# Effectiveness on level of consciousness of non-invasive neuromodulation therapy in patients with disorders of consciousness: a systematic review and meta-analysis

**DOI:** 10.3389/fnhum.2023.1129254

**Published:** 2023-05-24

**Authors:** Zhenyu Liu, Xintong Zhang, Binbin Yu, Jiayue Wang, Xiao Lu

**Affiliations:** Department of Rehabilitation Medicine, The First Affiliated Hospital of Nanjing Medical University, Nanjing, Jiangsu, China

**Keywords:** disorders of consciousness, coma, non-invasive neuromodulation therapy, transcranial Direct Current Stimulation, Transcranial Magnetic Stimulation, median nerve stimulation

## Abstract

**Background:**

Disorders of consciousness (DoC) commonly occurs secondary to severe neurological injury. A considerable volume of research has explored the effectiveness of different non-invasive neuromodulation therapy (NINT) on awaking therapy, however, equivocal findings were reported.

**Objective:**

The aim of this study was to systematically investigate the effectiveness on level of consciousness of different NINT in patients with DoC and explore optimal stimulation parameters and characteristics of patients.

**Methods:**

PubMed, Embase, Web of Science, Scopus, and Cochrane central register of controlled trials were searched from their inception through November 2022. Randomized controlled trials, that investigated effectiveness on level of consciousness of NINT, were included. Mean difference (MD) with 95% confidence interval (CI) was evaluated as effect size. Risk of bias was assessed with revised Cochrane risk-of-bias tool.

**Results:**

A total of 15 randomized controlled trials with 345 patients were included. Meta-analysis was performed on 13 out of 15 reviewed trials indicating that transcranial Direct Current Stimulation (tDCS), Transcranial Magnetic Stimulation (TMS), and median nerve stimulation (MNS) all had a small but significant effect (MD 0.71 [95% CI 0.28, 1.13]; MD 1.51 [95% CI 0.87, 2.15]; MD 3.20 [95%CI: 1.45, 4.96]) on level of consciousness. Subgroup analyses revealed that patients with traumatic brain injury, higher initial level of consciousness (minimally conscious state), and shorter duration of prolonged DoC (subacute phase of DoC) reserved better awaking ability after tDCS. TMS also showed encouraging awaking effect when stimulation was applied on dorsolateral prefrontal cortex in patients with prolonged DoC.

**Conclusion:**

tDCS and TMS appear to be effective interventions for improving level of consciousness of patients with prolonged DoC. Subgroup analyses identified the key parameters required to enhance the effects of tDCS and TMS on level of consciousness. Etiology of DoC, initial level of consciousness, and phase of DoC could act as significant characteristics of patients related to the effectiveness of tDCS. Stimulation site could act as significant stimulation parameter related to the effectiveness of TMS. There is insufficient evidence to support the use of MNS in clinical practice to improve level of consciousness in patients with coma.

**Systematic review registration:**

https://www.crd.york.ac.uk/PROSPERO/display_record.php?RecordID=337780, identifier: CRD42022337780.

## Introduction

During the past several years, with the development of resuscitation techniques and intensive care, the mortality rate of patients after traumatic brain injury (TBI), cerebral vascular accident (CVA), hypoxic-ischemic encephalopathy (HIE), or other severe neurological injury has decreased gradually (Stein et al., 2010; Fugate et al., 2012; Mensah et al., 2017; Jiang et al., 2019). However, patients who have survived may still suffer from a range of cognitive, emotional, and behavioral sequelae secondary to the injury (Howlett et al., 2022). Disorders of consciousness (DoC), as one of these sequelae, are characterized by the reduction of wakefulness and/or awareness (Sergi and Bilotta, 2020). The former refers to the state of consciousness, which is characterized by individual eyes-opening readiness to respond to stimuli in such a way as to favor continued health (Sergi and Bilotta, 2020). The latter refers to the content of consciousness, which is characterized by a serially time-ordered, organized, and reflective awareness of self and the environment (James, 1894). Coma, as the most severe stage of DoC, is characterized by the complete loss of wakefulness and awareness (Sergi and Bilotta, 2020). Such patients commonly exhibit eyes-closing and lack a normal sleep-wake cycle. Coma typically lasts only a few hours, days, or weeks and transitions into either a vegetative state/unresponsive wakefulness syndrome (VS/UWS) or a minimally conscious state (MCS). The fundamental difference between VS/UWS and MCS is whether the patient has inconsistent but clearly discernible behavioral evidence of self or environmental awareness, including simple command-following, gestural or verbal “yes/no” response, and purposeful motional or affective behaviors that occur concerning relevant environmental stimuli (Giacino et al., 2002; Porcaro et al., 2021). Although some patients with MCS may follow commands to a certain extent, functional communication remains challenging, which causes severe distress to their families. Furthermore, the cost of lifetime rehabilitation care for patients with DoC places heavy economic load on individuals and society (Adan Ali and Farah Yusuf Mohamud, 2022).

Although there are various treatments for DoC currently, only a small part of them have demonstrated a strong level of evidence (Thibaut et al., 2019). Regarding medical therapy, amantadine is the only medicine that has been recommended as effective treatment for patients with DoC after TBI between 4 and 16 weeks in American guidelines (Giacino et al., 2018b). However, its efficacy is still limited by specific population and duration of DoC, and cannot be extended to a broader group of patients with DoC at present. As for neuromodulation therapy, it can be further divided into invasive neuromodulation therapy and non-invasive neuromodulation therapy (NINT). The former usually applies direct stimulation of the brain or nerves through invasive approaches such as implanted electrodes. A recent open-label study of central thalamic deep brain stimulation in patients with DoC reported that four out of fourteen patients with VS/UWS or MCS showed positive effects on clinical recovery (Chudy et al., 2018). However, given the high risk of invasive operation and high surgery cost, NINT has shown unique advantages because of convenience, safety, and economics.

Among different types of non-invasive interventions, the most common one that is applied in clinical practice is non-invasive brain stimulation which includes transcranial Direct Current Stimulation (tDCS) and Transcranial Magnetic Stimulation (TMS). Both tDCS and TMS have the effect of regulating functional connectivity between different brain regions by modulating cortical excitability and neuroplasticity (Cirillo et al., 2017). In addition, non-invasive peripheral neuromodulation therapy such as median nerve stimulation (MNS), and transcutaneous auricular vagus nerve stimulation (taVNS) is also proposed for awaking therapy. In contrast to non-invasive brain stimulation, peripheral neuromodulation commonly regulates functional brain activity by modulating the impulses sent from peripheral nerves to the central nerve and indirectly adjusting the electrophysiological activity of cortical neurons (Briand et al., 2020).

Several systematic reviews and meta-analyses have examined whether tDCS, rTMS, or MNS can be applied as an effective awaking therapy in patients with DoC (Zaninotto et al., 2019; Feng et al., 2020; Feller et al., 2021; O'Neal et al., 2021). A recent meta-analysis on twelve trials indicated that tDCS could be expected to improve the level of consciousness in patients with DoC whereas TMS had no clear evidence (Feng et al., 2020). Remarkably, the conclusion of this review was limited to the effectiveness of tDCS but lacked further exploration for optimal stimulation parameters and characteristics of patients. Meanwhile, the conclusion for TMS was based on only two TMS studies. As a result, a high risk of bias of this conclusion induced by the limited number of studies could exist. In addition, a review conducted by Feller et al. (2021) only reported a qualitative result and drew an indefinite conclusion about the effectiveness of MNS. As a result, the objective of this review was to explore the effectiveness of different NINT and find out their optimal stimulation parameters and characteristics of patients with DoC.

## Methods

The systematic review and meta-analysis followed the Preferred Reporting Items for Systematic Reviews and Meta-Analyses (PRISMA) framework (Page et al., 2021) and was registered with PROSPERO (CRD42022337780).

### Eligible criteria

The Patient-Intervention-Comparison-Outcome-Study Design (PICOS) framework was used to organize the inclusion criteria.

(P) Studies recruited patients diagnosed with DoC by coma recovery scale-revised (CRS-R) or Glasgow coma scale (GCS). (I) Studies using tDCS, TMS, MNS, or any other type of NINT to investigate its effectiveness on level of consciousness in patients with DoC. (C) Studies adopting control conditions such as sham stimulation, no stimulation, or any active control intervention. (O) Studies adopted at least one of the clinical behavioral scales, neurophysiology evaluation, neuroimaging evaluation, or any other measures to assess level of consciousness. (S) Randomized controlled trials (RCTs) with parallel or cross-over design.

In addition to the above criteria, we only included studies reported in English. Review articles, conference abstracts, expert opinion papers, and editorials were excluded. Meanwhile, studies reporting on less than five patients or providing only one session intervention were excluded.

### Information sources

Electronic databases were searched from their inception through November 2022, including PubMed, Embase, Web of Science, Scopus, and Cochrane central register of controlled trials. Meanwhile, we searched the reference lists of included studies to identify further studies. The search was performed using the following keywords: (“disorders of consciousness” OR DoC OR Coma OR “Vegetative State” OR VS OR “unresponsive wakefulness syndrome” OR UWS or “minimally conscious state” OR MCS) AND (neuromodulation OR “non-invasive brain” OR “transcranial electrical current stimulation” OR TES OR “transcranial Direct Current Stimulation” OR tDCS OR “transcranial alternating current stimulation” OR tACS OR “transcranial random noise stimulation” OR tRNS OR “transcranial magnetic stimulation” OR TMS OR “theta burst stimulation” OR TBS OR “low-intensity focused ultrasound” OR LIFU OR “transcutaneous auricular vagus nerve stimulation” OR taVNS OR “Near-infrared laser stimulation” OR N-LT OR “focused shock wave therapy” OR F-SWT OR “median nerve stimulation” OR MNS). The detailed searching strategies were shown in Appendix S1.

### Selection process

After removing duplicates, two authors (B.B.Y, and J.Y.W.) independently screened all eligible articles and cross-checked the information. In a case where the eligibility of inclusion was conflicted, a third senior author (X.L.) was consulted to solve the dispute, and a final decision was made by consensus.

### Data collection process

Two authors (Z.Y.L. and X.T.Z.) independently extracted data using a standardized form for each eligible study. This form includes information concerning general information (e.g. title, first author, year of publication), methodology (e.g. study design, duration of the study), demographics (e.g. age, gender, time from injury to intervention), interventions (e.g. type of intervention, stimulation parameter), outcomes (e.g. outcome measures, evaluation timepoint) and adverse events. Disagreements between the two authors were resolved by discussion with a third senior author (X.L.).

### Study risk of bias assessment

We assessed the risk of bias in all included studies using revised Cochrane risk-of-bias tool (ROB version 2.0) (Sterne et al., 2019). Aspects of randomization process, deviations from the intended interventions, missing outcome data, measurement of the outcomes, and selection of the reported result were evaluated. Bias arising from period and carryover effects were additionally evaluated for cross-over studies. Each domain was assessed as “low risks”, “some concerns”, or “high risks”. Two authors (Z.Y.L. and X.T.Z.) independently accomplished assessment and resolved any discrepancy through discussion with a third senior author (X.L.).

### Effect measures

With the assistance of R statistical software, library “meta” was used to perform meta-analysis if at least two studies assessed one specific outcome. For outcomes based on continuous data obtained at the end of the intervention, we adopted mean difference (MD) with 95% confidence interval (CI) as effect size. For dichotomous outcomes obtained at the end of the intervention, we adopted risk ratio (RR) with 95% CI. For ordinal outcomes, if there was a cut-off point that could be obtained, we transformed the data into dichotomous data. Otherwise, it was calculated as continuous data.

### Synthesis methods

Meta-analyses were calculated following the methods suggested by the Cochrane Review (Higgins et al., 2011). Combined design meta-analytic formula, using the methods suggested by Elbourne et al. (Curtin et al., 2002; Elbourne et al., 2002), were used to combine parallel and cross-over trial results. With no carry-over effects in cross-over studies, trial results were combined with parallel studies by the combined design meta-analytic formula or included in qualitative analysis, depending on whether the study reported the order of crossover and individual specific different time-point results. Otherwise, only data from the first phase in cross-over studies was included and combined with parallel studies.

Statistical heterogeneity of included trials was evaluated with I^2^ statistic and between-study variance (τ^2^) (Higgins and Thompson, 2002). Studies with an I^2^ of 0 to 24% were considered as low heterogeneity; I^2^ of 25% to 49% as moderate heterogeneity; I^2^ of 50%-74% as substantial heterogeneity and 75%-100% as high heterogeneity (Higgins et al., 2003). When I^2^ was greater than 50%, it was assumed that there was considerable heterogeneity between studies, therefore random-effects model was applied. Sensitivity analyses were conducted by the “leave-one-out” method and omitting studies with high risk of bias.

To examine the differential effects of confounders, pre-planned subgroup analyses were conducted by certain parameters if data was available, including etiology of Doc (TBI, CVA or HIE), initial level of consciousness (Coma, VS/UWS or MCS), phase of DoC (Acute, Subacute or Chronic), and stimulation site (primary motor cortex, dorsolateral prefrontal cortex or others).

Moderator effects were examined by meta-regression using stimulation parameters as predictor variables. For tDCS, these parameters included number of total sessions and total stimulation time. For TMS, these parameters included frequency of stimulation, number of sessions, number of pulses per session, and total stimulation number of pulses.

### Reporting bias assessment

Reporting biases were assessed by a contour-enhanced funnel plot (Peters et al., 2008). Based on the effect sizes and standard errors of included studies, the significance of any effect size could be calculated and relevant study could be distributed to special color regions representing different significance levels. An asymmetrical appearance of the plots represents the existence of bias. When bias was detected through funnel plot, we used a trim and fill algorithm to adjust (Duval and Tweedie, 2000). The adjusted results obtained by the algorithm could balance the bias in the overall results of studies that were unpublished due to insignificant effects. Adjusted results combined with primary results were used to determine whether the bias remarkably affected effect size estimation.

## Results

### Study selection

**Figure 1:**
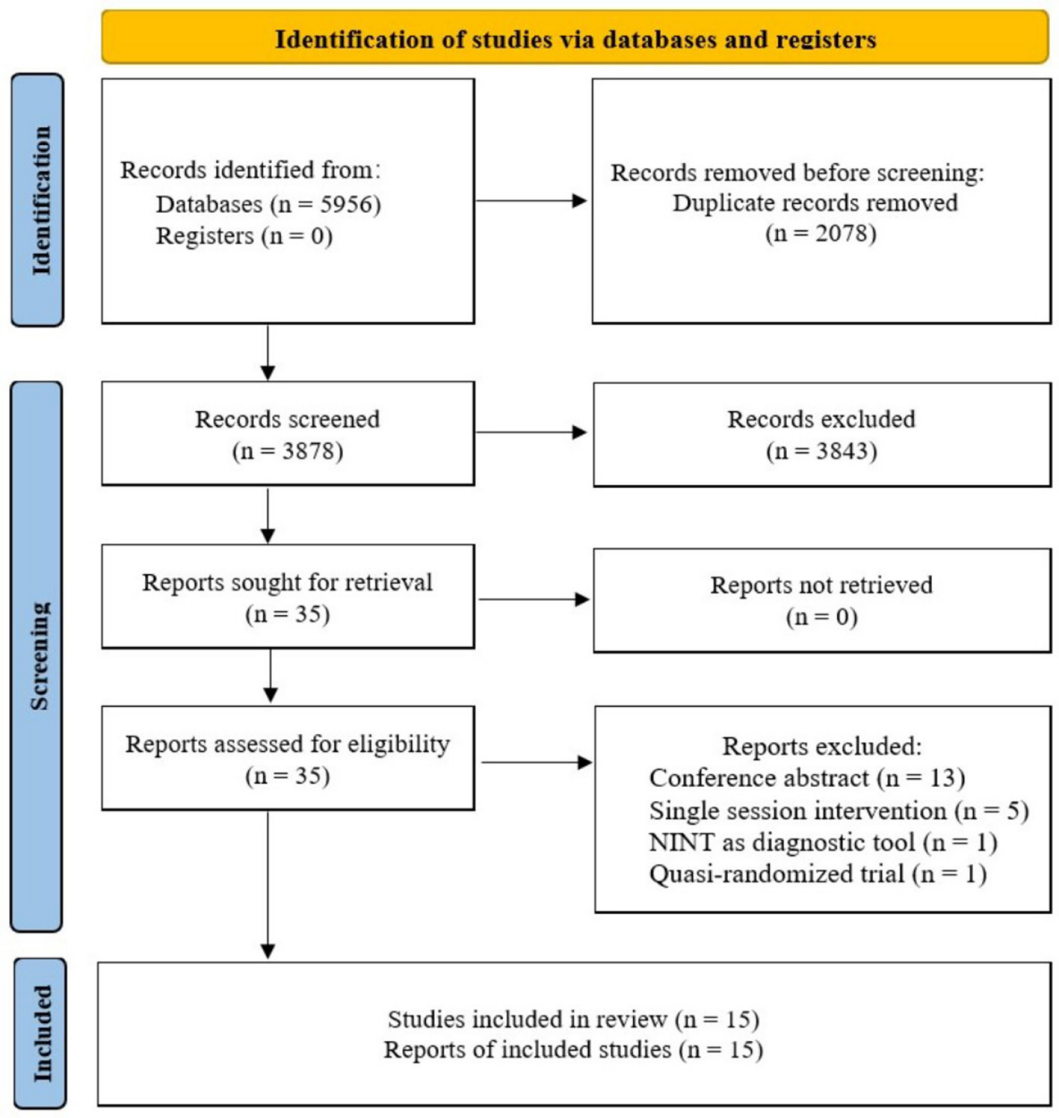
Flowchart summarizing study selection process. NINT, non-invasive neuromodulation therapy.

Process of study selection was summarized in Figure 1. A total of 5,956 studies were retrieved from the databases. After duplication elimination, 3,878 studies were obtained. Fifteen studies met the eligibility criteria and were finally included in the systematic review. Two studies were excluded from quantitative synthesis due to lack of sufficient data for obtaining effect sizes. A PDF document of the comprehensive search results including all the records was shown in Appendix S2.

### Study characteristics

Table 1 summarized characteristics of studies included in this review. A total of 15 randomized controlled studies with sample size ranging from 6 to 50 patients were included in this review. Regarding patients' initial level of consciousness, three studies included only patients with coma, four included patients with VS/UWS, three included patients with MCS, and the remaining five did not specify the type of DoC in their eligibility criteria.

**Table 1:** Characteristics of included studies.

**References**	**Country**	**Study design**	**Sample size**	**Intervention**	**Target**	**Stimulation parameter (intensity/duration/total sessions/pulses)**	**Control**	**Outcome**
Estraneo et al. (2017)	Italy	Cross-over	13	tDCS	LDLPFC	2 mA ^*^ 20 min/session ^*^ 5 sessions	Sham tDCS	CRS-R EEG
Huang et al. (2017)	Belgium	Cross-over	33	tDCS	PPC	2 mA ^*^ 20 min/session ^*^ 5 sessions	Sham tDCS	CRS-R
Thibaut et al. (2017)	Belgium	Cross-over	16	tDCS	LDLPFC	2 mA ^*^ 20 min/session ^*^ 5 sessions	Sham tDCS	CRS-R
Zhang et al. (2017)	China	Parallel	26	tDCS	LDLPFC	1 or 2 mA ^*^ 20 min/session ^*^ 20 sessions	Sham tDCS	CRS-R ERP
Martens et al. (2018)	Belgium	Cross-over	27	tDCS	LDLPFC	2mA ^*^ 20 min/session ^*^ 20 sessions	Sham tDCS	CRS-R
Cavinato et al. (2019)	Italy	Cross-over	24	tDCS	LDLPFC	2 mA ^*^ 20 min/session ^*^ 10 sessions	Sham tDCS	EEG CRS-R WNSSP
Wu et al. (2019)	China	Parallel	15	tDCS	LDLPFC or RDLPFC	2 mA ^*^ 20 min/session ^*^ 10 sessions	Sham tDCS	CRS-R EEG GCS-E
Cincotta et al. (2015)	Italy	Cross-over	11	TMS	LM1	90% RMT ^*^ 1000pulses/session ^*^ 5sessions Frequency: 20 Hz	Sham TMS	CRS-R CGI-I EEG
He et al. (2018)	China	Cross-over	6	TMS	LM1	100%RMT ^*^ 1,000pulses/session ^*^ 5 sessions Frequency: 20 Hz	Sham TMS	CRS-R EEG
Zhang et al. (2021)	China	Parallel	48	TMS	LDLPFC	80% RMT ^*^ 2,000 pulses/session ^*^ 40 sessions Frequency: 5 Hz	Sham TMS	CRS-R EEG
Chen et al. (2022)	China	Parallel	50	TMS	LDLPFC	90%RMT ^*^ 1,000 pulses/session ^*^ 30sessions; Frequency: 10Hz	Sham TMS	CRS-R GCS SEP BAEP
Fan et al. (2022)	China	Parallel	40	TMS	LDLPFC	100%RMT ^*^ 2,000 pulses/session ^*^ 20sessions Frequency: 20 Hz	Sham TMS	CRS-R
Cooper et al. (1999)	USA	Parallel	6	MNS	RMN	20mA ^*^ 8 or 12 h/session ^*^ 14sessions Frequency: 40 Hz Waveform: asymmetric biphasic	Not mentioned	GCS Days spent in ICU GOS
Peri et al. (2001)	USA	Parallel	10	MNS	LMN or RMN	15-20 mA ^*^ 8h/session ^*^ 14 sessions. Frequency: 40Hz Waveform: asymmetric biphasic	Sham MNS	Time out of coma GCS GOS FIM
Nekkanti et al. (2016)	India	Parallel	20	MNS	RMN	20 mA ^*^ 30 min/session ^*^ 30 sessions Frequency: 40 Hz. Waveform: asymmetric biphasic	Regular medication	GCS

tDCS, transcranial Direct Current Stimulation; LDLPFC, left dorsolateral prefrontal cortex; min, minute; CRS-R, coma recovery scale-revised; EEG, electroencephalography; PPC, posterior parietal cortex; ERP, event-related potential; WNSSP, Western Neurosensory Stimulation Profile; RDLPFC, right dorsolateral prefrontal cortex; GCS-E, Glasgow coma scale-extended; TMS, Transcranial Magnetic Stimulation; LM1, left primary motor cortex; RMT, resting motor threshold; CGI-I, clinical global impression scale-improvement; GCS, Glasgow coma scale; SEP, somatosensory evoked potential; BAEP, brainstem auditory evoked potential; USA, United States of America; MNS, median nerve stimulation; RMN, right median nerve; h, hour; GOS, Glasgow outcome scale; LMN, left median nerve; FIM, function independent measure.

As for the intervention of studies, seven studies used tDCS. Five studies used TMS. The remaining three studies used MNS. In addition, in terms of the design of studies, we included seven randomized cross-over controlled studies and eight randomized parallel controlled studies.

### tDCS studies

Seven tDCS studies enrolled 154 patients with VS/UWS or MCS. Clinical behavioral scales like CRS-R, Western Neurosensory Stimulation Profile (WNSSP), or neurophysiology evaluation like electroencephalogram (EEG), event-related potential (ERP) were evaluated as the outcome. Among them, Huang et al. (2017) and Thibaut et al. (2017) reported significant effects (*p* < 0.05) on CRS-R in patients with MCS. Both studies used a unilateral monopolar montage protocol and selected right supraorbital region as the reference cathode. It is worth noting that the former selected posterior parietal cortex (PPC) as the anodic stimulation site, whereas the latter chose the left dorsolateral prefrontal cortex (DLPFC). Cavinato et al. (2019) reported that only a proportion of patients with MCS had significant effects on WNSSP when the left DLPFC and the right deltoid muscle were selected as the anode and cathode respectively (*p* < 0.05). None of the remaining four studies reported any significant effects (Estraneo et al., 2017; Zhang et al., 2017; Martens et al., 2018; Wu et al., 2019).

### TMS studies

Five TMS studies recruited 155 patients with VS/UWS or MCS. CRS-R was the only commonly used outcome. In addition, neurophysiological evaluation such as EEG, somatosensory evoked potential (SEP), or brainstem auditory evoked potential (BAEP) was also used to assess patients' level of consciousness. Cincotta et al. (2015) and He et al. (2018) applied TMS on left primary motor cortex (M1) and reported no significant effect on CRS-R and EEG. Zhang et al. (2021) investigated the effects of TMS with left DLPFC on CRSR, EEG, and BAEP in patients with DoC and reported a significant effect (*p* < 0.05). Similarly, Chen et al. (2022) and Fan et al. (2022) selected left DLPFC as stimulation target and showed significant effects (*p* < 0.05) of TMS on CRS-R either.

### MNS studies

Three MNS studies enrolled 36 patients with coma. Cooper et al. (1999) and Nekkanti et al. (2016) both applied MNS on right median nerve and reported significant effects on GCS. However, Peri et al. (2001) used MNS on unilateral median nerve according to patients' injured brain hemisphere and reported no significant effect on GCS.

### Risk of bias in studies

**Figure 2:**
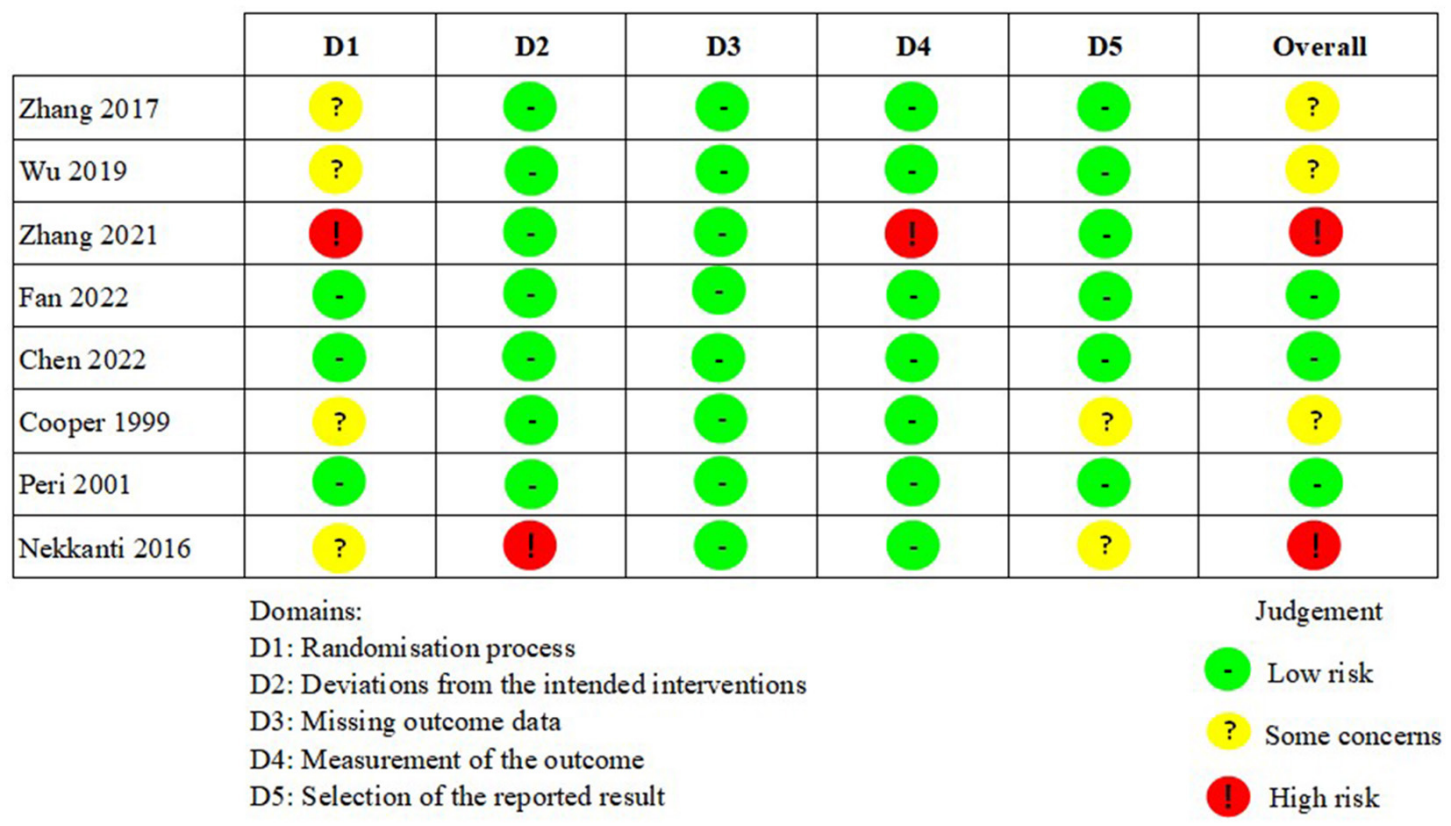
The risk of bias in parallel studies.

**Figure 3:**
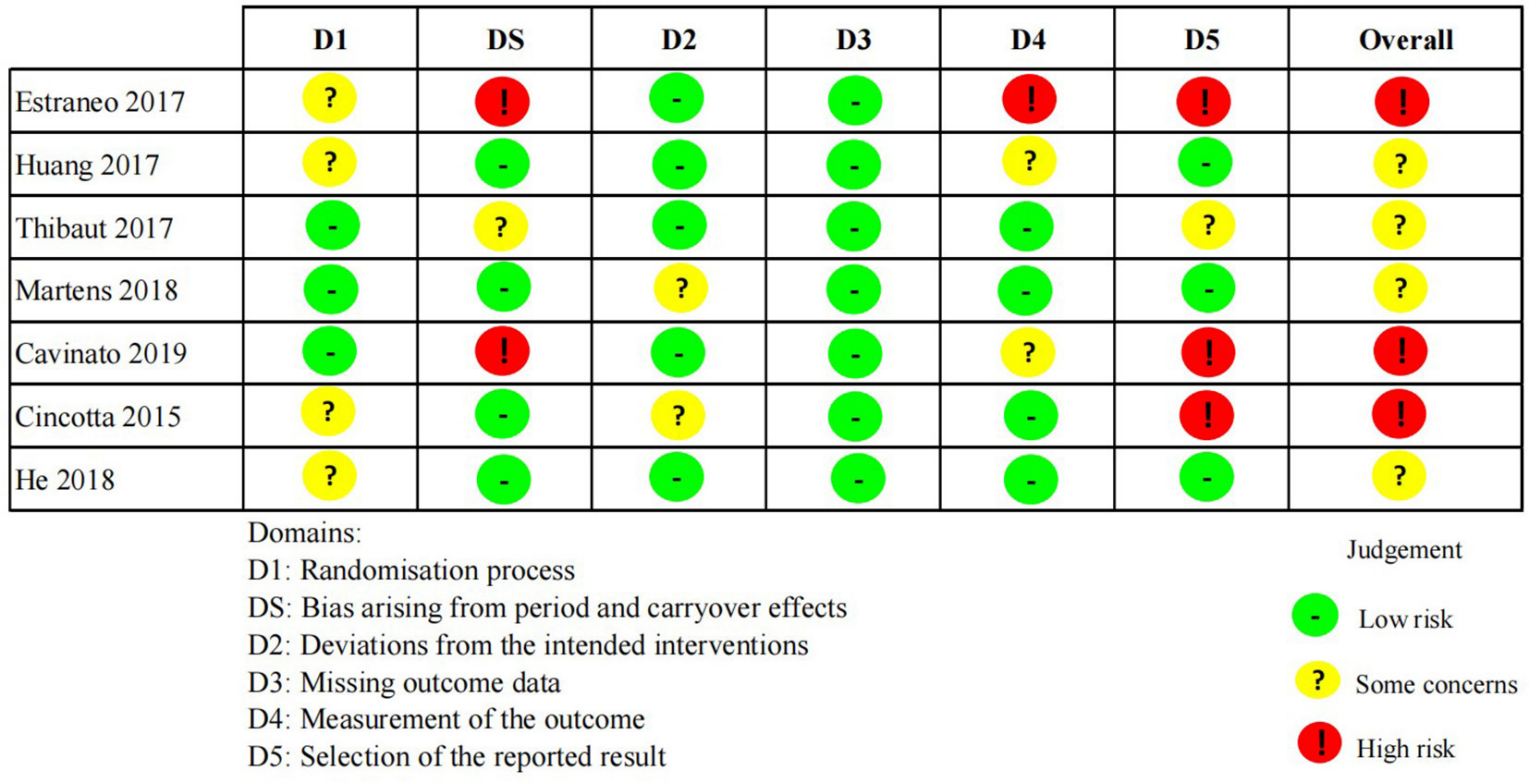
The risk of bias in cross-over studies.

Results from assessment of bias using revised Cochrane risk-of-bias tool for parallel and cross-over studies were presented in Figures 2, 3. Only three studies were assessed as “low risks” (Peri et al., 2001; Chen et al., 2022; Fan et al., 2022). In addition, the majority of included studies (*n* = 7) were assessed as “some concerns” because of indistinct illustration of randomization process or other relatively rare reasons (Cooper et al., 1999; Huang et al., 2017; Thibaut et al., 2017; Zhang et al., 2017; He et al., 2018; Martens et al., 2018; Wu et al., 2019). Five studies were assessed as “high risks” on account of significant bias in at least one domain (Cincotta et al., 2015; Nekkanti et al., 2016; Estraneo et al., 2017; Cavinato et al., 2019; Zhang et al., 2021).

### Synthesis of results

Thirteen studies were included in the meta-analysis. CRS-R was the only commonly used outcome in six tDCS studies and five TMS studies. Similarly, GCS was the only outcome that could be extracted from two MNS studies. Separate meta-analyses were conducted for tDCS, TMS, and MNS studies.

### Effectiveness of tDCS on level of consciousness

**Figure 4:**
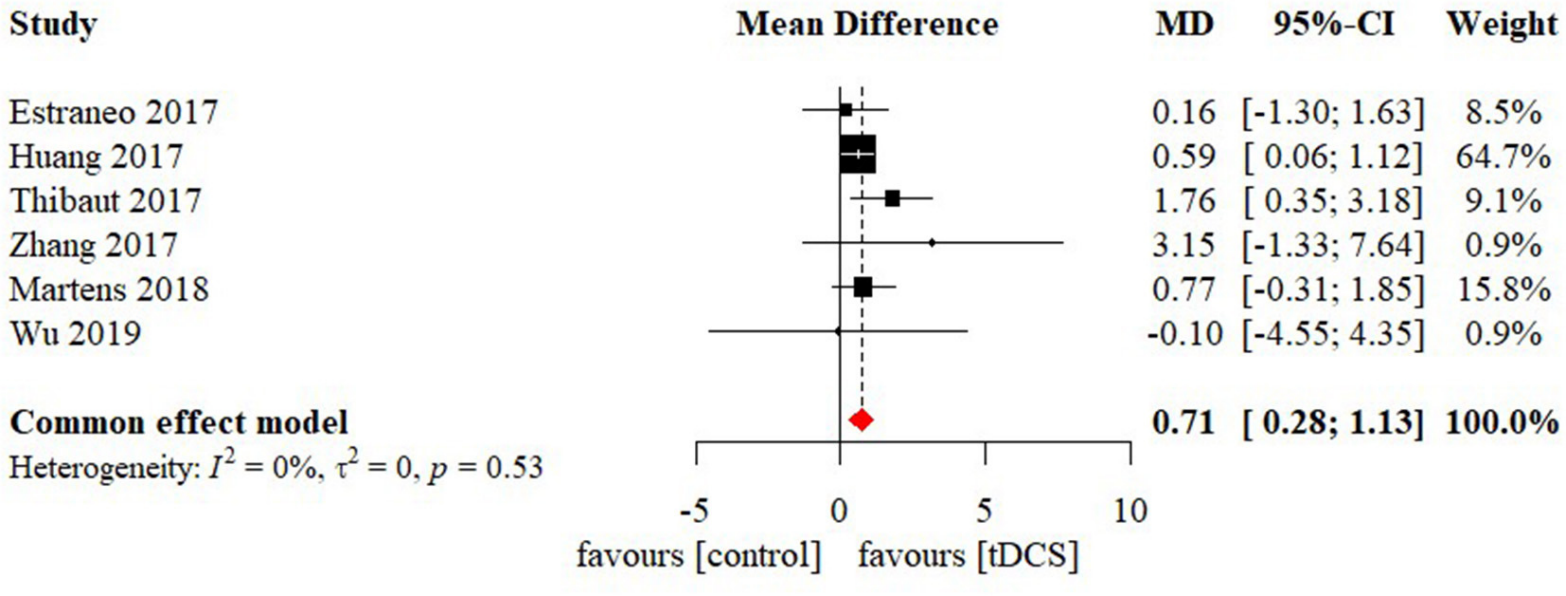
Statistical summary and forest plot of effect of tDCS studies. tDCS, transcranial Direct Current Stimulation; MD, mean difference; CI, confidence interval.

**Figure 5:**
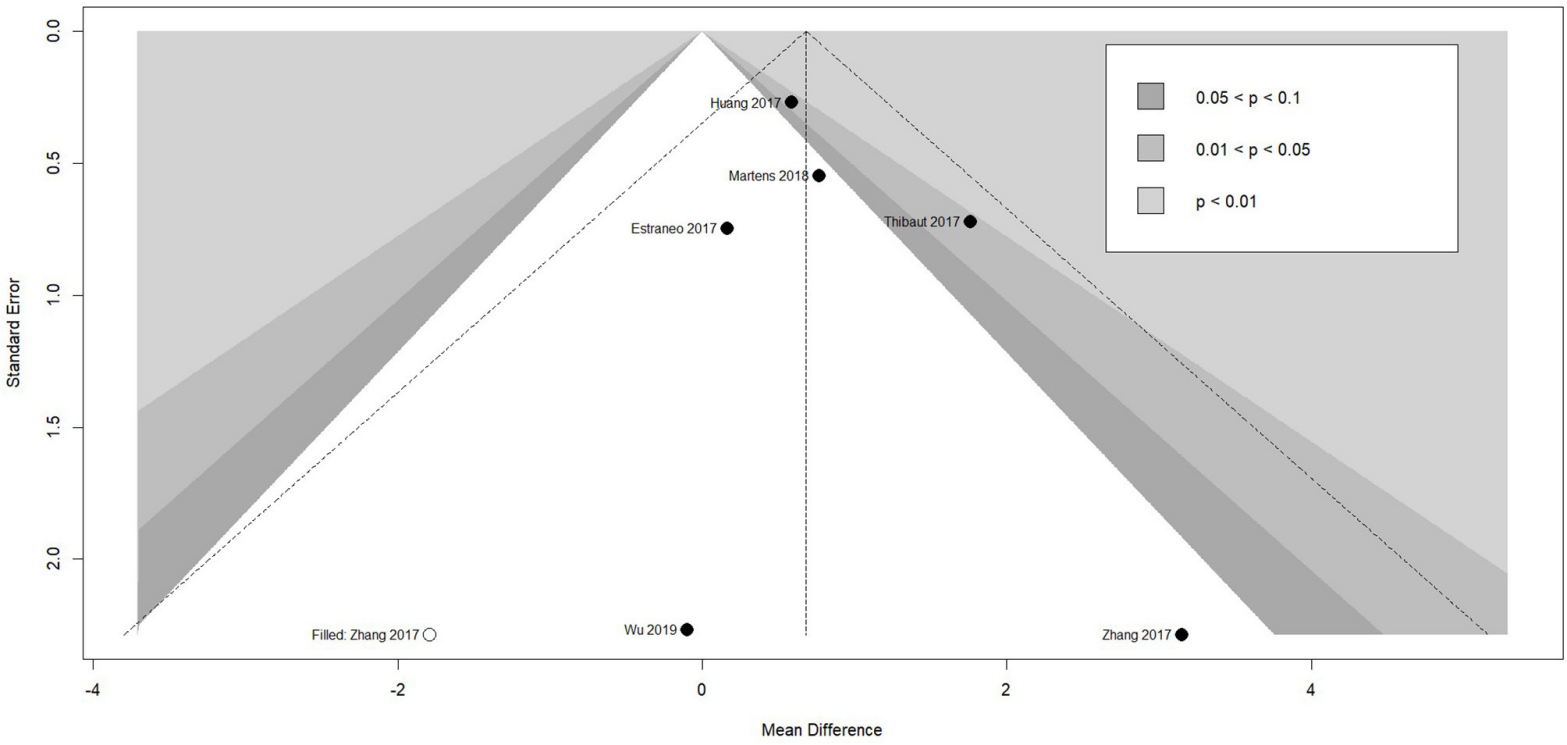
Funnel plot of the reporting biases in tDCS studies. tDCS, transcranial Direct Current Stimulation.

Meta-analysis of effectiveness of tDCS on CRS-R of six studies was presented in Figure 4. There was a small but significant effect size (MD 0.71 [95% CI 0.28, 1.13], p < 0.01) without significant heterogeneity (I^2^ = 0.0%, *p* = 0.53). Furthermore, result was stable when we adopted sensitivity analysis (Table 2). The contour-enhanced funnel plot (Figure 5) with the trim and fill method did not show evidence of reporting bias (MD 0.68 [95% CI 0.26, 1.11], *p* < 0.01).

**Table 2:** Sensitivity analyses in tDCS and TMS studies.

**Studies omitted**	**MMD**	**95% CI**	***P* value**	**Weight in total synthesis**
**tDCS studies**				
Estraneo et al. (2017)^†^	0.76	0.31–1.20	< 0.01	8.50%
Huang et al. (2017)	0.92	0.20–1.64	0.01	64.70%
Thibaut et al. (2017)	0.60	0.15–1.05	< 0.01	9.10%
Zhang et al. (2017)	0.68	0.25–1.11	< 0.01	0.90%
Martens et al. (2018)	0.69	0.23–1.16	< 0.01	15.80%
Wu et al. (2019)	0.71	0.28–1.14	< 0.01	0.90%
High risks	0.76	0.31–1.20	< 0.01	8.50%
**TMS studies**				
Cincotta et al. (2015)^†^	1.74	1.11–2.37	< 0.01	14.40%
He et al. (2018)	1.59	0.99–2.19	< 0.01	6.20%
Zhang et al. (2021)^†^	1.65	0.82–2.48	< 0.01	50.50%
Chen et al. (2022)	1.51	0.87–2.15	< 0.01	16.30%
Fan et al. (2022)	1.51	0.88–2.13	< 0.01	12.50%
High risks	2.03	1.04–3.02	< 0.01	64.90%

^†^Studies with a high risk of bias.

tDCS, transcranial Direct Current Stimulation; TMS, Transcranial Magnetic Stimulation; MMD, modified mean difference; CI, confidence interval.

To examine the differential effects of confounders, subgroup analyses were conducted. As shown in Table 3, the subgroup analysis by the etiology of DoC revealed that among patients with TBI, tDCS showed a positive and significant effect size on CRS-R (MD 1.09 [95%CI 0.37, 1.82], *p* = 0.003), while patients with CVA had a positive but insignificant effect size (MD 0.53 [95%CI−0.10, 1.163], *p* = 0.10) and patients with HIE only showed a negative and insignificant effect size (MD−0.30 [95%CI−1.50, 0.91], *p* = 0.63).

**Table 3:** Subgroup analyses for tDCS and TMS studies on CRS-R.

**Subgroup analyses**	**Category**	**Studies**	**MD**	**95% CI**	**P value**	**I^2^**
**tDCS studies**						
Etiology of DoC	TBI	5	1.09	0.37–1.82	< 0.01	21.50%
	CVA	3	0.53	−0.10–1.16	0.10	0.00%
	HIE	3	−0.30	−1.50–0.91	0.63	0.00%
Initial level of consciousness	MCS	6	1.08	0.40–1.77	< 0.01	50.30%
	VS/UWS	4	−0.10	−1.45–1.24	0.88	0.00%
Phase of DoC	Subacute	3	0.97	0.13–1.81	0.02	43.90%
	Chronic	6	0.55	−0.03–1.13	0.06	41.80%
Stimulation site	DLPFC	5	0.92	0.20–1.64	0.01	0.00%
	PPC	1	0.59	0.06–1.12	N/A	N/A
**TMS studies**						
Stimulation site	DLPFC	3	1.75	1.09–2.40	< 0.01	0.00%
	M1	2	1.01	−0.28–2.30	0.13	0.00%

tDCS, transcranial Direct Current Stimulation; TMS, Transcranial Magnetic Stimulation; CRS-R, coma recovery scale-revised; MD, mean difference; CI, confidence interval; DoC, disorders of consciousness; TBI, traumatic brain injury; CVA, cerebral vascular accident; HIE, hypoxic-ischemic encephalopathy; MCS, minimally conscious state; VS/UWS, vegetative state/unresponsive wakefulness syndrome; DLPFC, dorsolateral prefrontal cortex; PPC, posterior parietal cortex; M1, primary motor cortex.

Factors that showed a significant effect size favoring tDCS include initial level of consciousness among patients with MCS (MD 1.08 [95%CI 0.40, 1.77], *p* = 0.004), subacute phase of DoC (MD 0.97 [95%CI 0.13, 1.81], p = 0.02) and DLPFC as the stimulation target (MD 0.92 [95%CI 0.20, 1.64], p = 0.01).

As shown in Table 4, the meta-regression analysis showed that none of the between-study variables significantly predicted the effects of tDCS (number of total sessions: β = 0.01, *p* = 0.71; total stimulation time: β = 0.00, *p* = 0.71).

**Table 4:** Results from meta-regression analyses examining the effects of stimulation parameters.

**Study type/predictor variable**	**Beta**	**95% CI**	***P* value**	**I^2^**
**tDCS studies**				
Number of total sessions	0.01	−0.06–0.09	0.71	0.00%
Total stimulation time	0.00	−0.01–0.01	0.71	0.00%
**TMS studies**				
Frequency of stimulation	−0.01	−0.09–0.08	0.88	0.00%
Number of sessions	0.01	−0.03– 0.05	0.63	0.00%
Number of pulses per session	0.00	−0.01–0.01	0.74	0.00%
Total stimulation number of pulses	0.00	−0.01–0.01	0.80	0.00%

CI, confidence interval; tDCS, transcranial Direct Current Stimulation; TMS, Transcranial Magnetic Stimulation.

### Effectiveness of TMS on level of consciousness

**Figure 6:**
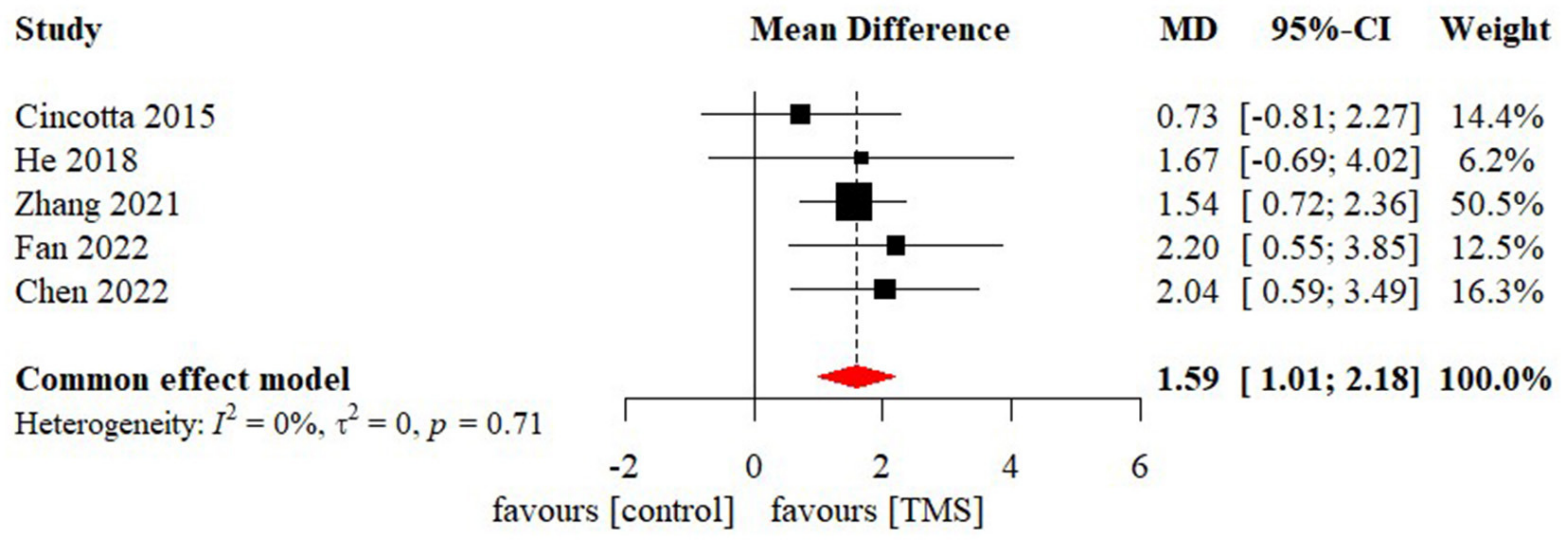
Statistical summary and forest plot of effect of TMS studies. TMS, Transcranial Magnetic Stimulation; MD, mean difference; CI, confidence interval.

**Figure 7:**
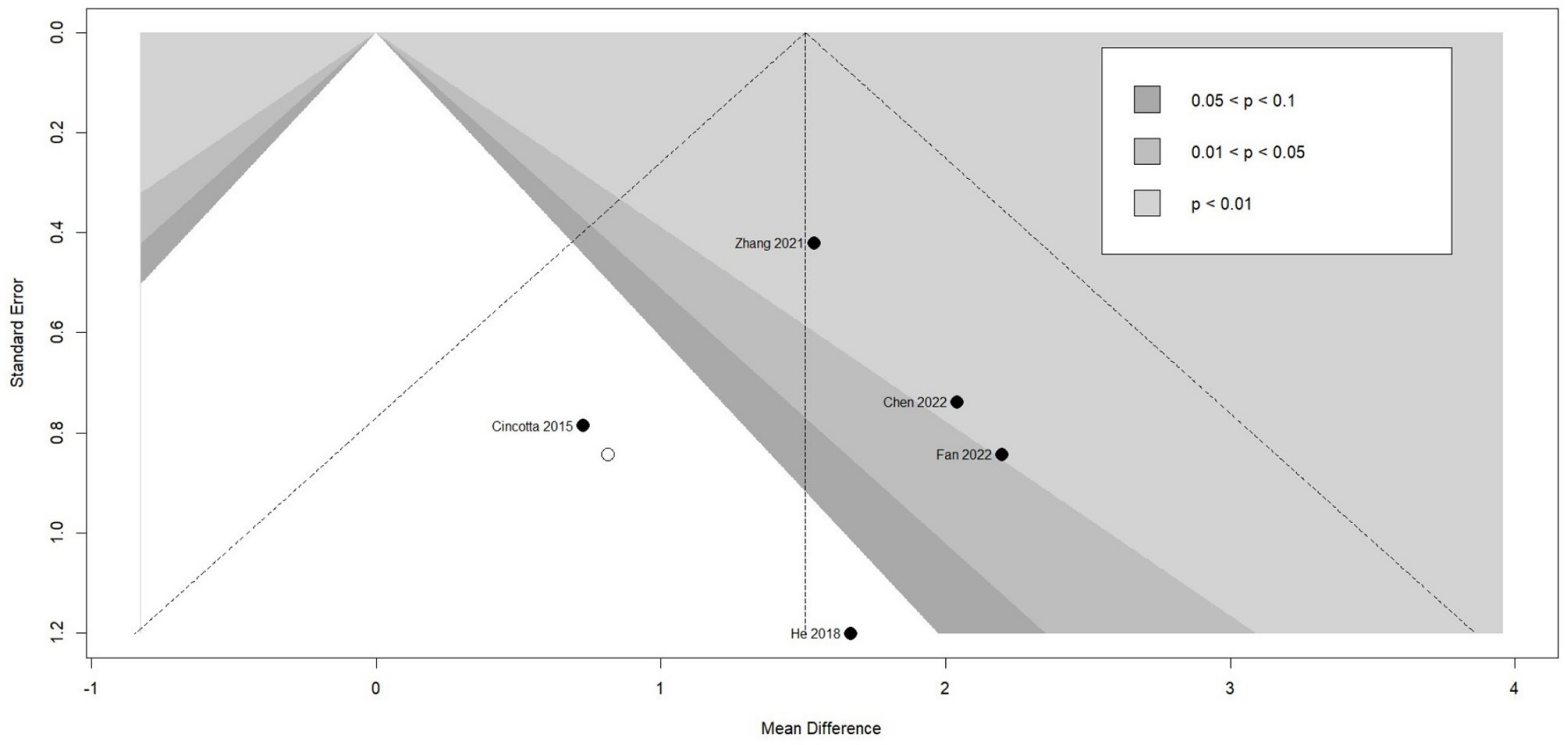
Funnel plot of the reporting biases in TMS studies. TMS, Transcranial Magnetic Stimulation.

Meta-analysis of effectiveness of TMS on CRS-R of five studies was presented in Figure 6. There was a small but significant effect size (MD 1.59 [95% CI 1.01, 2.18], p < 0.01). Non-significant level of heterogeneity (I^2^ = 0.0%, *p* = 0.71) was found. The result was further confirmed by using sensitivity analysis (Table 2). The contour-enhanced funnel plot (Figure 7) with the trim and fill method did not show evidence of reporting bias (MD 1.51 [95% CI 0.96, 2.06], *p* < 0.01).

To examine the differential effects of confounders, subgroup analyses were conducted. None of the four included TMS studies specified subjects from their etiology, initial level of consciousness, and duration of DoC. Individual patient data related to the above variables could not be extracted either. As a result, subgroup analysis was only conducted for stimulation site. As shown in Table 3, only patients who applied TMS on DLPFC showed a positive and significant effect size (MD 1.75 [95%CI 1.09, 2.40], p < 0.01), while patients who applied TMS on M1 had a small but insignificant effect size (MD 1.01 [95%CI−0.28, 2.30], *p* = 0.13).

As shown in Table 4, the meta-regression analysis showed that none of the between-study variables significantly predicted the effects of TMS (frequency of stimulation: β = −0.01, *p* = 0.88; number of sessions: β = 0.01, *p* = 0.63; number of pulses per session: β = 0.00, *p* = 0.74; total stimulation number of pulses: β = 0.00, *p* = 0.80).

### Effectiveness of MNS on level of consciousness

**Figure 8:**
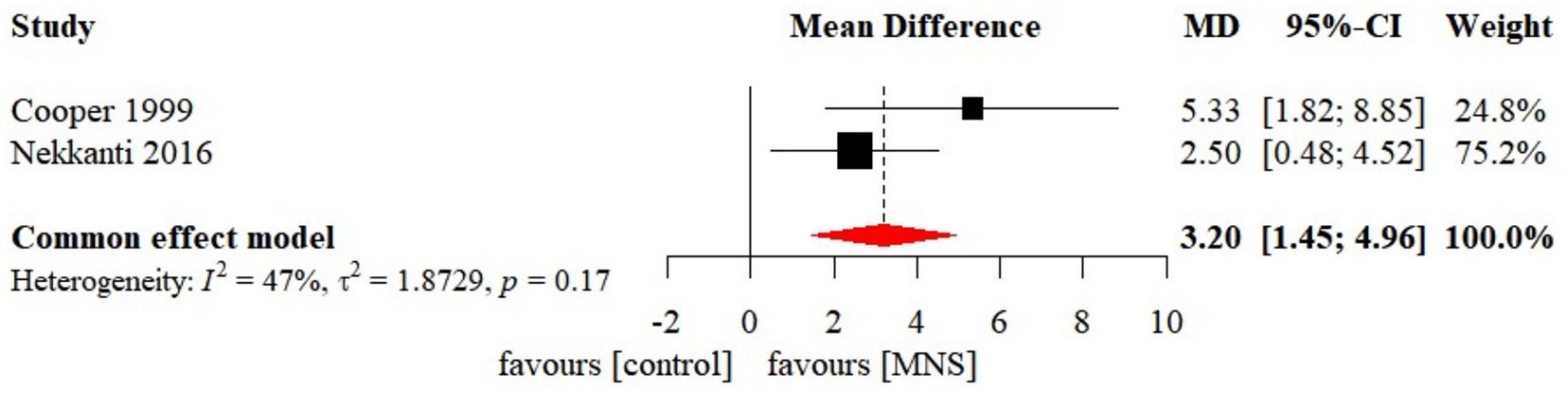
Statistical summary and forest plot of effect of MNS studies. MNS, median nerve stimulation; MD, mean difference; CI, confidence interval.

Meta-analysis of effects of MNS on GCS was presented in Figure 8. Only two MNS studies were included in the meta-analysis of effects of MNS on GCS. There was a significant effect size in GCS (MD 3.20 [95%CI: 1.45, 4.96], *p* < 0.001) favoring the MNS group. Sensitivity analysis, reporting bias, subgroup analysis, and meta-regression were not conducted due to the limited number of studies.

## Discussion

The current study evaluated the effect of NINT on various neurobehavioral or electrophysiological evaluation in patients with DoC. Compared to sham intervention, the synthesized results revealed small but significant effects in favor of tDCS, TMS, and MNS. Notably, the mechanisms of recovery of consciousness among different phases of DoC are distinct. Based on the current cellular and circuit hypothesis, the recovery of consciousness depends on the recovery of neural activities of cortex, thalamus, and striatum and the re-emergence of dynamic interactions between multiple cerebral networks, such as the mesocircuit, frontoparietal network, and ascending reticular activating system (ARAS) (Edlow et al., 2021). A common pathophysiological mechanism of coma is widespread impairment of cortical neuronal excitatory activity, which may stem from structural lesions of cerebral cortex and/or insufficient input from the ARAS to the mesocircuit and frontoparietal network (Steriade et al., 1993; Timofeev et al., 2000). With the recovery of condition, patients with coma gradually transition into prolonged DoC (i.e., VS/UWS and MCS), which refers to any DoC that has lasted for more than 4 weeks following sudden brain injury of any cause (Giacino et al., 2018b; Physicians, 2020). The pathophysiology of prolonged DoC is typically characterized by functional recovery of ARAS, whereas the connectivity between functional networks critical for processing intrinsic thoughts and extrinsic stimuli remains disjointed (Steriade, 1996; Silva et al., 2010). In addition, the variability of stimulation parameters across different protocols may also contribute to the difference in awaking effect. Therefore, considering the difference in patients and stimulation parameters of these included studies, the effectiveness of any single intervention cannot be simply extended to the entire population of patients with DoC. This further highlights the necessity to conduct subgroup analysis and meta-regression to explore the optimal characteristics of patients and stimulation parameters.

### Effectiveness of tDCS on level of consciousness

The meta-analysis of the effect of tDCS on level of consciousness in patients with prolonged DoC indicated a positive, albeit, small significant effect size. Subgroup analyses revealed that only patients with TBI presented significant improvement in the level of consciousness compared to patients with CVA or HIE. Moreover, we also found that higher initial level of consciousness (MCS) and shorter duration of prolonged DoC (Subacute phase of DoC) may be associated with better clinical awaking effects.

Regarding the stimulation parameter, almost all studies adopt the same stimulation intensity and stimulation time per session. The results of meta-regression also showed a non-significant “dose-dependent” correlation between total stimulation duration and effectiveness. A possible explanation for this might be that the current result was based only on the short-term effects. Whether the benefit of tDCS in long-term effects improves with an increasing number of sessions remains to be discussed in future studies. As for the stimulation sites, five studies selected DLPFC as the anodic stimulation site and only one study selected PPC. As a result, only the effectiveness of tDCS applied on DLPFC could be confirmed. The effectiveness of tDCS applied on other sites remains to be explored.

Compared to previously published reviews (Zaninotto et al., 2019; Feng et al., 2020), our finding was consistent with that of Feng et al. who reported a positive effect of tDCS in patients with MCS. In addition to specific initial level of consciousness of patients, we found that etiology and phase of DoC could be significant factors for effectiveness of tDCS. This finding was also consistent with the current consensus that patients with MCS or TBI had a better prognosis compared to other diagnostic or aetiologic subtypes (Giacino et al., 2018a). Our results, while preliminary, suggested that the above characteristics of patients could contribute to the effectiveness of tDCS.

### Effectiveness of TMS on level of consciousness

The meta-analysis of the effect of TMS on level of consciousness in patients with prolonged DoC indicated a small but significant effect size. Regarding the stimulation parameter, the result of meta-regression showed no linear relationship between stimulation frequency, stimulation duration, or number of stimulation pulses and effectiveness. One reason for this result might be that all included studies utilized high-frequency (5–20 Hz) TMS. Similar excitatory effect on the cortex was produced with the long-term potentiation induced by high-frequency stimulation (Pascual-Leone et al., 1994). On the other hand, the absence of “dose-dependent” correlation might also be attributed to the lack of long-term follow-up data. Whether the benefit of TMS in long-term effects improves with an increasing number of sessions or pulses remains to be discussed in future studies.

Subgroup analysis revealed that only patients who applied TMS on DLPFC presented significant improvement in the level of consciousness compared to patients who applied TMS on M1. Interestingly, this finding was partly consistent with that of Feng et al. A non-significant awaking effect was found by two studies that applied TMS on M1 (Feng et al., 2020). Compared to this previous review, the reason that caused the difference stemmed from three newly included TMS studies in our study. Given the results of subgroup analysis and meta-regression, the main factor for the opposite conclusions might be attributed to the different stimulation sites of TMS. As compared to M1, it seems that TMS has an awaking effect via DLPFC. A possible explanation for this hypothesis could be linked to the function of different cerebral networks. DLPFC, as a critical component of executive control network (ECN), plays a vital role in mediating environmental awareness and repairing the imbalance between the ECN and default mode network (DMN) (Seeley et al., 2007). Therefore, it can be assumed that stimulation of DLPFC could modulate internetwork connectivity between ECN and DMN via salience network and accelerate patients' transition from VS/UWS to MCS. However, this assumption needs to be verified further. Future work is required to determine whether DLPFC is the most optimal stimulation site for TMS.

### Effectiveness of MNS on level of consciousness

The meta-analysis of the effect of MNS on level of consciousness in patients with coma indicated a small but significant effect size. Notably, the results should be interpreted cautiously, considering the high risk induced by the limited number of available studies. In contrast to previous studies, one systematic review reported qualitative results and expressed concerns about the effectiveness of MNS (Feller et al., 2021). Currently, the mechanism regarding the awaking effect of MNS remains unclear. One possible mechanism is that MNS enhances ARAS activity by stimulating the locus coeruleus and dorsal raphe nucleus, which represents the origins of the noradrenergic and serotonergic neurotransmitter systems, respectively (Kayama and Koyama, 1998). Whether MNS has an awaking effect in patients with prolonged DoC also lacks evidence from research. Nonetheless, the convenience and economics of MNS allow caregivers to provide beside therapy without the assistance of medical staff. As a result, the effectiveness of MNS on level of consciousness remains to be explored in future studies.

### Discernible effects of NINT on level of consciousness

Our finding, while preliminary, further supported the validity of the cellular and circuit mechanism (Edlow et al., 2021). Both central neuromodulation applied on the DLPFC (e.g., tDCS and TMS) and peripheral neuromodulation applied on the median nerve were involved in the reorganization of dynamic interactions between multiple cerebral networks. Notably, as consciousness was dominated by complex cerebral networks, the stimulation of a single neural circuit might not extend to other neural networks. Therefore, compared with the single NINT commonly used in clinical research, whether the combination of multiple neuromodulation therapies can achieve better awaking effects by activating widespread functional connectivity between brain regions remains to be further investigated.

### Limitation

Our review cannot be ruled out with limitations. Firstly, although most studies applied assessments other than neurobehavioral evaluation as outcomes, it is difficult to combine these results into synthesis analysis because of their varying collection and analysis methods. Therefore, our meta-analyses were based only on CRS-R and GCS. Secondly, due to few studies reported follow-up results, our finding was only applied to short-term effects. Future studies need to further explore the effectiveness of NINT in long-term awaking effects. Thirdly, limited by the fact that included tDCS studies and TMS studies had only one common outcome, as well as the lack of direct comparison between tDCS and TMS, it is difficult to conduct a network meta-analysis. As a result, it is hard to draw a definite ranking list of the superiority of the two interventions. Finally, although several new studies of NINT such as taVNS, low-intensity focused stimulations (LIFUS), and focused shock wave therapy (F-SWT) have been published in recent years and both have reported encouraging results in awaking therapy (Hesse et al., 2016; Cain et al., 2021, 2022). Most of them are still case series and need further validation through more high-quality randomized controlled trials. Therefore, only tDCS, TMS, and MNS were included in our review.

## Conclusion

In light of the findings of this review, based on the limited neurobehavioral outcomes measured by CRS-R or GCS, the existing evidence shows that tDCS and TMS may be advantageous to the recovery of consciousness in patients with prolonged DoC. Etiology of DoC, initial level of consciousness, and phase of DoC could act as significant characteristics of patients related to the effectiveness of tDCS. Stimulation site could act as significant stimulation parameter related to the effectiveness of TMS. In addition, there is limited evidence to suggest that MNS may improve level of consciousness in patients with coma. Considering the convenience and better tolerability, MNS may also have a promising role in awaking therapy in the future. Further research should investigate the optimal parameters and ranking list of different NINT through more high-quality randomized controlled trials.

## Data availability statement

The raw data supporting the conclusions of this article will be made available by the authors, without undue reservation.

## Author contributions

XL was responsible for conception and design of the study. ZL and XZ were responsible for data extraction and data analysis and drafted the manuscript. BY and JW were responsible for trial screening. All authors contributed to manuscript revision, read, and approved the submitted version.
