# Supplementary material for: Effectiveness on level of consciousness of non-invasive neuromodulation therapy in patients with disorders of consciousness: a systematic review and meta-analysis
Source: Front Hum Neurosci. 2023 May 24;17:1129254. doi: 10.3389/fnhum.2023.1129254 (PMC10246452; doi:10.3389/fnhum.2023.1129254)
Supplement: Supplementary file 1 [file Data_Sheet_1.PDF]

**Searching strategies**

**1. Searching Strategy for Medline**

#1 "Consciousness Disorders"[Mesh]

#2 Unconsciousness[Title/Abstract] OR Coma[Title/Abstract] OR Persistent Vegetative State[Title/Abstract] OR stupor[Title/Abstract] OR Syncope[Title/Abstract] OR Consciousness Disorder[Title/Abstract] OR Consciousness, Level Depressed[Title/Abstract] OR Depressed Level of Consciousness[Title/Abstract] OR Semiconsciousness[Title/Abstract] OR Consciousness, Level Altered[Title/Abstract] OR Altered Level of Consciousness[Title/Abstract] OR "disorders of consciousness"[Title/Abstract] OR "disorder of consciousness" [Title/Abstract] OR DOC[Title/Abstract] OR "Vegetative State"[Title/Abstract] OR "unresponsive wakefulness syndrome"[Title/Abstract] OR UWS[Title/Abstract] OR "minimally consciousness state"[Title/Abstract] OR MCS[Title/Abstract] OR "minimally conscious"[Title/Abstract]

#3 #1 OR #2

#4 "Transcutaneous Electric Nerve Stimulation"[Mesh] OR "Transcranial Direct Current Stimulation"[Mesh] OR "Transcranial Magnetic Stimulation"[Mesh]

#5 neuromodulation[Title/Abstract] OR "neuromodulation therapy"[Title/Abstract] OR "neuromodulation techniques"[Title/Abstract] OR "neuromodulatory techniques" [Title/Abstract] OR "non-invasive brain"[Title/Abstract] OR NIBI[Title/Abstract] OR NIBS[Title/Abstract] OR "transcranial electrical current stimulation"[Title/Abstract] OR tECS[Title/Abstract] OR "transcranial direct current stimulation"[Title/Abstract] OR tDCS[Title/Abstract] OR "transcranial alternating current stimulation"[Title/Abstract] OR tACS[Title/Abstract] OR "transcranial magnetic stimulation"[Title/Abstract] OR TMS[Title/Abstract] OR "repetitive transcranial magnetic stimulation"[Title/Abstract] OR rTMS[Title/Abstract] OR "theta burst stimulation"[Title/Abstract] OR TBS[Title/Abstract] OR cTBS[Title/Abstract] OR iTBS[Title/Abstract] OR "low-intensity focused ultrasound"[Title/Abstract] OR LIFUS[Title/Abstract] OR "transcutaneous auricular vagus nerve stimulation"[Title/Abstract] OR taVNS[Title/Abstract] OR "Near-infrared laser stimulation"[Title/Abstract] OR

N-LT[Title/Abstract] OR "focused shock wave therapy"[Title/Abstract] OR F-SWT[Title/Abstract] OR "median nerve stimulation"[Title/Abstract] OR MNS[Title/Abstract] OR rMNS[Title/Abstract]

#6 4# or 5#

#7 3# and 6#

**2. Searching Strategy for EMBASE**

#1 'consciousness disorder'/exp

#2 unconsciousness:ab,kw,ti OR coma:ab,kw,ti OR 'persistent vegetative state':ab,kw,ti OR stupor:ab,kw,ti OR syncope:ab,kw,ti OR 'consciousness disorder':ab,kw,ti OR 'consciousness disorders':ab,kw,ti OR 'consciousness, level depressed':ab,kw,ti OR 'depressed level of consciousness':ab,kw,ti OR 'semiconsciousness':ab,kw,ti OR 'consciousness, level altered':ab,kw,ti OR 'altered level of consciousness':ab,kw,ti OR 'disorders of consciousness':ab,kw,ti OR 'disorder of consciousness':ab,kw,ti OR doc:ab,kw,ti OR 'vegetative state':ab,kw,ti OR 'unresponsive wakefulness syndrome':ab,kw,ti OR uws:ab,kw,ti OR 'minimally consciousness state':ab,kw,ti OR mcs:ab,kw,ti

#3 #1 or #2

#4 'transcutaneous electrical nerve stimulation'/exp or 'transcranial direct current stimulation'/exp or 'transcranial magnetic stimulation'/exp

#5 neuromodulation:ab,kw,ti OR 'neuromodulation therapy':ab,kw,ti OR 'neuromodulation techniques':ab,kw,ti OR 'neuromodulatory techniques':ab,kw,ti OR 'non-invasive brain':ab,kw,ti OR nibi:ab,kw,ti OR nibs:ab,kw,ti OR 'transcranial electrical current stimulation':ab,kw,ti OR tecs:ab,kw,ti OR 'transcranial direct current stimulation':ab,kw,ti OR tdcs:ab,kw,ti OR 'transcranial alternating current stimulation':ab,kw,ti OR tacs:ab,kw,ti OR 'transcranial magnetic stimulation':ab,kw,ti OR tms:ab,kw,ti OR 'repetitive transcranial magnetic stimulation':ab,kw,ti OR rtms:ab,kw,ti OR 'theta burst stimulation':ab,kw,ti OR tbs:ab,kw,ti OR ctbs:ab,kw,ti OR itbs:ab,kw,ti OR 'low-intensity focused ultrasound':ab,kw,ti OR lifus:ab,kw,ti OR 'transcutaneous auricular vagus nerve stimulation':ab,kw,ti OR tavns:ab,kw,ti OR 'near-infrared laser stimulation':ab,kw,ti OR 'n lt':ab,kw,ti OR 'focused shock wave therapy':ab,kw,ti OR 'f swt':ab,kw,ti OR 'median nerve stimulation':ab,kw,ti OR mns:ab,kw,ti OR rmns:ab,kw,ti

#6 #4 or #5

#7 [embase]/lim NOT ([embase]/lim AND [medline]/lim)

#8 #3 and #6 and #7

**3. Searching Strategy for Web of Science**

#1 TS=("Consciousness Disorders" OR "Unconsciousness" OR "Coma" OR "Persistent Vegetative State" OR "stupor" OR "Syncope" OR "Consciousness Disorder" OR "Depressed Level of Consciousness" OR "Semiconsciousness" OR "Altered Level of Consciousness" OR "disorders of consciousness" OR “disorder of consciousness” OR DOC OR "Vegetative State" OR "unresponsive wakefulness syndrome" OR UWS OR "minimally consciousness state" OR MCS)

#2 TS=("Transcutaneous Electric Nerve Stimulation" OR "Transcranial Direct Current Stimulation" OR "Transcranial Magnetic Stimulation" OR "neuromodulation" OR "neuromodulation therapy" OR "neuromodulation techniques" OR "neuromodulatory techniques" OR "non-invasive brain" OR NIBS OR NIBI OR "transcranial electrical current stimulation" OR tECS OR "transcranial direct current stimulation" OR tDCS OR "transcranial alternating current stimulation" OR tACS or "transcranial magnetic stimulation" or TMS OR "repetitive transcranial magnetic stimulation" OR rTMS OR "theta burst stimulation" OR TBS OR cTBS OR iTBS OR "low-intensity focused ultrasound" OR LIFUS OR "transcutaneous auricular vagus nerve stimulation" OR taVNS or "Near-infrared laser stimulation" or N-LT OR “focused shock wave therapy” OR F-SWT OR "median nerve stimulation" OR MNS OR rMNS)

#3 1# AND 2#

**4. Searching Strategy for Scopus**

#1 TITLE-ABS-KEY ("Consciousness Disorders" OR "Unconsciousness" OR "Coma" OR "Persistent Vegetative State" OR "stupor" OR "Syncope" OR "Consciousness Disorder" OR "Depressed Level of Consciousness" OR "Semiconsciousness" OR "Altered Level of Consciousness" OR "disorders of consciousness" OR "disorder of consciousness" OR doc OR "Vegetative State" OR "unresponsive wakefulness syndrome" OR uws OR "minimally consciousness state" OR mcs )

#2 TITLE-ABS-KEY ( "Transcutaneous Electric Nerve Stimulation" OR "Transcranial Direct Current Stimulation" OR "Transcranial Magnetic Stimulation" OR "neuromodulation" OR "neuromodulation therapy" OR "neuromodulation techniques" OR "neuromodulatory techniques" OR "non-invasive brain" OR nibs OR nibi OR "transcranial electrical current stimulation" OR tecs OR "transcranial direct current stimulation" OR tdcs OR "transcranial alternating current stimulation" OR tacs OR "transcranial magnetic stimulation" OR tms OR "repetitive transcranial magnetic stimulation" OR rtms OR "theta burst stimulation" OR tbs OR ctbs OR itbs OR "low-intensity focused ultrasound" OR lifus OR "transcutaneous auricular vagus nerve stimulation" OR tavns OR "Near-infrared laser stimulation" OR n-lt OR "focused shock wave therapy" OR f-swt OR "median nerve stimulation" OR mns OR rmns )

#3 LIMIT-TO (DOCTYPE , "ar") OR LIMIT-TO (DOCTYPE , "cp")

#4 LIMIT-TO (EXACTKEYWORD , "Human")

#5 #1 AND #2 AND #3 AND #4

**5. Searching Strategy for Cochrane central register of controlled trials**

#1 MeSH descriptor: [Consciousness Disorders] explode all trees

#2 (Unconsciousness):ti,ab,kw OR (Coma):ti,ab,kw OR (Persistent Vegetative State):ti,ab,kw OR (stupor):ti,ab,kw OR (Syncope):ti,ab,kw OR (Consciousness Disorder):ti,ab,kw (Consciousness, Level Depressed):ti,ab,kw OR (Depressed Level of Consciousness):ti,ab,kw OR (Semiconsciousness):ti,ab,kw OR (Consciousness, Level Altered):ti,ab,kw OR (Altered Level of Consciousness):ti,ab,kw OR (“disorders of consciousness”):ti,ab,kw OR (“disorder of consciousness”):ti,ab,kw OR (DOC):ti,ab,kw OR (“Vegetative State”):ti,ab,kw OR (“unresponsive wakefulness syndrome”):ti,ab,kw OR (UWS):ti,ab,kw OR (“minimally consciousness state”):ti,ab,kw OR (MCS):ti,ab,kw

#3 #1 OR #2

#4 MeSH descriptor: [Transcutaneous Electric Nerve Stimulation] explode all trees OR MeSH descriptor: [Transcranial Direct Current Stimulation] explode all trees OR MeSH descriptor: [Transcranial Magnetic Stimulation] explode all trees

#5 (neuromodulation):ti,ab,kw OR ("neuromodulation therapy"):ti,ab,kw OR ("neuromodulation techniques"):ti,ab,kw OR (“neuromodulatory techniques”):ti,ab,kw OR ("non-invasive brain"):ti,ab,kw OR (NIBI):ti,ab,kw OR (NIBS):ti,ab,kw OR ("transcranial electrical current stimulation"):ti,ab,kw OR (tECS):ti,ab,kw OR ("transcranial direct current stimulation"):ti,ab,kw OR (tDCS):ti,ab,kw OR ("transcranial alternating current stimulation"):ti,ab,kw OR (tACS):ti,ab,kw OR ("transcranial magnetic stimulation"):ti,ab,kw OR (TMS):ti,ab,kw OR ("repetitive transcranial magnetic stimulation"):ti,ab,kw OR (rTMS):ti,ab,kw OR ("theta burst stimulation"):ti,ab,kw OR (TBS):ti,ab,kw OR (cTBS):ti,ab,kw OR (iTBS):ti,ab,kw OR ("low-intensity focused ultrasound"):ti,ab,kw OR (LIFUS):ti,ab,kw OR ("transcutaneous auricular vagus nerve stimulation"):ti,ab,kw OR (taVNS):ti,ab,kw OR ("Near-infrared laser stimulation"):ti,ab,kw OR (N-LT):ti,ab,kw OR (“focused shock wave therapy”):ti,ab,kw OR (F-SWT):ti,ab,kw OR ("median nerve stimulation"):ti,ab,kw OR (MNS):ti,ab,kw OR (rMNS):ti,ab,kw

#6 #4 OR #5

#7 #3 AND #6
