# Supplementary material for: Effectiveness on level of consciousness of non-invasive neuromodulation therapy in patients with disorders of consciousness: a systematic review and meta-analysis
Source: Front Hum Neurosci. 2023 May 24;17:1129254. doi: 10.3389/fnhum.2023.1129254 (PMC10246452; doi:10.3389/fnhum.2023.1129254)
Supplement: Supplementary file 2 [file Data_Sheet_2.pdf]

1. **Studies screened from five databases**

(1) **Pubmed (1060 studies)**

1. Hume AL, Cant BR, Shaw NA. Central somatosensory conduction time in comatose patients. Ann Neurol. 1979;5(4):379-84. Epub 1979/04/01. doi: 10.1002/ana.410050412. PubMed PMID: 443771.

2. Anziska BJ, Cracco RQ. Short latency somatosensory evoked potentials in brain dead patients. Arch Neurol. 1980;37(4):222-5. Epub 1980/04/01. doi: 10.1001/archneur.1980.00500530060009. PubMed PMID: 7362487.

3. Borucki Z. [Results of rheoencephalographic studies in chronic subdural hematoma]. Neurol Neurochir Pol. 1980;14(6):633-7. Epub 1980/01/01. PubMed PMID: 7242783.

4. Anziska BJ, Cracco RQ. Short latency SEPs to median nerve stimulation: comparison of recording methods and origin of components. Electroencephalogr Clin Neurophysiol. 1981;52(6):531-9. Epub 1981/12/01. doi: 10.1016/0013-4694(81)91428-0. PubMed PMID: 6172253.

5. Yoshida Y, Kouka M, Otose H, Hashiguchi K, Kamitsuchihasi H, Igata A. [A Lance-Adams syndrome of a diver (author's transl)]. No To Shinkei. 1981;33(10):1051-6. Epub 1981/10/01. PubMed PMID: 6797451.

6. Torres B, Gómez C, Delgado-García JM. [Effects of axotomy on the activity of the motor neurons of the external ocular motor nucleus during saccadic movements]. Rev Esp Fisiol. 1985;41(1):73-81. Epub 1985/03/01. PubMed PMID: 4001546.
7. Berić A, Dimitrijević MR, Prevec TS, Sherwood AM. Epidurally recorded cervical somatosensory evoked potential in humans. Electroencephalogr Clin Neurophysiol. 1986;65(2):94-101. Epub 1986/03/01. doi: 10.1016/0168-5597(86)90041-9. PubMed PMID: 2419108.
8. Shaw NA. The origin of the N20 component of the somatosensory evoked potential in man. Aust J Exp Biol Med Sci. 1986;64 (Pt 5):471-5. Epub 1986/10/01. doi: 10.1038/icb.1986.50. PubMed PMID: 3579741.
9. Takata M, Nagahama T. Cortically and lingually induced postsynaptic potentials in trigeminal motoneurons after axotomy. Exp Brain Res. 1986;61(2):272-9. Epub 1986/01/01. doi: 10.1007/bf00239517. PubMed PMID: 3005014.
10. Thomas JG. Computer generation of templates for evaluating the amplitude and latency parameters of two superimposed constituent components of early somatosensory evoked responses. J Neurosci Methods. 1986;15(4):329-34. Epub 1986/02/01. doi: 10.1016/0165-0270(86)90146-9. PubMed PMID: 3515056.

11. Ganji S, Peters G, Frazier E. Alpha-coma: clinical and evoked potential studies. Clin Electroencephalogr. 1987;18(3):103-13. Epub 1987/07/01. PubMed PMID: 3652461.

12. Jabbari B, Vance SC, Harper MG, Salazar AM, Smutok MA, Amin D. Clinical and radiological correlates of somatosensory evoked potentials in the late phase of head injury: a study of 500 Vietnam veterans. Electroencephalogr Clin Neurophysiol. 1987;67(4):289-97. Epub 1987/10/01. doi: 10.1016/0013-4694(87)90115-5. PubMed PMID: 2441962.

13. Sonnet ML, Floret D, Guillaume C, Fournet A, Motin J. [Evoked potentials in comatose children]. Pediatrie. 1987;42(1):45-50. Epub 1987/01/01. PubMed PMID: 3588178.

14. Stöhr M, Riffel B, Trost E, Ullrich A. Short-latency somatosensory evoked potentials in brain death. J Neurol. 1987;234(4):211-4. Epub 1987/05/01. doi: 10.1007/bf00618252. PubMed PMID: 3612191.

15. Stöhr M, Riffel B, Trost E, Wengert P. [Significance of somatosensory evoked potentials in determining brain death]. Anasth Intensivther Notfallmed. 1987;22(1):21-5. Epub 1987/02/01. PubMed PMID: 3578718.

16. Takahashi H, Yasue M, Ishijima B. [Changes in short latency SEPs (S-SEPs) to median nerve stimulation in brain dead patients].

No To Shinkei. 1987;39(6):543-9. Epub 1987/06/01. PubMed PMID: 3620222.
17. Buchner H, Ferbert A, Hacke W. Serial recording of median nerve stimulated subcortical somatosensory evoked potentials (SEPs) in developing brain death. Electroencephalogr Clin Neurophysiol. 1988;69(1):14-23. Epub 1988/01/01. doi: 10.1016/0013-4694(88)90031-4. PubMed PMID: 2448117.
18. Moryś J, Słoniewski P, Narkiewicz O. Somatosensory evoked potentials following lesions of the claustrum. Acta Physiol Pol. 1988;39(5-6):475-83. Epub 1988/09/01. PubMed PMID: 3257060.
19. Nakamura Y, Toya S, Nakatsukasa M, Ibata Y, Tamura K, Takase M. [A case of alpha coma in acute brainstem dysfunction--consecutive electroencephalograms and evoked potentials]. No To Shinkei. 1988;40(8):753-8. Epub 1988/08/01. PubMed PMID: 3179089.
20. Rumpl E, Prugger M, Battista HJ, Badry F, Gerstenbrand F, Dienstl F. Short latency somatosensory evoked potentials and brain-stem auditory evoked potentials in coma due to CNS depressant drug poisoning. Preliminary observations. Electroencephalogr Clin Neurophysiol. 1988;70(6):482-9. Epub 1988/12/01. doi: 10.1016/0013-4694(88)90146-0. PubMed PMID: 2461282.

21. Rumpl E, Prugger M, Gerstenbrand F, Brunhuber W, Badry F, Hackl JM. Central somatosensory conduction time and acoustic brainstem transmission time in post-traumatic coma. J Clin Neurophysiol. 1988;5(3):237-60. Epub 1988/07/01. doi: 10.1097/00004691-198807000-00002. PubMed PMID: 2844849.
22. Schriefer TN, Mills KR, Murray NM, Hess CW. Evaluation of proximal facial nerve conduction by transcranial magnetic stimulation. J Neurol Neurosurg Psychiatry. 1988;51(1):60-6. Epub 1988/01/01. doi: 10.1136/jnnp.51.1.60. PubMed PMID: 3351531; PubMed Central PMCID: PMCPMC1032714.
23. Urasaki E, Fukumura A, Itho Y, Itoyama Y, Yamada M, Ushio Y, et al. [Lazarus' sign and respiratory-like movement in a patient with brain death]. No To Shinkei. 1988;40(12):1111-6. Epub 1988/12/01. PubMed PMID: 3248188.
24. Reilly RP, Findley TW. Research in physical medicine and rehabilitation. IV. Some practical designs in applied research. Am J Phys Med Rehabil. 1989;68(4):196-201. Epub 1989/08/01. doi: 10.1097/00002060-198908000-00009. PubMed PMID: 2788436.
25. Riffel B, Stöhr M, Graser W, Trost E, Baumgärtner H. [Early prognosis in severe cranio-cerebral trauma using the Glasgow Coma Score and evoked potentials]. Anaesthesist. 1989;38(2):51-8. Epub 1989/02/01. PubMed PMID: 2929965.

26. Unger WG. Mediation of the ocular response to injury and irritation: peptides versus prostaglandins. Prog Clin Biol Res. 1989;312:293-328. Epub 1989/01/01. PubMed PMID: 2678145.
27. Facco E, Casartelli Liviero M, Munari M, Toffoletto F, Baratto F, Giron GP. Short latency evoked potentials: new criteria for brain death? J Neurol Neurosurg Psychiatry. 1990;53(4):351-3. Epub 1990/04/01. doi: 10.1136/jnnp.53.4.351. PubMed PMID: 2341851; PubMed Central PMCID: PMCPMC1014178.
28. Gacek RR. Arachnoid granulation cerebrospinal fluid otorrhea. Ann Otol Rhinol Laryngol. 1990;99(11):854-62. Epub 1990/11/01. doi: 10.1177/000348949009901102. PubMed PMID: 2241009.
29. Heydenreich A. [Hypothetical aspects of the preventive effectiveness of controlled reflex and stimulation therapy (exemplified by acupuncture and PuTENS) in migraine]. Psychiatr Neurol Med Psychol (Leipz). 1990;42(8):500-7. Epub 1990/08/01. PubMed PMID: 2247531.
30. Zeitlhofer J, Mayr N, Auff E. [The value of evoked potentials in brain death diagnosis]. Wien Med Wochenschr. 1990;140(23-24):564-7. Epub 1990/01/01. PubMed PMID: 2085011.
31. Facco E, Munari M, Donà B, Baratto F, Fiore D, Behr AU, et al. Spatial mapping of SEP in comatose patients: improved

outcome prediction by combined parietal N20 and frontal N30 analysis. Brain Topogr. 1991;3(4):447-55. Epub 1991/01/01. doi: 10.1007/bf01129004. PubMed PMID: 1742161.

32. Grimm G, Madl C, Oder W, Druml W, Schneeweiss B, Laggner AN, et al. Evoked potentials in severe herpes simplex encephalitis. Intensive Care Med. 1991;17(2):94-7. Epub 1991/01/01. doi: 10.1007/bf01691430. PubMed PMID: 1865043.

33. Jouvet M, Albarede JL, Lubin S, Meyrignac C. [Noradrenaline and cerebral aging]. Encephale. 1991;17(3):187-95. Epub 1991/05/01. PubMed PMID: 1864252.

34. Liska A, Kazibutowska Z, Rogoz B, Pierzchała K, Rusiecki Z, Totuszyński M. [Multimodal evoked potentials in the early period after cranio-cerebral trauma]. Neurol Neurochir Pol. 1991;25(5):647-55. Epub 1991/09/01. PubMed PMID: 1808527.

35. Werba A, Weinstabl C, Petricek W, Plainer B, Spiss CK. [Requisite muscle relaxation using vecuronium for tracheobronchial suction in neurosurgical intensive care patients]. Anaesthesist. 1991;40(6):328-31. Epub 1991/06/01. PubMed PMID: 1679303.

36. Zhou D. [Comparison of the effects of 4 psychiatric treatments on rat brain beta 5HT2 receptors]. Zhonghua Shen Jing Jing Shen Ke Za Zhi. 1991;24(4):207-9, 51-2. Epub 1991/08/01. PubMed

PMID: 1659512.
37. Cusumano S, Paolin A, Di Paola F, Boccaletto F, Simini G, Palermo F, et al. Assessing brain function in post-traumatic coma by means of bit-mapped SEPs, BAEPs, CT, SPET and clinical scores. Prognostic implications. Electroencephalogr Clin Neurophysiol. 1992;84(6):499-514. Epub 1992/11/01. doi: 10.1016/0168-5597(92)90039-e. PubMed PMID: 1280196.
38. Jaspert A, Kotterba S, Tegenthoff M, Malin JP. [Transcranial magnetic stimulation in the cranial nerve region in cranio-cerebral injuries]. EEG EMG Z Elektroenzephalogr Elektromyogr Verwandte Geb. 1992;23(1):37-42. Epub 1992/03/01. PubMed PMID: 1587232.
39. Liepert J, Tegenthoff M. [Transcranial magnetic stimulation of patients with a single epileptic seizure]. Nervenarzt. 1992;63(8):492-4. Epub 1992/08/01. PubMed PMID: 1522935.
40. Netz J, Hömberg V. Intact conduction of fastest corticospinal efferents is not sufficient for normal voluntary muscle strength: transcranial motor cortex stimulation in patients with tetraplegia. Neurosci Lett. 1992;146(1):29-32. Epub 1992/10/26. doi: 10.1016/0304-3940(92)90164-3. PubMed PMID: 1475046.
41. Shutov AA, Sherman MA. [A psychoautonomic syndrome in the residual period of a modern "mild" military craniocerebral

trauma]. Zh Nevropatol Psikhiatr Im S S Korsakova. 1992;92(5-12):13-5. Epub 1992/01/01. PubMed PMID: 1338144.

42. Urasaki E, Tokimura T, Kumai J, Wada S, Yokota A. Preserved spinal dorsal horn potentials in a brain-dead patient with Lazarus' sign. Case report. J Neurosurg. 1992;76(4):710-3. Epub 1992/04/01. doi: 10.3171/jns.1992.76.4.0710. PubMed PMID: 1545268.

43. Ying Z, Schmid UD, Schmid J, Hess CW. Motor and somatosensory evoked potentials in coma: analysis and relation to clinical status and outcome. J Neurol Neurosurg Psychiatry. 1992;55(6):470-4. Epub 1992/06/01. doi: 10.1136/jnnp.55.6.470. PubMed PMID: 1619414; PubMed Central PMCID: PMCPMC1014903.

44. Bach-y-Rita P. Nonsynaptic diffusion neurotransmission (NDN) in the brain. Neurochem Int. 1993;23(4):297-318. Epub 1993/10/01. doi: 10.1016/0197-0186(93)90074-f. PubMed PMID: 8220172.

45. Devinsky O. Electrical and magnetic stimulation of the central nervous system. Historical overview. Adv Neurol. 1993;63:1-16. Epub 1993/01/01. PubMed PMID: 8279294.

46. Kapuściński A. Effect of clinical death on inositol 1,4,5-trisphosphate in the rat brain. Neuropatol Pol. 1993;31(1-2):91-6. Epub 1993/01/01. PubMed PMID: 8208444.

47. Kiers L, Cros D, Chiappa KH, Fang J. Variability of motor potentials evoked by transcranial magnetic stimulation. Electroencephalogr Clin Neurophysiol. 1993;89(6):415-23. Epub 1993/12/01. doi: 10.1016/0168-5597(93)90115-6. PubMed PMID: 7507428.

48. Kotterba S, Tegenthoff M, Malin JP. [Transcranial magnetic stimulation in perioperative damage to the facial nerve]. Laryngorhinootologie. 1993;72(1):32-5. Epub 1993/01/01. doi: 10.1055/s-2007-997849. PubMed PMID: 8439354.

49. Champagnat J, Richter DW. The roles of K+ conductance in expiratory pattern generation in anaesthetized cats. J Physiol. 1994;479 (Pt 1)(Pt 1):127-38. Epub 1994/08/15. doi: 10.1113/jphysiol.1994.sp020282. PubMed PMID: 7990029; PubMed Central PMCID: PMCPMC1155730.

50. Kong WJ, Egg G, Hussl B, Seyr M, Schrott-Fischer A. A study of neurotransmitters in human inner ear. Preservation of human temporal bone and value of organ donation for inner ear research. Acta Otolaryngol. 1994;114(3):245-53. Epub 1994/05/01. doi: 10.3109/00016489409126051. PubMed PMID: 7915484.

51. Mazziotta JC. Mapping human brain activity in vivo. West J Med. 1994;161(3):273-8. Epub 1994/09/01. PubMed PMID: 7975566; PubMed Central PMCID: PMCPMC1011409.

52. Kaneko M. [Prognostic evaluation of patients with severe head injury by motor evoked potentials induced by transcranial magnetic stimulation--combined analysis with brainstem auditory evoked potentials]. No To Shinkei. 1995;47(5):491-6. Epub 1995/05/01. PubMed PMID: 7786626.

53. Mesejo A, Núñez C, Simó M, Blanquer J, Pérez PL, Ruiz F, et al. [Neuroleptic malignant syndrome, multiorgan failure and peripheral polyneuropathy]. Rev Neurol. 1995;23(119):136-8. Epub 1995/01/01. PubMed PMID: 8548608.

54. Schrader M, Schrader V. [Reliability of magnetic stimulation in the diagnosis of peripheral facial paralysis of idiopathic origin]. Rev Laryngol Otol Rhinol (Bord). 1995;116(2):123-7. Epub 1995/01/01. PubMed PMID: 7569373.

55. Sader R, Meyer BU, Horch HH, Deppe H, Zeilhofer HF, Herzog M, et al. [Neurologic examinations for facial nerve damage in surgically treated mandibular collum fractures]. Fortschr Kiefer Gesichtschir. 1996;41:143-5. Epub 1996/01/01. PubMed PMID: 8755427.

56. Wagner W. Scalp, earlobe and nasopharyngeal recordings of the median nerve somatosensory evoked P14 potential in coma and brain death. Detailed latency and amplitude analysis in 181 patients. Brain. 1996;119 (Pt 5):1507-21. Epub 1996/10/01. doi:

10.1093/brain/119.5.1507. PubMed PMID: 8931576.

57. Heinemann F, Assion HJ, Hermes G, Ehrlich M. [Paroxetine-induced neuroleptic malignant syndrome]. Nervenarzt. 1997;68(8):664-6. Epub 1997/08/01. doi: 10.1007/s001150050178. PubMed PMID: 9380213.

58. Kotterba S, Tegenthoff M, Malin JP. Perioperative lesions of the facial nerve: follow-up investigations using transcranial magnetic stimulation. Eur Arch Otorhinolaryngol. 1997;254(3):140-4. Epub 1997/01/01. doi: 10.1007/bf02471278. PubMed PMID: 9112034.

59. Maegaki Y, Maeoka Y, Ishii S, Shiota M, Takeuchi A, Yoshino K, et al. Mechanisms of central motor reorganization in pediatric hemiplegic patients. Neuropediatrics. 1997;28(3):168-74. Epub 1997/06/01. doi: 10.1055/s-2007-973695. PubMed PMID: 9266555.

60. Msikita W, Yaninek JS, Ahounou M, Baimey H, Fagbemissi R. First Report of Nattrassia mangiferae Root and Stem Rot of Cassava in West Africa. Plant Dis. 1997;81(11):1332. Epub 1997/11/01. doi: 10.1094/pdis.1997.81.11.1332a. PubMed PMID: 30861748.

61. Orlando LR, Luthi-Carter R, Standaert DG, Coyle JT, Penney JB, Jr., Young AB. N-acetylaspartylglutamate (NAAG) protects

against rat striatal quinolinic acid lesions in vivo. Neurosci Lett. 1997;236(2):91-4. Epub 1997/12/24. doi: 10.1016/s0304-3940(97)00769-6. PubMed PMID: 9404819.

62. Raungratanaamporn O, Bhuripanyo K, Krittayaphong R, Aroonpruksakul S, Toomtong P, Chaithiraphan S. Radiofrequency catheter ablation in type I atrial flutter: preliminary experience of 10 cases. J Med Assoc Thai. 1997;80(7):466-72. Epub 1997/07/01. PubMed PMID: 9277077.

63. Taub E, Munz M, Tasker RR. Chronic electrical stimulation of the gasserian ganglion for the relief of pain in a series of 34 patients. J Neurosurg. 1997;86(2):197-202. Epub 1997/02/01. doi: 10.3171/jns.1997.86.2.0197. PubMed PMID: 9010419.

64. Vargo CP, Hickman DM. Cluster-like signs and symptoms respond to myofascial/craniomandibular treatment: a report of two cases. Cranio. 1997;15(1):89-93. Epub 1997/01/01. doi: 10.1080/08869634.1997.11745997. PubMed PMID: 9586493.

65. Butler JD, Miles J. Dysaesthetic neck pain with syncope. Pain. 1998;75(2-3):395-7. Epub 1998/05/16. doi: 10.1016/s0304-3959(97)00195-4. PubMed PMID: 9583777.

66. Chistyakov AV, Soustiel JF, Hafner H, Elron M, Feinsod M. Altered excitability of the motor cortex after minor head injury revealed by transcranial magnetic stimulation. Acta Neurochir

(Wien). 1998;140(5):467-72. Epub 1998/09/05. doi: 10.1007/s007010050126. PubMed PMID: 9728247.

67. Classen J, Knorr U, Werhahn KJ, Schlaug G, Kunesch E, Cohen LG, et al. Multimodal output mapping of human central motor representation on different spatial scales. J Physiol. 1998;512 (Pt 1)(Pt 1):163-79. Epub 1998/09/08. doi: 10.1111/j.1469-7793.1998.163bf.x. PubMed PMID: 9729626; PubMed Central PMCID: PMCPMC2231178.

68. McMillan AS, Watson C, Walshaw D. Transcranial magnetic-stimulation mapping of the cortical topography of the human masseter muscle. Arch Oral Biol. 1998;43(12):925-31. Epub 1999/01/07. doi: 10.1016/s0003-9969(98)00081-8. PubMed PMID: 9877323.

69. Chistyakov AV, Hafner H, Soustiel JF, Trubnik M, Levy G, Feinsod M. Dissociation of somatosensory and motor evoked potentials in non-comatose patients after head injury. Clin Neurophysiol. 1999;110(6):1080-9. Epub 1999/07/13. doi: 10.1016/s1388-2457(99)00029-2. PubMed PMID: 10402095.

70. Elron M, Levi G, Trovnick M, Soustiel JF, Hafner H, Chistyakov A, et al. [Exhaustion of motor cortex after head injury--revealed by transcranial magnetic stimulation]. Harefuah. 1999;136(5):355-8, 419. Epub 2000/07/29. PubMed PMID:

10914237.

71. Ilmoniemi RJ, Ruohonen J, Karhu J. Transcranial magnetic stimulation--a new tool for functional imaging of the brain. Crit Rev Biomed Eng. 1999;27(3-5):241-84. Epub 2000/06/23. PubMed PMID: 10864281.

72. Kobayashi M, Ohira T, Ochiai M, Mihara B, Kawase T. Transcranial magnetic stimulation of the central and peripheral motor pathways to the lingual muscles in cats. Clin Neurophysiol. 1999;110(3):445-8. Epub 1999/06/11. doi: 10.1016/s1388-2457(98)00015-7. PubMed PMID: 10363768.

73. Koscielniak-Nielsen ZJ, Nielsen PR, Nielsen SL, Gardi T, Hermann C. Comparison of transarterial and multiple nerve stimulation techniques for axillary block using a high dose of mepivacaine with adrenaline. Acta Anaesthesiol Scand. 1999;43(4):398-404. Epub 1999/05/04. doi: 10.1034/j.1399-6576.1999.430406.x. PubMed PMID: 10225072.

74. Moosavi SH, Ellaway PH, Catley M, Stokes MJ, Haque N. Corticospinal function in severe brain injury assessed using magnetic stimulation of the motor cortex in man. J Neurol Sci. 1999;164(2):179-86. Epub 1999/07/13. doi: 10.1016/s0022-510x(99)00065-9. PubMed PMID: 10402031.

75. Palmieri MG, Iani C, Scalise A, Desiato MT, Loberti M,

Telera S, et al. The effect of benzodiazepines and flumazenil on motor cortical excitability in the human brain. Brain Res. 1999;815(2):192-9. Epub 1999/01/08. doi: 10.1016/s0006-8993(98)01164-0. PubMed PMID: 9878733.
76. Pascual-Leone A, Bartres-Faz D, Keenan JP. Transcranial magnetic stimulation: studying the brain-behaviour relationship by induction of 'virtual lesions'. Philos Trans R Soc Lond B Biol Sci. 1999;354(1387):1229-38. Epub 1999/08/31. doi: 10.1098/rstb.1999.0476. PubMed PMID: 10466148; PubMed Central PMCID: PMCPMC1692644.
77. Steenerson RL, Cronin GW. Treatment of tinnitus with electrical stimulation. Otolaryngol Head Neck Surg. 1999;121(5):511-3. Epub 1999/11/05. doi: 10.1016/s0194-5998(99)70048-3. PubMed PMID: 10547461.
78. Taub E, Uswatte G, Pidikiti R. Constraint-Induced Movement Therapy: a new family of techniques with broad application to physical rehabilitation--a clinical review. J Rehabil Res Dev. 1999;36(3):237-51. Epub 2000/02/05. PubMed PMID: 10659807.
79. Terao Y, Ugawa Y, Hanajima R, Furubayashi T, Machii K, Enomoto H, et al. Air-puff-induced facilitation of motor cortical excitability studied in patients with discrete brain lesions. Brain. 1999;122 (Pt 12):2259-77. Epub 1999/12/03. doi:

10.1093/brain/122.12.2259. PubMed PMID: 10581221.

80. Zanetta JP, Timmerman P, Leroy Y. Gas-liquid chromatography of the heptafluorobutyrate derivatives of the O-methyl-glycosides on capillary columns: a method for the quantitative determination of the monosaccharide composition of glycoproteins and glycolipids. Glycobiology. 1999;9(3):255-66. Epub 1999/02/20. doi: 10.1093/glycob/9.3.255. PubMed PMID: 10024663.

81. Couper FJ, Logan BK. Determination of gamma-hydroxybutyrate (GHB) in biological specimens by gas chromatography--mass spectrometry. J Anal Toxicol. 2000;24(1):1-7. Epub 2000/02/02. doi: 10.1093/jat/24.1.1. PubMed PMID: 10654561.

82. Cowey A, Walsh V. Magnetically induced phosphenes in sighted, blind and blindsighted observers. Neuroreport. 2000;11(14):3269-73. Epub 2000/10/24. doi: 10.1097/00001756-200009280-00044. PubMed PMID: 11043562.

83. Fins JJ. A proposed ethical framework for interventional cognitive neuroscience: a consideration of deep brain stimulation in impaired consciousness. Neurol Res. 2000;22(3):273-8. Epub 2000/04/19. doi: 10.1080/01616412.2000.11740670. PubMed PMID: 10769820.

84. García-Larrea L, Peyron R, Mertens P, Laurent B, Mauguière F, Sindou M. Functional imaging and neurophysiological assessment of spinal and brain therapeutic modulation in humans. Arch Med Res. 2000;31(3):248-57. Epub 2000/10/19. doi: 10.1016/s0188-4409(00)00083-7. PubMed PMID: 11036174.

85. Gómez-Fernández L. [Cortical plasticity and restoration of neurologic functions: an update on this topic]. Rev Neurol. 2000;31(8):749-56. Epub 2000/11/18. PubMed PMID: 11082885.

86. Har-El G, McPhee JR. Transcranial magnetic stimulation in acute facial nerve injury. Laryngoscope. 2000;110(7):1105-11. Epub 2000/07/13. doi: 10.1097/00005537-200007000-00007. PubMed PMID: 10892678.

87. Kudoh A, Matsuki A. Current perception thresholds of epileptic patients treated with valproate. Seizure. 2000;9(7):498-501. Epub 2000/10/18. doi: 10.1053/seiz.2000.0449. PubMed PMID: 11034875.

88. Oliveri M, Rossini PM, Filippi MM, Traversa R, Cicinelli P, Palmieri MG, et al. Time-dependent activation of parieto-frontal networks for directing attention to tactile space. A study with paired transcranial magnetic stimulation pulses in right-brain-damaged patients with extinction. Brain. 2000;123 (Pt 9):1939-47. Epub 2000/08/26. doi: 10.1093/brain/123.9.1939.

PubMed PMID: 10960057.
89. Restuccia D. Anatomic origin of P13 and P14 scalp far-field potentials. J Clin Neurophysiol. 2000;17(3):246-57. Epub 2000/08/06. doi: 10.1097/00004691-200005000-00003. PubMed PMID: 10928637.
90. Schiff ND, Rezai AR, Plum F. A neuromodulation strategy for rational therapy of complex brain injury states. Neurol Res. 2000;22(3):267-72. Epub 2000/04/19. doi: 10.1080/01616412.2000.11740669. PubMed PMID: 10769819.
91. Sharma JC, Fletcher S, Vassallo M, Ross I. Prognostic value of CT scan features in acute ischaemic stroke and relationship with clinical stroke syndromes. Int J Clin Pract. 2000;54(8):514-8. Epub 2001/02/24. PubMed PMID: 11198729.
92. Sindou M, Mertens P. Neurosurgical management of neuropathic pain. Stereotact Funct Neurosurg. 2000;75(2-3):76-80. Epub 2001/12/12. doi: 10.1159/000048386. PubMed PMID: 11740173.
93. Uclés P, Serrano JL, Rosa F. Central conduction time of magnetic brain stimulation in attention-deficit hyperactivity disorder. J Child Neurol. 2000;15(11):723-8. Epub 2000/12/07. doi: 10.1177/088307380001501103. PubMed PMID: 11108505.
94. Abo M, Chen Z, Lai LJ, Reese T, Bjelke B. Functional

recovery after brain lesion--contralateral neuromodulation: an fMRI study. Neuroreport. 2001;12(7):1543-7. Epub 2001/06/05. doi: 10.1097/00001756-200105250-00048. PubMed PMID: 11388445.
95. Blume WT. Current trends in electroencephalography. Curr Opin Neurol. 2001;14(2):193-7. Epub 2001/03/23. doi: 10.1097/00019052-200104000-00010. PubMed PMID: 11262735.
96. Chistyakov AV, Soustiel JF, Hafner H, Trubnik M, Levy G, Feinsod M. Excitatory and inhibitory corticospinal responses to transcranial magnetic stimulation in patients with minor to moderate head injury. J Neurol Neurosurg Psychiatry. 2001;70(5):580-7. Epub 2001/04/20. doi: 10.1136/jnnp.70.5.580. PubMed PMID: 11309450; PubMed Central PMCID: PMCPMC1737339.
97. Hill NM, Sleigh JW. Transient epidermal flushing in a head injured patient: a case report. Crit Care Resusc. 2001;3(3):170-2. Epub 2006/04/01. PubMed PMID: 16573498.
98. Iwasaki M, Nakasato N, Kanno A, Hatanaka K, Nagamatsu K, Nagamine Y, et al. Somatosensory evoked fields in comatose survivors after severe traumatic brain injury. Clin Neurophysiol. 2001;112(1):205-11. Epub 2001/01/04. doi: 10.1016/s1388-2457(00)00506-x. PubMed PMID: 11137679.

99. Mogilner AY, Rezai AR. Epidural motor cortex stimulation with functional imaging guidance. Neurosurg Focus. 2001;11(3):E4. Epub 2006/03/08. doi: 10.3171/foc.2001.11.3.5. PubMed PMID: 16519424.

100. Moritz CH, Rowley HA, Haughton VM, Swartz KR, Jones J, Badie B. Functional MR imaging assessment of a non-responsive brain injured patient. Magn Reson Imaging. 2001;19(8):1129-32. Epub 2001/11/17. doi: 10.1016/s0730-725x(01)00432-5. PubMed PMID: 11711238.

101. Peri CV, Shaffrey ME, Farace E, Cooper E, Alves WM, Cooper JB, et al. Pilot study of electrical stimulation on median nerve in comatose severe brain injured patients: 3-month outcome. Brain Inj. 2001;15(10):903-10. Epub 2001/10/12. doi: 10.1080/02699050110065709. PubMed PMID: 11595086.

102. Plewnia C, Bartels M, Cohen L, Gerloff C. Noradrenergic modulation of human cortex excitability by the presynaptic alpha(2)-antagonist yohimbine. Neurosci Lett. 2001;307(1):41-4. Epub 2001/08/23. doi: 10.1016/s0304-3940(01)01921-8. PubMed PMID: 11516570.

103. Bevilaqua-Grosso D, Monteiro-Pedro V, Guirro RR, Bérzin F. A physiotherapeutic approach to craniomandibular disorders: a case report. J Oral Rehabil. 2002;29(3):268-73. Epub 2002/03/19.

doi: 10.1046/j.1365-2842.2002.00832.x. PubMed PMID: 11896844.

104. Bütefisch CM, Davis BC, Sawaki L, Waldvogel D, Classen J, Kopylev L, et al. Modulation of use-dependent plasticity by d-amphetamine. Ann Neurol. 2002;51(1):59-68. Epub 2002/01/10. doi: 10.1002/ana.10056. PubMed PMID: 11782985.

105. Carkeet A, Luo HD, Tong L, Saw SM, Tan DT. Refractive error and monochromatic aberrations in Singaporean children. Vision Res. 2002;42(14):1809-24. Epub 2002/07/20. doi: 10.1016/s0042-6989(02)00114-1. PubMed PMID: 12127112.

106. Fraser C, Power M, Hamdy S, Rothwell J, Hobday D, Hollander I, et al. Driving plasticity in human adult motor cortex is associated with improved motor function after brain injury. Neuron. 2002;34(5):831-40. Epub 2002/06/14. doi: 10.1016/s0896-6273(02)00705-5. PubMed PMID: 12062028.

107. Kahveci F, Bekar A, Tamgac F. Tc-99 HMPAO cerebral SPECT imaging in brain death patients with complex spinal automatism. Ulus Travma Derg. 2002;8(4):198-201. Epub 2002/11/05. PubMed PMID: 12415498.

108. Krames E. Implantable devices for pain control: spinal cord stimulation and intrathecal therapies. Best Pract Res Clin Anaesthesiol. 2002;16(4):619-49. Epub 2003/01/09. doi:

10.1053/bean.2002.0263. PubMed PMID: 12516895.

109. Oliveri M, Rossini PM, Filippi MM, Traversa R, Cicinelli P, Caltagirone C. Specific forms of neural activity associated with tactile space awareness. Neuroreport. 2002;13(8):997-1001. Epub 2002/06/13. doi: 10.1097/00001756-200206120-00002. PubMed PMID: 12060795.

110. Piroth MD, Zierhut D, van Kampen M, Sroka-Perez G, Wannenmacher M. [Lung dose depending on exact patient positioning during total body irradiation (TBI). Isoeffectiveness considerations to assess the risk of interstitial pneumonia after TBI]. Strahlenther Onkol. 2002;178(1):43-9. Epub 2002/04/30. doi: 10.1007/s00066-002-0876-0. PubMed PMID: 11977391.

111. Schiff ND, Plum F, Rezai AR. Developing prosthetics to treat cognitive disabilities resulting from acquired brain injuries. Neurol Res. 2002;24(2):116-24. Epub 2002/03/07. doi: 10.1179/016164102101199576. PubMed PMID: 11877893.

112. Staudt M, Grodd W, Gerloff C, Erb M, Stitz J, Krägeloh-Mann I. Two types of ipsilateral reorganization in congenital hemiparesis: a TMS and fMRI study. Brain. 2002;125(Pt 10):2222-37. Epub 2002/09/24. doi: 10.1093/brain/awf227. PubMed PMID: 12244080.

113. van Dijk KR, Scherder EJ, Scheltens P, Sergeant JA. Effects

of transcutaneous electrical nerve stimulation (TENS) on non-pain related cognitive and behavioural functioning. Rev Neurosci. 2002;13(3):257-70. Epub 2002/10/31. doi: 10.1515/revneuro.2002.13.3.257. PubMed PMID: 12405228.

114. Akamatsu N, Tsuji S. [Current diagnosis and treatment of status epilepticus]. Rinsho Shinkeigaku. 2003;43(11):799-801. Epub 2004/05/22. PubMed PMID: 15152468.

115. Cooper EB, Cooper JB. Electrical treatment of coma via the median nerve. Acta Neurochir Suppl. 2003;87:7-10. Epub 2003/10/02. doi: 10.1007/978-3-7091-6081-7_2. PubMed PMID: 14518514.

116. Fagade OO, Obilade TO. Therapeutic effect of TENS on post-IMF trismus and pain. Afr J Med Med Sci. 2003;32(4):391-4. Epub 2004/07/21. PubMed PMID: 15259924.

117. Fins JJ. From psychosurgery to neuromodulation and palliation: history's lessons for the ethical conduct and regulation of neuropsychiatric research. Neurosurg Clin N Am. 2003;14(2):303-19, ix-x. Epub 2003/07/15. doi: 10.1016/s1042-3680(02)00118-3. PubMed PMID: 12856496.

118. Ishiyama Y. [Current neurophysiological tests and revised JSCN technical standards for clinical EEG]. Rinsho Byori. 2003;51(6):562-7. Epub 2003/07/30. PubMed PMID: 12884744.

119. Liu JT, Wang CH, Chou IC, Sun SS, Koa CH, Cooper E. Regaining consciousness for prolonged comatose patients with right median nerve stimulation. Acta Neurochir Suppl. 2003;87:11-4. Epub 2003/10/02. doi: 10.1007/978-3-7091-6081-7_3. PubMed PMID: 14518515.
120. Ragauskas A, Daubaris G, Ragaisis V, Petkus V. Implementation of non-invasive brain physiological monitoring concepts. Med Eng Phys. 2003;25(8):667-78. Epub 2003/08/06. doi: 10.1016/s1350-4533(03)00082-1. PubMed PMID: 12900182.
121. Taub E, Uswatte G, Morris DM. Improved motor recovery after stroke and massive cortical reorganization following Constraint-Induced Movement therapy. Phys Med Rehabil Clin N Am. 2003;14(1 Suppl):S77-91, ix. Epub 2003/03/11. doi: 10.1016/s1047-9651(02)00052-9. PubMed PMID: 12625639.
122. Théoret H, Kobayashi M, Valero-Cabré A, Pascual-Leone A. Exploring paradoxical functional facilitation with TMS. Suppl Clin Neurophysiol. 2003;56:211-9. Epub 2003/12/18. doi: 10.1016/s1567-424x(09)70224-7. PubMed PMID: 14677397.
123. Urban PP. Transcranial magnetic stimulation in brainstem lesions and lesions of the cranial nerves. Suppl Clin Neurophysiol. 2003;56:341-57. Epub 2003/12/18. doi: 10.1016/s1567-424x(09)70238-7. PubMed PMID: 14677411.

124. Wedekind C, Klug N. Facial F wave recording: a novel and effective technique for extra- and intraoperative diagnosis of facial nerve function in acoustic tumor disease. Otolaryngol Head Neck Surg. 2003;129(1):114-20. Epub 2003/07/19. doi: 10.1016/s0194-5998(03)00475-3. PubMed PMID: 12869926.
125. Breitmeyer BG, Ro T, Ogmen H. A comparison of masking by visual and transcranial magnetic stimulation: implications for the study of conscious and unconscious visual processing. Conscious Cogn. 2004;13(4):829-43. Epub 2004/11/04. doi: 10.1016/j.concog.2004.08.007. PubMed PMID: 15522634.
126. Dobkin BH. Neurobiology of rehabilitation. Ann N Y Acad Sci. 2004;1038:148-70. Epub 2005/04/20. doi: 10.1196/annals.1315.024. PubMed PMID: 15838110; PubMed Central PMCID: PMCPMC4164200.
127. Jääskeläinen SK. The utility of clinical neurophysiological and quantitative sensory testing for trigeminal neuropathy. J Orofac Pain. 2004;18(4):355-9. Epub 2005/01/08. PubMed PMID: 15636020.
128. Lenaerts ME, Couch JR, Couch JR. Posttraumatic Headache. Curr Treat Options Neurol. 2004;6(6):507-17. Epub 2004/10/06. doi: 10.1007/s11940-004-0008-5. PubMed PMID: 15461928.
129. Martelletti P, van Suijlekom H. Cervicogenic headache:

practical approaches to therapy. CNS Drugs. 2004;18(12):793-805. Epub 2004/09/21. doi: 10.2165/00023210-200418120-00004. PubMed PMID: 15377169.

130. Nandi D, Aziz TZ. Deep brain stimulation in the management of neuropathic pain and multiple sclerosis tremor. J Clin Neurophysiol. 2004;21(1):31-9. Epub 2004/04/21. doi: 10.1097/00004691-200401000-00005. PubMed PMID: 15097292.

131. Ro T, Shelton D, Lee OL, Chang E. Extrageniculate mediation of unconscious vision in transcranial magnetic stimulation-induced blindsight. Proc Natl Acad Sci U S A. 2004;101(26):9933-5. Epub 2004/06/24. doi: 10.1073/pnas.0403061101. PubMed PMID: 15210947; PubMed Central PMCID: PMCPMC470776.

132. Suleiman Y, Amm M, Duncker GI, Nölle B. [Prognosis of corneal transplantation after penetrating eye injury]. Klin Monbl Augenheilkd. 2004;221(8):658-73. Epub 2004/09/03. doi: 10.1055/s-2004-813536. PubMed PMID: 15343450.

133. Wagner T, Gangitano M, Romero R, Théoret H, Kobayashi M, Anschel D, et al. Intracranial measurement of current densities induced by transcranial magnetic stimulation in the human brain. Neurosci Lett. 2004;354(2):91-4. Epub 2003/12/31. doi: 10.1016/s0304-3940(03)00861-9. PubMed PMID: 14698446.

134. Yamashita M, Horikoshi S, Asanuma K, Takahara H, Shirato I,

Tomino Y. Tensin is potentially involved in extracellular matrix production in mesangial cells. Histochem Cell Biol. 2004;121(3):245-54. Epub 2004/03/03. doi: 10.1007/s00418-004-0626-8. PubMed PMID: 14991332.

135. Zhao Z, Alam S, Oppenheim RW, Prevette DM, Evenson A, Parsadanian A. Overexpression of glial cell line-derived neurotrophic factor in the CNS rescues motoneurons from programmed cell death and promotes their long-term survival following axotomy. Exp Neurol. 2004;190(2):356-72. Epub 2004/11/09. doi: 10.1016/j.expneurol.2004.06.015. PubMed PMID: 15530875.

136. Zito G, Mattia D, Tomaiuolo F, Oliveri M, Castriota-Scanderbeg A, Caltagirone C, et al. Functional anatomy of motor recovery after early brain damage. Neurocase. 2004;10(3):265-9. Epub 2005/03/25. doi: 10.1080/13554790490495212. PubMed PMID: 15788263.

137. Aoyagi M. [Accuracy of the prognostic diagnosis in acute peripheral facial palsy]. Nihon Jibiinkoka Gakkai Kaiho. 2005;108(1):1-7. Epub 2005/02/17. doi: 10.3950/jibiinkoka.108.1. PubMed PMID: 15712490.

138. Centonze D, Palmieri MG, Boffa L, Pierantozzi M, Stanzione P, Brusa L, et al. Cortical hyperexcitability in post-traumatic stress

disorder secondary to minor accidental head trauma: a neurophysiologic study. J Psychiatry Neurosci. 2005;30(2):127-32. Epub 2005/03/31. PubMed PMID: 15798788; PubMed Central PMCID: PMCPMC551170.

139. Cooper EB, Scherder EJ, Cooper JB. Electrical treatment of reduced consciousness: experience with coma and Alzheimer's disease. Neuropsychol Rehabil. 2005;15(3-4):389-405. Epub 2005/12/15. doi: 10.1080/09602010443000317. PubMed PMID: 16350980.

140. Crossley M, Shiel A, Wilson B, Coleman MR, Gelling L, Fryer T, et al. Monitoring emergence from coma following severe brain injury in an octogenarian using behavioural indicators, electrophysiological measures and metabolic studies: a demonstration of the potential for good recovery in older adults. Brain Inj. 2005;19(9):729-37. Epub 2005/10/01. doi: 10.1080/02699050400013733. PubMed PMID: 16195187.

141. Fagade OO, Oginni FO, Obilade TO. Comparative study of the therapeutic effect of a systemic analgesic and transcutaneous electrical nerve stimulation (TENS) on post-IMF trismus and pain in Nigerian patients. Niger Postgrad Med J. 2005;12(2):97-101. Epub 2005/07/06. PubMed PMID: 15997257.

142. Foley DP, Fernandez LA, Leverson G, Chin LT, Krieger N,

Cooper JT, et al. Donation after cardiac death: the University of Wisconsin experience with liver transplantation. Ann Surg. 2005;242(5):724-31. Epub 2005/10/26. doi: 10.1097/01.sla.0000186178.07110.92. PubMed PMID: 16244547; PubMed Central PMCID: PMCPMC1409855.

143. Hiraoka T, Matsumoto Y, Okamoto F, Yamaguchi T, Hirohara Y, Mihashi T, et al. Corneal higher-order aberrations induced by overnight orthokeratology. Am J Ophthalmol. 2005;139(3):429-36. Epub 2005/03/16. doi: 10.1016/j.ajo.2004.10.006. PubMed PMID: 15767050.

144. Hirshberg LM, Chiu S, Frazier JA. Emerging brain-based interventions for children and adolescents: overview and clinical perspective. Child Adolesc Psychiatr Clin N Am. 2005;14(1):1-19, v. Epub 2004/11/27. doi: 10.1016/j.chc.2004.07.011. PubMed PMID: 15564050.

145. Hjortdal J, Møller-Pedersen T, Ivarsen A, Ehlers N. Corneal power, thickness, and stiffness: results of a prospective randomized controlled trial of PRK and LASIK for myopia. J Cataract Refract Surg. 2005;31(1):21-9. Epub 2005/02/22. doi: 10.1016/j.jcrs.2004.09.043. PubMed PMID: 15721693.

146. Jang SH, Cho SH, Kim YH, You SH, Kim SH, Kim O, et al. Motor recovery mechanism of diffuse axonal injury: a combined

study of transcranial magnetic stimulation and functional MRI. Restor Neurol Neurosci. 2005;23(1):51-6. Epub 2005/04/23. PubMed PMID: 15846032.

147. Karaca K, Etöz A, Kahveci R. Facial nerve identification using a disposable nerve block needle as a nerve stimulator. Plast Reconstr Surg. 2005;115(2):667-8. Epub 2005/02/05. doi: 10.1097/01.prs.0000149062.45157.9b. PubMed PMID: 15692393.

148. Khopade AJ, Arulsudar N, Khopade SA, Hartmann J. Ultrathin antibiotic walled microcapsules. Biomacromolecules. 2005;6(1):229-34. Epub 2005/01/11. doi: 10.1021/bm049554a. PubMed PMID: 15638525.

149. Li C, Maier DL, Cross B, Doherty JJ, Christian EP. Fimbria-fornix lesions compromise the induction of long-term potentiation at the Schaffer collateral-CA1 synapse in the rat in vivo. J Neurophysiol. 2005;93(5):3001-6. Epub 2005/04/23. doi: 10.1152/jn.00546.2004. PubMed PMID: 15846002.

150. Mazarr-Proo S, Kerrigan S. Distribution of GHB in tissues and fluids following a fatal overdose. J Anal Toxicol. 2005;29(5):398-400. Epub 2005/08/18. doi: 10.1093/jat/29.5.398. PubMed PMID: 16105269.

151. Walker N, Michaud K, Wolfe F. Work limitations among working persons with rheumatoid arthritis: results, reliability, and

validity of the work limitations questionnaire in 836 patients. J Rheumatol. 2005;32(6):1006-12. Epub 2005/06/09. PubMed PMID: 15940759.

152. Ward NS. Mechanisms underlying recovery of motor function after stroke. Postgrad Med J. 2005;81(958):510-4. Epub 2005/08/09. doi: 10.1136/pgmj.2004.030809. PubMed PMID: 16085742; PubMed Central PMCID: PMCPMC1743338.

153. André-Obadia N, Peyron R, Mertens P, Mauguière F, Laurent B, Garcia-Larrea L. Transcranial magnetic stimulation for pain control. Double-blind study of different frequencies against placebo, and correlation with motor cortex stimulation efficacy. Clin Neurophysiol. 2006;117(7):1536-44. Epub 2006/06/07. doi: 10.1016/j.clinph.2006.03.025. PubMed PMID: 16753335.

154. Aydin-Abidin S, Moliadze V, Eysel UT, Funke K. Effects of repetitive TMS on visually evoked potentials and EEG in the anaesthetized cat: dependence on stimulus frequency and train duration. J Physiol. 2006;574(Pt 2):443-55. Epub 2006/05/13. doi: 10.1113/jphysiol.2006.108464. PubMed PMID: 16690713; PubMed Central PMCID: PMCPMC1817766.

155. Barba C, Formisano R, Sabatini U, Cicinelli P, Elisabeth Hagberg G, Marconi B, et al. Dysfunction of a structurally normal motor pathway in a brain injury patient as revealed by multimodal

integrated techniques. Neurocase. 2006;12(4):232-5. Epub 2006/09/27. doi: 10.1080/13554790600878879. PubMed PMID: 17000593.

156. Basso D, Lotze M, Vitale L, Ferreri F, Bisiacchi P, Olivetti Belardinelli M, et al. The role of prefrontal cortex in visuo-spatial planning: A repetitive TMS study. Exp Brain Res. 2006;171(3):411-5. Epub 2006/05/20. doi: 10.1007/s00221-006-0457-z. PubMed PMID: 16710684.

157. Bütefisch CM. Neurobiological bases of rehabilitation. Neurol Sci. 2006;27 Suppl 1:S18-23. Epub 2006/05/19. doi: 10.1007/s10072-006-0540-z. PubMed PMID: 16708176.

158. Cohen Kadosh R, Walsh V. Cognitive neuroscience: rewired or crosswired brains? Curr Biol. 2006;16(22):R962-3. Epub 2006/11/23. doi: 10.1016/j.cub.2006.10.017. PubMed PMID: 17113378.

159. Duffau H. Brain plasticity: from pathophysiological mechanisms to therapeutic applications. J Clin Neurosci. 2006;13(9):885-97. Epub 2006/10/20. doi: 10.1016/j.jocn.2005.11.045. PubMed PMID: 17049865.

160. Epstein CM. Seizure or convulsive syncope during 1-Hz rTMS? Clin Neurophysiol. 2006;117(11):2566-7; author reply 7-8. Epub 2006/09/26. doi: 10.1016/j.clinph.2006.07.262. PubMed

PMID: 16996304.
161. Fujiki M, Hikawa T, Abe T, Ishii K, Kobayashi H. Reduced short latency afferent inhibition in diffuse axonal injury patients with memory impairment. Neurosci Lett. 2006;405(3):226-30. Epub 2006/08/12. doi: 10.1016/j.neulet.2006.07.005. PubMed PMID: 16901641.
162. Giacino JT, Hirsch J, Schiff N, Laureys S. Functional neuroimaging applications for assessment and rehabilitation planning in patients with disorders of consciousness. Arch Phys Med Rehabil. 2006;87(12 Suppl 2):S67-76. Epub 2006/12/05. doi: 10.1016/j.apmr.2006.07.272. PubMed PMID: 17140882.
163. Hirai S, Cureoglu S, Schachern PA, Hayashi H, Paparella MM, Harada T. Large vestibular aqueduct syndrome: a human temporal bone study. Laryngoscope. 2006;116(11):2007-11. Epub 2006/11/01. doi: 10.1097/01.mlg.0000237673.94781.0a. PubMed PMID: 17075417.
164. Karnath HO, Dieterich M. Spatial neglect--a vestibular disorder? Brain. 2006;129(Pt 2):293-305. Epub 2005/12/24. doi: 10.1093/brain/awh698. PubMed PMID: 16371409.
165. Kofler M. Levetiracetam suppresses long-loop reflexes at the cortical level. Muscle Nerve. 2006;33(6):785-91. Epub 2006/03/07. doi: 10.1002/mus.20533. PubMed PMID: 16518861.

166. Nyffeler T, Müri R, Pflugshaupt T, Wartburg R, Hess CW. Cortical reorganization after brain damage: the oculomotor model. Eur J Neurosci. 2006;23(5):1397-402. Epub 2006/03/24. doi: 10.1111/j.1460-9568.2006.04648.x. PubMed PMID: 16553801.

167. Osenbach RK. Motor cortex stimulation for intractable pain. Neurosurg Focus. 2006;21(6):E7. Epub 2007/03/08. doi: 10.3171/foc.2006.21.6.12. PubMed PMID: 17341051.

168. Pape TL, Rosenow J, Lewis G. Transcranial magnetic stimulation: a possible treatment for TBI. J Head Trauma Rehabil. 2006;21(5):437-51. Epub 2006/09/20. doi: 10.1097/00001199-200609000-00063. PubMed PMID: 16983227.

169. Parsadanian A, Pan Y, Li W, Myckatyn TM, Brakefield D. Astrocyte-derived transgene GDNF promotes complete and long-term survival of adult facial motoneurons following avulsion and differentially regulates the expression of transcription factors of AP-1 and ATF/CREB families. Exp Neurol. 2006;200(1):26-37. Epub 2006/02/25. doi: 10.1016/j.expneurol.2006.01.014. PubMed PMID: 16497298.

170. Rais D, Nowack B, Schulin R, Luster J. Sorption of trace metals by standard and micro suction cups in the absence and presence of dissolved organic carbon. J Environ Qual. 2006;35(1):50-60. Epub 2006/01/05. doi: 10.2134/jeq2005.0040.

PubMed PMID: 16391276.

171. Robakis TK, Hirsch LJ. Literature review, case report, and expert discussion of prolonged refractory status epilepticus. Neurocrit Care. 2006;4(1):35-46. Epub 2006/02/25. doi: 10.1385/ncc:4:1:035. PubMed PMID: 16498194.

172. Saitoh Y, Hirayama A, Kishima H, Oshino S, Hirata M, Kato A, et al. Stimulation of primary motor cortex for intractable deafferentation pain. Acta Neurochir Suppl. 2006;99:57-9. Epub 2007/03/21. doi: 10.1007/978-3-211-35205-2_11. PubMed PMID: 17370765.

173. Sharova EV, Mel'nikov AV, Novikova MR, Kulikov MA, Grechenko TN, Shekhter ED, et al. [Changes in the brain spontaneous bioelectrical activity during transcranial electrical and electromagnetic stimulation]. Zh Vyssh Nerv Deiat Im I P Pavlova. 2006;56(3):363-70. Epub 2006/07/28. PubMed PMID: 16869271.

174. Shields CB, Ping Zhang Y, Shields LB, Burke DA, Glassman SD. Objective assessment of cervical spinal cord injury levels by transcranial magnetic motor-evoked potentials. Surg Neurol. 2006;66(5):475-83; discussion 83. Epub 2006/11/07. doi: 10.1016/j.surneu.2006.04.009. PubMed PMID: 17084191.

175. Takata K, Yamauchi H, Tatsuno H, Hashimoto K, Abo M. Is the ipsilateral cortex surrounding the lesion or the non-injured

contralateral cortex important for motor recovery in rats with photochemically induced cortical lesions? Eur Neurol. 2006;56(2):106-12. Epub 2006/09/09. doi: 10.1159/000095700. PubMed PMID: 16960450.

176. Takeuchi N, Ikoma K, Chuma T, Matsuo Y. Measurement of transcallosal inhibition in traumatic brain injury by transcranial magnetic stimulation. Brain Inj. 2006;20(9):991-6. Epub 2006/10/19. doi: 10.1080/02699050600909771. PubMed PMID: 17046801.

177. Wang IJ, Sun YC, Lee YC, Hou YC, Hu FR. The relationship between anterior corneal aberrations and contrast sensitivity in conventional LASIK. Curr Eye Res. 2006;31(7-8):563-8. Epub 2006/08/01. doi: 10.1080/02713680600814761. PubMed PMID: 16877264.

178. Ward NS, Frackowiak RS. The functional anatomy of cerebral reorganisation after focal brain injury. J Physiol Paris. 2006;99(4-6):425-36. Epub 2006/06/06. doi: 10.1016/j.jphysparis.2006.03.002. PubMed PMID: 16750616.

179. Zumsteg D, Wennberg R, Gütling E, Hess K. Whiplash and concussion: similar acute changes in middle-latency SEPs. Can J Neurol Sci. 2006;33(4):379-86. Epub 2006/12/16. doi: 10.1017/s0317167100005333. PubMed PMID: 17168163.

180. Belardinelli P, Ciancetta L, Staudt M, Pizzella V, Londei A, Birbaumer N, et al. Cerebro-muscular and cerebro-cerebral coherence in patients with pre- and perinatally acquired unilateral brain lesions. Neuroimage. 2007;37(4):1301-14. Epub 2007/08/03. doi: 10.1016/j.neuroimage.2007.05.053. PubMed PMID: 17669666.
181. Canavero S, Bonicalzi V. Extradural cortical stimulation for movement disorders. Acta Neurochir Suppl. 2007;97(Pt 2):223-32. Epub 2007/08/19. doi: 10.1007/978-3-211-33081-4_25. PubMed PMID: 17691308.
182. Canavero S, Bonicalzi V. Extradural cortical stimulation for central pain. Acta Neurochir Suppl. 2007;97(Pt 2):27-36. Epub 2007/08/19. doi: 10.1007/978-3-211-33081-4_3. PubMed PMID: 17691286.
183. Cioni B, Meglio M. Motor cortex stimulation for chronic non-malignant pain: current state and future prospects. Acta Neurochir Suppl. 2007;97(Pt 2):45-9. Epub 2007/08/19. doi: 10.1007/978-3-211-33081-4_5. PubMed PMID: 17691288.
184. Cioni B, Meglio M, Perotti V, De Bonis P, Montano N. Neurophysiological aspects of chronic motor cortex stimulation. Neurophysiol Clin. 2007;37(6):441-7. Epub 2007/12/18. doi: 10.1016/j.neucli.2007.10.007. PubMed PMID: 18083500.

185. Cruccu G, Aziz TZ, Garcia-Larrea L, Hansson P, Jensen TS, Lefaucheur JP, et al. EFNS guidelines on neurostimulation therapy for neuropathic pain. Eur J Neurol. 2007;14(9):952-70. Epub 2007/08/28. doi: 10.1111/j.1468-1331.2007.01916.x. PubMed PMID: 17718686.

186. De Beaumont L, Lassonde M, Leclerc S, Théoret H. Long-term and cumulative effects of sports concussion on motor cortex inhibition. Neurosurgery. 2007;61(2):329-36; discussion 36-7. Epub 2007/09/01. doi: 10.1227/01.Neu.0000280000.03578.B6. PubMed PMID: 17762745.

187. Favier I, van Vliet JA, Roon KI, Witteveen RJ, Verschuuren JJ, Ferrari MD, et al. Trigeminal autonomic cephalgias due to structural lesions: a review of 31 cases. Arch Neurol. 2007;64(1):25-31. Epub 2007/01/11. doi: 10.1001/archneur.64.1.25. PubMed PMID: 17210806.

188. Floel A, Cohen LG. Contribution of noninvasive cortical stimulation to the study of memory functions. Brain Res Rev. 2007;53(2):250-9. Epub 2006/10/07. doi: 10.1016/j.brainresrev.2006.08.006. PubMed PMID: 17023050.

189. Fodstad H, Hariz M. Electricity in the treatment of nervous system disease. Acta Neurochir Suppl. 2007;97(Pt 1):11-9. Epub

2007/08/19. doi: 10.1007/978-3-211-33079-1_2. PubMed PMID: 17691352.

190. Gimranov RF, Moskalenko SN, Gimranova Zh V. [Investigation of evoked acoustic brainstem potentials in norm and pathology]. Voen Med Zh. 2007;328(2):24-7. Epub 2007/05/19. PubMed PMID: 17508606.

191. Ibekwe TS, Ijaduola GT, Nwaorgu OG. Tympanic membrane perforation among adults in West Africa. Otol Neurotol. 2007;28(3):348-52. Epub 2007/04/07. doi: 10.1097/MAO.0b013e3180311605. PubMed PMID: 17414040.

192. Jafri B, Li X, Yang H, Rabinowitz YS. Higher order wavefront aberrations and topography in early and suspected keratoconus. J Refract Surg. 2007;23(8):774-81. Epub 2007/11/08. doi: 10.3928/1081-597x-20071001-06. PubMed PMID: 17985796.

193. Kleim JA, Kleim ED, Cramer SC. Systematic assessment of training-induced changes in corticospinal output to hand using frameless stereotaxic transcranial magnetic stimulation. Nat Protoc. 2007;2(7):1675-84. Epub 2007/07/21. doi: 10.1038/nprot.2007.206. PubMed PMID: 17641632.

194. Lazorthes Y, Sol JC, Fowo S, Roux FE, Verdié JC. Motor cortex stimulation for neuropathic pain. Acta Neurochir Suppl. 2007;97(Pt 2):37-44. Epub 2007/08/19. doi:

10.1007/978-3-211-33081-4_4. PubMed PMID: 17691287.

195. Lepilin AV, Bakhteeva GR, Erokina NL. [Transcutaneous electric stimulation of nerves use in comprehensive treatment of patients with mandibular fractures]. Stomatologiia (Mosk). 2007;86(2):54-6. Epub 2007/09/11. PubMed PMID: 17828071.

196. Luo MF, He JN, Guo Y, Li CH, Zhang JL. [Effect of electroacupuncture and moxibustion of "Dazhui" (GV14) on the number and distribution of degranulated mast cells in GV14 region]. Zhen Ci Yan Jiu. 2007;32(5):327-9. Epub 2007/12/07. PubMed PMID: 18050625.

197. Moliavchikova OV, Cherevashchenko LA, Grinzaĭd Iu M, Aĭvazov VN, Zhuravlev ME. [Effects of combined action of radon baths and transcranial magnetotherapy on cerebral circulation in patients in an intermediate period of a mild craniocerebral trauma]. Vopr Kurortol Fizioter Lech Fiz Kult. 2007(3):19-22. Epub 2007/07/25. PubMed PMID: 17647311.

198. Park OH, Lee KJ, Rhyu IJ, Geum D, Kim H, Buss R, et al. Bax-dependent and -independent death of motoneurons after facial nerve injury in adult mice. Eur J Neurosci. 2007;26(6):1421-32. Epub 2007/09/08. doi: 10.1111/j.1460-9568.2007.05787.x. PubMed PMID: 17822434.

199. Payne CM, Weber C, Crowley-Skillicorn C, Dvorak K,

Bernstein H, Bernstein C, et al. Deoxycholate induces mitochondrial oxidative stress and activates NF-kappaB through multiple mechanisms in HCT-116 colon epithelial cells. Carcinogenesis. 2007;28(1):215-22. Epub 2006/08/05. doi: 10.1093/carcin/bgl139. PubMed PMID: 16887864.

200. Petropoulou KB, Panourias IG, Rapidi CA, Sakas DE. The importance of neurorehabilitation to the outcome of neuromodulation in spasticity. Acta Neurochir Suppl. 2007;97(Pt 1):243-50. Epub 2007/08/19. doi: 10.1007/978-3-211-33079-1_33. PubMed PMID: 17691383.

201. Riós-Romenets S, Castaño-Monsalve B, Bernabeu-Guitart M. [Pharmacotherapy of the cognitive sequelae secondary to traumatic brain injury]. Rev Neurol. 2007;45(9):563-70. Epub 2007/11/06. PubMed PMID: 17979087.

202. Sakas DE, Panourias IG, Simpson BA, Krames ES. An introduction to operative neuromodulation and functional neuroprosthetics, the new frontiers of clinical neuroscience and biotechnology. Acta Neurochir Suppl. 2007;97(Pt 1):3-10. Epub 2007/08/19. doi: 10.1007/978-3-211-33079-1_1. PubMed PMID: 17691351.

203. Sharova EV, Mel'nikov AV, Novikova MR, Kulikov MA, Grechenko TN, Shekhter ED, et al. Changes in spontaneous brain

bioelectrical activity during transcranial electrical and electromagnetic stimulation. Neurosci Behav Physiol. 2007;37(5):451-7. Epub 2007/05/17. doi: 10.1007/s11055-007-0034-3. PubMed PMID: 17505794.

204. Tani N, Saitoh Y, Kishima H, Oshino S, Hatazawa J, Hashikawa K, et al. Motor cortex stimulation for levodopa-resistant akinesia: case report. Mov Disord. 2007;22(11):1645-9. Epub 2007/06/09. doi: 10.1002/mds.21593. PubMed PMID: 17557343.

205. Voropaev AA, Troshin VD, Mochalov AD. [Craniospinal electromagnetic therapy in the neurological practice]. Zh Nevrol Psikhiatr Im S S Korsakova. 2007;107(12):16-20. Epub 2008/04/23. PubMed PMID: 18427454.

206. Yasokawa YT, Shinoda J, Okumura A, Nakayama N, Miwa K, Iwama T. Correlation between diffusion-tensor magnetic resonance imaging and motor-evoked potential in chronic severe diffuse axonal injury. J Neurotrauma. 2007;24(1):163-73. Epub 2007/02/01. doi: 10.1089/neu.2006.0073. PubMed PMID: 17263680.

207. Battelli L, Walsh V, Pascual-Leone A, Cavanagh P. The 'when' parietal pathway explored by lesion studies. Curr Opin Neurobiol. 2008;18(2):120-6. Epub 2008/08/19. doi:

10.1016/j.conb.2008.08.004. PubMed PMID: 18708141; PubMed Central PMCID: PMCPMC5076376.

208. Dabydeen L, Thomas JE, Aston TJ, Hartley H, Sinha SK, Eyre JA. High-energy and -protein diet increases brain and corticospinal tract growth in term and preterm infants after perinatal brain injury. Pediatrics. 2008;121(1):148-56. Epub 2008/01/02. doi: 10.1542/peds.2007-1267. PubMed PMID: 18166569.

209. Gleason D, Fallon JH, Guerra M, Liu JC, Bryant PJ. Ependymal stem cells divide asymmetrically and transfer progeny into the subventricular zone when activated by injury. Neuroscience. 2008;156(1):81-8. Epub 2008/08/07. doi: 10.1016/j.neuroscience.2008.06.065. PubMed PMID: 18682279.

210. Hill K, Dutta P, Zareba A, Eldridge ML, Sepaniak MJ. Morphological and chemical optimization of microcantilever surfaces for thyroid system biosensing and beyond. Anal Chim Acta. 2008;625(1):55-62. Epub 2008/08/30. doi: 10.1016/j.aca.2008.07.005. PubMed PMID: 18721540.

211. Hosomi K, Saitoh Y, Kishima H, Oshino S, Hirata M, Tani N, et al. Electrical stimulation of primary motor cortex within the central sulcus for intractable neuropathic pain. Clin Neurophysiol. 2008;119(5):993-1001. Epub 2008/03/11. doi: 10.1016/j.clinph.2007.12.022. PubMed PMID: 18329955.

212. Isagulian ED, Shabalov VA. [Neuromodulation--controlled analgesia. Criteria for long efficiency]. Zh Vopr Neirokhir Im N N Burdenko. 2008(2):18-26; discussion -8. Epub 2008/08/30. PubMed PMID: 18720727.

213. Jarema M. A few facts from the history of selected biological treatment methods in psychiatry. Neuro Endocrinol Lett. 2008;29 Suppl 1:5-9. Epub 2008/11/26. PubMed PMID: 19029880.

214. Jin Y, Hilgetag CC. Perturbation of visuospatial attention by high-frequency offline rTMS. Exp Brain Res. 2008;189(1):121-8. Epub 2008/06/20. doi: 10.1007/s00221-008-1449-y. PubMed PMID: 18563400.

215. Kao CD, Chen JT, Lai KL, Chang JB, Wu ZA, Liao KK. Gabapentin for decerebrate rigidity: a case report. Clin Drug Investig. 2008;28(1):67-70. Epub 2007/12/18. doi: 10.2165/00044011-200828010-00008. PubMed PMID: 18081362.

216. Kirton A, Deveber G, Gunraj C, Chen R. Neurocardiogenic syncope complicating pediatric transcranial magnetic stimulation. Pediatr Neurol. 2008;39(3):196-7. Epub 2008/08/30. doi: 10.1016/j.pediatrneurol.2008.06.004. PubMed PMID: 18725065.

217. Liu JT, Lee JK, Chang PJ, Sun CM. Increased regional cerebral perfusion in contralateral motor and somatosensory areas after median nerve stimulation therapy. Acta Neurochir Suppl.

2008;101:65-70. Epub 2008/07/23. doi: 10.1007/978-3-211-78205-7_11. PubMed PMID: 18642636.
218. Liu JT, Lee JK, Tyan YS, Liu CY, Lin TB. Change in cerebral perfusion of patients with coma after treatment with right median nerve stimulation and hyperbaric oxygen. Neuromodulation. 2008;11(4):296-301. Epub 2008/10/01. doi: 10.1111/j.1525-1403.2008.00179.x. PubMed PMID: 22151144.
219. Miniussi C, Cappa SF, Cohen LG, Floel A, Fregni F, Nitsche MA, et al. Efficacy of repetitive transcranial magnetic stimulation/transcranial direct current stimulation in cognitive neurorehabilitation. Brain Stimul. 2008;1(4):326-36. Epub 2008/10/01. doi: 10.1016/j.brs.2008.07.002. PubMed PMID: 20633391.
220. Müri RM, Nyffeler T. Neurophysiology and neuroanatomy of reflexive and volitional saccades as revealed by lesion studies with neurological patients and transcranial magnetic stimulation (TMS). Brain Cogn. 2008;68(3):284-92. Epub 2008/10/11. doi: 10.1016/j.bandc.2008.08.018. PubMed PMID: 18845373.
221. Sandin M, Thörn SE, Dahlqvist A, Wattwil L, Axelsson K, Wattwil M. Effects of pain stimulation on bispectral index, heart rate and blood pressure at different minimal alveolar concentration values of sevoflurane. Acta Anaesthesiol Scand. 2008;52(3):420-6.

Epub 2008/02/14. doi: 10.1111/j.1399-6576.2007.01569.x. PubMed PMID: 18269392.

222. Serruya MD, Kahana MJ. Techniques and devices to restore cognition. Behav Brain Res. 2008;192(2):149-65. Epub 2008/06/10. doi: 10.1016/j.bbr.2008.04.007. PubMed PMID: 18539345; PubMed Central PMCID: PMCPMC3051349.

223. Shaĭkhaev GO, Kuz'mina NS, Miazin AE, Panushkina OG, Rubanovich AV, Khaĭmovich TI, et al. [The analysis of mutations at mini- and microsatellite DNA loci in the family members of a group of occupationally exposed to tritium and tritium oxide plant workers]. Radiats Biol Radioecol. 2008;48(6):690-7. Epub 2009/01/31. PubMed PMID: 19178046.

224. Shaladi A, Crestani F, Saltari R, Piva B. [Percutaneous electrical nerve stimulation of peripheral nerve for the intractable occipital neuralgia]. Recenti Prog Med. 2008;99(6):295-301. Epub 2008/08/20. PubMed PMID: 18710060.

225. Staudt M, Ticini LF, Grodd W, Krägeloh-Mann I, Karnath HO. Functional topography of early periventricular brain lesions in relation to cytoarchitectonic probabilistic maps. Brain Lang. 2008;106(3):177-83. Epub 2008/02/16. doi: 10.1016/j.bandl.2008.01.007. PubMed PMID: 18275996.

226. Visocchi M. Neuromodulation of cerebral blood flow by

spinal cord electrical stimulation: the role of the Italian school and state of art. J Neurosurg Sci. 2008;52(2):41-7. Epub 2008/05/27. PubMed PMID: 18500217.

227. Wang JL, Ren QS, Shen CC, Xie WX, Zheng RX, Ni JW. [Effect of transcutaneous acupoint electrical stimulation on blood bioactive compounds involving cerebral injury during craniotomy]. Zhen Ci Yan Jiu. 2008;33(1):26-30. Epub 2008/04/05. PubMed PMID: 18386641.

228. Wu HG, Jiang B, Zhou EH, Shi Z, Shi DR, Cui YH, et al. Regulatory mechanism of electroacupuncture in irritable bowel syndrome: preventing MC activation and decreasing SP VIP secretion. Dig Dis Sci. 2008;53(6):1644-51. Epub 2007/11/14. doi: 10.1007/s10620-007-0062-4. PubMed PMID: 17999187.

229. Yoo WK, Kim DS, Kwon YH, Jang SH. Kernohan's notch phenomenon demonstrated by diffusion tensor imaging and transcranial magnetic stimulation. J Neurol Neurosurg Psychiatry. 2008;79(11):1295-7. Epub 2008/10/23. doi: 10.1136/jnnp.2007.138131. PubMed PMID: 18940992.

230. Baad-Hansen L, Blicher JU, Lapitskaya N, Nielsen JF, Svensson P. Intra-cortical excitability in healthy human subjects after tongue training. J Oral Rehabil. 2009;36(6):427-34. Epub 2009/05/09. doi: 10.1111/j.1365-2842.2009.01955.x. PubMed

PMID: 19422433.

231. Bernabeu M, Demirtas-Tatlidede A, Opisso E, Lopez R, Tormos JM, Pascual-Leone A. Abnormal corticospinal excitability in traumatic diffuse axonal brain injury. J Neurotrauma. 2009;26(12):2185-93. Epub 2009/07/17. doi: 10.1089/neu.2008.0859. PubMed PMID: 19604100; PubMed Central PMCID: PMCPMC2824228.

232. Bikson M, Datta A, Elwassif M. Establishing safety limits for transcranial direct current stimulation. Clin Neurophysiol. 2009;120(6):1033-4. Epub 2009/04/28. doi: 10.1016/j.clinph.2009.03.018. PubMed PMID: 19394269; PubMed Central PMCID: PMCPMC2754807.

233. Bolognini N, Pascual-Leone A, Fregni F. Using non-invasive brain stimulation to augment motor training-induced plasticity. J Neuroeng Rehabil. 2009;6:8. Epub 2009/03/19. doi: 10.1186/1743-0003-6-8. PubMed PMID: 19292910; PubMed Central PMCID: PMCPMC2667408.

234. Boly M, Massimini M, Tononi G. Theoretical approaches to the diagnosis of altered states of consciousness. Prog Brain Res. 2009;177:383-98. Epub 2009/10/13. doi: 10.1016/s0079-6123(09)17727-0. PubMed PMID: 19818916.

235. Chen MJ, Peng ML, Chen LW, Fan HL, Liou YM, Liao JM.

Pressor effects on blood pressure induced by isovolumic bladder distension and electro-acupuncture stimulations in anesthetized rats. Chin J Physiol. 2009;52(6):426-31. Epub 2010/03/27. doi: 10.4077/cjp.2009.amh069. PubMed PMID: 20337150.

236. Chen XY, Zhu Y, Huang XS. [Effect of strong stimulation of acupuncture at twelve Jing-well points as main for neurosurgery patients with disorder of consciousness]. Zhongguo Zhen Jiu. 2009;29(8):619-22. Epub 2009/12/02. PubMed PMID: 19947263.

237. Chou P, Chu H, Lin JG. Effects of electroacupuncture treatment on impaired cognition and quality of life in Taiwanese stroke patients. J Altern Complement Med. 2009;15(10):1067-73. Epub 2010/01/06. PubMed PMID: 20050300.

238. De Beaumont L, Théoret H, Mongeon D, Messier J, Leclerc S, Tremblay S, et al. Brain function decline in healthy retired athletes who sustained their last sports concussion in early adulthood. Brain. 2009;132(Pt 3):695-708. Epub 2009/01/30. doi: 10.1093/brain/awn347. PubMed PMID: 19176544.

239. De FP, Fellus J, Polito MZ, Thompson JW, Moser RS, DeLuca J. The new neuroscience frontier: promoting neuroplasticity and brain repair in traumatic brain injury. Clin Neuropsychol. 2009;23(8):1391-9. Epub 2009/11/03. doi: 10.1080/13854040903058978. PubMed PMID: 19882477.

240. Fu YY, Cao SQ, Zhuang JX, Hu L, Chen DK, Gu FJ. [Observation on electroacupuncture combined with routine western medicine therapy for promoting consciousness of the patient with coma caused by craniocerebral trauma]. Zhongguo Zhen Jiu. 2009;29(2):107-10. Epub 2009/04/28. PubMed PMID: 19391532.

241. Gao J, Youn S, Hovsepyan A, Llaneza VL, Wang Y, Bitton G, et al. Dispersion and toxicity of selected manufactured nanomaterials in natural river water samples: effects of water chemical composition. Environ Sci Technol. 2009;43(9):3322-8. Epub 2009/06/19. doi: 10.1021/es803315v. PubMed PMID: 19534153.

242. Gurskaia OE, Ponomarev VA. [Methods of localization of equivalent EEG sources in the diagnostics of protracted loss of consciousness]. Zh Nevrol Psikhiatr Im S S Korsakova. 2009;109(4):36-42. Epub 2009/06/06. PubMed PMID: 19491808.

243. Hiraoka T, Mihashi T, Okamoto C, Okamoto F, Hirohara Y, Oshika T. Influence of induced decentered orthokeratology lens on ocular higher-order wavefront aberrations and contrast sensitivity function. J Cataract Refract Surg. 2009;35(11):1918-26. Epub 2009/11/03. doi: 10.1016/j.jcrs.2009.06.018. PubMed PMID: 19878824.

244. Hwang SC, Im SB, Kim BT, Shin WH. Safe entry point for

twist-drill craniostomy of a chronic subdural hematoma. J Neurosurg. 2009;110(6):1265-70. Epub 2008/12/23. doi: 10.3171/2008.9.Jns08359. PubMed PMID: 19099378.

245. Johnston MV. Plasticity in the developing brain: implications for rehabilitation. Dev Disabil Res Rev. 2009;15(2):94-101. Epub 2009/06/03. doi: 10.1002/ddrr.64. PubMed PMID: 19489084.

246. Lapitska N, Gosseries O, Delvaux V, Overgaard M, Nielsen F, Maertens de Noordhout A, et al. Transcranial magnetic stimulation in disorders of consciousness. Rev Neurosci. 2009;20(3-4):235-50. Epub 2010/02/18. doi: 10.1515/revneuro.2009.20.3-4.235. PubMed PMID: 20157993.

247. Lapitskaya N, Coleman MR, Nielsen JF, Gosseries O, de Noordhout AM. Disorders of consciousness: further pathophysiological insights using motor cortex transcranial magnetic stimulation. Prog Brain Res. 2009;177:191-200. Epub 2009/10/13. doi: 10.1016/s0079-6123(09)17713-0. PubMed PMID: 19818902.

248. Lepilin AV, Erokina NL, Rogatina TV, Khlusov I. [Using dynamic magnetotherapy and transdermal electroneurostimulation in the combined treatment of patients with mandibular fractures and concomitant periodontal inflammatory diseases]. Vopr Kurortol Fizioter Lech Fiz Kult. 2009(4):37-40. Epub 2009/08/28.

PubMed PMID: 19711518.

249. Liebetanz D, Koch R, Mayenfels S, König F, Paulus W, Nitsche MA. Safety limits of cathodal transcranial direct current stimulation in rats. Clin Neurophysiol. 2009;120(6):1161-7. Epub 2009/05/01. doi: 10.1016/j.clinph.2009.01.022. PubMed PMID: 19403329.

250. Liu JT, Lee JK, Tyan YS, Liu CY, Chen YH, Lin TB. Neuromodulation on cervical spinal cord combined with hyperbaric oxygen in comatose patients--a preliminary report. Surg Neurol. 2009;72 Suppl 2:S28-34; discussion S-5. Epub 2009/08/12. doi: 10.1016/j.surneu.2009.05.029. PubMed PMID: 19665182.

251. Louise-Bender Pape T, Rosenow J, Lewis G, Ahmed G, Walker M, Guernon A, et al. Repetitive transcranial magnetic stimulation-associated neurobehavioral gains during coma recovery. Brain Stimul. 2009;2(1):22-35. Epub 2009/01/01. doi: 10.1016/j.brs.2008.09.004. PubMed PMID: 20633400.

252. Lusardi TA. Adenosine neuromodulation and traumatic brain injury. Curr Neuropharmacol. 2009;7(3):228-37. Epub 2010/03/02. doi: 10.2174/157015909789152137. PubMed PMID: 20190964; PubMed Central PMCID: PMCPMC2769006.

253. MacDonald SW, Li SC, Bäckman L. Neural underpinnings of within-person variability in cognitive functioning. Psychol Aging.

2009;24(4):792-808. Epub 2009/12/23. doi: 10.1037/a0017798. PubMed PMID: 20025396.
254. Maguire S, Pickerd N, Farewell D, Mann M, Tempest V, Kemp AM. Which clinical features distinguish inflicted from non-inflicted brain injury? A systematic review. Arch Dis Child. 2009;94(11):860-7. Epub 2009/06/18. doi: 10.1136/adc.2008.150110. PubMed PMID: 19531526.
255. Massimini M, Boly M, Casali A, Rosanova M, Tononi G. A perturbational approach for evaluating the brain's capacity for consciousness. Prog Brain Res. 2009;177:201-14. Epub 2009/10/13. doi: 10.1016/s0079-6123(09)17714-2. PubMed PMID: 19818903.
256. Ozturk M, Komurcu S, Kilic S, Ozet A, Arpaci F, Ozturk B, et al. Self-reported experience of mucositis in cancer patients who underwent conditioning regimen and stem cell transplantation. Support Care Cancer. 2009;17(10):1295-9. Epub 2009/02/10. doi: 10.1007/s00520-009-0586-6. PubMed PMID: 19198890.
257. Stasiukyniene V, Pilvinis V, Reingardiene D, Janauskaite L. [Epileptic seizures in critically ill patients]. Medicina (Kaunas). 2009;45(6):501-7. Epub 2009/07/17. PubMed PMID: 19605972.
258. Suppiej A, Cappellari A, Cogo PE. Prognostic role of somatosensory and auditory evoked potentials in paediatric hypoxic-ischemic encephalopathy managed with hypothermia: an

illustrative case. Neurophysiol Clin. 2009;39(2):101-5. Epub 2009/05/27. doi: 10.1016/j.neucli.2009.02.003. PubMed PMID: 19467440.

259. Vandermeeren Y, Davare M, Duque J, Olivier E. Reorganization of cortical hand representation in congenital hemiplegia. Eur J Neurosci. 2009;29(4):845-54. Epub 2009/02/10. doi: 10.1111/j.1460-9568.2009.06619.x. PubMed PMID: 19200077.

260. Williams KM, Mella H, Lucas PJ, Williams JA, Telford W, Gress RE. Single cell analysis of complex thymus stromal cell populations: rapid thymic epithelia preparation characterizes radiation injury. Clin Transl Sci. 2009;2(4):279-85. Epub 2009/09/15. doi: 10.1111/j.1752-8062.2009.00128.x. PubMed PMID: 19750208; PubMed Central PMCID: PMCPMC2741333.

261. Yuan Q. Theta bursts in the olfactory nerve paired with beta-adrenoceptor activation induce calcium elevation in mitral cells: a mechanism for odor preference learning in the neonate rat. Learn Mem. 2009;16(11):676-81. Epub 2009/10/28. doi: 10.1101/lm.1569309. PubMed PMID: 19858361; PubMed Central PMCID: PMCPMC2775516.

262. Zaghi S, Heine N, Fregni F. Brain stimulation for the treatment of pain: A review of costs, clinical effects, and

mechanisms of treatment for three different central neuromodulatory approaches. J Pain Manag. 2009;2(3):339-52. Epub 2010/06/30. PubMed PMID: 20585474; PubMed Central PMCID: PMCPMC2888303.

263. Alfieri FM, de Jesus Guirro RR, Teodori RM. Postural stability of elderly submmitted to multisensorial physical therapy intervention. Electromyogr Clin Neurophysiol. 2010;50(2):113-9. Epub 2010/04/22. PubMed PMID: 20405787.

264. Barañano CF, Sweitzer RS, Mahalak ML, Alexander NS, Woolley AL. The management of myringotomy tubes in pediatric cochlear implant recipients. Arch Otolaryngol Head Neck Surg. 2010;136(6):557-60. Epub 2010/06/23. doi: 10.1001/archoto.2010.80. PubMed PMID: 20566905.

265. Bashir S, Mizrahi I, Weaver K, Fregni F, Pascual-Leone A. Assessment and modulation of neural plasticity in rehabilitation with transcranial magnetic stimulation. Pm r. 2010;2(12 Suppl 2):S253-68. Epub 2011/02/09. doi: 10.1016/j.pmrj.2010.10.015. PubMed PMID: 21172687; PubMed Central PMCID: PMCPMC3951769.

266. Cosentino G, Giglia G, Palermo A, Panetta ML, Lo Baido R, Brighina F, et al. A case of post-traumatic complex auditory hallucinosis treated with rTMS. Neurocase. 2010;16(3):267-72.

Epub 2010/01/28. doi: 10.1080/13554790903456191. PubMed PMID: 20104391.
267. Dagi TF. Consenting to the ineffable: the problem of neuromodulation and altered consciousness. J Clin Ethics. 2010;21(2):140-2. Epub 2010/09/28. PubMed PMID: 20866020.
268. Datta A, Bikson M, Fregni F. Transcranial direct current stimulation in patients with skull defects and skull plates: high-resolution computational FEM study of factors altering cortical current flow. Neuroimage. 2010;52(4):1268-78. Epub 2010/05/04. doi: 10.1016/j.neuroimage.2010.04.252. PubMed PMID: 20435146; PubMed Central PMCID: PMCPMC2910315.
269. DeFina PA, Fellus J, Thompson JW, Eller M, Moser RS, Frisina PG, et al. Improving outcomes of severe disorders of consciousness. Restor Neurol Neurosci. 2010;28(6):769-80. Epub 2011/01/07. doi: 10.3233/rnn-2010-0548. PubMed PMID: 21209492.
270. Driver J, Blankenburg F, Bestmann S, Ruff CC. New approaches to the study of human brain networks underlying spatial attention and related processes. Exp Brain Res. 2010;206(2):153-62. Epub 2010/04/01. doi: 10.1007/s00221-010-2205-7. PubMed PMID: 20354681; PubMed Central PMCID: PMCPMC2940032.

271. Ferrarelli F, Massimini M, Sarasso S, Casali A, Riedner BA, Angelini G, et al. Breakdown in cortical effective connectivity during midazolam-induced loss of consciousness. Proc Natl Acad Sci U S A. 2010;107(6):2681-6. Epub 2010/02/06. doi: 10.1073/pnas.0913008107. PubMed PMID: 20133802; PubMed Central PMCID: PMCPMC2823915.
272. Gall C, Fedorov AB, Ernst L, Borrmann A, Sabel BA. Repetitive transorbital alternating current stimulation in optic neuropathy. NeuroRehabilitation. 2010;27(4):335-41. Epub 2010/12/17. doi: 10.3233/nre-2010-0617. PubMed PMID: 21160123.
273. Georgiopoulos M, Katsakiori P, Kefalopoulou Z, Ellul J, Chroni E, Constantoyannis C. Vegetative state and minimally conscious state: a review of the therapeutic interventions. Stereotact Funct Neurosurg. 2010;88(4):199-207. Epub 2010/05/13. doi: 10.1159/000314354. PubMed PMID: 20460949.
274. Holmström L, Vollmer B, Tedroff K, Islam M, Persson JK, Kits A, et al. Hand function in relation to brain lesions and corticomotor-projection pattern in children with unilateral cerebral palsy. Dev Med Child Neurol. 2010;52(2):145-52. Epub 2009/10/08. doi: 10.1111/j.1469-8749.2009.03496.x. PubMed PMID: 19807768.

275. Jang SH, Hong JH, Ahn SH, Son SM, Cho YW. Motor function reorganization in a patient with a brainstem lesion: DTT, fMRI and TMS study. NeuroRehabilitation. 2010;26(2):167-71. Epub 2010/03/06. doi: 10.3233/nre-2010-0550. PubMed PMID: 20203384.

276. Leone M, Franzini A, Proietti Cecchini A, Mea E, Broggi G, Bussone G. Deep brain stimulation in trigeminal autonomic cephalalgias. Neurotherapeutics. 2010;7(2):220-8. Epub 2010/05/01. doi: 10.1016/j.nurt.2010.02.001. PubMed PMID: 20430322; PubMed Central PMCID: PMCPMC5084104.

277. Levy R, Deer TR, Henderson J. Intracranial neurostimulation for pain control: a review. Pain Physician. 2010;13(2):157-65. Epub 2010/03/24. PubMed PMID: 20309382.

278. Liu JP, Yang ZL, Wang MS, Shi R, Zhu BP. [Observation on therapeutic effect of electroacupuncture therapy for promoting consciousness of patients with coma]. Zhongguo Zhen Jiu. 2010;30(3):206-8. Epub 2010/05/26. PubMed PMID: 20496735.

279. Livingston SC, Saliba EN, Goodkin HP, Barth JT, Hertel JN, Ingersoll CD. A preliminary investigation of motor evoked potential abnormalities following sport-related concussion. Brain Inj. 2010;24(6):904-13. Epub 2010/05/04. doi: 10.3109/02699051003789245. PubMed PMID: 20433286.

280. Pallanti S. Unique contributions of brain stimulation to the study of consciousness: where neuroscience meets philosophy. CNS Spectr. 2010;15(3):154-6. Epub 2010/04/24. doi: 10.1017/s1092852900027425. PubMed PMID: 20414164.

281. Peng F, Chen ZQ, Luo JK. [Clinical observation on continuous electroacupuncture at Neiguan (PC 6) for arousing consciousness of comatose patients with severe craniocerebral trauma]. Zhongguo Zhen Jiu. 2010;30(6):465-8. Epub 2010/06/29. PubMed PMID: 20578383.

282. Polanowska K, Seniów J. [Influence of transcranial direct current stimulation on cognitive functioning of patients with brain injury]. Neurol Neurochir Pol. 2010;44(6):580-90. Epub 2011/01/13. doi: 10.1016/s0028-3843(14)60156-0. PubMed PMID: 21225521.

283. Robinson LR, Micklesen PJ. Does stimulus rate matter when performing somatosensory evoked potentials for coma patients? Neurocrit Care. 2010;12(1):69-73. Epub 2009/02/20. doi: 10.1007/s12028-009-9191-2. PubMed PMID: 19225910.

284. Shah SA, Schiff ND. Central thalamic deep brain stimulation for cognitive neuromodulation - a review of proposed mechanisms and investigational studies. Eur J Neurosci. 2010;32(7):1135-44. Epub 2010/11/03. doi: 10.1111/j.1460-9568.2010.07420.x.

PubMed PMID: 21039953; PubMed Central PMCID: PMCPMC3058925.

285. Stoeckel MC, Binkofski F. The role of ipsilateral primary motor cortex in movement control and recovery from brain damage. Exp Neurol. 2010;221(1):13-7. Epub 2009/11/10. doi: 10.1016/j.expneurol.2009.10.021. PubMed PMID: 19896482.

286. Tumakaev RF, Guseva DS, Garaev RS. [Dimephosphon restores functions of the injured spinal cord]. Eksp Klin Farmakol. 2010;73(5):41-4. Epub 2010/07/06. PubMed PMID: 20597371.

287. Burnstock G, Krügel U, Abbracchio MP, Illes P. Purinergic signalling: from normal behaviour to pathological brain function. Prog Neurobiol. 2011;95(2):229-74. Epub 2011/09/13. doi: 10.1016/j.pneurobio.2011.08.006. PubMed PMID: 21907261.

288. Connon FF, Namdarian B, Ee JL, Drummond KJ, Miller JA. Do routinely repeated computed tomography scans in traumatic brain injury influence management? A prospective observational study in a level 1 trauma center. Ann Surg. 2011;254(6):1028-31. Epub 2011/11/25. doi: 10.1097/SLA.0b013e318219727f. PubMed PMID: 22112983.

289. De Beaumont L, Mongeon D, Tremblay S, Messier J, Prince F, Leclerc S, et al. Persistent motor system abnormalities in formerly concussed athletes. J Athl Train. 2011;46(3):234-40. Epub

2011/06/15. doi: 10.4085/1062-6050-46.3.234. PubMed PMID: 21669091; PubMed Central PMCID: PMCPMC3419550.

290. Edwardson M, Fetz EE, Avery DH. Seizure produced by 20 Hz transcranial magnetic stimulation during isometric muscle contraction in a healthy subject. Clin Neurophysiol. 2011;122(11):2326-7. Epub 2011/05/10. doi: 10.1016/j.clinph.2011.04.005. PubMed PMID: 21550843; PubMed Central PMCID: PMCPMC3372095.

291. Fitzgerald PB, Hoy KE, Maller JJ, Herring S, Segrave R, McQueen S, et al. Transcranial magnetic stimulation for depression after a traumatic brain injury: a case study. J ect. 2011;27(1):38-40. Epub 2010/10/13. doi: 10.1097/YCT.0b013e3181eb30c6. PubMed PMID: 20938348.

292. Gerretsen P, Diaz P, Mamo D, Kavanagh D, Menon M, Pollock BG, et al. Transient insight induction with electroconvulsive therapy in a patient with refractory schizophrenia: a case report and systematic literature review. J ect. 2011;27(3):247-50. Epub 2010/10/23. doi: 10.1097/YCT.0b013e3181f816f6. PubMed PMID: 20966768.

293. Hirnstein M, Bayer U, Ellison A, Hausmann M. TMS over the left angular gyrus impairs the ability to discriminate left from right. Neuropsychologia. 2011;49(1):29-33. Epub 2010/11/03. doi:

10.1016/j.neuropsychologia.2010.10.028. PubMed PMID: 21035475.

294. Holt RL, Mikati MA. Care for child development: basic science rationale and effects of interventions. Pediatr Neurol. 2011;44(4):239-53. Epub 2011/03/15. doi: 10.1016/j.pediatrneurol.2010.11.009. PubMed PMID: 21397164.

295. Kratz O, Studer P, Barth W, Wangler S, Hoegl T, Heinrich H, et al. Seizure in a nonpredisposed individual induced by single-pulse transcranial magnetic stimulation. J ect. 2011;27(1):48-50. Epub 2010/03/31. doi: 10.1097/YCT.0b013e3181d2ef85. PubMed PMID: 20351571.

296. Kwon HG, Lee DG, Choi BY, Chang CH, Kim SH, Jang SH. Recovery of the corticospinal tract after injury by transtentorial herniation: a case report. NeuroRehabilitation. 2011;29(3):243-6. Epub 2011/12/07. doi: 10.3233/nre-2011-0699. PubMed PMID: 22142757.

297. Lamy JC, Boakye M. Non-invasive tools to promote spinal plasticity in humans. Clin Neurophysiol. 2011;122(11):2114-5. Epub 2011/04/26. doi: 10.1016/j.clinph.2011.03.024. PubMed PMID: 21514881.

298. Lee KY, Nyein MK, Moore DF, Joannopoulos JD, Socrate S, Imholt T, et al. Blast-induced electromagnetic fields in the brain

from bone piezoelectricity. Neuroimage. 2011;54 Suppl 1:S30-6. Epub 2010/06/16. doi: 10.1016/j.neuroimage.2010.05.042. PubMed PMID: 20547228.

299. León L, Casanova-Molla J, Lauria G, Valls-Solé J. The somatosensory blink reflex in upper and lower brainstem lesions. Muscle Nerve. 2011;43(2):196-202. Epub 2011/01/22. doi: 10.1002/mus.21810. PubMed PMID: 21254084.

300. Li S, Rymer WZ. Voluntary breathing influences corticospinal excitability of nonrespiratory finger muscles. J Neurophysiol. 2011;105(2):512-21. Epub 2010/12/17. doi: 10.1152/jn.00946.2010. PubMed PMID: 21160006; PubMed Central PMCID: PMCPMC3059159.

301. Liu MC, Kobeissy F, Zheng W, Zhang Z, Hayes RL, Wang KK. Dual vulnerability of tau to calpains and caspase-3 proteolysis under neurotoxic and neurodegenerative conditions. ASN Neuro. 2011;3(1):e00051. Epub 2011/03/02. doi: 10.1042/an20100012. PubMed PMID: 21359008; PubMed Central PMCID: PMCPMC3040574.

302. Miller BT, Vytlacil J, Fegen D, Pradhan S, D'Esposito M. The prefrontal cortex modulates category selectivity in human extrastriate cortex. J Cogn Neurosci. 2011;23(1):1-10. Epub 2010/07/01. doi: 10.1162/jocn.2010.21516. PubMed PMID:

20586702.

303. Miniussi C, Rossini PM. Transcranial magnetic stimulation in cognitive rehabilitation. Neuropsychol Rehabil. 2011;21(5):579-601. Epub 2011/04/05. doi: 10.1080/09602011.2011.562689. PubMed PMID: 21462081.

304. Miniussi C, Vallar G. Brain stimulation and behavioural cognitive rehabilitation: a new tool for neurorehabilitation? Neuropsychol Rehabil. 2011;21(5):553-9. Epub 2011/10/21. doi: 10.1080/09602011.2011.622435. PubMed PMID: 22011015.

305. Mishra NK, Russmann H, Granziera C, Maeder P, Annoni JM. Mutism and amnesia following high-voltage electrical injury: psychogenic symptomatology triggered by organic dysfunction? Eur Neurol. 2011;66(4):229-34. Epub 2011/09/29. doi: 10.1159/000330953. PubMed PMID: 21952143.

306. Nardone R, Bergmann J, Kunz A, Caleri F, Seidl M, Tezzon F, et al. Cortical excitability changes in patients with sleep-wake disturbances after traumatic brain injury. J Neurotrauma. 2011;28(7):1165-71. Epub 2011/03/31. doi: 10.1089/neu.2010.1748. PubMed PMID: 21446790.

307. Pachalska M, Łukowicz M, Kropotov JD, Herman-Sucharska I, Talar J. Evaluation of differentiated neurotherapy programs for a patient after severe TBI and long term coma using event-related

potentials. Med Sci Monit. 2011;17(10):Cs120-8. Epub 2011/10/01. doi: 10.12659/msm.881970. PubMed PMID: 21959618; PubMed Central PMCID: PMCPMC3539468.

308. Paulus W. Transcranial electrical stimulation (tES - tDCS; tRNS, tACS) methods. Neuropsychol Rehabil. 2011;21(5):602-17. Epub 2011/08/09. doi: 10.1080/09602011.2011.557292. PubMed PMID: 21819181.

309. Piccione F, Cavinato M, Manganotti P, Formaggio E, Storti SF, Battistin L, et al. Behavioral and neurophysiological effects of repetitive transcranial magnetic stimulation on the minimally conscious state: a case study. Neurorehabil Neural Repair. 2011;25(1):98-102. Epub 2010/07/22. doi: 10.1177/1545968310369802. PubMed PMID: 20647501.

310. Reddy GS, Sujatha M. A Rare Case of Short-Chain Acyl-COA Dehydrogenase Deficiency: The Apparent Rarity of the Disorder Results in Under Diagnosis. Indian J Clin Biochem. 2011;26(3):312-5. Epub 2012/07/04. doi: 10.1007/s12291-011-0139-x. PubMed PMID: 22754199; PubMed Central PMCID: PMCPMC3162956.

311. Renton T, Yilmaz Z. Profiling of patients presenting with posttraumatic neuropathy of the trigeminal nerve. J Orofac Pain. 2011;25(4):333-44. Epub 2012/01/17. PubMed PMID: 22247929.

312. Seo MH, Park SH, Ko MH, Seo JH. Motor evoked potentials of trunk muscles in traumatic brain injury patients. Ann Rehabil Med. 2011;35(4):557-64. Epub 2012/04/17. doi: 10.5535/arm.2011.35.4.557. PubMed PMID: 22506173; PubMed Central PMCID: PMCPMC3309240.

313. Smith M. Shedding light on the adult brain: a review of the clinical applications of near-infrared spectroscopy. Philos Trans A Math Phys Eng Sci. 2011;369(1955):4452-69. Epub 2011/10/19. doi: 10.1098/rsta.2011.0242. PubMed PMID: 22006901.

314. Sokal P, Harat M, Paczkowski D, Rudaś M, Birski M, Litwinowicz A. Results of neuromodulation for the management of chronic pain. Neurol Neurochir Pol. 2011;45(5):445-51. Epub 2011/12/01. doi: 10.1016/s0028-3843(14)60312-1. PubMed PMID: 22127939.

315. Song XJ, Luo MF, Jiang J, Zhang JL. [Effect of acupoint-injection of stem cell factor antibody on electroacupuncture induced changes of count and degranulation of subcutaneous mast cells in the local region in rats]. Zhen Ci Yan Jiu. 2011;36(4):247-51. Epub 2011/09/29. PubMed PMID: 21942176.

316. Strzelczyk A, Kepenek Y, Rindock H, Müller-Mazzotta J, Oertel WH, Mylius V, et al. Benign paroxysmal positional vertigo

following diagnostic transcranial magnetic stimulation. Neurol Sci. 2011;32(3):503-5. Epub 2011/03/05. doi: 10.1007/s10072-011-0511-x. PubMed PMID: 21374092.

317. Supp GG, Siegel M, Hipp JF, Engel AK. Cortical hypersynchrony predicts breakdown of sensory processing during loss of consciousness. Curr Biol. 2011;21(23):1988-93. Epub 2011/11/22. doi: 10.1016/j.cub.2011.10.017. PubMed PMID: 22100063.

318. Tanaka S, Sandrini M, Cohen LG. Modulation of motor learning and memory formation by non-invasive cortical stimulation of the primary motor cortex. Neuropsychol Rehabil. 2011;21(5):650-75. Epub 2011/09/29. doi: 10.1080/09602011.2011.605589. PubMed PMID: 21942897.

319. Tremblay S, de Beaumont L, Lassonde M, Théoret H. Evidence for the specificity of intracortical inhibitory dysfunction in asymptomatic concussed athletes. J Neurotrauma. 2011;28(4):493-502. Epub 2011/01/12. doi: 10.1089/neu.2010.1615. PubMed PMID: 21219259.

320. Wei JW, Huang Y, Wang JG, Liu M, Wong LK, Huang Q, et al. Current management of intracerebral haemorrhage in China: a national, multi-centre, hospital register study. BMC Neurol. 2011;11:16. Epub 2011/02/01. doi: 10.1186/1471-2377-11-16.

PubMed PMID: 21276264; PubMed Central PMCID: PMCPMC3040709.
321. Yoo SS, Kim H, Min BK, Franck E, Park S. Transcranial focused ultrasound to the thalamus alters anesthesia time in rats. Neuroreport. 2011;22(15):783-7. Epub 2011/08/31. doi: 10.1097/WNR.0b013e32834b2957. PubMed PMID: 21876461; PubMed Central PMCID: PMCPMC3174273.
322. Zhang Y, Su YY, Ye H, Xiao SY, Chen WB, Zhao JW. Predicting comatose patients with acute stroke outcome using middle-latency somatosensory evoked potentials. Clin Neurophysiol. 2011;122(8):1645-9. Epub 2011/02/15. doi: 10.1016/j.clinph.2010.11.016. PubMed PMID: 21317029.
323. Zhang YJ, Li JZ. [Severe sensorineural deafness after cerebral trauma by electroacupuncture and scalp acupuncture: a report of 19 cases]. Zhongguo Zhong Xi Yi Jie He Za Zhi. 2011;31(10):1427-8. Epub 2011/11/22. PubMed PMID: 22097221.
324. Zhou F, Guo J, Cheng J, Wu G, Xia Y. Electroacupuncture increased cerebral blood flow and reduced ischemic brain injury: dependence on stimulation intensity and frequency. J Appl Physiol (1985). 2011;111(6):1877-87. Epub 2011/08/13. doi: 10.1152/japplphysiol.00313.2011. PubMed PMID: 21836043; PubMed Central PMCID: PMCPMC3233896.

325. Arendash GW. Transcranial electromagnetic treatment against Alzheimer's disease: why it has the potential to trump Alzheimer's disease drug development. J Alzheimers Dis. 2012;32(2):243-66. Epub 2012/07/20. doi: 10.3233/jad-2012-120943. PubMed PMID: 22810103.

326. Auxemery Y. Etiopathogenic perspectives on chronic psycho traumatic and chronic psychotic symptoms: the hypothesis of a hyperdopaminergic endophenotype of PTSD. Med Hypotheses. 2012;79(5):667-72. Epub 2012/09/04. doi: 10.1016/j.mehy.2012.08.005. PubMed PMID: 22939767.

327. Auxéméry Y. [Posttraumatic stress disorder (PTSD) as a consequence of the interaction between an individual genetic susceptibility, a traumatogenic event and a social context]. Encephale. 2012;38(5):373-80. Epub 2012/10/16. doi: 10.1016/j.encep.2011.12.003. PubMed PMID: 23062450.

328. Bagnato S, Boccagni C, Sant'Angelo A, Prestandrea C, Rizzo S, Galardi G. Patients in a vegetative state following traumatic brain injury display a reduced intracortical modulation. Clin Neurophysiol. 2012;123(10):1937-41. Epub 2012/05/09. doi: 10.1016/j.clinph.2012.03.014. PubMed PMID: 22560638.

329. Bashir S, Vernet M, Yoo WK, Mizrahi I, Theoret H, Pascual-Leone A. Changes in cortical plasticity after mild

traumatic brain injury. Restor Neurol Neurosci. 2012;30(4):277-82. Epub 2012/05/19. doi: 10.3233/rnn-2012-110207. PubMed PMID: 22596356; PubMed Central PMCID: PMCPMC3951777.

330. Boly M, Massimini M, Garrido MI, Gosseries O, Noirhomme Q, Laureys S, et al. Brain connectivity in disorders of consciousness. Brain Connect. 2012;2(1):1-10. Epub 2012/04/20. doi: 10.1089/brain.2011.0049. PubMed PMID: 22512333.

331. Cavinato M, Iaia V, Piccione F. Repeated sessions of sub-threshold 20-Hz rTMS. Potential cumulative effects in a brain-injured patient. Clin Neurophysiol. 2012;123(9):1893-5. Epub 2012/03/13. doi: 10.1016/j.clinph.2012.02.066. PubMed PMID: 22405994.

332. De Beaumont L, Tremblay S, Poirier J, Lassonde M, Théoret H. Altered bidirectional plasticity and reduced implicit motor learning in concussed athletes. Cereb Cortex. 2012;22(1):112-21. Epub 2011/05/17. doi: 10.1093/cercor/bhr096. PubMed PMID: 21572090.

333. De Salvo S, Bramanti P, Marino S. Clinical differentiation and outcome evaluation in vegetative and minimally conscious state patients:the neurophysiological approach. Funct Neurol. 2012;27(3):155-62. Epub 2013/02/14. PubMed PMID: 23402676; PubMed Central PMCID: PMCPMC3812768.

334. Demirtas-Tatlidede A, Vahabzadeh-Hagh AM, Bernabeu M, Tormos JM, Pascual-Leone A. Noninvasive brain stimulation in traumatic brain injury. J Head Trauma Rehabil. 2012;27(4):274-92. Epub 2011/06/22. doi: 10.1097/HTR.0b013e318217df55. PubMed PMID: 21691215; PubMed Central PMCID: PMCPMC3342413.
335. Dumas R, Richieri R, Guedj E, Auquier P, Lancon C, Boyer L. Improvement of health-related quality of life in depression after transcranial magnetic stimulation in a naturalistic trial is associated with decreased perfusion in precuneus. Health Qual Life Outcomes. 2012;10:87. Epub 2012/07/31. doi: 10.1186/1477-7525-10-87. PubMed PMID: 22838740; PubMed Central PMCID: PMCPMC3492154.
336. Golaszewski SM, Bergmann J, Christova M, Kunz AB, Kronbichler M, Rafolt D, et al. Modulation of motor cortex excitability by different levels of whole-hand afferent electrical stimulation. Clin Neurophysiol. 2012;123(1):193-9. Epub 2011/07/19. doi: 10.1016/j.clinph.2011.06.010. PubMed PMID: 21764634.
337. Hadar AA, Makris S, Yarrow K. Single-pulse TMS related syncopal spell in a healthy subject. Brain Stimul. 2012;5(4):652-3. Epub 2011/09/21. doi: 10.1016/j.brs.2011.08.001. PubMed PMID: 21930450.

338. Inoue M, Kojima Y, Mima T, Sawamoto N, Matsuhashi M, Fumuro T, et al. Pathophysiology of unilateral asterixis due to thalamic lesion. Clin Neurophysiol. 2012;123(9):1858-64. Epub 2012/03/20. doi: 10.1016/j.clinph.2012.01.021. PubMed PMID: 22425586.

339. Ivarsen A, Hjortdal J. Seven-year changes in corneal power and aberrations after PRK or LASIK. Invest Ophthalmol Vis Sci. 2012;53(10):6011-6. Epub 2012/08/11. doi: 10.1167/iovs.12-10208. PubMed PMID: 22879417.

340. Kang EK, Kim DY, Paik NJ. Transcranial direct current stimulation of the left prefrontal cortex improves attention in patients with traumatic brain injury: a pilot study. J Rehabil Med. 2012;44(4):346-50. Epub 2012/03/22. doi: 10.2340/16501977-0947. PubMed PMID: 22434324.

341. Kantak SS, Stinear JW, Buch ER, Cohen LG. Rewiring the brain: potential role of the premotor cortex in motor control, learning, and recovery of function following brain injury. Neurorehabil Neural Repair. 2012;26(3):282-92. Epub 2011/09/20. doi: 10.1177/1545968311420845. PubMed PMID: 21926382; PubMed Central PMCID: PMCPMC4886541.

342. Kawasaki Y, Fujiki M, Ooba H, Sugita K, Hikawa T, Abe T, et al. Short latency afferent inhibition associated with cortical

compression and memory impairment in patients with chronic subdural hematoma. Clin Neurol Neurosurg. 2012;114(7):976-80. Epub 2012/04/10. doi: 10.1016/j.clineuro.2012.02.037. PubMed PMID: 22482870.

343. Krishnan M, Smith N, Donders J. Use of the Tower of London - Drexel University, Second Edition (TOLDX) in adults with traumatic brain injury. Clin Neuropsychol. 2012;26(6):951-64. Epub 2012/08/02. doi: 10.1080/13854046.2012.708166. PubMed PMID: 22849344.

344. Livingston SC, Goodkin HP, Hertel JN, Saliba EN, Barth JT, Ingersoll CD. Differential rates of recovery after acute sport-related concussion: electrophysiologic, symptomatic, and neurocognitive indices. J Clin Neurophysiol. 2012;29(1):23-32. Epub 2012/02/23. doi: 10.1097/WNP.0b013e318246ae46. PubMed PMID: 22353982.

345. Mandat T, Koziara H, Barszcz S, Rola R, Karliński M, Sliwińska A, et al. Motor cortex stimulation in the treatment of neuropathic pain. Neurol Neurochir Pol. 2012;46(5):428-35. Epub 2012/11/20. doi: 10.5114/ninp.2012.31352. PubMed PMID: 23161186.

346. Massimini M, Ferrarelli F, Sarasso S, Tononi G. Cortical mechanisms of loss of consciousness: insight from TMS/EEG studies. Arch Ital Biol. 2012;150(2-3):44-55. Epub 2012/11/21. doi:

10.4449/aib.v150i2.1361. PubMed PMID: 23165870.

347. Monsalve GA. Motor cortex stimulation for facial chronic neuropathic pain: A review of the literature. Surg Neurol Int. 2012;3(Suppl 4):S290-311. Epub 2012/12/12. doi: 10.4103/2152-7806.103023. PubMed PMID: 23230534; PubMed Central PMCID: PMCPMC3514920.

348. Mylius V, Ayache SS, Teepker M, Kappus C, Kolodziej M, Rosenow F, et al. [Transcranial magnetic stimulation and motor cortex stimulation in neuropathic pain]. Schmerz. 2012;26(6):655-60. Epub 2012/11/28. doi: 10.1007/s00482-012-1243-4. PubMed PMID: 23183989.

349. Nizard J, Lefaucheur JP, Helbert M, de Chauvigny E, Nguyen JP. Non-invasive stimulation therapies for the treatment of refractory pain. Discov Med. 2012;14(74):21-31. Epub 2012/08/01. PubMed PMID: 22846200.

350. Nizard J, Raoul S, Nguyen JP, Lefaucheur JP. Invasive stimulation therapies for the treatment of refractory pain. Discov Med. 2012;14(77):237-46. Epub 2012/11/02. PubMed PMID: 23114579.

351. Oliviero A, Arevalo-Martin A, Rotondi M, García-Ovejero D, Mordillo-Mateos L, Lozano-Sicilia A, et al. CB1 receptor antagonism/inverse agonism increases motor system excitability in

humans. Eur Neuropsychopharmacol. 2012;22(1):27-35. Epub 2011/05/17. doi: 10.1016/j.euroneuro.2011.04.004. PubMed PMID: 21571507.

352. Plow EB, Pascual-Leone A, Machado A. Brain stimulation in the treatment of chronic neuropathic and non-cancerous pain. J Pain. 2012;13(5):411-24. Epub 2012/04/10. doi: 10.1016/j.jpain.2012.02.001. PubMed PMID: 22484179; PubMed Central PMCID: PMCPMC3348447.

353. Railo H, Salminen-Vaparanta N, Henriksson L, Revonsuo A, Koivisto M. Unconscious and conscious processing of color rely on activity in early visual cortex: a TMS study. J Cogn Neurosci. 2012;24(4):819-29. Epub 2011/12/01. doi: 10.1162/jocn_a_00172. PubMed PMID: 22126671.

354. Renton T, Yilmaz Z, Gaballah K. Evaluation of trigeminal nerve injuries in relation to third molar surgery in a prospective patient cohort. Recommendations for prevention. Int J Oral Maxillofac Surg. 2012;41(12):1509-18. Epub 2012/09/29. doi: 10.1016/j.ijom.2012.06.025. PubMed PMID: 23017786.

355. Rokyta R, Fricová J. Neurostimulation methods in the treatment of chronic pain. Physiol Res. 2012;61(Suppl 2):S23-31. Epub 2012/11/21. doi: 10.33549/physiolres.932392. PubMed PMID: 23130900.

356. Rosanova M, Gosseries O, Casarotto S, Boly M, Casali AG, Bruno MA, et al. Recovery of cortical effective connectivity and recovery of consciousness in vegetative patients. Brain. 2012;135(Pt 4):1308-20. Epub 2012/01/10. doi: 10.1093/brain/awr340. PubMed PMID: 22226806; PubMed Central PMCID: PMCPMC3326248.

357. Rothwell JC. Clinical applications of noninvasive electrical stimulation: problems and potential. Clin EEG Neurosci. 2012;43(3):209-14. Epub 2012/06/21. doi: 10.1177/1550059412444973. PubMed PMID: 22715492.

358. Sharova EV, Mel'nikov AV, Novikova MR, Kulikov MA, Mats VN, Shishkina LV, et al. [Functional effects of transcranial electromagnetic stimulation (60 and 70 Hz) of the brain of intact rats and rats with acute brainstem damage]. Zh Vyssh Nerv Deiat Im I P Pavlova. 2012;62(1):65-78. Epub 2012/05/10. PubMed PMID: 22567988.

359. Stidd DA, Wuollet AL, Bowden K, Price T, Patwardhan A, Barker S, et al. Peripheral nerve stimulation for trigeminal neuropathic pain. Pain Physician. 2012;15(1):27-33. Epub 2012/01/25. PubMed PMID: 22270735; PubMed Central PMCID: PMCPMC4664452.

360. Tallus J, Lioumis P, Hämäläinen H, Kähkönen S, Tenovuo O.

Long-lasting TMS motor threshold elevation in mild traumatic brain injury. Acta Neurol Scand. 2012;126(3):178-82. Epub 2011/11/23. doi: 10.1111/j.1600-0404.2011.01623.x. PubMed PMID: 22103909.

361. van Putten MJ. The N20 in post-anoxic coma: are you listening? Clin Neurophysiol. 2012;123(7):1460-4. Epub 2011/12/17. doi: 10.1016/j.clinph.2011.10.049. PubMed PMID: 22172767.

362. Villamar MF, Santos Portilla A, Fregni F, Zafonte R. Noninvasive brain stimulation to modulate neuroplasticity in traumatic brain injury. Neuromodulation. 2012;15(4):326-38. Epub 2012/08/14. doi: 10.1111/j.1525-1403.2012.00474.x. PubMed PMID: 22882244.

363. Wahab RA, Choi M, Liu Y, Krauthamer V, Zderic V, Myers MR. Mechanical bioeffects of pulsed high intensity focused ultrasound on a simple neural model. Med Phys. 2012;39(7):4274-83. Epub 2012/07/27. doi: 10.1118/1.4729712. PubMed PMID: 22830761.

364. Wang Q, Wang F, Li X, Yang Q, Li X, Xu N, et al. Electroacupuncture pretreatment attenuates cerebral ischemic injury through α7 nicotinic acetylcholine receptor-mediated inhibition of high-mobility group box 1 release in rats. J

Neuroinflammation. 2012;9:24. Epub 2012/01/27. doi: 10.1186/1742-2094-9-24. PubMed PMID: 22277256; PubMed Central PMCID: PMCPMC3297509.

365. Xiang YM, Hao CH, Wang J. [Electroacupuncture combined with acupoint injection for 40 cases of comatose due to craniocerebral trauma]. Zhongguo Zhen Jiu. 2012;32(5):475-6. Epub 2012/06/02. PubMed PMID: 22650144.

366. Xie Y, Zhang T. Repetitive transcranial magnetic stimulation improves consciousness disturbance in stroke patients: A quantitative electroencephalography spectral power analysis. Neural Regen Res. 2012;7(31):2465-72. Epub 2012/11/05. doi: 10.3969/j.issn.1673-5374.2012.31.008. PubMed PMID: 25337097; PubMed Central PMCID: PMCPMC4200721.

367. Zakrison TL, Pereira BM, Marttos AC, Jr., Fraga GP, Nascimento B, Jr., Rizoli S. Venous thromboembolism prophylaxis in patients with traumatic brain injury. Rev Col Bras Cir. 2012;39(6):553-7. Epub 2013/01/26. doi: 10.1590/s0100-69912012000600018. PubMed PMID: 23348655.

368. Zhang W, Sun YH, Shi QW, Peng XH, Yang G, Li Y. [Effect of electroacupuncture on ultrastructural changes of facial nerve in rabbits with facial nerve injury]. Zhen Ci Yan Jiu. 2012;37(4):296-301. Epub 2012/11/13. PubMed PMID: 23140051.

369. Andrade C. Once- to twice-daily, 3-year domiciliary maintenance transcranial direct current stimulation for severe, disabling, clozapine-refractory continuous auditory hallucinations in schizophrenia. J ect. 2013;29(3):239-42. Epub 2013/02/05. doi: 10.1097/YCT.0b013e3182843866. PubMed PMID: 23377748.

370. Andrade DC, Borges I, Bravo GL, Bolognini N, Fregni F. Therapeutic time window of noninvasive brain stimulation for pain treatment: inhibition of maladaptive plasticity with early intervention. Expert Rev Med Devices. 2013;10(3):339-52. Epub 2013/05/15. doi: 10.1586/erd.12.90. PubMed PMID: 23668706.

371. Bezuidenhout AF, Hurter D, Maydell AT, van Niekerk F, de Figueiredo SA, Harvey J, et al. The Kimberley Hospital Rule (KHR) for urgent computed tomography of the brain in a resource-limited environment. S Afr Med J. 2013;103(9):646-51. Epub 2013/12/05. doi: 10.7196/samj.6876. PubMed PMID: 24300685.

372. Bhalerao SU, Geurtjens C, Thomas GR, Kitamura CR, Zhou C, Marlborough M. Understanding the neuropsychiatric consequences associated with significant traumatic brain injury. Brain Inj. 2013;27(7-8):767-74. Epub 2013/06/26. doi: 10.3109/02699052.2013.793396. PubMed PMID: 23789861.

373. Block CK, West SE. Psychotherapeutic treatment of survivors

of traumatic brain injury: review of the literature and special considerations. Brain Inj. 2013;27(7-8):775-88. Epub 2013/05/02. doi: 10.3109/02699052.2013.775487. PubMed PMID: 23631508.

374. Bodart O, Laureys S, Gosseries O. Coma and disorders of consciousness: scientific advances and practical considerations for clinicians. Semin Neurol. 2013;33(2):83-90. Epub 2013/07/28. doi: 10.1055/s-0033-1348965. PubMed PMID: 23888393.

375. Bonnì S, Mastropasqua C, Bozzali M, Caltagirone C, Koch G. Theta burst stimulation improves visuo-spatial attention in a patient with traumatic brain injury. Neurol Sci. 2013;34(11):2053-6. Epub 2013/03/28. doi: 10.1007/s10072-013-1412-y. PubMed PMID: 23532550.

376. Casali AG, Gosseries O, Rosanova M, Boly M, Sarasso S, Casali KR, et al. A theoretically based index of consciousness independent of sensory processing and behavior. Sci Transl Med. 2013;5(198):198ra05. Epub 2013/08/16. doi: 10.1126/scitranslmed.3006294. PubMed PMID: 23946194.

377. Chen W, Shan CL, Guo B, Yuan T. Mirror neuron system activation for conscious states assessment: for patients in vegetative and minimally conscious states. Med Hypotheses. 2013;81(1):144-5. Epub 2013/05/07. doi: 10.1016/j.mehy.2013.04.014. PubMed PMID: 23643706.

378. Chuang CH, Hsu YC, Wang CC, Hu C, Kuo JR. Cerebral blood flow and apoptosis-associated factor with electroacupuncture in a traumatic brain injury rat model. Acupunct Med. 2013;31(4):395-403. Epub 2013/09/24. doi: 10.1136/acupmed-2013-010406. PubMed PMID: 24055977.

379. Della Pepa GM, Fukaya C, La Rocca G, Zhong J, Visocchi M. Neuromodulation of vegetative state through spinal cord stimulation: where are we now and where are we going? Stereotact Funct Neurosurg. 2013;91(5):275-87. Epub 2013/06/26. doi: 10.1159/000348271. PubMed PMID: 23797266.

380. Furness S, Bryan G, McMillan R, Birchenough S, Worthington HV. Interventions for the management of dry mouth: non-pharmacological interventions. Cochrane Database Syst Rev. 2013;2013(9):Cd009603. Epub 2013/09/06. doi: 10.1002/14651858.CD009603.pub3. PubMed PMID: 24006231; PubMed Central PMCID: PMCPMC7100870 Worthington: no interests to declare. Roddy McMillan: no interests to declare. Sarah Birchenough: no interests to declare.

381. Gillebert CR, Mantini D. Functional connectivity in the normal and injured brain. Neuroscientist. 2013;19(5):509-22. Epub 2012/10/16. doi: 10.1177/1073858412463168. PubMed PMID: 23064084.

382. Henrich-Noack P, Lazik S, Sergeeva E, Wagner S, Voigt N, Prilloff S, et al. Transcorneal alternating current stimulation after severe axon damage in rats results in "long-term silent survivor" neurons. Brain Res Bull. 2013;95:7-14. Epub 2013/03/19. doi: 10.1016/j.brainresbull.2013.02.008. PubMed PMID: 23500179.

383. Héron A, Dubayle D. A focus on mast cells and pain. J Neuroimmunol. 2013;264(1-2):1-7. Epub 2013/10/16. doi: 10.1016/j.jneuroim.2013.09.018. PubMed PMID: 24125568.

384. Hömberg V. Neurorehabilitation approaches to facilitate motor recovery. Handb Clin Neurol. 2013;110:161-73. Epub 2013/01/15. doi: 10.1016/b978-0-444-52901-5.00014-9. PubMed PMID: 23312639.

385. Kim JH, Choi KH, Jang YJ, Kim HN, Bae SS, Choi BT, et al. Electroacupuncture preconditioning reduces cerebral ischemic injury via BDNF and SDF-1α in mice. BMC Complement Altern Med. 2013;13:22. Epub 2013/01/30. doi: 10.1186/1472-6882-13-22. PubMed PMID: 23356671; PubMed Central PMCID: PMCPMC3562247.

386. Kirton A. Modeling developmental plasticity after perinatal stroke: defining central therapeutic targets in cerebral palsy. Pediatr Neurol. 2013;48(2):81-94. Epub 2013/01/23. doi: 10.1016/j.pediatrneurol.2012.08.001. PubMed PMID: 23337000.

387. Kojovic M, Pareés I, Kassavetis P, Palomar FJ, Mir P, Teo JT, et al. Secondary and primary dystonia: pathophysiological differences. Brain. 2013;136(Pt 7):2038-49. Epub 2013/06/19. doi: 10.1093/brain/awt150. PubMed PMID: 23771342.
388. Kreuzer PM, Landgrebe M, Frank E, Langguth B. Repetitive transcranial magnetic stimulation for the treatment of chronic tinnitus after traumatic brain injury: a case study. J Head Trauma Rehabil. 2013;28(5):386-9. Epub 2012/06/13. doi: 10.1097/HTR.0b013e318254736e. PubMed PMID: 22688213.
389. Lapitskaya N, Gosseries O, De Pasqua V, Pedersen AR, Nielsen JF, de Noordhout AM, et al. Abnormal corticospinal excitability in patients with disorders of consciousness. Brain Stimul. 2013;6(4):590-7. Epub 2013/02/14. doi: 10.1016/j.brs.2013.01.002. PubMed PMID: 23403267.
390. Lapitskaya N, Moerk SK, Gosseries O, Nielsen JF, de Noordhout AM. Corticospinal excitability in patients with anoxic, traumatic, and non-traumatic diffuse brain injury. Brain Stimul. 2013;6(2):130-7. Epub 2012/05/01. doi: 10.1016/j.brs.2012.03.010. PubMed PMID: 22542389.
391. Lee N, Rydyznski CE, Spearry RP, Robitz R, Maclennan AJ. The contribution of ciliary neurotrophic factor receptors to adult motor neuron survival in vivo is specific to insult type and distinct

from that for embryonic motor neurons. J Comp Neurol. 2013;521(14):3217-25. Epub 2013/05/23. doi: 10.1002/cne.23341. PubMed PMID: 23695797; PubMed Central PMCID: PMCPMC3986355.

392. Lefaucheur JP. Pain. Handb Clin Neurol. 2013;116:423-40. Epub 2013/10/12. doi: 10.1016/b978-0-444-53497-2.00035-8. PubMed PMID: 24112914.

393. Lloyd DA, Abrahamyan A, Harris JA. Brain-stimulation induced blindsight: unconscious vision or response bias? PLoS One. 2013;8(12):e82828. Epub 2013/12/11. doi: 10.1371/journal.pone.0082828. PubMed PMID: 24324837; PubMed Central PMCID: PMCPMC3855787.

394. Louppe JM, Nguyen JP, Robert R, Buffenoir K, de Chauvigny E, Riant T, et al. Motor cortex stimulation in refractory pelvic and perineal pain: report of two successful cases. Neurourol Urodyn. 2013;32(1):53-7. Epub 2012/06/08. doi: 10.1002/nau.22269. PubMed PMID: 22674567.

395. Manganotti P, Formaggio E, Storti SF, Fiaschi A, Battistin L, Tonin P, et al. Effect of high-frequency repetitive transcranial magnetic stimulation on brain excitability in severely brain-injured patients in minimally conscious or vegetative state. Brain Stimul. 2013;6(6):913-21. Epub 2013/08/10. doi:

10.1016/j.brs.2013.06.006. PubMed PMID: 23928101.

396. Megha M, Harpreet S, Nayeem Z. Effect of frequency of multimodal coma stimulation on the consciousness levels of traumatic brain injury comatose patients. Brain Inj. 2013;27(5):570-7. Epub 2013/03/12. doi: 10.3109/02699052.2013.767937. PubMed PMID: 23473238.

397. Nguyen J, Brown J, Mold JW, Welborn TL. In patients with concussions, is the probability of permanent neurological damage predicted better by total number of concussions than by severity and duration of individual concussions? J Okla State Med Assoc. 2013;106(11):431-2. Epub 2014/03/07. PubMed PMID: 24596983; PubMed Central PMCID: PMCPMC4930876.

398. Pandi-Perumal SR, BaHammam AS, Brown GM, Spence DW, Bharti VK, Kaur C, et al. Melatonin antioxidative defense: therapeutical implications for aging and neurodegenerative processes. Neurotox Res. 2013;23(3):267-300. Epub 2012/06/29. doi: 10.1007/s12640-012-9337-4. PubMed PMID: 22739839.

399. Papuć E, Rejdak K. The role of neurostimulation in the treatment of neuropathic pain. Ann Agric Environ Med. 2013;Spec no. 1:14-7. Epub 2014/07/09. PubMed PMID: 25000835.

400. Peters KM, Killinger KA, Gilleran J, Boura JA. Does patient age impact outcomes of neuromodulation? Neurourol Urodyn.

2013;32(1):30-6. Epub 2012/06/08. doi: 10.1002/nau.22268. PubMed PMID: 22674536.

401. Pinchuk D, Pinchuk O, Sirbiladze K, Shugar O. Clinical effectiveness of primary and secondary headache treatment by transcranial direct current stimulation. Front Neurol. 2013;4:25. Epub 2013/03/23. doi: 10.3389/fneur.2013.00025. PubMed PMID: 23519166; PubMed Central PMCID: PMCPMC3604631.

402. Pistoia F, Sacco S, Carolei A, Sarà M. Corticomotor facilitation in vegetative state: results of a pilot study. Arch Phys Med Rehabil. 2013;94(8):1599-606. Epub 2013/02/07. doi: 10.1016/j.apmr.2013.01.019. PubMed PMID: 23385107.

403. Ragazzoni A, Pirulli C, Veniero D, Feurra M, Cincotta M, Giovannelli F, et al. Vegetative versus minimally conscious states: a study using TMS-EEG, sensory and event-related potentials. PLoS One. 2013;8(2):e57069. Epub 2013/03/06. doi: 10.1371/journal.pone.0057069. PubMed PMID: 23460826; PubMed Central PMCID: PMCPMC3584112.

404. Reyns N, Derambure P, Duhamel A, Bourriez JL, Blond S, Houdayer E. Motor cortex stimulation modulates defective central beta rhythms in patients with neuropathic pain. Clin Neurophysiol. 2013;124(4):761-9. Epub 2012/11/16. doi: 10.1016/j.clinph.2012.10.011. PubMed PMID: 23151426.

405. Rushmore RJ, DeSimone C, Valero-Cabré A. Multiple sessions of transcranial direct current stimulation to the intact hemisphere improves visual function after unilateral ablation of visual cortex. Eur J Neurosci. 2013;38(12):3799-807. Epub 2013/10/15. doi: 10.1111/ejn.12373. PubMed PMID: 24118563; PubMed Central PMCID: PMCPMC3934645.
406. Rutiku R, Einberg A, Imanaka K, Bachmann T. The effect of task-irrelevant visual backgrounds on human transcranial magnetic stimulation-evoked electroencephalography responses and cortical alpha activity. Eur J Neurosci. 2013;38(12):3768-77. Epub 2013/10/15. doi: 10.1111/ejn.12374. PubMed PMID: 24118584.
407. Saitoh Y, Maruo T, Yokoe M, Matsuzaki T, Sekino M. Electrical or repetitive transcranial magnetic stimulation of primary motor cortex for intractable neuropathic pain. Annu Int Conf IEEE Eng Med Biol Soc. 2013;2013:6163-6. Epub 2013/10/11. doi: 10.1109/embc.2013.6610960. PubMed PMID: 24111147.
408. Sawicki M, Bohatyrewicz R, Safranow K, Walecka A, Walecki J, Rowinski O, et al. Dynamic evaluation of stasis filling phenomenon with computed tomography in diagnosis of brain death. Neuroradiology. 2013;55(9):1061-9. Epub 2013/06/04. doi: 10.1007/s00234-013-1210-5. PubMed PMID: 23728070; PubMed Central PMCID: PMCPMC3761089.

409. Sczesny-Kaiser M, Höffken O, Tegenthoff M, Schwenkreis P. Convulsive syncope after single-pulse TMS. Brain Stimul. 2013;6(5):830. Epub 2013/03/07. doi: 10.1016/j.brs.2013.01.018. PubMed PMID: 23462345.

410. Shear DA, Tortella FC. A military-centered approach to neuroprotection for traumatic brain injury. Front Neurol. 2013;4:73. Epub 2013/06/20. doi: 10.3389/fneur.2013.00073. PubMed PMID: 23781213; PubMed Central PMCID: PMCPMC3679469.

411. Steinberg H. A pioneer work on electric brain stimulation in psychotic patients. Rudolph Gottfried Arndt and his 1870s studies. Brain Stimul. 2013;6(4):477-81. Epub 2012/12/26. doi: 10.1016/j.brs.2012.11.004. PubMed PMID: 23266132.

412. Tallus J, Lioumis P, Hämäläinen H, Kähkönen S, Tenovuo O. Transcranial magnetic stimulation-electroencephalography responses in recovered and symptomatic mild traumatic brain injury. J Neurotrauma. 2013;30(14):1270-7. Epub 2013/02/07. doi: 10.1089/neu.2012.2760. PubMed PMID: 23384582; PubMed Central PMCID: PMCPMC3713436.

413. Tan T, Xie J, Liu T, Chen X, Zheng X, Tong Z, et al. Low-frequency (1 Hz) repetitive transcranial magnetic stimulation (rTMS) reverses Aβ(1-42)-mediated memory deficits in rats. Exp Gerontol. 2013;48(8):786-94. Epub 2013/05/15. doi:

10.1016/j.exger.2013.05.001. PubMed PMID: 23665072.

414. Tanaka Y, Matsuwaki T, Yamanouchi K, Nishihara M. Increased lysosomal biogenesis in activated microglia and exacerbated neuronal damage after traumatic brain injury in progranulin-deficient mice. Neuroscience. 2013;250:8-19. Epub 2013/07/09. doi: 10.1016/j.neuroscience.2013.06.049. PubMed PMID: 23830905.

415. Tran MT, Skovbjerg S, Arendt-Nielsen L, Christensen KB, Elberling J. Transcranial pulsed electromagnetic fields for multiple chemical sensitivity: study protocol for a randomized, double-blind, placebo-controlled trial. Trials. 2013;14:256. Epub 2013/08/21. doi: 10.1186/1745-6215-14-256. PubMed PMID: 23947742; PubMed Central PMCID: PMCPMC3765111.

416. Velazquez R, Ash JA, Powers BE, Kelley CM, Strawderman M, Luscher ZI, et al. Maternal choline supplementation improves spatial learning and adult hippocampal neurogenesis in the Ts65Dn mouse model of Down syndrome. Neurobiol Dis. 2013;58:92-101. Epub 2013/05/07. doi: 10.1016/j.nbd.2013.04.016. PubMed PMID: 23643842; PubMed Central PMCID: PMCPMC4029409.

417. Wong V, Cheuk DK, Lee S, Chu V. Acupuncture for acute management and rehabilitation of traumatic brain injury. Cochrane Database Syst Rev. 2013(3):Cd007700. Epub 2013/04/02. doi:

10.1002/14651858.CD007700.pub3. PubMed PMID: 23543554.
418. Yeo SS, Jang SH. Motor recovery via aberrant pyramidal tract in a patient with traumatic brain injury: A diffusion tensor tractography study. Neural Regen Res. 2013;8(1):90-4. Epub 2013/01/05. doi: 10.3969/j.issn.1673-5374.2013.01.012. PubMed PMID: 25206377; PubMed Central PMCID: PMCPMC4107495.
419. Yeo SS, Jang SH. Corticospinal tract recovery in a patient with traumatic transtentorial herniation. Neural Regen Res. 2013;8(5):469-73. Epub 2013/02/15. doi: 10.3969/j.issn.1673-5374.2013.05.011. PubMed PMID: 25206689; PubMed Central PMCID: PMCPMC4146130.
420. Yeo SS, Kim SH, Jang SH. Proximal weakness due to injury of the corticoreticular pathway in a patient with traumatic brain injury. NeuroRehabilitation. 2013;32(3):665-9. Epub 2013/05/08. doi: 10.3233/nre-130889. PubMed PMID: 23648621.
421. Zhang YM, Chen AL, Tang CZ, Zhang YQ, Yin HB, Chen SX. [Clinical observation on electroacupuncture for arousing consciousness of comatose patients with severe trauma brain injury]. Zhen Ci Yan Jiu. 2013;38(2):158-62. Epub 2013/07/04. PubMed PMID: 23819221.
422. Alampay MM, Haigney MC, Flanagan MC, Perito RM, Love KM, Grammer GG. Transcranial magnetic stimulation as an

antidepressant alternative in a patient with Brugada syndrome and recurrent syncope. Mayo Clin Proc. 2014;89(11):1584-7. Epub 2014/12/03. doi: 10.1016/j.mayocp.2014.08.010. PubMed PMID: 25444490.

423. Alhelal MA, Palaska I, Panagiotidou S, Letourneau R, Theoharides TC. Trigeminal nerve stimulation triggers oral mast cell activation and vascular permeability. Ann Allergy Asthma Immunol. 2014;112(1):40-5. Epub 2013/12/18. doi: 10.1016/j.anai.2013.10.011. PubMed PMID: 24331392.

424. André-Obadia N, Mertens P, Lelekov-Boissard T, Afif A, Magnin M, Garcia-Larrea L. Is Life better after motor cortex stimulation for pain control? Results at long-term and their prediction by preoperative rTMS. Pain Physician. 2014;17(1):53-62. Epub 2014/01/24. PubMed PMID: 24452645.

425. Angelakis E, Liouta E, Andreadis N, Korfias S, Ktonas P, Stranjalis G, et al. Transcranial direct current stimulation effects in disorders of consciousness. Arch Phys Med Rehabil. 2014;95(2):283-9. Epub 2013/09/17. doi: 10.1016/j.apmr.2013.09.002. PubMed PMID: 24035769.

426. Barlow KM, Brooks BL, MacMaster FP, Kirton A, Seeger T, Esser M, et al. A double-blind, placebo-controlled intervention trial of 3 and 10 mg sublingual melatonin for post-concussion syndrome

in youths (PLAYGAME): study protocol for a randomized controlled trial. Trials. 2014;15:271. Epub 2014/07/09. doi: 10.1186/1745-6215-15-271. PubMed PMID: 25001947; PubMed Central PMCID: PMCPMC4227124.

427. Barry MD, Boddington LJ, Igelström KM, Gray JP, Shemmell J, Tseng KY, et al. Utility of intracerebral theta burst electrical stimulation to attenuate interhemispheric inhibition and to promote motor recovery after cortical injury in an animal model. Exp Neurol. 2014;261:258-66. Epub 2014/06/07. doi: 10.1016/j.expneurol.2014.05.023. PubMed PMID: 24905955.

428. Brody AL, Okita K, Shieh J, Liang L, Hubert R, Mamoun M, et al. Radiation dosimetry and biodistribution of the translocator protein radiotracer [(11)C]DAA1106 determined with PET/CT in healthy human volunteers. Nucl Med Biol. 2014;41(10):871-5. Epub 2014/08/27. doi: 10.1016/j.nucmedbio.2014.07.004. PubMed PMID: 25156039; PubMed Central PMCID: PMCPMC4192057.

429. Castel-Lacanal E, Tarri M, Loubinoux I, Gasq D, de Boissezon X, Marque P, et al. Transcranial magnetic stimulation in brain injury. Ann Fr Anesth Reanim. 2014;33(2):83-7. Epub 2014/01/01. doi: 10.1016/j.annfar.2013.11.006. PubMed PMID: 24378049.

430. Cossu G. Therapeutic options to enhance coma arousal after

traumatic brain injury: state of the art of current treatments to improve coma recovery. Br J Neurosurg. 2014;28(2):187-98. Epub 2013/10/05. doi: 10.3109/02688697.2013.841845. PubMed PMID: 24090192.

431. Deer TR, Mekhail N, Petersen E, Krames E, Staats P, Pope J, et al. The appropriate use of neurostimulation: stimulation of the intracranial and extracranial space and head for chronic pain. Neuromodulation Appropriateness Consensus Committee. Neuromodulation. 2014;17(6):551-70; discussion 70. Epub 2014/08/13. doi: 10.1111/ner.12215. PubMed PMID: 25112890.

432. Di Perri C, Heine L, Amico E, Soddu A, Laureys S, Demertzi A. Technology-based assessment in patients with disorders of consciousness. Ann Ist Super Sanita. 2014;50(3):209-20. Epub 2014/10/09. doi: 10.4415/ann_14_03_03. PubMed PMID: 25292268.

433. Di Perri C, Stender J, Laureys S, Gosseries O. Functional neuroanatomy of disorders of consciousness. Epilepsy Behav. 2014;30:28-32. Epub 2013/10/09. doi: 10.1016/j.yebeh.2013.09.014. PubMed PMID: 24100252.

434. Di Perri C, Thibaut A, Heine L, Soddu A, Demertzi A, Laureys S. Measuring consciousness in coma and related states. World J Radiol. 2014;6(8):589-97. Epub 2014/08/30. doi:

10.4329/wjr.v6.i8.589. PubMed PMID: 25170396; PubMed Central PMCID: PMCPMC4147439.

435. Dougherty ET, Turner JC, Vogel F. Multiscale coupling of transcranial direct current stimulation to neuron electrodynamics: modeling the influence of the transcranial electric field on neuronal depolarization. Comput Math Methods Med. 2014;2014:360179. Epub 2014/11/19. doi: 10.1155/2014/360179. PubMed PMID: 25404950; PubMed Central PMCID: PMCPMC4227389.

436. Elahi F, Reddy C. High cervical epidural neurostimulation for post-traumatic headache management. Pain Physician. 2014;17(4):E537-41. Epub 2014/07/24. PubMed PMID: 25054404.

437. Fox MD, Buckner RL, Liu H, Chakravarty MM, Lozano AM, Pascual-Leone A. Resting-state networks link invasive and noninvasive brain stimulation across diverse psychiatric and neurological diseases. Proc Natl Acad Sci U S A. 2014;111(41):E4367-75. Epub 2014/10/01. doi: 10.1073/pnas.1405003111. PubMed PMID: 25267639; PubMed Central PMCID: PMCPMC4205651.

438. Fridman EA, Schiff ND. Neuromodulation of the conscious state following severe brain injuries. Curr Opin Neurobiol. 2014;29:172-7. Epub 2014/10/07. doi: 10.1016/j.conb.2014.09.008. PubMed PMID: 25285395; PubMed Central PMCID:

PMCPMC6519077.

439. George MS, Raman R, Benedek DM, Pelic CG, Grammer GG, Stokes KT, et al. A two-site pilot randomized 3 day trial of high dose left prefrontal repetitive transcranial magnetic stimulation (rTMS) for suicidal inpatients. Brain Stimul. 2014;7(3):421-31. Epub 2014/04/16. doi: 10.1016/j.brs.2014.03.006. PubMed PMID: 24731434.

440. Gosseries O, Thibaut A, Boly M, Rosanova M, Massimini M, Laureys S. Assessing consciousness in coma and related states using transcranial magnetic stimulation combined with electroencephalography. Ann Fr Anesth Reanim. 2014;33(2):65-71. Epub 2014/01/08. doi: 10.1016/j.annfar.2013.11.002. PubMed PMID: 24393302.

441. Guerra A, Costantini EM, Maatta S, Ponzo D, Ferreri F. Disorders of consciousness and electrophysiological treatment strategies: a review of the literature and new perspectives. Curr Pharm Des. 2014;20(26):4248-67. Epub 2013/09/13. PubMed PMID: 24025061.

442. Guller Y, Giacino J. Potential applications of concurrent transcranial magnetic stimulation and functional magnetic resonance imaging in acquired brain injury and disorders of consciousness. Brain Inj. 2014;28(9):1190-6. Epub 2014/08/08. doi:

10.3109/02699052.2014.920527. PubMed PMID: 25099023.

443. Herrold AA, Kletzel SL, Harton BC, Chambers RA, Jordan N, Pape TL. Transcranial magnetic stimulation: potential treatment for co-occurring alcohol, traumatic brain injury and posttraumatic stress disorders. Neural Regen Res. 2014;9(19):1712-30. Epub 2014/11/26. doi: 10.4103/1673-5374.143408. PubMed PMID: 25422632; PubMed Central PMCID: PMCPMC4238159.

444. Huang B, Zhou ZL, Wang LL, Zuo C, Lu Y, Chen Y. Electrical response grading versus House-Brackmann scale for evaluation of facial nerve injury after Bell's palsy: a comparative study. J Integr Med. 2014;12(4):367-71. Epub 2014/07/31. doi: 10.1016/s2095-4964(14)60036-4. PubMed PMID: 25074886.

445. Kronenburg A, van Doormaal T, van Eijsden P, van der Zwan A, Leijten F, Han KS. Surgery for a giant arteriovenous malformation without motor deterioration: preoperative transcranial magnetic stimulation in a non-cooperative patient. J Neurosurg Pediatr. 2014;14(1):38-42. Epub 2014/05/29. doi: 10.3171/2014.4.Peds13592. PubMed PMID: 24866824.

446. Lairamore CI, Garrison MK, Bourgeon L, Mennemeier M. Effects of functional electrical stimulation on gait recovery post-neurological injury during inpatient rehabilitation. Percept Mot Skills. 2014;119(2):591-608. Epub 2014/08/26. doi:

10.2466/15.25.PMS.119c19z5. PubMed PMID: 25153616; PubMed Central PMCID: PMCPMC4341891.

447. Lemaire JJ, Sontheimer A, Nezzar H, Pontier B, Luauté J, Roche B, et al. Electrical modulation of neuronal networks in brain-injured patients with disorders of consciousness: a systematic review. Ann Fr Anesth Reanim. 2014;33(2):88-97. Epub 2013/12/24. doi: 10.1016/j.annfar.2013.11.007. PubMed PMID: 24361283.

448. Leśniak M, Polanowska K, Seniów J, Członkowska A. Effects of repeated anodal tDCS coupled with cognitive training for patients with severe traumatic brain injury: a pilot randomized controlled trial. J Head Trauma Rehabil. 2014;29(3):E20-9. Epub 2013/06/13. doi: 10.1097/HTR.0b013e318292a4c2. PubMed PMID: 23756431.

449. Lipinski LJ, Van Gompel J, Smith J, Spinner RJ. Spinal accessory neuropathy following dual tunneling of lead extensions for motor cortex stimulation. Stereotact Funct Neurosurg. 2014;92(3):178-81. Epub 2014/06/20. doi: 10.1159/000360863. PubMed PMID: 24943465.

450. Lohse-Busch H, Reime U, Falland R. Symptomatic treatment of unresponsive wakefulness syndrome with transcranially focused extracorporeal shock waves. NeuroRehabilitation.

2014;35(2):235-44. Epub 2014/07/06. doi: 10.3233/nre-141115. PubMed PMID: 24990026.

451. López-Liria R, Fernández-Alonso M, Vega-Ramírez FA, Salido-Campos M, Padilla-Góngora D. [Treatment and rehabilitation of dysphagia following cerebrovascular disease]. Rev Neurol. 2014;58(6):259-67. Epub 2014/03/13. PubMed PMID: 24610693.

452. Maller JJ. Neuroplasticity in normal and brain injured patients: potential relevance of ear wiggling locus of control and cortical projections. Med Hypotheses. 2014;83(6):838-43. Epub 2014/12/04. doi: 10.1016/j.mehy.2014.11.003. PubMed PMID: 25468045.

453. Mason RA, Prat CS, Just MA. Neurocognitive brain response to transient impairment of Wernicke's area. Cereb Cortex. 2014;24(6):1474-84. Epub 2013/01/17. doi: 10.1093/cercor/bhs423. PubMed PMID: 23322403; PubMed Central PMCID: PMCPMC4014179.

454. Merlo L, Cimino F, Angileri FF, La Torre D, Conti A, Cardali SM, et al. Alteration in synaptic junction proteins following traumatic brain injury. J Neurotrauma. 2014;31(16):1375-85. Epub 2014/03/26. doi: 10.1089/neu.2014.3385. PubMed PMID: 24661152.

455. Middleton A, Fritz SL, Liuzzo DM, Newman-Norlund R, Herter TM. Using clinical and robotic assessment tools to examine the feasibility of pairing tDCS with upper extremity physical therapy in patients with stroke and TBI: a consideration-of-concept pilot study. NeuroRehabilitation. 2014;35(4):741-54. Epub 2014/10/18. doi: 10.3233/nre-141178. PubMed PMID: 25323084; PubMed Central PMCID: PMCPMC4268358.
456. Miller NR, Yasen AL, Maynard LF, Chou LS, Howell DR, Christie AD. Acute and longitudinal changes in motor cortex function following mild traumatic brain injury. Brain Inj. 2014;28(10):1270-6. Epub 2014/05/21. doi: 10.3109/02699052.2014.915987. PubMed PMID: 24841536.
457. Min KS, Lee CJ, Jun SB, Kim J, Lee SE, Shin J, et al. A liquid crystal polymer-based neuromodulation system: an application on animal model of neuropathic pain. Neuromodulation. 2014;17(2):160-9. Epub 2013/09/13. doi: 10.1111/ner.12093. PubMed PMID: 24024655.
458. Moore NZ, Lempka SF, Machado A. Central neuromodulation for refractory pain. Neurosurg Clin N Am. 2014;25(1):77-83. Epub 2013/11/23. doi: 10.1016/j.nec.2013.08.011. PubMed PMID: 24262901; PubMed Central PMCID: PMCPMC3867973.
459. Moreno-Duarte I, Morse LR, Alam M, Bikson M, Zafonte R,

Fregni F. Targeted therapies using electrical and magnetic neural stimulation for the treatment of chronic pain in spinal cord injury. Neuroimage. 2014;85 Pt 3:1003-13. Epub 2013/06/04. doi: 10.1016/j.neuroimage.2013.05.097. PubMed PMID: 23727533.
460. Napolitani M, Bodart O, Canali P, Seregni F, Casali A, Laureys S, et al. Transcranial magnetic stimulation combined with high-density EEG in altered states of consciousness. Brain Inj. 2014;28(9):1180-9. Epub 2014/08/08. doi: 10.3109/02699052.2014.920524. PubMed PMID: 25099022.
461. Nardone R, Höller Y, Leis S, Höller P, Thon N, Thomschewski A, et al. Invasive and non-invasive brain stimulation for treatment of neuropathic pain in patients with spinal cord injury: a review. J Spinal Cord Med. 2014;37(1):19-31. Epub 2013/10/05. doi: 10.1179/2045772313y.0000000140. PubMed PMID: 24090372; PubMed Central PMCID: PMCPMC4066547.
462. Naryshkin AG, Gorelik AL, Skoromets TA, Egorov A, Vtorov AV, Martynov IV. [On possibility of partial restore integrative brain activity in patients with vegetative state]. Fiziol Cheloveka. 2014;40(5):69-79. Epub 2015/02/26. PubMed PMID: 25711097.
463. Notaro P, Buratti E, Meroni A, Montagna MC, Rubino FG, Voltolini A. The effects of peripheral occipital nerve stimulation for the treatment of patients suffering from chronic migraine: a single

center experience. Pain Physician. 2014;17(3):E369-74. Epub 2014/05/23. PubMed PMID: 24850118.

464. Odinak MM, Zhivolupov SA, Panomarev VV, Rashidov NA, Samartsev IN. Recovery of consciousness as manifestation of neuroplasticity. Zh Vopr Neirokhir Im N N Burdenko. 2014;78(1):33-41; discussion Epub 2014/04/26. PubMed PMID: 24761594.

465. Ostergard T, Miller JP. Deep brain stimulation: new directions. J Neurosurg Sci. 2014;58(4):191-8. Epub 2014/11/25. PubMed PMID: 25418273.

466. Pan B, Liu J, Zhang Y, Sun Y, Wu Q, Zhao K, et al. Acute ablation of DP thymocytes induces up-regulation of IL-22 and Foxn1 in TECs. Clin Immunol. 2014;150(1):101-8. Epub 2013/12/18. doi: 10.1016/j.clim.2013.11.002. PubMed PMID: 24333537.

467. Papa L, LaMee A, Tan CN, Hill-Pryor C. Systematic review and meta-analysis of noninvasive cranial nerve neuromodulation for nervous system disorders. Arch Phys Med Rehabil. 2014;95(12):2435-43. Epub 2014/05/13. doi: 10.1016/j.apmr.2014.04.018. PubMed PMID: 24814561.

468. Pape TL, Rosenow JM, Patil V, Steiner M, Harton B, Guernon A, et al. RTMS safety for two subjects with disordered

consciousness after traumatic brain injury. Brain Stimul. 2014;7(4):620-2. Epub 2014/05/20. doi: 10.1016/j.brs.2014.03.007. PubMed PMID: 24836500.

469. Parmar VK, Gee L, Smith H, Pilitsis JG. Supraspinal stimulation for treatment of refractory pain. Clin Neurol Neurosurg. 2014;123:155-63. Epub 2014/06/24. doi: 10.1016/j.clineuro.2014.05.026. PubMed PMID: 24956545.

470. Pearce AJ, Hoy K, Rogers MA, Corp DT, Maller JJ, Drury HG, et al. The long-term effects of sports concussion on retired Australian football players: a study using transcranial magnetic stimulation. J Neurotrauma. 2014;31(13):1139-45. Epub 2014/03/04. doi: 10.1089/neu.2013.3219. PubMed PMID: 24579780.

471. Powers KC, Cinelli ME, Kalmar JM. Cortical hypoexcitability persists beyond the symptomatic phase of a concussion. Brain Inj. 2014;28(4):465-71. Epub 2014/04/08. doi: 10.3109/02699052.2014.888759. PubMed PMID: 24702432.

472. Railo H, Andersson E, Kaasinen V, Laine T, Koivisto M. Unlike in clinical blindsight patients, unconscious processing of chromatic information depends on early visual cortex in healthy humans. Brain Stimul. 2014;7(3):415-20. Epub 2014/04/05. doi: 10.1016/j.brs.2014.01.060. PubMed PMID: 24698972.

473. Ribeiro AS, Ramos A, Bermejo E, Casero M, Corrales JM, Grantham S. Effects of different musical stimuli in vital signs and facial expressions in patients with cerebral damage: a pilot study. J Neurosci Nurs. 2014;46(2):117-24. Epub 2014/02/22. doi: 10.1097/jnn.0000000000000037. PubMed PMID: 24556659.
474. Sarasso S, Rosanova M, Casali AG, Casarotto S, Fecchio M, Boly M, et al. Quantifying cortical EEG responses to TMS in (un)consciousness. Clin EEG Neurosci. 2014;45(1):40-9. Epub 2014/01/10. doi: 10.1177/1550059413513723. PubMed PMID: 24403317.
475. Shin DS, Hwang SC, Kim BT, Jeong JH, Im SB, Shin WH. Serial Brain CT Scans in Severe Head Injury without Intracranial Pressure Monitoring. Korean J Neurotrauma. 2014;10(1):26-30. Epub 2014/04/01. doi: 10.13004/kjnt.2014.10.1.26. PubMed PMID: 27169029; PubMed Central PMCID: PMCPMC4852589.
476. Shin SS, Dixon CE, Okonkwo DO, Richardson RM. Neurostimulation for traumatic brain injury. J Neurosurg. 2014;121(5):1219-31. Epub 2014/08/30. doi: 10.3171/2014.7.Jns131826. PubMed PMID: 25170668.
477. Silva D, Colvin L, Hagemann E, Stanley F, Bower C. Children diagnosed with attention deficit disorder and their hospitalisations: population data linkage study. Eur Child Adolesc Psychiatry.

2014;23(11):1043-50. Epub 2014/04/29. doi: 10.1007/s00787-014-0545-8. PubMed PMID: 24770488.

478. Song XJ, Luo MF, Jiang J, Zhang JL. [Effects of electroacupuncture stimulation of "Zusanli" (ST 36) on the regional vascular intercellular Adhesion Molecule-1 mRNA expression and mast cell activity in normal rats]. Zhen Ci Yan Jiu. 2014;39(6):461-5. Epub 2015/01/31. PubMed PMID: 25632570.

479. Sukul VV, Slavin KV. Deep brain and motor cortex stimulation. Curr Pain Headache Rep. 2014;18(7):427. Epub 2014/05/13. doi: 10.1007/s11916-014-0427-2. PubMed PMID: 24817154.

480. Sweet JA, Eakin KC, Munyon CN, Miller JP. Improved learning and memory with theta-burst stimulation of the fornix in rat model of traumatic brain injury. Hippocampus. 2014;24(12):1592-600. Epub 2014/08/05. doi: 10.1002/hipo.22338. PubMed PMID: 25087862.

481. Tang Y, Shang Q, Zhou LH. [Vegetative state treated with acupoint injection combined with plum-blossom needle in children: a randomized controlled trial]. Zhongguo Zhen Jiu. 2014;34(5):421-5. Epub 2014/07/16. PubMed PMID: 25022107.

482. Thibaut A, Bruno MA, Ledoux D, Demertzi A, Laureys S. tDCS in patients with disorders of consciousness: sham-controlled

randomized double-blind study. Neurology. 2014;82(13):1112-8. Epub 2014/02/28. doi: 10.1212/wnl.0000000000000260. PubMed PMID: 24574549.

483. Thibaut A, Bruno MA, Ledoux D, Demertzi A, Laureys S. tDCS in patients with disorders of consciousness: sham-controlled randomized double-blind study. Neurology. 2014;82(13):1112-8. Epub 2014/02/28. doi: 10.1212/wnl.0000000000000260. PubMed PMID: 24574549.

484. Treffert DA. Accidental genius. Sci Am. 2014;311(2):52-7. Epub 2014/08/07. doi: 10.1038/scientificamerican0814-52. PubMed PMID: 25095469.

485. Tremblay S, Beaulé V, Proulx S, Tremblay S, Marjańska M, Doyon J, et al. Multimodal assessment of primary motor cortex integrity following sport concussion in asymptomatic athletes. Clin Neurophysiol. 2014;125(7):1371-9. Epub 2014/01/28. doi: 10.1016/j.clinph.2013.11.040. PubMed PMID: 24462505; PubMed Central PMCID: PMCPMC4381958.

486. Urbanski M, Coubard OA, Bourlon C. Visualizing the blind brain: brain imaging of visual field defects from early recovery to rehabilitation techniques. Front Integr Neurosci. 2014;8:74. Epub 2014/10/18. doi: 10.3389/fnint.2014.00074. PubMed PMID: 25324739; PubMed Central PMCID: PMCPMC4179723.

487. Whyte J. Disorders of consciousness: the changing landscape of treatment. Neurology. 2014;82(13):1106-7. Epub 2014/02/28. doi: 10.1212/wnl.0000000000000276. PubMed PMID: 24574547.

488. Yeung ST, Myczek K, Kang AP, Chabrier MA, Baglietto-Vargas D, Laferla FM. Impact of hippocampal neuronal ablation on neurogenesis and cognition in the aged brain. Neuroscience. 2014;259:214-22. Epub 2013/12/10. doi: 10.1016/j.neuroscience.2013.11.054. PubMed PMID: 24316470; PubMed Central PMCID: PMCPMC4438704.

489. Young SJ, Bertucco M, Sanger TD. Cathodal transcranial direct current stimulation in children with dystonia: a sham-controlled study. J Child Neurol. 2014;29(2):232-9. Epub 2013/06/14. doi: 10.1177/0883073813492385. PubMed PMID: 23760989.

490. Zhang X, Bian JS. Hydrogen sulfide: a neuromodulator and neuroprotectant in the central nervous system. ACS Chem Neurosci. 2014;5(10):876-83. Epub 2014/09/18. doi: 10.1021/cn500185g. PubMed PMID: 25230373.

491. Zhou HJ, Tang T, Zhong JH, Luo JK, Cui HJ, Zhang QM, et al. Electroacupuncture improves recovery after hemorrhagic brain injury by inducing the expression of angiopoietin-1 and -2 in rats. BMC Complement Altern Med. 2014;14:127. Epub 2014/04/09.

doi: 10.1186/1472-6882-14-127. PubMed PMID: 24708627; PubMed Central PMCID: PMCPMC4012070.

492. Büyükçoban S, Arıcı MA, Koca U, Kalkan Ş. A Case Report of Toxic Brain Syndrome Caused by Methyl Bromide. Turk J Anaesthesiol Reanim. 2015;43(2):134-7. Epub 2015/04/01. doi: 10.5152/tjar.2014.84756. PubMed PMID: 27366483; PubMed Central PMCID: PMCPMC4917157.

493. Calzolari E, Bolognini N, Casati C, Marzoli SB, Vallar G. Restoring abnormal aftereffects of prismatic adaptation through neuromodulation. Neuropsychologia. 2015;74:162-9. Epub 2015/04/29. doi: 10.1016/j.neuropsychologia.2015.04.022. PubMed PMID: 25912762.

494. Chaieb L, Antal A, Masurat F, Paulus W. Neuroplastic effects of transcranial near-infrared stimulation (tNIRS) on the motor cortex. Front Behav Neurosci. 2015;9:147. Epub 2015/06/18. doi: 10.3389/fnbeh.2015.00147. PubMed PMID: 26082699; PubMed Central PMCID: PMCPMC4451368.

495. Chung MG, Lo WD. Noninvasive brain stimulation: the potential for use in the rehabilitation of pediatric acquired brain injury. Arch Phys Med Rehabil. 2015;96(4 Suppl):S129-37. Epub 2014/12/03. doi: 10.1016/j.apmr.2014.10.013. PubMed PMID: 25448248.

496. Cincotta M, Giovannelli F, Chiaramonti R, Bianco G, Godone M, Battista D, et al. No effects of 20 Hz-rTMS of the primary motor cortex in vegetative state: A randomised, sham-controlled study. Cortex. 2015;71:368-76. Epub 2015/08/25. doi: 10.1016/j.cortex.2015.07.027. PubMed PMID: 26301875.

497. Danilov Y, Kaczmarek K, Skinner K, Tyler M. Frontiers in Neuroengineering
Cranial Nerve Noninvasive Neuromodulation: New Approach to Neurorehabilitation. In: Kobeissy FH, editor. Brain Neurotrauma: Molecular, Neuropsychological, and Rehabilitation Aspects. Boca Raton (FL): CRC Press/Taylor & Francis
© 2015 by Taylor & Francis Group, LLC.; 2015.

498. Dhaliwal SK, Meek BP, Modirrousta MM. Non-Invasive Brain Stimulation for the Treatment of Symptoms Following Traumatic Brain Injury. Front Psychiatry. 2015;6:119. Epub 2015/09/18. doi: 10.3389/fpsyt.2015.00119. PubMed PMID: 26379560; PubMed Central PMCID: PMCPMC4549551.

499. Espahbodi M, Sweeney AD, Lennon KJ, Wanna GB. Facial nerve stimulation associated with cochlear implant use following temporal bone fractures. Am J Otolaryngol. 2015;36(4):578-82. Epub 2015/05/02. doi: 10.1016/j.amjoto.2015.04.003. PubMed PMID: 25929977.

500. Fecteau S, Dickler M, Pelayo R, Kumru H, Bernabeu M, Opisso Salleras E, et al. Cortical Excitability During Passive Action Observation in Hospitalized Adults With Subacute Moderate to Severe Traumatic Brain Injury: A Preliminary TMS Study. Neurorehabil Neural Repair. 2015;29(6):548-56. Epub 2014/12/17. doi: 10.1177/1545968314558603. PubMed PMID: 25505219; PubMed Central PMCID: PMCPMC4465410.

501. Fins JJ. Nanotechnology, neuromodulation & the immune response: discourse, materiality & ethics. Biomed Microdevices. 2015;17(2):28. Epub 2015/02/15. doi: 10.1007/s10544-015-9934-0. PubMed PMID: 25681046.

502. Gertler P, Tate RL, Cameron ID. Non-pharmacological interventions for depression in adults and children with traumatic brain injury. Cochrane Database Syst Rev. 2015;2015(12):Cd009871. Epub 2015/12/15. doi: 10.1002/14651858.CD009871.pub2. PubMed PMID: 26663136; PubMed Central PMCID: PMCPMC8761477.

503. Gilbert KS, Kark SM, Gehrman P, Bogdanova Y. Sleep disturbances, TBI and PTSD: Implications for treatment and recovery. Clin Psychol Rev. 2015;40:195-212. Epub 2015/07/15. doi: 10.1016/j.cpr.2015.05.008. PubMed PMID: 26164549; PubMed Central PMCID: PMCPMC5153364.

504. Gillick BT, Rich T, Chen M, Meekins GD. Case report of vasovagal syncope associated with single pulse transcranial magnetic stimulation in a healthy adult participant. BMC Neurol. 2015;15:248. Epub 2015/12/03. doi: 10.1186/s12883-015-0510-2. PubMed PMID: 26627895; PubMed Central PMCID: PMCPMC4666029.

505. Goldwasser T, Bressan S, Oakley E, Arpone M, Babl FE. Use of sedation in children receiving computed tomography after head injuries. Eur J Emerg Med. 2015;22(6):413-8. Epub 2014/09/16. doi: 10.1097/mej.0000000000000201. PubMed PMID: 25222427.

506. Gosseries O, Sarasso S, Casarotto S, Boly M, Schnakers C, Napolitani M, et al. On the cerebral origin of EEG responses to TMS: insights from severe cortical lesions. Brain Stimul. 2015;8(1):142-9. Epub 2014/12/08. doi: 10.1016/j.brs.2014.10.008. PubMed PMID: 25481074.

507. Gummadavelli A, Kundishora AJ, Willie JT, Andrews JP, Gerrard JL, Spencer DD, et al. Neurostimulation to improve level of consciousness in patients with epilepsy. Neurosurg Focus. 2015;38(6):E10. Epub 2015/06/02. doi: 10.3171/2015.3.Focus1535. PubMed PMID: 26030698; PubMed Central PMCID: PMCPMC6300138.

508. Guo T, Li H, Lv Y, Lu H, Niu J, Sun J, et al. Pulsed

Transcranial Ultrasound Stimulation Immediately After The Ischemic Brain Injury is Neuroprotective. IEEE Trans Biomed Eng. 2015;62(10):2352-7. Epub 2015/05/03. doi: 10.1109/tbme.2015.2427339. PubMed PMID: 25935023.

509. Isagulyan ED, Tomsky AA, Dekopov AV, Salova EM, Troshina EM, Dorokhov EV, et al. Results of motor cortex stimulation in the treatment of chronic pain syndromes. Zh Vopr Neirokhir Im N N Burdenko. 2015;79(6):46-60. Epub 2015/01/01. doi: 10.17116/neiro201579646-60. PubMed PMID: 26977794.

510. Koski L, Kolivakis T, Yu C, Chen JK, Delaney S, Ptito A. Noninvasive brain stimulation for persistent postconcussion symptoms in mild traumatic brain injury. J Neurotrauma. 2015;32(1):38-44. Epub 2014/06/24. doi: 10.1089/neu.2014.3449. PubMed PMID: 24955920.

511. Lee DJ, Gurkoff GG, Izadi A, Seidl SE, Echeverri A, Melnik M, et al. Septohippocampal Neuromodulation Improves Cognition after Traumatic Brain Injury. J Neurotrauma. 2015;32(22):1822-32. Epub 2015/06/23. doi: 10.1089/neu.2014.3744. PubMed PMID: 26096267; PubMed Central PMCID: PMCPMC4702430.

512. Lefebvre G, Tremblay S, Théoret H. Probing the effects of mild traumatic brain injury with transcranial magnetic stimulation of the primary motor cortex. Brain Inj. 2015;29(9):1032-43. Epub

2015/05/23. doi: 10.3109/02699052.2015.1028447. PubMed PMID: 25996466.

513. Lei J, Wang L, Gao G, Cooper E, Jiang J. Right Median Nerve Electrical Stimulation for Acute Traumatic Coma Patients. J Neurotrauma. 2015;32(20):1584-9. Epub 2015/02/11. doi: 10.1089/neu.2014.3768. PubMed PMID: 25664378.

514. Lemmens S, Brône B, Dooley D, Hendrix S, Geurts N. Alpha-adrenoceptor modulation in central nervous system trauma: pain, spasms, and paralysis--an unlucky triad. Med Res Rev. 2015;35(4):653-77. Epub 2014/12/30. doi: 10.1002/med.21337. PubMed PMID: 25546087.

515. Li S, Zaninotto AL, Neville IS, Paiva WS, Nunn D, Fregni F. Clinical utility of brain stimulation modalities following traumatic brain injury: current evidence. Neuropsychiatr Dis Treat. 2015;11:1573-86. Epub 2015/07/15. doi: 10.2147/ndt.S65816. PubMed PMID: 26170670; PubMed Central PMCID: PMCPMC4494620.

516. Lu H, Kobilo T, Robertson C, Tong S, Celnik P, Pelled G. Transcranial magnetic stimulation facilitates neurorehabilitation after pediatric traumatic brain injury. Sci Rep. 2015;5:14769. Epub 2015/10/07. doi: 10.1038/srep14769. PubMed PMID: 26440604; PubMed Central PMCID: PMCPMC4594036.

517. Major BP, Rogers MA, Pearce AJ. Using transcranial magnetic stimulation to quantify electrophysiological changes following concussive brain injury: a systematic review. Clin Exp Pharmacol Physiol. 2015;42(4):394-405. Epub 2015/01/22. doi: 10.1111/1440-1681.12363. PubMed PMID: 25603731.
518. Miller JP, Sweet JA, Bailey CM, Munyon CN, Luders HO, Fastenau PS. Visual-spatial memory may be enhanced with theta burst deep brain stimulation of the fornix: a preliminary investigation with four cases. Brain. 2015;138(Pt 7):1833-42. Epub 2015/06/25. doi: 10.1093/brain/awv095. PubMed PMID: 26106097.
519. Nardone A, Sampson E, Stauffer C, Leopold A, Jacobs K, Hendricks DJ, et al. Project Career: A qualitative examination of five college students with traumatic brain injuries. NeuroRehabilitation. 2015;37(3):459-69. Epub 2015/11/01. doi: 10.3233/nre-151274. PubMed PMID: 26518535.
520. Naro A, Calabrò RS, Russo M, Leo A, Pollicino P, Quartarone A, et al. Can transcranial direct current stimulation be useful in differentiating unresponsive wakefulness syndrome from minimally conscious state patients? Restor Neurol Neurosci. 2015;33(2):159-76. Epub 2015/01/16. doi: 10.3233/rnn-140448. PubMed PMID: 25588461.

521. Naro A, Leo A, Bramanti P, Calabrò RS. Moving Toward Conscious Pain Processing Detection in Chronic Disorders of Consciousness: Anterior Cingulate Cortex Neuromodulation. J Pain. 2015;16(10):1022-31. Epub 2015/07/26. doi: 10.1016/j.jpain.2015.06.014. PubMed PMID: 26208761.
522. Naro A, Leo A, Cannavò A, Buda A, Bruno R, Salviera C, et al. Audiomotor Integration in Minimally Conscious State: Proof of Concept! Neural Plast. 2015;2015:391349. Epub 2015/10/02. doi: 10.1155/2015/391349. PubMed PMID: 26425370; PubMed Central PMCID: PMCPMC4573993.
523. Naro A, Leo A, Filoni S, Bramanti P, Calabrò RS. Visuo-motor integration in unresponsive wakefulness syndrome: A piece of the puzzle towards consciousness detection? Restor Neurol Neurosci. 2015;33(4):447-60. Epub 2015/09/28. doi: 10.3233/rnn-150525. PubMed PMID: 26409404; PubMed Central PMCID: PMCPMC4923741.
524. Naro A, Leo A, Russo M, Quartarone A, Bramanti P, Calabrò RS. Shaping thalamo-cortical plasticity: a marker of cortical pain integration in patients with post-anoxic unresponsive wakefulness syndrome? Brain Stimul. 2015;8(1):97-104. Epub 2014/09/28. doi: 10.1016/j.brs.2014.09.001. PubMed PMID: 25260422.
525. Naro A, Pisani LR, Leo A, Molonia F, Bramanti P, Calabrò RS.

Treatment of refractory generalized status epilepticus in a patient with unresponsive wakefulness syndrome: Is neuromodulation the future? Epilepsy Behav. 2015;50:96-7. Epub 2015/07/27. doi: 10.1016/j.yebeh.2015.06.038. PubMed PMID: 26209943.

526. Naro A, Russo M, Leo A, Bramanti P, Quartarone A, Calabrò RS. A Single Session of Repetitive Transcranial Magnetic Stimulation Over the Dorsolateral Prefrontal Cortex in Patients With Unresponsive Wakefulness Syndrome: Preliminary Results. Neurorehabil Neural Repair. 2015;29(7):603-13. Epub 2014/12/30. doi: 10.1177/1545968314562114. PubMed PMID: 25539781.

527. Neville IS, Hayashi CY, El Hajj SA, Zaninotto AL, Sabino JP, Sousa LM, Jr., et al. Repetitive Transcranial Magnetic Stimulation (rTMS) for the cognitive rehabilitation of traumatic brain injury (TBI) victims: study protocol for a randomized controlled trial. Trials. 2015;16:440. Epub 2015/10/07. doi: 10.1186/s13063-015-0944-2. PubMed PMID: 26438108; PubMed Central PMCID: PMCPMC4594992.

528. Nielson DM, McKnight CA, Patel RN, Kalnin AJ, Mysiw WJ. Preliminary guidelines for safe and effective use of repetitive transcranial magnetic stimulation in moderate to severe traumatic brain injury. Arch Phys Med Rehabil. 2015;96(4 Suppl):S138-44. Epub 2014/10/05. doi: 10.1016/j.apmr.2014.09.010. PubMed

PMID: 25281871.

529. Page SJ, Cunningham DA, Plow E, Blazak B. It takes two: noninvasive brain stimulation combined with neurorehabilitation. Arch Phys Med Rehabil. 2015;96(4 Suppl):S89-93. Epub 2015/03/31. doi: 10.1016/j.apmr.2014.09.019. PubMed PMID: 25813373; PubMed Central PMCID: PMCPMC4445084.

530. Pearce AJ, Hoy K, Rogers MA, Corp DT, Davies CB, Maller JJ, et al. Acute motor, neurocognitive and neurophysiological change following concussion injury in Australian amateur football. A prospective multimodal investigation. J Sci Med Sport. 2015;18(5):500-6. Epub 2014/08/12. doi: 10.1016/j.jsams.2014.07.010. PubMed PMID: 25104044.

531. Pisani LR, Naro A, Leo A, Aricò I, Pisani F, Silvestri R, et al. Repetitive transcranial magnetic stimulation induced slow wave activity modification: A possible role in disorder of consciousness differential diagnosis? Conscious Cogn. 2015;38:1-8. Epub 2015/10/27. doi: 10.1016/j.concog.2015.09.012. PubMed PMID: 26496476.

532. Potter-Baker KA, Bonnett CE, Chabra P, Roelle S, Varnerin N, Cunningham DA, et al. A game of hide and seek: Is it possible to recruit more patients for NIBS studies in stroke? J Neurol Sci. 2015;358(1-2):472-4. Epub 2015/09/01. doi:

10.1016/j.jns.2015.08.030. PubMed PMID: 26320611; PubMed Central PMCID: PMCPMC4629798.

533. Rao VR, Parko KL. Clinical approach to posttraumatic epilepsy. Semin Neurol. 2015;35(1):57-63. Epub 2015/02/26. doi: 10.1055/s-0035-1544239. PubMed PMID: 25714868.

534. Reti IM, Schwarz N, Bower A, Tibbs M, Rao V. Transcranial magnetic stimulation: A potential new treatment for depression associated with traumatic brain injury. Brain Inj. 2015;29(7-8):789-97. Epub 2015/05/08. doi: 10.3109/02699052.2015.1009168. PubMed PMID: 25950260.

535. Rodger J, Sherrard RM. Optimising repetitive transcranial magnetic stimulation for neural circuit repair following traumatic brain injury. Neural Regen Res. 2015;10(3):357-9. Epub 2015/04/17. doi: 10.4103/1673-5374.153676. PubMed PMID: 25878575; PubMed Central PMCID: PMCPMC4396089.

536. Seo JP, Jang SH. Traumatic axonal injury of the corticospinal tract in the subcortical white matter in patients with mild traumatic brain injury. Brain Inj. 2015;29(1):110-4. Epub 2014/10/31. doi: 10.3109/02699052.2014.973447. PubMed PMID: 25356741.

537. Sergeeva EG, Bola M, Wagner S, Lazik S, Voigt N, Mawrin C, et al. Repetitive Transcorneal Alternating Current Stimulation Reduces Brain Idling State After Long-term Vision Loss. Brain

Stimul. 2015;8(6):1065-73. Epub 2015/07/07. doi: 10.1016/j.brs.2015.06.006. PubMed PMID: 26145756.
538. Sokal P, Harat M, Zieliński P, Furtak J, Paczkowski D, Rusinek M. Motor cortex stimulation in patients with chronic central pain. Adv Clin Exp Med. 2015;24(2):289-96. Epub 2015/05/02. doi: 10.17219/acem/40452. PubMed PMID: 25931362.
539. Speth J, Speth C, Harley TA. Transcranial direct current stimulation of the motor cortex in waking resting state induces motor imagery. Conscious Cogn. 2015;36:298-305. Epub 2015/07/24. doi: 10.1016/j.concog.2015.07.005. PubMed PMID: 26204566.
540. Takamiya A, Kishimoto T, Watanabe K, Mimura M, Kito S. Transcranial Magnetic Stimulation for Bipolar Disorder with Catatonic Stupor: A Case Report. Brain Stimul. 2015;8(6):1236-7. Epub 2015/10/03. doi: 10.1016/j.brs.2015.09.004. PubMed PMID: 26428351.
541. Tang AD, Makowiecki K, Bartlett C, Rodger J. Low intensity repetitive transcranial magnetic stimulation does not induce cell survival or regeneration in a mouse optic nerve crush model. PLoS One. 2015;10(5):e0126949. Epub 2015/05/21. doi: 10.1371/journal.pone.0126949. PubMed PMID: 25993112;

PubMed Central PMCID: PMCPMC4438867.

542. Thibaut A, Di Perri C, Chatelle C, Bruno MA, Bahri MA, Wannez S, et al. Clinical Response to tDCS Depends on Residual Brain Metabolism and Grey Matter Integrity in Patients With Minimally Conscious State. Brain Stimul. 2015;8(6):1116-23. Epub 2015/10/17. doi: 10.1016/j.brs.2015.07.024. PubMed PMID: 26471400.

543. Tremblay S, Vernet M, Bashir S, Pascual-Leone A, Théoret H. Theta burst stimulation to characterize changes in brain plasticity following mild traumatic brain injury: A proof-of-principle study. Restor Neurol Neurosci. 2015;33(5):611-20. Epub 2015/03/05. doi: 10.3233/rnn-140459. PubMed PMID: 25735241; PubMed Central PMCID: PMCPMC4850740.

544. Ulam F, Shelton C, Richards L, Davis L, Hunter B, Fregni F, et al. Cumulative effects of transcranial direct current stimulation on EEG oscillations and attention/working memory during subacute neurorehabilitation of traumatic brain injury. Clin Neurophysiol. 2015;126(3):486-96. Epub 2014/06/21. doi: 10.1016/j.clinph.2014.05.015. PubMed PMID: 24947595.

545. Yoon KJ, Lee YT, Chung PW, Lee YK, Kim DY, Chun MH. Effects of Repetitive Transcranial Magnetic Stimulation on Behavioral Recovery during Early Stage of Traumatic Brain Injury

in Rats. J Korean Med Sci. 2015;30(10):1496-502. Epub 2015/10/02. doi: 10.3346/jkms.2015.30.10.1496. PubMed PMID: 26425049; PubMed Central PMCID: PMCPMC4575941.

546. Yoon YS, Cho KH, Kim ES, Lee MS, Lee KJ. Effect of Epidural Electrical Stimulation and Repetitive Transcranial Magnetic Stimulation in Rats With Diffuse Traumatic Brain Injury. Ann Rehabil Med. 2015;39(3):416-24. Epub 2015/07/15. doi: 10.5535/arm.2015.39.3.416. PubMed PMID: 26161348; PubMed Central PMCID: PMCPMC4496513.

547. Yuan J, Wu Y, Li JY, Zhang L, Chen X, Chen HX. [Protection of Transcutaneous Acupoint Electrical Stimulation for Brain Injury Undergoing Intervention: a Clinical Observation]. Zhongguo Zhong Xi Yi Jie He Za Zhi. 2015;35(8):971-4. Epub 2015/10/22. PubMed PMID: 26485913.

548. Zanakis S, Tasoulas J, Angelidis I, Dendrinos C. Tooth in the line of angle fractures: the impact in the healing process. A retrospective study of 112 patients. J Craniomaxillofac Surg. 2015;43(1):113-6. Epub 2014/12/04. doi: 10.1016/j.jcms.2014.10.020. PubMed PMID: 25465485.

549. Zhao W, Wang C, Li Z, Chen L, Li J, Cui W, et al. Efficacy and safety of transcutaneous electrical acupoint stimulation to treat muscle spasticity following brain injury: a double-blinded,

multicenter, randomized controlled trial. PLoS One. 2015;10(2):e0116976. Epub 2015/02/03. doi: 10.1371/journal.pone.0116976. PubMed PMID: 25643051; PubMed Central PMCID: PMCPMC4314074.

550. Zhong YJ, Feng Z, Wang L, Wei TQ. Wake-promoting actions of median nerve stimulation in TBI-induced coma: An investigation of orexin-A and orexin receptor 1 in the hypothalamic region. Mol Med Rep. 2015;12(3):4441-7. Epub 2015/06/11. doi: 10.3892/mmr.2015.3898. PubMed PMID: 26059340.

551. Bai Y, Xia X, Kang J, Yin X, Yang Y, He J, et al. Evaluating the Effect of Repetitive Transcranial Magnetic Stimulation on Disorders of Consciousness by Using TMS-EEG. Front Neurosci. 2016;10:473. Epub 2016/11/05. doi: 10.3389/fnins.2016.00473. PubMed PMID: 27812319; PubMed Central PMCID: PMCPMC5071327.

552. Bick SK, Eskandar EN. Neuromodulation for restoring memory. Neurosurg Focus. 2016;40(5):E5. Epub 2016/05/03. doi: 10.3171/2016.3.Focus162. PubMed PMID: 27132526.

553. Brill J, Shao Z, Puche AC, Wachowiak M, Shipley MT. Serotonin increases synaptic activity in olfactory bulb glomeruli. J Neurophysiol. 2016;115(3):1208-19. Epub 2015/12/15. doi: 10.1152/jn.00847.2015. PubMed PMID: 26655822; PubMed

Central PMCID: PMCPMC4808087.
554. Casarotto S, Comanducci A, Rosanova M, Sarasso S, Fecchio M, Napolitani M, et al. Stratification of unresponsive patients by an independently validated index of brain complexity. Ann Neurol. 2016;80(5):718-29. Epub 2016/11/03. doi: 10.1002/ana.24779. PubMed PMID: 27717082; PubMed Central PMCID: PMCPMC5132045.
555. Cavaliere C, Aiello M, Di Perri C, Amico E, Martial C, Thibaut A, et al. Functional Connectivity Substrates for tDCS Response in Minimally Conscious State Patients. Front Cell Neurosci. 2016;10:257. Epub 2016/11/20. doi: 10.3389/fncel.2016.00257. PubMed PMID: 27857682; PubMed Central PMCID: PMCPMC5093112.
556. Chen HI, Burke JF, Cohen AS. Editorial: Traumatic Brain Injury As a Systems Neuroscience Problem. Front Syst Neurosci. 2016;10:100. Epub 2016/12/27. doi: 10.3389/fnsys.2016.00100. PubMed PMID: 28018187; PubMed Central PMCID: PMCPMC5145880.
557. Clayton E, Kinley-Cooper SK, Weber RA, Adkins DL. Brain stimulation: Neuromodulation as a potential treatment for motor recovery following traumatic brain injury. Brain Res. 2016;1640(Pt A):130-8. Epub 2016/02/09. doi: 10.1016/j.brainres.2016.01.056.

PubMed PMID: 26855256; PubMed Central PMCID: PMCPMC5090977.

558. Cruccu G, Garcia-Larrea L, Hansson P, Keindl M, Lefaucheur JP, Paulus W, et al. EAN guidelines on central neurostimulation therapy in chronic pain conditions. Eur J Neurol. 2016;23(10):1489-99. Epub 2016/08/12. doi: 10.1111/ene.13103. PubMed PMID: 27511815.

559. Davidson TW, Tremblay F. Evidence of alterations in transcallosal motor inhibition as a possible long-term consequence of concussions in sports: A transcranial magnetic stimulation study. Clin Neurophysiol. 2016;127(10):3364-75. Epub 2016/08/18. doi: 10.1016/j.clinph.2016.07.012. PubMed PMID: 27531393.

560. Debruyne P. "Cardio-Neuromodulation" With a Multielectrode Irrigated Catheter: A Potential New Approach for Patients With Cardio-Inhibitory Syncope. J Cardiovasc Electrophysiol. 2016;27(9):1110-3. Epub 2016/06/17. doi: 10.1111/jce.13031. PubMed PMID: 27307200.

561. Di Virgilio TG, Hunter A, Wilson L, Stewart W, Goodall S, Howatson G, et al. Evidence for Acute Electrophysiological and Cognitive Changes Following Routine Soccer Heading. EBioMedicine. 2016;13:66-71. Epub 2016/10/30. doi: 10.1016/j.ebiom.2016.10.029. PubMed PMID: 27789273; PubMed

Central PMCID: PMCPMC5264439.

562. DosSantos MF, Ferreira N, Toback RL, Carvalho AC, DaSilva AF. Potential Mechanisms Supporting the Value of Motor Cortex Stimulation to Treat Chronic Pain Syndromes. Front Neurosci. 2016;10:18. Epub 2016/02/24. doi: 10.3389/fnins.2016.00018. PubMed PMID: 26903788; PubMed Central PMCID: PMCPMC4749700.

563. Feng Z, Du Q. Mechanisms responsible for the effect of median nerve electrical stimulation on traumatic brain injury-induced coma: orexin-A-mediated N-methyl-D-aspartate receptor subunit NR1 upregulation. Neural Regen Res. 2016;11(6):951-6. Epub 2016/08/03. doi: 10.4103/1673-5374.184494. PubMed PMID: 27482224; PubMed Central PMCID: PMCPMC4962593.

564. Formaggio E, Cavinato M, Storti SF, Tonin P, Piccione F, Manganotti P. Assessment of Event-Related EEG Power After Single-Pulse TMS in Unresponsive Wakefulness Syndrome and Minimally Conscious State Patients. Brain Topogr. 2016;29(2):322-33. Epub 2015/11/23. doi: 10.1007/s10548-015-0461-3. PubMed PMID: 26590568.

565. Gall C, Schmidt S, Schittkowski MP, Antal A, Ambrus GG, Paulus W, et al. Alternating Current Stimulation for Vision

Restoration after Optic Nerve Damage: A Randomized Clinical Trial. PLoS One. 2016;11(6):e0156134. Epub 2016/06/30. doi: 10.1371/journal.pone.0156134. PubMed PMID: 27355577; PubMed Central PMCID: PMCPMC4927182.

566. Girgis F, Pace J, Sweet J, Miller JP. Hippocampal Neurophysiologic Changes after Mild Traumatic Brain Injury and Potential Neuromodulation Treatment Approaches. Front Syst Neurosci. 2016;10:8. Epub 2016/02/24. doi: 10.3389/fnsys.2016.00008. PubMed PMID: 26903824; PubMed Central PMCID: PMCPMC4746250.

567. Gosseries O, Pistoia F, Charland-Verville V, Carolei A, Sacco S, Laureys S. The Role of Neuroimaging Techniques in Establishing Diagnosis, Prognosis and Therapy in Disorders of Consciousness. Open Neuroimag J. 2016;10:52-68. Epub 2016/06/28. doi: 10.2174/1874440001610010052. PubMed PMID: 27347265; PubMed Central PMCID: PMCPMC4894918.

568. Honey CM, Tronnier VM, Honey CR. Deep brain stimulation versus motor cortex stimulation for neuropathic pain: A minireview of the literature and proposal for future research. Comput Struct Biotechnol J. 2016;14:234-7. Epub 2016/07/15. doi: 10.1016/j.csbj.2016.06.003. PubMed PMID: 27413477; PubMed Central PMCID: PMCPMC4925438.

569. Kesar TM, McDonald HS, Eicholtz SP, Borich MR. Case Report of Syncope During a Single Pulse Transcranial Magnetic Stimulation Experiment in a Healthy Adult Participant. Brain Stimul. 2016;9(3):471-2. Epub 2016/04/07. doi: 10.1016/j.brs.2016.03.013. PubMed PMID: 27050115; PubMed Central PMCID: PMCPMC4928708.
570. Kim JW, Bae KY, Kim SW, Kang HJ, Shin IS, Yoon JS, et al. Treatment-Resistant Depression Entering Remission Following a Seizure during the Course of Repetitive Transcranial Magnetic Stimulation. Psychiatry Investig. 2016;13(4):468-71. Epub 2016/08/03. doi: 10.4306/pi.2016.13.4.468. PubMed PMID: 27482250; PubMed Central PMCID: PMCPMC4965659.
571. Kondratyeva EA, Avdunina IA, Kondratyev AN, Ulitin AU, Ivanova NE, Petrova MV, et al. [Vegetative State: Difficulty in Identifying Consciousness and Predicting Outcome]. Vestn Ross Akad Med Nauk. 2016;71(4):273-80. Epub 2016/01/01. doi: 10.15690/vramn728. PubMed PMID: 29297644.
572. Långsjö JW, Lehtimäki K, Ruohonen J, Sajanti A, Sandell S, Heikkilä HT, et al. Critical neural targets for (the level of) human consciousness: Arousal arrest and unconsciousness after sumatriptan administration. Brain Inj. 2016;30(13-14):1731-6. Epub 2016/09/30. doi: 10.1080/02699052.2016.1199908. PubMed

PMID: 27680211.
573. Leung A, Fallah A, Shukla S, Lin L, Tsia A, Song D, et al. rTMS in Alleviating Mild TBI Related Headaches--A Case Series. Pain Physician. 2016;19(2):E347-54. Epub 2016/01/28. PubMed PMID: 26815263.
574. Leung A, Shukla S, Fallah A, Song D, Lin L, Golshan S, et al. Repetitive Transcranial Magnetic Stimulation in Managing Mild Traumatic Brain Injury-Related Headaches. Neuromodulation. 2016;19(2):133-41. Epub 2015/11/12. doi: 10.1111/ner.12364. PubMed PMID: 26555886.
575. Liu P, Gao J, Pan S, Meng F, Pan G, Li J, et al. Effects of High-Frequency Repetitive Transcranial Magnetic Stimulation on Cerebral Hemodynamics in Patients with Disorders of Consciousness: A Sham-Controlled Study. Eur Neurol. 2016;76(1-2):1-7. Epub 2016/06/23. doi: 10.1159/000447325. PubMed PMID: 27332827.
576. Marangolo P, Fiori V, Sabatini U, De Pasquale G, Razzano C, Caltagirone C, et al. Bilateral Transcranial Direct Current Stimulation Language Treatment Enhances Functional Connectivity in the Left Hemisphere: Preliminary Data from Aphasia. J Cogn Neurosci. 2016;28(5):724-38. Epub 2016/01/26. doi: 10.1162/jocn_a_00927. PubMed PMID: 26807842.

577. Martino Cinnera A, Bonnì S, Iosa M, Ponzo V, Fusco A, Caltagirone C, et al. Clinical effects of non-invasive cerebellar magnetic stimulation treatment combined with neuromotor rehabilitation in traumatic brain injury. A single case study. Funct Neurol. 2016;31(2):117-20. Epub 2016/07/01. PubMed PMID: 27358225.

578. Matsumoto-Miyazaki J, Asano Y, Yonezawa S, Nomura Y, Ikegame Y, Aki T, et al. Acupuncture Increases the Excitability of the Cortico-Spinal System in Patients with Chronic Disorders of Consciousness Following Traumatic Brain Injury. J Altern Complement Med. 2016;22(11):887-94. Epub 2016/09/24. doi: 10.1089/acm.2014.0356. PubMed PMID: 27662495.

579. Matteo BM, Viganò B, Cerri CG, Perin C. Visual field restorative rehabilitation after brain injury. J Vis. 2016;16(9):11. Epub 2016/07/30. doi: 10.1167/16.9.11. PubMed PMID: 27472498.

580. Meng L, Jiang J, Jin C, Liu J, Zhao Y, Wang W, et al. Trauma-specific Grey Matter Alterations in PTSD. Sci Rep. 2016;6:33748. Epub 2016/09/22. doi: 10.1038/srep33748. PubMed PMID: 27651030; PubMed Central PMCID: PMCPMC5030628.

581. Momosaki R, Kinoshita S, Kakuda W, Yamada N, Abo M. Noninvasive brain stimulation for dysphagia after acquired brain

injury: a systematic review. J Med Invest. 2016;63(3-4):153-8. Epub 2016/09/21. doi: 10.2152/jmi.63.153. PubMed PMID: 27644550.

582. Morishita T, Inoue T. Brain Stimulation Therapy for Central Post-Stroke Pain from a Perspective of Interhemispheric Neural Network Remodeling. Front Hum Neurosci. 2016;10:166. Epub 2016/05/06. doi: 10.3389/fnhum.2016.00166. PubMed PMID: 27148019; PubMed Central PMCID: PMCPMC4838620.

583. Naro A, Bramanti P, Leo A, Russo M, Calabrò RS. Transcranial Alternating Current Stimulation in Patients with Chronic Disorder of Consciousness: A Possible Way to Cut the Diagnostic Gordian Knot? Brain Topogr. 2016;29(4):623-44. Epub 2016/04/12. doi: 10.1007/s10548-016-0489-z. PubMed PMID: 27062669.

584. Naro A, Leo A, Buda A, Manuli A, Bramanti A, Bramanti P, et al. Do you see me? The role of visual fixation in chronic disorders of consciousness differential diagnosis. Brain Res. 2016;1653:59-66. Epub 2016/10/25. doi: 10.1016/j.brainres.2016.10.015. PubMed PMID: 27773729.

585. Naro A, Leo A, Buda A, Manuli A, Bramanti A, Bramanti P, et al. Unravelling motor networks in patients with chronic disorders of consciousness: A promising minimally invasive approach. Brain

Res. 2016;1646:262-8. Epub 2016/06/12. doi: 10.1016/j.brainres.2016.06.012. PubMed PMID: 27288702.

586. Naro A, Russo M, Leo A, Cannavò A, Manuli A, Bramanti A, et al. Cortical connectivity modulation induced by cerebellar oscillatory transcranial direct current stimulation in patients with chronic disorders of consciousness: A marker of covert cognition? Clin Neurophysiol. 2016;127(3):1845-54. Epub 2016/01/13. doi: 10.1016/j.clinph.2015.12.010. PubMed PMID: 26754875.

587. Neren D, Johnson MD, Legon W, Bachour SP, Ling G, Divani AA. Vagus Nerve Stimulation and Other Neuromodulation Methods for Treatment of Traumatic Brain Injury. Neurocrit Care. 2016;24(2):308-19. Epub 2015/09/25. doi: 10.1007/s12028-015-0203-0. PubMed PMID: 26399249.

588. Nieminen JO, Gosseries O, Massimini M, Saad E, Sheldon AD, Boly M, et al. Consciousness and cortical responsiveness: a within-state study during non-rapid eye movement sleep. Sci Rep. 2016;6:30932. Epub 2016/08/06. doi: 10.1038/srep30932. PubMed PMID: 27491799; PubMed Central PMCID: PMCPMC4974655.

589. Padilla R, Domina A. Effectiveness of Sensory Stimulation to Improve Arousal and Alertness of People in a Coma or Persistent Vegetative State After Traumatic Brain Injury: A Systematic Review. Am J Occup Ther. 2016;70(3):7003180030p1-8. Epub

2016/04/19. doi: 10.5014/ajot.2016.021022. PubMed PMID: 27089287.

590. Pearce AJ. The Neurophysiological Response Following Sub-Concussive Soccer Heading. EBioMedicine. 2016;13:3-4. Epub 2016/11/08. doi: 10.1016/j.ebiom.2016.10.043. PubMed PMID: 27818040; PubMed Central PMCID: PMCPMC5264430.

591. Pevzner A, Izadi A, Lee DJ, Shahlaie K, Gurkoff GG. Making Waves in the Brain: What Are Oscillations, and Why Modulating Them Makes Sense for Brain Injury. Front Syst Neurosci. 2016;10:30. Epub 2016/04/20. doi: 10.3389/fnsys.2016.00030. PubMed PMID: 27092062; PubMed Central PMCID: PMCPMC4823270.

592. Pruitt DT, Schmid AN, Kim LJ, Abe CM, Trieu JL, Choua C, et al. Vagus Nerve Stimulation Delivered with Motor Training Enhances Recovery of Function after Traumatic Brain Injury. J Neurotrauma. 2016;33(9):871-9. Epub 2015/06/11. doi: 10.1089/neu.2015.3972. PubMed PMID: 26058501; PubMed Central PMCID: PMCPMC4860663.

593. Pycroft L, Boccard SG, Owen SLF, Stein JF, Fitzgerald JJ, Green AL, et al. Brainjacking: Implant Security Issues in Invasive Neuromodulation. World Neurosurg. 2016;92:454-62. Epub 2016/05/18. doi: 10.1016/j.wneu.2016.05.010. PubMed PMID:

27184896.
594. Rahimpour S, Lad SP. Frontiers in Neuroscience
Traumatic Brain Injury and Potential for Neuromodulation. In: Laskowitz D, Grant G, editors. Translational Research in Traumatic Brain Injury. Boca Raton (FL): CRC Press/Taylor and Francis Group
© 2016 by Taylor & Francis Group, LLC.; 2016.
595. Rajan TS, Cuzzocrea S, Bruschetta D, Quartarone A. Repetitive Transcranial Magnetic Stimulation as a Novel Therapy in Animal Models of Traumatic Brain Injury. Methods Mol Biol. 2016;1462:433-43. Epub 2016/09/09. doi: 10.1007/978-1-4939-3816-2_24. PubMed PMID: 27604732.
596. Rasche D, Tronnier VM. Clinical Significance of Invasive Motor Cortex Stimulation for Trigeminal Facial Neuropathic Pain Syndromes. Neurosurgery. 2016;79(5):655-66. Epub 2016/10/21. doi: 10.1227/neu.0000000000001353. PubMed PMID: 27465843.
597. Rigon A, Voss MW, Turkstra LS, Mutlu B, Duff MC. Frontal and Temporal Structural Connectivity Is Associated with Social Communication Impairment Following Traumatic Brain Injury. J Int Neuropsychol Soc. 2016;22(7):705-16. Epub 2016/07/14. doi: 10.1017/s1355617716000539. PubMed PMID: 27405965; PubMed Central PMCID: PMCPMC5823263.

598. Roberts A, Sithole A, Sedghi M, Walker CA, Quinn TM. Minimal adverse effects profile following implantation of periauricular percutaneous electrical nerve field stimulators: a retrospective cohort study. Med Devices (Auckl). 2016;9:389-93. Epub 2016/11/16. doi: 10.2147/mder.S107426. PubMed PMID: 27843360; PubMed Central PMCID: PMCPMC5098779.

599. Sacco K, Galetto V, Dimitri D, Geda E, Perotti F, Zettin M, et al. Concomitant Use of Transcranial Direct Current Stimulation and Computer-Assisted Training for the Rehabilitation of Attention in Traumatic Brain Injured Patients: Behavioral and Neuroimaging Results. Front Behav Neurosci. 2016;10:57. Epub 2016/04/12. doi: 10.3389/fnbeh.2016.00057. PubMed PMID: 27065823; PubMed Central PMCID: PMCPMC4814724.

600. Sasso V, Bisicchia E, Latini L, Ghiglieri V, Cacace F, Carola V, et al. Repetitive transcranial magnetic stimulation reduces remote apoptotic cell death and inflammation after focal brain injury. J Neuroinflammation. 2016;13(1):150. Epub 2016/06/16. doi: 10.1186/s12974-016-0616-5. PubMed PMID: 27301743; PubMed Central PMCID: PMCPMC4908713.

601. Schiff ND. Central thalamic deep brain stimulation to support anterior forebrain mesocircuit function in the severely injured brain. J Neural Transm (Vienna). 2016;123(7):797-806. Epub 2016/04/27.

doi: 10.1007/s00702-016-1547-0. PubMed PMID: 27113938.

602. Sharma M, Naik V, Deogaonkar M. Emerging applications of deep brain stimulation. J Neurosurg Sci. 2016;60(2):242-55. Epub 2016/01/21. PubMed PMID: 26788743.

603. Sharpee TO, Destexhe A, Kawato M, Sekulić V, Skinner FK, Wójcik DK, et al. 25th Annual Computational Neuroscience Meeting: CNS-2016. BMC Neurosci. 2016;17 Suppl 1(Suppl 1):54. Epub 2016/08/19. doi: 10.1186/s12868-016-0283-6. PubMed PMID: 27534393; PubMed Central PMCID: PMCPMC5001212.

604. Speth J, Speth C. Motor imagery in REM sleep is increased by transcranial direct current stimulation of the left motor cortex (C3). Neuropsychologia. 2016;86:57-65. Epub 2016/04/16. doi: 10.1016/j.neuropsychologia.2016.04.010. PubMed PMID: 27079954.

605. Tang WC, Hsu YC, Wang CC, Hu CY, Chio CC, Kuo JR. Early electroacupuncture treatment ameliorates neuroinflammation in rats with traumatic brain injury. BMC Complement Altern Med. 2016;16(1):470. Epub 2016/11/18. doi: 10.1186/s12906-016-1457-6. PubMed PMID: 27852302; PubMed Central PMCID: PMCPMC5112630.

606. Turner DA. Frontiers in Neuroscience

Enhanced Functional Outcome from Traumatic Brain Injury with

Brain–Machine Interface Neuromodulation: Neuroprosthetic Scaling in Relation to Injury Severity. In: Laskowitz D, Grant G, editors. Translational Research in Traumatic Brain Injury. Boca Raton (FL): CRC Press/Taylor and Francis Group
© 2016 by Taylor & Francis Group, LLC.; 2016.
607. Werner C, Byhahn M, Hesse S. Non-invasive brain stimulation to promote alertness and awareness in chronic patients with disorders of consciousness: Low-level, near-infrared laser stimulation vs. focused shock wave therapy. Restor Neurol Neurosci. 2016;34(4):561-9. Epub 2016/04/16. doi: 10.3233/rnn-150624. PubMed PMID: 27080072.
608. Wolf JA, Koch PF. Disruption of Network Synchrony and Cognitive Dysfunction After Traumatic Brain Injury. Front Syst Neurosci. 2016;10:43. Epub 2016/06/01. doi: 10.3389/fnsys.2016.00043. PubMed PMID: 27242454; PubMed Central PMCID: PMCPMC4868948.
609. Yang N, Yue Y, Pan JZ, Zuo MZ, Shi Y, Zhou SZ, et al. Changes in the Bispectral Index in Response to Loss of Consciousness and No Somatic Movement to Nociceptive Stimuli in Elderly Patients. Chin Med J (Engl). 2016;129(4):410-6. Epub 2016/02/18. doi: 10.4103/0366-6999.176083. PubMed PMID: 26879014; PubMed Central PMCID: PMCPMC4800841.

610. Yoon KJ, Lee YT, Chae SW, Park CR, Kim DY. Effects of anodal transcranial direct current stimulation (tDCS) on behavioral and spatial memory during the early stage of traumatic brain injury in the rats. J Neurol Sci. 2016;362:314-20. Epub 2016/03/06. doi: 10.1016/j.jns.2016.02.005. PubMed PMID: 26944170.
611. Bai Y, Xia X, Kang J, Yang Y, He J, Li X. TDCS modulates cortical excitability in patients with disorders of consciousness. Neuroimage Clin. 2017;15:702-9. Epub 2017/07/14. doi: 10.1016/j.nicl.2017.01.025. PubMed PMID: 28702347; PubMed Central PMCID: PMCPMC5487253.
612. Bodart O, Gosseries O, Wannez S, Thibaut A, Annen J, Boly M, et al. Measures of metabolism and complexity in the brain of patients with disorders of consciousness. Neuroimage Clin. 2017;14:354-62. Epub 2017/02/28. doi: 10.1016/j.nicl.2017.02.002. PubMed PMID: 28239544; PubMed Central PMCID: PMCPMC5318348.
613. Chantsoulis M, Półrola P, Góral-Półrola J, Hajdukiewicz A, Supiński J, Kropotov JD, et al. Application of ERPs neuromarkers for assessment and treatment of a patient with chronic crossed aphasia after severe TBI and long-term coma - Case Report. Ann Agric Environ Med. 2017;24(1):141-7. Epub 2017/04/06. doi: 10.5604/12321966.1232770 PubMed PMID: 28378976.

614. Chari A, Hentall ID, Papadopoulos MC, Pereira EA. Surgical Neurostimulation for Spinal Cord Injury. Brain Sci. 2017;7(2). Epub 2017/02/18. doi: 10.3390/brainsci7020018. PubMed PMID: 28208601; PubMed Central PMCID: PMCPMC5332961.

615. Conde V, Andreasen SH, Petersen TH, Larsen KB, Madsen K, Andersen KW, et al. Alterations in the brain's connectome during recovery from severe traumatic brain injury: protocol for a longitudinal prospective study. BMJ Open. 2017;7(6):e016286. Epub 2017/06/16. doi: 10.1136/bmjopen-2017-016286. PubMed PMID: 28615277; PubMed Central PMCID: PMCPMC5541610.

616. Crosson B, Hampstead BM, Krishnamurthy LC, Krishnamurthy V, McGregor KM, Nocera JR, et al. Advances in neurocognitive rehabilitation research from 1992 to 2017: The ascension of neural plasticity. Neuropsychology. 2017;31(8):900-20. Epub 2017/09/01. doi: 10.1037/neu0000396. PubMed PMID: 28857600; PubMed Central PMCID: PMCPMC5788715.

617. Dang B, Chen W, He W, Chen G. Rehabilitation Treatment and Progress of Traumatic Brain Injury Dysfunction. Neural Plast. 2017;2017:1582182. Epub 2017/05/12. doi: 10.1155/2017/1582182. PubMed PMID: 28491478; PubMed Central PMCID: PMCPMC5405588.

618. de Amorim RLO, Brunoni AR, de Oliveira MAF, Zaninotto ALC, Nagumo MM, Guirado VMP, et al. Transcranial Direct Current Stimulation for Post-Concussion Syndrome: Study Protocol for a Randomized Crossover Trial. Front Neurol. 2017;8:164. Epub 2017/05/18. doi: 10.3389/fneur.2017.00164. PubMed PMID: 28512443; PubMed Central PMCID: PMCPMC5411433.
619. Debruyne P, Wijns W. Cardio-Neuromodulation: The Right-Sided Approach. JACC Clin Electrophysiol. 2017;3(9):1056-7. Epub 2018/05/16. doi: 10.1016/j.jacep.2016.12.027. PubMed PMID: 29759711.
620. Dimitri D, De Filippis D, Galetto V, Zettin M. Evaluation of the effectiveness of transcranial direct current stimulation (tDCS) and psychosensory stimulation through DOCS scale in a minimally conscious subject. Neurocase. 2017;23(2):96-104. Epub 2017/03/30. doi: 10.1080/13554794.2017.1305112. PubMed PMID: 28347207.
621. Edwards EK, Christie AD. Assessment of motor cortex excitability and inhibition during a cognitive task in individuals with concussion. Brain Inj. 2017;31(10):1348-55. Epub 2017/06/29. doi: 10.1080/02699052.2017.1327671. PubMed PMID: 28657363.
622. Estraneo A, Pascarella A, Moretta P, Masotta O, Fiorenza S,

Chirico G, et al. Repeated transcranial direct current stimulation in prolonged disorders of consciousness: A double-blind cross-over study. J Neurol Sci. 2017;375:464-70. Epub 2017/03/23. doi: 10.1016/j.jns.2017.02.036. PubMed PMID: 28320187.
623. Gao YL, Zhang Y, Cao JP, Wu SB, Cai XH, Zhang YC, et al. Regulation of the endoplasmic reticulum stress response and neuroprotective effects of acupuncture on brain injury caused by heroin addiction. Acupunct Med. 2017;35(5):366-73. Epub 2017/05/27. doi: 10.1136/acupmed-2016-011220. PubMed PMID: 28546432.
624. Hara T, Abo M, Sasaki N, Yamada N, Niimi M, Kenmoku M, et al. Improvement of higher brain dysfunction after brain injury by repetitive transcranial magnetic stimulation and intensive rehabilitation therapy: case report. Neuroreport. 2017;28(13):800-7. Epub 2017/07/14. doi: 10.1097/wnr.0000000000000830. PubMed PMID: 28704292.
625. Hsieh TH, Lee HHC, Hameed MQ, Pascual-Leone A, Hensch TK, Rotenberg A. Trajectory of Parvalbumin Cell Impairment and Loss of Cortical Inhibition in Traumatic Brain Injury. Cereb Cortex. 2017;27(12):5509-24. Epub 2016/12/03. doi: 10.1093/cercor/bhw318. PubMed PMID: 27909008; PubMed Central PMCID: PMCPMC6075565.

626. Huang MX, Swan AR, Quinto AA, Matthews S, Harrington DL, Nichols S, et al. A pilot treatment study for mild traumatic brain injury: Neuroimaging changes detected by MEG after low-intensity pulse-based transcranial electrical stimulation. Brain Inj. 2017;31(13-14):1951-63. Epub 2017/09/20. doi: 10.1080/02699052.2017.1363409. PubMed PMID: 28925734.
627. Huang Z, Thiebaud N, Fadool DA. Differential serotonergic modulation across the main and accessory olfactory bulbs. J Physiol. 2017;595(11):3515-33. Epub 2017/02/24. doi: 10.1113/jp273945. PubMed PMID: 28229459; PubMed Central PMCID: PMCPMC5451723.
628. Hurme M, Koivisto M, Revonsuo A, Railo H. Early processing in primary visual cortex is necessary for conscious and unconscious vision while late processing is necessary only for conscious vision in neurologically healthy humans. Neuroimage. 2017;150:230-8. Epub 2017/03/04. doi: 10.1016/j.neuroimage.2017.02.060. PubMed PMID: 28254455.
629. Ismail FY, Fatemi A, Johnston MV. Cerebral plasticity: Windows of opportunity in the developing brain. Eur J Paediatr Neurol. 2017;21(1):23-48. Epub 2016/08/28. doi: 10.1016/j.ejpn.2016.07.007. PubMed PMID: 27567276.
630. Jablawi F, Kweider H, Nikoubashman O, Clusmann H,

Schubert GA. Twist Drill Procedure for Chronic Subdural Hematoma Evacuation: An Analysis of Predictors for Treatment Success. World Neurosurg. 2017;100:480-6. Epub 2017/01/23. doi: 10.1016/j.wneu.2017.01.037. PubMed PMID: 28109862.

631. Kamins J, Bigler E, Covassin T, Henry L, Kemp S, Leddy JJ, et al. What is the physiological time to recovery after concussion? A systematic review. Br J Sports Med. 2017;51(12):935-40. Epub 2017/04/30. doi: 10.1136/bjsports-2016-097464. PubMed PMID: 28455363.

632. Kim HJ, Han SJ. Anodal Transcranial Direct Current Stimulation Provokes Neuroplasticity in Repetitive Mild Traumatic Brain Injury in Rats. Neural Plast. 2017;2017:1372946. Epub 2017/08/05. doi: 10.1155/2017/1372946. PubMed PMID: 28770112; PubMed Central PMCID: PMCPMC5523234.

633. Kovacic K, Hainsworth K, Sood M, Chelimsky G, Unteutsch R, Nugent M, et al. Neurostimulation for abdominal pain-related functional gastrointestinal disorders in adolescents: a randomised, double-blind, sham-controlled trial. Lancet Gastroenterol Hepatol. 2017;2(10):727-37. Epub 2017/08/23. doi: 10.1016/s2468-1253(17)30253-4. PubMed PMID: 28826627.

634. Kukke SN, Brewer CC, Zalewski C, King KA, Damiano D, Alter KE, et al. Hearing Safety From Single- and Double-Pulse

Transcranial Magnetic Stimulation in Children and Young Adults. J Clin Neurophysiol. 2017;34(4):340-7. Epub 2017/06/24. doi: 10.1097/wnp.0000000000000372. PubMed PMID: 28644204; PubMed Central PMCID: PMCPMC5484061.
635. Kurt E, Henssen D, Steegers M, Staal M, Beese U, Maarrawi J, et al. Motor Cortex Stimulation in Patients Suffering from Chronic Neuropathic Pain: Summary of Expert Meeting and Premeeting Questionnaire, Combined with Literature Review. World Neurosurg. 2017;108:254-63. Epub 2017/09/09. doi: 10.1016/j.wneu.2017.08.168. PubMed PMID: 28882715.
636. Landgraf MN, König H, Hannibal I, Langhagen T, Bonfert MV, Klose B, et al. [Migraine in children and adolescents-brain and muscle? : Another example of why children are not small adults]. Nervenarzt. 2017;88(12):1402-10. Epub 2017/09/29. doi: 10.1007/s00115-017-0428-6. PubMed PMID: 28956079.
637. Lavano A, Guzzi G, M DER, Romano M, Della Torre A, Vescio G, et al. Minimally invasive motor cortex stimulation for Parkinson's disease. J Neurosurg Sci. 2017;61(1):77-87. Epub 2015/04/18. doi: 10.23736/s0390-5616.16.03246-x. PubMed PMID: 25881652.
638. Lewis GN, Hume PA, Stavric V, Brown SR, Taylor D. New Zealand rugby health study: motor cortex excitability in retired

elite and community level rugby players. N Z Med J. 2017;130(1448):34-44. Epub 2017/01/13. PubMed PMID: 28081555.

639. Liu LA, Wang ZQ, Fu JJ, Du QQ, Zhang YY, Jiao CC, et al. [Comparative Observation on Electroacupuncture and Manual Acupuncture in Rabbits with Facial Nerve Injury by Electron Microscope]. Zhen Ci Yan Jiu. 2017;42(5):423-8. Epub 2017/11/07. PubMed PMID: 29105471.

640. Lu X, Bao X, Li J, Zhang G, Guan J, Gao Y, et al. High-frequency repetitive transcranial magnetic stimulation for treating moderate traumatic brain injury in rats: A pilot study. Exp Ther Med. 2017;13(5):2247-54. Epub 2017/06/02. doi: 10.3892/etm.2017.4283. PubMed PMID: 28565833; PubMed Central PMCID: PMCPMC5443170.

641. Mancuso M, Abbruzzese L, Canova S, Landi G, Rossi S, Santarnecchi E. Transcranial Random Noise Stimulation Does Not Improve Behavioral and Neurophysiological Measures in Patients with Subacute Vegetative-Unresponsive Wakefulness State (VS-UWS). Front Hum Neurosci. 2017;11:524. Epub 2017/11/23. doi: 10.3389/fnhum.2017.00524. PubMed PMID: 29163104; PubMed Central PMCID: PMCPMC5681535.

642. Markiewcz R. The use of EEG Biofeedback/Neurofeedback in

psychiatric rehabilitation. Psychiatr Pol. 2017;51(6):1095-106. Epub 2018/02/13. doi: 10.12740/pp/68919. PubMed PMID: 29432505.

643. Mattogno PP, Barbagallo G, Iacopino G, Pappalardo G, G LAR, Signorelli F, et al. Recovery from Chronic Diseases of Consciousness: State of the Art in Neuromodulation for Persistent Vegetative State and Minimally Conscious State. Acta Neurochir Suppl. 2017;124:19-25. Epub 2017/01/26. doi: 10.1007/978-3-319-39546-3_4. PubMed PMID: 28120048.

644. Meehan SK, Mirdamadi JL, Martini DN, Broglio SP. Changes in Cortical Plasticity in Relation to a History of Concussion during Adolescence. Front Hum Neurosci. 2017;11:5. Epub 2017/02/02. doi: 10.3389/fnhum.2017.00005. PubMed PMID: 28144218; PubMed Central PMCID: PMCPMC5239801.

645. Mitchell C, Bowen A, Tyson S, Butterfint Z, Conroy P. Interventions for dysarthria due to stroke and other adult-acquired, non-progressive brain injury. Cochrane Database Syst Rev. 2017;1(1):Cd002088. Epub 2017/01/26. doi: 10.1002/14651858.CD002088.pub3. PubMed PMID: 28121021; PubMed Central PMCID: PMCPMC6464736 Institute for Health Research Doctoral Research Fellowship (DRF-2014-07-043) and is registered with the Health and Care Professions Council, UK.

Audrey Bowen's salary is part funded by Stroke Association and partly by the National Institute for Health Research Collaboration for Leadership in Applied Health Research and Care (NIHR CLAHRC) Greater Manchester. Audrey Bowen has been involved in a study included in this review (Bowen 2012). She did not contribute to the assessment or interpretation of this study. Sarah Tyson: none known. Zoe Butterfint: none known. Paul Conroy is a speech and language therapist, member of the Royal College of Speech and Language Therapists, and is registered with the Health and Care Professions Council, UK. This report presents independent research funded by the National Institute for Health Research (NIHR). The views expressed are those of the review authors and not necessarily those of the NHS, the NIHR, or the Department of Health.

646. Murphy C, Zafar H, Sharif F. An updated review of cardiac devices in heart failure. Ir J Med Sci. 2017;186(4):909-19. Epub 2017/03/28. doi: 10.1007/s11845-017-1597-9. PubMed PMID: 28343276.

647. Naro A, Bruno R, Leo A, Russo M, Salviera C, Bramanti A, et al. Twist and turn into chronic disorders of consciousness: Potential role of the auditory stapedial reflex. Restor Neurol Neurosci. 2017;35(1):77-85. Epub 2017/01/07. doi: 10.3233/rnn-160655.

PubMed PMID: 28059800.

648. Naro A, Leo A, Manuli A, Cannavò A, Bramanti A, Bramanti P, et al. How far can we go in chronic disorders of consciousness differential diagnosis? The use of neuromodulation in detecting internal and external awareness. Neuroscience. 2017;349:165-73. Epub 2017/03/14. doi: 10.1016/j.neuroscience.2017.02.053. PubMed PMID: 28285941.

649. Naro A, Leo A, Russo M, Casella C, Buda A, Crespantini A, et al. Breakthroughs in the spasticity management: Are non-pharmacological treatments the future? J Clin Neurosci. 2017;39:16-27. Epub 2017/03/07. doi: 10.1016/j.jocn.2017.02.044. PubMed PMID: 28262404.

650. O'Neil-Pirozzi TM, Doruk D, Thomson JM, Fregni F. Immediate memory and electrophysiologic effects of prefrontal cortex transcranial direct current stimulation on neurotypical individuals and individuals with chronic traumatic brain injury: a pilot study. Int J Neurosci. 2017;127(7):592-600. Epub 2016/07/28. doi: 10.1080/00207454.2016.1216415. PubMed PMID: 27453334.

651. Pearn ML, Niesman IR, Egawa J, Sawada A, Almenar-Queralt A, Shah SB, et al. Pathophysiology Associated with Traumatic Brain Injury: Current Treatments and Potential Novel Therapeutics. Cell Mol Neurobiol. 2017;37(4):571-85. Epub 2016/07/08. doi:

10.1007/s10571-016-0400-1. PubMed PMID: 27383839.

652. Ragazzoni A, Cincotta M, Giovannelli F, Cruse D, Young GB, Miniussi C, et al. Clinical neurophysiology of prolonged disorders of consciousness: From diagnostic stimulation to therapeutic neuromodulation. Clin Neurophysiol. 2017;128(9):1629-46. Epub 2017/07/21. doi: 10.1016/j.clinph.2017.06.037. PubMed PMID: 28728060.

653. Santarnecchi E, Pascual-Leone A. The Illusion of the Perfect Brain Enhancer. Cerebrum. 2017;2017. Epub 2018/09/14. PubMed PMID: 30210662; PubMed Central PMCID: PMCPMC6132045.

654. Schnakers C, Monti MM. Disorders of consciousness after severe brain injury: therapeutic options. Curr Opin Neurol. 2017;30(6):573-9. Epub 2017/09/14. doi: 10.1097/wco.0000000000000495. PubMed PMID: 28901969.

655. Seeger TA, Kirton A, Esser MJ, Gallagher C, Dunn J, Zewdie E, et al. Cortical excitability after pediatric mild traumatic brain injury. Brain Stimul. 2017;10(2):305-14. Epub 2016/12/06. doi: 10.1016/j.brs.2016.11.011. PubMed PMID: 27916406.

656. Shin SS, Pelled G. Novel Neuromodulation Techniques to Assess Interhemispheric Communication in Neural Injury and Neurodegenerative Diseases. Front Neural Circuits. 2017;11:15. Epub 2017/03/25. doi: 10.3389/fncir.2017.00015. PubMed PMID:

28337129; PubMed Central PMCID: PMCPMC5343068.
657. Srejic U, Larson P, Bickler PE. Little Black Boxes: Noncardiac Implantable Electronic Medical Devices and Their Anesthetic and Surgical Implications. Anesth Analg. 2017;125(1):124-38. Epub 2017/05/11. doi: 10.1213/ane.0000000000001983. PubMed PMID: 28489640.
658. Thibaut A, Wannez S, Donneau AF, Chatelle C, Gosseries O, Bruno MA, et al. Controlled clinical trial of repeated prefrontal tDCS in patients with chronic minimally conscious state. Brain Inj. 2017;31(4):466-74. Epub 2017/03/11. doi: 10.1080/02699052.2016.1274776. PubMed PMID: 28281845.
659. Tu-Chan AP, Natraj N, Godlove J, Abrams G, Ganguly K. Effects of somatosensory electrical stimulation on motor function and cortical oscillations. J Neuroeng Rehabil. 2017;14(1):113. Epub 2017/11/15. doi: 10.1186/s12984-017-0323-1. PubMed PMID: 29132379; PubMed Central PMCID: PMCPMC5683582.
660. Vanhoecke J, Hariz M. Deep brain stimulation for disorders of consciousness: Systematic review of cases and ethics. Brain Stimul. 2017;10(6):1013-23. Epub 2017/10/03. doi: 10.1016/j.brs.2017.08.006. PubMed PMID: 28966051.
661. Vanzan S, Wilkinson D, Ferguson H, Pullicino P, Sakel M. Behavioural improvement in a minimally conscious state after

caloric vestibular stimulation: evidence from two single case studies. Clin Rehabil. 2017;31(4):500-7. Epub 2016/04/29. doi: 10.1177/0269215516646167. PubMed PMID: 27121862.

662. Verdugo-Diaz L, Estrada-Rojo F, Garcia-Espinoza A, Hernandez-Lopez E, Hernandez-Chavez A, Guzman-Uribe C, et al. Effect of Intermediate-Frequency Repetitive Transcranial Magnetic Stimulation on Recovery following Traumatic Brain Injury in Rats. Biomed Res Int. 2017;2017:4540291. Epub 2018/01/11. doi: 10.1155/2017/4540291. PubMed PMID: 29318150; PubMed Central PMCID: PMCPMC5727566.

663. Wilke S, List J, Mekle R, Lindenberg R, Bukowski M, Ott S, et al. No Effect of Anodal Transcranial Direct Current Stimulation on Gamma-Aminobutyric Acid Levels in Patients with Recurrent Mild Traumatic Brain Injury. J Neurotrauma. 2017;34(2):281-90. Epub 2016/07/10. doi: 10.1089/neu.2016.4399. PubMed PMID: 27393381.

664. Wu X, Zhang C, Feng J, Mao Q, Gao G, Jiang J. Right median nerve electrical stimulation for acute traumatic coma (the Asia Coma Electrical Stimulation trial): study protocol for a randomised controlled trial. Trials. 2017;18(1):311. Epub 2017/07/12. doi: 10.1186/s13063-017-2045-x. PubMed PMID: 28693604; PubMed Central PMCID: PMCPMC5504835.

665. Xia X, Bai Y, Zhou Y, Yang Y, Xu R, Gao X, et al. Effects of 10 Hz Repetitive Transcranial Magnetic Stimulation of the Left Dorsolateral Prefrontal Cortex in Disorders of Consciousness. Front Neurol. 2017;8:182. Epub 2017/05/19. doi: 10.3389/fneur.2017.00182. PubMed PMID: 28515709; PubMed Central PMCID: PMCPMC5413493.

666. Xia X, Liu Y, Bai Y, Liu Z, Yang Y, Guo Y, et al. Long-lasting repetitive transcranial magnetic stimulation modulates electroencephalography oscillation in patients with disorders of consciousness. Neuroreport. 2017;28(15):1022-9. Epub 2017/09/14. doi: 10.1097/wnr.0000000000000886. PubMed PMID: 28902713.

667. Yamamoto T, Watanabe M, Obuchi T, Kobayashi K, Oshima H, Fukaya C, et al. Spinal Cord Stimulation for Vegetative State and Minimally Conscious State: Changes in Consciousness Level and Motor Function. Acta Neurochir Suppl. 2017;124:37-42. Epub 2017/01/26. doi: 10.1007/978-3-319-39546-3_6. PubMed PMID: 28120050.

668. Yasen AL, Howell DR, Chou LS, Pazzaglia AM, Christie AD. Cortical and Physical Function after Mild Traumatic Brain Injury. Med Sci Sports Exerc. 2017;49(6):1066-71. Epub 2017/05/17. doi: 10.1249/mss.0000000000001217. PubMed PMID: 28509819.

669. Yu YT, Yang Y, Wang LB, Fang JL, Chen YY, He JH, et al.

Transcutaneous auricular vagus nerve stimulation in disorders of consciousness monitored by fMRI: The first case report. Brain Stimul. 2017;10(2):328-30. Epub 2016/12/27. doi: 10.1016/j.brs.2016.12.004. PubMed PMID: 28017322.

670. Zhang X, Hu Y, Tao W, Zhu H, Xiao D, Li Y. The Effect of Motor Cortex Stimulation on Central Poststroke Pain in a Series of 16 Patients With a Mean Follow-Up of 28 Months. Neuromodulation. 2017;20(5):492-6. Epub 2017/01/20. doi: 10.1111/ner.12547. PubMed PMID: 28102925.

671. Zhang Y, Song W, Du J, Huo S, Shan G, Li R. Transcranial Direct Current Stimulation in Patients with Prolonged Disorders of Consciousness: Combined Behavioral and Event-Related Potential Evidence. Front Neurol. 2017;8:620. Epub 2017/12/07. doi: 10.3389/fneur.2017.00620. PubMed PMID: 29209270; PubMed Central PMCID: PMCPMC5702306.

672. Zhu Y, Deng L, Tang H, Gao X, Wang Y, Guo K, et al. Electroacupuncture improves neurobehavioral function and brain injury in rat model of intracerebral hemorrhage. Brain Res Bull. 2017;131:123-32. Epub 2017/04/12. doi: 10.1016/j.brainresbull.2017.04.003. PubMed PMID: 28395933.

673. Bai Y, Xia X, Wang Y, Guo Y, Yang Y, He J, et al. Fronto-parietal coherence response to tDCS modulation in patients

with disorders of consciousness. Int J Neurosci. 2018;128(7):587-94. Epub 2017/11/22. doi: 10.1080/00207454.2017.1403440. PubMed PMID: 29160761.

674. Bell AH, Bultitude JH. Methods matter: A primer on permanent and reversible interference techniques in animals for investigators of human neuropsychology. Neuropsychologia. 2018;115:211-9. Epub 2017/09/26. doi: 10.1016/j.neuropsychologia.2017.09.019. PubMed PMID: 28943365; PubMed Central PMCID: PMCPMC6018620.

675. Bestelmeyer PEG, Davis NJ, Poliva O, Rafal RD. Neuromodulation of Right Auditory Cortex Selectively Increases Activation in Speech-Related Brain Areas in Brainstem Auditory Agnosia. Cogn Behav Neurol. 2018;31(3):151-5. Epub 2018/09/22. doi: 10.1097/wnn.0000000000000162. PubMed PMID: 30239465.

676. Black L, Gaebler-Spira D. Nonsurgical Treatment Options for Upper Limb Spasticity. Hand Clin. 2018;34(4):455-64. Epub 2018/10/06. doi: 10.1016/j.hcl.2018.06.003. PubMed PMID: 30286960.

677. Bodart O, Amico E, Gómez F, Casali AG, Wannez S, Heine L, et al. Global structural integrity and effective connectivity in patients with disorders of consciousness. Brain Stimul. 2018;11(2):358-65. Epub 2017/11/23. doi:

10.1016/j.brs.2017.11.006. PubMed PMID: 29162503.

678. Bragina OA, Lara DA, Nemoto EM, Shuttleworth CW, Semyachkina-Glushkovskaya OV, Bragin DE. Increases in Microvascular Perfusion and Tissue Oxygenation via Vasodilatation After Anodal Transcranial Direct Current Stimulation in the Healthy and Traumatized Mouse Brain. Adv Exp Med Biol. 2018;1072:27-31. Epub 2018/09/05. doi: 10.1007/978-3-319-91287-5_5. PubMed PMID: 30178319; PubMed Central PMCID: PMCPMC6294145.

679. Cavanna F, Vilas MG, Palmucci M, Tagliazucchi E. Dynamic functional connectivity and brain metastability during altered states of consciousness. Neuroimage. 2018;180(Pt B):383-95. Epub 2017/10/08. doi: 10.1016/j.neuroimage.2017.09.065. PubMed PMID: 28986208.

680. Choi GS, Kwak SG, Lee HD, Chang MC. Effect of high-frequency repetitive transcranial magnetic stimulation on chronic central pain after mild traumatic brain injury: A pilot study. J Rehabil Med. 2018;50(3):246-52. Epub 2018/02/03. doi: 10.2340/16501977-2321. PubMed PMID: 29392332.

681. Corbett M, South E, Harden M, Eldabe S, Pereira E, Sedki I, et al. Brain and spinal stimulation therapies for phantom limb pain: a systematic review. Health Technol Assess. 2018;22(62):1-94.

Epub 2018/11/09. doi: 10.3310/hta22620. PubMed PMID: 30407905; PubMed Central PMCID: PMCPMC6253324 USA) and personal fees from Abbott Laboratories (Chicago, IL, USA) and Boston Scientific (Marlborough, MA, USA) outside the submitted work.

682. Coyle HL, Ponsford J, Hoy KE. Understanding individual variability in symptoms and recovery following mTBI: A role for TMS-EEG? Neurosci Biobehav Rev. 2018;92:140-9. Epub 2018/06/10. doi: 10.1016/j.neubiorev.2018.05.027. PubMed PMID: 29885426.

683. Damar U, Gersner R, Johnstone JT, Kapur K, Collins S, Schachter S, et al. Alterations in the Timing of Huperzine A Cerebral Pharmacodynamics in the Acute Traumatic Brain Injury Setting. J Neurotrauma. 2018;35(2):393-7. Epub 2017/10/22. doi: 10.1089/neu.2017.5258. PubMed PMID: 29054135; PubMed Central PMCID: PMCPMC5784790.

684. Darracq M, Funk CM, Polyakov D, Riedner B, Gosseries O, Nieminen JO, et al. Evoked Alpha Power is Reduced in Disconnected Consciousness During Sleep and Anesthesia. Sci Rep. 2018;8(1):16664. Epub 2018/11/11. doi: 10.1038/s41598-018-34957-9. PubMed PMID: 30413741; PubMed Central PMCID: PMCPMC6226534 well as honoraria from

Medtronic during the past five years. V.B. is the Editor-in-Chief of the Acta Anaesthesiologica Belgica. G.T. receives unrestricted grant funding from Philips Healthcare and serves on the advisory board of the Allen Institute for Brain Science. All other authors declare no competing interests.

685. Debruyne P, Rossenbacker T, Collienne C, Roosen J, Ector B, Janssens L, et al. Unifocal Right-Sided Ablation Treatment for Neurally Mediated Syncope and Functional Sinus Node Dysfunction Under Computed Tomographic Guidance. Circ Arrhythm Electrophysiol. 2018;11(9):e006604. Epub 2018/10/26. doi: 10.1161/circep.118.006604. PubMed PMID: 30354289.

686. Duchalais E, Guilluy C, Nedellec S, Touvron M, Bessard A, Touchefeu Y, et al. Colorectal Cancer Cells Adhere to and Migrate Along the Neurons of the Enteric Nervous System. Cell Mol Gastroenterol Hepatol. 2018;5(1):31-49. Epub 2017/12/01. doi: 10.1016/j.jcmgh.2017.10.002. PubMed PMID: 29188232; PubMed Central PMCID: PMCPMC5696385.

687. Evans LR, Fitzgerald MC, Varma D, Mitra B. A novel approach to improving the interpretation of CT brain in trauma. Injury. 2018;49(1):56-61. Epub 2017/09/09. doi: 10.1016/j.injury.2017.08.056. PubMed PMID: 28882376.

688. Gao F, Zhang Q, Li Y, Tai Y, Xin X, Wang X, et al.

Transcutaneous electrical acupoint stimulation for prevention of postoperative delirium in geriatric patients with silent lacunar infarction: a preliminary study. Clin Interv Aging. 2018;13:2127-34. Epub 2018/11/15. doi: 10.2147/cia.S183698. PubMed PMID: 30425466; PubMed Central PMCID: PMCPMC6205526.

689. Guger C, Spataro R, Pellas F, Allison BZ, Heilinger A, Ortner R, et al. Assessing Command-Following and Communication With Vibro-Tactile P300 Brain-Computer Interface Tools in Patients With Unresponsive Wakefulness Syndrome. Front Neurosci. 2018;12:423. Epub 2018/07/17. doi: 10.3389/fnins.2018.00423. PubMed PMID: 30008659; PubMed Central PMCID: PMCPMC6034093.

690. Guzmán-Mejía F, López-Rubalcava C, González-Espinosa C. Stimulation of nAchRα7 Receptor Inhibits TNF Synthesis and Secretion in Response to LPS Treatment of Mast Cells by Targeting ERK1/2 and TACE Activation. J Neuroimmune Pharmacol. 2018;13(1):39-52. Epub 2017/08/20. doi: 10.1007/s11481-017-9760-7. PubMed PMID: 28822039.

691. Hayashi CY, Neville IS, Rodrigues PA, Galhardoni R, Brunoni AR, Zaninotto AL, et al. Altered Intracortical Inhibition in Chronic Traumatic Diffuse Axonal Injury. Front Neurol. 2018;9:189. Epub 2018/04/13. doi: 10.3389/fneur.2018.00189. PubMed PMID:

29643831; PubMed Central PMCID: PMCPMC5882787.

692. He F, Wu M, Meng F, Hu Y, Gao J, Chen Z, et al. Effects of 20 Hz Repetitive Transcranial Magnetic Stimulation on Disorders of Consciousness: A Resting-State Electroencephalography Study. Neural Plast. 2018;2018:5036184. Epub 2018/05/18. doi: 10.1155/2018/5036184. PubMed PMID: 29770146; PubMed Central PMCID: PMCPMC5889874.

693. Hussein AE, Esfahani DR, Moisak GI, Rzaev JA, Slavin KV. Motor Cortex Stimulation for Deafferentation Pain. Curr Pain Headache Rep. 2018;22(6):45. Epub 2018/05/26. doi: 10.1007/s11916-018-0697-1. PubMed PMID: 29796941.

694. Iliceto A, Seiler RL, Sarkar K. Repetitive Transcranial Magnetic Stimulation for Treatment of Depression in a Patient With Severe Traumatic Brain Injury. Ochsner J. 2018;18(3):264-7. Epub 2018/10/03. doi: 10.31486/toj.17.0075. PubMed PMID: 30275792; PubMed Central PMCID: PMCPMC6162118.

695. Ishai R, Knoll RM, Chen JX, Wong K, Reinshagen KL, Nadol JB, Jr., et al. Otopathologic Changes in the Cochlea following Head Injury without Temporal Bone Fracture. Otolaryngol Head Neck Surg. 2018;159(3):526-34. Epub 2018/06/13. doi: 10.1177/0194599818769861. PubMed PMID: 29890895.

696. Jang SH, Kwon Y. Injury of leg somatotopy of corticospinal

tract at corona radiata by ventriculoperitoneal shunt: A case report. Medicine (Baltimore). 2018;97(10):e9983. Epub 2018/03/09. doi: 10.1097/md.0000000000009983. PubMed PMID: 29517704; PubMed Central PMCID: PMCPMC5882449 through the National Research Foundation of Korea (NRF) funded by the Ministry of Science, ICT, and Future Planning.Disclosures: Financial disclosure statements have been obtained, and no conflicts of interest have been reported by the authors or by any individuals in control of the content of this article.

697. Jones W, Silkworth W, Dusto N, Pelak VS, Berliner J, Buard I, et al. Central Visual Oscillopsia: Case Report and Review of the Literature. Cogn Behav Neurol. 2018;31(2):86-95. Epub 2018/06/22. doi: 10.1097/wnn.0000000000000151. PubMed PMID: 29927799.

698. Joutsa J, Shih LC, Horn A, Reich MM, Wu O, Rost NS, et al. Identifying therapeutic targets from spontaneous beneficial brain lesions. Ann Neurol. 2018;84(1):153-7. Epub 2018/07/18. doi: 10.1002/ana.25285. PubMed PMID: 30014594.

699. Kim H, Koo YS, Shin MJ, Kim SY, Shin YB, Choi BT, et al. Combination of Constraint-Induced Movement Therapy with Electroacupuncture Improves Functional Recovery following Neonatal Hypoxic-Ischemic Brain Injury in Rats. Biomed Res Int.

2018;2018:8638294. Epub 2018/03/24. doi: 10.1155/2018/8638294. PubMed PMID: 29568769; PubMed Central PMCID: PMCPMC5820667.

700. Koenig MA, Kaplan PW. Measuring Depth in Still Water: Electrophysiologic Indicators of Residual Consciousness in the Unresponsive Patient. Epilepsy Curr. 2018;18(3):147-50. Epub 2018/06/29. doi: 10.5698/1535-7597.18.3.147. PubMed PMID: 29950932; PubMed Central PMCID: PMCPMC6017691.

701. Kuffler DP. Coping with Phantom Limb Pain. Mol Neurobiol. 2018;55(1):70-84. Epub 2017/09/19. doi: 10.1007/s12035-017-0718-9. PubMed PMID: 28921418.

702. Kuffler DP. Origins of Phantom Limb Pain. Mol Neurobiol. 2018;55(1):60-9. Epub 2017/09/19. doi: 10.1007/s12035-017-0717-x. PubMed PMID: 28921474.

703. Kundu B, Brock AA, Englot DJ, Butson CR, Rolston JD. Deep brain stimulation for the treatment of disorders of consciousness and cognition in traumatic brain injury patients: a review. Neurosurg Focus. 2018;45(2):E14. Epub 2018/08/02. doi: 10.3171/2018.5.Focus18168. PubMed PMID: 30064315; PubMed Central PMCID: PMCPMC6193266.

704. Lee SA, Kim MK. Effect of Low Frequency Repetitive Transcranial Magnetic Stimulation on Depression and Cognition of

Patients with Traumatic Brain Injury: A Randomized Controlled Trial. Med Sci Monit. 2018;24:8789-94. Epub 2018/12/05. doi: 10.12659/msm.911385. PubMed PMID: 30513530; PubMed Central PMCID: PMCPMC6289027.

705. Legarreta AD, Monk SH, Kirby PW, Brett BL, Yengo-Kahn AM, Bhatia A, et al. Long-Term Neuroimaging Findings in American Football Players: Systematic Review. World Neurosurg. 2018;120:e365-e79. Epub 2018/08/26. doi: 10.1016/j.wneu.2018.08.079. PubMed PMID: 30144599.

706. Leung A, Metzger-Smith V, He Y, Cordero J, Ehlert B, Song D, et al. Left Dorsolateral Prefrontal Cortex rTMS in Alleviating MTBI Related Headaches and Depressive Symptoms. Neuromodulation. 2018;21(4):390-401. Epub 2017/05/31. doi: 10.1111/ner.12615. PubMed PMID: 28557049.

707. Li F, Ball S, Katz B, Smith A. Case report of syncope during a transcranial direct current stimulation experiment in a healthy adult participant. Brain Stimul. 2018;11(5):1201-2. Epub 2018/08/28. doi: 10.1016/j.brs.2018.06.008. PubMed PMID: 30146042.

708. Li Y, Luo X, Wan M, Li J, Wang H, Wei D, et al. The effectiveness of non-invasive brain stimulation on arousal and alertness in patients in coma or persistent vegetative state after traumatic brain injury: Protocol of systematic review and network

meta-analysis. Medicine (Baltimore). 2018;97(37):e12321. Epub 2018/09/15. doi: 10.1097/md.0000000000012321. PubMed PMID: 30212977; PubMed Central PMCID: PMCPMC6155968.

709. Liu J, Xue X, Wu Y, Yang C, Li N, Li H. Efficacy and safety of electro-acupuncture treatment in improving the consciousness of patients with traumatic brain injury: study protocol for a randomized controlled trial. Trials. 2018;19(1):296. Epub 2018/05/31. doi: 10.1186/s13063-018-2687-3. PubMed PMID: 29843761; PubMed Central PMCID: PMCPMC5975471.

710. Liu X, Meng F, Gao J, Zhang L, Zhou Z, Pan G, et al. Behavioral and Resting State Functional Connectivity Effects of High Frequency rTMS on Disorders of Consciousness: A Sham-Controlled Study. Front Neurol. 2018;9:982. Epub 2018/12/07. doi: 10.3389/fneur.2018.00982. PubMed PMID: 30519211; PubMed Central PMCID: PMCPMC6258881.

711. Martens G, Lejeune N, O'Brien AT, Fregni F, Martial C, Wannez S, et al. Randomized controlled trial of home-based 4-week tDCS in chronic minimally conscious state. Brain Stimul. 2018;11(5):982-90. Epub 2018/05/16. doi: 10.1016/j.brs.2018.04.021. PubMed PMID: 29759943.

712. Mayer AR, Kaushal M, Dodd AB, Hanlon FM, Shaff NA, Mannix R, et al. Advanced biomarkers of pediatric mild traumatic

brain injury: Progress and perils. Neurosci Biobehav Rev. 2018;94:149-65. Epub 2018/08/14. doi: 10.1016/j.neubiorev.2018.08.002. PubMed PMID: 30098989; PubMed Central PMCID: PMCPMC6221189.

713. Naro A, Bramanti A, Leo A, Bramanti P, Calabrò RS. Metaplasticity: A Promising Tool to Disentangle Chronic Disorders of Consciousness Differential Diagnosis. Int J Neural Syst. 2018;28(6):1750059. Epub 2018/01/27. doi: 10.1142/s0129065717500599. PubMed PMID: 29370729.

714. Naro A, Bramanti A, Leo A, Cacciola A, Manuli A, Bramanti P, et al. Shedding new light on disorders of consciousness diagnosis: The dynamic functional connectivity. Cortex. 2018;103:316-28. Epub 2018/04/24. doi: 10.1016/j.cortex.2018.03.029. PubMed PMID: 29684751.

715. Naro A, Calabrò RS, Leo A, Russo M, Milardi D, Cannavò A, et al. Bridging the Gap Towards Awareness Detection in Disorders of Consciousness: An Experimental Study on the Mirror Neuron System. Brain Topogr. 2018;31(4):623-39. Epub 2018/02/09. doi: 10.1007/s10548-018-0628-9. PubMed PMID: 29417320.

716. Nelson DV, Esty ML. Minute Pulsed Electromagnetic Neurostimulation for Mixed Trauma Syndromes. J Evid Based Integr Med. 2018;23:2515690x18770136. Epub 2018/04/26. doi:

10.1177/2515690x18770136. PubMed PMID: 29692181; PubMed Central PMCID: PMCPMC5987890.

717. Paxman E, Stilling J, Mercier L, Debert CT. Repetitive transcranial magnetic stimulation (rTMS) as a treatment for chronic dizziness following mild traumatic brain injury. BMJ Case Rep. 2018;2018. Epub 2018/11/07. doi: 10.1136/bcr-2018-226698. PubMed PMID: 30396889; PubMed Central PMCID: PMCPMC6229180.

718. Pearce AJ, Rist B, Fraser CL, Cohen A, Maller JJ. Neurophysiological and cognitive impairment following repeated sports concussion injuries in retired professional rugby league players. Brain Inj. 2018;32(4):498-505. Epub 2018/02/02. doi: 10.1080/02699052.2018.1430376. PubMed PMID: 29388850.

719. Pei LX, Zhang W, Song YF, Geng H, Chen L, Wu XL, et al. [Electroacupuncture of "Tianshu" (ST 25) Suppresses Visceral Pain Possibly by Down-regulating Mast Cell Activation, and Tryptase and SP Expression in Rats with Post-infectious Irritable Bowel Syndrome]. Zhen Ci Yan Jiu. 2018;43(7):419-23. Epub 2018/08/11. doi: 10.13702/j.1000-0607.180211. PubMed PMID: 30094977.

720. Quesada C, Pommier B, Fauchon C, Bradley C, Créac'h C, Vassal F, et al. Robot-Guided Neuronavigated Repetitive Transcranial Magnetic Stimulation (rTMS) in Central Neuropathic

Pain. Arch Phys Med Rehabil. 2018;99(11):2203-15.e1. Epub 2018/05/12. doi: 10.1016/j.apmr.2018.04.013. PubMed PMID: 29750900.

721. Romeo-Guitart D, Leiva-Rodríguez T, Espinosa-Alcantud M, Sima N, Vaquero A, Domínguez-Martín H, et al. SIRT1 activation with neuroheal is neuroprotective but SIRT2 inhibition with AK7 is detrimental for disconnected motoneurons. Cell Death Dis. 2018;9(5):531. Epub 2018/05/12. doi: 10.1038/s41419-018-0553-6. PubMed PMID: 29748539; PubMed Central PMCID: PMCPMC5945655 under PCT extension.

722. Rosanova M, Fecchio M, Casarotto S, Sarasso S, Casali AG, Pigorini A, et al. Sleep-like cortical OFF-periods disrupt causality and complexity in the brain of unresponsive wakefulness syndrome patients. Nat Commun. 2018;9(1):4427. Epub 2018/10/26. doi: 10.1038/s41467-018-06871-1. PubMed PMID: 30356042; PubMed Central PMCID: PMCPMC6200777.

723. Ryan NP, Genc S, Beauchamp MH, Yeates KO, Hearps S, Catroppa C, et al. White matter microstructure predicts longitudinal social cognitive outcomes after paediatric traumatic brain injury: a diffusion tensor imaging study. Psychol Med. 2018;48(4):679-91. Epub 2017/08/07. doi: 10.1017/s0033291717002057. PubMed PMID: 28780927.

724. Sanders RD, Banks MI, Darracq M, Moran R, Sleigh J, Gosseries O, et al. Propofol-induced unresponsiveness is associated with impaired feedforward connectivity in cortical hierarchy. Br J Anaesth. 2018;121(5):1084-96. Epub 2018/10/20. doi: 10.1016/j.bja.2018.07.006. PubMed PMID: 30336853; PubMed Central PMCID: PMCPMC6208295.
725. Seven YB, Nichols NL, Kelly MN, Hobson OR, Satriotomo I, Mitchell GS. Compensatory plasticity in diaphragm and intercostal muscle utilization in a rat model of ALS. Exp Neurol. 2018;299(Pt A):148-56. Epub 2017/10/24. doi: 10.1016/j.expneurol.2017.10.015. PubMed PMID: 29056361; PubMed Central PMCID: PMCPMC5951687.
726. Shin SS, Krishnan V, Stokes W, Robertson C, Celnik P, Chen Y, et al. Transcranial magnetic stimulation and environmental enrichment enhances cortical excitability and functional outcomes after traumatic brain injury. Brain Stimul. 2018;11(6):1306-13. Epub 2018/08/08. doi: 10.1016/j.brs.2018.07.050. PubMed PMID: 30082198; PubMed Central PMCID: PMCPMC6204305.
727. Simon-Martinez C, Mailleux L, Ortibus E, Fehrenbach A, Sgandurra G, Cioni G, et al. Combining constraint-induced movement therapy and action-observation training in children with unilateral cerebral palsy: a randomized controlled trial. BMC

Pediatr. 2018;18(1):250. Epub 2018/08/02. doi: 10.1186/s12887-018-1228-2. PubMed PMID: 30064396; PubMed Central PMCID: PMCPMC6069849.

728. Sueri C, Gasparini S, Balestrini S, Labate A, Gambardella A, Russo E, et al. Diagnostic Biomarkers of Epilepsy. Curr Pharm Biotechnol. 2018;19(6):440-50. Epub 2018/07/14. doi: 10.2174/1389201019666180713095251. PubMed PMID: 30003857.

729. Tanei T, Kajita Y, Maesawa S, Nakatsubo D, Aoki K, Noda H, et al. Long-term Effect and Predictive Factors of Motor Cortex and Spinal Cord Stimulation for Chronic Neuropathic Pain. Neurol Med Chir (Tokyo). 2018;58(10):422-34. Epub 2018/08/31. doi: 10.2176/nmc.oa.2018-0106. PubMed PMID: 30158352; PubMed Central PMCID: PMCPMC6186764.

730. Thibaut A, Chennu S, Chatelle C, Martens G, Annen J, Cassol H, et al. Theta network centrality correlates with tDCS response in disorders of consciousness. Brain Stimul. 2018;11(6):1407-9. Epub 2018/09/23. doi: 10.1016/j.brs.2018.09.002. PubMed PMID: 30241773.

731. Tjen ALSC, Fu LW, Guo ZL, Longhurst JC. Modulation of Neurally Mediated Vasodepression and Bradycardia by Electroacupuncture through Opioids in Nucleus Tractus Solitarius.

Sci Rep. 2018;8(1):1900. Epub 2018/02/01. doi: 10.1038/s41598-018-19672-9. PubMed PMID: 29382866; PubMed Central PMCID: PMCPMC5789879.

732. Tremblay S, Pascual-Leone A, Théoret H. A review of the effects of physical activity and sports concussion on brain function and anatomy. Int J Psychophysiol. 2018;132(Pt A):167-75. Epub 2017/09/13. doi: 10.1016/j.ijpsycho.2017.09.005. PubMed PMID: 28893565.

733. Trofimov AO, Kalentiev G, Karelsky M, Ksenofontova C, Ruzavina A, Yuriev M, et al. Cerebral Hemodynamics After Transcranial Direct Current Stimulation (tDCS) in Patients with Consequences of Traumatic Brain Injury. Adv Exp Med Biol. 2018;1072:59-62. Epub 2018/09/05. doi: 10.1007/978-3-319-91287-5_10. PubMed PMID: 30178324; PubMed Central PMCID: PMCPMC6312702.

734. Vargas-Saturno L, Ayala-Grosso C. Adaptive neurogenesis in the cerebral cortex and contralateral subventricular zone induced by unilateral cortical devascularization: Possible modulation by dopamine neurotransmission. Eur J Neurosci. 2018;48(12):3514-33. Epub 2018/11/08. doi: 10.1111/ejn.14260. PubMed PMID: 30402991.

735. Verma MK, Goel R, Krishnadas N, Nemmani KVS. Targeting

glucose-dependent insulinotropic polypeptide receptor for neurodegenerative disorders. Expert Opin Ther Targets. 2018;22(7):615-28. Epub 2018/06/19. doi: 10.1080/14728222.2018.1487952. PubMed PMID: 29911915.

736. Wu M, Wu Y, Yu Y, Gao J, Meng F, He F, et al. Effects of theta burst stimulation of the left dorsolateral prefrontal cortex in disorders of consciousness. Brain Stimul. 2018;11(6):1382-4. Epub 2018/08/19. doi: 10.1016/j.brs.2018.07.055. PubMed PMID: 30120046.

737. Xia X, Yang Y, Guo Y, Bai Y, Dang Y, Xu R, et al. Current Status of Neuromodulatory Therapies for Disorders of Consciousness. Neurosci Bull. 2018;34(4):615-25. Epub 2018/06/20. doi: 10.1007/s12264-018-0244-4. PubMed PMID: 29916112; PubMed Central PMCID: PMCPMC6060218.

738. Yu K, Seal ML, Reyes J, Godfrey C, Anderson V, Adamson C, et al. Brain volumetric correlates of inhibition and cognitive flexibility 16 years following childhood traumatic brain injury. J Neurosci Res. 2018;96(4):642-51. Epub 2017/07/05. doi: 10.1002/jnr.24087. PubMed PMID: 28675465.

739. Yu KP, Yoon YS, Lee JG, Oh JS, Lee JS, Seog T, et al. Effects of Electric Cortical Stimulation (ECS) and Transcranial Direct Current Stimulation (tDCS) on Rats With a Traumatic Brain Injury.

Ann Rehabil Med. 2018;42(4):502-13. Epub 2018/09/06. doi: 10.5535/arm.2018.42.4.502. PubMed PMID: 30180518; PubMed Central PMCID: PMCPMC6129703.

740. Zhang D, Fu Q, Dionigi G, Wang T, Xin J, Zhang J, et al. Intraoperative Neural Monitoring in Endoscopic Thyroidectomy Via Bilateral Areola Approach. Surg Laparosc Endosc Percutan Tech. 2018;28(5):303-8. Epub 2018/06/12. doi: 10.1097/sle.0000000000000542. PubMed PMID: 29889110.

741. Zhang Y, Song W. Transcranial direct current stimulation in disorders of consciousness: a review. Int J Neurosci. 2018;128(3):255-61. Epub 2017/09/19. doi: 10.1080/00207454.2017.1381094. PubMed PMID: 28918680.

742. Zhang Y, Yang Y, Si J, Xia X, He J, Jiang T. Influence of inter-stimulus interval of spinal cord stimulation in patients with disorders of consciousness: A preliminary functional near-infrared spectroscopy study. Neuroimage Clin. 2018;17:1-9. Epub 2018/04/06. doi: 10.1016/j.nicl.2017.09.017. PubMed PMID: 29619317; PubMed Central PMCID: PMCPMC5883216.

743. Zheng MX, Hua XY, Feng JT, Li T, Lu YC, Shen YD, et al. Trial of Contralateral Seventh Cervical Nerve Transfer for Spastic Arm Paralysis. N Engl J Med. 2018;378(1):22-34. Epub 2017/12/21. doi: 10.1056/NEJMoa1615208. PubMed PMID:

29262271.
744. Allart E, Devanne H, Delval A. Contribution of transcranial magnetic stimulation in assessing parietofrontal connectivity during gesture production in healthy individuals and brain-injured patients. Neurophysiol Clin. 2019;49(2):115-23. Epub 2019/01/03. doi: 10.1016/j.neucli.2018.12.005. PubMed PMID: 30600138.
745. Ansado J, Blunt A, Chen JK, Koski L, Ptito A. Impact of non-invasive brain stimulation on transcallosal modulation in mild traumatic brain injury: a multimodal pilot investigation. Brain Inj. 2019;33(8):1021-31. Epub 2019/06/07. doi: 10.1080/02699052.2019.1605620. PubMed PMID: 31170014.
746. Bensaid S, Modolo J, Merlet I, Wendling F, Benquet P. COALIA: A Computational Model of Human EEG for Consciousness Research. Front Syst Neurosci. 2019;13:59. Epub 2019/12/05. doi: 10.3389/fnsys.2019.00059. PubMed PMID: 31798421; PubMed Central PMCID: PMCPMC6863981.
747. Berger P. A review of physical modalities and the potential to expand the treatment of patients with traumatic brain injury. Acupunct Med. 2019;37(6):365-9. Epub 2019/04/30. doi: 10.1177/0964528419844264. PubMed PMID: 31032621.
748. Berger P. A rehabilitation approach to a patient with traumatic brain injury. Acupunct Med. 2019;37(6):370-1. Epub 2019/11/19.

doi: 10.1177/0964528419884947. PubMed PMID: 31735056.

749. Bourdillon P, Hermann B, Sitt JD, Naccache L. Electromagnetic Brain Stimulation in Patients With Disorders of Consciousness. Front Neurosci. 2019;13:223. Epub 2019/04/03. doi: 10.3389/fnins.2019.00223. PubMed PMID: 30936822; PubMed Central PMCID: PMCPMC6432925.

750. Burchiel KJ, Raslan AM. Contemporary concepts of pain surgery. J Neurosurg. 2019;130(4):1039-49. Epub 2019/04/02. doi: 10.3171/2019.1.Jns181620. PubMed PMID: 30933905.

751. Cai T, Xia X, Zhang H, Guo Y, Bai Y. High-definition transcranial direct current stimulation modulates neural activities in patients with prolonged disorders of consciousness. Brain Stimul. 2019;12(6):1619-21. Epub 2019/09/15. doi: 10.1016/j.brs.2019.08.017. PubMed PMID: 31519492.

752. Cavinato M, Genna C, Formaggio E, Gregorio C, Storti SF, Manganotti P, et al. Behavioural and electrophysiological effects of tDCS to prefrontal cortex in patients with disorders of consciousness. Clin Neurophysiol. 2019;130(2):231-8. Epub 2018/12/24. doi: 10.1016/j.clinph.2018.10.018. PubMed PMID: 30580246.

753. Colombo MA, Napolitani M, Boly M, Gosseries O, Casarotto S, Rosanova M, et al. The spectral exponent of the resting EEG

indexes the presence of consciousness during unresponsiveness induced by propofol, xenon, and ketamine. Neuroimage. 2019;189:631-44. Epub 2019/01/15. doi: 10.1016/j.neuroimage.2019.01.024. PubMed PMID: 30639334.

754. Di Virgilio TG, Ietswaart M, Wilson L, Donaldson DI, Hunter AM. Understanding the Consequences of Repetitive Subconcussive Head Impacts in Sport: Brain Changes and Dampened Motor Control Are Seen After Boxing Practice. Front Hum Neurosci. 2019;13:294. Epub 2019/09/26. doi: 10.3389/fnhum.2019.00294. PubMed PMID: 31551732; PubMed Central PMCID: PMCPMC6746992.

755. Dixon B, Turner R, Christou C. Assessment of a Non-Invasive Brain Oximeter in a Sheep Model of Acute Brain Injury. Med Devices (Auckl). 2019;12:479-87. Epub 2019/12/12. doi: 10.2147/mder.S235804. PubMed PMID: 31824197; PubMed Central PMCID: PMCPMC6900466.

756. Dobney DM, Miller MB, Tufts E. Non-pharmacological rehabilitation interventions for concussion in children: a scoping review. Disabil Rehabil. 2019;41(6):727-39. Epub 2017/11/22. doi: 10.1080/09638288.2017.1400595. PubMed PMID: 29157025.

757. Dunn MS, Beck B, Simpson PM, Cameron PA, Kennedy M, Maiden M, et al. Comparing the outcomes of isolated, serious

traumatic brain injury in older adults managed at major trauma centres and neurosurgical services: A registry-based cohort study. Injury. 2019;50(9):1534-9. Epub 2019/06/18. doi: 10.1016/j.injury.2019.06.012. PubMed PMID: 31204027.

758. Elbanna ST, Elshennawy S, Ayad MN. Noninvasive Brain Stimulation for Rehabilitation of Pediatric Motor Disorders Following Brain Injury: Systematic Review of Randomized Controlled Trials. Arch Phys Med Rehabil. 2019;100(10):1945-63. Epub 2019/05/13. doi: 10.1016/j.apmr.2019.04.009. PubMed PMID: 31078616.

759. Faye A, Jacquin-Courtois S, Reynaud E, Lesourd M, Besnard J, Osiurak F. Numerical cognition: A meta-analysis of neuroimaging, transcranial magnetic stimulation and brain-damaged patients studies. Neuroimage Clin. 2019;24:102053. Epub 2019/12/05. doi: 10.1016/j.nicl.2019.102053. PubMed PMID: 31795045; PubMed Central PMCID: PMCPMC6978218.

760. Feldmann LK, Le Prieult F, Felzen V, Thal SC, Engelhard K, Behl C, et al. Proteasome and Autophagy-Mediated Impairment of Late Long-Term Potentiation (l-LTP) after Traumatic Brain Injury in the Somatosensory Cortex of Mice. Int J Mol Sci. 2019;20(12). Epub 2019/06/27. doi: 10.3390/ijms20123048. PubMed PMID: 31234472; PubMed Central PMCID: PMCPMC6627835.

761. Fridman EA, Osborne JR, Mozley PD, Victor JD, Schiff ND. Presynaptic dopamine deficit in minimally conscious state patients following traumatic brain injury. Brain. 2019;142(7):1887-93. Epub 2019/09/11. doi: 10.1093/brain/awz118. PubMed PMID: 31505542; PubMed Central PMCID: PMCPMC6598636.

762. Guo Y, Bai Y, Xia X, Li J, Wang X, Dai Y, et al. Effects of Long-Lasting High-Definition Transcranial Direct Current Stimulation in Chronic Disorders of Consciousness: A Pilot Study. Front Neurosci. 2019;13:412. Epub 2019/05/23. doi: 10.3389/fnins.2019.00412. PubMed PMID: 31114475; PubMed Central PMCID: PMCPMC6502996.

763. Hameed MQ, Hsieh TH, Morales-Quezada L, Lee HHC, Damar U, MacMullin PC, et al. Ceftriaxone Treatment Preserves Cortical Inhibitory Interneuron Function via Transient Salvage of GLT-1 in a Rat Traumatic Brain Injury Model. Cereb Cortex. 2019;29(11):4506-18. Epub 2018/12/28. doi: 10.1093/cercor/bhy328. PubMed PMID: 30590449; PubMed Central PMCID: PMCPMC7150617.

764. He DP, Zhang J, Bai ZF. Percutaneous Electrical Nerve Stimulation for Chronic Knee Pain: A Randomized, Sham-controlled Trial. Altern Ther Health Med. 2019;25(2):30-4. Epub 2017/11/05. PubMed PMID: 29101777.

765. Hofer AS, Schwab ME. Enhancing rehabilitation and functional recovery after brain and spinal cord trauma with electrical neuromodulation. Curr Opin Neurol. 2019;32(6):828-35. Epub 2019/10/01. doi: 10.1097/wco.0000000000000750. PubMed PMID: 31567546; PubMed Central PMCID: PMCPMC6855343.
766. Houghton DC, Uhde TW, Borckardt JJ, Cortese BM. Exploratory Investigation of a Brief Cognitive Behavioral Intervention and Transcranial Direct Current Stimulation on Odor Sensitivity. Psychosom Med. 2019;81(4):389-95. Epub 2019/02/15. doi: 10.1097/psy.0000000000000679. PubMed PMID: 30762663; PubMed Central PMCID: PMCPMC6961818.
767. Hoy KE, McQueen S, Elliot D, Herring SE, Maller JJ, Fitzgerald PB. A Pilot Investigation of Repetitive Transcranial Magnetic Stimulation for Post-Traumatic Brain Injury Depression: Safety, Tolerability, and Efficacy. J Neurotrauma. 2019;36(13):2092-8. Epub 2019/02/05. doi: 10.1089/neu.2018.6097. PubMed PMID: 30712461.
768. Huang X, Luo Y, Liu Z, Zhang C, Zhong H, Xue J, et al. Influence of Two-Stage Combinations of Constructed Wetlands on the Removal of Antibiotics, Antibiotic Resistance Genes and Nutrients from Goose Wastewater. Int J Environ Res Public Health. 2019;16(20). Epub 2019/10/24. doi: 10.3390/ijerph16204030.

PubMed PMID: 31640268; PubMed Central PMCID: PMCPMC6843979.

769. Hurme M, Koivisto M, Revonsuo A, Railo H. V1 activity during feedforward and early feedback processing is necessary for both conscious and unconscious motion perception. Neuroimage. 2019;185:313-21. Epub 2018/10/27. doi: 10.1016/j.neuroimage.2018.10.058. PubMed PMID: 30366074.

770. Jang SH, Kwon YH. Neuroimaging characterization of recovery of impaired consciousness in patients with disorders of consciousness. Neural Regen Res. 2019;14(7):1202-7. Epub 2019/02/26. doi: 10.4103/1673-5374.251299. PubMed PMID: 30804246; PubMed Central PMCID: PMCPMC6425825.

771. Jang SH, Seo YS. Diagnosis of Conversion Disorder Using Diffusion Tensor Tractography and Transcranial Magnetic Stimulation in a Patient with Mild Traumatic Brain Injury. Diagnostics (Basel). 2019;9(4). Epub 2019/10/28. doi: 10.3390/diagnostics9040155. PubMed PMID: 31652549; PubMed Central PMCID: PMCPMC6963761.

772. Kataria P, Padmanabhan P, Gopalakrishnan A, Padmanaban V, Mahadik S, Ambrósio R, Jr. Accuracy of Scheimpflug-derived corneal biomechanical and tomographic indices for detecting subclinical and mild keratectasia in a South Asian population. J

Cataract Refract Surg. 2019;45(3):328-36. Epub 2018/12/12. doi: 10.1016/j.jcrs.2018.10.030. PubMed PMID: 30527442.

773. Khan F, Amatya B, Bensmail D, Yelnik A. Non-pharmacological interventions for spasticity in adults: An overview of systematic reviews. Ann Phys Rehabil Med. 2019;62(4):265-73. Epub 2017/10/19. doi: 10.1016/j.rehab.2017.10.001. PubMed PMID: 29042299.

774. Khan IS, D'Agostino EN, Calnan DR, Lee JE, Aronson JP. Deep Brain Stimulation for Memory Modulation: A New Frontier. World Neurosurg. 2019;126:638-46. Epub 2019/01/18. doi: 10.1016/j.wneu.2018.12.184. PubMed PMID: 30654156.

775. Kim WS, Lee K, Kim S, Cho S, Paik NJ. Transcranial direct current stimulation for the treatment of motor impairment following traumatic brain injury. J Neuroeng Rehabil. 2019;16(1):14. Epub 2019/01/27. doi: 10.1186/s12984-019-0489-9. PubMed PMID: 30683136; PubMed Central PMCID: PMCPMC6347832.

776. King R, Kirton A, Zewdie E, Seeger TA, Ciechanski P, Barlow KM. Longitudinal Assessment of Cortical Excitability in Children and Adolescents With Mild Traumatic Brain Injury and Persistent Post-concussive Symptoms. Front Neurol. 2019;10:451. Epub 2019/06/04. doi: 10.3389/fneur.2019.00451. PubMed PMID:

31156530; PubMed Central PMCID: PMCPMC6533942.
777. Knoll RM, Ishai R, Trakimas DR, Chen JX, Nadol JB, Jr., Rauch SD, et al. Peripheral Vestibular System Histopathologic Changes following Head Injury without Temporal Bone Fracture. Otolaryngol Head Neck Surg. 2019;160(1):122-30. Epub 2018/10/03. doi: 10.1177/0194599818795695. PubMed PMID: 30274548.
778. Knoll RM, Reinshagen KL, Barber SR, Ghanad I, Swanson R, Smith DH, et al. High Resolution Computed Tomography Atlas of the Porcine Temporal Bone and Skull Base: Anatomical Correlates for Traumatic Brain Injury Research. J Neurotrauma. 2019;36(7):1029-39. Epub 2018/07/05. doi: 10.1089/neu.2018.5808. PubMed PMID: 29969939; PubMed Central PMCID: PMCPMC8349728.
779. Kopachka MM, Sharova EV, Aleksandrova EV, Troshina EM, Zaytsev OS, Kravchuk AD, et al. [In search of an effective algorithm for rhythmic transcranial magnetic stimulation in neurorehabilitation after severe traumatic brain injury]. Zh Vopr Neirokhir Im N N Burdenko. 2019;83(6):111-9. Epub 2020/02/08. doi: 10.17116/neiro201983061111. PubMed PMID: 32031174.
780. Lee M, Baird B, Gosseries O, Nieminen JO, Boly M, Postle BR, et al. Connectivity differences between consciousness and

unconsciousness in non-rapid eye movement sleep: a TMS-EEG study. Sci Rep. 2019;9(1):5175. Epub 2019/03/28. doi: 10.1038/s41598-019-41274-2. PubMed PMID: 30914674; PubMed Central PMCID: PMCPMC6435892.

781. Legostaeva L, Poydasheva A, Iazeva E, Sinitsyn D, Sergeev D, Bakulin I, et al. Stimulation of the Angular Gyrus Improves the Level of Consciousness. Brain Sci. 2019;9(5). Epub 2019/05/09. doi: 10.3390/brainsci9050103. PubMed PMID: 31064138; PubMed Central PMCID: PMCPMC6562708.

782. Li H, Wang Z, He Y, Meng S, Xu Y, Chen S, et al. Rational synthesis of Mn(x)Cd(1-x)S for enhanced photocatalytic H(2) evolution: Effects of S precursors and the feed ratio of Mn/Cd on its structure and performance. J Colloid Interface Sci. 2019;535:469-80. Epub 2018/10/16. doi: 10.1016/j.jcis.2018.10.018. PubMed PMID: 30321782.

783. Li LM, Violante IR, Zimmerman K, Leech R, Hampshire A, Patel M, et al. Traumatic axonal injury influences the cognitive effect of non-invasive brain stimulation. Brain. 2019;142(10):3280-93. Epub 2019/09/11. doi: 10.1093/brain/awz252. PubMed PMID: 31504237; PubMed Central PMCID: PMCPMC6794939.

784. Li S, Dong X, Sun W, Zhao N, Yu G, Shuai L. Effects of

transcranial direct current stimulation on patients with disorders of consciousness after traumatic brain injury: study protocol for a randomized, double-blind controlled trial. Trials. 2019;20(1):596. Epub 2019/10/19. doi: 10.1186/s13063-019-3680-1. PubMed PMID: 31623656; PubMed Central PMCID: PMCPMC6796458.

785. Lim Y, Baek KH, Kim HJ, Lee S, Lee JW, Kang MI. Changes in trabecular bone score and bone mineral density following allogeneic hematopoietic stem cell transplantation. Bone. 2019;124:40-6. Epub 2019/04/09. doi: 10.1016/j.bone.2019.04.004. PubMed PMID: 30959188.

786. Lin Y, Liu T, Huang Q, Su Y, Chen W, Gao D, et al. Electroencephalography and Functional Magnetic Resonance Imaging-Guided Simultaneous Transcranial Direct Current Stimulation and Repetitive Transcranial Magnetic Stimulation in a Patient With Minimally Conscious State. Front Neurosci. 2019;13:746. Epub 2019/08/17. doi: 10.3389/fnins.2019.00746. PubMed PMID: 31417339; PubMed Central PMCID: PMCPMC6685103.

787. Lindsey GL, Yesen A, Christie AD. The Impact of Physical Activity and Sleep on Physiology Following a mTBI. Int J Exerc Sci. 2019;12(3):919-31. Epub 2019/09/17. PubMed PMID: 31523349; PubMed Central PMCID: PMCPMC6719821.

788. Liu H, Li Z, Yao Z, Liu Y, Zhang Q, Sun Y, et al. Designed MnS/Co(9)S(8) micro-flowers composites with serrate edges as high-performance electrodes for asymmetric supercapacitor. J Colloid Interface Sci. 2019;551:119-29. Epub 2019/05/11. doi: 10.1016/j.jcis.2019.05.024. PubMed PMID: 31075626.

789. Liu T, Lu Y, Yu J, Kuang W, Wang X, Jiang Y, et al. Effect of auricular electroacupuncture combined with body acupuncture in improving the consciousness of patients after traumatic brain injury: Study protocol for a randomized controlled trial. Medicine (Baltimore). 2019;98(30):e16587. Epub 2019/07/28. doi: 10.1097/md.0000000000016587. PubMed PMID: 31348298; PubMed Central PMCID: PMCPMC6709251.

790. Locatelli D, Veiceschi P, Castelnuovo P, Tanriover N, Evliyaoglu O, Canaz H, et al. Transsphenoidal surgery for pituitary adenomas in pediatric patients: a multicentric retrospective study. Childs Nerv Syst. 2019;35(11):2119-26. Epub 2019/05/13. doi: 10.1007/s00381-019-04179-z. PubMed PMID: 31079182.

791. Ly LT, Benthall KN, Pale T, McClellan AD. Restoration of the Topological Organization of the Trigeminal System Following Trigeminal Nerve Root Injury in the Lamprey. Neuroscience. 2019;423:216-31. Epub 2019/09/05. doi: 10.1016/j.neuroscience.2019.08.044. PubMed PMID: 31484046.

792. Martens G, Fregni F, Carrière M, Barra A, Laureys S, Thibaut A. Single tDCS session of motor cortex in patients with disorders of consciousness: a pilot study. Brain Inj. 2019;33(13-14):1679-83. Epub 2019/09/17. doi: 10.1080/02699052.2019.1667537. PubMed PMID: 31523995.

793. Martens KM, Pechacek KM, Modrak CG, Milleson VJ, Zhu B, Vonder Haar C. Cathodal Transcranial Direct-Current Stimulation Selectively Decreases Impulsivity after Traumatic Brain Injury in Rats. J Neurotrauma. 2019;36(19):2827-30. Epub 2019/05/11. doi: 10.1089/neu.2019.6470. PubMed PMID: 31072218; PubMed Central PMCID: PMCPMC6744944.

794. Mattavelli G, Pisoni A, Romero Lauro LJ, Marino BF, Bonomi M, Rosanova M, et al. TMS-EEG approach unveils brain mechanisms underlying conscious and unconscious face perception. Brain Stimul. 2019;12(4):1010-9. Epub 2019/03/11. doi: 10.1016/j.brs.2019.02.022. PubMed PMID: 30852122.

795. Mo JJ, Hu WH, Zhang C, Wang X, Liu C, Zhao BT, et al. Motor cortex stimulation: a systematic literature-based analysis of effectiveness and case series experience. BMC Neurol. 2019;19(1):48. Epub 2019/03/31. doi: 10.1186/s12883-019-1273-y. PubMed PMID: 30925914; PubMed Central PMCID: PMCPMC6440080.

796. Moretti A, de Sire A, Curci C, Toro G, Gimigliano F, Iolascon G. Effectiveness of denosumab on back pain-related disability and quality-of-life in patients with vertebral fragility fractures. Curr Med Res Opin. 2019;35(1):151-5. Epub 2018/11/09. doi: 10.1080/03007995.2018.1545636. PubMed PMID: 30406685.
797. Moussavi Z, Suleiman A, Rutherford G, Ranjbar Pouya O, Dastgheib Z, Zhang W, et al. A Pilot Randomised Double-Blind Study of the Tolerability and efficacy of repetitive Transcranial Magnetic Stimulation on Persistent Post-Concussion Syndrome. Sci Rep. 2019;9(1):5498. Epub 2019/04/04. doi: 10.1038/s41598-019-41923-6. PubMed PMID: 30940870; PubMed Central PMCID: PMCPMC6445141.
798. Nagel SJ, Helland L, Woodroffe RW, Frizon LA, Holland MT, Machado AG, et al. Durotomy Surrogate and Seals for Intradural Spinal Cord Stimulators: Apparatus and Review of Clinical Methods and Materials. Neuromodulation. 2019;22(8):916-29. Epub 2019/01/12. doi: 10.1111/ner.12913. PubMed PMID: 30632655.
799. Namgung E, Kim M, Yoon S. Repetitive transcranial magnetic stimulation in trauma-related conditions. Neuropsychiatr Dis Treat. 2019;15:701-12. Epub 2019/04/03. doi: 10.2147/ndt.S189498. PubMed PMID: 30936700; PubMed Central PMCID:

PMCPMC6430993.

800. Narayana S, Charles C, Collins K, Tsao JW, Stanfill AG, Baughman B. Neuroimaging and Neuropsychological Studies in Sports-Related Concussions in Adolescents: Current State and Future Directions. Front Neurol. 2019;10:538. Epub 2019/06/11. doi: 10.3389/fneur.2019.00538. PubMed PMID: 31178818; PubMed Central PMCID: PMCPMC6542940.

801. Nemanich ST, Chen CY, Chen M, Zorn E, Mueller B, Peyton C, et al. Safety and Feasibility of Transcranial Magnetic Stimulation as an Exploratory Assessment of Corticospinal Connectivity in Infants After Perinatal Brain Injury: An Observational Study. Phys Ther. 2019;99(6):689-700. Epub 2019/02/27. doi: 10.1093/ptj/pzz028. PubMed PMID: 30806664; PubMed Central PMCID: PMCPMC6545276.

802. Neville IS, Zaninotto AL, Hayashi CY, Rodrigues PA, Galhardoni R, Ciampi de Andrade D, et al. Repetitive TMS does not improve cognition in patients with TBI: A randomized double-blind trial. Neurology. 2019;93(2):e190-e9. Epub 2019/06/09. doi: 10.1212/wnl.0000000000007748. PubMed PMID: 31175209; PubMed Central PMCID: PMCPMC6656650.

803. Nicolini C, Toepp S, Harasym D, Michalski B, Fahnestock M, Gibala MJ, et al. No changes in corticospinal excitability,

biochemical markers, and working memory after six weeks of high-intensity interval training in sedentary males. Physiol Rep. 2019;7(11):e14140. Epub 2019/06/09. doi: 10.14814/phy2.14140. PubMed PMID: 31175708; PubMed Central PMCID: PMCPMC6555846.

804. Noller CM, Levine YA, Urakov TM, Aronson JP, Nash MS. Vagus Nerve Stimulation in Rodent Models: An Overview of Technical Considerations. Front Neurosci. 2019;13:911. Epub 2019/09/26. doi: 10.3389/fnins.2019.00911. PubMed PMID: 31551679; PubMed Central PMCID: PMCPMC6738225.

805. Opie GM, Foo N, Killington M, Ridding MC, Semmler JG. Transcranial Magnetic Stimulation-Electroencephalography Measures of Cortical Neuroplasticity Are Altered after Mild Traumatic Brain Injury. J Neurotrauma. 2019;36(19):2774-84. Epub 2019/03/09. doi: 10.1089/neu.2018.6353. PubMed PMID: 30848163.

806. Padua L, Cuccagna C, Pazzaglia C. Novel sensory paradigms for neuromodulation in disorders of consciousness in traumatic brain injury. Curr Opin Neurol. 2019;32(6):844-9. Epub 2019/10/01. doi: 10.1097/wco.0000000000000747. PubMed PMID: 31567499.

807. Pearce AJ, Tommerdahl M, King DA. Neurophysiological

abnormalities in individuals with persistent post-concussion symptoms. Neuroscience. 2019;408:272-81. Epub 2019/04/21. doi: 10.1016/j.neuroscience.2019.04.019. PubMed PMID: 31004695.
808. Pellegrini M, Zoghi M, Jaberzadeh S. Case Report of a Syncope Episode in a Healthy Male Adult Participant During Single-Pulse Transcranial Magnetic Stimulation. J ect. 2019;35(2):e11-e2. Epub 2018/08/17. doi: 10.1097/yct.0000000000000526. PubMed PMID: 30113987.
809. Rao V, Bechtold K, McCann U, Roy D, Peters M, Vaishnavi S, et al. Low-Frequency Right Repetitive Transcranial Magnetic Stimulation for the Treatment of Depression After Traumatic Brain Injury: A Randomized Sham-Controlled Pilot Study. J Neuropsychiatry Clin Neurosci. 2019;31(4):306-18. Epub 2019/04/26. doi: 10.1176/appi.neuropsych.17110338. PubMed PMID: 31018810.
810. Reilmann R, McGarry A, Grachev ID, Savola JM, Borowsky B, Eyal E, et al. Safety and efficacy of pridopidine in patients with Huntington's disease (PRIDE-HD): a phase 2, randomised, placebo-controlled, multicentre, dose-ranging study. Lancet Neurol. 2019;18(2):165-76. Epub 2018/12/20. doi: 10.1016/s1474-4422(18)30391-0. PubMed PMID: 30563778.
811. Ruiz de Miras J, Soler F, Iglesias-Parro S, Ibáñez-Molina AJ,

Casali AG, Laureys S, et al. Fractal dimension analysis of states of consciousness and unconsciousness using transcranial magnetic stimulation. Comput Methods Programs Biomed. 2019;175:129-37. Epub 2019/05/21. doi: 10.1016/j.cmpb.2019.04.017. PubMed PMID: 31104702.

812. Saleem GT, Ewen JB, Crasta JE, Slomine BS, Cantarero GL, Suskauer SJ. Single-arm, open-label, dose escalation phase I study to evaluate the safety and feasibility of transcranial direct current stimulation with electroencephalography biomarkers in paediatric disorders of consciousness: a study protocol. BMJ Open. 2019;9(8):e029967. Epub 2019/08/12. doi: 10.1136/bmjopen-2019-029967. PubMed PMID: 31401607; PubMed Central PMCID: PMCPMC6701812.

813. Sdoia S, Conversi D, Pecchinenda A, Ferlazzo F. Access to consciousness of briefly presented visual events is modulated by transcranial direct current stimulation of left dorsolateral prefrontal cortex. Sci Rep. 2019;9(1):10950. Epub 2019/07/31. doi: 10.1038/s41598-019-47527-4. PubMed PMID: 31358875; PubMed Central PMCID: PMCPMC6662720.

814. Sekar S, Zhang Y, Miranzadeh Mahabadi H, Parvizi A, Taghibiglou C. Low-Field Magnetic Stimulation Restores Cognitive and Motor Functions in the Mouse Model of Repeated

Traumatic Brain Injury: Role of Cellular Prion Protein. J Neurotrauma. 2019;36(22):3103-14. Epub 2019/04/26. doi: 10.1089/neu.2018.5918. PubMed PMID: 31020907.

815. Sha R, Zhang B, Han X, Peng J, Zheng C, Zhang F, et al. Electroacupuncture Alleviates Ischemic Brain Injury by Inhibiting the miR-223/NLRP3 Pathway. Med Sci Monit. 2019;25:4723-33. Epub 2019/06/27. doi: 10.12659/msm.917213. PubMed PMID: 31237865; PubMed Central PMCID: PMCPMC6607941.

816. Siddiqi SH, Trapp NT, Hacker CD, Laumann TO, Kandala S, Hong X, et al. Repetitive Transcranial Magnetic Stimulation with Resting-State Network Targeting for Treatment-Resistant Depression in Traumatic Brain Injury: A Randomized, Controlled, Double-Blinded Pilot Study. J Neurotrauma. 2019;36(8):1361-74. Epub 2018/11/02. doi: 10.1089/neu.2018.5889. PubMed PMID: 30381997; PubMed Central PMCID: PMCPMC6909726.

817. Siddiqi SH, Trapp NT, Shahim P, Hacker CD, Laumann TO, Kandala S, et al. Individualized Connectome-Targeted Transcranial Magnetic Stimulation for Neuropsychiatric Sequelae of Repetitive Traumatic Brain Injury in a Retired NFL Player. J Neuropsychiatry Clin Neurosci. 2019;31(3):254-63. Epub 2019/04/05. doi: 10.1176/appi.neuropsych.18100230. PubMed PMID: 30945588.

818. Sohn E. Decoding the neuroscience of consciousness. Nature.

2019;571(7766):S2-s5. Epub 2019/07/26. doi: 10.1038/d41586-019-02207-1. PubMed PMID: 31341312.
819. Straudi S, Bonsangue V, Mele S, Craighero L, Montis A, Fregni F, et al. Bilateral M1 anodal transcranial direct current stimulation in post traumatic chronic minimally conscious state: a pilot EEG-tDCS study. Brain Inj. 2019;33(4):490-5. Epub 2019/01/12. doi: 10.1080/02699052.2019.1565894. PubMed PMID: 30632807.
820. Tang-Schomer MD, Kaplan DL, Whalen MJ. Film interface for drug testing for delivery to cells in culture and in the brain. Acta Biomater. 2019;94:306-19. Epub 2019/03/06. doi: 10.1016/j.actbio.2019.02.052. PubMed PMID: 30836199; PubMed Central PMCID: PMCPMC6642835.
821. Thibaut A, Piarulli A, Martens G, Chatelle C, Laureys S. Effect of multichannel transcranial direct current stimulation to reduce hypertonia in individuals with prolonged disorders of consciousness: A randomized controlled pilot study. Ann Phys Rehabil Med. 2019;62(6):418-25. Epub 2019/07/10. doi: 10.1016/j.rehab.2019.05.009. PubMed PMID: 31283989.
822. Thibaut A, Schiff N, Giacino J, Laureys S, Gosseries O. Therapeutic interventions in patients with prolonged disorders of consciousness. Lancet Neurol. 2019;18(6):600-14. Epub

2019/04/21. doi: 10.1016/s1474-4422(19)30031-6. PubMed PMID: 31003899.
823. Trakimas DR, Knoll RM, Ishai R, Lee DJ, Jung DH, Nadol JB, Jr., et al. Otopathology of Unilateral Cochlear Implantation in Patients With Bilateral Temporal Bone Fracture. Otol Neurotol. 2019;40(1):e14-e9. Epub 2018/12/12. doi: 10.1097/mao.0000000000002057. PubMed PMID: 30531637; PubMed Central PMCID: PMCPMC6939457.
824. Tyler M, Skinner K, Prabhakaran V, Kaczmarek K, Danilov Y. Translingual Neurostimulation for the Treatment of Chronic Symptoms Due to Mild-to-Moderate Traumatic Brain Injury. Arch Rehabil Res Clin Transl. 2019;1(3-4):100026. Epub 2019/09/27. doi: 10.1016/j.arrct.2019.100026. PubMed PMID: 33543056; PubMed Central PMCID: PMCPMC7853385.
825. Verley DR, Torolira D, Hessell BA, Sutton RL, Harris NG. Cortical Neuromodulation of Remote Regions after Experimental Traumatic Brain Injury Normalizes Forelimb Function but is Temporally Dependent. J Neurotrauma. 2019;36(5):789-801. Epub 2018/07/18. doi: 10.1089/neu.2018.5769. PubMed PMID: 30014759; PubMed Central PMCID: PMCPMC6387565.
826. Walker LAS, Lindsay-Brown AP, Berard JA. Cognitive Fatigability Interventions in Neurological Conditions: A Systematic

Review. Neurol Ther. 2019;8(2):251-71. Epub 2019/10/06. doi: 10.1007/s40120-019-00158-3. PubMed PMID: 31586303; PubMed Central PMCID: PMCPMC6858900.

827. Wang L, Zhang Y, Wang X, Ye Z. Electroacupuncture-induced cannabinoid receptor expression in repair of abducens nerve. Int J Neurosci. 2019;129(9):923-9. Epub 2019/03/20. doi: 10.1080/00207454.2019.1593980. PubMed PMID: 30889365.

828. Wang P, Zhang J, Yu J, Smith C, Feng W. Brain Modulatory Effects by Low-Intensity Transcranial Ultrasound Stimulation (TUS): A Systematic Review on Both Animal and Human Studies. Front Neurosci. 2019;13:696. Epub 2019/08/10. doi: 10.3389/fnins.2019.00696. PubMed PMID: 31396029; PubMed Central PMCID: PMCPMC6667677.

829. Wu M, Yu Y, Luo L, Wu Y, Gao J, Ye X, et al. Efficiency of Repetitive Transcranial Direct Current Stimulation of the Dorsolateral Prefrontal Cortex in Disorders of Consciousness: A Randomized Sham-Controlled Study. Neural Plast. 2019;2019:7089543. Epub 2019/07/17. doi: 10.1155/2019/7089543. PubMed PMID: 31308848; PubMed Central PMCID: PMCPMC6594311.

830. Wu X, Sun NN, Lü MH, Su SH, Wang DH, Zhang SJ, et al. [Effect of different courses of electroacupuncture intervention on

recognition memory and proliferation and differentiation of hippocampal neural stem cells in mice with radiation-induced brain injury]. Zhen Ci Yan Jiu. 2019;44(11):787-92. Epub 2019/11/30. doi: 10.13702/j.1000-0607.190017. PubMed PMID: 31777226.

831. Xia X, Wang Y, Li C, Li X, He J, Bai Y. Transcranial magnetic stimulation-evoked connectivity reveals modulation effects of repetitive transcranial magnetic stimulation on patients with disorders of consciousness. Neuroreport. 2019;30(18):1307-15. Epub 2019/11/13. doi: 10.1097/wnr.0000000000001362. PubMed PMID: 31714484.

832. Yang J, Shang B, Shi H, Zhu S, Lu G, Dai F. The role of toll-like receptor 4 and mast cell in the ameliorating effect of electroacupuncture on visceral hypersensitivity in rats. Neurogastroenterol Motil. 2019;31(6):e13583. Epub 2019/03/28. doi: 10.1111/nmo.13583. PubMed PMID: 30916854.

833. Yang Z, Zhang Y, Sun S. Deciphering the SUMO code in the kidney. J Cell Mol Med. 2019;23(2):711-9. Epub 2018/12/07. doi: 10.1111/jcmm.14021. PubMed PMID: 30506859; PubMed Central PMCID: PMCPMC6349152.

834. Yong Y, Guo J, Zheng D, Li Y, Chen W, Wang J, et al. Electroacupuncture pretreatment attenuates brain injury in a mouse model of cardiac arrest and cardiopulmonary resuscitation via the

AKT/eNOS pathway. Life Sci. 2019;235:116821. Epub 2019/09/03. doi: 10.1016/j.lfs.2019.116821. PubMed PMID: 31476306.
835. Zaninotto AL, El-Hagrassy MM, Green JR, Babo M, Paglioni VM, Benute GG, et al. Transcranial direct current stimulation (tDCS) effects on traumatic brain injury (TBI) recovery: A systematic review. Dement Neuropsychol. 2019;13(2):172-9. Epub 2019/07/10. doi: 10.1590/1980-57642018dn13-020005. PubMed PMID: 31285791; PubMed Central PMCID: PMCPMC6601308.
836. Ziliotto N, Marchetti G, Straudi S, Tisato V, Lavezzi S, Manfredini F, et al. Soluble neural cell adhesion molecule and behavioural recovery in minimally conscious patients undergoing transcranial direct current stimulation. Clin Chim Acta. 2019;495:374-6. Epub 2019/05/12. doi: 10.1016/j.cca.2019.05.008. PubMed PMID: 31077650.
837. Anderson J, Parr NJ, Vela K. VA Evidence-based Synthesis Program Reports. Evidence Brief: Transcranial Magnetic Stimulation (TMS) for Chronic Pain, PTSD, TBI, Opioid Addiction, and Sexual Trauma. Washington (DC): Department of Veterans Affairs (US); 2020.
838. Assecondi S, Hu R, Eskes G, Read M, Griffiths C, Shapiro K. BRAINSTORMING: A study protocol for a randomised double-blind clinical trial to assess the impact of concurrent brain

stimulation (tDCS) and working memory training on cognitive performance in Acquired Brain Injury (ABI). BMC Psychol. 2020;8(1):125. Epub 2020/11/28. doi: 10.1186/s40359-020-00454-w. PubMed PMID: 33243286; PubMed Central PMCID: PMCPMC7694939.

839. Beedham W, Belli A, Ingaralingam S, Haque S, Upthegrove R. The management of depression following traumatic brain injury: A systematic review with meta-analysis. Brain Inj. 2020;34(10):1287-304. Epub 2020/08/19. doi: 10.1080/02699052.2020.1797169. PubMed PMID: 32809854.

840. Begemann MJ, Brand BA, Ćurčić-Blake B, Aleman A, Sommer IE. Efficacy of non-invasive brain stimulation on cognitive functioning in brain disorders: a meta-analysis. Psychol Med. 2020;50(15):2465-86. Epub 2020/10/20. doi: 10.1017/s0033291720003670. PubMed PMID: 33070785; PubMed Central PMCID: PMCPMC7737055.

841. Bender Pape TL, Herrold AA, Guernon A, Aaronson A, Rosenow JM. Neuromodulatory Interventions for Traumatic Brain Injury. J Head Trauma Rehabil. 2020;35(6):365-70. Epub 2020/11/10. doi: 10.1097/htr.0000000000000643. PubMed PMID: 33165150.

842. Bragina OA, Semyachkina-Glushkovskaya OV, Nemoto EM,

Bragin DE. Anodal Transcranial Direct Current Stimulation Improves Impaired Cerebrovascular Reactivity in Traumatized Mouse Brain. Adv Exp Med Biol. 2020;1232:47-53. Epub 2020/01/02. doi: 10.1007/978-3-030-34461-0_7. PubMed PMID: 31893393; PubMed Central PMCID: PMCPMC7307636.

843. Briand MM, Gosseries O, Staumont B, Laureys S, Thibaut A. Transcutaneous Auricular Vagal Nerve Stimulation and Disorders of Consciousness: A Hypothesis for Mechanisms of Action. Front Neurol. 2020;11:933. Epub 2020/09/29. doi: 10.3389/fneur.2020.00933. PubMed PMID: 32982941; PubMed Central PMCID: PMCPMC7477388.

844. Buhagiar F, Fitzgerald M, Bell J, Allanson F, Pestell C. Neuromodulation for Mild Traumatic Brain Injury Rehabilitation: A Systematic Review. Front Hum Neurosci. 2020;14:598208. Epub 2020/12/29. doi: 10.3389/fnhum.2020.598208. PubMed PMID: 33362494; PubMed Central PMCID: PMCPMC7759622.

845. Burhan AM, Patience JA, Teselink JGP, Marlatt NM, Babapoor-Farrokhran S, Palaniyappan L. Bilateral sequential theta burst stimulation for multiple-therapy-resistant depression: A naturalistic observation study. J Psychiatr Res. 2020;130:342-6. Epub 2020/09/04. doi: 10.1016/j.jpsychires.2020.08.009. PubMed PMID: 32882575.

846. Cantarero G, Choynowski J, St Pierre M, Anaya M, Statton M, Stokes W, et al. Repeated Concussions Impair Behavioral and Neurophysiological Changes in the Motor Learning System. Neurorehabil Neural Repair. 2020;34(9):804-13. Epub 2020/07/30. doi: 10.1177/1545968320943578. PubMed PMID: 32723160; PubMed Central PMCID: PMCPMC7501144.
847. Carrière M, Mortaheb S, Raimondo F, Annen J, Barra A, Binda Fossati MC, et al. Neurophysiological Correlates of a Single Session of Prefrontal tDCS in Patients with Prolonged Disorders of Consciousness: A Pilot Double-Blind Randomized Controlled Study. Brain Sci. 2020;10(7). Epub 2020/07/28. doi: 10.3390/brainsci10070469. PubMed PMID: 32708119; PubMed Central PMCID: PMCPMC7408434.
848. Cotovio G, Talmasov D, Barahona-Corrêa JB, Hsu J, Senova S, Ribeiro R, et al. Mapping mania symptoms based on focal brain damage. J Clin Invest. 2020;130(10):5209-22. Epub 2020/08/25. doi: 10.1172/jci136096. PubMed PMID: 32831292; PubMed Central PMCID: PMCPMC7524493.
849. D'Arcy RCN, Greene T, Greene D, Frehlick Z, Fickling SD, Campbell N, et al. Portable neuromodulation induces neuroplasticity to re-activate motor function recovery from brain injury: a high-density MEG case study. J Neuroeng Rehabil.

2020;17(1):158. Epub 2020/12/03. doi: 10.1186/s12984-020-00772-5. PubMed PMID: 33261623; PubMed Central PMCID: PMCPMC7708191.

850. de Tommaso M, Betti V, Bocci T, Bolognini N, Di Russo F, Fattapposta F, et al. Pearl and pitfalls in brain functional analysis by event-related potentials: a narrative review by the Italian Psychophysiology and Cognitive Neuroscience Society on methodological limits and clinical reliability-part II. Neurol Sci. 2020;41(12):3503-15. Epub 2020/07/20. doi: 10.1007/s10072-020-04527-x. PubMed PMID: 32683566.

851. Delaney SL, Gendreau JL, D'Souza M, Feng AY, Ho AL. Optogenetic Modulation for the Treatment of Traumatic Brain Injury. Stem Cells Dev. 2020;29(4):187-97. Epub 2019/09/29. doi: 10.1089/scd.2019.0187. PubMed PMID: 31559914.

852. Ding Y, Li H, Hong T, Yao P. Efficacy of Pulsed Radiofrequency to Cervical Nerve Root for Postherpetic Neuralgia in Upper Extremity. Front Neurosci. 2020;14:377. Epub 2020/05/07. doi: 10.3389/fnins.2020.00377. PubMed PMID: 32372914; PubMed Central PMCID: PMCPMC7186594.

853. Dixon B, MacLeod DB. Assessment of a Non Invasive Brain Oximeter in Volunteers Undergoing Acute Hypoxia. Med Devices (Auckl). 2020;13:183-94. Epub 2020/07/17. doi:

10.2147/mder.S250102. PubMed PMID: 32669881; PubMed Central PMCID: PMCPMC7335769.

854. Dombovy-Johnson ML, Hagedorn JM, Wilson RE, Canzanello NC, Pingree MJ, Watson JC. Spinal Cord Stimulation for Neuropathic Pain Treatment in Brachial Plexus Avulsions: A Literature Review and Report of Two Cases. Neuromodulation. 2020;23(5):704-12. Epub 2020/02/19. doi: 10.1111/ner.13128. PubMed PMID: 32069374.

855. Edlow BL, Barra ME, Zhou DW, Foulkes AS, Snider SB, Threlkeld ZD, et al. Personalized Connectome Mapping to Guide Targeted Therapy and Promote Recovery of Consciousness in the Intensive Care Unit. Neurocrit Care. 2020;33(2):364-75. Epub 2020/08/15. doi: 10.1007/s12028-020-01062-7. PubMed PMID: 32794142; PubMed Central PMCID: PMCPMC8336723.

856. Elias GJB, De Vloo P, Germann J, Boutet A, Gramer RM, Joel SE, et al. Mapping the network underpinnings of central poststroke pain and analgesic neuromodulation. Pain. 2020;161(12):2805-19. Epub 2020/07/23. doi: 10.1097/j.pain.0000000000001998. PubMed PMID: 32694384.

857. Feng Y, Zhang J, Zhou Y, Bai Z, Yin Y. Noninvasive brain stimulation for patients with a disorder of consciousness: a systematic review and meta-analysis. Rev Neurosci. 2020. Epub

2020/08/28. doi: 10.1515/revneuro-2020-0033. PubMed PMID: 32845870.

858. Fickling SD, Greene T, Greene D, Frehlick Z, Campbell N, Etheridge T, et al. Brain Vital Signs Detect Cognitive Improvements During Combined Physical Therapy and Neuromodulation in Rehabilitation From Severe Traumatic Brain Injury: A Case Report. Front Hum Neurosci. 2020;14:347. Epub 2020/11/03. doi: 10.3389/fnhum.2020.00347. PubMed PMID: 33132868; PubMed Central PMCID: PMCPMC7513585.

859. Fisher BE. Beyond Limits: Unmasking Potential Through Movement Discovery. Phys Ther. 2020;100(5):747-56. Epub 2020/04/28. doi: 10.1093/ptj/pzaa018. PubMed PMID: 32339243.

860. Gosseries O, Fecchio M, Wolff A, Sanz LRD, Sombrun C, Vanhaudenhuyse A, et al. Behavioural and brain responses in cognitive trance: A TMS-EEG case study. Clin Neurophysiol. 2020;131(2):586-8. Epub 2019/12/18. doi: 10.1016/j.clinph.2019.11.011. PubMed PMID: 31843502.

861. Gu T, Wang RH, Wu T, Wang D, Yang Q, Yang Q, et al. [Effect of electroacupuncture on neuronal apoptosis in rats with traumatic brain injury based on PI3K/Akt signaling pathway]. Zhongguo Zhen Jiu. 2020;40(8):851-6. Epub 2020/09/02. doi: 10.13703/j.0255-2930.20191217-k0004. PubMed PMID:

32869594.

862. Gu T, Wang X, Yang H, She XN, Chen KH, Wu T, et al. [Impacts of electroacupuncture on neurological function and protein expressions of apoptosis-related Cyt-C and Caspase-9 in rats with traumatic brain injury]. Zhongguo Zhen Jiu. 2020;40(7):749-55. Epub 2020/07/11. doi: 10.13703/j.0255-2930.20190521-0001. PubMed PMID: 32648400.

863. Gu T, Wu T, Wang RH, Yang H, Ke ZH, Wang D, et al. [Effect of electroacupuncture on expression of corticocerebral autophagy-related proteins in rats with traumatic brain injury]. Zhen Ci Yan Jiu. 2020;45(7):524-8. Epub 2020/07/25. doi: 10.13702/j.1000-0607.190824. PubMed PMID: 32705824.

864. Hakon J, Moghiseh M, Poulsen I, Øland CML, Hansen CP, Sabers A. Transcutaneous Vagus Nerve Stimulation in Patients With Severe Traumatic Brain Injury: A Feasibility Trial. Neuromodulation. 2020;23(6):859-64. Epub 2020/04/01. doi: 10.1111/ner.13148. PubMed PMID: 32227429.

865. He R, Fan J, Wang H, Zhong Y, Ma J. Differentiating Responders and Non-responders to rTMS Treatment for Disorder of Consciousness Using EEG After-Effects. Front Neurol. 2020;11:583268. Epub 2020/12/18. doi: 10.3389/fneur.2020.583268. PubMed PMID: 33329325; PubMed

Central PMCID: PMCPMC7714935.

866. Hendriks T, Kunst HPM, Huppelschoten M, Doorduin J, Ter Laan M. TcMEP threshold change is superior to A-train detection when predicting facial nerve outcome in CPA tumour surgery. Acta Neurochir (Wien). 2020;162(5):1197-203. Epub 2020/03/09. doi: 10.1007/s00701-020-04275-z. PubMed PMID: 32146526; PubMed Central PMCID: PMCPMC7156349.

867. Hermann B, Raimondo F, Hirsch L, Huang Y, Denis-Valente M, Pérez P, et al. Combined behavioral and electrophysiological evidence for a direct cortical effect of prefrontal tDCS on disorders of consciousness. Sci Rep. 2020;10(1):4323. Epub 2020/03/11. doi: 10.1038/s41598-020-61180-2. PubMed PMID: 32152347; PubMed Central PMCID: PMCPMC7062738.

868. Herrold AA, Siddiqi SH, Livengood SL, Bender Pape TL, Higgins JP, Adamson MM, et al. Customizing TMS Applications in Traumatic Brain Injury Using Neuroimaging. J Head Trauma Rehabil. 2020;35(6):401-11. Epub 2020/11/10. doi: 10.1097/htr.0000000000000627. PubMed PMID: 33165153.

869. Hitti FL, Piazza M, Sinha S, Kvint S, Hudgins E, Baltuch G, et al. Surgical Outcomes in Post-Traumatic Epilepsy: A Single Institutional Experience. Oper Neurosurg (Hagerstown). 2020;18(1):12-8. Epub 2019/03/30. doi: 10.1093/ons/opz043.

PubMed PMID: 30924499; PubMed Central PMCID: PMCPMC6911733.
870. Howard L, Schwedt TJ. Posttraumatic headache: recent progress. Curr Opin Neurol. 2020;33(3):316-22. Epub 2020/04/19. doi: 10.1097/wco.0000000000000815. PubMed PMID: 32304441.
871. Hurtado-Puerto AM, Nestor K, Eldaief M, Camprodon JA. Safety Considerations for Cerebellar Theta Burst Stimulation. Clin Ther. 2020;42(7):1169-90.e1. Epub 2020/07/18. doi: 10.1016/j.clinthera.2020.06.001. PubMed PMID: 32674957.
872. Jang SH, Kwon HG. Relationship between depression and dorsolateral prefronto-thalamic tract injury in patients with mild traumatic brain injury. Sci Rep. 2020;10(1):19728. Epub 2020/11/14. doi: 10.1038/s41598-020-76889-3. PubMed PMID: 33184443; PubMed Central PMCID: PMCPMC7661494.
873. Jang SH, Kwon YH. Effect of repetitive transcranial magnetic stimulation on the ascending reticular activating system in a patient with disorder of consciousness: a case report. BMC Neurol. 2020;20(1):37. Epub 2020/01/31. doi: 10.1186/s12883-020-1607-9. PubMed PMID: 31996169; PubMed Central PMCID: PMCPMC6988329.
874. Jang SH, Seo YS. Recovery of Injured Optic Radiations in a Patient with Hypoxic-Ischaemic Brain Injury. Neuroophthalmology.

2020;44(4):270-3. Epub 2020/10/06. doi: 10.1080/01658107.2019.1676263. PubMed PMID: 33012915; PubMed Central PMCID: PMCPMC7518302.

875. Jang SH, Seo YS, Lee SJ. Increased thalamocortical connectivity to the medial prefrontal cortex with recovery of impaired consciousness in a stroke patient: A case report. Medicine (Baltimore). 2020;99(18):e19937. Epub 2020/05/03. doi: 10.1097/md.0000000000019937. PubMed PMID: 32358365; PubMed Central PMCID: PMCPMC7440307.

876. Kaga T, Kato H, Hatano Y, Kawaguchi M, Furui T, Morishige KI, et al. Can MRI features differentiate ovarian mucinous carcinoma from mucinous borderline tumor? Eur J Radiol. 2020;132:109281. Epub 2020/09/23. doi: 10.1016/j.ejrad.2020.109281. PubMed PMID: 32961452.

877. Kang DH, Kim GW. Changes in Diffuse Tensor Imaging and Therapeutic Effect of Repetitive Transcranial Magnetic Stimulation in Traumatic Brain Injury with Central Pain. Brain Sci. 2020;10(12). Epub 2020/12/06. doi: 10.3390/brainsci10120929. PubMed PMID: 33276440; PubMed Central PMCID: PMCPMC7759834.

878. Kletzel SL, Aaronson AL, Guernon A, Carbone C, Chaudhry N, Walsh E, et al. Safety Considerations for the Use of Transcranial

Magnetic Stimulation as Treatment for Coma Recovery in People With Severe Traumatic Brain Injury. J Head Trauma Rehabil. 2020;35(6):430-8. Epub 2020/11/10. doi: 10.1097/htr.0000000000000636. PubMed PMID: 33165155; PubMed Central PMCID: PMCPMC8908773.

879. Knoll RM, Ishai R, Lubner RJ, Trakimas DR, Brodsky JR, Jung DH, et al. Peripheral Vestibular Organ Degeneration After Temporal Bone Fracture: A Human Otopathology Study. Laryngoscope. 2020;130(3):752-60. Epub 2019/05/11. doi: 10.1002/lary.28010. PubMed PMID: 31074866.

880. Kondziella D, Bender A, Diserens K, van Erp W, Estraneo A, Formisano R, et al. European Academy of Neurology guideline on the diagnosis of coma and other disorders of consciousness. Eur J Neurol. 2020;27(5):741-56. Epub 2020/02/25. doi: 10.1111/ene.14151. PubMed PMID: 32090418.

881. Lang S, Yan X, Wang C, Wu Y, Liu X, Ma C, et al. The poly-ADP ribose polymerase-1/apoptosis-inducing factor pathway may help mediate the protective effect of electroacupuncture on early brain injury after subarachnoid hemorrhage. Neuroreport. 2020;31(8):605-12. Epub 2020/04/18. doi: 10.1097/wnr.0000000000001445. PubMed PMID: 32301816.

882. Latchoumane CV, Barany DA, Karumbaiah L, Singh T.

Neurostimulation and Reach-to-Grasp Function Recovery Following Acquired Brain Injury: Insight From Pre-clinical Rodent Models and Human Applications. Front Neurol. 2020;11:835. Epub 2020/08/28. doi: 10.3389/fneur.2020.00835. PubMed PMID: 32849253; PubMed Central PMCID: PMCPMC7396659.

883. Leung A, Shirvalkar P, Chen R, Kuluva J, Vaninetti M, Bermudes R, et al. Transcranial Magnetic Stimulation for Pain, Headache, and Comorbid Depression: INS-NANS Expert Consensus Panel Review and Recommendation. Neuromodulation. 2020;23(3):267-90. Epub 2020/03/27. doi: 10.1111/ner.13094. PubMed PMID: 32212288.

884. Levy-Lamdan O, Zifman N, Sasson E, Efrati S, Hack DC, Tanne D, et al. Evaluation of White Matter Integrity Utilizing the DELPHI (TMS-EEG) System. Front Neurosci. 2020;14:589107. Epub 2021/01/08. doi: 10.3389/fnins.2020.589107. PubMed PMID: 33408607; PubMed Central PMCID: PMCPMC7779791.

885. Liu J, Wang XL, Zi L, Yang CH, Li HP, Li N. [Effect of early electroacupuncture intervention on conscious state of patients after traumatic brain injury surgery]. Zhongguo Zhen Jiu. 2020;40(5):479-82. Epub 2020/05/13. doi: 10.13703/j.0255-2930.20190506-0005. PubMed PMID: 32394653.

886. Lucente G, Valls-Sole J, Murillo N, Rothwell J, Coll J,

Davalos A, et al. Noninvasive Brain Stimulation and Noninvasive Peripheral Stimulation for Neglect Syndrome Following Acquired Brain Injury. Neuromodulation. 2020;23(3):312-23. Epub 2019/11/15. doi: 10.1111/ner.13062. PubMed PMID: 31725939.

887. Luo F, Li Y, Norgbey E, Li R, Ya Z, Nwankwegu AS, et al. A study on the occurrence of black water in reservoirs in Eucalyptus Plantation region. Environ Sci Pollut Res Int. 2020;27(28):34927-40. Epub 2020/06/25. doi: 10.1007/s11356-020-09613-3. PubMed PMID: 32577983.

888. Lutkenhoff ES, Johnson MA, Casarotto S, Massimini M, Monti MM. Subcortical atrophy correlates with the perturbational complexity index in patients with disorders of consciousness. Brain Stimul. 2020;13(5):1426-35. Epub 2020/07/28. doi: 10.1016/j.brs.2020.07.012. PubMed PMID: 32717393.

889. Mailleux L, Simon-Martinez C, Radwan A, Blommaert J, Gooijers J, Wenderoth N, et al. White matter characteristics of motor, sensory and interhemispheric tracts underlying impaired upper limb function in children with unilateral cerebral palsy. Brain Struct Funct. 2020;225(5):1495-509. Epub 2020/04/23. doi: 10.1007/s00429-020-02070-1. PubMed PMID: 32318818.

890. Maj R, Osório TG, Borio G, Iacopino S, Ströker E, Sieira J, et al. A novel strategy to treat vaso-vagal syncope: Cardiac

neuromodulation by cryoballoon pulmonary vein isolation. Indian Pacing Electrophysiol J. 2020;20(4):154-9. Epub 2020/04/01. doi: 10.1016/j.ipej.2020.03.008. PubMed PMID: 32224089; PubMed Central PMCID: PMCPMC7371945.

891. Makhdoom SN, Campbell KM, Carvalho RM, Manso AP. Effects of curing modes on depth of cure and microtensile bond strength of bulk fill composites to dentin. J Appl Oral Sci. 2020;28:e20190753. Epub 2020/07/09. doi: 10.1590/1678-7757-2019-0753. PubMed PMID: 32638829; PubMed Central PMCID: PMCPMC7340207.

892. Marion S, Leonid C, Belinda B, Joanne D, Elise H, Leeanne C, et al. Effects of modified-release fampridine on upper limb impairment in patients with Multiple Sclerosis. Mult Scler Relat Disord. 2020;40:101971. Epub 2020/02/18. doi: 10.1016/j.msard.2020.101971. PubMed PMID: 32062444.

893. Martens G, Kroupi E, Bodien Y, Frasso G, Annen J, Cassol H, et al. Behavioral and electrophysiological effects of network-based frontoparietal tDCS in patients with severe brain injury: A randomized controlled trial. Neuroimage Clin. 2020;28:102426. Epub 2020/09/26. doi: 10.1016/j.nicl.2020.102426. PubMed PMID: 32977212; PubMed Central PMCID: PMCPMC7511767.

894. Mencarelli L, Biagi MC, Salvador R, Romanella S, Ruffini G,

Rossi S, et al. Network Mapping of Connectivity Alterations in Disorder of Consciousness: Towards Targeted Neuromodulation. J Clin Med. 2020;9(3). Epub 2020/03/22. doi: 10.3390/jcm9030828. PubMed PMID: 32197485; PubMed Central PMCID: PMCPMC7141258.

895. Mensen A, Bodart O, Thibaut A, Wannez S, Annen J, Laureys S, et al. Decreased Evoked Slow-Activity After tDCS in Disorders of Consciousness. Front Syst Neurosci. 2020;14:62. Epub 2020/10/27. doi: 10.3389/fnsys.2020.00062. PubMed PMID: 33100977; PubMed Central PMCID: PMCPMC7546425.

896. Miller CP, Prener M, Dissing S, Paulson OB. Transcranial low-frequency pulsating electromagnetic fields (T-PEMF) as post-concussion syndrome treatment. Acta Neurol Scand. 2020;142(6):597-604. Epub 2020/06/20. doi: 10.1111/ane.13300. PubMed PMID: 32559313; PubMed Central PMCID: PMCPMC7689717.

897. Miller SM. Endoscopic recurrent laryngeal neuropathy grade prevalence in a sample of thoroughbred yearlings at public auction in South Africa (2013-2019). J S Afr Vet Assoc. 2020;91(0):e1-e5. Epub 2020/05/07. doi: 10.4102/jsava.v91i0.2013. PubMed PMID: 32370531; PubMed Central PMCID: PMCPMC7203182.

898. Motes MA, Spence JS, Yeatman K, Jones PM, Lutrell M,

O'Hair R, et al. High-Definition Transcranial Direct Current Stimulation to Improve Verbal Retrieval Deficits in Chronic Traumatic Brain Injury. J Neurotrauma. 2020;37(1):170-7. Epub 2019/07/30. doi: 10.1089/neu.2018.6331. PubMed PMID: 31354040.

899. Murovec N, Heilinger A, Xu R, Ortner R, Spataro R, La Bella V, et al. Effects of a Vibro-Tactile P300 Based Brain-Computer Interface on the Coma Recovery Scale-Revised in Patients With Disorders of Consciousness. Front Neurosci. 2020;14:294. Epub 2020/04/25. doi: 10.3389/fnins.2020.00294. PubMed PMID: 32327970; PubMed Central PMCID: PMCPMC7161577.

900. Narapareddy BR, Narapareddy L, Lin A, Wigh S, Nanavati J, Dougherty J, 3rd, et al. Treatment of Depression After Traumatic Brain Injury: A Systematic Review Focused on Pharmacological and Neuromodulatory Interventions. Psychosomatics. 2020;61(5):481-97. Epub 2020/07/15. doi: 10.1016/j.psym.2020.04.012. PubMed PMID: 32660873.

901. Nardone R, Sebastianelli L, Brigo F, Golaszewski S, Trinka E, Pucks-Faes E, et al. Effects of intrathecal baclofen therapy in subjects with disorders of consciousness: a reappraisal. J Neural Transm (Vienna). 2020;127(9):1209-15. Epub 2020/07/28. doi: 10.1007/s00702-020-02233-8. PubMed PMID: 32710152.

902. Nardone R, Sebastianelli L, Versace V, Brigo F, Golaszewski S, Manganotti P, et al. Repetitive transcranial magnetic stimulation in traumatic brain injury: Evidence from animal and human studies. Brain Res Bull. 2020;159:44-52. Epub 2020/04/07. doi: 10.1016/j.brainresbull.2020.03.016. PubMed PMID: 32251693.
903. Oberman LM, Exley S, Philip NS, Siddiqi SH, Adamson MM, Brody DL. Use of Repetitive Transcranial Magnetic Stimulation in the Treatment of Neuropsychiatric and Neurocognitive Symptoms Associated With Concussion in Military Populations. J Head Trauma Rehabil. 2020;35(6):388-400. Epub 2020/11/10. doi: 10.1097/htr.0000000000000628. PubMed PMID: 33165152; PubMed Central PMCID: PMCPMC7664843.
904. Pagnozzi AM, Pannek K, Fripp J, Fiori S, Boyd RN, Rose S. Understanding the impact of bilateral brain injury in children with unilateral cerebral palsy. Hum Brain Mapp. 2020;41(10):2794-807. Epub 2020/03/07. doi: 10.1002/hbm.24978. PubMed PMID: 32134174; PubMed Central PMCID: PMCPMC7294067.
905. Park JH, Lee JH, Lee S, Shin JY, Kim TH. Adverse events related to electroacupuncture: a systematic review of single case studies and case series. Acupunct Med. 2020;38(6):407-16. Epub 2020/05/19. doi: 10.1177/0964528420920287. PubMed PMID: 32418438.

906. Pearce AJ, Kidgell DJ, Frazer AK, King DA, Buckland ME, Tommerdahl M. Corticomotor correlates of somatosensory reaction time and variability in individuals with post concussion symptoms. Somatosens Mot Res. 2020;37(1):14-21. Epub 2019/12/07. doi: 10.1080/08990220.2019.1699045. PubMed PMID: 31809669.

907. Phillips A, Sami S, Adamson M. Sex Differences in Neuromodulation Treatment Approaches for Traumatic Brain Injury: A Scoping Review. J Head Trauma Rehabil. 2020;35(6):412-29. Epub 2020/11/10. doi: 10.1097/htr.0000000000000631. PubMed PMID: 33165154.

908. Phillips N, Dalziel SR, Borland ML, Dalton S, Lyttle MD, Bressan S, et al. Imaging and admission practices in paediatric head injury across emergency departments in Australia and New Zealand: A PREDICT study. Emerg Med Australas. 2020;32(2):240-9. Epub 2019/11/28. doi: 10.1111/1742-6723.13396. PubMed PMID: 31773866.

909. Pommier B, Quesada C, Nuti C, Peyron R, Vassal F. Is the analgesic effect of motor cortex stimulation somatotopically driven or not? Neurophysiol Clin. 2020;50(3):195-203. Epub 2020/05/22. doi: 10.1016/j.neucli.2020.04.002. PubMed PMID: 32434693.

910. Qian Q, Ling YT, Zhong H, Zheng YP, Alam M. Restoration of arm and hand functions via noninvasive cervical cord

neuromodulation after traumatic brain injury: a case study. Brain Inj. 2020;34(13-14):1771-80. Epub 2020/12/03. doi: 10.1080/02699052.2020.1850864. PubMed PMID: 33264033.

911. Quinn DK, Upston J, Jones T, Brandt E, Story-Remer J, Fratzke V, et al. Cerebral Perfusion Effects of Cognitive Training and Transcranial Direct Current Stimulation in Mild-Moderate TBI. Front Neurol. 2020;11:545174. Epub 2020/10/30. doi: 10.3389/fneur.2020.545174. PubMed PMID: 33117255; PubMed Central PMCID: PMCPMC7575722.

912. Rodrigues PA, Zaninotto AL, Ventresca HM, Neville IS, Hayashi CY, Brunoni AR, et al. The Effects of Repetitive Transcranial Magnetic Stimulation on Anxiety in Patients With Moderate to Severe Traumatic Brain Injury: A Post-hoc Analysis of a Randomized Clinical Trial. Front Neurol. 2020;11:564940. Epub 2020/12/22. doi: 10.3389/fneur.2020.564940. PubMed PMID: 33343483; PubMed Central PMCID: PMCPMC7746857.

913. Romero-Martínez Á, Bressanutti S, Moya-Albiol L. A Systematic Review of the Effectiveness of Non-Invasive Brain Stimulation Techniques to Reduce Violence Proneness by Interfering in Anger and Irritability. J Clin Med. 2020;9(3). Epub 2020/03/28. doi: 10.3390/jcm9030882. PubMed PMID: 32213818; PubMed Central PMCID: PMCPMC7141522.

914. Rosenstock T, Picht T, Schneider H, Vajkoczy P, Thomale UW. Pediatric navigated transcranial magnetic stimulation motor and language mapping combined with diffusion tensor imaging tractography: clinical experience. J Neurosurg Pediatr. 2020;26(5):583-93. Epub 2020/07/25. doi: 10.3171/2020.4.Peds20174. PubMed PMID: 32707554.

915. Sarasso S, D'Ambrosio S, Fecchio M, Casarotto S, Viganò A, Landi C, et al. Local sleep-like cortical reactivity in the awake brain after focal injury. Brain. 2020;143(12):3672-84. Epub 2020/11/15. doi: 10.1093/brain/awaa338. PubMed PMID: 33188680; PubMed Central PMCID: PMCPMC7805800.

916. Senatus P, Zurek S, Deogaonkar M. Deep Brain Stimulation and Motor Cortex Stimulation for Chronic Pain. Neurol India. 2020;68(Supplement):S235-s40. Epub 2020/12/16. doi: 10.4103/0028-3886.302471. PubMed PMID: 33318357.

917. Sinitsyn DO, Poydasheva AG, Bakulin IS, Legostaeva LA, Iazeva EG, Sergeev DV, et al. Detecting the Potential for Consciousness in Unresponsive Patients Using the Perturbational Complexity Index. Brain Sci. 2020;10(12). Epub 2020/12/03. doi: 10.3390/brainsci10120917. PubMed PMID: 33260944; PubMed Central PMCID: PMCPMC7760168.

918. Srivastav AK, Sharma N, Samuel AJ. tDCS combined with

cognitive training in a patient with chronic traumatic head injury. Neurophysiol Clin. 2020;50(2):133-4. Epub 2020/03/10. doi: 10.1016/j.neucli.2020.02.004. PubMed PMID: 32147282.

919. Stilling J, Paxman E, Mercier L, Gan LS, Wang M, Amoozegar F, et al. Treatment of Persistent Post-Traumatic Headache and Post-Concussion Symptoms Using Repetitive Transcranial Magnetic Stimulation: A Pilot, Double-Blind, Randomized Controlled Trial. J Neurotrauma. 2020;37(2):312-23. Epub 2019/09/19. doi: 10.1089/neu.2019.6692. PubMed PMID: 31530227.

920. Stokes W, Runnalls K, Choynowki J, St Pierre M, Anaya M, Statton MA, et al. Altered corticomotor latencies but normal motor neuroplasticity in concussed athletes. J Neurophysiol. 2020;123(5):1600-5. Epub 2020/02/20. doi: 10.1152/jn.00774.2019. PubMed PMID: 32073936; PubMed Central PMCID: PMCPMC7444927.

921. Strüber D, Herrmann CS. Modulation of gamma oscillations as a possible therapeutic tool for neuropsychiatric diseases: A review and perspective. Int J Psychophysiol. 2020;152:15-25. Epub 2020/04/03. doi: 10.1016/j.ijpsycho.2020.03.003. PubMed PMID: 32240665.

922. Stultz DJ, Osburn S, Burns T, Pawlowska-Wajswol S, Walton

R. Transcranial Magnetic Stimulation (TMS) Safety with Respect to Seizures: A Literature Review. Neuropsychiatr Dis Treat. 2020;16:2989-3000. Epub 2020/12/17. doi: 10.2147/ndt.S276635. PubMed PMID: 33324060; PubMed Central PMCID: PMCPMC7732158.

923. Sun J, Shi J, Hou J, Guo C, Heng X, Qi G. Electroacupuncture protects rats from ischemic brain injury via coffilin in mice. J Cell Biochem. 2020;121(1):43-8. Epub 2019/10/11. doi: 10.1002/jcb.27781. PubMed PMID: 31599049.

924. Talbot CE, Zhao K, Ward M, Kandinov A, Mammis A, Paskhover B. Neuromodulation of the lingual nerve: a novel technique. J Neurosurg. 2020;134(3):1271-5. Epub 2020/04/25. doi: 10.3171/2020.2.Jns193109. PubMed PMID: 32330885.

925. Texakalidis P, Tora MS, McMahon JT, Greven A, Anthony CL, Nagarajan P, et al. Percutaneous Trigeminal Stimulation for Intractable Facial Pain: A Case Series. Neurosurgery. 2020;87(3):547-54. Epub 2020/04/04. doi: 10.1093/neuros/nyaa065. PubMed PMID: 32243534.

926. Tseng CT, Brougher J, Gaulding SJ, Hassan BS, Thorn CA. Vagus nerve stimulation promotes cortical reorganization and reduces task-dependent calorie intake in male and female rats. Brain Res. 2020;1748:147099. Epub 2020/09/09. doi:

10.1016/j.brainres.2020.147099. PubMed PMID: 32896520.

927. Tzur R, David M, Barron J, Jaul E, Meiron O. Prefrontal cortex transcranial direct-current stimulation putatively enhances electroencephalography classification resolution of binary responses in a minimal conscious state patient. Clin Neurophysiol. 2020;131(9):2148-9. Epub 2020/07/19. doi: 10.1016/j.clinph.2020.06.009. PubMed PMID: 32682242.

928. Vallence AM, Fujiyama H, Rurak BK, Wansbrough K, Haigh Y. Suspected vasovagal syncope during single-pulse TMS in healthy adults: Three case reports. Clin Neurophysiol. 2020;131(11):2540-1. Epub 2020/09/15. doi: 10.1016/j.clinph.2020.08.007. PubMed PMID: 32927208.

929. Van Dam J, Goldsworthy M. Case report of a vasovagal pre-syncope event during single-pulse transcranial magnetic stimulation in a healthy adult participant. Clin Neurophysiol. 2020;131(4):981-2. Epub 2020/02/06. doi: 10.1016/j.clinph.2019.12.401. PubMed PMID: 32008908.

930. Wang X, Guo Y, Zhang Y, Li J, Gao Z, Li Y, et al. Combined Behavioral and Mismatch Negativity Evidence for the Effects of Long-Lasting High-Definition tDCS in Disorders of Consciousness: A Pilot Study. Front Neurosci. 2020;14:381. Epub 2020/05/16. doi: 10.3389/fnins.2020.00381. PubMed PMID: 32410950; PubMed

Central PMCID: PMCPMC7198816.

931. Wende T, Hoffmann KT, Meixensberger J. Tractography in Neurosurgery: A Systematic Review of Current Applications. J Neurol Surg A Cent Eur Neurosurg. 2020;81(5):442-55. Epub 2020/03/17. doi: 10.1055/s-0039-1691823. PubMed PMID: 32176926.

932. Wu XL, Liu LX, Yang LY, Zhang T. Comprehensive rehabilitation in a patient with corpus callosum syndrome after traumatic brain injury: Case report. Medicine (Baltimore). 2020;99(28):e21218. Epub 2020/07/16. doi: 10.1097/md.0000000000021218. PubMed PMID: 32664173; PubMed Central PMCID: PMCPMC7360258.

933. Wu Y, Wang L, Yang F, Xi W. Neural Circuits for Sleep-Wake Regulation. Adv Exp Med Biol. 2020;1284:91-112. Epub 2020/08/28. doi: 10.1007/978-981-15-7086-5_8. PubMed PMID: 32852742.

934. Xu G, Sheng Q, Xin Q, Song Y, Zhang G, Yuan L, et al. EEG Assessment in a 2-Year-Old Child with Prolonged Disorders of Consciousness: 3 Years' Follow-up. Comput Intell Neurosci. 2020;2020:8826238. Epub 2020/12/10. doi: 10.1155/2020/8826238. PubMed PMID: 33293944; PubMed Central PMCID: PMCPMC7718066.

935. Yang Q, An PF, Wang RH, Wang D, Zhang RC. [Effect of electroacupuncture at different stages on the expression of Fas and FasL in brain tissue of rats with traumatic brain injury]. Zhen Ci Yan Jiu. 2020;45(9):714-9. Epub 2020/09/23. doi: 10.13702/j.1000-0607.190863. PubMed PMID: 32959553.
936. Yasen AL, Lim MM, Weymann KB, Christie AD. Excitability, Inhibition, and Neurotransmitter Levels in the Motor Cortex of Symptomatic and Asymptomatic Individuals Following Mild Traumatic Brain Injury. Front Neurol. 2020;11:683. Epub 2020/08/09. doi: 10.3389/fneur.2020.00683. PubMed PMID: 32765402; PubMed Central PMCID: PMCPMC7381132.
937. Zewdie E, Ciechanski P, Kuo HC, Giuffre A, Kahl C, King R, et al. Safety and tolerability of transcranial magnetic and direct current stimulation in children: Prospective single center evidence from 3.5 million stimulations. Brain Stimul. 2020;13(3):565-75. Epub 2020/04/15. doi: 10.1016/j.brs.2019.12.025. PubMed PMID: 32289678.
938. Zhang D, Fu Y, Dionigi G, Pontin A, Caruso E, Antonella P, et al. Human cadaveric model for studying the preservation of mental nerve during transoral endoscopic thyroidectomy. Surg Radiol Anat. 2020;42(1):55-62. Epub 2019/08/25. doi: 10.1007/s00276-019-02306-8. PubMed PMID: 31444547.

939. Zhang D, Li S, Dionigi G, Wang T, Zhang J, Xue G, et al. Consequences of Trocar Dynamics on Mental Nerve in Transoral Thyroidectomy. Surg Laparosc Endosc Percutan Tech. 2020;30(4):305-11. Epub 2020/05/28. doi: 10.1097/sle.0000000000000795. PubMed PMID: 32459705.

940. Zhang Q, Liu J, Cao R, Jin Y. Acupuncture for Patients in Coma after Traumatic Brain Injury: Systematic Review and Meta-Analysis. Altern Ther Health Med. 2020;26(4):50-7. Epub 2020/02/24. PubMed PMID: 32088667.

941. Zhang R, Zhang L, Guo Y, Shi L, Gao J, Wang X, et al. Effects of High-Definition Transcranial Direct-Current Stimulation on Resting-State Functional Connectivity in Patients With Disorders of Consciousness. Front Hum Neurosci. 2020;14:560586. Epub 2020/10/27. doi: 10.3389/fnhum.2020.560586. PubMed PMID: 33100996; PubMed Central PMCID: PMCPMC7546763.

942. Zhang X, Liu B, Li N, Li Y, Hou J, Duan G, et al. Transcranial Direct Current Stimulation Over Prefrontal Areas Improves Psychomotor Inhibition State in Patients With Traumatic Brain Injury: A Pilot Study. Front Neurosci. 2020;14:386. Epub 2020/06/09. doi: 10.3389/fnins.2020.00386. PubMed PMID: 32508560; PubMed Central PMCID: PMCPMC7251071.

943. Zhang XH, Li FF, Qi Y, Ming CR, Li Y, Pan ST, et al.

[Electroacupuncture improves cutaneous allergic reaction by inhibiting degranulation of intrape-ritoneal mast cells, MAPK signaling and inflammatory factor levels in urticaria rats]. Zhen Ci Yan Jiu. 2020;45(4):299-304. Epub 2020/04/26. doi: 10.13702/j.1000-0607.190263. PubMed PMID: 32333535.

944. Zhang Y, Lu J, Du J, Huo S, Li R, Song W. Neural correlates of different behavioral response to transcranial direct current stimulation between patients in the unresponsive wakefulness syndrome and minimally conscious state. Neurol Sci. 2020;41(1):75-82. Epub 2019/08/20. doi: 10.1007/s10072-019-04034-8. PubMed PMID: 31422504.

945. Zheng SM, Zhao FL, Luo YY, Lin XF, Wen MY. [Clinical effect of electroacupuncture at Baihui and Shuigou points in treatment of brain injury in patients with sepsis-associated encephalopathy]. Zhen Ci Yan Jiu. 2020;45(5):402-6. Epub 2020/05/25. doi: 10.13702/j.1000-0607.190781. PubMed PMID: 32447856.

946. Ahorsu DK, Adjaottor ES, Lam BYH. Intervention Effect of Non-Invasive Brain Stimulation on Cognitive Functions among People with Traumatic Brain Injury: A Systematic Review and Meta-Analysis. Brain Sci. 2021;11(7). Epub 2021/07/03. doi: 10.3390/brainsci11070840. PubMed PMID: 34202739; PubMed

Central PMCID: PMCPMC8301762.
947. Aksu T, Davila A, Gupta D. The "heart brain" and neuromodulation for vasovagal syncope. Auton Neurosci. 2021;236:102892. Epub 2021/10/20. doi: 10.1016/j.autneu.2021.102892. PubMed PMID: 34666205.
948. Aksu T, Turagam M, Gautam S, Futyma P, Akella K, Baysal E, et al. Effects of permanent cardiac pacing on ventricular repolarization when compared to cardioneuroablation. J Electrocardiol. 2021;67:13-8. Epub 2021/05/14. doi: 10.1016/j.jelectrocard.2021.04.006. PubMed PMID: 33984570.
949. Aloi D, Della Rocchetta AI, Ditchfield A, Coulborn S, Fernández-Espejo D. Therapeutic Use of Transcranial Direct Current Stimulation in the Rehabilitation of Prolonged Disorders of Consciousness. Front Neurol. 2021;12:632572. Epub 2021/04/27. doi: 10.3389/fneur.2021.632572. PubMed PMID: 33897592; PubMed Central PMCID: PMCPMC8058460.
950. Arai N, Nakanishi T, Nakajima S, Li X, Wada M, Daskalakis ZJ, et al. Insights of neurophysiology on unconscious state using combined transcranial magnetic stimulation and electroencephalography: A systematic review. Neurosci Biobehav Rev. 2021;131:293-312. Epub 2021/09/24. doi: 10.1016/j.neubiorev.2021.09.029. PubMed PMID: 34555384.

951. Argyriou AA, Mitsikostas DD, Mantovani E, Litsardopoulos P, Panagiotopoulos V, Tamburin S. An updated brief overview on post-traumatic headache and a systematic review of the non-pharmacological interventions for its management. Expert Rev Neurother. 2021;21(4):475-90. Epub 2021/03/09. doi: 10.1080/14737175.2021.1900734. PubMed PMID: 33682560.
952. Bai Y, Lin Y, Ziemann U. Managing disorders of consciousness: the role of electroencephalography. J Neurol. 2021;268(11):4033-65. Epub 2020/09/12. doi: 10.1007/s00415-020-10095-z. PubMed PMID: 32915309; PubMed Central PMCID: PMCPMC8505374.
953. Barth BB, Travis L, Spencer NJ, Grill WM. Control of colonic motility using electrical stimulation to modulate enteric neural activity. Am J Physiol Gastrointest Liver Physiol. 2021;320(4):G675-g87. Epub 2021/02/25. doi: 10.1152/ajpgi.00463.2020. PubMed PMID: 33624530; PubMed Central PMCID: PMCPMC8238160.
954. Boissonnault È, Higgins J, LaGarde G, Barthélemy D, Lamarre C, J HD. Brain stimulation in attention deficits after traumatic brain injury: a literature review and feasibility study. Pilot Feasibility Stud. 2021;7(1):115. Epub 2021/06/02. doi: 10.1186/s40814-021-00859-3. PubMed PMID: 34059152; PubMed

Central PMCID: PMCPMC8165970.
955. Cambiaghi M, Cherchi L, Masin L, Infortuna C, Briski N, Caviasco C, et al. High-frequency repetitive transcranial magnetic stimulation enhances layer II/III morphological dendritic plasticity in mouse primary motor cortex. Behav Brain Res. 2021;410:113352. Epub 2021/05/13. doi: 10.1016/j.bbr.2021.113352. PubMed PMID: 33979657.
956. Chantigian DP, Larson M, Chen M, Gillick BT, Cross TJ, Keller-Ross ML. Heart rate and blood pressure responses to corticomotor stimulation in young, healthy adults. Clin Neurophysiol Pract. 2021;6:206-8. Epub 2021/08/12. doi: 10.1016/j.cnp.2021.06.004. PubMed PMID: 34377873; PubMed Central PMCID: PMCPMC8327479.
957. Chen B, Lin WQ, Li ZF, Zhong XY, Wang J, You XF, et al. Electroacupuncture Attenuates Ischemic Brain Injury and Cellular Apoptosis via Mitochondrial Translocation of Cofilin. Chin J Integr Med. 2021;27(9):705-12. Epub 2021/03/13. doi: 10.1007/s11655-021-3335-4. PubMed PMID: 33709239.
958. Chen XL, Yu LP, Zhu Y, Wang TY, Han J, Chen XY, et al. Combined effect of hydrotherapy and transcranial direct-current stimulation on children with cerebral palsy: A protocol for a randomized controlled trial. Medicine (Baltimore).

2021;100(49):e27962. Epub 2021/12/11. doi: 10.1097/md.0000000000027962. PubMed PMID: 34889241; PubMed Central PMCID: PMCPMC8663893.

959. Chiang HS, Motes M, O'Hair R, Vanneste S, Kraut M, Hart J, Jr. Baseline delayed verbal recall predicts response to high definition transcranial direct current stimulation targeting the superior medial frontal cortex. Neurosci Lett. 2021;764:136204. Epub 2021/09/04. doi: 10.1016/j.neulet.2021.136204. PubMed PMID: 34478816.

960. Chiang HS, Shakal S, Vanneste S, Kraut M, Hart J, Jr. Case Report: Improving Verbal Retrieval Deficits With High Definition Transcranial Direct Current Stimulation Targeting the Pre-Supplementary Motor Area in a Patient With Chronic Traumatic Brain Injury. Front Neurol. 2021;12:678518. Epub 2021/08/03. doi: 10.3389/fneur.2021.678518. PubMed PMID: 34335447; PubMed Central PMCID: PMCPMC8322436.

961. De Andrés J, Navarrete-Rueda F, Fabregat G, García-Gutiérrez MS, Monsalve-Dolz V, Harutyunyan A, et al. Differences in Gene Expression of Endogenous Opioid Peptide Precursor, Cannabinoid 1 and 2 Receptors and Interleukin Beta in Peripheral Blood Mononuclear Cells of Patients With Refractory Failed Back Surgery Syndrome Treated With Spinal Cord

Stimulation: Markers of Therapeutic Outcomes? Neuromodulation. 2021;24(1):49-60. Epub 2020/02/07. doi: 10.1111/ner.13111. PubMed PMID: 32027775.

962. De Freitas DJ, De Carvalho D, Paglioni VM, Brunoni AR, Valiengo L, Thome-Souza MS, et al. Effects of transcranial direct current stimulation (tDCS) and concurrent cognitive training on episodic memory in patients with traumatic brain injury: a double-blind, randomised, placebo-controlled study. BMJ Open. 2021;11(8):e045285. Epub 2021/08/28. doi: 10.1136/bmjopen-2020-045285. PubMed PMID: 34446480; PubMed Central PMCID: PMCPMC8395342.

963. Deblieck C, Smeijers S, Morlion B, Datta A, Thomas C, Theys T. Case Report: Initial Evidence of Safety and Efficacy of High Definition-Transcranial Direct Current Stimulation in a Patient With Neuropathic Pain and Implanted Spinal Cord Stimulator. Front Pain Res (Lausanne). 2021;2:753464. Epub 2022/03/18. doi: 10.3389/fpain.2021.753464. PubMed PMID: 35295503; PubMed Central PMCID: PMCPMC8915614.

964. Di Gregorio F, La Porta F, Lullini G, Casanova E, Petrone V, Simoncini L, et al. Efficacy of Repetitive Transcranial Magnetic Stimulation Combined With Visual Scanning Treatment on Cognitive-Behavioral Symptoms of Unilateral Spatial Neglect in

Patients With Traumatic Brain Injury: Study Protocol for a Randomized Controlled Trial. Front Neurol. 2021;12:702649. Epub 2021/08/03. doi: 10.3389/fneur.2021.702649. PubMed PMID: 34335455; PubMed Central PMCID: PMCPMC8317998.

965. Dong J, Wang L, Xiong R, Liu X, Guo Z, Sun W, et al. [A 180-day mortality predictive score based on frailty syndrome in elderly patients with sepsis: a Logistic regression analysis model]. Zhonghua Wei Zhong Bing Ji Jiu Yi Xue. 2021;33(3):257-62. Epub 2021/04/10. doi: 10.3760/cma.j.cn121430-20201027-00688. PubMed PMID: 33834964.

966. Eilam-Stock T, George A, Charvet LE. Cognitive Telerehabilitation with Transcranial Direct Current Stimulation Improves Cognitive and Emotional Functioning Following a Traumatic Brain Injury: A Case Study. Arch Clin Neuropsychol. 2021;36(3):442-53. Epub 2021/04/23. doi: 10.1093/arclin/acaa059. PubMed PMID: 33885138.

967. Eskildsen SJ, Poulsen I, Jakobsen D, Riberholt CG, Curtis DJ. Scoping review to identify and map non-pharmacological, non-surgical treatments for dysphagia following moderate-to-severe acquired brain injury. BMJ Open. 2021;11(12):e053244. Epub 2021/12/04. doi: 10.1136/bmjopen-2021-053244. PubMed PMID: 34857571;

PubMed Central PMCID: PMCPMC8640633.
968. Fekete K, Tóth J, Horváth L, Márton S, Héja M, Csiba L, et al. Neurophysiological Examinations as Adjunctive Tool to Imaging Techniques in Spontaneous Intracerebral Hemorrhage: IRONHEART Study. Front Neurol. 2021;12:757078. Epub 2021/11/16. doi: 10.3389/fneur.2021.757078. PubMed PMID: 34777228; PubMed Central PMCID: PMCPMC8578792.
969. Feller D, Vinante C, Trentin F, Innocenti T. The effectiveness of median nerve electrical stimulation in patients with disorders of consciousness: a systematic review. Brain Inj. 2021;35(4):385-94. Epub 2021/02/23. doi: 10.1080/02699052.2021.1887522. PubMed PMID: 33617359.
970. Formica C, De Salvo S, Corallo F, Alagna A, Logiudice AL, Todaro A, et al. Role of neurorehabilitative treatment using transcranial magnetic stimulation in disorders of consciousness. J Int Med Res. 2021;49(2):300060520976472. Epub 2021/02/05. doi: 10.1177/0300060520976472. PubMed PMID: 33535855; PubMed Central PMCID: PMCPMC7869152.
971. Furlan JC, Pakosh M, Craven BC, Popovic MR. Insights on the Potential Mechanisms of Action of Functional Electrical Stimulation Therapy in Combination With Task-Specific Training: A Scoping Review. Neuromodulation. 2021. Epub 2021/05/26. doi:

10.1111/ner.13403. PubMed PMID: 34031937.

972. Galafassi GZ, Simm Pires de Aguiar PH, Simm RF, Franceschini PR, Filho MP, Pagura JR, et al. Neuromodulation for Medically Refractory Neuropathic Pain: Spinal Cord Stimulation, Deep Brain Stimulation, Motor Cortex Stimulation, and Posterior Insula Stimulation. World Neurosurg. 2021;146:246-60. Epub 2020/11/21. doi: 10.1016/j.wneu.2020.11.048. PubMed PMID: 33217591.

973. Gatzinsky K, Bergh C, Liljegren A, Silander H, Samuelsson J, Svanberg T, et al. Repetitive transcranial magnetic stimulation of the primary motor cortex in management of chronic neuropathic pain: a systematic review. Scand J Pain. 2021;21(1):8-21. Epub 2020/09/07. doi: 10.1515/sjpain-2020-0054. PubMed PMID: 32892189.

974. Ge X, Zhang Y, Xin T, Luan X. Effects of 10 Hz repetitive transcranial magnetic stimulation of the right dorsolateral prefrontal cortex in the vegetative state. Exp Ther Med. 2021;21(3):206. Epub 2021/01/28. doi: 10.3892/etm.2021.9626. PubMed PMID: 33500699; PubMed Central PMCID: PMCPMC7818534.

975. Hara T, Shanmugalingam A, McIntyre A, Burhan AM. The Effect of Non-Invasive Brain Stimulation (NIBS) on Executive

Functioning, Attention and Memory in Rehabilitation Patients with Traumatic Brain Injury: A Systematic Review. Diagnostics (Basel). 2021;11(4). Epub 2021/04/04. doi: 10.3390/diagnostics11040627. PubMed PMID: 33807188; PubMed Central PMCID: PMCPMC8066265.

976. He RH, Wang HJ, Zhou Z, Fan JZ, Zhang SQ, Zhong YH. The influence of high-frequency repetitive transcranial magnetic stimulation on endogenous estrogen in patients with disorders of consciousness. Brain Stimul. 2021;14(3):461-6. Epub 2021/03/08. doi: 10.1016/j.brs.2021.02.014. PubMed PMID: 33677157.

977. Heslot C, Cogné M, Guillouët E, Perdrieau V, Lefevre-Dognin C, Glize B, et al. Management of unfavorable outcome after mild traumatic brain injury: Review of physical and cognitive rehabilitation and of psychological care in post-concussive syndrome. Neurochirurgie. 2021;67(3):283-9. Epub 2020/10/14. doi: 10.1016/j.neuchi.2020.09.001. PubMed PMID: 33049290.

978. Horton GA, Ibrahim O, Jansen M, Trier J, Milev R, Beyea JA. Repetitive Transcranial Magnetic Stimulation For The Treatment of Chronic Tinnitus: A Preliminary Study of The Influence of Traumatic Brain Injury on Treatment Response. Int Tinnitus J. 2021;25(1):51-8. Epub 2021/08/20. doi: 10.5935/0946-5448.20210011. PubMed PMID: 34410080.

979. Hosseinian T, Yavari F, Biagi MC, Kuo MF, Ruffini G, Nitsche MA, et al. External induction and stabilization of brain oscillations in the human. Brain Stimul. 2021;14(3):579-87. Epub 2021/03/31. doi: 10.1016/j.brs.2021.03.011. PubMed PMID: 33781955; PubMed Central PMCID: PMCPMC8144019.

980. Juárez-Belaúnde A, Orcajo E, Lejarreta S, Davila-Pérez P, León N, Oliviero A. Fatigue in patients with acquired brain damage. Neurologia (Engl Ed). 2021. Epub 2021/09/21. doi: 10.1016/j.nrl.2021.05.011. PubMed PMID: 34538507.

981. Kolchina AN, Yatsyshina EE, Malysheva LV, Ledentsova EE, Lidyaeva EE, Khaletskaya OV. Diagnostics of Inherited Metabolic Diseases in Newborns with the Hyperammonemia Syndrome at the Onset of Disease (Pilot Study). Sovrem Tekhnologii Med. 2021;13(1):59-64. Epub 2021/09/14. doi: 10.17691/stm2021.13.1.07. PubMed PMID: 34513067; PubMed Central PMCID: PMCPMC8353695.

982. Kuo CW, Chang MY, Liu HH, He XK, Chan SY, Huang YZ, et al. Cortical Electrical Stimulation Ameliorates Traumatic Brain Injury-Induced Sensorimotor and Cognitive Deficits in Rats. Front Neural Circuits. 2021;15:693073. Epub 2021/07/02. doi: 10.3389/fncir.2021.693073. PubMed PMID: 34194304; PubMed Central PMCID: PMCPMC8236591.

983. Kurtin DL, Violante IR, Zimmerman K, Leech R, Hampshire A, Patel MC, et al. Investigating the interaction between white matter and brain state on tDCS-induced changes in brain network activity. Brain Stimul. 2021;14(5):1261-70. Epub 2021/08/27. doi: 10.1016/j.brs.2021.08.004. PubMed PMID: 34438046; PubMed Central PMCID: PMCPMC8460997.

984. Li J, Curley WH, Guerin B, Dougherty DD, Dalca AV, Fischl B, et al. Mapping the subcortical connectivity of the human default mode network. Neuroimage. 2021;245:118758. Epub 2021/11/29. doi: 10.1016/j.neuroimage.2021.118758. PubMed PMID: 34838949; PubMed Central PMCID: PMCPMC8945548.

985. Li LP, Chen JK, Coelho DH. Optimizing Location of Subdermal Recording Electrodes for Intraoperative Facial Nerve Monitoring. Laryngoscope. 2021;131(7):E2329-e34. Epub 2021/03/23. doi: 10.1002/lary.29518. PubMed PMID: 33749869.

986. Li W, Yang Y, Huang J, Zhang H, Zeng D, Jiang L, et al. Electroacupuncture-induced plasticity between face and hand representations in motor cortex is associated with recovery of function after facial nerve injury. Acupunct Med. 2021;39(1):75-7. Epub 2020/06/17. doi: 10.1177/0964528420920298. PubMed PMID: 32539425.

987. Lv Y, Dai DC, Jiang HN, Wang L. [Effect of

electroacupuncture on lumbar disc herniation with different multifidus fatty infiltration rates]. Zhongguo Zhen Jiu. 2021;41(6):593-7. Epub 2021/06/05. doi: 10.13703/j.0255-2930.20200817-k0006. PubMed PMID: 34085473.

988. Maeda Y, Otsuka T, Mitsuhara T, Okazaki T, Yuge L, Takeda M. A novel bone-thinning technique for transcranial stimulation motor-evoked potentials in rats. Sci Rep. 2021;11(1):12496. Epub 2021/06/16. doi: 10.1038/s41598-021-91780-5. PubMed PMID: 34127706; PubMed Central PMCID: PMCPMC8203740.

989. Mao B, Wang Y, Balasubramanian T, Urioste R, Wafa T, Fitzgerald TS, et al. Assessment of auditory and vestibular damage in a mouse model after single and triple blast exposures. Hear Res. 2021;407:108292. Epub 2021/07/03. doi: 10.1016/j.heares.2021.108292. PubMed PMID: 34214947; PubMed Central PMCID: PMCPMC8276524.

990. Martens G, Ibáñez-Soria D, Barra A, Soria-Frisch A, Piarulli A, Gosseries O, et al. A novel closed-loop EEG-tDCS approach to promote responsiveness of patients in minimally conscious state: A study protocol. Behav Brain Res. 2021;409:113311. Epub 2021/04/21. doi: 10.1016/j.bbr.2021.113311. PubMed PMID: 33878429.

991. Meek BP, Hill S, Modirrousta M. Accelerated repetitive transcranial magnetic stimulation in the treatment of post-concussion symptoms due to mild traumatic brain injury: a pilot study. Brain Inj. 2021;35(1):48-58. Epub 2020/12/11. doi: 10.1080/02699052.2020.1857837. PubMed PMID: 33297788.

992. Milosevic M, Nakanishi T, Sasaki A, Yamaguchi A, Nomura T, Popovic MR, et al. Cortical Re-organization After Traumatic Brain Injury Elicited Using Functional Electrical Stimulation Therapy: A Case Report. Front Neurosci. 2021;15:693861. Epub 2021/09/08. doi: 10.3389/fnins.2021.693861. PubMed PMID: 34489624; PubMed Central PMCID: PMCPMC8417438.

993. Mollica A, Safavifar F, Fralick M, Giacobbe P, Lipsman N, Burke MJ. Transcranial Magnetic Stimulation for the Treatment of Concussion: A Systematic Review. Neuromodulation. 2021;24(5):803-12. Epub 2020/11/14. doi: 10.1111/ner.13319. PubMed PMID: 33184973.

994. Moreau-Debord I, Serrano É, Quessy S, Dancause N. Rapid and Bihemispheric Reorganization of Neuronal Activity in Premotor Cortex after Brain Injury. J Neurosci. 2021;41(44):9112-28. Epub 2021/09/25. doi: 10.1523/jneurosci.0196-21.2021. PubMed PMID: 34556488; PubMed Central PMCID: PMCPMC8570830.

995. Norgbey E, Li Y, Zhu Y, Nwankwegu AS, Bofah-Buah R, Nuamah L, et al. Combined use of high-resolution dialysis, diffusive gradient in thin films (DGT) technique, and conventional methods to assess trace metals in reservoir sediments. Environ Monit Assess. 2021;193(8):469. Epub 2021/07/07. doi: 10.1007/s10661-021-09247-z. PubMed PMID: 34226963.
996. O'Leary GH, Jenkins DD, Coker-Bolt P, George MS, Kautz S, Bikson M, et al. From adults to pediatrics: A review noninvasive brain stimulation (NIBS) to facilitate recovery from brain injury. Prog Brain Res. 2021;264:287-322. Epub 2021/06/26. doi: 10.1016/bs.pbr.2021.01.019. PubMed PMID: 34167660; PubMed Central PMCID: PMCPMC8855635.
997. O'Neal CM, Schroeder LN, Wells AA, Chen S, Stephens TM, Glenn CA, et al. Patient Outcomes in Disorders of Consciousness Following Transcranial Magnetic Stimulation: A Systematic Review and Meta-Analysis of Individual Patient Data. Front Neurol. 2021;12:694970. Epub 2021/09/04. doi: 10.3389/fneur.2021.694970. PubMed PMID: 34475848; PubMed Central PMCID: PMCPMC8407074.
998. Park G, Suh JH, Han SJ. Transcranial direct current stimulation for balance and gait in repetitive mild traumatic brain injury in rats. BMC Neurosci. 2021;22(1):26. Epub 2021/04/19.

doi: 10.1186/s12868-021-00633-4. PubMed PMID: 33865318; PubMed Central PMCID: PMCPMC8052850.

999. Patterson Gentile C, Shah R, Irwin SL, Greene K, Szperka CL. Acute and chronic management of posttraumatic headache in children: A systematic review. Headache. 2021;61(10):1475-92. Epub 2021/12/05. doi: 10.1111/head.14236. PubMed PMID: 34862612.

1000. Pearce AJ, Kidgell DJ, Tommerdahl MA, Frazer AK, Rist B, Mobbs R, et al. Chronic Neurophysiological Effects of Repeated Head Trauma in Retired Australian Male Sport Athletes. Front Neurol. 2021;12:633320. Epub 2021/03/27. doi: 10.3389/fneur.2021.633320. PubMed PMID: 33767661; PubMed Central PMCID: PMCPMC7985524.

1001. Pincherle A, Rosanova M, Diserens K. Editorial: New Advances in Diagnostic Tools and Rehabilitation of Disorders of Consciousness in the Acute Phase. Front Neurol. 2021;12:770791. Epub 2021/12/07. doi: 10.3389/fneur.2021.770791. PubMed PMID: 34867758; PubMed Central PMCID: PMCPMC8635187.

1002. Pink AE, Williams C, Alderman N, Stoffels M. The use of repetitive transcranial magnetic stimulation (rTMS) following traumatic brain injury (TBI): A scoping review. Neuropsychol Rehabil. 2021;31(3):479-505. Epub 2019/12/28. doi:

10.1080/09602011.2019.1706585. PubMed PMID: 31880207.

1003. Rosalia L, Ozturk C, Shoar S, Fan Y, Malone G, Cheema FH, et al. Device-Based Solutions to Improve Cardiac Physiology and Hemodynamics in Heart Failure With Preserved Ejection Fraction. JACC Basic Transl Sci. 2021;6(9-10):772-95. Epub 2021/11/11. doi: 10.1016/j.jacbts.2021.06.002. PubMed PMID: 34754993; PubMed Central PMCID: PMCPMC8559325.

1004. Rudroff T, Workman CD. Transcranial Direct Current Stimulation as a Treatment Tool for Mild Traumatic Brain Injury. Brain Sci. 2021;11(6). Epub 2021/07/03. doi: 10.3390/brainsci11060806. PubMed PMID: 34207004; PubMed Central PMCID: PMCPMC8235194.

1005. Rushby JA, De Blasio FM, Logan JA, Wearne T, Kornfeld E, Wilson EJ, et al. tDCS effects on task-related activation and working memory performance in traumatic brain injury: A within group randomized controlled trial. Neuropsychol Rehabil. 2021;31(5):814-36. Epub 2020/03/03. doi: 10.1080/09602011.2020.1733620. PubMed PMID: 32114899.

1006. Saepang K, Li SK, Chantasart D. Passive and iontophoretic transport of pramipexole dihydrochloride across human skin microchannels created by microneedles in vitro. Int J Pharm. 2021;609:121092. Epub 2021/09/17. doi:

10.1016/j.ijpharm.2021.121092. PubMed PMID: 34530098.

1007. Sanz LRD, Thibaut A, Edlow BL, Laureys S, Gosseries O. Update on neuroimaging in disorders of consciousness. Curr Opin Neurol. 2021;34(4):488-96. Epub 2021/06/01. doi: 10.1097/wco.0000000000000951. PubMed PMID: 34054109; PubMed Central PMCID: PMCPMC8938964.

1008. Schwarm FP, Ott M, Nagl J, Bender M, Stein M, Uhl E, et al. The Predictive Value of Transcutaneous Electrical Nerve Stimulation for Patient Selection in Peripheral Nerve Field Stimulation for Chronic Low Back Pain: A Prospective Study. Neuromodulation. 2021;24(6):1051-8. Epub 2020/08/07. doi: 10.1111/ner.13244. PubMed PMID: 32757257.

1009. Serruya MD, Rosenwasser RH. An artificial nervous system to treat chronic stroke. Artif Organs. 2021;45(8):804-12. Epub 2021/06/23. doi: 10.1111/aor.13998. PubMed PMID: 34156104.

1010. Shirvani S, Davoudi M, Shirvani M, Koleini P, Hojat Panah S, Shoshtari F, et al. Comparison of the effects of transcranial direct current stimulation and mindfulness-based stress reduction on mental fatigue, quality of life and aggression in mild traumatic brain injury patients: a randomized clinical trial. Ann Gen Psychiatry. 2021;20(1):33. Epub 2021/06/17. doi:

10.1186/s12991-021-00355-1. PubMed PMID: 34130721; PubMed Central PMCID: PMCPMC8207739.

1011. Shou Z, Li Z, Wang X, Chen M, Bai Y, Di H. Non-invasive brain intervention techniques used in patients with disorders of consciousness. Int J Neurosci. 2021;131(4):390-404. Epub 2020/04/03. doi: 10.1080/00207454.2020.1744598. PubMed PMID: 32238043.

1012. Skalický P, Masopust V. Bilateral cortical stimulation for bilateral refractory atypical trigeminal neuropathic pain: Case report. Neuro Endocrinol Lett. 2021;42(2):87-90. Epub 2021/07/05. PubMed PMID: 34217165.

1013. Stephens TM, Young IM, O'Neal CM, Dadario NB, Briggs RG, Teo C, et al. Akinetic mutism reversed by inferior parietal lobule repetitive theta burst stimulation: Can we restore default mode network function for therapeutic benefit? Brain Behav. 2021;11(8):e02180. Epub 2021/06/20. doi: 10.1002/brb3.2180. PubMed PMID: 34145791; PubMed Central PMCID: PMCPMC8413751.

1014. Szarka G, Balogh M, Tengölics Á J, Ganczer A, Völgyi B, Kovács-Öller T. The role of gap junctions in cell death and neuromodulation in the retina. Neural Regen Res. 2021;16(10):1911-20. Epub 2021/03/02. doi:

10.4103/1673-5374.308069. PubMed PMID: 33642359; PubMed Central PMCID: PMCPMC8343308.

1015. Trofimov AO, Agarkova DI, Kopylov AA, Dubrovin A, Trofimova KA, Sheludyakov A, et al. NIRS-Based Assessment of Cerebral Oxygenation During High-Definition Anodal Transcranial Direct Current Stimulation in Patients with Posttraumatic Encephalopathy. Adv Exp Med Biol. 2021;1339:27-31. Epub 2022/01/14. doi: 10.1007/978-3-030-78787-5_4. PubMed PMID: 35023086.

1016. Trofimov AO, Kopylov AA, Martynov DS, Zorkova AV, Trofimova K, Cheremuhin PN, et al. The Changes in Brain Oxygenation During Transcranial Alternating Current Stimulation as Consequences of Traumatic Brain Injury: A Near-Infrared Spectroscopy Study. Adv Exp Med Biol. 2021;1269:235-9. Epub 2021/05/10. doi: 10.1007/978-3-030-48238-1_37. PubMed PMID: 33966223.

1017. Tsai PY, Chen YC, Wang JY, Chung KH, Lai CH. Effect of repetitive transcranial magnetic stimulation on depression and cognition in individuals with traumatic brain injury: a systematic review and meta-analysis. Sci Rep. 2021;11(1):16940. Epub 2021/08/22. doi: 10.1038/s41598-021-95838-2. PubMed PMID: 34417481; PubMed Central PMCID: PMCPMC8379199.

1018. Uchiyama M, Monsanto RDC, Sancak IG, Park GS, Schachern P, Kobayashi H, et al. Temporal Bone Pathology Secondary to Head Trauma-A Human Temporal Bone Study. Otol Neurotol. 2021;42(8):e1152-e9. Epub 2021/07/06. doi: 10.1097/mao.0000000000003192. PubMed PMID: 34224545.
1019. Vaninetti M, Lim M, Khalaf A, Metzger-Smith V, Flowers M, Kunnel A, et al. fMRI findings in MTBI patients with headaches following rTMS. Sci Rep. 2021;11(1):9573. Epub 2021/05/07. doi: 10.1038/s41598-021-89118-2. PubMed PMID: 33953315; PubMed Central PMCID: PMCPMC8100290.
1020. Wang H, Gao H. Protective effects of repetitive transcranial magnetic stimulation against 6-OHDA-induced Parkinson's symptoms in a mice model: the key role of miR-409-3p/PDHB axis. Int J Neurosci. 2021:1-8. Epub 2021/06/04. doi: 10.1080/00207454.2021.1938037. PubMed PMID: 34078216.
1021. Wang Q, Sun W, Qu Y, Feng C, Wang D, Yin H, et al. Development and Application of Medicine-Engineering Integration in the Rehabilitation of Traumatic Brain Injury. Biomed Res Int. 2021;2021:9962905. Epub 2021/07/09. doi: 10.1155/2021/9962905. PubMed PMID: 34235225; PubMed Central PMCID: PMCPMC8216791 publication of the paper.

1022. Wu X, Li YH, Zhang WJ, Zhang SJ, Gao JF. [Effects of electroacupuncture with different courses on the synaptic structure and synaptic function-related proteins in mice with radiation-induced brain injury]. Sheng Li Xue Bao. 2021;73(6):909-16. Epub 2021/12/29. PubMed PMID: 34961865.
1023. Wu YH, Yu J, Hong LR, Luo BY. Neuromodulatory therapies for patients with prolonged disorders of consciousness. Chin Med J (Engl). 2021;134(7):765-76. Epub 2021/04/03. doi: 10.1097/cm9.0000000000001377. PubMed PMID: 33797467; PubMed Central PMCID: PMCPMC8104219.
1024. Xing X, Jiang R, Lei S, Xu Q, Zhu M, Zhi Y, et al. [Electroacupuncture in the treatment of acute gastrointestinal injury in patients with severe traumatic brain injury: a prospective randomized controlled trial]. Zhonghua Wei Zhong Bing Ji Jiu Yi Xue. 2021;33(1):95-9. Epub 2021/02/11. doi: 10.3760/cma.j.cn121430-20200804-00562. PubMed PMID: 33565409.
1025. Xu H, Wang Y, Luo Y. OTULIN is a new target of EA treatment in the alleviation of brain injury and glial cell activation via suppression of the NF-κB signalling pathway in acute ischaemic stroke rats. Mol Med. 2021;27(1):37. Epub 2021/04/11. doi: 10.1186/s10020-021-00297-0. PubMed PMID: 33836646;

PubMed Central PMCID: PMCPMC8035756.

1026. Yang D, Shin YI, Hong KS. Systemic Review on Transcranial Electrical Stimulation Parameters and EEG/fNIRS Features for Brain Diseases. Front Neurosci. 2021;15:629323. Epub 2021/04/13. doi: 10.3389/fnins.2021.629323. PubMed PMID: 33841079; PubMed Central PMCID: PMCPMC8032955.

1027. Yao L, Baker JL, Schiff ND, Purpura KP, Shoaran M. Predicting task performance from biomarkers of mental fatigue in global brain activity. J Neural Eng. 2021;18(3). Epub 2020/10/28. doi: 10.1088/1741-2552/abc529. PubMed PMID: 33108778; PubMed Central PMCID: PMCPMC8122624.

1028. Yu Y, Yang Y, Gan S, Guo S, Fang J, Wang S, et al. Cerebral Hemodynamic Correlates of Transcutaneous Auricular Vagal Nerve Stimulation in Consciousness Restoration: An Open-Label Pilot Study. Front Neurol. 2021;12:684791. Epub 2021/08/03. doi: 10.3389/fneur.2021.684791. PubMed PMID: 34335449; PubMed Central PMCID: PMCPMC8319239.

1029. Zhang X, Liu B, Li Y, Duan G, Hou J, Wu D. Multi-Target and Multi-Session Transcranial Direct Current Stimulation in Patients With Prolonged Disorders of Consciousness: A Controlled Study. Front Neurosci. 2021;15:641951. Epub 2021/09/28. doi: 10.3389/fnins.2021.641951. PubMed PMID: 34566555; PubMed

Central PMCID: PMCPMC8456025.

1030. Zhang XH, Han P, Zeng YY, Wang YL, Lv HL. The Clinical Effect of Repetitive Transcranial Magnetic Stimulation on the Disturbance of Consciousness in Patients in a Vegetative State. Front Neurosci. 2021;15:647517. Epub 2021/05/18. doi: 10.3389/fnins.2021.647517. PubMed PMID: 33994925; PubMed Central PMCID: PMCPMC8119637.

1031. Zhou L, Huang X, Li H, Guo R, Wang J, Zhang Y, et al. Rehabilitation effect of rTMS combined with cognitive training on cognitive impairment after traumatic brain injury. Am J Transl Res. 2021;13(10):11711-7. Epub 2021/11/18. PubMed PMID: 34786098; PubMed Central PMCID: PMCPMC8581933.

1032. Zhu D, Wang M, Feng F, Nan N, Liu Y, Shi J, et al. Correlation between clinical features and total maturation score by magnetic resonance imaging in very low birth weight premature infants with brain injury. Ann Palliat Med. 2021;10(2):2089-97. Epub 2021/03/18. doi: 10.21037/apm-21-55. PubMed PMID: 33725767.

1033. Aloi D, Jalali R, Tilsley P, Miall RC, Fernández-Espejo D. tDCS modulates effective connectivity during motor command following; a potential therapeutic target for disorders of consciousness. Neuroimage. 2022;247:118781. Epub 2021/12/09.

doi: 10.1016/j.neuroimage.2021.118781. PubMed PMID: 34879252; PubMed Central PMCID: PMCPMC8803542.

1034. Chiang HS, Shakal S, Strain JF, Womack K, Kraut M, Vanneste S, et al. Reversal of unilateral hand movement dysfunction by high definition transcranial direct current stimulation in a patient with chronic traumatic brain injury. Brain Stimul. 2022;15(2):283-5. Epub 2022/01/24. doi: 10.1016/j.brs.2022.01.010. PubMed PMID: 35066190.

1035. Cho YS, Ryu O, Cho K, Kim D, Lim J, Hong SH, et al. The effect of charge-balanced transcutaneous electrical nerve stimulation on rodent facial nerve regeneration. Sci Rep. 2022;12(1):1388. Epub 2022/01/28. doi: 10.1038/s41598-022-05542-y. PubMed PMID: 35082405; PubMed Central PMCID: PMCPMC8791984.

1036. DaSilva AF, Datta A, Swami J, Kim DJ, Patil PG, Bikson M. The Concept, Development, and Application of a Home-Based High-Definition tDCS for Bilateral Motor Cortex Modulation in Migraine and Pain. Front Pain Res (Lausanne). 2022;3:798056. Epub 2022/03/18. doi: 10.3389/fpain.2022.798056. PubMed PMID: 35295794; PubMed Central PMCID: PMCPMC8915734.

1037. du Plessis S, Oni IK, Lapointe AP, Campbell C, Dunn JF, Debert CT. Treatment of Persistent Postconcussion Syndrome With

Repetitive Transcranial Magnetic Stimulation Using Functional Near-Infrared Spectroscopy as a Biomarker of Response: Protocol for a Randomized Controlled Clinical Trial. JMIR Res Protoc. 2022;11(3):e31308. Epub 2022/03/23. doi: 10.2196/31308. PubMed PMID: 35315783.

1038. Eapen BC, Bowles AO, Sall J, Lang AE, Hoppes CW, Stout KC, et al. The management and rehabilitation of post-acute mild traumatic brain injury. Brain Inj. 2022:1-10. Epub 2022/02/15. doi: 10.1080/02699052.2022.2033848. PubMed PMID: 35152817.

1039. Ek-Huchim JP, Árcega-Cabrera F, May-Tec AL, Améndola-Pimenta M, Ceja-Moreno V, Rodríguez-Canul R. Red Blood Cell Cytotoxicity Associated to Heavy Metals and Hydrocarbons Exposure in Flounder Fish from Two Regions of the Gulf of Mexico. Bull Environ Contam Toxicol. 2022;108(1):78-84. Epub 2021/03/25. doi: 10.1007/s00128-021-03176-w. PubMed PMID: 33759008.

1040. Fan J, Zhong Y, Wang H, Aierken N, He R. Repetitive transcranial magnetic stimulation improves consciousness in some patients with disorders of consciousness. Clin Rehabil. 2022:2692155221089455. Epub 2022/03/25. doi: 10.1177/02692155221089455. PubMed PMID: 35322709.

1041. Franke LM, Gitchel GT, Perera RA, Hadimani RL,

Holloway KL, Walker WC. Randomized trial of rTMS in traumatic brain injury: improved subjective neurobehavioral symptoms and increases in EEG delta activity. Brain Inj. 2022:1-10. Epub 2022/02/11. doi: 10.1080/02699052.2022.2033845. PubMed PMID: 35143365.

1042. Hac NEF, Gold DR. Neuro-Visual and Vestibular Manifestations of Concussion and Mild TBI. Curr Neurol Neurosci Rep. 2022. Epub 2022/03/03. doi: 10.1007/s11910-022-01184-9. PubMed PMID: 35235169.

1043. Keswani T, Obeidallah A, Nieves E, Sidoli S, Fazzari M, Taylor T, et al. Pipecolic Acid, a Putative Mediator of the Encephalopathy of Cerebral Malaria and the Experimental Model of Cerebral Malaria. J Infect Dis. 2022;225(4):705-14. Epub 2021/12/22. doi: 10.1093/infdis/jiab615. PubMed PMID: 34932816; PubMed Central PMCID: PMCPMC8844588.

1044. Mabaso SH, Bhana-Nathoo D, Lucas S. An audit of CT brain findings in adults with new-onset seizures in a resource restricted setting in South Africa. SA J Radiol. 2022;26(1):2294. Epub 2022/02/17. doi: 10.4102/sajr.v26i1.2294. PubMed PMID: 35169503; PubMed Central PMCID: PMCPMC8831926.

1045. Manganotti P, Ajčević M, Buoite Stella A. EEG as a marker of brain plasticity in clinical applications. Handb Clin

Neurol. 2022;184:91-104. Epub 2022/01/18. doi: 10.1016/b978-0-12-819410-2.00029-1. PubMed PMID: 35034760.

1046. Mollica A, Dey A, Cairncross M, Silverberg N, Burke MJ. Neuropsychiatric Treatment for Mild Traumatic Brain Injury: Nonpharmacological Approaches. Semin Neurol. 2022. Epub 2022/02/04. doi: 10.1055/s-0041-1742143. PubMed PMID: 35114694.

1047. Mollica A, Greben R, Oriuwa C, Siddiqi SH, Burke MJ. Neuromodulation Treatments for Mild Traumatic Brain Injury and Post-concussive Symptoms. Curr Neurol Neurosci Rep. 2022. Epub 2022/02/18. doi: 10.1007/s11910-022-01183-w. PubMed PMID: 35175543.

1048. Navot-Mintzer D, Ekstein D, Nof E, Fogelman R, Beinart R, Honig A, et al. Rare Cause of Syncope in an Athlete, Matter of Brain and Heart Electricity. Clin J Sport Med. 2022;32(1):e104-e5. Epub 2021/08/28. doi: 10.1097/jsm.0000000000000967. PubMed PMID: 34446650.

1049. Nousia A, Martzoukou M, Liampas I, Siokas V, Bakirtzis C, Nasios G, et al. The Effectiveness of Non-Invasive Brain Stimulation Alone or Combined with Cognitive Training on the Cognitive Performance of Patients With Traumatic Brain Injury: A Systematic Review. Arch Clin Neuropsychol. 2022;37(2):497-512.

Epub 2021/06/23. doi: 10.1093/arclin/acab047. PubMed PMID: 34155517.

1050. Peng Y, Zhao J, Lu X, Dong J, Zhang S, Zhang J, et al. Efficacy of Transcranial Direct Current Stimulation Over Dorsolateral Prefrontal Cortex in Patients With Minimally Conscious State. Front Neurol. 2022;13:821286. Epub 2022/03/08. doi: 10.3389/fneur.2022.821286. PubMed PMID: 35250824; PubMed Central PMCID: PMCPMC8894202.

1051. Qureshi AY, Stevens RD. Mapping the Unconscious Brain: Insights From Advanced Neuroimaging. J Clin Neurophysiol. 2022;39(1):12-21. Epub 2021/09/03. doi: 10.1097/wnp.0000000000000846. PubMed PMID: 34474430.

1052. Ramos-Fresnedo A, Perez-Vega C, Domingo RA, Cheshire WP, Middlebrooks EH, Grewal SS. Motor Cortex Stimulation for Pain: A Narrative Review of Indications, Techniques, and Outcomes. Neuromodulation. 2022;25(2):211-21. Epub 2022/02/08. doi: 10.1016/j.neurom.2021.10.025. PubMed PMID: 35125140.

1053. Srihagulang C, Vongsfak J, Vaniyapong T, Chattipakorn N, Chattipakorn SC. Potential roles of vagus nerve stimulation on traumatic brain injury: Evidence from in vivo and clinical studies. Exp Neurol. 2022;347:113887. Epub 2021/10/09. doi: 10.1016/j.expneurol.2021.113887. PubMed PMID: 34624329.

1054. Sui K, Meneghetti M, Kaur J, Sørensen RJF, Berg RW, Markos C. Adaptive polymer fiber neural device for drug delivery and enlarged illumination angle for neuromodulation. J Neural Eng. 2022;19(1). Epub 2022/02/08. doi: 10.1088/1741-2552/ac5267. PubMed PMID: 35130533.

1055. Tiotiu A, Badi Y, Kermani NZ, Sanak M, Kolmert J, Wheelock CE, et al. Association of Differential Mast Cell Activation with Granulocytic Inflammation in Severe Asthma. Am J Respir Crit Care Med. 2022;205(4):397-411. Epub 2021/11/24. doi: 10.1164/rccm.202102-0355OC. PubMed PMID: 34813381.

1056. Wang Y, Niu Z, Xia X, Bai Y, Liang Z, He J, et al. Application of Fast Perturbational Complexity Index to the Diagnosis and Prognosis for Disorders of Consciousness. IEEE Trans Neural Syst Rehabil Eng. 2022;30:509-18. Epub 2022/02/26. doi: 10.1109/tnsre.2022.3154772. PubMed PMID: 35213312.

1057. Wong JK, Deuschl G, Wolke R, Bergman H, Muthuraman M, Groppa S, et al. Proceedings of the Ninth Annual Deep Brain Stimulation Think Tank: Advances in Cutting Edge Technologies, Artificial Intelligence, Neuromodulation, Neuroethics, Pain, Interventional Psychiatry, Epilepsy, and Traumatic Brain Injury. Front Hum Neurosci. 2022;16:813387. Epub 2022/03/22. doi: 10.3389/fnhum.2022.813387. PubMed PMID: 35308605; PubMed

Central PMCID: PMCPMC8931265.

1058. Wu M, Wu Y, Yu Y, Gao J, Meng F, He F, et al. Corrigendum to: "Effects of theta burst stimulation of the left dorsolateral prefrontal cortex in disorders of consciousness". Brain Stimul. 2022;15(2):426. Epub 2022/02/15. doi: 10.1016/j.brs.2022.02.001. PubMed PMID: 35158110.

1059. Yang S, Kwak SG, Choi GS, Chang MC. Short-term Effect of Repetitive Transcranial Magnetic Stimulation on Diabetic Peripheral Neuropathic Pain. Pain Physician. 2022;25(2):E203-e9. Epub 2022/03/25. PubMed PMID: 35322973.

1060. Zhang Y, Chen W, Zhang T, Du J, Li R, Huo R, et al. P300 correlates with tDCS response in minimally conscious state patients. Neurosci Lett. 2022;774:136534. Epub 2022/02/20. doi: 10.1016/j.neulet.2022.136534. PubMed PMID: 35181480.

(2) Embase （812 studies）

1. Winter A. Coma and spinal cord dysfunction: Treatment with naloxone, hyperbaric oxygen, transcutaneous nerve stimulation, and electrophrenic pacemaker. Journal of Neurological and Orthopaedic Medicine and Surgery. 1991;12(2):124-8.

2. Pizzamiglio L, Vallar G, Magnotti L. Transcutaneous electrical stimulation of the neck muscles and hemineglect rehabilitation. Restorative Neurology and Neuroscience. 1996;10(4):197-203. doi: 10.3233/rnn-1996-10402.

3. Bolton CF. Summary: Electrophysiology in the ICU. Canadian Journal of Neurological Sciences. 1998;25(SUPPL. 1):S43-S4. doi: 10.1017/s0317167100034739.

4. Philips M. Brainstem origins of the N18 component of the somatosensory evoked response. Skull Base Surgery. 1998;8(3):133-40. doi: 10.1055/s-2008-1058572.

5. Hasselmo ME. Neuromodulation: Acetylcholine and memory consolidation. Trends in Cognitive Sciences. 1999;3(9):351-9. doi: 10.1016/S1364-6613(99)01365-0.

6. Maini S, Deogaonkar SC. Transdermal electrical stimulation in sensorineural tinnitus. Indian Journal of Otolaryngology and Head and Neck Surgery. 1999;51(2):37-9. doi: 10.1007/BF02997987.

7. Mayr J, Fechter M, Grechenig W. Functional electrical stimulation (FES) for treatment of head injury sequelae in children. Acta Chirurgica Austriaca. 1999;31(3):167-9. doi: 10.1007/BF02619996.

8. Ravishankar M, Parthasarathy S, Saravanan P. Labour analgesia. Journal of Anaesthesiology Clinical Pharmacology. 1999;15(3):225-52.

9. Hallett M. Brain plasticity and recovery from hemiplegia. Journal of Medical Speech-Language Pathology. 2001;9(2):107-15.

10. Miall C. Stimulating times for TMS. Trends in Cognitive Sciences. 2001;5(8):329. doi: 10.1016/s1364-6613(00)01737-x.

11. Block F. Treatment of neuropathic pain. Therapiewoche. 2002;18(9):416-22.

12. Post RM, Speer AM, Obrocea GV, Leverich GS. Acute and prophylactic effects of anticonvulsants in bipolar depression. Clinical Neuroscience Research. 2002;2(3-4):228-51. doi: 10.1016/S1566-2772(02)00047-6.

13. Canavero S, Bonicalzi V. Neuromodulation for central pain. Expert Review of Neurotherapeutics. 2003;3(5):591-607. PubMed Central PMCID: PMCBertek(United States)

Elan(Ireland)

Novartis(United States).

14. Feketova S. Central pain. Bolest. 2003;6(4):212-7.

15. Halligan PW, Fink GR, Marshall JC, Vallar G. Spatial cognition: Evidence from visual neglect. Trends in Cognitive Sciences. 2003;7(3):125-33. doi: 10.1016/S1364-6613(03)00032-9.

16. Koehler J. Brainstem diagnosis via somatosensory-evoked potentials following stimulation of the median nerve. Klinische Neurophysiologie. 2003;34(1):2-7. doi: 10.1055/s-2003-37745.

17. Owen AM. HERA today, gone tomorrow? Trends in Cognitive Sciences. 2003;7(9):383-4. doi: 10.1016/S1364-6613(03)00195-5.

18. Parsons LM. Superior parietal cortices and varieties of mental rotation. Trends in Cognitive Sciences. 2003;7(12):515-7. doi: 10.1016/j.tics.2003.10.002.

19. Tyrer S, Lievesley A. Pain following traumatic brain injury: Assessment and management. Neuropsychological Rehabilitation. 2003;13(1-2):189-210. doi: 10.1080/09602010244000381.

20. Urban PP. Transcranial magnetic stimulation (TMS) in brainstem lesions and lesions of the peripheral cranial nerves. Klinische Neurophysiologie. 2003;34(1):21-31. doi: 10.1055/s-2003-37749.

21. Wegner DM. The mind's best trick: How we experience conscious will. Trends in Cognitive Sciences. 2003;7(2):65-9. doi: 10.1016/S1364-6613(03)00002-0.

22. Williams WH, Evans JJ. Brain injury and emotion: An overview to a special issue on biopsychosocial approaches in neurorehabilitation. Neuropsychological Rehabilitation. 2003;13(1-2):1-11.

23. Byblow W, Carson R. Sensorimotor coordination: Behavioural modes and neural mechanisms. Human Movement Science. 2004;23(3-4 SPE. ISS.):235-8. doi: 10.1016/j.humov.2004.08.005.

24. Liepert J. Relevance of TMS for investigations in rehabilitation. Neurologie und Rehabilitation. 2004;10(5):227-32.

25. Pageler L. Diagnosis and treatment of trigeminal autonomic cephalgias. Notfall und Hausarztmedizin. 2004;30(9):418-23. doi: 10.1055/s-2004-837354.

26. Pilipenko P, Ierusalimsky A, Vostrikova E, Feigin V. Siberia: Novosibirsk's Neurological School. Practical Neurology. 2004;4(4):248-51. doi: 10.1111/j.1474-7766.2004.12-234.x.

27. Left and right parietal lobes: Different roles in mental imagery and neglect. Neuroscientist. 2005;11(4):271. doi: 10.1177/107385840501100403.

28. Fregni F, Pascual-Leone A, Valente KDR. A therapeutic window for transcranial magnetic stimulation in refractory epilepsy. Journal of Epilepsy and Clinical Neurophysiology. 2005;11(4):177-81.

29. He XK, You CJ, Wang YF, Ai ZB. Effects of low-frequency repetitive transcranial magnetic stimulation at different intension on praxiology of Parkinson rats. Chinese Journal of Clinical Rehabilitation. 2005;9(41):11-3.
30. Knight CA. Applications of the H reflex and transcranial magnetic stimulation. Athletic Therapy Today. 2005;10(3):22-4. doi: 10.1123/att.10.3.22.
31. Nitsche MA, Liebetanz D, Paulus W, Tergau F. Pharmacological characterisation and modulation of neuroplasticity in humans. Current Neuropharmacology. 2005;3(3):217-29. doi: 10.2174/1570159054368268.
32. Singh AN. Multidisciplinary management of chronic pain. International Medical Journal. 2005;12(2):111-6.
33. Tyrer S, Davis E. Central pain states: Etiology and management in rehabilitation. Critical Reviews in Physical and Rehabilitation Medicine. 2005;17(2):131-62. doi: 10.1615/CritRevPhysRehabilMed.v17.i2.30.
34. Chokron S. Contributions of functional neuroimaging to clinical and cognitive neuropsychology: A selective review. Current Medical Imaging Reviews. 2006;2(1):59-69. doi: 10.2174/157340506775541703.
35. MacDonald DB. Safety of transcranial, cortical and

subcortical electrical stimulation using different neurophysiological techniques. Rivista Medica. 2006;12(1-2):39-43.

36. Platz T, Liepert J, Seitz RJ, Bütefisch CM, Müller K, Suchan B, et al. Motor system recovery after brain damage - Mechanisms of recovery and development of restorative therapy - Initiatives and results from the rehabilitation subnet of the German competence net stroke. Nervenheilkunde. 2006;25(11):933-40. doi: 10.1055/s-0038-1626802.

37. Abo-Krysha N, Farouk AA. Utility of clinical assessment and electrophysiological study of the respiratory impairment in Egyptian patients with multiple sclerosis. Journal of Neurological Sciences. 2007;24(4):296-306.

38. Fujiki M, Hikawa T, Abe T, Anan M, Sugita K, Kobayashi H. Navigated brain stimulation for preoperative anatomic and functional identification of impaired motor cortex in a patient with meningioma. Neurosurgery Quarterly. 2007;17(1):33-9. doi: 10.1097/WNQ.0b013e3180329833.

39. Haddadan K, Krames ES. Technique that better localizes the dura mater during intrathecal catheterization should reduce inadvertent multiple dural punctures and reduce complications. Neuromodulation. 2007;10(2):164-6. doi: 10.1111/j.1525-1403.2007.00105.x.

40. Keenan JP, Gorman J. The causal role of the right hemisphere in self-awareness: It is the brain that is selective. Cortex. 2007;43(8):1074-82. doi: 10.1016/S0010-9452(08)70705-6.

41. Khouri AA, El Houni A, Afandi B. A complicated case of prolactinoma. Emirates Medical Journal. 2007;25(2):241-3.

42. Lamme VAF. Sue Ned Block!: Making a better case for P-consciousness. Behavioral and Brain Sciences. 2007;30(5-6):511-2. doi: 10.1017/S0140525X07002920.

43. Nersesyan H, Slavin KV. Current aproach to cancer pain management: Availability and implications of different treatment options. Therapeutics and Clinical Risk Management. 2007;3(3):381-400.

44. Ng YS. Clinical features and rehabilitation of strokes in the less common vascular territories: Anterior and posterior cerebral artery strokes. Critical Reviews in Physical and Rehabilitation Medicine. 2007;19(4):295-329. doi: 10.1615/CritRevPhysRehabilMed.v19.i4.30.

45. Sasso A. The value of somatosensory evoked potentials in prediction of neurodevelopmental outcome. Paediatria Croatica, Supplement. 2007;51(1):130-4.

46. Staudt M. (Re-) organization of sensorimotor and language functions after periventricular brain lesions. Journal fur Neurologie,

Neurochirurgie und Psychiatrie. 2007;8(4):7-9.

47. Wiedemann C, Whalley K, Welberg L. From the editors. Nature Reviews Neuroscience. 2007;8(7):489. doi: 10.1038/nrn2184.

48. Allred RP, Jones TA. Experience: A double-edged sword for restorative neural plasticity after brain damage. Future Neurology. 2008;3(2):189-98. doi: 10.2217/14796708.3.2.189.

49. Andreani JCM, Guma C. New animal model to mimic spastic cerebral palsy: The brain-damaged pig preparation. Neuromodulation. 2008;11(3):196-201. doi: 10.1111/j.1525-1403.2008.00166.x.

50. Andreani JCM, Guma C. Lateral cord stimulation decreases spastic electromyographic spreading: Responses in a brain-damaged pig preparation. Neuromodulation. 2008;11(3):202-7. doi: 10.1111/j.1525-1403.2008.00167.x.

51. Flanagan SR, Cantor JB, Ashman TA. Traumatic brain injury: Future assessment tools and treatment prospects. Neuropsychiatric Disease and Treatment. 2008;4(5):877-92. doi: 10.2147/ndt.s1985.

52. Herraiz C. Assessing the cause of tinnitus for therapeutic options. Expert Opinion on Medical Diagnostics. 2008;2(10):1183-96. doi: 10.1517/17530059.2.10.1183.

53. Lenzi D, Raz E, Pantano P. FMRI and multiple sclerosis.

Current Medical Imaging Reviews. 2008;4(3):163-9. doi: 10.2174/157340508785294507.

54. Sagar SM, Wong RK. Chinese medicine and biomodulation in cancer patients - Part two. Current Oncology. 2008;15(2):8-30. doi: 10.3747/co.v15i2.238.

55. Akhtar J, Carlson JN, Abesamis MG, Katz KD. Somatosensory evoked potentials and brain-stem auditory evoked potentials in coma due venlafaxine overdose. Clinical Toxicology. 2009;47(5):453. doi: 10.1080/15563650902952273.

56. Baseler HA, Gouws A, Morland AB. The organization of the visual cortex in patients with scotomata resulting from lesions of the central retina. Neuro-Ophthalmology. 2009;33(3):149-57. doi: 10.1080/01658100903050053.

57. Braet W, Humphreys GW. The role of reentrant processes in feature binding: Evidence from neuropsychology and TMS on late onset illusory conjunctions. Visual Cognition. 2009;17(1-2):25-47. doi: 10.1080/13506280802193318.

58. Braun C, Demarchi G, Papadelis C. Cortical reorganization after damage to the central nervous system. Neuro-Ophthalmology. 2009;33(3):142-8. doi: 10.1080/01658100902825521.

59. Castro G, Ramirez A, Bastidas J, Godinez N. Transcranial magnetic stimulation in neuropathic pain and its potential use as

screening test in patients candidates to motor cortex stimulation. Pain Practice. 2009;9:45. doi: 10.1111/j.1533-2500.2009.00266.x.

60. John Y, Mark N, Edmonds M, Badawy R, Macdonell R. Transcranial magnetic stimulation as a diagnostic tool in first seizure patients. Journal of Clinical Neuroscience. 2009;16(11):1523-4. doi: 10.1016/j.jocn.2009.07.029.

61. Kljajević V. fMRI in language recovery after stroke. Acta Facultatis Medicae Naissensis. 2009;26(4):217-23.

62. Lapitskaya N, Gosseries O, Pasqua VD, De Noordhout AM, Nielsen JF, Moonen G, et al. Reduced short latency afferent inhibition in severe brain injury patients with impaired consciousness. Journal of Neurology. 2009;256:S20. doi: 10.1007/s00415-009-5161-z.

63. Livingston SC, Hertel JN, Saliba EN, Barth JT, Goodkin HP, Ingersoll CD. Motor-evoked potential amplitudes are correlated with prior number of concussions in the acutely concussed collegiate athlete. Journal of Head Trauma Rehabilitation. 2009;24(5):400-1. doi: 10.1097/HTR.0b013e3181bd6769.

64. Moerk SK, Feldbæk J, Lapitskaya N. Cortical excitability in patients with brain damage after anoxia. European Journal of Neurology. 2009;16(S3):317. doi: 10.1111/j.1468-1331.2009.02821.x.

65. Monier A, Pelc K, Willems A, Dachy B, Guissard G, Dan B. Paediatric brainstem stroke: A rare life-threatening condition. European Journal of Paediatric Neurology. 2009;13:S96. doi: 10.1016/S1090-3798(09)70300-9.

66. Nowak DA. Brain stimulation in the rehabilitation of impaired hand function following stroke. Aktuelle Neurologie. 2009;36(8):397-411. doi: 10.1055/s-0029-1220365.

67. Park S, Ko M, Seo J. Motor reorganisation with transcranial stimulation of motor cortex in children with hemiplegic cerebral palsy. Developmental Medicine and Child Neurology. 2009;51:57. doi: 10.1111/j.1469-8749.2009.03452-2.x.

68. Peyrot M, Rubin RR. Patient reported outcomes in adults with type 2 diabetes using mealtime AFRESA™ (Inhaled technosphere® insulin) or Metformin+Secretagogue or both. Diabetes. 2009;58.

69. Rasche D, Tronnier VM. Invasive treatment of chronic neuropathic pain syndromes: Epidural stimulation of the motor cortex. Journal of Pain Management. 2009;2(3):315-26.

70. Rizvi SJ, Kennedy SH, McNeely H, Giacobbe P, Mayberg HS, Lozano AM. Functional outcome after 12 months of deep brain stimulation for treatment resistant major depressive disorder. European Neuropsychopharmacology. 2009;19:S388-S9. doi:

10.1016/S0924-977X(09)70598-X.

71. Struppler A, Angerer B, Gebhard B. Repetitive peripheral magnetic stimulation (RPMS) as a method for the rehabilitation of sensorimotor deficits of hand and arm following cerebral lesions. Neurologie und Rehabilitation. 2009;15(1):28-38.

72. Vollmer B. Investigation of neural correlates of cerebral palsy. Developmental Medicine and Child Neurology. 2009;51:11. doi: 10.1111/j.1469-8749.2009.03328-1.x.

73. Vollmer B, Holmström L, Lennartsson F, Tedroff K, Forssberg H, Persson J, et al. Fractional anisotropy in the cortico-spinal tract, motor projection patterns, and hand motor outcome in children with unilateral cerebral palsy (CP) - Preliminary report. Developmental Medicine and Child Neurology. 2009;51:78-9. doi: 10.1111/j.1469-8749.2008.03237.x.

74. Watts P, Bowden N, Farewell D, Maguire S, Kemp A. Retinal hemorrhages defining the probability of inflicted brain injury: A systematic review. Journal of AAPOS. 2009;13(1):e30-e1. doi: 10.1016/j.jaapos.2008.12.092.

75. Selected Abstracts of Conference Presentations at the 2010 International Society for Neurofeedback and Research, ISNR, 18th Annual Conference. Journal of Neurotherapy. 2010;14(4).

76. Society Proceedings: 9th Congress of Clinical

Neurophysiology Society of Serbia and Montenegro with International Participation. Clinical Neurophysiology. 2010;121(4).

77. Andrade P, Carrillo-Ruiz JD, Ramírez Y, Jiménez F. Effects of thalamic reticular nucleus electrical stimulation in rats in a t-maze perseverative behavior model induced by 8-OH-DPAT. Neuromodulation. 2010;13(1):2-9. doi: 10.1111/j.1525-1403.2009.00242.x.

78. Belverud S, Mogilner A, Schulder M. Intrathecal drug delivery by implanted pumps. 2010. p. 137-53.

79. Cakar E, Isilak Z, Kiralp MZ, Akyuz G. Low frequency rTMS is a safe method for poststroke patients who had atrial fibrillation: A case report. Brain Injury. 2010;24(3):433. doi: 10.3109/02699051003648227.

80. Camprodon J, Sack A, Pascual-Leone A. Developing strategies for therapeutic neuromodulation: Simultaneous transcranial magnetic stimulation and functional magnetic resonance imaging to study the mechanisms of hemispatial neglect recovery. Neuropsychopharmacology. 2010;35:S272. doi: 10.1038/npp.2010.217.

81. Chang P, Levy RM. High lateral cervical spinal cord stimulation (SCS) for neuropathic facial pain: Report of 10 cases. Neurosurgery. 2010;67(2):550. doi:

10.1227/01.NEU.0000387015.37565.43.

82. Cohen L. Physiological substrates underlying recovery of motor function after stroke. Clinical Neurophysiology. 2010;121:S48. doi: 10.1016/S1388-2457(10)60202-7.

83. Cooper E, Cooper B. Right median nerve stimulation for coma treatment. Brain Injury. 2010;24(3):223-4. doi: 10.3109/02699051003648227.

84. De Fina P, Thompson J, Fellus J, Moser R, Schatz P, Eller M, et al. EEG correlates of improved outcomes from SDOC using a novel treatment protocol. Journal of Neurotherapy. 2010;14(4):355-7. doi: 10.1080/10874208.2010.52335.

85. Derakhshan I, Reesman S. A reappraisal of the kernohan and woltman's article on ipsilateral pyramidal signs: New insight denies role of herniation in favor of deafferentation of the minor hemisphere. Brain Injury. 2010;24(3):118-9. doi: 10.3109/02699051003648227.

86. Dinomais M, Gröschel S, Wilke M, Staudt M, Krägeloh-Mann I. Somatosensory system in congenital hemiparesis: Clinical assessment and resting state functional connectivity MRI analysis. Neuropediatrics. 2010;41(2). doi: 10.1055/s-0030-1265521.

87. Fountoulakis KN. The emerging modern face of mood disorders: A didactic editorial with a detailed presentation of data

and definitions. Annals of General Psychiatry. 2010;9. doi: 10.1186/1744-859X-9-14.

88. Gamé X, Khan S, Panicker J, Hamid R, Elneil S, Fowler C. 2-stage sacral neuromodulation for treating chronic urinary retention in women: Outcome and response prognostic factors. Neurourology and Urodynamics. 2010;29(6):1081-3. doi: 10.1002/nau.20973.

89. Gao G, Jiang J, Cooper E. Traumatic coma: What to do after we made it. Brain Injury. 2010;24(3):153. doi: 10.3109/02699051003648227.

90. Garcia-Larrea L. Motor cortex stimulation (MCS) for neuropathic pain: Can we proceed from phenomenology to mechanisms? European Journal of Pain Supplements. 2010;4(1):11. doi: 10.1016/S1754-3207(10)70039-0.

91. Hummel F. Mechanisms of stroke recovery: Non-invasive brain stimulation to enhance neuronal plasticity and functional recovery. Clinical Neurophysiology. 2010;121:S49. doi: 10.1016/S1388-2457(10)60204-0.

92. Irina S, Tatyana B, Tatyana I, Vadim I, Tatyana S, Konstantin L. Application of transcranial magnetic stimulation in diagnostics and treatment of patients in vegetative state. Brain Injury. 2010;24(3):316. doi: 10.3109/02699051003648227.

93. Jayasekeran V, Rothwell J, Hamdy S. A neurostimulation study of the human cerebellum in the control of swallowing motor pathways. Gastroenterology. 2010;138(5):S371.

94. Jayasekeran V, Rothwell J, Hamdy S. Facilitation of human cortical swallowing pathways by stimulation of the cerebellum. Gut. 2010;59:A29-A30. doi: 10.1136/gut.2009.208975s.

95. Kimiskidis VK, Kugiumtzis D, Papagiannopoulos SK, Kazis DA, Vasiliadis G, Oikonomidi A, et al. Transcranial Magnetic Stimulation terminates epileptiform discharges in patients with partial epilepsy: A combined EEG-TMS study. Clinical Neurophysiology. 2010;121:S168. doi: 10.1016/S1388-2457(10)60691-8.

96. Laureys S. Brain imaging studies of self processing. Journal of Neurology, Neurosurgery and Psychiatry. 2010;81(10):e12-e3. doi: 10.1136/jnnp.2010.217554.30.

97. Lauritzen M, Wanscher B, Smith T. Predictive value of median nerve SEP and EEG after anoxic brain injury. Clinical Neurophysiology. 2010;121:S186-S7. doi: 10.1016/S1388-2457(10)60766-3.

98. Miyai I. Neural mechanisms underlying functional recovery after stroke. Clinical Neurophysiology. 2010;121:S48-S9. doi: 10.1016/S1388-2457(10)60203-9.

99. Paust HJ, Riedel JH, Turner JE, Peters A, Steinmetz OM, Stahl RAK, et al. IFNγ and IL-17 have contrary effects on Th1-and Th17-cell-attracting chemokines. NDT Plus. 2010;3:iii124.

100. Rees G, Seth AK. The cognitive neuroscience of consciousness. Cognitive Neuroscience. 2010;1(3):153-4. doi: 10.1080/17588928.2010.503602.

101. Rose S, Guzzetta A, Tournier D, Burke S, Sakzewski L, Jackson G, et al. Relationship between brain microstructure and upper limb motor and sensory function in congenital hemiplegia. Developmental Medicine and Child Neurology. 2010;52:32-3. doi: 10.1111/j.1469-8749.2010.03682.x.

102. Rossi S, Ragazzoni A, Pirulli C, Veniero D, Miniussi C, Feurra M, et al. Cortical functional connectivity in the Vegetative State and Minimally Conscious State: An investigation with TMS-Evoked Potentials (TEPs). Clinical Neurophysiology. 2010;121:S170. doi: 10.1016/S1388-2457(10)60696-7.

103. Rossini PM. Neuronal plasticity in human. Clinical Neurophysiology. 2010;121:S1. doi: 10.1016/S1388-2457(10)60004-1.

104. Schambra H, Abe M, Luckenbaugh D, Reis J, Cohen L. Motor skill learning is differentially facilitated by left and right motor cortex (MC) stimulation. Annals of Neurology. 2010;68:S60. doi:

10.1002/ana.22175.
105. Tallus J, Lioumis P, Hämäläinen H, Kähkönen S, Tenovuo O. Mild TBI causes a long-lasting elevation of the transcranial magnetic stimulation motor threshold. Brain Injury. 2010;24(3):177. doi: 10.3109/02699051003648227.
106. Tallus J, Lioumis P, Hämäläinen H, Kähkönen S, Tenovuo O. Differences in TMS-evoked EEG responses between healthy controls and symptomatic and recovered patients with mild TBI. Brain Injury. 2010;24(3):177-8. doi: 10.3109/02699051003648227.
107. Tawfik S, Attia M, Flanagan E S, Kapoor R, Ellard S, Hussain K. Congenital hyperinsulinism in infant with homozygous novel ABCC8 missense mutation G1376R. Hormone Research in Paediatrics. 2010;74:240. doi: 10.1159/000321348.
108. Watt J. Ciliary neurotrophic factor as a treatment for neuroendocrine abnormalities associated with traumatic brain injury. Brain Injury. 2010;24(3):341. doi: 10.3109/02699051003648227.
109. Weber K, Holthausen H, Staudt M. Asymmetrical localisation of the motor cortex after perinatally acquired intracerebral hemorrhage: Volume shift versus neuroplasticity. Neuropediatrics. 2010;41(2). doi: 10.1055/s-0030-1265615.
110. Wei JW, Heeley EL, Wang J, Huang Y, Wong LKS, Li Z, et al.

Comparison of recovery trajectories and prognostic indicators for ischemic and hemorrhagic stroke in china: The chinaquest registry study. Cerebrovascular Diseases. 2010;29:259. doi: 10.1159/000321266.

111. Zeller D, Dang SY, Classen J. LTD-like central motor plasticity is not compromised in multiple sclerosis patients. Klinische Neurophysiologie. 2010;41(1). doi: 10.1055/s-0030-1250978.

112. Annual Meeting of the Swiss Society of Clinical Neurophysiology. Clinical Neurophysiology. 2011;122(4).

113. Al-Jehani HM, Al-Issawi W, Jacques L. Duration of intra-operative stimulation on trial as a predictor of success of spinal cord stimulation for chronic pain syndromes. Canadian Journal of Neurological Sciences. 2011;38(3):S60.

114. Andreani JCM. Lateral cord stimulation. A new tool for spastic syndrome treatment. Neuromodulation. 2011;14(5):470. doi: 10.1111/j.1525-1403.2011.00364.x.

115. Angelakis E. EEG, fMRI, and TDCS/tACS applications for disorders of consciousness and movement disorders. Journal of Neurotherapy. 2011;15(4):409-10. doi: 10.1080/10874208.2011.623098.

116. Ansari AH, Jain S, Kumar U, Mathur R. Effects of transcranial

magnetic stimulation on pain and anxiety in fibromyalgia patients. Indian Journal of Physiology and Pharmacology. 2011;55(5):291.

117. Bagnato S, Boccagni C, Sant'Angelo A, Prestandrea C, Galardi G. Reduced intracortical modulation in patients in vegetative state after a traumatic brain injury. Clinical Neurophysiology. 2011;122:S192-S3. doi: 10.1016/S1388-2457(11)60696-2.

118. Battaglia F, Freedberg D. Augmenting physical therapy through modulation of dorsal premotor-motor connectivity. Archives of Physical Medicine and Rehabilitation. 2011;92(10):1736.

119. Blythe LaGasse A, Knight A. Rhythm and music in rehabilitation: A critical review of current research. Critical Reviews in Physical and Rehabilitation Medicine. 2011;23(1-4):49-67. doi: 10.1615/critrevphysrehabilmed.v23.i1-4.40.

120. Borckardt J, Bikson M, Frohman H, Reeves S, Datta A, Bansal V, et al. A pilot study of the tolerability, safety and effects of high-definition transcranial direct current stimulation (HD-tDCS) on pain perception. Journal of Pain. 2011;12(4):P52. doi: 10.1016/j.jpain.2011.02.212.

121. Bourguignon M, Jousmki V, Opde Beeck M, Van Bogaert P,

Nouali M, Metens T, et al. Novel multimodal functional neuroimaging approach for noninvasive primary sensorimotor cortex mapping. Epilepsia. 2011;52:166-7. doi: 10.1111/j.1528-1167.2011.03207.x.

122. Camprodon JA, Sack A, Pascal-Leone A. Simultaneous TMS and fMRI to study mechanisms of hemispatial neglect recovery and strategies for therapeutic neuromodulation. Journal of Neuropsychiatry and Clinical Neurosciences. 2011;23(2):31.

123. Castellanos NP, Bajo R, Cuesta P, Antoniovillacorta-Atienza J, Paúl N, Garcia-Prieto J, et al. Alteration and reorganization of functional networks: A new perspective in brain injury study. Frontiers in Human Neuroscience. 2011(SEPTEMBER). doi: 10.3389/fnhum.2011.00090.

124. Chawla A, Fang L, Wang K. TBI biomarker UCH-L1 protein monomer and oligomers overexpressed in E.coli as recombinant soluable protein calibrator for human gfap biomarker assay. Journal of Neurotrauma. 2011;28(6):A39-A40. doi: 10.1089/neu.2011.9945.

125. Ciaramella A, De Carolis G. The effectiveness of motor cortex stimulation for neuropathic pain can be predicted by psychiatric comorbidity. Case report. Neuromodulation. 2011;14(4):378. doi: 10.1111/j.1525-1403.2011.00363.x.

126. Cohen LG. Non-invasive brain stimulation in the study of mechanisms and modulation of motor learning. Clinical Neurophysiology. 2011;122:S54-S5. doi: 10.1016/S1388-2457(11)60184-3.

127. Cook G, Garderet L, Van Biezen A, LeBlond V, Rovira M, Bordessoule D, et al. The role of high-dose chemotherapy and autologous stem cell transplantation in patients with poems syndrome: A retrospective study of the MM subcommittee of the chronic leukaemia working party of the EBMT. Bone Marrow Transplantation. 2011;46:S32-S3. doi: 10.1038/bmt.2011.47.

128. Danilov YP, Tyler ME, Kaczmarek KA. Rehabilitation of multiple sclerosis and Parkinson's symptoms using cranial nerve non-invasive neuromodulation, (CN-NINM). Movement Disorders. 2011;26:S3-S4. doi: 10.1002/mds.23764.

129. Deering T, Bernardes A, Veiga A, Gartenlaub O, Goncalves A, Jimenez A, et al. Increase of ventricular pacing demand in patients with suspected and documented atrio-ventricular blocks implanted with dual-chamber pacemaker. Europace. 2011;13. doi: 10.1093/europace/eur229.

130. Dinomais M. Reorganization types of the sensori-motor cortex after unilateral early brain lesion: Case of congenital hemiparesis. Annals of Physical and Rehabilitation Medicine. 2011;54:e197. doi:

10.1016/j.rehab.2011.07.442.

131. Dombovy ML. Introduction: The evolving field of neurorehabilitation. CONTINUUM Lifelong Learning in Neurology. 2011;17(3):443-8. doi: 10.1212/01.CON.0000399065.23826.f0.

132. Dykukha S. Surgical correction of complex anatomical and haemodynamical forms of aortic coarctation with partial left ventricular use of bypass. Interactive Cardiovascular and Thoracic Surgery. 2011;12:S131. doi: 10.1510/icvts.2011.0000S1.

133. Eakin K, Ostergard T, Wu O, Hoffer B, Miller J. Electrophysiological abnormalities after minimal traumatic brain injury. Journal of Neurotrauma. 2011;28(6):A10-A1. doi: 10.1089/neu.2011.9945.

134. Eddy CM, Rickards HE, Cavanna AE. Review: Treatment strategies for tics in Tourette syndrome. Therapeutic Advances in Neurological Disorders. 2011;4(1):25-45. doi: 10.1177/1756285610390261.

135. Eisner W, Bauer R. Loss of effectivity of motor cortex stimulation for treatment of neuropathic pain syndromes. Acta Neurochirurgica. 2011;153(3):715. doi: 10.1007/s00701-010-0919-8.

136. El-Wahab N, Robinson N. Analgesia and anaesthesia in labour.

Obstetrics, Gynaecology and Reproductive Medicine. 2011;21(5):137-41. doi: 10.1016/j.ogrm.2011.02.006.
137. Golby AJ, McLaren Black P. Preface. Neurosurgery Clinics of North America. 2011;22(2):xiii-xiv. doi: 10.1016/j.nec.2011.01.005.
138. Hallböök T, Thordstein M, Kohler S, Lundgren J, Bergstrand S, Pegenius G, et al. A case-report using high precision, navigated transcranial magnetic stimulation. Epilepsy Currents. 2011;11(1).
139. Hsieh TH, Dhamne S, Chen JJ, Pascual-Leone A, Jensen F, Rotenberg A. Loss of cortical inhibition following traumatic brain injury detected by mechanomyography and paired-pulse transcranial magnetic stimulation in unanesthetized rats. Clinical Neurophysiology. 2011;122:S148. doi: 10.1016/S1388-2457(11)60531-2.
140. Jacobson L, Lavidor M. Inhibition and facilitation of cognitive functions. Journal of Molecular Neuroscience. 2011;45:S56. doi: 10.1007/s12031-011-9491-9.
141. Jacobson L, Lavidor M. Activation of inhibition: Diminishing impulsive behavior by direct current stimulation over the inferior frontal gyrus. Clinical Neurophysiology. 2011;122:S122-S3. doi: 10.1016/S1388-2457(11)60435-5.
142. Koffman RL. Downrange acupuncture. Medical Acupuncture.

2011;23(4):215-8. doi: 10.1089/acu.2011.0860.

143. Kugiumtzis D, Kimiskidis VK. Effective connectivity in partial epilepsy: Modulation by transcranial magnetic stimuli. Neuroscience Letters. 2011;500:e9. doi: 10.1016/j.neulet.2011.05.088.

144. Kutluay S. Neural plasticity after stroke and functional recovery. Turkiye Fiziksel Tip ve Rehabilitasyon Dergisi. 2011;57:10.

145. Ladd FVL, Ladd AAB, Passos AO, Mattos KC, Brum PC, Coppi AA. Haemodynamic and design-based stereological assessment of the heart in 3-NP-induced mice. Clinical Genetics. 2011;80:20. doi: 10.1111/j.1399-0004.2011.01737.x.

146. Lemaire JJ, Pontier B, Coste J, Gillart T, Sakka L, Feschet F, et al. Conscious behavior after traumatic brain injury: Anatomo-functional support and therapeutic prospects. Annals of Physical and Rehabilitation Medicine. 2011;54:e213-e4. doi: 10.1016/j.rehab.2011.07.387.

147. McLarnon A. Motility: Magnetic stimulation of the cerebellum could restore swallowing. Nature Reviews Gastroenterology and Hepatology. 2011;8(10):538. doi: 10.1038/nrgastro2011157.

148. Messina G, Cordella R, Dones I, Tringali G, Franzini A.

Improvement of secondary fixed dystonia of the upper limb after chronic extradural motor Cortex stimulation in 10 patients. Neuromodulation. 2011;14(4):358. doi: 10.1111/j.1525-1403.2011.00363.x.
149. Mistry S. Unravelling and manipulating the cerebral control of human swallowing using non-invasive brain imaging modalities. Annals of Physical and Rehabilitation Medicine. 2011;54:e35. doi: 10.1016/j.rehab.2011.07.855.
150. Pirulli C, Ragazzoni A, Veniero D, Rossi S, Feurra M, Cincotta M, et al. Functional cortical connectivity in vegetative state and minimally conscious state: An investigation by transcranial magnetic stimulation-evoked potential. Clinical Neurophysiology. 2011;122:S180. doi: 10.1016/S1388-2457(11)60650-0.
151. Quartarone A. Brain plasticity and mechanisms of neural encoding of skills. Clinical Neurophysiology. 2011;122:S43. doi: 10.1016/S1388-2457(11)60142-9.
152. Rasche D. Neurostimulation of the precentral gyrus for the treatment of chronic neuropathic pain syndromes. Neuromodulation. 2011;14(4):380. doi: 10.1111/j.1525-1403.2011.00363.x.
153. Rasche D, Tronnier V. Invasive pain therapy and clinical

significance of intracranial neuromodulation for chronic pain. European Journal of Pain Supplements. 2011;5(1):96-7. doi: 10.1016/S1754-3207(11)70329-7.

154. Rönnefarth M, Schmidt S, Fleischmann R, Picht T, Brandt SA. Interneuronal assemblies reflect early neural reorganization after operative revascularization. Klinische Neurophysiologie. 2011;42(1). doi: 10.1055/s-0031-1272764.

155. Rosanova M, Gosseries O, Casarotto S, Boly M, Casali AG, Bruno MA, et al. Assessing cortical effective connectivity in patients with disorders of consciousness. Journal of Neurology. 2011;258:S42-S3. doi: 10.1007/s00415-011-6026-9.

156. Rubio-Morell B, Rotenberg A, Herreros O, Monzon J, Exposito S, Pascual-Leone A. Transcranial current direct stimulation (tDCS): Therapeutic challenges for ADHD. European Child and Adolescent Psychiatry. 2011;20:S126. doi: 10.1007/s00787-011-0181-5.

157. Skuban T, Hardenacke K, Woopen C, Kuhn J. Informed consent in deep brain stimulation - ethical considerations in a stress field of pride and prejudice. Frontiers in Integrative Neuroscience. 2011;5. doi: 10.3389/fnint.2011.00007.

158. Stam CJ. Exploring brain connectivity. Clinical Neurophysiology. 2011;122:S52. doi:

10.1016/S1388-2457(11)60175-2.

159. Toba M, Rastelli F, Tchokothe C, Nierat MC, Migliaccio R, Vincent S, et al. Using repetitive Transcranial Magnetic Stimulation (rTMS) for the rehabilitation of visuospatial neglect following right hemisphere strokes: Theoretical background, initial experiences and preliminary data and from the project PHRC regional “NEGLECT”. Annals of Physical and Rehabilitation Medicine. 2011;54:e219. doi: 10.1016/j.rehab.2011.07.404.

160. Urgesi C, Avenanti A. Functional and epiphenomenal modulation of neural activity in body-selective visual areas. Cognitive Neuroscience. 2011;2(3-4):212-4. doi: 10.1080/17588928.2011.604725.

161. Van't Wout M. Game theory and the brain: Neural modulation and lesion studies as a tool to examine interpersonal decision-making. Nervenheilkunde. 2011;30(6):411-8. doi: 10.1055/s-0038-1628371.

162. Westlake KP, Nagarajan SS. Functional connectivity in relation to motor performance and recovery after stroke. Frontiers in Systems Neuroscience. 2011(MARCH 2011). doi: 10.3389/fnsys.2011.00008.

163. Zdibska O, Novikova I. Presentation of metabolic diseases among patients of pediatric clinics. Clinical Biochemistry.

2011;44(7):549. doi: 10.1016/j.clinbiochem.2011.03.125.

164. Zinke-Cerwenka W, Rohn A, Posch U, Beham-Schmid C, Linkesch W, Wölfler A, et al. Allogeneic stem cell transplantation with reduced intensity conditioning in patients with therapy-related myeloid neoplasms. European Surgery - Acta Chirurgica Austriaca. 2011;43:22. doi: 10.1007/s10353-011-0041-z.

165. 58th Congress of the Czech and Slovak Society of Clinical Neurophysiology. Clinical Neurophysiology. 2012;123(3).

166. Abo M, Kakuda W, Watanabe M, Senoo A. Does the combination of intensive speech therapy and therapeutic low-frequency transcranial magnetic stimulation based on functional MRI findings and analysis of type of aphasia improve the language function in poststroke patients with chronic aphasia? Brain Injury. 2012;26(4-5):397-8. doi: 10.3109/02699052.2012.660091.

167. Alagoz A, Gunal Sazak H, Ulus F, Kokulu S, Pehlivanoglu P, Sahin S, et al. The evaluation of two hundred thoracic epidural catheterizations in terms of anesthesia residency grade. Pain Practice. 2012;12:138. doi: 10.1111/j.1533-2500.2011.00528.x.

168. Angelakis E, Korfias S, Andreadis N, Liouta E, Sakas D. Cognitive electrophysiological responses of non-responsive patients before and after transcranial alternating current stimulation.

Stereotactic and Functional Neurosurgery. 2012;90:123. doi: 10.1159/000342226.

169. Angelakis E, Liouta E, Andreadis N, Korfias S, Stranjalis G, Ktonas P, et al. Clinical improvement in non-responsive patients with transcranial direct current stimulation. Stereotactic and Functional Neurosurgery. 2012;90:4. doi: 10.1159/000342226.

170. Bartolommei N, Lattanzi L, Callari A, Cosentino L, Luchini F, Mauri M. Catatonia: A critical review and therapeutic recommendations. Journal of Psychopathology. 2012;18(3):234-46.

171. Belkin A, Lescovets V, Lescovets E, Vikulova D, Voronchychina S, Souslov S. Navigated brain stimulation in diagnosis of minimally conscious state. European Journal of Anaesthesiology. 2012;29:S22-S3. doi: 10.1097/01.EJA.0000412518.25201.32.

172. Bellande B, Winicur Z, Gallo B, Turner M, Saulino M, Heath S, et al. Competency-based curricula in DBS and ITB therapies for movement disorders. Neurology. 2012;78(1). doi: 10.1212/WNL.78.1.

173. Best SR, Pavel DG, Chang YC. Role of high definition brain SPECT in the evaluation of non-convulsive epilepsy co-morbid to mental disorder. Epilepsy and Behavior. 2012;24(2):185. doi: 10.1016/j.yebeh.2011.04.021.

174. Birbaumer N, Liberati G. Motor and spatial congnition in minimally consciouos states. Cognitive Processing. 2012;13(1):S7. doi: 10.1007/s10339-012-0510-8.

175. Boly M. Cerebral connectivity in disorders of consciousness. British Journal of Anaesthesia. 2012;108(2):337P-8P. doi: 10.1093/bja/aer442.

176. Cantone M, Lanza G, Pennisi M, Puglisi V, Vinciguerra L, Ricceri R, et al. Motor cortex excitability in patients with vascular cognitive impairment-no dementia: A follow-up study. European Journal of Neurology. 2012;19:225. doi: 10.1111/j.1468-1331.2012.03888.x.

177. Chew E, Teh I, Ang KK, Phua KS, Zhao L, Chang W, et al. Combined transcranial direct current stimulation and brain-computer interface rehabilitation - A new approach to motor rehabilitation. Brain Injury. 2012;26(4-5):782. doi: 10.3109/02699052.2012.660091.

178. Claßen J. Neuroplasticity in MS (#152). Multiple Sclerosis. 2012;18(5):S9. doi: 10.1177/1352458512444204.

179. Conti A. Comment. Acta Neurochirurgica. 2012;154(6):1104. doi: 10.1007/s00701-012-1326-0.

180. Delnooz CCS, Van De Warrenburg BPC. Current and future medical treatment in primary dystonia. Therapeutic Advances in

Neurological Disorders. 2012;5(4):221-40. doi: 10.1177/1756285612447261.
181. Dinn NA, Houlden DA, Potapova E, Mainprize TG. A comparison of SSEP amplitude recorded from the scalp and brain surface. Canadian Journal of Neurological Sciences. 2012;39(3):S26.
182. Eggermont JJ, Roberts LE. The neuroscience of tinnitus: Understanding abnormal and normal auditory perception. Frontiers in Systems Neuroscience. 2012(JULY 2012). doi: 10.3389/fnsys.2012.00053.
183. Espinoza J, Cortes C, Valenzuela J, Sandoval R, Gómez R, Céspedes P, et al. Respiratory syncytial virus infection in mice reduces spatial learning capacity and long term potentiation. Journal of Immunology. 2012;188(1).
184. Gaede G, Tiede M, Lorenz I, Zimmermann H, Pfueller CF, Dörr J, et al. Safety and efficacy of repetitive deep transcranial magnetic stimulation in multiple sclerosis. Journal of Neurology. 2012;259(1):S190-S1. doi: 10.1007/s00415-012-6524-4.
185. Gall C, Schmidt S, Brandt SA, Fedorov AB, Sabel BA. Non-invasive alternating current stimulation improves subjective visual functioning in optic neuropathy. Brain Injury. 2012;26(4-5):521-2. doi: 10.3109/02699052.2012.660091.

186. Ghaffarieh A, Ghaffarpasand F, Dehghankhalili M, Honarpisheh N, Nirumandi S, Tanideh N. Effect of transcutaneous electrical stimulation on rabbit corneal epithelial cell migration. Cornea. 2012;31(5):559-63. doi: 10.1097/ICO.0b013e31823f8b2a.
187. Gibbons CJ, Young CA. Assessing and managing depression and fatigue in motor neuron disease. Neurodegenerative Disease Management. 2012;2(4):401-9. doi: 10.2217/nmt.12.42.
188. Golaszewski SM, Seidl M, Kunz AB, Caleri F, Lochner P, Cataldo S, et al. Functional involvement of cerebral cortex in patients with sleep-wake disturbances after traumatic brain injury: A TMS study. Neurorehabilitation and Neural Repair. 2012;26(4):427. doi: 10.1177/1545968312447071.
189. Hamdy S. Transcranial magnetic stimulation - A diagnostic and therapeutic tool in swallowing disorders. Neurogastroenterology and Motility. 2012;24:9. doi: 10.1111/j.1365-2982.2012.01995.x.
190. Han TR, Oh BM, Kim JY. Molecular mechanism for cumulative effects of repetitive transcranial magnetic stimulation on neural plasticity in rats. Brain Injury. 2012;26(4-5):598-9. doi: 10.3109/02699052.2012.660091.
191. Heesen M, Veeser M. Analgesia in obstetrics. Geburtshilfe und Frauenheilkunde. 2012;72(7):596-601. doi:

10.1055/s-0031-1298444.

192. Horovitz SG, Horwitz B. Introduction to research topic - Brain connectivity analysis: Investigating brain disorders. Part 2: Original research articles. Frontiers in Systems Neuroscience. 2012(FEBRUARY 2012). doi: 10.3389/fnsys.2012.00004.

193. Horwitz B, Horovitz SG. Introduction to research topic - Brain connectivity analysis: Investigating brain disorders. Part 1: The review articles. Frontiers in Systems Neuroscience. 2012(FEBRUARY 2012). doi: 10.3389/fnsys.2012.00003.

194. Hsieh TH, Hameed MQ, Chen JJ, Wang Y, Amandusson A, Pascual-Leone A, et al. Progressive loss of intracortical inhibition following traumatic brain injury detected by transcranial magnetic stimulation and mechanomyogram in rats. Epilepsy Currents. 2012;12(1).

195. Hummel F. What can we learn from system neurosciences (fMRI, TMS) (#149). Multiple Sclerosis. 2012;18(5):S9. doi: 10.1177/1352458512444204.

196. Isagulyan E, Shabalov V, Salova E, Khitj M. Motor cortex stimulation in the treatment of neurogenic pain syndromes. Stereotactic and Functional Neurosurgery. 2012;90:80. doi: 10.1159/000342226.

197. Izadi A, Lee D, Gurkoff G, Echeverri A, Schwartzkroin P,

Berman R, et al. TBI leads to alterations in hippocampal theta, beta and gamma frequencies during rest and exploration in rats. Journal of Neurotrauma. 2012;29(10):A111. doi: 10.1089/neu.2012.9943.

198. Jadhav T, Cross JH. Surgical approaches to treating epilepsy in children. Current Treatment Options in Neurology. 2012;14(6):620-9. doi: 10.1007/s11940-012-0203-8.

199. James JE. Potential advances in knowledge from cross-fertilization between different fields of caffeine research. Journal of Caffeine Research. 2012;2(1):1-2. doi: 10.1089/jcr.2012.1216.

200. Jasek L, Siger M, Berkowicz T, Selmaj K. Multiparametric MRI analysis of patients with late multiple sclerosis. Neurology. 2012;78(1). doi: 10.1212/WNL.78.1.

201. Ju H, Van Dongen AMJ, Xu JX. A neural network model for motor rehabilitation. Proceedings of Singapore Healthcare. 2012;21:S102. doi: 10.1177/201010581202100S101.

202. Kantak S, Liao WW, Wittenberg GF, Waller SM. Posture-related modulation in motor cortical excitability of proximal and distal upper extremity muscles. Archives of Physical Medicine and Rehabilitation. 2012;93(10):E15-E6. doi: 10.1016/j.apmr.2012.08.038.

203. Kidgell D, Daly R, Tooley G, Lum J, Pearce A. Time-course

changes in motor cortical excitability following low level constant transcranial direct current stimulation. Journal of Science and Medicine in Sport. 2012;15:S119. doi: 10.1016/j.jsams.2012.11.290.

204. Kim HM, Chun MH, Lee SI, Lee SJ, Kim HJ, Kim SB. The effect of repetitive transcranial magnetic stimulation on brain injured patients with dysphagia. Neurorehabilitation and Neural Repair. 2012;26(4):407. doi: 10.1177/1545968312447071.

205. Kiziltan ME, Sevindik MS, Bozluolcay M. Auditory startle reflex in cerebrovascular disease. Clinical Neurophysiology. 2012;123(2):e3. doi: 10.1016/j.clinph.2011.07.011.

206. Kunz AB, Christova M, Gallasch E, Seidl M, Bergmann J, Krenn Y, et al. Modulatory effects on human sensorimotor cortex activation by whole hand vibration stimulation. European Journal of Neurology. 2012;19:221. doi: 10.1111/j.1468-1331.2012.03888.x.

207. Kurata J. Corticocortical-evoked potential monitoring as a tool to investigate anaesthetic-induced unconsciousness: A preliminary report. British Journal of Anaesthesia. 2012;108(2):346P-7P. doi: 10.1093/bja/aer442.

208. Lee DJ, Gurkoff G, Izadi A, Schwartzkroin P, Berman R, Muizelaar J, et al. Medial septal theta frequency stimulation

improves spatial working memory following traumatic brain injury. Journal of Neurosurgery. 2012;117(2):A422.

209. Lee DJ, Izadi A, Gurkoff G, Berman R, Ekstrom A, Schwartzkroin P, et al. Frequency-dependent effects of medial septal stimulation for cognitive improvement following traumatic brain injury. Journal of Neurotrauma. 2012;29(10):A10-A1. doi: 10.1089/neu.2012.9943.

210. Lee YH, Ko A, Kim Y, Yong S. Effect of combined peripheral motor electrical stimulation and transcranial direct current stimulation(TDCS) to finger movement in patients with hemiparesis. Brain Injury. 2012;26(4-5):442-3. doi: 10.3109/02699052.2012.660091.

211. Livingston S. Electrophysiologic evidence for the effects of acute cerebral concussion in a collegiatewomen's soccer player. Journal of Head Trauma Rehabilitation. 2012;27(5):E12-E3. doi: 10.1097/HTR.0b013e3182690d04.

212. McDonald S, Anderson V, Ponsford J, Tate R, Togher L, Morgan A, et al. Moving ahead: A new centre of research excellence in brain recovery, focusing on psychosocial reintegration following traumatic brain injury. Brain Impairment. 2012;13(2):256-70. doi: 10.1017/BrImp.2012.21.

213. Messina G, Franzini A, Dones I, Broggi G. Motor cortex

stimulation for upper limb fixed dystonia. Stereotactic and Functional Neurosurgery. 2012;90:41. doi: 10.1159/000342226.
214. Miyai I, Mihara M, Hattori N, Hatakenaka M, Kawano T, Yagura H. Contribution of brain function analysis to the evolution of neurorehabilitation. Clinical Neurology. 2012;52(11):1174-7. doi: 10.5692/clinicalneurol.52.1174.
215. Moennich D, Troost EGC, Kaanders JHAM, Oyen WJG, Alber M, Thorwarth D. Modelling and simulation of the influence of acute and chronic hypoxia on [18F]Fmiso PET imaging. Radiotherapy and Oncology. 2012;103:S335. doi: 10.1016/S0167-8140(12)71190-8.
216. Mohasseb D, Abou Alfa A, Reda M, Khalil I, El Gafi R. Utility of electrophysiological studies in assessing prognosis and recovery of traumatic spinal cord injuries. Journal of Neurology. 2012;259(1):S185. doi: 10.1007/s00415-012-6524-4.
217. Munoz Marron E, Redolar-Ripoll D, Boixados M, Andreu LL. New approaches to the treatment of neglect: Transcranial magnetic stimulation. Neurorehabilitation and Neural Repair. 2012;26(4):420-1. doi: 10.1177/1545968312447071.
218. Muñoz-Marron E, Robles N, Andreu-Barrachina L, Boixados-Angles M, Redolar-Ripoll D. Brain stimulation techniques: Non-invasive treatments for depression. European

Psychiatry. 2012;27. doi: 10.1016/S0924-9338(12)75545-1.

219. Murray LL. Direct and indirect treatment approaches for addressing short-term or working memory deficits in aphasia. Aphasiology. 2012;26(3-4):317-37. doi: 10.1080/02687038.2011.589894.

220. Niimi M, Kakuda W, Abo M. Brain plasticity and stroke rehabilitation. Tokyo Jikeikai Medical Journal. 2012;127(4):151-67.

221. Nunes KF, Manzano GM, Nobrega JAM, Giuliano LMP. Motor skills were affected by intrauterine somatosensory brain injury that mimics a brachial plexus palsy. Clinical Neurophysiology. 2012;123(6):e22-e3. doi: 10.1016/j.clinph.2011.11.103.

222. Pape TLB. Safety and efficacy of repetitive transcranial magnetic stimulation (rTMS) for two persons in vegetative state. Archives of Physical Medicine and Rehabilitation. 2012;93(10):E15. doi: 10.1016/j.apmr.2012.08.035.

223. Piccione F, Genna C, Dotta S, Fusaro P, Silverio F, Cavinato M. Transcranial direct current stimulation can promote awareness in patients with alterate state of consciousness: A preliminary study. Cognitive Processing. 2012;13(1):S8-S9. doi: 10.1007/s10339-012-0510-8.

224. Pralong E, Diserens K, Vuadens P, Levivier M, Bloch J. Measure of spasticity modulation by cervical epidural stimulation in a case of hemispasticity. Stereotactic and Functional Neurosurgery. 2012;90:143. doi: 10.1159/000342226.
225. Rastelli F, Toba M, Tchokotche C, Vincent S, Pradat-Diehl P, Valero-Cabré A. Repetitive transcranial magnetic stimulation (RTMS) for the rehabilitation of visuospatial neglect following right hemisphere strokes: Details from an ongoing multicenter double blind clinical trial. Journal of Head Trauma Rehabilitation. 2012;27(5):E8. doi: 10.1097/HTR.0b013e3182690d04.
226. Rossetti AO. Hypothermia in refractory status epilepticus. Critical Care. 2012;16.
227. Semashko V, Kistsen V, Evstigneev V. Blink reflex significance at estimation of epilepsy hypersynchronous activity. European Journal of Neurology. 2012;19:258. doi: 10.1111/j.1468-1331.2012.03888.x.
228. Siqueira F, Ibrahim V, Pineda CG, Pohlman D. Rehabilitation outcome for the treatment of trauma-induced trismus: A case report. PM and R. 2012;4(10):S221.
229. Takada K, Ito Y. Transdermal insulin application system with dissolving microneedles. Journal of Diabetes Science and Technology. 2012;6(2):A176-A7.

230. Thibaut A, Ledoux D, Demertzi A, Ropars C, Laureys S, Bruno MA. Improvement of consciousness after transcranial direct current stimulation-a sham-controlled double blind study. Brain Injury. 2012;26(4-5):655. doi: 10.3109/02699052.2012.660091.

231. Vanbellingen T, Bertschi M, Nyffeler T, Cazzoli D, Wiest R, Bassetti C, et al. Theta burst stimulation over left posterior parietal cortex interferes with imitation of gestures. Clinical Neurophysiology. 2012;123(10):e101-e2. doi: 10.1016/j.clinph.2012.03.052.

232. Vesper J, Rieck S, Wille C, Wojtecki L, Kinfe T, Schu S. Chronic motor cortex stimulation (MCS) and predictive value of rtms. Pain Practice. 2012;12:181. doi: 10.1111/j.1533-2500.2011.00528.x.

233. Viganò A, De Pasqua V, Magis D, Auvé M, Sava SL, Di Piero V, et al. Theta burst and quadripulse repetitive transcranial magnetic stimulation (rTMS) may have therapeutic potentials in migraine prevention: A proof-of-concept study in healthy volunteers. European Journal of Neurology. 2012;19:632. doi: 10.1111/j.1468-1331.2012.03889.x.

234. Wilke M, Dechent P, Schmidt-Samoa C. Experimental models for spatial neglect (Studies in human and non-human primates). Neuroforum. 2012;18(1):178-89.

235. Xu WD, Hua XY, Zheng MX, Xu JG, Gu YD. Peripheral nerve crossing treats the spastic upper extremity caused by central neurological injury. Brain Injury. 2012;26(4-5):522. doi: 10.3109/02699052.2012.660091.
236. Yamada SM, Yamada S, Nakaguchi H, Murakami M, Hoya K, Matsuno A, et al. A case of tumefactive multiple sclerosis. Japanese Journal of Neurosurgery. 2012;21(5):427-32.
237. 5th International Congress on Psychopharmacology & International Symposium on Child and Adolescent Psychopharmacology. Bulletin of Clinical Psychopharmacology. 2013;23:S1.
238. North American Brain Injury Society's 11th Annual Conference on Brain Injury, NABIS 2013. Journal of Head Trauma Rehabilitation. 2013;28(5).
239. Abdullah JM. All the vice chancellor's neuroscientists: Unity to achieve success in solving Malaysia's diseases via upgrading clinical services and neuroscience research. Malaysian Journal of Medical Sciences. 2013;20(3):1-5.
240. Akbar MJ, Mehmood S, Ullah S, Avery G, Sedman P, Macfie J. Permanent gastric neuromodulation for drug refractory gastroparesis, and persistent nausea and vomiting. European Surgical Research. 2013;50:63-4. doi: 10.1159/000351978.

241. Akbar MJ, Ullah S, Mehmood S, Sedman P, MacFie J. Gastric neuromodulation for drug resistant gastroparesis, and persistent nausea and vomiting. European Surgical Research. 2013;50(2):199. doi: 10.1159/000351963.

242. Antal A, Chaieb L, Paulus W. Transcranial near-infrared stimulation induces neuroplastic changes in the intact human cortex. Clinical EEG and Neuroscience. 2013;44(4):E30. doi: 10.1177/1550059413507209.

243. Assenza G, Pellegrino G, Tombini M, Tecchio F, Tomasevic L, Di Pino G, et al. Delta waves increase after cortical plasticity induction during wakefulness. Clinical Neurophysiology. 2013;124(10):e71-e2. doi: 10.1016/j.clinph.2013.04.094.

244. Baxter MG. Mediodorsal thalamus and cognition in nonhuman primates. Frontiers in Systems Neuroscience. 2013(JUL). doi: 10.3389/fnsys.2013.00038.

245. Bosco E, Zanatta P, Baldanzi F, Sorbara C. Effects of median nerve stimulation intensity on somatosensory evoked potentials in comatose patients. Clinical Neurophysiology. 2013;124(11):e211. doi: 1016/j.clinph.2013.06.128.

246. Burnstock G. Introduction and perspective, historical note. Frontiers in Cellular Neuroscience. 2013;7(NOV). doi: 10.3389/fncel.2013.00227.

247. Campostrini S, Cavinato M, Piccione F. Transcranial direct current stimulation increases long-range fronto-parietal connections in patients with disorders of consciousness: A preliminary study. Clinical Neurophysiology. 2013;124(11):e210. doi: 10.1016/j.clinph.2013.06.124.
248. Carvalho D, Teixeira S, Lucas M, Yuan TF, Chaves F, Peressutti C, et al. The mirror neuron system in post-stroke rehabilitation. International Archives of Medicine. 2013;6(1). doi: 10.1186/1755-7682-6-41.
249. Casali S, Renzi C, D'Angelo E, Vecchi T, Cattaneo Z. Online rTMS over cerebellar vermis affects motion discrimination in healthy individuals. Clinical Neurophysiology. 2013;124(10):e135. doi: 10.1016/j.clinph.2013.04.227.
250. Celnik P. Using non-invasive brain stimulation to study cerebellar contributions to motor learning. Clinical Neurophysiology. 2013;124(10):e45. doi: 10.1016/j.clinph.2013.04.038.
251. Dargie R, Olma M, Behrens J, Kraft A, Irlbacher K, Fahle M, et al. Serial anodal tDCS over V1 induces long-term effects on colour discrimination in V4 measured in the unimpaired hemifield of patients with occipital stroke. Clinical Neurophysiology. 2013;124(10):e138. doi: 10.1016/j.clinph.2013.04.232.

252. Davis LA, Ku BS, Griffin B, Fields JM. Prevalence of needle phobia in patients with difficult venous access. Academic Emergency Medicine. 2013;20(5):S141. doi: 10.1111/acem.12115.
253. Fan B. Neuropathic pain and neuromodulation techniques. Journal of the Neurological Sciences. 2013;333:e535. doi: 10.1016/j.jns.2013.07.1885.
254. Fasotti L, van Kessel M. Novel insights in the rehabilitation of neglect. Frontiers in Human Neuroscience. 2013(NOV). doi: 10.3389/fnhum.2013.00780.
255. Fink E, Tasker R, Beca J, Bell MJ, Clark RS, Hutchison J, et al. Prevalence of acute critical neurological disease in children: A global epidemiological assessment (PANGEA). Critical Care. 2013;17:S131-S2. doi: 10.1186/cc12284.
256. Frank S, Waschke A, Reichart R, Kalff R. Transcranial magnetic stimulation in neuropathic facial pain-A prospective study of 17 patients. Clinical Neurophysiology. 2013;124(10):e122. doi: 10.1016/j.clinph.2013.04.200.
257. Fricova J, Klirova M, Novak T, Vérebová K, Rokyta R. The advantage of 20 HZ RTMS stimulation in the treatment of orofacial pain. Neuromodulation. 2013;16(5):e169. doi: 10.1111/ner.12106.
258. Georgy M, Padwal J, Georgy B. Spinal cord stimulators in outpatient interventional neuroradiology practice. Neuroradiology.

2013;55(6):790-1. doi: 10.1007/s00234-013-1209-y.
259. Gilmore EJ, Breteler MJM, Hirsch LJ, Zaveri H, Gaspard N. Early quantitative EEG findings correlate with neurological dysfunction, hemodynamics and predict outcome in patients with severe sepsis. Neurocritical Care. 2013;19(1):S5. doi: 10.1007/s12028-013-9895-1.
260. Golbe LI. Diagnosis and management of progressive supranuclear palsy. Neurodegenerative Disease Management. 2013;3(1):81-90. doi: 10.2217/nmt.13.1.
261. Hartmann A, Kalis R, Matthäus L, Weber F, Rommel T. Robot guided positioning of a magnetic coil for rTMS over the cortex in patients with cerebral lesions. Clinical Neurophysiology. 2013;124(10):e173-e4. doi: 10.1016/j.clinph.2013.04.303.
262. Hirabayashi H. Surgical treatment strategy for intractable pain due to the stroke: Application of neuromodulation therapy. Neuromodulation. 2013;16(5):e108. doi: 10.1111/ner.12105.
263. Hoffmann M. The human frontal lobes and frontal network systems: An evolutionary, clinical, and treatment perspective. ISRN Neurology. 2013;1(1). doi: 10.1155/2013/892459.
264. Hsu E, Cohen SP. Postamputation pain: Epidemiology, mechanisms, and treatment. Journal of Pain Research. 2013;6:121-36. doi: 10.2147/JPR.S32299.

265. Hummel F. Non-invasive brain stimulation to support neuroregeneration of the motor system after stroke. Behavioural Neurology. 2013;27(3):325. doi: 10.3233/BEN-139900.

266. Janice Lin CH, Chiang MC. Applying magnetic resonance imaging to structural and functional brain research. Journal of Neuroscience and Neuroengineering. 2013;2(1):29-37. doi: 10.1166/jnsne.2013.1032.

267. Kandel M, Beis JM, Le Ray M, Fraser A, Le Chapelain L, Datié AM, et al. Transcranial direct current stimulation improves function for stroke patients with pure motor neglect: A case report. Annals of Physical and Rehabilitation Medicine. 2013;56:e60-e1. doi: 10.1016/j.rehab.2013.07.059.

268. Kashiwa K, Bennett M, El-Jabali F, Papastergiou G, Waite K. Transcutaneous microcurrent electrical stimulation (MCS) in the therapy of previously untreatable retinal diseases. Investigative Ophthalmology and Visual Science. 2013;54(15).

269. Klinger E, Kadri A, Sorita E, Le Guiet JL, Coignard P, Fuchs MP, et al. Personalized rehabilitation of cognitive functions based on simulated Activities o Daily Living (sADL). Annals of Physical and Rehabilitation Medicine. 2013;56:e335. doi: 10.1016/j.rehab.2013.07.1075.

270. Korfias S, Angelakis E, Stranjalis G, Sakas D. Anatomic

circuits of consciousness. neuromodulation prospects for patients in Coma, PVS or MCS? Neuromodulation. 2013;16(5):e202. doi: 10.1111/ner.12106.

271. Kumar N, Jha S. Repetitive Transcranial Magnetic Stimulation (RTMS) in organic personality and behavioral disorder, secondary to suicide attempt by hanging. Clinical Neurophysiology. 2013;124(10):e165-e6. doi: 10.1016/j.clinph.2013.04.290.

272. Lee D, Shahlaie K, Izadi A, Melnik M, Seidl S, Berman R, et al. Stimulation of medial septum restores hippocampal theta coherence following traumatic brain injury. Journal of Neurotrauma. 2013;30(15):A74-A5. doi: 10.1089/neu.2013.9938.

273. Lee J, Ku J, Han K, Park J, Lee H, Kim KR, et al. rTMS over bilateral inferior parietal cortex induces decrement of spatial sustained attention. Frontiers in Human Neuroscience. 2013(FEB). doi: 10.3389/fnhum.2013.00026.

274. Lesniak M, Polanowska K, Seniów J. Repeated anodal tDCS coupled with cognitive training for patients with severe traumatic brain injury-a pilot RCT. Clinical Neurophysiology. 2013;124(10):e179-e80. doi: 10.1016/j.clinph.2013.04.314.

275. Mall V, Jung NH. Motor cortex: Anatomy, Functions and injury. Brain Research Journal. 2013;6(2):209-20.

276. Marron EM, Redolar-Ripoll D. Transcranial magnetic

stimulation: A new rehabilitation tool for neglect. Behavioural Neurology. 2013;27(3):394. doi: 10.3233/BEN-139900.

277. Meier K, Flink M, Simonsen R, Sørensen JC. The aarhus neuromodulation database. Neuromodulation. 2013;16(5):e211. doi: 10.1111/ner.12106.

278. Mueske N, Ryan D, Van Speybroeck A, Wren T. Fat distribution in children and adolescents with myelomeningocele. Developmental Medicine and Child Neurology. 2013;55:28. doi: 10.1111/dmcn.12247.

279. Müri RM, Cazzoli D, Nef T, Mosimann UP, Nyffeler SHT. Non-invasive brain stimulation in neglect rehabilitation: An update. Frontiers in Human Neuroscience. 2013(MAY). doi: 10.3389/fnhum.2013.00248.

280. Nitsche MA. Timing-dependent effects of brain stimulation on motor learning. Clinical Neurophysiology. 2013;124(10):e43. doi: 10.1016/j.clinph.2013.04.030.

281. Ogura M, Nishibayashi H, Kakishita K, Nakao N. Motor cortex stimulation for freezing of gait. Stereotactic and Functional Neurosurgery. 2013;91:204. doi: 10.1159/000351990.

282. Pernet L, Juchters A, Kerckhofs E. The effectiveness of conservative treatment modalities in right hemisphere stroke patients with hemineglect, measured by neuropsychological and/or

functional tests: A systematic review. Cerebrovascular Diseases. 2013;35:823. doi: 10.1159/000353129.

283. Petrosyan V, Ameerally P. Third molar surgery: Changes in patient demographics over 18 years and the effect of the nice guidelines. International Journal of Oral and Maxillofacial Surgery. 2013;42(10):1241. doi: 10.1016/j.ijom.2013.07.243.

284. Pitzalis S, Spinelli D, Vallar G, Di Russo F. Transcutaneous electrical nerve stimulation effects on neglect: A visual-evoked potential study. Frontiers in Human Neuroscience. 2013(MAR). doi: 10.3389/fnhum.2013.00111.

285. Pradhan A, McGuire B, Charles A. Characterization of a novel model for chronic migraine. Journal of Headache and Pain. 2013;14(1).

286. Rajapakse T, Kirton A. Non-invasive brain stimulation in children: Applications and future directions. Translational Neuroscience. 2013;4(2):217-33. doi: 10.2478/s13380-013-0116-3.

287. Rosanova M. TMS-EEG to measure excitability and connectivity of human cortical circuits. Clinical Neurophysiology. 2013;124(10):e47. doi: 10.1016/j.clinph.2013.04.043.

288. Sarikaya-Seiwert S, Beez T, Stoffels G, Janßen G, Steiger HJ, Hänggi D. O-(2-[18F]fluorethyl)-L-tyrosine PET in the predictive clinical evaluation of primary pediatric brain tumors. Child's

Nervous System. 2013;29(9):1736. doi: 10.1007/s00381-013-2261-y.

289. Seregni F, Fecchio M, Rosanova M, Sarasso S, Pigorini A, Napolitani M, et al. Cortical bistability in pathological loss of consciousness after brain injury: A TMS/EEG study. Clinical Neurophysiology. 2013;124(11):e211. doi: 10.1016/j.clinph.2013.06.127.

290. Shabalov VA, Isagulyan E, Khitj MA. Motor cortex stimulation for the treatment of severe pain. Stereotactic and Functional Neurosurgery. 2013;91:111. doi: 10.1159/000351783.

291. Sharova E, Kopachka, Oknina LB, Chelyapina, Gavrilov VM, Romanov. Changes of mental activity and acoustic evoked potentials (AEP) under the influence of repetitive transcranial magnetic stimulation of premotor cortex on normal subjects and patients after severe head injury. Clinical Neurophysiology. 2013;124(10):e181-e2. doi: 10.1016/j.clinph.2013.04.319.

292. Shkirkova K, Belkin A, Lescovetc V. Functional plasticity of the motor cortical structures demonstrated by Navigated Brain Stimulation in patients with stroke and brain damage. Cerebrovascular Diseases. 2013;35:784. doi: 10.1159/000353129.

293. Skripka-Serry J. The great neuro-pipeline 'brain drain' (and why Big Pharma hasn't given up on CNS disorders). Drug

Discovery World. 2013;14(4):9-16. PubMed Central PMCID: PMCHoffmann La Roche
Merck.
294. Sohtaoglu Sevindik M, Erdemir Kiziltan M, Bozluolcay M. Time related changes in electrophysiologic findings of two patients with pons infarctions: Auditory startle reflex and transcranial magnetic stimulation parameters. Journal of the Neurological Sciences. 2013;333:e640-e1. doi: 10.1016/j.jns.2013.07.2225.
295. Starr SA, Baysinger CL. Inhaled Nitrous Oxide for Labor Analgesia. Anesthesiology Clinics. 2013;31(3):623-34. doi: 10.1016/j.anclin.2013.04.001.
296. Stefani A, Peppe A, Galati S, Bassi SM, D'Angelo V, Pierantozzi M. The serendipity case of the pedunculopontine nucleus low-frequency brain stimulation: Chasing a gait response, finding sleep, and cognition improvement. Frontiers in Neurology. 2013;4 JUN. doi: 10.3389/fneur.2013.00068.
297. Sweet JA, Miller JP. Cognitive deficits after traumatic brain injury are associated with decreased hippocampal bursting and improved with burst stimulation of the fornix. Clinical Neurosurgery. 2013;60:166.
298. Tomasino B, Rumiati RI. Introducing the special topic "The when and why of sensorimotor processes in conceptual knowledge

and abstract concepts". Frontiers in Human Neuroscience. 2013(AUG). doi: 10.3389/fnhum.2013.00498.

299. Vandenberghe R, Gillebert CR. Dissociations between spatial-attentional processes within parietal cortex: Insights from hybrid spatial cueing and change detection paradigms. Frontiers in Human Neuroscience. 2013(JUN). doi: 10.3389/fnhum.2013.00366.

300. Xu G, Lan Y, Zhao J, Huang D, Ding M, He X. Disruption of front oparietal network by low frequency repetitive transcranial magnetic stimulation. Journal of Head Trauma Rehabilitation. 2013;28(5):E32. doi: 10.1097/HTR.0b013e3182a2e849.

301. 2014 American Neuropsychiatric Association Annual Meeting Abstracts, ANPA 2014. Journal of Neuropsychiatry and Clinical Neurosciences. 2014;26(2).

302. Ammann C, Gruart A, Delgado-García JM, Márquez-Ruíz J. Testing thermal epidural effects during transcranial direct current stimulation in alert rabbits. Acta Physiologica. 2014;212:89-90. doi: 10.1111/apha.12388.

303. Auld M, Russo R, Moseley GL, Johnston L. Tactile interventions for children with cerebral palsy: A systematic review. Developmental Medicine and Child Neurology. 2014;56:22. doi: 10.1111/dmcn.12368.

304. Burke E, Dodakian L, See J, Riley JD, McKenzie A, Le V, et al. Different predictors of treatment gains in lacunar and non-lacunar stroke. Stroke. 2014;45.
305. Coslett H, Faseyitan O, Medina J, Iannacone M. Effects of gaze, head and hand location on motor function: Evidence from TMS in normal subjects. Neurology. 2014;82(10).
306. Crescentini C, Aglioti SM, Fabbro F, Urgesi C. Virtual lesions of the inferior parietal cortex induce fast changes of implicit religiousness/spirituality. Cortex. 2014;54(1):1-15. doi: 10.1016/j.cortex.2014.01.023.
307. Deer T. Spinal mapping and neuromodulation of the DRG. Regional Anesthesia and Pain Medicine. 2014;39(5):e35-e6. doi: 10.1097/AAP.0000000000000142.
308. Defina P, Zakrzewski C, Prestigiacomo C, Machado C, Bernad P, Halper J, et al. Re-evaluating brain death: The potential for treatment and recovery after brain injury. Neurology. 2014;82(10).
309. Du Plooy W, Mathibe L. Hospitalization of patients due to traditional medicine poisoning. Basic and Clinical Pharmacology and Toxicology. 2014;115:357.
310. Eisner W, Fiegele T, Sohm F. Possible reason for the loss of effectivity of motor cortex stimulation for treatment of neuropathic pain syndromes. Stereotactic and Functional Neurosurgery.

2014;92:s37. doi: 10.1159/000366073.

311. Ekusheva EV. Ipsilateral motor evoked potentials to transcranial magnetic stimulation in right and left hemisphere stroke. European Journal of Neurology. 2014;21:486.

312. Ekusheva EV. Ipsilateral motor evoked potentials to transcranial magnetic stimulation in right-and left hemisphere stroke. Journal of Neurology. 2014;261:S322. doi: 10.1007/s00415-014-7337-4.

313. Etheredge S, Zasler N. Cefaly neuromodulation for posttraumatic neuralgic headache. Brain Injury. 2014;28(5-6):791. doi: 10.3109/02699052.2014.892379.

314. Fayet G, Pegat B, Kassam R, Magot A, Roualdes V, N'Guyen JP, et al. Quantitative assessment of the cerebello-cortical inhibition by double-pulse transcranial magnetic stimulation in patients with cerebellar alterations. Clinical Neurophysiology. 2014;125:S241.

315. Formisano R, Zasler N. Post-traumatic parkinsonism. Brain Injury. 2014;28(5-6):586. doi: 10.3109/02699052.2014.892379.

316. Fox M, Buckner R, Lozano A, Pascual-Leone A. Connecting invasive and noninvasive brain stimulation. Neurology. 2014;82(10).

317. Frank S, Waschke A, Reichart R. RTMS in treatment of

neuropathic facial pain-a prospective study of 20 patients. Clinical Neurophysiology. 2014;125:S242.

318. Galldiks N, Ruge MI, Hamisch C, Stoffels G, Ceccon G, Rapp M, et al. Differentiation of local recurrent brain metastasis from radiation-induced changes using O-(2-[18F]fluoroethyl)-l-tyrosine PET. Neuro-Oncology. 2014;16:ii16. doi: 10.1093/neuonc/nou174.

319. Goldwasser T, Bressan S, Oakley E, Babl F. Use of sedation in children requiring a CT scan after head injury. EMA - Emergency Medicine Australasia. 2014;26:9-10. doi: 10.1111/1742-6723.12243.

320. Harton B, Wang X, Pape T, Parrish T. EEG power spectrum changes induced by transcranial magnetic stimulation in two vegetative patients. Brain Injury. 2014;28(5-6):772. doi: 10.3109/02699052.2014.892379.

321. Heathcock J, Lo W, Chaudhari A. Endpoint kinematics and hand strength following transcranial magnetic stimulation in children with hemiparesis. Developmental Medicine and Child Neurology. 2014;56:64-5. doi: 10.1111/dmcn.12368.

322. Hwang HS, Yi HJ, Lee SG, Sheen SH, Moon SM, Shin IY. Paroxysmal autonomic instability in traumatic brain injuries at neurosurgical intensive care unit. Journal of Neurotrauma. 2014;31(5):A64. doi: 10.1089/neu.2014.9937.

323. Jeon HA. Hierarchical processing in the prefrontal cortex in a variety of cognitive domains. Frontiers in Systems Neuroscience. 2014;8(NOV):1-8. doi: 10.3389/fnsys.2014.00223.
324. Jolly S, Pringle N, Zhuang H, Li H, Richardson WD. Studies of a glial-specific G-protein-coupled receptor GPR37L1. FEBS Journal. 2014;281:382-3. doi: 10.1111/febs.12919.
325. Julkunen P, Koenoenen M, Tarkka I, Maeaettae S, Vanninen R, Jaekaelae P, et al. Changes in cortical reactivity during stroke recovery-a TMS-EEG study. Clinical Neurophysiology. 2014;125:S142-S3.
326. Kakuda W, Abo M, Shimizu M, Sasanuma J, Okamoto T, Hara H, et al. Combination treatment of lowfrequency repetitive transcranial magnetic stimulation and intensive occupational therapy for upper limb hemiparesis after stroke: Clinical results in more than a thousand patients. Brain Injury. 2014;28(5-6):586-7. doi: 10.3109/02699052.2014.892379.
327. Khan B, Hodics T, Hervey N, Kondraske G, Stowe AM, Alexandrakis G. Personalizing non-invasive cortical stimulation for improving motor performance with perturbation transcranial direct current stimulation (ptDCS). Neuromodulation. 2014;17(5):e114. doi: 10.1111/ner.12232.
328. Kirton A, Anderson J, Herrero M, Carsolio L, Mineyko A,

Keess J, et al. Repetitive transcranial magnetic stimulation for perinatal stroke-induced cerebral palsy: Shortterm outcomes from the PLASTIC CHAMPS trial. Developmental Medicine and Child Neurology. 2014;56:84. doi: 10.1111/dmcn.12540.
329. Koganemaru S, Ikeda A, Takahashi R, Fukuyama H, Mima T. Case report: Seizure induction by single-pulse transcranial magnetic stimulation after anodal transcranial direct current stimulation in a stroke patient. Clinical Neurophysiology. 2014;125:S225.
330. Kukurin G. The use of non-invasive brain stimulation in mTBI treatment: A case series. Brain Injury. 2014;28(5-6):526-7. doi: 10.3109/02699052.2014.892379.
331. Leal B, Zenatti L, Rangel R, Chaves J, Carvalho C, Bettencourt A, et al. Adenosinergic system in mesial temporal lobe epilepsy. Epilepsia. 2014;55:80. doi: 10.1111/epi.12675.
332. Liberati G, Hünefeldt T, Belardinelli MO. Questioning the dichotomy between vegetative state and minimally conscious state: A review of the statistical evidence. 2014;8(November):1-14. doi: 10.3389/fnhum.2014.00865.
333. Lippa CF. Head injury and neuropsychological issues. American Journal of Alzheimer's Disease and other Dementias. 2014;29(3):197-8. doi: 10.1177/1533317514531362.

334. Maggioni G, Passaro I, Colombo R, Sterpi I, Giustini A, Pistarini C. Robotic rehabilitation therapy combined with transcranial direct current neuromodulation for the recovery of the upper limb in the subacute phase of stroke. Annals of Physical and Rehabilitation Medicine. 2014;57:e12. doi: 10.1016/j.rehab.2014.03.038.

335. Mahayana IT, Hartono, Tcheang L, Chen CY, Juan CH, Muggleton NG. Posterior parietal cortex and visuospatial control in near and far space. Translational Neuroscience. 2014;5(4):269-74. doi: 10.2478/s13380-014-0229-3.

336. Mall V. Neuromodulation in hand function training. Developmental Medicine and Child Neurology. 2014;56:5-6. doi: 10.1111/dmcn.12482-1.

337. Mazzi C, Mancini F, Savazzi S. Can IPS reach visual awareness without V1? Evidence from TMS in healthy subjects and hemianopic patients. Neuropsychologia. 2014;64:134-44. doi: 10.1016/j.neuropsychologia.2014.09.026.

338. Middleton A, Liuzzo D, Newman-Norlund R, Fritz S, Herter T. Quantifying the feasibility of pairing tDCS with standard physical therapy following neurological insult, using clinical and robotic assessment tools. Neurorehabilitation and Neural Repair. 2014;28(4):NP1-NP2. doi: 10.1177/1545968314529927.

339. Mistry S, Michou E, Vasant D, Ghannouchi I, Verin E, Hamdy S. New techniques-future. Journal of Gastroenterology and Hepatology Research. 2014;3(5):1080-7. doi: 10.6051/j.issn.2224-3992.2014.03.408-14.

340. Momosaki R, Abo M, Watanabe S, Kakuda W, Yamada N, Mochio K. Influence of functional magnetic stimulation on swallowing function. Brain Injury. 2014;28(5-6):562. doi: 10.3109/02699052.2014.892379.

341. Moy SL. Concussion in athletes: From pathophysiology to current guidelines and recommendations. Osteopathic Family Physician. 2014;6(2):8-13. doi: 10.1016/ofp.v6i2.11.

342. Najib U, Horvath JC. Transcranial Magnetic Stimulation (TMS) safety considerations and recommendations. 2014. p. 15-30.

343. Nam HJ, Shin WC, Kwon YN, Lee JS. Cortical excitability difference in postictal state of epileptic seizure and syncope associated with loss of consciousness. Epilepsy Currents. 2014;14:45.

344. Nishijima DK. Tackling the persistent effects of concussion. Science Translational Medicine. 2014;6(255). doi: 10.1126/scitranslmed.3010413.

345. Pape T. rTMS related changes in neural network functional connectivity for subjects in vegetative state after TBI. Archives of

Physical Medicine and Rehabilitation. 2014;95(10):e73-e4.

346. Pearce AJ, Corp DT, Davies CB, Major BP, Maller JJ. Second time around: Corticospinal responses following repeated sports-related concussions within the same season. A transcranial magnetic stimulation study. Journal of Acute Disease. 2014;3(3):186-93. doi: 10.1016/S2221-6189(14)60042-1.

347. Pérez De Los Cobos F, Chatterjee M, Egger V. Long-term plasticity at the olfactory bulb mitral-granule cell synapse. Chemical Senses. 2014;39(1):107. doi: 10.1093/chemse/bjt055.

348. Pistoia F, Sacco S, Sarà M, Franceschini M, Carolei A. Intrathecal Baclofen: Effects on Spasticity, Pain, and Consciousness in Disorders of Consciousness and Locked-in Syndrome. Current Pain and Headache Reports. 2014;19(1). doi: 10.1007/s11916-014-0466-8.

349. Prager J, Deer T, Levy R, Bruel B, Buchser E, Caraway D, et al. Best practices for intrathecal drug delivery for pain. Neuromodulation. 2014;17(4):354-72. doi: 10.1111/ner.12146.

350. Raicevic A. Clinical modeling of neurolysis. Annals of Physical and Rehabilitation Medicine. 2014;57:e22. doi: 10.1016/j.rehab.2014.03.077.

351. Sabel BA, Brösel D, Krohn F, Schlaug C, Rossini PM, Tatlisumak T, et al. REVIS-network: Restoration of vision after

stroke with noninvasive alternating current stimulation to improve visual impairment. Brain Injury. 2014;28(5-6):764. doi: 10.3109/02699052.2014.892379.

352. Shah PP, Norise C, Garcia G, Torres J, Faseyitan O, Hamilton RH. Lesion correlates of transcranial direct current stimulation in chronic nonfluent aphasia. Annals of Neurology. 2014;76:S25-S6. doi: 10.1002/ana.24247.

353. Sidyakina I, Dobrushina O, Degterev D. Navigated fMRI-guided rTMS in rehabilitation of swallowing: A case demonstration. Annals of Physical and Rehabilitation Medicine. 2014;57:e265. doi: 10.1016/j.rehab.2014.03.962.

354. Silvanto J. The use of brain stimulation in the rehabilitation of visual and attentional functions. Clinical Neurophysiology. 2014;125:S5.

355. Singh J, Kaur M, Kulshrestha A, Bajwa SJS. Recent advances in pre-eclampsia management: An anesthesiologist's perspective! Anaesthesia, Pain and Intensive Care. 2014;18(2):209-14.

356. Smorenburg A, Kuo H, Ferre CL, Brandão M, Carmel J, Gordon A, et al. Wired for recovery? How corticospinal system connectivity impacts on the efficacy of bimanual therapy in children with unilateral cerebral palsy. Developmental Medicine and Child Neurology. 2014;56:81-2. doi: 10.1111/dmcn.12540.

357. Su J, Wang W, Sun L, Li T, Kong D, Kang J. Raphe serotonergic neurons modulate genioglossus corticomotor activity in intermittent hypoxic rats. Respiratory Research. 2014;15(1). doi: 10.1186/1465-9921-15-76.

358. Suykerbuyk J, Demeester M. Duloxetine as a treatment of mood instability after acquired brain injury. Brain Injury. 2014;28(5-6):581. doi: 10.3109/02699052.2014.892379.

359. Swick D, Chatham CH. Ten years of inhibition revisited. Frontiers in Human Neuroscience. 2014;8(MAY). doi: 10.3389/fnhum.2014.00329.

360. Taub E, Uswatte G, Mark VW. The functional significance of cortical reorganization and the parallel development of CI therapy. Frontiers in Human Neuroscience. 2014;8(JUNE). doi: 10.3389/fnhum.2014.00396.

361. Tonello F, Francesco C, Tebaldi F, Cantagallo A. High technology for aphasia rehabilitation. Annals of Physical and Rehabilitation Medicine. 2014;57:e144. doi: 10.1016/j.rehab.2014.03.1595.

362. Tracey I, Flower R. The warrior in the machine: Neuroscience goes to war. Nature Reviews Neuroscience. 2014;15(12):825-34. doi: 10.1038/nrn3835.

363. Ulyanova AV, Koch PF, Grovola MR, Harris JP, Cullen DK,

Wolf JA. The development of epileptogenic activity after diffuse brain injury in swine. Journal of Neurotrauma. 2014;31(12):A2. doi: 10.1089/neu.2014.9935.abstracts.

364. Vanzan S, Ferguson H, Bodani M, Sakel M. The effects of caloric vestibular stimulation (CVS) on low awareness states. Archives of Physical Medicine and Rehabilitation. 2014;95(10):e70-e1.

365. Vernet M, Quentin R, Chanes L, Mitsumasu A, Valero-Cabré A. Frontal eye field, where art thou? Anatomy, function, and non-invasive manipulation of frontal regions involved in eye movements and associated cognitive operations. Frontiers in Integrative Neuroscience. 2014;8(AUG). doi: 10.3389/fnint.2014.00066.

366. Vidal AC, Banca P, Gil Pascoal A, Cordeiro G, Sargento-Freitas J, Gouveia A, et al. Acute neuroimaging cortical interhemispheric modulation by motion induced interactions of the upper limbs in acute ischemic stroke. International Journal of Stroke. 2014;9:58. doi: 10.1111/ijs.12375.

367. Wolf JA, Browne K, Grovola M, Cullen DK, Ulyanova AV. Hippocampal circuitry disruptions following diffuse brain injury in swine. Epilepsy Currents. 2014;14:17.

368. Xu G, Lan Y, Zhang Q, He X. Acute modulatory effects of low

frequency repetitive transcranial magnetic stimulation on the frontoparietal network. Brain Injury. 2014;28(5-6):676. doi: 10.3109/02699052.2014.892379.
369. Yamada N, Kakuda W, Kondo T, Shimizu M, Mitani S, Abo M. Consecutive application of continuous theta burst stimulation combined with intensive occupational therapy for upper limb hemiparesis after stroke: A preliminary study. Brain Injury. 2014;28(5-6):620-1. doi: 10.3109/02699052.2014.892379.
370. Zado ES, Pammer M, Marchlinski FE. First report of a shock from a subcutaneous ICD during transcutaneous electrical nerve stimulation (TENS). Heart Rhythm. 2014;11(5):S1. doi: 10.1016/j.hrthm.2014.03.025.
371. Zanetti O. Non-pharmacological intervention for memory decline. Neurobiology of Aging. 2014;35:S26.
372. Abdullah JM, Idris Z, Ahmad AL, Omar H. Transcranial magnetic stimulation (TMS) for neurorehabilitation and clinical application. Brain Stimulation. 2015;8(2):413.
373. Alústiza I, Pujol N, Molero P, Ortuño F. Temporal processing in schizophrenia: Review. Schizophrenia Research: Cognition. 2015;2(4):185-8. doi: 10.1016/j.scog.2015.10.001.
374. Bogdanova Y, Martin P, Ho M, Yee M, Ho V, Knight J, et al. Improved sleep and cognition after transcranial or intranasal,

red/near-infrared led treatment in chronic TBI: Pilot case series. Journal of Head Trauma Rehabilitation. 2015;30(3):E101-E2. doi: 10.1097/HTR.0000000000000150.

375. Bolognini N, Russo C, Vallar G. Crossmodal illusions in neurorehabilitation. Frontiers in Behavioral Neuroscience. 2015;9(AUGUST). doi: 10.3389/fnbeh.2015.00212.

376. Culling B, Dahlgren N, Dillow J, Weinstein A. Neuromodulation for chronic bilateral occipital Neuralgia; a common technique with a not so common outcome. Regional Anesthesia and Pain Medicine. 2015;40(5).

377. Da Silva EU, Nilton Jr LA, Veiga JCE, Silva JMDA, De Souza HC. Use of tDCS as motor cortex stimulation predictor for gait disorders in advanced Parkinson's disease. Movement Disorders. 2015;30:S78. doi: 10.1002/mds.26295.

378. Da Silva EU, Nilton Jr LA, Veiga JCE, Silva JMDA, De Souza HC. Motor cortex stimulation for gait disorders in advanced Parkinson's disease. Movement Disorders. 2015;30:S214-S5. doi: 10.1002/mds.26295.

379. Danilov Y, Verbny Y, Kaczmarek K, Skinner K, Tyler M. Eye movement rehabilitation by CN-ninm intervention: A set of case studies. Journal of Head Trauma Rehabilitation. 2015;30(3):E77. doi: 10.1097/HTR.0000000000000150.

380. Dudakov J, Wertheimer T, Velardi E, Brede C, Young L, Ginsberg M, et al. Endothelial cells promote endogenous thymic regeneration via BMP4 signaling and activation of Foxn1 (HEM7P.224). Journal of Immunology. 2015;194(1).

381. Eghtesadi M, Boudreau G, Leroux E, Fournier Gosselin MP. Peripheral neuromodulation for headache attributed to an upper cervical radiculopathy. Cephalalgia. 2015;35(6):128. doi: 10.1177/0333102415581304.

382. Endisch C, Storm C, Ploner CJ, Leithner C. Somatosensory evoked high-frequency oscillations and prognostication after cardiac arrest. Critical Care. 2015;19:S151. doi: 10.1186/cc14511.

383. Ford JA, Orfila JE, Dietz RM, Yang Y, Haeger S, Herson PS, et al. The mechanistic investigation of neurocognitive dysfunction after sepsis. American Journal of Respiratory and Critical Care Medicine. 2015;191.

384. Fregni F, Nitsche MA, Loo CK, Brunoni AR, Marangolo P, Leite J, et al. Regulatory considerations for the clinical and research use of transcranial direct current stimulation (tDCS): Review and recommendations from an expert panel. Clinical Research and Regulatory Affairs. 2015;32(1):22-35. doi: 10.3109/10601333.2015.980944.

385. Fuente A. The olivocochlear system and protection from

acoustic trauma: A mini literature review. Frontiers in Systems Neuroscience. 2015;9(JUNE). doi: 10.3389/fnsys.2015.00094.
386. Guffens F, Maes A, Van Laere K, Goffin K. Dynamic O-(2-18F-Fluoroethyl)-l-Tyrosine PET improves differentiation of local recurrent brain metastasis from radiation necrosis. Journal of Nuclear Medicine. 2015;56.
387. Hameed MQ, Hsieh TH, Morales-Quezada JL, Goodrich GS, Rotenberg A. Ceftriaxone treatment in a rat post traumatic epilepsy model preserves cortical inhibitory interneuron function and parvalbumin expression. Epilepsy Currents. 2015;15:393.
388. Ille S, Sollmann N, Hauck T, Maurer S, Tanigawa N, Obermueller T, et al. Impairment of non-invasive language mapping by lesion location a fMRI, rTMS, and DCS study. Clinical Neurophysiology. 2015;126(8):e130. doi: 10.1016/j.clinph.2015.04.205.
389. Ille S, Wittig R, Kulchytska N, Sollmann N, Hauck T, Maurer S, et al. Correlation of hemispheric dominance of language examined by rTMS and postoperative course of language function in patients with left-sided Perisylvian brains lesions. Clinical Neurophysiology. 2015;126(8):e129-e30. doi: 10.1016/j.clinph.2015.04.204.
390. Jedlicka S, Ott S, Krammer M, Lumenta CB. The value of

navigated transcranial magnetic stimulation in clinical neurosurgical practice. Brain Stimulation. 2015;8(2):428.

391. Kopachka MM, Sharova EV, Troshina EM, Oknina LB, Romanov AC, Chelyapina MV, et al. Repetitive transcranial magnetic stimulation (RTMS)-induced asymmetric changes in acoustic event-related potentials (AERP) in normal subjects and in patients experiencing posttraumatic unconsciousness states. Asimmetria - Journal of Asymmetry. 2015;9(2):18-29.

392. Ksiazek-Winiarek DJ, Szpakowski P, Glabinski A. Neural Plasticity in Multiple Sclerosis: The Functional and Molecular Background. Neural Plasticity. 2015;2015. doi: 10.1155/2015/307175.

393. Làdavas E, Giulietti S, Avenanti A, Bertini C, Lorenzini E, Quinquinio C, et al. A-tDCS on the ipsilesional parietal cortex boosts the effects of prism adaptation treatment in neglect. Restorative Neurology and Neuroscience. 2015;33(5):647-62. doi: 10.3233/RNN-140464.

394. Lee A, Chang WH, Lee MJ, Kim MS, Kim YH. Dual-mode noninvasive brain stimulation over prefrontal cortices on working memory in stroke patients. Brain Stimulation. 2015;8(2):359.

395. Lefebvre J, Murray MM. Shaping pathological cortical dynamics with high-frequency neurostimulation. BMC

Neuroscience. 2015;16.

396. Liamlahi R, Hagmann C, Beck I, Latal B, Knirsch W. Delayed brain maturation correlates with motor impairment at one year of age in children with severe congenital heart disease. Cardiology in the Young. 2015;25:S27. doi: 10.1017/S1047951115000529.

397. Lõo S, Kuusela L, Valanne L, Vaalto S, Nevalainen P, Lauronen L, et al. Structural MRI, transcranial magnetic stimulation, magnetoencephalography and DTI tractography findings in relation to sensorimotor outcome after perinatal stroke. European Journal of Paediatric Neurology. 2015;19:S9-S10.

398. Morries LD, Cassano P, Henderson TA. Treatments for traumatic brain injury with emphasis on transcranial near-infrared laser phototherapy. Neuropsychiatric Disease and Treatment. 2015;11:2159-75. doi: 10.2147/NDT.S65809.

399. Nantes JC, Zhong J, Holmes SA, Narayanan S, Lapierre Y, Koski L. Intracortical inhibition in multiple sclerosis: Relation to clinical subtype, motor disability, and cortical damage. Multiple Sclerosis. 2015;23(11):223. doi: 10.1177/1352458515602642.

400. Picillo M, Moro E, Edwards M, Di Lazzaro V, Lozano AM, Fasano A. Subdural continuous theta burst stimulation of the motor cortex in essential tremor. Neurological Sciences. 2015;36(2):S190-S1. doi: 10.1007/s10072-015-2346-3.

401. Pistarini C. Robot therapy and tDCS for recovery of movement in stroke patients. Neurorehabilitation and Neural Repair. 2015;29(7):NP4. doi: 10.1177/1545968315589320.
402. Rabau S, Van De Heyning P. The added effect of transcranial direct current stimulation for the treatment of acute tinnitus. Brain Stimulation. 2015;8(2):410.
403. Sanders R, Bruno M, Bonhomme V, Rosanova M, Laureys S, Tononi G, et al. Propofol-induced unresponsiveness is associated with loss of deterministic high density electroencephalogram responses to transcranial magnetic stimulation. Journal of Neurosurgical Anesthesiology. 2015;27(4):447. doi: 10.1097/ANA.0000000000000217.
404. Scalone L, Zucco F, Ciampichini R, Lavano A, Costantini A, De Rose M, et al. Relationship between pain, functional disability and health-related quality of life in patient with failed back surgery syndrome undergoing spinal cord stimulation: Results from the precise study. Value in Health. 2015;18(7):A647.
405. Sharma V, Kaur H, Gupta D. Right median nerve stimulation for improving consciousness: A case series. Indian Journal of Neurotrauma. 2015;12(2):144-8. doi: 10.1055/s-0035-1569473.
406. Singh R, Telleria M, Patin D. Neuromodulation therapy for the treatment of refractory complex regional pain syndrome with

diagonal spread. Journal of Pain. 2015;16(4):S71.
407. Sollmann N, Hauck T, Ille S, Maurer S, Negwer C, Meyer B, et al. Preoperative repetitive navigated transcranial magnetic stimulation for language mapping and outcome improvement in brain tumor patients. Clinical Neurophysiology. 2015;126(8):e124. doi: 10.1016/j.clinph.2015.04.195.
408. Sollmann N, Ille S, Hauck T, Maurer S, Negwer C, Zimmer C, et al. The impact of preoperative language mapping by repetitive navigated transcranial magnetic stimulation on the clinical course of brain tumor patients. BMC Cancer. 2015;15(1). doi: 10.1186/s12885-015-1299-5.
409. Speth J, Frenzel C, Harley T. Transcranial direct current stimulation of areas above the motor cortex (C3) in waking resting state and REM sleep induces motor imagery. Brain Stimulation. 2015;8(2):404-5.
410. Sridhar K. A summary of some of the recently published, seminal papers in Neuroscience. Neurology India. 2015;63(2):250-4. doi: 10.4103/0028-3886.156294.
411. Storm C, Ploner CJ, Leithner C. Amplitudes of cortical somatosensory evoked potentials and outcome prediction after cardiac arrest. Critical Care. 2015;19:S153. doi: 10.1186/cc14516.
412. Urbano Da Silva E, Lara NAJR, Veiga JCE, De Souza HC.

Motor cortex stimlation in parkinsonian patients with refractory freezing of gait and balance disorders responsive to transcranial direct current stimulation. Neurodegenerative Diseases. 2015;15:1962. doi: 10.1159/000381736.
413. Varipapa RJ. Continuous theta burst stimulation (CTBs) applied to the dorsolateral prefrontal cortex modulates late somatosensory evoked potentials (SEP) in normal subjects. Journal of Clinical Neurophysiology. 2015;32(4):395. doi: 10.1097/WNP.0000000000000175.
414. Votta-Velis G. Refresher course: Phantom pain: Treatment algorithms. Regional Anesthesia and Pain Medicine. 2015;40(5):e9-e11. doi: 10.1097/AAP.0000000000000308.
415. Zimerman M, Weide S, Wessel M, Schulz R, Timmermann JE, Bönstrup M, et al. Interactions between primary and secondary motor areas for recovered hand functions after stroke. Clinical Neurophysiology. 2015;126(8):e84-e5. doi: 10.1016/j.clinph.2015.04.115.
416. Cyclops. Canadian Journal of Ophthalmology. 2016;51(3):135. doi: 10.1016/j.jcjo.2016.03.012.
417. Abstracts from the 19th Annual Meeting of the North American Neuromodulation Society, NANS 2015. Neuromodulation. 2016;19(3).

418. Amaro LJA, De Brito VF, De Ribamar Castro Veloso J, Tavares JR, De Sousa FLR, Neto FXP, et al. Deafness post traumatic brain injury. International Archives of Otorhinolaryngology. 2016;20:S26.

419. Avakov V, Shakhova I. Non-invasive brain and kidney cooling in severe multi system brain injury complicated by septic shock. Journal of Cerebral Blood Flow and Metabolism. 2016;36:802-3. doi: 10.1177/0271678X16645239.

420. Babyar S, Santos-Pontelli T, Will-Lemos T, Mazin S, Bikson M, Truong DQ, et al. Center of Pressure Speed Changes with tDCS Versus GVS in Patients with Lateropulsion after Stroke. Brain Stimulation. 2016;9(5):796-8. doi: 10.1016/j.brs.2016.06.053.

421. Boes AD, Stern AP, Bernstein M, Hooker JE, Connor A, Press DZ, et al. H-Coil Repetitive Transcranial Magnetic Stimulation Induced Seizure in an Adult with Major Depression: A Case Report. Brain Stimulation. 2016;9(4):632-3. doi: 10.1016/j.brs.2016.04.013.

422. Chevalier A, Armstrong K, Norwood-Williams C, Gokal R. DC Electroacupuncture Effects on Scars and Sutures of a Patient with Postconcussion Pain. Medical Acupuncture. 2016;28(4):223-9. doi: 10.1089/acu.2016.1188.

423. Cruccu G, Attal N. Neurostimulation in neuropathic pain.

European Journal of Neurology. 2016;23:902. doi: 10.1111/ene.13096.

424. Feldmann LK, Le Prieult F, Felzen V, Thal S, Behl C, Engelhard K, et al. Traumatic brain injury induces changes in synaptic longterm plasticity connected to alterations in protein degradation systems. Acta Physiologica. 2016;216:163.

425. Feng Z, Du Q. Mechanisms responsible for the effect of median nerve electrical stimulation on traumatic brain injuryinduced coma: Orexin-A-mediated N-methyl-D-aspartate receptor subunit NR1 upregulation. Neural Regeneration Research. 2016;11(6):951-6. doi: 10.4103/1673-5374.184494.

426. Flamand V, Smorenburg A, Kuo H, Marneweck M, Ferre C, Bleyenheuft Y, et al. Underpinnings of intracortical motor circuits physiology in children with unilateral cerebral palsy. Developmental Medicine and Child Neurology. 2016;58:70-1. doi: 10.1111/dmcn.51_13224.

427. Fujimoto S, Kon N, Otaka Y, Yamaguchi T, Nakayama T, Kondo K, et al. Transcranial direct current stimulation over the primary and secondary somatosensory cortices transiently improves tactile spatial discrimination in stroke patients. Frontiers in Neuroscience. 2016;10(MAR). doi: 10.3389/fnins.2016.00128.

428. Gaur GS. Concepts of synaptic plasticity, neuroplasticity and

neurogenesis. Indian Journal of Physiology and Pharmacology. 2016;60(5):12.

429. Goyal E, Kadiani A, Marella S, Saldanha D. Interventional psychiatry. history, current status and future directions. Indian Journal of Psychiatry. 2016;58(5):S14-S5.

430. Guo S, Lu X, Zhuo J, Xu S, Gullapalli R, Gandhi D. Water diffusivity changes with varying focused ultrasound exposure levels in rat brains. Journal of Therapeutic Ultrasound. 2016;4(1). doi: 10.1186/s40349-016-0076-5.

431. Ilić TV, Milanović S, Komatina N, Raičević R. Prefrontal transcranial direct current stimulation in patients with disorders of consciousness-a case series study. Clinical Neurophysiology. 2016;127(3):e98. doi: 10.1016/j.clinph.2015.11.330.

432. Ille S, Sollmann N, Ringel F, Meyer B, Krieg SM. Resection of highly language-eloquent brain lesions purely based on rTMS language mapping without awake surgery. Neuro-Oncology. 2016;18:vi191. doi: 10.1093/neuonc/now212.804.

433. Legostaeva LA, Zmeykina EA, Poydasheva AG, Sergeev DV, Sinitsyn DO, Kremneva EI, et al. A new approach to the study of consciousness from the theory of integrated information point of view. Sovremennye Tehnologii v Medicine. 2016;8(4):251-8. doi: 10.17691/stm2016.8.4.30.

434. León D, Rodríguez N, Pelayo R, Martinell M, Berbaneu M, Tormos JM. Treatment of chronic non-fluent aphasia with rTMS combined with intensive speech therapy. Brain Injury. 2016;30(5-6):621. doi: 10.3109/02699052.2016.1162060.

435. Lin N, Antiel R, Waqar L, Hoffman-Craven C, Monos S, D'Agostino J, et al. Brain maturation and neurodevelopmental outcomes in infants with congenital diaphragmatic hernia. Annals of Neurology. 2016;80:S401-S3. doi: 10.1002/pon.4272.

436. Luvizutto GJ, Dos Santos MRL, Rodrigues RT, Fogaroli MO, Braga GP, Bazan SGZ, et al. Relation between anthropometric variables and respiratory muscular strength in the acute phase of stroke. International Journal of Stroke. 2016;11:133. doi: 10.1177/1747493016670567.

437. Mahler DA. A Perspective on Acupuncture Techniques for Relief of Dyspnea in Chronic Obstructive Pulmonary Disease. Medical Acupuncture. 2016;28(1):28-32. doi: 10.1089/acu.2015.1117.

438. Maistreli S, Kourlaba G, Georgopoulos D, Maniadakis N. Budget impact analysis of the incorporation of a computerized temperature management system in the treatment of therapeutic hypothermia in Greece. Value in Health. 2016;19(7):A630-A1.

439. Meola A. Letter to the Editor. Brain Injury.

2016;30(13-14):1515-6. doi: 10.1080/02699052.2016.1199912.

440. Neville IS, Hayashi CY, Rodrigues P, Galhardoni RRG, Brunoni AR, Zaninotto AL, et al. Cortical excitability assessment on traumatic brain injury (10649). Neuromodulation. 2016;19(3):e2. doi: 10.1111/ner.12428.

441. Neville IS, Hayashi CY, Sousa Jr LM, Galhardoni RRG, Moraes TM, Rodrigues P, et al. Application of rTMS on victims of Bifrontal Cerebral Contusions. (10746). Neuromodulation. 2016;19(3):e71. doi: 10.1111/ner.12428.

442. Otal B, Dutta A, Foerster Á, Ripolles O, Kuceyeski A, Miranda PC, et al. Opportunities for guided multichannel non-invasive transcranial current stimulation in poststroke rehabilitation. Frontiers in Neurology. 2016;7(FEB). doi: 10.3389/fneur.2016.00021.

443. Panuccio G, Semprini M, Chiappalone M. Intelligent biohybrid systems for functional brain repair. New Horizons in Translational Medicine. 2016;3(3-4):162-74. doi: 10.1016/j.nhtm.2016.10.001.

444. Pape T, Rosenow J, Herrold A, Harton B, Patil V, Guernon A. Neurobiological model for use of rTMS + amantadine as a treatment to modulate and shape neural repair for persons in states of disordered consciousness. Brain Injury. 2016;30(5-6):615-6. doi:

10.3109/02699052.2016.1162060.

445. Parenti L, Barloscio D, Bartolotta M, Di Rollo A, Sartucci F, Bocci T. Intracortical and interhemispheric changes induced by Transcutaneous spinal Direct Current Stimulation (tsDCS). Clinical Neurophysiology. 2016;127(12):e339. doi: 10.1016/j.clinph.2016.10.077.

446. Rosa MA, Abdo GL, Rosa MO. Efficacy and safety of TMS during eight years in a Brazilian service: Are safety guidelines too strict? Journal of ECT. 2016;32(3):218.

447. Sasso V, Bisicchia E, Latini L, Ghiglieri V, Molinari M, Viscomi M. Repetitive transcranial magnetic stimulation protects neurons form remote degeneration after focal brain injury in rats. Clinical Neuropathology. 2016;35(3):146-7.

448. Scott R, Van-Ruiten H, Baker M, Mitra D, McFarland R, Ramesh V. Explosive, new-onset epilepsia partialis continua in a teenage girl caused by mitochondrial disease-treatment with transcranial direct current electrical stimulation. Developmental Medicine and Child Neurology. 2016;58:3. doi: 10.1111/dmcn.12997.

449. Seeger T, Kirton A, Barlow K. Cortical excitability after mild traumatic brain injury in children. Neurology. 2016;86(16).

450. Sobreira G, Aleixo MA, Moreia C, Oliveira J. Adverse effects

in repetitive transcranial magnetic stimulation-prevention and management. European Psychiatry. 2016;33:S555-S6. doi: 10.1016/j.eurpsy.2016.01.2052.

451. Sorato Bezerra O, Babis Magoulas C, Schuh Teixeira Da Rosa AL, Primo De Carvalho Alves L. Efficacy of low-frequency rTMS on motor recovery after-stroke. European Journal of Neurology. 2016;23:224. doi: 10.1111/ene.13092.

452. Staudt M, Küpper H, Pieper T, Gröschel S, Winkler P, Holthausen H, et al. Prediction of contralateral hand function after hemispherectomy for refractory epilepsies: Experience from 102 pediatric cases. Neuropediatrics. 2016;47. doi: 10.1055/s-0036-1583712.

453. Tanaka J. Editorial. Visual Cognition. 2016;24(1):1. doi: 10.1080/13506285.2016.1213000.

454. Tidoni E, Candidi M. Commentary: Understanding intentions from actions: Direct perception, inference, and the roles of mirror and mentalizing systems. Frontiers in Behavioral Neuroscience. 2016;10(FEB). doi: 10.3389/fnbeh.2016.00013.

455. Vaalto S, Loo S, Mãenpãã H. Lateralization differences in hand and leg motor representations in patients with cerebral palsy. Clinical Neurophysiology. 2016;127(3):e34. doi: 10.1016/j.clinph.2015.11.105.

456. van den Heuvel CNAM, Tijssen MAJ, van de Warrenburg BPC, Delnooz CCS. The Symptomatic Treatment of Acquired Dystonia: A Systematic Review. Movement Disorders Clinical Practice. 2016;3(6):548-58. doi: 10.1002/mdc3.12400.
457. Vannini B, Bocci T, Caleo M, Vergari M, Rossi S, Priori A, et al. An unexpected target of spinal direct current stimulation: Interhemispheric connectivity in humans. Clinical Neurophysiology. 2016;127(4):e158. doi: 10.1016/j.clinph.2015.09.122.
458. Walsh EM, Blabas B, Guernon A. Reliability of motor threshold testing. Archives of Physical Medicine and Rehabilitation. 2016;97(10):e80.
459. Abstracts of the 62nd National Congress of the Italian Society for Clinical Neurophysiology. Clinical Neurophysiology. 2017;128(12).
460. Abstracts of the 61st Scientific Annual Meeting of the German Society for Clinical Neurophysiology and Functional Imaging, DGKN 2017. Clinical Neurophysiology. 2017;128(10).
461. 2017 Symposium "Brain Plasticity in Epilepsy". Acta Neurologica Belgica. 2017;117(3).
462. International Society for ECT and Neurostimulation Annual Meeting Abstracts 2017. Journal of ECT. 2017;33(3).

463. Adamson M, Main K, Milazzo AC, Bickart K, Furst A, Ashford J, et al. Detection and treatment of chronic sequalae of brain injury in veterans. Journal of Head Trauma Rehabilitation. 2017;32(6):E95. doi: 10.1097/HTR.0000000000000241.
464. Ahn S, Son BC, Choi JG, Ha SW, Ko HC. Long term results of simultaneous trial of deep brain and motor cortex stimulation in refractory neuropathic pain. Stereotactic and Functional Neurosurgery. 2017;95:347. doi: 10.1159/000478281.
465. Attard Trevisan A, Rotonda M, Antigua L, Cavallari P. Towards a personalised model for transcranial direct current stimulation (tDCS). Brain Stimulation. 2017;10(2):378. doi: 10.1016/j.brs.2017.01.119.
466. Banús J, Martens G, Salvador R, Thibaut A, Ripolles O, Antonopoulos G, et al. The effects of lesions in e-field distribution during frontoparietal TDCS. Brain Stimulation. 2017;10(2):442-3. doi: 10.1016/j.brs.2017.01.317.
467. Bassi MS, Gilio L, Buttari F, Maffei P, Marfia GA, Restivo DA, et al. Remodeling functional connectivity in multiple sclerosis: A challenging therapeutic approach. Frontiers in Neuroscience. 2017;11(DEC). doi: 10.3389/fnins.2017.00710.
468. Bestmann S, Ward N. Are current flow models for transcranial electrical stimulation fit for purpose? Brain Stimulation.

2017;10(4):865-6. doi: 10.1016/j.brs.2017.04.002.

469. Bockbrader MA. Physician-monitored home tDCS for post-brain injury fatigue, depression and headache: A case report. Brain Stimulation. 2017;10(4):e33-e4. doi: 10.1016/j.brs.2017.04.052.

470. Bodart O, Amico E, Wannez S, Gomez F, Casarotto S, Rosanova M, et al. Global structural and effective connectivity in patients with chronic disorders of consciousness. Brain Stimulation. 2017;10(2):353. doi: 10.1016/j.brs.2017.01.035.

471. Boes A, Labar D, Kuceyeski A, Pascual-Leone A, Fox M, Edwards D. Lesion mapping of bilateral motor threshold in chronic post-stroke hemiparesis. Neurology. 2017;88(16).

472. Bologna M, Berardelli A. Cerebellum: An explanation for dystonia? Cerebellum and Ataxias. 2017;4(1). doi: 10.1186/s40673-017-0064-8.

473. Bragina OA, Lara DA, Yang Y, Nemoto EE, Shuttleworth CW, Semyachkina-Glushkovskaya OV, et al. Anodal transcranial direct current stimulation increases cerebral blood flow, tissue oxygenation and improves neurologic outcome in mice after traumatic brain injury. Brain Stimulation. 2017;10(4):e75-e6. doi: 10.1016/j.brs.2017.04.115.

474. Celnik P. The role of the cerebellum on motor recovery

following stroke. Brain Stimulation. 2017;10(2):500. doi: 10.1016/j.brs.2017.01.459.

475. Da Silva A. New insights in vivo into the treatment of migraine and other chronic pain disorders using non-invasive neuromodulation. Brain Stimulation. 2017;10(2):387. doi: 10.1016/j.brs.2017.01.143.

476. Dario A, Baldeschi GC, DeCarolis G, Luxardo N, Nosella P, Natale MNMNM, et al. New implantable neuromodulation system for peripheral nerve stimulation: Efficacy on pain and mental status. Neuromodulation. 2017;20(7):e210. doi: 10.1111/ner.12639.

477. Dong X, Wei T, Papa EV, Feng Z. Median nerve electrical stimulation causes wake-promotion by downregulating the expression of γ-aminobutyric acid b receptor via orexins pathway in comatose rats caused by traumatic brain injury. International Journal of Clinical and Experimental Medicine. 2017;10(10):14571-8.

478. Eisner W, Quirbach S, Sohm F, Kerschbaumer J. Possible reason for the loss of effectivity of motor cortex stimulation. Stereotactic and Functional Neurosurgery. 2017;95:368. doi: 10.1159/000478281.

479. Ferrari PF, Barbot A, Bianchi B, Ferri A, Garofalo G, Bruno N, et al. A proposal for new neurorehabilitative intervention on

Moebius Syndrome patients after 'smile surgery'. Proof of concept based on mirror neuron system properties and hand-mouth synergistic activity. Neuroscience and Biobehavioral Reviews. 2017;76:111-22. doi: 10.1016/j.neubiorev.2017.01.050.

480. Gilmore N, Dwyer M, Kiran S. A systematic review of standardized outcome measures used in aphasia rehabilitation, including meta-analyses. Archives of Physical Medicine and Rehabilitation. 2017;98(10):e137. doi: 10.1016/j.apmr.2017.08.447.

481. Giordano J, Bikson M, Kappenman ES, Clark VP, Coslett HB, Hamblin MR, et al. Mechanisms and effects of transcranial direct current stimulation. Dose-Response. 2017;15(1). doi: 10.1177/1559325816685467.

482. Guo F, Lou J, Han X, Deng Y, Huang X. Repetitive transcranial magnetic stimulation ameliorates cognitive impairment by enhancing neurogenesis and suppressing apoptosis in the hippocampus in rats with ischemic stroke. Frontiers in Physiology. 2017;8(AUG). doi: 10.3389/fphys.2017.00559.

483. Henrich-Noack P, Sergeeva EG, Sabel BA. Non-invasive electrical brain stimulation: From acute to late-stage treatment of central nervous system damage. Neural Regeneration Research. 2017;12(10):1590-4. doi: 10.4103/1673-5374.217322.

484. Hone-Blanchet A, Mondino M, Fecteau S. Repetitive transcranial magnetic stimulation reduces anxiety symptoms, drug cravings, and elevates 1H-MRS brain metabolites: A case report. Brain Stimulation. 2017;10(4):856-8. doi: 10.1016/j.brs.2017.03.007.
485. Ilyichev VP, Martynov IV, Gorelik AL, Naryshkin AG. Experience of complex application of neuromodulation technologies in cases of severe cerebral lesions. Brain Stimulation. 2017;10(2):406-7. doi: 10.1016/j.brs.2017.01.206.
486. Jalan A, Shinde D, Kudalkar K, Jalan R, Borugale M, Tawde R, et al. Carnitine palmitoyl transferase deficiency in Indian patients. Journal of Inborn Errors of Metabolism and Screening. 2017;5:219. doi: 10.1177/2326409817722292.
487. Khan IR, Rehan M, Badjatia N, Parikh G, Kon Z, Herr D, et al. Near infrared spectroscopy as a non-invasive brain injury monitor in adult extracorporeal membrane oxygenation. Neurocritical Care. 2017;27(2):S248. doi: 10.1007/s12028-017-0465-9.
488. King R, Seeger T, Zewdie E, Ciechanski P, Damji O, Kirton A, et al. Longitudinal follow-up of cortical excitability in paediatric traumatic brain injury. Brain Injury. 2017;31(6-7):955-6. doi: 10.1080/02699052.2017.1312145.
489. Kregel E, Walz M, Nelson A, Con-Way T. Intrathecal catheters

for targeted drug delivery: Computer modeling of bending the ascenda® catheter. Neuromodulation. 2017;20(7):e236-e7. doi: 10.1111/ner.12639.

490. Krieg S, Bulubas L, Sollmann N, Tanigawa N, Meyer B. Plasticity of motor representations in patients with brain lesions: A navigated TMS study. Journal of Neurosurgery. 2017;126(4):A1415. doi: 10.3171/2017.4.JNS.AANS2017abstracts.

491. Lee HY, Kim TW. Clinical effects of intermittent theta-burst stimulation of the anterior temporal lobe to promote visual motor integration and perceptual reasoning in pediatric traumatic brain injury: A case study. Brain Stimulation. 2017;10(2):492-3. doi: 10.1016/j.brs.2017.01.440.

492. Lefebvre S, Liew SL. Anatomical parameters of tDCS to modulate the motor system after stroke: A review. Frontiers in Neurology. 2017;8(FEB). doi: 10.3389/fneur.2017.00029.

493. Legostaeva L, Mochalova E, Poydasheva A, Chervyakov A, Kremneva E, Sergeev D, et al. Feasibility of individual diagnostic approach for patients with chronic disorders of consciousness. European Journal of Neurology. 2017;24:147. doi: 10.1111/ene.13367.

494. Li C, Amrit C, Afridi A, Narayan R. Trigeminal nerve

stimulation attenuates secondary brain injury after traumatic brain injury and hemorrhagic shock. Journal of Neurotrauma. 2017;34(13):A123. doi: 10.1089/neu.2017.29011.abstracts.

495. Love S, Jaouen V, Annan M, Cottier JP, Vercouillie J, Guilloteau D, et al. Robust supervised clustering analysis of 18F DPA-714 dynamic clinical PET data. Journal of Nuclear Medicine. 2017;58.

496. Madsen C, Vaughan M, Koehlmoos TP. Use of Integrative Medicine in the United States Military Health System. Evidence-based Complementary and Alternative Medicine. 2017;2017. doi: 10.1155/2017/9529257.

497. Marei A, Rashed H. One session of repetitive transcranial magnetic stimulation (rTMS) can improve catatonia features associated with major depressive disorder (MDD): Case study. Brain Stimulation. 2017;10(2):519. doi: 10.1016/j.brs.2017.01.515.

498. Maslenikov N, Tsukarzi EE, Mosolov SN. Treatment-resistant depression in schizophrenia: Clinical response predictors of repetitive transcranial magnetic stimulation (rTMS). European Neuropsychopharmacology. 2017;27:S693-S4.

499. Maurer-Karattup P, Morrissey AM. Comparison of treatment for disorders of consciousness (DOC) across countries-results of an international survey. Brain Injury. 2017;31(6-7):841. doi:

10.1080/02699052.2017.1312145.
500. Menolascino SM, Belgin M, Izzo GN, Fisher LE. Maintenance tDCS: A case of full and durable recovery from myalgic encephalomyelitis/chronic fatigue syndrome (ME/CFS). Brain Stimulation. 2017;10(4):e69-e71. doi: 10.1016/j.brs.2017.04.112.
501. Moisset X, Bouhassira D. Non-invasive brain neurostimulation for neuropathic pain. Douleurs. 2017;18(2):71-7. doi: 10.1016/j.douler.2017.03.007.
502. Nada M, Ghany WA, Fayed Z, Elbahy K, Ghanem E. Comparative study between the outcomes of neuroablative and neuromodulation techniques in the treatment of secondary dystonia. Stereotactic and Functional Neurosurgery. 2017;95:244. doi: 10.1159/000478281.
503. Nicoletti CG, Monteleone F, Marfia GA, Centonze D, Mori F. Enhancing brain plasticity to contrast clinical progression in MS: A pilot study assessing the safety and efficacy of D-Aspartate. Multiple Sclerosis Journal. 2017;23(3):657-8. doi: 10.1177/1352458517731406.
504. Nuta J, Itonaga M, Tamura T, Kitano M, Hirono S, Kawai M, et al. Usefulness of contrast-enhanced harmonic endoscopic ultrasonography for identification of mural nodules in intraductal

papillary mucinous neoplasms. Gastrointestinal Endoscopy. 2017;85(5):AB343.
505. Orfila J, Dietz R, Patsos O, Schmidt R, Traystman R, Herson P. Extended therapeutic window of a novel peptide inhibitor of TRPM2 channels on memory following traumatic brain injury. Journal of Cerebral Blood Flow and Metabolism. 2017;37(1):411. doi: 10.1177/0271678X17695986.
506. Parazzini M, Chiaramello E, Fiocchi S, Ravazzani P. Computational modelling studies for non-invasive brain stimulation. European Archives of Psychiatry and Clinical Neuroscience. 2017;267:S135-S6. doi: 10.1007/s00406-017-0856-0.
507. Pashkovskaya O, Polovnikov E. A study on the patient positioning accuracy for stereotactic radiotherapy of brain lesions. Radiotherapy and Oncology. 2017;123:S994.
508. Pearce AJ, Rist B, Cohen A. Electrophysiological changes in retired Australian rugby players with a history of repeated sports concussion: A study using transcranial magnetic stimulation. Brain Injury. 2017;31(6-7):825-6. doi: 10.1080/02699052.2017.1312145.
509. Peng CW, Chen SC, Hsieh TH. Influence of cortical electrical stimulation on neural plasticity in a rat model of traumatic brain injury. Brain Stimulation. 2017;10(2):362. doi:

10.1016/j.brs.2017.01.066.
510. Quoilin C, De Timary P, Duque J. Impaired inhibitory control in alcohol-dependence: Relationship with brain damage and relapse. Alcoholism: Clinical and Experimental Research. 2017;41:84A. doi: 10.1111/acer.13391.
511. Rothwell J. Understanding and improving mechanisms of non-invasive brain stimulation. Brain Stimulation. 2017;10(2):540. doi: 10.1016/j.brs.2017.01.572.
512. Rovlias A, Blionas A, Melissaris S, Papoutsakis D. Strategies and public health impact of head injuries prevention. Brain Injury. 2017;31(6-7):983-4. doi: 10.1080/02699052.2017.1312145.
513. Sánchez-Kuhn A, Medina Y, Pérez-García M, Martínez-Sola M, De Haro P, Flores P, et al. Neurorehabilitation treatment of dysphagia after-stroke with transcranial direct current stimulation: A clinical case. Brain Stimulation. 2017;10(2):431. doi: 10.1016/j.brs.2017.01.283.
514. Shamanur SB, Prabhu KV. Pseudohypoaldosteronism type 1: A challenging diagnosis. Hormone Research in Paediatrics. 2017;88:76. doi: 10.1159/000481424.
515. Sheehan D, Fickling S, Livingstone A, Tannouri P. Symstematic review and proposed study design for neuromodulation therapy in dementia. Irish Journal of Medical

Science. 2017;186(1):S15-S6. doi: 10.1007/s11845-016-1532-5.

516. Siddiqi SH, Hacker CD, Kandala S, Laumann TO, Trapp NT, Brody DL, et al. Individualized precision targeting of resting-state networks with transcranial magnetic stimulation. Journal of ECT. 2017;33(3):212. doi: 10.1097/YCT.0000000000000429.

517. Sollmann N, Wildschutz N, Boeckh-Behrens T, Meyer B, Krieg SM. Associations between clinical outcome and navigated TMS characteristics in patients with motor-eloquent brain lesions: A combined NTMS efiber tracking approach. Brain Stimulation. 2017;10(2):348. doi: 10.1016/j.brs.2017.01.016.

518. Stip E, Lespérance P, Farmer O, Fournier-Gosselin MP. First clinical use of epidural stimulation in catatonia. Brain Stimulation. 2017;10(4):859-61. doi: 10.1016/j.brs.2017.03.006.

519. Sun Y, Wong W, Farzan F, Blumberger DM, Daskalakis ZJ. Neuromodulation of subgenual cingulate activity localizable from EEG. BMC Neuroscience. 2017;18. doi: 10.1186/s12868-017-0371-2.

520. Suykerbuyk JK, Demeester MAUD. A double-blind placebo controlled pilot-study of duloxetine as treatment of mood instability after acquired brain injury (ABI). Journal of Head Trauma Rehabilitation. 2017;32(6):E76. doi: 10.1097/HTR.0000000000000241.

521. Tejale HG, Khandheria B, Smalligan RD. Eagle in a tailspin (stylohyoid syndrome leads to vertigo). Journal of General Internal Medicine. 2017;32(2):S491.

522. Trachsel DS, Tejada MA, Grosfjeld Christensen V, Pedersen PJ, Kanters JK, Calloe K, et al. Trimethoprim-sulfadiazine has Kv11.1 potassium channel blocking activitiy in horses. Journal of Veterinary Internal Medicine. 2017;31(2):611.

523. Trapp NT, Siddiqi SH, Carter AC, Brody DL. Functional magnetic resonance imaging-navigated repetitive transcranial magnetic stimulation of the dorsal attention network alters functional connectivity in a predictable manner. Journal of ECT. 2017;33(3):215. doi: 10.1097/YCT.0000000000000429.

524. Ulyanova A, Koch P, Grovola M, Russo R, Smith D, Johnson V, et al. In vivo electrophysiological alterations of the hippocampus in a porcine model of diffuse axonal injury. Journal of Neurotrauma. 2017;34(13):A119. doi: 10.1089/neu.2017.29011.abstracts.

525. Votta-Velis EG. Pro con debate 5: Intrathecal drug delivery is the way forward. Regional Anesthesia and Pain Medicine. 2017;42(5):e7. doi: 10.1097/AAP.0000000000000656.

526. Watanabe M, Michou E, Raginis-Zborowska A, Magara J, Inoue M, Hamdy S. Ten Hz repetitive cerebellar magnetic

stimulation reverses cortical suppression in the healthy human pharyngeal motor system. Gut. 2017;66:A269. doi: 10.1136/gutjnl-2017-314472.525.

527. Wise N. Transcranial photobiomodulation for stress: Why, how, and where to treat the brain to relax the body. Lasers in Medical Science. 2017;32(8):1703. doi: 10.1007/s10103-017-2327-2.

528. Zhang J. Multivariate analysis and machine learning in cerebral palsy research. Frontiers in Neurology. 2017;8(DEC). doi: 10.3389/fneur.2017.00715.

529. Abstracts of the 62nd Scientific Annual Meeting of the German Society for Clinical Neurophysiology and Functional Imaging, DGKN. Clinical Neurophysiology. 2018;129(8).

530. Anghinah R, Paiva WS, Falk TH, Fregni F. Editorial: NEUROTRAUMA: from emergency room to back to day-by-day life. Frontiers in Neurology. 2018;9(SEP). doi: 10.3389/fneur.2018.00776.

531. Barilka V, Matlan V, Shalay O, Yevstachevych Y, Prymak S, Loginsky V. The impaired production of TNF, TGF beta 1 as an important reason of defective natural killer ability in non-Hodgkin lymphoma patients with spleen affection. HemaSphere. 2018;2:1038-9. doi: 10.1097/HS9.0000000000000060.

532. Bodi I, Giamouriadis A, Sibtain N, Laxton R, King A, Vergani F. Primary intracerebral INI1-deficient rhabdoid tumor with CD34 immunopositivity in a young adult. Surgical Neurology International. 2018;9(1). doi: 10.4103/sni.sni_334_17.
533. Boly M. Quantifying consciousness. Clinical EEG and Neuroscience. 2018;49(6):NP9. doi: 10.1177/1550059418756508.
534. Bragina OA, Nemoto EM, Bragin DE. Anodal transcranial direct current stimulation (tDCS) improves impaired cerebrovascular reactivity in traumatized mouse brain. Journal of Neurotrauma. 2018;35(16):A264. doi: 10.1089/neu.2018.29013.abstracts.
535. Casarotto S, Comanducci A, Casali AG, Rosanova M, Massimini M. A non-invasive perturbation approach to disorders of consciousness. International Journal of Psychophysiology. 2018;131:S65. doi: 10.1016/j.ijpsycho.2018.07.193.
536. Chieffi S, Ilardi CR, Iavarone A. Parietal lobe dysfunction in schizophrenia: A review. Current Psychiatry Reviews. 2018;14(2):71-83. doi: 10.2174/1573400514666180703150804.
537. Christova M, Gallasch E, Pinter D. Cerebellar contributions to somatosensory induced neuroplasticity. Clinical Neurophysiology. 2018;129:e141. doi: 10.1016/j.clinph.2018.04.357.
538. Corr PJ, Mobbs D. From epiphenomenon to biologically

important phenomena. Personality Neuroscience. 2018;1:1-4. doi: 10.1017/pen.2017.1.

539. Danilov Y, Paltin D. Translingual neurostimulation (TLNS): Perspective on a novel approach to neurorehabilitation after brain injury. 2018. p. 307-27.

540. Domen K, Takebayashi T, Takahashi K, Moriwaki M. A pilot randomized controlled trial of constraint-induced movement therapy combined with transcranial direct current stimulation and peripheral neuromuscular stimulation. Neurorehabilitation and Neural Repair. 2018;32(4-5):340. doi: 10.1177/1545968318765497.

541. Epperson M, Horn P, Wu S, Pedapati E, Guthrie M, Huddleston D, et al. Motor cortex physiology of movement preparation in ADHD children. Developmental Medicine and Child Neurology. 2018;60:95-6. doi: 10.1111/dmcn.30_14018.

542. Grangeon L, O'Connor E, Matharu M. Cohort study of post-traumatic cluster headache. Headache. 2018;58:201-2.

543. Han D, She J, Guo H, Sun C, Li H. Ablation or pacing, which goes first for patients with tachycardia-bradycardia syndrome: Two case comparison and clinical enlightenment. Journal of Interventional Cardiac Electrophysiology. 2018;51(1):S112-S3. doi: 10.1007/s10840-018-0338-y.

544. Hartl E, Bötzel K, Mehrkens JH, Noachtar S. Seizure reductions outlast DBS explantation. Brain Stimulation. 2018;11(3):636-8. doi: 10.1016/j.brs.2018.01.033.
545. Henssen D, Dao J, Witkam R, Comes D, Van Cappellen Van Walsum AM, Kozicz T, et al. Phenotypical and neurosurgical characteristics of non-responders to motor cortex stimulation as a treatment of pain: A systematic review and metaanalysis of individual patient data. Pain Practice. 2018;18:131.
546. Hermann B, Raimondo F, Valente M, Rohaut B, Perez P, Sitt JD, et al. Electrophysiological effects of transcranial direct current stimulation in patients suffering from disorders of consciousness : An open-label pilot study. Neurocritical Care. 2018;29(1):S312. doi: 10.1007/s12028-018-0606-9.
547. Holeckova I, Vales J, Mracek J, Rihanek P, Moucek R, Mautner P, et al. The changes in motor cortex localisation and organisation in motor eloquent tumorous brain lesions detected by navigated transcranial magnetic stimulation. Clinical Neurophysiology. 2018;129:e46. doi: 10.1016/j.clinph.2018.04.114.
548. Jung KI, Ohn SH, Lee H. GLOBAL SYNCHRONIZATION INDEX AS A NEW INDICATOR FOR TRACKING COGNITIVE FUNCTION CHANGES BY TRANSCRANIAL DIRECT

CURRENT STIMULATION IN A TRAUMATIC BRAIN INJURED PATIENT: A CASE REPORT. Alzheimer's and Dementia. 2018;14(7):P289-P90. doi: 10.1016/j.jalz.2018.06.058.

549. Kaczmarek BLJ, Markiewicz K. Current and traditional views on the brain works. Acta Neuropsychologica. 2018;16(2):201-12. doi: 10.5604/01.3001.0012.2052.

550. Khodaei S, Avramescu S, Lecker I, Whissell P, Wang DS, Baker AJ, et al. Inhibiting α5GABAA receptors restores synaptic plasticity in the hippocampus after traumatic brain injury in mice. Anesthesia and Analgesia. 2018;126(4):775-6.

551. King D. Use of the King-Devick test and Brain Gauge for the management of concussion. Journal of Science and Medicine in Sport. 2018;21:S38-S9. doi: 10.1016/j.jsams.2018.09.089.

552. Laureys S. Neuromodulation: Outlook into the future. European Journal of Neurology. 2018;25:11.

553. Mak A, Dharmadhikari B, Wong LW, Sreedharan SK, Schwarz H. The differential impact of the abrogation of the costimulatory molecule CD137 ligand on renal and cerebral manifestations in C57BL/6.FASLPR-/- mice. Arthritis and Rheumatology. 2018;70:2318-9. doi: 10.1002/art.40700.

554. Manoj NK, Singh U, Shahid NA. Pilot study on outcome of brief right median nerve stimulation and multimodal sensory

stimulation in comatose patients. Neurorehabilitation and Neural Repair. 2018;32(4-5):475-6. doi: 10.1177/1545968318765498.
555. Matheson N, Fisher S, Shemmell J, Reynolds J. The cellular effects of rTMS in healthy and stroke-damaged brains: An in vivo intracellular electrophysiological study in the rat. Neurorehabilitation and Neural Repair. 2018;32(12):1080. doi: 10.1177/1545968318817151.
556. Nagai M, Tanaka N, Oouchida Y, Izumi SI. Effect of Cathodal Transcranial Direct Current Stimulation on a Child with Involuntary Movement after Hypoxic Encephalopathy. Case Reports in Medicine. 2018;2018. doi: 10.1155/2018/8934253.
557. Nemanich S, Rich T, Mueller B, Opitz A, Gillick B. Multimodal neurophysiologic and neuroanatomic assessment of brain connectivity in pediatric stroke. Developmental Medicine and Child Neurology. 2018;60:19-20. doi: 10.1111/dmcn.27_14017.
558. Orfila JE, Clevenger AC, Dietz RM, Patsos OP, Schroeder C, Schmidt R, et al. A novel peptide inhibitor of TRPM2 channels reduces memory deficits and improves LTP following traumatic brain injury in mice. Journal of Neurotrauma. 2018;35(16):A138. doi: 10.1089/neu.2018.29013.abstracts.
559. Oxley TJ, Opie NL, Rind GS, Liyanage K, John SE, Ronayne S, et al. An ovine model of cerebral catheter venography for

implantation of an endovascular neural interface. Journal of Neurophysiology. 2018;128(4):1020-7. doi: 10.3171/2016.11.JNS161754.
560. Piccillo GA, Mondati EGM, Gasbarrini G. A severe and rare kind of headache drastically reduced the quality of life of a young unfortunate woman…. Italian Journal of Medicine. 2018;12(2):95. doi: 10.4081/itjm.2018.s2.
561. Pixa NH, Pollok B. Effects of tDCS on bimanual motor skills: A brief review. Frontiers in Behavioral Neuroscience. 2018;12. doi: 10.3389/fnbeh.2018.00063.
562. Poydasheva A, Legostaeva L, Mochalova E, Kremneva E, Sinitsyn D, Sergeev D, et al. RTMS application in disorders of consciousness. Clinical Neurophysiology. 2018;129:e153. doi: 10.1016/j.clinph.2018.04.389.
563. Rosenow JM, Blabas BC, Keltzel S, Herrold A, Parrish T, Guernon A, et al. RTMS and amantadine for ppersons in chronic states of disordered consciousness after TBI. Neuromodulation. 2018;21(3):e79. doi: 10.1111/ner.12774.
564. Rossini PM. Connectivity disturbances in stroke as revealed by integrated multimodal neuroimaging by fMRI, EEG and TMS-EEG. European Journal of Neurology. 2018;25:633.
565. Schellack N, Masuku P, Mogale K, Mokwele N, Skosana P.

Hands on management of muscle pain and injuries. SA Pharmaceutical Journal. 2018;85(6):27-36.
566. Schmidt J, Brown KE, Feldman SJ, Babul S, Zwicker JG, Boyd LA. Evidence of altered interhemispheric communication in pediatric mild traumatic brain injury. Neurorehabilitation and Neural Repair. 2018;32(12):1102. doi: 10.1177/1545968318817151.
567. Sekar S, Zhang Y, Taghibiglou C. Role of low field magnetic stimulation on cognitive and motor functions in traumatic brain injury mouse model. Journal of Neurotrauma. 2018;35(16):A137. doi: 10.1089/neu.2018.29013.abstracts.
568. Sgandurra G, Biagi L, Guzzetta A, Fogassi L, Ferrari A, Cioni G, et al. Reorganization of action observation network in children with unilateral cerebral palsy: An fMRI study. Developmental Medicine and Child Neurology. 2018;60:16-7. doi: 10.1111/dmcn.22_14017.
569. Simon-Martinez C, Jaspers E, Mailleux L, Ortibus E, Klingels K, Wenderoth N, et al. Corticospinal tract wiring and brain lesion characteristics in unilateral cerebral palsy: Determinants of upper limb motor and sensory function. Neurorehabilitation and Neural Repair. 2018;32(12):1087. doi: 10.1177/1545968318817151.
570. Sokhadze EM, Lamina EV, Casanova EL, Kelly DP, Opris I,

Tasman A, et al. Exploratory study of rTMS neuromodulation effects on electrocortical functional measures of performance in an oddball test and behavioral symptoms in autism. Frontiers in Systems Neuroscience. 2018;12. doi: 10.3389/fnsys.2018.00020.

571. Sollmann N, Wildschuetz N, Kelm A, Conway N, Moser T, Bulubas L, et al. Associations between clinical outcome and navigatedtranscranial magnetic stimulation characteristics inpatients with motor-eloquent brain lesions: A combinednavigated transcranial magnetic stimulation-diffusiontensor imaging fiber tracking approach. Journal of Neurophysiology. 2018;128(3):800-10. doi: 10.3171/2016.11.JNS162322.

572. Steele VR, Maxwell AM, Ross TJ, Moussawi K, Abulseoud OO, Stein EA, et al. Report of transient events in a cocaine-dependent volunteer who received iTBS. Brain Stimulation. 2018;11(3):631-3. doi: 10.1016/j.brs.2018.01.004.

573. Stilling J, Duszynski C, Oni I, Paxman E, Dunn J, Debert C. Using functional near-infrared spectroscopy to study the effect of repetitive transcranial magnetic stimulation in concussion. Journal of Neurotrauma. 2018;35(16):A103-A4. doi: 10.1089/neu.2018.29013.abstracts.

574. Subramanian S, Fountain M, Hood A. Interventions to augment upper extremity motor improvement in individuals with a

traumatic brain injury: A systematic review. Neurorehabilitation and Neural Repair. 2018;32(12):1110-1. doi: 10.1177/1545968318817151.

575. Sundaravadhanan S. Neurotrauma: A Futuristic Perspective. Indian Journal of Neurotrauma. 2018;15(2-3):78-81. doi: 10.1055/s-0039-1694297.

576. Tasca CI, Lanznaster D, Oliveira KA, Fernández-Dueñas V, Ciruela F. Neuromodulatory effects of guanine-based purines in health and disease. Frontiers in Cellular Neuroscience. 2018;12. doi: 10.3389/fncel.2018.00376.

577. Thibaut A, Chatelle C, Vanhaudenhuyse A, Martens G, Cassol H, Martial C, et al. Transcranial direct current stimulation unveils covert consciousness. Brain Stimulation. 2018;11(3):642-4. doi: 10.1016/j.brs.2018.02.002.

578. Torrente A, Buscarnera S, Curto S, Di Stefano V, Cosentino G, Giglia G, et al. Transcranial direct current stimulation in post-stroke aphasia rehabilitation: Bilateral vs unilateral online stimulation. European Journal of Neurology. 2018;25:565.

579. Vyas S. A conversation with Mehmet Fatih Yanik. 2018. p. 290-2.

580. Wu V, Cooke B, Eitutis S, Simpson MTW, Beyea JA. Approach to tinnitus management. Canadian Family Physician.

2018;64(7):e293-e8.
581. Yamasaki F, Kolakshyapati M, Hashizume A, Ochi K, Ueno H, Kaichi Y, et al. Histogram analysis in ring-enhancing intracranial lesions. Neuroradiology. 2018;60(10):1119. doi: 10.1007/s00234-018-2086-1.
582. Yankevich DS, Borodin MM, Usoltseva NI, Shevtsova EE, Kovaleva GA, Gorbeshko GA. Personalized patient neurorehabilitation in early post-coma period. The uniqueness of a single case. International Journal of Pharmaceutical Research. 2018;10(1):305-19.
583. Zardouz S, Leung A. Long term feasibility and efficacy of RTMS in alleviating MTBI headaches. Pain Medicine (United States). 2018;19(4):855-6. doi: 10.1093/pm/pny044.
584. Zhou Y, Coughlin J, Du Y, Liu M, Leal J, Wong D, et al. A genetic polymorphism based kinetic analysis of [11C]DPA-713 dynamic PET for precision imaging of brain injury in National Football League players. Journal of Nuclear Medicine. 2018;59.
585. 2019 ACRM Annual Conference. Archives of Physical Medicine and Rehabilitation. 2019;100(10):A1-A22. doi: 10.1016/s0003-9993(19)31009-3.
586. European Congress of Clinical Neurophysiology. Clinical Neurophysiology. 2019;130(7):e21-e116. doi:

10.1016/j.clinph.2019.04.317.
587. 3rd International Brain Stimulation Conference. Brain Stimulation. 2019;12(2).
588. 63rd National Congress of the Italian Society for Clinical Neurophysiology. Clinical Neurophysiology. 2019;130(1).
589. Aanen IP, Elders BBLJ, Pullens B, Wijnen RMH. Tracheomalacia after esophageal repair: A systematic review. Diseases of the Esophagus. 2019;32:15. doi: 10.1093/dote/doz047.41.
590. Abraham M, Poulopoulos N, Larson E. Clinical Utility of Transcranial Direct Current Stimulation (tDCS) Following Traumatic Brain Injury and Stroke. Archives of Physical Medicine and Rehabilitation. 2019;100(10):e149. doi: 10.1016/j.apmr.2019.08.454.
591. Adamson M, Siddiqi S, McNerney W, Swaminath G, Wu L, Wortman K, et al. Plasticity mechanisms underlying repetitive transcranial magnetic stimulation for improving cognition in brain injury. Neuromodulation. 2019;22(7):e417. doi: 10.1111/ner.12958.
592. Ahmed M, Siddiqi S, Haque S, Chau J, Amin H. Safety and efficacy of repetitive Transcranial Magnetic Stimulation (rTMS) for depression in mild to moderate Traumatic Brain Injury (TBI): a non-controlled study. Brain Stimulation. 2019;12(2):585. doi:

10.1016/j.brs.2018.12.943.
593. Al-Sultan F, Sultan Alsultan A. Safety of transcranial direct current stimulation during fasting in healthy adults. Journal of Clinical Neurophysiology. 2019;36(6):503. doi: 10.1097/WNP.0000000000000642.
594. Almousa A, Alajaji R, Alaboudi M, Al-sultan F, Bashir S. Safety of transcranial direct current stimulation across three brain regions in fasting. Brain Stimulation. 2019;12(2):402. doi: 10.1016/j.brs.2018.12.296.
595. Ay H. Post-extubation dysphagia (PED). Turk Beyin Damar Hastaliklar Dergisi. 2019;25:91-5.
596. Badran B, Jenkins D, DeVries W, Dancy M, Cook D, Mappin G, et al. Development of Closed-loop Transcutaneous Auricular Vagus Nerve Stimulation (taVNS) as a Neurorehabilitation Tool. Brain Stimulation. 2019;12(2):523. doi: 10.1016/j.brs.2018.12.721.
597. Bakulin I, Poydasheva A, Lagoda D, Zabirova A, Evdokimov K, Suponeva N, et al. P79-S Tolerability of repetitive transcranial magnetic stimulation: Analysis of 727 sessions with standardized questionnaires. Clinical Neurophysiology. 2019;130(7):e116. doi: 10.1016/j.clinph.2019.04.607.
598. Barbero-Castillo A, Weinert JF, Camassa A, Perez-Mendez L, Caldas-Martinez S, Mattia M, et al. Proceedings #31: Cortical

Network Complexity under Different Levels of Excitability Controlled by Electric Fields. Brain Stimulation. 2019;12(2):e97-e9. doi: 10.1016/j.brs.2018.12.200.

599. Batista J, Porto H. Abstract #131: tDCS in Post Firearm Brain Injury - Case Report. Brain Stimulation. 2019;12(2):e45. doi: 10.1016/j.brs.2018.12.138.

600. Berger A, Horst F, Müller S, Steinberg F, Doppelmayr M. Current state and future prospects of EEG and fNIRS in robot-assisted gait rehabilitation: A brief review. Frontiers in Human Neuroscience. 2019;13. doi: 10.3389/fnhum.2019.00172.

601. Boyd R, Pagnozzi A, Fripp J, Rose S. Understanding the impact of bilateral brain injury in children with cerebral palsy. Developmental Medicine and Child Neurology. 2019;61:54. doi: 10.1111/dmcn.14353.

602. Burroughs L, Mitroi J, Bolbecker A, Moussa-Tooks A, Lundin N, O'Donnell B, et al. Transcranial direct current stimulation of the human cerebellum during associative learning. Brain Stimulation. 2019;12(2):478. doi: 10.1016/j.brs.2018.12.558.

603. Caglayan AB, Beker MC, Caglayan B, Yalcin E, Caglayan A, Yulug B, et al. Acute and post-acute neuromodulation induces stroke recovery by promoting survival signaling, neurogenesis, and pyramidal tract plasticity. Frontiers in Cellular Neuroscience.

2019;13. doi: 10.3389/fncel.2019.00144.

604. Cain J, Monti M. Altered cortical blood flow during sonication of high-order thalamus using low intensity focused ultrasound pulsation. Brain Stimulation. 2019;12(2):480-1. doi: 10.1016/j.brs.2018.12.568.

605. Capone F, Miccininilli S, Pellegrino G, Zollo L, Guglielmelli E, Sterzi S, et al. Combining Transcutaneous Vagus Nerve Stimulation and Upper-Limb Robotic Rehabilitation in Chronic Stroke Patients. Brain Stimulation. 2019;12(2):413. doi: 10.1016/j.brs.2018.12.334.

606. Castagna A, Di Tella S, Blasi V, Cabinio M, Baglio F. Integrity of Mirror Neuron System in cervical dystonia: An fMRI study. Movement Disorder. 2019;34:S775.

607. Chai Z, Ma C, Jin X. Cortical stimulation for treatment of neurological disorders of hyperexcitability: A role of homeostatic plasticity. Neural Regeneration Research. 2019;14(1):34-8. doi: 10.4103/1673-5374.243696.

608. Chattopadhyay A, Uikey MS, Verma R, Kumar N. Intermittent theta burst stimulation (iTBS) for managing treatment resistant depression. Indian Journal of Psychiatry. 2019;61(9):S624.

609. Coben R, Armes C. Meta-analysis of mulitvariate coherence neurofeedback research. NeuroRegulation. 2019;6(4):214-5. doi:

10.15540/nr.6.4.197.

610. Cunningham D, Rickert A, Plow E, Knutson J. Chronic stroke hemispheric dominance and task specific fmri laterality of cortical motor activation. Neurorehabilitation and Neural Repair. 2019;33(12):1082. doi: 10.1177/1545968319893871.

611. Cutsuridis V. Memory prosthesis: Is it time for a deep neuromimetic computing approach? Frontiers in Neuroscience. 2019;13(JUL). doi: 10.3389/fnins.2019.00667.

612. da Costa Ferreira Pinto PH, Nigri F, Caparelli-Dáquer EM, dos Santos Viana J. Computed tomography-guided navigated transcranial magnetic stimulation for preoperative brain motor mapping in brain lesion resection: A case report. Surgical Neurology International. 2019;10. doi: 10.25259/SNI-124-2019.

613. Debruyne P, Rossenbacker T, Collienne C, Janssens L, Roosen J, Vankelecom B, et al. Unifocal right-sided ablation for neurally mediated syncope and functional sinus node dysfunction. Acta Cardiologica. 2019;74:3-4. doi: 10.1080/00015385.2018.1558767.

614. Duarte N, Grecco L, Lopes J, Galli M, Oliveira C. The effect of transcranial direct current stimulation on mobility in a child with cerebral palsy: case report. Brain Stimulation. 2019;12(2):429. doi: 10.1016/j.brs.2018.12.390.

615. Ebbing J, van de Lindt D. Right Sided (RDLPFC) Low

Frequency (1Hz) rTMS in the Third Trimester of Pregnancy; A Case Report. Brain Stimulation. 2019;12(4):e134-e5. doi: 10.1016/j.brs.2019.03.041.

616. Fecchio M, Rosanova M, Casarotto S, Sarasso S, Casali AG, Pigorini A, et al. Sleep-like bistability, loss of causality and complexity in the cerebral cortex of unresponsive wakefulness syndrome patients. Brain Stimulation. 2019;12(2):432. doi: 10.1016/j.brs.2018.12.401.

617. Ferre C, Karen C, Gordon A, Friel K. Bilateral connectivity in children with unilateral brain injury: Implications for upper-extremity function. Developmental Medicine and Child Neurology. 2019;61:128. doi: 10.1111/dmcn.14354.

618. Gao S, Proekt A, Renier N, Calderon D, Pfaff D. Gigantocellular Neurons Awaken the Brain from Deep Pharmacologically-Induced Coma. Brain Stimulation. 2019;12(2):428. doi: 10.1016/j.brs.2018.12.386.

619. Giuffre A, Zewdie E, Kahl C, Kirton A. Robotic Transcranial Magnetic Stimulation (TMS) Motor Mapping in Children. Brain Stimulation. 2019;12(2):549. doi: 10.1016/j.brs.2018.12.813.

620. Glize B, Bigourdan A, Villain M, Munsch F, Tourdias T, de Gabory I, et al. Motor evoked potential of upper-limbs is predictive of aphasia recovery. Aphasiology. 2019;33(1):105-20. doi:

10.1080/02687038.2018.1444137.
621. Hara T, Burhan A, Abo M, Watanabe S, Akimoto H, Fukui R. Improvement of higher brain dysfunction after brain injurywith strategic application of repetitive transcranial magnetic stimulation and intensive rehabilitation therapy: a report of three cases. Brain Stimulation. 2019;12(2):391. doi: 10.1016/j.brs.2018.12.255.
622. Huang Y, Thomas C, Datta A, Parra LC. Proceedings #23: Inaccurate segmentation of lesioned brains can significantly affect targeted transcranial electrical stimulation on stroke patients. Brain Stimulation. 2019;12(2):e87-e9. doi: 10.1016/j.brs.2018.12.192.
623. Iovino L, Cooper K, Kinsella S, deRoos P, Jain R, Dudakov JA. Zinc Improves Thymic Regeneration after Allogeneic HSCT By Stimulating BMP4 Production from Endothelial Cells. Biology of Blood and Marrow Transplantation. 2019;25(3):S333. doi: 10.1016/j.bbmt.2018.12.537.
624. Kalita J, Kumar S, Misra UK. Effect of rTMS and amitriptyline versus rTMS alone in chronic migraine: A randomized controlled trial. European Journal of Neurology. 2019;26:218. doi: 10.1111/ene.14018.
625. Kaur J, Mania I. Deep transcranial magnetic stimulation (dTMS) for treatment of major depressive disorder (MDD) status post-surgical removal of medulloblastoma: A case report of safety.

Brain Stimulation. 2019;12(4):1061-2. doi: 10.1016/j.brs.2019.03.010.

626. Kawamura M, Takahashi N, Kobayashi Y. Effect of Repetitive Transcranial Magnetic Stimulation on the Right Superior Temporal Gyrus for Severe Aphasia Caused by Damage to the Left Inferior Frontal Gyrus. Case Reports in Neurology. 2019;11(2):189-98. doi: 10.1159/000500669.

627. Kaye HL, Block G, Zhang H, Sollmann N, Krieg S, Rotenberg A. Attraction of the motor map toward a seizure focus. Journal of Clinical Neurophysiology. 2019;36(6):485. doi: 10.1097/WNP.0000000000000642.

628. Khan OH. Incorporating Navigated Transcranial Magnetic Stimulation (nTMS) into the neurosurgical practice: Oncological, vascular and research potentials. Canadian Journal of Neurological Sciences. 2019;46:S38-S9. doi: 10.1017/cjn.2019.189.

629. Kopachka M, Sharova E, Alexandrova E, Troshina E, Zaitsev O, Chelyapina M, et al. P76-S Therapeutic possibilities of transcranial magnetic stimulation in patients after traumatic brain injury (updated report). Clinical Neurophysiology. 2019;130(7):e115. doi: 10.1016/j.clinph.2019.04.604.

630. Kowalski J, Nemanich S, Nawshin T, Sutter E, Chen M, Peyton C, et al. Corticospinal connectivity patterns and motor

outcome in infants with early brain injury. Developmental Medicine and Child Neurology. 2019;61:18-9. doi: 10.1111/dmcn.14244.
631. Krieger D, Soose R, Shepard P, Puccio A, Beers S, Schneider W, et al. Reliable magnetoencephalographic measures in chronic post-concussive syndrome-a teamtbi study. Journal of Neurotrauma. 2019;36(13):A115. doi: 10.1089/neu.2019.29100.abstracts.
632. Kumar D, Prabhakaran J, Kim J, Castrillon J, Molotkov A, Dileep H, et al. In vivo evaluation of microtubul ligand [11C]MPC-6827 in animal models of neurodegenerative disorders. Journal of Nuclear Medicine. 2019;60.
633. Levy R. Motor cortex stimulation for pain and related conditions: Pro. Neuromodulation. 2019;22(7):e316. doi: 10.1111/ner.12958.
634. Lin Y, Huang Q, Han T, Su Y, Gao D, Chen W, et al. Evaluating the Effect of Simultaneous Transcranial Direct Current stimulation and Repetitive Transcranial Magnetic Stimulation on Minimally conscious state by Using EEG and Functional MRI. Brain Stimulation. 2019;12(2):403-4. doi: 10.1016/j.brs.2018.12.303.
635. Martens G, Barra A, Laureys S, Thibaut A. Recent advance in the treatment of patients with disorders of consciousness: a review

of transcranial direct current stimulation efficacy. Brain Stimulation. 2019;12(2):428. doi: 10.1016/j.brs.2018.12.385.

636. Masuda F. Potentials of rTMS for neurodevelopmental disorders and road to clarification of TMS neuropathology. Brain Stimulation. 2019;12(2):476. doi: 10.1016/j.brs.2018.12.553.

637. Meek BP, Hill SR, Modirrousta M. Abstract #79: Efficacy of Low-Frequency rTMS over the Right Dorsolateral Prefrontal Cortex following Mild Traumatic Brain Injury. Brain Stimulation. 2019;12(2):e28. doi: 10.1016/j.brs.2018.12.086.

638. Michou E, Sasegbon A, Hamdy S. Direct and indirect therapy: Neurostimulation for the treatment of dysphagia after stroke. 2019. p. 731-61.

639. Mosiello G, Ludy Lopes D, Musciagna F, Del Conte F, Iacobelli B, Chiminello R, et al. Conservative treatment of bowel dysfunction in pediatric population: Role of a correct training for improving effectiveness of transanal irrigation. Neurourology and Urodynamics. 2019;38:S107-S8. doi: 10.1002/nau.24118.

640. Muir O, MacMillan C, Khan M, Sang L, Aron T. Safety and tolerability of dTMS treatment in 18-20 year old patients: case series. Brain Stimulation. 2019;12(4):e137-e8. doi: 10.1016/j.brs.2019.03.049.

641. Muresanu D. Advances in post stroke neurorehabilitation -

From neuro fundamentals to evidence based medicine. Journal of the Neurological Sciences. 2019;405:75. doi: 10.1016/j.jns.2019.10.209.

642. Nardone R, Langthaler P, Orioli A, Versace V, Scarano G, Brigo F, et al. Ipsilateral motor evoked potentials in a patient with unihemispheric cortical atrophy due to Rasmussen encephalitis. Neural Regeneration Research. 2019;14(6):1025-8. doi: 10.4103/1673-5374.250581.

643. Nawshin T, Behan M, Nemanich S, Kowalski J, Sutter E, Boxrud M, et al. Factors influencing parent and caregiver decisions to enroll children to participate in perceived high-risk brain research. Developmental Medicine and Child Neurology. 2019;61:28. doi: 10.1111/dmcn.14244.

644. Nemanich S, Zewdie E, Kuo HC, Francis S, MacKinnon C, Meekins G, et al. Atypical motor evoked potentials from single-pulse transcranial magnetic stimulation in children with hemiparetic cerebral palsy. Neurorehabilitation and Neural Repair. 2019;33(12):1073. doi: 10.1177/1545968319893871.

645. Nemanich ST, Kowalski JL, Chen M, Zorn E, Georgieff M, Gillick BT. Proceedings #1: Longitudinal Assessment of Cortical Excitability in an Infant with Perinatal Brain injury. Brain Stimulation. 2019;12(2):e56-e7. doi: 10.1016/j.brs.2018.12.170.

646. Oknina L, Zigmantovich A, Kopachka M, Zaitsev O, Masherov E, Alexandrova E. The reorganization of functional connectivity under transcranial magnetic stimulation in unconscious patients with severe brain injury. Folia Neuropathologica. 2019;57(3):308. doi: 10.5114/fn.2019.88492.
647. Ondek K, Izadi A, Girgis F, Saez I, Shahlaie K, Gurkoff G. Theta stimulation to treat cognitive dysfunction in rodent models of neurologic disorders. Brain Stimulation. 2019;12(2):582. doi: 10.1016/j.brs.2018.12.933.
648. Ort A, Preller K, Tononi G, Massimini M, Vollenweider FX. Characterization of psilocybin-induced altered state consciousness using TMS-EEG and integrated information theory (IIT). Neuropsychobiology. 2019;77(3):157. doi: 10.1159/000496817.
649. Papa L, Ptito A. Translingual neurostimulation with the portable neuromodulation stimulator (PoNS™) for the treatment of chronic balance deficit due to mild-to-moderate traumatic brain injury. PM and R. 2019;11:S89-S90. doi: 10.1002/pmrj.12271.
650. Peng CW, Shih-Ching C. Motor plasticity effects induced by cortical electrical stimulation in traumatic brain injured rats. Neuromodulation. 2019;22(7):e376. doi: 10.1111/ner.12958.
651. Pigorini A, Parmigiani S, Russo S, Mikulan E, Cattani A, Sarasso S, et al. P50-S State dependent changes in cortical

reactivity: Comparison between transcranial and intracranial stimulation. Clinical Neurophysiology. 2019;130(7):e109. doi: 10.1016/j.clinph.2019.04.586.

652. Podda M, Paciello F, Cocco S, Rolesi R, Troiani D, Fetoni A, et al. Transcranial direct current stimulation affects auditory cortex plasticity in normal-hearing and noise-exposed rats. Brain Stimulation. 2019;12(2):456. doi: 10.1016/j.brs.2018.12.481.

653. Portinho PC. High-risk patient capability for osteoporotic fractures / FUSEX ＊ proposal for the inclusion of patients in a national database. (relevance of FLS-fracture liaison services). Osteoporosis International. 2019;30(SUPPL 2):S454-S5. doi: 10.1007/s00198-019-04993-w.

654. Quinn D, Upston J, Jones T, Richardson J, Worth L, Fratzke V, et al. Abstract #1: Individualizing HD-tDCS With fMRI and E-Field Modeling: Pilot Data from the NAVIGATE-TBI Study. Brain Stimulation. 2019;12(2):e1. doi: 10.1016/j.brs.2018.12.008.

655. Quinn D, Upston J, Jones TR, Fratzke V, Brandt E, Gill D, et al. Cerebral perfusion and postconcussive symptom reduction after cognitive training in chronic mmTBI patients. Journal of Neurotrauma. 2019;36(13):A48-A9. doi: 10.1089/neu.2019.29100.abstracts.

656. Rezaeizadeh H, Rahimi R, Abbasi M. Fatigue due to multiple

sclerosis: A comparison between Persian medicine and conventional medicine. Galen Medical Journal. 2019;8. doi: 10.31661/gmj.v0i0.1139.

657. Rosenthal ZP, Raut R, Voss T, Bauer AQ, Culver J, Raichle M, et al. Local perturbations in cortical excitability propagate differentially through large-scale functional networks. Annals of Neurology. 2019;86:S42-S3.

658. Schlaug G. Abstract #161: When stimulating the unaffected (healthy) hemisphere in stroke patients makes sense. Brain Stimulation. 2019;12(2):e55. doi: 10.1016/j.brs.2018.12.168.

659. Schmidt J, Brown K, Feldman S, Babul S, Zwicker J, Boyd L. Evidence of altered inter-hemispheric communication in paediatric mild traumatic brain injury. Brain Stimulation. 2019;12(2):483. doi: 10.1016/j.brs.2018.12.576.

660. Sergeeva A, Kremneva E, Morozova S, Sinitsyn D, Legostaeva L, Poydasheva A, et al. Effect of repetitive transcranial magnetic stimulation on resting-state functional connectivity in patients with chronic disorders of consciousness. Neuroradiology. 2019;61(1):S75. doi: 10.1007/s00234-019-02263-4.

661. Seritan AL, Ureste P, Duong T, Ostrem JL. Psychopharmacology for patients with Parkinson’s disease and deep brain stimulation: Lessons learned in an academic center.

Current Psychopharmacology. 2019;8(1):41-54. doi: 10.2174/2211556007666180328142953.

662. Shinde A, Munsch F, Alsop D, Schlaug G. Abstract #18: Establishing a dose response for transcranial direct current stimulation. Brain Stimulation. 2019;12(2):e7. doi: 10.1016/j.brs.2018.12.025.

663. Simon-Martinez C, Decraene L, Van Hoof E, Zielinski I, Hoare B, Steenbergen B, et al. The impact of timing of the brain lesion and corticospinal tract organization on mirror movement characteristics in children with unilateral cerebral palsy. Developmental Medicine and Child Neurology. 2019;61:16. doi: 10.1111/dmcn.14244.

664. Sinitsyn D, Kremneva E, Legostaeva L, Yazeva E, Sergeev D, Poidasheva A, et al. TMS-EEG and resting-state fmri accuracy comparison in differentiation of chronic disorders of consciousness. Neuroradiology. 2019;61(1):S33-S4. doi: 10.1007/s00234-019-02263-4.

665. Skinner K, Prabhakaran V. Neurorehabilitation using the portable neuromodulation stimulator (PoNS™) for treating participants with chronic balance deficit due to mild-to-moderate traumatic brain injury: A 26-week study. PM and R. 2019;11:S34. doi: 10.1002/pmrj.12271.

666. Smith TJ, Kamson D, Mealy MA, D'Amato S. Treatment of central and spinal cord neuropathic pain with Scrambler Therapy. Journal of Clinical Oncology. 2019;37(31). doi: 10.1200/JCO.2019.37.31-suppl.127.
667. Sole JV. Transient loss of consciousness with brain stimulation. Brain Stimulation. 2019;12(2):435. doi: 10.1016/j.brs.2018.12.410.
668. Solingapuram Sai KK, Prabhakaran J, Hines J, Norman S, Rajagopal SA, Mintz A, et al. Synthesis and initial in vivo evaluations of [11C]WX-MB-18B, a microtubule imaging agent. Journal of Nuclear Medicine. 2019;60.
669. Sollmann N, Schramm S, Ille S, Meyer B, Krieg S. Application of navigated transcranial magnetic stimulation to map the supplementary motor area in healthy subjects. Brain Stimulation. 2019;12(2):466. doi: 10.1016/j.brs.2018.12.518.
670. Stultz DJ, Osburn S, Burns T, Walton R, Pawlowska-Wajswol S. TMS treatment in a patient with seizures and refractory depression. Brain Stimulation. 2019;12(6):1603-4. doi: 10.1016/j.brs.2019.08.009.
671. Stultz DJ, Thistlethwaite D, Voltin R, Osburn S, Walton R, Burns T, et al. Continued TMS Treatment After A Seizure. Brain Stimulation. 2019;12(4):e139. doi: 10.1016/j.brs.2019.03.056.
672. Uchiyama M, Park G, Monsanto RC, Paparella MM, Cureoglu

S. Pathologic changes of inner ear following head trauma. Otolaryngology - Head and Neck Surgery. 2019;161(2):P255-P6. doi: 10.1177/0194599819858142.
673. Uchiyama T, Fukawa N, Kato A. Long term follow up of intrathecal baclofen therapy for spastic and dystonic patients. Neuromodulation. 2019;22(7):e583. doi: 10.1111/ner.12958.
674. Vidal AC, Sequeira A, Costa C, Pinto S. Neuromodulation and physiotherapy: State of art. Annals of Medicine. 2019;51:S33-S4. doi: 10.1080/07853890.2018.1560076.
675. Vidal AC, Sequeira A, Costa C, Pinto S. Music as an acoustic neuromodulation tool in physiotherapy post-stroke: A case study. Annals of Medicine. 2019;51:S223. doi: 10.1080/07853890.2018.1560735.
676. Violante I, Li L, Sharp D. Theta-tACS normalizes brain network activity in patients with traumatic brain injury. Brain Stimulation. 2019;12(2):498. doi: 10.1016/j.brs.2018.12.629.
677. Wang W, Xu Y. Neuromodulation in minimally conscious state. Neuromodulation. 2019;22(7):e337. doi: 10.1111/ner.12958.
678. Weightman M, Brittain JS, Jenkinson N. Differentiating the roles of the cerebellum and motor cortex during visuomotor adaptation using either hand or whole arm reaching movements. Brain and Neuroscience Advances. 2019;3:134-5. doi:

10.1177/2398212819855490.
679. Yu Y, Yang Y, Gan S, Guo S, Fang J, Wang S, et al. Auditory-function-preserved patients with disorders of consciousness are reversible by transcutaneous auricular vagus nerve stimulation: A pilot study monitored by ASL fMRI. Brain Stimulation. 2019;12(2):388-9. doi: 10.1016/j.brs.2018.12.245.
680. Zewdie E, Ciechanski P, Kuo H, Giuffre A, Kahl C, King R, et al. Safety and tolerability of non-invasive neurostimulation in children. Brain Stimulation. 2019;12(2):550. doi: 10.1016/j.brs.2018.12.817.
681. Zhang Y, Wu S, Xie L, Yu S, Zhang L, Liu C, et al. Ketamine within clinically effective range inhibits glutamate transmission from astrocytes to neurons and disrupts synchronization of astrocytic SICs. Frontiers in Cellular Neuroscience. 2019;13. doi: 10.3389/fncel.2019.00240. PubMed Central PMCID: PMCFujian Gutian(China)
Jiangsu Nhwa(China)
Astra Zeneca(United States).
682. 40th International Symposium on Intensive Care and Emergency Medicine. Critical Care. 2020;24.
683. Ahn HJ, Bashir S, Martin P, Naeser MA, Pascual-Leone A, Yoo WK. cTBS effect on language reorganization verified by fMRI

and chronometry TMS in an aphasia patient. Neurology and Clinical Neuroscience. 2020;8(2):74-8. doi: 10.1111/ncn3.12353.
684. Ayache SS, Chalah MA. Fatigue and affective manifestations in multiple sclerosis—a cluster approach. Brain Sciences. 2020;10(1). doi: 10.3390/brainsci10010010.
685. Bakulin I, Legostaeva L, Poydasheva A, Iazeva E, Sinitsyn D, Sergeev D, et al. TMS-EEG analysis of rTMS efficacy in chronic disorders of consciousness. Clinical Neurophysiology. 2020;131(4):e107. doi: 10.1016/j.clinph.2019.12.276.
686. Bao SC, Khan A, Song R, Tong RKY. Rewiring the lesioned brain: Electrical stimulation for post-stroke motor restoration. Journal of Stroke. 2020;22(1):47-63. doi: 10.5853/jos.2019.03027.
687. Biagi MC, Salvador R, Thibaut A, Martens G, Laureys S, Santarnecchi E, et al. Targeting consciousness brain networks with transcranial current stimulation in minimally conscious state patients: what we gain from a personalized intervention. Clinical Neurophysiology. 2020;131(4):e33-e5. doi: 10.1016/j.clinph.2019.12.150.
688. Bolu A, Gündoğmuş I, Öznur T, Çelik C. Treatment of insomnia with repetitive transcranial magnetic stimulation (Rtms) in a patient with posttraumatic stress disorder (ptsd). Revista de Psiquiatria Clinica. 2020;47(5):157-8. doi:

10.1590/0101-60830000000252.
689. Campanella S, Schroder E, Vanderhasselt MA, Monnart A, Rabbijns M, Kornreich C, et al. Training inhibition through neuromodulation: Combined evidence from tdcs and erps through a go-nogo task in a healthy population. Clinical EEG and Neuroscience. 2020;51(3):NP16. doi: 10.1177/1550059415622686.
690. Campos R, Gomez R, Velarde L. Sacral neuromodulation with Interstim® for urinary voiding dysfunction in post-traumatic brain injury patients. Journal of Urology. 2020;203:e698.
691. Channagiri V, Aziz D, Iannaccone F, Kaufman A. Percutaneous peripheral nerve stimulation for treatment of brachial plexopathy secondary to pancoast tumor: A case report. Interventional Pain Management Reports. 2020;4(5):169-74. doi: 10.36076/PMCR.2020/4/169.
692. Chapman K, Van Roosendaal B, Schrippa F. Unilateral dorsal root ganglion stimulator (drg-s) lead placement with resolution of bilateral lower extremity symptoms in peripheral diabetic neuropathy: A case report. Pain Practice. 2020;20(SUPPL 1):42. doi: 10.1111/papr.12886.
693. Cotner B, Ching D, Nakase-Richardson R, Murphy J, Martin A, Takagishi SC, et al. An Environmental Scan to Identify Current Chronic Pain Treatment Practices for Persons with Traumatic Brain

Injury. Archives of Physical Medicine and Rehabilitation. 2020;101(11):e115. doi: 10.1016/j.apmr.2020.09.350.

694. Duong YN, Kilgard M, Hays S, Rennaker R, Pruitt D, Meyers E, et al. Usability of RePlay: An Automated Upper Limb Rehabilitation System for Brain Injury Tele-rehabilitation. Archives of Physical Medicine and Rehabilitation. 2020;101(11):e104. doi: 10.1016/j.apmr.2020.09.318.

695. Fard MT, Mansouri SS, Jafari A, Vousooghi N. Role of modafinil in the treatment of patients with methamphetamine dependence; An update on randomized, controlled clinical trial. Tropical Journal of Pharmaceutical Research. 2020;19(10):2179-85. doi: 10.4314/tjpr.v19i10.23.

696. Gao BY, Sun CC, Xia GH, Zhou ST, Zhang Y, Mao YR, et al. Paired associated magnetic stimulation promotes neural repair in the rat middle cerebral artery occlusion model of stroke. Neural Regeneration Research. 2020;15(11):2047-56. doi: 10.4103/1673-5374.282266.

697. Giuffre A, Zewdie E, Kuo HC, Carlson H, Kirton A. The effects of conventional and high definition transcranial direct current stimulation on motor learning and robotic Transcranial Magnetic Stimulation (TMS) motor maps in children. Clinical Neurophysiology. 2020;131(4):e147. doi:

10.1016/j.clinph.2019.12.342.

698. Gutiérrez M, Feijóo G, Delucchi LJ. Neurophysiological evaluation of canine congenital hydrocephalus in three dogs. Veterinary Record Case Reports. 2020;8(1). doi: 10.1136/vetreccr-2019-000949.

699. Haddad AF, Young JS, Berger MS, Tarapore PE. Preoperative Applications of Navigated Transcranial Magnetic Stimulation. Frontiers in Neurology. 2020;11. doi: 10.3389/fneur.2020.628903.

700. Harounian J, Berisha D, Mahmood A, Portenoy RK, Bikson M, Datta A, et al. TDCS-telehealth technology and protocol for successful TDCS administration by octogenarians for symptom control in home settings. Neuromodulation. 2020;23(5):e328-e30. doi: 10.1111/ner.13152.

701. Iovino L, Kinsella S, Cooper K, deRoos P, Ugrai TM, Gagnon A, et al. Thymic Reconstitution after Damage Is Influenced By Zinc Status. Biology of Blood and Marrow Transplantation. 2020;26(3):S304-S5. doi: 10.1016/j.bbmt.2019.12.403.

702. Jagtap P, Chawa M, Pasovic V, Gautam M. A Review of Transcranial Magnetic Stimulation for Cognitive Impairment: Evidence for Expansion of Clinical Indications. Brain Stimulation. 2020;13(6):1855. doi: 10.1016/j.brs.2020.06.058.

703. Jagtap P, Chawa M, Pasovic V, Gautam M. A review of

non-invasive brain stimulation for cognitive impairment: Evidence for novel and widespread applications. Neuromodulation. 2020;23(3):e67-e8. doi: 10.1111/ner.13133.
704. Janicak P, Heart K, McGugan B. Post market rate of seizures during TMS treatment with NeuroStar system appears to be lower than previously estimated. CNS Spectrums. 2020;25(2):306. doi: 10.1017/S1092852920000188.
705. Jing Z, Ling Y, Yi F. Multipoint transcutaneous electrical stimulation reduces median effective plasma concentration of propofol: A randomised clinical trial. Indian Journal of Anaesthesia. 2020;64(6):483-7. doi: 10.4103/ija.IJA_775_19.
706. Kaboodvand N, Griffiths J. Influence of anatomical connectivity and intrinsic dynamics in a connectome based neural mass model of TMS-evoked potentials. BMC Neuroscience. 2020;21(SUPPL 1). doi: 10.1186/s12868-020-00593-1.
707. Khaleghi A, Zarafshan H, Vand SR, Mohammadi MR. Effects of Non-invasive Neurostimulation on Autism Spectrum Disorder: A Systematic Review. Clinical Psychopharmacology and Neuroscience. 2020;18(4):527-52. doi: 10.9758/CPN.2020.18.4.527.
708. Kinfe T, Buchfelder M. Incisionless MR-guided focused ultrasound deep brain modulation: Technical considerations and

current therapeutic approaches in psychiatric disorders. Clinical Neurophysiology. 2020;131(4):e188-e9. doi: 10.1016/j.clinph.2019.12.030.

709. Knoll RM, Lubner RJ, Remenschneider AK, Kozin ED. Histopathology of audiovestibular dysfunction following head injury: A case report. Otolaryngology - Head and Neck Surgery. 2020;163(1 SUPPL):P233. doi: 10.1177/0194599820934781.

710. Kotob M. Adding illness to injury: A complicated football case. Clinical Journal of Sport Medicine. 2020;30(2):e44-e5. doi: 10.1097/JSM.0000000000000837.

711. Kretzschmar M, Reining M. Opioid withdrawal during DRGS treatment of neuropathic pain after PNI: 36-months followup in 21 patients. Neuromodulation. 2020;23(3):e23. doi: 10.1111/ner.13133.

712. Kutzer T, Dick M, Scudamore T, Wiener M, Schwartz T. Antidepressant efficacy and side effect burden: An updated guide for clinicians. Drugs in Context. 2020;9. doi: 10.7573/DIC.2020-2-2.

713. Luneva I, Ryabinkina J, Piradov MA. Locked-in syndrome after bacterial ventriculitis. European Journal of Neurology. 2020;27:1180.

714. Ma XJ, Cheng OM, Xiao H, Song D, Jiang QS, Qiu H.

Transcranial direct current stimulation promotes hippocampal neurogenesis in mice with cerebral ischemia involving up-regulation of nmda receptors. Chinese Pharmacological Bulletin. 2020;36(2):175-81. doi: 10.3969/j.issn.1001-1978.2020.02.07.

715. Nasios G, Bakirtzis C, Messinis L. Cognitive Impairment and Brain Reorganization in MS: Underlying Mechanisms and the Role of Neurorehabilitation. Frontiers in Neurology. 2020;11. doi: 10.3389/fneur.2020.00147.

716. Petrova M. Craniocerebral hypothermia as a neuromodulation method in chronically critically ill patients. Critical Care. 2020;24. doi: 10.1186/s13054-020-2772-3.

717. Pichini S, Di Trana A, Torrens M, Scherbaum N, Zaami S. Editorial: New Trends of Substance Abuse: Looking for New Psychotropic Effects of Chem Sex Drugs, Cognitive Enhancers, and New Psychoactive Substances. Frontiers in Psychiatry. 2020;11. doi: 10.3389/fpsyt.2020.612192.

718. Poydasheva A, Sergeeva A, Kremneva E, Morozova S, Legostaeva L, Bakulin I, et al. Functional connectivity changes after rTMS over angular gyrus in chronic DOC patients. Clinical Neurophysiology. 2020;131(4):e129-e30. doi: 10.1016/j.clinph.2019.12.314.

719. Read J, Kirsch I, McGrath L. Electroconvulsive Therapy for

Depression: A Review of the Quality of ECT versus Sham ECT Trials and Meta-Analyses. Ethical Human Psychology and Psychiatry. 2020;21(2):64-103. doi: 10.1891/EHPP-D-19-00014.

720. Rojas SS, Veiga VC, Carvalho J, Lisboa AC, Ordinola AA, Nogueira CP, et al. Non-invasive brain compliance findings in patients with COVID-19 in a Brazilian intensive care unit. Critical Care. 2020;24(SUPPL 2). doi: 10.1186/s13054-020-03187-9.

721. Rudroff T, Workman CD, Fietsam AC, Boles Ponto LL. Imaging transcranial direct current stimulation (TDCS) with positron emission tomography (PET). Brain Sciences. 2020;10(4). doi: 10.3390/brainsci10040236.

722. San Luciano M, Viehoever A, Bledsoe I, Dodenhoff K, Gittings M, Viser A, et al. Thalamic deep brain stimulation for acquired dystonia in children and young adults: A phase i clinical trial. Movement Disorders. 2020;35(SUPPL 1):S46. doi: 10.1002/mds.28268.

723. Santiago JE, Cameron AP, Navarrete RA. Addressing Sphincter Dysfunction in the Female with Neurogenic Lower Urinary Tract Dysfunction. Current Bladder Dysfunction Reports. 2020;15(3):142-52. doi: 10.1007/s11884-020-00597-x.

724. Scott E, Kidgell DJ, Frazer AK, Pearce AJ. The Neurophysiological Responses of Concussive Impacts: A

Systematic Review and Meta-Analysis of Transcranial Magnetic Stimulation Studies. Frontiers in Human Neuroscience. 2020;14. doi: 10.3389/fnhum.2020.00306.

725. Sendra C, Salvador R, Lekadir K, Ruffini G, Pedro JB. StarNEt: A convolutional neural network for automated white and gray matter segmentation from magnetic resonance imaging in the human brain. Clinical Neurophysiology. 2020;131(4):e55-e6. doi: 10.1016/j.clinph.2019.12.190.

726. Siddiqi SH, Horn A, Schaper F, Hsu J, Padmanabhan J, Cash RFH, et al. A convergent circuit for depression across brain lesions and brain stimulation. Journal of Neuropsychiatry and Clinical Neurosciences. 2020;32(3):e22.

727. Simon-Martinez C, Decraene L, Van Hoof E, Zielinski I, Hoare B, Steenbergen B, et al. The impact of brain lesion features and corticospinal tract wiring pattern on mirror movement characteristics in children with unilateral cerebral palsy. Developmental Medicine and Child Neurology. 2020;62(SUPPL 4):32-3. doi: 10.1111/dmcn.14688.

728. Simon-Martinez C, Mailleux L, Hoskens J, Ortibus E, Jaspers E, Wenderoth N, et al. Randomized controlled trial combining constraint-induced movement therapy and action-observation training in unilateral cerebral palsy: clinical effects and influencing

factors of treatment response. Therapeutic Advances in Neurological Disorders. 2020;13. doi: 10.1177/1756286419898065.
729. Singla RK, Guimarães AG, Zengin G. Editorial: Application of Plant Secondary Metabolites to Pain Neuromodulation. Frontiers in Pharmacology. 2020;11. doi: 10.3389/fphar.2020.623399.
730. Sollmann N, Fratini A, Zhang H, Zimmer C, Meyer B, Krieg SM. Associations between clinical outcome and tractography based on navigated transcranial magnetic stimulation in patients with language-eloquent brain lesions. Journal of Neurosurgery. 2020;132(4):1033-42. doi: 10.3171/2018.12.JNS182988.
731. Stevenson R, Samokhina E, Rossetti I, Morley JW, Buskila Y. Neuromodulation of Glial Function During Neurodegeneration. Frontiers in Cellular Neuroscience. 2020;14. doi: 10.3389/fncel.2020.00278.
732. Talmasov D, Cotovio G, Barahona-Corrêa JB, Hsu J, Senova S, Ribeiro R, et al. Lesion network mapping of mania symptoms caused by focal brain lesions. Annals of Neurology. 2020;88(SUPPL 25):S70-S1. doi: 10.1002/ana.25865.
733. Talmasov D, Cotovio G, Bernardo BarahonaCorrêa J, Hsu J, Senova S, Ribeiro R, et al. Network localization of mania symptoms based on focal bain lesions. Journal of Neuropsychiatry

and Clinical Neurosciences. 2020;32(3):e23.

734. Tegeler CL, Howard LJ, Brown KL, Kellar DC, Shaltout HA, Gerdes L, et al. Exploratory use of artificial intelligence driven acoustic neuromodulation improved sleep, depression, anxiety, and stress in adults with persisting post-concussion symptoms. Sleep. 2020;43(SUPPL 1):A207-A8. doi: 10.1093/sleep/zsaa056.539.

735. Texakalidis P, Tora M, McMahon T, Greven A, Anthony C, Boulis N. Percutaneous trigeminal ganglion stimulation for intractable facial pain. Journal of Neurosurgery. 2020;132(4):97. doi: 10.3171/2020.4.JNS.AANS2020abstracts.

736. Tiotiu A, Badi Y, Kermani NZ, Hansbro P, Wheelock C, Dahlen SE, et al. Differential mast cell activation by transcriptomic signature analysis in the U-BIOPRED severe asthma cohort. European Respiratory Journal. 2020;56. doi: 10.1183/13993003.congress-2020.3827.

737. Trapp N, Bruss J, Abu-Halawa O, Li S, Albazron F, Manzel K, et al. Large-scale lesion symptom mapping of depression: Anatomy of risk and resilience. Neuropsychopharmacology. 2020;45:316-7. doi: 10.1038/s41386-020-00892-5.

738. 2021 American Neuropsychiatric Association Annual Meeting Abstracts. Journal of Neuropsychiatry and Clinical Neurosciences. 2021;33(3).

739. Anasobi AT, Macel LP, Hirsch AR. Inhibition of hallucinations through electrical stimulation of the cerebellum. Annals of Neurology. 2021;90(SUPPL 27):S40. doi: 10.1002/ana.26180.
740. Asimakidou E, Abut PA, Raabe A, Seidel K. Motor evoked potential warning criteria in supratentorial surgery: A scoping review. Cancers. 2021;13(11). doi: 10.3390/cancers13112803.
741. Chakraborty S, Saetta G, Simon C, Lenggenhager B, Ruddy K. Could Brain–Computer Interface Be a New Therapeutic Approach for Body Integrity Dysphoria? Frontiers in Human Neuroscience. 2021;15. doi: 10.3389/fnhum.2021.699830.
742. Chan H, Hogue O, Newcomb M, Hermann J, Floden D, Machado A, et al. Prefrontal improvement by deep cerebellar stimulation in a traumatic brain injury model of rat. Brain Stimulation. 2021;14(6):1714-5. doi: 10.1016/j.brs.2021.10.416.
743. Chen S, Xu D, Fan L, Fang Z, Wang X, Li M. Roles of N-Methyl-D-Aspartate Receptors (NMDARs) in Epilepsy. Frontiers in Molecular Neuroscience. 2021;14. doi: 10.3389/fnmol.2021.797253.
744. Chiang HS, Kraut M, Vanneste S, Hart J. Modulation of the superior medial frontal cortex using anodal high definition transcranial direct current stimulation affects frontal midline theta oscillations during a Go-Nogo task in veterans with chronic

traumatic brain injury. Brain Stimulation. 2021;14(6):1635. doi: 10.1016/j.brs.2021.10.152.
745. Chou P, Kuo CC. Anticonvulsant vs. Proconvulsant Effect of in situ Deep Brain Stimulation at the Epileptogenic Focus. Frontiers in Systems Neuroscience. 2021;15. doi: 10.3389/fnsys.2021.607450.
746. Deli A, He S, Huang Y, Martin S, Aziz T, Denison T, et al. Entraining Human Wakefulness: Selective, Closed-Loop Stimulation of Brainstem Arousal Circuits. Stereotactic and Functional Neurosurgery. 2021;99(SUPPL 1):109. doi: 10.1159/000520618.
747. Deltombe T, Schillebeeckx F, Ip A, Schinwelski M, Teixeira JM, Ashford S, et al. Worldwide clinician survey on practice patterns and perceptions on use of adjunct therapies following botulinum toxin injection for limb spasticity. Toxicon. 2021;190:S15-S6. doi: 10.1016/j.toxicon.2020.11.371.
748. Dolev I. Delphi in the detection of neurological conditions and white matter pathologies. Journal of the Neurological Sciences. 2021;429. doi: 10.1016/j.jns.2021.118327.
749. Engelhardt M, Karhu J, Picht T. Protocol for mapping of the supplementary motor area using repetitive navigated transcranial magnetic stimulation. Brain Stimulation. 2021;14(6):1670. doi:

10.1016/j.brs.2021.10.261.

750. Fatih P, Kucuker MU, Vande Voort JL, Doruk Camsari D, Farzan F, Croarkin PE. A Systematic Review of Long-Interval Intracortical Inhibition as a Biomarker in Neuropsychiatric Disorders. Frontiers in Psychiatry. 2021;12. doi: 10.3389/fpsyt.2021.678088.

751. Fernández-Galán E, Rodriguez M, García De Herreros M, Pineda E, Martínez D, Nomdedeu M, et al. Role of s100 in the diagnosis of bone marrow metastasis from glioblastoma: A case report. Clinical Chemistry and Laboratory Medicine. 2021;59(SUPPL 1):S268. doi: 10.1515/cclm-2021-5013.

752. Gracefire P. Advances in photobiomodulation using a closed-loop design. NeuroRegulation. 2021;8(4):203-4. doi: 10.15540/nr.8.4.198.

753. Granero OP, Cheema S, Lagrata S, Hyam J, Zrinzo L, Matharu M, et al. Long-Term Outcomes of Ventral Tegmental Area Deep Brain Stimulation for Trigeminal Autonomic Cephalalgias. Stereotactic and Functional Neurosurgery. 2021;99(SUPPL 1):128. doi: 10.1159/000520618.

754. Hall MR, Mathiasen R. Phonate a friend. Clinical Journal of Sport Medicine. 2021;31(2):e35. doi: 10.1097/JSM.0000000000000917.

755. Hara T, Shanmugalingam A, McIntyre A, Burhan A. The Effect of Non-Invasive Brain Stimulation (NIBS) on Executive Functioning, Attention and Memory in Rehabilitation Patients with Traumatic Brain Injury: a Systematic Review and Meta-analysis. Brain Stimulation. 2021;14(6):1716-7. doi: 10.1016/j.brs.2021.10.422.
756. Hardy DJ. Neuroergonomics: A perspective from neuropsychology, with a proposal about workload. Brain Sciences. 2021;11(5). doi: 10.3390/brainsci11050647.
757. Hartikainen KM. Emotion-attention interaction in the right hemisphere. Brain Sciences. 2021;11(8). doi: 10.3390/brainsci11081006.
758. Hays S, Swank C, Rennaker R, French J. Vagus nerve stimulation paired with rehabilitation to improve recovery of upper extremity function in stroke and spinal cord injury. Neurorehabilitation and Neural Repair. 2021;35(11):NP16. doi: 10.1177/15459683211027047.
759. Herrold A, Livengood S, Siddiqi S, Pape TB. Customizing rTMS treatment targeting for co-occurring alcohol use disorder and mild traumatic brain injury using multi-modal neuroimaging data as neurobiological markers. Brain Stimulation. 2021;14(6):1721. doi: 10.1016/j.brs.2021.10.437.

760. Huang LD, Sung KB. COVID-encephalopathy treatment with transcranial near infrared laser stimulation. Brain Stimulation. 2021;14(6):1660. doi: 10.1016/j.brs.2021.10.227.
761. Hunt S, Moss H, Dancy M, Badran BW, George MS, Jenkins D. Predicting response to transcutaneous auricular vagus nerve stimulation (taVNS) paired with oromotor feeding in neonates: CNS metabolite biomarkers by MR spectroscopy (MRS). Brain Stimulation. 2021;14(6):1664-5. doi: 10.1016/j.brs.2021.10.243.
762. Isagulyan E, Tomsky A, Makashova E, Mikhailova V, Dorochov E, Salova E. Neuromodulation in Treating Complex Regional Pain Syndrome. Stereotactic and Functional Neurosurgery. 2021;99(SUPPL 1):119. doi: 10.1159/000520618.
763. Jung JY. The neural mechanisms of plasticity in semantic cognition: Variable neuro-displacement hypothesis. Asia-Pacific Psychiatry. 2021;13(SUPPL 1). doi: 10.1111/appy.12459.
764. Kahana M, Wanda P, Ezzyat Y, Adamovich-Zeitlin R, Lega B, Jobst B, et al. Closed-Loop Neuromodulation Restores Memory after Traumatic Brain Injury. Neuromodulation. 2021;24(4):e249-e50. doi: 10.1111/ner.13385.
765. Kang X, Herron T, Coetzee J, Adamson M. Effect of rTMS on fractional anisotropy, radial diffusivity, and axonal volume in select fiber tracts in patients with traumatic brain injury. Brain

Stimulation. 2021;14(6):1639. doi: 10.1016/j.brs.2021.10.164.

766. Kavakcı M. Efficacy of non-invasive brain stimulation methods for the treatment of cognitive impairment in traumatic brain injury and stroke. Turk Noroloji Dergisi. 2021;27(1):17-20. doi: 10.4274/tnd.2021.90688.

767. Kierońska S, Świtońska M, Meder G, Piotrowska M, Sokal P. Tractography alterations in the arcuate and uncinate fasciculi in post-stroke aphasia. Brain Sciences. 2021;11(1):1-12. doi: 10.3390/brainsci11010053.

768. Kinfe T, Buchfelder M. Incisionless MR-Guided Focused Ultrasound: Technical Considerations and Current Approaches in Psychiatric Disorders. Stereotactic and Functional Neurosurgery. 2021;99(SUPPL 1):132-3. doi: 10.1159/000520618.

769. Köhnen M, Kriston L, Härter M, Baumeister H, Liebherz S. Effectiveness and acceptance of technology-based psychological interventions for the acute treatment of unipolar depression: Systematic review and meta-analysis. Journal of Medical Internet Research. 2021;23(6). doi: 10.2196/24584.

770. Lam D, Bogguri C, Cadena J, Ladd A, Sebastian A, Enright H, et al. Do reprogrammed brain cells hold the key to healing damaged networks after traumatic brain injury? Journal of Neurotrauma. 2021;38(14):A70. doi:

10.1089/neu.2021.29111.abstracts.
771. Larsen J, Güler DB, Andersen MS, Rich K, Damm MV, Zhang M, et al. Plasticity of cholinergic system in the spinal cord following traumatic brain injury. Clinical Neuropathology. 2021;40(4):S107-S8. doi: 10.5414/NPP40M01.
772. Leisman G, Machado C, Melillo R. Cortical visual impairment in childhood: 'blindsight' and the sprague effect revisited. Brain Sciences. 2021;11(10). doi: 10.3390/brainsci11101279.
773. Leung A. TMS for Persistent Post Traumatic Brain Injury Headache-A neuropathic Pain State. Brain Stimulation. 2021;14(6):1730. doi: 10.1016/j.brs.2021.10.470.
774. Li R. Study on the Process of Consciousness Recovery in DOC Patients Based on Microstate. International Journal of Psychophysiology. 2021;168:S204. doi: 10.1016/j.ijpsycho.2021.07.554.
775. Li Y, He J, Yang B, Zhang H, Yang Z, Fu J, et al. Guidelines of clinical diagnosis and neurorestorative treatment for chronic disorders of consciousness (2021 China version). Journal of Neurorestoratology. 2021;9(1):50-9. doi: 10.26599/JNR.2021.9040006.
776. Magnusson JL, Leventhal DK. Revisiting the "Paradox of Stereotaxic Surgery": Insights Into Basal Ganglia-Thalamic

Interactions. Frontiers in Systems Neuroscience. 2021;15. doi: 10.3389/fnsys.2021.725876.

777. Manto M, Kakei S, Mitoma H. The critical need to develop tools assessing cerebellar reserve for the delivery and assessment of non-invasive cerebellar stimulation. Cerebellum and Ataxias. 2021;8(1). doi: 10.1186/s40673-020-00126-w.

778. Mayorova LA, Petrova MV, Pryanikov IV, Grechko AV. Chronic Disorders of Consciousness: Diagnosis and Prognosis. Neuroscience and Behavioral Physiology. 2021;51(8):1132-47. doi: 10.1007/s11055-021-01173-4.

779. Molero-Chamizo A, Salas Sánchez Á, Álvarez Batista B, Cordero García C, Andújar Barroso R, Rivera-Urbina GN, et al. Bilateral Motor Cortex tDCS Effects on Post-Stroke Pain and Spasticity: A Three Cases Study. Frontiers in Pharmacology. 2021;12. doi: 10.3389/fphar.2021.624582.

780. Monti M. Ultrasonic stimulation in disorders of consciousness. Brain Stimulation. 2021;14(6):1746-7. doi: 10.1016/j.brs.2021.10.532.

781. Mosiello G, Safder S, Marshall D, Rolle U, Benninga MA. Neurogenic bowel dysfunction in children and adolescents. Journal of Clinical Medicine. 2021;10(8). doi: 10.3390/jcm10081669.

782. Mota F, Ruiz-Bedoya C, Tucker E, De Jesus P, Flavahan K,

Turner M, et al. Noninvasive Assessment of Intralesional Antimicrobial Concentration-Time Profiles in Pulmonary and Central Nervous System Tuberculosis using Dynamic 18F-Pretomanid Positron Emission Tomography. Open Forum Infectious Diseases. 2021;8(SUPPL 1):S789-S90. doi: 10.1093/ofid/ofab466.1603.

783. Munir A, Rymowicz R, Joshi P. A rare case of gamma-butyrolactone (GBL) withdrawal syndrome. American Journal on Addictions. 2021;30(3):238-9. doi: 10.1111/ajad.13173.

784. Muttalib F, Fonseca Y, Stein P, Doctor A. The association between baseline heart rate variability and neurological outcome among children hospitalized due to cerebral malaria in malawi: A pilot cohort study. Pediatric Critical Care Medicine. 2021;22(SUPPL 1):49. doi: 10.1097/01.pcc.0000738448.18543.3b.

785. Noda Y. Clinical neurophysiology, neuroimaging, and neuromodulation of neuropsychiatric disorders. Journal of Personalized Medicine. 2021;11(11). doi: 10.3390/jpm11111193.

786. Orakpo N, Vieux U, Castro-Nuñez C. Case Report: Virtual Reality Neurofeedback Therapy as a Novel Modality for Sustained Analgesia in Centralized Pain Syndromes. Frontiers in Psychiatry. 2021;12. doi: 10.3389/fpsyt.2021.660105.

787. Ortelli P, Ferrazzoli D, Sebastianelli L, Maestri R, Dezi S,

Spampinato D, et al. Fatigue and “brain fog” in the aftermath of mild COVID-19: A neuropsychological and TMS study. Journal of the Neurological Sciences. 2021;429. doi: 10.1016/j.jns.2021.119854.

788. Pan J, Wu J, Liu J, Wu J, Wang F. A systematic review of sleep in patients with disorders of consciousness: From diagnosis to prognosis. Brain Sciences. 2021;11(8). doi: 10.3390/brainsci11081072.

789. Pape TB. Preliminary evaluation of the clinical utility of miRNA as neurobiological markers of TBI diagnosis and rTMS treatment responsiveness. Brain Stimulation. 2021;14(6):1721. doi: 10.1016/j.brs.2021.10.438.

790. Pascarella A, Manzo L, Estraneo A. Long-term clinical, neurophysiological and neuroimaging follow-up of a patient with hemiparkinson-hemiatrophy syndrome. Journal of the Neurological Sciences. 2021;429. doi: 10.1016/j.jns.2021.119461.

791. Rezai A, Ranjan M, D'Haese PF, Haut M, Carpenter J, Najib U, et al. Opening of Blood Brain Barrier (BBB) in the Hippocampus and Entorhinal Cortex in Early Alzheimer's Disease with Focused Ultrasound (FUS). Stereotactic and Functional Neurosurgery. 2021;99(SUPPL 1):105-6. doi: 10.1159/000520618.

792. Rosanova M, Sarasso S. Sleep-like cortical bistability after

focal and multifocal brain lesions. Brain Stimulation. 2021;14(6):1743. doi: 10.1016/j.brs.2021.10.518.

793. Rouwhorst R, Dijkstra E, Van Oostrom I, Zwienenberg L, Sack A, Van Dijk H, et al. Vasovagal syncope as a specific side effect of rTMS: A frontal-vagal dose-finding study. Brain Stimulation. 2021;14(6):1648. doi: 10.1016/j.brs.2021.10.194.

794. Saleem G, Slomine B, Suskauer S. Transcranial Direct Current Stimulation Feasibility Challenges in Youth with Severe Brain Injury: A Case Report. Neuromodulation. 2021;24(4):e159. doi: 10.1111/ner.13385.

795. Sanesteban Beceiro E, Mayo Rodríguez P, García Yepes M, Gómez Esteban A, Cuadrado Pérez M, Porta-Etessam J, et al. Secondary Cluster Headache: Description of five cases. European Journal of Neurology. 2021;28(SUPPL 1):269. doi: 10.1111/ene.14968.

796. Santaniello S, McConnell GC, Gale JT, Faghih RT, Kemere C, Hilliard JD, et al. Editorial: Towards the Next Generation of Deep Brain Stimulation Therapies: Technological Advancements, Computational Methods, and New Targets. Frontiers in Neuroscience. 2021;15. doi: 10.3389/fnins.2021.737737.

797. Schaper F, Nordberg J, Cohen A, Hsu J, Lin C, Ferguson M, et al. An epilepsy network derived from human brain lesions and deep

brain stimulation. Brain Stimulation. 2021;14(6):1643-4. doi: 10.1016/j.brs.2021.10.178.

798. Sheline Y, Balderston N, Oathes D, Makhoul W, Duprat R, Beer J, et al. Intensive tms in treatment refractory patients with major depressive disorder: A pilot replication. Neuropsychopharmacology. 2021;46:284-5. doi: 10.1038/s41386-021-01237-6.

799. Siddiqi S. Precision targeting of brain networks in TBI. Brain Stimulation. 2021;14(6):1721-2. doi: 10.1016/j.brs.2021.10.439.

800. Siddiqi S. Connectome-targeted neuromodulation across symptoms and disorders. Brain Stimulation. 2021;14(6):1712. doi: 10.1016/j.brs.2021.10.407.

801. Storch S, Samantzis M, Balbi M. Driving Oscillatory Dynamics: Neuromodulation for Recovery After Stroke. Frontiers in Systems Neuroscience. 2021;15. doi: 10.3389/fnsys.2021.712664.

802. Vesia M. Targeting brain circuits for goal-directed movements with multi-focal transcranial magnetic stimulation. Brain Stimulation. 2021;14(6):1713. doi: 10.1016/j.brs.2021.10.410.

803. Villagomez A, Borja T, Pontón P, Barba P, Chiliquinga A, Yambarela I, et al. HISTOLOGICAL AND MICROBIOLOGICAL EVALUATION IN MICE OF CYANOACRYLATE

(HISTOACRYL) AS POSSIBLE REGENERATIVE AGENT TO REPLACE TRADITIONAL SUTURES. Cytotherapy. 2021;23(4):17. doi: 10.1016/j.jcyt.2021.02.052.
804. Voinescu A, Sui J, Stanton Fraser D. Virtual reality in neurorehabilitation: An umbrella review of meta-analyses. Journal of Clinical Medicine. 2021;10(7). doi: 10.3390/jcm10071478.
805. Wambaugh JL. An expanding apraxia of speech (AOS) treatment evidence base: An update of recent developments. Aphasiology. 2021;35(4):442-61. doi: 10.1080/02687038.2020.1732289.
806. Wampler S, Fuertes C, Reck A, Haar CV. Brain injury increases impulsivity on the rodent gambling task but is not treated by neuromodulation in female rats. Journal of Neurotrauma. 2021;38(14):A30. doi: 10.1089/neu.2021.29111.abstracts.
807. Wileczek A, Reichert A, Kluk M, Sledz S, Sledz A, Kosior J, et al. Non-invasive and invasive autonomic tests to facilitate cardioneuroablation and complex indications for transcutaneous lead extraction and discontinuation of permanent pacing. Europace. 2021;23(SUPPL 3):iii538. doi: 10.1093/europace/euab116.490.
808. Xing G, Zhang Y, Zhang Y, Adamson MM, Furst AJ, Ashford JW. Editorial: Evidence-Based Anti-Inflammatory, Anti-Hypoperfusion and Anti-Anxiety/Insomnia Therapies Show

Promises for TBI-Induced Post-Traumatic Symptoms and Cognitive Deficits: Advances in Diagnosis and Treatment of TBI-Induced Neurodegeneration and Cognitive Deficits. Frontiers in Neurology. 2021;12. doi: 10.3389/fneur.2021.695629.

809. Zettin M, Bondesan C, Nada G, Varini M, Dimitri D. Transcranial Direct-Current Stimulation and Behavioral Training, a Promising Tool for a Tailor-Made Post-stroke Aphasia Rehabilitation: A Review. Frontiers in Human Neuroscience. 2021;15. doi: 10.3389/fnhum.2021.742136.

810. Zimdahl JW, Thomas H, Bolland SJ, Leggett K, Barry KM, Rodger J, et al. Excitatory Repetitive Transcranial Magnetic Stimulation Over Prefrontal Cortex in a Guinea Pig Model Ameliorates Tinnitus. Frontiers in Neuroscience. 2021;15. doi: 10.3389/fnins.2021.693935.

811. D'urso G, Toscano E, Sanges V, Sauvaget A, Sheffer CE, Riccio MP, et al. Cerebellar transcranial direct current stimulation in children with autism spectrum disorder: A pilot study on efficacy, feasibility, safety, and unexpected outcomes in tic disorder and epilepsy. Journal of Clinical Medicine. 2022;11(1). doi: 10.3390/jcm11010143.

812. Kornfeld S, Kalambaheti E, Antonucci MM. Rehabilitation of persistent symptoms and neurocognitive deficits following sports

related concussion in a professional hockey player: Case study. Neurology. 2022;98(1 SUPPL). doi: 10.1212/01.wnl.0000801980.87446.22.

(3) Web of science (762 studies)

1. Obeso JA, Teijeira JM, Marti-Masso JF, Iragui MI, Maravi E, Carrera N, et al. Somatosensory evoked potentials: abnormalities in brainstem lesions. Electromyography and clinical neurophysiology. 1979;19(5):417-23. PubMed PMID: MEDLINE:527543.

2. Zeitlhofer J, Steiner M, Zadrobilek E, Hausl E, Sporn P, Asenbaum S, et al. Evoked potentials in the follow-up and prognosis of patients with craniocerebral trauma. Der Anaesthesist. 1989;38(1):10-5. PubMed PMID: MEDLINE:2919747.

3. Maccabee PJ, Eberle L, Amassian VE, Cracco RQ, Rudell A, Jayachandra M. Spatial distribution of the electric field induced in volume by round and figure '8' magnetic coils: relevance to activation of sensory nerve fibers. Electroencephalography and clinical neurophysiology. 1990;76(2):131-41. doi: 10.1016/0013-4694(90)90211-2. PubMed PMID: MEDLINE:1697241.

4. Wagner W. SEP testing in deeply comatose and brain dead patients: the role of nasopharyngeal, scalp and earlobe derivations in recording the P14 potential. Electroencephalography and clinical neurophysiology. 1991;80(5):352-63. doi: 10.1016/0168-5597(91)90082-9. PubMed PMID: MEDLINE:1716559.

5. Gilmore R. Somatosensory evoked potential testing in infants and children. Journal of clinical neurophysiology : official publication of the American Electroencephalographic Society. 1992;9(3):324-41. doi: 10.1097/00004691-199207010-00002. PubMed PMID: MEDLINE:1517402.

6. Guerit JM, Baele P, de Tourtchaninoff M, Soveges L, Dion R. Somatosensory evoked potentials in patients undergoing circulatory arrest under profound hypothermia. Neurophysiologie clinique = Clinical neurophysiology. 1993;23(2-3):193-208. doi: 10.1016/s0987-7053(05)80230-8. PubMed PMID: MEDLINE:8326930.

7. Rappaport M, Leonard J, Ruiz Portillo S. Somatosensory evoked potential peak latencies and amplitudes in contralateral and ipsilateral hemispheres in normal and severely traumatized brain-injured subjects. Brain injury. 1993;7(1):3-13. doi: 10.3109/02699059309008152. PubMed PMID: MEDLINE:8381044.

8. Robertson CS, Gopinath SP, Chance B. A new application for near-infrared spectroscopy: detection of delayed intracranial hematomas after head injury. Journal of neurotrauma. 1995;12(4):591-600. doi: 10.1089/neu.1995.12.591. PubMed PMID: MEDLINE:8683610.

9. Konig F, Loble M, Wolfersdorf M. Depressive stupor--malignant neuroleptic syndrome--serotonin syndrome. A case contribution to a difficult differential diagnosis. Der Nervenarzt. 1996;67(5):407-12. PubMed PMID: MEDLINE:9005353.

10. Triplett G, Hill C, Freeman L, Rajan U, Templer DI. Incidence of head injury: lasting effects among college students and working adults in the general population. Perceptual and motor skills. 1996;83(3 Pt 2):1344-6. doi: 10.2466/pms.1996.83.3f.1344. PubMed PMID: MEDLINE:9017750.

11. de Sousa RM, Regis FC, Koizumi MS. Traumatic brain injury: differences among pedestrians and motor vehicle occupants. Revista de saude publica. 1999;33(1):85-94. doi: 10.1590/s0034-89101999000100011. PubMed PMID: MEDLINE:10436625.

12. Ellaway PH, Moosavi SH, Stokes MJ, Catley M, Haque N. Indices of cortical motor function following severe brain injury in man. In: Binder MD, editor. Peripheral and Spinal Mechanisms in the Neural Control of Movement. Progress in Brain Research. 1231999. p. 473-9.

13. Ilmoniemi RJ, Ruohonen J, Karhu J. Transcranial magnetic stimulation - A new tool for functional imaging of the brain.

Critical Reviews in Biomedical Engineering. 1999;27(3-5):241-84. PubMed PMID: WOS:000087536900005.

14. Liepert J, Weiller C. Mapping plastic brain changes after acute lesions. Current Opinion in Neurology. 1999;12(6):709-13. doi: 10.1097/00019052-199912000-00009. PubMed PMID: WOS:000167920600009.

15. Murakami H, Harada H, Saito Y. Design and analysis of Ni-base superalloys on an atomistic basis. Journal of the Japan Institute of Metals. 1999;63(6):723-32. doi: 10.2320/jinstmet1952.63.6_723. PubMed PMID: WOS:000081359600008.

16. Robertson IH, Murre JMJ. Rehabilitation of brain damage: Brain plasticity and principles of guided recovery. Psychological Bulletin. 1999;125(5):544-75. doi: 10.1037/0033-2909.125.5.544. PubMed PMID: WOS:000082379600003.

17. Rohde V, Irle S, Hassler WE. Prediction of the post-comatose motor function by motor evoked potentials obtained in the acute phase of traumatic and non-traumatic coma. Acta Neurochirurgica. 1999;141(8):841-8. doi: 10.1007/s007010050385. PubMed PMID: WOS:000082257500012.

18. Roncucci P, Lepori P, Mok MS, Bayat A, Logi F, Marino A. Nasopharyngeal electrode recording of somatosensory evoked

potentials as an indicator in brain death. Anaesthesia and Intensive Care. 1999;27(1):20-5. doi: 10.1177/0310057x9902700104. PubMed PMID: WOS:000078637700004.
19. Rytz R, Hoffmann R. Chemical bonding in the ternary transition metal bismuthides Ti4TBi2 with T = Cr, Mn, Fe, Co, and Ni. Inorganic Chemistry. 1999;38(7):1609-17. doi: 10.1021/ic981075v. PubMed PMID: WOS:000079620400039.
20. Stohr M, Hartmann M. Evoked potentials: New clinical applications. Aktuelle Neurologie. 1999;26(4):147-51. doi: 10.1055/s-2007-1017624. PubMed PMID: WOS:000081194000001.
21. Taub E, Uswatte G, Pidikiti R. Constraint-Induced Movement Therapy: A new family of techniques with broad application to physical rehabilitation - A clinical review. Journal of Rehabilitation Research and Development. 1999;36(3):237-51. PubMed PMID: WOS:000085584900011.
22. Chen WJ, Li CC. Oceanic rain rate retrievals using TRMM microwave imager multi-channel brightness temperatures during the 1998 SCSMEX. Terrestrial Atmospheric and Oceanic Sciences. 2000;11(4):765-88. doi: 10.3319/tao.2000.11.4.765(a). PubMed PMID: WOS:000166314400002.
23. Couper FJ, Logan BK. Determination of

gamma-hydroxybutyrate (GHB) in biological specimens by cas chromatography - Mass spectrometry. Journal of Analytical Toxicology. 2000;24(1):1-7. doi: 10.1093/jat/24.1.1. PubMed PMID: WOS:000084875200001.
24. Gomez-Fernandez L. Cortical plasticity and neurological functional restoring: An update. Revista De Neurologia. 2000;31(8):749-56. doi: 10.33588/rn.3108.2000358. PubMed PMID: WOS:000165189400012.
25. Oliveri M, Rossini PM, Filippi MM, Traversa R, Cicinelli P, Palmieri MG, et al. Time-dependent activation of parieto-frontal networks for directing attention to tactile space - A study with paired transcranial magnetic stimulation pulses in right-brain-damaged patients with extinction. Brain. 2000;123:1939-47. doi: 10.1093/brain/123.9.1939. PubMed PMID: WOS:000089070100016.
26. Pilipenko P. Study of the pyramidal tract in comatose patients by transcranial magnetic stimulation. Brain Pathology. 2000;10(4):654-5. PubMed PMID: WOS:000088213000347.
27. Schwarz S, Hacke W, Schwab S. Magnetic evoked potentials in neurocritical care patients with acute brainstem lesions. Journal of the Neurological Sciences. 2000;172(1):30-7. doi: 10.1016/s0022-510x(99)00268-3. PubMed PMID:

WOS:000085188000006.

28. Sterr A, Schmalohr D, Kolbel S, Freivogel S. Transcranial magnetic stimulation-based investigation of motor cortex reorganization after repetitive training in brain-injured patients. Psychophysiology. 2000;37:S95-S. PubMed PMID: WOS:000089900500388.

29. Wrisley DM, Sparto PJ, Whitney SL, Furman JM. Cervicogenic dizziness: A review of diagnosis and treatment. Journal of Orthopaedic & Sports Physical Therapy. 2000;30(12):755-66. doi: 10.2519/jospt.2000.30.12.755. PubMed PMID: WOS:000166180200005.

30. Abo M, Chen ZG, Lai LJ, Reese T, Bjelke B. Functional recovery after brain lesion - contralateral neuromodulation: an fMRI study. Neuroreport. 2001;12(7):1543-7. doi: 10.1097/00001756-200105250-00048. PubMed PMID: WOS:000169077800044.

31. Blazar BR, Min DL, Chung B, Panoskaltsis-Mortari A, Farrell C, Lacey D, et al. Thymic epithelial cell (TEC) injury is inhibited by pre-BMT keratinocyte growth factor (KGF): A new approach to facilitate thymopoiesis in alloBMT recipients. Faseb Journal. 2001;15(4):A666-A. PubMed PMID: WOS:000167438103826.

32. Blum T, Hoheisel U, Unger T, Mense S. Fibroblast growth

factor-2 acutely influences the impulse activity of rat dorsal horn neurones. Neuroscience Research. 2001;40(2):115-23. doi: 10.1016/s0168-0102(01)00217-6. PubMed PMID: WOS:000169343600002.

33. Hamada N, Oshika T, Amano S, Tomidokoro A, Mihashi T, Maeda N. Corneal wavefront aberration and contrast sensitivity after photorefractive keratectomy. Investigative Ophthalmology & Visual Science. 2001;42(4):S896-S. PubMed PMID: WOS:000168392104777.

34. Nudo RJ, Plautz EJ, Frost SB. Role of adaptive plasticity in recovery of function after damage to motor cortex. Muscle & Nerve. 2001;24(8):1000-19. doi: 10.1002/mus.1104. PubMed PMID: WOS:000169947300003.

35. Savoy RL. History and future directions of human brain mapping and functional neuroimaging. Acta psychologica. 2001;107(1-3):9-42. doi: 10.1016/s0001-6918(01)00018-x. PubMed PMID: MEDLINE:11388144.

36. Thickbroom GW, Byrnes ML, Archer SA, Nagarajan L, Mastaglia FL. Differences in sensory and motor cortical organization following brain injury early in life. Annals of Neurology. 2001;49(3):320-7. doi: 10.1002/ana.68. PubMed PMID: WOS:000167250100008.

37. Chicurel M. Magnetic mind games. Nature. 2002;417(6885):114-6. doi: 10.1038/417114a. PubMed PMID: WOS:000175460200015.

38. Devulder J, Crombez E, Mortier E. Central pain: an overview. Acta Neurologica Belgica. 2002;102(3):97-103. PubMed PMID: WOS:000178782200001.

39. Drouot X, Nguyen JP, Peschanski M, Lefaucheur JP. The antalgic efficacy of chronic motor cortex stimulation is related to sensory changes in the painful zone. Brain. 2002;125:1660-4. doi: 10.1093/brain/awf161. PubMed PMID: WOS:000176783500021.

40. Heitger MH, Anderson TJ, Jones RD. Saccade sequences as markers for cerebral dysfunction following mild closed head injury. In: Hyona J, Munoz DP, Heide W, Radach R, editors. Brain's Eye: Neurobiological and Clinical Aspects of Oculomotor Research. Progress in Brain Research. 1402002. p. 433-48.

41. Hoppner J, Bollow K, Lehmann C, Buchmann J, Schlafke D, Ernst K. EEG and cardiovascular symptoms during convulsive syncope. Nervenheilkunde. 2002;21(1):49-52. PubMed PMID: WOS:000174263200009.

42. Luft AR, Kaelin-Lang A, Hauser TK, Buitrago MM, Thakor NV, Hanley DF, et al. Modulation of rodent cortical motor excitability by somatosensory input. Experimental Brain Research.

2002;142(4):562-9. doi: 10.1007/s00221-001-0952-1. PubMed PMID: WOS:000174362100011.

43. Montes C, Mertens P, Convers P, Peyron R, Sindou M, Laurent B, et al. Cognitive effects of precentral cortical stimulation for pain control: an ERP study. Neurophysiologie Clinique-Clinical Neurophysiology. 2002;32(5):313-25. doi: 10.1016/s0987-7053(02)00340-4. PubMed PMID: WOS:000180085400004.

44. Piroth MD, Zierhut D, van Kampen M, Sroka-Perez G, Wannenmacher M. Lung dose depending on exact patient positioning during total body irradiation (TBI) - Isoeffective considerations to assess the risk of interstitial pneumonitis after TBI. Strahlentherapie Und Onkologie. 2002;178(1):43-9. doi: 10.1007/s00066-002-0876-0. PubMed PMID: WOS:000174223700007.

45. Scheufler KM, Zentner J. Motor-evoked potential facilitation during progressive cortical suppression by propofol. Anesthesia and Analgesia. 2002;94(4):907-12. doi: 10.1097/00000539-200204000-00025. PubMed PMID: WOS:000174528600025.

46. Staudt M, Grodd W, Gerloff C, Erb M, Stitz J, Krageloh-Mann I. Two types of ipsilateral reorganization in congenital hemiparesis

- A TMS and fMRI study. Brain. 2002;125:2222-37. doi: 10.1093/brain/awf227. PubMed PMID: WOS:000178613700006.

47. Di Lazzaro V, Oliviero A, Mazzone P, Pilato F, Saturno E, Dileone M, et al. Generation of I waves in the human: spinal recordings. Supplements to Clinical neurophysiology. 2003;56:143-52. PubMed PMID: MEDLINE:14677388.

48. Kanno A, Nakasato N, Hatanaka K, Yoshimoto T. Ipsilateral area 3b responses to median nerve somatosensory stimulation. Neuroimage. 2003;18(1):169-77. doi: 10.1006/nimg.2002.1283. PubMed PMID: WOS:000180015200015.

49. Reisner AD. The electroconvulsive therapy controversy: Evidence and ethics. Neuropsychology Review. 2003;13(4):199-219. doi: 10.1023/b:Nerv.0000009484.76564.58. PubMed PMID: WOS:000188844300002.

50. Shimbo Y, Sakata M, Hayano M, Mori S. Topographical relationships between the Brainstem auditory and somatosensory evoked potentials and the location of lesions in posterior fossa stroke. Neurologia Medico-Chirurgica. 2003;43(6):282-91. doi: 10.2176/nmc.43.282. PubMed PMID: WOS:000183587400004.

51. Tomita Y, Fukuda C, Maegaki Y, Hanaki K, Kitagawa K, Sanpei M. Re-evaluation of short latency somatosensory evoked potentials (P13, P14 and N18) for brainstem function in children

who once suffered from deep coma. Brain & Development. 2003;25(5):352-6. doi: 10.1016/s0387-7604(03)00023-8. PubMed PMID: WOS:000184309900010.

52. Bernabeu M, Orient F, Tormos JM, Pascual-Leone A. Seizure induced by fast repetitive transcranial magnetic stimulation. Clinical Neurophysiology. 2004;115(7):1714-5. doi: 10.1016/j.clinph.2004.02.021. PubMed PMID: WOS:000222814800025.

53. Han SH, Jiang Y, Gu H, Rao HY, Mao LH, Cui Y, et al. The role of human parietal cortex in attention networks. Brain. 2004;127:650-9. doi: 10.1093/brain/awh071. PubMed PMID: WOS:000220108800016.

54. Heitger MH, Anderson TJ, Jones RD, Dalrymple-Alford JC, Frampton CM, Ardagh MW. Eye movement and visuomotor arm movement deficits following mild closed head injury. Brain. 2004;127:575-90. doi: 10.1093/brain/awh066. PubMed PMID: WOS:000220108800011.

55. Hiraoka T, Matsumoto Y, Okamoto F, Yamaguchi T, Hirohara Y, Mihashi T, et al. Corneal higher-order aberrations induced by overnight orthokerstology. Investigative Ophthalmology & Visual Science. 2004;45:U577-U. PubMed PMID: WOS:000223338001509.

56. Martelletti P, van Suijlekom H. Cervicogenic headache - Practical approaches to therapy. Cns Drugs. 2004;18(12):793-805. doi: 10.2165/00023210-200418120-00004. PubMed PMID: WOS:000224601900004.

57. Morecraft RJ, Stilwell-Morecraft KS, Rossing WR. The motor cortex and facial expression: New insights from neuroscience. Neurologist. 2004;10(5):235-49. doi: 10.1097/01.nrl.0000138734.45742.8d. PubMed PMID: WOS:000223868900002.

58. Paine P, Hamdy S, Hobson A, Mistry S, Gardener E, Aziz Q. Exploring the temporal characteristics of excitability in the human brain gut axis using transcranial magnetic stimulation. Gastroenterology. 2004;126(4):A589-A90. PubMed PMID: WOS:000220890202963.

59. Xu P, Wang Z, Wu Y. Primary Study on Median Nerve Stimulation Therapy in Improving the Level of Consciousness of Patients in Coma Caused by head Traumas. Suzhou University Journal of Medical Science. 2004;24(2):199-202. PubMed PMID: CSCD:1624092.

60. Fins JJ. Clinical pragmatism and the care of brain damaged patients: toward a palliative neuroethics for disorders of consciousness. In: Laureys S, editor. Boundaries of Consciousness:

Neurobiology and Neuropathology. Progress in Brain Research. 1502005. p. 565-82.
61. Hammoud DA, Endres CJ, Chander AR, Guilarte TR, Wong DF, Sacktor NC, et al. Imaging glial cell activation with C-11 -R-PK11195 in patients with AIDS. Journal of Neurovirology. 2005;11(4):346-55. doi: 10.1080/13550280500187351. PubMed PMID: WOS:000231877000004.
62. Jafri B, Li X, Yang H, Rabinowitz YS. Higher order wavefront aberrations in early keratoconus and keratoconus suspects. Investigative Ophthalmology & Visual Science. 2005;46. PubMed PMID: WOS:000227980405263.
63. Lee J, Oh J-K, 이현정, 김호성, 권기욱. Preventive effects of swimming training in cold water on cerebral infarct volume and motor functions in MCAo rats. Exercise Science. 2005;14(3):417-28. PubMed PMID: KJD:ART001137123.
64. Pascual-Leone A, Amedi A, Fregni F, Merabet LB. The plastic human brain cortex. Annual Review of Neuroscience. 2005;28:377-401. doi: 10.1146/annurev.neuro.27.070203.144216. PubMed PMID: WOS:000231235700015.
65. Ribeiro JA. What can Adenosine Neuromodulation do for Neuroprotection? Cns & Neurological Disorders-Drug Targets. 2005;4(4):325-9. doi: 10.2174/1568007054546090. PubMed PMID:

WOS:000497842800003.
66. Rusina R, Vaculin S, Yamamotova A, Barek S, Dvorakova H, Rokyta R. The effect of motor cortex stimulation in deafferentated rats. Neuroendocrinology Letters. 2005;26(4):283-8. PubMed PMID: WOS:000233233000002.
67. Senapati AK, Huntington PJ, Peng YB. Spinal dorsal horn neuron response to mechanical stimuli is decreased by electrical stimulation of the primary motor cortex. Brain Research. 2005;1036(1-2):173-9. doi: 10.1016/j.brainres.2004.12.043. PubMed PMID: WOS:000227577400021.
68. Takahashi CD, Der Yeghiaian L, Cramer SC. Stroke recovery and its imaging. Neuroimaging Clinics of North America. 2005;15(3):681-+. doi: 10.1016/j.nic.2005.08.006. PubMed PMID: WOS:000234303800015.
69. Carden P. Managed communication services architecture framework. Alcatel Telecommunications Review. 2006(1):67-72. PubMed PMID: WOS:000236508500016.
70. Ivins BJ, Schwab KA, Baker G, Warden DL. Hospital admissions associated with traumatic brain injury in the US army during peacetime: 1990s Trends. Neuroepidemiology. 2006;27(3):154-63. doi: 10.1159/000096127. PubMed PMID: WOS:000241773900006.

71. Karnath HO, Dieterich M. Spatial neglect - a vestibular disorder? Brain. 2006;129:293-305. doi: 10.1093/brain/awh698. PubMed PMID: WOS:000234965500004.

72. Lazar RM. Why does neuropsychology need a review journal? An editorial. Neuropsychology Review. 2006;16(1):1-2. doi: 10.1007/s11065-006-9004-8. PubMed PMID: WOS:000239015200001.

73. Lefaucheur JP. New insights into the therapeutic potential of non-invasive transcranial cortical stimulation in chronic neuropathic pain. Pain. 2006;122(1-2):11-3. doi: 10.1016/j.pain.2006.02.024. PubMed PMID: WOS:000237549100005.

74. Melander C, Adden R, Brinkmalm G, Gorton L, Mischnick P. New approaches to the analysis of enzymatically hydrolyzed methyl cellulose. Part 2. Comparison of various enzyme preparations. Biomacromolecules. 2006;7(5):1410-21. doi: 10.1021/bm0509422. PubMed PMID: WOS:000237593600006.

75. Nowak DA, Connemann BJ. Reply to "Seizure or convulsive syncope during 1-Hz rTMS?". Clinical Neurophysiology. 2006;117(11):2567-8. doi: 10.1016/j.clinph.2006.07.263. PubMed PMID: WOS:000242249800023.

76. Pascual-Leone A. Disrupting the brain to guide plasticity and

improve behavior. In: Moller AR, editor. Reprogramming the Brain. Progress in Brain Research. 1572006. p. 315-+.
77. Rafal RD. Oculomotor functions of the parietal lobe: Effects of chronic lesions in humans. Cortex. 2006;42(5):730-9. doi: 10.1016/s0010-9452(08)70411-8. PubMed PMID: WOS:000239547500010.
78. Sedmak B, Elersek T. Microcystins induce morphological and physiological changes in selected representative phytoplanktons. Microbial Ecology. 2006;51(4):508-15. doi: 10.1007/s00248-006-9045-9. PubMed PMID: WOS:000238520800011.
79. Terhoeven-Urselmans T, Michel K, Helfrich M, Flessa H, Ludwig B. Near-infrared spectroscopy can predict the composition of organic matter in soil and litter. Journal of Plant Nutrition and Soil Science. 2006;169(2):168-74. doi: 10.1002/jpln.200521712. PubMed PMID: WOS:000236991000003.
80. Zappasodi F, Pasqualetti P, Tombini M, Ercolani M, Pizzella V, Rossini PM, et al. Hand cortical representation at rest and during activation: Gender and age effects in the two hemispheres. Clinical Neurophysiology. 2006;117(7):1518-28. doi: 10.1016/j.clinph.2006.03.016. PubMed PMID: WOS:000239227100014.

81. Ansari S, Chaudhri K, Al Moutaery KA. Vagus nerve stimulation: indications and limitations. Acta neurochirurgica Supplement. 2007;97(Pt 2):281-6. PubMed PMID: MEDLINE:17691314.

82. Chiu F-C, Lai C-S, Lee R-S. Synthesis functional poly(carbonate-b-ester) copolymers and micellar characterizations. Journal of Applied Polymer Science. 2007;106(1):283-92. doi: 10.1002/app.26454. PubMed PMID: WOS:000248609900037.

83. Cioni B. Motor cortex stimulation for Parkinson's disease. Acta neurochirurgica Supplement. 2007;97(Pt 2):233-8. PubMed PMID: MEDLINE:17691309.

84. Desmurget M, Bonnetblanc F, Duffau H. Contrasting acute and slow-growing lesions: a new door to brain plasticity. Brain. 2007;130:898-914. doi: 10.1093/brain/awl300. PubMed PMID: WOS:000246293300004.

85. Favier I, van Vliet JA, Roon KI, Witteveen RJW, Verschuuren JJGM, Ferrari MD, et al. Trigeminal autonomic cephalgias due to structural lesions - A review of 31 cases. Archives of Neurology. 2007;64(1):25-31. doi: 10.1001/archneur.64.1.25. PubMed PMID: WOS:000243336500004.

86. Kishima H, Saitoh Y, Osaki Y, Nishimura H, Kato A, Hatazawa J, et al. Motor cortex stimulation in patients with

deafferentation pain: activation of the posterior insula and thalamus. Journal of Neurosurgery. 2007;107(1):43-8. doi: 10.3171/jns-07/07/0043. PubMed PMID: WOS:000247799900008.
87. Kunert HJ, Norra C, Hoff P. Theories of delusional disorders - An update and review. Psychopathology. 2007;40(3):191-202. doi: 10.1159/000100367. PubMed PMID: WOS:000245269800009.
88. Manola L, Holsheimer J. Motor cortex stimulation: role of computer modeling. Acta neurochirurgica Supplement. 2007;97(Pt 2):497-503. PubMed PMID: MEDLINE:17691340.
89. McDannold N, Vykhodtseva N, Hynynen K. Use of ultrasound pulses combined with definity for targeted blood-brain barrier disruption: A feasibility study. Ultrasound in Medicine and Biology. 2007;33(4):584-90. doi: 10.1016/j.ultrasmedbio.2006.10.004. PubMed PMID: WOS:000245645400012.
90. Morita I, Keith MW, Kanno T. Dorsal column stimulation for persistent vegetative state. Acta neurochirurgica Supplement. 2007;97(Pt 1):455-9. PubMed PMID: MEDLINE:17691409.
91. Nelissen N, Vandenbulcke M, Fannes K, Verbruggen A, Peeters R, Dupont P, et al. A beta amyloid deposition in the language system and how the brain responds. Brain. 2007;130:2055-69. doi: 10.1093/brain/awm133. PubMed PMID: WOS:000249097700011.

92. Panourias IG, Themistocleous M, Sakas DE. Intrathecal baclofen in current neuromodulatory practice: established indications and emerging applications. Acta neurochirurgica Supplement. 2007;97(Pt 1):145-54. PubMed PMID: MEDLINE:17691370.

93. Poggel DA, Mueller-Oehring EM, Gothe J, Kenkel S, Kasten E, Sabel BA. Visual hallucinations during spontaneous and training-induced visual field recovery. Neuropsychologia. 2007;45(11):2598-607. doi: 10.1016/j.neuropsychologia.2007.03.005. PubMed PMID: WOS:000247865800020.

94. Richard I, Menei P. Intrathecal baclofen in the treatment of spasticity, dystonia and vegetative disorders. Acta neurochirurgica Supplement. 2007;97(Pt 1):213-8. PubMed PMID: MEDLINE:17691379.

95. Scherder EJA, Vuijk P-J, Swaab DF, van Someren EJW. Estimating the effects of right median nerve stimulation on memory in Alzheimer's disease: A randomized controlled pilot study. Experimental Aging Research. 2007;33(2):177-86. doi: 10.1080/03610730701238915. PubMed PMID: WOS:000245435500005.

96. Schiff ND, Fins JJ. Deep brain stimulation and cognition:

moving from animal to patient. Current Opinion in Neurology. 2007;20(6):638-42. doi: 10.1097/WCO.0b013e3282f1c6e4. PubMed PMID: WOS:000251209300006.

97. Sgouros S. Surgical management of spasticity of cerebral origin in children. Acta neurochirurgica Supplement. 2007;97(Pt 1):193-203. PubMed PMID: MEDLINE:17691377.

98. Staudt M. (Re-)organization of the developing human brain following periventricular white matter lesions. Neuroscience and Biobehavioral Reviews. 2007;31(8):1150-6. doi: 10.1016/j.neubiorev.2007.05.005. PubMed PMID: WOS:000251873300009.

99. Wu AK, McCairn KW, Zada G, Wu T, Turner RS. Motor cortex stimulation: mild transient benefit in a primate model of Parkinson disease. Journal of Neurosurgery. 2007;106(4):695-700. doi: 10.3171/jns.2007.106.4.695. PubMed PMID: WOS:000245586900028.

100. Bushnik T, Englander J, Wright J. Patterns of fatigue and its correlates over the first 2 years after traumatic brain injury. The Journal of head trauma rehabilitation. 2008;23(1):25-32. doi: 10.1097/01.HTR.0000308718.88214.bb. PubMed PMID: MEDLINE:18219232.

101. Chen J-K, Johnston KM, Petrides M, Ptito A. Neural

substrates of symptoms of depression following concussion in male athletes with persisting postconcussion symptoms. Archives of General Psychiatry. 2008;65(1):81-9. doi: 10.1001/archgenpsychiatry.2007.8. PubMed PMID: WOS:000252176400011.

102. Dijkerman HC, Vargha-Khadem F, Polkey CE, Weiskrantz L. Ipsilesional and contralesional sensorimotor function after hemispherectomy: Differences between distal and proximal function. Neuropsychologia. 2008;46(3):886-901. doi: 10.1016/j.neuropsychologia.2007.11.023. PubMed PMID: WOS:000254731700013.

103. Frye RE, Rotenberg A, Ousley M, Pascual-Leone A. Transcranial Magnetic Stimulation in Child Neurology: Current and Future Directions. Journal of Child Neurology. 2008;23(1):79-96. doi: 10.1177/0883073807307972. PubMed PMID: WOS:000262554000013.

104. Isagulyan ED, Shabalov VA. Neuromodulation is controlled analgesia: Criteria for long efficiency. Problems of Neurosurgery named after NN Burdenko. 2008(2):18-28. PubMed PMID: RSCI:11655387.

105. Kao C-D, Chen J-T, Lai K-L, Chang J-B, Wu Z-A, Liao K-K. Gabapentin for decerebrate rigidity - A case report. Clinical Drug

Investigation. 2008;28(1):67-70. doi: 10.2165/00044011-200828010-00008. PubMed PMID: WOS:000252174300008.

106. Koenigs M, Huey ED, Calamia M, Raymont V, Tranel D, Grafman J. Distinct Regions of Prefrontal Cortex Mediate Resistance and Vulnerability to Depression. Journal of Neuroscience. 2008;28(47):12341-8. doi: 10.1523/jneurosci.2324-08.2008. PubMed PMID: WOS:000261191000020.

107. Koenigs M, Huey ED, Raymont V, Cheon B, Solomon J, Wassermann EM, et al. Focal brain damage protects against post-traumatic stress disorder in combat veterans. Nature Neuroscience. 2008;11(2):232-7. doi: 10.1038/nn2032. PubMed PMID: WOS:000252712100018.

108. Masino SA, Geiger JD. Are purines mediators of the anticonvulsant/neuroprotective effects of ketogenic diets? Trends in Neurosciences. 2008;31(6):273-8. doi: 10.1016/j.tins.2008.02.009. PubMed PMID: WOS:000257002600002.

109. McDannold N, Vykhodtseva N, Hynynen K. Effects of acoustic parameters and ultrasound contrast agent dose on focused-ultrasound induced blood-brain barrier disruption. Ultrasound in Medicine and Biology. 2008;34(6):930-7. doi:

10.1016/j.ultrasmedbio.2007.11.009. PubMed PMID: WOS:000256485200009.

110. Miniussi C, Cappa SF, Cohen LG, Floel A, Fregni F, Nitsche MA, et al. Efficacy of repetitive transcranial magnetic stimulation/transcranial direct current stimulation in cognitive neurorehabititation. Brain Stimulation. 2008;1(4):326-36. doi: 10.1016/j.brs.2008.07.002. PubMed PMID: WOS:000262717000002.

111. Park J-H, von Maltzahn G, Ruoslahti E, Bhatia SN, Sailor MJ. Micellar hybrid nanoparticles for simultaneous magnetofluorescent imaging and drug delivery. Angewandte Chemie-International Edition. 2008;47(38):7284-8. doi: 10.1002/anie.200801810. PubMed PMID: WOS:000259382900026.

112. Solak O, Kavuncu V. Current Approaches in Therapeutic Management of Chronic Neuropathic Pain. Romatizma-Rheumatism. 2008;23(4):135-42. PubMed PMID: WOS:000262426100006.

113. Weder ND, Muralee S, Penland H, Tampi RR. Catatonia: A Review. Annals of Clinical Psychiatry. 2008;20(2):97-107. doi: 10.1080/10401230802017092. PubMed PMID: WOS:000261639600006.

114. Badawy RAB, Jackson GD, Berkovic SF, Newton MR,

Macdonell RAL. Diagnosis of epilepsy after the first seizure: a role for transcranial magnetic stimulation? Journal of the Neurological Sciences. 2009;285:S248-S. doi: 10.1016/s0022-510x(09)70944-x. PubMed PMID: WOS:000272521300874.

115. Bledsoe JM, Kimble CJ, Covey DP, Blaha CD, Agnesi F, Mohseni P, et al. Development of the Wireless Instantaneous Neurotransmitter Concentration System for intraoperative neurochemical monitoring using fast-scan cyclic voltammetry Technical note. Journal of Neurosurgery. 2009;111(4):712-23. doi: 10.3171/2009.3.Jns081348. PubMed PMID: WOS:000270550000013.

116. Chang WH, Kim Y-B, Ohn SH, Park C-h, Kim ST, Kim Y-H. Double decussated ipsilateral corticospinal tract in schizencephaly. Neuroreport. 2009;20(16):1434-8. doi: 10.1097/WNR.0b013e328331483d. PubMed PMID: WOS:000271292400008.

117. Dobkin BH. Motor rehabilitation after stroke, traumatic brain, and spinal cord injury: common denominators within recent clinical trials. Current Opinion in Neurology. 2009;22(6):563-9. doi: 10.1097/WCO.0b013e3283314b11. PubMed PMID: WOS:000272544100001.

118. Fontaine D, Bruneto JL, El Fakir H, Paquis P, Lanteri-Minet

M. Short-term restoration of facial sensory loss by motor cortex stimulation in peripheral post-traumatic neuropathic pain. Journal of Headache and Pain. 2009;10(3):203-6. doi: 10.1007/s10194-009-0115-5. PubMed PMID: WOS:000265924700009.

119. Heitger MH, Jones RD, Macleod AD, Snell DL, Frampton CM, Anderson TJ. Impaired eye movements in post-concussion syndrome indicate suboptimal brain function beyond the influence of depression, malingering or intellectual ability. Brain. 2009;132:2850-70. doi: 10.1093/brain/awp181. PubMed PMID: WOS:000270685600031.

120. Jang SH. Review of motor recovery in patients with traumatic brain injury. NeuroRehabilitation. 2009;24(4):349-53. doi: 10.3233/nre-2009-0489. PubMed PMID: MEDLINE:19597273.

121. Jang SH, Kim D-S, Son SM, Cho YW, Kim SH, Kim OL, et al. Clinical application of diffusion tensor tractography for elucidation of the causes of motor weakness in patients with traumatic brain injury. NeuroRehabilitation. 2009;24(3):273-8. doi: 10.3233/nre-2009-0478. PubMed PMID: MEDLINE:19458435.

122. Koenigs M, Barbey AK, Postle BR, Grafman J. Superior Parietal Cortex Is Critical for the Manipulation of Information in Working Memory. Journal of Neuroscience. 2009;29(47):14980-6.

doi: 10.1523/jneurosci.3706-09.2009. PubMed PMID: WOS:000272185100030.

123. Liu J-T, Lee J-K, Tyan Y-S, Liu C-Y, Chen Y-H, Lin T-B. Neuromodulation on cervical spinal cord combined with hyperbaric oxygen in comatose patients-a preliminary report. Surgical Neurology. 2009;72:28-35. doi: 10.1016/j.surneu.2009.05.029. PubMed PMID: WOS:000279234100004.

124. Mori F, Koch G, Foti C, Bernardi G, Centonze D. The use of repetitive transcranial magnetic stimulation (rTMS) for the treatment of spasticity. In: Verhaagen J, Hol EM, Huitenga I, Wijnholds J, Bergen AB, Boer GJ, et al., editors. Neurotherapy: Progress in Restorative Neuroscience and Neurology. Progress in Brain Research. 1752009. p. 429-39.

125. Proto D, Pella RD, Hill BD, Gouvier WD. Assessment and rehabilitation of acquired visuospatial and proprioceptive deficits associated with visuospatial neglect. NeuroRehabilitation. 2009;24(2):145-57. doi: 10.3233/nre-2009-0463. PubMed PMID: MEDLINE:19339753.

126. Reis J, Schambra HM, Cohen LG, Buch ER, Fritsch B, Zarahn E, et al. Noninvasive cortical stimulation enhances motor skill acquisition over multiple days through an effect on consolidation.

Proceedings of the National Academy of Sciences of the United States of America. 2009;106(5):1590-5. doi: 10.1073/pnas.0805413106. PubMed PMID: WOS:000263074600054.

127. Rotenberg A, Pascual-Leone A. Safety of 1 Hz repetitive transcranial magnetic stimulation (rTMS) in patients with titanium skull plates. Clinical Neurophysiology. 2009;120(7):1417-. doi: 10.1016/j.clinph.2009.05.004. PubMed PMID: WOS:000268841700026.

128. Singh S, Mistry S, Jefferson S, Davies K, Rothwell J, Williams S, et al. A Magnetic Resonance Spectroscopy Study of Brain Glutamate in a Model of Plasticity in Human Pharyngeal Motor Cortex. Gastroenterology. 2009;136(2):417-24. doi: 10.1053/j.gastro.2008.10.087. PubMed PMID: WOS:000262967700013.

129. Tsuchida A, Fellows LK. Lesion Evidence That Two Distinct Regions within Prefrontal Cortex are Critical for n-Back Performance in Humans. Journal of Cognitive Neuroscience. 2009;21(12):2263-75. doi: 10.1162/jocn.2008.21172. PubMed PMID: WOS:000272351200001.

130. Xu W, Gao Q, Xu Y, Wu D, Sun Y, Shen W, et al. Controllable release of ibuprofen from size-adjustable and surface hydrophobic

mesoporous silica spheres. Powder Technology. 2009;191(1-2):13-20. doi: 10.1016/j.powtec.2008.09.001. PubMed PMID: WOS:000264656900003.

131. Yamashita O, Odahara H, Ochi T, Shigeto J-i. Dependence of the resultant Seebeck coefficient on the thickness of Bi0.88Sb0.12 alloy in welded M/Bi-Sb/M (M = Cu and Ni) composites. Applied Physics a-Materials Science & Processing. 2009;97(1):133-41. doi: 10.1007/s00339-009-5300-5. PubMed PMID: WOS:000269206600019.

132. Zafonte R, Hammond F, Dennison A, Chew E. Pharmacotherapy to enhance arousal: what is known and what is not. In: Laureys S, Schiff ND, Owen AM, editors. Coma Science: Clinical and Ethical Implications. Progress in Brain Research. 1772009. p. 293-316.

133. Carmel JB, Berrol LJ, Brus-Ramer M, Martin JH. Chronic Electrical Stimulation of the Intact Corticospinal System after Unilateral Injury Restores Skilled Locomotor Control and Promotes Spinal Axon Outgrowth. Journal of Neuroscience. 2010;30(32):10918-26. doi: 10.1523/jneurosci.1435-10.2010. PubMed PMID: WOS:000280795900031.

134. Casali AG, Casarotto S, Rosanova M, Mariotti M, Massimini M. General indices to characterize the electrical response of the

cerebral cortex to TMS. Neuroimage. 2010;49(2):1459-68. doi: 10.1016/j.neuroimage.2009.09.026. PubMed PMID: WOS:000272808400030.

135. D'Agati D, Bloch Y, Levkovitz Y, Reti I. rTMS for adolescents: Safety and efficacy considerations. Psychiatry Research. 2010;177(3):280-5. doi: 10.1016/j.psychres.2010.03.004. PubMed PMID: WOS:000278659600002.

136. Fleming MK, Stinear CM, Byblow WD. Bilateral parietal cortex function during motor imagery. Experimental Brain Research. 2010;201(3):499-508. doi: 10.1007/s00221-009-2062-4. PubMed PMID: WOS:000275414500013.

137. George MS, Aston-Jones G. Noninvasive techniques for probing neurocircuitry and treating illness: vagus nerve stimulation (VNS), transcranial magnetic stimulation (TMS) and transcranial direct current stimulation (tDCS). Neuropsychopharmacology. 2010;35(1):301-16. doi: 10.1038/npp.2009.87. PubMed PMID: WOS:000272784600016.

138. Gooch CM, Wiener M, Wencil EB, Coslett HB. Interval timing disruptions in subjects with cerebellar lesions. Neuropsychologia. 2010;48(4):1022-31. doi: 10.1016/j.neuropsychologia.2009.11.028. PubMed PMID: WOS:000275933500020.

139. Harvey RL. Central Poststroke Pain Syndrome. Topics in Stroke Rehabilitation. 2010;17(3):163-72. doi: 10.1310/tsr1703-163. PubMed PMID: WOS:000280229200003.

140. Jang SH. Unusual long-term motor recovery in a patient with corona radiata infarct. Neural Regeneration Research. 2010;5(17):1353-6. doi: 10.3969/j.issn.1673-5374.2010.17.013. PubMed PMID: WOS:000283641200013.

141. Knikou M. Neural control of locomotion and training-induced plasticity after spinal and cerebral lesions. Clinical Neurophysiology. 2010;121(10):1655-68. doi: 10.1016/j.clinph.2010.01.039. PubMed PMID: WOS:000281435800007.

142. Lancioni GE, Bosco A, Belardinelli MO, Singh NN, O'Reilly MF, Sigafoos J. An overview of intervention options for promoting adaptive behavior of persons with acquired brain injury and minimally conscious state. Research in developmental disabilities. 2010;31(6):1121-34. doi: 10.1016/j.ridd.2010.06.019. PubMed PMID: MEDLINE:20663643.

143. Lee R-S, Li H-R, Chiu F-C. Synthesis of Amphiphilic Pseudopolyamino Acid-Containing ABC-Triblock Copolymers and Micellar Characterizations. Journal of Applied Polymer Science. 2010;115(5):2556-64. doi: 10.1002/app.31213. PubMed PMID:

WOS:000274114000004.
144. Lou HC, Gross J, Biermann-Ruben K, Kjaer TW, Schnitzler A. Coherence in Consciousness: Paralimbic Gamma Synchrony of Self-Reference Links Conscious Experiences. Human Brain Mapping. 2010;31(2):185-92. doi: 10.1002/hbm.20855. PubMed PMID: WOS:000274460900002.
145. Maller JJ, Thomson RHS, Lewis PM, Rose SE, Pannek K, Fitzgerald PB. Traumatic brain injury, major depression, and diffusion tensor imaging: Making connections. Brain Research Reviews. 2010;64(1):213-40. doi: 10.1016/j.brainresrev.2010.04.003. PubMed PMID: WOS:000279291800009.
146. Plowman EK, Kleim JA. Motor cortex reorganization across the lifespan. Journal of communication disorders. 2010;43(4):286-94. doi: 10.1016/j.jcomdis.2010.04.005. PubMed PMID: MEDLINE:20478572.
147. Polito MZ, Thompson JWG, DeFina PA. A review of the International Brain Research Foundation novel approach to mild traumatic brain injury presented at the International Conference on Behavioral Health and Traumatic Brain Injury. Journal of the American Academy of Nurse Practitioners. 2010;22(9):504-9. doi: 10.1111/j.1745-7599.2010.00540.x. PubMed PMID:

WOS:000281660700009.
148. Pope C, Mechoulam R, Parsons L. Endocannabinoid signaling in neurotoxicity and neuroprotection. Neurotoxicology. 2010;31(5):562-71. doi: 10.1016/j.neuro.2009.12.002. PubMed PMID: WOS:000281939200020.
149. Reidler JS, Nascimento BC, Wu DSK, Carvas M, Massuyama BK, Oliveira BMRd, et al. Modulation of inhibitory systems to enhance motor rehabilitation: insights for the use of noninvasive brain stimulation. Psychology & Neuroscience. 2010;3(2):151-60. doi: 10.3922/j.psns.2010.2.004. PubMed PMID: SCIELO:S1983-32882010000200004.
150. Alonso-Alonso M, Chang B, Press DZ, Rotenberg A, Pascual-Leone A. Commentary on Kratz et al "Seizure in a Nonpredisposed Individual Induced by Single-Pulse Transcranial Magnetic Stimulation". Journal of Ect. 2011;27(2):176-7. doi: 10.1097/YCT.0b013e3181e6336e. PubMed PMID: WOS:000290789300021.
151. Babiloni C, Vecchio F, Lizio R, Ferri R, Rodriguez G, Marzano N, et al. Resting State Cortical Rhythms in Mild Cognitive Impairment and Alzheimer's Disease: Electroencephalographic Evidence. Journal of Alzheimers Disease. 2011;26:201-14. doi: 10.3233/jad-2011-0051. PubMed PMID:

WOS:000297842800014.

152. Cook G, Garderet L, van Biezen A, Henseler A, Leblond V, Rovira M, et al. The Roel of High Dose Chemotherapy and Autologous Stem Cell Transplantation (ASCT) In PATIENTS with POEMS SYNDROME: A Retrospective study of the MM Subcommittee of the Chronic Leukemia Working Party of the EBMT. Blood. 2011;118(21):1758-9. PubMed PMID: WOS:000299597106283.

153. De Ridder D, van der Loo E, Vanneste S, Gais S, Plazier M, Kovacs S, et al. Theta-gamma dysrhythmia and auditory phantom perception Case report. Journal of Neurosurgery. 2011;114(4):912-21. doi: 10.3171/2010.11.Jns10335. PubMed PMID: WOS:000288725900005.

154. Dudakkov JA, Hanash AM, Young LF, Singer NV, West ML, Jenq RR, et al. Innate Lymphoid Cell-Derived IL-22 Mediates Endogenous Thymic Repair Under the Control of IL-23. Blood. 2011;118(21):69-. PubMed PMID: WOS:000299597100144.

155. Fonoff ET, Hamani C, de Andrade DC, Yeng LT, Marcolin MA, Teixeira MJ. Pain Relief and Functional Recovery in Patients with Complex Regional Pain Syndrome after Motor Cortex Stimulation. Stereotactic and Functional Neurosurgery. 2011;89(3):167-72. doi: 10.1159/000324895. PubMed PMID:

WOS:000290307000006.
156. Gao G, Cooper E, Jiang J. RIGHT MEDIAN NERVE STIMULATION IN POST-TRAUMATIC COMA. Journal of Neurotrauma. 2011;28(5):A39-A. PubMed PMID: WOS:000290472100116.
157. Gillebert CR, Mantini D, Thijs V, Sunaert S, Dupont P, Vandenberghe R. Lesion evidence for the critical role of the intraparietal sulcus in spatial attention. Brain. 2011;134:1694-709. doi: 10.1093/brain/awr085. PubMed PMID: WOS:000291063900012.
158. Grechanina OY, Zdibska OP, Novikova IV. FATTY ACID OXIDATION DEFECT, DETECTED USING SELECTIVE SCREENING. Journal of Inherited Metabolic Disease. 2011;34:S157-S. PubMed PMID: WOS:000309837800281.
159. Hansen H-C. Sooner or later ...? In search of the most useful components of the somatosensory evoked potential to define prognosis in critically ill patients with acute stroke. Clinical Neurophysiology. 2011;122(8):1482-4. doi: 10.1016/j.clinph.2011.01.038. PubMed PMID: WOS:000292633300002.
160. Hosp JA, Hertler B, Atiemo CO, Luft AR. Dopaminergic modulation of receptive fields in rat sensorimotor cortex.

Neuroimage. 2011;54(1):154-60. doi: 10.1016/j.neuroimage.2010.07.029. PubMed PMID: WOS:000283825000019.

161. Jagodic HK, Jankovic VN, Pregelj P. Up-todate treatment with electroconvulsive therapy. Zdravniski Vestnik-Slovenian Medical Journal. 2011;80(3):203-14. PubMed PMID: WOS:000288646600011.

162. Jang SH. Diffusion tensor imaging studies on corticospinal tract injury following traumatic brain injury: a review. NeuroRehabilitation. 2011;29(4):339-45. doi: 10.3233/nre-2011-0710. PubMed PMID: MEDLINE:22207060.

163. Jean-Paul N, Nizard J, Keravel Y, Lefaucheur J-P. Invasive brain stimulation for the treatment of neuropathic pain. Nature Reviews Neurology. 2011;7(12):699-709. doi: 10.1038/nrneurol.2011.138. PubMed PMID: WOS:000298149000008.

164. Kleim JA. Neural plasticity and neurorehabilitation: teaching the new brain old tricks. Journal of communication disorders. 2011;44(5):521-8. doi: 10.1016/j.jcomdis.2011.04.006. PubMed PMID: MEDLINE:21600589.

165. Kratz O. Reply to the Letter to the Editor "Response to Kratz et al, Seizure in a Nonpredisposed Individual Induced by

Single-Pulse Transcranial Magnetic Stimulation". Journal of Ect. 2011;27(2):177-. doi: 10.1097/YCT.0b013e3181ec0d8a. PubMed PMID: WOS:000290789300022.

166. Lambooij E, Anil H, Butler SR, Reimert H, Workel L, Hindle V. Transcranial magnetic stunning of broilers: a preliminary trial to induce unconsciousness. Animal Welfare. 2011;20(3):407-12. PubMed PMID: WOS:000293305900010.

167. Livingston SC. Head Injury - Baseball. Persistent Electrophysiologic Changes Associated with Acute Cerebral Concussion in a Collegiate Baseball Player. Medicine and Science in Sports and Exercise. 2011;43(5):196-. doi: 10.1249/01.MSS.0000400522.18851.f6. PubMed PMID: WOS:000209079500494.

168. Lucas JM, Ji Y, Masri R. Motor cortex stimulation reduces hyperalgesia in an animal model of central pain. Pain. 2011;152(6):1398-407. doi: 10.1016/j.pain.2011.02.025. PubMed PMID: WOS:000290710100028.

169. Ma Y, Hu Y, Valentin N, Geocadin RG, Thakor NV, Jia X. Time jitter of somatosensory evoked potentials in recovery from hypoxic-ischemic brain injury. Journal of Neuroscience Methods. 2011;201(2):355-60. doi: 10.1016/j.jneumeth.2011.08.025. PubMed PMID: WOS:000295998900011.

170. Magnani B, Oliveri M, Mancuso G, Galante E, Frassinetti F. Time and spatial attention: Effects of prism adaptation on temporal deficits in brain damaged patients. Neuropsychologia. 2011;49(5):1016-23. doi: 10.1016/j.neuropsychologia.2010.12.014. PubMed PMID: WOS:000290649200027.

171. Migalska K, Morrow DIJ, Garland MJ, Thakur R, Woolfson AD, Donnelly RF. Laser-Engineered Dissolving Microneedle Arrays for Transdermal Macromolecular Drug Delivery. Pharmaceutical Research. 2011;28(8):1919-30. doi: 10.1007/s11095-011-0419-4. PubMed PMID: WOS:000292518800011.

172. Miniussi C, Vallar G. Brain stimulation and behavioural cognitive rehabilitation: A new tool for neurorehabilitation? Foreword. Neuropsychological Rehabilitation. 2011;21(5):553-9. doi: 10.1080/09602011.2011.622435. PubMed PMID: WOS:000299789100001.

173. Missitzi J, Gentner R, Geladas N, Politis P, Karandreas N, Classen J, et al. Plasticity in human motor cortex is in part genetically determined. Journal of Physiology-London. 2011;589(2):297-306. doi: 10.1113/jphysiol.2010.200600. PubMed PMID: WOS:000286213900004.

174. Morishita T, Yamashita A, Katayama Y, Oshima H, Nishizaki

Y, Shijo K, et al. Chronological Changes in Astrocytes Induced by Chronic Electrical Sensorimotor Cortex Stimulation in Rats. Neurologia Medico-Chirurgica. 2011;51(7):496-502. doi: 10.2176/nmc.51.496. PubMed PMID: WOS:000292853000004.

175. Musayev AV, Huseynova SG, Mustafayeva EE. MAGNETIC STIMULATION IN REHABILITATION OF PATIENTS SUFFERING FROM TRAUMATIC NEUROPATHY. European Journal of Neurology. 2011;18:325-. PubMed PMID: WOS:000294806600590.

176. Nagarwal RC, Kumar R, Dhanawat M, Pandit JK. Modified PLA nano in situ gel: A potential ophthalmic drug delivery system. Colloids and Surfaces B-Biointerfaces. 2011;86(1):28-34. doi: 10.1016/j.colsurfb.2011.03.023. PubMed PMID: WOS:000291458100005.

177. Pape TL-B. Diagnostic and therapeutic challenges of persons with severe traumatic brain injuries. Faseb Journal. 2011;25. PubMed PMID: WOS:000310708402343.

178. Paulus W. Transcranial stimulation techniques: which genetics is the best for which purpose? Journal of Physiology-London. 2011;589(6):1245-. doi: 10.1113/jphysiol.2011.206680. PubMed PMID: WOS:000288376800003.

179. Pitcher JB, Schneider LA, Drysdale JL, Ridding MC, Owens

JA. Motor System Development of the Preterm and Low Birthweight Infant. Clinics in Perinatology. 2011;38(4):605-+. doi: 10.1016/j.clp.2011.08.010. PubMed PMID: WOS:000298767800004.

180. Reis J, Fritsch B. Modulation of motor performance and motor learning by transcranial direct current stimulation. Current Opinion in Neurology. 2011;24(6):590-6. doi: 10.1097/WCO.0b013e32834c3db0. PubMed PMID: WOS:000296850900012.

181. Rossetti AO, Lowenstein DH. Management of refractory status epilepticus in adults: still more questions than answers. Lancet Neurology. 2011;10(10):922-30. doi: 10.1016/s1474-4422(11)70187-9. PubMed PMID: WOS:000295814600013.

182. Rossi S, Hallett M, Rossini PM, Pascual-Leone A. Screening questionnaire before TMS: An update. Clinical Neurophysiology. 2011;122(8):1686-. doi: 10.1016/j.clinph.2010.12.037. PubMed PMID: WOS:000292633300030.

183. Shorvon S. Super-refractory status epilepticus: An approach to therapy in this difficult clinical situation. Epilepsia. 2011;52:53-6. doi: 10.1111/j.1528-1167.2011.03238.x. PubMed PMID: WOS:000296065200018.

184. Timofeev I. Neuronal plasticity and thalamocortical sleep and waking oscillations. In: VanSomeren EJW, VanDerWerf YD, Roelfsema PR, Mansvelder HD, DaSilva FHL, editors. Slow Brain Oscillations of Sleep, Resting State and Vigilance. Progress in Brain Research. 1932011. p. 121-44.

185. Tomassini V, Jbabdi S, Kincses ZT, Bosnell R, Douaud G, Pozzilli C, et al. Structural and Functional Bases for Individual Differences in Motor Learning. Human Brain Mapping. 2011;32(3):494-508. doi: 10.1002/hbm.21037. PubMed PMID: WOS:000287375500013.

186. Xia LY, Zhang MQ, Yuan Ce, Rong MZ. A facile heteroaggregate-template route to hollow magnetic mesoporous spheres with tunable shell structures. Journal of Materials Chemistry. 2011;21(25):9020-6. doi: 10.1039/c1jm10126a. PubMed PMID: WOS:000291611600015.

187. You X, Chang J-h, Ju B-K, Pak JJ. Rapidly dissolving fibroin microneedles for transdermal drug delivery. Materials Science & Engineering C-Materials for Biological Applications. 2011;31(8):1632-6. doi: 10.1016/j.msec.2011.06.010. PubMed PMID: WOS:000297400800003.

188. Arnsten AFT, Wang MJ, Paspalas CD. Neuromodulation of Thought: Flexibilities and Vulnerabilities in Prefrontal Cortical

Network Synapses. Neuron. 2012;76(1):223-39. doi: 10.1016/j.neuron.2012.08.038. PubMed PMID: WOS:000309799000016.

189. Bates KA, Clark VW, Meloni BP, Dunlop SA, Rodger J. Short-term low intensity PMF does not improve functional or histological outcomes in a rat model of transient focal cerebral ischemia. Brain Research. 2012;1458:76-85. doi: 10.1016/j.brainres.2012.04.006. PubMed PMID: WOS:000305365900008.

190. Bikson M, Rahman A, Datta A, Fregni F, Merabet L. High-Resolution Modeling Assisted Design of Customized and Individualized Transcranial Direct Current Stimulation Protocols. Neuromodulation. 2012;15(4):306-15. doi: 10.1111/j.1525-1403.2012.00481.x. PubMed PMID: WOS:000307219900006.

191. Bogdanova Y, Verfaellie M. Cognitive Sequelae of Blast-Induced Traumatic Brain Injury: Recovery and Rehabilitation. Neuropsychology Review. 2012;22(1):4-20. doi: 10.1007/s11065-012-9192-3. PubMed PMID: WOS:000300892100003.

192. Borckardt JJ, Bikson M, Frohman H, Reeves ST, Datta A, Bansal V, et al. A Pilot Study of the Tolerability and Effects of

High-Definition Transcranial Direct Current Stimulation (HD-tDCS) on Pain Perception. Journal of Pain. 2012;13(2):112-20. doi: 10.1016/j.jpain.2011.07.001. PubMed PMID: WOS:000300594200002.

193. Burton SD, Ermentrout GB, Urban NN. Intrinsic heterogeneity in oscillatory dynamics limits correlation-induced neural synchronization. Journal of Neurophysiology. 2012;108(8):2115-33. doi: 10.1152/jn.00362.2012. PubMed PMID: WOS:000310057500004.

194. Chung H, Dai T, Sharma SK, Huang Y-Y, Carroll JD, Hamblin MR. The Nuts and Bolts of Low-level Laser (Light) Therapy. Annals of Biomedical Engineering. 2012;40(2):516-33. doi: 10.1007/s10439-011-0454-7. PubMed PMID: WOS:000300485100019.

195. Costa V. USE OF NONINVASIVE CEREBRAL STIMULATION TECHNIQUES IN APHASIA: AN UPDATING. Acta Medica Mediterranea. 2012;28(2):105-8. PubMed PMID: WOS:000310121900008.

196. Danilov Y, Tyler M, Kazcmarek K. NEW APPROACH TO CHRONIC TBI REHABILITATION: CRANIAL NERVE NONINVASIVE NEUROMODULATION (CN-NINM TECHNOLOGY). Journal of Neurotrauma. 2012;29(10):A97-A.

PubMed PMID: WOS:000306244400163.

197. de Haan B, Karnath H-O, Driver J. Mechanisms and anatomy of unilateral extinction after brain injury. Neuropsychologia. 2012;50(6):1045-53. doi: 10.1016/j.neuropsychologia.2012.02.015. PubMed PMID: WOS:000304724500005.

198. Dutta D, Sharma S, Das J, Gairola RM. Development of a regional rain retrieval algorithm for exclusive mesoscale convective systems over peninsular India. Advances in Space Research. 2012;49(12):1750-62. doi: 10.1016/j.asr.2012.02.028. PubMed PMID: WOS:000304787900012.

199. Gao G, Jiang J, Cooper E. Short term right median nerve stimulation arouse profound variation of brain blood perfusion and microRNA expression on traumatic coma patients. Brain Injury. 2012;26(4-5):401-2. PubMed PMID: WOS:000304104600175.

200. Giacino J, Fins JJ, Machado A, Schiff ND. Central Thalamic Deep Brain Stimulation to Promote Recovery from Chronic Posttraumatic Minimally Conscious State: Challenges and Opportunities. Neuromodulation. 2012;15(4):339-49. doi: 10.1111/j.1525-1403.2012.00458.x. PubMed PMID: WOS:000307219900009.

201. Gordon AL, di Maggio A. Rehabilitation for Children After Acquired Brain Injury: Current and Emerging Approaches.

Pediatric Neurology. 2012;46(6):339-44. doi: 10.1016/j.pediatrneurol.2012.02.029. PubMed PMID: WOS:000305301800001.

202. Hughes JR. THE SAVANT SYNDROME AND ITS POSSIBLE RELATIONSHIP TO EPILEPSY. In: Ahmad SI, editor. Neurodegenerative Diseases. Advances in Experimental Medicine and Biology. 7242012. p. 332-43.

203. Kahali P, Tadayon SH, Zali A, Abbasian A. Transcranial direct current stimulation in a vegetative state patient: a case study. European Journal of Neurology. 2012;19:579-. PubMed PMID: WOS:000309359704006.

204. Kemmerer D, Rudrauf D, Manzel K, Tranel D. Behavioral patterns and lesion sites associated with impaired processing of lexical and conceptual knowledge of actions. Cortex. 2012;48(7):826-48. doi: 10.1016/j.cortex.2010.11.001. PubMed PMID: WOS:000305710600004.

205. Khandan O, Famili A, Kahook MY, Rao MP. Titanium-based, fenestrated, in-plane microneedles for passive ocular drug delivery. Annual International Conference of the IEEE Engineering in Medicine and Biology Society IEEE Engineering in Medicine and Biology Society Annual International Conference. 2012;2012:6572-5. doi: 10.1109/embc.2012.6347500. PubMed

PMID: MEDLINE:23367435.

206. Kumaria A, Tolias CM. Is there a role for vagus nerve stimulation therapy as a treatment of traumatic brain injury? British Journal of Neurosurgery. 2012;26(3):316-20. doi: 10.3109/02688697.2012.663517. PubMed PMID: WOS:000304524300005.

207. Lancioni GE, Singh NN, O'Reilly MF, Sigafoos J, Amenduni MT, Navarro J, et al. Microswitch technology and contingent stimulation to promote adaptive engagement in persons with minimally conscious state: a case evaluation. Cognitive processing. 2012;13(2):133-7. doi: 10.1007/s10339-011-0428-6. PubMed PMID: MEDLINE:22131129.

208. Lancioni GE, Singh NN, O'Reilly MF, Sigafoos J, Belardinelli MO, Buonocunto F, et al. Promoting adaptive behavior in persons with acquired brain injury, extensive motor and communication disabilities, and consciousness disorders. Research in developmental disabilities. 2012;33(6):1964-74. doi: 10.1016/j.ridd.2012.05.027. PubMed PMID: MEDLINE:22738766.

209. Lu Y-J, Wei K-C, Ma C-CM, Yang S-Y, Chen J-P. Dual targeted delivery of doxorubicin to cancer cells using folate-conjugated magnetic multi-walled carbon nanotubes.

Colloids and Surfaces B-Biointerfaces. 2012;89:1-9. doi: 10.1016/j.colsurfb.2011.08.001. PubMed PMID: WOS:000297495000001.

210. Matsumoto H, Ugawa Y. Neurophysiological analyses of asterixis utilizing innovative approaches. Clinical Neurophysiology. 2012;123(9):1695-6. doi: 10.1016/j.clinph.2012.02.063. PubMed PMID: WOS:000307183800006.

211. Nuti C, Vassal F, Mertens P, Lemaire J-J, Magnin M, Peyron R. Improved Dexterity after Chronic Electrical Stimulation of the Motor Cortex for Central Pain: A Special Relevance for Thalamic Syndrome. Stereotactic and Functional Neurosurgery. 2012;90(6):370-8. doi: 10.1159/000338681. PubMed PMID: WOS:000310357500005.

212. Priori A, Mameli F, Ferrucci R, Barbieri S. Transcranial Direct Current Stimulation (tDCS) and Cognitive Decline. Journal of Alzheimers Disease. 2012;29:31-2. PubMed PMID: WOS:000303176400010.

213. Riccio A, Mattia D, Simione L, Olivetti M, Cincotti F. Eye-gaze independent EEG-based brain-computer interfaces for communication. Journal of Neural Engineering. 2012;9(4). doi: 10.1088/1741-2560/9/4/045001. PubMed PMID: WOS:000306759600002.

214. Singh TRR, Garland MJ, Migalska K, Salvador EC, Shaikh R, McCarthy HO, et al. Influence of a pore-forming agent on swelling, network parameters, and permeability of poly(ethylene glycol)-crosslinked poly(methyl vinyl ether-co-maleic acid) hydrogels: Application in transdermal delivery systems. Journal of Applied Polymer Science. 2012;125(4):2680-94. doi: 10.1002/app.36524. PubMed PMID: WOS:000303596200023.
215. Sterr A, Conforto AB. Plasticity of Adult Sensorimotor System in Severe Brain Infarcts: Challenges and Opportunities. Neural Plasticity. 2012;2012. doi: 10.1155/2012/970136. PubMed PMID: WOS:000302658500001.
216. Takeuchi N, Oouchida Y, Izumi S-I. Motor Control and Neural Plasticity through Interhemispheric Interactions. Neural Plasticity. 2012;2012. doi: 10.1155/2012/823285. PubMed PMID: WOS:000313169100001.
217. Tarkka IM, Stokic DS. Left prefrontal cortex influences motor imagery. European Journal of Neurology. 2012;19:217-. PubMed PMID: WOS:000309359701044.
218. Tarnawa I. EXPLORING THE ROLE OF SODIUM CHANNEL INHIBITORS IN NEUROLOGICAL AND PSYCHIATRIC DISORDERS. Drugs of the Future. 2012;37(7):499-521. doi: 10.1358/dof.2012.037.07.1811776.

PubMed PMID: WOS:000307112800005.
219. Tasset I, Medina FJ, Jimena I, Agueera E, Gascon F, Feijoo M, et al. NEUROPROTECTIVE EFFECTS OF EXTREMELY LOW-FREQUENCY ELECTROMAGNETIC FIELDS ON A HUNTINGTON'S DISEASE RAT MODEL: EFFECTS ON NEUROTROPHIC FACTORS AND NEURONAL DENSITY. Neuroscience. 2012;209:54-63. doi: 10.1016/j.neuroscience.2012.02.034. PubMed PMID: WOS:000303306600007.
220. Trost S, Gruber O. Evidence for a Double Dissociation of Articulatory Rehearsal and Non-Articulatory Maintenance of Phonological Information in Human Verbal Working Memory. Neuropsychobiology. 2012;65(3):133-40. doi: 10.1159/000332335. PubMed PMID: WOS:000302875300004.
221. Aleksandrova EV, Zaitsev OS, Chelyapina MV, Sharova EV, Sokolova EY, Gavrilov VM, et al. Emergence from protracted unconsciousness due to severe diffuse axonal brain injury: clinical observation and literature review. Nevrologiya, neiropsikhiatriya, psikhosomatika. 2013(2):51-8. PubMed PMID: RSCI:20277676.
222. Balzeau J, Pinier M, Berges R, Saulnier P, Benoit J-P, Eyer J. The effect of functionalizing lipid nanocapsules with NFL-TBS.40-63 peptide on their uptake by glioblastoma cells.

Biomaterials. 2013;34(13):3381-9. doi: 10.1016/j.biomaterials.2013.01.068. PubMed PMID: WOS:000316770100022.

223. Bashir S, Al-Hussain FA, Yoo W-K. Neural plasticity and hemispatial neglect in stroke. Journal of the Pakistan Medical Association. 2013;63(10):1299-301. PubMed PMID: WOS:000330328500019.

224. Baudic S, Attal N, Mhalla A, de Andrade DC, Perrot S, Bouhassira D. Unilateral repetitive transcranial magnetic stimulation of the motor cortex does not affect cognition in patients with fibromyalgia. Journal of Psychiatric Research. 2013;47(1):72-7. doi: 10.1016/j.jpsychires.2012.09.003. PubMed PMID: WOS:000312354400010.

225. Bose P, Hou J, Nelson R, Nissim N, Parmer R, Keener J, et al. Effects of Acute Intrathecal Baclofen in an Animal Model of TBI-Induced Spasticity, Cognitive, and Balance Disabilities. Journal of Neurotrauma. 2013;30(13):1177-91. doi: 10.1089/neu.2012.2740. PubMed PMID: WOS:000321186400009.

226. Carson RG, Kennedy NC. Modulation of human corticospinal excitability by paired associative stimulation. Frontiers in Human Neuroscience. 2013;7. doi: 10.3389/fnhum.2013.00823. PubMed PMID: WOS:000328151600001.

227. Carson RG, Nelson BD, Buick AR, Carroll TJ, Kennedy NC, Mac Cann R. Characterizing Changes in the Excitability of Corticospinal Projections to Proximal Muscles of the Upper Limb. Brain Stimulation. 2013;6(5):760-8. doi: 10.1016/j.brs.2013.01.016. PubMed PMID: WOS:000325046600009.
228. Cha M, Ji Y, Masri R. Motor Cortex Stimulation Activates the Incertothalamic Pathway in an Animal Model of Spinal Cord Injury. Journal of Pain. 2013;14(3):260-9. doi: 10.1016/j.jpain.2012.11.007. PubMed PMID: WOS:000316038200005.
229. Chen F, Li C, Zhu Y-J, Zhao X-Y, Lu B-Q, Wu J. Magnetic nanocomposite of hydroxyapatite ultrathin nanosheets/Fe3O4 nanoparticles: microwave-assisted rapid synthesis and application in pH-responsive drug release. Biomaterials Science. 2013;1(10):1074-81. doi: 10.1039/c3bm60086f. PubMed PMID: WOS:000330137700008.
230. Chen L, Sharma A, Guo J, Muresanu D, Huang H, Skaper S, et al. Conference Report Annual Advances in Neurorestoratology: A Summary of IANR VI and 10th GCNN Conference, Bucharest, Romania, April 4-7, 2013. Cns & Neurological Disorders-Drug Targets. 2013;12(5):547-9. PubMed PMID: WOS:000322202100001.

231. Cheyne DO, Fehlings D. Can neuroimaging help identify effective strategies for constraint therapy in congenital hemiparesis? Developmental Medicine and Child Neurology. 2013;55(10):882-3. doi: 10.1111/dmcn.12238. PubMed PMID: WOS:000324067100006.
232. Dudakov JA, Cole D, Hanash AM, Young LF, Kreines FM, Levy ER, et al. Intrathymic Innate Lymphoid Cells: Long-Lived Mediators Of Immune Regeneration. Blood. 2013;122(21). doi: 10.1182/blood.V122.21.289.289. PubMed PMID: WOS:000331385000316.
233. Ganguly K, Byl NN, Abrams GM. Neurorehabilitation: Motor Recovery After Stroke as an Example. Annals of Neurology. 2013;74(3):373-81. doi: 10.1002/ana.23994. PubMed PMID: WOS:000325463100013.
234. Garcia G, Norise C, Faseyitan O, Naeser MA, Hamilton RH. Utilizing Repetitive Transcranial Magnetic Stimulation to Improve Language Function in Stroke Patients with Chronic Non-fluent Aphasia. Jove-Journal of Visualized Experiments. 2013(77). doi: 10.3791/50228. PubMed PMID: WOS:000209227900006.
235. George MS, Massimini M. Using Brain Stimulation to Create Thoughts, Retrieve and Alter Memories, and Measure Consciousness - A Discussion of Recent Research. Brain

Stimulation. 2013;6(6):835-6. doi: 10.1016/j.brs.2013.09.011. PubMed PMID: WOS:000327908600001.

236. George MS, Raman R, Jain S, Benedek DM, Pelic CG, Grammer GG, et al. A Two-site Pilot Study Suggests that Three Days (9 Sessions) of High Dose Left Prefrontal Repetitive Transcranial Magnetic Stimulation (rTMS) is Feasible, Safe, and Reduces Suicidal Thinking in Suicidal Inpatients. Neuropsychopharmacology. 2013;38:S421-S2. PubMed PMID: WOS:000209477100662.

237. Gerardo-Nava J, Mayorenko II, Grehl T, Steinbusch HWM, Weis J, Brook GA. Differential pattern of neuroprotection in lumbar, cervical and thoracic spinal cord segments in an organotypic rat model of glutamate-induced excitotoxicity. Journal of Chemical Neuroanatomy. 2013;53:11-7. doi: 10.1016/j.jchemneu.2013.09.007. PubMed PMID: WOS:000329421900002.

238. Giacino JT, Katz DI, Whyte J. Neurorehabilitation in Disorders of Consciousness. Seminars in Neurology. 2013;33(2):142-56. doi: 10.1055/s-0033-1348960. PubMed PMID: WOS:000322225300008.

239. He J, Yang Y, Jiao H, Dang Y, Dai Y, Zhang Q, et al. Neruomodulation treatment for patients under persistent vegetative

state. Chinese Journal of Neuromedicine. 2013;12(12):1197-200. PubMed PMID: CSCD:5036754.

240. He X, Lan Y, Xu G, Mao Y, Chen Z, Huang D, et al. Frontoparietal regions may become hypoactive after intermittent theta burst stimulation over the contralateral homologous cortex in humans. Journal of Neurophysiology. 2013;110(12):2849-56. doi: 10.1152/jn.00369.2013. PubMed PMID: WOS:000328889700014.

241. Inguaggiato E, Sgandurra G, Perazza S, Guzzetta A, Cioni G. Brain Reorganization following Intervention in Children with Congenital Hemiplegia: A Systematic Review. Neural Plasticity. 2013;2013. doi: 10.1155/2013/356275. PubMed PMID: WOS:000328141300001.

242. Kaplitt MG. Spinal Cord Stimulation for Vegetative State: Is This Ready for Prime Time? Stereotactic and Functional Neurosurgery. 2013;91(5):288-9. doi: 10.1159/000348272. PubMed PMID: WOS:000323778500002.

243. Kapur N, Cole J, Manly T, Viskontas I, Ninteman A, Hasher L, et al. Positive Clinical Neuroscience: Explorations in Positive Neurology. Neuroscientist. 2013;19(4):354-69. doi: 10.1177/1073858412470976. PubMed PMID: WOS:000330518600008.

244. Kibria G, Hatakeyama H, Ohga N, Hida K, Harashima H. The

effect of liposomal size on the targeted delivery of doxorubicin to Integrin alpha v beta 3-expressing tumor endothelial cells. Biomaterials. 2013;34(22):5617-27. doi: 10.1016/j.biomaterials.2013.03.094. PubMed PMID: WOS:000319855600018.

245. Kozel FA, Croarkin PE, Mapes KS. A Non-Epileptiform Event in the Course of rTMS: A Case for Close Physician Monitoring. Brain Stimulation. 2013;6(6):970-2. doi: 10.1016/j.brs.2013.04.003. PubMed PMID: WOS:000327908600023.

246. Miller J, Eakin K, Sweet J. Theta Burst Stimulation Of The Hippocampus Via An Electrode Implanted In The Fornix Is Associated With Improved Cognitive Performance After Moderate Traumatic Brain Injury. Stereotactic and Functional Neurosurgery. 2013;91:78-. PubMed PMID: WOS:000209547400077.

247. Ng YS, Chew E, Samuel GS, Tan YL, Kong KH. Advances in rehabilitation medicine. Singapore Medical Journal. 2013;54(10):538-51. PubMed PMID: WOS:000327245300008.

248. Olson LD, Perry MS. LOCALIZATION OF EPILEPTIC FOCI USING MULTIMODALITY NEUROIMAGING. International Journal of Neural Systems. 2013;23(1). doi: 10.1142/s012906571230001x. PubMed PMID: WOS:000316807400002.

249. Ommaya AK, Adams KM, Allman RM, Collins EG, Cooper RA, Dixon CE, et al. Opportunities in rehabilitation research. Journal of Rehabilitation Research and Development. 2013;50(6):VII-XXXII. doi: 10.1682/jrrd.2012.09.0167. PubMed PMID: WOS:000324350200001.
250. Opisso E, Borau A, Rijkhoff NJM. Subject-Controlled Stimulation of Dorsal Genital Nerve to Treat Neurogenic Detrusor Overactivity at Home. Neurourology and Urodynamics. 2013;32(7):1004-9. doi: 10.1002/nau.22359. PubMed PMID: WOS:000323169800013.
251. Perreault M-C, Glover JC. Glutamatergic reticulospinal neurons in the mouse: developmental origins, axon projections, and functional connectivity. In: ZiskindConhaim L, MacDermott AB, Houle JD, Alvarez FJ, Hochman S, editors. Neurons, Circuitry, and Plasticity in the Spinal Cord and Brainstem. Annals of the New York Academy of Sciences. 12792013. p. 80-9.
252. Petzold A, Girbes A. Pain Management in Neurocritical Care. Neurocritical Care. 2013;19(2):232-56. doi: 10.1007/s12028-013-9851-0. PubMed PMID: WOS:000324644800016.
253. Porcher NJ, Solecki TJ. A narrative review of sports-related concussion and return-to-play testing with asymptomatic athletes.

Journal of chiropractic medicine. 2013;12(4):260-8. doi: 10.1016/j.jcm.2013.08.002. PubMed PMID: MEDLINE:24396328.
254. Qiu D, An X. Controllable release from magnetoliposomes by magnetic stimulation and thermal stimulation. Colloids and Surfaces B-Biointerfaces. 2013;104:326-9. doi: 10.1016/j.colsurfb.2012.11.033. PubMed PMID: WOS:000316645100047.
255. Schif ND. Making Waves in Consciousness Research. Science Translational Medicine. 2013;5(198). doi: 10.1126/scitranslmed.3007004. PubMed PMID: WOS:000323132200009.
256. Serpero LD, Bellissima V, Colivicchi M, Sabatini M, Frigiola A, Ricotti A, et al. Next generation biomarkers for brain injury. Journal of Maternal-Fetal & Neonatal Medicine. 2013;26:44-9. doi: 10.3109/14767058.2013.829688. PubMed PMID: WOS:000324833900013.
257. Song J-J, Punte AK, De Ridder D, Vanneste S, Van de Heyning P. Neural substrates predicting improvement of tinnitus after cochlear implantation in patients with single-sided deafness. Hearing Research. 2013;299:1-9. doi: 10.1016/j.heares.2013.02.001. PubMed PMID: WOS:000319483700001.

258. Tan T, Xie J, Liu T, Chen X, Zheng X, Tong Z, et al. Low-frequency (1Hz) repetitive transcranial magnetic stimulation (rTMS) reverses A beta(1-42)-mediated memory deficits in rats. Experimental Gerontology. 2013;48(8):786-94. doi: 10.1016/j.exger.2013.05.001. PubMed PMID: WOS:000321700300013.

259. Tarapore PE, Findlay AM, Honma SM, Mizuiri D, Houde JF, Berger MS, et al. Language mapping with navigated repetitive TMS: Proof of technique and validation. Neuroimage. 2013;82:260-72. doi: 10.1016/j.neuroimage.2013.05.018. PubMed PMID: WOS:000324568400027.

260. Tremblay S, Beaule V, Proulx S, de Beaumont L, Marjanska M, Doyon J, et al. Relationship between transcranial magnetic stimulation measures of intracortical inhibition and spectroscopy measures of GABA and glutamate plus glutamine. Journal of Neurophysiology. 2013;109(5):1343-9. doi: 10.1152/jn.00704.2012. PubMed PMID: WOS:000315681100012.

261. Tyler ME, Danilov YP, Kaczmarek K. CRANIAL NERVE NON-INVASIVE NEUROMODULATION FOR SYMPTOMATIC TREATMENT OF MILD AND MODERATE TRAUMATIC BRAIN INJURY. Journal of Neurotrauma. 2013;30(15):A51-A2. PubMed PMID: WOS:000322446800095.

262. Van Vleet TM, DeGutis JM. The Nonspatial Side of Spatial Neglect and Related Approaches to Treatment. In: Merzenich MM, Nahum M, VanVleet TM, editors. Changing Brains Applying Brain Plasticity to Advance and Recover Human Ability. Progress in Brain Research. 2072013. p. 327-49.
263. Wang P-C, Paik S-J, Chen S, Rajaraman S, Kim S-H, Allen MG. Fabrication and Characterization of Polymer Hollow Microneedle Array Using UV Lithography Into Micromolds. Journal of Microelectromechanical Systems. 2013;22(5):1041-53. doi: 10.1109/jmems.2013.2262587. PubMed PMID: WOS:000325410300011.
264. 윤상희, 윤서영, 이진석. Ultrasound-Aided Monolayer Assembly of Spherical Silica Nanobeads. Applied Science and Convergence Technology. 2013;22(6):298-305. PubMed PMID: KJD:ART001820151.
265. 30th International Congress of Clinical Neurophysiology of the International Federation of Clinical Neurophysiology, Berlin, Germany, March 20-23, 2014 Abstracts. Clinical Neurophysiology. 2014;125:S1-S339. PubMed PMID: WOS:000361971100001.
266. Allen CPG, Dunkley BT, Muthukumaraswamy SD, Edden R, Evans CJ, Sumner P, et al. Enhanced Awareness Followed Reversible Inhibition of Human Visual Cortex: A Combined TMS,

MRS and MEG Study. Plos One. 2014;9(6). doi: 10.1371/journal.pone.0100350. PubMed PMID: WOS:000338917900042.

267. Babiloni C, Vecchio F, Buffo P, Iacoboni M, Pistoia F, Sacco S, et al. Mechanisms of Cortical Neural Synchronization Related to Healthy and Impaired Consciousness: Evidence by Quantitative Electroencephalographic Studies. Current Pharmaceutical Design. 2014;20(26):4225-38. PubMed PMID: WOS:000338508000009.

268. Bachtiar V, Stagg CJ. THE ROLE OF INHIBITION IN HUMAN MOTOR CORTICAL PLASTICITY. Neuroscience. 2014;278:93-104. doi: 10.1016/j.neuroscience.2014.07.059. PubMed PMID: WOS:000342058700010.

269. Bate S, Bennetts RJ. The rehabilitation of face recognition impairments: a critical review and future directions. Frontiers in Human Neuroscience. 2014;8. doi: 10.3389/fnhum.2014.00491. PubMed PMID: WOS:000340564400001.

270. Birbaumer N. Neural Signatures of Modified Memories. Neuron. 2014;81(1):3-5. doi: 10.1016/j.neuron.2013.12.019. PubMed PMID: WOS:000329559000002.

271. Bonni S, Perri R, Fadda L, Tornaiuolo F, Koch G, Caltagirone C, et al. Selective deficit of spatial short-term memory: Role of storage and rehearsal mechanisms. Cortex. 2014;59:22-32. doi:

10.1016/j.cortex.2014.06.004. PubMed PMID: WOS:000343838500003.

272. Brody AL, Okita K, Shieh J, Liang L, Hubert R, Mamoun M, et al. Radiation dosimetry and biodistribution of the translocator protein radiotracer C-11 DAA1106 determined with PET/CT in healthy human volunteers. Nuclear Medicine and Biology. 2014;41(10):871-5. doi: 10.1016/j.nucmedbio.2014.07.004. PubMed PMID: WOS:000343614800013.

273. Burke E, Dodakian L, See J, McKenzie A, Riley JD, Le V, et al. A multimodal approach to understanding motor impairment and disability after stroke. Journal of Neurology. 2014;261(6):1178-86. doi: 10.1007/s00415-014-7341-8. PubMed PMID: WOS:000337750700017.

274. Cai B, Xia W, Bredenberg S, Engqvist H. Self-setting bioceramic microscopic protrusions for transdermal drug delivery. Journal of Materials Chemistry B. 2014;2(36):5992-8. doi: 10.1039/c4tb00764f. PubMed PMID: WOS:000341300300007.

275. Deer TR, Mekhail N, Petersen E, Krames E, Staats P, Pope J, et al. The Appropriate Use of Neurostimulation: Stimulation of the Intracranial and Extracranial Space and Head for Chronic Pain. Neuromodulation. 2014;17(6):551-70. doi: 10.1111/ner.12215. PubMed PMID: WOS:000340500200004.

276. Deng Y-L, Juang Y-J. Polydimethyl siloxane wet etching for three dimensional fabrication of microneedle array and high-aspect-ratio micropillars. Biomicrofluidics. 2014;8(2). doi: 10.1063/1.4871038. PubMed PMID: WOS:000336082100027.
277. Glannon W. Neuromodulation, Agency and Autonomy. Brain Topography. 2014;27(1):46-54. doi: 10.1007/s10548-012-0269-3. PubMed PMID: WOS:000329226500006.
278. Gosseries O, Laureys S. Current knowledge on severe acquired brain injury with disorders of consciousness. Brain Injury. 2014;28(9):1139-40. doi: 10.3109/02699052.2014.932554. PubMed PMID: WOS:000340455700001.
279. Gosseries O, Zasler ND, Laureys S. Recent advances in disorders of consciousness: Focus on the diagnosis. Brain Injury. 2014;28(9):1141-50. doi: 10.3109/02699052.2014.920522. PubMed PMID: WOS:000340455700002.
280. Grefkes C, Ward NS. Cortical Reorganization After Stroke: How Much and How Functional? Neuroscientist. 2014;20(1):56-70. doi: 10.1177/1073858413491147. PubMed PMID: WOS:000329919300011.
281. Guerra-Carrillo B, Mackey AP, Bunge SA. Resting-State fMRI: A Window into Human Brain Plasticity. Neuroscientist. 2014;20(5):522-33. doi: 10.1177/1073858414524442. PubMed

PMID: WOS:000342640800013.
282. Gun TK, Gul JD, Hee EP. EFFECTS OF DUAL MODE NON INVASIVE BRAIN STIMULATION ON MOTOR FUNCTION IN PATIENTS WITH CHRONIC TRAUMATIC BRAIN INJURY. Journal of Neurotrauma. 2014;31(12):A45-A. PubMed PMID: WOS:000338443600120.
283. Jang SH. THE CORTICOSPINAL TRACT FROM THE VIEWPOINT OF BRAIN REHABILITATION. Journal of Rehabilitation Medicine. 2014;46(3):193-9. doi: 10.2340/16501977-1782. PubMed PMID: WOS:000332903400001.
284. Jiang L, Ji Y, Voulalas PJ, Keaser M, Xu S, Gullapalli RP, et al. Motor Cortex Stimulation Suppresses Cortical Responses to Noxious Hindpaw Stimulation After Spinal Cord Lesion in Rats. Brain Stimulation. 2014;7(2):182-9. doi: 10.1016/j.brs.2013.12.013. PubMed PMID: WOS:000333442200005.
285. Kaczmarek K, Tyler M, Skinner K, Verbny Y, Danilov Y. Cranial nerve non-invasive neuromodulation (CN-NINM) for symptomatic treatment of mild and moderate traumatic brain injury. Brain Injury. 2014;28(5-6):605-. PubMed PMID: WOS:000335017000219.
286. Kelly C, Castellanos FX. Strengthening Connections:

Functional Connectivity and Brain Plasticity. Neuropsychology Review. 2014;24(1):63-76. doi: 10.1007/s11065-014-9252-y. PubMed PMID: WOS:000335812400006.
287. Kim DR, Wang E. Prevention of supine hypotensive syndrome in pregnant women treated with transcranial magnetic stimulation. Psychiatry Research. 2014;218(1-2):247-8. doi: 10.1016/j.psychres.2014.04.001. PubMed PMID: WOS:000338614500042.
288. Lancioni GE, Bosco A, Belardinelli MO, Singh NN, O'Reilly ME, Sigafoos J, et al. Technology-based intervention programs to promote stimulation control and communication in post-coma persons with different levels of disability. Frontiers in Human Neuroscience. 2014;8. doi: 10.3389/fnhum.2014.00048. PubMed PMID: WOS:000331945400001.
289. Langsjo JW, Revonsuo A, Scheinin H. Harnessing Anesthesia and Brain Imaging for the Study of Human Consciousness. Current Pharmaceutical Design. 2014;20(26):4211-24. PubMed PMID: WOS:000338508000008.
290. Lefaucheur J-P, Andre-Obadia N, Antal A, Ayache SS, Baeken C, Benninger DH, et al. Evidence-based guidelines on the therapeutic use of repetitive transcranial magnetic stimulation (rTMS). Clinical Neurophysiology. 2014;125(11):2150-206. doi:

10.1016/j.clinph.2014.05.021. PubMed PMID: WOS:000345519600005.

291. Leung A, Shukla S, Song DD, Lin L, Tsai AM, Polston G, et al. rTMS in Reducing Mild TBI Related Headache- a Pilot Study. Headache. 2014;54:57-. PubMed PMID: WOS:000337950600111.

292. Liew S-L, Santarnecchi E, Buch ER, Cohen LG. Non-invasive brain stimulation in neurorehabilitation: local and distant effects for motor recovery. Frontiers in Human Neuroscience. 2014;8. doi: 10.3389/fnhum.2014.00378. PubMed PMID: WOS:000339945300002.

293. Masopust V, Rokyta R, Benes V. Neuromodulation. Ceska a Slovenska Neurologie a Neurochirurgie. 2014;77(2):138-51. PubMed PMID: WOS:000333662100001.

294. Matsumoto-Miyazaki J, Yonezawa S, Nishiyama N, Okumura R, Fukuyama S, Kanematsu Y, et al. Acupuncture treatment increases motor evoked potentials induced by using transcranial magnetic stimulation in patients with chronic disorder of consciousness following severe traumatic brain injury. Brain Injury. 2014;28(5-6):597-8. PubMed PMID: WOS:000335017000202.

295. Muthukumaraswamy SD. The use of magnetoencephalography in the study of psychopharmacology (pharmaco-MEG). Journal of Psychopharmacology.

2014;28(9):815-29. doi: 10.1177/0269881114536790. PubMed PMID: WOS:000340182700001.
296. Neva JL, Singh AM, Vesia M, Staines WR. Selective modulation of left primary motor cortex excitability after continuous theta burst stimulation to right primary motor cortex and bimanual training. Behavioural Brain Research. 2014;269:138-46. doi: 10.1016/j.bbr.2014.04.041. PubMed PMID: WOS:000337866400018.
297. Neva JL, Vesia M, Singh AM, Staines WR. Modulation of left primary motor cortex excitability after bimanual training and intermittent theta burst stimulation to left dorsal premotor cortex. Behavioural Brain Research. 2014;261:289-96. doi: 10.1016/j.bbr.2013.12.029. PubMed PMID: WOS:000331497000036.
298. Pape T, Rosenow J, Patil V, Steiner M, Harton B, Wang X, et al. TMS related changes in functional connectivity of default mode network and associated neurobehavioural gains for subjects in vegetative state after traumatic brain injury. Brain Injury. 2014;28(5-6):708-. PubMed PMID: WOS:000335017000463.
299. Patuzzo S, Manganotti P. Deep Brain Stimulation in Persistent Vegetative States: Ethical Issues Governing Decision Making. Behavioural Neurology. 2014;2014. doi: 10.1155/2014/641213.

PubMed PMID: WOS:000333597000001.

300. Prichard G, Weiner C, Fritsch B, Reis J. Effects of Different Electrical Brain Stimulation Protocols on Subcomponents of Motor Skill Learning. Brain Stimulation. 2014;7(4):532-40. doi: 10.1016/j.brs.2014.04.005. PubMed PMID: WOS:000339984300006.

301. Rollnik JD, Altenmueller E. Music in disorders of consciousness. Frontiers in Neuroscience. 2014;8. doi: 10.3389/fnins.2014.00190. PubMed PMID: WOS:000346496900001.

302. Schintu S, Hadj-Bouziane F, Dal Monte O, Knutson KM, Pardini M, Wassermann EM, et al. Object and space perception - Is it a matter of hemisphere? Cortex. 2014;57:244-53. doi: 10.1016/j.cortex.2014.04.009. PubMed PMID: WOS:000340317500018.

303. Seidl SE, Izadi A, Lee DJ, Melnik M, Ekstrom AD, Shahlaie K, et al. STIMULATION OF THE MEDIAL SEPTUM DRIVES HIPPOCAMPAL THETA OSCILLATIONS LEADING TO PERSISTENT INCREASES IN THETA PHASE COHERENCE. Journal of Neurotrauma. 2014;31(12):A98-A9. PubMed PMID: WOS:000338443600267.

304. Thibaut A, Bruno M-A, Ledoux D, Demertzi A, Laureys S.

tDCS in patients with disorders of consciousness. Neurology. 2014;82(13):1112-8. doi: 10.1212/wnl.0000000000000260. PubMed PMID: WOS:000335977100006.

305. Thompson FJ, Hou J, Mustafa G, Wilkie Z, Tsuda S, Nelson R, et al. THERAPEUTIC TMS REDUCES TBI SPASTICITY, ANXIETY, AND PAIN. Journal of Neurotrauma. 2014;31(12):A47-A. PubMed PMID: WOS:000338443600126.

306. Tremblay S, Lepage J-F, Latulipe-Loiselle A, Fregni F, Pascual-Leone A, Theoret H. The Uncertain Outcome of Prefrontal tDCS. Brain Stimulation. 2014;7(6):773-83. doi: 10.1016/j.brs.2014.10.003. PubMed PMID: WOS:000346455200002.

307. Urgesi C, Candidi M, Avenanti A. Neuroanatonnical substrates of action perception and understanding: an anatomic likelihood estimation meta-analysis of lesion-symptom mapping studies in brain injured patients. Frontiers in Human Neuroscience. 2014;8. doi: 10.3389/fnhum.2014.00344. PubMed PMID: WOS:000339500600001.

308. Wang P-C, Paik S-J, Kim S-H, Allen MG. Hypodermic-Needle-Like Hollow Polymer Microneedle Array: Fabrication and Characterization. Journal of Microelectromechanical Systems. 2014;23(4):991-8. doi:

10.1109/jmems.2014.2307320. PubMed PMID: WOS:000341573300027.

309. Wertheimer T, Kloss CC, Velardi E, Brede C, Ginsberg M, Kreines FM, et al. Endothelial Cells Promote Endogenous Thymic Regeneration after Injury Via BMP4 Signaling. Blood. 2014;124(21). PubMed PMID: WOS:000349243501095.

310. Xerri C, Zennou-Azogui Y, Sadlaoud K, Sauvajon D. INTERPLAY BETWEEN INTRA- AND INTERHEMISPHERIC REMODELING OF NEURAL NETWORKS AS A SUBSTRATE OF FUNCTIONAL RECOVERY AFTER STROKE: ADAPTIVE VERSUS MALADAPTIVE REORGANIZATION. Neuroscience. 2014;283:178-201. doi: 10.1016/j.neuroscience.2014.06.066. PubMed PMID: WOS:000345513100013.

311. Yu Y, Liang E, Zhang L, Zhu Z, Yan H. Effect of early rehabilitation in patients with severe traumatic brain injury. Chinese Journal of Trauma. 2014;30(5):400-3. PubMed PMID: CSCD:5162287.

312. Zhang X, Chen H, Liang E. Neuromodulation (cervical spinal cord stimulation, cSCS) on a comatose patient due to traumatic brain injury-One case report. Brain Injury. 2014;28(5-6):531-. PubMed PMID: WOS:000335017000039.

313. Agashe S, Foreman J. The Efficacy of Transcranial Magnetic

Stimulation in Long Term Rehabilitation Following Traumatic Brain Injury - A Case Report. Annals of Neurology. 2015;78:S44-S. PubMed PMID: WOS:000362668600096.

314. Amen DG, Raji CA, Willeumier K, Taylor D, Tarzwell R, Newberg A, et al. Functional Neuroimaging Distinguishes Posttraumatic Stress Disorder from Traumatic Brain Injury in Focused and Large Community Datasets. Plos One. 2015;10(7). doi: 10.1371/journal.pone.0129659. PubMed PMID: WOS:000358153000031.

315. Bodart O, Wannez S, Thibaut A, Annen J, Casali AG, Casarotto S, et al. Multimodal TMS/EEG, FDG-PET and active fMRI assessments in patients with disorders of consciousness and locked-in syndrome. European Journal of Neurology. 2015;22:102-3. PubMed PMID: WOS:000357033500163.

316. Bolu A, Balikci A, Erdem M, Oznur T, Celik C, Uzun O. Cortical Excitability and Agressive Behavior in Post-Traumatic Stress Disorder. Noropsikiyatri Arsivi-Archives of Neuropsychiatry. 2015;52(1):73-7. doi: 10.5152/npa.2015.7031. PubMed PMID: WOS:000351851100015.

317. Broeer S, Loescher W. Novel combinations of phenotypic biomarkers predict development of epilepsy in the lithium-pilocarpine model of temporal lobe epilepsy in rats.

Epilepsy & Behavior. 2015;53:98-107. doi: 10.1016/j.yebeh.2015.09.028. PubMed PMID: WOS:000366328200017.

318. Chang S, Ranjan M, Slotty PJ, Honey CR. The Influence of Positioning and Muscle Activity on Motor Threshold during Motor Cortex Stimulation Programming. Stereotactic and Functional Neurosurgery. 2015;93(2):122-6. doi: 10.1159/000369355. PubMed PMID: WOS:000351486500007.

319. Chantsoulis M, Mirski A, Rasmus A, Kropotov JD, Pachalska M. Neuropsychological rehabilitation for traumatic brain injury patients. Annals of Agricultural and Environmental Medicine. 2015;22(2):368-79. doi: 10.5604/12321966.1152097. PubMed PMID: WOS:000356635800036.

320. Chen M-C, Ling M-H, Kusuma SJ. Poly-gamma-glutamic acid microneedles with a supporting structure design as a potential tool for transdermal delivery of insulin. Acta Biomaterialia. 2015;24:106-16. doi: 10.1016/j.actbio.2015.06.021. PubMed PMID: WOS:000360867700011.

321. Chen Y, Kosmas P, Anwar PS, Huang L. A Touch-Communication Framework for Drug Delivery Based on a Transient Microbot System. Ieee Transactions on Nanobioscience. 2015;14(4):397-408. doi: 10.1109/tnb.2015.2395539. PubMed

PMID: WOS:000359565800009.
322. Corbett D, Jeffers M, Nguemeni C, Gomez-Smith M, Livingston-Thomas J. Lost in translation: rethinking approaches to stroke recovery. In: Dancause N, Nadeau S, Rossignol S, editors. Sensorimotor Rehabilitation at the Crossroads of Basic and Clinical Sciences. Progress in Brain Research. 2182015. p. 413-34.
323. Guerriero RM, Giza CC, Rotenberg A. Glutamate and GABA Imbalance Following Traumatic Brain Injury. Current Neurology and Neuroscience Reports. 2015;15(5). doi: 10.1007/s11910-015-0545-1. PubMed PMID: WOS:000352317600009.
324. Johnen VM, Neubert F-X, Buch ER, Verhagen LM, O'Reilly J, Mars RB, et al. Causal manipulation of functional connectivity in a specific neural pathway during behaviour and at rest. Elife. 2015;4. doi: 10.7554/eLife.04585. PubMed PMID: WOS:000349460700002.
325. Lancioni GE, Singh NN, O'Reilly MF, Sigafoos J, D'Amico F, Buonocunto F, et al. Assistive technology to help persons in a minimally conscious state develop responding and stimulation control: Performance assessment and social rating. Neurorehabilitation. 2015;37(3):393-403. doi: 10.3233/nre-151269. PubMed PMID: WOS:000366324100006.

326. Lee IC, He J-S, Tsai M-T, Lin K-C. Fabrication of a novel partially dissolving polymer microneedle patch for transdermal drug delivery. Journal of Materials Chemistry B. 2015;3(2):276-85. doi: 10.1039/c4tb01555j. PubMed PMID: WOS:000346061000014.
327. Lemmens S, Brone B, Dooley D, Hendrix S, Geurts N. Alpha-Adrenoceptor Modulation in Central Nervous System Trauma: Pain, Spasms, and Paralysis - An Unlucky Triad. Medicinal Research Reviews. 2015;35(4):653-77. doi: 10.1002/med.21337. PubMed PMID: WOS:000356068800001.
328. Li LM, Uehara K, Hanakawa T. The contribution of interindividual factors to variability of response in transcranial direct current stimulation studies. Frontiers in Cellular Neuroscience. 2015;9. doi: 10.3389/fncel.2015.00181. PubMed PMID: WOS:000354803600003.
329. Litvinenko IV, Odinak MM, Emelin AY, Vorobyev SV, Yurin AA. Role of transcranial magnetic stimulation in diagnostics of pathophysiological mechanisms of cognitive impairment in traumatic brain injury. Bulletin of the Russian Military Medical Academy. 2015(3):249-54. PubMed PMID: RSCI:25808282.
330. Liu J, Zhao F, Wu J, Wu S, Yang A, Li X, et al. Features of survived casualties and treatment strategies in Kunshan August 2"

explosion aaccident. Chinese Journal of Emergency Medicine. 2015;24(9):952-7. PubMed PMID: CSCD:5510601.

331. Morey RA, Lancaster SC, Haswell CC. Trauma Re-experiencing Symptoms Modulate Topology of Intrinsic Functional Networks. Biological Psychiatry. 2015;78(3):156-8. doi: 10.1016/j.biopsych.2015.06.001. PubMed PMID: WOS:000357489000004.

332. Muralidharan P, Malapit M, Mallory E, Hayes D, Jr., Mansour HM. Inhalable nanoparticulate powders for respiratory delivery. Nanomedicine-Nanotechnology Biology and Medicine. 2015;11(5):1189-99. doi: 10.1016/j.nano.2015.01.007. PubMed PMID: WOS:000363967100016.

333. Neggers SFW, Petrov PI, Mandija S, Sommer IEC, van den Berg NAT. Understanding the biophysical effects of transcranial magnetic stimulation on brain tissue: The bridge between brain stimulation and cognition. In: Bestmann S, editor. Computational Neurostimulation. Progress in Brain Research. 2222015. p. 229-59.

334. Neva JL, Vesia M, Singh AM, Staines WR. Bilateral primary motor cortex circuitry is modulated due to theta burst stimulation to left dorsal premotor cortex and bimanual training. Brain Research. 2015;1618:61-74. doi: 10.1016/j.brainres.2015.05.028. PubMed PMID: WOS:000359957600008.

335. Nielson DM, McKnight CA, Patel RN, Kalnin AJ, Mysiw WJ. Preliminary Guidelines for Safe and Effective Use of Repetitive Transcraniat Magnetic Stimulation in Moderate to Severe Traumatic Brain Injury. Archives of Physical Medicine and Rehabilitation. 2015;96(4):S138-S44. doi: 10.1016/j.apmr.2014.09.010. PubMed PMID: WOS:000352179900007.
336. Page SJ, Cunningham DA, Plow E, Blazak B. It Takes Two: Noninvasive Brain Stimulation Combined With Neurorehabilitation INTRODUCTION. Archives of Physical Medicine and Rehabilitation. 2015;96(4):S89-S93. doi: 10.1016/j.apmr.2014.09.019. PubMed PMID: WOS:000352179900001.
337. Paiva WS, Neville IS, Fregni F, Teixeira MJ. Is transcranial magnetic stimulation useful in posttraumatic disorders? Neural Regeneration Research. 2015;10(9):1528-. doi: 10.4103/1673-5374.165529. PubMed PMID: WOS:000362625000041.
338. Quinlan EB, Dodakian L, See J, McKenzie A, Le V, Wojnowicz M, et al. Neural Function, Injury, and Stroke Subtype Predict Treatment Gains after Stroke. Annals of Neurology. 2015;77(1):132-45. doi: 10.1002/ana.24309. PubMed PMID:

WOS:000348423200012.

339. Raji CA, Willeumier K, Taylor D, Tarzwell R, Newberg A, Henderson TA, et al. Functional neuroimaging with default mode network regions distinguishes PTSD from TBI in a military veteran population. Brain Imaging and Behavior. 2015;9(3):527-34. doi: 10.1007/s11682-015-9385-5. PubMed PMID: WOS:000361604300013.

340. Sarasso S, Boly M, Napolitani M, Gosseries O, Charland-Verville V, Casarotto S, et al. Consciousness and Complexity during Unresponsiveness Induced by Propofol, Xenon, and Ketamine. Current Biology. 2015;25(23):3099-105. doi: 10.1016/j.cub.2015.10.014. PubMed PMID: WOS:000366388600025.

341. Schartner M, Seth A, Noirhomme Q, Boly M, Bruno M-A, Laureys S, et al. Complexity of Multi-Dimensional Spontaneous EEG Decreases during Propofol Induced General Anaesthesia. Plos One. 2015;10(8). doi: 10.1371/journal.pone.0133532. PubMed PMID: WOS:000359121100020.

342. Schatzberg AF. Issues encountered in recent attempts to develop novel antidepressant agents. In: Fink ME, Barchas JD, Sheikh JI, editors. Affective Disorders and Traumatic Brain Injury. Annals of the New York Academy of Sciences. 13452015. p.

67-73.

343. Sidiakina IV, Dobrushina OR, Liadov KV, Shapovalenko TV, Romashin OV. The role of evidence-based medicine in the neurorehabilitation: the innovative technologies (a review). Voprosy kurortologii, fizioterapii, i lechebnoi fizicheskoi kultury. 2015;92(3):53-6. doi: 10.17116/kurort2015353-56. PubMed PMID: MEDLINE:26285335.

344. Simon DT, Larsson KC, Nilsson D, Burstrom G, Galter D, Berggren M, et al. An organic electronic biomimetic neuron enables auto-regulated neuromodulation. Biosensors & Bioelectronics. 2015;71:359-64. doi: 10.1016/j.bios.2015.04.058. PubMed PMID: WOS:000356646000052.

345. Slotty PJ, Eisner W, Honey CR, Wille C, Vesper J. Long-Term Follow-Up of Motor Cortex Stimulation for Neuropathic Pain in 23 Patients. Stereotactic and Functional Neurosurgery. 2015;93(3):199-205. doi: 10.1159/000375177. PubMed PMID: WOS:000355200800008.

346. Sun M-K, Nelson TJ, Alkon DL. Towards universal therapeutics for memory disorders. Trends in Pharmacological Sciences. 2015;36(6):384-94. doi: 10.1016/j.tips.2015.04.004. PubMed PMID: WOS:000356636900007.

347. Syroezhkin FA, Nikitin NI, Dvorianchikov VV, Golovanov

AE, Morozova MV, Letyagin AI. Neural plasticity in current tinnitus conception: opportunity for audial training. Bulletin of the Russian Military Medical Academy. 2015(3):94-8. PubMed PMID: RSCI:25808252.

348. Tekriwal A, Baltuch G. Deep Brain Stimulation: Expanding Applications. Neurologia Medico-Chirurgica. 2015;55(12):861-77. doi: 10.2176/nmc.ra.2015-0172. PubMed PMID: WOS:000367428900001.

349. Wertheimer T, Velardi E, Brede C, Xiao S, Kloss CC, Beilhack A, et al. Production of BMP4 By Endothelial Cells Is Crucial for Endogenous Thymic Regeneration. Blood. 2015;126(23). PubMed PMID: WOS:000368019002074.

350. Wessel MJ, Zimerman M, Hummel FC. Non-invasive brain stimulation: an interventional tool for enhancing behavioral training after stroke. Frontiers in Human Neuroscience. 2015;9. doi: 10.3389/fnhum.2015.00265. PubMed PMID: WOS:000356060400001.

351. Xie Y, Zhang T, Chen ACN. Repetitive Transcranial Magnetic Stimulation for the Recovery of Stroke Patients With Disturbance of Consciousness. Brain Stimulation. 2015;8(3):674-5. doi: 10.1016/j.brs.2015.01.406. PubMed PMID: WOS:000355772300038.

352. Xu J, Wang D, Wang J, Wang J, Hu L. Preliminary Quantitative Evaluation of Potential Risk for HCCB TBS Tritium Extraction System Based on Fault Tree Analysis. Journal of Fusion Energy. 2015;34(6):1369-77. doi: 10.1007/s10894-015-9972-x. PubMed PMID: WOS:000363490000022.

353. Yin Z, Shen Y, Reinhardt JD, Chen C-F, Jiang X, Dai W, et al. 5 Hz Repetitive Transcranial Magnetic Stimulation with Maximum Voluntary Muscle Contraction Facilitates Cerebral Cortex Excitability of Normal Subjects. Cns & Neurological Disorders-Drug Targets. 2015;14(10):1298-303. doi: 10.2174/1871527315666151111124216. PubMed PMID: WOS:000365808000009.

354. Angelakis E, Konstantinidi M, Sakas D. Clinical improvement with transcranial direct current stimulation (tDCS) in disorders of consciousness due to TBI. Brain Injury. 2016;30(5-6):613-4. PubMed PMID: WOS:000376388200304.

355. Barlow KM. Postconcussion Syndrome: A Review. Journal of Child Neurology. 2016;31(1):57-67. doi: 10.1177/0883073814543305. PubMed PMID: WOS:000366650400006.

356. Bikson M, Grossman P, Thomas C, Zannou AL, Jiang J, Adnan T, et al. Safety of Transcranial Direct Current Stimulation:

Evidence Based Update 2016. Brain Stimulation. 2016;9(5):641-61. doi: 10.1016/j.brs.2016.06.004. PubMed PMID: WOS:000382499100002.

357. Bragin D, Bragina O, Statom G, Nemoto E. THERAPEUTIC EVALUATION OF TRANSCRANIAL DIRECT CURRENT STIMULATION IN A MOUSE MODEL OF TRAUMATIC BRAIN INJURY. Journal of Neurotrauma. 2016;33(13):A90-A1. PubMed PMID: WOS:000378336200247.

358. Cioni B, Tufo T, Bentivoglio A, Trevisi G, Piano C. Motor cortex stimulation for movement disorders. Journal of Neurosurgical Sciences. 2016;60(2):230-41. PubMed PMID: WOS:000384947200012.

359. Drougkas D, Konstantinidi M, Kalkanis D, Sakas DE, Datseris IE, Angelakis E. Changes in brain metabolism (18-FDG-PET) after transcranial direct current stimulation (Tdcs) in TBI patients with disorders of consciousness (DOC). Brain Injury. 2016;30(5-6):735-6. PubMed PMID: WOS:000376388200566.

360. Dumel G, Bourassa ME, Desjardins M, Voarino N, Charlebois-Plante C, Doyon J, et al. Multisession Anodal tDCS Protocol Improves Motor System Function in an Aging Population. Neural Plasticity. 2016;2016. doi: 10.1155/2016/5961362. PubMed PMID: WOS:000371058200001.

361. Economou N-T, Papoutsi SN, Ilias I, Konstantinidi M, Sakas D, Dikeos D, et al. Sleep in disorders of consciousness due to traumatic brain injury before and after transcranial direct current stimulation. Brain Injury. 2016;30(5-6):790-. PubMed PMID: WOS:000376388200686.

362. Economou NT, Papoutsi S, Konstantinidi M, Sakas D, Dikeos D, Angelakis E. Sleep structure changes in disorders of consciousness due to traumatic brain injury before and after transcranial direct current stimulation. Journal of Sleep Research. 2016;25:315-. PubMed PMID: WOS:000383445201330.

363. Endisch C, Waterstraat G, Storm C, Ploner CJ, Curio G, Leithner C. Cortical somatosensory evoked high-frequency (600 Hz) oscillations predict absence of severe hypoxic encephalopathy after resuscitation. Clinical Neurophysiology. 2016;127(7):2561-9. doi: 10.1016/j.clinph.2016.04.014. PubMed PMID: WOS:000377988900003.

364. Gallovic MD, Bandyopadhyay S, Borteh H, Montjoy DG, Collier MA, Peine KJ, et al. Microparticles formulated from a family of novel silylated polysaccharides demonstrate inherent immunostimulatory properties and tunable hydrolytic degradability. Journal of Materials Chemistry B. 2016;4(24):4302-12. doi: 10.1039/c6tb00745g. PubMed PMID: WOS:000378936400008.

365. Glannon W. Ethical issues in neuroprosthetics. Journal of Neural Engineering. 2016;13(2). doi: 10.1088/1741-2560/13/2/021002. PubMed PMID: WOS:000375683800003.
366. Glass L, Moody L, Grafman J, Krueger F. Neural signatures of third-party punishment: evidence from penetrating traumatic brain injury. Social Cognitive and Affective Neuroscience. 2016;11(2):253-62. doi: 10.1093/scan/nsv105. PubMed PMID: WOS:000374226900007.
367. Guan C, Guo G, Li Z. Transcranial magnetic stimulation promotes cognition among epileptics after traumatic brain injury. Chinese Journal of Physical Medicine and Rehabilitation. 2016;38(5):349-52. PubMed PMID: CSCD:5798451.
368. Hadanny A, Efrati S. Treatment of persistent post-concussion syndrome due to mild traumatic brain injury: current status and future directions. Expert Review of Neurotherapeutics. 2016;16(8):875-87. doi: 10.1080/14737175.2016.1205487. PubMed PMID: WOS:000381575200004.
369. Henkin RI, Abdelmeguid M, Knoppel AB, Potolicchio SJ. Phantageusia: The Presence of Taste Distortions Inhibited by Repetitive Transcranial Magnetic Stimulation (rTMS). Faseb Journal. 2016;30. PubMed PMID: WOS:000406444000300.

370. Iscan Z, Nazarova M, Fedele T, Blagovechtchenski E, Nikulin VV. Pre-stimulus Alpha Oscillations and Inter-subject Variability of Motor Evoked Potentials in Single- and Paired-Pulse TMS Paradigms. Frontiers in Human Neuroscience. 2016;10. doi: 10.3389/fnhum.2016.00504. PubMed PMID: WOS:000385888100001.
371. Juang Y-J, Deng Y-L, Lee IC. Membrane filtration: An unconventional route for fabrication of the flexible and dissolvable, polymer microneedle patches. Biomicrofluidics. 2016;10(4). doi: 10.1063/1.4960206. PubMed PMID: WOS:000383911400021.
372. Khandan O, Kahook MY, Rao MP. Fenestrated microneedles for ocular drug delivery. Sensors and Actuators B-Chemical. 2016;223:15-23. doi: 10.1016/j.snb.2015.09.071. PubMed PMID: WOS:000366458700003.
373. Kimura H, Kanahara N, Takase M, Yoshida T, Watanabe H, Iyo M. A randomized, sham-controlled study of high frequency rTMS for auditory hallucination in schizophrenia. Psychiatry Research. 2016;241:190-4. doi: 10.1016/j.psychres.2016.04.119. PubMed PMID: WOS:000379370900032.
374. Kotecha R, Berriochoa CA, Murphy ES, Machado AG, Chao ST, Suh JH, et al. Report of whole-brain radiation therapy in a patient with an implanted deep brain stimulator: important

neurosurgical considerations and radiotherapy practice principles. Journal of Neurosurgery. 2016;124(4):966-70. doi: 10.3171/2015.2.Jns142951. PubMed PMID: WOS:000372669100010.

375. Kusamori K, Katsumi H, Sakai R, Hayashi R, Hirai Y, Tanaka Y, et al. Development of a drug-coated microneedle array and its application for transdermal delivery of interferon alpha. Biofabrication. 2016;8(1). doi: 10.1088/1758-5090/8/1/015006. PubMed PMID: WOS:000373289000010.

376. Laureys S. Transcranial direct current stimulation: a promising new avenue. European Journal of Neurology. 2016;23:5-. PubMed PMID: WOS:000431634200012.

377. Lefaucheur J-P. A comprehensive database of published tDCS clinical trials (2005-2016). Neurophysiologie Clinique-Clinical Neurophysiology. 2016;46(6):319-98. doi: 10.1016/j.neucli.2016.10.002. PubMed PMID: WOS:000393255800001.

378. Legostaeva L, Zmeykina E, Kremneva E, Poydasheva A, Chervyakov A, Sergeev D, et al. FMRI-navigated rTMS original protocol for disorders of consciousness treatment. European Journal of Neurology. 2016;23:460-. PubMed PMID: WOS:000431634202217.

379. Legostaeva LA, Zmeykina EA, Poydasheva AG, Sergeev DV, Sinitsyn DO, Kremneva EI, et al. Новый подход к изучению сознания с позиции теории интегрированной информации (обзор)
A New Approach to the Study of Consciousness from the Theory of Integrated Information Point of View (Review). Modern Technologies in Medicine. 2016;8(4):251-9. doi: 10.17691/stm2016.8.4.30. PubMed PMID: RSCI:28290656.
380. Leung A, Fallah A, Shukla S, Lin L, Tsia A, Song D, et al. rTMS in Alleviating Mild TBI Related Headaches - A Case Series. Pain Physician. 2016;19(2):E347-E53. PubMed PMID: WOS:000368991000010.
381. Leung A, Shukla S, Yang E, Canlas B, Kadokana M, Heald J, et al. Diminished supraspinal pain modulation in patients with mild traumatic brain injury. Molecular Pain. 2016;12. doi: 10.1177/1744806916662661. PubMed PMID: WOS:000386444700001.
382. Liu Y, Yang H, Tang X, Bai W, Wang G, Tian X. Repetitive transcranial magnetic stimulation regulates L-type Ca2+ channel activity inhibited by early sevoflurane exposure. Brain Research. 2016;1646:207-18. doi: 10.1016/j.brainres.2016.05.045. PubMed PMID: WOS:000381844700023.

383. Loescher W. Fit for purpose application of currently existing animal models in the discovery of novel epilepsy therapies. Epilepsy Research. 2016;126:157-84. doi: 10.1016/j.eplepsyres.2016.05.016. PubMed PMID: WOS:000383825200022.
384. Manogaran P, Vavasour I, Borich M, Kolind SH, Lange AP, Rauscher A, et al. Corticospinal tract integrity measured using transcranial magnetic stimulation and magnetic resonance imaging in neuromyelitis optica and multiple sclerosis. Multiple Sclerosis Journal. 2016;22(1):43-50. doi: 10.1177/1352458515579441. PubMed PMID: WOS:000367252500010.
385. Morecraft RJ, Ge J, Stilwell-Morecraft KS, McNeal DW, Hynes SM, Pizzimenti MA, et al. Frontal and frontoparietal injury differentially affect the ipsilateral corticospinal projection from the nonlesioned hemisphere in monkey (Macaca mulatta). Journal of Comparative Neurology. 2016;524(2):380-407. doi: 10.1002/cne.23861. PubMed PMID: WOS:000366604700010.
386. Naro A, Leo A, Buda A, Manuli A, Bramanti A, Bramanti P, et al. Do you see me? The role of visual fixation consciousness differential diagnosis. Brain Research. 2016;1653:59-66. doi: 10.1016/j.brainres.2016.10.015. PubMed PMID: WOS:000389730100007.

387. Plenger P, Krishnan K, Cloud M, Bosworth C, Qualls D, de la Plata CM. fNIRS-based investigation of the Stroop task after TBI. Brain Imaging and Behavior. 2016;10(2):357-66. doi: 10.1007/s11682-015-9401-9. PubMed PMID: WOS:000382390000004.

388. Poole VN, Robinson ME, Singleton O, DeGutis J, Milberg WP, McGlinchey RE, et al. Intrinsic functional connectivity predicts individual differences in distractibility. Neuropsychologia. 2016;86:176-82. doi: 10.1016/j.neuropsychologia.2016.04.023. PubMed PMID: WOS:000378453400018.

389. Romero G, Christiansen MG, Barbosa LS, Garcia F, Anikeeva P. Localized Excitation of Neural Activity via Rapid Magnetothermal Drug Release. Advanced Functional Materials. 2016;26(35):6471-8. doi: 10.1002/adfm.201602189. PubMed PMID: WOS:000384809100013.

390. Rudisch J, Butler J, Izadi H, Zielinski IM, Aarts P, Birtles D, et al. Kinematic parameters of hand movement during a disparate bimanual movement task in children with unilateral Cerebral Palsy. Human Movement Science. 2016;46:239-50. doi: 10.1016/j.humov.2016.01.010. PubMed PMID: WOS:000370098600023.

391. Sahoo S, Rout CS. Facile Electrochemical Synthesis of Porous

Manganese-Cobalt-Sulfide Based Ternary Transition Metal Sulfide Nanosheets Architectures for High Performance Energy Storage Applications. Electrochimica Acta. 2016;220:57-66. doi: 10.1016/j.electacta.2016.10.043. PubMed PMID: WOS:000389090800007.

392. Schorr B, Schlee W, Arndt M, Bender A. Coherence in resting-state EEG as a predictor for the recovery from unresponsive wakefulness syndrome. Journal of Neurology. 2016;263(5):937-53. doi: 10.1007/s00415-016-8084-5. PubMed PMID: WOS:000376141000012.

393. Shen Q, Fang Y, Wu J, Li N, You P, Liu P, et al. Effect of hyperbaric oxygen combined with repetitive transcranial magnetic stimulation in the treatment of coma following craniocerebral trauma. Chinese Journal of Nautical Medicine and Hyperbaric Medicine. 2016;23(1):9-12. PubMed PMID: CSCD:5669180.

394. Vaessen MJ, Saj A, Loublad K-O, Gschwind M, Vuilleumier P. Structural white-matter connections mediating distinct behavioral components of spatial neglect in right brain-damaged patients. Cortex. 2016;77:54-68. doi: 10.1016/j.cortex.2015.12.008. PubMed PMID: WOS:000374081600005.

395. Ventura RE, Balcer LJ, Galetta SL, Rucker JC. Ocular motor assessment in concussion: Current status and future directions.

Journal of the Neurological Sciences. 2016;361:79-86. doi: 10.1016/j.jns.2015.12.010. PubMed PMID: WOS:000370093400016.

396. Wagner D, Eslinger PJ, Barrett AM. Decreased Leftward 'Aiming' Motor-Intentional Spatial Cuing in Traumatic Brain Injury. Neuropsychology. 2016;30(6):731-41. doi: 10.1037/neu0000252. PubMed PMID: WOS:000383879300008.

397. Wang QL, Zhu DD, Chen Y, Guo XD. A fabrication method of microneedle molds with controlled microstructures. Materials Science & Engineering C-Materials for Biological Applications. 2016;65:135-42. doi: 10.1016/j.msec.2016.03.097. PubMed PMID: WOS:000376833200016.

398. Willemse RB, Hillebrand A, Ronner HE, Vandertop WP, Stam CJ. Magnetoencephalographic study of hand and foot sensorimotor organization in 325 consecutive patients evaluated for tumor or epilepsy surgery. Neuroimage-Clinical. 2016;10:46-53. doi: 10.1016/j.nicl.2015.11.002. PubMed PMID: WOS:000373189000005.

399. Woods DL, Wyma JM, Herron TJ, Yund EW. The Effects of Repeat Testing, Malingering, and Traumatic Brain Injury on Computerized Measures of Visuospatial Memory Span. Frontiers in Human Neuroscience. 2016;9. doi: 10.3389/fnhum.2015.00690.

PubMed PMID: WOS:000367645700001.

400. Xia X, Yang Y, Bai Y, He J. Transcranial magnetic stimulation combined with high -density EEG in assessment of patients with disorders of consciousness. Chinese Journal of Neuromedicine. 2016;15(4):425-8. PubMed PMID: CSCD:5689961.

401. Yang C, Wang L, Feng Z. Arousal effects of median nerve electrical stimulation on coma patients after traumatic brain injury. Chinese Journal of Rehabilitation Medicine. 2016;31(11):1195-9,207. PubMed PMID: CSCD:5850560.

402. You W, Tang Q, Wang L, Lei J, Feng J, Mao Q, et al. Effect of right median nerve electric stimulation on brain electrical activity of traumatic coma patients. Chinese Journal of Trauma. 2016;32(2):120-3. PubMed PMID: CSCD:5626226.

403. Zhu DD, Wang QL, Liu XB, Guo XD. Rapidly separating microneedles for transdermal drug delivery. Acta Biomaterialia. 2016;41:312-9. doi: 10.1016/j.actbio.2016.06.005. PubMed PMID: WOS:000381950500029.

404. Zhu J, Shen Q, Cao Y, Chen X, Zhao X. The fabrication and property of a novel coated out-of-plane microneedle arrays. Microsystem Technologies-Micro-and Nanosystems-Information Storage and Processing Systems. 2016;22(1):143-9. doi: 10.1007/s00542-015-2448-0. PubMed PMID:

WOS:000367812600020.
405. Zou J, Wang M, Uchiumi O, Shui Y, Ishigaki Y, Liu X, et al. Learning impairment by minimal cortical injury in a mouse model of Alzheimer's disease. Brain Research. 2016;1637:56-63. doi: 10.1016/j.brainres.2016.02.017. PubMed PMID: WOS:000374610700005.
406. An M, Liu H. Dissolving Microneedle Arrays for Transdermal Delivery of Amphiphilic Vaccines. Small. 2017;13(26). doi: 10.1002/smll.201700164. PubMed PMID: WOS:000404986200005.
407. Bai Y, Xia X, Li X. A Review of Resting-State Electroencephalography Analysis in Disorders of Consciousness. Frontiers in Neurology. 2017;8. doi: 10.3389/fneur.2017.00471. PubMed PMID: WOS:000410076900002.
408. Bernier RA, Roy A, Venkatesan UM, Grossner EC, Brenner EK, Hillary FG. Dedifferentiation Does not account for hyperconnectivity after Traumatic Brain injury. Frontiers in Neurology. 2017;8. doi: 10.3389/fneur.2017.00297. PubMed PMID: WOS:000406481700001.
409. Boly M, Massimini M, Tsuchiya N, Postle BR, Koch C, Tononi G. Are the Neural Correlates of Consciousness in the Front or in the Back of the Cerebral Cortex? Clinical and Neuroimaging

Evidence. Journal of Neuroscience. 2017;37(40):9603-13. doi: 10.1523/jneurosci.3218-16.2017. PubMed PMID: WOS:000412182900002.

410. Bragina O, Lara D, Yang Y, Shuttleworth C, Nemoto E, Bragin D. REPETITIVE ANODAL TRANSCRANIAL DIRECT CURRENT STIMULATION IMPROVES OUTCOME AT THREE WEEKS BUT NOT ONE WEEK AFTER TBI IN MICE. Journal of Neurotrauma. 2017;34(13):A71-A2. PubMed PMID: WOS:000404530400193.

411. Bragina OA, Yang Y, Shuttleworth CW, Nemoto EM, Semyachkina-Glushkovskaya OV, Bragin DE. Repetitive anodal transcranial direct current stimulation (tDCS) improves neurologic outcome in mice after traumatic brain injury (TBI) by increasing cerebral blood flow and tissue oxygenation. Journal of Cerebral Blood Flow and Metabolism. 2017;37:408-. PubMed PMID: WOS:000400157400588.

412. Buch ER, Liew S-L, Cohen LG. Plasticity of Sensorimotor Networks: Multiple Overlapping Mechanisms. Neuroscientist. 2017;23(2):185-96. doi: 10.1177/1073858416638641. PubMed PMID: WOS:000398048800011.

413. Cakmak YO, Ekinci G, Heinecke A, Cavdar S. A Possible Role of Prolonged Whirling Episodes on Structural Plasticity of the

Cortical Networks and Altered Vertigo Perception: The Cortex of Sufi Whirling Dervishes. Frontiers in Human Neuroscience. 2017;11. doi: 10.3389/fnhum.2017.00003. PubMed PMID: WOS:000392444300001.

414. Cha M, Um SW, Kwon M, Nam TS, Lee BH. Repetitive motor cortex stimulation reinforces the pain modulation circuits of peripheral neuropathic pain. Scientific Reports. 2017;7. doi: 10.1038/s41598-017-08208-2. PubMed PMID: WOS:000407443300034.

415. Chantigian D, Rich T, Chen C-Y, Lixandrao M, Gillick B, Keller-Ross M. The Effect of Transcranial Magnetic Stimulation on the Autonomic Nervous System in Pediatric Stroke. Faseb Journal. 2017;31. PubMed PMID: WOS:000405986502579.

416. Chen M-C, Chan H-A, Ling M-H, Su L-C. Implantable polymeric microneedles with phototriggerable properties as a patient-controlled transdermal analgesia system. Journal of Materials Chemistry B. 2017;5(3):496-503. doi: 10.1039/c6tb02718k. PubMed PMID: WOS:000394431600012.

417. de Oliveira FF, Correia Marin SdM, Ferreira Bertolucci PH. Neurological impressions on the organization of language networks in the human brain. Brain Injury. 2017;31(2):140-50. doi: 10.1080/02699052.2016.1199914. PubMed PMID:

WOS:000395163500002.
418. Dong X, Wei T, Papa EV, Feng Z. Median nerve electrical stimulation causes wake-promotion by downregulating the expression of gamma-aminobutyric acid b receptor via orexins pathway in comatose rats caused by traumatic brain injury. International Journal of Clinical and Experimental Medicine. 2017;10(10):14571-8. PubMed PMID: WOS:000414299700046.
419. Du Q, Feng Z. The effects of electrical stimulation on the expression of H1 receptor in the prefrontal cortex of brain injured, comatose rats. Chinese Journal of Physical Medicine and Rehabilitation. 2017;39(2):81-5. PubMed PMID: CSCD:5932756.
420. Eapen BC, Murphy DP, Cifu DX. Neuroprosthetics in amputee and brain injury rehabilitation. Experimental Neurology. 2017;287:479-85. doi: 10.1016/j.expneurol.2016.08.004. PubMed PMID: WOS:000391158800006.
421. Gould TD, Georgiou P, Brenner LA, Brundin L, Can A, Courtet P, et al. Animal models to improve our understanding and treatment of suicidal behavior. Translational Psychiatry. 2017;7. doi: 10.1038/tp.2017.50. PubMed PMID: WOS:000402329700007.
422. Guo J, Garrido L, Liu RR, Susilo T, Barton JJS, Duchaine B. Normal voice processing after posterior superior temporal sulcus lesion. Neuropsychologia. 2017;105:215-22. doi:

10.1016/j.neuropsychologia.2017.03.008. PubMed PMID: WOS:000416185000019.

423. Henderson TA, Morries LD. Multi-Watt Near-Infrared Phototherapy for the Treatment of Comorbid Depression: An Open-Label Single-Arm Study. Frontiers in Psychiatry. 2017;8. doi: 10.3389/fpsyt.2017.00187. PubMed PMID: WOS:000411935500001.

424. Huang C-J, Huang Y-H, Lin C-H. Factors Related to the Changes in Quality of Life for Patients With Depression After an Acute Course of Electroconvulsive Therapy. Journal of Ect. 2017;33(2):126-33. doi: 10.1097/yct.0000000000000358. PubMed PMID: WOS:000401798400019.

425. Jackson MP, Bikson M, Liebetanz D, Nitsche M. Toward comprehensive tDCS safety standards. Brain Behavior and Immunity. 2017;66:413-. doi: 10.1016/j.bbi.2017.08.007. PubMed PMID: WOS:000414236600038.

426. Jung D, Rejinold NS, Kwak J-E, Park S-H, Kim Y-C. Nano-patterning of a stainless steel microneedle surface to improve the dip-coating efficiency of a DNA vaccine and its immune response. Colloids and Surfaces B-Biointerfaces. 2017;159:54-61. doi: 10.1016/j.colsurfb.2017.07.059. PubMed PMID: WOS:000418729600008.

427. Kendall DA, Yudowski GA. Cannabinoid Receptors in the Central Nervous System: Their Signaling and Roles in Disease. Frontiers in Cellular Neuroscience. 2017;10. doi: 10.3389/fncel.2016.00294. PubMed PMID: WOS:000391218600001.

428. Khan S, Fusco H. Review of Non-invasive Brain Stimulation and Stem Cells in the Treatment of Traumatic Brain Injury. Journal of Head Trauma Rehabilitation. 2017;32(6):E80-E. PubMed PMID: WOS:000418588100041.

429. Kimberley TJ, Novak I, Boyd L, Fowler E, Larsen D. Stepping Up to Rethink the Future of Rehabilitation: IV STEP Considerations and Inspirations. Journal of Neurologic Physical Therapy. 2017;41(3):S63-S72. doi: 10.1097/npt.0000000000000182. PubMed PMID: WOS:000404956200010.

430. Lascano AM, Lalive PH, Hardmeier M, Fuhr P, Seeck M. Clinical evoked potentials in neurology: a review of techniques and indications. Journal of Neurology Neurosurgery and Psychiatry. 2017;88(8):688-96. doi: 10.1136/jnnp-2016-314791. PubMed PMID: WOS:000405379100099.

431. Lee IC, Lin W-M, Shu J-C, Tsai S-W, Chen C-H, Tsai M-T. Formulation of two-layer dissolving polymeric microneedle

patches for insulin transdermal delivery in diabetic mice. Journal of Biomedical Materials Research Part A. 2017;105(1):84-93. doi: 10.1002/jbm.a.35869. PubMed PMID: WOS:000389145400009.

432. Lefaucheur J-P, Antal A, Ayache SS, Benninger DH, Brunelin J, Cogiamanian F, et al. Evidence-based guidelines on the therapeutic use of transcranial direct current stimulation (tDCS). Clinical Neurophysiology. 2017;128(1):56-92. doi: 10.1016/j.clinph.2016.10.087. PubMed PMID: WOS:000396377300008.

433. Li L, Violante I, Zimmerman K, Ross E, Leech R, Hampshire A, et al. EXPLORING THE TARGETED APPLICATION OF TDCS AFTER TBI. Journal of Neurology Neurosurgery and Psychiatry. 2017;88:A82-A. doi: 10.1136/jnnp-2017-ABN.286. PubMed PMID: WOS:000471110600287.

434. Li LM, Violante I, Zimmerman K, Ross E, Leech R, Hampshire A, et al. Promoting network recovery after traumatic brain injury: exploring the targeted application of transcranial direct current stimulation. Neurology. 2017;88. PubMed PMID: WOS:000577381500017.

435. Liao L, Teng X, Gao L, Gu L, Zhou B. Effect of Repetitive Transcranial Magnetic Stimulation Combined with Eye-acupuncture on Cognitive Dysfunction after Traumatic Brain

Injury. Chinese Journal of Rehabilitation Theory and Practice. 2017;23(1):92-6. PubMed PMID: CSCD:5922145.

436. Lin C-L, Dumont AS, Zhang JH, Zuccarello M, Chen C-S. Improving and Predicting Outcomes of Traumatic Brain Injury: Neuroplasticity, Imaging Modalities, and Perspective Therapy. Neural Plasticity. 2017;2017. doi: 10.1155/2017/4752546. PubMed PMID: WOS:000404928700001.

437. Lynall RC, Pietrosimone B, Kerr ZY, Mauntel TC, Mihalik JP, Guskiewicz KM. Osteoarthritis Prevalence in Retired National Football League Players With a History of Concussion and Lower Extremity Injury. Journal of Athletic Training. 2017;52(6):518-25. doi: 10.4085/1062-6050-52.2.03. PubMed PMID: WOS:000404154700005.

438. Mandal S, Khandalavala K, Pham R, Bruck P, Varghese M, Kochvar A, et al. Cellulose Acetate Phthalate and Antiretroviral Nanoparticle Fabrications for HIV Pre-Exposure Prophylaxis. Polymers. 2017;9(9). doi: 10.3390/polym9090423. PubMed PMID: WOS:000411524400037.

439. Mochalova E, Legostaeva L, Poydasheva A, Kremneva E, Chervyakov A, Sergeev D, et al. Decreased level of consciousness in thalamic hemorrhage patient. European Journal of Neurology. 2017;24:539-40. PubMed PMID: WOS:000405530101161.

440. O'Shea J, Revol P, Cousijn H, Near J, Petitet P, Jacquin-Courtois S, et al. Induced sensorimotor cortex plasticity remediates chronic treatment-resistant visual neglect. Elife. 2017;6. doi: 10.7554/eLife.26602. PubMed PMID: WOS:000410296800001.

441. Pape T, Rosenow J, Blabas B, Kletzel S, Herrold A, Parrish T, et al. Combining rTMS and amantadine to enable neurobehavioural recovery for persons living in chronic states of disordered consciousness after TBI. Brain Injury. 2017;31(6-7):994-5. PubMed PMID: WOS:000406734000550.

442. Pruitt DT, Danaphongse TT, Schmid AN, Morrison RA, Kilgard MP, Rennaker RL, II, et al. Traumatic Brain Injury Occludes Training-Dependent Cortical Reorganization in the Contralesional Hemisphere. Journal of Neurotrauma. 2017;34(17):2495-503. doi: 10.1089/neu.2016.4796. PubMed PMID: WOS:000408310200002.

443. Quinn D. TRANSCRANIAL DIRECT CURRENT STIMULATION FACILITATES WORKING MEMORY AFTER MILD-MODERATE TRAUMATIC BRAIN INJURY. Journal of Neurotrauma. 2017;34(13):A143-A. PubMed PMID: WOS:000404530400392.

444. Rizzo J-R, Hosseini M, Wong EA, Mackey WE, Fung JK,

Ahdoot E, et al. The Intersection between Ocular and Manual Motor Control: Eye-Hand Coordination in Acquired Brain Injury. Frontiers in Neurology. 2017;8. doi: 10.3389/fneur.2017.00227. PubMed PMID: WOS:000403264500001.

445. Scangos K, Boudewyn M, Roberts B, Ragland J, Ranganath C, Carter C. Effects of tDCS on Cognitive Control and Cortical Network Oscillations in Schizophrenia. Neuropsychopharmacology. 2017;42:S230-S. PubMed PMID: WOS:000416846301186.

446. Sebastianelli L, Saltuari L, Nardone R. How the brain can rewire itself after an injury: the lesson from hemispherectomy. Neural Regeneration Research. 2017;12(9):1426-7. doi: 10.4103/1673-5374.215247. PubMed PMID: WOS:000412978200008.

447. Simons A, Hamdy S. The Use of Brain Stimulation in Dysphagia Management. Dysphagia. 2017;32(2):209-15. doi: 10.1007/s00455-017-9789-z. PubMed PMID: WOS:000399169300001.

448. Song W, Martin JH. Spinal cord direct current stimulation differentially modulates neuronal activity in the dorsal and ventral spinal cord. Journal of Neurophysiology. 2017;117(3):1143-55. doi: 10.1152/jn.00584.2016. PubMed PMID: WOS:000398162500023.

449. Streijger F, Skinnider MA, Rogalski JC, Balshaw R, Shannon

CP, Prudova A, et al. A Targeted Proteomics Analysis of Cerebrospinal Fluid after Acute Human Spinal Cord Injury. Journal of Neurotrauma. 2017;34(12):2054-68. doi: 10.1089/neu.2016.4879. PubMed PMID: WOS:000402880600006.

450. Strisland F, Vedum J, Liverud A, Dalgard S, Brodreskift T, Albert B, et al. Portable qEEG and HD-tCS Device for Point-of-Injury Traumatic Brain Injury Diagnostics. Studies in health technology and informatics. 2017;237:198-203. PubMed PMID: MEDLINE:28479568.

451. Su L-C, Chen M-C. Efficient delivery of nanoparticles to deep skin layers using dissolvable microneedles with an extended-length design. Journal of Materials Chemistry B. 2017;5(18):3355-63. doi: 10.1039/c7tb00451f. PubMed PMID: WOS:000400988200011.

452. Suponeva NA, Bakulin IS, Poydasheva AG, Piradov MA. Safety of transcranial magnetic stimulation: review of international guidelines and new findings. Neuromuscular Diseases. 2017;7(2):21-36. doi: 10.17650/2222-8721-2017-7-2-21-36. PubMed PMID: RSCI:30013882.

453. Surmeli T, Eralp E, Mustafazade I, Kos IH, Ozer GE, Surmeli OH. Quantitative EEG Neurometric Analysis-Guided Neurofeedback Treatment in Postconcussion Syndrome (PCS): Forty Cases. How Is Neurometric Analysis Important for the

Treatment of PCS and as a Biomarker? Clinical Eeg and Neuroscience. 2017;48(3):217-30. doi: 10.1177/1550059416654849. PubMed PMID: WOS:000403282400007.
454. Takemi M, Castagnola E, Ansaldo A, Ricci D, Fadiga L, Taoka M, et al. Rapid Identification of Cortical Motor Areas in Rodents by High-Frequency Automatic Cortical Stimulation and Novel Motor Threshold Algorithm. Frontiers in Neuroscience. 2017;11. doi: 10.3389/fnins.2017.00580. PubMed PMID: WOS:000413158000001.
455. Than A, Liang K, Xu S, Sun L, Duan H, Xi F, et al. Transdermal Delivery of Anti-Obesity Compounds to Subcutaneous Adipose Tissue with Polymeric Microneedle Patches. Small Methods. 2017;1(11). doi: 10.1002/smtd.201700269. PubMed PMID: WOS:000417488900010.
456. Tobimatsu S. Neurophysiology CNS. Journal of the Neurological Sciences. 2017;381:26-. doi: 10.1016/j.jns.2017.08.115. PubMed PMID: WOS:000427450300091.
457. Wang D, Mai R, Wang M, Tan T, Zhu F, Chen S, et al. Effect of Low Frequency Repetitive Transcranial Magnetic Stimulation at Uninjured Side on Memory and Quality of Life in Patients with

Traumatic Brain Injury. Chinese Journal of Rehabilitation Theory and Practice. 2017;23(10):1180-4. PubMed PMID: CSCD:6107379.
458. Wang J, Lim Y-F, Ho GW. Manganese Copper Sulfide Nanocomposites: Structure Tailoring and Photo/Electrocatalytic Hydrogen Generation. Chemcatchem. 2017;9(21):4148-54. doi: 10.1002/cctc.201700865. PubMed PMID: WOS:000417441400019.
459. Wertheimer T, Velardi E, Tsai J, Cooper K, Ottmueller KJ, Mokthari Z, et al. Mechanisms Governing Endogenous Thymic Regeneration. Blood. 2017;130. PubMed PMID: WOS:000432419400121.
460. Xia X, Bai Y, Zhou Y, Yang Y, Xu R, Gao X, et al. Effect of 10 Hz Repetitive Transcranial Magnetic Stimulation of the Left Dorsolateral Prefrontal Cortex in Disorders of Consciousness. Frontiers in Neurology. 2017;8. doi: 10.3389/fneur.2017.00182. PubMed PMID: WOS:000400511900001.
461. Yan Y, Fu J, Wang T, Lu X. Controlled release of silyl ether camptothecin from thiol-ene click chemistry-functionalized mesoporous silica nanoparticles. Acta Biomaterialia. 2017;51:471-8. doi: 10.1016/j.actbio.2017.01.062. PubMed PMID: WOS:000398014000037.

462. Yang H, Jiang K, Fang D. Effect of Median Nerve Electrical Stimulation on Hemodynamics in Severe Brain Injury Patients with Coma. Acta Medicinae Universitatis Scientiae et Technologiae Huazhong. 2017;46(4):462-5. PubMed PMID: CSCD:6060883.
463. Yu W, Jiang G, Liu D, Li L, Chen H, Liu Y, et al. Fabrication of biodegradable composite microneedles based on calcium sulfate and gelatin for transdermal delivery of insulin. Materials Science & Engineering C-Materials for Biological Applications. 2017;71:725-34. doi: 10.1016/j.msec.2016.10.063. PubMed PMID: WOS:000390967200086.
464. Yu W, Jiang G, Liu D, Li L, Tong Z, Yao J, et al. Transdermal delivery of insulin with bioceramic composite microneedles fabricated by gelatin and hydroxyapatite. Materials Science & Engineering C-Materials for Biological Applications. 2017;73:425-8. doi: 10.1016/j.msec.2016.12.111. PubMed PMID: WOS:000394064800050.
465. Yu W, Jiang G, Zhang Y, Liu D, Xu B, Zhou J. Polymer microneedles fabricated from alginate and hyaluronate for transdermal delivery of insulin. Materials Science & Engineering C-Materials for Biological Applications. 2017;80:187-96. doi: 10.1016/j.msec.2017.05.143. PubMed PMID: WOS:000410254400025.

466. Yu Y, Rong P, Zhu B. Present Situation and Prospect of Transcutaneous Auricular Vagus Nerve Stimulation in Encephalopathy Treatment. Modernization of Traditional Chinese Medicine and Materia Medica--World Science and Technology. 2017;19(3):462-8. PubMed PMID: CSCD:6013096.
467. Zhang P, Cao Y, Chen H, Zhou B, Hu W, Zhang L. Preparation and evaluation of glycyrrhetinic acid-modified and honokiol-loaded acoustic nanodroplets for targeted tumor imaging and therapy with low-boiling-point phase-change perfluorocarbon. Journal of Materials Chemistry B. 2017;5(29):5845-53. doi: 10.1039/c7tb01215b. PubMed PMID: WOS:000406616400015.
468. Zhu DD, Chen BZ, He MC, Guo XD. Structural optimization of rapidly separating microneedles for efficient drug delivery. Journal of Industrial and Engineering Chemistry. 2017;51:178-84. doi: 10.1016/j.jiec.2017.02.030. PubMed PMID: WOS:000401208400019.
469. Alawieh A, Zhao J, Feng W. Factors affecting post-stroke motor recovery: Implications on neurotherapy after brain injury. Behavioural Brain Research. 2018;340:94-101. doi: 10.1016/j.bbr.2016.08.029. PubMed PMID: WOS:000424173400011.
470. Alsharidah M, Al-Hussain F, Iqbal M, Hamza A, Yoo WK,

Bashir S. The effect of transcranial direct current stimulation combined with functional task training on motor recovery in stroke patients. European Review for Medical and Pharmacological Sciences. 2018;22(21):7385-92. PubMed PMID: WOS:000451033900035.

471. Andersen TE, Andersen AJ, Petersen RS, Nielsen LH, Keller SS. Drug loaded biodegradable polymer microneedles fabricated by hot embossing. Microelectronic Engineering. 2018;195:57-61. doi: 10.1016/j.mee.2018.03.024. PubMed PMID: WOS:000433641200010.

472. Bianchi F, Malboubi M, Li Y, George JH, Jerusalem A, Szele F, et al. Rapid and efficient differentiation of functional motor neurons from human iPSC for neural injury modelling. Stem Cell Research. 2018;32:126-34. doi: 10.1016/j.scr.2018.09.006. PubMed PMID: WOS:000447301200019.

473. Bickart K, Main K, Milazzo A-C, Newsom M, Sours C, Ashford JW, et al. Large-Scale Frontotemporal Limbic Networks in TBI: Vulnerability and Response to Non-Invasive Brain Stimulation. Neurology. 2018;90. PubMed PMID: WOS:000453090806024.

474. Blandiaux S, Raimondo F, Wolff A, Sanz L, Bodart O, Barra A, et al. PCI & Auditory ERPs for the diagnosis of disorders of

consciousness: an EEG-based methods comparison study. European Journal of Neurology. 2018;25:399-. PubMed PMID: WOS:000435272002205.

475. Bui Quoc B, Ngoc Hoang L, Diem Huong Tran N, Tuong Vi T, Linh Phuong Tran P, Long Giang B, et al. Evolution and present scenario of multifunctionalized mesoporous nanosilica platform: A mini review. Materials Science & Engineering C-Materials for Biological Applications. 2018;91:912-28. doi: 10.1016/j.msec.2018.07.008. PubMed PMID: WOS:000442192000092.

476. Chen BZ, Ashfaq M, Zhu DD, Zhang XP, Guo XD. Controlled Delivery of Insulin Using Rapidly Separating Microneedles Fabricated from Genipin-Crosslinked Gelatin. Macromolecular Rapid Communications. 2018;39(20). doi: 10.1002/marc.201800075. PubMed PMID: WOS:000447653600005.

477. Chen C-H, Shyu VB-H, Chen C-T. Dissolving Microneedle Patches for Transdermal Insulin Delivery in Diabetic Mice: Potential for Clinical Applications. Materials. 2018;11(9). doi: 10.3390/ma11091625. PubMed PMID: WOS:000446395200141.

478. Chen M-C, Lai K-Y, Ling M-H, Lin C-W. Enhancing immunogenicity of antigens through sustained intradermal delivery

using chitosan microneedles with a patch-dissolvable design. Acta Biomaterialia. 2018;65:66-75. doi: 10.1016/j.actbio.2017.11.004. PubMed PMID: WOS:000418315900006.

479. Chi L, Li H, Zhao L, Pang Y. The clinical curative effect of transcranial direct current stimulation on patients with disorders of consciousness. Chinese Journal of Physical Medicine and Rehabilitation. 2018;40(9):652-6. PubMed PMID: CSCD:6459795.

480. D'Andola M, Rebollo B, Casali AG, Weinert JF, Pigorini A, Villa R, et al. Bistability, Causality, and Complexity in Cortical Networks: An In Vitro Perturbational Study. Cerebral Cortex. 2018;28(7):2233-42. doi: 10.1093/cercor/bhx122. PubMed PMID: WOS:000437165800002.

481. Dogra P, Adolphi NL, Wang Z, Lin Y-S, Butler KS, Durfee PN, et al. Establishing the effects of mesoporous silica nanoparticle properties on in vivo disposition using imaging-based pharmacokinetics. Nature Communications. 2018;9. doi: 10.1038/s41467-018-06730-z. PubMed PMID: WOS:000448807000022.

482. Fox MD. Mapping Symptoms to Brain Networks with the Human Connectome. New England Journal of Medicine. 2018;379(23):2237-45. doi: 10.1056/NEJMra1706158. PubMed PMID: WOS:000452259200009.

483. Fujioka H, Urasaki E, Izumihara A, Yamashita K. Epidural motor cortex stimulation for intractable leg pain. Clinical Neurophysiology. 2018;129(3):636-7. doi: 10.1016/j.clinph.2018.01.007. PubMed PMID: WOS:000425876800014.
484. Guzman Garcia L, Santamarina E, Toledo M, Raguer N, Sueiras M, Abraira del Fresno L, et al. Low Frequency Repetitive Transcranial Magnetic Stimulation for Refractory Status Epilepticus and Multifocal Highly Drug-Resistant Epilepsy. Epilepsia. 2018;59:S166-S. PubMed PMID: WOS:000451817900346.
485. Guzman-Mejia F, Lopez-Rubalcava C, Gonzalez-Espinosa C. Stimulation of nAchR alpha 7 Receptor Inhibits TNF Synthesis and Secretion in Response to LPS Treatment of Mast Cells by Targeting ERK1/2 and TACE Activation. Journal of Neuroimmune Pharmacology. 2018;13(1):39-52. doi: 10.1007/s11481-017-9760-7. PubMed PMID: WOS:000423591600004.
486. He F, Wu M, Meng F, Hu Y, Gao J, Chen Z, et al. Effects of 20Hz Repetitive Transcranial Magnetic Stimulation on Disorders of Consciousness: A Resting-State Electroencephalography Study. Neural Plasticity. 2018;2018. doi: 10.1155/2018/5036184. PubMed PMID: WOS:000429681300001.

487. Henssen DJHA, Kurt E, van Walsum A-MvC, Arnts I, Doorduin J, Kozicz T, et al. Long-term effect of motor cortex stimulation in patients suffering from chronic neuropathic pain: An observational study. Plos One. 2018;13(1). doi: 10.1371/journal.pone.0191774. PubMed PMID: WOS:000423617800034.

488. Hu L, Liao Z, Hu Q, Maffucci KG, Qu Y. Novel Bletilla striata polysaccharide microneedles: Fabrication, characterization, and in vitro transcutaneous drug delivery. International Journal of Biological Macromolecules. 2018;117:928-36. doi: 10.1016/j.ijbiomac.2018.05.097. PubMed PMID: WOS:000442057700107.

489. Iovino L, Cooper K, Kinsella S, DeRoos P, Jain R, Galimberti S, et al. Zinc Supplementation Improves T Cell Reconstitution after Allogeneic HSCT By Stimulating Endogenous Pathways of Thymic Regeneration. Blood. 2018;132. doi: 10.1182/blood-2018-99-112707. PubMed PMID: WOS:000454842801110.

490. Izrael M, Slutsky SG, Admoni T, Cohen L, Granit A, Hasson A, et al. Safety and efficacy of human embryonic stem cell-derived astrocytes following intrathecal transplantation in SOD1(G93A) and NSG animal models. Stem Cell Research & Therapy. 2018;9.

doi: 10.1186/s13287-018-0890-5. PubMed PMID: WOS:000434911700001.

491. Jang SH, Seo YS. Effect of Neuromuscular Electrical Stimulation Training on the Finger Extensor Muscles for the Contralateral Corticospinal Tract in Normal Subjects: A Diffusion Tensor Tractography Study. Frontiers in Human Neuroscience. 2018;12. doi: 10.3389/fnhum.2018.00432. PubMed PMID: WOS:000450705800001.

492. Jen E, Lin C-W, Hsieh T-H, Chiu Y-C, Lu T-C, Chen S-C, et al. Prototype Deep Brain Stimulation System with Closed-Loop Control Feedback for Modulating Bladder Functions in Traumatic Brain Injured Animals. Journal of Medical and Biological Engineering. 2018;38(3):337-49. doi: 10.1007/s40846-017-0326-1. PubMed PMID: WOS:000432631500001.

493. Jiang T. Recent Progress in Basic and Clinical Research on Disorders of Consciousness. Neuroscience Bulletin. 2018;34(4):589-91. doi: 10.1007/s12264-018-0264-0. PubMed PMID: WOS:000440164500001.

494. Karim R, Lepeltier E, Esnault L, Pigeon P, Lemaire L, Lepinoux-Chambaud C, et al. Enhanced and preferential internalization of lipid nanocapsules into human glioblastoma cells: effect of a surface-functionalizing NFL peptide. Nanoscale.

2018;10(28):13485-501. doi: 10.1039/c8nr02132e. PubMed PMID: WOS:000439319000022.

495. Kelchen MN, Brogden NK. Effect of dosing regimen and microneedle pretreatment on in vitro skin retention of topically applied beta-blockers. Biomedical Microdevices. 2018;20(4). doi: 10.1007/s10544-018-0348-7. PubMed PMID: WOS:000452462600002.

496. Kundu A, Ausaf T, Rajaraman S. 3D Printing, Ink Casting and Micromachined Lamination (3D PICLM): A Makerspace Approach to the Fabrication of Biological Microdevices. Micromachines. 2018;9(2). doi: 10.3390/mi9020085. PubMed PMID: WOS:000427530300045.

497. Kureshi N, Clarke DB. Neurotrauma Research at Dalhousie University: A Snapshot of Past and Present Developments. Journal of Neurotrauma. 2018;35(16):1993-4. PubMed PMID: WOS:000441527400762.

498. Latchoumane C-FV, Jackson L, Sendi MSE, Tehrani KF, Mortensen LJ, Stice SL, et al. Chronic Electrical Stimulation Promotes the Excitability and Plasticity of ESC-derived Neurons following Glutamate-induced Inhibition In vitro. Scientific Reports. 2018;8. doi: 10.1038/s41598-018-29069-3. PubMed PMID: WOS:000439102400013.

499. Lee G, Ma Y, Lee Y-h, Jung H. Clinical Evaluation of a Low-pain Long Microneedle for Subcutaneous Insulin Injection. Biochip Journal. 2018;12(4):309-16. doi: 10.1007/s13206-018-2411-0. PubMed PMID: WOS:000453682800007.

500. Lee HN, Oh Y-B, Kim G-W, Hui WY, Hwan KM, Hwan SJ, et al. Localization of Bilateral Hemisphere Lesion Using Combined Transcranial Magnetic Stimulation and Diffusion Tensor Imaging: Report of Two Cases. Journal of Electrodiagnosis and Neuromuscular Diseases. 2018;20(2):106-11. doi: 10.18214/jkaem.2018.20.2.106. PubMed PMID: KJD:ART002425419.

501. Lefebvre G, Chamard E, Proulx S, Tremblay S, Halko M, Soman S, et al. Increased Myo-Inositol in Primary Motor Cortex of Contact Sports Athletes without a History of Concussion. Journal of Neurotrauma. 2018;35(7):953-62. doi: 10.1089/neu.2017.5254. PubMed PMID: WOS:000424833600001.

502. Liao C, Sun W, Feng Z. Effects of transcranial direct current stimulation on consciousness and expression of brain-derived neurotrophic factor in coma rats following traumatic brain injury. Chinese Journal of Rehabilitation Medicine. 2018;33(3):269-73,85. PubMed PMID: CSCD:6203324.

503. Liu D, Yu B, Jiang G, Yu W, Zhang Y, Xu B. Fabrication of composite microneedles integrated with insulin-loaded CaCO3 microparticles and PVP for transdermal delivery in diabetic rats. Materials Science & Engineering C-Materials for Biological Applications. 2018;90:180-8. doi: 10.1016/j.msec.2018.04.055. PubMed PMID: WOS:000435619600019.
504. Liu J, Xu F, Huang J, Xu J, Liu Y, Yao Y, et al. Low-intensity focused ultrasound (LIFU)-activated nanodroplets as a theranostic agent for noninvasive cancer molecular imaging and drug delivery. Biomaterials Science. 2018;6(11). doi: 10.1039/c8bm00726h. PubMed PMID: WOS:000448822800004.
505. Liu X, Quan N. Microglia and CNS Interleukin-1: Beyond Immunological Concepts. Frontiers in Neurology. 2018;9. doi: 10.3389/fneur.2018.00008. PubMed PMID: WOS:000423072300002.
506. Luzuriaga MA, Berry DR, Reagan JC, Smaldone RA, Gassensmith JJ. Biodegradable 3D printed polymer microneedles for transdermal drug delivery. Lab on a Chip. 2018;18(8):1223-30. doi: 10.1039/c8lc00098k. PubMed PMID: WOS:000430406400007.
507. Maltese A, Salerno M, Cerroni F, Romano P, Di Folco A, Di Filippo T, et al. THE TRANSCRANIAL STIMULATION WITH

DIRECT CURRENTS (TDCS): AN HISTORICAL AND CONCEPTUAL MINIREVIEW. Acta Medica Mediterranea. 2018;34(3):697-702. doi: 10.19193/0393-6384_2018_3_107. PubMed PMID: WOS:000434980000012.

508. Marcelo G, Ariana-Machado J, Enea M, Carmo H, Rodriguez-Gonzalez B, Capelo JL, et al. Toxicological Evaluation of Luminescent Silica Nanoparticles as New Drug Nanocarriers in Different Cancer Cell Lines. Materials. 2018;11(8). doi: 10.3390/ma11081310. PubMed PMID: WOS:000444112800047.

509. Mishra R, Maiti TK, Bhattacharyya TK. Development of SU-8 hollow microneedles on a silicon substrate with microfluidic interconnects for transdermal drug delivery. Journal of Micromechanics and Microengineering. 2018;28(10). doi: 10.1088/1361-6439/aad301. PubMed PMID: WOS:000440308700002.

510. Mishra R, Matti TK, Bhattacharyya TK. Design and Scalable Fabrication of Hollow SU-8 Microneedles for Transdermal Drug Delivery. Ieee Sensors Journal. 2018;18(14):5635-44. doi: 10.1109/jsen.2018.2840335. PubMed PMID: WOS:000437024600002.

511. Neville IS, Gomes-Osman J, Amorim RLO, Hayashi CY, Galhardoni R, Zaninotto AL, et al. How can transcranial magnetic

stimulation change the way we treat traumatic brain injury? International Journal of Clinical and Experimental Medicine. 2018;11(8):7643-50. PubMed PMID: WOS:000443533700014.
512. Popova M, Trendafilova I, Szegedi A, Momekova D, Mihaly J, Momekov G, et al. Novel SO3H functionalized magnetic nanoporous silica/polymer nanocomposite as a carrier in a dual-drug delivery system for anticancer therapy. Microporous and Mesoporous Materials. 2018;263:96-105. doi: 10.1016/j.micromeso.2017.12.005. PubMed PMID: WOS:000434239700012.
513. Qu X, Dong Y, Jin H, Hou L. Research progress in non-invasive brain stimulation technique for rehabilitation of traumatic brain injury. Chinese Journal of Trauma. 2018;34(8):759-63. PubMed PMID: CSCD:6313390.
514. Rai S, Drislane FW. Treatment of Refractory and Super-refractory Status Epilepticus. Neurotherapeutics. 2018;15(3):697-712. doi: 10.1007/s13311-018-0640-5. PubMed PMID: WOS:000441999200014.
515. Rothen N, Schwartzman DJ, Bor D, Seth AK. Coordinated neural, behavioral, and phenomenological changes in perceptual plasticity through overtraining of synesthetic associations. Neuropsychologia. 2018;111:151-62. doi:

10.1016/j.neuropsychologia.2018.01.030. PubMed PMID: WOS:000430644300019.
516. Ryan K, Schiller K, Rezaie R, Baughman BC, Narayana S. A single season of soccer results in no significant neurophysiological changes in adolescent female athletes. Neurology. 2018;91(23). doi: 10.1212/01.wnl.0000550672.77974.75. PubMed PMID: WOS:000541962800039.
517. Schmidt J, Hayward KS, Brown KE, Zwicker JG, Ponsford J, van Donkelaar P, et al. Imaging in Pediatric Concussion: A Systematic Review. Pediatrics. 2018;141(5). doi: 10.1542/peds.2017-3406. PubMed PMID: WOS:000431231300028.
518. Schmidt J, Rubino C, Boyd LA, Virji-Babul N. The Role of Physical Activity in Recovery From Concussion in Youth: A Neuroscience Perspective. Journal of Neurologic Physical Therapy. 2018;42(3):155-62. doi: 10.1097/npt.0000000000000226. PubMed PMID: WOS:000436873100007.
519. Song M, Zhang Y, Cui Y, Yang Y, Jiang T. Brain Network Studies in Chronic Disorders of Consciousness: Advances and Perspectives. Neuroscience Bulletin. 2018;34(4):592-604. doi: 10.1007/s12264-018-0243-5. PubMed PMID: WOS:000440164500002.

520. Taghdiri F, Chung J, Irwin S, Multani N, Tarazi A, Ebraheem A, et al. Decreased Number of Self-Paced Saccades in Post-Concussion Syndrome Associated with Higher Symptom Burden and Reduced White Matter Integrity. Journal of Neurotrauma. 2018;35(5):719-29. doi: 10.1089/neu.2017.5274. PubMed PMID: WOS:000424839300001.
521. Takeuchi K, Kim B. Functionalized microneedles for continuous glucose monitoring. Nano Convergence. 2018;5. doi: 10.1186/s40580-018-0161-2. PubMed PMID: WOS:000451707500001.
522. Turolla A, Venneri A, Farina D, Cagnin A, Cheung VCK. Rehabilitation Induced Neural Plasticity after Acquired Brain Injury. Neural Plasticity. 2018;2018. doi: 10.1155/2018/6565418. PubMed PMID: WOS:000432801000001.
523. Ullah A, Kim CM, Kim GM. Porous polymer coatings on metal microneedles for enhanced drug delivery. Royal Society Open Science. 2018;5(4). doi: 10.1098/rsos.171609. PubMed PMID: WOS:000431110100020.
524. Vallhov H, Xia W, Engqvist H, Scheynius A. Bioceramic microneedle arrays are able to deliver OVA to dendritic cells in human skin. Journal of Materials Chemistry B. 2018;6(42):6808-16. doi: 10.1039/c8tb01476k. PubMed PMID:

WOS:000451177200011.
525. Whalen AJ, Xiao Y, Kadji H, Dahlem MA, Gluckman BJ, Schiff SJ. Control of Spreading Depression with Electrical Fields. Scientific Reports. 2018;8. doi: 10.1038/s41598-018-26986-1. PubMed PMID: WOS:000434640800023.
526. Wieckowska A, Gajewska-Wozniak O, Glowacka A, Ji B, Grycz K, Czarkowska-Bauch J, et al. Spinalization and locomotor training differentially affect muscarinic acetylcholine receptor type 2 abutting on -motoneurons innervating the ankle extensor and flexor muscles. Journal of Neurochemistry. 2018;147(3):361-79. doi: 10.1111/jnc.14567. PubMed PMID: WOS:000450112100005.
527. Yasen AL, Edwards EK, Eick G, Sterner K, Christie AD. Motor Cortex Function in Symptomatic and Asymptomatic Individuals Following Mild Traumatic Brain Injury. Medicine and Science in Sports and Exercise. 2018;50(5):564-. PubMed PMID: WOS:000456870502370.
528. Zhang X, Hou J, Duan G. Effects of transcranial direct current stimulation on psychomotor inhibition state in patients with traumatic brain injury. Chinese Journal of Rehabilitation Medicine. 2018;33(12):1405-9. PubMed PMID: CSCD:6395336.
529. Zhang X, Zhu H, Tao W, Li Y, Hu Y. Motor Cortex Stimulation Therapy for Relief of Central Post-Stroke Pain: A

Retrospective Study with Neuropathic Pain Symptom Inventory. Stereotactic and Functional Neurosurgery. 2018;96(4):239-43. doi: 10.1159/000492056. PubMed PMID: WOS:000446819900004.

530. Zhao J. Disorders of Consciousness in China. Neuroscience Bulletin. 2018;34(4):605-14. doi: 10.1007/s12264-018-0263-1. PubMed PMID: WOS:000440164500003.

531. Zhao J, Ni Y, Lin H. A study on resting state fMRI of brain function network in patients with minimally conscious state by transcranial direct current stimulation. Chinese Journal of Rehabilitation Medicine. 2018;33(3):259-63. PubMed PMID: CSCD:6203322.

532. Zhao J, Ni Y, Lin H, Liu H, Lu X, Dong J, et al. A comparative study of the effect of a single transcranial direct current stimulation on resting state of brain functional network in patients in a minimally conscious state. Chinese Journal of Physical Medicine and Rehabilitation. 2018;40(9):647-51. PubMed PMID: CSCD:6459794.

533. Zhao X, Luo W, Hu J, Zuo L, Wang J, Hu R, et al. Cardiomyocyte-targeted and 17 beta-estradiol-loaded acoustic nanoprobes as a theranostic platform for cardiac hypertrophy. Journal of Nanobiotechnology. 2018;16. doi: 10.1186/s12951-018-0360-3. PubMed PMID:

WOS:000428929500004.
534. Abubaker SS, Zhang Y. Optimization Design and Fabrication of Polymer Micro Needle by Hot Embossing Method. International Journal of Precision Engineering and Manufacturing. 2019;20(4):631-40. doi: 10.1007/s12541-019-00095-z. PubMed PMID: WOS:000464704300012.
535. Askalsky P, Iosifescu DV. Transcranial Photobiomodulation For The Management Of Depression: Current Perspectives. Neuropsychiatric Disease and Treatment. 2019;15:3255-72. doi: 10.2147/ndt.S188906. PubMed PMID: WOS:000500986900001.
536. Bai Y, Xia X, Wang Y, He J, Li X. Assessment Effects of Repetitive Transcranial Magnetic Stimulation in Patients with Disorders of Consciousness by EEG. Chinese Journal of Biomedical Engineering. 2019;38(6):687-94. PubMed PMID: CSCD:6645150.
537. Bareham CA, Allanson J, Roberts N, Hutchinson PJA, Pickard JD, Menon DK, et al. Longitudinal assessments highlight long-term behavioural recovery in disorders of consciousness. Brain communications. 2019;1(1):fcz017-fcz. doi: 10.1093/braincomms/fcz017. PubMed PMID: MEDLINE:31886461.
538. Beisegel J, Denkert C, Koehler E, Krnc M, Pivac N, Scheffler

R, et al. On the End-Vertex Problem of Graph Searches. Discrete Mathematics and Theoretical Computer Science. 2019;21(1). PubMed PMID: WOS:000480435300008.

539. Bernier RA, Hillary FG. Traumatic brain injury and frontal lobe plasticity. Handbook of clinical neurology. 2019;163:411-31. doi: 10.1016/b978-0-12-804281-6.00022-7. PubMed PMID: MEDLINE:31590744.

540. Bodnar C, Bachstetter A. Understanding interleukin-1 receptor 1 localization after traumatic brain injury. Journal of Neurochemistry. 2019;150:170-. PubMed PMID: WOS:000478639800423.

541. Carey L, Nilsson M, Boyd L. Learning following Brain Injury: Neural Plasticity Markers. Neural Plasticity. 2019;2019. doi: 10.1155/2019/4838159. PubMed PMID: WOS:000486427000001.

542. Cary JH, Li BS, Maibach HI. Dermatotoxicology of microneedles (MNs) in man. Biomedical Microdevices. 2019;21(3). doi: 10.1007/s10544-019-0371-3. PubMed PMID: WOS:000474371300001.

543. Chemali Z, Nehme R, Fricchione G. Sensory neurologic disorders: Tinnitus. Handbook of clinical neurology. 2019;165:365-81. doi: 10.1016/b978-0-444-64012-3.00022-8. PubMed PMID: MEDLINE:31727224.

544. Cole G, Ali AA, McErlean E, Mulholland EJ, Short A, McCrudden CM, et al. DNA vaccination via RALA nanoparticles in a microneedle delivery system induces a potent immune response against the endogenous prostate cancer stem cell antigen. Acta Biomaterialia. 2019;96:480-90. doi: 10.1016/j.actbio.2019.07.003. PubMed PMID: WOS:000486132900038.

545. Das N, Spence JS, Aslan S, Vanneste S, Mudar R, Rackley A, et al. Cognitive Training and Transcranial Direct Current Stimulation in Mild Cognitive Impairment: A Randomized Pilot Trial. Frontiers in Neuroscience. 2019;13. doi: 10.3389/fnins.2019.00307. PubMed PMID: WOS:000464451200001.

546. Dave A, Ganesh A, Adil MM, Tsao JW. Practice Current: How do you diagnose and treat post-concussive headache? Neurology Clinical practice. 2019;9(3):263-70. doi: 10.1212/cpj.0000000000000656. PubMed PMID: MEDLINE:31341715.

547. Fonseca DFS, Vilela C, Silvestre AJD, Freire CSR. A compendium of current developments on polysaccharide and protein-based microneedles. International Journal of Biological Macromolecules. 2019;136:704-28. doi:

10.1016/j.ijbiomac.2019.04.163. PubMed PMID: WOS:000482533000073.

548. Gaware SA, Rokade KA, Bala P, Kale SN. Microneedles of chitosan-porous carbon nanocomposites: Stimuli (pH and electric field)-initiated drug delivery and toxicological studies. Journal of Biomedical Materials Research Part A. 2019;107(8):1582-96. doi: 10.1002/jbm.a.36672. PubMed PMID: WOS:000471813900002.

549. Guo F, Ouyang T, Peng T, Zhang X, Xie B, Yang X, et al. Enhanced oral absorption of insulin using colon-specific nanoparticles co-modified with amphiphilic chitosan derivatives and cell-penetrating peptides. Biomaterials Science. 2019;7(4):1493-506. doi: 10.1039/c8bm01485j. PubMed PMID: WOS:000463746600021.

550. Guo S, Yin Z, Lu G, Huang X. A comparative study of repetitive transcranial magnetic stimulation at different frequencies in arousal for coma patients after brain stem injury. Chinese Journal of Neuromedicine. 2019;18(6):550-4. PubMed PMID: CSCD:6521188.

551. He R, Zheng Be, Wu H. The local change of EEG in the severe brain injury patients after rTMS intervention. Chinese Journal of Rehabilitation Medicine. 2019;34(12):1418-22. PubMed PMID: CSCD:6629230.

552. Herrold A, Jordan N, Parrish T, Cobia D, Chambers RA, Sander A, et al. Multi-Modal Neuroimaging Characterization of Co-Occurring Mild Traumatic Brain Injury and Alcohol Use Disorder. Biological Psychiatry. 2019;85(10):S293-S4. doi: 10.1016/j.biopsych.2019.03.744. PubMed PMID: WOS:000472661000719.
553. Herrold AA, Jordan N, Parrish T, Cobia D, Wang X, Chambers RA, et al. USING MULTI-MODAL NEUROIMAGING TO INFORM NEUROMODULATORY TREATMENT FOR CO-OCCURRING ALCOHOL USE DISORDER AND MILD TRAUMATIC BRAIN INJURY. Alcoholism-Clinical and Experimental Research. 2019;43:308A-A. PubMed PMID: WOS:000468963104199.
554. Horton G, Ibrahim O, Jansen M, Milev R, Beyea J, Trier J. Repetitive Trans-Cranial Magnetic Stimulation (rTMS) for the Treatment of Chronic Tinnitus in Patients with and without A History of Traumatic Brain Injury (TBI). Brain Injury. 2019;33:58-9. PubMed PMID: WOS:000466897000131.
555. Hu F, Zheng L, Liang E, Ding L, Wu L, Chen G, et al. Right anterior ganglionated plexus: The primary target of cardioneuroablation? Heart Rhythm. 2019;16(10):1545-51. doi: 10.1016/j.hrthm.2019.07.018. PubMed PMID:

WOS:000488200900015.
556. Iovino L, Kinsella S, Cooper K, Jain R, DeRoos P, Gagnon A, et al. Zinc Treatment Stimulates Thymic Regeneration after Bone Marrow Transplant. Blood. 2019;134. doi: 10.1182/blood-2019-129290. PubMed PMID: WOS:000577164603154.
557. Keller-Ross ML, Chantigian DP, Rich TL, Chen M, Chen C-Y, Gillick BT. Stability of the cardiovascular response during single-pulse TMS in perinatal stroke. Brain Stimulation. 2019;12(2):371-3. doi: 10.1016/j.brs.2018.11.010. PubMed PMID: WOS:000456437000023.
558. Kim Y, Bhattaccharjee SA, Beck-Broichsitter M, Banga AK. Fabrication and characterization of hyaluronic acid microneedles to enhance delivery of magnesium ascorbyl phosphate into skin. Biomedical Microdevices. 2019;21(4). doi: 10.1007/s10544-019-0455-0. PubMed PMID: WOS:000499456800002.
559. Lechner A, Spataro R, Guger C. Using vibro-tactile brain-computer interfaces in patients with unresponsive wakefulness syndrome. European Journal of Neurology. 2019;26:932-. PubMed PMID: WOS:000474481004141.
560. Legostaeva L, Sinitsyn D, Sergeev D, Iazeva E, Kremneva E,

Poydasheva A, et al. Comparison of the neurophysiological and neuroimaging data provides more accurate disorder of consciousness diagnostics. European Journal of Neurology. 2019;26:181-. PubMed PMID: WOS:000474481000286.
561. Li H, Wang Z, He Y, Meng S, Xu Y, Chen S, et al. Rational synthesis of MnxCd1-xS for enhanced photocatalytic H-2 evolution: Effects of S precursors and the feed ratio of Mn/Cd on its structure and performance. Journal of Colloid and Interface Science. 2019;535:469-80. doi: 10.1016/j.jcis.2018.10.018. PubMed PMID: WOS:000452811600050.
562. Li S, Lu K, Pan M, Zhang R. A new similarity analysis of EEG signals for automaticepileptic seizure detection. Journal of Northwest University Natural Science Edition. 2019;49(2):309-17. PubMed PMID: CSCD:6466355.
563. Li Z, Wang X, Wang X, Lin Y, Meng A, Yang L, et al. Mn-Cd-S@amorphous-Ni3S2 hybrid catalyst with enhanced photocatalytic property for hydrogen production and electrocatalytic OER. Applied Surface Science. 2019;491:799-806. doi: 10.1016/j.apsusc.2019.05.313. PubMed PMID: WOS:000479082900090.
564. Liu F, Lin Z, Jin Q, Wu Q, Yang C, Chen H-J, et al. Protection of Nanostructures-Integrated Microneedle Biosensor Using

Dissolvable Polymer Coating. Acs Applied Materials & Interfaces. 2019;11(5):4809-19. doi: 10.1021/acsami.8b18981. PubMed PMID: WOS:000458347900012.

565. Liu Q, Li R, Qu W, Li B, Yang W, Cui R. Pharmacological and non-pharmacological interventions of depression after traumatic brain injury: A systematic review. European Journal of Pharmacology. 2019;865. doi: 10.1016/j.ejphar.2019.172775. PubMed PMID: WOS:000500178300041.

566. Liu Y, Fan S, Huang J, Wang P, Yin Y, Xue L, et al. Clinical effect of hyperbaric oxygen combined with transcranial electrical stimulation and memantine hydrochloride on cognitive impairment in the patients with craniocerebral injury. Chinese Journal of Nautical Medicine and Hyperbaric Medicine. 2019;26(4):320-3. PubMed PMID: CSCD:6573524.

567. Luber B. Optimization of TMS Delivery in the Temporal Domain. Neuropsychopharmacology. 2019;44(SUPPL 1):74-5. PubMed PMID: WOS:000509665600159.

568. Luo Z, Sun W, Fang J, Lee K, Li S, Gu Z, et al. Biodegradable Gelatin Methacryloyl Microneedles for Transdermal Drug Delivery. Advanced Healthcare Materials. 2019;8(3). doi: 10.1002/adhm.201801054. PubMed PMID: WOS:000459627200002.

569. Martens K, Pechacek KP, Zhu B, Modrak CG, Milleson VJ, O'Hearn CO, et al. TRANSCRANIAL DIRECT-CURRENT STIMULATION REDUCES IMPULSIVITY AFTER EXPERIMENTAL TBI. Journal of Neurotrauma. 2019;36(13):A84-A. PubMed PMID: WOS:000473049600230.

570. McCall IC, Lau C, Minielly N, Illes J. Owning Ethical Innovation: Claims about Commercial Wearable Brain Technologies. Neuron. 2019;102(4):728-31. doi: 10.1016/j.neuron.2019.03.026. PubMed PMID: WOS:000468597000009.

571. Mensen A, Thibaut A, Bodart O, Wannez S, Laureys S, Gosseries O. Decreased Evoked Slow-Activity After tDCS in Patients with Severe Brain Injuries and Altered State of Consciousness. Brain Injury. 2019;33:16-. PubMed PMID: WOS:000466897000038.

572. Metzger-Smith V, Vaninetti M, Lim M, Khalaf A, Yang E, Song D, et al. PREFRONTAL MODULATORY FUNCTIONAL CHANGES AFTER RTMS IN MTBI PATIENTS WITH HEADACHES. Journal of Neurotrauma. 2019;36(13):A66-A. PubMed PMID: WOS:000473049600180.

573. Neville IS, Zaninotto AL, Hayashi CY, Rodrigues PA, Galhardoni R, de Andrade DC, et al. Repetitive TMS does not

improve cognition in patients with TBI. Neurology. 2019;93(2):E190-E9. doi: 10.1212/wnl.0000000000007748. PubMed PMID: WOS:000480783800009.
574. Oliveira E, Santos HM, Jorge S, Rodriguez-Gonzalez B, Novio F, Lorenzo J, et al. Sustainable synthesis of luminescent CdTe quantum dots coated with modified silica mesoporous nanoparticles: Towards new protein scavengers and smart drug delivery carriers. Dyes and Pigments. 2019;161:360-9. doi: 10.1016/j.dyepig.2018.09.047. PubMed PMID: WOS:000449241200044.
575. Padmanabhan JL, Cooke D, Joutsa J, Siddiqi SH, Ferguson M, Darby RR, et al. A Human Depression Circuit Derived From Focal Brain Lesions. Biological Psychiatry. 2019;86(10):749-58. doi: 10.1016/j.biopsych.2019.07.023. PubMed PMID: WOS:000491238900007.
576. Parravano DC, Ciampi DA, Fonoff ET, Monaco B, Navarro J, Yeng LT, et al. Quality of Life After Motor Cortex Stimulation: Clinical Results and Systematic Review of the Literature. Neurosurgery. 2019;84(2):451-6. doi: 10.1093/neuros/nyy060. PubMed PMID: WOS:000463732100026.
577. Paydar A, Schoenfield JS, Yan E, Kamali A, Kochan T, Harris NG. PROLONGED NEUROMODULATION AFTER

EXPERIMENTAL TBI ENHANCES FUNCTIONAL CONNECTIVITY BUT DOES NOT IMPROVE EVOKED ACTIVATION. Journal of Neurotrauma. 2019;36(13):A142-A3. PubMed PMID: WOS:000473049600393.

578. Pommier B, Quesada C, Fauchon C, Nuti C, Vassal F, Peyron R. Added value of multiple versus single sessions of repetitive transcranial magnetic stimulation in predicting motor cortex stimulation efficacy for refractory neuropathic pain. Journal of Neurosurgery. 2019;130(5):1750-61. doi: 10.3171/2017.12.Jns171333. PubMed PMID: WOS:000466401100040.

579. Poydasheva AG, Bakulin IS, Legostaeva LA, Suponeva NA, Piradov MA. TMS-EEG: CURRENT POSSIBILITIES AND FUTURE PROSPECTS. Zhurnal Vysshei Nervnoi Deyatelnosti Imeni I P Pavlova. 2019;69(3):267-79. doi: 10.1134/s0044467719030092. PubMed PMID: WOS:000473092700001.

580. Quentin R, Awosika O, Cohen LG. Plasticity and recovery of function. Handbook of clinical neurology. 2019;163:473-83. doi: 10.1016/b978-0-12-804281-6.00025-2. PubMed PMID: MEDLINE:31590747.

581. Rastogi P, Lee EG, Hadimani RL, Jiles DC. Transcranial

Magnetic Stimulation: Development of a Novel Deep-Brain Triple-Halo Coil. Ieee Magnetics Letters. 2019;10. doi: 10.1109/lmag.2019.2903993. PubMed PMID: WOS:000463473100001.
582. Rong P, Zhang Y, Li S, Wang Y. Present Status and Prospect of Transcutaneous Auricular Vagus Nerve Stimulation in the Treatment of Encephalopathy and Relating Diseases. Modernization of Traditional Chinese Medicine and Materia Medica--World Science and Technology. 2019;21(9):1799-804. PubMed PMID: CSCD:6672742.
583. Seon-Woo H-S, Kim HJ, Roh JY, Park J-H. Dissolving Microneedle Systems for the Oral Mucosal Delivery of Triamcinolone Acetonide to Treat Aphthous Stomatitis. Macromolecular Research. 2019;27(3):282-9. doi: 10.1007/s13233-019-7031-6. PubMed PMID: WOS:000467619900009.
584. Shen L, Ouyang H, Yang C. The effect of high- frequency repetitive transcranial magnetic stimulation on consciousness recovery in traumatic brain injury patients with disorders of consciousness. Chinese Journal of Rehabilitation Medicine. 2019;34(12):1411-7. PubMed PMID: CSCD:6629229.
585. Skinner K, Papa L, Ptito A. Cranial Nerve Noninvasive

Neuromodulation Using Translingual Stimulation with the Portable Neuromodulation Stimulator (PoNS (R)) for the Treatment of Chronic Balance Deficit Due to Mild-to-Moderate Traumatic Brain Injury. Brain Injury. 2019;33:317-8. PubMed PMID: WOS:000466897000639.

586. Sohn E. DECODING CONSCIOUSNESS. Nature. 2019;571(7766):S2-S5. doi: 10.1038/d41586-019-02207-1. PubMed PMID: WOS:000477016700019.

587. Stilling JM, Duszynski CC, Oni I, Paxman E, Dunn JF, Debert CT. Using Functional Near-Infrared Spectroscopy to Study the Effect of Repetitive Transcranial Magnetic Stimulation in Concussion: A Two-Patient Case Study. Frontiers in Neurology. 2019;10. doi: 10.3389/fneur.2019.00476. PubMed PMID: WOS:000467346400001.

588. Tanaka N, Tsutsumi R, Shirota Y, Shimizu T, Ohminami S, Terao Y, et al. Effects of L-DOPA on quadripulse magnetic stimulation-induced long-term potentiation in older adults. Neurobiology of Aging. 2019;84:217-24. doi: 10.1016/j.neurobiolaging.2019.08.005. PubMed PMID: WOS:000501576800024.

589. Tyler M, Skinner K, Prabhakaran V. Neurorehabilitation Using the Portable Neuromodulation Stimulator (PoNS (R)) for Treating

Patients with Chronic Balance Deficit Due to Mild-to-Moderate Traumatic Brain Injury (mTBI): A 26-Week Study. Brain Injury. 2019;33:228-9. PubMed PMID: WOS:000466897000464.

590. Wang Y, Sui G, Teng D, Wang Q, Qu J, Zhu L, et al. Low intensity focused ultrasound (LIFU) triggered drug release from cetuximab-conjugated phase-changeable nanoparticles for precision theranostics against anaplastic thyroid carcinoma. Biomaterials Science. 2019;7(1):196-210. doi: 10.1039/c8bm00970h. PubMed PMID: WOS:000454085700012.

591. Yang H, Wu X, Zhou Z, Chen X, Kong M. Enhanced transdermal lymphatic delivery of doxorubicin via hyaluronic acid based transfersomes/microneedle complex for tumor metastasis therapy. International Journal of Biological Macromolecules. 2019;125:9-16. doi: 10.1016/j.ijbiomac.2018.11.230. PubMed PMID: WOS:000458222000002.

592. Zhang X, Cheng J, Zeng J, Hong J, Chen F, Li M, et al. Predicting role of disturbance coefficient in post brain traumatic hydrocephalus. Chinese Journal of Neurosurgery. 2019;35(2):153-6. PubMed PMID: CSCD:6438369.

593. Zhang Y, Chai D, Gao M, Xu B, Jiang G. Thermal ablation of separable microneedles for transdermal delivery of metformin on diabetic rats. International Journal of Polymeric Materials and

Polymeric Biomaterials. 2019;68(14):850-8. doi: 10.1080/00914037.2018.1517347. PubMed PMID: WOS:000468504100006.

594. Zhong S, Zhang J, Zhang L, Ye X. Rencent advance in transcranial direct current stimulation in rehabilitation of patients with disorder of consciousness. Chinese Journal of Neuromedicine. 2019;18(3):297-301. PubMed PMID: CSCD:6470883.

595. Zhong Y, Zhang Y, Xu J, Zhou J, Liu J, Ye M, et al. Low-Intensity Focused Ultrasound-Responsive Phase-Transitional Nanoparticles for Thrombolysis without Vascular Damage: A Synergistic Nonpharmaceutical Strategy. Acs Nano. 2019;13(3):3387-403. doi: 10.1021/acsnano.8b09277. PubMed PMID: WOS:000462950500065.

596. Zurawski E, Behm K, Dunlap C, Koo J, Ismail F, Boulias C, et al. Interrater Reliability of the Modified Ashworth Scale with Standardized Movement Speeds: A Pilot Study. Physiotherapy Canada. 2019;71(4):348-54. doi: 10.3138/ptc-2018-0086. PubMed PMID: WOS:000501336200007.

597. Ahmed M, Balse A, Diep M, Amin H, Haque S. Oculomotor Dysfunction Improvement in Moderate TBI Patients With Repetitive Transcranial Magnetic Stimulation (rTMS). Journal of Head Trauma Rehabilitation. 2020;35(2):E228-E. PubMed PMID:

WOS:000525012500164.

598. Amer M, Chen RK. Self-Adhesive Microneedles with Interlocking Features for Sustained Ocular Drug Delivery. Macromolecular Bioscience. 2020;20(6). doi: 10.1002/mabi.202000089. PubMed PMID: WOS:000542595000003.

599. Angkawinitwong U, Courtenay AJ, Rodgers AM, Larraneta E, McCarthy HO, Brocchini S, et al. A Novel Transdermal Protein Delivery Strategy via Electrohydrodynamic Coating of PLGA Microparticles onto Microneedles. Acs Applied Materials & Interfaces. 2020;12(11):12478-88. doi: 10.1021/acsami.9b22425. PubMed PMID: WOS:000526543400010.

600. Badran BW, Jenkins DD, Cook D, Thompson S, Dancy M, DeVries WH, et al. Transcutaneous Auricular Vagus Nerve Stimulation-Paired Rehabilitation for Oromotor Feeding Problems in Newborns: An Open-Label Pilot Study. Frontiers in Human Neuroscience. 2020;14. doi: 10.3389/fnhum.2020.00077. PubMed PMID: WOS:000525569400001.

601. Bai S, Liao J, Zhang B, Zhao M, You B, Li P, et al. Multimodal and multifunctional nanoparticles with platelet targeting ability and phase transition efficiency for the molecular imaging and thrombolysis of coronary microthrombi. Biomaterials

Science. 2020;8(18):5047-60. doi: 10.1039/d0bm00818d. PubMed PMID: WOS:000569877500008.

602. Bapna A, Adin C, Engelman ZJ, Fudim M. Increasing Blood Pressure by Greater Splanchnic Nerve Stimulation: a Feasibility Study. Journal of Cardiovascular Translational Research. 2020;13(4):509-18. doi: 10.1007/s12265-019-09929-7. PubMed PMID: WOS:000494380000001.

603. Billeri L, Filoni S, Russo EF, Portaro S, Militi D, Calabro RS, et al. Toward Improving Diagnostic Strategies in Chronic Disorders of Consciousness: An Overview on the (Re-)Emergent Role of Neurophysiology. Brain Sciences. 2020;10(1). doi: 10.3390/brainsci10010042. PubMed PMID: WOS:000513130500027.

604. Campos R, Gomez R, Velarde L. SACRAL NEUROMODULATION WITH INTERSTIM (R) FOR URINARY VOIDING DYSFUNCTION IN POST-TRAUMATIC BRAIN INJURY PATIENTS. Journal of Urology. 2020;203:E698-E. PubMed PMID: WOS:000527010303051.

605. Chae J-S, Yuwon C, Kwon KM. The Effects of Proprioceptive Exercise Combined with Cognitive Task on the Balance and Ankle Function of Chronic Ankle Instability Adult. Journal of the Korean Society of Physical Medicine. 2020;15(1):65-76. doi:

10.13066/kspm.2020.15.1.65. PubMed PMID: KJD:ART002564681.
606. Chen G, Cao Y, Tang Y, Yang X, Liu Y, Huang D, et al. Advanced Near-Infrared Light for Monitoring and Modulating the Spatiotemporal Dynamics of Cell Functions in Living Systems. Advanced Science. 2020;7(8). doi: 10.1002/advs.201903783. PubMed PMID: WOS:000516660100001.
607. Chen J, Wang Y. Personalized dynamic transport of magnetic nanorobots inside the brain vasculature. Nanotechnology. 2020;31(49). doi: 10.1088/1361-6528/abb392. PubMed PMID: WOS:000576444600001.
608. Chu S, Uplekar N, Liu S, Ghodssi R. Complementary Capillary System Integrated Microneedles for Autonomously Localized Therapeutics Loading. Journal of Microelectromechanical Systems. 2020;29(5):912-7. doi: 10.1109/jmems.2020.2999255. PubMed PMID: WOS:000576466500043.
609. Conti A, Kamimura HAS, Novell A, Duggento A, Toschi N. Magnetic Resonance Methods for Focused Ultrasound-Induced Blood-Brain Barrier Opening. Frontiers in Physics. 2020;8. doi: 10.3389/fphy.2020.547674. PubMed PMID: WOS:000579081100001.

610. Dwivedi P, Kiran S, Han S, Dwivedi M, Khatik R, Fan R, et al. Magnetic Targeting and Ultrasound Activation of Liposome-Microbubble Conjugate for Enhanced Delivery of Anticancer Therapies. Acs Applied Materials & Interfaces. 2020;12(21):23737-51. doi: 10.1021/acsami.0c05308. PubMed PMID: WOS:000537731900009.
611. Dwyer B, Zasler N. Post-traumatic cephalalgia. Neurorehabilitation. 2020;47(3):327-42. doi: 10.3233/nre-208006. PubMed PMID: WOS:000592868600007.
612. Ellison A, Dunne S, Lane AR. Real-world applications in vision and attention: How to help patients find their (golf) balls again. In: Parkin BL, editor. Real-World Applications in Cognitive Neuroscience. Progress in Brain Research. 2532020. p. 169-200.
613. Fang J-H, Liu C-H, Hsu R-S, Chen Y-Y, Chiang W-H, Wang H-MD, et al. Transdermal Composite Microneedle Composed of Mesoporous Iron Oxide Nanoraspberry and PVA for Androgenetic Alopecia Treatment. Polymers. 2020;12(6). doi: 10.3390/polym12061392. PubMed PMID: WOS:000550735500001.
614. Feng YH, Liu JL, Zhu DD, Hao YY, Guo XD. Multiscale simulations of drug distributions in polymer dissolvable microneedles. Colloids and Surfaces B-Biointerfaces. 2020;189.

doi: 10.1016/j.colsurfb.2020.110844. PubMed PMID: WOS:000527314700027.

615. Foysal KMR, Baker SN. Induction of plasticity in the human motor system by motor imagery and transcranial magnetic stimulation. Journal of Physiology-London. 2020;598(12):2385-96. doi: 10.1113/jp279794. PubMed PMID: WOS:000531304600001.

616. Franzini A, Messina G, Levi V, D'Ammando A, Cordella R, Moosa S, et al. Deep brain stimulation of the posterior limb of the internal capsule in the treatment of central poststroke neuropathic pain of the lower limb: case series with long-term follow-up and literature review. Journal of Neurosurgery. 2020;133(3):830-8. doi: 10.3171/2019.5.Jns19227. PubMed PMID: WOS:000586088300029.

617. Fujiki M, Yee KM, Steward O. Non-invasive High Frequency Repetitive Transcranial Magnetic Stimulation (hfrTMS) Robustly Activates Molecular Pathways Implicated in Neuronal Growth and Synaptic Plasticity in Select Populations of Neurons. Frontiers in Neuroscience. 2020;14. doi: 10.3389/fnins.2020.00558. PubMed PMID: WOS:000546087600001.

618. Gandois L, Hoyt AM, Mounier S, Le Roux G, Harvey CF, Claustres A, et al. From canals to the coast: dissolved organic matter and trace metal composition in rivers draining degraded

tropical peatlands in Indonesia. Biogeosciences. 2020;17(7):1897-909. doi: 10.5194/bg-17-1897-2020. PubMed PMID: WOS:000525367000001.

619. Hampstead BM, Bahar-Fuchs A. Neurophysiological mechanisms and outcomes of nonpharmacologic interventions for neurological disease or injury: Introduction to special issue. International Journal of Psychophysiology. 2020;154:1-2. doi: 10.1016/j.ijpsycho.2020.02.010. PubMed PMID: WOS:000540674600001.

620. Han D, Morde RS, Mariani S, La Mattina AA, Vignali E, Yang C, et al. 4D Printing of a Bioinspired Microneedle Array with Backward-Facing Barbs for Enhanced Tissue Adhesion. Advanced Functional Materials. 2020;30(11). doi: 10.1002/adfm.201909197. PubMed PMID: WOS:000510696800001.

621. Henderson TA, van Lierop MJ, McLean M, Uszler JM, Thornton JF, Siow Y-H, et al. Functional Neuroimaging in Psychiatry-Aiding in Diagnosis and Guiding Treatment. What the American Psychiatric Association Does Not Know. Frontiers in Psychiatry. 2020;11. doi: 10.3389/fpsyt.2020.00276. PubMed PMID: WOS:000530505100001.

622. Herrold AA, Siddiqi S, Parrish T, Cobia D, Chambers RA, Jordan N, et al. INTEGRATION OF CUE REACTIVITY TASK

AND RESTING STATE MRI DATA TO DETERMINE TMS SITE OF STIMULATION FOR CO-OCCURRING AUD & MILD TBI. Alcoholism-Clinical and Experimental Research. 2020;44:71-. PubMed PMID: WOS:000540372300257.

623. Hou J, Nelson R, Mohammad N, Mustafa G, Plant D, Thompson FJ, et al. Effect of Simultaneous Combined Treadmill Training and Magnetic Stimulation on Spasticity and Gait Impairments after Cervical Spinal Cord Injury. Journal of Neurotrauma. 2020;37(18):1999-2013. doi: 10.1089/neu.2019.6961. PubMed PMID: WOS:000556431300001.

624. Houshangi A, Jamehbozorgi S, Yousefi M, Ghiasi R. Preparation of CoFe2O4/sawdust and NiFe2O4/sawdust magnetic nanocomposites for removal of oil from the water surface. Journal of the Chinese Chemical Society. 2020;67(2):288-97. doi: 10.1002/jccs.201800452. PubMed PMID: WOS:000481905000001.

625. Hsu W-L, Huang C-Y, Hsu Y-P, Hwang T-L, Chang S-H, Wang H-YJ, et al. On-skin glucose-biosensing and on-demand insulin-zinc hexamers delivery using microneedles for syringe-free diabetes management. Chemical Engineering Journal. 2020;398. doi: 10.1016/j.cej.2020.125536. PubMed PMID: WOS:000561588300007.

626. Hutton ARJ, McCrudden MTC, Larraneta E, Donnelly RF. Influence of molecular weight on transdermal delivery of model macromolecules using hydrogel-forming microneedles: potential to enhance the administration of novel low molecular weight biotherapeutics. Journal of Materials Chemistry B. 2020;8(19):4202-9. doi: 10.1039/d0tb00021c. PubMed PMID: WOS:000536814200004.

627. Iazeva E, Legostaeva L, Bakulin I, Poydasheva A, Shabalina A, Sergeev D, et al. RTMS increases BDNF levels in patients with posttraumatic chronic disorders of consciousness. European Journal of Neurology. 2020;27:399-. PubMed PMID: WOS:000534616801125.

628. Jiang Z, Xiong H, Yang S, Lu Y, Deng Y, Yao J, et al. Jet-Lagged Nanoparticles Enhanced Immunotherapy Efficiency through Synergistic Reconstruction of Tumor Microenvironment and Normalized Tumor Vasculature. Advanced Healthcare Materials. 2020;9(12). doi: 10.1002/adhm.202000075. PubMed PMID: WOS:000530633300001.

629. Kim M, Lee U. Alpha oscillation, criticality, and responsiveness in complex brain networks. Network Neuroscience. 2020;4(1):155-73. doi: 10.1162/netn_a_00113. PubMed PMID: WOS:000513793200007.

630. Kinsella S, Evandy C, Hopwo KS, Cooper K, Iovino L, Smith CW, et al. Damage-Induced Pyroptotic Cell Death Facilitates Regeneration of the Thymus. Blood. 2020;136. doi: 10.1182/blood-2020-143051. PubMed PMID: WOS:000607547203078.
631. Lei K, Kunnel A, Metzger-Smith V, Golshan S, Javors J, Wei J, et al. Diminished corticomotor excitability in Gulf War Illness related chronic pain symptoms; evidence from TMS study. Scientific Reports. 2020;10(1). doi: 10.1038/s41598-020-75006-8. PubMed PMID: WOS:000587657600016.
632. Leung A. Addressing chronic persistent headaches after MTBI as a neuropathic pain state. Journal of Headache and Pain. 2020;21(1). doi: 10.1186/s10194-020-01133-2. PubMed PMID: WOS:000543404700002.
633. Li Z, Zhang L, Zhang H, Yue L, Hu L. Pain-related gamma band oscillations: Progress and prospect. Chinese Science Bulletin. 2020;65(25):2752-62. PubMed PMID: CSCD:6818533.
634. Lim K-S, Chia Z-J, Myint M-Z, Ara KJ, Chee Y-C, Heng W-T, et al. Epilepsy in Southeast Asia, how much have we closed the management gap in past two decades? Neurology Asia. 2020;25(4):425-38. PubMed PMID: WOS:000616431700001.
635. Liu P, Du H, Chen Y, Wang H, Mao J, Zhang L, et al. Polymer

microneedles with interconnected porous structures via a phase inversion route for transdermal medical applications. Journal of Materials Chemistry B. 2020;8(10):2032-9. doi: 10.1039/c9tb02837d. PubMed PMID: WOS:000520502600017.

636. Liu S, Han X, Liu H, Zhao Y, Li H, Rupenthal DI, et al. Incorporation of ion exchange functionalized-montmorillonite into solid lipid nanoparticles with low irritation enhances drug bioavailability for glaucoma treatment. Drug Delivery. 2020;27(1):652-61. doi: 10.1080/10717544.2020.1756984. PubMed PMID: WOS:000530985800001.

637. Liu T, Jiang G, Song G, Zhu J, Yang Y. Fabrication of separable microneedles with phase change coating for NIR-triggered transdermal delivery of metformin on diabetic rats. Biomedical Microdevices. 2020;22(1). doi: 10.1007/s10544-019-0468-8. PubMed PMID: WOS:000511954500001.

638. Locke MB, Toepp SL, Turco CV, Harasym DH, Rathbone MP, Noseworthy MD, et al. Altered motor system function in post-concussion syndrome as assessed via transcranial magnetic stimulation. Clinical neurophysiology practice. 2020;5:157-64. doi: 10.1016/j.cnp.2020.07.004. PubMed PMID: MEDLINE:32939420.

639. Man VH, Li MS, Derreumaux P, Wang J, Nguyen TT, Nangia

S, et al. Molecular mechanism of ultrasound interaction with a blood brain barrier model. Journal of Chemical Physics. 2020;153(4). doi: 10.1063/5.0010667. PubMed PMID: WOS:000556316200003.

640. Modak M, Bobbala S, Lescott C, Liu Y-G, Nandwana V, Dravid VP, et al. Magnetic Nanostructure-Loaded Bicontinuous Nanospheres Support Multicargo Intracellular Delivery and Oxidation-Responsive Morphological Transitions. Acs Applied Materials & Interfaces. 2020;12(50):55584-95. doi: 10.1021/acsami.0c15920. PubMed PMID: WOS:000600202300005.

641. Moussi K, Bukhamsin A, Hidalgo T, Kosel J. Biocompatible 3D Printed Microneedles for Transdermal, Intradermal, and Percutaneous Applications. Advanced Engineering Materials. 2020;22(2). doi: 10.1002/adem.201901358. PubMed PMID: WOS:000502981900001.

642. Moussi K, Kavaldzhiev M, Perez JE, Alsharif N, Merzaban J, Kosel J. 3D Printed Microneedle Array for Electroporation. Annual International Conference of the IEEE Engineering in Medicine and Biology Society IEEE Engineering in Medicine and Biology Society Annual International Conference. 2020;2020:2202-5. doi: 10.1109/embc44109.2020.9175748. PubMed PMID:

MEDLINE:33018444.
643. Nabergoj M, Mauff K, Beelen DW, Ganser A, Kroger N, Bornhauser M, et al. Allogeneic Hematopoietic Cell Transplantation in Patients with Therapy-Related Myeloid Neoplasm after Breast Cancer: A Study of the Chronic Malignancies Working Party of the EBMT. Blood. 2020;136. doi: 10.1182/blood-2020-137773. PubMed PMID: WOS:000607547204058.
644. Noe E, Ferri J, Colomer C, Moliner B, O'Valle M, Ugart P, et al. Feasibility, safety and efficacy of transauricular vagus nerve stimulation in a cohort of patients with disorders of consciousness. Brain Stimulation. 2020;13(2):427-9. doi: 10.1016/j.brs.2019.12.005. PubMed PMID: WOS:000509471600028.
645. Pena J, Sampedro A, Ibarretxe-Bilbao N, Zubiaurre-Elorza L, Aizpurua A, Ojeda N. The effect of transcranial random noise stimulation (tRNS) over bilateral posterior parietal cortex on divergent and convergent thinking. Scientific Reports. 2020;10(1). doi: 10.1038/s41598-020-72532-3. PubMed PMID: WOS:000573849300087.
646. Plamadeala C, Gosain SR, Hischen F, Buchroithner B, Puthukodan S, Jacak J, et al. Bio-inspired microneedle design for

efficient drug/vaccine coating. Biomedical Microdevices. 2020;22(1). doi: 10.1007/s10544-019-0456-z. PubMed PMID: WOS:000511510400001.

647. Qu M, Kim H-J, Zhou X, Wang C, Jiang X, Zhu J, et al. Biodegradable microneedle patch for transdermal gene delivery. Nanoscale. 2020;12(32):16724-9. doi: 10.1039/d0nr02759f. PubMed PMID: WOS:000561429100009.

648. Rhoads M, Harper R, Snodgrass D, Yan-Go F, Sauerland E, Harper R. Neuromodulation of afferent fibers for Postural Orthostatic Trachycardia Syndrome (POTS). Faseb Journal. 2020;34. doi: 10.1096/fasebj.2020.34.s1.06800. PubMed PMID: WOS:000546107905210.

649. Roach AE, Hines C, Stafford J, Mooney S. Repetitive Transcranial Magnetic Stimulation for Treatment-Resistant Depression in Active-Duty Service Members Improves Depressive Symptoms. Journal of Ect. 2020;36(4):279-84. doi: 10.1097/yct.0000000000000680. PubMed PMID: WOS:000594475200022.

650. Siddiqi S, Horn A, Hsu J, Choi KS, Mayberg H, Grafman J, et al. A Convergent Causal Substrate for Depression Symptoms Across Lesions and Brain Stimulation. Biological Psychiatry. 2020;87(9):S46-S. PubMed PMID: WOS:000535308200110.

651. Stagg CJ. Exploring the infinite parameter space: rethinking assumptions underpinning the use of transcranial direct current stimulation to induce long-term effects. Journal of Physiology-London. 2020;598(4):621-2. doi: 10.1113/jp279295. PubMed PMID: WOS:000512369900001.
652. Stoppacher S, Scheruebel S, Ucal M, Kornmuller K, Glowacki E, Schindl R, et al. Modeling External Stimulation of Excitable Cells Using a Novel Light-Activated Organic Semiconductor Technology. Studies in health technology and informatics. 2020;271:9-16. doi: 10.3233/shti200068. PubMed PMID: MEDLINE:32578535.
653. Sydnor VJ, Bouix S, Pasternak O, Hartl E, Levin-Gleba L, Reid B, et al. Mild traumatic brain injury impacts associations between limbic system microstructure and post-traumatic stress disorder symptomatology. Neuroimage-Clinical. 2020;26. doi: 10.1016/j.nicl.2020.102190. PubMed PMID: WOS:000533149400014.
654. Tan M, Chen Y, Guo Y, Yang C, Liu M, Guo D, et al. A low-intensity focused ultrasound-assisted nanocomposite for advanced triple cancer therapy: local chemotherapy, therapeutic extracellular vesicles and combined immunotherapy. Biomaterials Science. 2020;8(23):6703-17. doi: 10.1039/d0bm00804d. PubMed

PMID: WOS:000592126300019.
655. Taylor RM, Maharjan D, Moreu F, Baca JT. Parametric study of 3D printed microneedle (MN) holders for interstitial fluid (ISF) extraction. Microsystem Technologies-Micro-and Nanosystems-Information Storage and Processing Systems. 2020;26(6):2067-73. doi: 10.1007/s00542-020-04758-0. PubMed PMID: WOS:000510351400003.
656. Teghil A, Di Vita A, Pietranelli V, Matano A, Boccia M. Duration reproduction in regular and irregular contexts after unilateral brain damage: Evidence from voxel-based lesion-symptom mapping and atlas-based hodological analysis. Neuropsychologia. 2020;147. doi: 10.1016/j.neuropsychologia.2020.107577. PubMed PMID: WOS:000581761400011.
657. Tucak A, Sirbubalo M, Hindija L, Rahic O, Hadziabdic J, Muhamedagic K, et al. Microneedles: Characteristics, Materials, Production Methods and Commercial Development. Micromachines. 2020;11(11). doi: 10.3390/mi11110961. PubMed PMID: WOS:000594026600001.
658. Volkers R, Giesen E, van der Heiden M, Kerperien M, Lange S, Kurt E, et al. Invasive Motor Cortex Stimulation Influences Intracerebral Structures in Patients With Neuropathic Pain: An

Activation Likelihood Estimation Meta-Analysis of Imaging Data. Neuromodulation. 2020;23(4):436-43. doi: 10.1111/ner.13119. PubMed PMID: WOS:000511365400001.

659. Wan H-P, Ni Y-Q. A New Approach for Interval Dynamic Analysis of Train-Bridge System Based on Bayesian Optimization. Journal of Engineering Mechanics. 2020;146(5). doi: 10.1061/(asce)em.1943-7889.0001735. PubMed PMID: WOS:000521194600005.

660. Wang H-y, Hou L, Li H-l, Wang X, Cao Y, Zhang B-y, et al. A nanosystem loaded with perfluorohexane and rose bengal coupled upconversion nanoparticles for multimodal imaging and synergetic chemo-photodynamic therapy of cancer. Biomaterials Science. 2020;8(9):2488-506. doi: 10.1039/c9bm02081k. PubMed PMID: WOS:000532529400010.

661. Wang M, Han Y, Yu X, Liang L, Chang H, Yeo DC, et al. Upconversion Nanoparticle Powered Microneedle Patches for Transdermal Delivery of siRNA. Advanced Healthcare Materials. 2020;9(2). doi: 10.1002/adhm.201900635. PubMed PMID: WOS:000499713200001.

662. Wang M, Yang Q, Li M, Zou H, Wang Z, Ran H, et al. Multifunctional Nanoparticles for Multimodal Imaging-Guided Low-Intensity Focused Ultrasound/Immunosynergistic

Retinoblastoma Therapy. Acs Applied Materials & Interfaces. 2020;12(5):5642-57. doi: 10.1021/acsami.9b22072. PubMed PMID: WOS:000512216900044.

663. Wang Y, Bai Y, Xia X, Yang Y, He J, Li X. Spinal cord stimulation modulates complexity of neural activities in patients with disorders of consciousness. International Journal of Neuroscience. 2020;130(7):662-70. doi: 10.1080/00207454.2019.1702543. PubMed PMID: WOS:000503531500001.

664. Wang Y, Wang H, Zhu XX, Guan Y, Zhang Y. Smart microneedle patches for rapid, and painless transdermal insulin delivery. Journal of Materials Chemistry B. 2020;8(40):9335-42. doi: 10.1039/d0tb01822h. PubMed PMID: WOS:000580584800012.

665. Wang Y, Xia X, Yang Y, Bai Y, He J, Li X. Recent advance in non-invasive neuromodulation in disorders of consciousness. Chinese Journal of Neuromedicine. 2020;19(9):966-71. PubMed PMID: CSCD:6807579.

666. Wilson C, Selwyn R, Quinn D, Elojeimy S. Detection of Regional Neurometabolic Differences in Individual TBI Subjects After tDCS Therapy. Journal of Nuclear Medicine. 2020;61. PubMed PMID: WOS:000568290501467.

667. Xie L, Zeng H, Sun J, Qian W. Engineering Microneedles for Therapy and Diagnosis: A Survey. Micromachines. 2020;11(3). doi: 10.3390/mi11030271. PubMed PMID: WOS:000526546000102.

668. Xu C-M, Tang M, Feng J, Xia H-F, Wu L-L, Pang D-W, et al. A liquid biopsy-guided drug release system for cancer theranostics: integrating rapid circulating tumor cell detection and precision tumor therapy. Lab on a Chip. 2020;20(8):1418-25. doi: 10.1039/d0lc00149j. PubMed PMID: WOS:000527017200003.

669. Yang A, Qiao B, Strohm EM, Cao J, Wang Z, Yuan X, et al. Thrombin-responsive engineered nanoexcavator with full-thickness infiltration capability for pharmaceutical-free deep venous thrombosis theranostics. Biomaterials Science. 2020;8(16):4545-58. doi: 10.1039/d0bm00917b. PubMed PMID: WOS:000558225900013.

670. Yang S, Chang MC. Effect of Repetitive Transcranial Magnetic Stimulation on Pain Management: A Systematic Narrative Review. Frontiers in Neurology. 2020;11. doi: 10.3389/fneur.2020.00114. PubMed PMID: WOS:000518616400001.

671. Yao W, Li D, Zhao Y, Zhan Z, Jin G, Liang H, et al. 3D Printed Multi-Functional Hydrogel Microneedles Based on High-Precision Digital Light Processing. Micromachines.

2020;11(1). doi: 10.3390/mi11010017. PubMed PMID: WOS:000514309100017.
672. Yasen AL, Eick GN, Sterner KN, Christie AD. The Influence Of Apoe Genotype On Motor Cortex Function Following Mtbi. Medicine and Science in Sports and Exercise. 2020;52(17):942-. PubMed PMID: WOS:000590026304178.
673. Zare EN, Jamaledin R, Naserzadeh P, Afjeh-Dana E, Ashtari B, Hosseinzadeh M, et al. Metal-Based Nanostructures/PLGA Nanocomposites: Antimicrobial Activity, Cytotoxicity, and Their Biomedical Applications. Acs Applied Materials & Interfaces. 2020;12(3):3279-300. doi: 10.1021/acsami.9b19435. PubMed PMID: WOS:000509428300001.
674. Zhang K-Y, Rui G, Zhang J-P, Guo L, An G-Z, Lin J-J, et al. Cathodal tDCS exerts neuroprotective effect in rat brain after acute ischemic stroke. Bmc Neuroscience. 2020;21(1). doi: 10.1186/s12868-020-00570-8. PubMed PMID: WOS:000535612200002.
675. Zhang X, Chen G, Liu Y, Sun L, Sun L, Zhao Y. Black Phosphorus-Loaded Separable Microneedles as Responsive Oxygen Delivery Carriers for Wound Healing. Acs Nano. 2020;14(5):5901-8. doi: 10.1021/acsnano.0c01059. PubMed PMID: WOS:000537682300073.

676. Zhang Y, Du J, Li R. The application of P300 in evaluating therapeutic efficacy of transcranial direct current stimulation in patients with minimally conscious state. Chinese Journal of Rehabilitation Medicine. 2020;35(1):22-8. PubMed PMID: CSCD:6656605.

677. Zhao K, Liu A. Effect of Right Median Nerve Stimulation on Clinical Efficacy and Prognosis of Patients with Consciousness Disorder Caused by Traumatic Brain Injury. Rehabilitation Medicine. 2020;30(3):192-6. PubMed PMID: CSCD:6757067.

678. Zhao X, Zhang S, Yang G, Zhou Z, Gao Y. Exploring Trehalose on the Release of Levonorgestrel from Implantable PLGA Microneedles. Polymers. 2020;12(1). doi: 10.3390/polym12010059. PubMed PMID: WOS:000519848300059.

679. Akram Z, Daood U, Aati S, Ngo H, Fawzy AS. Formulation of pH-sensitive chlorhexidine-loaded/mesoporous silica nanoparticles modified experimental dentin adhesive. Materials Science & Engineering C-Materials for Biological Applications. 2021;122. doi: 10.1016/j.msec.2021.111894. PubMed PMID: WOS:000624405100002.

680. An Y, Yang R, Wang X, Han Y, Jia G, Hu C, et al. Facile Assembly of Thermosensitive Liposomes for Active Targeting

Imaging and Synergetic Chemo-/Magnetic Hyperthermia Therapy. Frontiers in Bioengineering and Biotechnology. 2021;9. doi: 10.3389/fbioe.2021.691091. PubMed PMID: WOS:000687220800001.

681. Ayache SS, Chalah MA. Cognitive fatigability in the healthy brain: Neurophysiological substrates and the use of tDCS. Clinical Neurophysiology. 2021;132(7):1714-5. doi: 10.1016/j.clinph.2021.03.018. PubMed PMID: WOS:000659518700011.

682. Azizi S, Irani A, Shahrokhi A, Rahimian E, Mirbagheri MM. Contribution of altered corticospinal microstructure to gait impairment in children with cerebral palsy. Clinical Neurophysiology. 2021;132(9):2211-21. doi: 10.1016/j.clinph.2021.06.016. PubMed PMID: WOS:000691755500026.

683. Babity S, Laszlo E, Brambilla D. Polymer-Based Microneedles for Decentralized Diagnostics and Monitoring: Concepts, Potentials, and Challenges. Chemistry of Materials. 2021;33(18):7148-59. doi: 10.1021/acs.chemmater.1c01866. PubMed PMID: WOS:000703532600002.

684. Bareham CA, Oxner M, Gastrell T, Carmel D. Beyond the neural correlates of consciousness: using brain stimulation to

elucidate causal mechanisms underlying conscious states and contents. Journal of the Royal Society of New Zealand. 2021;51(1):143-70. doi: 10.1080/03036758.2020.1840405. PubMed PMID: WOS:000588817900001.

685. Barra A, Martens G, Pacheco-Barrios K, Gosseries O, Fregni F, Laureys S, et al. Neuromodulation to promote recovery in patients with disorders of consciousness: a systematic review and meta-analysis. European Journal of Neurology. 2021;28:443-. PubMed PMID: WOS:000663065901002.

686. Beisegel J, Denkert C, Koehler E, Krnc M, Pivac N, Scheffler R, et al. THE RECOGNITION PROBLEM OF GRAPH SEARCH TREES. Siam Journal on Discrete Mathematics. 2021;35(2):1418-46. doi: 10.1137/20m1313301. PubMed PMID: WOS:000674142200039.

687. Bian N, Yuan Y, Li Y, Liu M, Li X. Low-Intensity Pulsed Ultrasound Stimulation Inhibits Cortical Spreading Depression. Cerebral Cortex. 2021;31(8):3872-80. doi: 10.1093/cercor/bhab055. PubMed PMID: WOS:000674446400021.

688. Campos ACP, Berzuino MB, Barbosa GR, Freire HMRC, Lopes PS, Assis DV, et al. Motor Cortex Stimulation Reversed Hypernociception, Increased Serotonin in Raphe Neurons, and Caused Inhibition of Spinal Astrocytes in a Parkinson's Disease Rat

Model. Cells. 2021;10(5). doi: 10.3390/cells10051158. PubMed PMID: WOS:000654652600001.

689. Chen Z, Liu J, Zhao X, Zheng Z, Bai L, Yang D, et al. Effects of Electroacupuncture on Consciousness Disorders Based on Transcranial Magnetic Stimulation: Study with Mismatch Negativity. Chinese Journal of Rehabilitation Theory and Practice. 2021;27(1):102-6. PubMed PMID: CSCD:6900230.

690. Claassen J, Akbari Y, Alexander S, Bader MK, Bell K, Bleck TP, et al. Proceedings of the First Curing Coma Campaign NIH Symposium: Challenging the Future of Research for Coma and Disorders of Consciousness. Neurocritical Care. 2021;35(SUPPL 1):4-23. doi: 10.1007/s12028-021-01260-x. PubMed PMID: WOS:000671346400002.

691. Daood U, Balasankar MP, Ibrahim MA, Pichika MR, Mak K-K, Fawzy AS. PLGA nanoparticles loaded with quaternary ammonium silane and riboflavin for potential applications in adhesive dentistry. International Journal of Adhesion and Adhesives. 2021;105. doi: 10.1016/j.ijadhadh.2020.102797. PubMed PMID: WOS:000611906600006.

692. Darmani G, Nieminen JO, Bergmann TO, Ramezanpour H, Ziemann U. A degraded state of consciousness in healthy awake humans? Brain Stimulation. 2021;14(3):710-2. doi:

10.1016/j.brs.2021.04.012. PubMed PMID: WOS:000654712000033.

693. Di Natale C, De Rosa D, Profeta M, Jamaledin R, Attanasio A, Lagreca E, et al. Design of biodegradable bi-compartmental microneedles for the stabilization and the controlled release of the labile molecule collagenase for skin healthcare. Journal of Materials Chemistry B. 2021;9(2):392-403. doi: 10.1039/d0tb02279a. PubMed PMID: WOS:000609867400016.

694. Diep D, Lam ACL, Ko G. A Review of the Evidence and Current Applications of Portable Translingual Neurostimulation Technology. Neuromodulation. 2021;24(8):1377-87. doi: 10.1111/ner.13260. PubMed PMID: WOS:000566476900001.

695. Economidou SN, Uddin MJ, Marques MJ, Douroumis D, Sow WT, Li H, et al. A novel 3D printed hollow microneedle microelectromechanical system for controlled, personalized transdermal drug delivery. Additive Manufacturing. 2021;38. doi: 10.1016/j.addma.2020.101815. PubMed PMID: WOS:000617202300043.

696. Edlow BL, Sanz LRD, Polizzotto L, Pouratian N, Rolston JD, Snider SB, et al. Therapies to Restore Consciousness in Patients with Severe Brain Injuries: A Gap Analysis and Future Directions. Neurocritical Care. 2021;35(SUPPL 1):68-85. doi:

10.1007/s12028-021-01227-y. PubMed PMID: WOS:000671346400007.

697. Eilam-Stock T, George A, Charvet L. Telerehabilitation of Cognitive Functioning with Combined Transcranial Direct Current Stimulation and Computerized Adaptive Cognitive Training in Traumatic Brain Injury: A Case Report. Neurology. 2021;96(15). PubMed PMID: WOS:000729283604283.

698. Feng YH, Zhang XP, Li WX, Guo XD. Stability and Diffusion Properties of Insulin in Dissolvable Microneedles: A Multiscale Simulation Study. Langmuir. 2021;37(30):9244-52. doi: 10.1021/acs.langmuir.1c01434. PubMed PMID: WOS:000683367100038.

699. Fonseca DFS, Vilela C, Pinto RJB, Bastos V, Oliveira H, Catarino J, et al. Bacterial nanocellulose-hyaluronic acid microneedle patches for skin applications: In vitro and in vivo evaluation. Materials Science & Engineering C-Materials for Biological Applications. 2021;118. doi: 10.1016/j.msec.2020.111350. PubMed PMID: WOS:000600850700004.

700. Frohlich J, Toker D, Monti MM. Consciousness among delta waves: a paradox? Brain. 2021;144:2257-77. doi: 10.1093/brain/awab095. PubMed PMID: WOS:000710930500015.

701. Gazaille C, Sicot M, Akiki M, Lautram N, Dupont A, Saulnier P, et al. Characterization of Biological Material Adsorption to the Surface of Nanoparticles without a Prior Separation Step: a Case Study of Glioblastoma-Targeting Peptide and Lipid Nanocapsules. Pharmaceutical Research. 2021;38(4):681-91. doi: 10.1007/s11095-021-03034-8. PubMed PMID: WOS:000637660100002.

702. Guo Y, Liu C, Yang Y, Li Y, Shan Q, Xie J, et al. Effect of long-lasting repeated high-definition transcranial direct current stimulation on consciousness recovery in minimally conscious state patients. Journal of Third Military Medical University. 2021;43(15):1454-9. PubMed PMID: CSCD:7015498.

703. Han K, Dang H, Zhang H. Efficacy of Repetitive Transcranial Magnetic Stimulation on Cognitive Dysfunction after Traumatic Brain Injury: AMeta-analysis. Chinese Journal of Rehabilitation Theory and Practice. 2021;27(11):1282-90. PubMed PMID: CSCD:7111816.

704. Johnstone A, Grigoras I, Petitet P, Capitao LP, Stagg CJ. A single, clinically relevant dose of the GABA(B) agonist baclofen impairs visuomotor learning. Journal of Physiology-London. 2021;599(1):307-22. doi: 10.1113/jp280378. PubMed PMID: WOS:000584821600001.

705. Li D, Hu D, Xu H, Patra HK, Liu X, Zhou Z, et al. Progress and perspective of microneedle system for anti-cancer drug delivery. Biomaterials. 2021;264. doi: 10.1016/j.biomaterials.2020.120410. PubMed PMID: WOS:000587813600012.
706. Li S, Xia D, Prausnitz MR. Efficient Drug Delivery into Skin Using a Biphasic Dissolvable Microneedle Patch with Water-Insoluble Backing. Advanced Functional Materials. 2021;31(44). doi: 10.1002/adfm.202103359. PubMed PMID: WOS:000664463100001.
707. Lisanby S. Non-invasive brain stimulation and psychiatric symptoms in neurological disorders. Journal of the Neurological Sciences. 2021;429. doi: 10.1016/j.jns.2021.117962. PubMed PMID: WOS:000713637300325.
708. Liu L, Wang Q, Liao H, Ye J, Huang J, Li S, et al. Soluble microneedle patch with photothermal and NO-release properties for painless and precise treatment of ischemic perforator flaps. Journal of Materials Chemistry B. 2021;9(37):7725-33. doi: 10.1039/d1tb00491c. PubMed PMID: WOS:000692365400001.
709. Lo Re V, Varoli E, Hassan G, Avorio F, Mamone G, De Salvo S, et al. Role of transcranial magnetic stimulation (TMS) combined with electroencephalography (EEG) in disorders of consciousness

(DOC). Journal of the Neurological Sciences. 2021;429. doi: 10.1016/j.jns.2021.118507. PubMed PMID: WOS:000713637301325.

710. Lopez-Ramirez MA, Kupor D, Marchiori L, Soto F, Rueda R, Reynoso M, et al. Combinatorial microneedle patch with tunable release kinetics and dual fast-deep/sustained release capabilities. Journal of Materials Chemistry B. 2021;9(9):2189-99. doi: 10.1039/d1tb00141h. PubMed PMID: WOS:000627506200004.

711. Ma C. The Research Progress of Neuromodulation on Wake-promoting in Patients with Disorders of Consciousness. Journal of Sun Yat-sen University Medical Sciences. 2021;42(4):487-93. PubMed PMID: CSCD:7036612.

712. Mayorova LA, Petrova MV, Pryanikov IV, Grechko AV. CHRONIC DISORDERS OF CONSCIOUSNESS: DIAGNOSIS AND PROGNOSTICATION. Zhurnal Vysshei Nervnoi Deyatelnosti Imeni I P Pavlova. 2021;71(2):213-36. doi: 10.31857/s0044467721020076. PubMed PMID: WOS:000649024000005.

713. Mikellides G, Michael P, Schuhmann T, Sack AT. TMS-Induced Seizure during FDA-Approved Bilateral DMPFC Protocol for Treating OCD: A Case Report. Case reports in neurology. 2021;13(3):584-90. doi: 10.1159/000518999. PubMed

PMID: MEDLINE:34703446.
714. Moussi K, Haneef AA, Alsiary RA, Diallo EM, Boone MA, Abu-Araki H, et al. A Microneedles Balloon Catheter for Endovascular Drug Delivery. Advanced Materials Technologies. 2021;6(8). doi: 10.1002/admt.202100037. PubMed PMID: WOS:000655686800001.
715. Ntikas M, Hunter AM, Gallagher IJ, Di Virgilio TG. Longer Neurophysiological vs. Clinical Recovery Following Sport Concussion. Frontiers in sports and active living. 2021;3:737712-. doi: 10.3389/fspor.2021.737712. PubMed PMID: MEDLINE:34957396.
716. Omidi M, Mansouri V, Amirabad LM, Tayebi L. Impact of Lipid/Magnesium Hydroxide Hybrid Nanoparticles on the Stability of Vascular Endothelial Growth Factor-Loaded PLGA Microspheres. Acs Applied Materials & Interfaces. 2021;13(21):24370-84. doi: 10.1021/acsami.0c22140. PubMed PMID: WOS:000659315800003.
717. Onose G, Anghelescu A, Blendea CD, Ciobanu V, Daia CO, Firan FC, et al. Non-invasive, non-pharmacological/bio-technological interventions towards neurorestoration upshot after ischemic stroke, in adults-systematic, synthetic, literature review. Frontiers in Bioscience-Landmark.

2021;26(11):1204-39. doi: 10.52586/5020. PubMed PMID: WOS:000727662100021.
718. Petersen A, Soderstrom M, Saha B, Sharma P. Animal models of traumatic brain injury: a review of pathophysiology to biomarkers and treatments. Experimental Brain Research. 2021;239(10):2939-50. doi: 10.1007/s00221-021-06178-6. PubMed PMID: WOS:000679277000001.
719. Ptito A, Papa L, Gregory K, Folmer RL, Walker WC, Prabhakaran V, et al. A Prospective, Multicenter Study to Assess the Safety and Efficacy of Translingual Neurostimulation Plus Physical Therapy for the Treatment of a Chronic Balance Deficit Due to Mild-to-Moderate Traumatic Brain Injury. Neuromodulation. 2021;24(8):1412-21. doi: 10.1111/ner.13159. PubMed PMID: WOS:000529223000001.
720. Qiu W, Bouakaz A, Konofagou EE, Zheng H. Ultrasound for the Brain: A Review of Physical and Engineering Principles, and Clinical Applications. Ieee Transactions on Ultrasonics Ferroelectrics and Frequency Control. 2021;68(1):6-20. doi: 10.1109/tuffc.2020.3019932. PubMed PMID: WOS:000602706700002.
721. Rao J, Hu G, Yang W, Yu J, Xue C, Tian L, et al. Recovery of aphemia with pure word dumbness after treatment with repetitive

transcranial magnetic stimulation: A case report. Journal of Neurolinguistics. 2021;59. doi: 10.1016/j.jneuroling.2021.100991. PubMed PMID: WOS:000656680400012.

722. Requena MB, Permana AD, Vollet-Filho JD, Gonzalez-Vazquez P, Garcia MR, Goncalves De Faria CM, et al. Dissolving microneedles containing aminolevulinic acid improves protoporphyrinIXdistribution. Journal of Biophotonics. 2021;14(1). doi: 10.1002/jbio.202000128. PubMed PMID: WOS:000575099600001.

723. Ruan L, Song G, Zhang X, Liu T, Sun Y, Zhu J, et al. Transdermal delivery of multifunctional CaO2@Mn-PDA nanoformulations by microneedles for NIR-induced synergistic therapy against skin melanoma. Biomaterials Science. 2021;9(20):6830-41. doi: 10.1039/d1bm01117k. PubMed PMID: WOS:000693154800001.

724. San Luciano M, Robichaux-Viehoever A, Dodenhoff KA, Gittings ML, Viser AC, Racine CA, et al. Thalamic deep brain stimulation for acquired dystonia in children and young adults: a phase 1 clinical trial. Journal of Neurosurgery-Pediatrics. 2021;27(2):203-12. doi: 10.3171/2020.7.Peds20348. PubMed PMID: WOS:000615948400004.

725. Schmidt J, Brown KE, Feldman SJ, Babul S, Zwicker JG,

Boyd LA. Evidence of altered interhemispheric communication after pediatric concussion. Brain Injury. 2021;35(10):1143-61. doi: 10.1080/02699052.2021.1929485. PubMed PMID: WOS:000684719800001.

726. Seeni RZ, Zheng M, Lio DCS, Wiraja C, Yusoff MFBM, Koh WTY, et al. Targeted Delivery of Anesthetic Agents to Bone Tissues using Conductive Microneedles Enhanced Iontophoresis for Painless Dental Anesthesia. Advanced Functional Materials. 2021;31(47). doi: 10.1002/adfm.202105686. PubMed PMID: WOS:000686916800001.

727. Siddappaji KK, Gopal S. Molecular mechanisms in Alzheimer's disease and the impact of physical exercise with advancements in therapeutic approaches. AIMS neuroscience. 2021;8(3):357-89. doi: 10.3934/Neuroscience.2021020. PubMed PMID: MEDLINE:34183987.

728. Song C, Gao C, Zhao J, Wang Z. Construction of long-circulation EpCAM targeted drug delivery system and its application in the diagnosis and treatment of breast cancer. Journal of Biomaterials Applications. 2021;35(8):947-57. doi: 10.1177/0885328220965135. PubMed PMID: WOS:000622799000005.

729. Song F, Gao H, Li D, Petrov AV, Petrov VV, Wen D, et al.

Low intensity focused ultrasound responsive microcapsules for non-ablative ultrafast intracellular release of small molecules. Journal of Materials Chemistry B. 2021;9(10). doi: 10.1039/d0tb02788j. PubMed PMID: WOS:000629733200003.

730. Spindler LRB, Luppi AI, Adapa RM, Craig MM, Coppola P, Peattie ARD, et al. Dopaminergic brainstem disconnection is common to pharmacological and pathological consciousness. Proceedings of the National Academy of Sciences of the United States of America. 2021;118(30). doi: 10.1073/pnas.2026289118. PubMed PMID: WOS:000685039000004.

731. Stavropoulos I, Selway R, Hasegawa H, Hughes E, Rittey C, Jimenez-Jimenez D, et al. Low frequency centromedian thalamic nuclei deep brain stimulation for the treatment of super refractory status epilepticus: A case report and a review of the literature. Brain Stimulation. 2021;14(2):226-9. doi: 10.1016/j.brs.2020.12.013. PubMed PMID: WOS:000632697000004.

732. Su Y, Zhang X, Lei L, Liu B, Wu S, Shen J. Tumor Microenvironment-Activatable Cyclic Cascade Reaction to Reinforce Multimodal Combination Therapy by Destroying the Extracellular Matrix. Acs Applied Materials & Interfaces. 2021;13(11):12960-71. doi: 10.1021/acsami.1c02011. PubMed

PMID: WOS:000634759500033.
733. Tennant JR, Adams MS, Brown KE, Staines WR. Long-term effects of concussion on relevancy-based modulation of somatosensory-evoked potentials. Clinical Neurophysiology. 2021;132(10):2431-9. doi: 10.1016/j.clinph.2021.06.027. PubMed PMID: WOS:000704914800013.
734. Vaz AR, Vizinha D, Morais H, Colaco AR, Loch-Neckel G, Barbosa M, et al. Overexpression of miR-124 in Motor Neurons Plays a Key Role in ALS Pathological Processes. International Journal of Molecular Sciences. 2021;22(11). doi: 10.3390/ijms22116128. PubMed PMID: WOS:000660210700001.
735. Wang J, Zhu Jn, Ma X, Guo X, Huang D, Zheng Y. Application progress of low-intensity ultrasound in the treatment of neurological diseases. Journal of Ultrasound in Medicine. 2021;23(3):220-2. PubMed PMID: CSCD:6940599.
736. Wang Y, Bai Y, Xia X, Niu Z, Yang Y, He J, et al. Comparison of synchrosqueezing transform to alternative methods for time-frequency analysis of TMS-evoked EEG oscillations. Biomedical Signal Processing and Control. 2021;70. doi: 10.1016/j.bspc.2021.102975. PubMed PMID: WOS:000696963000002.
737. Wen T, Lin Z, Zhao Y, Zhou Y, Niu B, Shi C, et al.

Bioresponsive Nanoarchitectonics-Integrated Microneedles for Amplified Chemo-Photodynamic Therapy against Acne Vulgaris. Acs Applied Materials & Interfaces. 2021;13(41):48433-48. doi: 10.1021/acsami.1c15673. PubMed PMID: WOS:000710924900010.

738. Won-Seok K, Nam-Jong P. Safety Review for Clinical Application of Repetitive Transcranial Magnetic Stimulation. Brain & NeuroRehabilitation. 2021;14(1):1-8. doi: 10.12786/bn.2021.14.e6. PubMed PMID: KJD:ART002700635.

739. Xin Z, Abe Y, Liu S, Tanaka KF, Hosomi K, Saitoh Y, et al. Direct Impact of Motor Cortical Stimulation on the Blood Oxygen-level Dependent Response in Rats. Magnetic Resonance in Medical Sciences. 2021;20(1):83-90. doi: 10.2463/mrms.mp.2019-0156. PubMed PMID: WOS:000628817600011.

740. Yan Q, Weng J, Shen S, Wang Y, Fang M, Zheng G, et al. Finite Element Analysis for Biodegradable Dissolving Microneedle Materials on Skin Puncture and Mechanical Performance Evaluation. Polymers. 2021;13(18). doi: 10.3390/polym13183043. PubMed PMID: WOS:000701147700001.

741. Yang D, Chen M, Sun Y, Jin Y, Lu C, Pan X, et al. Microneedle-mediated transdermal drug delivery for treating

diverse skin diseases. Acta Biomaterialia. 2021;121:119-33. doi: 10.1016/j.actbio.2020.12.004. PubMed PMID: WOS:000614529200007.

742. Yang Y, Xu L, Jiang D, Chen BZ, Luo R, Liu Z, et al. Self-Powered Controllable Transdermal Drug Delivery System. Advanced Functional Materials. 2021;31(36). doi: 10.1002/adfm.202104092. PubMed PMID: WOS:000665868700001.

743. Yao S, Chi J, Wang Y, Zhao Y, Luo Y, Wang Y. Zn-MOF Encapsulated Antibacterial and Degradable Microneedles Array for Promoting Wound Healing. Advanced Healthcare Materials. 2021;10(12). doi: 10.1002/adhm.202100056. PubMed PMID: WOS:000646236000001.

744. Yasen AL, Eick GN, Sterner KN, Christie AD. Motor Cortex Function in APOE4 Carriers and Noncarriers. Journal of Clinical Neurophysiology. 2021;38(6):553-7. doi: 10.1097/wnp.0000000000000738. PubMed PMID: WOS:000756261600018.

745. Yu W, Li X, Huang Y, Chen Y, Gao Q, Wang Y, et al. Build an implanted "arsenal": detachable microneedles for NIR-triggered cancer photothermo-chemotherapy. Biomaterials Science. 2021;9(13):4737-45. doi: 10.1039/d1bm00520k. PubMed PMID:

WOS:000653853500001.
746. Yuan A, Xia F, Bian Q, Wu H, Gu Y, Wang T, et al. Ceria Nanozyme-Integrated Microneedles Reshape the Perifollicular Microenvironment for Androgenetic Alopecia Treatment. Acs Nano. 2021;15(8):13759-69. doi: 10.1021/acsnano.1c05272. PubMed PMID: WOS:000693105500108.
747. Zardkhoshoui AM, Ameri B, Davarani SSH. alpha-MnS@Co3S4 hollow nanospheres assembled from nanosheets for hybrid supercapacitors. Chemical Engineering Journal. 2021;422. doi: 10.1016/j.cej.2021.129953. PubMed PMID: WOS:000669703700002.
748. Zare MR, Khorram M, Barzegar S, Sarkari B, Asgari Q, Ahadian S, et al. Dissolvable carboxymethyl cellulose/polyvinylpyrrolidone microneedle arrays for transdermal delivery of Amphotericin B to treat cutaneous leishmaniasis. International Journal of Biological Macromolecules. 2021;182:1310-21. doi: 10.1016/j.ijbiomac.2021.05.075. PubMed PMID: WOS:000669497600014.
749. Zhang J, Kucyi A, Raya J, Nielsen AN, Nomi JS, Damoiseaux JS, et al. What have we really learned from functional connectivity in clinical populations? Neuroimage. 2021;242. doi: 10.1016/j.neuroimage.2021.118466. PubMed PMID:

WOS:000693332400010.

750. Zhang P, Ilagan R, Bai Y, Zhang X, Deng Y, Ding Y. Editorial: Plasticity and Reconstruction of Neural Network in Brain Injury. Frontiers in Cellular Neuroscience. 2021;15. doi: 10.3389/fncel.2021.710499. PubMed PMID: WOS:000669842700001.

751. Zhang Y, Wu M, Tan D, Liu Q, Xia R, Chen M, et al. A dissolving and glucose-responsive insulin-releasing microneedle patch for type 1 diabetes therapy. Journal of Materials Chemistry B. 2021;9(3). doi: 10.1039/d0tb02133d. PubMed PMID: WOS:000612856700008.

752. Zigmantovich AS, Oknina LB, Kopachka MM, Masherow EL, Alexandrova EV. Functional Wavelet-Connectivity in Resting State Reflects Consciousness Recovery in Patients with Severe Traumatic Brain Injury. Fiziologiya cheloveka. 2021;47(2):22-31. doi: 10.31857/s0131164621020144. PubMed PMID: RSCI:44810855.

753. Aloi D, Lafanechere M, Jalali R, Miall RC, Fernandez-Espejo D. tDCS Combined with Passive Mobilization Increases Brain Activity During Command-Following; a Proposed Intervention to Increase Behavioral Responsiveness in Prolonged Disorders of Consciousness. Brain Injury. 2022;36:130-. PubMed PMID:

WOS:000761085700258.
754. Borders JC, Grande AA, Troche MS. Statistical Power and Swallowing Rehabilitation Research: Current Landscape and Next Steps. Dysphagia. 2022. doi: 10.1007/s00455-022-10428-2. PubMed PMID: WOS:000762171900002.
755. Chang Y, Venton BJ. Dual-Channel Electrochemical Measurements Reveal Rapid Adenosine is Localized in Brain Slices. Acs Chemical Neuroscience. 2022;13(4):477-85. doi: 10.1021/acschemneuro.1c00679. PubMed PMID: WOS:000756681000005.
756. Garcia-Pallero M, Cardona D, Rueda-Ruzafa L, Rodriguez-Arrastia M, Roman P. Central nervous system stimulation therapies in phantom limb pain: a systematic review of clinical trials. Neural Regeneration Research. 2022;17(1):59-64. doi: 10.4103/1673-5374.314288. PubMed PMID: WOS:000661581500010.
757. Jang SH, Byun DH. A Review of Studies on the Role of Diffusion Tensor Magnetic Resonance Imaging Tractography in the Evaluation of the Fronto-Subcortical Circuit in Patients with Akinetic Mutism. Medical Science Monitor. 2022;28. doi: 10.12659/msm.936251. PubMed PMID: WOS:000758334200001.
758. Krieger D, Shepard P, Soose R, Puccio A, Beers S, Schneider

W, et al. MEG-Derived Symptom-Sensitive Biomarkers with Long-Term Test-Retest Reliability. Diagnostics. 2022;12(1). doi: 10.3390/diagnostics12010084. PubMed PMID: WOS:000758893600001.

759. Nousia A, Martzoukou M, Liampas I, Siokas V, Bakirtzis C, Nasios G, et al. The Effectiveness of Non-Invasive Brain Stimulation Alone or Combined with Cognitive Training on the Cognitive Performance of Patients With Traumatic Brain Injury: A Systematic Review. Archives of Clinical Neuropsychology. 2022;37(2):497-512. doi: 10.1093/arclin/acab047. PubMed PMID: WOS:000755899200001.

760. Penezic A, Gasparovic B, Cuculic V, Strmecki S, Djakovac T, Mlakar M. Dissolved Trace Metals and Organic Matter Distribution in the Northern Adriatic, an Increasingly Oligotrophic Shallow Sea. Water. 2022;14(3). doi: 10.3390/w14030349. PubMed PMID: WOS:000755544900001.

761. Porcaro C, Nemirovsky IE, Riganello F, Mansour Z, Cerasa A, Tonin P, et al. Diagnostic Developments in Differentiating Unresponsive Wakefulness Syndrome and the Minimally Conscious State. Frontiers in Neurology. 2022;12. doi: 10.3389/fneur.2021.778951. PubMed PMID: WOS:000752778200001.

762. Xiong M, Qin Y, Chai B, Yan J, Fan G, Xu F, et al. Unveiling the role of Mn-Cd-S solid solution and MnS in MnxCd1-xS photocatalysts and decorating with CoP nanoplates for enhanced photocatalytic H-2 evolution. Chemical Engineering Journal. 2022;428. doi: 10.1016/j.cej.2021.131069. PubMed PMID: WOS:000711644200002.

(4) Scopus (934 studies)

1. Sugita M, Sugita K, Furukawa T, Abe H. Studies on the Transport Mechanism of Aminoacids in the Renal Tubules: Part I Studies on the Mechanism of Aminoaciduria from the Analytical Standpoint of Titration Curve. JAPANESE CIRCULATION JOURNAL. 1967;31(3):405-14. doi: 10.1253/jcj.31.405.
2. French AP, Schmid AC, Ingalls E. Single case study: Transcendental meditation, altered reality testing, and behavioral change: A case report. Journal of Nervous and Mental Disease. 1975;161(1):55-8. doi: 10.1097/00005053-197507000-00007.
3. Singh AN, Saxena B, Gent M, Nelson HL. Maprotiline (Ludiomil, Ciba 34,276 BA) and imipramine in depressed outpatients: a double blind clinical study. Current Therapeutic Research - Clinical and Experimental. 1976;19(4):451-62.
4. Ide K, Harano K, Totoki T. Clinico-experimental studies on total spinal block with special reference to EEG findings. Japanese Journal of Anesthesiology. 1977;26(9):1029-40.
5. Mauguière F, Courjon J. The origins of short-latency somatosensory evoked potentials in humans. Annals of Neurology. 1981;9(6):607-11. doi: 10.1002/ana.410090616.
6. Bollini C, De Maria R, Trento L. Posttraumatic epilepsy: Notes on 2 groups of patients studied before and after the

introduction of computer tomography. Bollettino - Lega Italiana contro l'Epilessia. 1982;No. 39:76-7.
7. D'Alessandro R, Tinuper P, Ferrara R. Comparison between clinicaland computer tomographic findings in calculation of risk of posttraumatic epilepsy. Bollettino - Lega Italiana contro l'Epilessia. 1982;No. 39:69-70.
8. Gambi D, Colangelo U, Di Paolo A. Posttraumatic epilepsy: Correlations between EEG and computer tomography in cases of blunt trauma. Bollettino - Lega Italiana contro l'Epilessia. 1982;No. 39:68.
9. Moilanen A, Pitkanen M. Panoramic zonography in the radiographic diagnosis of facial and related structures. A clinical study. Rontgen-Blatter. 1984;37(2):95-9.
10. Adsett DB, Fitz CR, Hill A. HYPOXIC-ISCHAEMIC CEREBRAL INJURY IN THE TERM NEWBORN: CORRÉLATION OF CT FINDINGS WITH NEUROLOGICAL OUTCOME. Developmental Medicine & Child Neurology. 1985;27(2):155-60. doi: 10.1111/j.1469-8749.1985.tb03764.x.
11. Boureux C. Echocardiography in investigation of syncopes. Lyon Mediterranee Medical. 1985;21(14):10117-20.
12. Drechsler F. Short latency sep to median nerve stimulation: Recording methods, origin of components and clinical application.

Electromyography and Clinical Neurophysiology. 1985;25(2-3):115-34.

13. Drexler HG, Gignac SM, Minowada J. Subsets of normal mononuclear cells in the peripheral blocd defined by monoclonal antibodies. Immunological Investigations. 1985;14(4):315-21. doi: 10.3109/08820138509022666.

14. Frank Y, Zimmerman R, Leeds NMD. NEUROLOGICAL MANIFESTATIONS IN ABUSED CHILDREN WHO HAVE BÉEN SHAKEN. Developmental Medicine & Child Neurology. 1985;27(3):312-6. doi: 10.1111/j.1469-8749.1985.tb04541.x.

15. Noetzel MJ. Theophylline Neurotoxicity Resulting in Significant Unilateral Brain-damage. Developmental Medicine & Child Neurology. 1985;27(2):242-5. doi: 10.1111/j.1469-8749.1985.tb03775.x.

16. Scandiffio T, Contuzzi V, Tesler U, Tantalo L. Pulsed Doppler transmitral flow in a case of myxoma of the left atrium. Giornale italiano di cardiologia. 1985;15(9):921-4.

17. Amici A, Castellucci G, Bragetti P, Poletti C, Santucci A, Troianello G, et al. Cranial injuries in childhood. Experience in 500 cases. Pediatria Medica e Chirurgica. 1986;8(4):555-8.

18. Kirkpatrick JS, Gower DJ, Chauvenet A, Kelly DL. Subgaleal Hematoma in a Child, without Skull Fracture. Developmental

Medicine & Child Neurology. 1986;28(4):511-4. doi: 10.1111/j.1469-8749.1986.tb14291.x.

19. Matsuo Y, Sagawa K, Morita M, Minowada J, Yokoyama MM. Establishment of a hairy cell leukemia cell line carrying Tac antigen and phagocytic activity with B-cell characteristics. Journal of the National Cancer Institute. 1986;76(2):207-16.

20. Tanaka H, Shimoda T, Uchida K, Suzuki T, Ishikawa E. IMMUNOHISTOCHEMICAL STUDY ON THE DISTRIBUTION AND SIGNIFICANCE OF MONONUCLEAR CELLS IN HUMAN BREAST CARCINOMA. Pathology International. 1986;36(10):1455-68. doi: 10.1111/j.1440-1827.1986.tb02817.x.

21. Theodore WH, Holmes MD, Dorwart RH, Porter RJ, Chiro GD, Sato S, et al. Complex Partial Seizures: Cerebral Structure and Cerebral Function. Epilepsia. 1986;27(5):576-82. doi: 10.1111/j.1528-1157.1986.tb03587.x.

22. Borromei A, Caramelli R, Cipriani G, Giancola LC, Guerra L, Lozito A. Neurotraumatology and post-traumatic epilepsy: Prophylaxis, treatment and long-term follow-up. Minerva Medica. 1987;78(22):1687-705.

23. Martins IP, Ferro JM. Acquired Conduction Aphasia in a Child. Developmental Medicine & Child Neurology. 1987;29(4):532-6. doi: 10.1111/j.1469-8749.1987.tb02514.x.

24. Momma F, Sabin HI, Symon L, Branston NM. Clinical evidence supporting a subthalamic origin of the P15 wave of the somatosensory evoked potentials to median nerve stimulation. Electroencephalography and Clinical Neurophysiology. 1987;67(2):134-9. doi: 10.1016/0013-4694(87)90035-6.
25. Stain C, Stockinger H, Scharf M, Jager U, Gossinger H, Lechner K, et al. Human blood basophils display a unique phenotype including activation linked membrane structures. Blood. 1987;70(6):1872-9. doi: 10.1182/blood.v70.6.1872.bloodjournal7061872.
26. Bensaid J, Gueret P, Blanc P, Virot P, Doumeix JJ. Barlow's disease and risk for life. Archives des Maladies du Coeur et des Vaisseaux. 1988;81(2):213-8.
27. Hayashi M, Saisho S, Suzuki H, Shimozawa K, Iwakawa Y. Sleep disturbances in children with congenital and acquired hypothyroidism. No To Hattatsu. 1988;20(4):294-300.
28. Kedar E, Or R, Naparstek E, Zeira E, Slavin S. Preliminary characterization of functional residual host-type I lymphocytes following conditioning for allogeneic HLA-matched bone marrow transplantation (BMT). Bone Marrow Transplantation. 1988;3(2):129-40.
29. MacKay-Lyons M. Low-load, prolonged stretch in treatment

of elbow flexion contractures secondary to head trauma: A case report. Physical Therapy. 1989;69(4):292-6. doi: 10.1093/ptj/69.4.292.

30. Pierluisi GJ, Terndrup TE. Influence of topical anesthesia on the sedation of pediatric emergency department patients with lacerations. Pediatric Emergency Care. 1989;5(4):211-5. doi: 10.1097/00006565-198912000-00002.

31. Riffel B, Stohr M, Graser W, Trost E, Baumgartner H. Early outcome prediction in patients with severe head injury by Glasgow Coma Score and evoked potentials. Anaesthesist. 1989;38(2):51-8.

32. Zeitlhofer J, Steiner M, Zadrobilek E, Hausl E, Sporn P, Asenbaum S, et al. Evoked potentials in the course and prognosis of patients after severe head trauma. Anaesthesist. 1989;38(1):10-5.

33. Greene SA, Jefferson IG, Baum JD. Cerebral Oedema Complicating Diabetic Ketoacidosis. Developmental Medicine & Child Neurology. 1990;32(7):633-8. doi: 10.1111/j.1469-8749.1990.tb08548.x.

34. Yamaguchi S, Knight RT. Gating of somatosensory input by human prefrontal cortex. Brain Research. 1990;521(1-2):281-8. doi: 10.1016/0006-8993(90)91553-S.

35. Jouvet M, Albarede JL, Lubin S, Meyrignac C. Norepinephrine and cerebral aging. Encephale. 1991;17(3):187-95.

36. Thompson NM, Ewing-Cobbs L, Fletcher JM, Miner ME, Levin HS. Left Unilateral Neglect in a Preschool Child. Developmental Medicine & Child Neurology. 1991;33(7):640-4. doi: 10.1111/j.1469-8749.1991.tb14935.x.

37. Werba A, Weinstabl C, Petricek W, Plainer B, Spiss CK. Vecuronium prevents increases in intracranial pressure during routine tracheobronchial suctioning in neurosurgical patients. Anaesthesist. 1991;40(6):328-31.

38. West JNW, Townend JN, Davies P, Sheridan JJ, Stallard TJ, Davies MK, et al. Effect of unrestricted activity on accuracy of ambulatory blood pressure measurement. Hypertension. 1991;18(5):593-7. doi: 10.1161/01.HYP.18.5.593.

39. Beretta L, Citerio G, Dell'Acqua A, Napolitano L, Piccoli S, Torri G. Diffuse axonal injury (DAI): diagnostic assessment with nuclear magnetic resonance (NMR). Minerva Anestesiologica. 1992;58(10):795-6.

40. Cameron PD, Wallace SJ, Munro J. HERPES SIMPLEX VIRUS ENCEPHALITIS: PROBLEMS IN DIAGNOSIS. Developmental Medicine & Child Neurology. 1992;34(2):134-40. doi: 10.1111/j.1469-8749.1992.tb14979.x.

41. Franssen H, Stegeman DF, Wieneke GH. Attenuation of dipoles modelled from SEP due to a lacunar infarct or altered

stimulus rate. Brain Topography. 1992;5(2):159-63. doi: 10.1007/BF01129044.

42. Jaspert A, Kotterba S, Tegenthoff M, Malin JP. Transcranial magnetic stimulation of the facial nerve in patients with traumatic brain lesions. EEG-EMG Zeitschrift fur Elektroenzephalographie Elektromyographie und Verwandte Gebiete. 1992;23(1):37-42.

43. Kutalek SP, Schuster MM, Hessen SE, Sheppard R, Maquilan M, Nydegger C. Clinical Experience with a New Multiprogrammable Dual Chamber Pacemaker. Pacing and Clinical Electrophysiology. 1992;15(11):1830-5. doi: 10.1111/j.1540-8159.1992.tb02977.x.

44. Liepert J, Tegenthoff M. Transcranial magnetic stimulation in patients with single epileptic seizures. Nervenarzt. 1992;63(8):492-4.

45. Osenbach RK. Neurosurgical applications of somatosensory and motor evoked potentials. Neurosurgery Quarterly. 1992;2(4):280-95.

46. Shutov AA, Sherman MA. Psychovegetative syndrome in the residual period of modern 'light' military craniocerebral injury. Zhurnal Nevropatologii i Psikhiatrii Imeni SSKorsakova. 1992;92(5-12):13-5.

47. Ten Harkel ADJ, Baisch F, Karemaker JM. Increased

orthostatic blood pressure variability after prolonged head-down tilt. Acta Physiologica Scandinavica, Supplement. 1992;144(604):89-99.

48. Van Lieshout EJ, Van Lieshout JJ, Krol J, Simons M, Karemaker JM. Assessment of cardiovascular reflexes is of limited value in predicting maximal + Gz-tolerance. Aviation Space and Environmental Medicine. 1992;63(1):21-6.

49. Bastos PG, Sun X, Wagner DP, Wu AW, Knaus WA. Glasgow Coma Scale score in the evaluation of outcome in the intensive care unit: Findings from the Acute Physiology and Chronic Health Evaluation III study. Critical Care Medicine. 1993;21(10):1459-65. doi: 10.1097/00003246-199310000-00012.

50. Ghiazza G, Piovano P, Ferraro G, D'Arco M, Pellegrini V. A case of hemiballismus developed after a hyperosmolar coma in an elderly diabetic woman. European Review for Medical and Pharmacological Sciences. 1993;15(2):83-6.

51. Gray PH, Tudehope DI, Masel JP, Burns YR, Mohay HA, O'Callaghan MJ, et al. PERINATAL HYPOXIC-ISCHAEMIC BRAIN INJURY: PRDICTION OF OUTCOME. Developmental Medicine & Child Neurology. 1993;35(11):965-73. doi: 10.1111/j.1469-8749.1993.tb11578.x.

52. Krägeloh-Mann I, Grodd W, Schöning M, Marquard K,

Nägele T, Ruitenbeek W. PROTON SPECTROSCOPY IN FIVE PATIENTS WITH LEIGH'S DISEASE AND MITOCHONDRIAL ENZYME DEFICIENCY. Developmental Medicine & Child Neurology. 1993;35(9):769-76. doi: 10.1111/j.1469-8749.1993.tb11728.x.

53. Masur H, Papke K, Oberwittler C. Suppression of visual perception by transcranial magnetic stimulation - experimental findings in healthy subjects and patients with optic neuritis. Electroencephalography and Clinical Neurophysiology. 1993;86(4):259-67. doi: 10.1016/0013-4694(93)90107-7.

54. Takeshita M, Masuda Y, Sumiyoshi Y, Ohshima K, Kikuchi M, Kimura N, et al. Clinicopathologic, enzyme and hisotchemical studies of centrocytic (mantle cell) lymphoma: Comparison with other types of low-grade B cell lymphoma bases on the updated Kiel classification. Pathology International. 1993;43(5):244-52. doi: 10.1111/j.1440-1827.1993.tb01139.x.

55. Veccia S, Giacometti A, Cinti B, Fiorentini A, Petroni S, Scalise G. Adenosine deaminase activity in cerebrospinal fluid from AIDS patients with cerebral lesions. Giornale di Malattie Infettive e Parassitarie. 1993;45(7-8):804-5.

56. Boehme MWJ, Werle E, Kommerell B, Raeth U. Serum levels of adhesion molecules and thrombomodulin as indicators of

vascular injury in severe Plasmodium falciparum malaria. The Clinical Investigator. 1994;72(8):598-603. doi: 10.1007/BF00227452.
57. Canivet JL, Gustad K, Leclercq P, Damas P, Lamy M. Massive ketonuria during sedation with propofol in a 12 year old girl with severe head trauma. Acta Anaesthesiologica Belgica. 1994;45(1):19-22.
58. Dichter MA. Emerging Insights Into Mechanisms of Epilepsy: Implications for New Antiepileptic Drug Development. Epilepsia. 1994;35:S51-S7. doi: 10.1111/j.1528-1157.1994.tb05956.x.
59. Goddard MJ, Dean BZ, King JC. 1. Basic science, acute pain, and neuropathic pain. Archives of Physical Medicine and Rehabilitation. 1994;75(5 SUPPL.):S4-S8. doi: 10.1016/0003-9993(94)90371-9.
60. Muizelaar JP. Clinical trials with dismutec(TM) (pegorgotein; polyethylene glycol- conjugated superoxide dismutase; PEG-SOD) in the treatment of severe closed head injury. Advances in Experimental Medicine and Biology1994. p. 389-400.
61. Probert TCS, Wiesenfeld D, Reade PC. Temporomandibular pain dysfunction disorder resulting from road traffic accidents - An Australian study. International Journal of Oral and Maxillofacial Surgery. 1994;23(6 PART 1):338-41. doi:

10.1016/S0901-5027(05)80049-9.

62. von Giesen HJ, Roick H, Benecke R. Inhibitory actions of motor cortex following unilateral brain lesions as studied by magnetic brain stimulation. Experimental Brain Research. 1994;99(1):84-96. doi: 10.1007/BF00241414.

63. Buchner H, Waberski TD, Fuchs M, Wischmann HA, Beckmann R, Rienäcker A. Origin of P16 median nerve SEP component identified by dipole source analysis - subthalamic or within the thalamo-cortical radiation? Experimental Brain Research. 1995;104(3):511-8. doi: 10.1007/BF00231985.

64. Eken P, de Vries LS, van der Graaf Y, Meiners LC, van Nieuwenhuizen O. HAEMORRHAGIC-ISCHAEMIC LESIONS OF THE NEONATAL BRAIN: CORRELATION BETWEEN CEREBRAL VISUAL IMPAIRMENT, NEURODEVELOP-MENTAL OUTCOME AND MRI IN INFANCY. Developmental Medicine & Child Neurology. 1995;37(1):41-55. doi: 10.1111/j.1469-8749.1995.tb11931.x.

65. Ernst AA, Marvez-Valls E, Nick TG, Weiss SJ. LAT (lidocaine-adrenaline-tetracaine) versus TAC (tetracaine-adrenaline-cocaine) for topical anesthesia in face and scalp lacerations. American Journal of Emergency Medicine. 1995;13(2):151-4. doi: 10.1016/0735-6757(95)90082-9.

66. Gambaretti R, Orlandi S, Pozzoni R. Diagnosis of meniscal and ligament lesions of the knee. Comparison between clinical and instrumental examination. Minerva Ortopedica e Traumatologica. 1995;46(3):105-7.

67. Gessner JE, Radeke HH, Uciechowski P, Resch K, Schmidt RE. Activation-dependent expression of Fc receptors for IgG on glomerular mesangial cells. Immunitat und Infektion. 1995;23(2):67-9.

68. Kaneko M. Prognostic evaluation of patients with severe head injury by motor evoked potentials induced by transcranial magnetic stimulation - Combined analysis with brainstem auditory evoked potentials. Brain and Nerve. 1995;47(5):491-6.

69. Kapelushnik J, Or R, Filon D, Nagler A, Cividalli G, Aker M, et al. Analysis of β-globin mutations shows stable mixed chimerism in patients with thalassemia after bone marrow transplantation. Blood. 1995;86(8):3241-6. doi: 10.1182/blood.v86.8.3241.bloodjournal8683241.

70. Karnath HO. Transcutaneous electrical stimulation and vibration of neck muscles in neglect. Experimental Brain Research. 1995;105(2):321-4. doi: 10.1007/BF00240969.

71. Morioka T, Yamamoto T, Mizushima A, Tombimatsu S, Shigeto H, Hasuo K, et al. Comparison of

magnetoencephalography, functional MRI, and motor evoked potentials in the localization of the sensory-motor cortex. Neurological Research. 1995;17(5):361-7. doi: 10.1080/01616412.1995.11740343.

72. Naka D, Funahashi K, Ogura M, Kuwata T, Nakai M, Nakai K, et al. Identification of motor area by transcranial magnetic stimulation using an 'eight-figure' coil in patients with motor paralysis. Neurological Surgery. 1995;23(1):27-34.

73. Naruse S, Takaya K, Yoshioka H. Metabolic and Functional Magnetic Resonance Imaging of the Brain : Clinical Application to Pediatric Brain Diseases. No To Hattatsu. 1995;27(2):138-45. doi: 10.11251/ojjscn1969.27.138.

74. Schrader M, Schrader V. Reliability of magnetic stimulation in the diagnosis of idiopathic peripheral facial palsies. Revue de Laryngologie Otologie Rhinologie. 1995;116(2):123-7.

75. Snell GI, Salamonsen RF, Bergin P, Esmore DS, Khan S, Williams TJ. Inhaled nitric oxide used as a bridge to heart-lung transplantation in a patient with end-stage pulmonary hypertension. American Journal of Respiratory and Critical Care Medicine. 1995;151(4):1263-6. doi: 10.1164/ajrccm.151.4.7697264.

76. Tassi C, Gallai V, Rossi A. Serum creatine kinase BB isoenzyme in subjects evaluated by trancranial magnetic

stimulation: Preliminary observations. Clinical Chemistry and Enzymology Communications. 1995;7(1):29-34.

77. Terao Y, Hayashi H, Shimizu T, Tanabe H, Hanajima R, Ugawa Y. Altered motor cortical excitability to magnetic stimulation in a patient with a lesion in globus pallidus. Journal of the Neurological Sciences. 1995;129(2):175-8. doi: 10.1016/0022-510X(94)00273-Q.

78. Vallar G, Rusconi ML, Barozzi S, Bernardini B, Ovadia D, Papagno C, et al. Improvement of left visuo-spatial hemineglect by left-sided transcutaneous electrical stimulation. Neuropsychologia. 1995;33(1):73-82. doi: 10.1016/0028-3932(94)00088-7.

79. Walker-Batson D, Smith P, Curtis S, Unwin H, Greenlee R. Amphetamine paired with physical therapy accelerates motor recovery after stroke; further evidence. Stroke. 1995;26(12):2254-9. doi: 10.1161/01.STR.26.12.2254.

80. Werhahn KJ, Classen J, Benecke R. The silent period induced by transcranial magnetic stimulation in muscles supplied by cranial nerves: Normal data and changes in patients. Journal of Neurology, Neurosurgery and Psychiatry. 1995;59(6):586-96. doi: 10.1136/jnnp.59.6.586.

81. Worley G, Hoffman JM, Paine SS, Kalman SL, Claerhout SJ, Boyko OB, et al. 18-FLUORODEOXYGLUCOSE POSITRON

EMISSION TOMOGRAPHY IN CHILDREN AND ADOLESCENTS WITH TRAUMATIC BRAIN INJURY. Developmental Medicine & Child Neurology. 1995;37(3):213-20. doi: 10.1111/j.1469-8749.1995.tb11994.x.

82. Karmel BZ, Gardner JM, Freedland RL. Arousal-modulated attention at four months as a function of intrauterine cocaine exposure and central nervous system injury. Journal of Pediatric Psychology. 1996;21(6):821-32. doi: 10.1093/jpepsy/21.6.821.

83. Kawamura T, Nakasato N, Seki K, Kanno A, Fujita S, Fujiwara S, et al. Neuromagnetic evidence of pre- and post-central cortical sources of somatosensory evoked responses. Electroencephalography and Clinical Neurophysiology - Evoked Potentials. 1996;100(1):44-50. doi: 10.1016/0168-5597(95)00217-0.

84. König F, Löble M, Wolfersdorf M. Depressive stupor - Malignant neuroleptic syndrome - Serotonin syndrome. A case report illustrating a problem in differential diagnosis. Nervenarzt. 1996;67(5):407-12.

85. Liepert J, Kotterba S, Tegenthoff M, Malin JP. Central fatigue assessed by transcranial magnetic stimulation. Muscle and Nerve. 1996;19(11):1429-34. doi: 10.1002/(SICI)1097-4598(199611)19:11<1429::AID-MUS7>3.0.C

O;2-E.
86. Pascual-Castroviejo I. Plasticidad cerebral. Revista de Neurologia. 1996;24(135):1361-6.
87. Urban PP, Hopf HC, Connemann B, Hundemer HP, Koehler J. The course of cortico-hypoglossal projections in the human brainstem. Functional testing using transcranial magnetic stimulation. Brain. 1996;119(3):1031-8. doi: 10.1093/brain/119.3.1031.
88. Verhagen HJM, Heijnen-Snyder GJ, Pronk A, Vroom TM, Van Vroonhoven TJMV, Eikelboom BC, et al. Thrombomodulin activity on mesothelial cells: Perspectives for mesothelial cells as an alternative for endothelial cells for cell seeding on vascular grafts. British Journal of Haematology. 1996;95(3):542-9. doi: 10.1046/j.1365-2141.1996.d01-1935.x.
89. Yuya H, Nagata K, Takanashi Y, Satoh Y, Watahiki Y, Hirata Y, et al. Scalp topography of SEP late components in patients with supra-tentorial lesions. Brain Topography. 1996;8(3):333-6. doi: 10.1007/BF01184794.
90. Charbel FT, Hoffman WE, Misra M, Hannigan K, Ausman JI. Cerebral interstitial tissue oxygen tension, pH, HCO3, CO2. Surgical Neurology. 1997;48(4):414-7. doi: 10.1016/S0090-3019(96)00473-9.

91. Davis CH, Fardanesh L, Rubner D, Wanlass RL, McDonald CM. Profiles of functional recovery in fifty traumatically brain-injured patients after acute rehabilitation. American Journal of Physical Medicine and Rehabilitation. 1997;76(3):213-8. doi: 10.1097/00002060-199705000-00011.

92. Deal BJ, Strieper M, Scagliotti D, Hulse E, Auld D, Campbell R, et al. The medical therapy of cardioinhibitory syncope in pediatric patients. PACE - Pacing and Clinical Electrophysiology. 1997;20(7):1759-61. doi: 10.1111/j.1540-8159.1997.tb03563.x.

93. Goodwin GM. Neuropsychological and neuroimaging evidence for the involvement of the frontal lobes in depression. Journal of Psychopharmacology. 1997;11(2):115-22. doi: 10.1177/026988119701100204.

94. Hirashima F, Yokota T. Influence of peripheral nerve stimulation on human motor cortical excitability in patients with ventrolateral thalamic lesion. Archives of Neurology. 1997;54(5):619-24. doi: 10.1001/archneur.1997.00550170091019.

95. Izumi SI, Takase M, Arita M, Masakado Y, Kimura A, Chino N. Transcranial magnetic stimulation-induced changes in EEG and responses recorded from the scalp of healthy humans. Electroencephalography and Clinical Neurophysiology. 1997;103(2):319-22. doi: 10.1016/S0013-4694(97)00007-2.

96. Lechner C, Behse F, Meisenzohl E, Brunkhorst F. Recovery time of Median nerve SEP following cerebral hypoxia in man. EEG-EMG Zeitschrift fur Elektroenzephalographie Elektromyographie und Verwandte Gebiete. 1997;28(3):136-40.
97. Pruna A, Peyri N, Berard M, Boffa MC. Thrombomodulin is synthesized by human mesangial cells. Kidney International. 1997;51(3):687-93. doi: 10.1038/ki.1997.99.
98. Taggart DP, Bhattacharya K, Meston N, Standing SJ, Kay JDS, Pillai R, et al. Serum S-100 protein concentration after cardiac surgery: A randomized trial of arterial line filtration. European Journal of Cardio-thoracic Surgery. 1997;11(4):645-9. doi: 10.1016/S1010-7940(96)01103-7.
99. Tobita M, Kuroiwa Y, Komiyama A, Hasegawa O, Miyasaki H, Sugiyama M, et al. Use of cortical SEP for the prediction of neurological prognosis after cardiopulmonary resuscitation. Brain and Nerve. 1997;49(2):131-6.
100. Urban PP, Hopf HC, Fleischer S, Zorowka PG, Müller-Forell W. Impaired cortico-bulbar tract function in dysarthria due to hemispheric stroke. Functional testing using transcranial magnetic stimulation. Brain. 1997;120(6):1077-84. doi: 10.1093/brain/120.6.1077.
101. Ustarroz JT, De Goicoechea FPNL, Ferreras AA, Arbonies AC.

Ecological validity of neuropsychological tests in executive processes: A report of a cese. Anales de Psiquiatria. 1997;13(8):339-44.
102. Valeriani M, Restuccia D, Di Lazzaro V, Le Pera D, Scerrati M, Tonali P, et al. Giant central N20-P22 with normal area 3b N20-P20: An argument in favour of an area 3a generator of early median nerve cortical SEPs? Electroencephalography and Clinical Neurophysiology - Evoked Potentials. 1997;104(1):60-7. doi: 10.1016/S0168-5597(96)96660-5.
103. Woiciechowsky C. Neuropsychological and neurophysiological consequences of partial callosotomy. Journal of Neurosurgical Sciences. 1997;41(1):75-80.
104. Bertolasi L, Priori A, Tinazzi M, Bertasi V, Rothwell JC. Inhibitory action of forearm flexor muscle afferents on corticospinal outputs to antagonist muscles in humans. Journal of Physiology. 1998;511(3):947-56. doi: 10.1111/j.1469-7793.1998.947bg.x.
105. Caramia MD, Telera S, Palmieri MG, Wilson-Jones M, Scalise A, Iani C, et al. Ipsilateral motor activation in patients with cerebral gliomas. Neurology. 1998;51(1):196-202. doi: 10.1212/WNL.51.1.196.
106. Carrillo Esper R, Aguilar Ramirez MDP, Gargallo Hernandez

JJ, Duran Vega HC, Alvarado Martinez C, Nunez Monroy FN, et al. Osborn wave in subarachnoid hemorrhage. Medicina Interna de Mexico. 1998;14(6):287-90.

107. Donaghy S, Wass PJ. Interrater reliability of the functional assessment measure in a brain injury rehabilitation program. Archives of Physical Medicine and Rehabilitation. 1998;79(10):1231-6. doi: 10.1016/S0003-9993(98)90267-2.

108. Hughes JR, Daaboul Y, Fino JJ, Shaw GL. The “Mozart Effect” on Epileptiform Activity. Clinical EEG and Neuroscience. 1998;29(3):109-19. doi: 10.1177/155005949802900301.

109. Ilmoniemi RJ. Neuromagnetic imaging and stimulation. Clinical Physiology. 1998;18(3):308.

110. Juneja G, Czyrny JJ, Linn RT. Admission balance and outcomes of patients admitted for acute inpatient rehabilitation. American Journal of Physical Medicine and Rehabilitation. 1998;77(5):388-93. doi: 10.1097/00002060-199809000-00005.

111. Kobayashi M, Ohira T, Ishihara M, Kawase T, Takase M. Transcranial magnetic stimulation of the oculomotor and abducens nerves: Determining the site of excitation in the cat. Journal of Clinical Neurophysiology. 1998;15(4):358-63. doi: 10.1097/00004691-199807000-00008.

112. Maccabee PJ, Nagarajan SS, Amassian VE, Durand DM,

Szabo AZ, Ahad AB, et al. Influence of pulse sequence, polarity and amplitude on magnetic stimulation of human and porcine peripheral nerve. Journal of Physiology. 1998;513(2):571-85. doi: 10.1111/j.1469-7793.1998.571bb.x.

113. Mosqueda-Garcia R, Fernandez-Violante R, Tank J, Snell M, Cunningham G, Furlan R. Yohimbine in neurally mediated syncope: Pathophysiological implications. Journal of Clinical Investigation. 1998;102(10):1824-30. doi: 10.1172/JCI3050.

114. Pérennou DA, Amblard B, Leblond C, Pélissier J. Biased postural vertical in humans with hemispheric cerebral lesions. Neuroscience Letters. 1998;252(2):75-8. doi: 10.1016/S0304-3940(98)00501-1.

115. Porst H. Transurethral application of Alprostadil with MUSE(TM) (Medicated Urethral System for Erection). Actual overview and personal experiences. Urologe - Ausgabe A. 1998;37(4):410-6. doi: 10.1007/s001200050199.

116. Schluter ND, Rushworth MFS, Passingham RE, Mills KR. Temporary interference in human lateral premotor cortex suggests dominance for the selection of movements. A study using transcranial magnetic stimulation. Brain. 1998;121(5):785-99. doi: 10.1093/brain/121.5.785.

117. Triggs WJ, Tesar DW, Young MS. Ipsilateral motor control in

pathological left-handedness. Neurocase. 1998;4(1):65-9. doi: 10.1093/neucas/4.1.65.

118. Zuhosky JP, Dugan SA, Young JL, Bode RK, Kelly JP. A retrospective review of the incidence and rehabilitation outcome of concomitant traumatic brain injury and ligamentous knee injury. Archives of Physical Medicine and Rehabilitation. 1998;79(7):805-10. doi: 10.1016/S0003-9993(98)90360-4.

119. Béliveau A, Leclerc S, Rouleau M, Guérin SL. Multiple cloning sites from mammalian expression vectors interfere with gene promoter studies in vitro. European Journal of Biochemistry. 1999;261(2):585-90. doi: 10.1046/j.1432-1327.1999.00362.x.

120. Bischoff SC, Sellge G, Schwengberg S, Lorentz A, Manns MP. Stem cell factor-dependent survival, proliferation and enhanced releasability of purified mature mast cells isolated from human intestinal tissue. International Archives of Allergy and Immunology. 1999;118(2-4):104-7. doi: 10.1159/000024041.

121. Chevion S, Or R, Berry EM. The antioxidant status of patients subjected to total body irradiation. Biochemistry and Molecular Biology International. 1999;47(6):1019-27.

122. Dunai J, Bennett K, Fotiades A, Kritikos A, Castiello U. Modulation of unilateral neglect as a function of direction of object motion. NeuroReport. 1999;10(5):1041-7. doi:

10.1097/00001756-199904060-00027.

123. Escada P, Pratas Vital J, Capucho C, Lima C, Madeira Da Silva J, Silva Penha R. Meningoencephalic herniation into the middle ear. Revue de Laryngologie Otologie Rhinologie. 1999;120(1):47-52.

124. Godefroy O, Cabaret M, Petit-Chenal V, Pruvo JP, Rousseaux M. Control functions of the frontal lobes. Modularity of the central-supervisory system? Cortex. 1999;35(1):1-20. doi: 10.1016/S0010-9452(08)70782-2.

125. Goldstein LB. Pharmacological approach to functional reorganization: The role of norepinephrine. Revue Neurologique. 1999;155(9):731-6.

126. Gusmão SS, Pittella JEH. Congestive brain swelling in victims of fatal road traffic accidents: Frequency and association with other head injury lesions. Arquivos de Neuro-Psiquiatria. 1999;57(4):965-70. doi: 10.1590/s0004-282x1999000600011.

127. Halper AS, Cherney LR, Cichowski K, Zhang M. Dysphagia after head trauma: The effect of cognitive-communicative impairments on functional outcomes. Journal of Head Trauma Rehabilitation. 1999;14(5):486-96. doi: 10.1097/00001199-199910000-00009.

128. James JD, Hauer-Jensen M. Effects of fixative and fixation

time for quantitative computerized image analysis of immunohistochemical staining. Journal of Histotechnology. 1999;22(2):109-11. doi: 10.1179/his.1999.22.2.109.

129. Kelly KM, Littlefield TR, Pomatto JK, Manwaring KH, Beals SP. Cranial growth unrestricted during treatment of deformational plagiocephaly. Pediatric Neurosurgery. 1999;30(4):193-9. doi: 10.1159/000028794.

130. Kikushima S, Kobayashi Y, Nakagawa H, Katagiri T. Triggering mechanism for neurally mediated syncope induced by head-up tilt test: Role of catecholamines and response to propranolol. Journal of the American College of Cardiology. 1999;33(2):350-7. doi: 10.1016/S0735-1097(98)00567-1.

131. Maegaki Y, Maeoka Y, Ishii S, Eda I, Ohtagaki A, Kitahara TJ, et al. Central motor reorganization in cerebral palsy patients with bilateral cerebral lesions. Pediatric Research. 1999;45(4):559-67. doi: 10.1203/00006450-199904010-00016.

132. Marchand-Pauvert V, Mazevet D, Pierrot-Deseilligny E, Pol S, Pradat-Diehl P. Handedness-related asymmetry in transmission in a system of human cervical premotoneurones. Experimental Brain Research. 1999;125(3):323-34. doi: 10.1007/s002210050688.

133. Marmarou A, Nichols J, Burgess J, Newell D, Troha J, Burnham D, et al. Effects of the bradykinin antagonist

Bradycor(TM) (Deltibant, CP-1027) in severe traumatic brain injury: Results of a multi-center, randomized, placebo-controlled trial. Journal of Neurotrauma. 1999;16(6):431-44. doi: 10.1089/neu.1999.16.431.

134. Marret S, Bonnier C, Raymackers JM, Delpech A, Evrard P, Gressens P. Glycine antagonist and NO°synthase inhibitor protect the developing mouse brain against neonatal excitotoxic lesions. Pediatric Research. 1999;45(3):337-42. doi: 10.1203/00006450-199903000-00008.

135. Nezu A, Kimura S, Takeshita S, Tanaka M. Functional recovery in hemiplegic cerebral palsy: Ipsilateral electromyographic responses to focal transcranial magnetic stimulation. Brain and Development. 1999;21(3):162-5. doi: 10.1016/S0387-7604(98)00094-1.

136. Nico D. Effectiveness of sensory stimulation on tactile extinction. Experimental Brain Research. 1999;127(1):75-82. doi: 10.1007/s002210050775.

137. Nudo RJ, Friel KM. Cortical plasticity after stroke: Implications for rehabilitation. Revue Neurologique. 1999;155(9):713-7.

138. Oliveri M, Rossini PM, Traversa R, Cicinelli P, Filippi MM, Pasqualetti P, et al. Left frontal transcranial magnetic stimulation

reduces contralesional extinction in patients with unilateral right brain damage. Brain. 1999;122(9):1731-9. doi: 10.1093/brain/122.9.1731.

139. Piovan E, Zampieri PG, Alessandrini F, Rosta L, Maschio A, Beltramello A. Xenon-CT in skull-head injury. Rivista Medica. 1999;5(1-2):53-9.

140. Rogers RD, Everitt BJ, Baldacchino A, Blackshaw AJ, Swainson R, Wynne K, et al. Dissociable deficits in the decision-making cognition of chronic amphetamine abusers, opiate abusers, patients with focal damage to prefrontal cortex, and tryptophan-depleted normal volunteersEvidence for monoaminergic mechanisms. Neuropsychopharmacology. 1999;20(4):322-39. doi: 10.1016/S0893-133X(98)00091-8.

141. Rona R, Piantanida S, Bolis C, Cuccia S Protti E, Cantoni A, Rivolta G. (Caso clinico). Acta Anaesthesiologica Italica / Anaesthesia and Intensive Care in Italy. 1999;50(2):129-32.

142. Al-Wahadneh AM, Khriesat IA, Kuda EH. Adverse reactions of intravenous immunoglobulin. Saudi Medical Journal. 2000;21(10):953-6.

143. Balbi P, Trojano L, Ragno M, Perretti A, Santoro L. Patterns of motor control reorganization in a patient with mirror movements. Clinical Neurophysiology. 2000;111(2):318-25. doi:

10.1016/S1388-2457(99)00239-4.
144. Colletti V, Fiorino FG, Carner M, Giarbini N, Sacchetto L, Cumer G. Advantages of the retrosigmoid approach in auditory brain stem implantation. Skull Base Surgery. 2000;10(4):165-70.
145. Couper FJ, Logan BK. Determination of γ-hydroxybutyrate (GHB) in biological specimens by gas chromatography-mass spectrometry. Journal of Analytical Toxicology. 2000;24(1):1-7. doi: 10.1093/jat/24.1.1.
146. Fitzpatrick SM, Rothman DL. A James S. McDonnell Foundation Workshop: Meeting report: Transcranial magnetic stimulation and studies of human cognition. Journal of Cognitive Neuroscience. 2000;12(4):704-9. doi: 10.1162/089892900562327.
147. Frackowiak RS. Forthergillian Lecture. Imaging human brain function. Transactions of the Medical Society of London. 2000;117:53-63.
148. Gray DS, Burnham RS. Preliminary outcome analysis of a long-term rehabilitation program for severe acquired brain injury. Archives of Physical Medicine and Rehabilitation. 2000;81(11):1447-56. doi: 10.1053/apmr.2000.16343.
149. Hobson JA, Pace-Schott EF, Stickgold R. Dreaming and the brain: Toward a cognitive neuroscience of conscious states. Behavioral and Brain Sciences. 2000;23(6):793-842. doi:

10.1017/S0140525X00003976.

150. Holloway V, Gadian DG, Vargha-Khadem F, Porter DA, Boyd SG, Connelly A. The reorganization of sensorimotor function in children after hemispherectomy. A functional MRI and somatosensory evoked potential study. Brain. 2000;123(12):2432-44. doi: 10.1093/brain/123.12.2432.

151. Karataş M, Dilek A, Erkan H, Yavuz N, Sözay S, Akman N. Functional outcome in stroke patients with atrial fibrillation. Archives of Physical Medicine and Rehabilitation. 2000;81(8):1025-9. doi: 10.1053/apmr.2000.6981.

152. Kasahata N, Shiota JI, Kawamura M, Miyazawa Y, Sugita K. Pure sensory stroke due to a pontine lesion: A study of clinical symptomatology and somatosensory evoked potential findings. Brain and Nerve. 2000;52(10):913-7.

153. Kastrup O, Leonhardt G, Kurthen M, Hufnagel A. Cortical motor reorganization following early brain damage and hemispherectomy demonstrated by transcranial magnetic stimulation. Clinical Neurophysiology. 2000;111(8):1346-52. doi: 10.1016/S1388-2457(00)00339-4.

154. Kessels RPC, D'Alfonso AAL, Postma A, De Haan EHF. Spatial working memory performance after high-frequency repetitive transcranial magnetic stimulation of the left and right

posterior parietal cortex in humans. Neuroscience Letters. 2000;287(1):68-70. doi: 10.1016/S0304-3940(00)01146-0.
155. Laine M. The learning brain. Brain and Language. 2000;71(1):132-4. doi: 10.1006/brln.1999.2232.
156. Oliveri M, Rossini PM, Cicinelli P, Traversa R, Pasqualetti P, Filippi MM, et al. Neurophysiological evaluation of tactile space perception deficits through transcranial magnetic stimulation. Brain Research Protocols. 2000;5(1):25-9. doi: 10.1016/S1385-299X(99)00055-0.
157. Philbert MA, Billingsley ML, Reuhl KR. Mechanisms of Injury in the Central Nervous System. Toxicologic Pathology. 2000;28(1):43-53. doi: 10.1177/019262330002800107.
158. Röricht S, Meyer BU. Residual function in motor cortex contralateral to amputated hand. Neurology. 2000;54(4):984-7. doi: 10.1212/WNL.54.4.984.
159. Shimizu T, Nariai T, Maehara T, Hino T, Komori T, Shimizu H, et al. Enhanced motor cortical excitability in the unaffected hemisphere after hemispherectomy. NeuroReport. 2000;11(14):3077-84. doi: 10.1097/00001756-200009280-00009.
160. Stowe CD, Lee KR, Storgion SA, Phelps SJ. Altered phenytoin pharmacokinetics in children with severe, acute traumatic brain injury. Journal of Clinical Pharmacology.

2000;40(12 II):1452-61. doi: 10.1177/00912700004001216.

161. Walker-Batson D. Use of pharmacotherapy in the treatment of aphasia. Brain and Language. 2000;71(1):252-4. doi: 10.1006/brln.1999.2262.

162. Wood EM, Colton E, Yomtovian RA, Currie LM, Connor J, Anderson JM. Prevention of monocyte adhesion and inflammatory cytokine production during blood platelet storage: An in vitro model with implications for transfusion practice. Journal of Biomedical Materials Research. 2000;51(2):147-54. doi: 10.1002/(SICI)1097-4636(200008)51:2<147::AID-JBM1>3.0.CO;2-Y.

163. Anthony DC, Blond D, Dempster R, Perry VH. Chemokine targets in acute brain injury and disease. Progress in Brain Research. 2001;132:507-24. doi: 10.1016/S0079-6123(01)32099-X.

164. Bezzi P, Domercq M, Vesce S, Volterra A. Neuron-astrocyte cross-talk during synaptic transmission: Physiological and neuropathological implications. Progress in Brain Research2001. p. 255-65.

165. Evans RG, Ventura S, Dampney RA, Ludbrook J. Neural mechanisms in the cardiovascular responses to acute central hypovolaemia. Clinical and Experimental Pharmacology and

Physiology. 2001;28(5-6):479-87. doi: 10.1046/j.1440-1681.2001.03473.x.

166. Gorji A, Scheller D, Straub H, Tegtmeier F, Köhling R, Höhling JM, et al. Spreading depression in human neocortical slices. Brain Research. 2001;906(1-2):74-83. doi: 10.1016/S0006-8993(01)02557-4.

167. Hallett M. Functional reorganization after lesions of the human brain: Studies with transcranial magnetic stimulation. Revue Neurologique. 2001;157(8-9 I):822-6.

168. Herrmann CS, Knight RT. Mechanisms of human attention: Event-related potentials and oscillations. Neuroscience and Biobehavioral Reviews. 2001;25(6):465-76. doi: 10.1016/S0149-7634(01)00027-6.

169. Hilgetag CC, Théoret H, Pascual-Leone A. Enhanced visual spatial attention ipsilateral to rTMS-induced 'virtual lesions' of human parietal cortex. Nature Neuroscience. 2001;4(9):953-7. doi: 10.1038/nn0901-953.

170. Kakkar A. Blast injury ear in a low intensity conflict. Indian Journal of Otolaryngology and Head and Neck Surgery. 2001;53(3):221-4.

171. Manthey D, Heck S, Engert S, Behl C. Estrogen induces a rapid secretion of amyloid β precursor protein via the

mitogen-activated protein kinase pathway. European Journal of Biochemistry. 2001;268(15):4285-91. doi: 10.1046/j.1432-1327.2001.02346.x.

172. McAllister TW, Sparling MB, Flashman LA, Guerin SJ, Mamourian AC, Saykin AJ. Differential working memory load effects after mild traumatic brain injury. NeuroImage. 2001;14(5):1004-12. doi: 10.1006/nimg.2001.0899.

173. Oliveri M, Bisiach E, Brighina F, Piazza A, La Bua V, Buffa D, et al. rTMS of the unaffected hemisphere transiently reduces contralesional visuospatial heineglect. Neurology. 2001;57(7):1338-40. doi: 10.1212/WNL.57.7.1338.

174. Piovan E, Cordopatri D, Cristofori L, Gambin R, Vivenza C, Beltramello A. Cerebral blood flow measured by Xenon-CT in severe head injury: Preliminary findings. Rivista di Neuroradiologia. 2001;14(4):395-405. doi: 10.1177/197140090101400404.

175. Piovan E, Grazioli A, Cordopatri D, Gambin R, Cristofori L, Beltramello A, et al. Xenon-CT in severe head injury. Rivista di Neuroradiologia. 2001;14(SUPPL. 3):175-82. doi: 10.1177/19714009010140s333.

176. Plewnia C, Bartels M, Cohen L, Gerloff C. Noradrenergic modulation of human cortex excitability by the presynaptic

α2-antagonist yohimbine. Neuroscience Letters. 2001;307(1):41-4. doi: 10.1016/S0304-3940(01)01921-8.

177. Ramos Rincón JM, García Ruipérez D, Aliaga Matas F, Lozano Cutillas MC, Llanos Llanos R, Herrero Huerta F. Specific mortality rates by DRG and main diagnosis according to CIE-9-MC at a level-II hospital. Anales de medicina interna (Madrid, Spain : 1984). 2001;18(10):510-6.

178. Restuccia D, Valeriani M, Barba C, Le Pera D, Capecci M, Filippini V, et al. Functional changes of the primary somatosensory cortex in patients with unilateral cerebellar lesions. Brain. 2001;124(4):757-68. doi: 10.1093/brain/124.4.757.

179. Ridding MC, Taylor JL. Mechanisms of motor-evoked potential facilitation following prolonged dual peripheral and central stimulation in humans. Journal of Physiology. 2001;537(2):623-31. doi: 10.1111/j.1469-7793.2001.00623.x.

180. Rollnik JD. Electrical stimulation of the sural nerve partially compensates effects of central fatigue. Acta Neurochirurgica, Supplement2001. p. 113-5.

181. Röricht S, Machetanz J, Irlbacher K, Niehaus L, Biemer E, Meyer BU. Reorganization of human motor cortex after hand replantation. Annals of Neurology. 2001;50(2):240-9. doi: 10.1002/ana.1091.

182. Satsou D, Kouakam C, Vaksmann G, Klug D, Kacet S, Rey C. Value of an implantable ECG monitor for the diagnosis of syncope due to arrhythmias in childhood. Archives des Maladies du Coeur et des Vaisseaux. 2001;94(5):527-30.
183. Sonde L, Bronge L, Kalimo H, Viitanen M. Can the site of brain lesion predict improved motor function after Low-TENS treatment on the post-stroke paretic arm? Clinical Rehabilitation. 2001;15(5):545-51. doi: 10.1191/026921501680425261.
184. Tran TD, Kubota M, Takeshita K, Yanagisawa M, Sakakihara Y. Varicella-associated acute necrotizing encephalopathy with a good prognosis. Brain and Development. 2001;23(1):54-7. doi: 10.1016/S0387-7604(00)00199-6.
185. Vecchio F, Menegazzo E, Salimbeni CF. A diagnostic-therapeutic protocol for head injury. Rivista di Neurobiologia. 2001;47(6):431-4.
186. Walker-Batson D, Curtis S, Natarajan R, Ford J, Dronkers N, Salmeron E, et al. A double-blind, placebo-controlled study of the use of amphetamine in the treatment of aphasia. Stroke. 2001;32(9):2093-7. doi: 10.1161/hs0901.095720.
187. Bechmann I, Nitsch R. Plasticity following lesion: Help and harm from the immune system. Restorative Neurology and Neuroscience. 2002;19(3-4):189-98.

188. Brighina F, Bisiach E, Piazza A, Oliveri M, La Bua V, Daniele O, et al. Perceptual and response bias in visuospatial neglect due to frontal and parietal repetitive transcranial magnetic stimulation in normal subjects. NeuroReport. 2002;13(18):2571-5. doi: 10.1097/00001756-200212200-00038.
189. Faella FS, Rossi M, Pagliano P, Attanasio V, Briante V, Fusco U, et al. Non post-operative spondylodiskitis. Our experience during the period 1990-2001. Infezioni in Medicina. 2002;10(3):157-62.
190. Ghika J, Vingerhoets F. Are oestrogens neuroprotective? Medecine et Hygiene. 2002;60(2390):901-4.
191. Johansen-Berg H, Rushworth MFS, Bogdanovic MD, Kischka U, Wimalaratna S, Matthews PM. The role of ipsilateral premotor cortex in hand movement after stroke. Proceedings of the National Academy of Sciences of the United States of America. 2002;99(22):14518-23. doi: 10.1073/pnas.222536799.
192. John ER. Editorial: Letter from the President. Clinical EEG Electroencephalography. 2002;33(3). doi: 10.1177/155005940203300303.
193. Katayama Y, Yamamoto T, Kobayashi K, Kasai M, Oshima H, Fukaya C. Motor cortex stimulation for phantom limb pain: Comprehensive therapy with spinal cord and thalamic stimulation.

Stereotactic and Functional Neurosurgery. 2002;77(1-4):159-62. doi: 10.1159/000064593.
194. Knecht S, Flöel A, Dräger B, Breitenstein C, Sommer J, Henningsen H, et al. Degree of language lateralization determines susceptibility to unilateral brain lesions. Nature Neuroscience. 2002;5(7):695-9. doi: 10.1038/nn868.
195. Legatt AD. Current practice of motor evoked potential monitoring: Results of a survey. Journal of Clinical Neurophysiology. 2002;19(5):454-60. doi: 10.1097/00004691-200210000-00008.
196. Lin YY, Forss N. Functional characterization of human second somatosensory cortex by magnetoencephalography. Behavioural Brain Research. 2002;135(1-2):141-5. doi: 10.1016/S0166-4328(02)00143-2.
197. Marshall JC, Fink GR, Halligan PW, Vallar G. Spatial awareness: A function of the posterior parietal lobe? Cortex. 2002;38(2):253-7. doi: 10.1016/S0010-9452(08)70654-3.
198. Meunier S, Katz R, Simonetta-Moreau M. Central nervous system lesions and segmental activity. Advances in Experimental Medicine and Biology2002. p. 309-13.
199. Montorsi F. The role of apomorphine SL in the treatment of erectile dysfunction. European Urology, Supplement.

2002;1(3):4-11. doi: 10.1016/S1569-9056(02)00008-8.

200. Münchau A, Orth M, Rothwell JC, Di Lazzaro V, Oliviero A, Profice P, et al. Intracortical inhibition is reduced in a patient with a lesion in the posterolateral thalamus. Movement Disorders. 2002;17(1):208-12. doi: 10.1002/mds.1264.

201. Phillips LL, Reeves TM. Interactive pathology following traumatic brain injury modifies hippocampal plasticity. Restorative Neurology and Neuroscience. 2002;19(3-4):213-35.

202. Price CJ, Friston KJ. Degeneracy and cognitive anatomy. Trends in Cognitive Sciences. 2002;6(10):416-21. doi: 10.1016/S1364-6613(02)01976-9.

203. Ragazzoni A, Cincotta M, Borgheresi A, Zaccara G, Ziemann U. Congenital hemiparesis: Different functional reorganization of somatosensory and motor pathways. Clinical Neurophysiology. 2002;113(8):1273-8. doi: 10.1016/S1388-2457(02)00150-5.

204. Ravaglia S, Costa A, Ratti MT, Savoldi F, Bo P, Moglia A. Cognitive impairment and central motor conduction time in chronic alcoholics. Functional Neurology. 2002;17(2):83-6.

205. Ringholm A, Fredriksson R, Poliakova N, Yan YL, Postlethwait JH, Larhammar D, et al. One melanocortin 4 and two melanocortin 5 receptors from zebrafish show remarkable conservation in structure and pharmacology. Journal of

Neurochemistry. 2002;82(1):6-18. doi: 10.1046/j.1471-4159.2002.00934.x.
206. Schiff ND, Purpura KP. Towards a neurophysiological foundation for cognitive neuromodulation through deep brain stimulation. Thalamus and Related Systems. 2002;2(1):55-69. doi: 10.1016/S1472-9288(02)00028-6.
207. Segers S, Van Sassenbroeck D, Verstraete A, Martens P. Gamma-hydroxybutyric acid: From anaesthetic to illegal recreational drug. Tijdschrift voor Geneeskunde. 2002;58(1):49-54. doi: 10.2143/tvg.58.1.1001227.
208. Sommer M, Lang N, Tergau F, Paulus W. Neuronal tissue polarization induced by repetitive transcranial magnetic stimulation? NeuroReport. 2002;13(6):809-11. doi: 10.1097/00001756-200205070-00015.
209. Wang XM, Fu HM, Long CG. The clinical research on the treatment of depression in patients with mild traumatic brain injury with repetitive transcranial magnetic stimulation. Chinese Journal of Clinical Rehabilitation. 2002;6(3).
210. Wong AMF, Tweed D, Sharpe JA. Adaptive neural mechanism for Listing's law revealed in patients with sixth nerve palsy. Investigative Ophthalmology and Visual Science. 2002;43(1):112-9.

211. Yokota H, Naoe Y, Nakabayashi M, Unemoto K, Kushimoto S, Kurokawa A, et al. Cerebral endothelial injury in severe head injury: The significance of measurements of serum thrombomodulin and the von Willebrand factor. Journal of Neurotrauma. 2002;19(9):1007-15. doi: 10.1089/089771502760341929.

212. Ziviani J, Ottenbacher KJ, Shephard K, Foreman S, Astbury W, Ireland P. Concurrent Validity of the Functional Independence Measure for Children (WeeFIM(™)) and the Pediatric Evaluation of Disabilities Inventory in Children with Developmental Disabilities and Acquired Brain Injuries. Physical and Occupational Therapy in Pediatrics. 2002;21(42038):91-101. doi: 10.1080/J006v21n02_08.

213. Abbott KC, Bernet VJ, Agodoa LY, Yuan CM. Diabetic ketoacidosis and hyperglycemic hyperosmolar syndrome after renal tranplantation in the United States. BMC Endocrine Disorders. 2003;3. doi: 10.1186/1472-6823-3-1.

214. Agudelo-Florez P, Palacio LG. Prevalence of Taenia solium antibodies in humans and in pigs in an endemic area of Colombia. Revista de Neurologia. 2003;36(8):706-9. doi: 10.33588/rn.3608.2002323.

215. Andrews, Bowyer J, Paule M, Doyle A, Todorovic V, Alves, et

al. Questions and answers: Session I: Clinical trials and beyond. Annals of the New York Academy of Sciences2003. p. 48-53.

216. Begley DJ. Understanding and circumventing the blood-brain barrier. Acta Paediatrica, International Journal of Paediatrics, Supplement. 2003;92(443):83-91. doi: 10.1111/j.1651-2227.2003.tb00226.x.

217. Brighina F, Bisiach E, Oliveri M, Piazza A, La Bua V, Daniele O, et al. 1 Hz repetitive transcranial magnetic stimulation of the unaffected hemisphere ameliorates contralesional visuospatial neglect in humans. Neuroscience Letters. 2003;336(2):131-3. doi: 10.1016/S0304-3940(02)01283-1.

218. Bütefisch CM, Netz J, Weßling M, Seitz RJ, Hömberg V. Remote changes in cortical excitability after stroke. Brain. 2003;126(2):470-81. doi: 10.1093/brain/awg044.

219. Campidelli C, Di Tommaso L, Zanetti G. Aneurysmal bone cyst of nasal cavity. A case report and review of the literature. Pathologica. 2003;95(2):103-7.

220. Coubard O, Kapoula Z, Müri R, Rivaud-Péchoux S. Effects of TMS over the right prefrontal cortex on latency of saccades and convergence. Investigative Ophthalmology and Visual Science. 2003;44(2):600-9. doi: 10.1167/iovs.02-0188.

221. Frederick RI. Review of the validity indicator profile. Journal

of Forensic Neuropsychology. 2003;2(3-4):125-45. doi: 10.1300/J151v02n03_06.

222. Frisch S, Kotz SA, Von Cramon DY, Friederici AD. Why the P600 is not just a P300: The role of the basal ganglia. Clinical Neurophysiology. 2003;114(2):336-40. doi: 10.1016/S1388-2457(02)00366-8.

223. Greig P, Lilly L, Scudamore C, Erb S, Yoshida E, Kneteman N, et al. Early steroid withdrawal after liver transplantation: The Canadian tacrolimus versus microemulsion cyclosporin a trial: 1 year follow-up. Liver Transplantation. 2003;9(6):587-95. doi: 10.1053/jlts.2003.50102.

224. He BX, Wang MD, Yan WP, Guo SW, Jiang HT, Bao G, et al. Protective effect of neuronavigation microsurgery on brain function and its accuracy in the treatment of intracranial minute occupying-space lesions. Chinese Journal of Clinical Rehabilitation. 2003;7(28):3858-9.

225. Holst B, Schwartz TW. Molecular mechanism of agonism and inverse agonism in the melanocortin receptors: Zn2+ as a structural and functional probe. Annals of the New York Academy of Sciences2003. p. 1-11.

226. Ilić TV, Korchounov A, Ziemann U. Methylphenidate facilitates and disinhibits the motor cortex in intact humans.

NeuroReport. 2003;14(5):773-6. doi: 10.1097/00001756-200304150-00023.
227. Jaspert A, Grehl H. Principles of transcranial magnetic stimulation. Neurophysiologie-Labor. 2003;25(3):121-37.
228. Kamada K, Houkin K, Takeuchi F, Ishii N, Ikeda J, Sawamura Y, et al. Visualization of the eloquent motor system by integration of MEG, functional, and anisotropic diffusion-weighted MRI in functional neuronavigation. Surgical Neurology. 2003;59(5):352-60. doi: 10.1016/S0090-3019(03)00018-1.
229. Koch G, Oliveri M, Torriero S, Caltagirone C. Underestimation of time perception after repetitive transcranial magnetic stimulation. Neurology. 2003;60(11):1844-6. doi: 10.1212/WNL.60.11.1844.
230. Kolb B. Overview of cortical plasticity and recovery from brain injury. Physical Medicine and Rehabilitation Clinics of North America. 2003;14(1 SUPPL.):S7. doi: 10.1016/S1047-9651(02)00056-6.
231. Kotz SA, Frisch S, von Cramon DY, Friederici AD. Syntactic language processing: ERP lesion data on the role of the basal ganglia. Journal of the International Neuropsychological Society. 2003;9(7):1053-60. doi: 10.1017/S1355617703970093.
232. Longhi L, Roncati Zanier E, Valeriani V, Ghisoni L, Besozzi A,

Ferrari C, et al. Brain vulnerability and its modulation. Minerva Anestesiologica. 2003;69(4):227-31.
233. Lu XR, Huang ZC, Man Y, Xia XH. Evaluation of cortex function in patients with migraine by transcranial magnetic stimulation. Chinese Journal of Clinical Rehabilitation. 2003;7(16):2298-9.
234. Miller KL. Urinary incontinence in elderly women. Primary Care Update for Ob/Gyns. 2003;10(5):242-6. doi: 10.1016/S1068-607X(03)00047-7.
235. Muramoto M, Yamazaki T, Nishimura S, Kita Y. Detailed in vitro pharmacological analysis of FK506-induced neuroprotection. Neuropharmacology. 2003;45(3):394-403. doi: 10.1016/S0028-3908(03)00168-0.
236. Nudo RJ. Adaptive plasticity in motor cortex: Implications for rehabilitation after brain injury. Journal of Rehabilitation Medicine, Supplement. 2003(41):7-10.
237. Özben T. Pathology of cerebral ischaemia: Mechanisms involved in neuronal damage. Jugoslovenska Medicinska Biokemija. 2003;22(2):85-92. doi: 10.2298/JMH0302085O.
238. Pniewski J, Chodakowska-Zebrowska M, Woźniak R, Stępień K, Stafiej A. Plasma homocysteine level and the course of ischemic stroke. Acta Neurobiologiae Experimentalis. 2003;63(2):127-30.

239. Rushworth MFS, Johansen-Berg H, Göbel SM, Devlin JT. The left parietal and premotor cortices: Motor attention and selection. NeuroImage. 2003;20(SUPPL. 1):S89-S100. doi: 10.1016/j.neuroimage.2003.09.011.

240. Shibaki H, Shibaki A. Analysis of dermatophyte flora at a private clinic in Sapporo during the period 1992 to 2001. Japanese Journal of Medical Mycology. 2003;44(3):209-16. doi: 10.3314/jjmm.44.209.

241. Štětkářová I, Veselá M, Kryštůfková O. Early detection of nervous system dysfunction in patients with systemic lupus erythematosus by evoked potentials - A case report. Ceska Revmatologie. 2003;11(2):99-103.

242. Steyvers M, Levin O, Van Baelen M, Swinnen SP. Corticospinal excitability changes following prolonged muscle tendon vibration. NeuroReport. 2003;14(15):1901-5. doi: 10.1097/00001756-200310270-00004.

243. Stürmer B, Leuthold H. Control over response priming in visuomotor processing: A lateralized event-related potential study. Experimental Brain Research. 2003;153(1):35-44. doi: 10.1007/s00221-003-1579-1.

244. Sun T. Reorganization of brain function in children after hemispherectomy. Chinese Journal of Clinical Rehabilitation.

2003;7(4):680.

245. Tomietto P, Annese V, D'Agostini S, Gremese E, Di Poi E, Ferraccioli G. Neuropsychological tests, MRI and SPECT in the evaluation of CNS involvement in SLE. Reumatismo. 2003;55(3):171-80.

246. Yang L. Mechanisms of central motor reorganization with transcranial magnetic stimulation in cerebral palsy. Chinese Journal of Clinical Rehabilitation. 2003;7(13):1938-9.

247. Bell EC, Baker GB, Poag C, Bellavance F, Khudabux J, Le Mellédo JM. Response to flumazenil in the late luteal phase and follicular phase of the menstrual cycle in healthy control females. Psychopharmacology. 2004;172(3):248-54. doi: 10.1007/s00213-003-1660-2.

248. Bueti D, Costantini M, Forster B, Aglioti SM. Uni- and cross-modal temporal modulation of tactile extinction in right brain damaged patients. Neuropsychologia. 2004;42(12):1689-96. doi: 10.1016/j.neuropsychologia.2004.04.005.

249. Cramer SC. Functional imaging in stroke recovery. Stroke. 2004;35(11 SUPPL. 1):2695-8. doi: 10.1161/01.STR.0000143326.36847.b0.

250. Deakin J, Aitken M, Robbins T, Sahakian BJ. Risk taking during decision-making in normal volunteers changes with age.

Journal of the International Neuropsychological Society. 2004;10(4):590-8. doi: 10.1017/S1355617704104104.

251. Dumas HM, Haley SM, Ludlow LH, Carey TM. Recovery of Ambulation during Inpatient Rehabilitation: Physical Therapist Prognosis for Children and Adolescents with Traumatic Brain Injury. Physical Therapy. 2004;84(3):232-42. doi: 10.1093/ptj/84.3.232.

252. Ellison A, Schindler I, Pattison LL, Milner AD. An exploration of the role of the superior temporal gyrus in visual search and spatial perception using TMS. Brain. 2004;127(10):2307-15. doi: 10.1093/brain/awh244.

253. Feydy A, Krainik A, Bussel B, Maier MA. Cortical Reorganization Allows for Motor Recovery after Crossed Cerebrocerebellar Atrophy. Journal of Neuroimaging. 2004;14(1):49-53. doi: 10.1177/1051228403259867.

254. Gorbach AM. Local alternated temperature gradients as footprints of cortical functional activation. Journal of Thermal Biology. 2004;29(7-8 SPEC. ISS.):589-98. doi: 10.1016/j.jtherbio.2004.08.072.

255. Jeong HJ, Chung HS, Kim YH, Moon BS, Sung KK, Bai SJ, et al. Differential regulation by Seogak Jihwang-Tang on cytokines production in peripheral blood mononuclear cells from the cerebral

infarction patients presenting with altered consciousness. Journal of Ethnopharmacology. 2004;94(2-3):289-94. doi: 10.1016/j.jep.2004.05.014.
256. Lafosse C, Kerckhofs E, Troch M, Santens P, Vandenbussche E. Graviceptive misperception of the postural vertical after right hemisphere damage. NeuroReport. 2004;15(5):887-91. doi: 10.1097/00001756-200404090-00031.
257. Landers M. Treatment-induced neuroplasticity following focal injury to the motor cortex. International Journal of Rehabilitation Research. 2004;27(1):1-5. doi: 10.1097/00004356-200403000-00001.
258. Lee CC, Chang WT, Fang CC, Tsai IL, Chen WJ. Sudden death caused by dissecting thoracic aortic aneurysm in a patient with autosomal dominant polycystic kidney disease. Resuscitation. 2004;63(1):93-6. doi: 10.1016/j.resuscitation.2004.03.019.
259. Lefaucheur JP, Fnelon G, Mnard-Lefaucheur I, Wendling S, Nguyen JP. Low-frequency repetitive TMS of premotor cortex can reduce painful axial spasms in generalized secondary dystonia: A pilot study of three patients. Neurophysiologie Clinique. 2004;34(3-4):141-5. doi: 10.1016/j.neucli.2004.07.003.
260. Liepert J, Hamzei F, Weiller C. Lesion-induced and training-induced brain reorganization. Restorative Neurology and

Neuroscience. 2004;22(3-4):269-77.

261. Marshall RS. Neuroimaging in stroke recovery: A position paper from the first international workshop on neuroimaging and stroke recovery. Cerebrovascular Diseases. 2004;18(3):260-7. doi: 10.1159/000080293.

262. Masuda J, Takayama E, Satoh A, Kojima-Aikawa K, Suzuki K, Matsumoto I. A novel expression vector, designated as pHisJM, for producing recombinant His-fusion proteins. Biotechnology Letters. 2004;26(20):1543-8. doi: 10.1023/B:BILE.0000045650.90384.b2.

263. Merabet L, Thut G, Murray B, Andrews J, Hsiao S, Pascual-Leone A. Feeling by sight or seeing by touch? Neuron. 2004;42(1):173-9. doi: 10.1016/S0896-6273(04)00147-3.

264. Obhi SS. Bimanual coordination: An unbalanced field of research. Motor Control. 2004;8(2):111-20. doi: 10.1123/mcj.8.2.111.

265. Rollnik JD, Schröder C, Rodríguez-Fornells A, Kurzbuch AR, Däuper J, Möller J, et al. Functional lesions and human action monitoring: Combining repetitive transcranial magnetic stimulation and event-related brain potentials. Clinical Neurophysiology. 2004;115(1):145-53. doi: 10.1016/j.clinph.2003.05.001.

266. Spence C, Pavani F, Maravita A, Holmes N. Multisensory contributions to the 3-D representation of visuotactile peripersonal

space in humans: Evidence from the crossmodal congruency task. Journal of Physiology Paris. 2004;98(1-3 SPEC. ISS.):171-89. doi: 10.1016/j.jphysparis.2004.03.008.
267. Staudt M, Gerloff C, Grodd W, Holthausen H, Niemann G, Krägeloh-Mann I. Reorganization in congenital hemiparesis acquired at different gestational ages. Annals of Neurology. 2004;56(6):854-63. doi: 10.1002/ana.20297.
268. Stinear JW, Byblow WD. Modulation of human cervical premotoneurons during bilateral voluntary contraction of upper-limb muscles. Muscle and Nerve. 2004;29(4):506-14. doi: 10.1002/mus.20003.
269. Strafella AP, Vanderwerf Y, Sadikot AF. Transcranial magnetic stimulation of the human motor cortex influences the neuronal activity of subthalamic nucleus. European Journal of Neuroscience. 2004;20(8):2245-9. doi: 10.1111/j.1460-9568.2004.03669.x.
270. Takayama K, Kawai N, Kawanishi M, Masada T, Jinnai T, Matsumoto Y, et al. Effects of Transcranial Magnetic Stimulation on Patients with Prolonged Consciousness Disturbance. Japanese Journal of Neurosurgery. 2004;13(1):32-8. doi: 10.7887/jcns.13.32.
271. Torbey MT, Geocadin R, Bhardwaj A. Brain arrest neurological outcome scale (BrANOS): Predicting mortality and severe disability following cardiac arrest. Resuscitation.

2004;63(1):55-63. doi: 10.1016/j.resuscitation.2004.03.021.

272. Ward NS, Frackowiak RSJ. Towards a new mapping of brain cortex function. Cerebrovascular Diseases. 2004;17(SUPPL. 3):35-8. doi: 10.1159/000075303.

273. Weddell RA. Subcortical modulation of spatial attention including evidence that the Sprague effect extends to man. Brain and Cognition. 2004;55(3):497-506. doi: 10.1016/j.bandc.2004.02.075.

274. White PF, Tang J, Ma H, Wender RH, Sloninsky A, Kariger R. Is the patient state analyzer with the PSArray2 a cost-effective alternative to the bispectral index monitor during the perioperative period? Anesthesia and Analgesia. 2004;99(5):1429-35. doi: 10.1213/01.ANE.0000132784.57622.CC.

275. Yago E, Duarte A, Wong T, Barceló F, Knight RT. Temporal kinetics of prefrontal modulation of the extrastriate cortex during visual attention. Cognitive, Affective and Behavioral Neuroscience. 2004;4(4):609-17. doi: 10.3758/CABN.4.4.609.

276. Young RL, Ridding MC, Morrell TL. Switching skills on by turning off part of the brain. Neurocase. 2004;10(3):215-22. doi: 10.1080/13554790490495140.

277. Abbott KC, Bernet VJ, Agodoa LY, Yuan CM. Differing manifestations of hepatitis C and tacrolimus on hospitalized

diabetes mellitus occurring after kidney transplantation. Annals of Epidemiology. 2005;15(8):558-63. doi: 10.1016/j.annepidem.2004.10.003.

278. Berkowitsch A, Greiss H, Vukajlovic D, Kuniss M, Neumann T, Zaltsberg S, et al. Usefulness of atrial fibrillation burden as a predictor for success of pulmonary vein isolation. PACE - Pacing and Clinical Electrophysiology. 2005;28(12):1292-301. doi: 10.1111/j.1540-8159.2005.00270.x.

279. Beuzeron-Mangina JH, Fotiou F. Editorial preface. International Journal of Psychophysiology. 2005;58(2-3 SPEC. ISS.):109-10. doi: 10.1016/j.ijpsycho.2005.07.002.

280. Bogousslavsky J. Artistic creativity, style and brain disorders. European Neurology. 2005;54(2):103-11. doi: 10.1159/000088645.

281. Bojinova V, Dimova P, Gergueltcheva V, Petkov R, Belopitova L, Yankov T. Peculiarities of multiple sclerosis in childhood - Frequency, clinical characteristics, diagnosis, prognosis, treatment. Pediatriya. 2005;45(4):21-5+3-4.

282. Carter OL, Pettigrew JD, Hasler F, Wallis GM, Liu GB, Hell D, et al. Modulating the rate and rhythmicity of perceptual rivalry alternations with the mixed 5-HT2A and 5-HT1A agonist psilocybin. Neuropsychopharmacology. 2005;30(6):1154-62. doi: 10.1038/sj.npp.1300621.

283. Gallegos DR, Tranel D. Positive facial affect facilitates the identification of famous faces. Brain and Language. 2005;93(3):338-48. doi: 10.1016/j.bandl.2004.11.001.

284. Hammoud DA, Endres CJ, Chander AR, Guilarte TR, Wong DF, Sacktor NC, et al. Imaging glial cell activation with [11C]-R-PK11195 in patients with AIDS. Journal of NeuroVirology. 2005;11(4):346-55. doi: 10.1080/13550280500187351.

285. Kovacic P, Cooksy A. Iminium metabolite mechanism for nicotine toxicity and addiction: Oxidative stress and electron transfer. Medical Hypotheses. 2005;64(1):104-11. doi: 10.1016/j.mehy.2004.03.048.

286. Liepert J, Restemeyer C, Münchau A, Weiller C. Motor cortex excitability after thalamic infarction. Clinical Neurophysiology. 2005;116(7):1621-7. doi: 10.1016/j.clinph.2005.03.002.

287. Lobato RD, Alen JF, Perez-Nuñez A, Alday R, Gómez PA, Pascual B, et al. Value of serial CT scanning and intracranial pressure monitoring for detecting new intracranial mass effect in severe head injury patients showing lesions type I-II in the initial CT scan. Neurocirugia. 2005;16(3):217-34. doi: 10.1016/s1130-1473(05)70405-x.

288. Manconi M, Sferraza B, Iannaccone S, Massimo A, Zucconi M, Ferini-Strambi L. Case of symptomatic propriospinal

myoclonus evolving toward acute "myoclonic status". Movement Disorders. 2005;20(12):1646-50. doi: 10.1002/mds.20645.

289. Mansur CG, Fregni F, Boggio PS, Riberto M, Gallucci-Neto J, Santos CM, et al. A sham stimulation-controlled trial of rTMS of the unaffected hemisphere in stroke patients. Neurology. 2005;64(10):1802-4. doi: 10.1212/01.WNL.0000161839.38079.92.

290. Murase N, Rothwell JC, Kaji R, Urushihara R, Nakamura K, Murayama N, et al. Subthreshold low-frequency repetitive transcranial magnetic stimulation over the premotor cortex modulates writer's cramp. Brain. 2005;128(1):104-15. doi: 10.1093/brain/awh315.

291. Naeser MA, Martin PI, Nicholas M, Baker EH, Seekins H, Kobayashi M, et al. Improved picture naming in chronic aphasia after TMS to part of right Broca's area: An open-protocol study. Brain and Language. 2005;93(1):95-105. doi: 10.1016/j.bandl.2004.08.004.

292. Pickard JD. Part II: Functional imaging, electrophysiology and mechanical intervention - Foreword. Neuropsychological Rehabilitation. 2005;15(3-4):272-5. doi: 10.1080/09602010443000641.

293. Rektorova I, Megova S, Bares M, Rektor I. Cognitive functioning after repetitive transcranial magnetic stimulation in

patients with cerebrovascular disease without dementia: A pilot study of seven patients. Journal of the Neurological Sciences. 2005;229-230:157-61. doi: 10.1016/j.jns.2004.11.021.
294. Rex E, Hapiak V, Hobson R, Smith K, Xiao H, Komuniecki R. TYRA-2 (F01E11.5): A Caenorhabditis elegans tyramine receptor expressed in the MC and NSM pharyngeal neurons. Journal of Neurochemistry. 2005;94(1):181-91. doi: 10.1111/j.1471-4159.2005.03180.x.
295. Seitz RJ, Kleiser R, Bütefisch CM. Reorganization of cerebral circuits in human brain lesion. Acta Neurochirurgica, Supplementum2005. p. 65-70.
296. Staudt M, Krägeloh-Mann I, Grodd W. Ipsilateral corticospinal pathways in congenital hemiparesis on routine magnetic resonance imaging. Pediatric Neurology. 2005;32(1):37-9. doi: 10.1016/j.pediatrneurol.2004.07.005.
297. Stefan K, Cohen LG, Duque J, Mazzocchio R, Celnik P, Sawaki L, et al. Formation of a motor memory by action observation. Journal of Neuroscience. 2005;25(41):9339-46. doi: 10.1523/JNEUROSCI.2282-05.2005.
298. Strafella AP, Ko JH, Grant J, Fraraccio M, Monchi O. Corticostriatal functional interactions in Parkinson's disease: A rTMS/[11C]raclopride PET study. European Journal of

Neuroscience. 2005;22(11):2946-52. doi: 10.1111/j.1460-9568.2005.04476.x.

299. Viveros MP, Marco EM, File SE. Endocannabinoid system and stress and anxiety responses. Pharmacology Biochemistry and Behavior. 2005;81(2 SPEC. ISS.):331-42. doi: 10.1016/j.pbb.2005.01.029.

300. Voets NL, Matthews PM. Clinical applications of functional magnetic resonance imaging. Imaging Decisions MRI. 2005;9(1):16-22. doi: 10.1111/j.1617-0830.2005.00037.x.

301. Ward NS. Plasticity and the functional reorganization of the human brain. International Journal of Psychophysiology. 2005;58(2-3 SPEC. ISS.):158-61. doi: 10.1016/j.ijpsycho.2005.02.009.

302. Yates BJ, Bronstein AM. The effects of vestibular system lesions on autonomic regulation: Observations, mechanisms, and clinical implications. Journal of Vestibular Research: Equilibrium and Orientation. 2005;15(3):119-29.

303. Chemerinski E, Levine SR. Neuropsychiatric disorders following vascular brain injury. Mount Sinai Journal of Medicine. 2006;73(7):1006-14.

304. Čumlivski R, Redl G, Strobl W, Girsch W, Krebs A, Machowetz P. Pharmacological neuromodulation of the spasticity

by using of intrathecal applied baclofen in the children. Bolest. 2006;9(2):88-98.

305. Durazzo TC, Gazdzinski S, Rothlind JC, Banys P, Meyerhoff DJ. Brain metabolite concentrations and neurocognition during short-term recovery from alcohol dependence: Preliminary evidence of the effects of concurrent chronic cigarette smoking. Alcoholism: Clinical and Experimental Research. 2006;30(3):539-51. doi: 10.1111/j.1530-0277.2006.00060.x.

306. Galat A. Involvement of some large immunophilins and their ligands in the protection and regeneration of neurons: A hypothetical mode of action. Computational Biology and Chemistry. 2006;30(5):348-59. doi: 10.1016/j.compbiolchem.2006.08.001.

307. Ichinose M, Saito M, Fujii N, Kondo N, Nishiyasu T. Modulation of the control of muscle sympathetic nerve activity during severe orthostatic stress. Journal of Physiology. 2006;576(3):947-58. doi: 10.1113/jphysiol.2006.117507.

308. Kao CD, Guo WY, Chen JT, Wu ZA, Liao KK. MR findings of decerebrate rigidity with preservation of consciousness. American Journal of Neuroradiology. 2006;27(5):1074-5.

309. Koethe D, Gerth CW, Neatby MA, Haensel A, Thies M, Schneider U, et al. Disturbances of visual information processing

in early states of psychosis and experimental delta-9-tetrahydrocannabinol altered states of consciousness. Schizophrenia Research. 2006;88(1-3):142-50. doi: 10.1016/j.schres.2006.07.023.

310. Laganaro M, Di Pietro M, Schnider A. What does recovery from anomia tell us about the underlying impairment: The case of similar anomic patterns and different recovery. Neuropsychologia. 2006;44(4):534-45. doi: 10.1016/j.neuropsychologia.2005.07.005.

311. Lippert-Grüner M, Švestková O, Angerová Y, Brtnická P, Pfeiffer J. Mechanisms of neuroplasticity and its influence on rehabilitation of patients in case of brain injuries. Eurorehab. 2006;16(3-4):78-83.

312. Lomarev MP, Kanchana S, Bara-Jimenez W, Iyer M, Wassermann EM, Hallett M. Placebo-controlled study of rTMS for the treatment of Parkinson's disease. Movement Disorders. 2006;21(3):325-31. doi: 10.1002/mds.20713.

313. Meinzer M, Flaisch T, Obleser J, Assadollahi R, Djundja D, Barthel G, et al. Brain regions essential for improved lexical access in an aged aphasic patient: A case report. BMC Neurology. 2006;6. doi: 10.1186/1471-2377-6-28.

314. Neto FC, De Oliveira E, Paschoal E, Cendes F, Santana Filho M. Morphological characteristics from the insula's lobe in patients

with medial temporal lobe epilepsy. Arquivos de Neuro-Psiquiatria. 2006;64(3 A).

315. Peers PV, Cusack R, Duncan J. Modulation of spatial bias in the dual task paradigm: Evidence from patients with unilateral parietal lesions and controls. Neuropsychologia. 2006;44(8):1325-35. doi: 10.1016/j.neuropsychologia.2006.01.033.

316. Perlstein WM, Larson MJ, Dotson VM, Kelly KG. Temporal dissociation of components of cognitive control dysfunction in severe TBI: ERPs and the cued-Stroop task. Neuropsychologia. 2006;44(2):260-74. doi: 10.1016/j.neuropsychologia.2005.05.009.

317. Stein RB, Chong S, Everaert DG, Rolf R, Thompson AK, Whittaker M, et al. A multicenter trial of a footdrop stimulator controlled by a tilt sensor. Neurorehabilitation and Neural Repair. 2006;20(3):371-9. doi: 10.1177/1545968306289292.

318. Šupe S, Matijević V, Kondić L, Alvir D. Series of seizures as a sign of development of recurrent malignant neuroleptic syndrome - A case report. Psychiatria Danubina. 2006;18(1-2):97-101.

319. Thiel A, Schumacher B, Wienhard K, Gairing S, Kracht LW, Wagner R, et al. Direct demonstration of transcallosal disinhibition in language networks. Journal of Cerebral Blood Flow and Metabolism. 2006;26(9):1122-7. doi: 10.1038/sj.jcbfm.9600350.

320. Tyvaert L, Houdayer E, Devanne H, Monaca C, Cassim F,

Derambure P. The effect of repetitive transcranial magnetic stimulation on dystonia: a clinical and pathophysiological approach. Neurophysiologie Clinique. 2006;36(3):135-43. doi: 10.1016/j.neucli.2006.08.007.
321. Urushihara R, Murase N, Rothwell JC, Harada M, Hosono Y, Asanuma K, et al. Effect of repetitive transcranial magnetic stimulation applied over the premotor cortex on somatosensory-evoked potentials and regional cerebral blood flow. NeuroImage. 2006;31(2):699-709. doi: 10.1016/j.neuroimage.2005.12.027.
322. Wiese H, Tönnes C, de Greiff A, Nebel K, Diener HC, Stude P. Self-initiated movements in chronic prefrontal traumatic brain injury: An event-related functional MRI study. NeuroImage. 2006;30(4):1292-301. doi: 10.1016/j.neuroimage.2005.11.012.
323. Ernst S, Chun JKR, Ujeyl A, Ouyang F, Kuck KH. "Sequential" mapping mimicking "simultaneous" mapping using magnetic navigation during catheter ablation of supraventricular tachycardia: Results of the single DX study. Journal of Cardiovascular Electrophysiology. 2007;18(SUPPL. 1):S11-S7. doi: 10.1111/j.1540-8167.2007.00705.x.
324. Fried M, Hixson KK, Anderson L, Ogata Y, Mutabingwa TK, Duffy PE. The distinct proteome of placental malaria parasites.

Molecular and Biochemical Parasitology. 2007;155(1):57-65. doi: 10.1016/j.molbiopara.2007.05.010.
325. Guzzetta A, Bonanni P, Biagi L, Tosetti M, Montanaro D, Guerrini R, et al. Reorganisation of the somatosensory system after early brain damage. Clinical Neurophysiology. 2007;118(5):1110-21. doi: 10.1016/j.clinph.2007.02.014.
326. Han S, Humphreys GW. The fronto-parietal network and top-down modulation of perceptual grouping. Neurocase. 2007;13(4):278-89. doi: 10.1080/13554790701649930.
327. Holsheimer J, Khan YN, Raza SS, Khan EA. Effects of electrode positioning on perception threshold and paresthesia coverage in spinal cord stimulation. Neuromodulation. 2007;10(1):34-41. doi: 10.1111/j.1525-1403.2007.00085.x.
328. Hu J, Yang S, Xuan Y, Jiang Q, Yang Y, Haacke EM. Simultaneous detection of resolved glutamate, glutamine, and γ-aminobutyric acid at 4 T. Journal of Magnetic Resonance. 2007;185(2):204-13. doi: 10.1016/j.jmr.2006.12.010.
329. Li Y, Wang Z, Wang HL, Yu XZ. An analysis of the early diagnostic criteria for tuberculosis meningitis. Zhonghua nei ke za zhi [Chinese journal of internal medicine]. 2007;46(3):217-9.
330. Lomarev MP, Kim DY, Richardson SP, Voller B, Hallett M. Safety study of high-frequency transcranial magnetic stimulation in

patients with chronic stroke. Clinical Neurophysiology. 2007;118(9):2072-5. doi: 10.1016/j.clinph.2007.06.016.
331. Medvedovsky M, Taulu S, Bikmullina R, Paetau R. Artifact and head movement compensation in MEG. Neurology, Neurophysiology and Neuroscience. 2007;2007:1-10.
332. Nickel J, Jorgens S, Bussfeld P, Seitz RJ. Asymptomatic motor cortex displacement due to a giant arachnoid cyst. Neurocase. 2007;13(5-6):328-33. doi: 10.1080/13554790701849910.
333. Panettieri V, Wennberg B, Gagliardi G, Duch MA, Ginjaume M, Lax I. SBRT of lung tumours: Monte Carlo simulation with PENELOPE of dose distributions including respiratory motion and comparison with different treatment planning systems. Physics in Medicine and Biology. 2007;52(14):4265-81. doi: 10.1088/0031-9155/52/14/016.
334. Payne CM, Weber C, Crowley-Skillicorn C, Dvorak K, Bernstein H, Bernstein C, et al. Deoxycholate induces mitochondrial oxidative stress and activates NF-κB through multiple mechanisms in HCT-116 colon epithelial cells. Carcinogenesis. 2007;28(1):215-22. doi: 10.1093/carcin/bgl139.
335. Ross ED, Prodan CI, Monnot M. Human facial expressions are organized functionally across the upper-lower facial axis. Neuroscientist. 2007;13(5):433-46. doi:

10.1177/1073858407305618.
336. van Kuijk AA, Hendricks HT, Pasman JW, Kremer BH, Geurts AC. Are neuroradiological or neurophysiological characteristics associated with upper-extremity hypertonia in severe ischaemic supratentorial stroke? Journal of Rehabilitation Medicine. 2007;39(1):38-42. doi: 10.2340/16501977-0010.
337. Yildirim A, Kotan D, Yildirim S, Aygül R, Akçay F. Increased lipid peroxidation and decreased antioxidant response in serum and cerebrospinal fluid in acute ischemic stroke. Turkish Journal of Medical Sciences. 2007;37(2):75-81.
338. Yokota H, Atsumi T, Araki T, Fuse A, Sato H, Kushimoto S, et al. Cerebral endothelial injury in elderly patients with severe head injury measured by serum thrombomodulin and von Willebrand factor. Neurologia Medico-Chirurgica. 2007;47(9):383-8. doi: 10.2176/nmc.47.383.
339. Alkire MT. Loss of effective connectivity during general anesthesia. International Anesthesiology Clinics. 2008;46(3):55-73. doi: 10.1097/AIA.0b013e3181755dc6.
340. Arango-Lasprilla JC, Ketchum JM, Williams K, Kreutzer JS, Marquez de la Plata CD, O'Neil-Pirozzi TM, et al. Racial Differences in Employment Outcomes After Traumatic Brain Injury. Archives of Physical Medicine and Rehabilitation.

2008;89(5):988-95. doi: 10.1016/j.apmr.2008.02.012.

341. Butterworth RF. Pathophysiology of hepatic encephalopathy: The concept of synergism. Hepatology Research. 2008;38(SUPPL. 1):S116-S21. doi: 10.1111/j.1872-034X.2008.00436.x.

342. Carey JR, Evans CD, Anderson DC, Bhatt E, Nagpal A, Kimberley TJ, et al. Safety of 6-Hz primed low-frequency rTMS in stroke. Neurorehabilitation and Neural Repair. 2008;22(2):185-92. doi: 10.1177/1545968307305458.

343. Clement EA, Richard A, Thwaites M, Ailon J, Peters S, Dickson CT. Cyclic and sleep-like spontaneous alternations of brain state under urethane anaesthesia. PLoS ONE. 2008;3(4). doi: 10.1371/journal.pone.0002004.

344. Cologno D, Buzzi MG, Cicinelli P, Caltagirone C, Spalletta G. Symptomatic cluster-like headache triggered by forehead lipoma: A case report and review of the literature. Neurological Sciences. 2008;29(5):331-5. doi: 10.1007/s10072-008-0990-6.

345. Cummings JL. Progress in neurotherapeutics and neuropsychopharmacology 2008. Progress in Neurotherapeutics and Neuropsychopharmacology. 2008;3(1):1-11. doi: 10.1017/S1748232107000183.

346. Gómez Herrero H, García Asensio S, Martínez-Berganza Asensio MT, Sarría Octavio De Toledo L, Cozcolluela Cabrejas R,

Forradellas Morales A. Indirect CT venography in patients with clinical suspicion of pulmonary thromboembolism: Our experience and possible indications for the test. Radiologia. 2008;50(2):147-52. doi: 10.1016/S0033-8338(08)71948-9.

347. Gruessner RWG, Sutherland DER, Kandaswamy R, Gruessner AC. Over 500 solitary pancreas transplants in nonuremic patients with brittle diabetes mellitus. Transplantation. 2008;85(1):42-7. doi: 10.1097/01.tp.0000296820.46978.3f.

348. Hossain MA, Afroj S, Rahman MR, Yunus EB, Samad R, Asna ZH, et al. Evaluation of alternative diagnostic techniques for diagnosis of cerebral malaria in a tertiary referral hospital in Bangladesh. Mymensingh medical journal : MMJ. 2008;17(2):180-5.

349. Ji YK, Eun JC, Won YL, Hee YS, Gyeong HL, Choe YS, et al. Therapeutic effect of repetitive transcranial magnetic stimulation in Parkinson's disease: Analysis of [11C] raclopride PET study. Movement Disorders. 2008;23(2):207-11. doi: 10.1002/mds.21787.

350. Knott C. Research into cannabinoid medicines may suggest potential therapeutic opportunities. Pharmacy in Practice. 2008;18(3):96-109.

351. Marquez de la Plata CD, Hart T, Hammond FM, Frol AB, Hudak A, Harper CR, et al. Impact of Age on Long-Term Recovery

From Traumatic Brain Injury. Archives of Physical Medicine and Rehabilitation. 2008;89(5):896-903. doi: 10.1016/j.apmr.2007.12.030.

352. Naito T, Mino Y, Otsuka A, Ushiyama T, Ozono S, Kagawa Y, et al. Influence of metal cations on plasma trough concentration of mycophenolic acid and its glucuronide in tacrolimus-treated and cyclosporine-treated kidney transplant recipients. Biological and Pharmaceutical Bulletin. 2008;31(6):1292-6. doi: 10.1248/bpb.31.1292.

353. Obuchi T, Katayama Y, Kobayashi K, Oshima H, Fukaya C, Yamamoto T. Direction and predictive factors for the shift of brain structure during deep brain stimulation electrode implantation for advanced Parkinson's disease. Neuromodulation. 2008;11(4):302-10. doi: 10.1111/j.1525-1403.2008.00180.x.

354. Poreisz C, Csifcsák G, Antal A, Levold M, Hillers F, Paulus W. Theta burst stimulation of the motor cortex reduces laser-evoked pain perception. NeuroReport. 2008;19(2):193-6. doi: 10.1097/WNR.0b013e3282f45498.

355. Ricci R, Ramsey D, Johnson K, Borckardt JJ, Vallejo M, Roberts DR, et al. A pilot feasibility study of daily rTMS to modify corticospinal excitability during lower limb immobilization. Therapeutics and Clinical Risk Management. 2008;4(5):1127-34.

356. Rubí Callejón J, Arjona Padillo A, Alonso Verdegay G, Huete Hurtado A, Naranjo C, Serrano Castro P, et al. Trigeminal-autonomic cephalalgias: International Headache Society diagnostic criteria does not allow for a correct classification. Neurologia. 2008;23(4):203-8.

357. Schiff ND. Central thalamic contributions to arousal regulation and neurological disorders of consciousness. Annals of the New York Academy of Sciences2008. p. 105-18.

358. Sedlackova S, Rektorova I, Fanfrdlova Z, Rektor I. Neurocognitive effects of repetitive transcranial magnetic stimulation in patients with cerebrovascular disease without dementia. Journal of Psychophysiology. 2008;22(1):14-9. doi: 10.1027/0269-8803.22.1.14.

359. Song YG, Hao FT, Zhu J, Li A, Xia YJ, Wang DX. [Clinical study on pneumoconiosis with pulmonary thromboembolism]. Zhonghua lao dong wei sheng zhi ye bing za zhi = Zhonghua laodong weisheng zhiyebing zazhi = Chinese journal of industrial hygiene and occupational diseases. 2008;26(1):39-42.

360. Strangman GE, O'Neil-Pirozzi TM, Goldstein R, Kelkar K, Katz DI, Burke D, et al. Prediction of Memory Rehabilitation Outcomes in Traumatic Brain Injury by Using Functional Magnetic Resonance Imaging. Archives of Physical Medicine and

Rehabilitation. 2008;89(5):974-81. doi: 10.1016/j.apmr.2008.02.011.

361. Zitnay GA, Zitnay KM, Povlishock JT, Hall ED, Marion DW, Trudel T, et al. Traumatic brain injury research priorities: The conemaugh international brain injury symposium. Journal of Neurotrauma. 2008;25(10):1135-52. doi: 10.1089/neu.2008.0599.

362. Blicher JU, Nielsen JF. Cortical and spinal excitability changes after robotic gait training in healthy participants. Neurorehabilitation and Neural Repair. 2009;23(2):143-9. doi: 10.1177/1545968308317973.

363. Caramia F, Francia A, Mainero C, Tinelli E, Palmieri MG, Colonnese C, et al. Neurophysiological and functional MRI evidence of reorganization of cortical motor areas in cerebral arteriovenous malformation. Magnetic Resonance Imaging. 2009;27(10):1360-9. doi: 10.1016/j.mri.2009.05.029.

364. Carlson T, Andréll P, Ekre O, Edvardsson N, Holmgren C, Jacobsson F, et al. Interference of transcutaneous electrical nerve stimulation with permanent ventricular stimulation: A new clinical problem? Europace. 2009;11(3):364-9. doi: 10.1093/europace/eun351.

365. Edwards DJ, Krebs HI, Rykman A, Zipse J, Thickbroom GW, Mastaglia FL, et al. Raised corticomotor excitability of M1 forearm

area following anodal tDCS is sustained during robotic wrist therapy in chronic stroke. Restorative Neurology and Neuroscience. 2009;27(3):199-207. doi: 10.3233/RNN-2009-0470.

366. Jardri R. Transcranial magnetic stimulation for the management of hallucinations in children with early-onset schizophrenia. Neuropsychiatrie de l'Enfance et de l'Adolescence. 2009;57(1):38-43. doi: 10.1016/j.neurenf.2007.07.017.

367. Kanno T, Morita I, Yamaguchi S, Yokoyama T, Kamei Y, Anil SM, et al. Dorsal column stimulation in persistent vegetative state. Neuromodulation. 2009;12(1):33-8. doi: 10.1111/j.1525-1403.2009.00185.x.

368. Kranz G, Shamim EA, Lin PT, Kranz GS, Voller B, Hallett M. Blepharospasm and the modulation of cortical excitability in primary and secondary motor areas. Neurology. 2009;73(23):2031-6. doi: 10.1212/WNL.0b013e3181c5b42d.

369. Lo YL, Fook-Chong S, Chan LL, Ong WY. Cerebellar control of motor activation and cancellation in humans: An electrophysiological study. Cerebellum. 2009;8(3):302-11. doi: 10.1007/s12311-009-0095-7.

370. Martin RCG, Joshi J, Robbins K, Tomalty D, O'Hara R, Tatum C. Transarterial chemoembolization of metastatic colorectal carcinoma with drug-eluting beads, irinotecan (DEBIRI):

Multi-institutional registry. Journal of Oncology. 2009. doi: 10.1155/2009/539795.

371. Meera P, Olsen RW, Otis TS, Wallner M. Etomidate, propofol and the neurosteroid THDOC increase the GABA efficacy of recombinant α4β3δ and α4β3 GABAA receptors expressed in HEK cells. Neuropharmacology. 2009;56(1):155-60. doi: 10.1016/j.neuropharm.2008.08.011.

372. Morrison LJ, Rizoli SB, Schwartz B, Rhind SG, Simitciu M, Perreira T, et al. The Toronto prehospital hypertonic resuscitation-head injury and multi organ dysfunction trial (TOPHR HIT) - Methods and data collection tools. Trials. 2009;10. doi: 10.1186/1745-6215-10-105.

373. Muresanu DF. Neuromodulation with pleiotropic and multimodal drugs - Future approaches to treatment of neurological disorders. Acta Neurochirurgica, Supplementum2009. p. 291-4.

374. Sirén AL, Faßhauer T, Bartels C, Ehrenreich H. Therapeutic Potential of Erythropoietin and its Structural or Functional Variants in the Nervous System. Neurotherapeutics. 2009;6(1):108-27. doi: 10.1016/j.nurt.2008.10.041.

375. Xie GH, Fang XM. Importance of recognizing and managing delirium in intensive care unit. Chinese Journal of Traumatology - English Edition. 2009;12(6):370-4. doi:

10.3760/cma.j.issn.1008-1275.2009.06.009.

376. Abo M, Kakuda W. Neuroimaging and neurorehabilitation for aphasia. Brain and Nerve. 2010;62(2):141-9.

377. An HS, Cho BM, Kang JH, Kim MK, Oh SM, Park SH. Efficacy of low dose barbiturate coma therapy for the patients with intractable intracranial hypertension using the bispectral™ index monitoring. Journal of Korean Neurosurgical Society. 2010;47(4):252-7. doi: 10.3340/jkns.2010.47.4.252.

378. Finn DP. International Cannabinoid Research Society - 20th annual symposium. IDrugs. 2010;13(10):677-9.

379. George MS, Lisanby SH, Avery D, McDonald WM, Durkalski V, Pavlicova M, et al. Daily left prefrontal transcranial magnetic stimulation therapy for major depressive disorder: A sham-controlled randomized trial. Archives of General Psychiatry. 2010;67(5):507-16. doi: 10.1001/archgenpsychiatry.2010.46.

380. Henderson JM, Heit G, Fisher RS. Recurrent seizures related to motor cortex stimulator programming. Neuromodulation. 2010;13(1):37-43. doi: 10.1111/j.1525-1403.2009.00256.x.

381. Hirota N, Yasuda D, Hashidate T, Yamamoto T, Yamaguchi S, Nagamune T, et al. Amino acid residues critical for endoplasmic reticulum export and trafficking of platelet-activating factor receptor. Journal of Biological Chemistry. 2010;285(8):5931-40.

doi: 10.1074/jbc.M109.066282.
382. Hochman G, Yechiam E, Bechara A. Recency gets larger as lesions move from anterior to posterior locations within the ventromedial prefrontal cortex. Behavioural Brain Research. 2010;213(1):27-34. doi: 10.1016/j.bbr.2010.04.023.
383. Hori T, Oike F, Hata K, Nishikiori M, Ogura Y, Ogawa K, et al. Hashimoto's encephalopathy after interferon therapy for hepatitis C virus in adult liver transplant recipient accompanied by post-transplant lymphoproliferative disorder related to Epstein-Barr virus infection: Case report. Transplant Infectious Disease. 2010;12(4):347-52. doi: 10.1111/j.1399-3062.2010.00508.x.
384. Kang DW, Kang JH, Lee MS, Chang JW. Posttraumatic hemidystonia treated with unilateral globus pallidus interna stimulation: Long-term follow-up and radiologic features. Neuromodulation. 2010;13(4):261-4. doi: 10.1111/j.1525-1403.2010.00306.x.
385. Kantelhardt SR, Fadini T, Finke M, Kallenberg K, Siemerkus J, Bockermann V, et al. Robot-assisted image-guided transcranial magnetic stimulation for somatotopic mapping of the motor cortex: A clinical pilot study. Acta Neurochirurgica. 2010;152(2):333-43. doi: 10.1007/s00701-009-0565-1.
386. Koivisto M, Mäntylä T, Silvanto J. The role of early visual

cortex (V1/V2) in conscious and unconscious visual perception. NeuroImage. 2010;51(2):828-34. doi: 10.1016/j.neuroimage.2010.02.042.

387. Liuzzi G, Freundlieb N, Ridder V, Hoppe J, Heise K, Zimerman M, et al. The involvement of the left motor cortex in learning of a novel action word lexicon. Current Biology. 2010;20(19):1745-51. doi: 10.1016/j.cub.2010.08.034.

388. Nuutinen S, Panula P. Histamine in neurotransmission and brain diseases. Advances in Experimental Medicine and Biology2010. p. 95-107.

389. Quemada JI, De Francisco ML, Echeburúa E, Bulbena A. Impact of psychiatric and neuropsychological consequences of traumatic brain injury on the duration of the legal periods. Actas Espanolas de Psiquiatria. 2010;38(2):101-7.

390. Roth TM. Sacral neuromodulation and cardiac pacemakers. International Urogynecology Journal. 2010;21(8):1035-7. doi: 10.1007/s00192-009-1091-7.

391. Scavarda D, Gabaudan C, Ughetto F, Lamy F, Imada V, Lena G, et al. Initial predictive factors of outcome in severe non-accidental head trauma in children. Child's Nervous System. 2010;26(11):1555-61. doi: 10.1007/s00381-010-1150-x.

392. Yang FG, Fuller J, Khodaparast N, Krawczyk DC. Figurative

language processing after traumatic brain injury in adults: A preliminary study. Neuropsychologia. 2010;48(7):1923-9. doi: 10.1016/j.neuropsychologia.2010.03.011.
393. Zorrilla-Zubilete MA, Maur DG, Palumbo ML, Genaro AM. Role of nitric oxide signaling pathways in brain injuries. Current Chemical Biology. 2010;4(3):250-61. doi: 10.2174/187231310792062666.
394. Abdel-Salam OME. Stem cell therapy for Alzheimer's disease. CNS and Neurological Disorders - Drug Targets. 2011;10(4):459-85. doi: 10.2174/187152711795563976.
395. Billino J, Braun DI, Bremmer F, Gegenfurtner KR. Challenges to normal neural functioning provide insights into separability of motion processing mechanisms. Neuropsychologia. 2011;49(12):3151-63. doi: 10.1016/j.neuropsychologia.2011.07.009.
396. Blom RM, Figee M, Vulink N, Denys D. Update on repetitive transcranial magnetic stimulation in obsessive-compulsive disorder: Different targets. Current Psychiatry Reports. 2011;13(4):289-94. doi: 10.1007/s11920-011-0205-3.
397. Buiten MS, Dejongste MJL, Beese U, Kliphuis C, Durenkamp A, Staal MJ. Subcutaneous electrical nerve stimulation: A feasible and new method for the treatment of patients with refractory

angina. Neuromodulation. 2011;14(3):258-65. doi: 10.1111/j.1525-1403.2011.00355.x.

398. Burnstock G, Fredholm BB, Verkhratsky A. Adenosine and ATP receptors in the brain. Current Topics in Medicinal Chemistry. 2011;11(8):973-1011. doi: 10.2174/156802611795347627.

399. Charash WE, Caputo MP, Clark H, Callas PW, Rogers FB, Crookes BA, et al. Telemedicine to a moving ambulance improves outcome after trauma in simulated patients. Journal of Trauma - Injury, Infection and Critical Care. 2011;71(1):49-54. doi: 10.1097/TA.0b013e31821e4690.

400. Clauss RP. Neurotransmitters in disorders of consciousness and brain damage. Medical Hypotheses. 2011;77(2):209-13. doi: 10.1016/j.mehy.2011.04.014.

401. Eiermann W, Pienkowski T, Crown J, Sadeghi S, Martin M, Chan A, et al. Phase III study of doxorubicin/cyclophosphamide with concomitant versus sequential docetaxel as adjuvant treatment in patients with human epidermal growth factor receptor 2-normal, node-positive breast cancer: BCIRG-005 trial. Journal of Clinical Oncology. 2011;29(29):3877-84. doi: 10.1200/JCO.2010.28.5437.

402. Engels PT, Rezende-Neto JB, Al Mahroos M, Scarpelini S, Rizoli SB, Tien HC. The natural history of trauma-related coagulopathy: Implications for treatment. Journal of Trauma -

Injury, Infection and Critical Care. 2011;71(5 SUPPL.1):S448-S55. doi: 10.1097/TA.0b013e318232e6ac.

403. Fathali N, Khatibi NH, Ostrowski RP, Zhang JH. The evolving landscape of neuroinflammation after neonatal hypoxia-ischemia. Acta Neurochirurgica, Supplementum2011. p. 93-100.

404. Hirabayashi H, Kawata K, Hoshida T, Tamura K, Youngsu P, Nakase H. Neuromodulation therapy for neuropathic pain. Japanese Journal of Neurosurgery. 2011;20(2):93-102. doi: 10.7887/jcns.20.93.

405. Insola A, Padua L, Valeriani M. Absent median nerve P14 far-field somatosensory evoked potential with persistent tibial nerve P30 component in a patient with ischemic pontine lesion. Neurophysiologie Clinique. 2011;41(2):81-4. doi: 10.1016/j.neucli.2010.12.003.

406. Jan JE, Ribary U, Wong PKH, Reiter RJ, Bax MCO, Wasdell MB. Cerebral modulation of circadian sleep-wake rhythms. Journal of Clinical Neurophysiology. 2011;28(2):165-9. doi: 10.1097/WNP.0b013e31821219e3.

407. Jotterand F, Giordano J. Transcranial magnetic stimulation, deep brain stimulation and personal identity: Ethical questions, and neuroethical approaches for medical practice. International Review of Psychiatry. 2011;23(5):476-85. doi:

10.3109/09540261.2011.616189.

408. Kao CD, Lin KP, Chen JT, Chang JB, Guo WY, Lin YY, et al. Preserved motor-evoked potentials but without good motor recovery in a patient with decerebrate rigidity. Journal of the Chinese Medical Association. 2011;74(1):37-9. doi: 10.1016/j.jcma.2011.01.005.

409. Keep RF, Xi G, Hua Y, Xiang J. Clot formation, vascular repair and hematoma resolution after ICH, a coordinating role for thrombin? Acta Neurochirurgica, Supplementum2011. p. 71-5.

410. Krasomski G. The role of cervical factor in preterm labour etiopathogenesis. Ginekologia i Poloznictwo. 2011;21(3):87-91.

411. Levens SM, Devinsky O, Phelps EA. Role of the left amygdala and right orbital frontal cortex in emotional interference resolution facilitation in working memory. Neuropsychologia. 2011;49(12):3201-12. doi: 10.1016/j.neuropsychologia.2011.07.021.

412. Panina OS, Zaytseva GA, Pokazan'eva SA. Rehabilitation of newborns with perinatal lesions of the central nervous system using transcranial magnetic therapy. Zhurnal Nevrologii i Psihiatrii imeni SS Korsakova. 2011;111(4):87-9.

413. Park YS, Kim JP, Chang WS, Chang JW. Management of a DBS system in patients with traumatic brain injury: Case report.

Neuromodulation. 2011;14(3):214-8. doi: 10.1111/j.1525-1403.2011.00348.x.
414. Pascual-Leone A, Freitas C, Oberman L, Horvath JC, Halko M, Eldaief M, et al. Characterizing brain cortical plasticity and network dynamics across the age-span in health and disease with TMS-EEG and TMS-fMRI. Brain Topography. 2011;24(3-4):302-15. doi: 10.1007/s10548-011-0196-8.
415. Piech RM, McHugo M, Smith SD, Dukic MS, Van Der Meer J, Abou-Khalil B, et al. Attentional capture by emotional stimuli is preserved in patients with amygdala lesions. Neuropsychologia. 2011;49(12):3314-9. doi: 10.1016/j.neuropsychologia.2011.08.004.
416. Piriz J, Muller A, Trejo JL, Torres-Aleman I. IGF-I and the aging mammalian brain. Experimental Gerontology. 2011;46(2-3):96-9. doi: 10.1016/j.exger.2010.08.022.
417. Schwartz TL, Nihalani N. Neuronal pathways implicated in executive dysfunction. Clinical Neuropsychiatry. 2011;8(3):212-8.
418. Sironi VA. Origin and evolution of deep brain stimulation. Frontiers in Integrative Neuroscience. 2011;5. doi: 10.3389/fnint.2011.00042.
419. Sperlágh B, Vizi ES. The role of extracellular adenosine in chemical neurotransmission in the hippocampus and basal ganglia: Pharmacological and clinical aspects. Current Topics in Medicinal

Chemistry. 2011;11(8):1034-46. doi: 10.2174/156802611795347564.

420. Stenson K, Brownrigg JRW, Mohamed M. Occurrence and multiple recurrence of severe vasospasm of the upper extremity following thorascopic sympathectomy for hyperhidrosis. European Journal of Vascular and Endovascular Surgery. 2011;41(2):264-6. doi: 10.1016/j.ejvs.2010.11.003.

421. Thordstein M, Hallböök T, Lundgren J, Van Westen D, Elam M. Transfer of cortical motor representation after a perinatal cerebral insult. Pediatric Neurology. 2011;44(2):131-4. doi: 10.1016/j.pediatrneurol.2010.08.017.

422. Van Der Aa NE, Debode S, Van Diessen E, Verhage CH, Van Nieuwenhuizen O, Ramsey NF, et al. Cortical reorganisation in a preterm born child with unilateral watershed infarction. European Journal of Paediatric Neurology. 2011;15(6):554-7. doi: 10.1016/j.ejpn.2011.04.008.

423. van der Harst JJ, Luijckx GJ. Treatment of central nervous system tuberculosis infections and neurological complications of tuberculosis treatment. Current Pharmaceutical Design. 2011;17(27):2940-7. doi: 10.2174/138161211797470237.

424. Vetrugno R, Montagna P. From REM sleep behaviour disorder to status dissociatus: Insights into the maze of states of being.

Sleep Medicine. 2011;12(SUPPL. 2):S68-S71. doi: 10.1016/j.sleep.2011.10.015.
425. Zeller D, Dang SY, Stefan K, Biller A, Bartsch A, Saur D, et al. Functional role of ipsilateral motor areas in multiple sclerosis. Journal of Neurology, Neurosurgery and Psychiatry. 2011;82(5):578-83. doi: 10.1136/jnnp.2010.219964.
426. Zisa M, Di Carlo I, Pulvirenti E, Guastella T. Complex partial seizure in patient with insulinoma: importance of early diagnosis. Il Giornale di chirurgia. 2011;32(1-2):45-7.
427. Achard S, Delon-Martin C, Vértes PE, Renard F, Schenck M, Schneider F, et al. Hubs of brain functional networks are radically reorganized in comatose patients. Proceedings of the National Academy of Sciences of the United States of America. 2012;109(50):20608-13. doi: 10.1073/pnas.1208933109.
428. Agudelo M, Yoo C, Nair MP. Alcohol-induced serotonergic modulation: The role of histone deacetylases. Alcohol. 2012;46(7):635-42. doi: 10.1016/j.alcohol.2012.03.005.
429. Alkan F, Altinbaş K. Detection of demografic and clinical risk factors associated with neuroleptic malignant syndrome: Evaluation of cases in the Turkish literature. Dusunen Adam. 2012;25(1):35-42. doi: 10.5350/DAJPN2012250104.
430. Beigh Z, Ahmad MM, Yousuf A, Ahmad RP. Role of topical

heparine in healing of traumatic tympanic membrane perforations. Indian Journal of Otology. 2012;18(3):143-7. doi: 10.4103/0971-7749.103442.
431. Çakir EDP, Mutlu FŞ, Eren E, Paşa AÖ, Sağlam H, Tarim Ö. Testicular adrenal rest tumors in patients with congenital adrenal hyperplasia. JCRPE Journal of Clinical Research in Pediatric Endocrinology. 2012;4(2):94-100. doi: 10.4274/jcrpe.563.
432. Chollet F, Albucher JF. Strategies to augment recovery after stroke. Current Treatment Options in Neurology. 2012;14(6):531-40. doi: 10.1007/s11940-012-0196-3.
433. De Freitas GR. Aphasia and other language disorders. Frontiers of Neurology and Neuroscience2012. p. 41-5.
434. de Haan B, Karnath HO. Objections against the view of visual extinction as an attentional disengagement deficit: The interaction between spatial position and temporal modulation. Cortex. 2012;48(8):972-9. doi: 10.1016/j.cortex.2011.03.011.
435. Frenkel-Toledo S, Liebermann DG, Bentin S, Soroker N. Dysfunction of the Human Mirror Neuron System in Ideomotor Apraxia: Evidence from Mu Suppression. Journal of Cognitive Neuroscience. 2012;10:775-91. doi: 10.1162/jocn_a_00936.
436. Gheysarzadeh A, Yazdanparast R. Inhibition of H2O2-induced cell death through FOXO1 modulation by EUK-172 in SK-N-MC

cells. European Journal of Pharmacology. 2012;697(1-3):47-52. doi: 10.1016/j.ejphar.2012.09.036.
437. Hansen N, Seiler C, Rumpf J, Kraft P, Dlaske H, Abele-Horn M, et al. Human tuberculous meningitis caused by mycobacterium caprae. Case Reports in Neurology. 2012;4(1):54-60. doi: 10.1159/000337299.
438. Hong JH, Lee J, Cho YW, Byun WM, Cho HK, Son SM, et al. Limb apraxia in a patient with cerebral infarct: Diffusion tensor tractography study. NeuroRehabilitation. 2012;30(4):255-9. doi: 10.3233/NRE-2012-0753.
439. Huang H, Chen L, Sanberg PR. Clinical achievements, obstacles, falsehoods, and future directions of cell-based neurorestoratology. Cell Transplantation. 2012;21(SUPPL. 1):S3-S11. doi: 10.3727/096368912X633725.
440. Kaindl AM, Degos V, Peineau S, Gouadon E, Chhor V, Loron G, et al. Activation of microglial N-methyl-D-aspartate receptors triggers inflammation and neuronal cell death in the developing and mature brain. Annals of Neurology. 2012;72(4):536-49. doi: 10.1002/ana.23626.
441. Kang EK, Kim DY, Paik NJ. Transcranial direct curent stimulation of the left prefrontal cortex improves attention in patients with traumatic brain injury: A pilot study. Journal of

Rehabilitation Medicine. 2012;44(4):346-50. doi: 10.2340/16501977-0947.

442. Kim J, Whyte J, Patel S, Europa E, Wang J, Coslett HB, et al. Methylphenidate modulates sustained attention and cortical activation in survivors of traumatic brain injury: A perfusion fMRI study. Psychopharmacology. 2012;222(1):47-57. doi: 10.1007/s00213-011-2622-8.

443. Koivisto M, Henriksson L, Revonsuo A, Railo H. Unconscious response priming by shape depends on geniculostriate visual projection. European Journal of Neuroscience. 2012;35(3-4):623-33. doi: 10.1111/j.1460-9568.2011.07973.x.

444. Lin RS, Wang W, Guo A. Management of distal posterior inferior cerebellar artery aneurysms. Journal of Craniofacial Surgery. 2012;23(5):1388-90. doi: 10.1097/SCS.0b013e31825653ce.

445. Martin MJ, Hatch Q, Cotton B, Holcomb J. The use of temporary abdominal closure in low-risk trauma patients: Helpful or harmful? Journal of Trauma and Acute Care Surgery. 2012;72(3):601-8. doi: 10.1097/TA.0b013e31824483b7.

446. McIntyre WF, Pérez-Riera AR, Femenía F, Baranchuk A. Coexisting early repolarization pattern and Brugada syndrome: Recognition of potentially overlapping entities. Journal of

Electrocardiology. 2012;45(3):195-8. doi: 10.1016/j.jelectrocard.2011.10.008.

447. Oberdorfer P, Kongthavonsakul K, Intachumpoo J, Odell S. A 14-year-old girl with tuberculous otitis media and brain abscess. BMJ Case Reports. 2012. doi: 10.1136/bcr-2012-006618.

448. Pang T, Drislane FW. Treatment of nonconvulsive status epilepticus. Current Treatment Options in Neurology. 2012;14(4):307-21. doi: 10.1007/s11940-012-0179-4.

449. Panina OS, Zaitseva GA, Pokazanieva SA. Rehabilitation of neonates with perinatal injuries to the central nervous system using transcranial magnetotherapy. Neuroscience and Behavioral Physiology. 2012;42(7):745-7. doi: 10.1007/s11055-012-9628-5.

450. Patel V, Griffith NC, Blackwood E, Dias M, Cordato DJ. Spectrum disorder of neuromyelitis optica in a patient presenting with intractable vomiting and hiccups, transverse myelitis and acute encephalopathy. Journal of Clinical Neuroscience. 2012;19(11):1576-8. doi: 10.1016/j.jocn.2012.03.007.

451. Perruchoud C, Bovy M, Durrer A, Rosato M, Rutschmann B, Mustaki JP, et al. Severe hypertension following accidental clonidine overdose during the refilling of an implanted intrathecal drug delivery system. Neuromodulation. 2012;15(1):31-4. doi: 10.1111/j.1525-1403.2011.00392.x.

452. Skaper SD. 1 st workshop on "palmitoylethanolamide: Biochemistry, Pharmacology and therapeutic use of a Pleiotropic anti-inflammatory lipid Mediator". CNS and Neurological Disorders - Drug Targets. 2012;11(3):3.
453. Soto E, Hao J, Knotkova H, Cruciani RA. Central neurostimulation techniques for primary headaches. Techniques in Regional Anesthesia and Pain Management. 2012;16(1):47-56. doi: 10.1053/j.trap.2012.11.007.
454. Taira T. Tinnitus: From traditional concept to challenges to loosen the gordian knot. World Neurosurgery. 2012;77(5-6):651-3. doi: 10.1016/j.wneu.2011.10.017.
455. Taksande A, Prabhu S, Venkatesh S. Cardiovascular aspect of beta-thalassaemia. Cardiovascular and Hematological Agents in Medicinal Chemistry. 2012;10(1):25-30. doi: 10.2174/187152512799201172.
456. Van Koningsbruggen MG, Peelen MV, Davies E, Rafal RD. Neural control of voluntary eye closure: A case study and an fMRI investigation of blinking and winking. Behavioural Neurology. 2012;25(2):103-9. doi: 10.3233/BEN-2011-0355.
457. Wong M. mTOR as a potential treatment target for epilepsy. Future Neurology. 2012;7(5):537-45. doi: 10.2217/fnl.12.45.
458. Xiong ZG, Xu TL. The role of ASICs in cerebral ischemia.

Wiley Interdisciplinary Reviews: Membrane Transport and Signaling. 2012;1(5):655-62. doi: 10.1002/wmts.57.

459. Zafonte R. Brain Injury: Refinement and Restoration-The Light Is Coming On. PM and R. 2012;4(9):639-41. doi: 10.1016/j.pmrj.2012.07.008.

460. Zelante G, Catalano A, Ricceri R, Rampello L, Rampello L. Subcortical vascular dementia: Lights and shadows. Acta Medica Mediterranea. 2012;28(1):45-52.

461. Zeller D, Dang SY, Weise D, Rieckmann P, Toyka KV, Classen J. Excitability decreasing central motor plasticity is retained in multiple sclerosis patients. BMC Neurology. 2012;12. doi: 10.1186/1471-2377-12-92.

462. Zhou FA, Davis G. Momentary Conscious Pairing Eliminates Unconscious-Stimulus Influences on Task Selection. PLoS ONE. 2012;7(9). doi: 10.1371/journal.pone.0046320.

463. Zittel S, Kroeger J, van der Vegt JPM, Siebner HR, Brüggemann N, Ramirez A, et al. Motor pathway excitability in ATP13A2 mutation carriers: A transcranial magnetic stimulation study. Parkinsonism and Related Disorders. 2012;18(5):590-4. doi: 10.1016/j.parkreldis.2011.10.020.

464. Arslan D, Kalkan M, Yazgan H, Ünüvar U, Şahin C. Collective circumcision performed in Sudan: Evaluation in terms

of early complications and alternative practice. Urology. 2013;81(4):864-8. doi: 10.1016/j.urology.2012.11.070.

465. Baguley D, McFerran D, Hall D. Tinnitus. The Lancet. 2013;382(9904):1600-7. doi: 10.1016/S0140-6736(13)60142-7.

466. Balconi M, Bortolotti A. Conscious and unconscious face recognition is improved by high-frequency rTMS on pre-motor cortex. Consciousness and Cognition. 2013;22(3):771-8. doi: 10.1016/j.concog.2013.04.013.

467. Barbas AS, Rege AS, Castleberry AW, Gommer J, Ellis MJ, Brennan TV, et al. Posterior reversible encephalopathy syndrome independently associated with tacrolimus and sirolimus after multivisceral transplantation. American Journal of Transplantation. 2013;13(3):808-10. doi: 10.1111/ajt.12061.

468. Barnes VA, Rigg JL, Williams JJ. Clinical case series: Treatment of PTSD with transcendental meditation in active duty military personnel. Military Medicine. 2013;178(7):e836-e40. doi: 10.7205/MILMED-D-12-00426.

469. Barrett AM, Oh-Park M, Chen P, Ifejika NL. Neurorehabilitation: Five new things. Neurology: Clinical Practice. 2013;3(6):484-92. doi: 10.1212/01.CPJ.0000437088.98407.fa.

470. Bick AS, Leker RR, Ben-Hur T, Levin N. Implementing novel imaging methods for improved diagnosis of disorder of

consciousness patients. Journal of the Neurological Sciences. 2013;334(1-2):130-8. doi: 10.1016/j.jns.2013.08.009.
471. Blakemore RL, Hyland BI, Hammond-Tooke GD, Anson JG. Distinct Modulation of Event-Related Potentials during Motor Preparation in Patients with Motor Conversion Disorder. PLoS ONE. 2013;8(4). doi: 10.1371/journal.pone.0062539.
472. Brukamp K. Motor-independent communication by severely physically challenged patients. Neuroscientific research results and patient autonomy. Nervenarzt. 2013;84(10):1190-5. doi: 10.1007/s00115-013-3813-9.
473. Brukamp K. Right (to a) diagnosis? Establishing correct diagnoses in chronic disorders of consciousness. Neuroethics. 2013;6(1):5-11. doi: 10.1007/s12152-012-9154-8.
474. Chen L, Sharma A, Guo J, Muresanu D, Huang H, Skaper S, et al. Annual advances in neurorestoratology: A summary of IANR VI and 10th GCNN Conference, Bucharest, Romania, april 4-7, 2013. CNS and Neurological Disorders - Drug Targets. 2013;12(5):547-9. doi: 10.2174/18715273113120500001.
475. Cheng CA, Chu H, Cheng CC, Hsu CH, Lin CC, Tsai CL, et al. Modifications of autonomic activity and baroreceptor response on posture challenge in patients with vasovagal syncope. Journal of Medical Sciences (Taiwan). 2013;33(4):199-204. doi:

10.6136/JMS.2013.33(4).199.
476. Diaz-Ledezma C, Lichstein PM, Maltenfort M, Restrepo C, Parvizi J. Pattern of impact of femoroacetabular impingement upon healthrelated quality of life: The determinant role of extra-articular factors. Quality of Life Research. 2013;22(9):2323-30. doi: 10.1007/s11136-013-0359-z.
477. Do KH, Yeo SS, Lee J, Jang SH. Injury of the corticoreticular pathway in patients with proximal weakness following cerebral infarct: Diffusion tensor tractography study. Neuroscience Letters. 2013;546:21-5. doi: 10.1016/j.neulet.2013.04.040.
478. Hashimoto T, Taoka M, Obayashi S, Hara Y, Tanaka M, Iriki A. Modulation of cortical vestibular processing by somatosensory inputs in the posterior insula. Brain Injury. 2013;27(13-14):1685-91. doi: 10.3109/02699052.2013.831128.
479. Hillert L, Jovanovic H, Åhs F, Savic I. Women with Multiple Chemical Sensitivity Have Increased Harm Avoidance and Reduced 5-HT1A Receptor Binding Potential in the Anterior Cingulate and Amygdala. PLoS ONE. 2013;8(1). doi: 10.1371/journal.pone.0054781.
480. Höller Y, Bergmann J, Thomschewski A, Kronbichler M, Holler P, Crone JS, et al. Comparison of EEG-features and classification methods for motor imagery in patients with disorders

of consciousness. PLoS ONE. 2013;8(11). doi: 10.1371/journal.pone.0080479.

481. Indovino S, Maccora S, Baschi R, Puma A, Cosentino G. Visuospatial ability in healthy subjects and in patients with left or right hemisphere damage. Acta Medica Mediterranea. 2013;29(2):209-12.

482. Ji RR, Berta T, Nedergaard M. Glia and pain: Is chronic pain a gliopathy? Pain. 2013;154(SUPPL. 1):S10-S28. doi: 10.1016/j.pain.2013.06.022.

483. Kirton A. Can noninvasive brain stimulation measure and modulate developmental plasticity to improve function in stroke-induced cerebral palsy? Seminars in Pediatric Neurology. 2013;20(2):116-26. doi: 10.1016/j.spen.2013.06.004.

484. Kulkarni J, Gavrilidis E, Worsley R, Rheenen TV, Hayes E. The role of estrogen in the treatment of men with schizophrenia. International Journal of Endocrinology and Metabolism. 2013;11(3):129-36. doi: 10.5812/ijem.6615.

485. Kwon HG, Choi BY, Chang CH, Kim SH, Jung YJ, Jang SH. Recovery of an injured corticospinal tract during a critical period in a patient with intracerebral hemorrhage. NeuroRehabilitation. 2013;32(1):27-32. doi: 10.3233/NRE-130820.

486. La Rubia M, Rus A, Molina F, del Moral ML. Is

fibromyalgia-related oxidative stress implicated in the decline of physical and mental health status? Clinical and Experimental Rheumatology. 2013;31(SUPPL.79):S121-S7.

487. Lamy JC, Boakye M. BDNF Val66Met polymorphism alters spinal DC stimulation-induced plasticity in humans. Journal of Neurophysiology. 2013;110(1):109-16. doi: 10.1152/jn.00116.2013.

488. Liley DTJ, Walsh M. The mesoscopic modelling of burst suppression during anaesthesia. Frontiers in Computational Neuroscience. 2013(APR 2013). doi: 10.3389/fncom.2013.00046.

489. Lloréns R, Colomer-Font C, Alcañiz M, Noé-Sebastián E. BioTrak virtual reality system: Effectiveness and satisfaction analysis for balance rehabilitation in patients with brain injury. Neurologia. 2013;28(5):268-75. doi: 10.1016/j.nrl.2012.04.016.

490. Machado A. New frontier: The brain machine interface. Neuromodulation. 2013;16(1):6-7. doi: 10.1111/ner.12021.

491. Manganotti P, Del Felice A. New perspectives in transcranial magnetic stimulation: Epilepsy, consciousness and the perturbational approach. Behavioural Neurology. 2013;27(2):155-67. doi: 10.3233/BEN-2012-120263.

492. Mańko G, Olszewski H, Krawczyński M, Tłokiński W. Evaluation of differentiated neurotherapy programs for patients

recovering from severe TBI and long term coma. Acta Neuropsychologica. 2013;11(1):9-18.

493. Mateer CA. Reconceptualizing brain injury rehabilitation in the future: A peek over the horizon. Journal of Head Trauma Rehabilitation. 2013;28(3):232-6. doi: 10.1097/HTR.0b013e318291b046.

494. Michou E, Mistry S, Rothwell J, Hamdy S. Priming pharyngeal motor cortex by repeated paired associative stimulation: Implications for dysphagia neurorehabilitation. Neurorehabilitation and Neural Repair. 2013;27(4):355-62. doi: 10.1177/1545968312469837.

495. Müller-Dahlhaus F. Induction and increase of cortico-cortical reorganisation post stroke. Klinische Neurophysiologie. 2013;44(4):257-62. doi: 10.1055/s-0033-1357213.

496. Muscari A, Puddu GM, Serafini C, Fabbri E, Vizioli L, Zoli M. Predictors of short-term improvement of ischemic stroke. Neurological Research. 2013;35(6):594-601. doi: 10.1179/1743132813Y.0000000181.

497. Nudo RJ. Recovery after brain injury: Mechanisms and principles. Frontiers in Human Neuroscience. 2013;7(DEC). doi: 10.3389/fnhum.2013.00887.

498. Othmer S, Othmer SF, Kaiser DA, Putman J. Endogenous

neuromodulation at infralow frequencies. Seminars in Pediatric Neurology. 2013;20(4):246-57. doi: 10.1016/j.spen.2013.10.006.

499. Picht T, Krieg SM, Sollmann N, Rösler J, Niraula B, Neuvonen T, et al. A comparison of language mapping by preoperative navigated transcranial magnetic stimulation and direct cortical stimulation during awake surgery. Neurosurgery. 2013;72(5):808-19. doi: 10.1227/NEU.0b013e3182889e01.

500. Rajkowska G, Stockmeier CA. Astrocyte pathology in major depressive disorder: Insights from human postmortem brain tissue. Current Drug Targets. 2013;14(11):1225-36. doi: 10.2174/13894501113149990156.

501. Richter L, Trillenberg P, Schweikard A, Schlaefer A. Stimulus intensity for hand held and robotic transcranial magnetic stimulation. Brain Stimulation. 2013;6(3):315-21. doi: 10.1016/j.brs.2012.06.002.

502. Saroka KS, Persinger MA. Potential production of Hughlings Jackson's "parasitic consciousness" by physiologically-patterned weak transcerebral magnetic fields: QEEG and source localization. Epilepsy and Behavior. 2013;28(3):395-407. doi: 10.1016/j.yebeh.2013.05.023.

503. Shaqiri A, Anderson B. Priming and statistical learning in right brain damaged patients. Neuropsychologia.

2013;51(13):2526-33. doi: 10.1016/j.neuropsychologia.2013.09.024.
504. Shetty AK. Prospects of levetiracetam as a neuroprotective drug against status epilepticus, traumatic brain injury, and stroke. Frontiers in Neurology. 2013;4 NOV. doi: 10.3389/fneur.2013.00172.
505. Sung KW, Son MH, Lee SH, Yoo KH, Koo HH, Kim JY, et al. Tandem high-dose chemotherapy and autologous stem cell transplantation in patients with high-risk neuroblastoma: Results of SMC NB-2004 study. Bone Marrow Transplantation. 2013;48(1):68-73. doi: 10.1038/bmt.2012.86.
506. Tsaousides T, Ashman TA, Gordon WA. Diagnosis and treatment of depression following traumatic brain injury. Brain Impairment. 2013;14(1):63-76. doi: 10.1017/BrImp.2013.8.
507. Van der Aa NE, Verhage CH, Groenendaal F, Vermeulen RJ, De Bode S, Van Nieuwenhuizen O, et al. Neonatal neuroimaging predicts recruitment of contralesional corticospinal tracts following perinatal brain injury. Developmental Medicine and Child Neurology. 2013;55(8):707-12. doi: 10.1111/dmcn.12160.
508. Varelas PN, Spanaki MV, Mirski MA. Status epilepticus: An update. Current Neurology and Neuroscience Reports. 2013;13(7). doi: 10.1007/s11910-013-0357-0.

509. White NM, Packard MG, McDonald RJ. Dissociation of memory systems: The story unfolds. Behavioral Neuroscience. 2013;127(6):813-34. doi: 10.1037/a0034859.
510. Amico E, Gomez F, Di Perri C, Vanhaudenhuyse A, Lesenfants D, Boveroux P, et al. Posterior cingulate cortex-related co-activation patterns: A resting state fMRI study in propofol-induced loss of consciousness. PLoS ONE. 2014;9(6). doi: 10.1371/journal.pone.0100012.
511. Bécsi B, Kiss A, Erdodi F. Interaction of protein phosphatase inhibitors with membrane lipids assessed by surface plasmon resonance based binding technique. Chemistry and Physics of Lipids. 2014;183:68-76. doi: 10.1016/j.chemphyslip.2014.05.009.
512. Bolton DAE, Staines WR. Attention-based modulation of tactile stimuli: A comparison between prefrontal lesion patients and healthy age-matched controls. Neuropsychologia. 2014;57(1):101-11. doi: 10.1016/j.neuropsychologia.2014.03.003.
513. Chen L, Zhu Y, Yang D, Zou R, Wu J, Tian H. Synthesis and antibacterial activities of antibacterial peptides with a spiropyran fluorescence probe. Scientific Reports. 2014;4. doi: 10.1038/srep06860.
514. Chennu S, Finoia P, Kamau E, Allanson J, Williams GB, Monti MM, et al. Spectral Signatures of Reorganised Brain

Networks in Disorders of Consciousness. PLoS Computational Biology. 2014;10(10). doi: 10.1371/journal.pcbi.1003887.

515. Corbetta M. Hemispatial neglect: Clinic, pathogenesis, and treatment. Seminars in Neurology. 2014;34(5):514-23. doi: 10.1055/s-0034-1396005.

516. Dominguez LG, Radhu N, Farzan F, Daskalakis ZJ. Characterizing long interval cortical inhibition over the time-frequency domain. PLoS ONE. 2014;9(3). doi: 10.1371/journal.pone.0092354.

517. Farzan F. Single-pulse transcranial magnetic stimulation (TMS) protocols and outcome measures. Neuromethods2014. p. 69-115.

518. Finsterer J, Stöllberger C, Güler N. Non-compaction delineates amyotrophic lateral sclerosis from metabolic myopathy. International Journal of Cardiology. 2014;176(1):277-9. doi: 10.1016/j.ijcard.2014.06.092.

519. Gaede G, Hellweg R, Zimmermann H, Brandt AU, Dörr J, Bellmann-Strobl J, et al. Effects of deep repetitive transcranial magnetic stimulation on brain-derived neurotrophic factor serum concentration in healthy volunteers. Neuropsychobiology. 2014;69(2):112-9. doi: 10.1159/000358088.

520. Gimeno H, Tustin K, Lumsden D, Ashkan K, Selway R, Lin

JP. Evaluation of functional goal outcomes using the Canadian Occupational Performance Measure (COPM) following Deep Brain Stimulation (DBS) in childhood dystonia. European Journal of Paediatric Neurology. 2014;18(3):308-16. doi: 10.1016/j.ejpn.2013.12.010.
521. Gobbo M, Falciati L. Neuromodulation and cognitive rehabilitation: Addressing the methodological issue of circadian rhythms. Frontiers in Psychiatry. 2014;5(OCT). doi: 10.3389/fpsyt.2014.00150.
522. Gonullu H, Aslan M, Karadas S, Kat C, Duran L, Milanlioglu A, et al. Serum prolidase enzyme activity and oxidative stress levels in patients with acute hemorrhagic stroke. Scandinavian Journal of Clinical and Laboratory Investigation. 2014;74(3):199-205. doi: 10.3109/00365513.2013.873949.
523. Hu H, Zhou Y, Leng T, Liu A, Wang Y, You X, et al. The major cholesterol metabolite cholestane-3β,5α,6β-triol functions as an endogenous neuroprotectant. Journal of Neuroscience. 2014;34(34):11426-38. doi: 10.1523/JNEUROSCI.0344-14.2014.
524. Jennings S, Russell N, Jennings B, Slee V, Sterling L, Castells M, et al. The mastocytosis society survey on mast cell disorders: Patient experiences and perceptions. Journal of Allergy and Clinical Immunology: In Practice. 2014;2(1):70-6. doi:

10.1016/j.jaip.2013.09.004.
525. Jiménez-Ortega L, García-Milla M, Fondevila S, Casado P, Hernández-Gutiérrez D, Martín-Loeches M. Automaticity of higher cognitive functions: Neurophysiological evidence for unconscious syntactic processing of masked words. Biological Psychology. 2014;103:83-91. doi: 10.1016/j.biopsycho.2014.08.011.
526. Klein PA, Petitjean C, Olivier E, Duque J. Top-down suppression of incompatible motor activations during response selection under conflict. NeuroImage. 2014;86:138-49. doi: 10.1016/j.neuroimage.2013.08.005.
527. Kronenburg A, Van Doormaal T, Van Eijsden P, Van Der Zwan A, Leijten F, Han KS. Surgery for a giant arteriovenous malformation without motor deterioration: Preoperative transcranial magnetic stimulation in a non-cooperative patient: Case report. Journal of Neurosurgery: Pediatrics. 2014;14(1):38-42. doi: 10.3171/2014.4.PEDS13592.
528. Kwon HG, Jang SH. Delayed gait disturbance due to injury of the corticoreticular pathway in a patient with mild traumatic brain injury. Brain Injury. 2014;28(4):511-4. doi: 10.3109/02699052.2014.887228.
529. Lau BK, Vaughan CW. Targeting the endogenous cannabinoid system to treat neuropathic pain. Frontiers in Pharmacology.

2014;5 MAR. doi: 10.3389/fphar.2014.00028.
530. León-Carrión J, León-Domínguez U, Halper J, Pollonini L, Zouridakis G, Domínguez-Morales MR. Restoring cortical connectivity directionality and synchronization is essential to treating disorder of consciousness. Current Pharmaceutical Design. 2014;20(26):4268-74.
531. Li H, Zhang T. A synopsis of recent influential papers published in mental health journals (2012-2013) in Mainland China. Asian Journal of Psychiatry. 2014;10:105-8. doi: 10.1016/j.ajp.2014.01.012.
532. McCullagh P, Lightbody G, Zygierewicz J, Kernohan WG. Ethical challenges associated with the development and deployment of brain computer interface technology. Neuroethics. 2014;7(2):109-22. doi: 10.1007/s12152-013-9188-6.
533. Minter MR, Zhang M, Ates RC, Taylor JM, Crack PJ. Type-1 interferons contribute to oxygen glucose deprivation induced neuro-inflammation in BE(2)M17 human neuroblastoma cells. Journal of Neuroinflammation. 2014;11. doi: 10.1186/1742-2094-11-43.
534. Mitchell AS, Murray Sherman S, Sommer MA, Mair RG, Vertes RP, Chudasama Y. Advances in understanding mechanisms of thalamic relays in cognition and behavior. Journal of

Neuroscience. 2014;34(46):15340-6. doi: 10.1523/JNEUROSCI.3289-14.2014.

535. Moeller DR, Duffey JM, Goolsby AM, Gallimore JT. Use of a Removable Mandibular Neuroprosthesis for the Reduction of Posttraumatic Stress Disorder (PTSD) and Mild Traumatic Brain Injury/PTSD/Associated Nightmares, Headaches, and Sleep Disturbances. Journal of special operations medicine : a peer reviewed journal for SOF medical professionals. 2014;14(3):64-73.

536. Morton DL, El-Deredy W, Jones AK. Placebo analgesia: Cognition or perception. Handbook of Experimental Pharmacology2014. p. 71-80.

537. O'Neill B, Gardani M, Findlay G, Whyte T, Cullen T. Challenging behaviour and sleep cycle disorder following brain injury: A preliminary response to agomelatine treatment. Brain Injury. 2014;28(3):378-81. doi: 10.3109/02699052.2013.865264.

538. Pąchalska M, Buliński L, Jauer-Niworowska O, Rasmus A, Daniluk B, Mirski A, et al. Neuropsychological functioning after traumatic injury to the orbito-frontal area: A Polish Phineas Gage. Acta Neuropsychologica. 2014;12(4):503-16. doi: 10.5604/17307503.1138851.

539. Preston C, Newport R. Noisy visual feedback training impairs detection of self-generated movement error: Implications for

anosognosia for hemiplegia. Frontiers in Human Neuroscience. 2014;8(JUNE). doi: 10.3389/fnhum.2014.00456.
540. Reddy A, Cho J, Ling S, Reddy V, Shlykov M, Saier MH. Reliability of nine programs of topological predictions and their application to integral membrane channel and carrier proteins. Journal of Molecular Microbiology and Biotechnology. 2014;24(3):161-90. doi: 10.1159/000363506.
541. Rezai AR, Sharma M. Deep Brain Stimulation (DBS): Current and emerging applications. Japanese Journal of Neurosurgery. 2014;23(8):648-60. doi: 10.7887/jcns.23.648.
542. Roberts L, Cacace AT. Jendrassik maneuver facilitates cVEMP amplitude: Some preliminary observations. Journal of the American Academy of Audiology. 2014;25(3):237-43. doi: 10.3766/jaaa.25.3.2.
543. Roebuck H, Bourke P, Guo K. Role of lateral and feedback connections in primary visual cortex in the processing of spatiotemporal regularity - A TMS study. Neuroscience. 2014;263:231-9. doi: 10.1016/j.neuroscience.2014.01.027.
544. Sachs AJ, Babu H, Su YF, Miller KJ, Henderson JM. Lack of efficacy of motor cortex stimulation for the treatment of neuropathic pain in 14 patients. Neuromodulation. 2014;17(4):303-11. doi: 10.1111/ner.12181.

545. Solbakk AK, Funderud I, Løvstad M, Endestad T, Meling T, Lindgren M, et al. Impact of orbitofrontal lesions on electrophysiological signals in a stop signal task. Journal of Cognitive Neuroscience. 2014;26(7):1528-45. doi: 10.1162/jocn_a_00561.
546. Spreij LA, Visser-Meily JMA, van Heugten CM, Nijboer TCW. Novel insights into the rehabilitation of memory post acquired brain injury: A systematic review. Frontiers in Human Neuroscience. 2014;8(DEC). doi: 10.3389/fnhum.2014.00993.
547. Strigo IA, Spadoni AD, Lohr J, Simmons AN. Too hard to control: Compromised pain anticipation and modulation in mild traumatic brain injury. Translational Psychiatry. 2014;4. doi: 10.1038/tp.2013.116.
548. Tran MTD, Skovbjerg S, Arendt-Nielsen L, Bech P, Lunde M, Elberling J. Two of three patients with multiple chemical sensitivity had less symptoms and secondary hyperalgesia after transcranially applied pulsed electromagnetic fields. Scandinavian Journal of Pain. 2014;5(2):104-9. doi: 10.1016/j.sjpain.2013.11.008.
549. Uhrig L, Dehaene S, Jarraya B. Cerebral mechanisms of general anesthesia. Annales Francaises d'Anesthesie et de Reanimation. 2014;33(2):72-82. doi: 10.1016/j.annfar.2013.11.005.

550. Urgesi C, Candidi M, Avenanti A. Neuroanatomical substrates of action perception and understanding: An anatomic likelihood estimation meta-analysis of lesion-symptom mapping studies in brain injured patients. Frontiers in Human Neuroscience. 2014;8(MAY). doi: 10.3389/fnhum.2014.00344.
551. Volman V, Ng LJ. Primary paranode demyelination modulates slowly developing axonal depolarization in a model of axonal injury. Journal of Computational Neuroscience. 2014;37(3):439-57. doi: 10.1007/s10827-014-0515-7.
552. Waninger KN, Gloyeske BM, Hauth JM, Vanic KA, Yen DM. Intratympanic hemorrhage and concussion in a football offensive lineman. Journal of Emergency Medicine. 2014;46(3):371-2. doi: 10.1016/j.jemermed.2013.08.043.
553. Weatherall A, Garner A, Lovell N, Redmond S, Lee A, Skowno J, et al. Study protocol for the PHANTOM study: Prehospital assessment of noninvasive tissue oximetry monitoring. Scandinavian Journal of Trauma, Resuscitation and Emergency Medicine. 2014;22(1). doi: 10.1186/s13049-014-0057-z.
554. Wojtecki L, Petri D, Elben S, Hirschmann J, Yelnik J, Eickhoff S, et al. Modulation of central thalamic oscillations during emotional-cognitive processing in chronic disorder of consciousness. Cortex. 2014;60:94-102. doi:

10.1016/j.cortex.2014.09.007.
555. Wu Q, Marescaux C, Qin X, Kessler R, Yang J. Heterogeneity of radiological spectrum in tacrolimus-associated encephalopathy after lung transplantation. Behavioural Neurology. 2014;2014. doi: 10.1155/2014/931808.
556. Zhang X, Fu LD, Geng YH, Zhai X, Liu YH. Analysis of the effect of repeated-pulse transcranial magnetic stimulation at the Guangming point on electroencephalograms. Neural Regeneration Research. 2014;9(5):549-54. doi: 10.4103/1673-5374.130082.
557. Zimerman M, Heise KF, Gerloff C, Cohen LG, Hummel FC. Disrupting the ipsilateral motor cortex interferes with training of a complex motor task in older adults. Cerebral Cortex. 2014;24(4):1030-6. doi: 10.1093/cercor/bhs385.
558. Ayata C, Lauritzen M. Spreading depression, spreading depolarizations, and the cerebral vasculature. Physiological Reviews. 2015;95(3):953-93. doi: 10.1152/physrev.00027.2014.
559. Bhaskaran A, Barry MAT, I. Al Raisi S, Chik W, Nguyen DT, Pouliopoulos J, et al. Magnetic guidance versus manual control: comparison of radiofrequency lesion dimensions and evaluation of the effect of heart wall motion in a myocardial phantom. Journal of Interventional Cardiac Electrophysiology. 2015;44(1):1-8. doi: 10.1007/s10840-015-0023-3.

560. Blume C, del Giudice R, Wislowska M, Lechinger J, Schabus M. Across the consciousness continuum—from unresponsive wakefulness to sleep. Frontiers in Human Neuroscience. 2015;9(MAR). doi: 10.3389/fnhum.2015.00105.

561. Burnstock G. Physiopathological roles of P2X receptors in the central nervous system. Current Medicinal Chemistry. 2015;22(7):819-44. doi: 10.2174/0929867321666140706130415.

562. Carman AJ, Ferguson R, Cantu R, Comstock RD, Dacks PA, Dekosky ST, et al. Expert consensus document: Mind the gaps-advancing research into short-term and long-term neuropsychological outcomes of youth sports-related concussions. Nature Reviews Neurology. 2015;11(4):230-44. doi: 10.1038/nrneurol.2015.30.

563. Chen GS, Huang KF, Huang CC, Wang JY. Thaliporphine derivative improves acute lung injury after traumatic brain injury. BioMed Research International. 2015;2015. doi: 10.1155/2015/729831.

564. Cipollari S, Veniero D, Razzano C, Caltagirone C, Koch G, Marangolo P. Combining TMS-EEG with transcranial direct current stimulation language treatment in aphasia. Expert Review of Neurotherapeutics. 2015;15(7):833-45. doi: 10.1586/14737175.2015.1049998.

565. Costa V, Lugert S, Jagasia R. Role of adult hippocampal neurogenesis in cognition in physiology and disease: pharmacological targets and biomarkers. Handbook of Experimental Pharmacology2015. p. 99-155.

566. Defrin R, Riabinin M, Feingold Y, Schreiber S, Pick CG. Deficient pain modulatory systems in patients with mild traumatic brain and chronic post-traumatic headache: Implications for its mechanism. Journal of Neurotrauma. 2015;32(1):28-37. doi: 10.1089/neu.2014.3359.

567. Durka PJ, Malinowska U, Zieleniewska M, O'Reilly C, Różański PT, Żygierewicz J. Spindles in Svarog: Framework and software for parametrization of EEG transients. Frontiers in Human Neuroscience. 2015;9(MAY). doi: 10.3389/fnhum.2015.00258.

568. Fecteau S, Dickler M, Pelayo R, Kumru H, Bernabeu M, Opisso Salleras E, et al. Cortical Excitability during Passive Action Observation in Hospitalized Adults with Subacute Moderate to Severe Traumatic Brain Injury. Neurorehabilitation and Neural Repair. 2015;29(6):548-56. doi: 10.1177/1545968314558603.

569. Fischer DB, Truog RD. What is a reflex? A guide for understanding disorders of consciousness. Neurology. 2015;85(6):543-8. doi: 10.1212/WNL.0000000000001748.

570. Fritz JV, McVige J, Mechtler L. Coding in behavioral

neurology and neuropsychiatry. CONTINUUM Lifelong Learning in Neurology. 2015;21(3):844-60. doi: 10.1212/01.CON.0000466672.94853.19.

571. Gentile A, Mori F, Bernardini S, Centonze D. Role of amyloid-β CSF levels in cognitive deficit in MS. Clinica Chimica Acta. 2015;449:23-30. doi: 10.1016/j.cca.2015.01.035.

572. Goldsworthy MR, Pitcher JB, Ridding MC. Spaced Noninvasive Brain Stimulation: Prospects for Inducing Long-Lasting Human Cortical Plasticity. Neurorehabilitation and Neural Repair. 2015;29(8):714-21. doi: 10.1177/1545968314562649.

573. Heise CW, Beutler D, Bosak A, Orme G, Loli A, Graeme K. Massive Atenolol, Lisinopril, and Chlorthalidone Overdose Treated with Endoscopic Decontamination, Hemodialysis, Impella Percutaneous Left Ventricular Assist Device, and ECMO. Journal of Medical Toxicology. 2015;11(1):110-4. doi: 10.1007/s13181-014-0419-y.

574. Hilz MJ, Aurnhammer F, Flanagan SR, Intravooth T, Wang R, Hösl KM, et al. Eyeball Pressure Stimulation Unveils Subtle Autonomic Cardiovascular Dysfunction in Persons with a History of Mild Traumatic Brain Injury. Journal of Neurotrauma. 2015;32(22):1796-804. doi: 10.1089/neu.2014.3842.

575. Ji G, Liu M, Zhao XF, Liu XY, Guo QL, Guan ZF, et al. NF-κB Signaling is Involved in the Effects of Intranasally Engrafted Human Neural Stem Cells on Neurofunctional Improvements in Neonatal Rat Hypoxic-Ischemic Encephalopathy. CNS Neuroscience and Therapeutics. 2015;21(12):926-35. doi: 10.1111/cns.12441.

576. Karuppagounder SS, Kumar A, Shao DS, Zille M, Bourassa MW, Caulfield JT, et al. Metabolism and epigenetics in the nervous system: Creating cellular fitness and resistance to neuronal death in neurological conditions via modulation of oxygen-, iron-, and 2-oxoglutarate-dependent dioxygenases. Brain Research. 2015;1628:273-87. doi: 10.1016/j.brainres.2015.07.030.

577. Ketelslegers IA, Van Pelt ED, Bryde S, Neuteboom RF, Catsman-Berrevoets CE, Hamann D, et al. Anti-MOG antibodies plead against MS diagnosis in an Acquired Demyelinating Syndromes cohort. Multiple Sclerosis. 2015;21(12):1513-20. doi: 10.1177/1352458514566666.

578. Kim SK, Nam SJ, Jang H, Kim A, Lee JJ. TM-MC: A database of medicinal materials and chemical compounds in Northeast Asian traditional medicine. BMC Complementary and Alternative Medicine. 2015;15(1). doi: 10.1186/s12906-015-0758-5.

579. Kornfeld S, Rodríguez JAD, Everts R, Kaelin-Lang A, Wiest R, Weisstanner C, et al. Cortical reorganisation of cerebral networks after childhood stroke: Impact on outcome. BMC Neurology. 2015;15(1). doi: 10.1186/s12883-015-0309-1.

580. Leong SR, Liang WC, Wu Y, Crocker L, Cheng E, Sampath D, et al. An Anti-B7-H4 antibody-drug conjugate for the treatment of breast cancer. Molecular Pharmaceutics. 2015;12(6):1717-29. doi: 10.1021/mp5007745.

581. Liao Z, Pu T, Gu H, Liu W. Coupled motion of cervical spine in three level hybrid constructs. Bio-Medical Materials and Engineering. 2015;26:S637-S45. doi: 10.3233/BME-151355.

582. Lin SC, Brown RE, Shuler MGH, Petersen CCH, Kepecs A. Optogenetic dissection of the basal forebrain neuromodulatory control of cortical activation, plasticity, and cognition. Journal of Neuroscience. 2015;35(41):13896-903. doi: 10.1523/JNEUROSCI.2590-15.2015.

583. Liu CY, Law M, Romano R. Rational approach to understanding and preventing sports-related traumatic brain injuries. World Neurosurgery. 2015;84(6):1556-7. doi: 10.1016/j.wneu.2015.07.076.

584. Liu Z, Lee Y, Jang JH, Li Y, Han X, Yokoi K, et al. Microfluidic cytometric analysis of cancer cell transportability and

invasiveness. Scientific Reports. 2015;5. doi: 10.1038/srep14272.
585. Lorente L, Martín MM, Almeida T, Abreu-González P, Ramos L, Argueso M, et al. Total antioxidant capacity is associated with mortality of patients with severe traumatic brain injury. BMC Neurology. 2015;15(1). doi: 10.1186/s12883-015-0378-1.
586. Luauté J, Morlet D, Mattout J. BCI in patients with disorders of consciousness: Clinical perspectives. Annals of Physical and Rehabilitation Medicine. 2015;58(1):29-34. doi: 10.1016/j.rehab.2014.09.015.
587. Marek Gołe'biewski W, Cyrta M, Michalczyk A. Synthesis and fungistatic activity of aryl aldoxime derivatives. Journal de Mycologie Medicale. 2015;25(1):50-6. doi: 10.1016/j.mycmed.2014.10.026.
588. Marins TF, Rodrigues EC, Engel A, Hoefle S, Basílio R, Lent R, et al. Enhancing motor network activity using real-time functional mri neurofeedback of left premotor cortex. Frontiers in Behavioral Neuroscience. 2015;9(DEC). doi: 10.3389/fnbeh.2015.00341.
589. Palhano-Fontes F, Andrade KC, Tofoli LF, Jose ACS, Crippa AS, Hallak JEC, et al. The psychedelic state induced by Ayahuasca modulates the activity and connectivity of the Default Mode Network. PLoS ONE. 2015;10(2). doi:

10.1371/journal.pone.0118143.

590. Pathak R, Shao L, Ghosh SP, Zhou D, Boerma M, Weiler H, et al. Thrombomodulin contributes to gamma tocotrienol-mediated lethality protection and hematopoietic cell recovery in irradiated mice. PLoS ONE. 2015;10(4). doi: 10.1371/journal.pone.0122511.

591. Richardson SP, Tinaz S, Chen R. Repetitive transcranial magnetic stimulation in cervical dystonia: Effect of site and repetition in a randomized pilot trial. PLoS ONE. 2015;10(4). doi: 10.1371/journal.pone.0124937.

592. Sánchez-Porras R, Zheng Z, Sakowitz OW. Pharmacological modulation of spreading depolarizations. Acta Neurochirurgica, Supplementum2015. p. 153-7.

593. Sato N, Morishita R. The roles of lipid and glucose metabolism in modulation of β-amyloid, tau, and neurodegeneration in the pathogenesis of Alzheimer disease. Frontiers in Aging Neuroscience. 2015;7(OCT). doi: 10.3389/fnagi.2015.00199.

594. Shyshov AS, Petrova IS, Grishina TP, Frolova KS, Blank IA, Inozemtseva AB, et al. Cerebrospinal fluid in clinical aspects of tuberculosis of the central nervous system. Zhurnal Nevrologii i Psihiatrii imeni SS Korsakova. 2015;2015(3):17-24. doi: 10.17116/jnevro20151153117-24.

595. Spagnuolo MS, Mollica MP, Maresca B, Cavaliere G, Cefaliello C, Trinchese G, et al. High fat diet and inflammation – Modulation of haptoglobin level in rat brain. Frontiers in Cellular Neuroscience. 2015;9(DEC). doi: 10.3389/fncel.2015.00479.
596. Wilkinson L, Steel A, Mooshagian E, Zimmermann T, Keisler A, Lewis JD, et al. Online feedback enhances early consolidation of motor sequence learning and reverses recall deficit from transcranial stimulation of motor cortex. Cortex. 2015;71:134-47. doi: 10.1016/j.cortex.2015.06.012.
597. Xu T, Zhang WG, Sun J, Zhang Y, Lu JF, Han HB, et al. Protective effects of thrombomodulin on microvascular permeability after subarachnoid hemorrhage in mouse model. Neuroscience. 2015;299:18-27. doi: 10.1016/j.neuroscience.2015.04.058.
598. Zakhvataev VE. Possible scenarios of the influence of low-dose ionizing radiation on neural functioning. Medical Hypotheses. 2015;85(6):723-35. doi: 10.1016/j.mehy.2015.10.020.
599. Zhou J, Zhou C, Li J, Zhang M. Cognitive style modulates conscious but not unconscious thought: Comparing the deliberation-without-attention effect in analytics and wholists. Consciousness and Cognition. 2015;36:54-60. doi: 10.1016/j.concog.2015.05.018.

600. Zhou X, Wang YH, Ghosh S, Tang WF, Pang BB, Liu MQ, et al. Genomic characterization of G3P[6], G4P[6] and G4P[8] human rotaviruses from Wuhan, China: Evidence for interspecies transmission and reassortment events. Infection, Genetics and Evolution. 2015;33:55-71. doi: 10.1016/j.meegid.2015.04.010.

601. A Skutchanova A, Saniova B. The Enigma of Consciousness - Philosophic and Scientific Approach. Acta Medica Martiniana. 2016;16(2):37-43. doi: 10.1515/acm-2016-0010.

602. Balazs S, Kermanshahi K, Binder H, Rattay F, Bodis-Wollner I. Gamma-Band Modulation and Coherence in the EEG by Involuntary Eye Movements in Patients in Unresponsive Wakefulness Syndrome. Clinical EEG and Neuroscience. 2016;47(3):196-206. doi: 10.1177/1550059415601756.

603. Biagi L, Cioni G, Fogassi L, Guzzetta A, Sgandurra G, Tosetti M. Action observation network in childhood: a comparative fMRI study with adults. Developmental Science. 2016;19(6):1075-86. doi: 10.1111/desc.12353.

604. Burnett CJ, Krashes MJ. Resolving behavioral output via chemogenetic designer receptors exclusively activated by designer drugs. Journal of Neuroscience. 2016;36(36):9268-82. doi: 10.1523/JNEUROSCI.1333-16.2016.

605. Charbit J, Lakhal K, Deras P, Dehon A, Latry P, Boissier E, et

al. Influence of surgical bleeding on the relationship between admission coagulopathy and risk of massive transfusion: lesson from 704 severe trauma patients. Vox Sanguinis. 2016;111(2):151-60. doi: 10.1111/vox.12401.

606. Colella M, Biran V, Baud O. Melatonin and the newborn brain. Early Human Development. 2016;102:1-3. doi: 10.1016/j.earlhumdev.2016.09.001.

607. de Tommaso M. Response: Commentary: Cortical responses to salient nociceptive and not nociceptive stimuli in vegetative and minimal conscious state. Frontiers in Human Neuroscience. 2016;10(JAN2016). doi: 10.3389/fnhum.2016.00012.

608. Del Bigio MR, Di Curzio DL. Nonsurgical therapy for hydrocephalus: A comprehensive and critical review. Fluids and Barriers of the CNS. 2016;13(1). doi: 10.1186/s12987-016-0025-2.

609. Dubljević V. 21 Selected Abstracts from the Montreal Neuroethics Conference for Young Researchers. Neuroethics. 2016;9(2):137-45. doi: 10.1007/s12152-016-9262-y.

610. Elzamarany E, Afifi L, El-Fayoumy NM, Salah H, Nada M. Motor cortex rTMS improves dexterity in relapsing-remitting and secondary progressive multiple sclerosis. Acta Neurologica Belgica. 2016;116(2):145-50. doi: 10.1007/s13760-015-0540-y.

611. Flamez A, Cordenier A, De Raedt S, Michiels V, Smetcoren S,

Van Merhaegen-Wieleman A, et al. Bilateral low frequency rTMS of the primary motor cortex may not be a suitable treatment for levodopa-induced dyskinesias in late stage Parkinson's disease. Parkinsonism and Related Disorders. 2016;22:62-7. doi: 10.1016/j.parkreldis.2015.11.009.

612. González-Saldaña N, Hernández-Porras M, Macías-Parra M, Monroy-Colín VA, Acebo-Arcentales JJ, Juárez-Olguín H. Tuberculous meningitis: Symptoms, diagnosis and evaluation experienced in 532 patients in a pediatric hospital. Asian Pacific Journal of Tropical Disease. 2016;6(3):208-11. doi: 10.1016/S2222-1808(15)61015-5.

613. Goulet-Kennedy J, Labbe S, Fecteau S. The involvement of the striatum in decision making. Dialogues in Clinical Neuroscience. 2016;18(1):55-63.

614. Grecco LAC, Oliveira CS, Galli M, Cosmo C, Duarte NAC, Zanon N, et al. Spared primary motor cortex and the presence of MEP in cerebral palsy dictate the responsiveness to tDCS during gait training. Frontiers in Human Neuroscience. 2016;10. doi: 10.3389/fnhum.2016.00361.

615. Gu JJ, Chen JB, Zhang JH, Zhang H, Wang SS. Recombinant human soluble thrombomodulin protects against brain injury in a CVST rat model, via downregulation of the HMGB1-RAGE axis.

Molecular Medicine Reports. 2016;14(6):5217-22. doi: 10.3892/mmr.2016.5891.

616. Hampel KG, Jahanbekam A, Elger CE, Surges R. Seizure-related modulation of systemic arterial blood pressure in focal epilepsy. Epilepsia. 2016;57(10):1709-18. doi: 10.1111/epi.13504.

617. Heiss WD. Imaging effects related to language improvements by rTMS. Restorative Neurology and Neuroscience. 2016;34(4):531-6. doi: 10.3233/RNN-150631.

618. Kang IS, Lee W, Choi BW, Choi D, Hong MK, Jang Y, et al. Semiquantitative assessment of tibial artery calcification by computed tomography angiography and its ability to predict infrapopliteal angioplasty outcomes. Journal of Vascular Surgery. 2016;64(5):1335-43. doi: 10.1016/j.jvs.2016.04.047.

619. Keane KM, Haskell-Ramsay CF, Veasey RC, Howatson G. Montmorency Tart cherries (Prunus cerasus L.) modulate vascular function acutely, in the absence of improvement in cognitive performance. British Journal of Nutrition. 2016;116(11):1935-44. doi: 10.1017/S0007114516004177.

620. Kim MO, Adji A, O'Rourke MF, Avolio AP, Smielewski P, Pickard JD, et al. Change in pulsatile cerebral arterial pressure and flow waves as a therapeutic strategy? Acta Neurochirurgica,

Supplementum2016. p. 167-70.

621. Leung A, Fallah A, Shukla S, Lin L, Tsia A, Song D, et al. RTMS in alleviating mild TBI related headaches – A case series. Pain Physician. 2016;19(2):E347-E53.

622. Li J, Meng XM, Li RY, Zhang R, Zhang Z, Du YF. Effects of different frequencies of repetitive transcranial magnetic stimulation on the recovery of upper limb motor dysfunction in patients with subacute cerebral infarction. Neural Regeneration Research. 2016;11(10):1584-90. doi: 10.4103/1673-5374.193236.

623. Lorente L, Martín MM, Pérez-Cejas A, Abreu-González P, Ramos L, Argueso M, et al. Association between total antioxidant capacity and mortality in ischemic stroke patients. Annals of Intensive Care. 2016;6(1). doi: 10.1186/s13613-016-0143-7.

624. Magrone T, Russo MA, Jirillo E. Role of immune cells in the course of central nervous system injury: Modulation with natural products. Current Pharmaceutical Design. 2016;22(6):701-8. doi: 10.2174/1381612822666151204000959.

625. Matsushima K, Inaba K, Cho J, Mohammed H, Herr K, Leichtle S, et al. Therapeutic anticoagulation in patients with traumatic brain injury. Journal of Surgical Research. 2016;205(1):186-91. doi: 10.1016/j.jss.2016.06.042.

626. Montenegro RA, Farinatti PTV, De Lima PFM, Okano AH,

Menêses AL, De Oliveira-Neto L, et al. Motor cortex tDCS does not modulate perceived exertion within multiple-sets of resistance exercises. Isokinetics and Exercise Science. 2016;24(1):17-24. doi: 10.3233/IES-150593.

627. Naim-Feil J, Bradshaw JL, Rogasch NC, Daskalakis ZJ, Sheppard DM, Lubman DI, et al. Cortical inhibition within motor and frontal regions in alcohol dependence post-detoxification: A pilot TMS-EEG study. World Journal of Biological Psychiatry. 2016;17(7):547-56. doi: 10.3109/15622975.2015.1066512.

628. Naros G, Geyer M, Koch S, Mayr L, Ellinger T, Grimm F, et al. Enhanced motor learning with bilateral transcranial direct current stimulation: Impact of polarity or current flow direction? Clinical Neurophysiology. 2016;127(4):2119-26. doi: 10.1016/j.clinph.2015.12.020.

629. Okie S. TBI's long-term follow-up-slow progress in science and recovery. New England Journal of Medicine. 2016;375(2):180-4. doi: 10.1056/NEJMms1604272.

630. Rabie A, Metman LV, Fakhry M, Eassa AYE, Fouad W, Shakal A, et al. Improvement of advanced Parkinson’s disease manifestations with deep brain stimulation of the subthalamic nucleus: A single institution experience. Brain Sciences. 2016;6(4). doi: 10.3390/brainsci6040058.

631. Ranft A, Golkowski D, Kiel T, Riedl V, Kohl P, Rohrer G, et al. Neural Correlates of Sevoflurane-induced Unconsciousness Identified by Simultaneous Functional Magnetic Resonance Imaging and Electroencephalography. Anesthesiology. 2016;125(5):861-72. doi: 10.1097/ALN.0000000000001322.
632. Raucci U, Tomasello C, Marri M, Salzano M, Gasparini A, Conicella E. Scrambler Therapy® MC-5A for Complex Regional Pain Syndrome: Case Reports. Pain Practice. 2016;16(7):E103-E9. doi: 10.1111/papr.12474.
633. Sbicigo JB, Piccolo LR, Becker N, Vedana SN, Rodrigues JC, Salles JF, et al. Current perspectives on the anatomo-clinical method in neuropsychology. Psychology and Neuroscience. 2016;9(2):198-214. doi: 10.1037/pne0000050.
634. Shehata HS, Esmail EH, Abdelalim A, El-Jaafary S, Elmazny A, Sabbah A, et al. Repetitive transcranial magnetic stimulation versus botulinum toxin injection in chronic migraine prophylaxis: A pilot randomized trial. Journal of Pain Research. 2016;9:771-7. doi: 10.2147/JPR.S116671.
635. Singh H, Cooper R, Lee CW, Dempsey L, Brigadoi S, Edwards A, et al. Neurovascular interactions in the neurologically compromised neonatal brain. Advances in Experimental Medicine and Biology2016. p. 485-92.

636. Sollmann N, Kubitscheck A, Maurer S, Ille S, Hauck T, Kirschke JS, et al. Preoperative language mapping by repetitive navigated transcranial magnetic stimulation and diffusion tensor imaging fiber tracking and their comparison to intraoperative stimulation. Neuroradiology. 2016;58(8):807-18. doi: 10.1007/s00234-016-1685-y.
637. Suntrup-Krueger S, Bittner S, Recker S, Meuth SG, Warnecke T, Suttrup I, et al. Electrical pharyngeal stimulation increases substance P level in saliva. Neurogastroenterology and Motility. 2016;28(6):855-60. doi: 10.1111/nmo.12783.
638. Takechi K, Hori S, Yamashiro K, Yuki T, Hori A. A case of successful drug-eluting microsphere transcatheter arterial embolization for hepatocellular carcinoma with associated tumor thrombus in the inferior vena cava and right atrium. Japanese Journal of Cancer and Chemotherapy. 2016;43(11):1385-8.
639. Tan CSH, Ho CFY, Heng SS, Wu JS, Tan BKH, Ng YK, et al. Clinacanthus nutans Extracts Modulate Epigenetic Link to Cytosolic Phospholipase A2 Expression in SH-SY5Y Cells and Primary Cortical Neurons. NeuroMolecular Medicine. 2016;18(3):441-52. doi: 10.1007/s12017-016-8404-z.
640. Thiel A, Zumbansen A. The pathophysiology of post-stroke aphasia: A network approach. Restorative Neurology and

Neuroscience. 2016;34(4):507-18. doi: 10.3233/RNN-150632.

641. Tsujita-Inoue K, Hirota M, Atobe T, Ashikaga T, Tokura Y, Kouzuki H. Development of novel in vitro photosafety assays focused on the Keap1-Nrf2-ARE pathway. Journal of Applied Toxicology. 2016;36(7):956-68. doi: 10.1002/jat.3234.

642. Valenza MC, Torres-Sánchez I, Cabrera-Martos I, Valenza-Demet G, Cano-Cappellacci M. Acute Effects of Contract-Relax Stretching vs. TENS in Young Subjects with Anterior Knee Pain: A Randomized Controlled Trial. Journal of Strength and Conditioning Research. 2016;30(8):2271-8. doi: 10.1097/JSC.0000000000000215.

643. Watson JC, Sandroni P. Central Neuropathic Pain Syndromes. Mayo Clinic Proceedings. 2016;91(3):372-85. doi: 10.1016/j.mayocp.2016.01.017.

644. Yang B, Wang Z, Sheng C, Wang Y, Zhou J, Xiong XG, et al. Evidence-based review of oral traditional Chinese medicine compound recipe administration for treating weight drop-induced experimental traumatic brain injury. BMC Complementary and Alternative Medicine. 2016;16(1). doi: 10.1186/s12906-016-1076-2.

645. Yang Y, Cheng X, Xu Q, Li R, Liu Z, Wang L, et al. The maintenance of modified electroconvulsive therapy combined with

risperidone is better than risperidone alone in preventing relapse of schizophrenia and improving cognitive function. Arquivos de Neuro-Psiquiatria. 2016;74(10):823-8. doi: 10.1590/0004-282X20160130.

646. Zarea A, Charbonnier C, Rovelet-Lecrux A, Nicolas G, Rousseau S, Borden A, et al. Seizures in dominantly inherited Alzheimer disease. Neurology. 2016;87(9):912-9. doi: 10.1212/WNL.0000000000003048.

647. Zong H, Ma F, Zhang L, Lu H, Gong J, Cai M, et al. Hindlimb spasticity after unilateral motor cortex lesion in rats is reduced by contralateral nerve root transfer. Bioscience Reports. 2016;36(6). doi: 10.1042/BSR20160412.

648. Andrabi SS, Parvez S, Tabassum H. Neurosteroids and ischemic stroke: Progesterone a promising agent in reducing the brain injury in Ischemic stroke. Journal of Environmental Pathology, Toxicology and Oncology. 2017;36(3):191-205. doi: 10.1615/JEnvironPatholToxicolOncol.2017017156.

649. Antal A, Paulus W, Rohde V. New Results on Brain Stimulation in Chronic Pain. Aktuelle Neurologie. 2017;44(10):728-32. doi: 10.1055/s-0043-119975.

650. Bilotta F, Lauretta MP, Tewari A, Haque M, Hara N, Uchino H, et al. Insulin and the Brain : A Sweet Relationship with Intensive

Care. Journal of Intensive Care Medicine. 2017;32(1):48-58. doi: 10.1177/0885066615594341.

651. Bivona G, Sasso BL, Agnello L, Ciaccio M. From the blood-brain barrier to behavior. Acta Neuropsychologica. 2017;15(1):63-7. doi: 10.5604/12321966.1237451.

652. Bognár A, Csete G, Németh M, Csibri P, Kincses TZ, Sáry G. Transcranial stimulation of the orbitofrontal cortex affects decisions about magnocellular optimized stimuli. Frontiers in Neuroscience. 2017;11(APR). doi: 10.3389/fnins.2017.00234.

653. Branscheidt M, Hoppe J, Freundlieb N, Zwitserlood P, Liuzzi G. tDCS over the motor cortex shows differential effects on action and object words in associative word learning in healthy aging. Frontiers in Aging Neuroscience. 2017;9(MAY). doi: 10.3389/fnagi.2017.00137.

654. Chantsoulis M, Półrola P, Góral-Półrola J, Hajdukiewicz A, Supiński J, Kropotov JD, et al. Application of ERPs neuromarkers for assessment and treatment of a patient with chronic crossed aphasia after severe TBI and long-term coma – Case report. Annals of Agricultural and Environmental Medicine. 2017;24(1):141-7. doi: 10.5604/12321966.1232770.

655. Cho IT, Cho YW, Kwak SG, Chang MC. Comparison between ultrasound-guided interfascial pulsed radiofrequency and

ultrasound-guided interfascial block with local anesthetic in myofascial pain syndrome of trapezius muscle. Medicine (United States). 2017;96(5). doi: 10.1097/MD.0000000000006019.

656. D'Imperio D, Tomelleri G, Moretto G, Moro V. Modulation of somatoparaphrenia following left-hemisphere damage. Neurocase. 2017;23(2):162-70. doi: 10.1080/13554794.2017.1329444.

657. Demuth HU, Dijkhuizen RM, Farr TD, Gelderblom M, Horsburgh K, Iadecola C, et al. Recent progress in translational research on neurovascular and neurodegenerative disorders. Restorative Neurology and Neuroscience. 2017;35(1):87-103. doi: 10.3233/RNN-160690.

658. Fomina T, Weichwald S, Synofzik M, Just J, Schols L, Scholkopf B, et al. Absence of EEG correlates of self-referential processing depth in ALS. PLoS ONE. 2017;12(6). doi: 10.1371/journal.pone.0180136.

659. Garcia-Rill E. Bottom-up gamma and stages of waking. Medical Hypotheses. 2017;104:58-62. doi: 10.1016/j.mehy.2017.05.023.

660. Ginanneschi F, Mignarri A, Lucchiari S, Ulzi G, Comi GP, Rossi A, et al. Neuromuscular excitability changes produced by sustained voluntary contraction and response to mexiletine in myotonia congenita. Neurophysiologie Clinique.

2017;47(3):247-52. doi: 10.1016/j.neucli.2017.01.003.
661. Goadsby PJ, Holland PR, Martins-Oliveira M, Hoffmann J, Schankin C, Akerman S. Pathophysiology of migraine: A disorder of sensory processing. Physiological Reviews. 2017;97(2):553-622. doi: 10.1152/physrev.00034.2015.
662. Higginson CI, Thompson T, Benjamin AE, Rosales AC, Zane KL, Valenti M, et al. Construct validity of the Functional Assessment of Verbal Reasoning and Executive Strategies (FAVRES). Brain Injury. 2017;31(13-14):1807-12. doi: 10.1080/02699052.2017.1347278.
663. Jobst BC, Kapur R, Barkley GL, Bazil CW, Berg MJ, Bergey GK, et al. Brain-responsive neurostimulation in patients with medically intractable seizures arising from eloquent and other neocortical areas. Epilepsia. 2017;58(6):1005-14. doi: 10.1111/epi.13739.
664. Kraeutner SN, Ingram TGJ, Boe SG. The effector independent nature of motor imagery: Evidence from rTMS induced inhibition to the primary motor cortices. Neuropsychologia. 2017;97:1-8. doi: 10.1016/j.neuropsychologia.2017.01.023.
665. Lee JW, Lee S, Cho HW, Ma Y, Yoo KH, Sung KW, et al. Incorporation of high-dose 131I-metaiodobenzylguanidine treatment into tandem high-dose chemotherapy and autologous

stem cell transplantation for high-risk neuroblastoma: results of the SMC NB-2009 study. Journal of Hematology and Oncology. 2017;10(1). doi: 10.1186/s13045-017-0477-0.

666. Lorente L, Martín MM, Abreu-González P, Pérez-Cejas A, Ramos L, Argueso M, et al. Serum melatonin levels in survivor and non-survivor patients with traumatic brain injury. BMC Neurology. 2017;17(1). doi: 10.1186/s12883-017-0922-2.

667. Lu CC, Dong SL, Lin HH, Ni YC, Jan ML, Chuang KS. Noninvasive measurement of radiopharmaceutical time-activity data using external thermoluminescent dosimeters (TLDs). Physics in Medicine and Biology. 2017;62(4):N58-N72. doi: 10.1088/1361-6560/aa54a9.

668. Meng ZY, Song WQ. Low frequency repetitive transcranial magnetic stimulation improves motor dysfunction after cerebral infarction. Neural Regeneration Research. 2017;12(4):610-3. doi: 10.4103/1673-5374.205100.

669. Moisset X. Noninvasive brain stimulation: a new therapeutic option in chronic pain? Douleur et Analgesie. 2017;30(4):192-8. doi: 10.1007/s11724-017-0532-1.

670. Moore N, Patel B, Zuabi N, Langdorf MI, Rodriguez RM. Feasibility of Informed Consent for Computed Tomography in Acute Trauma Patients. Academic Emergency Medicine.

2017;24(5):637-40. doi: 10.1111/acem.13164.

671. Nagy A, Lipoldová J, Novák M, Štěpánová R. Occurrence of implantable cardioverter-defibrillator therapy in clinical practice. Cor et Vasa. 2017;59(3):e215-e21. doi: 10.1016/j.crvasa.2016.12.014.

672. Naros G, Gharabaghi A. Physiological and behavioral effects of β-tACS on brain self-regulation in chronic stroke. Brain Stimulation. 2017;10(2):251-9. doi: 10.1016/j.brs.2016.11.003.

673. Oakley E, May R, Hoeppner T, Sinn K, Furyk J, Craig S, et al. Computed tomography for head injuries in children: Change in Australian usage rates over time. EMA - Emergency Medicine Australasia. 2017;29(2):192-7. doi: 10.1111/1742-6723.12732.

674. Odegaard B, Knight RT, Lau H. Should a few null findings falsify prefrontal theories of conscious perception? Journal of Neuroscience. 2017;37(40):9593-602. doi: 10.1523/JNEUROSCI.3217-16.2017.

675. Ohi K, Kuwata A, Shimada T, Yasuyama T, Nitta Y, Uehara T, et al. Response to benzodiazepines and the clinical course in malignant catatonia associated with schizophrenia: A case report. Medicine (United States). 2017;96(16). doi: 10.1097/MD.0000000000006566.

676. Okamura Y, Mishima S, Kashiwakura JI, Sasaki-Sakamoto T,

Toyoshima S, Kuroda K, et al. The dual regulation of substance P-mediated inflammation via human synovial mast cells in rheumatoid arthritis. Allergology International. 2017;66:S9-S20. doi: 10.1016/j.alit.2017.03.002.

677. Oliveira-Maia AJ, Press D, Pascual-Leone A. Modulation of motor cortex excitability predicts antidepressant response to prefrontal cortex repetitive transcranial magnetic stimulation. Brain Stimulation. 2017;10(4):787-94. doi: 10.1016/j.brs.2017.03.013.

678. Ortenzi M, Ghiselli R, Cardinali L, Guerrieri M. Surgical treatment of gastric stromal tumors: laparoscopic versus open approach. Annali italiani di chirurgia. 2017;88.

679. Rajczak E, Pecoraro VL, Juskowiak B. Sm(iii)[12-MCGa(III)shi-4] as a luminescent probe for G-quadruplex structures. Metallomics. 2017;9(12):1735-44. doi: 10.1039/c7mt00232g.

680. Saadipour K, MacLean M, Pirkle S, Ali S, Lopez-Redondo ML, Stokes DL, et al. The transmembrane domain of the p75 neurotrophin receptor stimulates phosphorylation of the TrkB tyrosine kinase receptor. Journal of Biological Chemistry. 2017;292(40):16594-604. doi: 10.1074/jbc.M117.788729.

681. Sato K, Kobayashi Y, Nakamura A, Fukushima D, Satomi S. Early post-transplant hyperbilirubinemia is a possible predictive

factor for developing neurological complications in pediatric living donor liver transplant patients receiving tacrolimus. Pediatric Transplantation. 2017;21(2). doi: 10.1111/petr.12843.

682. Sender NS, Govindan RB, Whitehead MT, Massaro AN, Metzler M, Wang J, et al. Cerebral modulation of the autonomic nervous system in term infants. Journal of Perinatology. 2017;37(5):558-62. doi: 10.1038/jp.2016.248.

683. Soplata AE, McCarthy MM, Sherfey J, Lee S, Purdon PL, Brown EN, et al. Thalamocortical control of propofol phase-amplitude coupling. PLoS Computational Biology. 2017;13(12). doi: 10.1371/journal.pcbi.1005879.

684. Tendler A, Roth Y, Barnea-Ygael N, Zangen A. How to use the H1 deep transcranial magnetic stimulation coil for conditions other than depression. Journal of Visualized Experiments. 2017;2017(119). doi: 10.3791/55100.

685. Theus MH, Brickler T, Meza AL, Coutermarsh-Ott S, Hazy A, Gris D, et al. Loss of NLRX1 exacerbates neural tissue damage and NF-κB signaling following brain injury. Journal of Immunology. 2017;199(10):3547-58. doi: 10.4049/jimmunol.1700251.

686. Wilkerson GB, Grooms DR, Acocello SN. Neuromechanical Considerations for Postconcussion Musculoskeletal Injury Risk Management. Current Sports Medicine Reports. 2017;16(6):419-27.

doi: 10.1249/JSR.0000000000000430.
687. Winkelman MJ. The mechanisms of psychedelic visionary experiences: Hypotheses from evolutionary psychology. Frontiers in Neuroscience. 2017;11(SEP). doi: 10.3389/fnins.2017.00539.
688. Young LR, Yu W, Holloway M, Rodgers BN, Chapman SB, Krawczyk DC. Amygdala activation as a marker for selective attention toward neutral faces in a chronic traumatic brain injury population. Neuropsychologia. 2017;104:214-22. doi: 10.1016/j.neuropsychologia.2017.08.026.
689. Aima M, DeWerd LA, Mitch MG, Hammer CG, Culberson WS. Dosimetric characterization of a new directional low-dose rate brachytherapy source. Medical Physics. 2018;45(8):3848-60. doi: 10.1002/mp.12994.
690. Alkanfari I, Gupta K, Jahan T, Ali H. Naturally occurring missense MRGPRX2 variants display loss of function phenotype for mast cell degranulation in response to substance P, hemokinin-1, human b-defensin-3, and icatibant. Journal of Immunology. 2018;201(2):343-9. doi: 10.4049/jimmunol.1701793.
691. Baltus A, Wagner S, Wolters CH, Herrmann CS. Optimized auditory transcranial alternating current stimulation improves individual auditory temporal resolution. Brain Stimulation. 2018;11(1):118-24. doi: 10.1016/j.brs.2017.10.008.

692. Branscheidt M, Hoppe J, Zwitserlood P, Liuzzi G. TDCS over the motor cortex improves lexical retrieval of action words in poststroke aphasia. Journal of Neurophysiology. 2018;119(2):621-30. doi: 10.1152/jn.00285.2017.
693. Catić-Đorđević A, Pavlović I, Pavlović D, Stefanović N, Mikov M, Cvetković T, et al. Evaluation of gender-based limited sampling methods for tacrolimus exposure after renal transplantation using the Monte Carlo simulation. Pharmazie. 2018;73(8):482-5. doi: 10.1691/ph.2018.8461.
694. de Assis Aquino Gondim F, Bastante P, Gondim WC, Filha JGH, Thomas FP. Medical and neuropsychiatric phenomena depicted in two Spanish medieval texts of Marian miracles. Neurological Sciences. 2018;39(3):565-9. doi: 10.1007/s10072-017-3183-3.
695. Demeulenaere M, Devreese K, Vanbelleghem H, De Zaeytijd J, Vande Walle J, Van Biesen W, et al. Thrombomodulin and Endothelial Dysfunction: A Disease-Modifier Shared between Malignant Hypertension and Atypical Hemolytic Uremic Syndrome. Nephron. 2018;140(1):63-73. doi: 10.1159/000490201.
696. Dieguez S, Kaeser M, Roux C, Cottet J, Annoni JM, Schmidlin E. Birth and death of a phantom. Annals of Clinical and Translational Neurology. 2018;5(1):98-101. doi: 10.1002/acn3.495.

697. Faried A, Ramdan A, Arifin MZ, Nataprawira HM. Characteristics and surgical outcomes of tuberculous meningitis and of tuberculous spondylitis in pediatric patients at Dr. Hasan Sadikin Hospital, Bandung: A single center experience. Interdisciplinary Neurosurgery: Advanced Techniques and Case Management. 2018;11:37-40. doi: 10.1016/j.inat.2017.09.008.
698. Finley J. Transposable elements, placental development, and oocyte activation: Cellular stress and AMPK links jumping genes with the creation of human life. Medical Hypotheses. 2018;118:44-54. doi: 10.1016/j.mehy.2018.05.015.
699. Fouzia NA, Edison ES, Lakshmi KM, Korula A, Velayudhan SR, Balasubramanian P, et al. Long-term outcome of mixed chimerism after stem cell transplantation for thalassemia major conditioned with busulfan and cyclophosphamide. Bone Marrow Transplantation. 2018;53(2):169-74. doi: 10.1038/bmt.2017.231.
700. Fox KCR, Yih J, Raccah O, Pendekanti SL, Limbach LE, Maydan DD, et al. Changes in subjective experience elicited by direct stimulation of the human orbitofrontal cortex. Neurology. 2018;91(16):E1519-E27. doi: 10.1212/WNL.0000000000006358.
701. Gallagher CN, Esser MJ, Nguyen R, Casha S, Sutherland G, Yeates KO. Neurotrauma Research at the University of Calgary. Journal of Neurotrauma. 2018;35(16):1990-2.

702. Gallasch E, Rafolt D, Postruznik M, Fresnoza S, Christova M. Decrease of motor cortex excitability following exposure to a 20 Hz magnetic field as generated by a rotating permanent magnet. Clinical Neurophysiology. 2018;129(7):1397-402. doi: 10.1016/j.clinph.2018.03.045.

703. Geraldo AF, Pereira J, Nunes P, Reimão S, Sousa R, Castelo-Branco M, et al. Beyond fractional anisotropy in amyotrophic lateral sclerosis: the value of mean, axial, and radial diffusivity and its correlation with electrophysiological conductivity changes. Neuroradiology. 2018;60(5):505-15. doi: 10.1007/s00234-018-2012-6.

704. Gincberg G, Shohami E, Trembovler V, Alexandrovich AG, Lazarovici P, Elchalal U. Nerve growth factor plays a role in the neurotherapeutic effect of a CD45 + pan-hematopoietic subpopulation derived from human umbilical cord blood in a traumatic brain injury model. Cytotherapy. 2018;20(2):245-61. doi: 10.1016/j.jcyt.2017.11.008.

705. Hamoudi M, Schambra HM, Fritsch B, Schoechlin-Marx A, Weiller C, Cohen LG, et al. Transcranial Direct Current Stimulation Enhances Motor Skill Learning but Not Generalization in Chronic Stroke. Neurorehabilitation and Neural Repair. 2018;32(4-5):295-308. doi: 10.1177/1545968318769164.

706. Irvine KA, David Clark J. Chronic pain after traumatic brain injury: Pathophysiology and pain mechanisms. Pain Medicine (United States). 2018;19(7):1315-33. doi: 10.1093/pm/pnx153.
707. Jasey N, Ward I, Lequerica A, Chiaravalloti ND. The therapeutic value of cranioplasty in individuals with brain injury. Brain Injury. 2018;32(3):318-24. doi: 10.1080/02699052.2017.1419283.
708. Johansson M, Ricci F, Aung N, Sutton R, Melander O, Fedorowski A. Proteomic Profiling for Cardiovascular Biomarker Discovery in Orthostatic Hypotension. Hypertension. 2018;71(3):465-72. doi: 10.1161/HYPERTENSIONAHA.117.10365.
709. Kharchenko EP, Tel'nova MN. Brain Plasticity: Limitations and Possibilities. Neuroscience and Behavioral Physiology. 2018;48(5):603-7. doi: 10.1007/s11055-018-0606-4.
710. Khatoun A, Breukers J, De Beeck SO, Nica IG, Aerts JM, Seynaeve L, et al. Using high-amplitude and focused transcranial alternating current stimulation to entrain physiological tremor. Scientific Reports. 2018;8(1). doi: 10.1038/s41598-018-23290-w.
711. Kim C, Park JM, Kong TH, Lee S, Seo KW, Choi Y, et al. Double-Injected Human Stem Cells Enhance Rehabilitation in TBI Mice Via Modulation of Survival and Inflammation. Molecular

Neurobiology. 2018;55(6):4870-84. doi: 10.1007/s12035-017-0683-3.

712. Kim CH, Shin JE. Hemorrhage within the tympanic membrane without perforation. Journal of Otolaryngology - Head and Neck Surgery. 2018;47(1). doi: 10.1186/s40463-018-0300-0.

713. Maines M, Zorzi A, Tomasi G, Angheben C, Catanzariti D, Piffer L, et al. Clinical impact, safety, and accuracy of the remotely monitored implantable loop recorder Medtronic Reveal LINQ TM. Europace. 2018;20(6):1050-7. doi: 10.1093/europace/eux187.

714. Meiron O, Gale R, Namestnic J, Bennet-Back O, David J, Gebodh N, et al. High-Definition transcranial direct current stimulation in early onset epileptic encephalopathy: a case study. Brain Injury. 2018;32(1):135-43. doi: 10.1080/02699052.2017.1390254.

715. Naro A, Chillura A, Portaro S, Bramanti A, De Luca R, Bramanti P, et al. Novel approaches to the diagnosis of chronic disorders of consciousness: Detecting peripersonal space by using ultrasonics. Frontiers in Neurology. 2018;9(FEB). doi: 10.3389/fneur.2018.00047.

716. Portaro S, Naro A, Bramanti A, Leo A, Manuli A, Balletta T, et al. Beyond the muscular involvement in non-dystrophic myotonias: The emerging role of neuromodulation. Restorative

Neurology and Neuroscience. 2018;36(4):459-67. doi: 10.3233/RNN-170796.

717. Quinn DK, Mayer AR, Master CL, Fann JR. Prolonged postconcussive symptoms. American Journal of Psychiatry. 2018;175(2):103-11. doi: 10.1176/appi.ajp.2017.17020235.

718. Raij T, Nummenmaa A, Marin MF, Porter D, Furtak S, Setsompop K, et al. Prefrontal Cortex Stimulation Enhances Fear Extinction Memory in Humans. Biological Psychiatry. 2018;84(2):129-37. doi: 10.1016/j.biopsych.2017.10.022.

719. Raine A. Antisocial Personality as a Neurodevelopmental Disorder. Annual Review of Clinical Psychology2018. p. 259-89.

720. Ramirez-Zamora A, Giordano JJ, Gunduz A, Brown P, Sanchez JC, Foote KD, et al. Evolving applications, technological challenges and future opportunities in neuromodulation: Proceedings of the fifth annual deep brain stimulation think tank. Frontiers in Neuroscience. 2018;11(JAN). doi: 10.3389/fnins.2017.00734.

721. Rivera-Lillo G, Rojas-Líbano D, Burgos P, Egaña JI, Chennu S, Maldonado PE. Reduced delta-band modulation underlies the loss of P300 responses in disorders of consciousness. Clinical Neurophysiology. 2018;129(12):2613-22. doi: 10.1016/j.clinph.2018.09.104.

722. Ryan K, Wawrzyn K, Gati JS, Chronik BA, Wong D, Duggal N, et al. 1H MR spectroscopy of the motor cortex immediately following transcranial direct current stimulation at 7 Tesla. PLoS ONE. 2018;13(8). doi: 10.1371/journal.pone.0198053.
723. Sanger TD, Liker M, Arguelles E, Deshpande R, Maskooki A, Ferman D, et al. Pediatric deep brain stimulation using awake recording and stimulation for target selection in, we inpatient neuromodulation monitoring unit. Brain Sciences. 2018;8(7). doi: 10.3390/brainsci8070135.
724. Sedaghat MR, Momeni-Moghaddam H, Naroo SS, Ghavamsaeedi H, Vahedi A. Dysfunctional lens syndrome. International Ophthalmology. 2018;38(4):1759-63. doi: 10.1007/s10792-017-0622-3.
725. Servia L, Serrano JCE, Pamplona R, Badia M, Montserrat N, Portero-Otin M, et al. Location-dependent effects of trauma on oxidative stress in humans. PLoS ONE. 2018;13(10). doi: 10.1371/journal.pone.0205519.
726. Smith AT, Gorassini MA. Hyperexcitability of brain stem pathways in cerebral palsy. Journal of Neurophysiology. 2018;120(3):1428-37. doi: 10.1152/jn.00185.2018.
727. Surowiecka-Pastewka A, Witkowski W, Kawecki M. A new triage method for burn disasters: Fast triage in burns (FTB).

Medical Science Monitor. 2018;24:1894-901. doi: 10.12659/MSM.905197.

728. Vitale A, Lai Q, Farinati F, Bucci L, Giannini EG, Napoli L, et al. Utility of Tumor Burden Score to Stratify Prognosis of Patients with Hepatocellular Cancer: Results of 4759 Cases from ITA.LI.CA Study Group. Journal of Gastrointestinal Surgery. 2018;22(5):859-71. doi: 10.1007/s11605-018-3688-y.

729. Wang T, Mantini D, Gillebert CR. The potential of real-time fMRI neurofeedback for stroke rehabilitation: A systematic review. Cortex. 2018;107:148-65. doi: 10.1016/j.cortex.2017.09.006.

730. Weinstein M, Green D, Rudisch J, Zielinski IM, Benthem-Muñiz M, Jongsma MLA, et al. Understanding the relationship between brain and upper limb function in children with unilateral motor impairments: A multimodal approach. European Journal of Paediatric Neurology. 2018;22(1):143-54. doi: 10.1016/j.ejpn.2017.09.012.

731. Woytowicz EJ, Sours C, Gullapalli RP, Rosenberg J, Westlake KP. Modulation of working memory load distinguishes individuals with and without balance impairments following mild traumatic brain injury. Brain Injury. 2018;32(2):191-9. doi: 10.1080/02699052.2017.1403045.

732. Yagi N, Itoh H, Hisamatsu T, Tomita Y, Kimura H, Fujii Y, et

al. A challenge for mutation specific risk stratification in long QT syndrome type 1. Journal of Cardiology. 2018;72(1):56-65. doi: 10.1016/j.jjcc.2017.12.011.
733. Yavari F, van Thriel C, Nitsche MA, Kuo MF. Effect of acute exposure to toluene on cortical excitability, neuroplasticity, and motor learning in healthy humans. Archives of Toxicology. 2018;92(10):3149-62. doi: 10.1007/s00204-018-2277-x.
734. Zarshenas S, Cullen N. Association between cognitive ability and functional outcomes following traumatic brain injury- using a computer-based neurocognitive battery. Brain Injury. 2018;32(13-14):1678-83. doi: 10.1080/02699052.2018.1517223.
735. Zhang XY, Sui YF, Guo TC, Wang SH, Hu Y, Lu YS. Effect of Paired Associative Stimulation on Motor Cortex Excitability in Rats. Current Medical Science. 2018;38(5):903-9. doi: 10.1007/s11596-018-1960-8.
736. Akyüz O, Bodakçi MN, Tefekli AH. Thermal cautery-assisted circumcision and principles of its use to decrease complication rates. Journal of Pediatric Urology. 2019;15(2):186.e1-.e8. doi: 10.1016/j.jpurol.2019.01.003.
737. Belkin VA, Pozdnyakov DG, Belkin AA. Diagnosis of the phenomenon of cognitive-motor dissociation in patients with chronic consciousness disorders. Nevrologiya, Neiropsikhiatriya,

Psikhosomatika. 2019;11:46-51. doi: 10.14412/2074-2711-2019-3S-46-51.
738. Brenner M, Messing A, Olsen ML. AP-1 and the injury response of the GFAP gene. Journal of Neuroscience Research. 2019;97(2):149-61. doi: 10.1002/jnr.24338.
739. Cacciola A, Naro A, Milardi D, Bramanti A, Malatacca L, Spitaleri M, et al. Functional brain network topology discriminates between patients with minimally conscious state and unresponsivewakefulness syndrome. Journal of Clinical Medicine. 2019;8(3). doi: 10.3390/jcm8030306.
740. Comolatti R, Pigorini A, Casarotto S, Fecchio M, Faria G, Sarasso S, et al. A fast and general method to empirically estimate the complexity of brain responses to transcranial and intracranial stimulations. Brain Stimulation. 2019;12(5):1280-9. doi: 10.1016/j.brs.2019.05.013.
741. Dodeja AK, Thomas C, Daniels CJ, Kertesz N, Kamp A. Detection of arrhythmias in adult congenital heart disease patients with LINQTM implantable loop recorder. Congenital Heart Disease. 2019;14(5):745-51. doi: 10.1111/chd.12815.
742. Etkin A, Maron-Katz A, Wu W, Fonzo GA, Huemer J, Vértes PE, et al. Using fMRI connectivity to define a treatment-resistant form of post-traumatic stress disorder. Science Translational

Medicine. 2019;11(486). doi: 10.1126/scitranslmed.aal3236.
743. Fernandes SR, Pereira M, Salvador R, Miranda PC, De Carvalho M. Cervical trans-spinal direct current stimulation: A modelling-experimental approach. Journal of NeuroEngineering and Rehabilitation. 2019;16(1). doi: 10.1186/s12984-019-0589-6.
744. Filla MS, Faralli JA, Desikan H, Peotter JL, Wannow AC, Peters DM. Activation of αvβ3 integrin alters fibronectin fibril formation in human trabecular meshwork cells in a ROCK-independent manner. Investigative Ophthalmology and Visual Science. 2019;60(12):3897-913. doi: 10.1167/iovs.19-27171.
745. Ghonimi NAM, Mahdy ME, Abdel Salam OA. Total Antioxidant Capacity Predicts Outcome in Acute Ischemic Stroke Subtypes in Egyptian Patients. Journal of Stroke and Cerebrovascular Diseases. 2019;28(7):1911-7. doi: 10.1016/j.jstrokecerebrovasdis.2019.03.053.
746. Goldman JS, Tort-Colet N, di Volo M, Susin E, Bouté J, Dali M, et al. Bridging Single Neuron Dynamics to Global Brain States. Frontiers in Systems Neuroscience. 2019;13. doi: 10.3389/fnsys.2019.00075.
747. Gupte R, Christian S, Keselman P, Habiger J, Brooks WM, Harris JL. Evaluation of taurine neuroprotection in aged rats with

traumatic brain injury. Brain Imaging and Behavior. 2019;13(2):461-71. doi: 10.1007/s11682-018-9865-5.

748. Hagelskjær V, Krohn M, Christensen PS, Christensen JR. Canadian occupational performance measure supported by talking mats: An evaluation of the clinical utility. Occupational Therapy International. 2019;2019. doi: 10.1155/2019/9367315.

749. Hall BR, Armijo PR, Leinicke JA, Langenfeld SJ, Oleynikov D. Prolonged non-operative management of clostridium difficile colitis is associated with increased mortality, complications, and cost. American Journal of Surgery. 2019;217(6):1042-6. doi: 10.1016/j.amjsurg.2019.01.017.

750. Hannah R, Iacovou A, Rothwell JC. Direction of TDCS current flow in human sensorimotor cortex influences behavioural learning. Brain Stimulation. 2019;12(3):684-92. doi: 10.1016/j.brs.2019.01.016.

751. Hansson JHS. A hypothesis regarding how sleep can calibrate neuronal excitability in the central nervous system and thereby offer stability, sensitivity and the best possible cognitive function. Medical Hypotheses. 2019;131. doi: 10.1016/j.mehy.2019.109307.

752. Henriques IAD, Lattari E, Torres G, Rodrigues GM, Oliveira BRR, Neto GAM, et al. Can transcranial direct current stimulation improve range of motion and modulate pain perception in healthy

individuals? Neuroscience Letters. 2019;707. doi: 10.1016/j.neulet.2019.134311.

753. Huo C, Li X, Jing J, Ma Y, Li W, Wang Y, et al. Median Nerve Electrical Stimulation–Induced Changes in Effective Connectivity in Patients With Stroke as Assessed With Functional Near-Infrared Spectroscopy. Neurorehabilitation and Neural Repair. 2019;33(12):1008-17. doi: 10.1177/1545968319875952.

754. Jha KA, Pentecost M, Lenin R, Gentry J, Klaic L, Del Mar N, et al. TSG-6 in conditioned media from adipose mesenchymal stem cells protects against visual deficits in mild traumatic brain injury model through neurovascular modulation. Stem Cell Research and Therapy. 2019;10(1). doi: 10.1186/s13287-019-1436-1.

755. Kalita J, Misra UK, Dubey AK. Role of Oxidative Stress in Tuberculous Meningitis: a Clinico-Radiological Correlation. Journal of Molecular Neuroscience. 2019;68(2):287-94. doi: 10.1007/s12031-019-01304-0.

756. Kaltiainen H, Liljeström M, Helle L, Salo A, Hietanen M, Renvall H, et al. Mild traumatic brain injury affects cognitive processing and modifies oscillatory brain activity during attentional tasks. Journal of Neurotrauma. 2019;36(14):2222-32. doi: 10.1089/neu.2018.6306.

757. Karamyan VT. Peptidase neurolysin is an endogenous

cerebroprotective mechanism in acute neurodegenerative disorders. Medical Hypotheses. 2019;131. doi: 10.1016/j.mehy.2019.109309.
758. Khammash D, Simmonite M, Polk TA, Taylor SF, Meehan SK. Probing short-latency cortical inhibition in the visual cortex with transcranial magnetic stimulation: A reliability study. Brain Stimulation. 2019;12(3):702-4. doi: 10.1016/j.brs.2019.01.013.
759. Kim SK, Lee S, Lee MK, Lee S. A systems pharmacology approach to investigate the mechanism of Oryeong-san formula for the treatment of hypertension. Journal of Ethnopharmacology. 2019;244. doi: 10.1016/j.jep.2019.112129.
760. Koh S, Ambrósio R, Jr., Inoue R, Maeda N, Miki A, Nishida K. Detection of subclinical corneal ectasia using corneal tomographic and biomechanical assessments in a Japanese population. Journal of Refractive Surgery. 2019;35(6):383-90. doi: 10.3928/1081597X-20190417-01.
761. Kulju T, Haapasalo J, Rainesalo S, Lehtimäki K, Peltola J. Autostimulation in Vagus Nerve Stimulator Treatment: Modulating Neuromodulation. Neuromodulation. 2019;22(5):630-7. doi: 10.1111/ner.12897.
762. Kulyk AV. The system of differentiated medical neurorehabilitation in the traumatic post-coma conditions of long-term disorders of consciousness. Acta Neuropsychologica.

2019;17(1):9-24. doi: 10.5604/01.3001.0013.1405.

763. Lee KM, Greenhalgh WM, Sargent P, Chae H, Klimp S, Engel S, et al. Unique Features of the US Department of Defense Multidisciplinary Concussion Clinics. Journal of Head Trauma Rehabilitation. 2019;34(6):402-8. doi: 10.1097/HTR.0000000000000526.

764. Lee M, Baird B, Gosseries O, Nieminen JO, Boly M, Postle BR, et al. Connectivity differences between consciousness and unconsciousness in non-rapid eye movement sleep: a TMS–EEG study. Scientific Reports. 2019;9(1). doi: 10.1038/s41598-019-41274-2.

765. Leopold A, Rumrill P, Hendricks DJ, Nardone A, Sampson E, Minton D, et al. A mixed-methodological examination of participant experiences, activities, and outcomes in a technology and employment project for postsecondary students with traumatic brain injuries. Journal of Vocational Rehabilitation. 2019;50(1):3-11. doi: 10.3233/JVR-180983.

766. Levi V, Cordella R, D’Ammando A, Tringali G, Dones I, Messina G, et al. Dorsal anterior cingulate cortex (ACC) deep brain stimulation (DBS): a promising surgical option for the treatment of refractory thalamic pain syndrome (TPS). Acta Neurochirurgica. 2019;161(8):1579-88. doi:

10.1007/s00701-019-03975-5.
767. Li D, Mashour GA. Cortical dynamics during psychedelic and anesthetized states induced by ketamine. NeuroImage. 2019;196:32-40. doi: 10.1016/j.neuroimage.2019.03.076.
768. Liu J, Fan Y, Kim D, Zhong T, Yi P, Fan C, et al. Neuroprotective effect of catechins derivatives isolated from Anhua dark tea on NMDA-induced excitotoxicity in SH-SY5Y cells. Fitoterapia. 2019;137. doi: 10.1016/j.fitote.2019.104240.
769. Mahncke HW, Kim SJ, Rose A, Stasio C, Buckley P, Caroff S, et al. Evaluation of a plasticity-based cognitive training program in schizophrenia: Results from the eCaesar trial. Schizophrenia Research. 2019;208:182-9. doi: 10.1016/j.schres.2019.03.006.
770. Marcó-García S, Ferrer-Quintero M, Usall J, Ochoa S, Del Cacho N, Huerta-Ramos E. Facial emotion recognition in neurological disorders: A narrative review. Revista de Neurologia. 2019;69(5):207-19. doi: 10.33588/rn.6905.2019047.
771. Meng H, Zhao H, Cao X, Hao J, Zhang H, Liu Y, et al. Double-negative T cells remarkably promote neuroinflammation after ischemic stroke. Proceedings of the National Academy of Sciences of the United States of America. 2019;116(12):5558-63. doi: 10.1073/pnas.1814394116.
772. Nemanich ST, Mueller BA, Gillick BT. Neurite orientation

dispersion and density imaging quantifies corticospinal tract microstructural organization in children with unilateral cerebral palsy. Human Brain Mapping. 2019;40(17):4888-900. doi: 10.1002/hbm.24744.

773. Park JS, Cho Y, You Y, Min JH, Jeong W, Ahn HJ, et al. Optimal timing to measure optic nerve sheath diameter as a prognostic predictor in post-cardiac arrest patients treated with targeted temperature management. Resuscitation. 2019;143:173-9. doi: 10.1016/j.resuscitation.2019.07.004.

774. Popescu M, Popescu EA, DeGraba TJ, Fernandez-Fidalgo DJ, Riedy G, Hughes JD. Post-traumatic stress disorder is associated with altered modulation of prefrontal alpha band oscillations during working memory. Clinical Neurophysiology. 2019;130(10):1869-81. doi: 10.1016/j.clinph.2019.06.227.

775. Rauca VF, Vlase L, Casian T, Sesarman A, Gheldiu AM, Mocan A, et al. Biologically active Ajuga species extracts modulate supportive processes for cancer cell development. Frontiers in Pharmacology. 2019;10(APR). doi: 10.3389/fphar.2019.00334.

776. Sa M, Singh R, Pujar S, D'Arco F, Desai N, Eltze C, et al. Centromedian thalamic nuclei deep brain stimulation and Anakinra treatment for FIRES – Two different outcomes. European Journal of Paediatric Neurology. 2019;23(5):749-54. doi:

10.1016/j.ejpn.2019.08.001.
777. Scott G, Carhart-Harris RL. Psychedelics as a treatment for disorders of consciousness. Neuroscience of Consciousness. 2019;2019(1). doi: 10.1093/nc/niz003.
778. Scott-King JM, Tieu S, Chiew AL, Lui J, Kirby KA, Chan BS. Clinical decision rule for non-traumatic computed tomography of the brain. EMA - Emergency Medicine Australasia. 2019;31(6):974-81. doi: 10.1111/1742-6723.13292.
779. Shelley-Tremblay JF, Eyer JC, Hill BD. A laboratory word memory test analogue differentiates intentional feigning from true responding using the p300 event-related potential. Brain Sciences. 2019;9(5). doi: 10.3390/brainsci9050109.
780. Tay EMY, Preisz P, Day RO. Role and impact of brain computed tomography in the management of drug overdoses and guideline recommendations. EMA - Emergency Medicine Australasia. 2019;31(6):1053-8. doi: 10.1111/1742-6723.13316.
781. Toccaceli G, Barbagallo G, Peschillo S. Low-intensity focused ultrasound for the treatment of brain diseases: Safety and feasibility. Theranostics. 2019;9(2):537-9. doi: 10.7150/thno.31765.
782. Wang N, Ke Y, Du J, Liu W, Kong L, Zhao X, et al., editors. High-Definition Transcranial Direct Current Stimulation (HD-tDCS) enhances working memory training. Proceedings of the

Annual International Conference of the IEEE Engineering in Medicine and Biology Society, EMBS; 2019.
783. Wang WT, Sun L, Sun CH. PDIA3-regulted inflammation and oxidative stress contribute to the traumatic brain injury (TBI) in mice. Biochemical and Biophysical Research Communications. 2019;518(4):657-63. doi: 10.1016/j.bbrc.2019.08.100.
784. Wilson RL, François M, Jankovic-Karasoulos T, McAninch D, McCullough D, Leifert WR, et al. Characterization of 5-methylcytosine and 5-hydroxymethylcytosine in human placenta cell types across gestation. Epigenetics. 2019;14(7):660-71. doi: 10.1080/15592294.2019.1609866.
785. Xu J, Branscheidt M, Schambra H, Steiner L, Widmer M, Diedrichsen J, et al. Rethinking interhemispheric imbalance as a target for stroke neurorehabilitation. Annals of Neurology. 2019;85(4):502-13. doi: 10.1002/ana.25452.
786. Xu Y, Li P, Zhang S, Wang Y, Zhao X, Wang X, et al. Cervical Spinal Cord Stimulation for the Vegetative State: A Preliminary Result of 12 Cases. Neuromodulation. 2019;22(3):347-54. doi: 10.1111/ner.12903.
787. Ye L, Zhang D, Shao M, Zhao P, Yin B, Zhuang J, et al. Lower posttraumatic α-synuclein level associated with altered default mode network connectivity following acute mild traumatic

brain injury. Frontiers in Neural Circuits. 2019;13. doi: 10.3389/fncir.2019.00026.

788. Zhang J, Shi C, Wang H, Gao C, Chang P, Chen X, et al. Hydrogen sulfide protects against cell damage through modulation of PI3K/Akt/Nrf2 signaling. International Journal of Biochemistry and Cell Biology. 2019;117. doi: 10.1016/j.biocel.2019.105636.

789. Zheng H, Zhang Z, Jiang S, Yan B, Shi X, Xie Y, et al. A shape-memory and spiral light-emitting device for precise multisite stimulation of nerve bundles. Nature Communications. 2019;10(1). doi: 10.1038/s41467-019-10418-3.

790. Al-Hussainy ADO, Al-Gazally ME, Ewadh W. Oxidative stress, Anti-melanocyte and anti tyrosinase antibody in vitiligo and response to treatment. Annals of Tropical Medicine and Public Health. 2020;23(5):500-7. doi: 10.36295/ASRO.2020.23524.

791. Arnsten AFT. Guanfacine's mechanism of action in treating prefrontal cortical disorders: Successful translation across species. Neurobiology of Learning and Memory. 2020;176. doi: 10.1016/j.nlm.2020.107327.

792. Bagnato S, Galardi G, Ribaudo F, Boccagni C, Fiorilla TV, Rubino F, et al. Serum BDNF Levels Are Reduced in Patients with Disorders of Consciousness and Are Not Modified by Verticalization with Robot-Assisted Lower-Limb Training. Neural

Plasticity. 2020;2020. doi: 10.1155/2020/5608145.

793. Baik SW, Kim GW, Ko MH, Seo JH, Won YH, Park SH. An Unusual, Intermediate-Sized Lesion Affecting Motor Organization in a Patient With Schizencephaly: A Case Report. Frontiers in Human Neuroscience. 2020;14. doi: 10.3389/fnhum.2020.00258.

794. Balcik ZE, Senadim S, Keskek A, Ozudogru A, Koksal A, Soysal A, et al. Does restricted diffusion in the splenium indicate an acute infarct? Acta Neurologica Belgica. 2020;120(5):1085-9. doi: 10.1007/s13760-017-0876-6.

795. Bernini A, Masoodi M, Solari D, Miroz JP, Carteron L, Christinat N, et al. Modulation of cerebral ketone metabolism following traumatic brain injury in humans. Journal of Cerebral Blood Flow and Metabolism. 2020;40(1):177-86. doi: 10.1177/0271678X18808947.

796. Buday J, Albrecht J, Podgorná G, Mareš T, Le TH, Čapek V, et al. Seizure threshold manipulation in electroconvulsive therapy via repetitive transcranial magnetic stimulation. A novel way of augmentation? Brain Stimulation. 2020;13(6):1631-8. doi: 10.1016/j.brs.2020.09.008.

797. Bulygin KV, Beeraka NM, Saitgareeva AR, Nikolenko VN, Gareev I, Beylerli O, et al. Can mirnas be considered as diagnostic and therapeutic molecules in ischemic stroke

pathogenesis?—current status. International Journal of Molecular Sciences. 2020;21(18):1-25. doi: 10.3390/ijms21186728.
798. Cassidy JM, Wodeyar A, Wu J, Kaur K, Masuda AK, Srinivasan R, et al. Low-Frequency Oscillations Are a Biomarker of Injury and Recovery after Stroke. Stroke. 2020:1442-50. doi: 10.1161/STROKEAHA.120.028932.
799. Castricum J, Tulen JHM, Taal W, Ottenhoff MJ, Kushner SA, Elgersma Y. Motor cortical excitability and plasticity in patients with neurofibromatosis type 1. Clinical Neurophysiology. 2020;131(11):2673-81. doi: 10.1016/j.clinph.2020.08.016.
800. Chiabi A, Djimafo ANM, Nguefack S, Mah E, Nguefack Dongmo F, Angwafo F, III. Severe malaria in Cameroon: Pattern of disease in children at the Yaounde Gynaeco-Obstetric and Pediatric hospital. Journal of Infection and Public Health. 2020;13(10):1469-72. doi: 10.1016/j.jiph.2020.02.038.
801. Chowdhury FA, Caciagli L, Whatley BP, McLaughlin C, Sanders B, Wehner T, et al. Preoperative language mapping using navigated TMS compared with extra-operative direct cortical stimulation using intracranial electrodes: A case report. Seizure. 2020;76:96-9. doi: 10.1016/j.seizure.2020.01.019.
802. de Almeida AC, Massote MA, Ichinose RM, Miranda de Sá AMFL. Spectral F Test for detecting TMS/EEG responses.

Biomedical Signal Processing and Control. 2020;58. doi: 10.1016/j.bspc.2019.101840.

803. Fong ZH, Sündermann O. Modulating disgust in mental contamination: Experimental evidence for the role of disgust. Journal of Behavior Therapy and Experimental Psychiatry. 2020;68. doi: 10.1016/j.jbtep.2020.101567.

804. Fornia L, Puglisi G, Leonetti A, Bello L, Berti A, Cerri G, et al. Direct electrical stimulation of the premotor cortex shuts down awareness of voluntary actions. Nature Communications. 2020;11(1). doi: 10.1038/s41467-020-14517-4.

805. Gao F, Ostrem JL, Wang DD. Treatment of post-hypoxic myoclonus using pallidal deep brain stimulation placed using interventional MRI methods. Tremor and Other Hyperkinetic Movements. 2020;10:1-6. doi: 10.5334/tohm.544.

806. Giummarra MJ, Murgatroyd D, Tran Y, Adie S, Mittal R, Ponsford J, et al. Health and return to work in the first two years following road traffic injury: a comparison of outcomes between compensation claimants in Victoria and New South Wales, Australia. Injury. 2020;51(10):2199-208. doi: 10.1016/j.injury.2020.07.002.

807. Gruen DS, Brown JB, Guyette FX, Vodovotz Y, Johansson PI, Stensballe J, et al. Prehospital plasma is associated with distinct

biomarker expression following injury. JCI Insight. 2020;5(8). doi: 10.1172/jci.insight.135350.

808. Gudmundsson L, Vohryzek J, Fornari E, Clarke S, Hagmann P, Crottaz-Herbette S. A brief exposure to rightward prismatic adaptation changes resting-state network characteristics of the ventral attentional system. PLoS ONE. 2020;15(6). doi: 10.1371/journal.pone.0234382.

809. Han JM, Hong KO, Yang IH, Ahn CH, Jin B, Lee W, et al. Oridonin induces the apoptosis of mucoepidermoid carcinoma cell lines in a myeloid cell leukemia-1-dependent manner. International Journal of Oncology. 2020;57(1):385-2020. doi: 10.3892/ijo.2020.5061.

810. Handy JD, Wright WG, Haskell A, Servatius L, Servatius RJ. Enhanced Acquisition and Retention of Conditioned Eyeblink Responses in Veterans Expressing PTSD Symptoms: Modulation by Lifetime History of Mild Traumatic Brain Injury. Frontiers in Behavioral Neuroscience. 2020;14. doi: 10.3389/fnbeh.2020.595007.

811. Harter K, Hammel G, Fleming M, Traidl-Hoffmann C. Multiple chemical sensitivity (MCS) – a guide for dermatologists on how to manage affected individuals. JDDG - Journal of the German Society of Dermatology. 2020;18(2):119-30. doi:

10.1111/ddg.14027.
812. Hill NM, Dewald JPA. The Upper Extremity Flexion Synergy Is Minimally Expressed in Young Individuals With Unilateral Cerebral Palsy Following an Early Brain Injury. Frontiers in Human Neuroscience. 2020;14. doi: 10.3389/fnhum.2020.590198.
813. Hollis A, Zewdie E, Nettel-Aguirre A, Hilderley A, Kuo HC, Carlson HL, et al. Transcranial Static Magnetic Field Stimulation of the Motor Cortex in Children. Frontiers in Neuroscience. 2020;14. doi: 10.3389/fnins.2020.00464.
814. Huang Y, Hu K, Green AL, Ma X, Gillies MJ, Wang S, et al. Dynamic changes in rhythmic and arrhythmic neural signatures in the subthalamic nucleus induced by anaesthesia and tracheal intubation. British Journal of Anaesthesia. 2020;125(1):67-76. doi: 10.1016/j.bja.2020.03.014.
815. Iazeva EG, Legostaeva LA, Bakulin IS, Poydasheva AG, Abaimov DA, Suponeva NA, et al. Effect of neuromodulation on neurotrophic factors in patients with chronic disorders of conciousness. Bulletin of Russian State Medical University. 2020(5):37-43. doi: 10.24075/brsmu.2020.056.
816. Iwanski S, Lesniak M, Polanowska K, Bembenek J, Czepiel W, Seniów J. Neuronavigated 1 Hz rTMS of the left angular gyrus combined with visuospatial therapy in post-stroke neglect.

NeuroRehabilitation. 2020;46(1):83-93. doi: 10.3233/NRE-192951.

817. Khan A, Peechatka A, Dias NR, Lima V, Seddo M, Inja A, et al. Patient perspective in the development of electronic patient-reported outcomes (ePROs) in seizure disorders: A patient-centric approach. Patient Preference and Adherence. 2020;14:13-21. doi: 10.2147/PPA.S222642.

818. King ML, Manzel K, Bruss J, Tranel D. Neural correlates of improvements in personality and behavior following a neurological event. Neuropsychologia. 2020;145. doi: 10.1016/j.neuropsychologia.2017.11.023.

819. Kojima Y, Sendo R, Ohno S, Sugimura M. Ultrasound-guided inferior alveolar nerve block for trismus during dental treatment: a case report. JA Clinical Reports. 2020;6(1). doi: 10.1186/s40981-020-00400-0.

820. Krewer C, Thibaut A. Non-invasive brain stimulation for treatment of severe disorders of consciousness in people with acquired brain injury. Cochrane Database of Systematic Reviews. 2020;2020(2). doi: 10.1002/14651858.CD013533.

821. León-Lozano MZ, Arnaez J, Valls A, Arca G, Agut T, Alarcón A, et al. Cerebrospinal fluid levels of neuron-specific enolase predict the severity of brain damage in newborns with neonatal

hypoxic-ischemic encephalopathy treated with hypothermia. PLoS ONE. 2020;15(6). doi: 10.1371/journal.pone.0234082.
822. Liepert J, Stürner J, Büsching I, Sehle A, Schoenfeld MA. Effects of a single mental chronometry training session in subacute stroke patients – a randomized controlled trial. BMC Sports Science, Medicine and Rehabilitation. 2020;12(1). doi: 10.1186/s13102-020-00212-w.
823. Litvinenko IV, Zhivolupov SA, Samartsev IN, Kravchuk AY, Vorobyova MN, Yakovlev EV, et al. The Cholinergic Profile as a Target for Rational Therapy of Central Nervous System Diseases and Injuries. Neuroscience and Behavioral Physiology. 2020;50(9):1112-8. doi: 10.1007/s11055-020-01012-y.
824. Liu L, Feigin V, Sacco RL, Koroshetz WJ. Promoting global collaboration for brain health research. The BMJ. 2020;371. doi: 10.1136/bmj.m3753.
825. Lorente L, Martín MM, Pérez-Cejas A, González-Rivero AF, Abreu-González P, Ramos L, et al. Traumatic brain injury patients mortality and serum total antioxidant capacity. Brain Sciences. 2020;10(2). doi: 10.3390/brainsci10020110.
826. McMillan A, Kocharyan A, Dekker SE, Kikano EG, Garg A, Huang VW, et al. Comparison of Materials Used for 3D-Printing Temporal Bone Models to Simulate Surgical Dissection. Annals of

Otology, Rhinology and Laryngology. 2020. doi: 10.1177/0003489420918273.

827. Meng HJ, Zhang LL, Luo SS, Cao N, Zhang J, Pi YL. Modulation of hand motor skill performance induced by motor practice combined with matched or mismatched hand posture motor imagery. Physiology and Behavior. 2020;225. doi: 10.1016/j.physbeh.2020.113084.

828. Mukherjee B, Nayak S. Nonorganic visual loss in a child. Saudi Journal of Ophthalmology. 2020;34(3):220-2. doi: 10.4103/1319-4534.310419.

829. Naros G, Lehnertz T, Leão MT, Ziemann U, Gharabaghi A. Brain State-dependent Gain Modulation of Corticospinal Output in the Active Motor System. Cerebral Cortex. 2020;30(1):371-81. doi: 10.1093/cercor/bhz093.

830. Nguyen S, Santhakumar V. From Plugging the Dam to Fueling the Firing: Platelets Breach the Barrier to Seize the Brain. Epilepsy Currents. 2020;20(5):300-2. doi: 10.1177/1535759720948117.

831. Nieves MD, Furmanski O, Doughty ML. Host sex and transplanted human induced pluripotent stem cell phenotype interact to influence sensorimotor recovery in a mouse model of cortical contusion injury. Brain Research. 2020;1748. doi: 10.1016/j.brainres.2020.147120.

832. Okidi R, Ogwang DM, Okello TR, Ezati D, Kyegombe W, Nyeko D, et al. Factors affecting mortality after traumatic brain injury in a resource-poor setting. BJS open. 2020;4(2):320-5. doi: 10.1002/bjs5.50243.
833. Olaya J, Noé E, Navarro MD, O'valle M, Colomer C, Moliner B, et al. When, how, and to what extent are individuals with unresponsive wakefulness syndrome able to progress? Functional independence. Brain Sciences. 2020;10(12):1-14. doi: 10.3390/brainsci10120990.
834. Osali A. Aerobic exercise and nano-curcumin supplementation improve inflammation in elderly females with metabolic syndrome. Diabetology and Metabolic Syndrome. 2020;12(1). doi: 10.1186/s13098-020-00532-4.
835. Pender SC, Smith AM, Finnoff JT, Huston J, Stuart MJ. Concussions in Ice Hockey - Moving Toward Objective Diagnoses and Point-of-care Treatment: A Review. Current Sports Medicine Reports. 2020;19(9):380-6. doi: 10.1249/JSR.0000000000000752.
836. Perin C, Vigano B, Piscitelli D, Matteo BM, Meroni R, Cerri CG. Non-invasive current stimulation in vision recovery: A review of the literature. Restorative Neurology and Neuroscience. 2020;38(3):239-50. doi: 10.3233/RNN-190948.
837. Pushchina EV, Marinina KS, Myasoyedov SD. Hydrogen

Sulfide and Pathophysiology of the CNS. Neurophysiology. 2020;52(4):308-21. doi: 10.1007/s11062-021-09887-4.
838. Ronconi L, Melcher D, Junghöfer M, Wolters CH, Busch NA. Testing the effect of tACS over parietal cortex in modulating endogenous alpha rhythm and temporal integration windows in visual perception. European Journal of Neuroscience. 2020. doi: 10.1111/ejn.15017.
839. Roy R, Pattnaik S, Sivagurunathan S, Chidambaram S. Small ncRNA binding protein, PIWI: A potential molecular bridge between blood brain barrier and neuropathological conditions. Medical Hypotheses. 2020;138. doi: 10.1016/j.mehy.2020.109609.
840. Schwedt TJ, Digre K, Tepper SJ, Spare NM, Ailani J, Birlea M, et al. The American Registry for Migraine Research: Research Methods and Baseline Data for an Initial Patient Cohort. Headache. 2020;60(2):337-47. doi: 10.1111/head.13688.
841. Sorouri Khorashad B, Khazai B, Talaei A, Acar F, Hudson AR, Borji N, et al. Neuroanatomy of transgender persons in a Non-Western population and improving reliability in clinical neuroimaging. Journal of Neuroscience Research. 2020;98(11):2166-77. doi: 10.1002/jnr.24702.
842. Sun C, Li Y, Yates EA, Fernig DG. SimpleDSFviewer: A tool to analyze and view differential scanning fluorimetry data for

characterizing protein thermal stability and interactions. Protein Science. 2020;29(1):19-27. doi: 10.1002/pro.3703.
843. Swanson BT, Creighton D. The degenerative lumbar disc: not a disease, but still an important consideration for OMPT practice: a review of the history and science of discogenic instability. Journal of Manual and Manipulative Therapy. 2020;28(4):191-200. doi: 10.1080/10669817.2020.1758520.
844. Tegeler CL, Shaltout HA, Lee SW, Simpson SL, Gerdes L, Tegeler CH. High-resolution, relational, resonance-based, electroencephalic mirroring (HIRREM) improves symptoms and autonomic function for insomnia: A randomized, placebo-controlled clinical trial. Brain and Behavior. 2020;10(11). doi: 10.1002/brb3.1826.
845. Tsilimigras DI, Mehta R, Guglielmi A, Ratti F, Marques HP, Soubrane O, et al. Recurrence beyond the Milan criteria after curative-intent resection of hepatocellular carcinoma: A novel tumor-burden based prediction model. Journal of Surgical Oncology. 2020;122(5):955-63. doi: 10.1002/jso.26091.
846. Udovin LD, Kobiec T, Herrera MI, Toro-Urrego N, Kusnier CF, Kölliker-Frers RA, et al. Partial Reversal of Striatal Damage by Palmitoylethanolamide Administration Following Perinatal Asphyxia. Frontiers in Neuroscience. 2020;13. doi:

10.3389/fnins.2019.01345.
847. Webler RD, Hamady C, Molnar C, Johnson K, Bonilha L, Anderson BS, et al. Decreased interhemispheric connectivity and increased cortical excitability in unmedicated schizophrenia: A prefrontal interleaved TMS fMRI study. Brain Stimulation. 2020;13(5):1467-75. doi: 10.1016/j.brs.2020.06.017.
848. Wei S, Vaska P. Evaluation of quantitative, efficient image reconstruction for VersaPET, a compact PET system. Medical Physics. 2020;47(7):2852-68. doi: 10.1002/mp.14158.
849. Wilson CL, Tavender EJ, Phillips NT, Hearps SJC, Foster K, O'Brien SL, et al. Variation in CT use for paediatric head injuries across different types of emergency departments in Australia and New Zealand. Emergency Medicine Journal. 2020;37(11):686-9. doi: 10.1136/emermed-2020-209719.
850. Yuan X, Yang Y, Cao N, Jiang C. Promotion of poststroke motor-function recovery with repetitive transcranial magnetic stimulation by regulating the interhemispheric imbalance. Brain Sciences. 2020;10(9):1-14. doi: 10.3390/brainsci10090648.
851. Zurita Saavedra M, Pérez Alonso A, Pérez Cabrera B, Haro García AM, Ruiz García VM, Mirón Pozo B, et al. Management of Splenic Injuries Utilizing a Multidisciplinary Protocol in 110 Consecutive Patients at a Level II Hospital. Cirugia Espanola.

2020;98(3):143-8. doi: 10.1016/j.ciresp.2019.08.007.
852. Akkoc Altinok DC, Votinov M, Henzelmann F, Jo H, Eisert A, Habel U, et al. A combined administration of testosterone and arginine vasopressin affects aggressive behavior in males. Brain Sciences. 2021;11(12). doi: 10.3390/brainsci11121623.
853. Alpsoy MY, Sönmez S, Orhan Z, Orhan EK, Aslıyüksek H, Orhan KS. Evaluation of patients with post-traumatic hearing loss: A retrospective review of 506 cases. Journal of International Advanced Otology. 2021;17(3):239-44. doi: 10.5152/iao.2021.9089.
854. Auxéméry Y, Gayraud F. Post-concussion syndrome: Still forgotten? From the disappearance of the DSM-5 to a multidisciplinary consultation. Encephale. 2021;47(5):491-4. doi: 10.1016/j.encep.2020.06.017.
855. Boltzmann M, Schmidt SB, Gutenbrunner C, Krauss JK, Stangel M, Höglinger GU, et al. Auditory Stimulation Modulates Resting-State Functional Connectivity in Unresponsive Wakefulness Syndrome Patients. Frontiers in Neuroscience. 2021;15. doi: 10.3389/fnins.2021.554194.
856. Bouton C, Bhagat N, Chandrasekaran S, Herrero J, Markowitz N, Espinal E, et al. Decoding Neural Activity in Sulcal and White Matter Areas of the Brain to Accurately Predict Individual Finger

Movement and Tactile Stimuli of the Human Hand. Frontiers in Neuroscience. 2021;15. doi: 10.3389/fnins.2021.699631.
857. Campbell AM, Elbogen EB, Johnson JL, Hamer RM, Belger A. Event related potentials indexing the influence of emotion on cognitive processing in veterans with comorbid post-traumatic stress disorder and traumatic brain injury. Clinical Neurophysiology. 2021;132(7):1389-97. doi: 10.1016/j.clinph.2021.03.017.
858. Chavarría AP, Vázquez RRV, Cherit JGD, Bello HH, Suastegui HC, Moreno-Castañeda L, et al. Antioxidants and pentoxifylline as coadjuvant measures to standard therapy to improve prognosis of patients with pneumonia by COVID-19. Computational and Structural Biotechnology Journal. 2021;19:1379-90. doi: 10.1016/j.csbj.2021.02.009.
859. Choi Y, Jung IC, Kim AR, Park HJ, Kwon O, Lee JH, et al. Feasibility and effect of electroacupuncture on cognitive function domains in patients with mild cognitive impairment: A pilot exploratory randomized controlled trial. Brain Sciences. 2021;11(6). doi: 10.3390/brainsci11060756.
860. Conte G, Sbaraini S, Morelli C, Casale S, Caschera L, Contarino VE, et al. A susceptibility-weighted imaging qualitative score of the motor cortex may be a useful tool for distinguishing

clinical phenotypes in amyotrophic lateral sclerosis. European Radiology. 2021;31(3):1281-9. doi: 10.1007/s00330-020-07239-0.

861. Contreras Lopez WO, Navarro PA, Gouveia FV, Fonoff ET, Lebrun I, Auada AVV, et al. Directional Deep Brain Stimulation of the Posteromedial Hypothalamus for Refractory Intermittent Explosive Disorder: A Case Series Using a Novel Neurostimulation Device and Intraoperative Microdialysis. World Neurosurgery. 2021;155:e19-e33. doi: 10.1016/j.wneu.2021.07.086.

862. Coulborn S, Taylor C, Naci L, Owen AM, Fernández-Espejo D. Disruptions in effective connectivity within and between default mode network and anterior forebrain mesocircuit in prolonged disorders of consciousness. Brain Sciences. 2021;11(6). doi: 10.3390/brainsci11060749.

863. D'Arcy RCN, Sandhu JK, Marshall S, Besemann M. Mitigating Long-Term COVID-19 Consequences on Brain Health. Frontiers in Neurology. 2021;12. doi: 10.3389/fneur.2021.630986.

864. Daia C, Scheau C, Spinu A, Andone I, Popescu C, Toader C, et al. Modulated neuroprotection in unresponsive wakefulness syndrome after severe traumatic brain injury. Brain Sciences. 2021;11(8). doi: 10.3390/brainsci11081044.

865. Das S, Nalini A, Laxmi TR, Raju TR. ALS-CSF-induced structural changes in spinal motor neurons of rat pups cause

deficits in motor behaviour. Experimental Brain Research. 2021;239(1):315-27. doi: 10.1007/s00221-020-05969-7.

866. De Martino ML, De Bartolo M, Leemhuis E, Pazzaglia M. Rebuilding body–brain interaction from the vagal network in spinal cord injuries. Brain Sciences. 2021;11(8). doi: 10.3390/brainsci11081084.

867. Evans TC, DeGutis J, Rothlein D, Jagger-Rickels A, Yamashita A, Fortier CB, et al. Punishment and reward normalize error-related cognitive control in PTSD by modulating salience network activation and connectivity. Cortex. 2021;145:295-314. doi: 10.1016/j.cortex.2021.09.004.

868. Fan K, Zeng L, Guo J, Xie S, Yu Y, Chen J, et al. Visualized podocyte-targeting and focused ultrasound responsive glucocorticoid nano-delivery system against immune-associated nephropathy without glucocorticoid side effect. Theranostics. 2021;11(6):2670-90. doi: 10.7150/THNO.53083.

869. Fragío Gil JJ, Grau García E, Fernández Matilla M, Ortiz Sanjuan FM, Rodrigo Nicolás R, Román Ivorra JA. Decreased DNA hydroxymethylation and increased DNA demethylation are associated with high antioxidant response in systemic lupus erythematosus patients. Medicina Clinica. 2021;157(12):575-9. doi: 10.1016/j.medcli.2020.10.023.

870. Herrity AN, Aslan SC, Ugiliweneza B, Mohamed AZ, Hubscher CH, Harkema SJ. Improvements in Bladder Function Following Activity-Based Recovery Training With Epidural Stimulation After Chronic Spinal Cord Injury. Frontiers in Systems Neuroscience. 2021;14. doi: 10.3389/fnsys.2020.614691.
871. Hümmer K, Coenen J, Konstantinidis I, Lausberg H, Helmich I. Impaired recognition of nonverbal expressions of emotions in depressed individuals. Psychiatry Research. 2021;302. doi: 10.1016/j.psychres.2021.114031.
872. Huynh-Le MP, Tibbs MD, Karunamuni R, Salans M, Tringale KR, Yip A, et al. Microstructural Injury to Corpus Callosum and Intrahemispheric White Matter Tracts Correlate With Attention and Processing Speed Decline After Brain Radiation. International Journal of Radiation Oncology Biology Physics. 2021;110(2):337-47. doi: 10.1016/j.ijrobp.2020.12.046.
873. Imperatore JP, McCalley DM, Borckardt JJ, Brady KT, Hanlon CA. Non-invasive brain stimulation as a tool to decrease chronic pain in current opiate users: A parametric evaluation of two promising cortical targets. Drug and Alcohol Dependence. 2021;218. doi: 10.1016/j.drugalcdep.2020.108409.
874. Inanici F, Brighton LN, Samejima S, Hofstetter CP, Moritz CT. Transcutaneous Spinal Cord Stimulation Restores Hand and Arm

Function after Spinal Cord Injury. IEEE Transactions on Neural Systems and Rehabilitation Engineering. 2021;29:310-9. doi: 10.1109/TNSRE.2021.3049133.

875. Ishii D, Ishibashi K, Takeda K, Yuine H, Yamamoto S, Kaku Y, et al. Interaction of the Left–Right Somatosensory Pathways in Patients With Thalamic Hemorrhage: A Case Report. Frontiers in Human Neuroscience. 2021;15. doi: 10.3389/fnhum.2021.761186.

876. Ito S, Itoh H, Rakugi H, Okuda Y, Iijima S. Antihypertensive effects and safety of esaxerenone in patients with moderate kidney dysfunction. Hypertension Research. 2021;44(5):489-97. doi: 10.1038/s41440-020-00585-y.

877. Jerban S, Kasibhatla A, Ma Y, Wu M, Chen Y, Guo T, et al. Detecting Articular Cartilage and Meniscus Deformation Effects Using Magnetization Transfer Ultrashort Echo Time (MT-UTE) Modeling during Mechanical Load Application: Ex Vivo Feasibility Study. Cartilage. 2021;13(1_suppl):665S-73S. doi: 10.1177/1947603520976771.

878. Jerban S, Ma Y, Kasibhatla A, Wu M, Szeverenyi N, Guma M, et al. Ultrashort echo time adiabatic T1ρ (UTE-Adiab-T1ρ) is sensitive to human cadaveric knee joint deformation induced by mechanical loading and unloading. Magnetic Resonance Imaging. 2021;80:98-105. doi: 10.1016/j.mri.2021.04.014.

879. Kalita J, Kumar S, Singh VK, Misra UK. A randomized controlled trial of high rate rtms versus rtms and amitriptyline in chronic migraine. Pain Physician. 2021;24(6):E733-E41. doi: 10.36076/ppj.2021.24.e733.
880. Kamali AM, Gholamzadeh MJ, Mousavi SZ, Gharamaleki MV, Nami M. Improved Visual Function in a Case of Ultra-low Vision following Ischemic Encephalopathy Using Transcranial Electrical Stimulation; A Brief Report. Developmental Neurorehabilitation. 2021;24(1):68-72. doi: 10.1080/17518423.2020.1839978.
881. Krenzlin H, Wesp D, Schmitt J, Frenz C, Kurz E, Masomi-Bornwasser J, et al. Decreased superoxide dismutase concentrations (Sod) in plasma and csf and increased circulating total antioxidant capacity (tac) are associated with unfavorable neurological outcome after aneurysmal subarachnoid hemorrhage. Journal of Clinical Medicine. 2021;10(6):1-10. doi: 10.3390/jcm10061188.
882. Kullakçi H, Sonkaya AR. The investigation of the effects of repetitive transcranialmagnetic stimulation treatment on taste and smell sensations in depressed patients. Noropsikiyatri Arsivi. 2021;58(1):26-33. doi: 10.29399/npa.25087.
883. Lan Q, Guo R, Chang J, Zheng J, Yu K, Chen J, editors. Fast Prediction of RF-induced Heating for Sacral Neuromodulation

System Exposed to Multi-Channel 2 RF Field at 3T MRI. Proceedings of the Annual International Conference of the IEEE Engineering in Medicine and Biology Society, EMBS; 2021.

884. Longley V, Hazelton C, Heal C, Pollock A, Woodward-Nutt K, Mitchell C, et al. Non-pharmacological interventions for spatial neglect or inattention following stroke and other non-progressive brain injury. Cochrane Database of Systematic Reviews. 2021;2021(7). doi: 10.1002/14651858.CD003586.pub4.

885. Lorente L, Martín MM, Pérez-Cejas A, González-Rivero AF, Sabatel R, Ramos-Gómez L, et al. High serum levels of TAC and early mortality in patients with spontaneous intracerebral haemorrhage. Neurological Sciences. 2021;42(4):1491-7. doi: 10.1007/s10072-020-04692-z.

886. Loveridge A, Smith DS, McGeer JC. Dissolved Organic Matter Mitigates the Acute Toxicity of Thulium to Hyalella azteca but Ca, Mg and Na Do Not. Archives of Environmental Contamination and Toxicology. 2021;81(4):637-47. doi: 10.1007/s00244-021-00898-0.

887. Luo Y, Granzhan A, Verga D, Mergny JL. FRET-MC: A fluorescence melting competition assay for studying G4 structures in vitro. Biopolymers. 2021;112(4). doi: 10.1002/bip.23415.

888. Mansur A, Rabiner EA, Tsukada H, Comley RA, Lewis Y,

Huiban M, et al. Test–retest variability and reference region-based quantification of 18F-BCPP-EF for imaging mitochondrial complex I in the human brain. Journal of Cerebral Blood Flow and Metabolism. 2021;41(4):771-9. doi: 10.1177/0271678X20928149.

889. Martel MM, Eng AG, Bansal PS, Smith TE, Elkins AR, Goh PK. Multiple informant average integration of ADHD symptom ratings predictive of concurrent and longitudinal impairment. Psychological Assessment. 2021;33(5):443-51. doi: 10.1037/pas0000994.

890. Max SM, Plewnia C, Zipfel S, Giel KE, Schag K. Combined antisaccade task and transcranial direct current stimulation to increase response inhibition in binge eating disorder. European Archives of Psychiatry and Clinical Neuroscience. 2021;271(1):17-28. doi: 10.1007/s00406-020-01164-5.

891. Mehta UM, Ektare A, Jakhar J, Basavaraju R, Sanjay TN, Naik SS, et al. A transdiagnostic evaluation of cortical inhibition in severe mental disorders using Transcranial Magnetic Stimulation. Journal of Psychiatric Research. 2021;143:364-9. doi: 10.1016/j.jpsychires.2021.09.049.

892. Mitra B, Ruggles T, Seah J, Miller C, Fitzgerald MC. Delayed intracranial hemorrhage after trauma. Brain Injury. 2021;35(4):484-9. doi: 10.1080/02699052.2021.1887520.

893. Mülling K, Fischer AJ, Siakaeva E, Richter M, Bordbari S, Spyra I, et al. Neutrophil dynamics, plasticity and function in acute neurodegeneration following neonatal hypoxia–ischemia. Brain, Behavior, and Immunity. 2021;92:234-44. doi: 10.1016/j.bbi.2020.12.012.

894. Nagy SA, Kürtös Z, Németh N, Perlaki G, Csernela E, Lakner FE, et al. Childhood maltreatment results in altered deactivation of reward processing circuits in depressed patients: A functional magnetic resonance imaging study of a facial emotion recognition task. Neurobiology of Stress. 2021;15. doi: 10.1016/j.ynstr.2021.100399.

895. Nejati V, Movahed Alavi M, Nitsche MA. The Impact of Attention Deficit-hyperactivity Disorder Symptom Severity on the Effectiveness of Transcranial Direct Current Stimulation (tDCS) on Inhibitory Control. Neuroscience. 2021;466:248-57. doi: 10.1016/j.neuroscience.2021.05.008.

896. Noé E, Ferri J, Olaya J, Navarro MD, O'valle M, Colomer C, et al. When, how, and to what extent are individuals with unresponsive wakefulness syndrome able to progress? Neurobehavioral progress. Brain Sciences. 2021;11(1):1-16. doi: 10.3390/brainsci11010126.

897. O'Donnell DM, Agin A. Management of headaches in children

and adolescents. Current Problems in Pediatric and Adolescent Health Care. 2021;51(7). doi: 10.1016/j.cppeds.2021.101034.

898. Orlando B, Morano A, Irelli EC, Fanella M, Giallonardo AT, Di Bonaventura C. Unilateral catalepsy in non-convulsive status epilepticus. Epileptic Disorders. 2021;23(5):760-4. doi: 10.1684/epd.2021.1323.

899. Qian F, Han Y, Han Z, Zhang D, Zhang L, Zhao G, et al. In Situ implantable, post-trauma microenvironment-responsive, ROS Depletion Hydrogels for the treatment of Traumatic brain injury. Biomaterials. 2021;270. doi: 10.1016/j.biomaterials.2021.120675.

900. Rr P, Douch C, Aan Koh MJ, Lai AHM, Lim CT, Hartley L, et al. Speckled brain lesions in Incontinentia Pigmenti patients with acquired brain syndromes. European Journal of Paediatric Neurology. 2021;33:106-11. doi: 10.1016/j.ejpn.2021.05.012.

901. Saetia S, Yoshimura N, Koike Y. Constructing Brain Connectivity Model Using Causal Network Reconstruction Approach. Frontiers in Neuroinformatics. 2021;15. doi: 10.3389/fninf.2021.619557.

902. Schwab PJ, Miller A, Raphail AM, Levine A, Haslam C, Coslett HB, et al. Virtual reality tools for assessing unilateral spatial neglect: A novel opportunity for data collection. Journal of Visualized Experiments. 2021;2021(169). doi: 10.3791/61951.

903. Shen Y, Wang J, Yuan J, Yang L, Yu F, Wang X, et al. Anemia among Chinese patients with chronic kidney disease and its association with quality of life - results from the Chinese cohort study of chronic kidney disease (C-STRIDE). BMC nephrology. 2021;22(1):64. doi: 10.1186/s12882-021-02247-8.
904. Shi Y, Song R, Wang Z, Zhang H, Zhu J, Yue Y, et al. Potential clinical value of circular RNAs as peripheral biomarkers for the diagnosis and treatment of major depressive disorder. EBioMedicine. 2021;66. doi: 10.1016/j.ebiom.2021.103337.
905. Singhal D, Hahn CN, Feurstein S, Wee LYA, Moma L, Kutyna MM, et al. Targeted gene panels identify a high frequency of pathogenic germline variants in patients diagnosed with a hematological malignancy and at least one other independent cancer. Leukemia. 2021;35(11):3245-56. doi: 10.1038/s41375-021-01246-w.
906. Spindler LRB, Luppi AI, Adapa RM, Craig MM, Coppola P, Peattie ARD, et al. Dopaminergic brainstem disconnection is common to pharmacological and pathological consciousness perturbation. Proceedings of the National Academy of Sciences of the United States of America. 2021;118(30). doi: 10.1073/pnas.2026289118.
907. Sun Q, Fang Y, Shi Y, Wang L, Peng X, Tan L. Inhibitory

Top-Down Control Deficits in Schizophrenia With Auditory Verbal Hallucinations: A Go/NoGo Task. Frontiers in Psychiatry. 2021;12. doi: 10.3389/fpsyt.2021.544746.

908. Taing AS, Mundy ME, Ponsford JL, Spitz G. Aberrant modulation of brain activity underlies impaired working memory following traumatic brain injury. NeuroImage: Clinical. 2021;31. doi: 10.1016/j.nicl.2021.102777.

909. Talas DÜ, Beger O, Çömelekoğlu Ü, Çakır S, Taghipour P, Vayisoğlu Y. An insight to tympanic membrane perforation pressure through morphometry: A cadaver study. Diving and Hyperbaric Medicine. 2021;51(1):10-7. doi: 10.28920/dhm51.1.10-17.

910. Toyohara J, Sakata M, Ishibashi K, Mossel P, Imai M, Wagatsuma K, et al. First clinical assessment of [18F]MC225, a novel fluorine-18 labelled PET tracer for measuring functional P-glycoprotein at the blood–brain barrier. Annals of Nuclear Medicine. 2021;35(11):1240-52. doi: 10.1007/s12149-021-01666-9.

911. van den Bos MAJ, Higashihara M, Geevasinga N, Menon P, Kiernan MC, Vucic S. Pathophysiological associations of transcallosal dysfunction in ALS. European Journal of Neurology. 2021;28(4):1172-80. doi: 10.1111/ene.14653.

912. Vuralli D, Karatas H, Yemisci M, Bolay H. Updated review on the link between cortical spreading depression and headache disorders. Expert Review of Neurotherapeutics. 2021;21(10):1069-84. doi: 10.1080/14737175.2021.1947797.
913. Wang P, Peng ZL, Liu L, An L, Liu YX, Cao QJ, et al. Neural response to trauma-related and trauma-unrelated negative stimuli in remitted and persistent pediatric post-traumatic stress disorder. Brain and Behavior. 2021;11(7). doi: 10.1002/brb3.2173.
914. Wang Y, Zhang W, Wu X, Luo X, Li S, Zhu G, et al. Cerebral Structural Abnormalities and Their Associations With Peripheral Cytokine Levels in a Group of Untreated Patients With Nasopharyngeal Carcinoma. Frontiers in Oncology. 2021;11. doi: 10.3389/fonc.2021.740033.
915. Wilson CL, Hearps SJC, Tavender EJ, Phillips NT, Lawton B, Kinnear F, et al. Factors predictive for computed tomography use and abnormality in paediatric head injuries in Australia and New Zealand. EMA - Emergency Medicine Australasia. 2021;33(1):157-60. doi: 10.1111/1742-6723.13694.
916. Yanming Q, Peng W, Xuanxuan X, Zheng L. Analysis of risk factors of acute respiratory distress syndrome secondary to severe multiple trauma. Zhonghua Wei Zhong Bing Ji Jiu Yi Xue. 2021;33(3):299-304. doi:

10.3760/cma.j.cn121430-20201023-00685.
917. Yu J, Meng F, He F, Chen F, Bao W, Yu Y, et al. Metabolic abnormalities in patients with chronic disorders of consciousness. Aging and Disease. 2021;12(2):386-403. doi: 10.14336/AD.2020.0812.
918. Zamunér AR, Minonzio M, Shiffer D, Fornerone R, Cairo B, Porta A, et al. Relationships Between Cardiovascular Autonomic Profile and Work Ability in Patients With Pure Autonomic Failure. Frontiers in Human Neuroscience. 2021;15. doi: 10.3389/fnhum.2021.761501.
919. Zhang D, Wang JLJ, Wang P, Cheng Y, Yuan Y, Song W. Efficacy comparison of noninvasive brain stimulation techniques on picture naming ability of non-fluent aphasia patients after stroke with normal expression capacity. Chinese Journal of Cerebrovascular Diseases. 2021;18(2):84-90. doi: 10.3969/j.issn.1672-5921.2021.02.002.
920. Zhang Y, Du W, Yin Y, Li H, Liu Z, Yang Y, et al. Impaired cerebral vascular and metabolic responses to parametric N-back tasks in subjective cognitive decline. Journal of Cerebral Blood Flow and Metabolism. 2021;41(10):2743-55. doi: 10.1177/0271678X211012153.
921. Zhong M, Cywiak C, Metto AC, Liu X, Qian C, Pelled G.

Multi-session delivery of synchronous rTMS and sensory stimulation induces long-term plasticity. Brain Stimulation. 2021;14(4):884-94. doi: 10.1016/j.brs.2021.05.005.
922. Bhome R, Zarkali A, Thomas GEC, Iglesias JE, Cole JH, Weil RS. Thalamic white matter macrostructure and subnuclei volumes in Parkinson’s disease depression. npj Parkinson's Disease. 2022;8(1). doi: 10.1038/s41531-021-00270-y.
923. Duan YJ, Hua XY, Zheng MX, Wu JJ, Xing XX, Li YL, et al. Corticocortical paired associative stimulation for treating motor dysfunction after stroke: Study protocol for a randomised sham-controlled double-blind clinical trial. BMJ Open. 2022;12(1). doi: 10.1136/bmjopen-2021-053991.
924. Khan A, Antonakakis M, Vogenauer N, Haueisen J, Wolters CH. Individually optimized multi-channel tDCS for targeting somatosensory cortex. Clinical Neurophysiology. 2022;134:9-26. doi: 10.1016/j.clinph.2021.10.016.
925. Koh DTS, Chen JY, Tan SM, Tay KS, Singh IR, Yeo NEM. Mid-Term Functional and Radiological Outcomes of Total Ankle Replacement in an Asian Cohort. Journal of Foot and Ankle Surgery. 2022;61(2):363-8. doi: 10.1053/j.jfas.2021.09.014.
926. Nguyen AL, Kirkwood B, Hackett L, Buntine P. Validation of the ECHS non trauma cranial CT rule in Australia: A prospective

cohort study. American Journal of Emergency Medicine. 2022;52:225-31. doi: 10.1016/j.ajem.2021.12.004.

927. Papa A, Pasquini S, Contri C, Gemma S, Campiani G, Butini S, et al. Polypharmacological Approaches for CNS Diseases: Focus on Endocannabinoid Degradation Inhibition. Cells. 2022;11(3). doi: 10.3390/cells11030471.

928. Patel H, Naish K, Soreni N, Amlung M. The Effects of a Single Transcranial Direct Current Stimulation Session on Impulsivity and Risk Among a Sample of Adult Recreational Cannabis Users. Frontiers in Human Neuroscience. 2022;16. doi: 10.3389/fnhum.2022.758285.

929. Pavitt S, Gelfand AA, Zorrilla N, Allen I, Riggins N. Efficacy and Safety of Repetitive Intravenous Sodium Valproate in Pediatric Patients With Refractory Chronic Headache Disorders: A Retrospective Review. Pediatric Neurology. 2022;128:52-7. doi: 10.1016/j.pediatrneurol.2021.12.008.

930. Ramage AE, Ray KL, Franz HM, Tate DF, Lewis JD, Robin DA. Cingulo-Opercular and Frontoparietal Network Control of Effort and Fatigue in Mild Traumatic Brain Injury. Frontiers in Human Neuroscience. 2022;15. doi: 10.3389/fnhum.2021.788091.

931. Stunnenberg BC, Berends J, Griggs RC, Statland J, Drost G, Nikles J, et al. N-of-1 Trials in Neurology A Systematic Review.

Neurology. 2022;98(2):E174-E85. doi: 10.1212/WNL.0000000000012998.

932. Winters B, Kuluris B, Pathmanaban R, Vanderwalt H, Thibaut A, Schnakers C. Changes of Spasticity across Time in Prolonged Disorders of Consciousness: A Retrospective Study. Brain Sciences. 2022;12(2). doi: 10.3390/brainsci12020295.

933. Xu R, Zhu GY, Zhu J, Wang Y, Xing XX, Chen LY, et al. Using Hebbian-Type Stimulation to Rescue Arm Function After Stroke: Study Protocol for a Randomized Clinical Trial. Frontiers in Neural Circuits. 2022;15. doi: 10.3389/fncir.2021.789095.

934. Zhang T, Song B, Li Y, Duan R, Gong Z, Jing L, et al. Neurofilament Light Chain as a Biomarker for Monitoring the Efficacy of Transcranial Magnetic Stimulation on Alcohol Use Disorder. Frontiers in Behavioral Neuroscience. 2022;16. doi: 10.3389/fnbeh.2022.831901.

(5) Conchrane central register of controlled trials (310 studies)

1. Oliver KM, Hemenger RP, Corbett MC, O'Brart DP, Verma S, Tomlinson A, et al. Corneal aberrations one year after photo refractive keratectomy for three types of ablation zone. American academy of optometry. 1995:183. PubMed PMID: CN-00518398.

2. Brouwer B, Hopkins-Rosseel DH. Motor cortical mapping of proximal upper extremity muscles following spinal cord injury. Spinal cord. 1997;35(4):205-12. doi: 10.1038/sj.sc.3100384. PubMed PMID: CN-00139385.

3. Hjortdal JO, Olsen H, Ehlers N. A prospective randomized comparative study of the optical performance of the cornea one year after radial keratotomy or photorefractive keratectomy. IOVS. 2000;41:ARVO Abstract 3612. PubMed PMID: CN-00679029.

4. Hjortdal JO, MÃ¸ller P, Iversen A. Corneal Aberrations after PRK and LASIK for Myopia. Results from a Prospective Randomised Trial. IOVS. 2003:ARVO E-abstract 2637. PubMed PMID: CN-00718134.

5. He CS, Yu Q, Yang DJ, Yang M. Interventional effects of low-frequency repetitive transcranial magnetic stimulation on patients with depression after traumatic brain injury. Chinese journal of clinical rehabilitation. 2004;8(28):6044-5. PubMed PMID: CN-01708746.

6. Xu JJ, Wei ZH, Yang LM, Zhang XJ, Diao HM, Li YT, et al. Effect of median nerve electrical stimulation against vegetative state of patients after craniocerebral injury. Chinese journal of clinical rehabilitation. 2004;8(28):6040-3. PubMed PMID: CN-01708745.

7. Hjortdal O, MÃ¸ller-Pedersen T, Ivarsen A. Biomechanical Changes in the Human Cornea After PRK and LASIK. IOVS. 2005;46:ARVO E-abstract 2735. PubMed PMID: CN-00599072.

8. Nct. Moving a Paralyzed Hand Through Use of a Brain-Computer Interface. https://clinicaltrialsgov/show/NCT00242242. 2005. PubMed PMID: CN-01582520.

9. Nct. A Double-Blind Placebo Controlled Trial of Repetitive Transcranial Magnetic Stimulation (rTMS) in the Treatment of Negative Symptoms in Schizophrenia. https://clinicaltrialsgov/show/NCT00168311. 2005. PubMed PMID: CN-01478111.

10. Nct. A Randomised Double-Blind Trial of Targeted Repetitive Transcranial Magnetic Stimulation in Major Depressive Disorder. https://clinicaltrialsgov/show/NCT00168376. 2005. PubMed PMID: CN-02013652.

11. Nct. Priming rTMS In Major Depression.

https://clinicaltrialsgov/show/NCT00168272. 2005. PubMed PMID: CN-01478109.

12. Fregni F, Boggio PS, Lima MC, Ferreira MJ, Wagner T, Rigonatti SP, et al. A sham-controlled, phase II trial of transcranial direct current stimulation for the treatment of central pain in traumatic spinal cord injury. Pain. 2006;122(1-2):197-209. doi: 10.1016/j.pain.2006.02.023. PubMed PMID: CN-00563646.

13. Nct. Long-Term Improvement in Motor Learning by Transcranial Direct Current Stimulation. https://clinicaltrialsgov/show/NCT00314769. 2006. PubMed PMID: CN-01481637.

14. Wallace MS, Charapata SG, Fisher R, Byas-Smith M, Staats PS, Mayo M, et al. Intrathecal ziconotide in the treatment of chronic nonmalignant pain: a randomized, double-blind, placebo-controlled clinical trial. Neuromodulation. 2006;9(2):75-86. doi: 10.1111/j.1525-1403.2006.00055.x. PubMed PMID: CN-00622675.

15. Nct. TMS in the Treatment of the Sequelae of Closed Brain Injury. https://clinicaltrialsgov/show/NCT00531258. 2007. PubMed PMID: CN-02014393.

16. Nct. Migraine--Investigational Treatment of Migraine With Noninvasive Brain Stimulation. (tDCS- Migraine).

https://clinicaltrialsgov/show/NCT00521196. 2007. PubMed PMID: CN-01590594.

17. Candidi M, Urgesi C, Ionta S, Aglioti SM. Virtual lesion of ventral premotor cortex impairs visual perception of biomechanically possible but not impossible actions. Social neuroscience. 2008;3(3-4):388-400. doi: 10.1080/17470910701676269. PubMed PMID: CN-00684893.

18. Nct. Using Repetitive Transcranial Magnetic Stimulation (rTMS) in the Rehabilitation of Chronic Spatial Neglect After Stroke. https://clinicaltrialsgov/show/NCT01000103. 2009. PubMed PMID: CN-01526424.

19. Nct. Transcranial Magnetic Stimulation (TMS) for Suicidal Ideation. https://clinicaltrialsgov/show/NCT01212848. 2010. PubMed PMID: CN-02022083.

20. Nct. Effects of rTMS on the Cognition of Elderly With Mild Memory Complaints. https://clinicaltrialsgov/show/NCT01292382. 2010. PubMed PMID: CN-01531886.

21. Cioni B, Bentivoglio A, Conforti G, Daniele A, De Simone C, Fasano A, et al. Motor cortex stimulation for Parkinson's disease. A prospective double-blind randomized study with cross-over. Preliminary results. Neuromodulation. 2011;14(4):364. doi: 10.1111/j.1525-1403.2011.00363.x. PubMed PMID:

CN-01029477.
22. Irct138903254191N. Repetitive transcranial magnetic stimulation and intermittent theta burst stimulation in schizophrenia. https://trialsearchwhoint/Trial2aspx?TrialID=IRCT138903254191N1. 2011. PubMed PMID: CN-01799008.
23. Nct. Transcranial Direct Current Stimulation (tDCS) and Motor Training for Traumatic Brain Injury (TBI) Survivors. https://clinicaltrialsgov/show/NCT01749735. 2011. PubMed PMID: CN-01539339.
24. Nct. Paraorbital-Occipital Alternating Current Stimulation Therapy of Patients With Post-Chiasmatic Lesions. https://clinicaltrialsgov/show/NCT01418820. 2011. PubMed PMID: CN-01488182.
25. Nct. A Double Blind Sham-controled Study to Evaluate the Influence of Low Frequency Repetitive Transcranial Stimulation (r-TMS) on Motor and Cognitive Measurements in Patients With Asymmetric Parkinson's Disease. https://clinicaltrialsgov/show/NCT01367782. 2011. PubMed PMID: CN-01486872.
26. Irct2012102311234N. Effects of Transcranial Direct Current Stimulation (tDCS) over Craving in abstinence methamphetamine

user.
https://trialsearchwhoint/Trial2aspx?TrialID=IRCT2012102311234N1. 2012. PubMed PMID: CN-01806758.

27. Nct. Effects of Huperzine A in Treatment of Moderate to Severe TBI. https://clinicaltrialsgov/show/NCT01676311. 2012. PubMed PMID: CN-01585265.

28. Nct. Relationship Between Attention and Emotional Regulation Post-Traumatic Brain Injury (TBI). https://clinicaltrialsgov/show/NCT01681589. 2012. PubMed PMID: CN-01537517.

29. Nct. Combined Transcranial Magnetic Stimulation and Cognitive Treatment in Blast Traumatic Brain Injury. https://clinicaltrialsgov/show/NCT01596569. 2012. PubMed PMID: CN-01596380.

30. Nct. Improving Spinal Cord Injury Rehabilitation Interventions by Retraining the Brain With Stimulation. https://clinicaltrialsgov/show/NCT01539109. 2012. PubMed PMID: CN-02044113.

31. Nct. Directing Neuroplasticity to Improve Rehabilitative Outcomes of the Upper Limb in Incomplete Quadriplegia. https://clinicaltrialsgov/show/NCT01539109. 2012. PubMed PMID: CN-01591278.

32. Nct. Precision Versus Endurance Training to Improve Walking After Chronic Incomplete SCI. https://clinicaltrialsgov/show/NCT01765153. 2012. PubMed PMID: CN-02035526.

33. Chi CT. Exploratory study of peripheral nerve rewiring surgery for rescuing the hemiplegic hand after central neurologic injuries by inducing brain reorganization. https://trialsearchwhoint/Trial2aspx?TrialID=ChiCTR-TRC-13004466. 2013. PubMed PMID: CN-01833292.

34. Chiaramonti R, Giovannelli F, Bianco G, Godone M, Battista D, Cardinali C, et al. Lack of behavioural effects of high-frequency rTMS in vegetative state: a randomised, double blind, sham-controlled, crossover study. Clinical neurophysiology. 2013;124(11):e211. doi: 10.1016/j.clinph.2013.06.126. PubMed PMID: CN-01060114.

35. Hensley-Judge H, Quigg M, Barbaro NM, Newman SA, Ward MM, Chang EF, et al. Visual field defects after radiosurgery for mesial temporal lobe epilepsy. Epilepsia. 2013;54(8):1376-80. doi: 10.1111/epi.12215. PubMed PMID: CN-00963913.

36. Irct2013042313100N. study of transcranial magnetic stimulation effect on motor imagery rehabilitation and movement improvment in multiple sclerosis patients.

https://trialsearchwhoint/Trial2aspx?TrialID=IRCT2013042313100 N1. 2013. PubMed PMID: CN-01805100.

37. Jetté F, Côté I, Meziane HB, Mercier C. Effect of single-session repetitive transcranial magnetic stimulation applied over the hand versus leg motor area on pain after spinal cord injury. Neurorehabilitation and neural repair. 2013;27(7):636-43. doi: 10.1177/1545968313484810. PubMed PMID: CN-00919324.

38. Jprn U. Before-after study of a transcranial direct current stimulation (tDCS) effects for rehabilitative treatments in subacute brain damaged patients. https://trialsearchwhoint/Trial2aspx?TrialID=JPRN-UMIN0000114 44. 2013. PubMed PMID: CN-01796444.

39. Midaglia L, Fraga A, Rodriguez Acevedo B, Lion S, Ribeiro S, Dieguez C, et al. Repetitive transcranial magnetic stimulation in refractory spasticity treatment in patients with multiple sclerosis. Multiple sclerosis (Houndmills, Basingstoke, England). 2013;19(11 SUPPL. 1):279-80. doi: 10.1177/1352458513502429. PubMed PMID: CN-01058244.

40. Nct. tDCS in MCS: Repeated Stimulations. https://clinicaltrialsgov/show/NCT02019615. 2013. PubMed PMID: CN-01479956.

41. Nct. Amantadine + rTMS as a Neurotherapeutic for

Disordered Consciousness. https://clinicaltrialsgov/show/NCT02025439. 2013. PubMed PMID: CN-01480121.

42. Nct. Improving Functions in MTBI Patients With Headache by rTMS. https://clinicaltrialsgov/show/NCT01948947. 2013. PubMed PMID: CN-01590508.

43. Nct. Transcranial Brain Stimulation in Vegetative State Patients. https://clinicaltrialsgov/show/NCT01820923. 2013. PubMed PMID: CN-01541309.

44. Nct. Effects of Repetitive Transcranial Magnetic Stimulation in the Treatment of Phantom Limb Pain in Landmine Victims: ANTARES. https://clinicaltrialsgov/show/NCT01872481. 2013. PubMed PMID: CN-01542664.

45. Nct. Improving Motor Function After Spinal Cord Injury. https://clinicaltrialsgov/show/NCT01915095. 2013. PubMed PMID: CN-01579207.

46. Nct. Skilled Motor Training and tDCS to Improve Leg Function After Spinal Cord Injury. https://clinicaltrialsgov/show/NCT01962675. 2013. PubMed PMID: CN-01536776.

47. Nct. Antispastic Effect of Transcranial Magnetic Stimulation in Patients With Cerebral and Spinal Spasticity.

https://clinicaltrialsgov/show/NCT01786005. 2013. PubMed PMID: CN-01579005.

48. Gao GY, Bao Y, Liang Y, Ding S, Jiang J. Right median nerve electrical stimulation improves the outcome of traumatic coma patients. Journal of neurotrauma. 2014;31(5):A43. doi: 10.1089/neu.2014.9937. PubMed PMID: CN-01058918.

49. Gibson N, Lay B, Blair E, Hamer P. The functional benefit and peripheral and central pathway changes derived from resistance training augmented with botulinum toxin A for the upper limb in children with spastic hemiplegia: a randomised controlled trial. Developmental medicine and child neurology. 2014;56:53-4. doi: 10.1111/dmcn.12368. PubMed PMID: CN-01023244.

50. Giovannelli F, Chiaramonti R, Bianco G, Godone M, Battista D, Cardinali C, et al. Lack of behavioural effects of high-frequency rTMS in vegetative state: a randomised, double blind, sham-controlled, cross-over study. Clinical neurophysiology. 2014;125:S243. PubMed PMID: CN-01059912.

51. Irct2014042817468N. The effect of transcranial direct current stimulation on pain and pain anxiety of burn in Patients with burns. https://trialsearchwhoint/Trial2aspx?TrialID=IRCT2014042817468N1. 2014. PubMed PMID: CN-01851089.

52. Irct2014062318192N. Effect of transcranial Direct Current

stimulation on inhibitory control. https://trialsearchwhoint/Trial2aspx?TrialID=IRCT2014062318192N1. 2014. PubMed PMID: CN-01846680.

53. Jensen MP, Sherlin LH, Fregni F, Gianas A, Howe JD, Hakimian S. Baseline brain activity predicts response to neuromodulatory pain treatment. Pain medicine (Malden, Mass). 2014;15(12):2055-63. doi: 10.1111/pme.12546. PubMed PMID: CN-01113278.

54. Nct. rTMS to Improve Cognitive Function in TBI. https://clinicaltrialsgov/show/NCT02152540. 2014. PubMed PMID: CN-01596932.

55. Nct. Brain Stimulation for Traumatic Brain Injury. https://clinicaltrialsgov/show/NCT02167971. 2014. PubMed PMID: CN-02037454.

56. Nct. Brain Stimulation for Mild Traumatic Brain Injury. https://clinicaltrialsgov/show/NCT02292589. 2014. PubMed PMID: CN-01550264.

57. Nct. Noninvasive Neuromodulation for Treatment of Symptoms Due to Mild or Moderate Traumatic Brain Injury. https://clinicaltrialsgov/show/NCT02158494. 2014. PubMed PMID: CN-01546282.

58. Nct. Cranial-nerve Non-invasive Neuromodulation

(CN-NINM) for Balance Deficits After Mild Traumatic Brain Injury (Full Trial). https://clinicaltrialsgov/show/NCT02125591. 2014. PubMed PMID: CN-01545356.

59. Nct. Cranial-nerve Non-invasive Neuromodulation (CN-NINM) for Balance Deficits After Mild Traumatic Brain Injury (Pilot Trial). https://clinicaltrialsgov/show/NCT02109198. 2014. PubMed PMID: CN-01544918.

60. Nct. Brain Mechanisms Underlying Reading Improvement in Central Alexia. https://clinicaltrialsgov/show/NCT02062619. 2014. PubMed PMID: CN-01586876.

61. Nct. Action Observation Treatment (AOT) as a Rehabilitation Tool. https://clinicaltrialsgov/show/NCT02235350. 2014. PubMed PMID: CN-01548556.

62. Nct. Prolonged-release Fampridine as Adjunct Therapy to Active Motor Training in MS Patients. https://clinicaltrialsgov/show/NCT02146534. 2014. PubMed PMID: CN-01545966.

63. Nct. Treating Refractory Schizophrenia With rTMS. https://clinicaltrialsgov/show/NCT02242578. 2014. PubMed PMID: CN-01548763.

64. Nct. The Effects of Exercise Training on Shoulder Neuromuscular Control.

https://clinicaltrialsgov/show/NCT02164305. 2014. PubMed PMID: CN-01546444.

65. Plow EB, Cunningham DA, Varnerin N, Janini D, Sakaie K, Frost F. Cortical plasticity to improve rehabilitative outcomes in patients with quadriplegia. Journal of spinal cord medicine. 2014;37(4):473-4. doi: 10.1179/1079026814Z. PubMed PMID: CN-01056930.

66. Shabalov V, Isagulyan E, Salova E, Tomskiy A. Central Cortex Stimulation (CCS) in the complex treatment of severe pain. Stereotactic and functional neurosurgery Conference: 21st congress of the european society for stereotactic and functional neurosurgery: implementing research in clinical applications maastricht netherlands Conference start: 20140917 conference end: 20140920 Conference publication: (varpagings). 2014;92:s122. doi: 10.1159/000367644. PubMed PMID: CN-01173725.

67. Tazoe T, Perez MA. Selective activation of ipsilateral motor pathways in intact humans. Journal of neuroscience. 2014;34(42):13924-34. doi: 10.1523/JNEUROSCI.1648-14.2014. PubMed PMID: CN-01053633.

68. Yoon EJ, Kim YK, Kim HR, Kim SE, Lee Y, Shin HI. Transcranial direct current stimulation to lessen neuropathic pain after spinal cord injury: a mechanistic PET study.

Neurorehabilitation and neural repair. 2014;28(3):250-9. doi: 10.1177/1545968313507632. PubMed PMID: CN-01120453.

69. Bogdanova Y, Gilbert KS, Kark S, Ho VT, Yee MK, Brown TA, et al. Sleep problems, treatment and recovery in veterans with blast exposure, TBI and PTSD. Archives of physical medicine and rehabilitation. 2015;96(10):e3-e4. PubMed PMID: CN-01164070.

70. Burns N, Bavishi S, Bockbrader M, Basobas B, Nielsen AV. Transcranial direct current stimulation for minimally conscious state: a case report. PM and r. 2015;7(9):S110-. PubMed PMID: CN-01106589.

71. Chi CI. The improvement of the episodic memory in the older adults and its cognitive neural mechanism. http://wwwchictrorgcn/showprojaspx?proj=11410. 2015. PubMed PMID: CN-01308112.

72. Irct2014031611234N. Cumulative effects of transcranial direct current stimulation on meth craving. https://trialsearchwhoint/Trial2aspx?TrialID=IRCT2014031611234N2. 2015. PubMed PMID: CN-01804843.

73. Jprn U. Acupuncture treatment for patients with chronic disorder of consciousness and severe cognitive disorder (higher brain dysfunction) following traffic traumatic brain injury. https://trialsearchwhoint/Trial2aspx?TrialID=JPRN-UMIN0000187

97. 2015. PubMed PMID: CN-01881418.

74. Martial C, Thibaut A, Bruno MA, Wannez S, Chatelle C, Anne-Francoise D, et al. Controlled clinical trial of repeated left prefrontal tDCS in patients with chronic minimally conscious state. European journal of neurology. 2015;22:408-9. doi: 10.1111/ene.12807. PubMed PMID: CN-01086684.

75. Nct. Asia Coma Electrical Stimulation (the ACES Trial). https://clinicaltrialsgov/show/NCT02645578. 2015. PubMed PMID: CN-01554851.

76. Nct. LED Treatment to Improve Cognition and Promote Recovery in TBI. https://clinicaltrialsgov/show/NCT02404402. 2015. PubMed PMID: CN-01598036.

77. Nct. Treatment and Recovery Monitoring of Post TBI Symptoms. https://clinicaltrialsgov/show/NCT02426749. 2015. PubMed PMID: CN-01591562.

78. Nct. Transcranial Magnetic Stimulation (TMS) to Treat mTBI and PTSD. https://clinicaltrialsgov/show/NCT02458521. 2015. PubMed PMID: CN-01590261.

79. Nct. rTMS: A Treatment to Restore Function After Severe TBI. https://clinicaltrialsgov/show/NCT02366754. 2015. PubMed PMID: CN-01480415.

80. Nct. Transcranial Direct Current Stimulation for the Treatment

of Deficits After Traumatic Brain Injury. https://clinicaltrialsgov/show/NCT02613936. 2015. PubMed PMID: CN-01589330.

81. Nct. Effect of Repeated tDCS Sessions on Cognitive Improvement in Patients With Disorders of Consciousness. https://clinicaltrialsgov/show/NCT02394691. 2015. PubMed PMID: CN-01597943.

82. Nct. Fronto-parietal tDCS in Severely Brain Injured Patients With Disorders of Consciousness. https://clinicaltrialsgov/show/NCT02626403. 2015. PubMed PMID: CN-01598970.

83. Nct. The Consciousness Variation of Deep Brain and Transcranial Magnetic Stimulation for Disorders of Consciousness Patients. https://clinicaltrialsgov/show/NCT02667899. 2015. PubMed PMID: CN-01555416.

84. Nct. Conditioning Neural Circuits to Improve Upper Extremity Function. https://clinicaltrialsgov/show/NCT02611375. 2015. PubMed PMID: CN-01553883.

85. Nct. Effects of the Transcranial Stimulation on Prevention of Cognitive Dysfunction in Cardiac Surgery. https://clinicaltrialsgov/show/NCT02549560. 2015. PubMed PMID: CN-01492241.

86. Nct. Primed vs Unprimed Facilitatory and Depressive Paired Associative Stimulation. https://clinicaltrialsgov/show/NCT02619643. 2015. PubMed PMID: CN-01589390.

87. Ngernyam N, Jensen MP, Arayawichanon P, Auvichayapat N, Tiamkao S, Janjarasjitt S, et al. The effects of transcranial direct current stimulation in patients with neuropathic pain from spinal cord injury. Clinical neurophysiology. 2015;126(2):382-90. doi: 10.1016/j.clinph.2014.05.034. PubMed PMID: CN-01048957.

88. Cassano P, Petrie S, Mischoulon D, Ionescu D, Cusin C, Katnani H, et al. Transcranial photobiomodulation for the treatment of major depressive disorder: the ELATED-2 clinical trial. Neuropsychopharmacology Conference: 55th annual meeting of the american college of neuropsychopharmacology, ACNP 2016 United states Conference start: 20161204 Conference end: 20161208. 2016;41:S354-S5. doi: 10.1038/npp.2016.241. PubMed PMID: CN-01296356.

89. Chi CI. An applied research of peripheral nerve rewiring in treating hemiplegic hand after central neurologic injury. https://trialsearchwhoint/Trial2aspx?TrialID=ChiCTR-IIR-16007919. 2016. PubMed PMID: CN-01815991.

90. De Laat F, Santegoets K, Peijnenburg R, Zuiderwijk E, Utens

C. Median nerve stimulation in children and young adults in vegetative or minimally conscious state: study protocol for a randomiszed controlled trial. Brain injury. 2016;30(5-6):508-. doi: 10.3109/02699052.2016.1162060. PubMed PMID: CN-01304359.

91. Hesse S, Werner C, Byhahn M. Non-invasive brain stimulation to improve alertness and awareness of patients with disorders of consciousness: transcranial near-infrared laser therapy vs. Transcranial shock-wave therapy. Neurologie und rehabilitation. 2016;22(4):293-300. PubMed PMID: CN-01298758.

92. Irct201605299014N. Effect of transcranial direct current stimulation versus no stimulation on working memory in elders with normal cognitive status. https://trialsearchwhoint/Trial2aspx?TrialID=IRCT201605299014N101. 2016. PubMed PMID: CN-01902289.

93. Irct2015120625384N. Efficacy of transcranial direct current stimulation in reduction of anxiety and crystalline heroin craving. https://trialsearchwhoint/Trial2aspx?TrialID=IRCT2015120625384N1. 2016. PubMed PMID: CN-01805923.

94. Irct2016012526189N. Evaluation of TDCS for augmentation treatment of major depressive disorder with poor treatment response.
https://trialsearchwhoint/Trial2aspx?TrialID=IRCT2016012526189

N1. 2016. PubMed PMID: CN-01878489.

95. Irct2016013026257N. Effect of transcranial stimulation on healthy older subjects function. https://trialsearchwhoint/Trial2aspx?TrialID=IRCT2016013026257N1. 2016. PubMed PMID: CN-01874119.

96. Jprn U. the effect of transcranial magnetic stimulation therapy in higher brain dysfunction patients. https://trialsearchwhoint/Trial2aspx?TrialID=JPRN-UMIN000023189. 2016. PubMed PMID: CN-01827678.

97. Martens G. Double blind randomized controlled clinical trial of fronto-parietal tdcs in patients with disorders of consciousness. Archives of physical medicine and rehabilitation. 2016;97(10):e33-e4. PubMed PMID: CN-01721885.

98. Martial C, Thibaut A, Bruno MA, Wannez S, Chatelle C, Donneau AF, et al. Five days of left prefrontal transcranial direct current stimulation improve signs of consciousness in 50% of chronic minimally conscious state. Brain injury. 2016;30(5-6):520-1. doi: 10.3109/02699052.2016.1162060. PubMed PMID: CN-01304309.

99. Nct. tDCS on Precuneus in MCS: Repeated Stimulations. https://clinicaltrialsgov/show/NCT02702362. 2016. PubMed PMID: CN-01556348.

100. Nct. fMRI-neuronavigated rTMS for the Treatment of Major Depression Associated With TBI. https://clinicaltrialsgov/show/NCT02980484. 2016. PubMed PMID: CN-02039937.

101. Nct. Neurorehabilitation After Traumatic Brain Injury: Functional Magnetic Resonance Imaging Study. https://clinicaltrialsgov/show/NCT02698449. 2016. PubMed PMID: CN-01556236.

102. Nct. Transcranial Direct Current Stimulation (TDCS) as an Intervention for Patients With Traumatic Brain Injury. https://clinicaltrialsgov/show/NCT02849223. 2016. PubMed PMID: CN-01583032.

103. Nct. Two Intensities of Transcranial Direct Current Stimulation to Improve Consciousness in Severely Brain Injured Patients. https://clinicaltrialsgov/show/NCT03007784. 2016. PubMed PMID: CN-01560967.

104. Nct. Corticospinal Excitability After rTMS in Spinal Cord Injury Patients. https://clinicaltrialsgov/show/NCT03014999. 2016. PubMed PMID: CN-01592507.

105. Nct. Impact of Celecoxib on Electrophysiological Property in Brain of Healthy Volunteer. https://clinicaltrialsgov/show/NCT02711579. 2016. PubMed PMID:

CN-02036454.

106. Nct. tDCS Associated With Peripheral Electrical Stimulation for Pain Control in Individuals With Sickle Cell Disease. https://clinicaltrialsgov/show/NCT02813629. 2016. PubMed PMID: CN-02042100.

107. Nct. Investigating the Physiological, Cognitive, and Psychological Effects of a Cognitive-Based Intervention. https://clinicaltrialsgov/show/NCT02906449. 2016. PubMed PMID: CN-01520917.

108. Nct. Augmentation of Locomotor Adaptation Post-Stroke. https://clinicaltrialsgov/show/NCT02892084. 2016. PubMed PMID: CN-01580117.

109. Nekkanti S, Shaik R, Mondem S, Meruva N, Elumalai G. Effect of right median nerve stimulation on level of consciousness in traumatic brain injury subjects. Asian journal of pharmaceutical research and health care. 2016;8(3):67-71. doi: 10.18311/ajprhc/2016/751. PubMed PMID: CN-01165502.

110. Neville IS, Hayashi CY, Rodrigues P, Galhardoni RRG, Sousa Jr LM, Rios RM. High-frequency rTMS for traumatic brain injury: tolerability and safety considerations (10743). Neuromodulation. 2016;19(3):e71. doi: 10.1111/ner.12428. PubMed PMID: CN-01214457.

111. Raithatha R, Carrico C, Powell ES, Westgate PM, Chelette Ii KC, Lee K, et al. Non-invasive brain stimulation and robot-assisted gait training after incomplete spinal cord injury: a randomized pilot study. NeuroRehabilitation. 2016;38(1):15-25. doi: 10.3233/NRE-151291. PubMed PMID: CN-01137986.
112. Simpson M, Churilov L, Bardsley B, Heriot E, Dimovitis J, Carey L, et al. The Effects of modified-release fampridine on upper limb impairment in multiple sclerosis. Multiple sclerosis (Houndmills, Basingstoke, England). 2016;22:685-. doi: 10.1177/1352458516663086. PubMed PMID: CN-01212377.
113. Thibaut A. Controlled clinical trial of 4 weeks of home-based tDCS in patients with chronic minimally conscious state. Archives of physical medicine and rehabilitation. 2016;97(10):e23-. PubMed PMID: CN-01784253.
114. Yozbatiran N, Keser Z, Davis M, Stampas A, O'Malley MK, Cooper-Hay C, et al. Transcranial direct current stimulation (tDCS) of the primary motor cortex and robot-assisted arm training in chronic incomplete cervical spinal cord injury: a proof of concept sham-randomized clinical study. NeuroRehabilitation. 2016;39(3):401-11. doi: 10.3233/NRE-161371. PubMed PMID: CN-01210967.
115. 25. Jahrestagung der Deutschen Gesellschaft fur

Neurorehabilitation e. V., DGNR 2017. Neurologie und rehabilitation. 2017;23. PubMed PMID: CN-01462366.
116. Abreu V, Vaz R, Rebelo V, Rosas MJ, Chamadoira C, Gillies MJ, et al. Thalamic Deep Brain Stimulation for Neuropathic Pain: efficacy at Three Years' Follow-Up. Neuromodulation. 2017;20(5):504-13. doi: 10.1111/ner.12620. PubMed PMID: CN-01396083.
117. Actrn. Theta Burst Stimulation in fibromyalgia. https://trialsearchwhoint/Trial2aspx?TrialID=ACTRN12617000487314. 2017. PubMed PMID: CN-01817044.
118. Alohali M, Alhussien A, Alghamdi F, Alatawi A, Almusned T, Al-Hussain F, et al. Safety and tolerability of transcranial direct current stimulation in midle east. Brain stimulation. 2017;10(4):e46-e7. doi: 10.1016/j.brs.2017.04.094. PubMed PMID: CN-01399454.
119. Ctri. Effect of Magnet treatment in Head injury patients. https://trialsearchwhoint/Trial2aspx?TrialID=CTRI/2017/02/007789. 2017. PubMed PMID: CN-01818882.
120. Estraneo A, Fiorenza S, Pascarella A, Moretta P, Masotta O, Loreto V, et al. Transcranial direct current stimulation in prolonged disorders of consciousness. Clinical neurophysiology. 2017;128(12):e417-. doi: 10.1016/j.clinph.2017.09.016. PubMed

PMID: CN-01438055.

121. Estraneo A, Pascarella A, Moretta P, Masotta O, Fiorenza S, Chirico G, et al. Transcranial direct current stimulation in prolonged disorders of consciousness: a randomized controlled pilot study. Brain injury. 2017;31(6-7):855-. doi: 10.1080/02699052.2017.1312145. PubMed PMID: CN-01399411.

122. Hanna K, Sambrook P, Armfield J, Brennan D. Online resources provision and wisdom teeth extractions: a pilot randomized controlled trial. Journal of dental research. 2017;96(Spec Iss B). PubMed PMID: CN-02344379.

123. Irct2017011932053N. Investigating the effect of rehabilitation with and without rTMS (repetitive transcranial magnetic stimulation) on upper extremity motor deficit of traumatic brain injury. https://trialsearchwhoint/Trial2aspx?TrialID=IRCT2017011932053N1. 2017. PubMed PMID: CN-01807694.

124. Irct2017020311885N. Effects of Transcranial direct currency stimulation (tDCS) on the treatment of depressive and anxiety symptoms and improve quality of sleep in patients with chronic renal diseases on dialysis. https://trialsearchwhoint/Trial2aspx?TrialID=IRCT2017020311885N8. 2017. PubMed PMID: CN-01840221.

125. Irct2017030532884N. Effect of combining tDCS and behavioral intervention on children with autism spectrum disorder. https://trialsearchwhoint/Trial2aspx?TrialID=IRCT2017030532884N1. 2017. PubMed PMID: CN-01815633.
126. Kaminski E, Hoff M, Rjosk V, Steele CJ, Gundlach C, Sehm B, et al. Anodal transcranial direct current stimulation does not facilitate dynamic balance task learning in healthy old adults. Frontiers in human neuroscience. 2017;11(no pagination). doi: 10.3389/fnhum.2017.00016. PubMed PMID: CN-01336954.
127. Martens G, Thibaut A, Huang W, Di H, Laureys S. Repeated stimulation of the posterior parietal cortex in patients in minimally conscious state: a sham-controlled randomized clinical trial. Brain stimulation. 2017;10(2):370-. doi: 10.1016/j.brs.2017.01.090. PubMed PMID: CN-01361661.
128. Martens G, Thibaut A, Huang W, Laureys S. Repeated transcranial direct current stimulation of the posterior parietal cortex in patients in minimally conscious state: a shamcontrolled randomized clinical trial. Brain injury. 2017;31(6-7):753-. doi: 10.1080/02699052.2017.1312145. PubMed PMID: CN-01399420.
129. Martens G, Thibaut A, Laureys S. Controlled clinical trial of 4 weeks of homebased tDCS in patients with chronic minimally conscious state. Brain injury. 2017;31(6-7):753-4. doi:

10.1080/02699052.2017.1312145. PubMed PMID: CN-01399419.

130. McGarry A, Kieburtz K, Abler V, Grachev ID, Gandhi S, Auinger P, et al. Safety and Exploratory Efficacy at 36 Months in Open-HART, an Open-Label Extension Study of Pridopidine in Huntington's Disease. Journal of huntington's disease. 2017;6(3):189-99. doi: 10.3233/JHD-170241. PubMed PMID: CN-01422679.

131. Moustafa IM, Diab AA, Harrison DE. The effect of normalizing the sagittal cervical configuration on dizziness, neck pain, and cervicocephalic kinesthetic sensibility: a 1-year randomized controlled study. European journal of physical and rehabilitation medicine. 2017;53(1):57-71. doi: 10.23736/S1973-9087.16.04179-4. PubMed PMID: CN-01413906.

132. Nct. Transcranial Direct Current Stimulation and Computer-based Training for the Traumatic Brain Injury Rehabilitation. https://clinicaltrialsgov/show/NCT03382626. 2017. PubMed PMID: CN-01566963.

133. Nct. Managing MTBI-related Headaches With rTMS. https://clinicaltrialsgov/show/NCT03314584. 2017. PubMed PMID: CN-01564973.

134. Nct. Transcranial Magnetic Stimulation in Patients With Disorders of Consciousness.

https://clinicaltrialsgov/show/NCT03385278. 2017. PubMed PMID: CN-02038968.

135. Nct. Neurophysiological Effects of tPCS and tDCS on Patients With Disorders of Consciousness. https://clinicaltrialsgov/show/NCT03115021. 2017. PubMed PMID: CN-01575917.

136. Nct. Long-term Effect of tDCS in Patients With Disorders of Consciousness. https://clinicaltrialsgov/show/NCT03114397. 2017. PubMed PMID: CN-01575908.

137. Nct. Transcranial Direct Current Stimulation to Enhance Rehabilitation in Individuals With Rotator Cuff Tendinopathy. https://clinicaltrialsgov/show/NCT03104218. 2017. PubMed PMID: CN-01563191.

138. Potter-Baker KA, Frost F, Plow EB. It's all in your head: driving cortical plasticity to improve muscle contraction below the level of injury. Journal of spinal cord medicine. 2017;40(5):592-. doi: 10.1080/10790268.2017.1351703. PubMed PMID: CN-01416408.

139. Rutherford G, Mansouri B, Zhang W, Wang X, Sulieman A, Ranjbar O, et al. RTMS as a treatment for mild traumatic brain injury. Brain stimulation. 2017;10(2):481-. doi: 10.1016/j.brs.2017.01.409. PubMed PMID: CN-01361738.

140. Shaw M, Dobbs B, Pawlak N, Pau W, Sherman K, Bikson M, et al. Updated safety and tolerability of remotely-supervised transcranial direct current stimulation (RS-tDCS). Brain stimulation. 2017;10(4):e60-e1. doi: 10.1016/j.brs.2017.04.106. PubMed PMID: CN-01399491.

141. Shin HW, Hallett M, Sohn Y. Low-frequency repetitive transcranial cerebellar magnetic stimulation as an 'add-on' therapy in patients with Essential Tremor. Movement disorders. 2017;32:507-9. doi: 10.1002/mds.27087. PubMed PMID: CN-01712971.

142. Siddiqi SH, Trapp NT, Kandala S, Hacker CD, Hong X, Carter AC, et al. Individualized connectome mapping to target rTMS for treatment-resistant depression in traumatic brain injury. Journal of neuropsychiatry and clinical neurosciences. 2017;29(3):e27-. doi: 10.1176/appi.neuropsych.16110299. PubMed PMID: CN-01470441.

143. Siddiqi SH, Trapp NT, Kandala S, Laumann TO, Hacker CD, Hong X, et al. Repetitive transcranial magnetic stimulation with resting-state network targeting for depression in traumatic brain injury. Journal of ECT. 2017;33(3):212-. doi: 10.1097/YCT.0000000000000429. PubMed PMID: CN-01461833.

144. Thibaut A, Martens G, Laureys S. Multichannel tDCS of the

frontoparietal network in patients with disorders of consciousness: a double blind sham controlled randomized clinical trial. Brain stimulation. 2017;10(4):e40-. doi: 10.1016/j.brs.2017.04.074. PubMed PMID: CN-01399464.

145. Tyler WJ. Transdermal electrical neuromodulation of the trigeminal sensory nuclear complex improves sleep quality. Sleep medicine. 2017;40:e330-. PubMed PMID: CN-01463341.

146. Wardini R, Moses M. Portable neuromodulation stimulation (PoNSTM) therapy efficacy for the treatment of traumatic brain injury compared to standard of care. Brain injury Conference: 12th world congress on brain injury of the international brain injury association United states. 2017;31(6-7):806. doi: 10.1080/02699052.2017.1312145. PubMed PMID: CN-01399406.

147. Baniqued PL, Gallen CL, Voss MW, Burzynska AZ, Wong CN, Cooke GE, et al. Brain network modularity predicts exercise-related executive function gains in older adults. Frontiers in aging neuroscience. 2018;9(JAN) (no pagination). doi: 10.3389/fnagi.2017.00426. PubMed PMID: CN-01449590.

148. ChiCtr. A prospective study of prognosis in Children with Disorder of consciousness and Wake-promoting actions of median nerve stimulation of DoC in children. https://trialsearchwhoint/Trial2aspx?TrialID=ChiCTR1800019330.

2018. PubMed PMID: CN-01950673.

149. ChiCtr. Effects of Transcranial Direct Current Stimulation on Patients With Disorders of Consciousness. https://trialsearchwhoint/Trial2aspx?TrialID=ChiCTR1800014808. 2018. PubMed PMID: CN-01899537.

150. Guernon A, Larson E, Sayyad A, Brady S, Bou S, Pape T. Innovative approach using neuromodulation techniques as a treatment for TBI related cognitive impairments. Journal of head trauma rehabilitation. 2018;33(3):E93-E4. doi: 10.1097/HTR.0000000000000401. PubMed PMID: CN-01612454.

151. Irct20170808035562N. ?Effect of transcranial direct current stimulation on drug craving of addicts. https://trialsearchwhoint/Trial2aspx?TrialID=IRCT2017080803556 2N2. 2018. PubMed PMID: CN-01909612.

152. Irct20171223038019N. Effect of rTMS on severity of Delirium. https://trialsearchwhoint/Trial2aspx?TrialID=IRCT2017122303801 9N1. 2018. PubMed PMID: CN-01902368.

153. Nct. Efficacy of Repetitive Transcranial Magnetic Stimulation for Improvement of Memory in Older Adults With TBI. https://clinicaltrialsgov/show/NCT03727737. 2018. PubMed PMID: CN-01794719.

154. Nct. fMRI-neuronavigated rTMS Treatment for Symptoms of Depression Associated With Concussive TBI in the Military Population. https://clinicaltrialsgov/show/NCT03523507. 2018. PubMed PMID: CN-01574383.
155. Nct. rTMS for Cognitive Rehabilitation After TBI. https://clinicaltrialsgov/show/NCT03642158. 2018. PubMed PMID: CN-01662504.
156. Nct. rTMS Treatment of Persistent Headache and Post Concussion Symptoms Attributed to Mild Traumatic Injury to the Head. https://clinicaltrialsgov/show/NCT03691272. 2018. PubMed PMID: CN-01663757.
157. Nct. The Effects of Low-intensity Blood-flow Restricted Exercise on Upper Limb Function Following Spinal Cord Injury. https://clinicaltrialsgov/show/NCT03690700. 2018. PubMed PMID: CN-01663748.
158. Nct. Investigation and Modulation of the Mu-opioid Mechanisms in TMD (in Vivo). https://clinicaltrialsgov/show/NCT03724032. 2018. PubMed PMID: CN-01964986.
159. Nct. Grasping Function After Spinal Cord Injury. https://clinicaltrialsgov/show/NCT03447509. 2018. PubMed PMID: CN-01589533.

160. Nct. Non-Invasive Stimulation for Improving Motor Function. https://clinicaltrialsgov/show/NCT03592173. 2018. PubMed PMID: CN-01661238.

161. Nct. The Effects of rTMS in Rehabilitation Following Spinal Cord Injury. https://clinicaltrialsgov/show/NCT03690726. 2018. PubMed PMID: CN-01663749.

162. Nct. Combined Robotic Training and tDCS in Chronic SCI. https://clinicaltrialsgov/show/NCT03555838. 2018. PubMed PMID: CN-01609221.

163. Nct. Treatment of Memory Disorders in Gulf War Illness With High Definition Transcranial Direct Cortical Stimulation. https://clinicaltrialsgov/show/NCT03542383. 2018. PubMed PMID: CN-01659983.

164. Nct. Activity-Dependent Transspinal Stimulation in SCI. https://clinicaltrialsgov/show/NCT03669302. 2018. PubMed PMID: CN-01663224.

165. Nct. Neuromodulation for Exercise Adherence. https://clinicaltrialsgov/show/NCT03733041. 2018. PubMed PMID: CN-01658712.

166. Nct. Post-concussion and Transcranial Direct Current Stimulation. https://clinicaltrialsgov/show/NCT03481153. 2018. PubMed PMID: CN-01589260.

167. Nct. CONsciousness Transcranial Electric STimulation. https://clinicaltrialsgov/show/NCT03576248. 2018. PubMed PMID: CN-01660835.

168. Padmanabhan P, Kataria P, Padmanaban V, Srinivas SP, Ambrosio R. Accuracy of integrated corneal biomechanical and tomographic data for detecting subclinical keratectasia. Investigative ophthalmology & visual science. 2018;59(9). PubMed PMID: CN-01960158.

169. Siddiqi SH, Trapp NT, Hacker CD, Laumann TO, Kandala S, Hong X, et al. Repetitive transcranial magnetic stimulation with resting state network targeting for treatment-resistant depression in TBI. Journal of neurotrauma. 2018;35(16):A15-. doi: 10.1089/neu.2018.29013.abstracts. PubMed PMID: CN-01647811.

170. Tctr. Comparison of the mindfulness and transcranial stimulation on brain injury. https://trialsearchwhoint/Trial2aspx?TrialID=TCTR20180827003. 2018. PubMed PMID: CN-01992880.

171. Thibaut A, Martens G, Kroupi E, Aureli Soria-Frisch A, Ruffini G, Laureys S. Stimulation of the frontoparietal network using tDCS in patients with disorders of consciousness: a double blind sham controlled randomised clinical trial. European journal of neurology. 2018;25:562-. PubMed PMID: CN-01630749.

172. Thomas R, Mathew R. Right median nerve electrical stimulation (RMNS) to improve arousal in acquired brain injury patients a randomised controlled trial. Neurorehabilitation and neural repair. 2018;32(4-5):476-. doi: 10.1177/1545968318765498. PubMed PMID: CN-01785701.

173. Yona Rabinovich Y, Shaklai S, Sacher Y, Cismariu Potash K, Ressel Zviely A. The effects of TDCS stimulation on motor learning in healthy and severe sub-acute traumatic brain injury adults. Annals of physical and rehabilitation medicine. 2018;(no pagination). doi: 10.1016/j.rehab.2018.05.086. PubMed PMID: CN-01610899.

174. CTMSS 2019 Annual Meeting. Brain stimulation. 2019;12(4):A1-A10. doi: 10.1016/s1935-861x(19)30251-7. PubMed PMID: CN-01976548.

175. Actrn. Effects of transcranial direct current stimulation for improving attention after traumatic brain injury. https://trialsearchwhoint/Trial2aspx?TrialID=ACTRN12619001523190. 2019. PubMed PMID: CN-02064925.

176. Actrn. A clinical research study investigating repetitive transcranial magnetic stimulation (rTMS) in treating persistent post-concussion symptoms in children compared to placebo/dummy treatment.

https://trialsearchwhoint/Trial2aspx?TrialID=ACTRN12619001118190. 2019. PubMed PMID: CN-01975102.

177. Awosika OO, Sandrini M, Volochayev R, Thompson RM, Fishman N, Wu T, et al. Transcutaneous spinal direct current stimulation improves locomotor learning in healthy humans. Brain Stimulation. 2019;12(3):628-34. doi: 10.1016/j.brs.2019.01.017. PubMed PMID: CN-01794166.

178. Choi YA, Shin HI. Effects of anodal transcutaneous spinal direct current stimulation on chronic neuropathic pain after spinal cord injury: a pilot study. Neuromodulation. 2019;22(7):e492-. doi: 10.1111/ner.12958. PubMed PMID: CN-02164317.

179. Chrysikou EG, Gorey C, Aupperle RL. Proceedings #19: effects of Transcranial Direct Current Stimulation over Prefrontal Cortex for Decision Making During Approach Avoidance Conflict. Brain stimulation. 2019;12(2):e81-e3. doi: 10.1016/j.brs.2018.12.188. PubMed PMID: CN-01788703.

180. De Launay KQ, Cheung ST, Riggs L, Reed N, Beal DS. Abstract #63: the Effect of tDCS on Cognitive Performance in Youth with Concussion: a Pilot Study. Brain stimulation. 2019;12(2):e22-e3. doi: 10.1016/j.brs.2018.12.070. PubMed PMID: CN-01791663.

181. Hayashi C, Neville IS, Rodrigues P, Galhardoni RR, de

Andrade DC, Zaninotto AL, et al. Abstract #77: repetitive Transcranial Magnetic Stimulation (rTMS) in chronic diffuse axonal injury: a randomized controlled trial. Brain stimulation. 2019;12(2):e27-. doi: 10.1016/j.brs.2018.12.084. PubMed PMID: CN-01787181.

182. Hosseini M, McConathey EM, Ungrady M, Grossman M, Coslett HB, Hamilton RH. Proceedings #10: transcranial Direct Current Stimulation Mediates Improvements in Verbal Fluency for Patients with Primary Progressive Aphasia. Brain stimulation. 2019;12(2):e69-e71. doi: 10.1016/j.brs.2018.12.179. PubMed PMID: CN-01793220.

183. Irct20130416013027N. The survey of transcranial direct current stimulation efficacy on the post-traumatic sleep quality, insomnia severity in the patients with mild traumatic brain injury during sub-acute phase. https://trialsearchwhoint/Trial2aspx?TrialID=IRCT20130416013027N2. 2019. PubMed PMID: CN-02068957.

184. Irct20171215037872N. Efficacy of Repetitive Transcranial Magnetic Stimulation to Reduce Muscle Spasticity. https://trialsearchwhoint/Trial2aspx?TrialID=IRCT20171215037872N1. 2019. PubMed PMID: CN-01972328.

185. Isrctn. Can treatment with Cerebrolysin and magnetic

stimulation of the brain improve recovery after traumatic brain injury? https://trialsearchwhoint/Trial2aspx?TrialID=ISRCTN10335247. 2019. PubMed PMID: CN-01947397.

186. Js G, E J, Ja T, Kh B, Kc O, Gover-Chamlou A, et al. Evolution of a Study of Bilateral Prefrontal Transcranial Magnetic Stimulation (TMS) to Treat the Symptoms of Mild TBI (mTBI) and PTSD: research Methods, Participant Demographics, and Tolerability. Brain stimulation. 2019;12(4):e135-. doi: 10.1016/j.brs.2019.03.042. PubMed PMID: CN-01962710.

187. Li L, Violante I, Zimmerman K, Leech R, Hampsire A, Carmichael D, et al. Exploring the targeted application of transcranial direct current stimulation (tDCS) for cognitive modulation after brain injury. Brain stimulation. 2019;12(2):454-. doi: 10.1016/j.brs.2018.12.475. PubMed PMID: CN-01790184.

188. Lowe J, Goodman C, Larson E, Guernon A. Safety and Feasibility of tDCS with Computerized Attention Training after TBI. Archives of physical medicine and rehabilitation. 2019;100(10):e118-. doi: 10.1016/j.apmr.2019.08.351. PubMed PMID: CN-01989744.

189. Martens G, Barra A, Carriere M, Soria-Frisch A, Ruffini G, Ibanez D, et al. Closed-loop application of tDCS to promote

responsiveness in patients with disorders of consciousness. Brain stimulation. 2019;12(2):459-. doi: 10.1016/j.brs.2018.12.493. PubMed PMID: CN-01787170.

190. Nct. Prefontal tDCS in Patients With Disorders of Consciousness: Neurophysiological and Behavioural Outcomes. https://clinicaltrialsgov/show/NCT03823508. 2019. PubMed PMID: CN-01918993.

191. Nct. Transcutaneous Auricular Vagal Nerve Stimulation for Post-coma Patients With Disorders of Consciousness. https://clinicaltrialsgov/show/NCT04065386. 2019. PubMed PMID: CN-01983669.

192. Nct. rTMS Target Identification for Functional Disability in AUD+mTBI. https://clinicaltrialsgov/show/NCT04043442. 2019. PubMed PMID: CN-01966108.

193. Nct. Pilot rTMS for AUD+mTBI. https://clinicaltrialsgov/show/NCT03995173. 2019. PubMed PMID: CN-01946067.

194. Nct. High-Definition Transcranial Direct Current Stimulation (HD-tDCS) for Sensory Deficits in Complex Traumatic Brain Injury. https://clinicaltrialsgov/show/NCT03799458. 2019. PubMed PMID: CN-01965210.

195. Nct. Neurofeedback Impact on Veterans With mTBI.

https://clinicaltrialsgov/show/NCT04195685. 2019. PubMed PMID: CN-02053115.

196. Nct. Neuromodulation and Neurorehabilitation for mTBI Plus PTSD. https://clinicaltrialsgov/show/NCT03819608. 2019. PubMed PMID: CN-01706661.

197. Nct. Closed-loop tDCS in Patients in Minimally Conscious State. https://clinicaltrialsgov/show/NCT03810079. 2019. PubMed PMID: CN-01795963.

198. Nct. Multichannel tDCS to Reduce Hypertonia in Patients With Prolonged DOC. https://clinicaltrialsgov/show/NCT03797573. 2019. PubMed PMID: CN-01795773.

199. Nct. Remotely Supervised Transcranial Direct Current Stimulation for Persistent Post-traumatic Headache. https://clinicaltrialsgov/show/NCT04012853. 2019. PubMed PMID: CN-01953145.

200. Nct. Spinal Cord Stimulation in Patients With Disorders of Consciousness. https://clinicaltrialsgov/show/NCT04010838. 2019. PubMed PMID: CN-01953095.

201. Nct. Cortical Contributions to Motor Sequence Learning. https://clinicaltrialsgov/show/NCT04138953. 2019. PubMed PMID: CN-02001358.

202. Nct. Computerised Working Memory Training in Acquired

Brain Injury. https://clinicaltrialsgov/show/NCT04010149. 2019. PubMed PMID: CN-01953080.

203. Nct. TMS and Exposure Therapy. https://clinicaltrialsgov/show/NCT04019054. 2019. PubMed PMID: CN-01953302.

204. Nct. Improving Hand Recovery With Neuromodulation in Tetraplegia. https://clinicaltrialsgov/show/NCT03954496. 2019. PubMed PMID: CN-01932763.

205. Nct. Subacromial Corticosteroid Injections and tDCS in Rotator Cuff Tendinopathy. https://clinicaltrialsgov/show/NCT03967574. 2019. PubMed PMID: CN-01945158.

206. Nct. Acute Effects of Pharmacological Neuromodulation on Leg Motor Activity in Patients With SCI Treated With EES. https://clinicaltrialsgov/show/NCT04052776. 2019. PubMed PMID: CN-01966345.

207. Nct. Therapeutic Effects of Transcranial Alternative Current Stimulation (tACS) in Chronic Post Stroke Hemianopia. https://clinicaltrialsgov/show/NCT04043689. 2019. PubMed PMID: CN-01966116.

208. Nct. Efficacy of Equine Assisted Therapy on Negative Symptoms in Patients With Schizophrenia.

https://clinicaltrialsgov/show/NCT03935243. 2019. PubMed PMID: CN-01931768.

209. Papa L, Ptito A. Translingual stimulation (TLNS) with the portable neuromodulation stimulator (PoNSTM) for the treatment of chronic balance deficit due to mild-to-moderate traumatic brain injury (mmTBI). Annals of neurology. 2019;86:S173-. PubMed PMID: CN-02095662.

210. Papa L, Ptito A. Translingual neurostimulation with the portable neuromodulation stimulator (PoNSâ„¢) for the treatment of chronic balance deficit due to mild-to-moderate traumatic brain injury. PM and r. 2019;11:S89-S90. doi: 10.1002/pmrj.12271. PubMed PMID: CN-02119961.

211. Papa L, Yochelson M, Walker WC. Improvements across functional outcomes in patients treated for chronic balance deficit after mild-tomoderate traumatic brain injury with translingual neurostimulation. Neuromodulation. 2019;22(7):e445-. doi: 10.1111/ner.12958. PubMed PMID: CN-02130515.

212. Pape TB, Rosenow J, Herrold A, Livengood S, Kletzel S, Guernon A, et al. ReEnabling ConsciOus behaViors by Engaging dopamineRgic pathwaYs (RECOVERY). Brain stimulation. 2019;12(2):559-. doi: 10.1016/j.brs.2018.12.849. PubMed PMID: CN-01790175.

213. Paxman E, Stilling J, Mercier L, Debert C. Repetitive Transcranial Magnetic Stimulation (rTMS) as an Effective Intervention for Chronic Dizziness Following Mild Traumatic Brain Injury: a Case Study. Brain stimulation. 2019;12(2):583-4. doi: 10.1016/j.brs.2018.12.937. PubMed PMID: CN-01788612.
214. Ptito A. Treatment of chronic symptoms of mild/moderate traumatic brain injury with the portable neuromodulation stimulator. Neuromodulation. 2019;22(3):E88-. doi: 10.1111/ner.12950. PubMed PMID: CN-01941442.
215. Ptito A, Papa L, Skinner K, Tyler M. Predicting therapeutic responses to cranial nerve noninvasive translingual neurostimulation in persons with mild-to-moderate traumatic brain injury using pooled data from two randomized clinical trials. Neuromodulation. 2019;22(7):e446-. doi: 10.1111/ner.12958. PubMed PMID: CN-02164356.
216. Ptito A, Skinner K, Papa L. The Portable Neuromodulation Stimulator for Chronic Symptoms Due to Mild-to-Moderate Traumatic Brain Injury. Archives of physical medicine and rehabilitation. 2019;100(10):e132-e3. doi: 10.1016/j.apmr.2019.08.402. PubMed PMID: CN-01987494.
217. Rackoll T, Darkow R, Wusten K, Floel A, Gorsler A. Proof of principle: traumatic brain injury speech rehabilitation under direct

current stimulation (BISCUIT). Clinical neurophysiology. 2019;130(8):e149-. doi: 10.1016/j.clinph.2019.04.663. PubMed PMID: CN-02081158.

218. Rosenow JM, Kletzel S, Herrold A, Guernon A, Patil V, Bender-Pape TL. Amantadine + RTMS as a neurotherapeutic for disordered consciousness after TBI: safety findings. Neuromodulation. 2019;22(3):E202-. doi: 10.1111/ner.12950. PubMed PMID: CN-01941712.

219. Saleh Velez F, Bonin Pinto C, Ortiz JG, Mansour A, Lazaridis C. The PASHT trial: randomized crossover sham-controlled trial evaluating safety and feasibility of transcranial direct current stimulation (TDCS) for paroxysmal sympathetic hyperactivity post stroke and TBI. European stroke journal. 2019;4:94-. doi: 10.1177/2396987319845578. PubMed PMID: CN-01961249.

220. Schenker E. Proceedings #7: trigeminal and Occipital Neuromodulation for Rapid Pain Reduction in Occipital Migraines. Brain stimulation. 2019;12(2):e65-e6. doi: 10.1016/j.brs.2018.12.176. PubMed PMID: CN-01925716.

221. Siddiqi S, Trapp N, Hacker C, Kandala S, Leuthardt E, Carter A, et al. Functional connectivity changes with targeted rTMS of the dorsal attention network in TBI-associated depression. Brain stimulation. 2019;12(2):538-. doi: 10.1016/j.brs.2018.12.773.

PubMed PMID: CN-01787184.
222. Siddiqi SH, Trapp NT, Hacker CD, Laumann TO, Kandala S, Hong X, et al. Abstract #156: functional connectivity changes with targeted rTMS of the dorsal attention network in TBI-associated depression. Brain stimulation. 2019;12(2):e53-. doi: 10.1016/j.brs.2018.12.163. PubMed PMID: CN-01788649.
223. Skinner K, Prabhakaran V. Neurorehabilitation using the portable neuromodulation stimulator (PoNSâ„¢) for treating participants with chronic balance deficit due to mild-to-moderate traumatic brain injury: a 26-week study. PM and r. 2019;11:S34-. doi: 10.1002/pmrj.12271. PubMed PMID: CN-02120108.
224. Skinner K, Tyler M. Long-Term Treatment of Balance Deficits After Mild-to-Moderate TBI With the Portable Neuromodulation Stimulator. Archives of physical medicine and rehabilitation. 2019;100(10):e9-. doi: 10.1016/j.apmr.2019.08.015. PubMed PMID: CN-01989746.
225. Straughn J, Denais Z. Application of Right Median Nerve Stimulation in an Inpatient Rehabilitation Setting for Disorders of Consciouness. Archives of physical medicine and rehabilitation. 2019;100(10):e44-. doi: 10.1016/j.apmr.2019.08.115. PubMed PMID: CN-01987488.
226. Tyler M, Skinner K, Prabhakaran V. Long-term treatment trial

of the portable neuromodulation stimulator (PoNSTM) for treatment of chronic balance deficit due to mild-to-moderate traumatic brain injury (mmTBI). Annals of neurology. 2019;86:S80-S1. PubMed PMID: CN-02095964.

227. Willms M, Brucar L, Muller A, Vila-Rodrigues F, Rosenblatt C, Babul NV. Exploring tDCS-induced changes in EEG power and network connectivity in youth concussion: preliminary findings. Brain stimulation. 2019;12(2):468-. doi: 10.1016/j.brs.2018.12.525. PubMed PMID: CN-01788611.

228. Actrn. Effect of strong magnetic fields on the communication between brain and arm after spinal cord injury. https://trialsearchwhoint/Trial2aspx?TrialID=ACTRN12620000746932. 2020. PubMed PMID: CN-02165428.

229. Actrn. Effect of individualised transcranial magnetic stimulation (TMS) on cognitive functioning in healthy volunteers. https://trialsearchwhoint/Trial2aspx?TrialID=ACTRN12620000162910. 2020. PubMed PMID: CN-02165087.

230. Adamson M, McNerney MW, Siddiqi S, Swaminath G, Wu LL, Hernandez B, et al. Default Mode Network Connectivity and Brain Derived Trophic Factor in Brain Stimulation of TBI patients. Biological psychiatry. 2020;87(9):S158-. doi: 10.1016/j.biopsych.2020.02.419. PubMed PMID: CN-02120338.

231. Carriere M, Barra A, Mortaheb S, Binda Fossati M, Martens G, Bodien Y, et al. Neurophysiological effects and behavioral outcomes after tPCS and tDCS in a patient in minimally conscious state. Clinical neurophysiology. 2020;131(4):e116-e7. doi: 10.1016/j.clinph.2019.12.292. PubMed PMID: CN-02096994.

232. Choi KJ, Nam GB, Zo JH, Lee JH, Cho MS, Kim J, et al. Comparison catheter ablation versus permanent pacemaker implantation in patients with tachycardia-bradycardia syndrome: prospective, randomized trial. European heart journal. 2020;41(SUPPL 2):712-. doi: 10.1093/ehjci/ehaa946.0712. PubMed PMID: CN-02243003.

233. Ctri. Non-invasive Brain Stimulation to Improve Cognition In Traumatic Brain Injury Patients. https://trialsearchwhoint/Trial2aspx?TrialID=CTRI/2020/04/024781. 2020. PubMed PMID: CN-02167794.

234. Ctri. Effect of direct current stimulation of brain on altered consciousness patients. https://trialsearchwhoint/Trial2aspx?TrialID=CTRI/2020/07/026553. 2020. PubMed PMID: CN-02185320.

235. Elmaadawi AZ, Robert Strawn J, Aaronson ST, Randolph Schrodt G, Holbert RC, Verdoliva S, et al. 24.3 HIGH-FREQUENCY TRANSCRANIAL MAGNETIC

STIMULATION FOR TREATMENT-RESISTANT DEPRESSION IN ADOLESCENTS: THE FIRST MULTICENTER ACTIVE VS SHAM MULTICENTER TRIAL. Journal of the American Academy of Child and Adolescent Psychiatry. 2020;59(10):S199-S200. doi: 10.1016/j.jaac.2020.08.235. PubMed PMID: CN-02192226.
236. Euctr DK. Aciclovir for treatment of meningitis caused by HSV-2 virus. https://trialsearchwhoint/Trial2aspx?TrialID=EUCTR2020-000033-41-DK. 2020. PubMed PMID: CN-02169561.
237. Garland JS, Gbade-Alabi O, Taylor JA, Atwal N, Pasquina PF. A Study of Bilateral Prefrontal Repetitive Transcranial Magnetic Stimulation (rTMS) to Treat the Symptoms of Mild TBI (mTBI) and PTSD: preliminary Tolerability and Effectiveness. Brain stimulation. 2020;13(6):1848-. doi: 10.1016/j.brs.2020.06.037. PubMed PMID: CN-02212144.
238. Garland JS, Jaskot EM, Taylor J, Buccellato K, Atwal N, Pasquina PF. 135 Evolution of a Study of Bilateral Prefrontal Transcranial Magnetic Stimulation (TMS) to Treat the Symptoms of Mild TBI (mTBI) and PTSD. CNS spectrums. 2020;25(2):285-6. doi: 10.1017/S1092852920000516. PubMed PMID: CN-02121330.
239. Garland JS, Jaskot EM, Taylor J, Buccellato K, Atwal N,

Pasquina PF. Evolution of a study of bilateral prefrontal transcranial magnetic stimulation (TMS) to treat the symptoms of mild TBI (mTBI) and PTSD. CNS spectrums. 2020;25(2):285-6. doi: 10.1017/S1092852920000188. PubMed PMID: CN-02121329.
240. Iovoli AJ, Ostrowski A, Rivers CI, Hermann GM, Groman A, Miller A, et al. Two- Versus Four-Times Weekly Acupuncture-Like Transcutaneous Electrical Nerve Stimulation for Treatment of Radiation-Induced Xerostomia: A Pilot Study. Journal of alternative and complementary medicine (New York, NY). 2020;26(4):323-8. doi: 10.1089/acm.2019.0131. PubMed PMID: CN-02073103.
241. Irct20191016045135N. impact of Transcranial direct current stimulation (TDCS) on moral decision making in stroke patients. https://trialsearchwhoint/Trial2aspx?TrialID=IRCT2019101604513 5N1. 2020. PubMed PMID: CN-02238533.
242. Irct20191229045931N. Effectiveness of Combined Transcranial Direct current Stimulation (tDCS) with Pharmacotherapy and Pharmacotherapy in Bipolar Disorders. https://trialsearchwhoint/Trial2aspx?TrialID=IRCT2019122904593 1N1. 2020. PubMed PMID: CN-02187673.
243. Kct. Effect of transcranial direct current stimulation on cognitive function in traumatic brain injury patients.

https://trialsearchwhoint/Trial2aspx?TrialID=KCT0005302. 2020. PubMed PMID: CN-02188991.

244. Kopell B, Leung A, Levy R, Poree L, Wasserman E, Adamson MM, et al. Transcranial Magnetic Stimulation for Pain, Headache and co-morbid depression: INS-NANS Expert Consensus Panel Review and Recommendation. Brain stimulation. 2020;13(6):1851-. doi: 10.1016/j.brs.2020.06.046. PubMed PMID: CN-02212142.

245. Leung A, Ettenhofer M, Sargent P, Liu J, Poree L, Kopell B, et al. Transcranial magnetic stimulation for persistent post-traumatic headache-an INS/NANS expert consensus panel review and recommendation. Neuromodulation. 2020;23(3):e35-e6. doi: 10.1111/ner.13133. PubMed PMID: CN-02243846.

246. McGarry A, Auinger P, Kieburtz K, Geva M, Mehra M, Abler V, et al. Additional Safety and Exploratory Efficacy Data at 48 and 60 Months from Open-HART, an Open-Label Extension Study of Pridopidine in Huntington Disease. Journal of huntington's disease. 2020;9(2):173-84. doi: 10.3233/JHD-190393. PubMed PMID: CN-02142063.

247. Nct. Spectral Correlates of Impulsivity in Patients With Traumatic Brain Injury. https://clinicaltrialsgov/show/NCT04239274. 2020. PubMed PMID: CN-02079648.

248. Nct. Repetitive Transcranial Magnetic Stimulation in Traumatic Brain Injury. https://clinicaltrialsgov/show/NCT04573413. 2020. PubMed PMID: CN-02181687.
249. Nct. Treatment of Post-concussion Syndrome With TMS: Using FNIRS as a Biomarker of Response. https://clinicaltrialsgov/show/NCT04568369. 2020. PubMed PMID: CN-02181558.
250. Nct. Noninvasive Brain Stimulation on Memory in Individuals With Mild Cognitive Impairment and History of Brain Injury. https://clinicaltrialsgov/show/NCT04504630. 2020. PubMed PMID: CN-02145932.
251. Nct. The Effects of Transcranial Direct Current Stimulation (tDCS) and Cognitive Training in Patients With TBI. https://clinicaltrialsgov/show/NCT04540783. 2020. PubMed PMID: CN-02148383.
252. Nct. Neural, Behavioural, and Clinical Effects of tDCS in PDOC; Feasibility Study. https://clinicaltrialsgov/show/NCT04248946. 2020. PubMed PMID: CN-02079911.
253. Nct. Transcranial Direct Current Stimulation (tDCS) Neuromodulation of Executive Function Across Neuropsychiatric

Populations. https://clinicaltrialsgov/show/NCT04296604. 2020. PubMed PMID: CN-02088785.
254. Nct. Does Physiotherapy Plus Non-invasive Brain Stimulation Improve Motor Recovery in Children With Acquired Brain Injury? https://clinicaltrialsgov/show/NCT04422886. 2020. PubMed PMID: CN-02125354.
255. Nct. BoMI for Muscle Control. https://clinicaltrialsgov/show/NCT04641793. 2020. PubMed PMID: CN-02209200.
256. Nct. Low Intensity Focused Ultrasound Pulses (LIFUP) to Modulate Pain. https://clinicaltrialsgov/show/NCT04339972. 2020. PubMed PMID: CN-02091502.
257. Nct. Physiological Changes Induced Through MEP Conditioning in People With SCI. https://clinicaltrialsgov/show/NCT04286191. 2020. PubMed PMID: CN-02088552.
258. Nct. Balance Training With tDCS for CAI. https://clinicaltrialsgov/show/NCT04390048. 2020. PubMed PMID: CN-02180589.
259. Nct. Treating Self Injurious Behavior: A Novel Brain Stimulation Approach. https://clinicaltrialsgov/show/NCT04244786. 2020. PubMed PMID:

CN-02079803.

260. Nct. The Effect of Dorsolateral Prefrontal Cortex Theta Burst Stimulation on Alcohol Cue Reactivity and Cognitive Control: a Double-blind, Sham Controlled Study of Heavy Alcohol Drinkers With a History of Alcohol Related Injury. https://clinicaltrialsgov/show/NCT04223154. 2020. PubMed PMID: CN-02079220.

261. Nct. rTMS and EEG in DOC Patients. https://clinicaltrialsgov/show/NCT04401319. 2020. PubMed PMID: CN-02180660.

262. Nct. Modulating Neuropathic Pain With Transcranial Direct Current Stimulation. https://clinicaltrialsgov/show/NCT04581525. 2020. PubMed PMID: CN-02174612.

263. Nct. Transcranial Direct Current Stimulation for Attention Deficit. https://clinicaltrialsgov/show/NCT04278430. 2020. PubMed PMID: CN-02088357.

264. Nct. Neuromuscular Control in Individuals Following ACL-Reconstruction. https://clinicaltrialsgov/show/NCT04495075. 2020. PubMed PMID: CN-02145754.

265. Rosenfelder M, Wolff A, Niimi M, Sanz L, Martens G, Laureys S, et al. Repetitive transcranial magnetic stimulation and electroencephalography in patients with disorders of consciousness:

protocol of a multi-center RCT. Clinical neurophysiology. 2020;131(4):e145-e6. doi: 10.1016/j.clinph.2019.12.338. PubMed PMID: CN-02100918.

266. Singh KR, Sharma RM, Bhattacharyya D. Role of rTMS as an adjunctive treatment in treatment resistant OCD: a randomised double blind and sham control trial. Indian journal of psychiatry. 2020;62(7):S97-S8. PubMed PMID: CN-02101348.

267. Vaninetti M, Lim M, Khalaf A, Smith VM, Flowers M, Kunnel A, et al. Cortical pain modulatory functional enhancement after rTMS associated with durable headache improvement in mTBI patients. Brain stimulation. 2020;13(6):1853-. doi: 10.1016/j.brs.2020.06.051. PubMed PMID: CN-02264756.

268. xmkyy RBR. Neurostimulation and memory training in depression. https://trialsearchwhoint/Trial2aspx?TrialID=RBR-3xmkyy. 2020. PubMed PMID: CN-02189333.

269. Zhao CG, Sun W, Ju F, Wang H, Sun XL, Mou X, et al. Analgesic Effects of Directed Repetitive Transcranial Magnetic Stimulation in Acute Neuropathic Pain After Spinal Cord Injury. Pain medicine (Malden, Mass). 2020;21(6):1216-23. doi: 10.1093/pm/pnz290. PubMed PMID: CN-02009667.

270. Adamson M, McNerney MW, Siddiqi S, Swaminath G, Wu

LL, Hernandez B, et al. Default mode network connectivity and brain derived neurotrophic factor in brain stimulation of traumatic brain injury patients. Brain stimulation. 2021;14(6):1633-. doi: 10.1016/j.brs.2021.10.146. PubMed PMID: CN-02345974.

271. Andre-Obadia N, Magnin M, Garcia-Larrea L. Theta-burst versus 20 Hz repetitive transcranial magnetic stimulation in neuropathic pain: A head-to-head comparison. Clinical Neurophysiology. 2021;132(10):2702-10. doi: 10.1016/j.clinph.2021.05.022. PubMed PMID: CN-02309283.

272. ChiCtr. The awakening effect of neuromodulation technology on patients with disorders of consciousness: a multi-center, double blind, randomized controlled clinical trial. https://trialsearchwhoint/Trial2aspx?TrialID=ChiCTR2100043784. 2021. PubMed PMID: CN-02280865.

273. ChiCtr. Repeated transcranial direct current stimulation in disorders of consciousness: a randomized, double-blind controlled trial study. https://trialsearchwhoint/Trial2aspx?TrialID=ChiCTR2100053108. 2021. PubMed PMID: CN-02349387.

274. ChiCtr. Effectiveness of transcranial magnetic stimulation on awaking patients with disorders of consciousness: a randomized controlled trial.

https://trialsearchwhoint/Trial2aspx?TrialID=ChiCTR2100044930. 2021. PubMed PMID: CN-02349205.

275. Deng ZD, McClintock S, Husain M, Lisanby S. Antidepressant response of electroconvulsive therapy and magnetic seizure therapy: response trajectories by symptom clusters. Neuropsychopharmacology. 2021;46:226-. doi: 10.1038/s41386-021-01237-6. PubMed PMID: CN-02356325.

276. Fann JR, Quinn DK, Hart T. Treatment of Psychiatric Problems After Traumatic Brain Injury. Biological psychiatry. 2021. doi: 10.1016/j.biopsych.2021.07.008. PubMed PMID: CN-02326463.

277. Flanagan SD, Proessl F, Dunn-Lewis C, Sterczala AJ, Connaboy C, Canino MC, et al. Differences in brain structure and theta burst stimulation-induced plasticity implicate the corticomotor system in loss of function after musculoskeletal injury. Journal of neurophysiology. 2021;125(4):1006-21. doi: 10.1152/jn.00689.2020. PubMed PMID: CN-02259029.

278. Huffman S, Badran B, Dancy M, Austelle C, Kautz S, George M. At-Home Telemedicine Controlled taVNS Twice Daily for 4 weeks is Feasible and Safe for Long COVID Symptoms. Brain stimulation. 2021;14(6):1702-3. doi: 10.1016/j.brs.2021.10.367. PubMed PMID: CN-02345944.

279. Kct. The effect of personalized and optimized transcranial direct current stimulation on consciousness in patients with severe unconsciousness (minimal unconsciousness state). https://trialsearchwhoint/Trial2aspx?TrialID=KCT0006665. 2021. PubMed PMID: CN-02351977.
280. Longo MG, Tan CO, Chan ST, Welt J, Ahmadi E, Ratai E, et al. Effect of Transcranial Low-Level Light Therapy Among Patients With Moderate Traumatic Brain Injury. Biological psychiatry. 2021;89(9):S68-S9. doi: 10.1016/j.biopsych.2021.02.186. PubMed PMID: CN-02298120.
281. Mahmud M, Prendergast M, Hadi Z, Seemungal B. Modulating balance with galvanic vestibular stimulation in traumatic brain injury patients. European journal of neurology. 2021;28(SUPPL 1):146-. doi: 10.1111/ene.14973. PubMed PMID: CN-02293585.
282. Nct. Noninvasive Modulation of Motivational Brain Regions in Healthy Volunteers. https://clinicaltrialsgov/show/NCT04972786. 2021. PubMed PMID: CN-02296894.
283. Nct. Foundations in Developing Precision Cognitive Restoration. https://clinicaltrialsgov/show/NCT05117502. 2021. PubMed PMID: CN-02352777.
284. Nct. tDCS and Cognitive Training in Active Duty Service

Members With Mild TBI: A Pilot Study. https://clinicaltrialsgov/show/NCT04925453. 2021. PubMed PMID: CN-02278434.

285. Nct. Treating Mild Traumatic Brain Injury With High Definition Transcranial Direct Current Stimulation. https://clinicaltrialsgov/show/NCT04869059. 2021. PubMed PMID: CN-02255043.

286. Nct. Effectiveness of NEUROM Combined With tDCS to Guide Motor Recovery in Paraplegics Patients. https://clinicaltrialsgov/show/NCT04790149. 2021. PubMed PMID: CN-02249762.

287. Nct. Brain State-dependent Stimulation to Improve Movement. https://clinicaltrialsgov/show/NCT05103176. 2021. PubMed PMID: CN-02341444.

288. Nct. Effects of Action Observation Training and Exercises Over 65 Years Old. https://clinicaltrialsgov/show/NCT04759690. 2021. PubMed PMID: CN-02249081.

289. Nct. Effects of tDCS With Gait Training on Leg Performance in Incomplete Spinal Cord Injury. https://clinicaltrialsgov/show/NCT04910412. 2021. PubMed PMID: CN-02278092.

290. Nct. Random Noise Stimulation to Enhance Cortical Drive &

Improve Hand Function. https://clinicaltrialsgov/show/NCT04777149. 2021. PubMed PMID: CN-02269153.

291. Nct. A Telerehabilitation Program for SCI. https://clinicaltrialsgov/show/NCT04977037. 2021. PubMed PMID: CN-02297003.

292. Nct. Transspinal Stimulation Plus Locomotor Training for SCI. https://clinicaltrialsgov/show/NCT04807764. 2021. PubMed PMID: CN-02253036.

293. Nct. Neural Basis of Sensory Learning: Brain Regions. https://clinicaltrialsgov/show/NCT05079516. 2021. PubMed PMID: CN-02340975.

294. Nct. The Study of a Neural Interface and a Neurostimulation in the Rehabilitation of Upper Limb Movement Impairments. https://clinicaltrialsgov/show/NCT05115149. 2021. PubMed PMID: CN-02352723.

295. Nct. Motor and Autonomic Concomitant Health Improvements With Neuromodulation and Exoskeleton Training: A RCT in Individuals With SCI. https://clinicaltrialsgov/show/NCT04726059. 2021. PubMed PMID: CN-02234463.

296. Nct. Efficacy of a Right-sided Ablation of the Anterior

Ganglionated Plexus for Neurally Mediated Syncope. https://clinicaltrialsgov/show/NCT04755101. 2021. PubMed PMID: CN-02235204.
297. Nct. Improving Visual Field Deficits With Noninvasive Brain Stimulation. https://clinicaltrialsgov/show/NCT05085210. 2021. PubMed PMID: CN-02341107.
298. Nct. Cerebellar tDCS for SRPCS Treatment. https://clinicaltrialsgov/show/NCT04760899. 2021. PubMed PMID: CN-02249114.
299. Nishikawa Y, Takahashi T, Kawade S, Maeda N, Maruyama H, Hyngstrom A. The effect of electrical muscle stimulation on muscle mass and balance in older adults with dementia. Brain Sciences. 2021;11(3):1-8. doi: 10.3390/brainsci11030339. PubMed PMID: CN-02264282.
300. Serradell-Ribe N, Romero Munoz JP, Viejo-Sobera R, Marron EM. Brain plasticity in frontal traumatic brain injury. Biomarker of severity and intervention efficacy. Brain Stimulation. 2021;14(6):1648-9. doi: 10.1016/j.brs.2021.10.195. PubMed PMID: CN-02370734.
301. Sippel LM, Flanagan JC, Holtzheimer PE, Moran-Santa-Maria MM, Brady KT, Joseph JE. Effects of intranasal oxytocin on threat- and reward-related functional connectivity in men and

women with and without childhood abuse-related PTSD. Psychiatry Research - Neuroimaging. 2021;317. doi: 10.1016/j.pscychresns.2021.111368. PubMed PMID: CN-02301315.

302. van 't Wout-Frank M, Philip NS. Simultaneous Application of Transcranial Direct Current Stimulation during Virtual Reality Exposure. Journal of visualized experiments : JoVE. 2021(167). doi: 10.3791/61795. PubMed PMID: CN-02271251.

303. Wessel MJ, Beanato E, Popa T, Windel F, Menoud P, Beliaeva V, et al. Evidence for temporal interference (TI) stimulation effects on motor striatum. Brain Stimulation. 2021;14(6):1684-. doi: 10.1016/j.brs.2021.10.307. PubMed PMID: CN-02370782.

304. Yeh N, Yang Y, Huang S, Ku P, Wang R. Effects of transcranial direct current stimulation followed by exercise on neuropathic pain in chronic spinal cord injury: a double-blinded randomized controlled pilot trial. Spinal cord. 2021;59(6):684-92. doi: 10.1038/s41393-020-00560-x. PubMed PMID: CN-02308880.

305. Nct. Control Network Neuromodulation to Enhance Cognitive Training in Complex Traumatic Brain Injury. https://clinicaltrialsgov/show/NCT05236010. 2022. PubMed PMID: CN-02367654.

306. Nct. Combined Transcranial Magnetic Stimulation and

Therapy for mTBI Related Headaches. https://clinicaltrialsgov/show/NCT05176392. 2022. PubMed PMID: CN-02355597.

307. Nct. Probing the Dorsolateral Prefrontal Cortex and Central Executive Network for Improving Neuromodulation in Depression. https://clinicaltrialsgov/show/NCT05224063. 2022. PubMed PMID: CN-02367408.

308. Nct. Effects of Individualized rTMS in DOC Patients. https://clinicaltrialsgov/show/NCT05187000. 2022. PubMed PMID: CN-02366658.

309. Nct. Long-term Effects of Repetitive TMS in Chronic Neuropathic Pain in People With SCI. https://clinicaltrialsgov/show/NCT05226676. 2022. PubMed PMID: CN-02367457.

310. Nct. Combining Cognitive Training With tDCS to Enhance Memory Inhibition in Young Binge Drinkers. https://clinicaltrialsgov/show/NCT05237414. 2022. PubMed PMID: CN-02367685.

2. **Emerged studies after duplication (3878 studies)**

1. Sugita M, Sugita K, Furukawa T, Abe H. Studies on the Transport Mechanism of Aminoacids in the Renal Tubules: Part I Studies on the Mechanism of Aminoaciduria from the Analytical Standpoint of Titration Curve. JAPANESE CIRCULATION JOURNAL. 1967;31(3):405-14. doi: 10.1253/jcj.31.405.

2. French AP, Schmid AC, Ingalls E. Single case study: Transcendental meditation, altered reality testing, and behavioral change: A case report. Journal of Nervous and Mental Disease. 1975;161(1):55-8. doi: 10.1097/00005053-197507000-00007.

3. Singh AN, Saxena B, Gent M, Nelson HL. Maprotiline (Ludiomil, Ciba 34,276 BA) and imipramine in depressed outpatients: a double blind clinical study. Current Therapeutic Research - Clinical and Experimental. 1976;19(4):451-62.

4. Ide K, Harano K, Totoki T. Clinico-experimental studies on total spinal block with special reference to EEG findings. Japanese Journal of Anesthesiology. 1977;26(9):1029-40.

5. Hume AL, Cant BR, Shaw NA. Central somatosensory conduction time in comatose patients. Ann Neurol. 1979;5(4):379-84. Epub 1979/04/01. doi: 10.1002/ana.410050412. PubMed PMID: 443771.

6. Obeso JA, Teijeira JM, Marti-Masso JF, Iragui MI, Maravi E,

Carrera N, et al. Somatosensory evoked potentials: abnormalities in brainstem lesions. Electromyography and clinical neurophysiology. 1979;19(5):417-23. PubMed PMID: MEDLINE:527543.

7. Anziska BJ, Cracco RQ. Short latency somatosensory evoked potentials in brain dead patients. Arch Neurol. 1980;37(4):222-5. Epub 1980/04/01. doi: 10.1001/archneur.1980.00500530060009. PubMed PMID: 7362487.

8. Borucki Z. [Results of rheoencephalographic studies in chronic subdural hematoma]. Neurol Neurochir Pol. 1980;14(6):633-7. Epub 1980/01/01. PubMed PMID: 7242783.

9. Anziska BJ, Cracco RQ. Short latency SEPs to median nerve stimulation: comparison of recording methods and origin of components. Electroencephalogr Clin Neurophysiol. 1981;52(6):531-9. Epub 1981/12/01. doi: 10.1016/0013-4694(81)91428-0. PubMed PMID: 6172253.

10. Mauguière F, Courjon J. The origins of short-latency somatosensory evoked potentials in humans. Annals of Neurology. 1981;9(6):607-11. doi: 10.1002/ana.410090616.

11. Yoshida Y, Kouka M, Otose H, Hashiguchi K, Kamitsuchihasi H, Igata A. [A Lance-Adams syndrome of a diver (author's transl)]. No To Shinkei. 1981;33(10):1051-6. Epub 1981/10/01. PubMed PMID: 6797451.

12. Bollini C, De Maria R, Trento L. Posttraumatic epilepsy: Notes on 2 groups of patients studied before and after the introduction of computer tomography. Bollettino - Lega Italiana contro l'Epilessia. 1982;No. 39:76-7.

13. D'Alessandro R, Tinuper P, Ferrara R. Comparison between clinicaland computer tomographic findings in calculation of risk of posttraumatic epilepsy. Bollettino - Lega Italiana contro l'Epilessia. 1982;No. 39:69-70.

14. Gambi D, Colangelo U, Di Paolo A. Posttraumatic epilepsy: Correlations between EEG and computer tomography in cases of blunt trauma. Bollettino - Lega Italiana contro l'Epilessia. 1982;No. 39:68.

15. Moilanen A, Pitkanen M. Panoramic zonography in the radiographic diagnosis of facial and related structures. A clinical study. Rontgen-Blatter. 1984;37(2):95-9.

16. Adsett DB, Fitz CR, Hill A. HYPOXIC-ISCHAEMIC CEREBRAL INJURY IN THE TERM NEWBORN: CORRÉLATION OF CT FINDINGS WITH NEUROLOGICAL OUTCOME. Developmental Medicine & Child Neurology. 1985;27(2):155-60. doi: 10.1111/j.1469-8749.1985.tb03764.x.

17. Boureux C. Echocardiography in investigation of syncopes. Lyon Mediterranee Medical. 1985;21(14):10117-20.

18. Drechsler F. Short latency sep to median nerve stimulation:

Recording methods, origin of components and clinical application. Electromyography and Clinical Neurophysiology. 1985;25(2-3):115-34.

19. Drexler HG, Gignac SM, Minowada J. Subsets of normal mononuclear cells in the peripheral blocd defined by monoclonal antibodies. Immunological Investigations. 1985;14(4):315-21. doi: 10.3109/08820138509022666.

20. Frank Y, Zimmerman R, Leeds NMD. NEUROLOGICAL MANIFESTATIONS IN ABUSED CHILDREN WHO HAVE BÉEN SHAKEN. Developmental Medicine & Child Neurology. 1985;27(3):312-6. doi: 10.1111/j.1469-8749.1985.tb04541.x.

21. Noetzel MJ. Theophylline Neurotoxicity Resulting in Significant Unilateral Brain-damage. Developmental Medicine & Child Neurology. 1985;27(2):242-5. doi: 10.1111/j.1469-8749.1985.tb03775.x.

22. Scandiffio T, Contuzzi V, Tesler U, Tantalo L. Pulsed Doppler transmitral flow in a case of myxoma of the left atrium. Giornale italiano di cardiologia. 1985;15(9):921-4.

23. Torres B, Gómez C, Delgado-García JM. [Effects of axotomy on the activity of the motor neurons of the external ocular motor nucleus during saccadic movements]. Rev Esp Fisiol. 1985;41(1):73-81. Epub 1985/03/01. PubMed PMID: 4001546.

24. Amici A, Castellucci G, Bragetti P, Poletti C, Santucci A, Troianello G, et al. Cranial injuries in childhood. Experience in 500 cases. Pediatria Medica e Chirurgica. 1986;8(4):555-8.
25. Berić A, Dimitrijević MR, Prevec TS, Sherwood AM. Epidurally recorded cervical somatosensory evoked potential in humans. Electroencephalogr Clin Neurophysiol. 1986;65(2):94-101. Epub 1986/03/01. doi: 10.1016/0168-5597(86)90041-9. PubMed PMID: 2419108.
26. Kirkpatrick JS, Gower DJ, Chauvenet A, Kelly DL. Subgaleal Hematoma in a Child, without Skull Fracture. Developmental Medicine & Child Neurology. 1986;28(4):511-4. doi: 10.1111/j.1469-8749.1986.tb14291.x.
27. Matsuo Y, Sagawa K, Morita M, Minowada J, Yokoyama MM. Establishment of a hairy cell leukemia cell line carrying Tac antigen and phagocytic activity with B-cell characteristics. Journal of the National Cancer Institute. 1986;76(2):207-16.
28. Shaw NA. The origin of the N20 component of the somatosensory evoked potential in man. Aust J Exp Biol Med Sci. 1986;64 (Pt 5):471-5. Epub 1986/10/01. doi: 10.1038/icb.1986.50. PubMed PMID: 3579741.
29. Takata M, Nagahama T. Cortically and lingually induced postsynaptic potentials in trigeminal motoneurons after axotomy. Exp

Brain Res. 1986;61(2):272-9. Epub 1986/01/01. doi: 10.1007/bf00239517. PubMed PMID: 3005014.
30. Tanaka H, Shimoda T, Uchida K, Suzuki T, Ishikawa E. IMMUNOHISTOCHEMICAL STUDY ON THE DISTRIBUTION AND SIGNIFICANCE OF MONONUCLEAR CELLS IN HUMAN BREAST CARCINOMA. Pathology International. 1986;36(10):1455-68. doi: 10.1111/j.1440-1827.1986.tb02817.x.
31. Theodore WH, Holmes MD, Dorwart RH, Porter RJ, Chiro GD, Sato S, et al. Complex Partial Seizures: Cerebral Structure and Cerebral Function. Epilepsia. 1986;27(5):576-82. doi: 10.1111/j.1528-1157.1986.tb03587.x.
32. Thomas JG. Computer generation of templates for evaluating the amplitude and latency parameters of two superimposed constituent components of early somatosensory evoked responses. J Neurosci Methods. 1986;15(4):329-34. Epub 1986/02/01. doi: 10.1016/0165-0270(86)90146-9. PubMed PMID: 3515056.
33. Borromei A, Caramelli R, Cipriani G, Giancola LC, Guerra L, Lozito A. Neurotraumatology and post-traumatic epilepsy: Prophylaxis, treatment and long-term follow-up. Minerva Medica. 1987;78(22):1687-705.
34. Ganji S, Peters G, Frazier E. Alpha-coma: clinical and evoked potential studies. Clin Electroencephalogr. 1987;18(3):103-13. Epub

1987/07/01. PubMed PMID: 3652461.

35. Jabbari B, Vance SC, Harper MG, Salazar AM, Smutok MA, Amin D. Clinical and radiological correlates of somatosensory evoked potentials in the late phase of head injury: a study of 500 Vietnam veterans. Electroencephalogr Clin Neurophysiol. 1987;67(4):289-97. Epub 1987/10/01. doi: 10.1016/0013-4694(87)90115-5. PubMed PMID: 2441962.

36. Martins IP, Ferro JM. Acquired Conduction Aphasia in a Child. Developmental Medicine & Child Neurology. 1987;29(4):532-6. doi: 10.1111/j.1469-8749.1987.tb02514.x.

37. Momma F, Sabin HI, Symon L, Branston NM. Clinical evidence supporting a subthalamic origin of the P15 wave of the somatosensory evoked potentials to median nerve stimulation. Electroencephalography and Clinical Neurophysiology. 1987;67(2):134-9. doi: 10.1016/0013-4694(87)90035-6.

38. Sonnet ML, Floret D, Guillaume C, Fournet A, Motin J. [Evoked potentials in comatose children]. Pediatrie. 1987;42(1):45-50. Epub 1987/01/01. PubMed PMID: 3588178.

39. Stain C, Stockinger H, Scharf M, Jager U, Gossinger H, Lechner K, et al. Human blood basophils display a unique phenotype including activation linked membrane structures. Blood. 1987;70(6):1872-9. doi: 10.1182/blood.v70.6.1872.bloodjournal7061872.

40. Stöhr M, Riffel B, Trost E, Ullrich A. Short-latency somatosensory evoked potentials in brain death. J Neurol. 1987;234(4):211-4. Epub 1987/05/01. doi: 10.1007/bf00618252. PubMed PMID: 3612191.

41. Stöhr M, Riffel B, Trost E, Wengert P. [Significance of somatosensory evoked potentials in determining brain death]. Anasth Intensivther Notfallmed. 1987;22(1):21-5. Epub 1987/02/01. PubMed PMID: 3578718.

42. Takahashi H, Yasue M, Ishijima B. [Changes in short latency SEPs (S-SEPs) to median nerve stimulation in brain dead patients]. No To Shinkei. 1987;39(6):543-9. Epub 1987/06/01. PubMed PMID: 3620222.

43. Bensaid J, Gueret P, Blanc P, Virot P, Doumeix JJ. Barlow's disease and risk for life. Archives des Maladies du Coeur et des Vaisseaux. 1988;81(2):213-8.

44. Buchner H, Ferbert A, Hacke W. Serial recording of median nerve stimulated subcortical somatosensory evoked potentials (SEPs) in developing brain death. Electroencephalogr Clin Neurophysiol. 1988;69(1):14-23. Epub 1988/01/01. doi: 10.1016/0013-4694(88)90031-4. PubMed PMID: 2448117.

45. Hayashi M, Saisho S, Suzuki H, Shimozawa K, Iwakawa Y. Sleep disturbances in children with congenital and acquired hypothyroidism.

No To Hattatsu. 1988;20(4):294-300.

46. Kedar E, Or R, Naparstek E, Zeira E, Slavin S. Preliminary characterization of functional residual host-type I lymphocytes following conditioning for allogeneic HLA-matched bone marrow transplantation (BMT). Bone Marrow Transplantation. 1988;3(2):129-40.

47. Moryś J, Słoniewski P, Narkiewicz O. Somatosensory evoked potentials following lesions of the claustrum. Acta Physiol Pol. 1988;39(5-6):475-83. Epub 1988/09/01. PubMed PMID: 3257060.

48. Nakamura Y, Toya S, Nakatsukasa M, Ibata Y, Tamura K, Takase M. [A case of alpha coma in acute brainstem dysfunction--consecutive electroencephalograms and evoked potentials]. No To Shinkei. 1988;40(8):753-8. Epub 1988/08/01. PubMed PMID: 3179089.

49. Rumpl E, Prugger M, Battista HJ, Badry F, Gerstenbrand F, Dienstl F. Short latency somatosensory evoked potentials and brain-stem auditory evoked potentials in coma due to CNS depressant drug poisoning. Preliminary observations. Electroencephalogr Clin Neurophysiol. 1988;70(6):482-9. Epub 1988/12/01. doi: 10.1016/0013-4694(88)90146-0. PubMed PMID: 2461282.

50. Rumpl E, Prugger M, Gerstenbrand F, Brunhuber W, Badry F, Hackl JM. Central somatosensory conduction time and acoustic brainstem transmission time in post-traumatic coma. J Clin

Neurophysiol. 1988;5(3):237-60. Epub 1988/07/01. doi: 10.1097/00004691-198807000-00002. PubMed PMID: 2844849.
51. Schriefer TN, Mills KR, Murray NM, Hess CW. Evaluation of proximal facial nerve conduction by transcranial magnetic stimulation. J Neurol Neurosurg Psychiatry. 1988;51(1):60-6. Epub 1988/01/01. doi: 10.1136/jnnp.51.1.60. PubMed PMID: 3351531; PubMed Central PMCID: PMCPMC1032714.
52. Urasaki E, Fukumura A, Itho Y, Itoyama Y, Yamada M, Ushio Y, et al. [Lazarus' sign and respiratory-like movement in a patient with brain death]. No To Shinkei. 1988;40(12):1111-6. Epub 1988/12/01. PubMed PMID: 3248188.
53. MacKay-Lyons M. Low-load, prolonged stretch in treatment of elbow flexion contractures secondary to head trauma: A case report. Physical Therapy. 1989;69(4):292-6. doi: 10.1093/ptj/69.4.292.
54. Pierluisi GJ, Terndrup TE. Influence of topical anesthesia on the sedation of pediatric emergency department patients with lacerations. Pediatric Emergency Care. 1989;5(4):211-5. doi: 10.1097/00006565-198912000-00002.
55. Reilly RP, Findley TW. Research in physical medicine and rehabilitation. IV. Some practical designs in applied research. Am J Phys Med Rehabil. 1989;68(4):196-201. Epub 1989/08/01. doi: 10.1097/00002060-198908000-00009. PubMed PMID: 2788436.

56. Riffel B, Stöhr M, Graser W, Trost E, Baumgärtner H. [Early prognosis in severe cranio-cerebral trauma using the Glasgow Coma Score and evoked potentials]. Anaesthesist. 1989;38(2):51-8. Epub 1989/02/01. PubMed PMID: 2929965.

57. Riffel B, Stohr M, Graser W, Trost E, Baumgartner H. Early outcome prediction in patients with severe head injury by Glasgow Coma Score and evoked potentials. Anaesthesist. 1989;38(2):51-8.

58. Unger WG. Mediation of the ocular response to injury and irritation: peptides versus prostaglandins. Prog Clin Biol Res. 1989;312:293-328. Epub 1989/01/01. PubMed PMID: 2678145.

59. Zeitlhofer J, Steiner M, Zadrobilek E, Hausl E, Sporn P, Asenbaum S, et al. Evoked potentials in the follow-up and prognosis of patients with craniocerebral trauma. Der Anaesthesist. 1989;38(1):10-5. PubMed PMID: MEDLINE:2919747.

60. Zeitlhofer J, Steiner M, Zadrobilek E, Hausl E, Sporn P, Asenbaum S, et al. Evoked potentials in the course and prognosis of patients after severe head trauma. Anaesthesist. 1989;38(1):10-5.

61. Facco E, Casartelli Liviero M, Munari M, Toffoletto F, Baratto F, Giron GP. Short latency evoked potentials: new criteria for brain death? J Neurol Neurosurg Psychiatry. 1990;53(4):351-3. Epub 1990/04/01. doi: 10.1136/jnnp.53.4.351. PubMed PMID: 2341851; PubMed Central PMCID: PMCPMC1014178.

62. Gacek RR. Arachnoid granulation cerebrospinal fluid otorrhea. Ann Otol Rhinol Laryngol. 1990;99(11):854-62. Epub 1990/11/01. doi: 10.1177/000348949009901102. PubMed PMID: 2241009.
63. Greene SA, Jefferson IG, Baum JD. Cerebral Oedema Complicating Diabetic Ketoacidosis. Developmental Medicine & Child Neurology. 1990;32(7):633-8. doi: 10.1111/j.1469-8749.1990.tb08548.x.
64. Heydenreich A. [Hypothetical aspects of the preventive effectiveness of controlled reflex and stimulation therapy (exemplified by acupuncture and PuTENS) in migraine]. Psychiatr Neurol Med Psychol (Leipz). 1990;42(8):500-7. Epub 1990/08/01. PubMed PMID: 2247531.
65. Maccabee PJ, Eberle L, Amassian VE, Cracco RQ, Rudell A, Jayachandra M. Spatial distribution of the electric field induced in volume by round and figure '8' magnetic coils: relevance to activation of sensory nerve fibers. Electroencephalography and clinical neurophysiology. 1990;76(2):131-41. doi: 10.1016/0013-4694(90)90211-2. PubMed PMID: MEDLINE:1697241.
66. Yamaguchi S, Knight RT. Gating of somatosensory input by human prefrontal cortex. Brain Research. 1990;521(1-2):281-8. doi: 10.1016/0006-8993(90)91553-S.

67. Zeitlhofer J, Mayr N, Auff E. [The value of evoked potentials in brain death diagnosis]. Wien Med Wochenschr. 1990;140(23-24):564-7. Epub 1990/01/01. PubMed PMID: 2085011.

68. Facco E, Munari M, Donà B, Baratto F, Fiore D, Behr AU, et al. Spatial mapping of SEP in comatose patients: improved outcome prediction by combined parietal N20 and frontal N30 analysis. Brain Topogr. 1991;3(4):447-55. Epub 1991/01/01. doi: 10.1007/bf01129004. PubMed PMID: 1742161.

69. Grimm G, Madl C, Oder W, Druml W, Schneeweiss B, Laggner AN, et al. Evoked potentials in severe herpes simplex encephalitis. Intensive Care Med. 1991;17(2):94-7. Epub 1991/01/01. doi: 10.1007/bf01691430. PubMed PMID: 1865043.

70. Jouvet M, Albarede JL, Lubin S, Meyrignac C. [Noradrenaline and cerebral aging]. Encephale. 1991;17(3):187-95. Epub 1991/05/01. PubMed PMID: 1864252.

71. Jouvet M, Albarede JL, Lubin S, Meyrignac C. Norepinephrine and cerebral aging. Encephale. 1991;17(3):187-95.

72. Liska A, Kazibutowska Z, Rogoz B, Pierzchała K, Rusiecki Z, Totuszyński M. [Multimodal evoked potentials in the early period after cranio-cerebral trauma]. Neurol Neurochir Pol. 1991;25(5):647-55. Epub 1991/09/01. PubMed PMID: 1808527.

73. Thompson NM, Ewing-Cobbs L, Fletcher JM, Miner ME, Levin

HS. Left Unilateral Neglect in a Preschool Child. Developmental Medicine & Child Neurology. 1991;33(7):640-4. doi: 10.1111/j.1469-8749.1991.tb14935.x.

74. Wagner W. SEP testing in deeply comatose and brain dead patients: the role of nasopharyngeal, scalp and earlobe derivations in recording the P14 potential. Electroencephalography and clinical neurophysiology. 1991;80(5):352-63. doi: 10.1016/0168-5597(91)90082-9. PubMed PMID: MEDLINE:1716559.

75. Werba A, Weinstabl C, Petricek W, Plainer B, Spiss CK. [Requisite muscle relaxation using vecuronium for tracheobronchial suction in neurosurgical intensive care patients]. Anaesthesist. 1991;40(6):328-31. Epub 1991/06/01. PubMed PMID: 1679303.

76. Werba A, Weinstabl C, Petricek W, Plainer B, Spiss CK. Vecuronium prevents increases in intracranial pressure during routine tracheobronchial suctioning in neurosurgical patients. Anaesthesist. 1991;40(6):328-31.

77. West JNW, Townend JN, Davies P, Sheridan JJ, Stallard TJ, Davies MK, et al. Effect of unrestricted activity on accuracy of ambulatory blood pressure measurement. Hypertension. 1991;18(5):593-7. doi: 10.1161/01.HYP.18.5.593.

78. Winter A. Coma and spinal cord dysfunction: Treatment with

naloxone, hyperbaric oxygen, transcutaneous nerve stimulation, and electrophrenic pacemaker. Journal of Neurological and Orthopaedic Medicine and Surgery. 1991;12(2):124-8.

79. Zhou D. [Comparison of the effects of 4 psychiatric treatments on rat brain beta 5HT2 receptors]. Zhonghua Shen Jing Jing Shen Ke Za Zhi. 1991;24(4):207-9, 51-2. Epub 1991/08/01. PubMed PMID: 1659512.

80. Beretta L, Citerio G, Dell'Acqua A, Napolitano L, Piccoli S, Torri G. Diffuse axonal injury (DAI): diagnostic assessment with nuclear magnetic resonance (NMR). Minerva Anestesiologica. 1992;58(10):795-6.

81. Cameron PD, Wallace SJ, Munro J. HERPES SIMPLEX VIRUS ENCEPHALITIS: PROBLEMS IN DIAGNOSIS. Developmental Medicine & Child Neurology. 1992;34(2):134-40. doi: 10.1111/j.1469-8749.1992.tb14979.x.

82. Cusumano S, Paolin A, Di Paola F, Boccaletto F, Simini G, Palermo F, et al. Assessing brain function in post-traumatic coma by means of bit-mapped SEPs, BAEPs, CT, SPET and clinical scores. Prognostic implications. Electroencephalogr Clin Neurophysiol. 1992;84(6):499-514. Epub 1992/11/01. doi: 10.1016/0168-5597(92)90039-e. PubMed PMID: 1280196.

83. Franssen H, Stegeman DF, Wieneke GH. Attenuation of dipoles

modelled from SEP due to a lacunar infarct or altered stimulus rate. Brain Topography. 1992;5(2):159-63. doi: 10.1007/BF01129044.
84. Gilmore R. Somatosensory evoked potential testing in infants and children. Journal of clinical neurophysiology : official publication of the American Electroencephalographic Society. 1992;9(3):324-41. doi: 10.1097/00004691-199207010-00002. PubMed PMID: MEDLINE:1517402.
85. Jaspert A, Kotterba S, Tegenthoff M, Malin JP. [Transcranial magnetic stimulation in the cranial nerve region in cranio-cerebral injuries]. EEG EMG Z Elektroenzephalogr Elektromyogr Verwandte Geb. 1992;23(1):37-42. Epub 1992/03/01. PubMed PMID: 1587232.
86. Jaspert A, Kotterba S, Tegenthoff M, Malin JP. Transcranial magnetic stimulation of the facial nerve in patients with traumatic brain lesions. EEG-EMG Zeitschrift fur Elektroenzephalographie Elektromyographie und Verwandte Gebiete. 1992;23(1):37-42.
87. Kutalek SP, Schuster MM, Hessen SE, Sheppard R, Maquilan M, Nydegger C. Clinical Experience with a New Multiprogrammable Dual Chamber Pacemaker. Pacing and Clinical Electrophysiology. 1992;15(11):1830-5. doi: 10.1111/j.1540-8159.1992.tb02977.x.
88. Liepert J, Tegenthoff M. [Transcranial magnetic stimulation of patients with a single epileptic seizure]. Nervenarzt. 1992;63(8):492-4. Epub 1992/08/01. PubMed PMID: 1522935.

89. Liepert J, Tegenthoff M. Transcranial magnetic stimulation in patients with single epileptic seizures. Nervenarzt. 1992;63(8):492-4.
90. Netz J, Hömberg V. Intact conduction of fastest corticospinal efferents is not sufficient for normal voluntary muscle strength: transcranial motor cortex stimulation in patients with tetraplegia. Neurosci Lett. 1992;146(1):29-32. Epub 1992/10/26. doi: 10.1016/0304-3940(92)90164-3. PubMed PMID: 1475046.
91. Osenbach RK. Neurosurgical applications of somatosensory and motor evoked potentials. Neurosurgery Quarterly. 1992;2(4):280-95.
92. Shutov AA, Sherman MA. [A psychoautonomic syndrome in the residual period of a modern "mild" military craniocerebral trauma]. Zh Nevropatol Psikhiatr Im S S Korsakova. 1992;92(5-12):13-5. Epub 1992/01/01. PubMed PMID: 1338144.
93. Shutov AA, Sherman MA. Psychovegetative syndrome in the residual period of modern 'light' military craniocerebral injury. Zhurnal Nevropatologii i Psikhiatrii Imeni SSKorsakova. 1992;92(5-12):13-5.
94. Ten Harkel ADJ, Baisch F, Karemaker JM. Increased orthostatic blood pressure variability after prolonged head-down tilt. Acta Physiologica Scandinavica, Supplement. 1992;144(604):89-99.
95. Urasaki E, Tokimura T, Kumai J, Wada S, Yokota A. Preserved spinal dorsal horn potentials in a brain-dead patient with Lazarus' sign.

Case report. J Neurosurg. 1992;76(4):710-3. Epub 1992/04/01. doi: 10.3171/jns.1992.76.4.0710. PubMed PMID: 1545268.
96. Van Lieshout EJ, Van Lieshout JJ, Krol J, Simons M, Karemaker JM. Assessment of cardiovascular reflexes is of limited value in predicting maximal + Gz-tolerance. Aviation Space and Environmental Medicine. 1992;63(1):21-6.
97. Ying Z, Schmid UD, Schmid J, Hess CW. Motor and somatosensory evoked potentials in coma: analysis and relation to clinical status and outcome. J Neurol Neurosurg Psychiatry. 1992;55(6):470-4. Epub 1992/06/01. doi: 10.1136/jnnp.55.6.470. PubMed PMID: 1619414; PubMed Central PMCID: PMCPMC1014903.
98. Bach-y-Rita P. Nonsynaptic diffusion neurotransmission (NDN) in the brain. Neurochem Int. 1993;23(4):297-318. Epub 1993/10/01. doi: 10.1016/0197-0186(93)90074-f. PubMed PMID: 8220172.
99. Bastos PG, Sun X, Wagner DP, Wu AW, Knaus WA. Glasgow Coma Scale score in the evaluation of outcome in the intensive care unit: Findings from the Acute Physiology and Chronic Health Evaluation III study. Critical Care Medicine. 1993;21(10):1459-65. doi: 10.1097/00003246-199310000-00012.
100. Devinsky O. Electrical and magnetic stimulation of the central nervous system. Historical overview. Adv Neurol. 1993;63:1-16. Epub

1993/01/01. PubMed PMID: 8279294.

101. Ghiazza G, Piovano P, Ferraro G, D'Arco M, Pellegrini V. A case of hemiballismus developed after a hyperosmolar coma in an elderly diabetic woman. European Review for Medical and Pharmacological Sciences. 1993;15(2):83-6.

102. Gray PH, Tudehope DI, Masel JP, Burns YR, Mohay HA, O'Callaghan MJ, et al. PERINATAL HYPOXIC-ISCHAEMIC BRAIN INJURY: PRDICTION OF OUTCOME. Developmental Medicine & Child Neurology. 1993;35(11):965-73. doi: 10.1111/j.1469-8749.1993.tb11578.x.

103. Guerit JM, Baele P, de Tourtchaninoff M, Soveges L, Dion R. Somatosensory evoked potentials in patients undergoing circulatory arrest under profound hypothermia. Neurophysiologie clinique = Clinical neurophysiology. 1993;23(2-3):193-208. doi: 10.1016/s0987-7053(05)80230-8. PubMed PMID: MEDLINE:8326930.

104. Kapuściński A. Effect of clinical death on inositol 1,4,5-trisphosphate in the rat brain. Neuropatol Pol. 1993;31(1-2):91-6. Epub 1993/01/01. PubMed PMID: 8208444.

105. Kiers L, Cros D, Chiappa KH, Fang J. Variability of motor potentials evoked by transcranial magnetic stimulation. Electroencephalogr Clin Neurophysiol. 1993;89(6):415-23. Epub

1993/12/01. doi: 10.1016/0168-5597(93)90115-6. PubMed PMID: 7507428.

106. Kotterba S, Tegenthoff M, Malin JP. [Transcranial magnetic stimulation in perioperative damage to the facial nerve]. Laryngorhinootologie. 1993;72(1):32-5. Epub 1993/01/01. doi: 10.1055/s-2007-997849. PubMed PMID: 8439354.

107. Krägeloh-Mann I, Grodd W, Schöning M, Marquard K, Nägele T, Ruitenbeek W. PROTON SPECTROSCOPY IN FIVE PATIENTS WITH LEIGH'S DISEASE AND MITOCHONDRIAL ENZYME DEFICIENCY. Developmental Medicine & Child Neurology. 1993;35(9):769-76. doi: 10.1111/j.1469-8749.1993.tb11728.x.

108. Masur H, Papke K, Oberwittler C. Suppression of visual perception by transcranial magnetic stimulation - experimental findings in healthy subjects and patients with optic neuritis. Electroencephalography and Clinical Neurophysiology. 1993;86(4):259-67. doi: 10.1016/0013-4694(93)90107-7.

109. Rappaport M, Leonard J, Ruiz Portillo S. Somatosensory evoked potential peak latencies and amplitudes in contralateral and ipsilateral hemispheres in normal and severely traumatized brain-injured subjects. Brain injury. 1993;7(1):3-13. doi: 10.3109/02699059309008152. PubMed PMID: MEDLINE:8381044.

110.Takeshita M, Masuda Y, Sumiyoshi Y, Ohshima K, Kikuchi M, Kimura N, et al. Clinicopathologic, enzyme and hisotchemical studies of centrocytic (mantle cell) lymphoma: Comparison with other types of low-grade B cell lymphoma bases on the updated Kiel classification. Pathology International. 1993;43(5):244-52. doi: 10.1111/j.1440-1827.1993.tb01139.x.

111.Veccia S, Giacometti A, Cinti B, Fiorentini A, Petroni S, Scalise G. Adenosine deaminase activity in cerebrospinal fluid from AIDS patients with cerebral lesions. Giornale di Malattie Infettive e Parassitarie. 1993;45(7-8):804-5.

112.Boehme MWJ, Werle E, Kommerell B, Raeth U. Serum levels of adhesion molecules and thrombomodulin as indicators of vascular injury in severe Plasmodium falciparum malaria. The Clinical Investigator. 1994;72(8):598-603. doi: 10.1007/BF00227452.

113.Canivet JL, Gustad K, Leclercq P, Damas P, Lamy M. Massive ketonuria during sedation with propofol in a 12 year old girl with severe head trauma. Acta Anaesthesiologica Belgica. 1994;45(1):19-22.

114.Champagnat J, Richter DW. The roles of K+ conductance in expiratory pattern generation in anaesthetized cats. J Physiol. 1994;479 (Pt 1)(Pt 1):127-38. Epub 1994/08/15. doi: 10.1113/jphysiol.1994.sp020282. PubMed PMID: 7990029; PubMed

Central PMCID: PMCPMC1155730.
115.Dichter MA. Emerging Insights Into Mechanisms of Epilepsy: Implications for New Antiepileptic Drug Development. Epilepsia. 1994;35:S51-S7. doi: 10.1111/j.1528-1157.1994.tb05956.x.
116.Goddard MJ, Dean BZ, King JC. 1. Basic science, acute pain, and neuropathic pain. Archives of Physical Medicine and Rehabilitation. 1994;75(5 SUPPL.):S4-S8. doi: 10.1016/0003-9993(94)90371-9.
117.Kong WJ, Egg G, Hussl B, Seyr M, Schrott-Fischer A. A study of neurotransmitters in human inner ear. Preservation of human temporal bone and value of organ donation for inner ear research. Acta Otolaryngol. 1994;114(3):245-53. Epub 1994/05/01. doi: 10.3109/00016489409126051. PubMed PMID: 7915484.
118.Mazziotta JC. Mapping human brain activity in vivo. West J Med. 1994;161(3):273-8. Epub 1994/09/01. PubMed PMID: 7975566; PubMed Central PMCID: PMCPMC1011409.
119.Muizelaar JP. Clinical trials with dismutec(TM) (pegorgotein; polyethylene glycol- conjugated superoxide dismutase; PEG-SOD) in the treatment of severe closed head injury. Advances in Experimental Medicine and Biology1994. p. 389-400.
120. Probert TCS, Wiesenfeld D, Reade PC. Temporomandibular pain dysfunction disorder resulting from road traffic accidents - An Australian study. International Journal of Oral and Maxillofacial

Surgery. 1994;23(6 PART 1):338-41. doi: 10.1016/S0901-5027(05)80049-9.

121. von Giesen HJ, Roick H, Benecke R. Inhibitory actions of motor cortex following unilateral brain lesions as studied by magnetic brain stimulation. Experimental Brain Research. 1994;99(1):84-96. doi: 10.1007/BF00241414.

122. Buchner H, Waberski TD, Fuchs M, Wischmann HA, Beckmann R, Rienäcker A. Origin of P16 median nerve SEP component identified by dipole source analysis - subthalamic or within the thalamo-cortical radiation? Experimental Brain Research. 1995;104(3):511-8. doi: 10.1007/BF00231985.

123. Eken P, de Vries LS, van der Graaf Y, Meiners LC, van Nieuwenhuizen O. HAEMORRHAGIC-ISCHAEMIC LESIONS OF THE NEONATAL BRAIN: CORRELATION BETWEEN CEREBRAL VISUAL IMPAIRMENT, NEURODEVELOP-MENTAL OUTCOME AND MRI IN INFANCY. Developmental Medicine & Child Neurology. 1995;37(1):41-55. doi: 10.1111/j.1469-8749.1995.tb11931.x.

124. Ernst AA, Marvez-Valls E, Nick TG, Weiss SJ. LAT (lidocaine-adrenaline-tetracaine) versus TAC (tetracaine-adrenaline-cocaine) for topical anesthesia in face and scalp lacerations. American Journal of Emergency Medicine.

1995;13(2):151-4. doi: 10.1016/0735-6757(95)90082-9.

125. Gambaretti R, Orlandi S, Pozzoni R. Diagnosis of meniscal and ligament lesions of the knee. Comparison between clinical and instrumental examination. Minerva Ortopedica e Traumatologica. 1995;46(3):105-7.

126. Gessner JE, Radeke HH, Uciechowski P, Resch K, Schmidt RE. Activation-dependent expression of Fc receptors for IgG on glomerular mesangial cells. Immunitat und Infektion. 1995;23(2):67-9.

127. Kaneko M. [Prognostic evaluation of patients with severe head injury by motor evoked potentials induced by transcranial magnetic stimulation--combined analysis with brainstem auditory evoked potentials]. No To Shinkei. 1995;47(5):491-6. Epub 1995/05/01. PubMed PMID: 7786626.

128. Kaneko M. Prognostic evaluation of patients with severe head injury by motor evoked potentials induced by transcranial magnetic stimulation - Combined analysis with brainstem auditory evoked potentials. Brain and Nerve. 1995;47(5):491-6.

129. Kapelushnik J, Or R, Filon D, Nagler A, Cividalli G, Aker M, et al. Analysis of β-globin mutations shows stable mixed chimerism in patients with thalassemia after bone marrow transplantation. Blood. 1995;86(8):3241-6. doi:

10.1182/blood.v86.8.3241.bloodjournal8683241.

130. Karnath HO. Transcutaneous electrical stimulation and vibration of neck muscles in neglect. Experimental Brain Research. 1995;105(2):321-4. doi: 10.1007/BF00240969.

131. Mesejo A, Núñez C, Simó M, Blanquer J, Pérez PL, Ruiz F, et al. [Neuroleptic malignant syndrome, multiorgan failure and peripheral polyneuropathy]. Rev Neurol. 1995;23(119):136-8. Epub 1995/01/01. PubMed PMID: 8548608.

132. Morioka T, Yamamoto T, Mizushima A, Tombimatsu S, Shigeto H, Hasuo K, et al. Comparison of magnetoencephalography, functional MRI, and motor evoked potentials in the localization of the sensory-motor cortex. Neurological Research. 1995;17(5):361-7. doi: 10.1080/01616412.1995.11740343.

133. Naka D, Funahashi K, Ogura M, Kuwata T, Nakai M, Nakai K, et al. Identification of motor area by transcranial magnetic stimulation using an 'eight-figure' coil in patients with motor paralysis. Neurological Surgery. 1995;23(1):27-34.

134. Naruse S, Takaya K, Yoshioka H. Metabolic and Functional Magnetic Resonance Imaging of the Brain : Clinical Application to Pediatric Brain Diseases. No To Hattatsu. 1995;27(2):138-45. doi: 10.11251/ojjscn1969.27.138.

135. Oliver KM, Hemenger RP, Corbett MC, O'Brart DP, Verma S,

Tomlinson A, et al. Corneal aberrations one year after photo refractive keratectomy for three types of ablation zone. American academy of optometry. 1995:183. PubMed PMID: CN-00518398.
136. Robertson CS, Gopinath SP, Chance B. A new application for near-infrared spectroscopy: detection of delayed intracranial hematomas after head injury. Journal of neurotrauma. 1995;12(4):591-600. doi: 10.1089/neu.1995.12.591. PubMed PMID: MEDLINE:8683610.
137. Schrader M, Schrader V. [Reliability of magnetic stimulation in the diagnosis of peripheral facial paralysis of idiopathic origin]. Rev Laryngol Otol Rhinol (Bord). 1995;116(2):123-7. Epub 1995/01/01. PubMed PMID: 7569373.
138. Schrader M, Schrader V. Reliability of magnetic stimulation in the diagnosis of idiopathic peripheral facial palsies. Revue de Laryngologie Otologie Rhinologie. 1995;116(2):123-7.
139. Snell GI, Salamonsen RF, Bergin P, Esmore DS, Khan S, Williams TJ. Inhaled nitric oxide used as a bridge to heart-lung transplantation in a patient with end-stage pulmonary hypertension. American Journal of Respiratory and Critical Care Medicine. 1995;151(4):1263-6. doi: 10.1164/ajrccm.151.4.7697264.
140. Tassi C, Gallai V, Rossi A. Serum creatine kinase BB isoenzyme in subjects evaluated by trancranial magnetic stimulation:

Preliminary observations. Clinical Chemistry and Enzymology Communications. 1995;7(1):29-34.
141. Terao Y, Hayashi H, Shimizu T, Tanabe H, Hanajima R, Ugawa Y. Altered motor cortical excitability to magnetic stimulation in a patient with a lesion in globus pallidus. Journal of the Neurological Sciences. 1995;129(2):175-8. doi: 10.1016/0022-510X(94)00273-Q.
142. Vallar G, Rusconi ML, Barozzi S, Bernardini B, Ovadia D, Papagno C, et al. Improvement of left visuo-spatial hemineglect by left-sided transcutaneous electrical stimulation. Neuropsychologia. 1995;33(1):73-82. doi: 10.1016/0028-3932(94)00088-7.
143. Walker-Batson D, Smith P, Curtis S, Unwin H, Greenlee R. Amphetamine paired with physical therapy accelerates motor recovery after stroke; further evidence. Stroke. 1995;26(12):2254-9. doi: 10.1161/01.STR.26.12.2254.
144. Werhahn KJ, Classen J, Benecke R. The silent period induced by transcranial magnetic stimulation in muscles supplied by cranial nerves: Normal data and changes in patients. Journal of Neurology, Neurosurgery and Psychiatry. 1995;59(6):586-96. doi: 10.1136/jnnp.59.6.586.
145. Worley G, Hoffman JM, Paine SS, Kalman SL, Claerhout SJ, Boyko OB, et al. 18-FLUORODEOXYGLUCOSE POSITRON

EMISSION TOMOGRAPHY IN CHILDREN AND ADOLESCENTS WITH TRAUMATIC BRAIN INJURY. Developmental Medicine & Child Neurology. 1995;37(3):213-20. doi: 10.1111/j.1469-8749.1995.tb11994.x.

146. Karmel BZ, Gardner JM, Freedland RL. Arousal-modulated attention at four months as a function of intrauterine cocaine exposure and central nervous system injury. Journal of Pediatric Psychology. 1996;21(6):821-32. doi: 10.1093/jpepsy/21.6.821.

147. Kawamura T, Nakasato N, Seki K, Kanno A, Fujita S, Fujiwara S, et al. Neuromagnetic evidence of pre- and post-central cortical sources of somatosensory evoked responses. Electroencephalography and Clinical Neurophysiology - Evoked Potentials. 1996;100(1):44-50. doi: 10.1016/0168-5597(95)00217-0.

148. Konig F, Loble M, Wolfersdorf M. Depressive stupor--malignant neuroleptic syndrome--serotonin syndrome. A case contribution to a difficult differential diagnosis. Der Nervenarzt. 1996;67(5):407-12. PubMed PMID: MEDLINE:9005353.

149. König F, Löble M, Wolfersdorf M. Depressive stupor - Malignant neuroleptic syndrome - Serotonin syndrome. A case report illustrating a problem in differential diagnosis. Nervenarzt. 1996;67(5):407-12.

150. Liepert J, Kotterba S, Tegenthoff M, Malin JP. Central fatigue

assessed by transcranial magnetic stimulation. Muscle and Nerve. 1996;19(11):1429-34. doi: 10.1002/(SICI)1097-4598(199611)19:11<1429::AID-MUS7>3.0.CO;2-E.

151. Pascual-Castroviejo I. Plasticidad cerebral. Revista de Neurologia. 1996;24(135):1361-6.

152. Pizzamiglio L, Vallar G, Magnotti L. Transcutaneous electrical stimulation of the neck muscles and hemineglect rehabilitation. Restorative Neurology and Neuroscience. 1996;10(4):197-203. doi: 10.3233/rnn-1996-10402.

153. Sader R, Meyer BU, Horch HH, Deppe H, Zeilhofer HF, Herzog M, et al. [Neurologic examinations for facial nerve damage in surgically treated mandibular collum fractures]. Fortschr Kiefer Gesichtschir. 1996;41:143-5. Epub 1996/01/01. PubMed PMID: 8755427.

154. Triplett G, Hill C, Freeman L, Rajan U, Templer DI. Incidence of head injury: lasting effects among college students and working adults in the general population. Perceptual and motor skills. 1996;83(3 Pt 2):1344-6. doi: 10.2466/pms.1996.83.3f.1344. PubMed PMID: MEDLINE:9017750.

155. Urban PP, Hopf HC, Connemann B, Hundemer HP, Koehler J. The course of cortico-hypoglossal projections in the human brainstem.

Functional testing using transcranial magnetic stimulation. Brain. 1996;119(3):1031-8. doi: 10.1093/brain/119.3.1031.

156. Verhagen HJM, Heijnen-Snyder GJ, Pronk A, Vroom TM, Van Vroonhoven TJMV, Eikelboom BC, et al. Thrombomodulin activity on mesothelial cells: Perspectives for mesothelial cells as an alternative for endothelial cells for cell seeding on vascular grafts. British Journal of Haematology. 1996;95(3):542-9. doi: 10.1046/j.1365-2141.1996.d01-1935.x.

157. Wagner W. Scalp, earlobe and nasopharyngeal recordings of the median nerve somatosensory evoked P14 potential in coma and brain death. Detailed latency and amplitude analysis in 181 patients. Brain. 1996;119 (Pt 5):1507-21. Epub 1996/10/01. doi: 10.1093/brain/119.5.1507. PubMed PMID: 8931576.

158. Yuya H, Nagata K, Takanashi Y, Satoh Y, Watahiki Y, Hirata Y, et al. Scalp topography of SEP late components in patients with supra-tentorial lesions. Brain Topography. 1996;8(3):333-6. doi: 10.1007/BF01184794.

159. Brouwer B, Hopkins-Rosseel DH. Motor cortical mapping of proximal upper extremity muscles following spinal cord injury. Spinal cord. 1997;35(4):205-12. doi: 10.1038/sj.sc.3100384. PubMed PMID: CN-00139385.

160. Charbel FT, Hoffman WE, Misra M, Hannigan K, Ausman JI.

Cerebral interstitial tissue oxygen tension, pH, HCO3, CO2. Surgical Neurology. 1997;48(4):414-7. doi: 10.1016/S0090-3019(96)00473-9.

161. Davis CH, Fardanesh L, Rubner D, Wanlass RL, McDonald CM. Profiles of functional recovery in fifty traumatically brain-injured patients after acute rehabilitation. American Journal of Physical Medicine and Rehabilitation. 1997;76(3):213-8. doi: 10.1097/00002060-199705000-00011.

162. Deal BJ, Strieper M, Scagliotti D, Hulse E, Auld D, Campbell R, et al. The medical therapy of cardioinhibitory syncope in pediatric patients. PACE - Pacing and Clinical Electrophysiology. 1997;20(7):1759-61. doi: 10.1111/j.1540-8159.1997.tb03563.x.

163. Goodwin GM. Neuropsychological and neuroimaging evidence for the involvement of the frontal lobes in depression. Journal of Psychopharmacology. 1997;11(2):115-22. doi: 10.1177/026988119701100204.

164. Heinemann F, Assion HJ, Hermes G, Ehrlich M. [Paroxetine-induced neuroleptic malignant syndrome]. Nervenarzt. 1997;68(8):664-6. Epub 1997/08/01. doi: 10.1007/s001150050178. PubMed PMID: 9380213.

165. Hirashima F, Yokota T. Influence of peripheral nerve stimulation on human motor cortical excitability in patients with ventrolateral thalamic lesion. Archives of Neurology.

1997;54(5):619-24. doi: 10.1001/archneur.1997.00550170091019.
166. Izumi SI, Takase M, Arita M, Masakado Y, Kimura A, Chino N. Transcranial magnetic stimulation-induced changes in EEG and responses recorded from the scalp of healthy humans. Electroencephalography and Clinical Neurophysiology. 1997;103(2):319-22. doi: 10.1016/S0013-4694(97)00007-2.
167. Kotterba S, Tegenthoff M, Malin JP. Perioperative lesions of the facial nerve: follow-up investigations using transcranial magnetic stimulation. Eur Arch Otorhinolaryngol. 1997;254(3):140-4. Epub 1997/01/01. doi: 10.1007/bf02471278. PubMed PMID: 9112034.
168. Lechner C, Behse F, Meisenzohl E, Brunkhorst F. Recovery time of Median nerve SEP following cerebral hypoxia in man. EEG-EMG Zeitschrift fur Elektroenzephalographie Elektromyographie und Verwandte Gebiete. 1997;28(3):136-40.
169. Maegaki Y, Maeoka Y, Ishii S, Shiota M, Takeuchi A, Yoshino K, et al. Mechanisms of central motor reorganization in pediatric hemiplegic patients. Neuropediatrics. 1997;28(3):168-74. Epub 1997/06/01. doi: 10.1055/s-2007-973695. PubMed PMID: 9266555.
170. Msikita W, Yaninek JS, Ahounou M, Baimey H, Fagbemissi R. First Report of Nattrassia mangiferae Root and Stem Rot of Cassava in West Africa. Plant Dis. 1997;81(11):1332. Epub 1997/11/01. doi: 10.1094/pdis.1997.81.11.1332a. PubMed PMID: 30861748.

171. Orlando LR, Luthi-Carter R, Standaert DG, Coyle JT, Penney JB, Jr., Young AB. N-acetylaspartylglutamate (NAAG) protects against rat striatal quinolinic acid lesions in vivo. Neurosci Lett. 1997;236(2):91-4. Epub 1997/12/24. doi: 10.1016/s0304-3940(97)00769-6. PubMed PMID: 9404819.

172. Pruna A, Peyri N, Berard M, Boffa MC. Thrombomodulin is synthesized by human mesangial cells. Kidney International. 1997;51(3):687-93. doi: 10.1038/ki.1997.99.

173. Raungratanaamporn O, Bhuripanyo K, Krittayaphong R, Aroonpruksakul S, Toomtong P, Chaithiraphan S. Radiofrequency catheter ablation in type I atrial flutter: preliminary experience of 10 cases. J Med Assoc Thai. 1997;80(7):466-72. Epub 1997/07/01. PubMed PMID: 9277077.

174. Taggart DP, Bhattacharya K, Meston N, Standing SJ, Kay JDS, Pillai R, et al. Serum S-100 protein concentration after cardiac surgery: A randomized trial of arterial line filtration. European Journal of Cardio-thoracic Surgery. 1997;11(4):645-9. doi: 10.1016/S1010-7940(96)01103-7.

175. Taub E, Munz M, Tasker RR. Chronic electrical stimulation of the gasserian ganglion for the relief of pain in a series of 34 patients. J Neurosurg. 1997;86(2):197-202. Epub 1997/02/01. doi: 10.3171/jns.1997.86.2.0197. PubMed PMID: 9010419.

176. Tobita M, Kuroiwa Y, Komiyama A, Hasegawa O, Miyasaki H, Sugiyama M, et al. Use of cortical SEP for the prediction of neurological prognosis after cardiopulmonary resuscitation. Brain and Nerve. 1997;49(2):131-6.

177. Urban PP, Hopf HC, Fleischer S, Zorowka PG, Müller-Forell W. Impaired cortico-bulbar tract function in dysarthria due to hemispheric stroke. Functional testing using transcranial magnetic stimulation. Brain. 1997;120(6):1077-84. doi: 10.1093/brain/120.6.1077.

178. Ustarroz JT, De Goicoechea FPNL, Ferreras AA, Arbonies AC. Ecological validity of neuropsychological tests in executive processes: A report of a cese. Anales de Psiquiatria. 1997;13(8):339-44.

179. Valeriani M, Restuccia D, Di Lazzaro V, Le Pera D, Scerrati M, Tonali P, et al. Giant central N20-P22 with normal area 3b N20-P20: An argument in favour of an area 3a generator of early median nerve cortical SEPs? Electroencephalography and Clinical Neurophysiology - Evoked Potentials. 1997;104(1):60-7. doi: 10.1016/S0168-5597(96)96660-5.

180. Vargo CP, Hickman DM. Cluster-like signs and symptoms respond to myofascial/craniomandibular treatment: a report of two cases. Cranio. 1997;15(1):89-93. Epub 1997/01/01. doi: 10.1080/08869634.1997.11745997. PubMed PMID: 9586493.

181. Woiciechowsky C. Neuropsychological and neurophysiological consequences of partial callosotomy. Journal of Neurosurgical Sciences. 1997;41(1):75-80.

182. Bertolasi L, Priori A, Tinazzi M, Bertasi V, Rothwell JC. Inhibitory action of forearm flexor muscle afferents on corticospinal outputs to antagonist muscles in humans. Journal of Physiology. 1998;511(3):947-56. doi: 10.1111/j.1469-7793.1998.947bg.x.

183. Bolton CF. Summary: Electrophysiology in the ICU. Canadian Journal of Neurological Sciences. 1998;25(SUPPL. 1):S43-S4. doi: 10.1017/s0317167100034739.

184. Butler JD, Miles J. Dysaesthetic neck pain with syncope. Pain. 1998;75(2-3):395-7. Epub 1998/05/16. doi: 10.1016/s0304-3959(97)00195-4. PubMed PMID: 9583777.

185. Caramia MD, Telera S, Palmieri MG, Wilson-Jones M, Scalise A, Iani C, et al. Ipsilateral motor activation in patients with cerebral gliomas. Neurology. 1998;51(1):196-202. doi: 10.1212/WNL.51.1.196.

186. Carrillo Esper R, Aguilar Ramirez MDP, Gargallo Hernandez JJ, Duran Vega HC, Alvarado Martinez C, Nunez Monroy FN, et al. Osborn wave in subarachnoid hemorrhage. Medicina Interna de Mexico. 1998;14(6):287-90.

187. Chistyakov AV, Soustiel JF, Hafner H, Elron M, Feinsod M.

Altered excitability of the motor cortex after minor head injury revealed by transcranial magnetic stimulation. Acta Neurochir (Wien). 1998;140(5):467-72. Epub 1998/09/05. doi: 10.1007/s007010050126. PubMed PMID: 9728247.

188. Classen J, Knorr U, Werhahn KJ, Schlaug G, Kunesch E, Cohen LG, et al. Multimodal output mapping of human central motor representation on different spatial scales. J Physiol. 1998;512 (Pt 1)(Pt 1):163-79. Epub 1998/09/08. doi: 10.1111/j.1469-7793.1998.163bf.x. PubMed PMID: 9729626; PubMed Central PMCID: PMCPMC2231178.

189. Donaghy S, Wass PJ. Interrater reliability of the functional assessment measure in a brain injury rehabilitation program. Archives of Physical Medicine and Rehabilitation. 1998;79(10):1231-6. doi: 10.1016/S0003-9993(98)90267-2.

190. Hughes JR, Daaboul Y, Fino JJ, Shaw GL. The “Mozart Effect” on Epileptiform Activity. Clinical EEG and Neuroscience. 1998;29(3):109-19. doi: 10.1177/155005949802900301.

191. Ilmoniemi RJ. Neuromagnetic imaging and stimulation. Clinical Physiology. 1998;18(3):308.

192. Juneja G, Czyrny JJ, Linn RT. Admission balance and outcomes of patients admitted for acute inpatient rehabilitation. American Journal of Physical Medicine and Rehabilitation.

1998;77(5):388-93. doi: 10.1097/00002060-199809000-00005.

193. Kobayashi M, Ohira T, Ishihara M, Kawase T, Takase M. Transcranial magnetic stimulation of the oculomotor and abducens nerves: Determining the site of excitation in the cat. Journal of Clinical Neurophysiology. 1998;15(4):358-63. doi: 10.1097/00004691-199807000-00008.

194. Maccabee PJ, Nagarajan SS, Amassian VE, Durand DM, Szabo AZ, Ahad AB, et al. Influence of pulse sequence, polarity and amplitude on magnetic stimulation of human and porcine peripheral nerve. Journal of Physiology. 1998;513(2):571-85. doi: 10.1111/j.1469-7793.1998.571bb.x.

195. McMillan AS, Watson C, Walshaw D. Transcranial magnetic-stimulation mapping of the cortical topography of the human masseter muscle. Arch Oral Biol. 1998;43(12):925-31. Epub 1999/01/07. doi: 10.1016/s0003-9969(98)00081-8. PubMed PMID: 9877323.

196. Mosqueda-Garcia R, Fernandez-Violante R, Tank J, Snell M, Cunningham G, Furlan R. Yohimbine in neurally mediated syncope: Pathophysiological implications. Journal of Clinical Investigation. 1998;102(10):1824-30. doi: 10.1172/JCI3050.

197. Pérennou DA, Amblard B, Leblond C, Pélissier J. Biased postural vertical in humans with hemispheric cerebral lesions.

Neuroscience Letters. 1998;252(2):75-8. doi: 10.1016/S0304-3940(98)00501-1.

198. Philips M. Brainstem origins of the N18 component of the somatosensory evoked response. Skull Base Surgery. 1998;8(3):133-40. doi: 10.1055/s-2008-1058572.

199. Porst H. Transurethral application of Alprostadil with MUSE(TM) (Medicated Urethral System for Erection). Actual overview and personal experiences. Urologe - Ausgabe A. 1998;37(4):410-6. doi: 10.1007/s001200050199.

200. Schluter ND, Rushworth MFS, Passingham RE, Mills KR. Temporary interference in human lateral premotor cortex suggests dominance for the selection of movements. A study using transcranial magnetic stimulation. Brain. 1998;121(5):785-99. doi: 10.1093/brain/121.5.785.

201. Triggs WJ, Tesar DW, Young MS. Ipsilateral motor control in pathological left-handedness. Neurocase. 1998;4(1):65-9. doi: 10.1093/neucas/4.1.65.

202. Zuhosky JP, Dugan SA, Young JL, Bode RK, Kelly JP. A retrospective review of the incidence and rehabilitation outcome of concomitant traumatic brain injury and ligamentous knee injury. Archives of Physical Medicine and Rehabilitation. 1998;79(7):805-10. doi: 10.1016/S0003-9993(98)90360-4.

203. Béliveau A, Leclerc S, Rouleau M, Guérin SL. Multiple cloning sites from mammalian expression vectors interfere with gene promoter studies in vitro. European Journal of Biochemistry. 1999;261(2):585-90. doi: 10.1046/j.1432-1327.1999.00362.x.

204. Bischoff SC, Sellge G, Schwengberg S, Lorentz A, Manns MP. Stem cell factor-dependent survival, proliferation and enhanced releasability of purified mature mast cells isolated from human intestinal tissue. International Archives of Allergy and Immunology. 1999;118(2-4):104-7. doi: 10.1159/000024041.

205. Chevion S, Or R, Berry EM. The antioxidant status of patients subjected to total body irradiation. Biochemistry and Molecular Biology International. 1999;47(6):1019-27.

206. Chistyakov AV, Hafner H, Soustiel JF, Trubnik M, Levy G, Feinsod M. Dissociation of somatosensory and motor evoked potentials in non-comatose patients after head injury. Clin Neurophysiol. 1999;110(6):1080-9. Epub 1999/07/13. doi: 10.1016/s1388-2457(99)00029-2. PubMed PMID: 10402095.

207. de Sousa RM, Regis FC, Koizumi MS. Traumatic brain injury: differences among pedestrians and motor vehicle occupants. Revista de saude publica. 1999;33(1):85-94. doi: 10.1590/s0034-89101999000100011. PubMed PMID: MEDLINE:10436625.

208. Dunai J, Bennett K, Fotiades A, Kritikos A, Castiello U. Modulation of unilateral neglect as a function of direction of object motion. NeuroReport. 1999;10(5):1041-7. doi: 10.1097/00001756-199904060-00027.

209. Ellaway PH, Moosavi SH, Stokes MJ, Catley M, Haque N. Indices of cortical motor function following severe brain injury in man. In: Binder MD, editor. Peripheral and Spinal Mechanisms in the Neural Control of Movement. Progress in Brain Research. 1231999. p. 473-9.

210. Elron M, Levi G, Trovnick M, Soustiel JF, Hafner H, Chistyakov A, et al. [Exhaustion of motor cortex after head injury--revealed by transcranial magnetic stimulation]. Harefuah. 1999;136(5):355-8, 419. Epub 2000/07/29. PubMed PMID: 10914237.

211.Escada P, Pratas Vital J, Capucho C, Lima C, Madeira Da Silva J, Silva Penha R. Meningoencephalic herniation into the middle ear. Revue de Laryngologie Otologie Rhinologie. 1999;120(1):47-52.

212. Godefroy O, Cabaret M, Petit-Chenal V, Pruvo JP, Rousseaux M. Control functions of the frontal lobes. Modularity of the central-supervisory system? Cortex. 1999;35(1):1-20. doi: 10.1016/S0010-9452(08)70782-2.

213. Goldstein LB. Pharmacological approach to functional

reorganization: The role of norepinephrine. Revue Neurologique. 1999;155(9):731-6.

214. Gusmão SS, Pittella JEH. Congestive brain swelling in victims of fatal road traffic accidents: Frequency and association with other head injury lesions. Arquivos de Neuro-Psiquiatria. 1999;57(4):965-70. doi: 10.1590/s0004-282x1999000600011.

215. Halper AS, Cherney LR, Cichowski K, Zhang M. Dysphagia after head trauma: The effect of cognitive-communicative impairments on functional outcomes. Journal of Head Trauma Rehabilitation. 1999;14(5):486-96. doi: 10.1097/00001199-199910000-00009.

216. Hasselmo ME. Neuromodulation: Acetylcholine and memory consolidation. Trends in Cognitive Sciences. 1999;3(9):351-9. doi: 10.1016/S1364-6613(99)01365-0.

217. Ilmoniemi RJ, Ruohonen J, Karhu J. Transcranial magnetic stimulation--a new tool for functional imaging of the brain. Crit Rev Biomed Eng. 1999;27(3-5):241-84. Epub 2000/06/23. PubMed PMID: 10864281.

218. Ilmoniemi RJ, Ruohonen J, Karhu J. Transcranial magnetic stimulation - A new tool for functional imaging of the brain. Critical Reviews in Biomedical Engineering. 1999;27(3-5):241-84. PubMed PMID: WOS:000087536900005.

219. James JD, Hauer-Jensen M. Effects of fixative and fixation

time for quantitative computerized image analysis of immunohistochemical staining. Journal of Histotechnology. 1999;22(2):109-11. doi: 10.1179/his.1999.22.2.109.
220. Kelly KM, Littlefield TR, Pomatto JK, Manwaring KH, Beals SP. Cranial growth unrestricted during treatment of deformational plagiocephaly. Pediatric Neurosurgery. 1999;30(4):193-9. doi: 10.1159/000028794.
221. Kikushima S, Kobayashi Y, Nakagawa H, Katagiri T. Triggering mechanism for neurally mediated syncope induced by head-up tilt test: Role of catecholamines and response to propranolol. Journal of the American College of Cardiology. 1999;33(2):350-7. doi: 10.1016/S0735-1097(98)00567-1.
222. Kobayashi M, Ohira T, Ochiai M, Mihara B, Kawase T. Transcranial magnetic stimulation of the central and peripheral motor pathways to the lingual muscles in cats. Clin Neurophysiol. 1999;110(3):445-8. Epub 1999/06/11. doi: 10.1016/s1388-2457(98)00015-7. PubMed PMID: 10363768.
223. Koscielniak-Nielsen ZJ, Nielsen PR, Nielsen SL, Gardi T, Hermann C. Comparison of transarterial and multiple nerve stimulation techniques for axillary block using a high dose of mepivacaine with adrenaline. Acta Anaesthesiol Scand. 1999;43(4):398-404. Epub 1999/05/04. doi:

10.1034/j.1399-6576.1999.430406.x. PubMed PMID: 10225072.

224. Liepert J, Weiller C. Mapping plastic brain changes after acute lesions. Current Opinion in Neurology. 1999;12(6):709-13. doi: 10.1097/00019052-199912000-00009. PubMed PMID: WOS:000167920600009.

225. Maegaki Y, Maeoka Y, Ishii S, Eda I, Ohtagaki A, Kitahara TJ, et al. Central motor reorganization in cerebral palsy patients with bilateral cerebral lesions. Pediatric Research. 1999;45(4):559-67. doi: 10.1203/00006450-199904010-00016.

226. Maini S, Deogaonkar SC. Transdermal electrical stimulation in sensorineural tinnitus. Indian Journal of Otolaryngology and Head and Neck Surgery. 1999;51(2):37-9. doi: 10.1007/BF02997987.

227. Marchand-Pauvert V, Mazevet D, Pierrot-Deseilligny E, Pol S, Pradat-Diehl P. Handedness-related asymmetry in transmission in a system of human cervical premotoneurones. Experimental Brain Research. 1999;125(3):323-34. doi: 10.1007/s002210050688.

228. Marmarou A, Nichols J, Burgess J, Newell D, Troha J, Burnham D, et al. Effects of the bradykinin antagonist Bradycor(TM) (Deltibant, CP-1027) in severe traumatic brain injury: Results of a multi-center, randomized, placebo-controlled trial. Journal of Neurotrauma. 1999;16(6):431-44. doi: 10.1089/neu.1999.16.431.

229. Marret S, Bonnier C, Raymackers JM, Delpech A, Evrard P,

Gressens P. Glycine antagonist and NO°synthase inhibitor protect the developing mouse brain against neonatal excitotoxic lesions. Pediatric Research. 1999;45(3):337-42. doi: 10.1203/00006450-199903000-00008.
230. Mayr J, Fechter M, Grechenig W. Functional electrical stimulation (FES) for treatment of head injury sequelae in children. Acta Chirurgica Austriaca. 1999;31(3):167-9. doi: 10.1007/BF02619996.
231. Moosavi SH, Ellaway PH, Catley M, Stokes MJ, Haque N. Corticospinal function in severe brain injury assessed using magnetic stimulation of the motor cortex in man. J Neurol Sci. 1999;164(2):179-86. Epub 1999/07/13. doi: 10.1016/s0022-510x(99)00065-9. PubMed PMID: 10402031.
232. Murakami H, Harada H, Saito Y. Design and analysis of Ni-base superalloys on an atomistic basis. Journal of the Japan Institute of Metals. 1999;63(6):723-32. doi: 10.2320/jinstmet1952.63.6_723. PubMed PMID: WOS:000081359600008.
233. Nezu A, Kimura S, Takeshita S, Tanaka M. Functional recovery in hemiplegic cerebral palsy: Ipsilateral electromyographic responses to focal transcranial magnetic stimulation. Brain and Development. 1999;21(3):162-5. doi:

10.1016/S0387-7604(98)00094-1.
234. Nico D. Effectiveness of sensory stimulation on tactile extinction. Experimental Brain Research. 1999;127(1):75-82. doi: 10.1007/s002210050775.
235. Nudo RJ, Friel KM. Cortical plasticity after stroke: Implications for rehabilitation. Revue Neurologique. 1999;155(9):713-7.
236. Oliveri M, Rossini PM, Traversa R, Cicinelli P, Filippi MM, Pasqualetti P, et al. Left frontal transcranial magnetic stimulation reduces contralesional extinction in patients with unilateral right brain damage. Brain. 1999;122(9):1731-9. doi: 10.1093/brain/122.9.1731.
237. Palmieri MG, Iani C, Scalise A, Desiato MT, Loberti M, Telera S, et al. The effect of benzodiazepines and flumazenil on motor cortical excitability in the human brain. Brain Res. 1999;815(2):192-9. Epub 1999/01/08. doi: 10.1016/s0006-8993(98)01164-0. PubMed PMID: 9878733.
238. Pascual-Leone A, Bartres-Faz D, Keenan JP. Transcranial magnetic stimulation: studying the brain-behaviour relationship by induction of 'virtual lesions'. Philos Trans R Soc Lond B Biol Sci. 1999;354(1387):1229-38. Epub 1999/08/31. doi: 10.1098/rstb.1999.0476. PubMed PMID: 10466148; PubMed Central PMCID: PMCPMC1692644.

239. Piovan E, Zampieri PG, Alessandrini F, Rosta L, Maschio A, Beltramello A. Xenon-CT in skull-head injury. Rivista Medica. 1999;5(1-2):53-9.

240. Ravishankar M, Parthasarathy S, Saravanan P. Labour analgesia. Journal of Anaesthesiology Clinical Pharmacology. 1999;15(3):225-52.

241. Robertson IH, Murre JMJ. Rehabilitation of brain damage: Brain plasticity and principles of guided recovery. Psychological Bulletin. 1999;125(5):544-75. doi: 10.1037/0033-2909.125.5.544. PubMed PMID: WOS:000082379600003.

242. Rogers RD, Everitt BJ, Baldacchino A, Blackshaw AJ, Swainson R, Wynne K, et al. Dissociable deficits in the decision-making cognition of chronic amphetamine abusers, opiate abusers, patients with focal damage to prefrontal cortex, and tryptophan-depleted normal volunteersEvidence for monoaminergic mechanisms. Neuropsychopharmacology. 1999;20(4):322-39. doi: 10.1016/S0893-133X(98)00091-8.

243. Rohde V, Irle S, Hassler WE. Prediction of the post-comatose motor function by motor evoked potentials obtained in the acute phase of traumatic and non-traumatic coma. Acta Neurochirurgica. 1999;141(8):841-8. doi: 10.1007/s007010050385. PubMed PMID: WOS:000082257500012.

244. Rona R, Piantanida S, Bolis C, Cuccia S Protti E, Cantoni A, Rivolta G. (Caso clinico). Acta Anaesthesiologica Italica / Anaesthesia and Intensive Care in Italy. 1999;50(2):129-32.

245. Roncucci P, Lepori P, Mok MS, Bayat A, Logi F, Marino A. Nasopharyngeal electrode recording of somatosensory evoked potentials as an indicator in brain death. Anaesthesia and Intensive Care. 1999;27(1):20-5. doi: 10.1177/0310057x9902700104. PubMed PMID: WOS:000078637700004.

246. Rytz R, Hoffmann R. Chemical bonding in the ternary transition metal bismuthides Ti4TBi2 with T = Cr, Mn, Fe, Co, and Ni. Inorganic Chemistry. 1999;38(7):1609-17. doi: 10.1021/ic981075v. PubMed PMID: WOS:000079620400039.

247. Steenerson RL, Cronin GW. Treatment of tinnitus with electrical stimulation. Otolaryngol Head Neck Surg. 1999;121(5):511-3. Epub 1999/11/05. doi: 10.1016/s0194-5998(99)70048-3. PubMed PMID: 10547461.

248. Stohr M, Hartmann M. Evoked potentials: New clinical applications. Aktuelle Neurologie. 1999;26(4):147-51. doi: 10.1055/s-2007-1017624. PubMed PMID: WOS:000081194000001.

249. Taub E, Uswatte G, Pidikiti R. Constraint-Induced Movement Therapy: a new family of techniques with broad application to physical rehabilitation--a clinical review. J Rehabil Res Dev.

1999;36(3):237-51. Epub 2000/02/05. PubMed PMID: 10659807.

250. Taub E, Uswatte G, Pidikiti R. Constraint-Induced Movement Therapy: A new family of techniques with broad application to physical rehabilitation - A clinical review. Journal of Rehabilitation Research and Development. 1999;36(3):237-51. PubMed PMID: WOS:000085584900011.

251. Terao Y, Ugawa Y, Hanajima R, Furubayashi T, Machii K, Enomoto H, et al. Air-puff-induced facilitation of motor cortical excitability studied in patients with discrete brain lesions. Brain. 1999;122 (Pt 12):2259-77. Epub 1999/12/03. doi: 10.1093/brain/122.12.2259. PubMed PMID: 10581221.

252. Zanetta JP, Timmerman P, Leroy Y. Gas-liquid chromatography of the heptafluorobutyrate derivatives of the O-methyl-glycosides on capillary columns: a method for the quantitative determination of the monosaccharide composition of glycoproteins and glycolipids. Glycobiology. 1999;9(3):255-66. Epub 1999/02/20. doi: 10.1093/glycob/9.3.255. PubMed PMID: 10024663.

253. Al-Wahadneh AM, Khriesat IA, Kuda EH. Adverse reactions of intravenous immunoglobulin. Saudi Medical Journal. 2000;21(10):953-6.

254. Balbi P, Trojano L, Ragno M, Perretti A, Santoro L. Patterns of motor control reorganization in a patient with mirror movements.

Clinical Neurophysiology. 2000;111(2):318-25. doi: 10.1016/S1388-2457(99)00239-4.
255. Chen WJ, Li CC. Oceanic rain rate retrievals using TRMM microwave imager multi-channel brightness temperatures during the 1998 SCSMEX. Terrestrial Atmospheric and Oceanic Sciences. 2000;11(4):765-88. doi: 10.3319/tao.2000.11.4.765(a). PubMed PMID: WOS:000166314400002.
256. Colletti V, Fiorino FG, Carner M, Giarbini N, Sacchetto L, Cumer G. Advantages of the retrosigmoid approach in auditory brain stem implantation. Skull Base Surgery. 2000;10(4):165-70.
257. Couper FJ, Logan BK. Determination of gamma-hydroxybutyrate (GHB) in biological specimens by gas chromatography--mass spectrometry. J Anal Toxicol. 2000;24(1):1-7. Epub 2000/02/02. doi: 10.1093/jat/24.1.1. PubMed PMID: 10654561.
258. Couper FJ, Logan BK. Determination of gamma-hydroxybutyrate (GHB) in biological specimens by cas chromatography - Mass spectrometry. Journal of Analytical Toxicology. 2000;24(1):1-7. doi: 10.1093/jat/24.1.1. PubMed PMID: WOS:000084875200001.
259. Couper FJ, Logan BK. Determination of γ-hydroxybutyrate (GHB) in biological specimens by gas chromatography-mass spectrometry. Journal of Analytical Toxicology. 2000;24(1):1-7. doi:

10.1093/jat/24.1.1.
260. Cowey A, Walsh V. Magnetically induced phosphenes in sighted, blind and blindsighted observers. Neuroreport. 2000;11(14):3269-73. Epub 2000/10/24. doi: 10.1097/00001756-200009280-00044. PubMed PMID: 11043562.
261. Fins JJ. A proposed ethical framework for interventional cognitive neuroscience: a consideration of deep brain stimulation in impaired consciousness. Neurol Res. 2000;22(3):273-8. Epub 2000/04/19. doi: 10.1080/01616412.2000.11740670. PubMed PMID: 10769820.
262. Fitzpatrick SM, Rothman DL. A James S. McDonnell Foundation Workshop: Meeting report: Transcranial magnetic stimulation and studies of human cognition. Journal of Cognitive Neuroscience. 2000;12(4):704-9. doi: 10.1162/089892900562327.
263. Frackowiak RS. Forthergillian Lecture. Imaging human brain function. Transactions of the Medical Society of London. 2000;117:53-63.
264. García-Larrea L, Peyron R, Mertens P, Laurent B, Mauguière F, Sindou M. Functional imaging and neurophysiological assessment of spinal and brain therapeutic modulation in humans. Arch Med Res. 2000;31(3):248-57. Epub 2000/10/19. doi: 10.1016/s0188-4409(00)00083-7. PubMed PMID: 11036174.

265. Gómez-Fernández L. [Cortical plasticity and restoration of neurologic functions: an update on this topic]. Rev Neurol. 2000;31(8):749-56. Epub 2000/11/18. PubMed PMID: 11082885.

266. Gomez-Fernandez L. Cortical plasticity and neurological functional restoring: An update. Revista De Neurologia. 2000;31(8):749-56. doi: 10.33588/rn.3108.2000358. PubMed PMID: WOS:000165189400012.

267. Gray DS, Burnham RS. Preliminary outcome analysis of a long-term rehabilitation program for severe acquired brain injury. Archives of Physical Medicine and Rehabilitation. 2000;81(11):1447-56. doi: 10.1053/apmr.2000.16343.

268. Har-El G, McPhee JR. Transcranial magnetic stimulation in acute facial nerve injury. Laryngoscope. 2000;110(7):1105-11. Epub 2000/07/13. doi: 10.1097/00005537-200007000-00007. PubMed PMID: 10892678.

269. Hjortdal JO, Olsen H, Ehlers N. A prospective randomized comparative study of the optical performance of the cornea one year after radial keratotomy or photorefractive keratectomy. IOVS. 2000;41:ARVO Abstract 3612. PubMed PMID: CN-00679029.

270. Hobson JA, Pace-Schott EF, Stickgold R. Dreaming and the brain: Toward a cognitive neuroscience of conscious states. Behavioral and Brain Sciences. 2000;23(6):793-842. doi:

10.1017/S0140525X00003976.
271. Holloway V, Gadian DG, Vargha-Khadem F, Porter DA, Boyd SG, Connelly A. The reorganization of sensorimotor function in children after hemispherectomy. A functional MRI and somatosensory evoked potential study. Brain. 2000;123(12):2432-44. doi: 10.1093/brain/123.12.2432.
272. Karataş M, Dilek A, Erkan H, Yavuz N, Sözay S, Akman N. Functional outcome in stroke patients with atrial fibrillation. Archives of Physical Medicine and Rehabilitation. 2000;81(8):1025-9. doi: 10.1053/apmr.2000.6981.
273. Kasahata N, Shiota JI, Kawamura M, Miyazawa Y, Sugita K. Pure sensory stroke due to a pontine lesion: A study of clinical symptomatology and somatosensory evoked potential findings. Brain and Nerve. 2000;52(10):913-7.
274. Kastrup O, Leonhardt G, Kurthen M, Hufnagel A. Cortical motor reorganization following early brain damage and hemispherectomy demonstrated by transcranial magnetic stimulation. Clinical Neurophysiology. 2000;111(8):1346-52. doi: 10.1016/S1388-2457(00)00339-4.
275. Kessels RPC, D'Alfonso AAL, Postma A, De Haan EHF. Spatial working memory performance after high-frequency repetitive transcranial magnetic stimulation of the left and right posterior parietal

cortex in humans. Neuroscience Letters. 2000;287(1):68-70. doi: 10.1016/S0304-3940(00)01146-0.

276. Kudoh A, Matsuki A. Current perception thresholds of epileptic patients treated with valproate. Seizure. 2000;9(7):498-501. Epub 2000/10/18. doi: 10.1053/seiz.2000.0449. PubMed PMID: 11034875.

277. Laine M. The learning brain. Brain and Language. 2000;71(1):132-4. doi: 10.1006/brln.1999.2232.

278. Oliveri M, Rossini PM, Cicinelli P, Traversa R, Pasqualetti P, Filippi MM, et al. Neurophysiological evaluation of tactile space perception deficits through transcranial magnetic stimulation. Brain Research Protocols. 2000;5(1):25-9. doi: 10.1016/S1385-299X(99)00055-0.

279. Oliveri M, Rossini PM, Filippi MM, Traversa R, Cicinelli P, Palmieri MG, et al. Time-dependent activation of parieto-frontal networks for directing attention to tactile space. A study with paired transcranial magnetic stimulation pulses in right-brain-damaged patients with extinction. Brain. 2000;123 (Pt 9):1939-47. Epub 2000/08/26. doi: 10.1093/brain/123.9.1939. PubMed PMID: 10960057.

280. Oliveri M, Rossini PM, Filippi MM, Traversa R, Cicinelli P, Palmieri MG, et al. Time-dependent activation of parieto-frontal

networks for directing attention to tactile space - A study with paired transcranial magnetic stimulation pulses in right-brain-damaged patients with extinction. Brain. 2000;123:1939-47. doi: 10.1093/brain/123.9.1939. PubMed PMID: WOS:000089070100016.

281. Philbert MA, Billingsley ML, Reuhl KR. Mechanisms of Injury in the Central Nervous System. Toxicologic Pathology. 2000;28(1):43-53. doi: 10.1177/019262330002800107.

282. Pilipenko P. Study of the pyramidal tract in comatose patients by transcranial magnetic stimulation. Brain Pathology. 2000;10(4):654-5. PubMed PMID: WOS:000088213000347.

283. Restuccia D. Anatomic origin of P13 and P14 scalp far-field potentials. J Clin Neurophysiol. 2000;17(3):246-57. Epub 2000/08/06. doi: 10.1097/00004691-200005000-00003. PubMed PMID: 10928637.

284. Röricht S, Meyer BU. Residual function in motor cortex contralateral to amputated hand. Neurology. 2000;54(4):984-7. doi: 10.1212/WNL.54.4.984.

285. Schiff ND, Rezai AR, Plum F. A neuromodulation strategy for rational therapy of complex brain injury states. Neurol Res. 2000;22(3):267-72. Epub 2000/04/19. doi: 10.1080/01616412.2000.11740669. PubMed PMID: 10769819.

286. Schwarz S, Hacke W, Schwab S. Magnetic evoked potentials

in neurocritical care patients with acute brainstem lesions. Journal of the Neurological Sciences. 2000;172(1):30-7. doi: 10.1016/s0022-510x(99)00268-3. PubMed PMID: WOS:000085188000006.

287. Sharma JC, Fletcher S, Vassallo M, Ross I. Prognostic value of CT scan features in acute ischaemic stroke and relationship with clinical stroke syndromes. Int J Clin Pract. 2000;54(8):514-8. Epub 2001/02/24. PubMed PMID: 11198729.

288. Shimizu T, Nariai T, Maehara T, Hino T, Komori T, Shimizu H, et al. Enhanced motor cortical excitability in the unaffected hemisphere after hemispherectomy. NeuroReport. 2000;11(14):3077-84. doi: 10.1097/00001756-200009280-00009.

289. Sindou M, Mertens P. Neurosurgical management of neuropathic pain. Stereotact Funct Neurosurg. 2000;75(2-3):76-80. Epub 2001/12/12. doi: 10.1159/000048386. PubMed PMID: 11740173.

290. Sterr A, Schmalohr D, Kolbel S, Freivogel S. Transcranial magnetic stimulation-based investigation of motor cortex reorganization after repetitive training in brain-injured patients. Psychophysiology. 2000;37:S95-S. PubMed PMID: WOS:000089900500388.

291. Stowe CD, Lee KR, Storgion SA, Phelps SJ. Altered

phenytoin pharmacokinetics in children with severe, acute traumatic brain injury. Journal of Clinical Pharmacology. 2000;40(12 II):1452-61. doi: 10.1177/009127000004001216.
292. Uclés P, Serrano JL, Rosa F. Central conduction time of magnetic brain stimulation in attention-deficit hyperactivity disorder. J Child Neurol. 2000;15(11):723-8. Epub 2000/12/07. doi: 10.1177/088307380001501103. PubMed PMID: 11108505.
293. Walker-Batson D. Use of pharmacotherapy in the treatment of aphasia. Brain and Language. 2000;71(1):252-4. doi: 10.1006/brln.1999.2262.
294. Wood EM, Colton E, Yomtovian RA, Currie LM, Connor J, Anderson JM. Prevention of monocyte adhesion and inflammatory cytokine production during blood platelet storage: An in vitro model with implications for transfusion practice. Journal of Biomedical Materials Research. 2000;51(2):147-54. doi: 10.1002/(SICI)1097-4636(200008)51:2<147::AID-JBM1>3.0.CO;2-Y.
295. Wrisley DM, Sparto PJ, Whitney SL, Furman JM. Cervicogenic dizziness: A review of diagnosis and treatment. Journal of Orthopaedic & Sports Physical Therapy. 2000;30(12):755-66. doi: 10.2519/jospt.2000.30.12.755. PubMed PMID: WOS:000166180200005.

296. Abo M, Chen Z, Lai LJ, Reese T, Bjelke B. Functional recovery after brain lesion--contralateral neuromodulation: an fMRI study. Neuroreport. 2001;12(7):1543-7. Epub 2001/06/05. doi: 10.1097/00001756-200105250-00048. PubMed PMID: 11388445.

297. Abo M, Chen ZG, Lai LJ, Reese T, Bjelke B. Functional recovery after brain lesion - contralateral neuromodulation: an fMRI study. Neuroreport. 2001;12(7):1543-7. doi: 10.1097/00001756-200105250-00048. PubMed PMID: WOS:000169077800044.

298. Anthony DC, Blond D, Dempster R, Perry VH. Chemokine targets in acute brain injury and disease. Progress in Brain Research. 2001;132:507-24. doi: 10.1016/S0079-6123(01)32099-X.

299. Bezzi P, Domercq M, Vesce S, Volterra A. Neuron-astrocyte cross-talk during synaptic transmission: Physiological and neuropathological implications. Progress in Brain Research2001. p. 255-65.

300. Blazar BR, Min DL, Chung B, Panoskaltsis-Mortari A, Farrell C, Lacey D, et al. Thymic epithelial cell (TEC) injury is inhibited by pre-BMT keratinocyte growth factor (KGF): A new approach to facilitate thymopoiesis in alloBMT recipients. Faseb Journal. 2001;15(4):A666-A. PubMed PMID: WOS:000167438103826.

301. Blum T, Hoheisel U, Unger T, Mense S. Fibroblast growth

factor-2 acutely influences the impulse activity of rat dorsal horn neurones. Neuroscience Research. 2001;40(2):115-23. doi: 10.1016/s0168-0102(01)00217-6. PubMed PMID: WOS:000169343600002.

302. Blume WT. Current trends in electroencephalography. Curr Opin Neurol. 2001;14(2):193-7. Epub 2001/03/23. doi: 10.1097/00019052-200104000-00010. PubMed PMID: 11262735.

303. Chistyakov AV, Soustiel JF, Hafner H, Trubnik M, Levy G, Feinsod M. Excitatory and inhibitory corticospinal responses to transcranial magnetic stimulation in patients with minor to moderate head injury. J Neurol Neurosurg Psychiatry. 2001;70(5):580-7. Epub 2001/04/20. doi: 10.1136/jnnp.70.5.580. PubMed PMID: 11309450; PubMed Central PMCID: PMCPMC1737339.

304. Evans RG, Ventura S, Dampney RA, Ludbrook J. Neural mechanisms in the cardiovascular responses to acute central hypovolaemia. Clinical and Experimental Pharmacology and Physiology. 2001;28(5-6):479-87. doi: 10.1046/j.1440-1681.2001.03473.x.

305. Gorji A, Scheller D, Straub H, Tegtmeier F, Köhling R, Höhling JM, et al. Spreading depression in human neocortical slices. Brain Research. 2001;906(1-2):74-83. doi: 10.1016/S0006-8993(01)02557-4.

306. Hallett M. Functional reorganization after lesions of the human brain: Studies with transcranial magnetic stimulation. Revue Neurologique. 2001;157(8-9 I):822-6.

307. Hallett M. Brain plasticity and recovery from hemiplegia. Journal of Medical Speech-Language Pathology. 2001;9(2):107-15.

308. Hamada N, Oshika T, Amano S, Tomidokoro A, Mihashi T, Maeda N. Corneal wavefront aberration and contrast sensitivity after photorefractive keratectomy. Investigative Ophthalmology & Visual Science. 2001;42(4):S896-S. PubMed PMID: WOS:000168392104777.

309. Herrmann CS, Knight RT. Mechanisms of human attention: Event-related potentials and oscillations. Neuroscience and Biobehavioral Reviews. 2001;25(6):465-76. doi: 10.1016/S0149-7634(01)00027-6.

310. Hilgetag CC, Théoret H, Pascual-Leone A. Enhanced visual spatial attention ipsilateral to rTMS-induced 'virtual lesions' of human parietal cortex. Nature Neuroscience. 2001;4(9):953-7. doi: 10.1038/nn0901-953.

311.Hill NM, Sleigh JW. Transient epidermal flushing in a head injured patient: a case report. Crit Care Resusc. 2001;3(3):170-2. Epub 2006/04/01. PubMed PMID: 16573498.

312. Iwasaki M, Nakasato N, Kanno A, Hatanaka K, Nagamatsu K,

Nagamine Y, et al. Somatosensory evoked fields in comatose survivors after severe traumatic brain injury. Clin Neurophysiol. 2001;112(1):205-11. Epub 2001/01/04. doi: 10.1016/s1388-2457(00)00506-x. PubMed PMID: 11137679.

313. Kakkar A. Blast injury ear in a low intensity conflict. Indian Journal of Otolaryngology and Head and Neck Surgery. 2001;53(3):221-4.

314. Manthey D, Heck S, Engert S, Behl C. Estrogen induces a rapid secretion of amyloid β precursor protein via the mitogen-activated protein kinase pathway. European Journal of Biochemistry. 2001;268(15):4285-91. doi: 10.1046/j.1432-1327.2001.02346.x.

315. McAllister TW, Sparling MB, Flashman LA, Guerin SJ, Mamourian AC, Saykin AJ. Differential working memory load effects after mild traumatic brain injury. NeuroImage. 2001;14(5):1004-12. doi: 10.1006/nimg.2001.0899.

316. Miall C. Stimulating times for TMS. Trends in Cognitive Sciences. 2001;5(8):329. doi: 10.1016/s1364-6613(00)01737-x.

317. Mogilner AY, Rezai AR. Epidural motor cortex stimulation with functional imaging guidance. Neurosurg Focus. 2001;11(3):E4. Epub 2006/03/08. doi: 10.3171/foc.2001.11.3.5. PubMed PMID: 16519424.

318. Moritz CH, Rowley HA, Haughton VM, Swartz KR, Jones J, Badie B. Functional MR imaging assessment of a non-responsive brain injured patient. Magn Reson Imaging. 2001;19(8):1129-32. Epub 2001/11/17. doi: 10.1016/s0730-725x(01)00432-5. PubMed PMID: 11711238.

319. Nudo RJ, Plautz EJ, Frost SB. Role of adaptive plasticity in recovery of function after damage to motor cortex. Muscle & Nerve. 2001;24(8):1000-19. doi: 10.1002/mus.1104. PubMed PMID: WOS:000169947300003.

320. Oliveri M, Bisiach E, Brighina F, Piazza A, La Bua V, Buffa D, et al. rTMS of the unaffected hemisphere transiently reduces contralesional visuospatial heineglect. Neurology. 2001;57(7):1338-40. doi: 10.1212/WNL.57.7.1338.

321. Peri CV, Shaffrey ME, Farace E, Cooper E, Alves WM, Cooper JB, et al. Pilot study of electrical stimulation on median nerve in comatose severe brain injured patients: 3-month outcome. Brain Inj. 2001;15(10):903-10. Epub 2001/10/12. doi: 10.1080/02699050110065709. PubMed PMID: 11595086.

322. Piovan E, Cordopatri D, Cristofori L, Gambin R, Vivenza C, Beltramello A. Cerebral blood flow measured by Xenon-CT in severe head injury: Preliminary findings. Rivista di Neuroradiologia. 2001;14(4):395-405. doi: 10.1177/197140090101400404.

323. Piovan E, Grazioli A, Cordopatri D, Gambin R, Cristofori L, Beltramello A, et al. Xenon-CT in severe head injury. Rivista di Neuroradiologia. 2001;14(SUPPL. 3):175-82. doi: 10.1177/19714009010140s333.

324. Plewnia C, Bartels M, Cohen L, Gerloff C. Noradrenergic modulation of human cortex excitability by the presynaptic alpha(2)-antagonist yohimbine. Neurosci Lett. 2001;307(1):41-4. Epub 2001/08/23. doi: 10.1016/s0304-3940(01)01921-8. PubMed PMID: 11516570.

325. Plewnia C, Bartels M, Cohen L, Gerloff C. Noradrenergic modulation of human cortex excitability by the presynaptic α2-antagonist yohimbine. Neuroscience Letters. 2001;307(1):41-4. doi: 10.1016/S0304-3940(01)01921-8.

326. Ramos Rincón JM, García Ruipérez D, Aliaga Matas F, Lozano Cutillas MC, Llanos Llanos R, Herrero Huerta F. Specific mortality rates by DRG and main diagnosis according to CIE-9-MC at a level-II hospital. Anales de medicina interna (Madrid, Spain : 1984). 2001;18(10):510-6.

327. Restuccia D, Valeriani M, Barba C, Le Pera D, Capecci M, Filippini V, et al. Functional changes of the primary somatosensory cortex in patients with unilateral cerebellar lesions. Brain. 2001;124(4):757-68. doi: 10.1093/brain/124.4.757.

328. Ridding MC, Taylor JL. Mechanisms of motor-evoked potential facilitation following prolonged dual peripheral and central stimulation in humans. Journal of Physiology. 2001;537(2):623-31. doi: 10.1111/j.1469-7793.2001.00623.x.

329. Rollnik JD. Electrical stimulation of the sural nerve partially compensates effects of central fatigue. Acta Neurochirurgica, Supplement2001. p. 113-5.

330. Röricht S, Machetanz J, Irlbacher K, Niehaus L, Biemer E, Meyer BU. Reorganization of human motor cortex after hand replantation. Annals of Neurology. 2001;50(2):240-9. doi: 10.1002/ana.1091.

331. Satsou D, Kouakam C, Vaksmann G, Klug D, Kacet S, Rey C. Value of an implantable ECG monitor for the diagnosis of syncope due to arrhythmias in childhood. Archives des Maladies du Coeur et des Vaisseaux. 2001;94(5):527-30.

332. Savoy RL. History and future directions of human brain mapping and functional neuroimaging. Acta psychologica. 2001;107(1-3):9-42. doi: 10.1016/s0001-6918(01)00018-x. PubMed PMID: MEDLINE:11388144.

333. Sonde L, Bronge L, Kalimo H, Viitanen M. Can the site of brain lesion predict improved motor function after Low-TENS treatment on the post-stroke paretic arm? Clinical Rehabilitation.

2001;15(5):545-51. doi: 10.1191/026921501680425261.

334. Thickbroom GW, Byrnes ML, Archer SA, Nagarajan L, Mastaglia FL. Differences in sensory and motor cortical organization following brain injury early in life. Annals of Neurology. 2001;49(3):320-7. doi: 10.1002/ana.68. PubMed PMID: WOS:000167250100008.

335. Tran TD, Kubota M, Takeshita K, Yanagisawa M, Sakakihara Y. Varicella-associated acute necrotizing encephalopathy with a good prognosis. Brain and Development. 2001;23(1):54-7. doi: 10.1016/S0387-7604(00)00199-6.

336. Vecchio F, Menegazzo E, Salimbeni CF. A diagnostic-therapeutic protocol for head injury. Rivista di Neurobiologia. 2001;47(6):431-4.

337. Walker-Batson D, Curtis S, Natarajan R, Ford J, Dronkers N, Salmeron E, et al. A double-blind, placebo-controlled study of the use of amphetamine in the treatment of aphasia. Stroke. 2001;32(9):2093-7. doi: 10.1161/hs0901.095720.

338. Bechmann I, Nitsch R. Plasticity following lesion: Help and harm from the immune system. Restorative Neurology and Neuroscience. 2002;19(3-4):189-98.

339. Bevilaqua-Grosso D, Monteiro-Pedro V, Guirro RR, Bérzin F. A physiotherapeutic approach to craniomandibular disorders: a case

report. J Oral Rehabil. 2002;29(3):268-73. Epub 2002/03/19. doi: 10.1046/j.1365-2842.2002.00832.x. PubMed PMID: 11896844.

340. Block F. Treatment of neuropathic pain. Therapiewoche. 2002;18(9):416-22.

341. Brighina F, Bisiach E, Piazza A, Oliveri M, La Bua V, Daniele O, et al. Perceptual and response bias in visuospatial neglect due to frontal and parietal repetitive transcranial magnetic stimulation in normal subjects. NeuroReport. 2002;13(18):2571-5. doi: 10.1097/00001756-200212200-00038.

342. Bütefisch CM, Davis BC, Sawaki L, Waldvogel D, Classen J, Kopylev L, et al. Modulation of use-dependent plasticity by d-amphetamine. Ann Neurol. 2002;51(1):59-68. Epub 2002/01/10. doi: 10.1002/ana.10056. PubMed PMID: 11782985.

343. Carkeet A, Luo HD, Tong L, Saw SM, Tan DT. Refractive error and monochromatic aberrations in Singaporean children. Vision Res. 2002;42(14):1809-24. Epub 2002/07/20. doi: 10.1016/s0042-6989(02)00114-1. PubMed PMID: 12127112.

344. Chicurel M. Magnetic mind games. Nature. 2002;417(6885):114-6. doi: 10.1038/417114a. PubMed PMID: WOS:000175460200015.

345. Devulder J, Crombez E, Mortier E. Central pain: an overview. Acta Neurologica Belgica. 2002;102(3):97-103. PubMed PMID:

WOS:000178782200001.

346. Drouot X, Nguyen JP, Peschanski M, Lefaucheur JP. The antalgic efficacy of chronic motor cortex stimulation is related to sensory changes in the painful zone. Brain. 2002;125:1660-4. doi: 10.1093/brain/awf161. PubMed PMID: WOS:000176783500021.

347. Faella FS, Rossi M, Pagliano P, Attanasio V, Briante V, Fusco U, et al. Non post-operative spondylodiskitis. Our experience during the period 1990-2001. Infezioni in Medicina. 2002;10(3):157-62.

348. Fraser C, Power M, Hamdy S, Rothwell J, Hobday D, Hollander I, et al. Driving plasticity in human adult motor cortex is associated with improved motor function after brain injury. Neuron. 2002;34(5):831-40. Epub 2002/06/14. doi: 10.1016/s0896-6273(02)00705-5. PubMed PMID: 12062028.

349. Ghika J, Vingerhoets F. Are oestrogens neuroprotective? Medecine et Hygiene. 2002;60(2390):901-4.

350. Heitger MH, Anderson TJ, Jones RD. Saccade sequences as markers for cerebral dysfunction following mild closed head injury. In: Hyona J, Munoz DP, Heide W, Radach R, editors. Brain's Eye: Neurobiological and Clinical Aspects of Oculomotor Research. Progress in Brain Research. 1402002. p. 433-48.

351. Hoppner J, Bollow K, Lehmann C, Buchmann J, Schlafke D, Ernst K. EEG and cardiovascular symptoms during convulsive

syncope. Nervenheilkunde. 2002;21(1):49-52. PubMed PMID: WOS:000174263200009.

352. Johansen-Berg H, Rushworth MFS, Bogdanovic MD, Kischka U, Wimalaratna S, Matthews PM. The role of ipsilateral premotor cortex in hand movement after stroke. Proceedings of the National Academy of Sciences of the United States of America. 2002;99(22):14518-23. doi: 10.1073/pnas.222536799.

353. John ER. Editorial: Letter from the President. Clinical EEG Electroencephalography. 2002;33(3). doi: 10.1177/155005940203300303.

354. Kahveci F, Bekar A, Tamgac F. Tc-99 HMPAO cerebral SPECT imaging in brain death patients with complex spinal automatism. Ulus Travma Derg. 2002;8(4):198-201. Epub 2002/11/05. PubMed PMID: 12415498.

355. Katayama Y, Yamamoto T, Kobayashi K, Kasai M, Oshima H, Fukaya C. Motor cortex stimulation for phantom limb pain: Comprehensive therapy with spinal cord and thalamic stimulation. Stereotactic and Functional Neurosurgery. 2002;77(1-4):159-62. doi: 10.1159/000064593.

356. Knecht S, Flöel A, Dräger B, Breitenstein C, Sommer J, Henningsen H, et al. Degree of language lateralization determines susceptibility to unilateral brain lesions. Nature Neuroscience.

2002;5(7):695-9. doi: 10.1038/nn868.

357. Krames E. Implantable devices for pain control: spinal cord stimulation and intrathecal therapies. Best Pract Res Clin Anaesthesiol. 2002;16(4):619-49. Epub 2003/01/09. doi: 10.1053/bean.2002.0263. PubMed PMID: 12516895.

358. Legatt AD. Current practice of motor evoked potential monitoring: Results of a survey. Journal of Clinical Neurophysiology. 2002;19(5):454-60. doi: 10.1097/00004691-200210000-00008.

359. Lin YY, Forss N. Functional characterization of human second somatosensory cortex by magnetoencephalography. Behavioural Brain Research. 2002;135(1-2):141-5. doi: 10.1016/S0166-4328(02)00143-2.

360. Luft AR, Kaelin-Lang A, Hauser TK, Buitrago MM, Thakor NV, Hanley DF, et al. Modulation of rodent cortical motor excitability by somatosensory input. Experimental Brain Research. 2002;142(4):562-9. doi: 10.1007/s00221-001-0952-1. PubMed PMID: WOS:000174362100011.

361. Marshall JC, Fink GR, Halligan PW, Vallar G. Spatial awareness: A function of the posterior parietal lobe? Cortex. 2002;38(2):253-7. doi: 10.1016/S0010-9452(08)70654-3.

362. Meunier S, Katz R, Simonetta-Moreau M. Central nervous system lesions and segmental activity. Advances in Experimental

Medicine and Biology2002. p. 309-13.
363. Montes C, Mertens P, Convers P, Peyron R, Sindou M, Laurent B, et al. Cognitive effects of precentral cortical stimulation for pain control: an ERP study. Neurophysiologie Clinique-Clinical Neurophysiology. 2002;32(5):313-25. doi: 10.1016/s0987-7053(02)00340-4. PubMed PMID: WOS:000180085400004.
364. Montorsi F. The role of apomorphine SL in the treatment of erectile dysfunction. European Urology, Supplement. 2002;1(3):4-11. doi: 10.1016/S1569-9056(02)00008-8.
365. Münchau A, Orth M, Rothwell JC, Di Lazzaro V, Oliviero A, Profice P, et al. Intracortical inhibition is reduced in a patient with a lesion in the posterolateral thalamus. Movement Disorders. 2002;17(1):208-12. doi: 10.1002/mds.1264.
366. Oliveri M, Rossini PM, Filippi MM, Traversa R, Cicinelli P, Caltagirone C. Specific forms of neural activity associated with tactile space awareness. Neuroreport. 2002;13(8):997-1001. Epub 2002/06/13. doi: 10.1097/00001756-200206120-00002. PubMed PMID: 12060795.
367. Phillips LL, Reeves TM. Interactive pathology following traumatic brain injury modifies hippocampal plasticity. Restorative Neurology and Neuroscience. 2002;19(3-4):213-35.

368. Piroth MD, Zierhut D, van Kampen M, Sroka-Perez G, Wannenmacher M. [Lung dose depending on exact patient positioning during total body irradiation (TBI). Isoeffectiveness considerations to assess the risk of interstitial pneumonia after TBI]. Strahlenther Onkol. 2002;178(1):43-9. Epub 2002/04/30. doi: 10.1007/s00066-002-0876-0. PubMed PMID: 11977391.

369. Piroth MD, Zierhut D, van Kampen M, Sroka-Perez G, Wannenmacher M. Lung dose depending on exact patient positioning during total body irradiation (TBI) - Isoeffective considerations to assess the risk of interstitial pneumonitis after TBI. Strahlentherapie Und Onkologie. 2002;178(1):43-9. doi: 10.1007/s00066-002-0876-0. PubMed PMID: WOS:000174223700007.

370. Post RM, Speer AM, Obrocea GV, Leverich GS. Acute and prophylactic effects of anticonvulsants in bipolar depression. Clinical Neuroscience Research. 2002;2(3-4):228-51. doi: 10.1016/S1566-2772(02)00047-6.

371. Price CJ, Friston KJ. Degeneracy and cognitive anatomy. Trends in Cognitive Sciences. 2002;6(10):416-21. doi: 10.1016/S1364-6613(02)01976-9.

372. Ragazzoni A, Cincotta M, Borgheresi A, Zaccara G, Ziemann U. Congenital hemiparesis: Different functional reorganization of somatosensory and motor pathways. Clinical Neurophysiology.

2002;113(8):1273-8. doi: 10.1016/S1388-2457(02)00150-5.
373. Ravaglia S, Costa A, Ratti MT, Savoldi F, Bo P, Moglia A. Cognitive impairment and central motor conduction time in chronic alcoholics. Functional Neurology. 2002;17(2):83-6.
374. Ringholm A, Fredriksson R, Poliakova N, Yan YL, Postlethwait JH, Larhammar D, et al. One melanocortin 4 and two melanocortin 5 receptors from zebrafish show remarkable conservation in structure and pharmacology. Journal of Neurochemistry. 2002;82(1):6-18. doi: 10.1046/j.1471-4159.2002.00934.x.
375. Scheufler KM, Zentner J. Motor-evoked potential facilitation during progressive cortical suppression by propofol. Anesthesia and Analgesia. 2002;94(4):907-12. doi: 10.1097/00000539-200204000-00025. PubMed PMID: WOS:000174528600025.
376. Schiff ND, Plum F, Rezai AR. Developing prosthetics to treat cognitive disabilities resulting from acquired brain injuries. Neurol Res. 2002;24(2):116-24. Epub 2002/03/07. doi: 10.1179/016164102101199576. PubMed PMID: 11877893.
377. Schiff ND, Purpura KP. Towards a neurophysiological foundation for cognitive neuromodulation through deep brain stimulation. Thalamus and Related Systems. 2002;2(1):55-69. doi:

10.1016/S1472-9288(02)00028-6.

378. Segers S, Van Sassenbroeck D, Verstraete A, Martens P. Gamma-hydroxybutyric acid: From anaesthetic to illegal recreational drug. Tijdschrift voor Geneeskunde. 2002;58(1):49-54. doi: 10.2143/tvg.58.1.1001227.

379. Sommer M, Lang N, Tergau F, Paulus W. Neuronal tissue polarization induced by repetitive transcranial magnetic stimulation? NeuroReport. 2002;13(6):809-11. doi: 10.1097/00001756-200205070-00015.

380. Staudt M, Grodd W, Gerloff C, Erb M, Stitz J, Krägeloh-Mann I. Two types of ipsilateral reorganization in congenital hemiparesis: a TMS and fMRI study. Brain. 2002;125(Pt 10):2222-37. Epub 2002/09/24. doi: 10.1093/brain/awf227. PubMed PMID: 12244080.

381. Staudt M, Grodd W, Gerloff C, Erb M, Stitz J, Krageloh-Mann I. Two types of ipsilateral reorganization in congenital hemiparesis - A TMS and fMRI study. Brain. 2002;125:2222-37. doi: 10.1093/brain/awf227. PubMed PMID: WOS:000178613700006.

382. van Dijk KR, Scherder EJ, Scheltens P, Sergeant JA. Effects of transcutaneous electrical nerve stimulation (TENS) on non-pain related cognitive and behavioural functioning. Rev Neurosci. 2002;13(3):257-70. Epub 2002/10/31. doi: 10.1515/revneuro.2002.13.3.257. PubMed PMID: 12405228.

383. Wang XM, Fu HM, Long CG. The clinical research on the treatment of depression in patients with mild traumatic brain injury with repetitive transcranial magnetic stimulation. Chinese Journal of Clinical Rehabilitation. 2002;6(3).

384. Wong AMF, Tweed D, Sharpe JA. Adaptive neural mechanism for Listing's law revealed in patients with sixth nerve palsy. Investigative Ophthalmology and Visual Science. 2002;43(1):112-9.

385. Yokota H, Naoe Y, Nakabayashi M, Unemoto K, Kushimoto S, Kurokawa A, et al. Cerebral endothelial injury in severe head injury: The significance of measurements of serum thrombomodulin and the von Willebrand factor. Journal of Neurotrauma. 2002;19(9):1007-15. doi: 10.1089/089771502760341929.

386. Ziviani J, Ottenbacher KJ, Shephard K, Foreman S, Astbury W, Ireland P. Concurrent Validity of the Functional Independence Measure for Children (WeeFIM(™)) and the Pediatric Evaluation of Disabilities Inventory in Children with Developmental Disabilities and Acquired Brain Injuries. Physical and Occupational Therapy in Pediatrics. 2002;21(42038):91-101. doi: 10.1080/J006v21n02_08.

387. Abbott KC, Bernet VJ, Agodoa LY, Yuan CM. Diabetic ketoacidosis and hyperglycemic hyperosmolar syndrome after renal tranplantation in the United States. BMC Endocrine Disorders. 2003;3. doi: 10.1186/1472-6823-3-1.

388. Agudelo-Florez P, Palacio LG. Prevalence of Taenia solium antibodies in humans and in pigs in an endemic area of Colombia. Revista de Neurologia. 2003;36(8):706-9. doi: 10.33588/rn.3608.2002323.

389. Akamatsu N, Tsuji S. [Current diagnosis and treatment of status epilepticus]. Rinsho Shinkeigaku. 2003;43(11):799-801. Epub 2004/05/22. PubMed PMID: 15152468.

390. Andrews, Bowyer J, Paule M, Doyle A, Todorovic V, Alves, et al. Questions and answers: Session I: Clinical trials and beyond. Annals of the New York Academy of Sciences2003. p. 48-53.

391. Begley DJ. Understanding and circumventing the blood-brain barrier. Acta Paediatrica, International Journal of Paediatrics, Supplement. 2003;92(443):83-91. doi: 10.1111/j.1651-2227.2003.tb00226.x.

392. Brighina F, Bisiach E, Oliveri M, Piazza A, La Bua V, Daniele O, et al. 1 Hz repetitive transcranial magnetic stimulation of the unaffected hemisphere ameliorates contralesional visuospatial neglect in humans. Neuroscience Letters. 2003;336(2):131-3. doi: 10.1016/S0304-3940(02)01283-1.

393. Bütefisch CM, Netz J, Weßling M, Seitz RJ, Hömberg V. Remote changes in cortical excitability after stroke. Brain. 2003;126(2):470-81. doi: 10.1093/brain/awg044.

394. Campidelli C, Di Tommaso L, Zanetti G. Aneurysmal bone cyst of nasal cavity. A case report and review of the literature. Pathologica. 2003;95(2):103-7.

395. Canavero S, Bonicalzi V. Neuromodulation for central pain. Expert Review of Neurotherapeutics. 2003;3(5):591-607. PubMed Central PMCID: PMCBertek(United States)
Elan(Ireland)
Novartis(United States).

396. Cooper EB, Cooper JB. Electrical treatment of coma via the median nerve. Acta Neurochir Suppl. 2003;87:7-10. Epub 2003/10/02. doi: 10.1007/978-3-7091-6081-7_2. PubMed PMID: 14518514.

397. Coubard O, Kapoula Z, Müri R, Rivaud-Péchoux S. Effects of TMS over the right prefrontal cortex on latency of saccades and convergence. Investigative Ophthalmology and Visual Science. 2003;44(2):600-9. doi: 10.1167/iovs.02-0188.

398. Di Lazzaro V, Oliviero A, Mazzone P, Pilato F, Saturno E, Dileone M, et al. Generation of I waves in the human: spinal recordings. Supplements to Clinical neurophysiology. 2003;56:143-52. PubMed PMID: MEDLINE:14677388.

399. Fagade OO, Obilade TO. Therapeutic effect of TENS on post-IMF trismus and pain. Afr J Med Med Sci. 2003;32(4):391-4. Epub 2004/07/21. PubMed PMID: 15259924.

400. Feketova S. Central pain. Bolest. 2003;6(4):212-7.

401. Fins JJ. From psychosurgery to neuromodulation and palliation: history's lessons for the ethical conduct and regulation of neuropsychiatric research. Neurosurg Clin N Am. 2003;14(2):303-19, ix-x. Epub 2003/07/15. doi: 10.1016/s1042-3680(02)00118-3. PubMed PMID: 12856496.

402. Frederick RI. Review of the validity indicator profile. Journal of Forensic Neuropsychology. 2003;2(3-4):125-45. doi: 10.1300/J151v02n03_06.

403. Frisch S, Kotz SA, Von Cramon DY, Friederici AD. Why the P600 is not just a P300: The role of the basal ganglia. Clinical Neurophysiology. 2003;114(2):336-40. doi: 10.1016/S1388-2457(02)00366-8.

404. Greig P, Lilly L, Scudamore C, Erb S, Yoshida E, Kneteman N, et al. Early steroid withdrawal after liver transplantation: The Canadian tacrolimus versus microemulsion cyclosporin a trial: 1 year follow-up. Liver Transplantation. 2003;9(6):587-95. doi: 10.1053/jlts.2003.50102.

405. Halligan PW, Fink GR, Marshall JC, Vallar G. Spatial cognition: Evidence from visual neglect. Trends in Cognitive Sciences. 2003;7(3):125-33. doi: 10.1016/S1364-6613(03)00032-9.

406. He BX, Wang MD, Yan WP, Guo SW, Jiang HT, Bao G, et al.

Protective effect of neuronavigation microsurgery on brain function and its accuracy in the treatment of intracranial minute occupying-space lesions. Chinese Journal of Clinical Rehabilitation. 2003;7(28):3858-9.

407. Hjortdal JO, MÃ¸ller P, Iversen A. Corneal Aberrations after PRK and LASIK for Myopia. Results from a Prospective Randomised Trial. IOVS. 2003:ARVO E-abstract 2637. PubMed PMID: CN-00718134.

408. Holst B, Schwartz TW. Molecular mechanism of agonism and inverse agonism in the melanocortin receptors: Zn2+ as a structural and functional probe. Annals of the New York Academy of Sciences2003. p. 1-11.

409. Ilić TV, Korchounov A, Ziemann U. Methylphenidate facilitates and disinhibits the motor cortex in intact humans. NeuroReport. 2003;14(5):773-6. doi: 10.1097/00001756-200304150-00023.

410. Ishiyama Y. [Current neurophysiological tests and revised JSCN technical standards for clinical EEG]. Rinsho Byori. 2003;51(6):562-7. Epub 2003/07/30. PubMed PMID: 12884744.

411.Jaspert A, Grehl H. Principles of transcranial magnetic stimulation. Neurophysiologie-Labor. 2003;25(3):121-37.

412. Kamada K, Houkin K, Takeuchi F, Ishii N, Ikeda J, Sawamura

Y, et al. Visualization of the eloquent motor system by integration of MEG, functional, and anisotropic diffusion-weighted MRI in functional neuronavigation. Surgical Neurology. 2003;59(5):352-60. doi: 10.1016/S0090-3019(03)00018-1.

413. Kanno A, Nakasato N, Hatanaka K, Yoshimoto T. Ipsilateral area 3b responses to median nerve somatosensory stimulation. Neuroimage. 2003;18(1):169-77. doi: 10.1006/nimg.2002.1283. PubMed PMID: WOS:000180015200015.

414. Koch G, Oliveri M, Torriero S, Caltagirone C. Underestimation of time perception after repetitive transcranial magnetic stimulation. Neurology. 2003;60(11):1844-6. doi: 10.1212/WNL.60.11.1844.

415. Koehler J. Brainstem diagnosis via somatosensory-evoked potentials following stimulation of the median nerve. Klinische Neurophysiologie. 2003;34(1):2-7. doi: 10.1055/s-2003-37745.

416. Kolb B. Overview of cortical plasticity and recovery from brain injury. Physical Medicine and Rehabilitation Clinics of North America. 2003;14(1 SUPPL.):S7. doi: 10.1016/S1047-9651(02)00056-6.

417. Kotz SA, Frisch S, von Cramon DY, Friederici AD. Syntactic language processing: ERP lesion data on the role of the basal ganglia. Journal of the International Neuropsychological Society.

2003;9(7):1053-60. doi: 10.1017/S1355617703970093.

418. Liu JT, Wang CH, Chou IC, Sun SS, Koa CH, Cooper E. Regaining consciousness for prolonged comatose patients with right median nerve stimulation. Acta Neurochir Suppl. 2003;87:11-4. Epub 2003/10/02. doi: 10.1007/978-3-7091-6081-7_3. PubMed PMID: 14518515.

419. Longhi L, Roncati Zanier E, Valeriani V, Ghisoni L, Besozzi A, Ferrari C, et al. Brain vulnerability and its modulation. Minerva Anestesiologica. 2003;69(4):227-31.

420. Lu XR, Huang ZC, Man Y, Xia XH. Evaluation of cortex function in patients with migraine by transcranial magnetic stimulation. Chinese Journal of Clinical Rehabilitation. 2003;7(16):2298-9.

421. Miller KL. Urinary incontinence in elderly women. Primary Care Update for Ob/Gyns. 2003;10(5):242-6. doi: 10.1016/S1068-607X(03)00047-7.

422. Muramoto M, Yamazaki T, Nishimura S, Kita Y. Detailed in vitro pharmacological analysis of FK506-induced neuroprotection. Neuropharmacology. 2003;45(3):394-403. doi: 10.1016/S0028-3908(03)00168-0.

423. Nudo RJ. Adaptive plasticity in motor cortex: Implications for rehabilitation after brain injury. Journal of Rehabilitation Medicine,

Supplement. 2003(41):7-10.

424. Owen AM. HERA today, gone tomorrow? Trends in Cognitive Sciences. 2003;7(9):383-4. doi: 10.1016/S1364-6613(03)00195-5.

425. Özben T. Pathology of cerebral ischaemia: Mechanisms involved in neuronal damage. Jugoslovenska Medicinska Biokemija. 2003;22(2):85-92. doi: 10.2298/JMH0302085O.

426. Parsons LM. Superior parietal cortices and varieties of mental rotation. Trends in Cognitive Sciences. 2003;7(12):515-7. doi: 10.1016/j.tics.2003.10.002.

427. Pniewski J, Chodakowska-Zebrowska M, Woźniak R, Stępień K, Stafiej A. Plasma homocysteine level and the course of ischemic stroke. Acta Neurobiologiae Experimentalis. 2003;63(2):127-30.

428. Ragauskas A, Daubaris G, Ragaisis V, Petkus V. Implementation of non-invasive brain physiological monitoring concepts. Med Eng Phys. 2003;25(8):667-78. Epub 2003/08/06. doi: 10.1016/s1350-4533(03)00082-1. PubMed PMID: 12900182.

429. Reisner AD. The electroconvulsive therapy controversy: Evidence and ethics. Neuropsychology Review. 2003;13(4):199-219. doi: 10.1023/b:Nerv.0000009484.76564.58. PubMed PMID: WOS:000188844300002.

430. Rushworth MFS, Johansen-Berg H, Göbel SM, Devlin JT. The left parietal and premotor cortices: Motor attention and selection.

NeuroImage. 2003;20(SUPPL. 1):S89-S100. doi: 10.1016/j.neuroimage.2003.09.011.

431. Shibaki H, Shibaki A. Analysis of dermatophyte flora at a private clinic in Sapporo during the period 1992 to 2001. Japanese Journal of Medical Mycology. 2003;44(3):209-16. doi: 10.3314/jjmm.44.209.

432. Shimbo Y, Sakata M, Hayano M, Mori S. Topographical relationships between the Brainstem auditory and somatosensory evoked potentials and the location of lesions in posterior fossa stroke. Neurologia Medico-Chirurgica. 2003;43(6):282-91. doi: 10.2176/nmc.43.282. PubMed PMID: WOS:000183587400004.

433. Štětkářová I, Veselá M, Kryštůfková O. Early detection of nervous system dysfunction in patients with systemic lupus erythematosus by evoked potentials - A case report. Ceska Revmatologie. 2003;11(2):99-103.

434. Steyvers M, Levin O, Van Baelen M, Swinnen SP. Corticospinal excitability changes following prolonged muscle tendon vibration. NeuroReport. 2003;14(15):1901-5. doi: 10.1097/00001756-200310270-00004.

435. Stürmer B, Leuthold H. Control over response priming in visuomotor processing: A lateralized event-related potential study. Experimental Brain Research. 2003;153(1):35-44. doi:

10.1007/s00221-003-1579-1.

436. Sun T. Reorganization of brain function in children after hemispherectomy. Chinese Journal of Clinical Rehabilitation. 2003;7(4):680.

437. Taub E, Uswatte G, Morris DM. Improved motor recovery after stroke and massive cortical reorganization following Constraint-Induced Movement therapy. Phys Med Rehabil Clin N Am. 2003;14(1 Suppl):S77-91, ix. Epub 2003/03/11. doi: 10.1016/s1047-9651(02)00052-9. PubMed PMID: 12625639.

438. Théoret H, Kobayashi M, Valero-Cabré A, Pascual-Leone A. Exploring paradoxical functional facilitation with TMS. Suppl Clin Neurophysiol. 2003;56:211-9. Epub 2003/12/18. doi: 10.1016/s1567-424x(09)70224-7. PubMed PMID: 14677397.

439. Tomietto P, Annese V, D'Agostini S, Gremese E, Di Poi E, Ferraccioli G. Neuropsychological tests, MRI and SPECT in the evaluation of CNS involvement in SLE. Reumatismo. 2003;55(3):171-80.

440. Tomita Y, Fukuda C, Maegaki Y, Hanaki K, Kitagawa K, Sanpei M. Re-evaluation of short latency somatosensory evoked potentials (P13, P14 and N18) for brainstem function in children who once suffered from deep coma. Brain & Development. 2003;25(5):352-6. doi: 10.1016/s0387-7604(03)00023-8. PubMed

PMID: WOS:000184309900010.

441. Tyrer S, Lievesley A. Pain following traumatic brain injury: Assessment and management. Neuropsychological Rehabilitation. 2003;13(1-2):189-210. doi: 10.1080/09602010244000381.

442. Urban PP. Transcranial magnetic stimulation in brainstem lesions and lesions of the cranial nerves. Suppl Clin Neurophysiol. 2003;56:341-57. Epub 2003/12/18. doi: 10.1016/s1567-424x(09)70238-7. PubMed PMID: 14677411.

443. Urban PP. Transcranial magnetic stimulation (TMS) in brainstem lesions and lesions of the peripheral cranial nerves. Klinische Neurophysiologie. 2003;34(1):21-31. doi: 10.1055/s-2003-37749.

444. Wedekind C, Klug N. Facial F wave recording: a novel and effective technique for extra- and intraoperative diagnosis of facial nerve function in acoustic tumor disease. Otolaryngol Head Neck Surg. 2003;129(1):114-20. Epub 2003/07/19. doi: 10.1016/s0194-5998(03)00475-3. PubMed PMID: 12869926.

445. Wegner DM. The mind's best trick: How we experience conscious will. Trends in Cognitive Sciences. 2003;7(2):65-9. doi: 10.1016/S1364-6613(03)00002-0.

446. Williams WH, Evans JJ. Brain injury and emotion: An overview to a special issue on biopsychosocial approaches in

neurorehabilitation. Neuropsychological Rehabilitation. 2003;13(1-2):1-11.

447. Yang L. Mechanisms of central motor reorganization with transcranial magnetic stimulation in cerebral palsy. Chinese Journal of Clinical Rehabilitation. 2003;7(13):1938-9.

448. Bell EC, Baker GB, Poag C, Bellavance F, Khudabux J, Le Mellédo JM. Response to flumazenil in the late luteal phase and follicular phase of the menstrual cycle in healthy control females. Psychopharmacology. 2004;172(3):248-54. doi: 10.1007/s00213-003-1660-2.

449. Bernabeu M, Orient F, Tormos JM, Pascual-Leone A. Seizure induced by fast repetitive transcranial magnetic stimulation. Clinical Neurophysiology. 2004;115(7):1714-5. doi: 10.1016/j.clinph.2004.02.021. PubMed PMID: WOS:000222814800025.

450. Breitmeyer BG, Ro T, Ogmen H. A comparison of masking by visual and transcranial magnetic stimulation: implications for the study of conscious and unconscious visual processing. Conscious Cogn. 2004;13(4):829-43. Epub 2004/11/04. doi: 10.1016/j.concog.2004.08.007. PubMed PMID: 15522634.

451. Bueti D, Costantini M, Forster B, Aglioti SM. Uni- and cross-modal temporal modulation of tactile extinction in right brain

damaged patients. Neuropsychologia. 2004;42(12):1689-96. doi: 10.1016/j.neuropsychologia.2004.04.005.

452. Byblow W, Carson R. Sensorimotor coordination: Behavioural modes and neural mechanisms. Human Movement Science. 2004;23(3-4 SPE. ISS.):235-8. doi: 10.1016/j.humov.2004.08.005.

453. Cramer SC. Functional imaging in stroke recovery. Stroke. 2004;35(11 SUPPL. 1):2695-8. doi: 10.1161/01.STR.0000143326.36847.b0.

454. Deakin J, Aitken M, Robbins T, Sahakian BJ. Risk taking during decision-making in normal volunteers changes with age. Journal of the International Neuropsychological Society. 2004;10(4):590-8. doi: 10.1017/S1355617704104104.

455. Dobkin BH. Neurobiology of rehabilitation. Ann N Y Acad Sci. 2004;1038:148-70. Epub 2005/04/20. doi: 10.1196/annals.1315.024. PubMed PMID: 15838110; PubMed Central PMCID: PMCPMC4164200.

456. Dumas HM, Haley SM, Ludlow LH, Carey TM. Recovery of Ambulation during Inpatient Rehabilitation: Physical Therapist Prognosis for Children and Adolescents with Traumatic Brain Injury. Physical Therapy. 2004;84(3):232-42. doi: 10.1093/ptj/84.3.232.

457. Ellison A, Schindler I, Pattison LL, Milner AD. An exploration of the role of the superior temporal gyrus in visual search

and spatial perception using TMS. Brain. 2004;127(10):2307-15. doi: 10.1093/brain/awh244.

458. Feydy A, Krainik A, Bussel B, Maier MA. Cortical Reorganization Allows for Motor Recovery after Crossed Cerebrocerebellar Atrophy. Journal of Neuroimaging. 2004;14(1):49-53. doi: 10.1177/1051228403259867.

459. Gorbach AM. Local alternated temperature gradients as footprints of cortical functional activation. Journal of Thermal Biology. 2004;29(7-8 SPEC. ISS.):589-98. doi: 10.1016/j.jtherbio.2004.08.072.

460. Han SH, Jiang Y, Gu H, Rao HY, Mao LH, Cui Y, et al. The role of human parietal cortex in attention networks. Brain. 2004;127:650-9. doi: 10.1093/brain/awh071. PubMed PMID: WOS:000220108800016.

461. He CS, Yu Q, Yang DJ, Yang M. Interventional effects of low-frequency repetitive transcranial magnetic stimulation on patients with depression after traumatic brain injury. Chinese journal of clinical rehabilitation. 2004;8(28):6044-5. PubMed PMID: CN-01708746.

462. Heitger MH, Anderson TJ, Jones RD, Dalrymple-Alford JC, Frampton CM, Ardagh MW. Eye movement and visuomotor arm movement deficits following mild closed head injury. Brain. 2004;127:575-90. doi: 10.1093/brain/awh066. PubMed PMID: WOS:000220108800011.

463. Hiraoka T, Matsumoto Y, Okamoto F, Yamaguchi T, Hirohara Y, Mihashi T, et al. Corneal higher-order aberrations induced by overnight orthokerstology. Investigative Ophthalmology & Visual Science. 2004;45:U577-U. PubMed PMID: WOS:000223338001509.

464. Jääskeläinen SK. The utility of clinical neurophysiological and quantitative sensory testing for trigeminal neuropathy. J Orofac Pain. 2004;18(4):355-9. Epub 2005/01/08. PubMed PMID: 15636020.

465. Jeong HJ, Chung HS, Kim YH, Moon BS, Sung KK, Bai SJ, et al. Differential regulation by Seogak Jihwang-Tang on cytokines production in peripheral blood mononuclear cells from the cerebral infarction patients presenting with altered consciousness. Journal of Ethnopharmacology. 2004;94(2-3):289-94. doi: 10.1016/j.jep.2004.05.014.

466. Lafosse C, Kerckhofs E, Troch M, Santens P, Vandenbussche E. Graviceptive misperception of the postural vertical after right hemisphere damage. NeuroReport. 2004;15(5):887-91. doi: 10.1097/00001756-200404090-00031.

467. Landers M. Treatment-induced neuroplasticity following focal injury to the motor cortex. International Journal of Rehabilitation Research. 2004;27(1):1-5. doi: 10.1097/00004356-200403000-00001.

468. Lee CC, Chang WT, Fang CC, Tsai IL, Chen WJ. Sudden death caused by dissecting thoracic aortic aneurysm in a patient with

autosomal dominant polycystic kidney disease. Resuscitation. 2004;63(1):93-6. doi: 10.1016/j.resuscitation.2004.03.019.

469. Lefaucheur JP, Fnelon G, Mnard-Lefaucheur I, Wendling S, Nguyen JP. Low-frequency repetitive TMS of premotor cortex can reduce painful axial spasms in generalized secondary dystonia: A pilot study of three patients. Neurophysiologie Clinique. 2004;34(3-4):141-5. doi: 10.1016/j.neucli.2004.07.003.

470. Lenaerts ME, Couch JR, Couch JR. Posttraumatic Headache. Curr Treat Options Neurol. 2004;6(6):507-17. Epub 2004/10/06. doi: 10.1007/s11940-004-0008-5. PubMed PMID: 15461928.

471. Liepert J. Relevance of TMS for investigations in rehabilitation. Neurologie und Rehabilitation. 2004;10(5):227-32.

472. Liepert J, Hamzei F, Weiller C. Lesion-induced and training-induced brain reorganization. Restorative Neurology and Neuroscience. 2004;22(3-4):269-77.

473. Marshall RS. Neuroimaging in stroke recovery: A position paper from the first international workshop on neuroimaging and stroke recovery. Cerebrovascular Diseases. 2004;18(3):260-7. doi: 10.1159/000080293.

474. Martelletti P, van Suijlekom H. Cervicogenic headache: practical approaches to therapy. CNS Drugs. 2004;18(12):793-805. Epub 2004/09/21. doi: 10.2165/00023210-200418120-00004. PubMed

PMID: 15377169.

475. Martelletti P, van Suijlekom H. Cervicogenic headache - Practical approaches to therapy. Cns Drugs. 2004;18(12):793-805. doi: 10.2165/00023210-200418120-00004. PubMed PMID: WOS:000224601900004.

476. Masuda J, Takayama E, Satoh A, Kojima-Aikawa K, Suzuki K, Matsumoto I. A novel expression vector, designated as pHisJM, for producing recombinant His-fusion proteins. Biotechnology Letters. 2004;26(20):1543-8. doi: 10.1023/B:BILE.0000045650.90384.b2.

477. Merabet L, Thut G, Murray B, Andrews J, Hsiao S, Pascual-Leone A. Feeling by sight or seeing by touch? Neuron. 2004;42(1):173-9. doi: 10.1016/S0896-6273(04)00147-3.

478. Morecraft RJ, Stilwell-Morecraft KS, Rossing WR. The motor cortex and facial expression: New insights from neuroscience. Neurologist. 2004;10(5):235-49. doi: 10.1097/01.nrl.0000138734.45742.8d. PubMed PMID: WOS:000223868900002.

479. Nandi D, Aziz TZ. Deep brain stimulation in the management of neuropathic pain and multiple sclerosis tremor. J Clin Neurophysiol. 2004;21(1):31-9. Epub 2004/04/21. doi: 10.1097/00004691-200401000-00005. PubMed PMID: 15097292.

480. Obhi SS. Bimanual coordination: An unbalanced field of

research. Motor Control. 2004;8(2):111-20. doi: 10.1123/mcj.8.2.111.

481. Pageler L. Diagnosis and treatment of trigeminal autonomic cephalgias. Notfall und Hausarztmedizin. 2004;30(9):418-23. doi: 10.1055/s-2004-837354.

482. Paine P, Hamdy S, Hobson A, Mistry S, Gardener E, Aziz Q. Exploring the temporal characteristics of excitability in the human brain gut axis using transcranial magnetic stimulation. Gastroenterology. 2004;126(4):A589-A90. PubMed PMID: WOS:000220890202963.

483. Pilipenko P, Ierusalimsky A, Vostrikova E, Feigin V. Siberia: Novosibirsk's Neurological School. Practical Neurology. 2004;4(4):248-51. doi: 10.1111/j.1474-7766.2004.12-234.x.

484. Ro T, Shelton D, Lee OL, Chang E. Extrageniculate mediation of unconscious vision in transcranial magnetic stimulation-induced blindsight. Proc Natl Acad Sci U S A. 2004;101(26):9933-5. Epub 2004/06/24. doi: 10.1073/pnas.0403061101. PubMed PMID: 15210947; PubMed Central PMCID: PMCPMC470776.

485. Rollnik JD, Schröder C, Rodríguez-Fornells A, Kurzbuch AR, Däuper J, Möller J, et al. Functional lesions and human action monitoring: Combining repetitive transcranial magnetic stimulation and event-related brain potentials. Clinical Neurophysiology. 2004;115(1):145-53. doi: 10.1016/j.clinph.2003.05.001.

486. Spence C, Pavani F, Maravita A, Holmes N. Multisensory contributions to the 3-D representation of visuotactile peripersonal space in humans: Evidence from the crossmodal congruency task. Journal of Physiology Paris. 2004;98(1-3 SPEC. ISS.):171-89. doi: 10.1016/j.jphysparis.2004.03.008.

487. Staudt M, Gerloff C, Grodd W, Holthausen H, Niemann G, Krägeloh-Mann I. Reorganization in congenital hemiparesis acquired at different gestational ages. Annals of Neurology. 2004;56(6):854-63. doi: 10.1002/ana.20297.

488. Stinear JW, Byblow WD. Modulation of human cervical premotoneurons during bilateral voluntary contraction of upper-limb muscles. Muscle and Nerve. 2004;29(4):506-14. doi: 10.1002/mus.20003.

489. Strafella AP, Vanderwerf Y, Sadikot AF. Transcranial magnetic stimulation of the human motor cortex influences the neuronal activity of subthalamic nucleus. European Journal of Neuroscience. 2004;20(8):2245-9. doi: 10.1111/j.1460-9568.2004.03669.x.

490. Suleiman Y, Amm M, Duncker GI, Nölle B. [Prognosis of corneal transplantation after penetrating eye injury]. Klin Monbl Augenheilkd. 2004;221(8):658-73. Epub 2004/09/03. doi: 10.1055/s-2004-813536. PubMed PMID: 15343450.

491. Takayama K, Kawai N, Kawanishi M, Masada T, Jinnai T,

Matsumoto Y, et al. Effects of Transcranial Magnetic Stimulation on Patients with Prolonged Consciousness Disturbance. Japanese Journal of Neurosurgery. 2004;13(1):32-8. doi: 10.7887/jcns.13.32.

492. Torbey MT, Geocadin R, Bhardwaj A. Brain arrest neurological outcome scale (BrANOS): Predicting mortality and severe disability following cardiac arrest. Resuscitation. 2004;63(1):55-63. doi: 10.1016/j.resuscitation.2004.03.021.

493. Wagner T, Gangitano M, Romero R, Théoret H, Kobayashi M, Anschel D, et al. Intracranial measurement of current densities induced by transcranial magnetic stimulation in the human brain. Neurosci Lett. 2004;354(2):91-4. Epub 2003/12/31. doi: 10.1016/s0304-3940(03)00861-9. PubMed PMID: 14698446.

494. Ward NS, Frackowiak RSJ. Towards a new mapping of brain cortex function. Cerebrovascular Diseases. 2004;17(SUPPL. 3):35-8. doi: 10.1159/000075303.

495. Weddell RA. Subcortical modulation of spatial attention including evidence that the Sprague effect extends to man. Brain and Cognition. 2004;55(3):497-506. doi: 10.1016/j.bandc.2004.02.075.

496. White PF, Tang J, Ma H, Wender RH, Sloninsky A, Kariger R. Is the patient state analyzer with the PSArray2 a cost-effective alternative to the bispectral index monitor during the perioperative period? Anesthesia and Analgesia. 2004;99(5):1429-35. doi:

10.1213/01.ANE.0000132784.57622.CC.

497. Xu JJ, Wei ZH, Yang LM, Zhang XJ, Diao HM, Li YT, et al. Effect of median nerve electrical stimulation against vegetative state of patients after craniocerebral injury. Chinese journal of clinical rehabilitation. 2004;8(28):6040-3. PubMed PMID: CN-01708745.

498. Xu P, Wang Z, Wu Y. Primary Study on Median Nerve Stimulation Therapy in Improving the Level of Consciousness of Patients in Coma Caused by head Traumas. Suzhou University Journal of Medical Science. 2004;24(2):199-202. PubMed PMID: CSCD:1624092.

499. Yago E, Duarte A, Wong T, Barceló F, Knight RT. Temporal kinetics of prefrontal modulation of the extrastriate cortex during visual attention. Cognitive, Affective and Behavioral Neuroscience. 2004;4(4):609-17. doi: 10.3758/CABN.4.4.609.

500. Yamashita M, Horikoshi S, Asanuma K, Takahara H, Shirato I, Tomino Y. Tensin is potentially involved in extracellular matrix production in mesangial cells. Histochem Cell Biol. 2004;121(3):245-54. Epub 2004/03/03. doi: 10.1007/s00418-004-0626-8. PubMed PMID: 14991332.

501. Young RL, Ridding MC, Morrell TL. Switching skills on by turning off part of the brain. Neurocase. 2004;10(3):215-22. doi: 10.1080/13554790490495140.

502. Zhao Z, Alam S, Oppenheim RW, Prevette DM, Evenson A, Parsadanian A. Overexpression of glial cell line-derived neurotrophic factor in the CNS rescues motoneurons from programmed cell death and promotes their long-term survival following axotomy. Exp Neurol. 2004;190(2):356-72. Epub 2004/11/09. doi: 10.1016/j.expneurol.2004.06.015. PubMed PMID: 15530875.
503. Zito G, Mattia D, Tomaiuolo F, Oliveri M, Castriota-Scanderbeg A, Caltagirone C, et al. Functional anatomy of motor recovery after early brain damage. Neurocase. 2004;10(3):265-9. Epub 2005/03/25. doi: 10.1080/13554790490495212. PubMed PMID: 15788263.
504. Left and right parietal lobes: Different roles in mental imagery and neglect. Neuroscientist. 2005;11(4):271. doi: 10.1177/107385840501100403.
505. Abbott KC, Bernet VJ, Agodoa LY, Yuan CM. Differing manifestations of hepatitis C and tacrolimus on hospitalized diabetes mellitus occurring after kidney transplantation. Annals of Epidemiology. 2005;15(8):558-63. doi: 10.1016/j.annepidem.2004.10.003.
506. Aoyagi M. [Accuracy of the prognostic diagnosis in acute peripheral facial palsy]. Nihon Jibiinkoka Gakkai Kaiho. 2005;108(1):1-7. Epub 2005/02/17. doi: 10.3950/jibiinkoka.108.1.

PubMed PMID: 15712490.

507. Berkowitsch A, Greiss H, Vukajlovic D, Kuniss M, Neumann T, Zaltsberg S, et al. Usefulness of atrial fibrillation burden as a predictor for success of pulmonary vein isolation. PACE - Pacing and Clinical Electrophysiology. 2005;28(12):1292-301. doi: 10.1111/j.1540-8159.2005.00270.x.

508. Beuzeron-Mangina JH, Fotiou F. Editorial preface. International Journal of Psychophysiology. 2005;58(2-3 SPEC. ISS.):109-10. doi: 10.1016/j.ijpsycho.2005.07.002.

509. Bogousslavsky J. Artistic creativity, style and brain disorders. European Neurology. 2005;54(2):103-11. doi: 10.1159/000088645.

510. Bojinova V, Dimova P, Gergueltcheva V, Petkov R, Belopitova L, Yankov T. Peculiarities of multiple sclerosis in childhood - Frequency, clinical characteristics, diagnosis, prognosis, treatment. Pediatriya. 2005;45(4):21-5+3-4.

511.Carter OL, Pettigrew JD, Hasler F, Wallis GM, Liu GB, Hell D, et al. Modulating the rate and rhythmicity of perceptual rivalry alternations with the mixed 5-HT2A and 5-HT1A agonist psilocybin. Neuropsychopharmacology. 2005;30(6):1154-62. doi: 10.1038/sj.npp.1300621.

512. Centonze D, Palmieri MG, Boffa L, Pierantozzi M, Stanzione P, Brusa L, et al. Cortical hyperexcitability in post-traumatic stress

disorder secondary to minor accidental head trauma: a neurophysiologic study. J Psychiatry Neurosci. 2005;30(2):127-32. Epub 2005/03/31. PubMed PMID: 15798788; PubMed Central PMCID: PMCPMC551170.

513. Cooper EB, Scherder EJ, Cooper JB. Electrical treatment of reduced consciousness: experience with coma and Alzheimer's disease. Neuropsychol Rehabil. 2005;15(3-4):389-405. Epub 2005/12/15. doi: 10.1080/09602010443000317. PubMed PMID: 16350980.

514. Crossley M, Shiel A, Wilson B, Coleman MR, Gelling L, Fryer T, et al. Monitoring emergence from coma following severe brain injury in an octogenarian using behavioural indicators, electrophysiological measures and metabolic studies: a demonstration of the potential for good recovery in older adults. Brain Inj. 2005;19(9):729-37. Epub 2005/10/01. doi: 10.1080/02699050400013733. PubMed PMID: 16195187.

515. Fagade OO, Oginni FO, Obilade TO. Comparative study of the therapeutic effect of a systemic analgesic and transcutaneous electrical nerve stimulation (TENS) on post-IMF trismus and pain in Nigerian patients. Niger Postgrad Med J. 2005;12(2):97-101. Epub 2005/07/06. PubMed PMID: 15997257.

516. Fins JJ. Clinical pragmatism and the care of brain damaged patients: toward a palliative neuroethics for disorders of consciousness.

In: Laureys S, editor. Boundaries of Consciousness: Neurobiology and Neuropathology. Progress in Brain Research. 1502005. p. 565-82.

517. Foley DP, Fernandez LA, Leverson G, Chin LT, Krieger N, Cooper JT, et al. Donation after cardiac death: the University of Wisconsin experience with liver transplantation. Ann Surg. 2005;242(5):724-31. Epub 2005/10/26. doi: 10.1097/01.sla.0000186178.07110.92. PubMed PMID: 16244547; PubMed Central PMCID: PMCPMC1409855.

518. Fregni F, Pascual-Leone A, Valente KDR. A therapeutic window for transcranial magnetic stimulation in refractory epilepsy. Journal of Epilepsy and Clinical Neurophysiology. 2005;11(4):177-81.

519. Gallegos DR, Tranel D. Positive facial affect facilitates the identification of famous faces. Brain and Language. 2005;93(3):338-48. doi: 10.1016/j.bandl.2004.11.001.

520. Hammoud DA, Endres CJ, Chander AR, Guilarte TR, Wong DF, Sacktor NC, et al. Imaging glial cell activation with C-11 -R-PK11195 in patients with AIDS. Journal of Neurovirology. 2005;11(4):346-55. doi: 10.1080/13550280500187351. PubMed PMID: WOS:000231877000004.

521. Hammoud DA, Endres CJ, Chander AR, Guilarte TR, Wong DF, Sacktor NC, et al. Imaging glial cell activation with [11C]-R-PK11195 in patients with AIDS. Journal of NeuroVirology.

2005;11(4):346-55. doi: 10.1080/13550280500187351.

522. He XK, You CJ, Wang YF, Ai ZB. Effects of low-frequency repetitive transcranial magnetic stimulation at different intension on praxiology of Parkinson rats. Chinese Journal of Clinical Rehabilitation. 2005;9(41):11-3.

523. Hiraoka T, Matsumoto Y, Okamoto F, Yamaguchi T, Hirohara Y, Mihashi T, et al. Corneal higher-order aberrations induced by overnight orthokeratology. Am J Ophthalmol. 2005;139(3):429-36. Epub 2005/03/16. doi: 10.1016/j.ajo.2004.10.006. PubMed PMID: 15767050.

524. Hirshberg LM, Chiu S, Frazier JA. Emerging brain-based interventions for children and adolescents: overview and clinical perspective. Child Adolesc Psychiatr Clin N Am. 2005;14(1):1-19, v. Epub 2004/11/27. doi: 10.1016/j.chc.2004.07.011. PubMed PMID: 15564050.

525. Hjortdal J, Møller-Pedersen T, Ivarsen A, Ehlers N. Corneal power, thickness, and stiffness: results of a prospective randomized controlled trial of PRK and LASIK for myopia. J Cataract Refract Surg. 2005;31(1):21-9. Epub 2005/02/22. doi: 10.1016/j.jcrs.2004.09.043. PubMed PMID: 15721693.

526. Hjortdal O, MÃ¸ller-Pedersen T, Ivarsen A. Biomechanical Changes in the Human Cornea After PRK and LASIK. IOVS.

2005;46:ARVO E-abstract 2735. PubMed PMID: CN-00599072.

527. Jafri B, Li X, Yang H, Rabinowitz YS. Higher order wavefront aberrations in early keratoconus and keratoconus suspects. Investigative Ophthalmology & Visual Science. 2005;46. PubMed PMID: WOS:000227980405263.

528. Jang SH, Cho SH, Kim YH, You SH, Kim SH, Kim O, et al. Motor recovery mechanism of diffuse axonal injury: a combined study of transcranial magnetic stimulation and functional MRI. Restor Neurol Neurosci. 2005;23(1):51-6. Epub 2005/04/23. PubMed PMID: 15846032.

529. Karaca K, Etöz A, Kahveci R. Facial nerve identification using a disposable nerve block needle as a nerve stimulator. Plast Reconstr Surg. 2005;115(2):667-8. Epub 2005/02/05. doi: 10.1097/01.prs.0000149062.45157.9b. PubMed PMID: 15692393.

530. Khopade AJ, Arulsudar N, Khopade SA, Hartmann J. Ultrathin antibiotic walled microcapsules. Biomacromolecules. 2005;6(1):229-34. Epub 2005/01/11. doi: 10.1021/bm049554a. PubMed PMID: 15638525.

531. Knight CA. Applications of the H reflex and transcranial magnetic stimulation. Athletic Therapy Today. 2005;10(3):22-4. doi: 10.1123/att.10.3.22.

532. Kovacic P, Cooksy A. Iminium metabolite mechanism for

nicotine toxicity and addiction: Oxidative stress and electron transfer. Medical Hypotheses. 2005;64(1):104-11. doi: 10.1016/j.mehy.2004.03.048.

533. Lee J, Oh J-K, 이현정, 김호성, 권기욱. Preventive effects of swimming training in cold water on cerebral infarct volume and motor functions in MCAo rats. Exercise Science. 2005;14(3):417-28. PubMed PMID: KJD:ART001137123.

534. Li C, Maier DL, Cross B, Doherty JJ, Christian EP. Fimbria-fornix lesions compromise the induction of long-term potentiation at the Schaffer collateral-CA1 synapse in the rat in vivo. J Neurophysiol. 2005;93(5):3001-6. Epub 2005/04/23. doi: 10.1152/jn.00546.2004. PubMed PMID: 15846002.

535. Liepert J, Restemeyer C, Münchau A, Weiller C. Motor cortex excitability after thalamic infarction. Clinical Neurophysiology. 2005;116(7):1621-7. doi: 10.1016/j.clinph.2005.03.002.

536. Lobato RD, Alen JF, Perez-Nuñez A, Alday R, Gómez PA, Pascual B, et al. Value of serial CT scanning and intracranial pressure monitoring for detecting new intracranial mass effect in severe head injury patients showing lesions type I-II in the initial CT scan. Neurocirugia. 2005;16(3):217-34. doi: 10.1016/s1130-1473(05)70405-x.

537. Manconi M, Sferraza B, Iannaccone S, Massimo A, Zucconi

M, Ferini-Strambi L. Case of symptomatic propriospinal myoclonus evolving toward acute "myoclonic status". Movement Disorders. 2005;20(12):1646-50. doi: 10.1002/mds.20645.

538. Mansur CG, Fregni F, Boggio PS, Riberto M, Gallucci-Neto J, Santos CM, et al. A sham stimulation-controlled trial of rTMS of the unaffected hemisphere in stroke patients. Neurology. 2005;64(10):1802-4. doi: 10.1212/01.WNL.0000161839.38079.92.

539. Mazarr-Proo S, Kerrigan S. Distribution of GHB in tissues and fluids following a fatal overdose. J Anal Toxicol. 2005;29(5):398-400. Epub 2005/08/18. doi: 10.1093/jat/29.5.398. PubMed PMID: 16105269.

540. Murase N, Rothwell JC, Kaji R, Urushihara R, Nakamura K, Murayama N, et al. Subthreshold low-frequency repetitive transcranial magnetic stimulation over the premotor cortex modulates writer's cramp. Brain. 2005;128(1):104-15. doi: 10.1093/brain/awh315.

541. Naeser MA, Martin PI, Nicholas M, Baker EH, Seekins H, Kobayashi M, et al. Improved picture naming in chronic aphasia after TMS to part of right Broca's area: An open-protocol study. Brain and Language. 2005;93(1):95-105. doi: 10.1016/j.bandl.2004.08.004.

542. Nct. Moving a Paralyzed Hand Through Use of a Brain-Computer Interface. https://clinicaltrialsgov/show/NCT00242242. 2005. PubMed PMID:

CN-01582520.

543. Nct. A Double-Blind Placebo Controlled Trial of Repetitive Transcranial Magnetic Stimulation (rTMS) in the Treatment of Negative Symptoms in Schizophrenia. https://clinicaltrialsgov/show/NCT00168311. 2005. PubMed PMID: CN-01478111.

544. Nct. A Randomised Double-Blind Trial of Targeted Repetitive Transcranial Magnetic Stimulation in Major Depressive Disorder. https://clinicaltrialsgov/show/NCT00168376. 2005. PubMed PMID: CN-02013652.

545. Nct. Priming rTMS In Major Depression. https://clinicaltrialsgov/show/NCT00168272. 2005. PubMed PMID: CN-01478109.

546. Nitsche MA, Liebetanz D, Paulus W, Tergau F. Pharmacological characterisation and modulation of neuroplasticity in humans. Current Neuropharmacology. 2005;3(3):217-29. doi: 10.2174/1570159054368268.

547. Pascual-Leone A, Amedi A, Fregni F, Merabet LB. The plastic human brain cortex. Annual Review of Neuroscience. 2005;28:377-401. doi: 10.1146/annurev.neuro.27.070203.144216. PubMed PMID: WOS:000231235700015.

548. Pickard JD. Part II: Functional imaging, electrophysiology and

mechanical intervention - Foreword. Neuropsychological Rehabilitation. 2005;15(3-4):272-5. doi: 10.1080/09602010443000641.

549. Rektorova I, Megova S, Bares M, Rektor I. Cognitive functioning after repetitive transcranial magnetic stimulation in patients with cerebrovascular disease without dementia: A pilot study of seven patients. Journal of the Neurological Sciences. 2005;229-230:157-61. doi: 10.1016/j.jns.2004.11.021.

550. Rex E, Hapiak V, Hobson R, Smith K, Xiao H, Komuniecki R. TYRA-2 (F01E11.5): A Caenorhabditis elegans tyramine receptor expressed in the MC and NSM pharyngeal neurons. Journal of Neurochemistry. 2005;94(1):181-91. doi: 10.1111/j.1471-4159.2005.03180.x.

551. Ribeiro JA. What can Adenosine Neuromodulation do for Neuroprotection? Cns & Neurological Disorders-Drug Targets. 2005;4(4):325-9. doi: 10.2174/1568007054546090. PubMed PMID: WOS:000497842800003.

552. Rusina R, Vaculin S, Yamamotova A, Barek S, Dvorakova H, Rokyta R. The effect of motor cortex stimulation in deafferentated rats. Neuroendocrinology Letters. 2005;26(4):283-8. PubMed PMID: WOS:000233233000002.

553. Seitz RJ, Kleiser R, Bütefisch CM. Reorganization of cerebral

circuits in human brain lesion. Acta Neurochirurgica, Supplementum2005. p. 65-70.

554. Senapati AK, Huntington PJ, Peng YB. Spinal dorsal horn neuron response to mechanical stimuli is decreased by electrical stimulation of the primary motor cortex. Brain Research. 2005;1036(1-2):173-9. doi: 10.1016/j.brainres.2004.12.043. PubMed PMID: WOS:000227577400021.

555. Singh AN. Multidisciplinary management of chronic pain. International Medical Journal. 2005;12(2):111-6.

556. Staudt M, Krägeloh-Mann I, Grodd W. Ipsilateral corticospinal pathways in congenital hemiparesis on routine magnetic resonance imaging. Pediatric Neurology. 2005;32(1):37-9. doi: 10.1016/j.pediatrneurol.2004.07.005.

557. Stefan K, Cohen LG, Duque J, Mazzocchio R, Celnik P, Sawaki L, et al. Formation of a motor memory by action observation. Journal of Neuroscience. 2005;25(41):9339-46. doi: 10.1523/JNEUROSCI.2282-05.2005.

558. Strafella AP, Ko JH, Grant J, Fraraccio M, Monchi O. Corticostriatal functional interactions in Parkinson's disease: A rTMS/[11C]raclopride PET study. European Journal of Neuroscience. 2005;22(11):2946-52. doi: 10.1111/j.1460-9568.2005.04476.x.

559. Takahashi CD, Der Yeghiaian L, Cramer SC. Stroke recovery

and its imaging. Neuroimaging Clinics of North America. 2005;15(3):681-+. doi: 10.1016/j.nic.2005.08.006. PubMed PMID: WOS:000234303800015.

560. Tyrer S, Davis E. Central pain states: Etiology and management in rehabilitation. Critical Reviews in Physical and Rehabilitation Medicine. 2005;17(2):131-62. doi: 10.1615/CritRevPhysRehabilMed.v17.i2.30.

561. Viveros MP, Marco EM, File SE. Endocannabinoid system and stress and anxiety responses. Pharmacology Biochemistry and Behavior. 2005;81(2 SPEC. ISS.):331-42. doi: 10.1016/j.pbb.2005.01.029.

562. Voets NL, Matthews PM. Clinical applications of functional magnetic resonance imaging. Imaging Decisions MRI. 2005;9(1):16-22. doi: 10.1111/j.1617-0830.2005.00037.x.

563. Walker N, Michaud K, Wolfe F. Work limitations among working persons with rheumatoid arthritis: results, reliability, and validity of the work limitations questionnaire in 836 patients. J Rheumatol. 2005;32(6):1006-12. Epub 2005/06/09. PubMed PMID: 15940759.

564. Ward NS. Mechanisms underlying recovery of motor function after stroke. Postgrad Med J. 2005;81(958):510-4. Epub 2005/08/09. doi: 10.1136/pgmj.2004.030809. PubMed PMID: 16085742; PubMed

Central PMCID: PMCPMC1743338.

565. Ward NS. Plasticity and the functional reorganization of the human brain. International Journal of Psychophysiology. 2005;58(2-3 SPEC. ISS.):158-61. doi: 10.1016/j.ijpsycho.2005.02.009.

566. Yates BJ, Bronstein AM. The effects of vestibular system lesions on autonomic regulation: Observations, mechanisms, and clinical implications. Journal of Vestibular Research: Equilibrium and Orientation. 2005;15(3):119-29.

567. André-Obadia N, Peyron R, Mertens P, Mauguière F, Laurent B, Garcia-Larrea L. Transcranial magnetic stimulation for pain control. Double-blind study of different frequencies against placebo, and correlation with motor cortex stimulation efficacy. Clin Neurophysiol. 2006;117(7):1536-44. Epub 2006/06/07. doi: 10.1016/j.clinph.2006.03.025. PubMed PMID: 16753335.

568. Aydin-Abidin S, Moliadze V, Eysel UT, Funke K. Effects of repetitive TMS on visually evoked potentials and EEG in the anaesthetized cat: dependence on stimulus frequency and train duration. J Physiol. 2006;574(Pt 2):443-55. Epub 2006/05/13. doi: 10.1113/jphysiol.2006.108464. PubMed PMID: 16690713; PubMed Central PMCID: PMCPMC1817766.

569. Barba C, Formisano R, Sabatini U, Cicinelli P, Elisabeth Hagberg G, Marconi B, et al. Dysfunction of a structurally normal

motor pathway in a brain injury patient as revealed by multimodal integrated techniques. Neurocase. 2006;12(4):232-5. Epub 2006/09/27. doi: 10.1080/13554790600878879. PubMed PMID: 17000593.

570. Basso D, Lotze M, Vitale L, Ferreri F, Bisiacchi P, Olivetti Belardinelli M, et al. The role of prefrontal cortex in visuo-spatial planning: A repetitive TMS study. Exp Brain Res. 2006;171(3):411-5. Epub 2006/05/20. doi: 10.1007/s00221-006-0457-z. PubMed PMID: 16710684.

571. Bütefisch CM. Neurobiological bases of rehabilitation. Neurol Sci. 2006;27 Suppl 1:S18-23. Epub 2006/05/19. doi: 10.1007/s10072-006-0540-z. PubMed PMID: 16708176.

572. Carden P. Managed communication services architecture framework. Alcatel Telecommunications Review. 2006(1):67-72. PubMed PMID: WOS:000236508500016.

573. Chemerinski E, Levine SR. Neuropsychiatric disorders following vascular brain injury. Mount Sinai Journal of Medicine. 2006;73(7):1006-14.

574. Chokron S. Contributions of functional neuroimaging to clinical and cognitive neuropsychology: A selective review. Current Medical Imaging Reviews. 2006;2(1):59-69. doi: 10.2174/157340506775541703.

575. Cohen Kadosh R, Walsh V. Cognitive neuroscience: rewired

or crosswired brains? Curr Biol. 2006;16(22):R962-3. Epub 2006/11/23. doi: 10.1016/j.cub.2006.10.017. PubMed PMID: 17113378.

576. Čumlivski R, Redl G, Strobl W, Girsch W, Krebs A, Machowetz P. Pharmacological neuromodulation of the spasticity by using of intrathecal applied baclofen in the children. Bolest. 2006;9(2):88-98.

577. Duffau H. Brain plasticity: from pathophysiological mechanisms to therapeutic applications. J Clin Neurosci. 2006;13(9):885-97. Epub 2006/10/20. doi: 10.1016/j.jocn.2005.11.045. PubMed PMID: 17049865.

578. Durazzo TC, Gazdzinski S, Rothlind JC, Banys P, Meyerhoff DJ. Brain metabolite concentrations and neurocognition during short-term recovery from alcohol dependence: Preliminary evidence of the effects of concurrent chronic cigarette smoking. Alcoholism: Clinical and Experimental Research. 2006;30(3):539-51. doi: 10.1111/j.1530-0277.2006.00060.x.

579. Epstein CM. Seizure or convulsive syncope during 1-Hz rTMS? Clin Neurophysiol. 2006;117(11):2566-7; author reply 7-8. Epub 2006/09/26. doi: 10.1016/j.clinph.2006.07.262. PubMed PMID: 16996304.

580. Fregni F, Boggio PS, Lima MC, Ferreira MJ, Wagner T,

Rigonatti SP, et al. A sham-controlled, phase II trial of transcranial direct current stimulation for the treatment of central pain in traumatic spinal cord injury. Pain. 2006;122(1-2):197-209. doi: 10.1016/j.pain.2006.02.023. PubMed PMID: CN-00563646.

581. Fujiki M, Hikawa T, Abe T, Ishii K, Kobayashi H. Reduced short latency afferent inhibition in diffuse axonal injury patients with memory impairment. Neurosci Lett. 2006;405(3):226-30. Epub 2006/08/12. doi: 10.1016/j.neulet.2006.07.005. PubMed PMID: 16901641.

582. Galat A. Involvement of some large immunophilins and their ligands in the protection and regeneration of neurons: A hypothetical mode of action. Computational Biology and Chemistry. 2006;30(5):348-59. doi: 10.1016/j.compbiolchem.2006.08.001.

583. Giacino JT, Hirsch J, Schiff N, Laureys S. Functional neuroimaging applications for assessment and rehabilitation planning in patients with disorders of consciousness. Arch Phys Med Rehabil. 2006;87(12 Suppl 2):S67-76. Epub 2006/12/05. doi: 10.1016/j.apmr.2006.07.272. PubMed PMID: 17140882.

584. Hirai S, Cureoglu S, Schachern PA, Hayashi H, Paparella MM, Harada T. Large vestibular aqueduct syndrome: a human temporal bone study. Laryngoscope. 2006;116(11):2007-11. Epub 2006/11/01. doi: 10.1097/01.mlg.0000237673.94781.0a. PubMed PMID:

17075417.
585. Ichinose M, Saito M, Fujii N, Kondo N, Nishiyasu T. Modulation of the control of muscle sympathetic nerve activity during severe orthostatic stress. Journal of Physiology. 2006;576(3):947-58. doi: 10.1113/jphysiol.2006.117507.
586. Ivins BJ, Schwab KA, Baker G, Warden DL. Hospital admissions associated with traumatic brain injury in the US army during peacetime: 1990s Trends. Neuroepidemiology. 2006;27(3):154-63. doi: 10.1159/000096127. PubMed PMID: WOS:000241773900006.
587. Kao CD, Guo WY, Chen JT, Wu ZA, Liao KK. MR findings of decerebrate rigidity with preservation of consciousness. American Journal of Neuroradiology. 2006;27(5):1074-5.
588. Karnath HO, Dieterich M. Spatial neglect--a vestibular disorder? Brain. 2006;129(Pt 2):293-305. Epub 2005/12/24. doi: 10.1093/brain/awh698. PubMed PMID: 16371409.
589. Karnath HO, Dieterich M. Spatial neglect - a vestibular disorder? Brain. 2006;129:293-305. doi: 10.1093/brain/awh698. PubMed PMID: WOS:000234965500004.
590. Koethe D, Gerth CW, Neatby MA, Haensel A, Thies M, Schneider U, et al. Disturbances of visual information processing in early states of psychosis and experimental

delta-9-tetrahydrocannabinol altered states of consciousness. Schizophrenia Research. 2006;88(1-3):142-50. doi: 10.1016/j.schres.2006.07.023.

591. Kofler M. Levetiracetam suppresses long-loop reflexes at the cortical level. Muscle Nerve. 2006;33(6):785-91. Epub 2006/03/07. doi: 10.1002/mus.20533. PubMed PMID: 16518861.

592. Laganaro M, Di Pietro M, Schnider A. What does recovery from anomia tell us about the underlying impairment: The case of similar anomic patterns and different recovery. Neuropsychologia. 2006;44(4):534-45. doi: 10.1016/j.neuropsychologia.2005.07.005.

593. Lazar RM. Why does neuropsychology need a review journal? An editorial. Neuropsychology Review. 2006;16(1):1-2. doi: 10.1007/s11065-006-9004-8. PubMed PMID: WOS:000239015200001.

594. Lefaucheur JP. New insights into the therapeutic potential of non-invasive transcranial cortical stimulation in chronic neuropathic pain. Pain. 2006;122(1-2):11-3. doi: 10.1016/j.pain.2006.02.024. PubMed PMID: WOS:000237549100005.

595. Lippert-Grüner M, Švestková O, Angerová Y, Brtnická P, Pfeiffer J. Mechanisms of neuroplasticity and its influence on rehabilitation of patients in case of brain injuries. Eurorehab. 2006;16(3-4):78-83.

596. Lomarev MP, Kanchana S, Bara-Jimenez W, Iyer M, Wassermann EM, Hallett M. Placebo-controlled study of rTMS for the treatment of Parkinson's disease. Movement Disorders. 2006;21(3):325-31. doi: 10.1002/mds.20713.

597. MacDonald DB. Safety of transcranial, cortical and subcortical electrical stimulation using different neurophysiological techniques. Rivista Medica. 2006;12(1-2):39-43.

598. Meinzer M, Flaisch T, Obleser J, Assadollahi R, Djundja D, Barthel G, et al. Brain regions essential for improved lexical access in an aged aphasic patient: A case report. BMC Neurology. 2006;6. doi: 10.1186/1471-2377-6-28.

599. Melander C, Adden R, Brinkmalm G, Gorton L, Mischnick P. New approaches to the analysis of enzymatically hydrolyzed methyl cellulose. Part 2. Comparison of various enzyme preparations. Biomacromolecules. 2006;7(5):1410-21. doi: 10.1021/bm0509422. PubMed PMID: WOS:000237593600006.

600. Nct. Long-Term Improvement in Motor Learning by Transcranial Direct Current Stimulation. https://clinicaltrialsgov/show/NCT00314769. 2006. PubMed PMID: CN-01481637.

601. Neto FC, De Oliveira E, Paschoal E, Cendes F, Santana Filho M. Morphological characteristics from the insula's lobe in patients

with medial temporal lobe epilepsy. Arquivos de Neuro-Psiquiatria. 2006;64(3 A).

602. Nowak DA, Connemann BJ. Reply to "Seizure or convulsive syncope during 1-Hz rTMS?". Clinical Neurophysiology. 2006;117(11):2567-8. doi: 10.1016/j.clinph.2006.07.263. PubMed PMID: WOS:000242249800023.

603. Nyffeler T, Müri R, Pflugshaupt T, Wartburg R, Hess CW. Cortical reorganization after brain damage: the oculomotor model. Eur J Neurosci. 2006;23(5):1397-402. Epub 2006/03/24. doi: 10.1111/j.1460-9568.2006.04648.x. PubMed PMID: 16553801.

604. Osenbach RK. Motor cortex stimulation for intractable pain. Neurosurg Focus. 2006;21(6):E7. Epub 2007/03/08. doi: 10.3171/foc.2006.21.6.12. PubMed PMID: 17341051.

605. Pape TL, Rosenow J, Lewis G. Transcranial magnetic stimulation: a possible treatment for TBI. J Head Trauma Rehabil. 2006;21(5):437-51. Epub 2006/09/20. doi: 10.1097/00001199-200609000-00063. PubMed PMID: 16983227.

606. Parsadanian A, Pan Y, Li W, Myckatyn TM, Brakefield D. Astrocyte-derived transgene GDNF promotes complete and long-term survival of adult facial motoneurons following avulsion and differentially regulates the expression of transcription factors of AP-1 and ATF/CREB families. Exp Neurol. 2006;200(1):26-37. Epub

2006/02/25. doi: 10.1016/j.expneurol.2006.01.014. PubMed PMID: 16497298.

607. Pascual-Leone A. Disrupting the brain to guide plasticity and improve behavior. In: Moller AR, editor. Reprogramming the Brain. Progress in Brain Research. 1572006. p. 315-+.

608. Peers PV, Cusack R, Duncan J. Modulation of spatial bias in the dual task paradigm: Evidence from patients with unilateral parietal lesions and controls. Neuropsychologia. 2006;44(8):1325-35. doi: 10.1016/j.neuropsychologia.2006.01.033.

609. Perlstein WM, Larson MJ, Dotson VM, Kelly KG. Temporal dissociation of components of cognitive control dysfunction in severe TBI: ERPs and the cued-Stroop task. Neuropsychologia. 2006;44(2):260-74. doi: 10.1016/j.neuropsychologia.2005.05.009.

610. Platz T, Liepert J, Seitz RJ, Bütefisch CM, Müller K, Suchan B, et al. Motor system recovery after brain damage - Mechanisms of recovery and development of restorative therapy - Initiatives and results from the rehabilitation subnet of the German competence net stroke. Nervenheilkunde. 2006;25(11):933-40. doi: 10.1055/s-0038-1626802.

611.Rafal RD. Oculomotor functions of the parietal lobe: Effects of chronic lesions in humans. Cortex. 2006;42(5):730-9. doi: 10.1016/s0010-9452(08)70411-8. PubMed PMID:

WOS:000239547500010.

612. Rais D, Nowack B, Schulin R, Luster J. Sorption of trace metals by standard and micro suction cups in the absence and presence of dissolved organic carbon. J Environ Qual. 2006;35(1):50-60. Epub 2006/01/05. doi: 10.2134/jeq2005.0040. PubMed PMID: 16391276.

613. Robakis TK, Hirsch LJ. Literature review, case report, and expert discussion of prolonged refractory status epilepticus. Neurocrit Care. 2006;4(1):35-46. Epub 2006/02/25. doi: 10.1385/ncc:4:1:035. PubMed PMID: 16498194.

614. Saitoh Y, Hirayama A, Kishima H, Oshino S, Hirata M, Kato A, et al. Stimulation of primary motor cortex for intractable deafferentation pain. Acta Neurochir Suppl. 2006;99:57-9. Epub 2007/03/21. doi: 10.1007/978-3-211-35205-2_11. PubMed PMID: 17370765.

615. Sedmak B, Elersek T. Microcystins induce morphological and physiological changes in selected representative phytoplanktons. Microbial Ecology. 2006;51(4):508-15. doi: 10.1007/s00248-006-9045-9. PubMed PMID: WOS:000238520800011.

616. Sharova EV, Mel'nikov AV, Novikova MR, Kulikov MA, Grechenko TN, Shekhter ED, et al. [Changes in the brain spontaneous bioelectrical activity during transcranial electrical and electromagnetic

stimulation]. Zh Vyssh Nerv Deiat Im I P Pavlova. 2006;56(3):363-70. Epub 2006/07/28. PubMed PMID: 16869271.

617. Shields CB, Ping Zhang Y, Shields LB, Burke DA, Glassman SD. Objective assessment of cervical spinal cord injury levels by transcranial magnetic motor-evoked potentials. Surg Neurol. 2006;66(5):475-83; discussion 83. Epub 2006/11/07. doi: 10.1016/j.surneu.2006.04.009. PubMed PMID: 17084191.

618. Stein RB, Chong S, Everaert DG, Rolf R, Thompson AK, Whittaker M, et al. A multicenter trial of a footdrop stimulator controlled by a tilt sensor. Neurorehabilitation and Neural Repair. 2006;20(3):371-9. doi: 10.1177/1545968306289292.

619. Šupe S, Matijević V, Kondić L, Alvir D. Series of seizures as a sign of development of recurrent malignant neuroleptic syndrome - A case report. Psychiatria Danubina. 2006;18(1-2):97-101.

620. Takata K, Yamauchi H, Tatsuno H, Hashimoto K, Abo M. Is the ipsilateral cortex surrounding the lesion or the non-injured contralateral cortex important for motor recovery in rats with photochemically induced cortical lesions? Eur Neurol. 2006;56(2):106-12. Epub 2006/09/09. doi: 10.1159/000095700. PubMed PMID: 16960450.

621. Takeuchi N, Ikoma K, Chuma T, Matsuo Y. Measurement of transcallosal inhibition in traumatic brain injury by transcranial

magnetic stimulation. Brain Inj. 2006;20(9):991-6. Epub 2006/10/19. doi: 10.1080/02699050600909771. PubMed PMID: 17046801.

622. Terhoeven-Urselmans T, Michel K, Helfrich M, Flessa H, Ludwig B. Near-infrared spectroscopy can predict the composition of organic matter in soil and litter. Journal of Plant Nutrition and Soil Science. 2006;169(2):168-74. doi: 10.1002/jpln.200521712. PubMed PMID: WOS:000236991000003.

623. Thiel A, Schumacher B, Wienhard K, Gairing S, Kracht LW, Wagner R, et al. Direct demonstration of transcallosal disinhibition in language networks. Journal of Cerebral Blood Flow and Metabolism. 2006;26(9):1122-7. doi: 10.1038/sj.jcbfm.9600350.

624. Tyvaert L, Houdayer E, Devanne H, Monaca C, Cassim F, Derambure P. The effect of repetitive transcranial magnetic stimulation on dystonia: a clinical and pathophysiological approach. Neurophysiologie Clinique. 2006;36(3):135-43. doi: 10.1016/j.neucli.2006.08.007.

625. Urushihara R, Murase N, Rothwell JC, Harada M, Hosono Y, Asanuma K, et al. Effect of repetitive transcranial magnetic stimulation applied over the premotor cortex on somatosensory-evoked potentials and regional cerebral blood flow. NeuroImage. 2006;31(2):699-709. doi: 10.1016/j.neuroimage.2005.12.027.

626. Wallace MS, Charapata SG, Fisher R, Byas-Smith M, Staats PS, Mayo M, et al. Intrathecal ziconotide in the treatment of chronic nonmalignant pain: a randomized, double-blind, placebo-controlled clinical trial. Neuromodulation. 2006;9(2):75-86. doi: 10.1111/j.1525-1403.2006.00055.x. PubMed PMID: CN-00622675.

627. Wang IJ, Sun YC, Lee YC, Hou YC, Hu FR. The relationship between anterior corneal aberrations and contrast sensitivity in conventional LASIK. Curr Eye Res. 2006;31(7-8):563-8. Epub 2006/08/01. doi: 10.1080/02713680600814761. PubMed PMID: 16877264.

628. Ward NS, Frackowiak RS. The functional anatomy of cerebral reorganisation after focal brain injury. J Physiol Paris. 2006;99(4-6):425-36. Epub 2006/06/06. doi: 10.1016/j.jphysparis.2006.03.002. PubMed PMID: 16750616.

629. Wiese H, Tönnes C, de Greiff A, Nebel K, Diener HC, Stude P. Self-initiated movements in chronic prefrontal traumatic brain injury: An event-related functional MRI study. NeuroImage. 2006;30(4):1292-301. doi: 10.1016/j.neuroimage.2005.11.012.

630. Zappasodi F, Pasqualetti P, Tombini M, Ercolani M, Pizzella V, Rossini PM, et al. Hand cortical representation at rest and during activation: Gender and age effects in the two hemispheres. Clinical Neurophysiology. 2006;117(7):1518-28. doi:

10.1016/j.clinph.2006.03.016. PubMed PMID: WOS:000239227100014.

631. Zumsteg D, Wennberg R, Gütling E, Hess K. Whiplash and concussion: similar acute changes in middle-latency SEPs. Can J Neurol Sci. 2006;33(4):379-86. Epub 2006/12/16. doi: 10.1017/s0317167100005333. PubMed PMID: 17168163.

632. Abo-Krysha N, Farouk AA. Utility of clinical assessment and electrophysiological study of the respiratory impairment in Egyptian patients with multiple sclerosis. Journal of Neurological Sciences. 2007;24(4):296-306.

633. Ansari S, Chaudhri K, Al Moutaery KA. Vagus nerve stimulation: indications and limitations. Acta neurochirurgica Supplement. 2007;97(Pt 2):281-6. PubMed PMID: MEDLINE:17691314.

634. Belardinelli P, Ciancetta L, Staudt M, Pizzella V, Londei A, Birbaumer N, et al. Cerebro-muscular and cerebro-cerebral coherence in patients with pre- and perinatally acquired unilateral brain lesions. Neuroimage. 2007;37(4):1301-14. Epub 2007/08/03. doi: 10.1016/j.neuroimage.2007.05.053. PubMed PMID: 17669666.

635. Canavero S, Bonicalzi V. Extradural cortical stimulation for movement disorders. Acta Neurochir Suppl. 2007;97(Pt 2):223-32. Epub 2007/08/19. doi: 10.1007/978-3-211-33081-4_25. PubMed

PMID: 17691308.
636. Canavero S, Bonicalzi V. Extradural cortical stimulation for central pain. Acta Neurochir Suppl. 2007;97(Pt 2):27-36. Epub 2007/08/19. doi: 10.1007/978-3-211-33081-4_3. PubMed PMID: 17691286.
637. Chiu F-C, Lai C-S, Lee R-S. Synthesis functional poly(carbonate-b-ester) copolymers and micellar characterizations. Journal of Applied Polymer Science. 2007;106(1):283-92. doi: 10.1002/app.26454. PubMed PMID: WOS:000248609900037.
638. Cioni B. Motor cortex stimulation for Parkinson's disease. Acta neurochirurgica Supplement. 2007;97(Pt 2):233-8. PubMed PMID: MEDLINE:17691309.
639. Cioni B, Meglio M. Motor cortex stimulation for chronic non-malignant pain: current state and future prospects. Acta Neurochir Suppl. 2007;97(Pt 2):45-9. Epub 2007/08/19. doi: 10.1007/978-3-211-33081-4_5. PubMed PMID: 17691288.
640. Cioni B, Meglio M, Perotti V, De Bonis P, Montano N. Neurophysiological aspects of chronic motor cortex stimulation. Neurophysiol Clin. 2007;37(6):441-7. Epub 2007/12/18. doi: 10.1016/j.neucli.2007.10.007. PubMed PMID: 18083500.
641. Cruccu G, Aziz TZ, Garcia-Larrea L, Hansson P, Jensen TS, Lefaucheur JP, et al. EFNS guidelines on neurostimulation therapy for

neuropathic pain. Eur J Neurol. 2007;14(9):952-70. Epub 2007/08/28. doi: 10.1111/j.1468-1331.2007.01916.x. PubMed PMID: 17718686.

642. De Beaumont L, Lassonde M, Leclerc S, Théoret H. Long-term and cumulative effects of sports concussion on motor cortex inhibition. Neurosurgery. 2007;61(2):329-36; discussion 36-7. Epub 2007/09/01. doi: 10.1227/01.Neu.0000280000.03578.B6. PubMed PMID: 17762745.

643. Desmurget M, Bonnetblanc F, Duffau H. Contrasting acute and slow-growing lesions: a new door to brain plasticity. Brain. 2007;130:898-914. doi: 10.1093/brain/awl300. PubMed PMID: WOS:000246293300004.

644. Ernst S, Chun JKR, Ujeyl A, Ouyang F, Kuck KH. "Sequential" mapping mimicking "simultaneous" mapping using magnetic navigation during catheter ablation of supraventricular tachycardia: Results of the single DX study. Journal of Cardiovascular Electrophysiology. 2007;18(SUPPL. 1):S11-S7. doi: 10.1111/j.1540-8167.2007.00705.x.

645. Favier I, van Vliet JA, Roon KI, Witteveen RJ, Verschuuren JJ, Ferrari MD, et al. Trigeminal autonomic cephalgias due to structural lesions: a review of 31 cases. Arch Neurol. 2007;64(1):25-31. Epub 2007/01/11. doi: 10.1001/archneur.64.1.25. PubMed PMID: 17210806.

646. Favier I, van Vliet JA, Roon KI, Witteveen RJW, Verschuuren JJGM, Ferrari MD, et al. Trigeminal autonomic cephalgias due to structural lesions - A review of 31 cases. Archives of Neurology. 2007;64(1):25-31. doi: 10.1001/archneur.64.1.25. PubMed PMID: WOS:000243336500004.

647. Floel A, Cohen LG. Contribution of noninvasive cortical stimulation to the study of memory functions. Brain Res Rev. 2007;53(2):250-9. Epub 2006/10/07. doi: 10.1016/j.brainresrev.2006.08.006. PubMed PMID: 17023050.

648. Fodstad H, Hariz M. Electricity in the treatment of nervous system disease. Acta Neurochir Suppl. 2007;97(Pt 1):11-9. Epub 2007/08/19. doi: 10.1007/978-3-211-33079-1_2. PubMed PMID: 17691352.

649. Fried M, Hixson KK, Anderson L, Ogata Y, Mutabingwa TK, Duffy PE. The distinct proteome of placental malaria parasites. Molecular and Biochemical Parasitology. 2007;155(1):57-65. doi: 10.1016/j.molbiopara.2007.05.010.

650. Fujiki M, Hikawa T, Abe T, Anan M, Sugita K, Kobayashi H. Navigated brain stimulation for preoperative anatomic and functional identification of impaired motor cortex in a patient with meningioma. Neurosurgery Quarterly. 2007;17(1):33-9. doi: 10.1097/WNQ.0b013e3180329833.

651. Gimranov RF, Moskalenko SN, Gimranova Zh V. [Investigation of evoked acoustic brainstem potentials in norm and pathology]. Voen Med Zh. 2007;328(2):24-7. Epub 2007/05/19. PubMed PMID: 17508606.

652. Guzzetta A, Bonanni P, Biagi L, Tosetti M, Montanaro D, Guerrini R, et al. Reorganisation of the somatosensory system after early brain damage. Clinical Neurophysiology. 2007;118(5):1110-21. doi: 10.1016/j.clinph.2007.02.014.

653. Haddadan K, Krames ES. Technique that better localizes the dura mater during intrathecal catheterization should reduce inadvertent multiple dural punctures and reduce complications. Neuromodulation. 2007;10(2):164-6. doi: 10.1111/j.1525-1403.2007.00105.x.

654. Han S, Humphreys GW. The fronto-parietal network and top-down modulation of perceptual grouping. Neurocase. 2007;13(4):278-89. doi: 10.1080/13554790701649930.

655. Holsheimer J, Khan YN, Raza SS, Khan EA. Effects of electrode positioning on perception threshold and paresthesia coverage in spinal cord stimulation. Neuromodulation. 2007;10(1):34-41. doi: 10.1111/j.1525-1403.2007.00085.x.

656. Hu J, Yang S, Xuan Y, Jiang Q, Yang Y, Haacke EM. Simultaneous detection of resolved glutamate, glutamine, and γ-aminobutyric acid at 4 T. Journal of Magnetic Resonance.

2007;185(2):204-13. doi: 10.1016/j.jmr.2006.12.010.

657. Ibekwe TS, Ijaduola GT, Nwaorgu OG. Tympanic membrane perforation among adults in West Africa. Otol Neurotol. 2007;28(3):348-52. Epub 2007/04/07. doi: 10.1097/MAO.0b013e3180311605. PubMed PMID: 17414040.

658. Jafri B, Li X, Yang H, Rabinowitz YS. Higher order wavefront aberrations and topography in early and suspected keratoconus. J Refract Surg. 2007;23(8):774-81. Epub 2007/11/08. doi: 10.3928/1081-597x-20071001-06. PubMed PMID: 17985796.

659. Keenan JP, Gorman J. The causal role of the right hemisphere in self-awareness: It is the brain that is selective. Cortex. 2007;43(8):1074-82. doi: 10.1016/S0010-9452(08)70705-6.

660. Khouri AA, El Houni A, Afandi B. A complicated case of prolactinoma. Emirates Medical Journal. 2007;25(2):241-3.

661. Kishima H, Saitoh Y, Osaki Y, Nishimura H, Kato A, Hatazawa J, et al. Motor cortex stimulation in patients with deafferentation pain: activation of the posterior insula and thalamus. Journal of Neurosurgery. 2007;107(1):43-8. doi: 10.3171/jns-07/07/0043. PubMed PMID: WOS:000247799900008.

662. Kleim JA, Kleim ED, Cramer SC. Systematic assessment of training-induced changes in corticospinal output to hand using frameless stereotaxic transcranial magnetic stimulation. Nat Protoc.

2007;2(7):1675-84. Epub 2007/07/21. doi: 10.1038/nprot.2007.206. PubMed PMID: 17641632.

663. Kunert HJ, Norra C, Hoff P. Theories of delusional disorders - An update and review. Psychopathology. 2007;40(3):191-202. doi: 10.1159/000100367. PubMed PMID: WOS:000245269800009.

664. Lamme VAF. Sue Ned Block!: Making a better case for P-consciousness. Behavioral and Brain Sciences. 2007;30(5-6):511-2. doi: 10.1017/S0140525X07002920.

665. Lazorthes Y, Sol JC, Fowo S, Roux FE, Verdié JC. Motor cortex stimulation for neuropathic pain. Acta Neurochir Suppl. 2007;97(Pt 2):37-44. Epub 2007/08/19. doi: 10.1007/978-3-211-33081-4_4. PubMed PMID: 17691287.

666. Lepilin AV, Bakhteeva GR, Erokina NL. [Transcutaneous electric stimulation of nerves use in comprehensive treatment of patients with mandibular fractures]. Stomatologiia (Mosk). 2007;86(2):54-6. Epub 2007/09/11. PubMed PMID: 17828071.

667. Li Y, Wang Z, Wang HL, Yu XZ. An analysis of the early diagnostic criteria for tuberculosis meningitis. Zhonghua nei ke za zhi [Chinese journal of internal medicine]. 2007;46(3):217-9.

668. Lomarev MP, Kim DY, Richardson SP, Voller B, Hallett M. Safety study of high-frequency transcranial magnetic stimulation in patients with chronic stroke. Clinical Neurophysiology.

2007;118(9):2072-5. doi: 10.1016/j.clinph.2007.06.016.

669. Luo MF, He JN, Guo Y, Li CH, Zhang JL. [Effect of electroacupuncture and moxibustion of "Dazhui" (GV14) on the number and distribution of degranulated mast cells in GV14 region]. Zhen Ci Yan Jiu. 2007;32(5):327-9. Epub 2007/12/07. PubMed PMID: 18050625.

670. Manola L, Holsheimer J. Motor cortex stimulation: role of computer modeling. Acta neurochirurgica Supplement. 2007;97(Pt 2):497-503. PubMed PMID: MEDLINE:17691340.

671. McDannold N, Vykhodtseva N, Hynynen K. Use of ultrasound pulses combined with definity for targeted blood-brain barrier disruption: A feasibility study. Ultrasound in Medicine and Biology. 2007;33(4):584-90. doi: 10.1016/j.ultrasmedbio.2006.10.004. PubMed PMID: WOS:000245645400012.

672. Medvedovsky M, Taulu S, Bikmullina R, Paetau R. Artifact and head movement compensation in MEG. Neurology, Neurophysiology and Neuroscience. 2007;2007:1-10.

673. Moliavchikova OV, Cherevashchenko LA, Grinzaĭd Iu M, Aĭvazov VN, Zhuravlev ME. [Effects of combined action of radon baths and transcranial magnetotherapy on cerebral circulation in patients in an intermediate period of a mild craniocerebral trauma]. Vopr Kurortol Fizioter Lech Fiz Kult. 2007(3):19-22. Epub

2007/07/25. PubMed PMID: 17647311.

674. Morita I, Keith MW, Kanno T. Dorsal column stimulation for persistent vegetative state. Acta neurochirurgica Supplement. 2007;97(Pt 1):455-9. PubMed PMID: MEDLINE:17691409.

675. Nct. TMS in the Treatment of the Sequelae of Closed Brain Injury. https://clinicaltrialsgov/show/NCT00531258. 2007. PubMed PMID: CN-02014393.

676. Nct. Migraine--Investigational Treatment of Migraine With Noninvasive Brain Stimulation. (tDCS- Migraine). https://clinicaltrialsgov/show/NCT00521196. 2007. PubMed PMID: CN-01590594.

677. Nelissen N, Vandenbulcke M, Fannes K, Verbruggen A, Peeters R, Dupont P, et al. A beta amyloid deposition in the language system and how the brain responds. Brain. 2007;130:2055-69. doi: 10.1093/brain/awm133. PubMed PMID: WOS:000249097700011.

678. Nersesyan H, Slavin KV. Current aproach to cancer pain management: Availability and implications of different treatment options. Therapeutics and Clinical Risk Management. 2007;3(3):381-400.

679. Ng YS. Clinical features and rehabilitation of strokes in the less common vascular territories: Anterior and posterior cerebral artery strokes. Critical Reviews in Physical and Rehabilitation Medicine.

2007;19(4):295-329. doi: 10.1615/CritRevPhysRehabilMed.v19.i4.30.
680. Nickel J, Jorgens S, Bussfeld P, Seitz RJ. Asymptomatic motor cortex displacement due to a giant arachnoid cyst. Neurocase. 2007;13(5-6):328-33. doi: 10.1080/13554790701849910.
681. Panettieri V, Wennberg B, Gagliardi G, Duch MA, Ginjaume M, Lax I. SBRT of lung tumours: Monte Carlo simulation with PENELOPE of dose distributions including respiratory motion and comparison with different treatment planning systems. Physics in Medicine and Biology. 2007;52(14):4265-81. doi: 10.1088/0031-9155/52/14/016.
682. Panourias IG, Themistocleous M, Sakas DE. Intrathecal baclofen in current neuromodulatory practice: established indications and emerging applications. Acta neurochirurgica Supplement. 2007;97(Pt 1):145-54. PubMed PMID: MEDLINE:17691370.
683. Park OH, Lee KJ, Rhyu IJ, Geum D, Kim H, Buss R, et al. Bax-dependent and -independent death of motoneurons after facial nerve injury in adult mice. Eur J Neurosci. 2007;26(6):1421-32. Epub 2007/09/08. doi: 10.1111/j.1460-9568.2007.05787.x. PubMed PMID: 17822434.
684. Payne CM, Weber C, Crowley-Skillicorn C, Dvorak K, Bernstein H, Bernstein C, et al. Deoxycholate induces mitochondrial oxidative stress and activates NF-kappaB through multiple

mechanisms in HCT-116 colon epithelial cells. Carcinogenesis. 2007;28(1):215-22. Epub 2006/08/05. doi: 10.1093/carcin/bgl139. PubMed PMID: 16887864.

685. Payne CM, Weber C, Crowley-Skillicorn C, Dvorak K, Bernstein H, Bernstein C, et al. Deoxycholate induces mitochondrial oxidative stress and activates NF-κB through multiple mechanisms in HCT-116 colon epithelial cells. Carcinogenesis. 2007;28(1):215-22. doi: 10.1093/carcin/bgl139.

686. Petropoulou KB, Panourias IG, Rapidi CA, Sakas DE. The importance of neurorehabilitation to the outcome of neuromodulation in spasticity. Acta Neurochir Suppl. 2007;97(Pt 1):243-50. Epub 2007/08/19. doi: 10.1007/978-3-211-33079-1_33. PubMed PMID: 17691383.

687. Poggel DA, Mueller-Oehring EM, Gothe J, Kenkel S, Kasten E, Sabel BA. Visual hallucinations during spontaneous and training-induced visual field recovery. Neuropsychologia. 2007;45(11):2598-607. doi: 10.1016/j.neuropsychologia.2007.03.005. PubMed PMID: WOS:000247865800020.

688. Richard I, Menei P. Intrathecal baclofen in the treatment of spasticity, dystonia and vegetative disorders. Acta neurochirurgica Supplement. 2007;97(Pt 1):213-8. PubMed PMID: MEDLINE:17691379.

689. Riós-Romenets S, Castaño-Monsalve B, Bernabeu-Guitart M. [Pharmacotherapy of the cognitive sequelae secondary to traumatic brain injury]. Rev Neurol. 2007;45(9):563-70. Epub 2007/11/06. PubMed PMID: 17979087.

690. Ross ED, Prodan CI, Monnot M. Human facial expressions are organized functionally across the upper-lower facial axis. Neuroscientist. 2007;13(5):433-46. doi: 10.1177/1073858407305618.

691. Sakas DE, Panourias IG, Simpson BA, Krames ES. An introduction to operative neuromodulation and functional neuroprosthetics, the new frontiers of clinical neuroscience and biotechnology. Acta Neurochir Suppl. 2007;97(Pt 1):3-10. Epub 2007/08/19. doi: 10.1007/978-3-211-33079-1_1. PubMed PMID: 17691351.

692. Sasso A. The value of somatosensory evoked potentials in prediction of neurodevelopmental outcome. Paediatria Croatica, Supplement. 2007;51(1):130-4.

693. Scherder EJA, Vuijk P-J, Swaab DF, van Someren EJW. Estimating the effects of right median nerve stimulation on memory in Alzheimer's disease: A randomized controlled pilot study. Experimental Aging Research. 2007;33(2):177-86. doi: 10.1080/03610730701238915. PubMed PMID: WOS:000245435500005.

694. Schiff ND, Fins JJ. Deep brain stimulation and cognition: moving from animal to patient. Current Opinion in Neurology. 2007;20(6):638-42. doi: 10.1097/WCO.0b013e3282f1c6e4. PubMed PMID: WOS:000251209300006.

695. Sgouros S. Surgical management of spasticity of cerebral origin in children. Acta neurochirurgica Supplement. 2007;97(Pt 1):193-203. PubMed PMID: MEDLINE:17691377.

696. Sharova EV, Mel'nikov AV, Novikova MR, Kulikov MA, Grechenko TN, Shekhter ED, et al. Changes in spontaneous brain bioelectrical activity during transcranial electrical and electromagnetic stimulation. Neurosci Behav Physiol. 2007;37(5):451-7. Epub 2007/05/17. doi: 10.1007/s11055-007-0034-3. PubMed PMID: 17505794.

697. Staudt M. (Re-)organization of the developing human brain following periventricular white matter lesions. Neuroscience and Biobehavioral Reviews. 2007;31(8):1150-6. doi: 10.1016/j.neubiorev.2007.05.005. PubMed PMID: WOS:000251873300009.

698. Staudt M. (Re-) organization of sensorimotor and language functions after periventricular brain lesions. Journal fur Neurologie, Neurochirurgie und Psychiatrie. 2007;8(4):7-9.

699. Tani N, Saitoh Y, Kishima H, Oshino S, Hatazawa J,

Hashikawa K, et al. Motor cortex stimulation for levodopa-resistant akinesia: case report. Mov Disord. 2007;22(11):1645-9. Epub 2007/06/09. doi: 10.1002/mds.21593. PubMed PMID: 17557343.
700. van Kuijk AA, Hendricks HT, Pasman JW, Kremer BH, Geurts AC. Are neuroradiological or neurophysiological characteristics associated with upper-extremity hypertonia in severe ischaemic supratentorial stroke? Journal of Rehabilitation Medicine. 2007;39(1):38-42. doi: 10.2340/16501977-0010.
701. Voropaev AA, Troshin VD, Mochalov AD. [Craniospinal electromagnetic therapy in the neurological practice]. Zh Nevrol Psikhiatr Im S S Korsakova. 2007;107(12):16-20. Epub 2008/04/23. PubMed PMID: 18427454.
702. Wiedemann C, Whalley K, Welberg L. From the editors. Nature Reviews Neuroscience. 2007;8(7):489. doi: 10.1038/nrn2184.
703. Wu AK, McCairn KW, Zada G, Wu T, Turner RS. Motor cortex stimulation: mild transient benefit in a primate model of Parkinson disease. Journal of Neurosurgery. 2007;106(4):695-700. doi: 10.3171/jns.2007.106.4.695. PubMed PMID: WOS:000245586900028.
704. Yasokawa YT, Shinoda J, Okumura A, Nakayama N, Miwa K, Iwama T. Correlation between diffusion-tensor magnetic resonance imaging and motor-evoked potential in chronic severe diffuse axonal

injury. J Neurotrauma. 2007;24(1):163-73. Epub 2007/02/01. doi: 10.1089/neu.2006.0073. PubMed PMID: 17263680.

705. Yildirim A, Kotan D, Yildirim S, Aygül R, Akçay F. Increased lipid peroxidation and decreased antioxidant response in serum and cerebrospinal fluid in acute ischemic stroke. Turkish Journal of Medical Sciences. 2007;37(2):75-81.

706. Yokota H, Atsumi T, Araki T, Fuse A, Sato H, Kushimoto S, et al. Cerebral endothelial injury in elderly patients with severe head injury measured by serum thrombomodulin and von Willebrand factor. Neurologia Medico-Chirurgica. 2007;47(9):383-8. doi: 10.2176/nmc.47.383.

707. Alkire MT. Loss of effective connectivity during general anesthesia. International Anesthesiology Clinics. 2008;46(3):55-73. doi: 10.1097/AIA.0b013e3181755dc6.

708. Allred RP, Jones TA. Experience: A double-edged sword for restorative neural plasticity after brain damage. Future Neurology. 2008;3(2):189-98. doi: 10.2217/14796708.3.2.189.

709. Andreani JCM, Guma C. New animal model to mimic spastic cerebral palsy: The brain-damaged pig preparation. Neuromodulation. 2008;11(3):196-201. doi: 10.1111/j.1525-1403.2008.00166.x.

710. Andreani JCM, Guma C. Lateral cord stimulation decreases spastic electromyographic spreading: Responses in a brain-damaged

pig preparation. Neuromodulation. 2008;11(3):202-7. doi: 10.1111/j.1525-1403.2008.00167.x.

711.Arango-Lasprilla JC, Ketchum JM, Williams K, Kreutzer JS, Marquez de la Plata CD, O'Neil-Pirozzi TM, et al. Racial Differences in Employment Outcomes After Traumatic Brain Injury. Archives of Physical Medicine and Rehabilitation. 2008;89(5):988-95. doi: 10.1016/j.apmr.2008.02.012.

712. Battelli L, Walsh V, Pascual-Leone A, Cavanagh P. The 'when' parietal pathway explored by lesion studies. Curr Opin Neurobiol. 2008;18(2):120-6. Epub 2008/08/19. doi: 10.1016/j.conb.2008.08.004. PubMed PMID: 18708141; PubMed Central PMCID: PMCPMC5076376.

713. Bushnik T, Englander J, Wright J. Patterns of fatigue and its correlates over the first 2 years after traumatic brain injury. The Journal of head trauma rehabilitation. 2008;23(1):25-32. doi: 10.1097/01.HTR.0000308718.88214.bb. PubMed PMID: MEDLINE:18219232.

714. Butterworth RF. Pathophysiology of hepatic encephalopathy: The concept of synergism. Hepatology Research. 2008;38(SUPPL. 1):S116-S21. doi: 10.1111/j.1872-034X.2008.00436.x.

715. Candidi M, Urgesi C, Ionta S, Aglioti SM. Virtual lesion of ventral premotor cortex impairs visual perception of biomechanically

possible but not impossible actions. Social neuroscience. 2008;3(3-4):388-400. doi: 10.1080/17470910701676269. PubMed PMID: CN-00684893.

716. Carey JR, Evans CD, Anderson DC, Bhatt E, Nagpal A, Kimberley TJ, et al. Safety of 6-Hz primed low-frequency rTMS in stroke. Neurorehabilitation and Neural Repair. 2008;22(2):185-92. doi: 10.1177/1545968307305458.

717. Chen J-K, Johnston KM, Petrides M, Ptito A. Neural substrates of symptoms of depression following concussion in male athletes with persisting postconcussion symptoms. Archives of General Psychiatry. 2008;65(1):81-9. doi: 10.1001/archgenpsychiatry.2007.8. PubMed PMID: WOS:000252176400011.

718. Clement EA, Richard A, Thwaites M, Ailon J, Peters S, Dickson CT. Cyclic and sleep-like spontaneous alternations of brain state under urethane anaesthesia. PLoS ONE. 2008;3(4). doi: 10.1371/journal.pone.0002004.

719. Cologno D, Buzzi MG, Cicinelli P, Caltagirone C, Spalletta G. Symptomatic cluster-like headache triggered by forehead lipoma: A case report and review of the literature. Neurological Sciences. 2008;29(5):331-5. doi: 10.1007/s10072-008-0990-6.

720. Cummings JL. Progress in neurotherapeutics and

neuropsychopharmacology 2008. Progress in Neurotherapeutics and Neuropsychopharmacology. 2008;3(1):1-11. doi: 10.1017/S1748232107000183.

721. Dabydeen L, Thomas JE, Aston TJ, Hartley H, Sinha SK, Eyre JA. High-energy and -protein diet increases brain and corticospinal tract growth in term and preterm infants after perinatal brain injury. Pediatrics. 2008;121(1):148-56. Epub 2008/01/02. doi: 10.1542/peds.2007-1267. PubMed PMID: 18166569.

722. Dijkerman HC, Vargha-Khadem F, Polkey CE, Weiskrantz L. Ipsilesional and contralesional sensorimotor function after hemispherectomy: Differences between distal and proximal function. Neuropsychologia. 2008;46(3):886-901. doi: 10.1016/j.neuropsychologia.2007.11.023. PubMed PMID: WOS:000254731700013.

723. Flanagan SR, Cantor JB, Ashman TA. Traumatic brain injury: Future assessment tools and treatment prospects. Neuropsychiatric Disease and Treatment. 2008;4(5):877-92. doi: 10.2147/ndt.s1985.

724. Frye RE, Rotenberg A, Ousley M, Pascual-Leone A. Transcranial Magnetic Stimulation in Child Neurology: Current and Future Directions. Journal of Child Neurology. 2008;23(1):79-96. doi: 10.1177/0883073807307972. PubMed PMID: WOS:000262554000013.

725. Gleason D, Fallon JH, Guerra M, Liu JC, Bryant PJ. Ependymal stem cells divide asymmetrically and transfer progeny into the subventricular zone when activated by injury. Neuroscience. 2008;156(1):81-8. Epub 2008/08/07. doi: 10.1016/j.neuroscience.2008.06.065. PubMed PMID: 18682279.

726. Gómez Herrero H, García Asensio S, Martínez-Berganza Asensio MT, Sarría Octavio De Toledo L, Cozcolluela Cabrejas R, Forradellas Morales A. Indirect CT venography in patients with clinical suspicion of pulmonary thromboembolism: Our experience and possible indications for the test. Radiologia. 2008;50(2):147-52. doi: 10.1016/S0033-8338(08)71948-9.

727. Gruessner RWG, Sutherland DER, Kandaswamy R, Gruessner AC. Over 500 solitary pancreas transplants in nonuremic patients with brittle diabetes mellitus. Transplantation. 2008;85(1):42-7. doi: 10.1097/01.tp.0000296820.46978.3f.

728. Herraiz C. Assessing the cause of tinnitus for therapeutic options. Expert Opinion on Medical Diagnostics. 2008;2(10):1183-96. doi: 10.1517/17530059.2.10.1183.

729. Hill K, Dutta P, Zareba A, Eldridge ML, Sepaniak MJ. Morphological and chemical optimization of microcantilever surfaces for thyroid system biosensing and beyond. Anal Chim Acta. 2008;625(1):55-62. Epub 2008/08/30. doi: 10.1016/j.aca.2008.07.005.

PubMed PMID: 18721540.

730. Hosomi K, Saitoh Y, Kishima H, Oshino S, Hirata M, Tani N, et al. Electrical stimulation of primary motor cortex within the central sulcus for intractable neuropathic pain. Clin Neurophysiol. 2008;119(5):993-1001. Epub 2008/03/11. doi: 10.1016/j.clinph.2007.12.022. PubMed PMID: 18329955.

731. Hossain MA, Afroj S, Rahman MR, Yunus EB, Samad R, Asna ZH, et al. Evaluation of alternative diagnostic techniques for diagnosis of cerebral malaria in a tertiary referral hospital in Bangladesh. Mymensingh medical journal : MMJ. 2008;17(2):180-5.

732. Isagulian ED, Shabalov VA. [Neuromodulation--controlled analgesia. Criteria for long efficiency]. Zh Vopr Neirokhir Im N N Burdenko. 2008(2):18-26; discussion -8. Epub 2008/08/30. PubMed PMID: 18720727.

733. Isagulyan ED, Shabalov VA. Neuromodulation is controlled analgesia: Criteria for long efficiency. Problems of Neurosurgery named after NN Burdenko. 2008(2):18-28. PubMed PMID: RSCI:11655387.

734. Jarema M. A few facts from the history of selected biological treatment methods in psychiatry. Neuro Endocrinol Lett. 2008;29 Suppl 1:5-9. Epub 2008/11/26. PubMed PMID: 19029880.

735. Ji YK, Eun JC, Won YL, Hee YS, Gyeong HL, Choe YS, et al.

Therapeutic effect of repetitive transcranial magnetic stimulation in Parkinson's disease: Analysis of [11C] raclopride PET study. Movement Disorders. 2008;23(2):207-11. doi: 10.1002/mds.21787.
736. Jin Y, Hilgetag CC. Perturbation of visuospatial attention by high-frequency offline rTMS. Exp Brain Res. 2008;189(1):121-8. Epub 2008/06/20. doi: 10.1007/s00221-008-1449-y. PubMed PMID: 18563400.
737. Kao C-D, Chen J-T, Lai K-L, Chang J-B, Wu Z-A, Liao K-K. Gabapentin for decerebrate rigidity - A case report. Clinical Drug Investigation. 2008;28(1):67-70. doi: 10.2165/00044011-200828010-00008. PubMed PMID: WOS:000252174300008.
738. Kao CD, Chen JT, Lai KL, Chang JB, Wu ZA, Liao KK. Gabapentin for decerebrate rigidity: a case report. Clin Drug Investig. 2008;28(1):67-70. Epub 2007/12/18. doi: 10.2165/00044011-200828010-00008. PubMed PMID: 18081362.
739. Kirton A, Deveber G, Gunraj C, Chen R. Neurocardiogenic syncope complicating pediatric transcranial magnetic stimulation. Pediatr Neurol. 2008;39(3):196-7. Epub 2008/08/30. doi: 10.1016/j.pediatrneurol.2008.06.004. PubMed PMID: 18725065.
740. Knott C. Research into cannabinoid medicines may suggest potential therapeutic opportunities. Pharmacy in Practice.

2008;18(3):96-109.

741. Koenigs M, Huey ED, Calamia M, Raymont V, Tranel D, Grafman J. Distinct Regions of Prefrontal Cortex Mediate Resistance and Vulnerability to Depression. Journal of Neuroscience. 2008;28(47):12341-8. doi: 10.1523/jneurosci.2324-08.2008. PubMed PMID: WOS:000261191000020.

742. Koenigs M, Huey ED, Raymont V, Cheon B, Solomon J, Wassermann EM, et al. Focal brain damage protects against post-traumatic stress disorder in combat veterans. Nature Neuroscience. 2008;11(2):232-7. doi: 10.1038/nn2032. PubMed PMID: WOS:000252712100018.

743. Lenzi D, Raz E, Pantano P. FMRI and multiple sclerosis. Current Medical Imaging Reviews. 2008;4(3):163-9. doi: 10.2174/157340508785294507.

744. Liu JT, Lee JK, Chang PJ, Sun CM. Increased regional cerebral perfusion in contralateral motor and somatosensory areas after median nerve stimulation therapy. Acta Neurochir Suppl. 2008;101:65-70. Epub 2008/07/23. doi: 10.1007/978-3-211-78205-7_11. PubMed PMID: 18642636.

745. Liu JT, Lee JK, Tyan YS, Liu CY, Lin TB. Change in cerebral perfusion of patients with coma after treatment with right median nerve stimulation and hyperbaric oxygen. Neuromodulation.

2008;11(4):296-301. Epub 2008/10/01. doi: 10.1111/j.1525-1403.2008.00179.x. PubMed PMID: 22151144.

746. Marquez de la Plata CD, Hart T, Hammond FM, Frol AB, Hudak A, Harper CR, et al. Impact of Age on Long-Term Recovery From Traumatic Brain Injury. Archives of Physical Medicine and Rehabilitation. 2008;89(5):896-903. doi: 10.1016/j.apmr.2007.12.030.

747. Masino SA, Geiger JD. Are purines mediators of the anticonvulsant/neuroprotective effects of ketogenic diets? Trends in Neurosciences. 2008;31(6):273-8. doi: 10.1016/j.tins.2008.02.009. PubMed PMID: WOS:000257002600002.

748. McDannold N, Vykhodtseva N, Hynynen K. Effects of acoustic parameters and ultrasound contrast agent dose on focused-ultrasound induced blood-brain barrier disruption. Ultrasound in Medicine and Biology. 2008;34(6):930-7. doi: 10.1016/j.ultrasmedbio.2007.11.009. PubMed PMID: WOS:000256485200009.

749. Miniussi C, Cappa SF, Cohen LG, Floel A, Fregni F, Nitsche MA, et al. Efficacy of repetitive transcranial magnetic stimulation/transcranial direct current stimulation in cognitive neurorehabititation. Brain Stimulation. 2008;1(4):326-36. doi: 10.1016/j.brs.2008.07.002. PubMed PMID: WOS:000262717000002.

750. Miniussi C, Cappa SF, Cohen LG, Floel A, Fregni F, Nitsche

MA, et al. Efficacy of repetitive transcranial magnetic stimulation/transcranial direct current stimulation in cognitive neurorehabilitation. Brain Stimul. 2008;1(4):326-36. Epub 2008/10/01. doi: 10.1016/j.brs.2008.07.002. PubMed PMID: 20633391.

751. Müri RM, Nyffeler T. Neurophysiology and neuroanatomy of reflexive and volitional saccades as revealed by lesion studies with neurological patients and transcranial magnetic stimulation (TMS). Brain Cogn. 2008;68(3):284-92. Epub 2008/10/11. doi: 10.1016/j.bandc.2008.08.018. PubMed PMID: 18845373.

752. Naito T, Mino Y, Otsuka A, Ushiyama T, Ozono S, Kagawa Y, et al. Influence of metal cations on plasma trough concentration of mycophenolic acid and its glucuronide in tacrolimus-treated and cyclosporine-treated kidney transplant recipients. Biological and Pharmaceutical Bulletin. 2008;31(6):1292-6. doi: 10.1248/bpb.31.1292.

753. Obuchi T, Katayama Y, Kobayashi K, Oshima H, Fukaya C, Yamamoto T. Direction and predictive factors for the shift of brain structure during deep brain stimulation electrode implantation for advanced Parkinson's disease. Neuromodulation. 2008;11(4):302-10. doi: 10.1111/j.1525-1403.2008.00180.x.

754. Park J-H, von Maltzahn G, Ruoslahti E, Bhatia SN, Sailor MJ. Micellar hybrid nanoparticles for simultaneous magnetofluorescent

imaging and drug delivery. Angewandte Chemie-International Edition. 2008;47(38):7284-8. doi: 10.1002/anie.200801810. PubMed PMID: WOS:000259382900026.

755. Poreisz C, Csifcsák G, Antal A, Levold M, Hillers F, Paulus W. Theta burst stimulation of the motor cortex reduces laser-evoked pain perception. NeuroReport. 2008;19(2):193-6. doi: 10.1097/WNR.0b013e3282f45498.

756. Ricci R, Ramsey D, Johnson K, Borckardt JJ, Vallejo M, Roberts DR, et al. A pilot feasibility study of daily rTMS to modify corticospinal excitability during lower limb immobilization. Therapeutics and Clinical Risk Management. 2008;4(5):1127-34.

757. Rubí Callejón J, Arjona Padillo A, Alonso Verdegay G, Huete Hurtado A, Naranjo C, Serrano Castro P, et al. Trigeminal-autonomic cephalalgias: International Headache Society diagnostic criteria does not allow for a correct classification. Neurologia. 2008;23(4):203-8.

758. Sagar SM, Wong RK. Chinese medicine and biomodulation in cancer patients - Part two. Current Oncology. 2008;15(2):8-30. doi: 10.3747/co.v15i2.238.

759. Sandin M, Thörn SE, Dahlqvist A, Wattwil L, Axelsson K, Wattwil M. Effects of pain stimulation on bispectral index, heart rate and blood pressure at different minimal alveolar concentration values of sevoflurane. Acta Anaesthesiol Scand. 2008;52(3):420-6. Epub

2008/02/14. doi: 10.1111/j.1399-6576.2007.01569.x. PubMed PMID: 18269392.

760. Schiff ND. Central thalamic contributions to arousal regulation and neurological disorders of consciousness. Annals of the New York Academy of Sciences2008. p. 105-18.

761. Sedlackova S, Rektorova I, Fanfrdlova Z, Rektor I. Neurocognitive effects of repetitive transcranial magnetic stimulation in patients with cerebrovascular disease without dementia. Journal of Psychophysiology. 2008;22(1):14-9. doi: 10.1027/0269-8803.22.1.14.

762. Serruya MD, Kahana MJ. Techniques and devices to restore cognition. Behav Brain Res. 2008;192(2):149-65. Epub 2008/06/10. doi: 10.1016/j.bbr.2008.04.007. PubMed PMID: 18539345; PubMed Central PMCID: PMCPMC3051349.

763. Shaĭkhaev GO, Kuz'mina NS, Miazin AE, Panushkina OG, Rubanovich AV, Khaĭmovich TI, et al. [The analysis of mutations at mini- and microsatellite DNA loci in the family members of a group of occupationally exposed to tritium and tritium oxide plant workers]. Radiats Biol Radioecol. 2008;48(6):690-7. Epub 2009/01/31. PubMed PMID: 19178046.

764. Shaladi A, Crestani F, Saltari R, Piva B. [Percutaneous electrical nerve stimulation of peripheral nerve for the intractable occipital neuralgia]. Recenti Prog Med. 2008;99(6):295-301. Epub

2008/08/20. PubMed PMID: 18710060.
765. Solak O, Kavuncu V. Current Approaches in Therapeutic Management of Chronic Neuropathic Pain. Romatizma-Rheumatism. 2008;23(4):135-42. PubMed PMID: WOS:000262426100006.
766. Song YG, Hao FT, Zhu J, Li A, Xia YJ, Wang DX. [Clinical study on pneumoconiosis with pulmonary thromboembolism]. Zhonghua lao dong wei sheng zhi ye bing za zhi = Zhonghua laodong weisheng zhiyebing zazhi = Chinese journal of industrial hygiene and occupational diseases. 2008;26(1):39-42.
767. Staudt M, Ticini LF, Grodd W, Krägeloh-Mann I, Karnath HO. Functional topography of early periventricular brain lesions in relation to cytoarchitectonic probabilistic maps. Brain Lang. 2008;106(3):177-83. Epub 2008/02/16. doi: 10.1016/j.bandl.2008.01.007. PubMed PMID: 18275996.
768. Strangman GE, O'Neil-Pirozzi TM, Goldstein R, Kelkar K, Katz DI, Burke D, et al. Prediction of Memory Rehabilitation Outcomes in Traumatic Brain Injury by Using Functional Magnetic Resonance Imaging. Archives of Physical Medicine and Rehabilitation. 2008;89(5):974-81. doi: 10.1016/j.apmr.2008.02.011.
769. Visocchi M. Neuromodulation of cerebral blood flow by spinal cord electrical stimulation: the role of the Italian school and state of art. J Neurosurg Sci. 2008;52(2):41-7. Epub 2008/05/27.

PubMed PMID: 18500217.

770. Wang JL, Ren QS, Shen CC, Xie WX, Zheng RX, Ni JW. [Effect of transcutaneous acupoint electrical stimulation on blood bioactive compounds involving cerebral injury during craniotomy]. Zhen Ci Yan Jiu. 2008;33(1):26-30. Epub 2008/04/05. PubMed PMID: 18386641.

771. Weder ND, Muralee S, Penland H, Tampi RR. Catatonia: A Review. Annals of Clinical Psychiatry. 2008;20(2):97-107. doi: 10.1080/10401230802017092. PubMed PMID: WOS:000261639600006.

772. Wu HG, Jiang B, Zhou EH, Shi Z, Shi DR, Cui YH, et al. Regulatory mechanism of electroacupuncture in irritable bowel syndrome: preventing MC activation and decreasing SP VIP secretion. Dig Dis Sci. 2008;53(6):1644-51. Epub 2007/11/14. doi: 10.1007/s10620-007-0062-4. PubMed PMID: 17999187.

773. Yoo WK, Kim DS, Kwon YH, Jang SH. Kernohan's notch phenomenon demonstrated by diffusion tensor imaging and transcranial magnetic stimulation. J Neurol Neurosurg Psychiatry. 2008;79(11):1295-7. Epub 2008/10/23. doi: 10.1136/jnnp.2007.138131. PubMed PMID: 18940992.

774. Zitnay GA, Zitnay KM, Povlishock JT, Hall ED, Marion DW, Trudel T, et al. Traumatic brain injury research priorities: The

conemaugh international brain injury symposium. Journal of Neurotrauma. 2008;25(10):1135-52. doi: 10.1089/neu.2008.0599.
775. Akhtar J, Carlson JN, Abesamis MG, Katz KD. Somatosensory evoked potentials and brain-stem auditory evoked potentials in coma due venlafaxine overdose. Clinical Toxicology. 2009;47(5):453. doi: 10.1080/15563650902952273.
776. Baad-Hansen L, Blicher JU, Lapitskaya N, Nielsen JF, Svensson P. Intra-cortical excitability in healthy human subjects after tongue training. J Oral Rehabil. 2009;36(6):427-34. Epub 2009/05/09. doi: 10.1111/j.1365-2842.2009.01955.x. PubMed PMID: 19422433.
777. Badawy RAB, Jackson GD, Berkovic SF, Newton MR, Macdonell RAL. Diagnosis of epilepsy after the first seizure: a role for transcranial magnetic stimulation? Journal of the Neurological Sciences. 2009;285:S248-S. doi: 10.1016/s0022-510x(09)70944-x. PubMed PMID: WOS:000272521300874.
778. Baseler HA, Gouws A, Morland AB. The organization of the visual cortex in patients with scotomata resulting from lesions of the central retina. Neuro-Ophthalmology. 2009;33(3):149-57. doi: 10.1080/01658100903050053.
779. Bernabeu M, Demirtas-Tatlidede A, Opisso E, Lopez R, Tormos JM, Pascual-Leone A. Abnormal corticospinal excitability in traumatic diffuse axonal brain injury. J Neurotrauma.

2009;26(12):2185-93. Epub 2009/07/17. doi: 10.1089/neu.2008.0859. PubMed PMID: 19604100; PubMed Central PMCID: PMCPMC2824228.

780. Bikson M, Datta A, Elwassif M. Establishing safety limits for transcranial direct current stimulation. Clin Neurophysiol. 2009;120(6):1033-4. Epub 2009/04/28. doi: 10.1016/j.clinph.2009.03.018. PubMed PMID: 19394269; PubMed Central PMCID: PMCPMC2754807.

781. Bledsoe JM, Kimble CJ, Covey DP, Blaha CD, Agnesi F, Mohseni P, et al. Development of the Wireless Instantaneous Neurotransmitter Concentration System for intraoperative neurochemical monitoring using fast-scan cyclic voltammetry Technical note. Journal of Neurosurgery. 2009;111(4):712-23. doi: 10.3171/2009.3.Jns081348. PubMed PMID: WOS:000270550000013.

782. Blicher JU, Nielsen JF. Cortical and spinal excitability changes after robotic gait training in healthy participants. Neurorehabilitation and Neural Repair. 2009;23(2):143-9. doi: 10.1177/1545968308317973.

783. Bolognini N, Pascual-Leone A, Fregni F. Using non-invasive brain stimulation to augment motor training-induced plasticity. J Neuroeng Rehabil. 2009;6:8. Epub 2009/03/19. doi: 10.1186/1743-0003-6-8. PubMed PMID: 19292910; PubMed Central

PMCID: PMCPMC2667408.

784. Boly M, Massimini M, Tononi G. Theoretical approaches to the diagnosis of altered states of consciousness. Prog Brain Res. 2009;177:383-98. Epub 2009/10/13. doi: 10.1016/s0079-6123(09)17727-0. PubMed PMID: 19818916.

785. Braet W, Humphreys GW. The role of reentrant processes in feature binding: Evidence from neuropsychology and TMS on late onset illusory conjunctions. Visual Cognition. 2009;17(1-2):25-47. doi: 10.1080/13506280802193318.

786. Braun C, Demarchi G, Papadelis C. Cortical reorganization after damage to the central nervous system. Neuro-Ophthalmology. 2009;33(3):142-8. doi: 10.1080/01658100902825521.

787. Caramia F, Francia A, Mainero C, Tinelli E, Palmieri MG, Colonnese C, et al. Neurophysiological and functional MRI evidence of reorganization of cortical motor areas in cerebral arteriovenous malformation. Magnetic Resonance Imaging. 2009;27(10):1360-9. doi: 10.1016/j.mri.2009.05.029.

788. Carlson T, Andréll P, Ekre O, Edvardsson N, Holmgren C, Jacobsson F, et al. Interference of transcutaneous electrical nerve stimulation with permanent ventricular stimulation: A new clinical problem? Europace. 2009;11(3):364-9. doi: 10.1093/europace/eun351.

789. Castro G, Ramirez A, Bastidas J, Godinez N. Transcranial

magnetic stimulation in neuropathic pain and its potential use as screening test in patients candidates to motor cortex stimulation. Pain Practice. 2009;9:45. doi: 10.1111/j.1533-2500.2009.00266.x.

790. Chang WH, Kim Y-B, Ohn SH, Park C-h, Kim ST, Kim Y-H. Double decussated ipsilateral corticospinal tract in schizencephaly. Neuroreport. 2009;20(16):1434-8. doi: 10.1097/WNR.0b013e328331483d. PubMed PMID: WOS:000271292400008.

791. Chen MJ, Peng ML, Chen LW, Fan HL, Liou YM, Liao JM. Pressor effects on blood pressure induced by isovolumic bladder distension and electro-acupuncture stimulations in anesthetized rats. Chin J Physiol. 2009;52(6):426-31. Epub 2010/03/27. doi: 10.4077/cjp.2009.amh069. PubMed PMID: 20337150.

792. Chen XY, Zhu Y, Huang XS. [Effect of strong stimulation of acupuncture at twelve Jing-well points as main for neurosurgery patients with disorder of consciousness]. Zhongguo Zhen Jiu. 2009;29(8):619-22. Epub 2009/12/02. PubMed PMID: 19947263.

793. Chou P, Chu H, Lin JG. Effects of electroacupuncture treatment on impaired cognition and quality of life in Taiwanese stroke patients. J Altern Complement Med. 2009;15(10):1067-73. Epub 2010/01/06. PubMed PMID: 20050300.

794. De Beaumont L, Théoret H, Mongeon D, Messier J, Leclerc S,

Tremblay S, et al. Brain function decline in healthy retired athletes who sustained their last sports concussion in early adulthood. Brain. 2009;132(Pt 3):695-708. Epub 2009/01/30. doi: 10.1093/brain/awn347. PubMed PMID: 19176544.

795. De FP, Fellus J, Polito MZ, Thompson JW, Moser RS, DeLuca J. The new neuroscience frontier: promoting neuroplasticity and brain repair in traumatic brain injury. Clin Neuropsychol. 2009;23(8):1391-9. Epub 2009/11/03. doi: 10.1080/13854040903058978. PubMed PMID: 19882477.

796. Dobkin BH. Motor rehabilitation after stroke, traumatic brain, and spinal cord injury: common denominators within recent clinical trials. Current Opinion in Neurology. 2009;22(6):563-9. doi: 10.1097/WCO.0b013e3283314b11. PubMed PMID: WOS:000272544100001.

797. Edwards DJ, Krebs HI, Rykman A, Zipse J, Thickbroom GW, Mastaglia FL, et al. Raised corticomotor excitability of M1 forearm area following anodal tDCS is sustained during robotic wrist therapy in chronic stroke. Restorative Neurology and Neuroscience. 2009;27(3):199-207. doi: 10.3233/RNN-2009-0470.

798. Fontaine D, Bruneto JL, El Fakir H, Paquis P, Lanteri-Minet M. Short-term restoration of facial sensory loss by motor cortex stimulation in peripheral post-traumatic neuropathic pain. Journal of

Headache and Pain. 2009;10(3):203-6. doi: 10.1007/s10194-009-0115-5. PubMed PMID: WOS:000265924700009.

799. Fu YY, Cao SQ, Zhuang JX, Hu L, Chen DK, Gu FJ. [Observation on electroacupuncture combined with routine western medicine therapy for promoting consciousness of the patient with coma caused by craniocerebral trauma]. Zhongguo Zhen Jiu. 2009;29(2):107-10. Epub 2009/04/28. PubMed PMID: 19391532.

800. Gao J, Youn S, Hovsepyan A, Llaneza VL, Wang Y, Bitton G, et al. Dispersion and toxicity of selected manufactured nanomaterials in natural river water samples: effects of water chemical composition. Environ Sci Technol. 2009;43(9):3322-8. Epub 2009/06/19. doi: 10.1021/es803315v. PubMed PMID: 19534153.

801. Gurskaia OE, Ponomarev VA. [Methods of localization of equivalent EEG sources in the diagnostics of protracted loss of consciousness]. Zh Nevrol Psikhiatr Im S S Korsakova. 2009;109(4):36-42. Epub 2009/06/06. PubMed PMID: 19491808.

802. Heitger MH, Jones RD, Macleod AD, Snell DL, Frampton CM, Anderson TJ. Impaired eye movements in post-concussion syndrome indicate suboptimal brain function beyond the influence of depression, malingering or intellectual ability. Brain. 2009;132:2850-70. doi: 10.1093/brain/awp181. PubMed PMID:

WOS:000270685600031.

803. Hiraoka T, Mihashi T, Okamoto C, Okamoto F, Hirohara Y, Oshika T. Influence of induced decentered orthokeratology lens on ocular higher-order wavefront aberrations and contrast sensitivity function. J Cataract Refract Surg. 2009;35(11):1918-26. Epub 2009/11/03. doi: 10.1016/j.jcrs.2009.06.018. PubMed PMID: 19878824.

804. Hwang SC, Im SB, Kim BT, Shin WH. Safe entry point for twist-drill craniostomy of a chronic subdural hematoma. J Neurosurg. 2009;110(6):1265-70. Epub 2008/12/23. doi: 10.3171/2008.9.Jns08359. PubMed PMID: 19099378.

805. Jang SH. Review of motor recovery in patients with traumatic brain injury. NeuroRehabilitation. 2009;24(4):349-53. doi: 10.3233/nre-2009-0489. PubMed PMID: MEDLINE:19597273.

806. Jang SH, Kim D-S, Son SM, Cho YW, Kim SH, Kim OL, et al. Clinical application of diffusion tensor tractography for elucidation of the causes of motor weakness in patients with traumatic brain injury. NeuroRehabilitation. 2009;24(3):273-8. doi: 10.3233/nre-2009-0478. PubMed PMID: MEDLINE:19458435.

807. Jardri R. Transcranial magnetic stimulation for the management of hallucinations in children with early-onset schizophrenia. Neuropsychiatrie de l'Enfance et de l'Adolescence.

2009;57(1):38-43. doi: 10.1016/j.neurenf.2007.07.017.

808. John Y, Mark N, Edmonds M, Badawy R, Macdonell R. Transcranial magnetic stimulation as a diagnostic tool in first seizure patients. Journal of Clinical Neuroscience. 2009;16(11):1523-4. doi: 10.1016/j.jocn.2009.07.029.

809. Johnston MV. Plasticity in the developing brain: implications for rehabilitation. Dev Disabil Res Rev. 2009;15(2):94-101. Epub 2009/06/03. doi: 10.1002/ddrr.64. PubMed PMID: 19489084.

810. Kanno T, Morita I, Yamaguchi S, Yokoyama T, Kamei Y, Anil SM, et al. Dorsal column stimulation in persistent vegetative state. Neuromodulation. 2009;12(1):33-8. doi: 10.1111/j.1525-1403.2009.00185.x.

811.Kljajević V. fMRI in language recovery after stroke. Acta Facultatis Medicae Naissensis. 2009;26(4):217-23.

812. Koenigs M, Barbey AK, Postle BR, Grafman J. Superior Parietal Cortex Is Critical for the Manipulation of Information in Working Memory. Journal of Neuroscience. 2009;29(47):14980-6. doi: 10.1523/jneurosci.3706-09.2009. PubMed PMID: WOS:000272185100030.

813. Kranz G, Shamim EA, Lin PT, Kranz GS, Voller B, Hallett M. Blepharospasm and the modulation of cortical excitability in primary and secondary motor areas. Neurology. 2009;73(23):2031-6. doi:

10.1212/WNL.0b013e3181c5b42d.

814. Lapitska N, Gosseries O, Delvaux V, Overgaard M, Nielsen F, Maertens de Noordhout A, et al. Transcranial magnetic stimulation in disorders of consciousness. Rev Neurosci. 2009;20(3-4):235-50. Epub 2010/02/18. doi: 10.1515/revneuro.2009.20.3-4.235. PubMed PMID: 20157993.

815. Lapitskaya N, Coleman MR, Nielsen JF, Gosseries O, de Noordhout AM. Disorders of consciousness: further pathophysiological insights using motor cortex transcranial magnetic stimulation. Prog Brain Res. 2009;177:191-200. Epub 2009/10/13. doi: 10.1016/s0079-6123(09)17713-0. PubMed PMID: 19818902.

816. Lapitskaya N, Gosseries O, Pasqua VD, De Noordhout AM, Nielsen JF, Moonen G, et al. Reduced short latency afferent inhibition in severe brain injury patients with impaired consciousness. Journal of Neurology. 2009;256:S20. doi: 10.1007/s00415-009-5161-z.

817. Lepilin AV, Erokina NL, Rogatina TV, Khlusov I. [Using dynamic magnetotherapy and transdermal electroneurostimulation in the combined treatment of patients with mandibular fractures and concomitant periodontal inflammatory diseases]. Vopr Kurortol Fizioter Lech Fiz Kult. 2009(4):37-40. Epub 2009/08/28. PubMed PMID: 19711518.

818. Liebetanz D, Koch R, Mayenfels S, König F, Paulus W,

Nitsche MA. Safety limits of cathodal transcranial direct current stimulation in rats. Clin Neurophysiol. 2009;120(6):1161-7. Epub 2009/05/01. doi: 10.1016/j.clinph.2009.01.022. PubMed PMID: 19403329.

819. Liu J-T, Lee J-K, Tyan Y-S, Liu C-Y, Chen Y-H, Lin T-B. Neuromodulation on cervical spinal cord combined with hyperbaric oxygen in comatose patients-a preliminary report. Surgical Neurology. 2009;72:28-35. doi: 10.1016/j.surneu.2009.05.029. PubMed PMID: WOS:000279234100004.

820. Liu JT, Lee JK, Tyan YS, Liu CY, Chen YH, Lin TB. Neuromodulation on cervical spinal cord combined with hyperbaric oxygen in comatose patients--a preliminary report. Surg Neurol. 2009;72 Suppl 2:S28-34; discussion S-5. Epub 2009/08/12. doi: 10.1016/j.surneu.2009.05.029. PubMed PMID: 19665182.

821. Livingston SC, Hertel JN, Saliba EN, Barth JT, Goodkin HP, Ingersoll CD. Motor-evoked potential amplitudes are correlated with prior number of concussions in the acutely concussed collegiate athlete. Journal of Head Trauma Rehabilitation. 2009;24(5):400-1. doi: 10.1097/HTR.0b013e3181bd6769.

822. Lo YL, Fook-Chong S, Chan LL, Ong WY. Cerebellar control of motor activation and cancellation in humans: An electrophysiological study. Cerebellum. 2009;8(3):302-11. doi:

10.1007/s12311-009-0095-7.
823. Louise-Bender Pape T, Rosenow J, Lewis G, Ahmed G, Walker M, Guernon A, et al. Repetitive transcranial magnetic stimulation-associated neurobehavioral gains during coma recovery. Brain Stimul. 2009;2(1):22-35. Epub 2009/01/01. doi: 10.1016/j.brs.2008.09.004. PubMed PMID: 20633400.
824. Lusardi TA. Adenosine neuromodulation and traumatic brain injury. Curr Neuropharmacol. 2009;7(3):228-37. Epub 2010/03/02. doi: 10.2174/157015909789152137. PubMed PMID: 20190964; PubMed Central PMCID: PMCPMC2769006.
825. MacDonald SW, Li SC, Bäckman L. Neural underpinnings of within-person variability in cognitive functioning. Psychol Aging. 2009;24(4):792-808. Epub 2009/12/23. doi: 10.1037/a0017798. PubMed PMID: 20025396.
826. Maguire S, Pickerd N, Farewell D, Mann M, Tempest V, Kemp AM. Which clinical features distinguish inflicted from non-inflicted brain injury? A systematic review. Arch Dis Child. 2009;94(11):860-7. Epub 2009/06/18. doi: 10.1136/adc.2008.150110. PubMed PMID: 19531526.
827. Martin RCG, Joshi J, Robbins K, Tomalty D, O'Hara R, Tatum C. Transarterial chemoembolization of metastatic colorectal carcinoma with drug-eluting beads, irinotecan (DEBIRI): Multi-institutional

registry. Journal of Oncology. 2009. doi: 10.1155/2009/539795.

828. Massimini M, Boly M, Casali A, Rosanova M, Tononi G. A perturbational approach for evaluating the brain's capacity for consciousness. Prog Brain Res. 2009;177:201-14. Epub 2009/10/13. doi: 10.1016/s0079-6123(09)17714-2. PubMed PMID: 19818903.

829. Meera P, Olsen RW, Otis TS, Wallner M. Etomidate, propofol and the neurosteroid THDOC increase the GABA efficacy of recombinant α4β3δ and α4β3 GABAA receptors expressed in HEK cells. Neuropharmacology. 2009;56(1):155-60. doi: 10.1016/j.neuropharm.2008.08.011.

830. Moerk SK, Feldbæk J, Lapitskaya N. Cortical excitability in patients with brain damage after anoxia. European Journal of Neurology. 2009;16(S3):317. doi: 10.1111/j.1468-1331.2009.02821.x.

831. Monier A, Pelc K, Willems A, Dachy B, Guissard G, Dan B. Paediatric brainstem stroke: A rare life-threatening condition. European Journal of Paediatric Neurology. 2009;13:S96. doi: 10.1016/S1090-3798(09)70300-9.

832. Mori F, Koch G, Foti C, Bernardi G, Centonze D. The use of repetitive transcranial magnetic stimulation (rTMS) for the treatment of spasticity. In: Verhaagen J, Hol EM, Huitenga I, Wijnholds J, Bergen AB, Boer GJ, et al., editors. Neurotherapy: Progress in Restorative Neuroscience and Neurology. Progress in Brain Research.

1752009. p. 429-39.
833. Morrison LJ, Rizoli SB, Schwartz B, Rhind SG, Simitciu M, Perreira T, et al. The Toronto prehospital hypertonic resuscitation-head injury and multi organ dysfunction trial (TOPHR HIT) - Methods and data collection tools. Trials. 2009;10. doi: 10.1186/1745-6215-10-105.
834. Muresanu DF. Neuromodulation with pleiotropic and multimodal drugs - Future approaches to treatment of neurological disorders. Acta Neurochirurgica, Supplementum2009. p. 291-4.
835. Nct. Using Repetitive Transcranial Magnetic Stimulation (rTMS) in the Rehabilitation of Chronic Spatial Neglect After Stroke. https://clinicaltrialsgov/show/NCT01000103. 2009. PubMed PMID: CN-01526424.
836. Nowak DA. Brain stimulation in the rehabilitation of impaired hand function following stroke. Aktuelle Neurologie. 2009;36(8):397-411. doi: 10.1055/s-0029-1220365.
837. Ozturk M, Komurcu S, Kilic S, Ozet A, Arpaci F, Ozturk B, et al. Self-reported experience of mucositis in cancer patients who underwent conditioning regimen and stem cell transplantation. Support Care Cancer. 2009;17(10):1295-9. Epub 2009/02/10. doi: 10.1007/s00520-009-0586-6. PubMed PMID: 19198890.
838. Park S, Ko M, Seo J. Motor reorganisation with transcranial stimulation of motor cortex in children with hemiplegic cerebral palsy.

Developmental Medicine and Child Neurology. 2009;51:57. doi: 10.1111/j.1469-8749.2009.03452-2.x.
839. Peyrot M, Rubin RR. Patient reported outcomes in adults with type 2 diabetes using mealtime AFRESA™ (Inhaled technosphere® insulin) or Metformin+Secretagogue or both. Diabetes. 2009;58.
840. Proto D, Pella RD, Hill BD, Gouvier WD. Assessment and rehabilitation of acquired visuospatial and proprioceptive deficits associated with visuospatial neglect. NeuroRehabilitation. 2009;24(2):145-57. doi: 10.3233/nre-2009-0463. PubMed PMID: MEDLINE:19339753.
841. Rasche D, Tronnier VM. Invasive treatment of chronic neuropathic pain syndromes: Epidural stimulation of the motor cortex. Journal of Pain Management. 2009;2(3):315-26.
842. Reis J, Schambra HM, Cohen LG, Buch ER, Fritsch B, Zarahn E, et al. Noninvasive cortical stimulation enhances motor skill acquisition over multiple days through an effect on consolidation. Proceedings of the National Academy of Sciences of the United States of America. 2009;106(5):1590-5. doi: 10.1073/pnas.0805413106. PubMed PMID: WOS:000263074600054.
843. Rizvi SJ, Kennedy SH, McNeely H, Giacobbe P, Mayberg HS, Lozano AM. Functional outcome after 12 months of deep brain stimulation for treatment resistant major depressive disorder.

European Neuropsychopharmacology. 2009;19:S388-S9. doi: 10.1016/S0924-977X(09)70598-X.

844. Rotenberg A, Pascual-Leone A. Safety of 1 Hz repetitive transcranial magnetic stimulation (rTMS) in patients with titanium skull plates. Clinical Neurophysiology. 2009;120(7):1417-. doi: 10.1016/j.clinph.2009.05.004. PubMed PMID: WOS:000268841700026.

845. Singh S, Mistry S, Jefferson S, Davies K, Rothwell J, Williams S, et al. A Magnetic Resonance Spectroscopy Study of Brain Glutamate in a Model of Plasticity in Human Pharyngeal Motor Cortex. Gastroenterology. 2009;136(2):417-24. doi: 10.1053/j.gastro.2008.10.087. PubMed PMID: WOS:000262967700013.

846. Sirén AL, Faßhauer T, Bartels C, Ehrenreich H. Therapeutic Potential of Erythropoietin and its Structural or Functional Variants in the Nervous System. Neurotherapeutics. 2009;6(1):108-27. doi: 10.1016/j.nurt.2008.10.041.

847. Stasiukyniene V, Pilvinis V, Reingardiene D, Janauskaite L. [Epileptic seizures in critically ill patients]. Medicina (Kaunas). 2009;45(6):501-7. Epub 2009/07/17. PubMed PMID: 19605972.

848. Struppler A, Angerer B, Gebhard B. Repetitive peripheral magnetic stimulation (RPMS) as a method for the rehabilitation of

sensorimotor deficits of hand and arm following cerebral lesions. Neurologie und Rehabilitation. 2009;15(1):28-38.

849. Suppiej A, Cappellari A, Cogo PE. Prognostic role of somatosensory and auditory evoked potentials in paediatric hypoxic-ischemic encephalopathy managed with hypothermia: an illustrative case. Neurophysiol Clin. 2009;39(2):101-5. Epub 2009/05/27. doi: 10.1016/j.neucli.2009.02.003. PubMed PMID: 19467440.

850. Tsuchida A, Fellows LK. Lesion Evidence That Two Distinct Regions within Prefrontal Cortex are Critical for n-Back Performance in Humans. Journal of Cognitive Neuroscience. 2009;21(12):2263-75. doi: 10.1162/jocn.2008.21172. PubMed PMID: WOS:000272351200001.

851. Vandermeeren Y, Davare M, Duque J, Olivier E. Reorganization of cortical hand representation in congenital hemiplegia. Eur J Neurosci. 2009;29(4):845-54. Epub 2009/02/10. doi: 10.1111/j.1460-9568.2009.06619.x. PubMed PMID: 19200077.

852. Vollmer B. Investigation of neural correlates of cerebral palsy. Developmental Medicine and Child Neurology. 2009;51:11. doi: 10.1111/j.1469-8749.2009.03328-1.x.

853. Vollmer B, Holmström L, Lennartsson F, Tedroff K, Forssberg H, Persson J, et al. Fractional anisotropy in the cortico-spinal tract,

motor projection patterns, and hand motor outcome in children with unilateral cerebral palsy (CP) - Preliminary report. Developmental Medicine and Child Neurology. 2009;51:78-9. doi: 10.1111/j.1469-8749.2008.03237.x.

854. Watts P, Bowden N, Farewell D, Maguire S, Kemp A. Retinal hemorrhages defining the probability of inflicted brain injury: A systematic review. Journal of AAPOS. 2009;13(1):e30-e1. doi: 10.1016/j.jaapos.2008.12.092.

855. Williams KM, Mella H, Lucas PJ, Williams JA, Telford W, Gress RE. Single cell analysis of complex thymus stromal cell populations: rapid thymic epithelia preparation characterizes radiation injury. Clin Transl Sci. 2009;2(4):279-85. Epub 2009/09/15. doi: 10.1111/j.1752-8062.2009.00128.x. PubMed PMID: 19750208; PubMed Central PMCID: PMCPMC2741333.

856. Xie GH, Fang XM. Importance of recognizing and managing delirium in intensive care unit. Chinese Journal of Traumatology - English Edition. 2009;12(6):370-4. doi: 10.3760/cma.j.issn.1008-1275.2009.06.009.

857. Xu W, Gao Q, Xu Y, Wu D, Sun Y, Shen W, et al. Controllable release of ibuprofen from size-adjustable and surface hydrophobic mesoporous silica spheres. Powder Technology. 2009;191(1-2):13-20. doi: 10.1016/j.powtec.2008.09.001. PubMed PMID:

WOS:000264656900003.
858. Yamashita O, Odahara H, Ochi T, Shigeto J-i. Dependence of the resultant Seebeck coefficient on the thickness of Bi0.88Sb0.12 alloy in welded M/Bi-Sb/M (M = Cu and Ni) composites. Applied Physics a-Materials Science & Processing. 2009;97(1):133-41. doi: 10.1007/s00339-009-5300-5. PubMed PMID: WOS:000269206600019.
859. Yuan Q. Theta bursts in the olfactory nerve paired with beta-adrenoceptor activation induce calcium elevation in mitral cells: a mechanism for odor preference learning in the neonate rat. Learn Mem. 2009;16(11):676-81. Epub 2009/10/28. doi: 10.1101/lm.1569309. PubMed PMID: 19858361; PubMed Central PMCID: PMCPMC2775516.
860. Zafonte R, Hammond F, Dennison A, Chew E. Pharmacotherapy to enhance arousal: what is known and what is not. In: Laureys S, Schiff ND, Owen AM, editors. Coma Science: Clinical and Ethical Implications. Progress in Brain Research. 1772009. p. 293-316.
861. Zaghi S, Heine N, Fregni F. Brain stimulation for the treatment of pain: A review of costs, clinical effects, and mechanisms of treatment for three different central neuromodulatory approaches. J Pain Manag. 2009;2(3):339-52. Epub 2010/06/30. PubMed PMID:

20585474; PubMed Central PMCID: PMCPMC2888303.

862. Selected Abstracts of Conference Presentations at the 2010 International Society for Neurofeedback and Research, ISNR, 18th Annual Conference. Journal of Neurotherapy. 2010;14(4).

863. Society Proceedings: 9th Congress of Clinical Neurophysiology Society of Serbia and Montenegro with International Participation. Clinical Neurophysiology. 2010;121(4).

864. Abo M, Kakuda W. Neuroimaging and neurorehabilitation for aphasia. Brain and Nerve. 2010;62(2):141-9.

865. Alfieri FM, de Jesus Guirro RR, Teodori RM. Postural stability of elderly submmitted to multisensorial physical therapy intervention. Electromyogr Clin Neurophysiol. 2010;50(2):113-9. Epub 2010/04/22. PubMed PMID: 20405787.

866. An HS, Cho BM, Kang JH, Kim MK, Oh SM, Park SH. Efficacy of low dose barbiturate coma therapy for the patients with intractable intracranial hypertension using the bispectral™ index monitoring. Journal of Korean Neurosurgical Society. 2010;47(4):252-7. doi: 10.3340/jkns.2010.47.4.252.

867. Andrade P, Carrillo-Ruiz JD, Ramírez Y, Jiménez F. Effects of thalamic reticular nucleus electrical stimulation in rats in a t-maze perseverative behavior model induced by 8-OH-DPAT. Neuromodulation. 2010;13(1):2-9. doi:

10.1111/j.1525-1403.2009.00242.x.

868. Barañano CF, Sweitzer RS, Mahalak ML, Alexander NS, Woolley AL. The management of myringotomy tubes in pediatric cochlear implant recipients. Arch Otolaryngol Head Neck Surg. 2010;136(6):557-60. Epub 2010/06/23. doi: 10.1001/archoto.2010.80. PubMed PMID: 20566905.

869. Bashir S, Mizrahi I, Weaver K, Fregni F, Pascual-Leone A. Assessment and modulation of neural plasticity in rehabilitation with transcranial magnetic stimulation. Pm r. 2010;2(12 Suppl 2):S253-68. Epub 2011/02/09. doi: 10.1016/j.pmrj.2010.10.015. PubMed PMID: 21172687; PubMed Central PMCID: PMCPMC3951769.

870. Belverud S, Mogilner A, Schulder M. Intrathecal drug delivery by implanted pumps. 2010. p. 137-53.

871. Cakar E, Isilak Z, Kiralp MZ, Akyuz G. Low frequency rTMS is a safe method for poststroke patients who had atrial fibrillation: A case report. Brain Injury. 2010;24(3):433. doi: 10.3109/02699051003648227.

872. Camprodon J, Sack A, Pascual-Leone A. Developing strategies for therapeutic neuromodulation: Simultaneous transcranial magnetic stimulation and functional magnetic resonance imaging to study the mechanisms of hemispatial neglect recovery. Neuropsychopharmacology. 2010;35:S272. doi:

10.1038/npp.2010.217.

873. Carmel JB, Berrol LJ, Brus-Ramer M, Martin JH. Chronic Electrical Stimulation of the Intact Corticospinal System after Unilateral Injury Restores Skilled Locomotor Control and Promotes Spinal Axon Outgrowth. Journal of Neuroscience. 2010;30(32):10918-26. doi: 10.1523/jneurosci.1435-10.2010. PubMed PMID: WOS:000280795900031.

874. Casali AG, Casarotto S, Rosanova M, Mariotti M, Massimini M. General indices to characterize the electrical response of the cerebral cortex to TMS. Neuroimage. 2010;49(2):1459-68. doi: 10.1016/j.neuroimage.2009.09.026. PubMed PMID: WOS:000272808400030.

875. Chang P, Levy RM. High lateral cervical spinal cord stimulation (SCS) for neuropathic facial pain: Report of 10 cases. Neurosurgery. 2010;67(2):550. doi: 10.1227/01.NEU.0000387015.37565.43.

876. Cohen L. Physiological substrates underlying recovery of motor function after stroke. Clinical Neurophysiology. 2010;121:S48. doi: 10.1016/S1388-2457(10)60202-7.

877. Cooper E, Cooper B. Right median nerve stimulation for coma treatment. Brain Injury. 2010;24(3):223-4. doi: 10.3109/02699051003648227.

878. Cosentino G, Giglia G, Palermo A, Panetta ML, Lo Baido R, Brighina F, et al. A case of post-traumatic complex auditory hallucinosis treated with rTMS. Neurocase. 2010;16(3):267-72. Epub 2010/01/28. doi: 10.1080/13554790903456191. PubMed PMID: 20104391.

879. D'Agati D, Bloch Y, Levkovitz Y, Reti I. rTMS for adolescents: Safety and efficacy considerations. Psychiatry Research. 2010;177(3):280-5. doi: 10.1016/j.psychres.2010.03.004. PubMed PMID: WOS:000278659600002.

880. Dagi TF. Consenting to the ineffable: the problem of neuromodulation and altered consciousness. J Clin Ethics. 2010;21(2):140-2. Epub 2010/09/28. PubMed PMID: 20866020.

881. Datta A, Bikson M, Fregni F. Transcranial direct current stimulation in patients with skull defects and skull plates: high-resolution computational FEM study of factors altering cortical current flow. Neuroimage. 2010;52(4):1268-78. Epub 2010/05/04. doi: 10.1016/j.neuroimage.2010.04.252. PubMed PMID: 20435146; PubMed Central PMCID: PMCPMC2910315.

882. De Fina P, Thompson J, Fellus J, Moser R, Schatz P, Eller M, et al. EEG correlates of improved outcomes from SDOC using a novel treatment protocol. Journal of Neurotherapy. 2010;14(4):355-7. doi: 10.1080/10874208.2010.52335.

883. DeFina PA, Fellus J, Thompson JW, Eller M, Moser RS, Frisina PG, et al. Improving outcomes of severe disorders of consciousness. Restor Neurol Neurosci. 2010;28(6):769-80. Epub 2011/01/07. doi: 10.3233/rnn-2010-0548. PubMed PMID: 21209492.

884. Derakhshan I, Reesman S. A reappraisal of the kernohan and woltman's article on ipsilateral pyramidal signs: New insight denies role of herniation in favor of deafferentation of the minor hemisphere. Brain Injury. 2010;24(3):118-9. doi: 10.3109/02699051003648227.

885. Dinomais M, Gröschel S, Wilke M, Staudt M, Krägeloh-Mann I. Somatosensory system in congenital hemiparesis: Clinical assessment and resting state functional connectivity MRI analysis. Neuropediatrics. 2010;41(2). doi: 10.1055/s-0030-1265521.

886. Driver J, Blankenburg F, Bestmann S, Ruff CC. New approaches to the study of human brain networks underlying spatial attention and related processes. Exp Brain Res. 2010;206(2):153-62. Epub 2010/04/01. doi: 10.1007/s00221-010-2205-7. PubMed PMID: 20354681; PubMed Central PMCID: PMCPMC2940032.

887. Ferrarelli F, Massimini M, Sarasso S, Casali A, Riedner BA, Angelini G, et al. Breakdown in cortical effective connectivity during midazolam-induced loss of consciousness. Proc Natl Acad Sci U S A. 2010;107(6):2681-6. Epub 2010/02/06. doi: 10.1073/pnas.0913008107. PubMed PMID: 20133802; PubMed

Central PMCID: PMCPMC2823915.

888. Finn DP. International Cannabinoid Research Society - 20th annual symposium. IDrugs. 2010;13(10):677-9.

889. Fleming MK, Stinear CM, Byblow WD. Bilateral parietal cortex function during motor imagery. Experimental Brain Research. 2010;201(3):499-508. doi: 10.1007/s00221-009-2062-4. PubMed PMID: WOS:000275414500013.

890. Fountoulakis KN. The emerging modern face of mood disorders: A didactic editorial with a detailed presentation of data and definitions. Annals of General Psychiatry. 2010;9. doi: 10.1186/1744-859X-9-14.

891. Gall C, Fedorov AB, Ernst L, Borrmann A, Sabel BA. Repetitive transorbital alternating current stimulation in optic neuropathy. NeuroRehabilitation. 2010;27(4):335-41. Epub 2010/12/17. doi: 10.3233/nre-2010-0617. PubMed PMID: 21160123.

892. Gamé X, Khan S, Panicker J, Hamid R, Elneil S, Fowler C. 2-stage sacral neuromodulation for treating chronic urinary retention in women: Outcome and response prognostic factors. Neurourology and Urodynamics. 2010;29(6):1081-3. doi: 10.1002/nau.20973.

893. Gao G, Jiang J, Cooper E. Traumatic coma: What to do after we made it. Brain Injury. 2010;24(3):153. doi: 10.3109/02699051003648227.

894. Garcia-Larrea L. Motor cortex stimulation (MCS) for neuropathic pain: Can we proceed from phenomenology to mechanisms? European Journal of Pain Supplements. 2010;4(1):11. doi: 10.1016/S1754-3207(10)70039-0.

895. George MS, Aston-Jones G. Noninvasive techniques for probing neurocircuitry and treating illness: vagus nerve stimulation (VNS), transcranial magnetic stimulation (TMS) and transcranial direct current stimulation (tDCS). Neuropsychopharmacology. 2010;35(1):301-16. doi: 10.1038/npp.2009.87. PubMed PMID: WOS:000272784600016.

896. George MS, Lisanby SH, Avery D, McDonald WM, Durkalski V, Pavlicova M, et al. Daily left prefrontal transcranial magnetic stimulation therapy for major depressive disorder: A sham-controlled randomized trial. Archives of General Psychiatry. 2010;67(5):507-16. doi: 10.1001/archgenpsychiatry.2010.46.

897. Georgiopoulos M, Katsakiori P, Kefalopoulou Z, Ellul J, Chroni E, Constantoyannis C. Vegetative state and minimally conscious state: a review of the therapeutic interventions. Stereotact Funct Neurosurg. 2010;88(4):199-207. Epub 2010/05/13. doi: 10.1159/000314354. PubMed PMID: 20460949.

898. Gooch CM, Wiener M, Wencil EB, Coslett HB. Interval timing disruptions in subjects with cerebellar lesions.

Neuropsychologia. 2010;48(4):1022-31. doi: 10.1016/j.neuropsychologia.2009.11.028. PubMed PMID: WOS:000275933500020.

899. Harvey RL. Central Poststroke Pain Syndrome. Topics in Stroke Rehabilitation. 2010;17(3):163-72. doi: 10.1310/tsr1703-163. PubMed PMID: WOS:000280229200003.

900. Henderson JM, Heit G, Fisher RS. Recurrent seizures related to motor cortex stimulator programming. Neuromodulation. 2010;13(1):37-43. doi: 10.1111/j.1525-1403.2009.00256.x.

901. Hirota N, Yasuda D, Hashidate T, Yamamoto T, Yamaguchi S, Nagamune T, et al. Amino acid residues critical for endoplasmic reticulum export and trafficking of platelet-activating factor receptor. Journal of Biological Chemistry. 2010;285(8):5931-40. doi: 10.1074/jbc.M109.066282.

902. Hochman G, Yechiam E, Bechara A. Recency gets larger as lesions move from anterior to posterior locations within the ventromedial prefrontal cortex. Behavioural Brain Research. 2010;213(1):27-34. doi: 10.1016/j.bbr.2010.04.023.

903. Holmström L, Vollmer B, Tedroff K, Islam M, Persson JK, Kits A, et al. Hand function in relation to brain lesions and corticomotor-projection pattern in children with unilateral cerebral palsy. Dev Med Child Neurol. 2010;52(2):145-52. Epub 2009/10/08.

doi: 10.1111/j.1469-8749.2009.03496.x. PubMed PMID: 19807768.

904. Hori T, Oike F, Hata K, Nishikiori M, Ogura Y, Ogawa K, et al. Hashimoto's encephalopathy after interferon therapy for hepatitis C virus in adult liver transplant recipient accompanied by post-transplant lymphoproliferative disorder related to Epstein-Barr virus infection: Case report. Transplant Infectious Disease. 2010;12(4):347-52. doi: 10.1111/j.1399-3062.2010.00508.x.

905. Hummel F. Mechanisms of stroke recovery: Non-invasive brain stimulation to enhance neuronal plasticity and functional recovery. Clinical Neurophysiology. 2010;121:S49. doi: 10.1016/S1388-2457(10)60204-0.

906. Irina S, Tatyana B, Tatyana I, Vadim I, Tatyana S, Konstantin L. Application of transcranial magnetic stimulation in diagnostics and treatment of patients in vegetative state. Brain Injury. 2010;24(3):316. doi: 10.3109/02699051003648227.

907. Jang SH. Unusual long-term motor recovery in a patient with corona radiata infarct. Neural Regeneration Research. 2010;5(17):1353-6. doi: 10.3969/j.issn.1673-5374.2010.17.013. PubMed PMID: WOS:000283641200013.

908. Jang SH, Hong JH, Ahn SH, Son SM, Cho YW. Motor function reorganization in a patient with a brainstem lesion: DTT, fMRI and TMS study. NeuroRehabilitation. 2010;26(2):167-71. Epub

2010/03/06. doi: 10.3233/nre-2010-0550. PubMed PMID: 20203384.

909. Jayasekeran V, Rothwell J, Hamdy S. A neurostimulation study of the human cerebellum in the control of swallowing motor pathways. Gastroenterology. 2010;138(5):S371.

910. Jayasekeran V, Rothwell J, Hamdy S. Facilitation of human cortical swallowing pathways by stimulation of the cerebellum. Gut. 2010;59:A29-A30. doi: 10.1136/gut.2009.208975s.

911.Kang DW, Kang JH, Lee MS, Chang JW. Posttraumatic hemidystonia treated with unilateral globus pallidus interna stimulation: Long-term follow-up and radiologic features. Neuromodulation. 2010;13(4):261-4. doi: 10.1111/j.1525-1403.2010.00306.x.

912. Kantelhardt SR, Fadini T, Finke M, Kallenberg K, Siemerkus J, Bockermann V, et al. Robot-assisted image-guided transcranial magnetic stimulation for somatotopic mapping of the motor cortex: A clinical pilot study. Acta Neurochirurgica. 2010;152(2):333-43. doi: 10.1007/s00701-009-0565-1.

913. Kimiskidis VK, Kugiumtzis D, Papagiannopoulos SK, Kazis DA, Vasiliadis G, Oikonomidi A, et al. Transcranial Magnetic Stimulation terminates epileptiform discharges in patients with partial epilepsy: A combined EEG-TMS study. Clinical Neurophysiology. 2010;121:S168. doi: 10.1016/S1388-2457(10)60691-8.

914. Knikou M. Neural control of locomotion and training-induced plasticity after spinal and cerebral lesions. Clinical Neurophysiology. 2010;121(10):1655-68. doi: 10.1016/j.clinph.2010.01.039. PubMed PMID: WOS:000281435800007.

915. Koivisto M, Mäntylä T, Silvanto J. The role of early visual cortex (V1/V2) in conscious and unconscious visual perception. NeuroImage. 2010;51(2):828-34. doi: 10.1016/j.neuroimage.2010.02.042.

916. Lancioni GE, Bosco A, Belardinelli MO, Singh NN, O'Reilly MF, Sigafoos J. An overview of intervention options for promoting adaptive behavior of persons with acquired brain injury and minimally conscious state. Research in developmental disabilities. 2010;31(6):1121-34. doi: 10.1016/j.ridd.2010.06.019. PubMed PMID: MEDLINE:20663643.

917. Laureys S. Brain imaging studies of self processing. Journal of Neurology, Neurosurgery and Psychiatry. 2010;81(10):e12-e3. doi: 10.1136/jnnp.2010.217554.30.

918. Lauritzen M, Wanscher B, Smith T. Predictive value of median nerve SEP and EEG after anoxic brain injury. Clinical Neurophysiology. 2010;121:S186-S7. doi: 10.1016/S1388-2457(10)60766-3.

919. Lee R-S, Li H-R, Chiu F-C. Synthesis of Amphiphilic

Pseudopolyamino Acid-Containing ABC-Triblock Copolymers and Micellar Characterizations. Journal of Applied Polymer Science. 2010;115(5):2556-64. doi: 10.1002/app.31213. PubMed PMID: WOS:000274114000004.

920. Leone M, Franzini A, Proietti Cecchini A, Mea E, Broggi G, Bussone G. Deep brain stimulation in trigeminal autonomic cephalalgias. Neurotherapeutics. 2010;7(2):220-8. Epub 2010/05/01. doi: 10.1016/j.nurt.2010.02.001. PubMed PMID: 20430322; PubMed Central PMCID: PMCPMC5084104.

921. Levy R, Deer TR, Henderson J. Intracranial neurostimulation for pain control: a review. Pain Physician. 2010;13(2):157-65. Epub 2010/03/24. PubMed PMID: 20309382.

922. Liu JP, Yang ZL, Wang MS, Shi R, Zhu BP. [Observation on therapeutic effect of electroacupuncture therapy for promoting consciousness of patients with coma]. Zhongguo Zhen Jiu. 2010;30(3):206-8. Epub 2010/05/26. PubMed PMID: 20496735.

923. Liuzzi G, Freundlieb N, Ridder V, Hoppe J, Heise K, Zimerman M, et al. The involvement of the left motor cortex in learning of a novel action word lexicon. Current Biology. 2010;20(19):1745-51. doi: 10.1016/j.cub.2010.08.034.

924. Livingston SC, Saliba EN, Goodkin HP, Barth JT, Hertel JN, Ingersoll CD. A preliminary investigation of motor evoked potential

abnormalities following sport-related concussion. Brain Inj. 2010;24(6):904-13. Epub 2010/05/04. doi: 10.3109/02699051003789245. PubMed PMID: 20433286.

925. Lou HC, Gross J, Biermann-Ruben K, Kjaer TW, Schnitzler A. Coherence in Consciousness: Paralimbic Gamma Synchrony of Self-Reference Links Conscious Experiences. Human Brain Mapping. 2010;31(2):185-92. doi: 10.1002/hbm.20855. PubMed PMID: WOS:000274460900002.

926. Maller JJ, Thomson RHS, Lewis PM, Rose SE, Pannek K, Fitzgerald PB. Traumatic brain injury, major depression, and diffusion tensor imaging: Making connections. Brain Research Reviews. 2010;64(1):213-40. doi: 10.1016/j.brainresrev.2010.04.003. PubMed PMID: WOS:000279291800009.

927. Miyai I. Neural mechanisms underlying functional recovery after stroke. Clinical Neurophysiology. 2010;121:S48-S9. doi: 10.1016/S1388-2457(10)60203-9.

928. Nct. Transcranial Magnetic Stimulation (TMS) for Suicidal Ideation. https://clinicaltrialsgov/show/NCT01212848. 2010. PubMed PMID: CN-02022083.

929. Nct. Effects of rTMS on the Cognition of Elderly With Mild Memory Complaints. https://clinicaltrialsgov/show/NCT01292382. 2010. PubMed PMID: CN-01531886.

930. Nuutinen S, Panula P. Histamine in neurotransmission and brain diseases. Advances in Experimental Medicine and Biology2010. p. 95-107.

931. Pallanti S. Unique contributions of brain stimulation to the study of consciousness: where neuroscience meets philosophy. CNS Spectr. 2010;15(3):154-6. Epub 2010/04/24. doi: 10.1017/s1092852900027425. PubMed PMID: 20414164.

932. Paust HJ, Riedel JH, Turner JE, Peters A, Steinmetz OM, Stahl RAK, et al. IFNγ and IL-17 have contrary effects on Th1-and Th17-cell-attracting chemokines. NDT Plus. 2010;3:iii124.

933. Peng F, Chen ZQ, Luo JK. [Clinical observation on continuous electroacupuncture at Neiguan (PC 6) for arousing consciousness of comatose patients with severe craniocerebral trauma]. Zhongguo Zhen Jiu. 2010;30(6):465-8. Epub 2010/06/29. PubMed PMID: 20578383.

934. Plowman EK, Kleim JA. Motor cortex reorganization across the lifespan. Journal of communication disorders. 2010;43(4):286-94. doi: 10.1016/j.jcomdis.2010.04.005. PubMed PMID: MEDLINE:20478572.

935. Polanowska K, Seniów J. [Influence of transcranial direct current stimulation on cognitive functioning of patients with brain injury]. Neurol Neurochir Pol. 2010;44(6):580-90. Epub 2011/01/13.

doi: 10.1016/s0028-3843(14)60156-0. PubMed PMID: 21225521.
936. Polito MZ, Thompson JWG, DeFina PA. A review of the International Brain Research Foundation novel approach to mild traumatic brain injury presented at the International Conference on Behavioral Health and Traumatic Brain Injury. Journal of the American Academy of Nurse Practitioners. 2010;22(9):504-9. doi: 10.1111/j.1745-7599.2010.00540.x. PubMed PMID: WOS:000281660700009.
937. Pope C, Mechoulam R, Parsons L. Endocannabinoid signaling in neurotoxicity and neuroprotection. Neurotoxicology. 2010;31(5):562-71. doi: 10.1016/j.neuro.2009.12.002. PubMed PMID: WOS:000281939200020.
938. Quemada JI, De Francisco ML, Echeburúa E, Bulbena A. Impact of psychiatric and neuropsychological consequences of traumatic brain injury on the duration of the legal periods. Actas Espanolas de Psiquiatria. 2010;38(2):101-7.
939. Rees G, Seth AK. The cognitive neuroscience of consciousness. Cognitive Neuroscience. 2010;1(3):153-4. doi: 10.1080/17588928.2010.503602.
940. Reidler JS, Nascimento BC, Wu DSK, Carvas M, Massuyama BK, Oliveira BMRd, et al. Modulation of inhibitory systems to enhance motor rehabilitation: insights for the use of noninvasive brain

stimulation. Psychology & Neuroscience. 2010;3(2):151-60. doi: 10.3922/j.psns.2010.2.004. PubMed PMID: SCIELO:S1983-32882010000200004.

941. Robinson LR, Micklesen PJ. Does stimulus rate matter when performing somatosensory evoked potentials for coma patients? Neurocrit Care. 2010;12(1):69-73. Epub 2009/02/20. doi: 10.1007/s12028-009-9191-2. PubMed PMID: 19225910.

942. Rose S, Guzzetta A, Tournier D, Burke S, Sakzewski L, Jackson G, et al. Relationship between brain microstructure and upper limb motor and sensory function in congenital hemiplegia. Developmental Medicine and Child Neurology. 2010;52:32-3. doi: 10.1111/j.1469-8749.2010.03682.x.

943. Rossi S, Ragazzoni A, Pirulli C, Veniero D, Miniussi C, Feurra M, et al. Cortical functional connectivity in the Vegetative State and Minimally Conscious State: An investigation with TMS-Evoked Potentials (TEPs). Clinical Neurophysiology. 2010;121:S170. doi: 10.1016/S1388-2457(10)60696-7.

944. Rossini PM. Neuronal plasticity in human. Clinical Neurophysiology. 2010;121:S1. doi: 10.1016/S1388-2457(10)60004-1.

945. Roth TM. Sacral neuromodulation and cardiac pacemakers. International Urogynecology Journal. 2010;21(8):1035-7. doi:

10.1007/s00192-009-1091-7.

946. Scavarda D, Gabaudan C, Ughetto F, Lamy F, Imada V, Lena G, et al. Initial predictive factors of outcome in severe non-accidental head trauma in children. Child's Nervous System. 2010;26(11):1555-61. doi: 10.1007/s00381-010-1150-x.

947. Schambra H, Abe M, Luckenbaugh D, Reis J, Cohen L. Motor skill learning is differentially facilitated by left and right motor cortex (MC) stimulation. Annals of Neurology. 2010;68:S60. doi: 10.1002/ana.22175.

948. Shah SA, Schiff ND. Central thalamic deep brain stimulation for cognitive neuromodulation - a review of proposed mechanisms and investigational studies. Eur J Neurosci. 2010;32(7):1135-44. Epub 2010/11/03. doi: 10.1111/j.1460-9568.2010.07420.x. PubMed PMID: 21039953; PubMed Central PMCID: PMCPMC3058925.

949. Stoeckel MC, Binkofski F. The role of ipsilateral primary motor cortex in movement control and recovery from brain damage. Exp Neurol. 2010;221(1):13-7. Epub 2009/11/10. doi: 10.1016/j.expneurol.2009.10.021. PubMed PMID: 19896482.

950. Tallus J, Lioumis P, Hämäläinen H, Kähkönen S, Tenovuo O. Mild TBI causes a long-lasting elevation of the transcranial magnetic stimulation motor threshold. Brain Injury. 2010;24(3):177. doi: 10.3109/02699051003648227.

951. Tallus J, Lioumis P, Hämäläinen H, Kähkönen S, Tenovuo O. Differences in TMS-evoked EEG responses between healthy controls and symptomatic and recovered patients with mild TBI. Brain Injury. 2010;24(3):177-8. doi: 10.3109/02699051003648227.

952. Tawfik S, Attia M, Flanagan E S, Kapoor R, Ellard S, Hussain K. Congenital hyperinsulinism in infant with homozygous novel ABCC8 missense mutation G1376R. Hormone Research in Paediatrics. 2010;74:240. doi: 10.1159/000321348.

953. Tumakaev RF, Guseva DS, Garaev RS. [Dimephosphon restores functions of the injured spinal cord]. Eksp Klin Farmakol. 2010;73(5):41-4. Epub 2010/07/06. PubMed PMID: 20597371.

954. Watt J. Ciliary neurotrophic factor as a treatment for neuroendocrine abnormalities associated with traumatic brain injury. Brain Injury. 2010;24(3):341. doi: 10.3109/02699051003648227.

955. Weber K, Holthausen H, Staudt M. Asymmetrical localisation of the motor cortex after perinatally acquired intracerebral hemorrhage: Volume shift versus neuroplasticity. Neuropediatrics. 2010;41(2). doi: 10.1055/s-0030-1265615.

956. Wei JW, Heeley EL, Wang J, Huang Y, Wong LKS, Li Z, et al. Comparison of recovery trajectories and prognostic indicators for ischemic and hemorrhagic stroke in china: The chinaquest registry study. Cerebrovascular Diseases. 2010;29:259. doi:

10.1159/000321266.
957. Yang FG, Fuller J, Khodaparast N, Krawczyk DC. Figurative language processing after traumatic brain injury in adults: A preliminary study. Neuropsychologia. 2010;48(7):1923-9. doi: 10.1016/j.neuropsychologia.2010.03.011.
958. Zeller D, Dang SY, Classen J. LTD-like central motor plasticity is not compromised in multiple sclerosis patients. Klinische Neurophysiologie. 2010;41(1). doi: 10.1055/s-0030-1250978.
959. Zorrilla-Zubilete MA, Maur DG, Palumbo ML, Genaro AM. Role of nitric oxide signaling pathways in brain injuries. Current Chemical Biology. 2010;4(3):250-61. doi: 10.2174/187231310792062666.
960. Annual Meeting of the Swiss Society of Clinical Neurophysiology. Clinical Neurophysiology. 2011;122(4).
961. Abdel-Salam OME. Stem cell therapy for Alzheimer's disease. CNS and Neurological Disorders - Drug Targets. 2011;10(4):459-85. doi: 10.2174/187152711795563976.
962. Al-Jehani HM, Al-Issawi W, Jacques L. Duration of intra-operative stimulation on trial as a predictor of success of spinal cord stimulation for chronic pain syndromes. Canadian Journal of Neurological Sciences. 2011;38(3):S60.
963. Alonso-Alonso M, Chang B, Press DZ, Rotenberg A,

Pascual-Leone A. Commentary on Kratz et al "Seizure in a Nonpredisposed Individual Induced by Single-Pulse Transcranial Magnetic Stimulation". Journal of Ect. 2011;27(2):176-7. doi: 10.1097/YCT.0b013e3181e6336e. PubMed PMID: WOS:000290789300021.

964. Andreani JCM. Lateral cord stimulation. A new tool for spastic syndrome treatment. Neuromodulation. 2011;14(5):470. doi: 10.1111/j.1525-1403.2011.00364.x.

965. Angelakis E. EEG, fMRI, and TDCS/tACS applications for disorders of consciousness and movement disorders. Journal of Neurotherapy. 2011;15(4):409-10. doi: 10.1080/10874208.2011.623098.

966. Ansari AH, Jain S, Kumar U, Mathur R. Effects of transcranial magnetic stimulation on pain and anxiety in fibromyalgia patients. Indian Journal of Physiology and Pharmacology. 2011;55(5):291.

967. Babiloni C, Vecchio F, Lizio R, Ferri R, Rodriguez G, Marzano N, et al. Resting State Cortical Rhythms in Mild Cognitive Impairment and Alzheimer's Disease: Electroencephalographic Evidence. Journal of Alzheimers Disease. 2011;26:201-14. doi: 10.3233/jad-2011-0051. PubMed PMID: WOS:000297842800014.

968. Bagnato S, Boccagni C, Sant'Angelo A, Prestandrea C, Galardi G. Reduced intracortical modulation in patients in vegetative

state after a traumatic brain injury. Clinical Neurophysiology. 2011;122:S192-S3. doi: 10.1016/S1388-2457(11)60696-2.

969. Battaglia F, Freedberg D. Augmenting physical therapy through modulation of dorsal premotor-motor connectivity. Archives of Physical Medicine and Rehabilitation. 2011;92(10):1736.

970. Billino J, Braun DI, Bremmer F, Gegenfurtner KR. Challenges to normal neural functioning provide insights into separability of motion processing mechanisms. Neuropsychologia. 2011;49(12):3151-63. doi: 10.1016/j.neuropsychologia.2011.07.009.

971. Blom RM, Figee M, Vulink N, Denys D. Update on repetitive transcranial magnetic stimulation in obsessive-compulsive disorder: Different targets. Current Psychiatry Reports. 2011;13(4):289-94. doi: 10.1007/s11920-011-0205-3.

972. Blythe LaGasse A, Knight A. Rhythm and music in rehabilitation: A critical review of current research. Critical Reviews in Physical and Rehabilitation Medicine. 2011;23(1-4):49-67. doi: 10.1615/critrevphysrehabilmed.v23.i1-4.40.

973. Borckardt J, Bikson M, Frohman H, Reeves S, Datta A, Bansal V, et al. A pilot study of the tolerability, safety and effects of high-definition transcranial direct current stimulation (HD-tDCS) on pain perception. Journal of Pain. 2011;12(4):P52. doi: 10.1016/j.jpain.2011.02.212.

974. Bourguignon M, Jousmki V, Opde Beeck M, Van Bogaert P, Nouali M, Metens T, et al. Novel multimodal functional neuroimaging approach for noninvasive primary sensorimotor cortex mapping. Epilepsia. 2011;52:166-7. doi: 10.1111/j.1528-1167.2011.03207.x.

975. Buiten MS, Dejongste MJL, Beese U, Kliphuis C, Durenkamp A, Staal MJ. Subcutaneous electrical nerve stimulation: A feasible and new method for the treatment of patients with refractory angina. Neuromodulation. 2011;14(3):258-65. doi: 10.1111/j.1525-1403.2011.00355.x.

976. Burnstock G, Fredholm BB, Verkhratsky A. Adenosine and ATP receptors in the brain. Current Topics in Medicinal Chemistry. 2011;11(8):973-1011. doi: 10.2174/156802611795347627.

977. Burnstock G, Krügel U, Abbracchio MP, Illes P. Purinergic signalling: from normal behaviour to pathological brain function. Prog Neurobiol. 2011;95(2):229-74. Epub 2011/09/13. doi: 10.1016/j.pneurobio.2011.08.006. PubMed PMID: 21907261.

978. Camprodon JA, Sack A, Pascal-Leone A. Simultaneous TMS and fMRI to study mechanisms of hemispatial neglect recovery and strategies for therapeutic neuromodulation. Journal of Neuropsychiatry and Clinical Neurosciences. 2011;23(2):31.

979. Castellanos NP, Bajo R, Cuesta P, Antoniovillacorta-Atienza J, Paúl N, Garcia-Prieto J, et al. Alteration and reorganization of

functional networks: A new perspective in brain injury study. Frontiers in Human Neuroscience. 2011(SEPTEMBER). doi: 10.3389/fnhum.2011.00090.

980. Charash WE, Caputo MP, Clark H, Callas PW, Rogers FB, Crookes BA, et al. Telemedicine to a moving ambulance improves outcome after trauma in simulated patients. Journal of Trauma - Injury, Infection and Critical Care. 2011;71(1):49-54. doi: 10.1097/TA.0b013e31821e4690.

981. Chawla A, Fang L, Wang K. TBI biomarker UCH-L1 protein monomer and oligomers overexpressed in E.coli as recombinant soluable protein calibrator for human gfap biomarker assay. Journal of Neurotrauma. 2011;28(6):A39-A40. doi: 10.1089/neu.2011.9945.

982. Ciaramella A, De Carolis G. The effectiveness of motor cortex stimulation for neuropathic pain can be predicted by psychiatric comorbidity. Case report. Neuromodulation. 2011;14(4):378. doi: 10.1111/j.1525-1403.2011.00363.x.

983. Cioni B, Bentivoglio A, Conforti G, Daniele A, De Simone C, Fasano A, et al. Motor cortex stimulation for Parkinson's disease. A prospective double-blind randomized study with cross-over. Preliminary results. Neuromodulation. 2011;14(4):364. doi: 10.1111/j.1525-1403.2011.00363.x. PubMed PMID: CN-01029477.

984. Clauss RP. Neurotransmitters in disorders of consciousness

and brain damage. Medical Hypotheses. 2011;77(2):209-13. doi: 10.1016/j.mehy.2011.04.014.

985. Cohen LG. Non-invasive brain stimulation in the study of mechanisms and modulation of motor learning. Clinical Neurophysiology. 2011;122:S54-S5. doi: 10.1016/S1388-2457(11)60184-3.

986. Connon FF, Namdarian B, Ee JL, Drummond KJ, Miller JA. Do routinely repeated computed tomography scans in traumatic brain injury influence management? A prospective observational study in a level 1 trauma center. Ann Surg. 2011;254(6):1028-31. Epub 2011/11/25. doi: 10.1097/SLA.0b013e318219727f. PubMed PMID: 22112983.

987. Cook G, Garderet L, van Biezen A, Henseler A, Leblond V, Rovira M, et al. The Roel of High Dose Chemotherapy and Autologous Stem Cell Transplantation (ASCT) In PATIENTS with POEMS SYNDROME: A Retrospective study of the MM Subcommittee of the Chronic Leukemia Working Party of the EBMT. Blood. 2011;118(21):1758-9. PubMed PMID: WOS:000299597106283.

988. Cook G, Garderet L, Van Biezen A, LeBlond V, Rovira M, Bordessoule D, et al. The role of high-dose chemotherapy and autologous stem cell transplantation in patients with poems syndrome:

A retrospective study of the MM subcommittee of the chronic leukaemia working party of the EBMT. Bone Marrow Transplantation. 2011;46:S32-S3. doi: 10.1038/bmt.2011.47.
989. Danilov YP, Tyler ME, Kaczmarek KA. Rehabilitation of multiple sclerosis and Parkinson's symptoms using cranial nerve non-invasive neuromodulation, (CN-NINM). Movement Disorders. 2011;26:S3-S4. doi: 10.1002/mds.23764.
990. De Beaumont L, Mongeon D, Tremblay S, Messier J, Prince F, Leclerc S, et al. Persistent motor system abnormalities in formerly concussed athletes. J Athl Train. 2011;46(3):234-40. Epub 2011/06/15. doi: 10.4085/1062-6050-46.3.234. PubMed PMID: 21669091; PubMed Central PMCID: PMCPMC3419550.
991. De Ridder D, van der Loo E, Vanneste S, Gais S, Plazier M, Kovacs S, et al. Theta-gamma dysrhythmia and auditory phantom perception Case report. Journal of Neurosurgery. 2011;114(4):912-21. doi: 10.3171/2010.11.Jns10335. PubMed PMID: WOS:000288725900005.
992. Deering T, Bernardes A, Veiga A, Gartenlaub O, Goncalves A, Jimenez A, et al. Increase of ventricular pacing demand in patients with suspected and documented atrio-ventricular blocks implanted with dual-chamber pacemaker. Europace. 2011;13. doi: 10.1093/europace/eur229.

993. Dinomais M. Reorganization types of the sensori-motor cortex after unilateral early brain lesion: Case of congenital hemiparesis. Annals of Physical and Rehabilitation Medicine. 2011;54:e197. doi: 10.1016/j.rehab.2011.07.442.

994. Dombovy ML. Introduction: The evolving field of neurorehabilitation. CONTINUUM Lifelong Learning in Neurology. 2011;17(3):443-8. doi: 10.1212/01.CON.0000399065.23826.f0.

995. Dudakkov JA, Hanash AM, Young LF, Singer NV, West ML, Jenq RR, et al. Innate Lymphoid Cell-Derived IL-22 Mediates Endogenous Thymic Repair Under the Control of IL-23. Blood. 2011;118(21):69-. PubMed PMID: WOS:000299597100144.

996. Dykukha S. Surgical correction of complex anatomical and haemodynamical forms of aortic coarctation with partial left ventricular use of bypass. Interactive Cardiovascular and Thoracic Surgery. 2011;12:S131. doi: 10.1510/icvts.2011.0000S1.

997. Eakin K, Ostergard T, Wu O, Hoffer B, Miller J. Electrophysiological abnormalities after minimal traumatic brain injury. Journal of Neurotrauma. 2011;28(6):A10-A1. doi: 10.1089/neu.2011.9945.

998. Eddy CM, Rickards HE, Cavanna AE. Review: Treatment strategies for tics in Tourette syndrome. Therapeutic Advances in Neurological Disorders. 2011;4(1):25-45. doi:

10.1177/1756285610390261.

999. Edwardson M, Fetz EE, Avery DH. Seizure produced by 20 Hz transcranial magnetic stimulation during isometric muscle contraction in a healthy subject. Clin Neurophysiol. 2011;122(11):2326-7. Epub 2011/05/10. doi: 10.1016/j.clinph.2011.04.005. PubMed PMID: 21550843; PubMed Central PMCID: PMCPMC3372095.

1000. Eiermann W, Pienkowski T, Crown J, Sadeghi S, Martin M, Chan A, et al. Phase III study of doxorubicin/cyclophosphamide with concomitant versus sequential docetaxel as adjuvant treatment in patients with human epidermal growth factor receptor 2-normal, node-positive breast cancer: BCIRG-005 trial. Journal of Clinical Oncology. 2011;29(29):3877-84. doi: 10.1200/JCO.2010.28.5437.

1001. Eisner W, Bauer R. Loss of effectivity of motor cortex stimulation for treatment of neuropathic pain syndromes. Acta Neurochirurgica. 2011;153(3):715. doi: 10.1007/s00701-010-0919-8.

1002. El-Wahab N, Robinson N. Analgesia and anaesthesia in labour. Obstetrics, Gynaecology and Reproductive Medicine. 2011;21(5):137-41. doi: 10.1016/j.ogrm.2011.02.006.

1003. Engels PT, Rezende-Neto JB, Al Mahroos M, Scarpelini S, Rizoli SB, Tien HC. The natural history of trauma-related coagulopathy: Implications for treatment. Journal of Trauma - Injury,

Infection and Critical Care. 2011;71(5 SUPPL.1):S448-S55. doi: 10.1097/TA.0b013e318232e6ac.

1004. Fathali N, Khatibi NH, Ostrowski RP, Zhang JH. The evolving landscape of neuroinflammation after neonatal hypoxia-ischemia. Acta Neurochirurgica, Supplementum2011. p. 93-100.

1005. Fitzgerald PB, Hoy KE, Maller JJ, Herring S, Segrave R, McQueen S, et al. Transcranial magnetic stimulation for depression after a traumatic brain injury: a case study. J ect. 2011;27(1):38-40. Epub 2010/10/13. doi: 10.1097/YCT.0b013e3181eb30c6. PubMed PMID: 20938348.

1006. Fonoff ET, Hamani C, de Andrade DC, Yeng LT, Marcolin MA, Teixeira MJ. Pain Relief and Functional Recovery in Patients with Complex Regional Pain Syndrome after Motor Cortex Stimulation. Stereotactic and Functional Neurosurgery. 2011;89(3):167-72. doi: 10.1159/000324895. PubMed PMID: WOS:000290307000006.

1007. Gao G, Cooper E, Jiang J. RIGHT MEDIAN NERVE STIMULATION IN POST-TRAUMATIC COMA. Journal of Neurotrauma. 2011;28(5):A39-A. PubMed PMID: WOS:000290472100116.

1008. Gerretsen P, Diaz P, Mamo D, Kavanagh D, Menon M, Pollock BG, et al. Transient insight induction with electroconvulsive

therapy in a patient with refractory schizophrenia: a case report and systematic literature review. J ect. 2011;27(3):247-50. Epub 2010/10/23. doi: 10.1097/YCT.0b013e3181f816f6. PubMed PMID: 20966768.

1009. Gillebert CR, Mantini D, Thijs V, Sunaert S, Dupont P, Vandenberghe R. Lesion evidence for the critical role of the intraparietal sulcus in spatial attention. Brain. 2011;134:1694-709. doi: 10.1093/brain/awr085. PubMed PMID: WOS:000291063900012.

1010. Golby AJ, McLaren Black P. Preface. Neurosurgery Clinics of North America. 2011;22(2):xiii-xiv. doi: 10.1016/j.nec.2011.01.005.

1011. Grechanina OY, Zdibska OP, Novikova IV. FATTY ACID OXIDATION DEFECT, DETECTED USING SELECTIVE SCREENING. Journal of Inherited Metabolic Disease. 2011;34:S157-S. PubMed PMID: WOS:000309837800281.

1012. Hallböök T, Thordstein M, Kohler S, Lundgren J, Bergstrand S, Pegenius G, et al. A case-report using high precision, navigated transcranial magnetic stimulation. Epilepsy Currents. 2011;11(1).

1013. Hansen H-C. Sooner or later ...? In search of the most useful components of the somatosensory evoked potential to define prognosis in critically ill patients with acute stroke. Clinical Neurophysiology. 2011;122(8):1482-4. doi: 10.1016/j.clinph.2011.01.038. PubMed PMID:

WOS:000292633300002.

1014. Hirabayashi H, Kawata K, Hoshida T, Tamura K, Youngsu P, Nakase H. Neuromodulation therapy for neuropathic pain. Japanese Journal of Neurosurgery. 2011;20(2):93-102. doi: 10.7887/jcns.20.93.

1015. Hirnstein M, Bayer U, Ellison A, Hausmann M. TMS over the left angular gyrus impairs the ability to discriminate left from right. Neuropsychologia. 2011;49(1):29-33. Epub 2010/11/03. doi: 10.1016/j.neuropsychologia.2010.10.028. PubMed PMID: 21035475.

1016. Holt RL, Mikati MA. Care for child development: basic science rationale and effects of interventions. Pediatr Neurol. 2011;44(4):239-53. Epub 2011/03/15. doi: 10.1016/j.pediatrneurol.2010.11.009. PubMed PMID: 21397164.

1017. Hosp JA, Hertler B, Atiemo CO, Luft AR. Dopaminergic modulation of receptive fields in rat sensorimotor cortex. Neuroimage. 2011;54(1):154-60. doi: 10.1016/j.neuroimage.2010.07.029. PubMed PMID: WOS:000283825000019.

1018. Hsieh TH, Dhamne S, Chen JJ, Pascual-Leone A, Jensen F, Rotenberg A. Loss of cortical inhibition following traumatic brain injury detected by mechanomyography and paired-pulse transcranial magnetic stimulation in unanesthetized rats. Clinical Neurophysiology. 2011;122:S148. doi: 10.1016/S1388-2457(11)60531-2.

1019. Insola A, Padua L, Valeriani M. Absent median nerve P14

far-field somatosensory evoked potential with persistent tibial nerve P30 component in a patient with ischemic pontine lesion. Neurophysiologie Clinique. 2011;41(2):81-4. doi: 10.1016/j.neucli.2010.12.003.

1020. Irct138903254191N. Repetitive transcranial magnetic stimulation and intermittent theta burst stimulation in schizophrenia. https://trialsearchwhoint/Trial2aspx?TrialID=IRCT138903254191N1. 2011. PubMed PMID: CN-01799008.

1021. Jacobson L, Lavidor M. Inhibition and facilitation of cognitive functions. Journal of Molecular Neuroscience. 2011;45:S56. doi: 10.1007/s12031-011-9491-9.

1022. Jacobson L, Lavidor M. Activation of inhibition: Diminishing impulsive behavior by direct current stimulation over the inferior frontal gyrus. Clinical Neurophysiology. 2011;122:S122-S3. doi: 10.1016/S1388-2457(11)60435-5.

1023. Jagodic HK, Jankovic VN, Pregelj P. Up-todate treatment with electroconvulsive therapy. Zdravniski Vestnik-Slovenian Medical Journal. 2011;80(3):203-14. PubMed PMID: WOS:000288646600011.

1024. Jan JE, Ribary U, Wong PKH, Reiter RJ, Bax MCO, Wasdell MB. Cerebral modulation of circadian sleep-wake rhythms. Journal of Clinical Neurophysiology. 2011;28(2):165-9. doi: 10.1097/WNP.0b013e31821219e3.

1025. Jang SH. Diffusion tensor imaging studies on corticospinal tract injury following traumatic brain injury: a review. NeuroRehabilitation. 2011;29(4):339-45. doi: 10.3233/nre-2011-0710. PubMed PMID: MEDLINE:22207060.

1026. Jean-Paul N, Nizard J, Keravel Y, Lefaucheur J-P. Invasive brain stimulation for the treatment of neuropathic pain. Nature Reviews Neurology. 2011;7(12):699-709. doi: 10.1038/nrneurol.2011.138. PubMed PMID: WOS:000298149000008.

1027. Jotterand F, Giordano J. Transcranial magnetic stimulation, deep brain stimulation and personal identity: Ethical questions, and neuroethical approaches for medical practice. International Review of Psychiatry. 2011;23(5):476-85. doi: 10.3109/09540261.2011.616189.

1028. Kao CD, Lin KP, Chen JT, Chang JB, Guo WY, Lin YY, et al. Preserved motor-evoked potentials but without good motor recovery in a patient with decerebrate rigidity. Journal of the Chinese Medical Association. 2011;74(1):37-9. doi: 10.1016/j.jcma.2011.01.005.

1029. Keep RF, Xi G, Hua Y, Xiang J. Clot formation, vascular repair and hematoma resolution after ICH, a coordinating role for thrombin? Acta Neurochirurgica, Supplementum2011. p. 71-5.

1030. Kleim JA. Neural plasticity and neurorehabilitation: teaching the new brain old tricks. Journal of communication disorders. 2011;44(5):521-8. doi: 10.1016/j.jcomdis.2011.04.006. PubMed

PMID: MEDLINE:21600589.
1031. Koffman RL. Downrange acupuncture. Medical Acupuncture. 2011;23(4):215-8. doi: 10.1089/acu.2011.0860.
1032. Krasomski G. The role of cervical factor in preterm labour etiopathogenesis. Ginekologia i Poloznictwo. 2011;21(3):87-91.
1033. Kratz O. Reply to the Letter to the Editor "Response to Kratz et al, Seizure in a Nonpredisposed Individual Induced by Single-Pulse Transcranial Magnetic Stimulation". Journal of Ect. 2011;27(2):177-. doi: 10.1097/YCT.0b013e3181ec0d8a. PubMed PMID: WOS:000290789300022.
1034. Kratz O, Studer P, Barth W, Wangler S, Hoegl T, Heinrich H, et al. Seizure in a nonpredisposed individual induced by single-pulse transcranial magnetic stimulation. J ect. 2011;27(1):48-50. Epub 2010/03/31. doi: 10.1097/YCT.0b013e3181d2ef85. PubMed PMID: 20351571.
1035. Kugiumtzis D, Kimiskidis VK. Effective connectivity in partial epilepsy: Modulation by transcranial magnetic stimuli. Neuroscience Letters. 2011;500:e9. doi: 10.1016/j.neulet.2011.05.088.
1036. Kutluay S. Neural plasticity after stroke and functional recovery. Turkiye Fiziksel Tip ve Rehabilitasyon Dergisi. 2011;57:10.
1037. Kwon HG, Lee DG, Choi BY, Chang CH, Kim SH, Jang SH. Recovery of the corticospinal tract after injury by transtentorial

herniation: a case report. NeuroRehabilitation. 2011;29(3):243-6. Epub 2011/12/07. doi: 10.3233/nre-2011-0699. PubMed PMID: 22142757.

1038. Ladd FVL, Ladd AAB, Passos AO, Mattos KC, Brum PC, Coppi AA. Haemodynamic and design-based stereological assessment of the heart in 3-NP-induced mice. Clinical Genetics. 2011;80:20. doi: 10.1111/j.1399-0004.2011.01737.x.

1039. Lambooij E, Anil H, Butler SR, Reimert H, Workel L, Hindle V. Transcranial magnetic stunning of broilers: a preliminary trial to induce unconsciousness. Animal Welfare. 2011;20(3):407-12. PubMed PMID: WOS:000293305900010.

1040. Lamy JC, Boakye M. Non-invasive tools to promote spinal plasticity in humans. Clin Neurophysiol. 2011;122(11):2114-5. Epub 2011/04/26. doi: 10.1016/j.clinph.2011.03.024. PubMed PMID: 21514881.

1041. Lee KY, Nyein MK, Moore DF, Joannopoulos JD, Socrate S, Imholt T, et al. Blast-induced electromagnetic fields in the brain from bone piezoelectricity. Neuroimage. 2011;54 Suppl 1:S30-6. Epub 2010/06/16. doi: 10.1016/j.neuroimage.2010.05.042. PubMed PMID: 20547228.

1042. Lemaire JJ, Pontier B, Coste J, Gillart T, Sakka L, Feschet F, et al. Conscious behavior after traumatic brain injury:

Anatomo-functional support and therapeutic prospects. Annals of Physical and Rehabilitation Medicine. 2011;54:e213-e4. doi: 10.1016/j.rehab.2011.07.387.
1043. León L, Casanova-Molla J, Lauria G, Valls-Solé J. The somatosensory blink reflex in upper and lower brainstem lesions. Muscle Nerve. 2011;43(2):196-202. Epub 2011/01/22. doi: 10.1002/mus.21810. PubMed PMID: 21254084.
1044. Levens SM, Devinsky O, Phelps EA. Role of the left amygdala and right orbital frontal cortex in emotional interference resolution facilitation in working memory. Neuropsychologia. 2011;49(12):3201-12. doi: 10.1016/j.neuropsychologia.2011.07.021.
1045. Li S, Rymer WZ. Voluntary breathing influences corticospinal excitability of nonrespiratory finger muscles. J Neurophysiol. 2011;105(2):512-21. Epub 2010/12/17. doi: 10.1152/jn.00946.2010. PubMed PMID: 21160006; PubMed Central PMCID: PMCPMC3059159.
1046. Liu MC, Kobeissy F, Zheng W, Zhang Z, Hayes RL, Wang KK. Dual vulnerability of tau to calpains and caspase-3 proteolysis under neurotoxic and neurodegenerative conditions. ASN Neuro. 2011;3(1):e00051. Epub 2011/03/02. doi: 10.1042/an20100012. PubMed PMID: 21359008; PubMed Central PMCID: PMCPMC3040574.

1047. Livingston SC. Head Injury - Baseball. Persistent Electrophysiologic Changes Associated with Acute Cerebral Concussion in a Collegiate Baseball Player. Medicine and Science in Sports and Exercise. 2011;43(5):196-. doi: 10.1249/01.MSS.0000400522.18851.f6. PubMed PMID: WOS:000209079500494.

1048. Lucas JM, Ji Y, Masri R. Motor cortex stimulation reduces hyperalgesia in an animal model of central pain. Pain. 2011;152(6):1398-407. doi: 10.1016/j.pain.2011.02.025. PubMed PMID: WOS:000290710100028.

1049. Ma Y, Hu Y, Valentin N, Geocadin RG, Thakor NV, Jia X. Time jitter of somatosensory evoked potentials in recovery from hypoxic-ischemic brain injury. Journal of Neuroscience Methods. 2011;201(2):355-60. doi: 10.1016/j.jneumeth.2011.08.025. PubMed PMID: WOS:000295998900011.

1050. Magnani B, Oliveri M, Mancuso G, Galante E, Frassinetti F. Time and spatial attention: Effects of prism adaptation on temporal deficits in brain damaged patients. Neuropsychologia. 2011;49(5):1016-23. doi: 10.1016/j.neuropsychologia.2010.12.014. PubMed PMID: WOS:000290649200027.

1051. McLarnon A. Motility: Magnetic stimulation of the cerebellum could restore swallowing. Nature Reviews

Gastroenterology and Hepatology. 2011;8(10):538. doi: 10.1038/nrgastro2011157.

1052. Messina G, Cordella R, Dones I, Tringali G, Franzini A. Improvement of secondary fixed dystonia of the upper limb after chronic extradural motor Cortex stimulation in 10 patients. Neuromodulation. 2011;14(4):358. doi: 10.1111/j.1525-1403.2011.00363.x.

1053. Migalska K, Morrow DIJ, Garland MJ, Thakur R, Woolfson AD, Donnelly RF. Laser-Engineered Dissolving Microneedle Arrays for Transdermal Macromolecular Drug Delivery. Pharmaceutical Research. 2011;28(8):1919-30. doi: 10.1007/s11095-011-0419-4. PubMed PMID: WOS:000292518800011.

1054. Miller BT, Vytlacil J, Fegen D, Pradhan S, D'Esposito M. The prefrontal cortex modulates category selectivity in human extrastriate cortex. J Cogn Neurosci. 2011;23(1):1-10. Epub 2010/07/01. doi: 10.1162/jocn.2010.21516. PubMed PMID: 20586702.

1055. Miniussi C, Rossini PM. Transcranial magnetic stimulation in cognitive rehabilitation. Neuropsychol Rehabil. 2011;21(5):579-601. Epub 2011/04/05. doi: 10.1080/09602011.2011.562689. PubMed PMID: 21462081.

1056. Miniussi C, Vallar G. Brain stimulation and behavioural cognitive rehabilitation: a new tool for neurorehabilitation?

Neuropsychol Rehabil. 2011;21(5):553-9. Epub 2011/10/21. doi: 10.1080/09602011.2011.622435. PubMed PMID: 22011015.

1057. Miniussi C, Vallar G. Brain stimulation and behavioural cognitive rehabilitation: A new tool for neurorehabilitation? Foreword. Neuropsychological Rehabilitation. 2011;21(5):553-9. doi: 10.1080/09602011.2011.622435. PubMed PMID: WOS:000299789100001.

1058. Mishra NK, Russmann H, Granziera C, Maeder P, Annoni JM. Mutism and amnesia following high-voltage electrical injury: psychogenic symptomatology triggered by organic dysfunction? Eur Neurol. 2011;66(4):229-34. Epub 2011/09/29. doi: 10.1159/000330953. PubMed PMID: 21952143.

1059. Missitzi J, Gentner R, Geladas N, Politis P, Karandreas N, Classen J, et al. Plasticity in human motor cortex is in part genetically determined. Journal of Physiology-London. 2011;589(2):297-306. doi: 10.1113/jphysiol.2010.200600. PubMed PMID: WOS:000286213900004.

1060. Mistry S. Unravelling and manipulating the cerebral control of human swallowing using non-invasive brain imaging modalities. Annals of Physical and Rehabilitation Medicine. 2011;54:e35. doi: 10.1016/j.rehab.2011.07.855.

1061. Morishita T, Yamashita A, Katayama Y, Oshima H, Nishizaki

Y, Shijo K, et al. Chronological Changes in Astrocytes Induced by Chronic Electrical Sensorimotor Cortex Stimulation in Rats. Neurologia Medico-Chirurgica. 2011;51(7):496-502. doi: 10.2176/nmc.51.496. PubMed PMID: WOS:000292853000004.

1062. Musayev AV, Huseynova SG, Mustafayeva EE. MAGNETIC STIMULATION IN REHABILITATION OF PATIENTS SUFFERING FROM TRAUMATIC NEUROPATHY. European Journal of Neurology. 2011;18:325-. PubMed PMID: WOS:000294806600590.

1063. Nagarwal RC, Kumar R, Dhanawat M, Pandit JK. Modified PLA nano in situ gel: A potential ophthalmic drug delivery system. Colloids and Surfaces B-Biointerfaces. 2011;86(1):28-34. doi: 10.1016/j.colsurfb.2011.03.023. PubMed PMID: WOS:000291458100005.

1064. Nardone R, Bergmann J, Kunz A, Caleri F, Seidl M, Tezzon F, et al. Cortical excitability changes in patients with sleep-wake disturbances after traumatic brain injury. J Neurotrauma. 2011;28(7):1165-71. Epub 2011/03/31. doi: 10.1089/neu.2010.1748. PubMed PMID: 21446790.

1065. Nct. Transcranial Direct Current Stimulation (tDCS) and Motor Training for Traumatic Brain Injury (TBI) Survivors. https://clinicaltrialsgov/show/NCT01749735. 2011. PubMed PMID:

CN-01539339.

1066. Nct. Paraorbital-Occipital Alternating Current Stimulation Therapy of Patients With Post-Chiasmatic Lesions. https://clinicaltrialsgov/show/NCT01418820. 2011. PubMed PMID: CN-01488182.

1067. Nct. A Double Blind Sham-controled Study to Evaluate the Influence of Low Frequency Repetitive Transcranial Stimulation (r-TMS) on Motor and Cognitive Measurements in Patients With Asymmetric Parkinson's Disease. https://clinicaltrialsgov/show/NCT01367782. 2011. PubMed PMID: CN-01486872.

1068. Pachalska M, Łukowicz M, Kropotov JD, Herman-Sucharska I, Talar J. Evaluation of differentiated neurotherapy programs for a patient after severe TBI and long term coma using event-related potentials. Med Sci Monit. 2011;17(10):Cs120-8. Epub 2011/10/01. doi: 10.12659/msm.881970. PubMed PMID: 21959618; PubMed Central PMCID: PMCPMC3539468.

1069. Panina OS, Zaytseva GA, Pokazan'eva SA. Rehabilitation of newborns with perinatal lesions of the central nervous system using transcranial magnetic therapy. Zhurnal Nevrologii i Psihiatrii imeni SS Korsakova. 2011;111(4):87-9.

1070. Pape TL-B. Diagnostic and therapeutic challenges of persons

with severe traumatic brain injuries. Faseb Journal. 2011;25. PubMed PMID: WOS:000310708402343.

1071. Park YS, Kim JP, Chang WS, Chang JW. Management of a DBS system in patients with traumatic brain injury: Case report. Neuromodulation. 2011;14(3):214-8. doi: 10.1111/j.1525-1403.2011.00348.x.

1072. Pascual-Leone A, Freitas C, Oberman L, Horvath JC, Halko M, Eldaief M, et al. Characterizing brain cortical plasticity and network dynamics across the age-span in health and disease with TMS-EEG and TMS-fMRI. Brain Topography. 2011;24(3-4):302-15. doi: 10.1007/s10548-011-0196-8.

1073. Paulus W. Transcranial electrical stimulation (tES - tDCS; tRNS, tACS) methods. Neuropsychol Rehabil. 2011;21(5):602-17. Epub 2011/08/09. doi: 10.1080/09602011.2011.557292. PubMed PMID: 21819181.

1074. Paulus W. Transcranial stimulation techniques: which genetics is the best for which purpose? Journal of Physiology-London. 2011;589(6):1245-. doi: 10.1113/jphysiol.2011.206680. PubMed PMID: WOS:000288376800003.

1075. Piccione F, Cavinato M, Manganotti P, Formaggio E, Storti SF, Battistin L, et al. Behavioral and neurophysiological effects of repetitive transcranial magnetic stimulation on the minimally

conscious state: a case study. Neurorehabil Neural Repair. 2011;25(1):98-102. Epub 2010/07/22. doi: 10.1177/1545968310369802. PubMed PMID: 20647501.
1076. Piech RM, McHugo M, Smith SD, Dukic MS, Van Der Meer J, Abou-Khalil B, et al. Attentional capture by emotional stimuli is preserved in patients with amygdala lesions. Neuropsychologia. 2011;49(12):3314-9. doi: 10.1016/j.neuropsychologia.2011.08.004.
1077. Piriz J, Muller A, Trejo JL, Torres-Aleman I. IGF-I and the aging mammalian brain. Experimental Gerontology. 2011;46(2-3):96-9. doi: 10.1016/j.exger.2010.08.022.
1078. Pirulli C, Ragazzoni A, Veniero D, Rossi S, Feurra M, Cincotta M, et al. Functional cortical connectivity in vegetative state and minimally conscious state: An investigation by transcranial magnetic stimulation-evoked potential. Clinical Neurophysiology. 2011;122:S180. doi: 10.1016/S1388-2457(11)60650-0.
1079. Pitcher JB, Schneider LA, Drysdale JL, Ridding MC, Owens JA. Motor System Development of the Preterm and Low Birthweight Infant. Clinics in Perinatology. 2011;38(4):605-+. doi: 10.1016/j.clp.2011.08.010. PubMed PMID: WOS:000298767800004.
1080. Quartarone A. Brain plasticity and mechanisms of neural encoding of skills. Clinical Neurophysiology. 2011;122:S43. doi: 10.1016/S1388-2457(11)60142-9.

1081. Rasche D. Neurostimulation of the precentral gyrus for the treatment of chronic neuropathic pain syndromes. Neuromodulation. 2011;14(4):380. doi: 10.1111/j.1525-1403.2011.00363.x.

1082. Rasche D, Tronnier V. Invasive pain therapy and clinical significance of intracranial neuromodulation for chronic pain. European Journal of Pain Supplements. 2011;5(1):96-7. doi: 10.1016/S1754-3207(11)70329-7.

1083. Reddy GS, Sujatha M. A Rare Case of Short-Chain Acyl-COA Dehydrogenase Deficiency: The Apparent Rarity of the Disorder Results in Under Diagnosis. Indian J Clin Biochem. 2011;26(3):312-5. Epub 2012/07/04. doi: 10.1007/s12291-011-0139-x. PubMed PMID: 22754199; PubMed Central PMCID: PMCPMC3162956.

1084. Reis J, Fritsch B. Modulation of motor performance and motor learning by transcranial direct current stimulation. Current Opinion in Neurology. 2011;24(6):590-6. doi: 10.1097/WCO.0b013e32834c3db0. PubMed PMID: WOS:000296850900012.

1085. Renton T, Yilmaz Z. Profiling of patients presenting with posttraumatic neuropathy of the trigeminal nerve. J Orofac Pain. 2011;25(4):333-44. Epub 2012/01/17. PubMed PMID: 22247929.

1086. Rönnefarth M, Schmidt S, Fleischmann R, Picht T, Brandt SA. Interneuronal assemblies reflect early neural reorganization after operative revascularization. Klinische Neurophysiologie. 2011;42(1).

doi: 10.1055/s-0031-1272764.
1087. Rosanova M, Gosseries O, Casarotto S, Boly M, Casali AG, Bruno MA, et al. Assessing cortical effective connectivity in patients with disorders of consciousness. Journal of Neurology. 2011;258:S42-S3. doi: 10.1007/s00415-011-6026-9.
1088. Rossetti AO, Lowenstein DH. Management of refractory status epilepticus in adults: still more questions than answers. Lancet Neurology. 2011;10(10):922-30. doi: 10.1016/s1474-4422(11)70187-9. PubMed PMID: WOS:000295814600013.
1089. Rossi S, Hallett M, Rossini PM, Pascual-Leone A. Screening questionnaire before TMS: An update. Clinical Neurophysiology. 2011;122(8):1686-. doi: 10.1016/j.clinph.2010.12.037. PubMed PMID: WOS:000292633300030.
1090. Rubio-Morell B, Rotenberg A, Herreros O, Monzon J, Exposito S, Pascual-Leone A. Transcranial current direct stimulation (tDCS): Therapeutic challenges for ADHD. European Child and Adolescent Psychiatry. 2011;20:S126. doi: 10.1007/s00787-011-0181-5.
1091. Schwartz TL, Nihalani N. Neuronal pathways implicated in executive dysfunction. Clinical Neuropsychiatry. 2011;8(3):212-8.
1092. Seo MH, Park SH, Ko MH, Seo JH. Motor evoked potentials of trunk muscles in traumatic brain injury patients. Ann Rehabil Med.

2011;35(4):557-64. Epub 2012/04/17. doi: 10.5535/arm.2011.35.4.557. PubMed PMID: 22506173; PubMed Central PMCID: PMCPMC3309240.

1093. Shorvon S. Super-refractory status epilepticus: An approach to therapy in this difficult clinical situation. Epilepsia. 2011;52:53-6. doi: 10.1111/j.1528-1167.2011.03238.x. PubMed PMID: WOS:000296065200018.

1094. Sironi VA. Origin and evolution of deep brain stimulation. Frontiers in Integrative Neuroscience. 2011;5. doi: 10.3389/fnint.2011.00042.

1095. Skuban T, Hardenacke K, Woopen C, Kuhn J. Informed consent in deep brain stimulation - ethical considerations in a stress field of pride and prejudice. Frontiers in Integrative Neuroscience. 2011;5. doi: 10.3389/fnint.2011.00007.

1096. Smith M. Shedding light on the adult brain: a review of the clinical applications of near-infrared spectroscopy. Philos Trans A Math Phys Eng Sci. 2011;369(1955):4452-69. Epub 2011/10/19. doi: 10.1098/rsta.2011.0242. PubMed PMID: 22006901.

1097. Sokal P, Harat M, Paczkowski D, Rudaś M, Birski M, Litwinowicz A. Results of neuromodulation for the management of chronic pain. Neurol Neurochir Pol. 2011;45(5):445-51. Epub 2011/12/01. doi: 10.1016/s0028-3843(14)60312-1. PubMed PMID:

22127939.
1098. Song XJ, Luo MF, Jiang J, Zhang JL. [Effect of acupoint-injection of stem cell factor antibody on electroacupuncture induced changes of count and degranulation of subcutaneous mast cells in the local region in rats]. Zhen Ci Yan Jiu. 2011;36(4):247-51. Epub 2011/09/29. PubMed PMID: 21942176.
1099. Sperlágh B, Vizi ES. The role of extracellular adenosine in chemical neurotransmission in the hippocampus and basal ganglia: Pharmacological and clinical aspects. Current Topics in Medicinal Chemistry. 2011;11(8):1034-46. doi: 10.2174/156802611795347564.
1100. Stam CJ. Exploring brain connectivity. Clinical Neurophysiology. 2011;122:S52. doi: 10.1016/S1388-2457(11)60175-2.
1101. Stenson K, Brownrigg JRW, Mohamed M. Occurrence and multiple recurrence of severe vasospasm of the upper extremity following thorascopic sympathectomy for hyperhidrosis. European Journal of Vascular and Endovascular Surgery. 2011;41(2):264-6. doi: 10.1016/j.ejvs.2010.11.003.
1102. Strzelczyk A, Kepenek Y, Rindock H, Müller-Mazzotta J, Oertel WH, Mylius V, et al. Benign paroxysmal positional vertigo following diagnostic transcranial magnetic stimulation. Neurol Sci. 2011;32(3):503-5. Epub 2011/03/05. doi: 10.1007/s10072-011-0511-x.

PubMed PMID: 21374092.

1103. Supp GG, Siegel M, Hipp JF, Engel AK. Cortical hypersynchrony predicts breakdown of sensory processing during loss of consciousness. Curr Biol. 2011;21(23):1988-93. Epub 2011/11/22. doi: 10.1016/j.cub.2011.10.017. PubMed PMID: 22100063.

1104. Tanaka S, Sandrini M, Cohen LG. Modulation of motor learning and memory formation by non-invasive cortical stimulation of the primary motor cortex. Neuropsychol Rehabil. 2011;21(5):650-75. Epub 2011/09/29. doi: 10.1080/09602011.2011.605589. PubMed PMID: 21942897.

1105. Thordstein M, Hallböök T, Lundgren J, Van Westen D, Elam M. Transfer of cortical motor representation after a perinatal cerebral insult. Pediatric Neurology. 2011;44(2):131-4. doi: 10.1016/j.pediatrneurol.2010.08.017.

1106. Timofeev I. Neuronal plasticity and thalamocortical sleep and waking oscillations. In: VanSomeren EJW, VanDerWerf YD, Roelfsema PR, Mansvelder HD, DaSilva FHL, editors. Slow Brain Oscillations of Sleep, Resting State and Vigilance. Progress in Brain Research. 1932011. p. 121-44.

1107. Toba M, Rastelli F, Tchokothe C, Nierat MC, Migliaccio R, Vincent S, et al. Using repetitive Transcranial Magnetic Stimulation (rTMS) for the rehabilitation of visuospatial neglect following right

hemisphere strokes: Theoretical background, initial experiences and preliminary data and from the project PHRC regional "NEGLECT". Annals of Physical and Rehabilitation Medicine. 2011;54:e219. doi: 10.1016/j.rehab.2011.07.404.

1108. Tomassini V, Jbabdi S, Kincses ZT, Bosnell R, Douaud G, Pozzilli C, et al. Structural and Functional Bases for Individual Differences in Motor Learning. Human Brain Mapping. 2011;32(3):494-508. doi: 10.1002/hbm.21037. PubMed PMID: WOS:000287375500013.

1109. Tremblay S, de Beaumont L, Lassonde M, Théoret H. Evidence for the specificity of intracortical inhibitory dysfunction in asymptomatic concussed athletes. J Neurotrauma. 2011;28(4):493-502. Epub 2011/01/12. doi: 10.1089/neu.2010.1615. PubMed PMID: 21219259.

1110. Urgesi C, Avenanti A. Functional and epiphenomenal modulation of neural activity in body-selective visual areas. Cognitive Neuroscience. 2011;2(3-4):212-4. doi: 10.1080/17588928.2011.604725.

1111. Van Der Aa NE, Debode S, Van Diessen E, Verhage CH, Van Nieuwenhuizen O, Ramsey NF, et al. Cortical reorganisation in a preterm born child with unilateral watershed infarction. European Journal of Paediatric Neurology. 2011;15(6):554-7. doi:

10.1016/j.ejpn.2011.04.008.

1112. van der Harst JJ, Luijckx GJ. Treatment of central nervous system tuberculosis infections and neurological complications of tuberculosis treatment. Current Pharmaceutical Design. 2011;17(27):2940-7. doi: 10.2174/138161211797470237.

1113. Van't Wout M. Game theory and the brain: Neural modulation and lesion studies as a tool to examine interpersonal decision-making. Nervenheilkunde. 2011;30(6):411-8. doi: 10.1055/s-0038-1628371.

1114. Vetrugno R, Montagna P. From REM sleep behaviour disorder to status dissociatus: Insights into the maze of states of being. Sleep Medicine. 2011;12(SUPPL. 2):S68-S71. doi: 10.1016/j.sleep.2011.10.015.

1115. Wei JW, Huang Y, Wang JG, Liu M, Wong LK, Huang Q, et al. Current management of intracerebral haemorrhage in China: a national, multi-centre, hospital register study. BMC Neurol. 2011;11:16. Epub 2011/02/01. doi: 10.1186/1471-2377-11-16. PubMed PMID: 21276264; PubMed Central PMCID: PMCPMC3040709.

1116. Westlake KP, Nagarajan SS. Functional connectivity in relation to motor performance and recovery after stroke. Frontiers in Systems Neuroscience. 2011(MARCH 2011). doi: 10.3389/fnsys.2011.00008.

1117. Xia LY, Zhang MQ, Yuan Ce, Rong MZ. A facile

heteroaggregate-template route to hollow magnetic mesoporous spheres with tunable shell structures. Journal of Materials Chemistry. 2011;21(25):9020-6. doi: 10.1039/c1jm10126a. PubMed PMID: WOS:000291611600015.

1118. Yoo SS, Kim H, Min BK, Franck E, Park S. Transcranial focused ultrasound to the thalamus alters anesthesia time in rats. Neuroreport. 2011;22(15):783-7. Epub 2011/08/31. doi: 10.1097/WNR.0b013e32834b2957. PubMed PMID: 21876461; PubMed Central PMCID: PMCPMC3174273.

1119. You X, Chang J-h, Ju B-K, Pak JJ. Rapidly dissolving fibroin microneedles for transdermal drug delivery. Materials Science & Engineering C-Materials for Biological Applications. 2011;31(8):1632-6. doi: 10.1016/j.msec.2011.06.010. PubMed PMID: WOS:000297400800003.

1120. Zdibska O, Novikova I. Presentation of metabolic diseases among patients of pediatric clinics. Clinical Biochemistry. 2011;44(7):549. doi: 10.1016/j.clinbiochem.2011.03.125.

1121. Zeller D, Dang SY, Stefan K, Biller A, Bartsch A, Saur D, et al. Functional role of ipsilateral motor areas in multiple sclerosis. Journal of Neurology, Neurosurgery and Psychiatry. 2011;82(5):578-83. doi: 10.1136/jnnp.2010.219964.

1122. Zhang Y, Su YY, Ye H, Xiao SY, Chen WB, Zhao JW.

Predicting comatose patients with acute stroke outcome using middle-latency somatosensory evoked potentials. Clin Neurophysiol. 2011;122(8):1645-9. Epub 2011/02/15. doi: 10.1016/j.clinph.2010.11.016. PubMed PMID: 21317029.

1123. Zhang YJ, Li JZ. [Severe sensorineural deafness after cerebral trauma by electroacupuncture and scalp acupuncture: a report of 19 cases]. Zhongguo Zhong Xi Yi Jie He Za Zhi. 2011;31(10):1427-8. Epub 2011/11/22. PubMed PMID: 22097221.

1124. Zhou F, Guo J, Cheng J, Wu G, Xia Y. Electroacupuncture increased cerebral blood flow and reduced ischemic brain injury: dependence on stimulation intensity and frequency. J Appl Physiol (1985). 2011;111(6):1877-87. Epub 2011/08/13. doi: 10.1152/japplphysiol.00313.2011. PubMed PMID: 21836043; PubMed Central PMCID: PMCPMC3233896.

1125. Zinke-Cerwenka W, Rohn A, Posch U, Beham-Schmid C, Linkesch W, Wölfler A, et al. Allogeneic stem cell transplantation with reduced intensity conditioning in patients with therapy-related myeloid neoplasms. European Surgery - Acta Chirurgica Austriaca. 2011;43:22. doi: 10.1007/s10353-011-0041-z.

1126. Zisa M, Di Carlo I, Pulvirenti E, Guastella T. Complex partial seizure in patient with insulinoma: importance of early diagnosis. Il Giornale di chirurgia. 2011;32(1-2):45-7.

1127. 58th Congress of the Czech and Slovak Society of Clinical Neurophysiology. Clinical Neurophysiology. 2012;123(3).

1128. Abo M, Kakuda W, Watanabe M, Senoo A. Does the combination of intensive speech therapy and therapeutic low-frequency transcranial magnetic stimulation based on functional MRI findings and analysis of type of aphasia improve the language function in poststroke patients with chronic aphasia? Brain Injury. 2012;26(4-5):397-8. doi: 10.3109/02699052.2012.660091.

1129. Achard S, Delon-Martin C, Vértes PE, Renard F, Schenck M, Schneider F, et al. Hubs of brain functional networks are radically reorganized in comatose patients. Proceedings of the National Academy of Sciences of the United States of America. 2012;109(50):20608-13. doi: 10.1073/pnas.1208933109.

1130. Agudelo M, Yoo C, Nair MP. Alcohol-induced serotonergic modulation: The role of histone deacetylases. Alcohol. 2012;46(7):635-42. doi: 10.1016/j.alcohol.2012.03.005.

1131. Alagoz A, Gunal Sazak H, Ulus F, Kokulu S, Pehlivanoglu P, Sahin S, et al. The evaluation of two hundred thoracic epidural catheterizations in terms of anesthesia residency grade. Pain Practice. 2012;12:138. doi: 10.1111/j.1533-2500.2011.00528.x.

1132. Alkan F, Altinbaş K. Detection of demografic and clinical risk factors associated with neuroleptic malignant syndrome: Evaluation of

cases in the Turkish literature. Dusunen Adam. 2012;25(1):35-42. doi: 10.5350/DAJPN2012250104.

1133. Angelakis E, Korfias S, Andreadis N, Liouta E, Sakas D. Cognitive electrophysiological responses of non-responsive patients before and after transcranial alternating current stimulation. Stereotactic and Functional Neurosurgery. 2012;90:123. doi: 10.1159/000342226.

1134. Angelakis E, Liouta E, Andreadis N, Korfias S, Stranjalis G, Ktonas P, et al. Clinical improvement in non-responsive patients with transcranial direct current stimulation. Stereotactic and Functional Neurosurgery. 2012;90:4. doi: 10.1159/000342226.

1135. Arendash GW. Transcranial electromagnetic treatment against Alzheimer's disease: why it has the potential to trump Alzheimer's disease drug development. J Alzheimers Dis. 2012;32(2):243-66. Epub 2012/07/20. doi: 10.3233/jad-2012-120943. PubMed PMID: 22810103.

1136. Arnsten AFT, Wang MJ, Paspalas CD. Neuromodulation of Thought: Flexibilities and Vulnerabilities in Prefrontal Cortical Network Synapses. Neuron. 2012;76(1):223-39. doi: 10.1016/j.neuron.2012.08.038. PubMed PMID: WOS:000309799000016.

1137. Auxemery Y. Etiopathogenic perspectives on chronic psycho

traumatic and chronic psychotic symptoms: the hypothesis of a hyperdopaminergic endophenotype of PTSD. Med Hypotheses. 2012;79(5):667-72. Epub 2012/09/04. doi: 10.1016/j.mehy.2012.08.005. PubMed PMID: 22939767.

1138. Auxéméry Y. [Posttraumatic stress disorder (PTSD) as a consequence of the interaction between an individual genetic susceptibility, a traumatogenic event and a social context]. Encephale. 2012;38(5):373-80. Epub 2012/10/16. doi: 10.1016/j.encep.2011.12.003. PubMed PMID: 23062450.

1139. Bagnato S, Boccagni C, Sant'Angelo A, Prestandrea C, Rizzo S, Galardi G. Patients in a vegetative state following traumatic brain injury display a reduced intracortical modulation. Clin Neurophysiol. 2012;123(10):1937-41. Epub 2012/05/09. doi: 10.1016/j.clinph.2012.03.014. PubMed PMID: 22560638.

1140. Bartolommei N, Lattanzi L, Callari A, Cosentino L, Luchini F, Mauri M. Catatonia: A critical review and therapeutic recommendations. Journal of Psychopathology. 2012;18(3):234-46.

1141. Bashir S, Vernet M, Yoo WK, Mizrahi I, Theoret H, Pascual-Leone A. Changes in cortical plasticity after mild traumatic brain injury. Restor Neurol Neurosci. 2012;30(4):277-82. Epub 2012/05/19. doi: 10.3233/rnn-2012-110207. PubMed PMID: 22596356; PubMed Central PMCID: PMCPMC3951777.

1142. Bates KA, Clark VW, Meloni BP, Dunlop SA, Rodger J. Short-term low intensity PMF does not improve functional or histological outcomes in a rat model of transient focal cerebral ischemia. Brain Research. 2012;1458:76-85. doi: 10.1016/j.brainres.2012.04.006. PubMed PMID: WOS:000305365900008.
1143. Beigh Z, Ahmad MM, Yousuf A, Ahmad RP. Role of topical heparine in healing of traumatic tympanic membrane perforations. Indian Journal of Otology. 2012;18(3):143-7. doi: 10.4103/0971-7749.103442.
1144. Belkin A, Lescovets V, Lescovets E, Vikulova D, Voronchychina S, Souslov S. Navigated brain stimulation in diagnosis of minimally conscious state. European Journal of Anaesthesiology. 2012;29:S22-S3. doi: 10.1097/01.EJA.0000412518.25201.32.
1145. Bellande B, Winicur Z, Gallo B, Turner M, Saulino M, Heath S, et al. Competency-based curricula in DBS and ITB therapies for movement disorders. Neurology. 2012;78(1). doi: 10.1212/WNL.78.1.
1146. Best SR, Pavel DG, Chang YC. Role of high definition brain SPECT in the evaluation of non-convulsive epilepsy co-morbid to mental disorder. Epilepsy and Behavior. 2012;24(2):185. doi: 10.1016/j.yebeh.2011.04.021.
1147. Bikson M, Rahman A, Datta A, Fregni F, Merabet L.

High-Resolution Modeling Assisted Design of Customized and Individualized Transcranial Direct Current Stimulation Protocols. Neuromodulation. 2012;15(4):306-15. doi: 10.1111/j.1525-1403.2012.00481.x. PubMed PMID: WOS:000307219900006.

1148. Birbaumer N, Liberati G. Motor and spatial congnition in minimally consciouos states. Cognitive Processing. 2012;13(1):S7. doi: 10.1007/s10339-012-0510-8.

1149. Bogdanova Y, Verfaellie M. Cognitive Sequelae of Blast-Induced Traumatic Brain Injury: Recovery and Rehabilitation. Neuropsychology Review. 2012;22(1):4-20. doi: 10.1007/s11065-012-9192-3. PubMed PMID: WOS:000300892100003.

1150. Boly M. Cerebral connectivity in disorders of consciousness. British Journal of Anaesthesia. 2012;108(2):337P-8P. doi: 10.1093/bja/aer442.

1151. Boly M, Massimini M, Garrido MI, Gosseries O, Noirhomme Q, Laureys S, et al. Brain connectivity in disorders of consciousness. Brain Connect. 2012;2(1):1-10. Epub 2012/04/20. doi: 10.1089/brain.2011.0049. PubMed PMID: 22512333.

1152. Borckardt JJ, Bikson M, Frohman H, Reeves ST, Datta A, Bansal V, et al. A Pilot Study of the Tolerability and Effects of

High-Definition Transcranial Direct Current Stimulation (HD-tDCS) on Pain Perception. Journal of Pain. 2012;13(2):112-20. doi: 10.1016/j.jpain.2011.07.001. PubMed PMID: WOS:000300594200002.

1153. Burton SD, Ermentrout GB, Urban NN. Intrinsic heterogeneity in oscillatory dynamics limits correlation-induced neural synchronization. Journal of Neurophysiology. 2012;108(8):2115-33. doi: 10.1152/jn.00362.2012. PubMed PMID: WOS:000310057500004.

1154. Çakir EDP, Mutlu FŞ, Eren E, Paşa AÖ, Sağlam H, Tarim Ö. Testicular adrenal rest tumors in patients with congenital adrenal hyperplasia. JCRPE Journal of Clinical Research in Pediatric Endocrinology. 2012;4(2):94-100. doi: 10.4274/jcrpe.563.

1155. Cantone M, Lanza G, Pennisi M, Puglisi V, Vinciguerra L, Ricceri R, et al. Motor cortex excitability in patients with vascular cognitive impairment-no dementia: A follow-up study. European Journal of Neurology. 2012;19:225. doi: 10.1111/j.1468-1331.2012.03888.x.

1156. Cavinato M, Iaia V, Piccione F. Repeated sessions of sub-threshold 20-Hz rTMS. Potential cumulative effects in a brain-injured patient. Clin Neurophysiol. 2012;123(9):1893-5. Epub 2012/03/13. doi: 10.1016/j.clinph.2012.02.066. PubMed PMID:

22405994.

1157. Chew E, Teh I, Ang KK, Phua KS, Zhao L, Chang W, et al. Combined transcranial direct current stimulation and brain-computer interface rehabilitation - A new approach to motor rehabilitation. Brain Injury. 2012;26(4-5):782. doi: 10.3109/02699052.2012.660091.

1158. Chollet F, Albucher JF. Strategies to augment recovery after stroke. Current Treatment Options in Neurology. 2012;14(6):531-40. doi: 10.1007/s11940-012-0196-3.

1159. Chung H, Dai T, Sharma SK, Huang Y-Y, Carroll JD, Hamblin MR. The Nuts and Bolts of Low-level Laser (Light) Therapy. Annals of Biomedical Engineering. 2012;40(2):516-33. doi: 10.1007/s10439-011-0454-7. PubMed PMID: WOS:000300485100019.

1160. Claßen J. Neuroplasticity in MS (#152). Multiple Sclerosis. 2012;18(5):S9. doi: 10.1177/1352458512444204.

1161. Conti A. Comment. Acta Neurochirurgica. 2012;154(6):1104. doi: 10.1007/s00701-012-1326-0.

1162. Costa V. USE OF NONINVASIVE CEREBRAL STIMULATION TECHNIQUES IN APHASIA: AN UPDATING. Acta Medica Mediterranea. 2012;28(2):105-8. PubMed PMID: WOS:000310121900008.

1163. Danilov Y, Tyler M, Kazcmarek K. NEW APPROACH TO

CHRONIC TBI REHABILITATION: CRANIAL NERVE NONINVASIVE NEUROMODULATION (CN-NINM TECHNOLOGY). Journal of Neurotrauma. 2012;29(10):A97-A. PubMed PMID: WOS:000306244400163.

1164. De Beaumont L, Tremblay S, Poirier J, Lassonde M, Théoret H. Altered bidirectional plasticity and reduced implicit motor learning in concussed athletes. Cereb Cortex. 2012;22(1):112-21. Epub 2011/05/17. doi: 10.1093/cercor/bhr096. PubMed PMID: 21572090.

1165. De Freitas GR. Aphasia and other language disorders. Frontiers of Neurology and Neuroscience2012. p. 41-5.

1166. de Haan B, Karnath H-O, Driver J. Mechanisms and anatomy of unilateral extinction after brain injury. Neuropsychologia. 2012;50(6):1045-53. doi: 10.1016/j.neuropsychologia.2012.02.015. PubMed PMID: WOS:000304724500005.

1167. de Haan B, Karnath HO. Objections against the view of visual extinction as an attentional disengagement deficit: The interaction between spatial position and temporal modulation. Cortex. 2012;48(8):972-9. doi: 10.1016/j.cortex.2011.03.011.

1168. De Salvo S, Bramanti P, Marino S. Clinical differentiation and outcome evaluation in vegetative and minimally conscious state patients:the neurophysiological approach. Funct Neurol. 2012;27(3):155-62. Epub 2013/02/14. PubMed PMID: 23402676;

PubMed Central PMCID: PMCPMC3812768.
1169. Delnooz CCS, Van De Warrenburg BPC. Current and future medical treatment in primary dystonia. Therapeutic Advances in Neurological Disorders. 2012;5(4):221-40. doi: 10.1177/1756285612447261.
1170. Demirtas-Tatlidede A, Vahabzadeh-Hagh AM, Bernabeu M, Tormos JM, Pascual-Leone A. Noninvasive brain stimulation in traumatic brain injury. J Head Trauma Rehabil. 2012;27(4):274-92. Epub 2011/06/22. doi: 10.1097/HTR.0b013e318217df55. PubMed PMID: 21691215; PubMed Central PMCID: PMCPMC3342413.
1171. Dinn NA, Houlden DA, Potapova E, Mainprize TG. A comparison of SSEP amplitude recorded from the scalp and brain surface. Canadian Journal of Neurological Sciences. 2012;39(3):S26.
1172. Dumas R, Richieri R, Guedj E, Auquier P, Lancon C, Boyer L. Improvement of health-related quality of life in depression after transcranial magnetic stimulation in a naturalistic trial is associated with decreased perfusion in precuneus. Health Qual Life Outcomes. 2012;10:87. Epub 2012/07/31. doi: 10.1186/1477-7525-10-87. PubMed PMID: 22838740; PubMed Central PMCID: PMCPMC3492154.
1173. Dutta D, Sharma S, Das J, Gairola RM. Development of a regional rain retrieval algorithm for exclusive mesoscale convective

systems over peninsular India. Advances in Space Research. 2012;49(12):1750-62. doi: 10.1016/j.asr.2012.02.028. PubMed PMID: WOS:000304787900012.
1174. Eggermont JJ, Roberts LE. The neuroscience of tinnitus: Understanding abnormal and normal auditory perception. Frontiers in Systems Neuroscience. 2012(JULY 2012). doi: 10.3389/fnsys.2012.00053.
1175. Espinoza J, Cortes C, Valenzuela J, Sandoval R, Gómez R, Céspedes P, et al. Respiratory syncytial virus infection in mice reduces spatial learning capacity and long term potentiation. Journal of Immunology. 2012;188(1).
1176. Frenkel-Toledo S, Liebermann DG, Bentin S, Soroker N. Dysfunction of the Human Mirror Neuron System in Ideomotor Apraxia: Evidence from Mu Suppression. Journal of Cognitive Neuroscience. 2012;10:775-91. doi: 10.1162/jocn_a_00936.
1177. Gaede G, Tiede M, Lorenz I, Zimmermann H, Pfueller CF, Dörr J, et al. Safety and efficacy of repetitive deep transcranial magnetic stimulation in multiple sclerosis. Journal of Neurology. 2012;259(1):S190-S1. doi: 10.1007/s00415-012-6524-4.
1178. Gall C, Schmidt S, Brandt SA, Fedorov AB, Sabel BA. Non-invasive alternating current stimulation improves subjective visual functioning in optic neuropathy. Brain Injury.

2012;26(4-5):521-2. doi: 10.3109/02699052.2012.660091.

1179. Gao G, Jiang J, Cooper E. Short term right median nerve stimulation arouse profound variation of brain blood perfusion and microRNA expression on traumatic coma patients. Brain Injury. 2012;26(4-5):401-2. PubMed PMID: WOS:000304104600175.

1180. Ghaffarieh A, Ghaffarpasand F, Dehghankhalili M, Honarpisheh N, Nirumandi S, Tanideh N. Effect of transcutaneous electrical stimulation on rabbit corneal epithelial cell migration. Cornea. 2012;31(5):559-63. doi: 10.1097/ICO.0b013e31823f8b2a.

1181. Gheysarzadeh A, Yazdanparast R. Inhibition of H2O2-induced cell death through FOXO1 modulation by EUK-172 in SK-N-MC cells. European Journal of Pharmacology. 2012;697(1-3):47-52. doi: 10.1016/j.ejphar.2012.09.036.

1182. Giacino J, Fins JJ, Machado A, Schiff ND. Central Thalamic Deep Brain Stimulation to Promote Recovery from Chronic Posttraumatic Minimally Conscious State: Challenges and Opportunities. Neuromodulation. 2012;15(4):339-49. doi: 10.1111/j.1525-1403.2012.00458.x. PubMed PMID: WOS:000307219900009.

1183. Gibbons CJ, Young CA. Assessing and managing depression and fatigue in motor neuron disease. Neurodegenerative Disease Management. 2012;2(4):401-9. doi: 10.2217/nmt.12.42.

1184. Golaszewski SM, Bergmann J, Christova M, Kunz AB, Kronbichler M, Rafolt D, et al. Modulation of motor cortex excitability by different levels of whole-hand afferent electrical stimulation. Clin Neurophysiol. 2012;123(1):193-9. Epub 2011/07/19. doi: 10.1016/j.clinph.2011.06.010. PubMed PMID: 21764634.
1185. Golaszewski SM, Seidl M, Kunz AB, Caleri F, Lochner P, Cataldo S, et al. Functional involvement of cerebral cortex in patients with sleep-wake disturbances after traumatic brain injury: A TMS study. Neurorehabilitation and Neural Repair. 2012;26(4):427. doi: 10.1177/1545968312447071.
1186. Gordon AL, di Maggio A. Rehabilitation for Children After Acquired Brain Injury: Current and Emerging Approaches. Pediatric Neurology. 2012;46(6):339-44. doi: 10.1016/j.pediatrneurol.2012.02.029. PubMed PMID: WOS:000305301800001.
1187. Hadar AA, Makris S, Yarrow K. Single-pulse TMS related syncopal spell in a healthy subject. Brain Stimul. 2012;5(4):652-3. Epub 2011/09/21. doi: 10.1016/j.brs.2011.08.001. PubMed PMID: 21930450.
1188. Hamdy S. Transcranial magnetic stimulation - A diagnostic and therapeutic tool in swallowing disorders. Neurogastroenterology and Motility. 2012;24:9. doi: 10.1111/j.1365-2982.2012.01995.x.

1189. Han TR, Oh BM, Kim JY. Molecular mechanism for cumulative effects of repetitive transcranial magnetic stimulation on neural plasticity in rats. Brain Injury. 2012;26(4-5):598-9. doi: 10.3109/02699052.2012.660091.

1190. Hansen N, Seiler C, Rumpf J, Kraft P, Dlaske H, Abele-Horn M, et al. Human tuberculous meningitis caused by mycobacterium caprae. Case Reports in Neurology. 2012;4(1):54-60. doi: 10.1159/000337299.

1191. Heesen M, Veeser M. Analgesia in obstetrics. Geburtshilfe und Frauenheilkunde. 2012;72(7):596-601. doi: 10.1055/s-0031-1298444.

1192. Hong JH, Lee J, Cho YW, Byun WM, Cho HK, Son SM, et al. Limb apraxia in a patient with cerebral infarct: Diffusion tensor tractography study. NeuroRehabilitation. 2012;30(4):255-9. doi: 10.3233/NRE-2012-0753.

1193. Horovitz SG, Horwitz B. Introduction to research topic - Brain connectivity analysis: Investigating brain disorders. Part 2: Original research articles. Frontiers in Systems Neuroscience. 2012(FEBRUARY 2012). doi: 10.3389/fnsys.2012.00004.

1194. Horwitz B, Horovitz SG. Introduction to research topic - Brain connectivity analysis: Investigating brain disorders. Part 1: The review articles. Frontiers in Systems Neuroscience. 2012(FEBRUARY 2012).

doi: 10.3389/fnsys.2012.00003.
1195. Hsieh TH, Hameed MQ, Chen JJ, Wang Y, Amandusson A, Pascual-Leone A, et al. Progressive loss of intracortical inhibition following traumatic brain injury detected by transcranial magnetic stimulation and mechanomyogram in rats. Epilepsy Currents. 2012;12(1).
1196. Huang H, Chen L, Sanberg PR. Clinical achievements, obstacles, falsehoods, and future directions of cell-based neurorestoratology. Cell Transplantation. 2012;21(SUPPL. 1):S3-S11. doi: 10.3727/096368912X633725.
1197. Hughes JR. THE SAVANT SYNDROME AND ITS POSSIBLE RELATIONSHIP TO EPILEPSY. In: Ahmad SI, editor. Neurodegenerative Diseases. Advances in Experimental Medicine and Biology. 7242012. p. 332-43.
1198. Hummel F. What can we learn from system neurosciences (fMRI, TMS) (#149). Multiple Sclerosis. 2012;18(5):S9. doi: 10.1177/1352458512444204.
1199. Inoue M, Kojima Y, Mima T, Sawamoto N, Matsuhashi M, Fumuro T, et al. Pathophysiology of unilateral asterixis due to thalamic lesion. Clin Neurophysiol. 2012;123(9):1858-64. Epub 2012/03/20. doi: 10.1016/j.clinph.2012.01.021. PubMed PMID: 22425586.

1200. Irct2012102311234N. Effects of Transcranial Direct Current Stimulation (tDCS) over Craving in abstinence methamphetamine user. https://trialsearchwhoint/Trial2aspx?TrialID=IRCT2012102311234N1. 2012. PubMed PMID: CN-01806758.

1201. Isagulyan E, Shabalov V, Salova E, Khitj M. Motor cortex stimulation in the treatment of neurogenic pain syndromes. Stereotactic and Functional Neurosurgery. 2012;90:80. doi: 10.1159/000342226.

1202. Ivarsen A, Hjortdal J. Seven-year changes in corneal power and aberrations after PRK or LASIK. Invest Ophthalmol Vis Sci. 2012;53(10):6011-6. Epub 2012/08/11. doi: 10.1167/iovs.12-10208. PubMed PMID: 22879417.

1203. Izadi A, Lee D, Gurkoff G, Echeverri A, Schwartzkroin P, Berman R, et al. TBI leads to alterations in hippocampal theta, beta and gamma frequencies during rest and exploration in rats. Journal of Neurotrauma. 2012;29(10):A111. doi: 10.1089/neu.2012.9943.

1204. Jadhav T, Cross JH. Surgical approaches to treating epilepsy in children. Current Treatment Options in Neurology. 2012;14(6):620-9. doi: 10.1007/s11940-012-0203-8.

1205. James JE. Potential advances in knowledge from cross-fertilization between different fields of caffeine research. Journal of Caffeine Research. 2012;2(1):1-2. doi: 10.1089/jcr.2012.1216.

1206. Jasek L, Siger M, Berkowicz T, Selmaj K. Multiparametric MRI analysis of patients with late multiple sclerosis. Neurology. 2012;78(1). doi: 10.1212/WNL.78.1.

1207. Ju H, Van Dongen AMJ, Xu JX. A neural network model for motor rehabilitation. Proceedings of Singapore Healthcare. 2012;21:S102. doi: 10.1177/201010581202100S101.

1208. Kahali P, Tadayon SH, Zali A, Abbasian A. Transcranial direct current stimulation in a vegetative state patient: a case study. European Journal of Neurology. 2012;19:579-. PubMed PMID: WOS:000309359704006.

1209. Kaindl AM, Degos V, Peineau S, Gouadon E, Chhor V, Loron G, et al. Activation of microglial N-methyl-D-aspartate receptors triggers inflammation and neuronal cell death in the developing and mature brain. Annals of Neurology. 2012;72(4):536-49. doi: 10.1002/ana.23626.

1210. Kang EK, Kim DY, Paik NJ. Transcranial direct current stimulation of the left prefrontal cortex improves attention in patients with traumatic brain injury: a pilot study. J Rehabil Med. 2012;44(4):346-50. Epub 2012/03/22. doi: 10.2340/16501977-0947. PubMed PMID: 22434324.

1211. Kang EK, Kim DY, Paik NJ. Transcranial direct curent stimulation of the left prefrontal cortex improves attention in patients

with traumatic brain injury: A pilot study. Journal of Rehabilitation Medicine. 2012;44(4):346-50. doi: 10.2340/16501977-0947.

1212. Kantak S, Liao WW, Wittenberg GF, Waller SM. Posture-related modulation in motor cortical excitability of proximal and distal upper extremity muscles. Archives of Physical Medicine and Rehabilitation. 2012;93(10):E15-E6. doi: 10.1016/j.apmr.2012.08.038.

1213. Kantak SS, Stinear JW, Buch ER, Cohen LG. Rewiring the brain: potential role of the premotor cortex in motor control, learning, and recovery of function following brain injury. Neurorehabil Neural Repair. 2012;26(3):282-92. Epub 2011/09/20. doi: 10.1177/1545968311420845. PubMed PMID: 21926382; PubMed Central PMCID: PMCPMC4886541.

1214. Kawasaki Y, Fujiki M, Ooba H, Sugita K, Hikawa T, Abe T, et al. Short latency afferent inhibition associated with cortical compression and memory impairment in patients with chronic subdural hematoma. Clin Neurol Neurosurg. 2012;114(7):976-80. Epub 2012/04/10. doi: 10.1016/j.clineuro.2012.02.037. PubMed PMID: 22482870.

1215. Kemmerer D, Rudrauf D, Manzel K, Tranel D. Behavioral patterns and lesion sites associated with impaired processing of lexical and conceptual knowledge of actions. Cortex. 2012;48(7):826-48. doi:

10.1016/j.cortex.2010.11.001. PubMed PMID: WOS:000305710600004.

1216. Khandan O, Famili A, Kahook MY, Rao MP. Titanium-based, fenestrated, in-plane microneedles for passive ocular drug delivery. Annual International Conference of the IEEE Engineering in Medicine and Biology Society IEEE Engineering in Medicine and Biology Society Annual International Conference. 2012;2012:6572-5. doi: 10.1109/embc.2012.6347500. PubMed PMID: MEDLINE:23367435.

1217. Kidgell D, Daly R, Tooley G, Lum J, Pearce A. Time-course changes in motor cortical excitability following low level constant transcranial direct current stimulation. Journal of Science and Medicine in Sport. 2012;15:S119. doi: 10.1016/j.jsams.2012.11.290.

1218. Kim HM, Chun MH, Lee SI, Lee SJ, Kim HJ, Kim SB. The effect of repetitive transcranial magnetic stimulation on brain injured patients with dysphagia. Neurorehabilitation and Neural Repair. 2012;26(4):407. doi: 10.1177/1545968312447071.

1219. Kim J, Whyte J, Patel S, Europa E, Wang J, Coslett HB, et al. Methylphenidate modulates sustained attention and cortical activation in survivors of traumatic brain injury: A perfusion fMRI study. Psychopharmacology. 2012;222(1):47-57. doi: 10.1007/s00213-011-2622-8.

1220. Kiziltan ME, Sevindik MS, Bozluolcay M. Auditory startle

reflex in cerebrovascular disease. Clinical Neurophysiology. 2012;123(2):e3. doi: 10.1016/j.clinph.2011.07.011.

1221. Koivisto M, Henriksson L, Revonsuo A, Railo H. Unconscious response priming by shape depends on geniculostriate visual projection. European Journal of Neuroscience. 2012;35(3-4):623-33. doi: 10.1111/j.1460-9568.2011.07973.x.

1222. Krishnan M, Smith N, Donders J. Use of the Tower of London - Drexel University, Second Edition (TOLDX) in adults with traumatic brain injury. Clin Neuropsychol. 2012;26(6):951-64. Epub 2012/08/02. doi: 10.1080/13854046.2012.708166. PubMed PMID: 22849344.

1223. Kumaria A, Tolias CM. Is there a role for vagus nerve stimulation therapy as a treatment of traumatic brain injury? British Journal of Neurosurgery. 2012;26(3):316-20. doi: 10.3109/02688697.2012.663517. PubMed PMID: WOS:000304524300005.

1224. Kunz AB, Christova M, Gallasch E, Seidl M, Bergmann J, Krenn Y, et al. Modulatory effects on human sensorimotor cortex activation by whole hand vibration stimulation. European Journal of Neurology. 2012;19:221. doi: 10.1111/j.1468-1331.2012.03888.x.

1225. Kurata J. Corticocortical-evoked potential monitoring as a tool to investigate anaesthetic-induced unconsciousness: A preliminary report. British Journal of Anaesthesia. 2012;108(2):346P-7P. doi:

10.1093/bja/aer442.

1226. Lancioni GE, Singh NN, O'Reilly MF, Sigafoos J, Amenduni MT, Navarro J, et al. Microswitch technology and contingent stimulation to promote adaptive engagement in persons with minimally conscious state: a case evaluation. Cognitive processing. 2012;13(2):133-7. doi: 10.1007/s10339-011-0428-6. PubMed PMID: MEDLINE:22131129.

1227. Lancioni GE, Singh NN, O'Reilly MF, Sigafoos J, Belardinelli MO, Buonocunto F, et al. Promoting adaptive behavior in persons with acquired brain injury, extensive motor and communication disabilities, and consciousness disorders. Research in developmental disabilities. 2012;33(6):1964-74. doi: 10.1016/j.ridd.2012.05.027. PubMed PMID: MEDLINE:22738766.

1228. Lee DJ, Gurkoff G, Izadi A, Schwartzkroin P, Berman R, Muizelaar J, et al. Medial septal theta frequency stimulation improves spatial working memory following traumatic brain injury. Journal of Neurosurgery. 2012;117(2):A422.

1229. Lee DJ, Izadi A, Gurkoff G, Berman R, Ekstrom A, Schwartzkroin P, et al. Frequency-dependent effects of medial septal stimulation for cognitive improvement following traumatic brain injury. Journal of Neurotrauma. 2012;29(10):A10-A1. doi: 10.1089/neu.2012.9943.

1230. Lee YH, Ko A, Kim Y, Yong S. Effect of combined peripheral motor electrical stimulation and transcranial direct current stimulation(TDCS) to finger movement in patients with hemiparesis. Brain Injury. 2012;26(4-5):442-3. doi: 10.3109/02699052.2012.660091.

1231. Lin RS, Wang W, Guo A. Management of distal posterior inferior cerebellar artery aneurysms. Journal of Craniofacial Surgery. 2012;23(5):1388-90. doi: 10.1097/SCS.0b013e31825653ce.

1232. Livingston S. Electrophysiologic evidence for the effects of acute cerebral concussion in a collegiatewomen's soccer player. Journal of Head Trauma Rehabilitation. 2012;27(5):E12-E3. doi: 10.1097/HTR.0b013e3182690d04.

1233. Livingston SC, Goodkin HP, Hertel JN, Saliba EN, Barth JT, Ingersoll CD. Differential rates of recovery after acute sport-related concussion: electrophysiologic, symptomatic, and neurocognitive indices. J Clin Neurophysiol. 2012;29(1):23-32. Epub 2012/02/23. doi: 10.1097/WNP.0b013e318246ae46. PubMed PMID: 22353982.

1234. Lu Y-J, Wei K-C, Ma C-CM, Yang S-Y, Chen J-P. Dual targeted delivery of doxorubicin to cancer cells using folate-conjugated magnetic multi-walled carbon nanotubes. Colloids and Surfaces B-Biointerfaces. 2012;89:1-9. doi: 10.1016/j.colsurfb.2011.08.001. PubMed PMID:

WOS:000297495000001.
1235. Mandat T, Koziara H, Barszcz S, Rola R, Karliński M, Sliwińska A, et al. Motor cortex stimulation in the treatment of neuropathic pain. Neurol Neurochir Pol. 2012;46(5):428-35. Epub 2012/11/20. doi: 10.5114/ninp.2012.31352. PubMed PMID: 23161186.
1236. Martin MJ, Hatch Q, Cotton B, Holcomb J. The use of temporary abdominal closure in low-risk trauma patients: Helpful or harmful? Journal of Trauma and Acute Care Surgery. 2012;72(3):601-8. doi: 10.1097/TA.0b013e31824483b7.
1237. Massimini M, Ferrarelli F, Sarasso S, Tononi G. Cortical mechanisms of loss of consciousness: insight from TMS/EEG studies. Arch Ital Biol. 2012;150(2-3):44-55. Epub 2012/11/21. doi: 10.4449/aib.v150i2.1361. PubMed PMID: 23165870.
1238. Matsumoto H, Ugawa Y. Neurophysiological analyses of asterixis utilizing innovative approaches. Clinical Neurophysiology. 2012;123(9):1695-6. doi: 10.1016/j.clinph.2012.02.063. PubMed PMID: WOS:000307183800006.
1239. McDonald S, Anderson V, Ponsford J, Tate R, Togher L, Morgan A, et al. Moving ahead: A new centre of research excellence in brain recovery, focusing on psychosocial reintegration following traumatic brain injury. Brain Impairment. 2012;13(2):256-70. doi:

10.1017/BrImp.2012.21.
1240. McIntyre WF, Pérez-Riera AR, Femenía F, Baranchuk A. Coexisting early repolarization pattern and Brugada syndrome: Recognition of potentially overlapping entities. Journal of Electrocardiology. 2012;45(3):195-8. doi: 10.1016/j.jelectrocard.2011.10.008.
1241. Messina G, Franzini A, Dones I, Broggi G. Motor cortex stimulation for upper limb fixed dystonia. Stereotactic and Functional Neurosurgery. 2012;90:41. doi: 10.1159/000342226.
1242. Miyai I, Mihara M, Hattori N, Hatakenaka M, Kawano T, Yagura H. Contribution of brain function analysis to the evolution of neurorehabilitation. Clinical Neurology. 2012;52(11):1174-7. doi: 10.5692/clinicalneurol.52.1174.
1243. Moennich D, Troost EGC, Kaanders JHAM, Oyen WJG, Alber M, Thorwarth D. Modelling and simulation of the influence of acute and chronic hypoxia on [18F]Fmiso PET imaging. Radiotherapy and Oncology. 2012;103:S335. doi: 10.1016/S0167-8140(12)71190-8.
1244. Mohasseb D, Abou Alfa A, Reda M, Khalil I, El Gafi R. Utility of electrophysiological studies in assessing prognosis and recovery of traumatic spinal cord injuries. Journal of Neurology. 2012;259(1):S185. doi: 10.1007/s00415-012-6524-4.
1245. Monsalve GA. Motor cortex stimulation for facial chronic

neuropathic pain: A review of the literature. Surg Neurol Int. 2012;3(Suppl 4):S290-311. Epub 2012/12/12. doi: 10.4103/2152-7806.103023. PubMed PMID: 23230534; PubMed Central PMCID: PMCPMC3514920.

1246. Munoz Marron E, Redolar-Ripoll D, Boixados M, Andreu LL. New approaches to the treatment of neglect: Transcranial magnetic stimulation. Neurorehabilitation and Neural Repair. 2012;26(4):420-1. doi: 10.1177/1545968312447071.

1247. Muñoz-Marron E, Robles N, Andreu-Barrachina L, Boixados-Angles M, Redolar-Ripoll D. Brain stimulation techniques: Non-invasive treatments for depression. European Psychiatry. 2012;27. doi: 10.1016/S0924-9338(12)75545-1.

1248. Murray LL. Direct and indirect treatment approaches for addressing short-term or working memory deficits in aphasia. Aphasiology. 2012;26(3-4):317-37. doi: 10.1080/02687038.2011.589894.

1249. Mylius V, Ayache SS, Teepker M, Kappus C, Kolodziej M, Rosenow F, et al. [Transcranial magnetic stimulation and motor cortex stimulation in neuropathic pain]. Schmerz. 2012;26(6):655-60. Epub 2012/11/28. doi: 10.1007/s00482-012-1243-4. PubMed PMID: 23183989.

1250. Nct. Effects of Huperzine A in Treatment of Moderate to

Severe TBI. https://clinicaltrialsgov/show/NCT01676311. 2012. PubMed PMID: CN-01585265.
1251. Nct. Relationship Between Attention and Emotional Regulation Post-Traumatic Brain Injury (TBI). https://clinicaltrialsgov/show/NCT01681589. 2012. PubMed PMID: CN-01537517.
1252. Nct. Combined Transcranial Magnetic Stimulation and Cognitive Treatment in Blast Traumatic Brain Injury. https://clinicaltrialsgov/show/NCT01596569. 2012. PubMed PMID: CN-01596380.
1253. Nct. Improving Spinal Cord Injury Rehabilitation Interventions by Retraining the Brain With Stimulation. https://clinicaltrialsgov/show/NCT01539109. 2012. PubMed PMID: CN-02044113.
1254. Nct. Directing Neuroplasticity to Improve Rehabilitative Outcomes of the Upper Limb in Incomplete Quadriplegia. https://clinicaltrialsgov/show/NCT01539109. 2012. PubMed PMID: CN-01591278.
1255. Nct. Precision Versus Endurance Training to Improve Walking After Chronic Incomplete SCI. https://clinicaltrialsgov/show/NCT01765153. 2012. PubMed PMID: CN-02035526.

1256. Niimi M, Kakuda W, Abo M. Brain plasticity and stroke rehabilitation. Tokyo Jikeikai Medical Journal. 2012;127(4):151-67.

1257. Nizard J, Lefaucheur JP, Helbert M, de Chauvigny E, Nguyen JP. Non-invasive stimulation therapies for the treatment of refractory pain. Discov Med. 2012;14(74):21-31. Epub 2012/08/01. PubMed PMID: 22846200.

1258. Nizard J, Raoul S, Nguyen JP, Lefaucheur JP. Invasive stimulation therapies for the treatment of refractory pain. Discov Med. 2012;14(77):237-46. Epub 2012/11/02. PubMed PMID: 23114579.

1259. Nunes KF, Manzano GM, Nobrega JAM, Giuliano LMP. Motor skills were affected by intrauterine somatosensory brain injury that mimics a brachial plexus palsy. Clinical Neurophysiology. 2012;123(6):e22-e3. doi: 10.1016/j.clinph.2011.11.103.

1260. Nuti C, Vassal F, Mertens P, Lemaire J-J, Magnin M, Peyron R. Improved Dexterity after Chronic Electrical Stimulation of the Motor Cortex for Central Pain: A Special Relevance for Thalamic Syndrome. Stereotactic and Functional Neurosurgery. 2012;90(6):370-8. doi: 10.1159/000338681. PubMed PMID: WOS:000310357500005.

1261. Oberdorfer P, Kongthavonsakul K, Intachumpoo J, Odell S. A 14-year-old girl with tuberculous otitis media and brain abscess. BMJ Case Reports. 2012. doi: 10.1136/bcr-2012-006618.

1262. Oliviero A, Arevalo-Martin A, Rotondi M, García-Ovejero D,

Mordillo-Mateos L, Lozano-Sicilia A, et al. CB1 receptor antagonism/inverse agonism increases motor system excitability in humans. Eur Neuropsychopharmacol. 2012;22(1):27-35. Epub 2011/05/17. doi: 10.1016/j.euroneuro.2011.04.004. PubMed PMID: 21571507.

1263. Pang T, Drislane FW. Treatment of nonconvulsive status epilepticus. Current Treatment Options in Neurology. 2012;14(4):307-21. doi: 10.1007/s11940-012-0179-4.

1264. Panina OS, Zaitseva GA, Pokazanieva SA. Rehabilitation of neonates with perinatal injuries to the central nervous system using transcranial magnetotherapy. Neuroscience and Behavioral Physiology. 2012;42(7):745-7. doi: 10.1007/s11055-012-9628-5.

1265. Pape TLB. Safety and efficacy of repetitive transcranial magnetic stimulation (rTMS) for two persons in vegetative state. Archives of Physical Medicine and Rehabilitation. 2012;93(10):E15. doi: 10.1016/j.apmr.2012.08.035.

1266. Patel V, Griffith NC, Blackwood E, Dias M, Cordato DJ. Spectrum disorder of neuromyelitis optica in a patient presenting with intractable vomiting and hiccups, transverse myelitis and acute encephalopathy. Journal of Clinical Neuroscience. 2012;19(11):1576-8. doi: 10.1016/j.jocn.2012.03.007.

1267. Perruchoud C, Bovy M, Durrer A, Rosato M, Rutschmann B,

Mustaki JP, et al. Severe hypertension following accidental clonidine overdose during the refilling of an implanted intrathecal drug delivery system. Neuromodulation. 2012;15(1):31-4. doi: 10.1111/j.1525-1403.2011.00392.x.

1268. Piccione F, Genna C, Dotta S, Fusaro P, Silverio F, Cavinato M. Transcranial direct current stimulation can promote awareness in patients with alterate state of consciousness: A preliminary study. Cognitive Processing. 2012;13(1):S8-S9. doi: 10.1007/s10339-012-0510-8.

1269. Plow EB, Pascual-Leone A, Machado A. Brain stimulation in the treatment of chronic neuropathic and non-cancerous pain. J Pain. 2012;13(5):411-24. Epub 2012/04/10. doi: 10.1016/j.jpain.2012.02.001. PubMed PMID: 22484179; PubMed Central PMCID: PMCPMC3348447.

1270. Pralong E, Diserens K, Vuadens P, Levivier M, Bloch J. Measure of spasticity modulation by cervical epidural stimulation in a case of hemispasticity. Stereotactic and Functional Neurosurgery. 2012;90:143. doi: 10.1159/000342226.

1271. Priori A, Mameli F, Ferrucci R, Barbieri S. Transcranial Direct Current Stimulation (tDCS) and Cognitive Decline. Journal of Alzheimers Disease. 2012;29:31-2. PubMed PMID: WOS:000303176400010.

1272. Railo H, Salminen-Vaparanta N, Henriksson L, Revonsuo A, Koivisto M. Unconscious and conscious processing of color rely on activity in early visual cortex: a TMS study. J Cogn Neurosci. 2012;24(4):819-29. Epub 2011/12/01. doi: 10.1162/jocn_a_00172. PubMed PMID: 22126671.

1273. Rastelli F, Toba M, Tchokotche C, Vincent S, Pradat-Diehl P, Valero-Cabré A. Repetitive transcranial magnetic stimulation (RTMS) for the rehabilitation of visuospatial neglect following right hemisphere strokes: Details from an ongoing multicenter double blind clinical trial. Journal of Head Trauma Rehabilitation. 2012;27(5):E8. doi: 10.1097/HTR.0b013e3182690d04.

1274. Renton T, Yilmaz Z, Gaballah K. Evaluation of trigeminal nerve injuries in relation to third molar surgery in a prospective patient cohort. Recommendations for prevention. Int J Oral Maxillofac Surg. 2012;41(12):1509-18. Epub 2012/09/29. doi: 10.1016/j.ijom.2012.06.025. PubMed PMID: 23017786.

1275. Riccio A, Mattia D, Simione L, Olivetti M, Cincotti F. Eye-gaze independent EEG-based brain-computer interfaces for communication. Journal of Neural Engineering. 2012;9(4). doi: 10.1088/1741-2560/9/4/045001. PubMed PMID: WOS:000306759600002.

1276. Rokyta R, Fricová J. Neurostimulation methods in the

treatment of chronic pain. Physiol Res. 2012;61(Suppl 2):S23-31. Epub 2012/11/21. doi: 10.33549/physiolres.932392. PubMed PMID: 23130900.

1277. Rosanova M, Gosseries O, Casarotto S, Boly M, Casali AG, Bruno MA, et al. Recovery of cortical effective connectivity and recovery of consciousness in vegetative patients. Brain. 2012;135(Pt 4):1308-20. Epub 2012/01/10. doi: 10.1093/brain/awr340. PubMed PMID: 22226806; PubMed Central PMCID: PMCPMC3326248.

1278. Rossetti AO. Hypothermia in refractory status epilepticus. Critical Care. 2012;16.

1279. Rothwell JC. Clinical applications of noninvasive electrical stimulation: problems and potential. Clin EEG Neurosci. 2012;43(3):209-14. Epub 2012/06/21. doi: 10.1177/1550059412444973. PubMed PMID: 22715492.

1280. Semashko V, Kistsen V, Evstigneev V. Blink reflex significance at estimation of epilepsy hypersynchronous activity. European Journal of Neurology. 2012;19:258. doi: 10.1111/j.1468-1331.2012.03888.x.

1281. Sharova EV, Mel'nikov AV, Novikova MR, Kulikov MA, Mats VN, Shishkina LV, et al. [Functional effects of transcranial electromagnetic stimulation (60 and 70 Hz) of the brain of intact rats and rats with acute brainstem damage]. Zh Vyssh Nerv Deiat Im I P

Pavlova. 2012;62(1):65-78. Epub 2012/05/10. PubMed PMID: 22567988.

1282. Singh TRR, Garland MJ, Migalska K, Salvador EC, Shaikh R, McCarthy HO, et al. Influence of a pore-forming agent on swelling, network parameters, and permeability of poly(ethylene glycol)-crosslinked poly(methyl vinyl ether-co-maleic acid) hydrogels: Application in transdermal delivery systems. Journal of Applied Polymer Science. 2012;125(4):2680-94. doi: 10.1002/app.36524. PubMed PMID: WOS:000303596200023.

1283. Siqueira F, Ibrahim V, Pineda CG, Pohlman D. Rehabilitation outcome for the treatment of trauma-induced trismus: A case report. PM and R. 2012;4(10):S221.

1284. Skaper SD. 1 st workshop on "palmitoylethanolamide: Biochemistry, Pharmacology and therapeutic use of a Pleiotropic anti-inflammatory lipid Mediator". CNS and Neurological Disorders - Drug Targets. 2012;11(3):3.

1285. Soto E, Hao J, Knotkova H, Cruciani RA. Central neurostimulation techniques for primary headaches. Techniques in Regional Anesthesia and Pain Management. 2012;16(1):47-56. doi: 10.1053/j.trap.2012.11.007.

1286. Sterr A, Conforto AB. Plasticity of Adult Sensorimotor System in Severe Brain Infarcts: Challenges and Opportunities. Neural

Plasticity. 2012;2012. doi: 10.1155/2012/970136. PubMed PMID: WOS:000302658500001.

1287. Stidd DA, Wuollet AL, Bowden K, Price T, Patwardhan A, Barker S, et al. Peripheral nerve stimulation for trigeminal neuropathic pain. Pain Physician. 2012;15(1):27-33. Epub 2012/01/25. PubMed PMID: 22270735; PubMed Central PMCID: PMCPMC4664452.

1288. Taira T. Tinnitus: From traditional concept to challenges to loosen the gordian knot. World Neurosurgery. 2012;77(5-6):651-3. doi: 10.1016/j.wneu.2011.10.017.

1289. Takada K, Ito Y. Transdermal insulin application system with dissolving microneedles. Journal of Diabetes Science and Technology. 2012;6(2):A176-A7.

1290. Takeuchi N, Oouchida Y, Izumi S-I. Motor Control and Neural Plasticity through Interhemispheric Interactions. Neural Plasticity. 2012;2012. doi: 10.1155/2012/823285. PubMed PMID: WOS:000313169100001.

1291. Taksande A, Prabhu S, Venkatesh S. Cardiovascular aspect of beta-thalassaemia. Cardiovascular and Hematological Agents in Medicinal Chemistry. 2012;10(1):25-30. doi: 10.2174/187152512799201172.

1292. Tallus J, Lioumis P, Hämäläinen H, Kähkönen S, Tenovuo O. Long-lasting TMS motor threshold elevation in mild traumatic brain

injury. Acta Neurol Scand. 2012;126(3):178-82. Epub 2011/11/23. doi: 10.1111/j.1600-0404.2011.01623.x. PubMed PMID: 22103909.

1293. Tarkka IM, Stokic DS. Left prefrontal cortex influences motor imagery. European Journal of Neurology. 2012;19:217-. PubMed PMID: WOS:000309359701044.

1294. Tarnawa I. EXPLORING THE ROLE OF SODIUM CHANNEL INHIBITORS IN NEUROLOGICAL AND PSYCHIATRIC DISORDERS. Drugs of the Future. 2012;37(7):499-521. doi: 10.1358/dof.2012.037.07.1811776. PubMed PMID: WOS:000307112800005.

1295. Tasset I, Medina FJ, Jimena I, Agueera E, Gascon F, Feijoo M, et al. NEUROPROTECTIVE EFFECTS OF EXTREMELY LOW-FREQUENCY ELECTROMAGNETIC FIELDS ON A HUNTINGTON'S DISEASE RAT MODEL: EFFECTS ON NEUROTROPHIC FACTORS AND NEURONAL DENSITY. Neuroscience. 2012;209:54-63. doi: 10.1016/j.neuroscience.2012.02.034. PubMed PMID: WOS:000303306600007.

1296. Thibaut A, Ledoux D, Demertzi A, Ropars C, Laureys S, Bruno MA. Improvement of consciousness after transcranial direct current stimulation-a sham-controlled double blind study. Brain Injury. 2012;26(4-5):655. doi: 10.3109/02699052.2012.660091.

1297. Trost S, Gruber O. Evidence for a Double Dissociation of Articulatory Rehearsal and Non-Articulatory Maintenance of Phonological Information in Human Verbal Working Memory. Neuropsychobiology. 2012;65(3):133-40. doi: 10.1159/000332335. PubMed PMID: WOS:000302875300004.

1298. Van Koningsbruggen MG, Peelen MV, Davies E, Rafal RD. Neural control of voluntary eye closure: A case study and an fMRI investigation of blinking and winking. Behavioural Neurology. 2012;25(2):103-9. doi: 10.3233/BEN-2011-0355.

1299. van Putten MJ. The N20 in post-anoxic coma: are you listening? Clin Neurophysiol. 2012;123(7):1460-4. Epub 2011/12/17. doi: 10.1016/j.clinph.2011.10.049. PubMed PMID: 22172767.

1300. Vanbellingen T, Bertschi M, Nyffeler T, Cazzoli D, Wiest R, Bassetti C, et al. Theta burst stimulation over left posterior parietal cortex interferes with imitation of gestures. Clinical Neurophysiology. 2012;123(10):e101-e2. doi: 10.1016/j.clinph.2012.03.052.

1301. Vesper J, Rieck S, Wille C, Wojtecki L, Kinfe T, Schu S. Chronic motor cortex stimulation (MCS) and predictive value of rtms. Pain Practice. 2012;12:181. doi: 10.1111/j.1533-2500.2011.00528.x.

1302. Viganò A, De Pasqua V, Magis D, Auvé M, Sava SL, Di Piero V, et al. Theta burst and quadripulse repetitive transcranial magnetic stimulation (rTMS) may have therapeutic potentials in migraine

prevention: A proof-of-concept study in healthy volunteers. European Journal of Neurology. 2012;19:632. doi: 10.1111/j.1468-1331.2012.03889.x.

1303. Villamar MF, Santos Portilla A, Fregni F, Zafonte R. Noninvasive brain stimulation to modulate neuroplasticity in traumatic brain injury. Neuromodulation. 2012;15(4):326-38. Epub 2012/08/14. doi: 10.1111/j.1525-1403.2012.00474.x. PubMed PMID: 22882244.

1304. Wahab RA, Choi M, Liu Y, Krauthamer V, Zderic V, Myers MR. Mechanical bioeffects of pulsed high intensity focused ultrasound on a simple neural model. Med Phys. 2012;39(7):4274-83. Epub 2012/07/27. doi: 10.1118/1.4729712. PubMed PMID: 22830761.

1305. Wang Q, Wang F, Li X, Yang Q, Li X, Xu N, et al. Electroacupuncture pretreatment attenuates cerebral ischemic injury through α7 nicotinic acetylcholine receptor-mediated inhibition of high-mobility group box 1 release in rats. J Neuroinflammation. 2012;9:24. Epub 2012/01/27. doi: 10.1186/1742-2094-9-24. PubMed PMID: 22277256; PubMed Central PMCID: PMCPMC3297509.

1306. Wilke M, Dechent P, Schmidt-Samoa C. Experimental models for spatial neglect (Studies in human and non-human primates). Neuroforum. 2012;18(1):178-89.

1307. Wong M. mTOR as a potential treatment target for epilepsy. Future Neurology. 2012;7(5):537-45. doi: 10.2217/fnl.12.45.

1308. Xiang YM, Hao CH, Wang J. [Electroacupuncture combined with acupoint injection for 40 cases of comatose due to craniocerebral trauma]. Zhongguo Zhen Jiu. 2012;32(5):475-6. Epub 2012/06/02. PubMed PMID: 22650144.

1309. Xie Y, Zhang T. Repetitive transcranial magnetic stimulation improves consciousness disturbance in stroke patients: A quantitative electroencephalography spectral power analysis. Neural Regen Res. 2012;7(31):2465-72. Epub 2012/11/05. doi: 10.3969/j.issn.1673-5374.2012.31.008. PubMed PMID: 25337097; PubMed Central PMCID: PMCPMC4200721.

1310. Xiong ZG, Xu TL. The role of ASICs in cerebral ischemia. Wiley Interdisciplinary Reviews: Membrane Transport and Signaling. 2012;1(5):655-62. doi: 10.1002/wmts.57.

1311. Xu WD, Hua XY, Zheng MX, Xu JG, Gu YD. Peripheral nerve crossing treats the spastic upper extremity caused by central neurological injury. Brain Injury. 2012;26(4-5):522. doi: 10.3109/02699052.2012.660091.

1312. Yamada SM, Yamada S, Nakaguchi H, Murakami M, Hoya K, Matsuno A, et al. A case of tumefactive multiple sclerosis. Japanese Journal of Neurosurgery. 2012;21(5):427-32.

1313. Zafonte R. Brain Injury: Refinement and Restoration-The Light Is Coming On. PM and R. 2012;4(9):639-41. doi:

10.1016/j.pmrj.2012.07.008.

1314. Zakrison TL, Pereira BM, Marttos AC, Jr., Fraga GP, Nascimento B, Jr., Rizoli S. Venous thromboembolism prophylaxis in patients with traumatic brain injury. Rev Col Bras Cir. 2012;39(6):553-7. Epub 2013/01/26. doi: 10.1590/s0100-69912012000600018. PubMed PMID: 23348655.

1315. Zelante G, Catalano A, Ricceri R, Rampello L, Rampello L. Subcortical vascular dementia: Lights and shadows. Acta Medica Mediterranea. 2012;28(1):45-52.

1316. Zeller D, Dang SY, Weise D, Rieckmann P, Toyka KV, Classen J. Excitability decreasing central motor plasticity is retained in multiple sclerosis patients. BMC Neurology. 2012;12. doi: 10.1186/1471-2377-12-92.

1317. Zhang W, Sun YH, Shi QW, Peng XH, Yang G, Li Y. [Effect of electroacupuncture on ultrastructural changes of facial nerve in rabbits with facial nerve injury]. Zhen Ci Yan Jiu. 2012;37(4):296-301. Epub 2012/11/13. PubMed PMID: 23140051.

1318. Zhou FA, Davis G. Momentary Conscious Pairing Eliminates Unconscious-Stimulus Influences on Task Selection. PLoS ONE. 2012;7(9). doi: 10.1371/journal.pone.0046320.

1319. Zittel S, Kroeger J, van der Vegt JPM, Siebner HR, Brüggemann N, Ramirez A, et al. Motor pathway excitability in

ATP13A2 mutation carriers: A transcranial magnetic stimulation study. Parkinsonism and Related Disorders. 2012;18(5):590-4. doi: 10.1016/j.parkreldis.2011.10.020.
1320. 5th International Congress on Psychopharmacology & International Symposium on Child and Adolescent Psychopharmacology. Bulletin of Clinical Psychopharmacology. 2013;23:S1.
1321. North American Brain Injury Society's 11th Annual Conference on Brain Injury, NABIS 2013. Journal of Head Trauma Rehabilitation. 2013;28(5).
1322. Abdullah JM. All the vice chancellor's neuroscientists: Unity to achieve success in solving Malaysia's diseases via upgrading clinical services and neuroscience research. Malaysian Journal of Medical Sciences. 2013;20(3):1-5.
1323. Akbar MJ, Mehmood S, Ullah S, Avery G, Sedman P, Macfie J. Permanent gastric neuromodulation for drug refractory gastroparesis, and persistent nausea and vomiting. European Surgical Research. 2013;50:63-4. doi: 10.1159/000351978.
1324. Akbar MJ, Ullah S, Mehmood S, Sedman P, MacFie J. Gastric neuromodulation for drug resistant gastroparesis, and persistent nausea and vomiting. European Surgical Research. 2013;50(2):199. doi: 10.1159/000351963.

1325. Aleksandrova EV, Zaitsev OS, Chelyapina MV, Sharova EV, Sokolova EY, Gavrilov VM, et al. Emergence from protracted unconsciousness due to severe diffuse axonal brain injury: clinical observation and literature review. Nevrologiya, neiropsikhiatriya, psikhosomatika. 2013(2):51-8. PubMed PMID: RSCI:20277676.

1326. Andrade C. Once- to twice-daily, 3-year domiciliary maintenance transcranial direct current stimulation for severe, disabling, clozapine-refractory continuous auditory hallucinations in schizophrenia. J ect. 2013;29(3):239-42. Epub 2013/02/05. doi: 10.1097/YCT.0b013e3182843866. PubMed PMID: 23377748.

1327. Andrade DC, Borges I, Bravo GL, Bolognini N, Fregni F. Therapeutic time window of noninvasive brain stimulation for pain treatment: inhibition of maladaptive plasticity with early intervention. Expert Rev Med Devices. 2013;10(3):339-52. Epub 2013/05/15. doi: 10.1586/erd.12.90. PubMed PMID: 23668706.

1328. Antal A, Chaieb L, Paulus W. Transcranial near-infrared stimulation induces neuroplastic changes in the intact human cortex. Clinical EEG and Neuroscience. 2013;44(4):E30. doi: 10.1177/1550059413507209.

1329. Arslan D, Kalkan M, Yazgan H, Ünüvar U, Şahin C. Collective circumcision performed in Sudan: Evaluation in terms of early complications and alternative practice. Urology.

2013;81(4):864-8. doi: 10.1016/j.urology.2012.11.070.

1330. Assenza G, Pellegrino G, Tombini M, Tecchio F, Tomasevic L, Di Pino G, et al. Delta waves increase after cortical plasticity induction during wakefulness. Clinical Neurophysiology. 2013;124(10):e71-e2. doi: 10.1016/j.clinph.2013.04.094.

1331. Baguley D, McFerran D, Hall D. Tinnitus. The Lancet. 2013;382(9904):1600-7. doi: 10.1016/S0140-6736(13)60142-7.

1332. Balconi M, Bortolotti A. Conscious and unconscious face recognition is improved by high-frequency rTMS on pre-motor cortex. Consciousness and Cognition. 2013;22(3):771-8. doi: 10.1016/j.concog.2013.04.013.

1333. Balzeau J, Pinier M, Berges R, Saulnier P, Benoit J-P, Eyer J. The effect of functionalizing lipid nanocapsules with NFL-TBS.40-63 peptide on their uptake by glioblastoma cells. Biomaterials. 2013;34(13):3381-9. doi: 10.1016/j.biomaterials.2013.01.068. PubMed PMID: WOS:000316770100022.

1334. Barbas AS, Rege AS, Castleberry AW, Gommer J, Ellis MJ, Brennan TV, et al. Posterior reversible encephalopathy syndrome independently associated with tacrolimus and sirolimus after multivisceral transplantation. American Journal of Transplantation. 2013;13(3):808-10. doi: 10.1111/ajt.12061.

1335. Barnes VA, Rigg JL, Williams JJ. Clinical case series:

Treatment of PTSD with transcendental meditation in active duty military personnel. Military Medicine. 2013;178(7):e836-e40. doi: 10.7205/MILMED-D-12-00426.

1336. Barrett AM, Oh-Park M, Chen P, Ifejika NL. Neurorehabilitation: Five new things. Neurology: Clinical Practice. 2013;3(6):484-92. doi: 10.1212/01.CPJ.0000437088.98407.fa.

1337. Bashir S, Al-Hussain FA, Yoo W-K. Neural plasticity and hemispatial neglect in stroke. Journal of the Pakistan Medical Association. 2013;63(10):1299-301. PubMed PMID: WOS:000330328500019.

1338. Baudic S, Attal N, Mhalla A, de Andrade DC, Perrot S, Bouhassira D. Unilateral repetitive transcranial magnetic stimulation of the motor cortex does not affect cognition in patients with fibromyalgia. Journal of Psychiatric Research. 2013;47(1):72-7. doi: 10.1016/j.jpsychires.2012.09.003. PubMed PMID: WOS:000312354400010.

1339. Baxter MG. Mediodorsal thalamus and cognition in nonhuman primates. Frontiers in Systems Neuroscience. 2013(JUL). doi: 10.3389/fnsys.2013.00038.

1340. Bezuidenhout AF, Hurter D, Maydell AT, van Niekerk F, de Figueiredo SA, Harvey J, et al. The Kimberley Hospital Rule (KHR) for urgent computed tomography of the brain in a resource-limited

environment. S Afr Med J. 2013;103(9):646-51. Epub 2013/12/05. doi: 10.7196/samj.6876. PubMed PMID: 24300685.

1341. Bhalerao SU, Geurtjens C, Thomas GR, Kitamura CR, Zhou C, Marlborough M. Understanding the neuropsychiatric consequences associated with significant traumatic brain injury. Brain Inj. 2013;27(7-8):767-74. Epub 2013/06/26. doi: 10.3109/02699052.2013.793396. PubMed PMID: 23789861.

1342. Bick AS, Leker RR, Ben-Hur T, Levin N. Implementing novel imaging methods for improved diagnosis of disorder of consciousness patients. Journal of the Neurological Sciences. 2013;334(1-2):130-8. doi: 10.1016/j.jns.2013.08.009.

1343. Blakemore RL, Hyland BI, Hammond-Tooke GD, Anson JG. Distinct Modulation of Event-Related Potentials during Motor Preparation in Patients with Motor Conversion Disorder. PLoS ONE. 2013;8(4). doi: 10.1371/journal.pone.0062539.

1344. Block CK, West SE. Psychotherapeutic treatment of survivors of traumatic brain injury: review of the literature and special considerations. Brain Inj. 2013;27(7-8):775-88. Epub 2013/05/02. doi: 10.3109/02699052.2013.775487. PubMed PMID: 23631508.

1345. Bodart O, Laureys S, Gosseries O. Coma and disorders of consciousness: scientific advances and practical considerations for clinicians. Semin Neurol. 2013;33(2):83-90. Epub 2013/07/28. doi:

10.1055/s-0033-1348965. PubMed PMID: 23888393.

1346. Bonnì S, Mastropasqua C, Bozzali M, Caltagirone C, Koch G. Theta burst stimulation improves visuo-spatial attention in a patient with traumatic brain injury. Neurol Sci. 2013;34(11):2053-6. Epub 2013/03/28. doi: 10.1007/s10072-013-1412-y. PubMed PMID: 23532550.

1347. Bosco E, Zanatta P, Baldanzi F, Sorbara C. Effects of median nerve stimulation intensity on somatosensory evoked potentials in comatose patients. Clinical Neurophysiology. 2013;124(11):e211. doi: 1016/j.clinph.2013.06.128.

1348. Bose P, Hou J, Nelson R, Nissim N, Parmer R, Keener J, et al. Effects of Acute Intrathecal Baclofen in an Animal Model of TBI-Induced Spasticity, Cognitive, and Balance Disabilities. Journal of Neurotrauma. 2013;30(13):1177-91. doi: 10.1089/neu.2012.2740. PubMed PMID: WOS:000321186400009.

1349. Brukamp K. Motor-independent communication by severely physically challenged patients. Neuroscientific research results and patient autonomy. Nervenarzt. 2013;84(10):1190-5. doi: 10.1007/s00115-013-3813-9.

1350. Brukamp K. Right (to a) diagnosis? Establishing correct diagnoses in chronic disorders of consciousness. Neuroethics. 2013;6(1):5-11. doi: 10.1007/s12152-012-9154-8.

1351. Burnstock G. Introduction and perspective, historical note. Frontiers in Cellular Neuroscience. 2013;7(NOV). doi: 10.3389/fncel.2013.00227.

1352. Campostrini S, Cavinato M, Piccione F. Transcranial direct current stimulation increases long-range fronto-parietal connections in patients with disorders of consciousness: A preliminary study. Clinical Neurophysiology. 2013;124(11):e210. doi: 10.1016/j.clinph.2013.06.124.

1353. Carson RG, Kennedy NC. Modulation of human corticospinal excitability by paired associative stimulation. Frontiers in Human Neuroscience. 2013;7. doi: 10.3389/fnhum.2013.00823. PubMed PMID: WOS:000328151600001.

1354. Carson RG, Nelson BD, Buick AR, Carroll TJ, Kennedy NC, Mac Cann R. Characterizing Changes in the Excitability of Corticospinal Projections to Proximal Muscles of the Upper Limb. Brain Stimulation. 2013;6(5):760-8. doi: 10.1016/j.brs.2013.01.016. PubMed PMID: WOS:000325046600009.

1355. Carvalho D, Teixeira S, Lucas M, Yuan TF, Chaves F, Peressutti C, et al. The mirror neuron system in post-stroke rehabilitation. International Archives of Medicine. 2013;6(1). doi: 10.1186/1755-7682-6-41.

1356. Casali AG, Gosseries O, Rosanova M, Boly M, Sarasso S,

Casali KR, et al. A theoretically based index of consciousness independent of sensory processing and behavior. Sci Transl Med. 2013;5(198):198ra05. Epub 2013/08/16. doi: 10.1126/scitranslmed.3006294. PubMed PMID: 23946194.

1357. Casali S, Renzi C, D'Angelo E, Vecchi T, Cattaneo Z. Online rTMS over cerebellar vermis affects motion discrimination in healthy individuals. Clinical Neurophysiology. 2013;124(10):e135. doi: 10.1016/j.clinph.2013.04.227.

1358. Celnik P. Using non-invasive brain stimulation to study cerebellar contributions to motor learning. Clinical Neurophysiology. 2013;124(10):e45. doi: 10.1016/j.clinph.2013.04.038.

1359. Cha M, Ji Y, Masri R. Motor Cortex Stimulation Activates the Incertothalamic Pathway in an Animal Model of Spinal Cord Injury. Journal of Pain. 2013;14(3):260-9. doi: 10.1016/j.jpain.2012.11.007. PubMed PMID: WOS:000316038200005.

1360. Chen F, Li C, Zhu Y-J, Zhao X-Y, Lu B-Q, Wu J. Magnetic nanocomposite of hydroxyapatite ultrathin nanosheets/Fe3O4 nanoparticles: microwave-assisted rapid synthesis and application in pH-responsive drug release. Biomaterials Science. 2013;1(10):1074-81. doi: 10.1039/c3bm60086f. PubMed PMID: WOS:000330137700008.

1361. Chen L, Sharma A, Guo J, Muresanu D, Huang H, Skaper S,

et al. Conference Report Annual Advances in Neurorestoratology: A Summary of IANR VI and 10th GCNN Conference, Bucharest, Romania, April 4-7, 2013. Cns & Neurological Disorders-Drug Targets. 2013;12(5):547-9. PubMed PMID: WOS:000322202100001.

1362. Chen L, Sharma A, Guo J, Muresanu D, Huang H, Skaper S, et al. Annual advances in neurorestoratology: A summary of IANR VI and 10th GCNN Conference, Bucharest, Romania, april 4-7, 2013. CNS and Neurological Disorders - Drug Targets. 2013;12(5):547-9. doi: 10.2174/18715273113120500001.

1363. Chen W, Shan CL, Guo B, Yuan T. Mirror neuron system activation for conscious states assessment: for patients in vegetative and minimally conscious states. Med Hypotheses. 2013;81(1):144-5. Epub 2013/05/07. doi: 10.1016/j.mehy.2013.04.014. PubMed PMID: 23643706.

1364. Cheng CA, Chu H, Cheng CC, Hsu CH, Lin CC, Tsai CL, et al. Modifications of autonomic activity and baroreceptor response on posture challenge in patients with vasovagal syncope. Journal of Medical Sciences (Taiwan). 2013;33(4):199-204. doi: 10.6136/JMS.2013.33(4).199.

1365. Cheyne DO, Fehlings D. Can neuroimaging help identify effective strategies for constraint therapy in congenital hemiparesis? Developmental Medicine and Child Neurology. 2013;55(10):882-3.

doi: 10.1111/dmcn.12238. PubMed PMID: WOS:000324067100006.

1366. Chi CT. Exploratory study of peripheral nerve rewiring surgery for rescuing the hemiplegic hand after central neurologic injuries by inducing brain reorganization. https://trialsearchwhoint/Trial2aspx?TrialID=ChiCTR-TRC-13004466. 2013. PubMed PMID: CN-01833292.

1367. Chiaramonti R, Giovannelli F, Bianco G, Godone M, Battista D, Cardinali C, et al. Lack of behavioural effects of high-frequency rTMS in vegetative state: a randomised, double blind, sham-controlled, crossover study. Clinical neurophysiology. 2013;124(11):e211. doi: 10.1016/j.clinph.2013.06.126. PubMed PMID: CN-01060114.

1368. Chuang CH, Hsu YC, Wang CC, Hu C, Kuo JR. Cerebral blood flow and apoptosis-associated factor with electroacupuncture in a traumatic brain injury rat model. Acupunct Med. 2013;31(4):395-403. Epub 2013/09/24. doi: 10.1136/acupmed-2013-010406. PubMed PMID: 24055977.

1369. Dargie R, Olma M, Behrens J, Kraft A, Irlbacher K, Fahle M, et al. Serial anodal tDCS over V1 induces long-term effects on colour discrimination in V4 measured in the unimpaired hemifield of patients with occipital stroke. Clinical Neurophysiology. 2013;124(10):e138. doi: 10.1016/j.clinph.2013.04.232.

1370. Davis LA, Ku BS, Griffin B, Fields JM. Prevalence of needle

phobia in patients with difficult venous access. Academic Emergency Medicine. 2013;20(5):S141. doi: 10.1111/acem.12115.

1371. Della Pepa GM, Fukaya C, La Rocca G, Zhong J, Visocchi M. Neuromodulation of vegetative state through spinal cord stimulation: where are we now and where are we going? Stereotact Funct Neurosurg. 2013;91(5):275-87. Epub 2013/06/26. doi: 10.1159/000348271. PubMed PMID: 23797266.

1372. Diaz-Ledezma C, Lichstein PM, Maltenfort M, Restrepo C, Parvizi J. Pattern of impact of femoroacetabular impingement upon healthrelated quality of life: The determinant role of extra-articular factors. Quality of Life Research. 2013;22(9):2323-30. doi: 10.1007/s11136-013-0359-z.

1373. Do KH, Yeo SS, Lee J, Jang SH. Injury of the corticoreticular pathway in patients with proximal weakness following cerebral infarct: Diffusion tensor tractography study. Neuroscience Letters. 2013;546:21-5. doi: 10.1016/j.neulet.2013.04.040.

1374. Dudakov JA, Cole D, Hanash AM, Young LF, Kreines FM, Levy ER, et al. Intrathymic Innate Lymphoid Cells: Long-Lived Mediators Of Immune Regeneration. Blood. 2013;122(21). doi: 10.1182/blood.V122.21.289.289. PubMed PMID: WOS:000331385000316.

1375. Fan B. Neuropathic pain and neuromodulation techniques.

Journal of the Neurological Sciences. 2013;333:e535. doi: 10.1016/j.jns.2013.07.1885.

1376. Fasotti L, van Kessel M. Novel insights in the rehabilitation of neglect. Frontiers in Human Neuroscience. 2013(NOV). doi: 10.3389/fnhum.2013.00780.

1377. Fink E, Tasker R, Beca J, Bell MJ, Clark RS, Hutchison J, et al. Prevalence of acute critical neurological disease in children: A global epidemiological assessment (PANGEA). Critical Care. 2013;17:S131-S2. doi: 10.1186/cc12284.

1378. Frank S, Waschke A, Reichart R, Kalff R. Transcranial magnetic stimulation in neuropathic facial pain-A prospective study of 17 patients. Clinical Neurophysiology. 2013;124(10):e122. doi: 10.1016/j.clinph.2013.04.200.

1379. Fricova J, Klirova M, Novak T, Vérebová K, Rokyta R. The advantage of 20 HZ RTMS stimulation in the treatment of orofacial pain. Neuromodulation. 2013;16(5):e169. doi: 10.1111/ner.12106.

1380. Furness S, Bryan G, McMillan R, Birchenough S, Worthington HV. Interventions for the management of dry mouth: non-pharmacological interventions. Cochrane Database Syst Rev. 2013;2013(9):Cd009603. Epub 2013/09/06. doi: 10.1002/14651858.CD009603.pub3. PubMed PMID: 24006231; PubMed Central PMCID: PMCPMC7100870 Worthington: no

interests to declare. Roddy McMillan: no interests to declare. Sarah Birchenough: no interests to declare.

1381. Ganguly K, Byl NN, Abrams GM. Neurorehabilitation: Motor Recovery After Stroke as an Example. Annals of Neurology. 2013;74(3):373-81. doi: 10.1002/ana.23994. PubMed PMID: WOS:000325463100013.

1382. Garcia G, Norise C, Faseyitan O, Naeser MA, Hamilton RH. Utilizing Repetitive Transcranial Magnetic Stimulation to Improve Language Function in Stroke Patients with Chronic Non-fluent Aphasia. Jove-Journal of Visualized Experiments. 2013(77). doi: 10.3791/50228. PubMed PMID: WOS:000209227900006.

1383. George MS, Massimini M. Using Brain Stimulation to Create Thoughts, Retrieve and Alter Memories, and Measure Consciousness - A Discussion of Recent Research. Brain Stimulation. 2013;6(6):835-6. doi: 10.1016/j.brs.2013.09.011. PubMed PMID: WOS:000327908600001.

1384. George MS, Raman R, Jain S, Benedek DM, Pelic CG, Grammer GG, et al. A Two-site Pilot Study Suggests that Three Days (9 Sessions) of High Dose Left Prefrontal Repetitive Transcranial Magnetic Stimulation (rTMS) is Feasible, Safe, and Reduces Suicidal Thinking in Suicidal Inpatients. Neuropsychopharmacology. 2013;38:S421-S2. PubMed PMID: WOS:000209477100662.

1385. Georgy M, Padwal J, Georgy B. Spinal cord stimulators in outpatient interventional neuroradiology practice. Neuroradiology. 2013;55(6):790-1. doi: 10.1007/s00234-013-1209-y.
1386. Gerardo-Nava J, Mayorenko II, Grehl T, Steinbusch HWM, Weis J, Brook GA. Differential pattern of neuroprotection in lumbar, cervical and thoracic spinal cord segments in an organotypic rat model of glutamate-induced excitotoxicity. Journal of Chemical Neuroanatomy. 2013;53:11-7. doi: 10.1016/j.jchemneu.2013.09.007. PubMed PMID: WOS:000329421900002.
1387. Giacino JT, Katz DI, Whyte J. Neurorehabilitation in Disorders of Consciousness. Seminars in Neurology. 2013;33(2):142-56. doi: 10.1055/s-0033-1348960. PubMed PMID: WOS:000322225300008.
1388. Gillebert CR, Mantini D. Functional connectivity in the normal and injured brain. Neuroscientist. 2013;19(5):509-22. Epub 2012/10/16. doi: 10.1177/1073858412463168. PubMed PMID: 23064084.
1389. Gilmore EJ, Breteler MJM, Hirsch LJ, Zaveri H, Gaspard N. Early quantitative EEG findings correlate with neurological dysfunction, hemodynamics and predict outcome in patients with severe sepsis. Neurocritical Care. 2013;19(1):S5. doi: 10.1007/s12028-013-9895-1.

1390. Golbe LI. Diagnosis and management of progressive supranuclear palsy. Neurodegenerative Disease Management. 2013;3(1):81-90. doi: 10.2217/nmt.13.1.

1391. Hartmann A, Kalis R, Matthäus L, Weber F, Rommel T. Robot guided positioning of a magnetic coil for rTMS over the cortex in patients with cerebral lesions. Clinical Neurophysiology. 2013;124(10):e173-e4. doi: 10.1016/j.clinph.2013.04.303.

1392. Hashimoto T, Taoka M, Obayashi S, Hara Y, Tanaka M, Iriki A. Modulation of cortical vestibular processing by somatosensory inputs in the posterior insula. Brain Injury. 2013;27(13-14):1685-91. doi: 10.3109/02699052.2013.831128.

1393. He J, Yang Y, Jiao H, Dang Y, Dai Y, Zhang Q, et al. Neruomodulation treatment for patients under persistent vegetative state. Chinese Journal of Neuromedicine. 2013;12(12):1197-200. PubMed PMID: CSCD:5036754.

1394. He X, Lan Y, Xu G, Mao Y, Chen Z, Huang D, et al. Frontoparietal regions may become hypoactive after intermittent theta burst stimulation over the contralateral homologous cortex in humans. Journal of Neurophysiology. 2013;110(12):2849-56. doi: 10.1152/jn.00369.2013. PubMed PMID: WOS:000328889700014.

1395. Henrich-Noack P, Lazik S, Sergeeva E, Wagner S, Voigt N, Prilloff S, et al. Transcorneal alternating current stimulation after

severe axon damage in rats results in "long-term silent survivor" neurons. Brain Res Bull. 2013;95:7-14. Epub 2013/03/19. doi: 10.1016/j.brainresbull.2013.02.008. PubMed PMID: 23500179.

1396. Hensley-Judge H, Quigg M, Barbaro NM, Newman SA, Ward MM, Chang EF, et al. Visual field defects after radiosurgery for mesial temporal lobe epilepsy. Epilepsia. 2013;54(8):1376-80. doi: 10.1111/epi.12215. PubMed PMID: CN-00963913.

1397. Héron A, Dubayle D. A focus on mast cells and pain. J Neuroimmunol. 2013;264(1-2):1-7. Epub 2013/10/16. doi: 10.1016/j.jneuroim.2013.09.018. PubMed PMID: 24125568.

1398. Hillert L, Jovanovic H, Åhs F, Savic I. Women with Multiple Chemical Sensitivity Have Increased Harm Avoidance and Reduced 5-HT1A Receptor Binding Potential in the Anterior Cingulate and Amygdala. PLoS ONE. 2013;8(1). doi: 10.1371/journal.pone.0054781.

1399. Hirabayashi H. Surgical treatment strategy for intractable pain due to the stroke: Application of neuromodulation therapy. Neuromodulation. 2013;16(5):e108. doi: 10.1111/ner.12105.

1400. Hoffmann M. The human frontal lobes and frontal network systems: An evolutionary, clinical, and treatment perspective. ISRN Neurology. 2013;1(1). doi: 10.1155/2013/892459.

1401. Höller Y, Bergmann J, Thomschewski A, Kronbichler M,

Höller P, Crone JS, et al. Comparison of EEG-features and classification methods for motor imagery in patients with disorders of consciousness. PLoS ONE. 2013;8(11). doi: 10.1371/journal.pone.0080479.

1402. Hömberg V. Neurorehabilitation approaches to facilitate motor recovery. Handb Clin Neurol. 2013;110:161-73. Epub 2013/01/15. doi: 10.1016/b978-0-444-52901-5.00014-9. PubMed PMID: 23312639.

1403. Hsu E, Cohen SP. Postamputation pain: Epidemiology, mechanisms, and treatment. Journal of Pain Research. 2013;6:121-36. doi: 10.2147/JPR.S32299.

1404. Hummel F. Non-invasive brain stimulation to support neuroregeneration of the motor system after stroke. Behavioural Neurology. 2013;27(3):325. doi: 10.3233/BEN-139900.

1405. Indovino S, Maccora S, Baschi R, Puma A, Cosentino G. Visuospatial ability in healthy subjects and in patients with left or right hemisphere damage. Acta Medica Mediterranea. 2013;29(2):209-12.

1406. Inguaggiato E, Sgandurra G, Perazza S, Guzzetta A, Cioni G. Brain Reorganization following Intervention in Children with Congenital Hemiplegia: A Systematic Review. Neural Plasticity. 2013;2013. doi: 10.1155/2013/356275. PubMed PMID: WOS:000328141300001.

1407. Irct2013042313100N. study of transcranial magnetic

stimulation effect on motor imagery rehabilitation and movement improvment in multiple sclerosis patients. https://trialsearchwhoint/Trial2aspx?TrialID=IRCT2013042313100N1. 2013. PubMed PMID: CN-01805100.

1408. Janice Lin CH, Chiang MC. Applying magnetic resonance imaging to structural and functional brain research. Journal of Neuroscience and Neuroengineering. 2013;2(1):29-37. doi: 10.1166/jnsne.2013.1032.

1409. Jetté F, Côté I, Meziane HB, Mercier C. Effect of single-session repetitive transcranial magnetic stimulation applied over the hand versus leg motor area on pain after spinal cord injury. Neurorehabilitation and neural repair. 2013;27(7):636-43. doi: 10.1177/1545968313484810. PubMed PMID: CN-00919324.

1410. Ji RR, Berta T, Nedergaard M. Glia and pain: Is chronic pain a gliopathy? Pain. 2013;154(SUPPL. 1):S10-S28. doi: 10.1016/j.pain.2013.06.022.

1411. Jprn U. Before-after study of a transcranial direct current stimulation (tDCS) effects for rehabilitative treatments in subacute brain damaged patients. https://trialsearchwhoint/Trial2aspx?TrialID=JPRN-UMIN000011444. 2013. PubMed PMID: CN-01796444.

1412. Kandel M, Beis JM, Le Ray M, Fraser A, Le Chapelain L,

Datié AM, et al. Transcranial direct current stimulation improves function for stroke patients with pure motor neglect: A case report. Annals of Physical and Rehabilitation Medicine. 2013;56:e60-e1. doi: 10.1016/j.rehab.2013.07.059.

1413. Kaplitt MG. Spinal Cord Stimulation for Vegetative State: Is This Ready for Prime Time? Stereotactic and Functional Neurosurgery. 2013;91(5):288-9. doi: 10.1159/000348272. PubMed PMID: WOS:000323778500002.

1414. Kapur N, Cole J, Manly T, Viskontas I, Ninteman A, Hasher L, et al. Positive Clinical Neuroscience: Explorations in Positive Neurology. Neuroscientist. 2013;19(4):354-69. doi: 10.1177/1073858412470976. PubMed PMID: WOS:000330518600008.

1415. Kashiwa K, Bennett M, El-Jabali F, Papastergiou G, Waite K. Transcutaneous microcurrent electrical stimulation (MCS) in the therapy of previously untreatable retinal diseases. Investigative Ophthalmology and Visual Science. 2013;54(15).

1416. Kibria G, Hatakeyama H, Ohga N, Hida K, Harashima H. The effect of liposomal size on the targeted delivery of doxorubicin to Integrin alpha v beta 3-expressing tumor endothelial cells. Biomaterials. 2013;34(22):5617-27. doi: 10.1016/j.biomaterials.2013.03.094. PubMed PMID:

WOS:000319855600018.

1417. Kim JH, Choi KH, Jang YJ, Kim HN, Bae SS, Choi BT, et al. Electroacupuncture preconditioning reduces cerebral ischemic injury via BDNF and SDF-1α in mice. BMC Complement Altern Med. 2013;13:22. Epub 2013/01/30. doi: 10.1186/1472-6882-13-22. PubMed PMID: 23356671; PubMed Central PMCID: PMCPMC3562247.

1418. Kirton A. Modeling developmental plasticity after perinatal stroke: defining central therapeutic targets in cerebral palsy. Pediatr Neurol. 2013;48(2):81-94. Epub 2013/01/23. doi: 10.1016/j.pediatrneurol.2012.08.001. PubMed PMID: 23337000.

1419. Kirton A. Can noninvasive brain stimulation measure and modulate developmental plasticity to improve function in stroke-induced cerebral palsy? Seminars in Pediatric Neurology. 2013;20(2):116-26. doi: 10.1016/j.spen.2013.06.004.

1420. Klinger E, Kadri A, Sorita E, Le Guiet JL, Coignard P, Fuchs MP, et al. Personalized rehabilitation of cognitive functions based on simulated Activities o Daily Living (sADL). Annals of Physical and Rehabilitation Medicine. 2013;56:e335. doi: 10.1016/j.rehab.2013.07.1075.

1421. Kojovic M, Pareés I, Kassavetis P, Palomar FJ, Mir P, Teo JT, et al. Secondary and primary dystonia: pathophysiological differences.

Brain. 2013;136(Pt 7):2038-49. Epub 2013/06/19. doi: 10.1093/brain/awt150. PubMed PMID: 23771342.

1422. Korfias S, Angelakis E, Stranjalis G, Sakas D. Anatomic circuits of consciousness. neuromodulation prospects for patients in Coma, PVS or MCS? Neuromodulation. 2013;16(5):e202. doi: 10.1111/ner.12106.

1423. Kozel FA, Croarkin PE, Mapes KS. A Non-Epileptiform Event in the Course of rTMS: A Case for Close Physician Monitoring. Brain Stimulation. 2013;6(6):970-2. doi: 10.1016/j.brs.2013.04.003. PubMed PMID: WOS:000327908600023.

1424. Kreuzer PM, Landgrebe M, Frank E, Langguth B. Repetitive transcranial magnetic stimulation for the treatment of chronic tinnitus after traumatic brain injury: a case study. J Head Trauma Rehabil. 2013;28(5):386-9. Epub 2012/06/13. doi: 10.1097/HTR.0b013e318254736e. PubMed PMID: 22688213.

1425. Kulkarni J, Gavrilidis E, Worsley R, Rheenen TV, Hayes E. The role of estrogen in the treatment of men with schizophrenia. International Journal of Endocrinology and Metabolism. 2013;11(3):129-36. doi: 10.5812/ijem.6615.

1426. Kumar N, Jha S. Repetitive Transcranial Magnetic Stimulation (RTMS) in organic personality and behavioral disorder, secondary to suicide attempt by hanging. Clinical Neurophysiology.

2013;124(10):e165-e6. doi: 10.1016/j.clinph.2013.04.290.

1427. Kwon HG, Choi BY, Chang CH, Kim SH, Jung YJ, Jang SH. Recovery of an injured corticospinal tract during a critical period in a patient with intracerebral hemorrhage. NeuroRehabilitation. 2013;32(1):27-32. doi: 10.3233/NRE-130820.

1428. La Rubia M, Rus A, Molina F, del Moral ML. Is fibromyalgia-related oxidative stress implicated in the decline of physical and mental health status? Clinical and Experimental Rheumatology. 2013;31(SUPPL.79):S121-S7.

1429. Lamy JC, Boakye M. BDNF Val66Met polymorphism alters spinal DC stimulation-induced plasticity in humans. Journal of Neurophysiology. 2013;110(1):109-16. doi: 10.1152/jn.00116.2013.

1430. Lapitskaya N, Gosseries O, De Pasqua V, Pedersen AR, Nielsen JF, de Noordhout AM, et al. Abnormal corticospinal excitability in patients with disorders of consciousness. Brain Stimul. 2013;6(4):590-7. Epub 2013/02/14. doi: 10.1016/j.brs.2013.01.002. PubMed PMID: 23403267.

1431. Lapitskaya N, Moerk SK, Gosseries O, Nielsen JF, de Noordhout AM. Corticospinal excitability in patients with anoxic, traumatic, and non-traumatic diffuse brain injury. Brain Stimul. 2013;6(2):130-7. Epub 2012/05/01. doi: 10.1016/j.brs.2012.03.010. PubMed PMID: 22542389.

1432. Lee D, Shahlaie K, Izadi A, Melnik M, Seidl S, Berman R, et al. Stimulation of medial septum restores hippocampal theta coherence following traumatic brain injury. Journal of Neurotrauma. 2013;30(15):A74-A5. doi: 10.1089/neu.2013.9938.

1433. Lee J, Ku J, Han K, Park J, Lee H, Kim KR, et al. rTMS over bilateral inferior parietal cortex induces decrement of spatial sustained attention. Frontiers in Human Neuroscience. 2013(FEB). doi: 10.3389/fnhum.2013.00026.

1434. Lee N, Rydyznski CE, Spearry RP, Robitz R, Maclennan AJ. The contribution of ciliary neurotrophic factor receptors to adult motor neuron survival in vivo is specific to insult type and distinct from that for embryonic motor neurons. J Comp Neurol. 2013;521(14):3217-25. Epub 2013/05/23. doi: 10.1002/cne.23341. PubMed PMID: 23695797; PubMed Central PMCID: PMCPMC3986355.

1435. Lefaucheur JP. Pain. Handb Clin Neurol. 2013;116:423-40. Epub 2013/10/12. doi: 10.1016/b978-0-444-53497-2.00035-8. PubMed PMID: 24112914.

1436. Lesniak M, Polanowska K, Seniów J. Repeated anodal tDCS coupled with cognitive training for patients with severe traumatic brain injury-a pilot RCT. Clinical Neurophysiology. 2013;124(10):e179-e80. doi: 10.1016/j.clinph.2013.04.314.

1437. Liley DTJ, Walsh M. The mesoscopic modelling of burst

suppression during anaesthesia. Frontiers in Computational Neuroscience. 2013(APR 2013). doi: 10.3389/fncom.2013.00046.

1438. Lloréns R, Colomer-Font C, Alcañiz M, Noé-Sebastián E. BioTrak virtual reality system: Effectiveness and satisfaction analysis for balance rehabilitation in patients with brain injury. Neurologia. 2013;28(5):268-75. doi: 10.1016/j.nrl.2012.04.016.

1439. Lloyd DA, Abrahamyan A, Harris JA. Brain-stimulation induced blindsight: unconscious vision or response bias? PLoS One. 2013;8(12):e82828. Epub 2013/12/11. doi: 10.1371/journal.pone.0082828. PubMed PMID: 24324837; PubMed Central PMCID: PMCPMC3855787.

1440. Louppe JM, Nguyen JP, Robert R, Buffenoir K, de Chauvigny E, Riant T, et al. Motor cortex stimulation in refractory pelvic and perineal pain: report of two successful cases. Neurourol Urodyn. 2013;32(1):53-7. Epub 2012/06/08. doi: 10.1002/nau.22269. PubMed PMID: 22674567.

1441. Machado A. New frontier: The brain machine interface. Neuromodulation. 2013;16(1):6-7. doi: 10.1111/ner.12021.

1442. Mall V, Jung NH. Motor cortex: Anatomy, Functions and injury. Brain Research Journal. 2013;6(2):209-20.

1443. Manganotti P, Del Felice A. New perspectives in transcranial magnetic stimulation: Epilepsy, consciousness and the perturbational

approach. Behavioural Neurology. 2013;27(2):155-67. doi: 10.3233/BEN-2012-120263.

1444. Manganotti P, Formaggio E, Storti SF, Fiaschi A, Battistin L, Tonin P, et al. Effect of high-frequency repetitive transcranial magnetic stimulation on brain excitability in severely brain-injured patients in minimally conscious or vegetative state. Brain Stimul. 2013;6(6):913-21. Epub 2013/08/10. doi: 10.1016/j.brs.2013.06.006. PubMed PMID: 23928101.

1445. Mańko G, Olszewski H, Krawczyński M, Tłokiński W. Evaluation of differentiated neurotherapy programs for patients recovering from severe TBI and long term coma. Acta Neuropsychologica. 2013;11(1):9-18.

1446. Marron EM, Redolar-Ripoll D. Transcranial magnetic stimulation: A new rehabilitation tool for neglect. Behavioural Neurology. 2013;27(3):394. doi: 10.3233/BEN-139900.

1447. Mateer CA. Reconceptualizing brain injury rehabilitation in the future: A peek over the horizon. Journal of Head Trauma Rehabilitation. 2013;28(3):232-6. doi: 10.1097/HTR.0b013e318291b046.

1448. Megha M, Harpreet S, Nayeem Z. Effect of frequency of multimodal coma stimulation on the consciousness levels of traumatic brain injury comatose patients. Brain Inj. 2013;27(5):570-7. Epub

2013/03/12. doi: 10.3109/02699052.2013.767937. PubMed PMID: 23473238.

1449. Meier K, Flink M, Simonsen R, Sørensen JC. The aarhus neuromodulation database. Neuromodulation. 2013;16(5):e211. doi: 10.1111/ner.12106.

1450. Michou E, Mistry S, Rothwell J, Hamdy S. Priming pharyngeal motor cortex by repeated paired associative stimulation: Implications for dysphagia neurorehabilitation. Neurorehabilitation and Neural Repair. 2013;27(4):355-62. doi: 10.1177/1545968312469837.

1451. Midaglia L, Fraga A, Rodriguez Acevedo B, Lion S, Ribeiro S, Dieguez C, et al. Repetitive transcranial magnetic stimulation in refractory spasticity treatment in patients with multiple sclerosis. Multiple sclerosis (Houndmills, Basingstoke, England). 2013;19(11 SUPPL. 1):279-80. doi: 10.1177/1352458513502429. PubMed PMID: CN-01058244.

1452. Miller J, Eakin K, Sweet J. Theta Burst Stimulation Of The Hippocampus Via An Electrode Implanted In The Fornix Is Associated With Improved Cognitive Performance After Moderate Traumatic Brain Injury. Stereotactic and Functional Neurosurgery. 2013;91:78-. PubMed PMID: WOS:000209547400077.

1453. Mueske N, Ryan D, Van Speybroeck A, Wren T. Fat

distribution in children and adolescents with myelomeningocele. Developmental Medicine and Child Neurology. 2013;55:28. doi: 10.1111/dmcn.12247.

1454. Müller-Dahlhaus F. Induction and increase of cortico-cortical reorganisation post stroke. Klinische Neurophysiologie. 2013;44(4):257-62. doi: 10.1055/s-0033-1357213.

1455. Müri RM, Cazzoli D, Nef T, Mosimann UP, Nyffeler SHT. Non-invasive brain stimulation in neglect rehabilitation: An update. Frontiers in Human Neuroscience. 2013(MAY). doi: 10.3389/fnhum.2013.00248.

1456. Muscari A, Puddu GM, Serafini C, Fabbri E, Vizioli L, Zoli M. Predictors of short-term improvement of ischemic stroke. Neurological Research. 2013;35(6):594-601. doi: 10.1179/1743132813Y.0000000181.

1457. Nct. tDCS in MCS: Repeated Stimulations. https://clinicaltrialsgov/show/NCT02019615. 2013. PubMed PMID: CN-01479956.

1458. Nct. Amantadine + rTMS as a Neurotherapeutic for Disordered Consciousness. https://clinicaltrialsgov/show/NCT02025439. 2013. PubMed PMID: CN-01480121.

1459. Nct. Improving Functions in MTBI Patients With Headache

by rTMS. https://clinicaltrialsgov/show/NCT01948947. 2013. PubMed PMID: CN-01590508.

1460. Nct. Transcranial Brain Stimulation in Vegetative State Patients. https://clinicaltrialsgov/show/NCT01820923. 2013. PubMed PMID: CN-01541309.

1461. Nct. Effects of Repetitive Transcranial Magnetic Stimulation in the Treatment of Phantom Limb Pain in Landmine Victims: ANTARES. https://clinicaltrialsgov/show/NCT01872481. 2013. PubMed PMID: CN-01542664.

1462. Nct. Improving Motor Function After Spinal Cord Injury. https://clinicaltrialsgov/show/NCT01915095. 2013. PubMed PMID: CN-01579207.

1463. Nct. Skilled Motor Training and tDCS to Improve Leg Function After Spinal Cord Injury. https://clinicaltrialsgov/show/NCT01962675. 2013. PubMed PMID: CN-01536776.

1464. Nct. Antispastic Effect of Transcranial Magnetic Stimulation in Patients With Cerebral and Spinal Spasticity. https://clinicaltrialsgov/show/NCT01786005. 2013. PubMed PMID: CN-01579005.

1465. Ng YS, Chew E, Samuel GS, Tan YL, Kong KH. Advances in rehabilitation medicine. Singapore Medical Journal.

2013;54(10):538-51. PubMed PMID: WOS:000327245300008.

1466. Nguyen J, Brown J, Mold JW, Welborn TL. In patients with concussions, is the probability of permanent neurological damage predicted better by total number of concussions than by severity and duration of individual concussions? J Okla State Med Assoc. 2013;106(11):431-2. Epub 2014/03/07. PubMed PMID: 24596983; PubMed Central PMCID: PMCPMC4930876.

1467. Nitsche MA. Timing-dependent effects of brain stimulation on motor learning. Clinical Neurophysiology. 2013;124(10):e43. doi: 10.1016/j.clinph.2013.04.030.

1468. Nudo RJ. Recovery after brain injury: Mechanisms and principles. Frontiers in Human Neuroscience. 2013;7(DEC). doi: 10.3389/fnhum.2013.00887.

1469. Ogura M, Nishibayashi H, Kakishita K, Nakao N. Motor cortex stimulation for freezing of gait. Stereotactic and Functional Neurosurgery. 2013;91:204. doi: 10.1159/000351990.

1470. Olson LD, Perry MS. LOCALIZATION OF EPILEPTIC FOCI USING MULTIMODALITY NEUROIMAGING. International Journal of Neural Systems. 2013;23(1). doi: 10.1142/s012906571230001x. PubMed PMID: WOS:000316807400002.

1471. Ommaya AK, Adams KM, Allman RM, Collins EG, Cooper

RA, Dixon CE, et al. Opportunities in rehabilitation research. Journal of Rehabilitation Research and Development. 2013;50(6):VII-XXXII. doi: 10.1682/jrrd.2012.09.0167. PubMed PMID: WOS:000324350200001.

1472. Opisso E, Borau A, Rijkhoff NJM. Subject-Controlled Stimulation of Dorsal Genital Nerve to Treat Neurogenic Detrusor Overactivity at Home. Neurourology and Urodynamics. 2013;32(7):1004-9. doi: 10.1002/nau.22359. PubMed PMID: WOS:000323169800013.

1473. Othmer S, Othmer SF, Kaiser DA, Putman J. Endogenous neuromodulation at infralow frequencies. Seminars in Pediatric Neurology. 2013;20(4):246-57. doi: 10.1016/j.spen.2013.10.006.

1474. Pandi-Perumal SR, BaHammam AS, Brown GM, Spence DW, Bharti VK, Kaur C, et al. Melatonin antioxidative defense: therapeutical implications for aging and neurodegenerative processes. Neurotox Res. 2013;23(3):267-300. Epub 2012/06/29. doi: 10.1007/s12640-012-9337-4. PubMed PMID: 22739839.

1475. Papuć E, Rejdak K. The role of neurostimulation in the treatment of neuropathic pain. Ann Agric Environ Med. 2013;Spec no. 1:14-7. Epub 2014/07/09. PubMed PMID: 25000835.

1476. Pernet L, Juchters A, Kerckhofs E. The effectiveness of conservative treatment modalities in right hemisphere stroke patients

with hemineglect, measured by neuropsychological and/or functional tests: A systematic review. Cerebrovascular Diseases. 2013;35:823. doi: 10.1159/000353129.

1477. Perreault M-C, Glover JC. Glutamatergic reticulospinal neurons in the mouse: developmental origins, axon projections, and functional connectivity. In: ZiskindConhaim L, MacDermott AB, Houle JD, Alvarez FJ, Hochman S, editors. Neurons, Circuitry, and Plasticity in the Spinal Cord and Brainstem. Annals of the New York Academy of Sciences. 12792013. p. 80-9.

1478. Peters KM, Killinger KA, Gilleran J, Boura JA. Does patient age impact outcomes of neuromodulation? Neurourol Urodyn. 2013;32(1):30-6. Epub 2012/06/08. doi: 10.1002/nau.22268. PubMed PMID: 22674536.

1479. Petrosyan V, Ameerally P. Third molar surgery: Changes in patient demographics over 18 years and the effect of the nice guidelines. International Journal of Oral and Maxillofacial Surgery. 2013;42(10):1241. doi: 10.1016/j.ijom.2013.07.243.

1480. Petzold A, Girbes A. Pain Management in Neurocritical Care. Neurocritical Care. 2013;19(2):232-56. doi: 10.1007/s12028-013-9851-0. PubMed PMID: WOS:000324644800016.

1481. Picht T, Krieg SM, Sollmann N, Rösler J, Niraula B,

Neuvonen T, et al. A comparison of language mapping by preoperative navigated transcranial magnetic stimulation and direct cortical stimulation during awake surgery. Neurosurgery. 2013;72(5):808-19. doi: 10.1227/NEU.0b013e3182889e01.

1482. Pinchuk D, Pinchuk O, Sirbiladze K, Shugar O. Clinical effectiveness of primary and secondary headache treatment by transcranial direct current stimulation. Front Neurol. 2013;4:25. Epub 2013/03/23. doi: 10.3389/fneur.2013.00025. PubMed PMID: 23519166; PubMed Central PMCID: PMCPMC3604631.

1483. Pistoia F, Sacco S, Carolei A, Sarà M. Corticomotor facilitation in vegetative state: results of a pilot study. Arch Phys Med Rehabil. 2013;94(8):1599-606. Epub 2013/02/07. doi: 10.1016/j.apmr.2013.01.019. PubMed PMID: 23385107.

1484. Pitzalis S, Spinelli D, Vallar G, Di Russo F. Transcutaneous electrical nerve stimulation effects on neglect: A visual-evoked potential study. Frontiers in Human Neuroscience. 2013(MAR). doi: 10.3389/fnhum.2013.00111.

1485. Porcher NJ, Solecki TJ. A narrative review of sports-related concussion and return-to-play testing with asymptomatic athletes. Journal of chiropractic medicine. 2013;12(4):260-8. doi: 10.1016/j.jcm.2013.08.002. PubMed PMID: MEDLINE:24396328.

1486. Pradhan A, McGuire B, Charles A. Characterization of a novel

model for chronic migraine. Journal of Headache and Pain. 2013;14(1).

1487. Qiu D, An X. Controllable release from magnetoliposomes by magnetic stimulation and thermal stimulation. Colloids and Surfaces B-Biointerfaces. 2013;104:326-9. doi: 10.1016/j.colsurfb.2012.11.033. PubMed PMID: WOS:000316645100047.

1488. Ragazzoni A, Pirulli C, Veniero D, Feurra M, Cincotta M, Giovannelli F, et al. Vegetative versus minimally conscious states: a study using TMS-EEG, sensory and event-related potentials. PLoS One. 2013;8(2):e57069. Epub 2013/03/06. doi: 10.1371/journal.pone.0057069. PubMed PMID: 23460826; PubMed Central PMCID: PMCPMC3584112.

1489. Rajapakse T, Kirton A. Non-invasive brain stimulation in children: Applications and future directions. Translational Neuroscience. 2013;4(2):217-33. doi: 10.2478/s13380-013-0116-3.

1490. Rajkowska G, Stockmeier CA. Astrocyte pathology in major depressive disorder: Insights from human postmortem brain tissue. Current Drug Targets. 2013;14(11):1225-36. doi: 10.2174/13894501113149990156.

1491. Reyns N, Derambure P, Duhamel A, Bourriez JL, Blond S, Houdayer E. Motor cortex stimulation modulates defective central beta rhythms in patients with neuropathic pain. Clin Neurophysiol.

2013;124(4):761-9. Epub 2012/11/16. doi: 10.1016/j.clinph.2012.10.011. PubMed PMID: 23151426.
1492. Richter L, Trillenberg P, Schweikard A, Schlaefer A. Stimulus intensity for hand held and robotic transcranial magnetic stimulation. Brain Stimulation. 2013;6(3):315-21. doi: 10.1016/j.brs.2012.06.002.
1493. Rosanova M. TMS-EEG to measure excitability and connectivity of human cortical circuits. Clinical Neurophysiology. 2013;124(10):e47. doi: 10.1016/j.clinph.2013.04.043.
1494. Rushmore RJ, DeSimone C, Valero-Cabré A. Multiple sessions of transcranial direct current stimulation to the intact hemisphere improves visual function after unilateral ablation of visual cortex. Eur J Neurosci. 2013;38(12):3799-807. Epub 2013/10/15. doi: 10.1111/ejn.12373. PubMed PMID: 24118563; PubMed Central PMCID: PMCPMC3934645.
1495. Rutiku R, Einberg A, Imanaka K, Bachmann T. The effect of task-irrelevant visual backgrounds on human transcranial magnetic stimulation-evoked electroencephalography responses and cortical alpha activity. Eur J Neurosci. 2013;38(12):3768-77. Epub 2013/10/15. doi: 10.1111/ejn.12374. PubMed PMID: 24118584.
1496. Saitoh Y, Maruo T, Yokoe M, Matsuzaki T, Sekino M. Electrical or repetitive transcranial magnetic stimulation of primary motor cortex for intractable neuropathic pain. Annu Int Conf IEEE

Eng Med Biol Soc. 2013;2013:6163-6. Epub 2013/10/11. doi: 10.1109/embc.2013.6610960. PubMed PMID: 24111147.
1497. Sarikaya-Seiwert S, Beez T, Stoffels G, Janßen G, Steiger HJ, Hänggi D. O-(2-[18F]fluorethyl)-L-tyrosine PET in the predictive clinical evaluation of primary pediatric brain tumors. Child's Nervous System. 2013;29(9):1736. doi: 10.1007/s00381-013-2261-y.
1498. Saroka KS, Persinger MA. Potential production of Hughlings Jackson's "parasitic consciousness" by physiologically-patterned weak transcerebral magnetic fields: QEEG and source localization. Epilepsy and Behavior. 2013;28(3):395-407. doi: 10.1016/j.yebeh.2013.05.023.
1499. Sawicki M, Bohatyrewicz R, Safranow K, Walecka A, Walecki J, Rowinski O, et al. Dynamic evaluation of stasis filling phenomenon with computed tomography in diagnosis of brain death. Neuroradiology. 2013;55(9):1061-9. Epub 2013/06/04. doi: 10.1007/s00234-013-1210-5. PubMed PMID: 23728070; PubMed Central PMCID: PMCPMC3761089.
1500. Schif ND. Making Waves in Consciousness Research. Science Translational Medicine. 2013;5(198). doi: 10.1126/scitranslmed.3007004. PubMed PMID: WOS:000323132200009.
1501. Sczesny-Kaiser M, Höffken O, Tegenthoff M, Schwenkreis P. Convulsive syncope after single-pulse TMS. Brain Stimul.

2013;6(5):830. Epub 2013/03/07. doi: 10.1016/j.brs.2013.01.018. PubMed PMID: 23462345.

1502. Seregni F, Fecchio M, Rosanova M, Sarasso S, Pigorini A, Napolitani M, et al. Cortical bistability in pathological loss of consciousness after brain injury: A TMS/EEG study. Clinical Neurophysiology. 2013;124(11):e211. doi: 10.1016/j.clinph.2013.06.127.

1503. Serpero LD, Bellissima V, Colivicchi M, Sabatini M, Frigiola A, Ricotti A, et al. Next generation biomarkers for brain injury. Journal of Maternal-Fetal & Neonatal Medicine. 2013;26:44-9. doi: 10.3109/14767058.2013.829688. PubMed PMID: WOS:000324833900013.

1504. Shabalov VA, Isagulyan E, Khitj MA. Motor cortex stimulation for the treatment of severe pain. Stereotactic and Functional Neurosurgery. 2013;91:111. doi: 10.1159/000351783.

1505. Shaqiri A, Anderson B. Priming and statistical learning in right brain damaged patients. Neuropsychologia. 2013;51(13):2526-33. doi: 10.1016/j.neuropsychologia.2013.09.024.

1506. Sharova E, Kopachka, Oknina LB, Chelyapina, Gavrilov VM, Romanov. Changes of mental activity and acoustic evoked potentials (AEP) under the influence of repetitive transcranial magnetic stimulation of premotor cortex on normal subjects and patients after

severe head injury. Clinical Neurophysiology. 2013;124(10):e181-e2. doi: 10.1016/j.clinph.2013.04.319.

1507. Shear DA, Tortella FC. A military-centered approach to neuroprotection for traumatic brain injury. Front Neurol. 2013;4:73. Epub 2013/06/20. doi: 10.3389/fneur.2013.00073. PubMed PMID: 23781213; PubMed Central PMCID: PMCPMC3679469.

1508. Shetty AK. Prospects of levetiracetam as a neuroprotective drug against status epilepticus, traumatic brain injury, and stroke. Frontiers in Neurology. 2013;4 NOV. doi: 10.3389/fneur.2013.00172.

1509. Shkirkova K, Belkin A, Lescovetc V. Functional plasticity of the motor cortical structures demonstrated by Navigated Brain Stimulation in patients with stroke and brain damage. Cerebrovascular Diseases. 2013;35:784. doi: 10.1159/000353129.

1510. Skripka-Serry J. The great neuro-pipeline 'brain drain' (and why Big Pharma hasn't given up on CNS disorders). Drug Discovery World. 2013;14(4):9-16. PubMed Central PMCID: PMCHoffmann La Roche

Merck.

1511. Sohtaoglu Sevindik M, Erdemir Kiziltan M, Bozluolcay M. Time related changes in electrophysiologic findings of two patients with pons infarctions: Auditory startle reflex and transcranial magnetic stimulation parameters. Journal of the Neurological Sciences.

2013;333:e640-e1. doi: 10.1016/j.jns.2013.07.2225.
1512. Song J-J, Punte AK, De Ridder D, Vanneste S, Van de Heyning P. Neural substrates predicting improvement of tinnitus after cochlear implantation in patients with single-sided deafness. Hearing Research. 2013;299:1-9. doi: 10.1016/j.heares.2013.02.001. PubMed PMID: WOS:000319483700001.
1513. Starr SA, Baysinger CL. Inhaled Nitrous Oxide for Labor Analgesia. Anesthesiology Clinics. 2013;31(3):623-34. doi: 10.1016/j.anclin.2013.04.001.
1514. Stefani A, Peppe A, Galati S, Bassi SM, D'Angelo V, Pierantozzi M. The serendipity case of the pedunculopontine nucleus low-frequency brain stimulation: Chasing a gait response, finding sleep, and cognition improvement. Frontiers in Neurology. 2013;4 JUN. doi: 10.3389/fneur.2013.00068.
1515. Steinberg H. A pioneer work on electric brain stimulation in psychotic patients. Rudolph Gottfried Arndt and his 1870s studies. Brain Stimul. 2013;6(4):477-81. Epub 2012/12/26. doi: 10.1016/j.brs.2012.11.004. PubMed PMID: 23266132.
1516. Sung KW, Son MH, Lee SH, Yoo KH, Koo HH, Kim JY, et al. Tandem high-dose chemotherapy and autologous stem cell transplantation in patients with high-risk neuroblastoma: Results of SMC NB-2004 study. Bone Marrow Transplantation.

2013;48(1):68-73. doi: 10.1038/bmt.2012.86.

1517. Sweet JA, Miller JP. Cognitive deficits after traumatic brain injury are associated with decreased hippocampal bursting and improved with burst stimulation of the fornix. Clinical Neurosurgery. 2013;60:166.

1518. Tallus J, Lioumis P, Hämäläinen H, Kähkönen S, Tenovuo O. Transcranial magnetic stimulation-electroencephalography responses in recovered and symptomatic mild traumatic brain injury. J Neurotrauma. 2013;30(14):1270-7. Epub 2013/02/07. doi: 10.1089/neu.2012.2760. PubMed PMID: 23384582; PubMed Central PMCID: PMCPMC3713436.

1519. Tan T, Xie J, Liu T, Chen X, Zheng X, Tong Z, et al. Low-frequency (1 Hz) repetitive transcranial magnetic stimulation (rTMS) reverses Aβ(1-42)-mediated memory deficits in rats. Exp Gerontol. 2013;48(8):786-94. Epub 2013/05/15. doi: 10.1016/j.exger.2013.05.001. PubMed PMID: 23665072.

1520. Tan T, Xie J, Liu T, Chen X, Zheng X, Tong Z, et al. Low-frequency (1Hz) repetitive transcranial magnetic stimulation (rTMS) reverses A beta(1-42)-mediated memory deficits in rats. Experimental Gerontology. 2013;48(8):786-94. doi: 10.1016/j.exger.2013.05.001. PubMed PMID: WOS:000321700300013.

1521. Tanaka Y, Matsuwaki T, Yamanouchi K, Nishihara M. Increased lysosomal biogenesis in activated microglia and exacerbated neuronal damage after traumatic brain injury in progranulin-deficient mice. Neuroscience. 2013;250:8-19. Epub 2013/07/09. doi: 10.1016/j.neuroscience.2013.06.049. PubMed PMID: 23830905.
1522. Tarapore PE, Findlay AM, Honma SM, Mizuiri D, Houde JF, Berger MS, et al. Language mapping with navigated repetitive TMS: Proof of technique and validation. Neuroimage. 2013;82:260-72. doi: 10.1016/j.neuroimage.2013.05.018. PubMed PMID: WOS:000324568400027.
1523. Tomasino B, Rumiati RI. Introducing the special topic "The when and why of sensorimotor processes in conceptual knowledge and abstract concepts". Frontiers in Human Neuroscience. 2013(AUG). doi: 10.3389/fnhum.2013.00498.
1524. Tran MT, Skovbjerg S, Arendt-Nielsen L, Christensen KB, Elberling J. Transcranial pulsed electromagnetic fields for multiple chemical sensitivity: study protocol for a randomized, double-blind, placebo-controlled trial. Trials. 2013;14:256. Epub 2013/08/21. doi: 10.1186/1745-6215-14-256. PubMed PMID: 23947742; PubMed Central PMCID: PMCPMC3765111.
1525. Tremblay S, Beaule V, Proulx S, de Beaumont L, Marjanska M, Doyon J, et al. Relationship between transcranial magnetic

stimulation measures of intracortical inhibition and spectroscopy measures of GABA and glutamate plus glutamine. Journal of Neurophysiology. 2013;109(5):1343-9. doi: 10.1152/jn.00704.2012. PubMed PMID: WOS:000315681100012.

1526. Tsaousides T, Ashman TA, Gordon WA. Diagnosis and treatment of depression following traumatic brain injury. Brain Impairment. 2013;14(1):63-76. doi: 10.1017/BrImp.2013.8.

1527. Tyler ME, Danilov YP, Kaczmarek K. CRANIAL NERVE NON-INVASIVE NEUROMODULATION FOR SYMPTOMATIC TREATMENT OF MILD AND MODERATE TRAUMATIC BRAIN INJURY. Journal of Neurotrauma. 2013;30(15):A51-A2. PubMed PMID: WOS:000322446800095.

1528. Van der Aa NE, Verhage CH, Groenendaal F, Vermeulen RJ, De Bode S, Van Nieuwenhuizen O, et al. Neonatal neuroimaging predicts recruitment of contralesional corticospinal tracts following perinatal brain injury. Developmental Medicine and Child Neurology. 2013;55(8):707-12. doi: 10.1111/dmcn.12160.

1529. Van Vleet TM, DeGutis JM. The Nonspatial Side of Spatial Neglect and Related Approaches to Treatment. In: Merzenich MM, Nahum M, VanVleet TM, editors. Changing Brains Applying Brain Plasticity to Advance and Recover Human Ability. Progress in Brain Research. 2072013. p. 327-49.

1530. Vandenberghe R, Gillebert CR. Dissociations between spatial-attentional processes within parietal cortex: Insights from hybrid spatial cueing and change detection paradigms. Frontiers in Human Neuroscience. 2013(JUN). doi: 10.3389/fnhum.2013.00366.

1531. Varelas PN, Spanaki MV, Mirski MA. Status epilepticus: An update. Current Neurology and Neuroscience Reports. 2013;13(7). doi: 10.1007/s11910-013-0357-0.

1532. Velazquez R, Ash JA, Powers BE, Kelley CM, Strawderman M, Luscher ZI, et al. Maternal choline supplementation improves spatial learning and adult hippocampal neurogenesis in the Ts65Dn mouse model of Down syndrome. Neurobiol Dis. 2013;58:92-101. Epub 2013/05/07. doi: 10.1016/j.nbd.2013.04.016. PubMed PMID: 23643842; PubMed Central PMCID: PMCPMC4029409.

1533. Wang P-C, Paik S-J, Chen S, Rajaraman S, Kim S-H, Allen MG. Fabrication and Characterization of Polymer Hollow Microneedle Array Using UV Lithography Into Micromolds. Journal of Microelectromechanical Systems. 2013;22(5):1041-53. doi: 10.1109/jmems.2013.2262587. PubMed PMID: WOS:000325410300011.

1534. White NM, Packard MG, McDonald RJ. Dissociation of memory systems: The story unfolds. Behavioral Neuroscience. 2013;127(6):813-34. doi: 10.1037/a0034859.

1535. Wong V, Cheuk DK, Lee S, Chu V. Acupuncture for acute management and rehabilitation of traumatic brain injury. Cochrane Database Syst Rev. 2013(3):Cd007700. Epub 2013/04/02. doi: 10.1002/14651858.CD007700.pub3. PubMed PMID: 23543554.

1536. Xu G, Lan Y, Zhao J, Huang D, Ding M, He X. Disruption of front oparietal network by low frequency repetitive transcranial magnetic stimulation. Journal of Head Trauma Rehabilitation. 2013;28(5):E32. doi: 10.1097/HTR.0b013e3182a2e849.

1537. Yeo SS, Jang SH. Motor recovery via aberrant pyramidal tract in a patient with traumatic brain injury: A diffusion tensor tractography study. Neural Regen Res. 2013;8(1):90-4. Epub 2013/01/05. doi: 10.3969/j.issn.1673-5374.2013.01.012. PubMed PMID: 25206377; PubMed Central PMCID: PMCPMC4107495.

1538. Yeo SS, Jang SH. Corticospinal tract recovery in a patient with traumatic transtentorial herniation. Neural Regen Res. 2013;8(5):469-73. Epub 2013/02/15. doi: 10.3969/j.issn.1673-5374.2013.05.011. PubMed PMID: 25206689; PubMed Central PMCID: PMCPMC4146130.

1539. Yeo SS, Kim SH, Jang SH. Proximal weakness due to injury of the corticoreticular pathway in a patient with traumatic brain injury. NeuroRehabilitation. 2013;32(3):665-9. Epub 2013/05/08. doi: 10.3233/nre-130889. PubMed PMID: 23648621.

1540. Zhang YM, Chen AL, Tang CZ, Zhang YQ, Yin HB, Chen SX. [Clinical observation on electroacupuncture for arousing consciousness of comatose patients with severe trauma brain injury]. Zhen Ci Yan Jiu. 2013;38(2):158-62. Epub 2013/07/04. PubMed PMID: 23819221.

1541. 윤상희, 윤서영, 이진석. Ultrasound-Aided Monolayer Assembly of Spherical Silica Nanobeads. Applied Science and Convergence Technology. 2013;22(6):298-305. PubMed PMID: KJD:ART001820151.

1542. 30th International Congress of Clinical Neurophysiology of the International Federation of Clinical Neurophysiology, Berlin, Germany, March 20-23, 2014 Abstracts. Clinical Neurophysiology. 2014;125:S1-S339. PubMed PMID: WOS:000361971100001.

1543. 2014 American Neuropsychiatric Association Annual Meeting Abstracts, ANPA 2014. Journal of Neuropsychiatry and Clinical Neurosciences. 2014;26(2).

1544. Alampay MM, Haigney MC, Flanagan MC, Perito RM, Love KM, Grammer GG. Transcranial magnetic stimulation as an antidepressant alternative in a patient with Brugada syndrome and recurrent syncope. Mayo Clin Proc. 2014;89(11):1584-7. Epub 2014/12/03. doi: 10.1016/j.mayocp.2014.08.010. PubMed PMID: 25444490.

1545. Alhelal MA, Palaska I, Panagiotidou S, Letourneau R, Theoharides TC. Trigeminal nerve stimulation triggers oral mast cell activation and vascular permeability. Ann Allergy Asthma Immunol. 2014;112(1):40-5. Epub 2013/12/18. doi: 10.1016/j.anai.2013.10.011. PubMed PMID: 24331392.

1546. Allen CPG, Dunkley BT, Muthukumaraswamy SD, Edden R, Evans CJ, Sumner P, et al. Enhanced Awareness Followed Reversible Inhibition of Human Visual Cortex: A Combined TMS, MRS and MEG Study. Plos One. 2014;9(6). doi: 10.1371/journal.pone.0100350. PubMed PMID: WOS:000338917900042.

1547. Amico E, Gomez F, Di Perri C, Vanhaudenhuyse A, Lesenfants D, Boveroux P, et al. Posterior cingulate cortex-related co-activation patterns: A resting state fMRI study in propofol-induced loss of consciousness. PLoS ONE. 2014;9(6). doi: 10.1371/journal.pone.0100012.

1548. Ammann C, Gruart A, Delgado-García JM, Márquez-Ruíz J. Testing thermal epidural effects during transcranial direct current stimulation in alert rabbits. Acta Physiologica. 2014;212:89-90. doi: 10.1111/apha.12388.

1549. André-Obadia N, Mertens P, Lelekov-Boissard T, Afif A, Magnin M, Garcia-Larrea L. Is Life better after motor cortex stimulation for pain control? Results at long-term and their prediction

by preoperative rTMS. Pain Physician. 2014;17(1):53-62. Epub 2014/01/24. PubMed PMID: 24452645.

1550. Angelakis E, Liouta E, Andreadis N, Korfias S, Ktonas P, Stranjalis G, et al. Transcranial direct current stimulation effects in disorders of consciousness. Arch Phys Med Rehabil. 2014;95(2):283-9. Epub 2013/09/17. doi: 10.1016/j.apmr.2013.09.002. PubMed PMID: 24035769.

1551. Auld M, Russo R, Moseley GL, Johnston L. Tactile interventions for children with cerebral palsy: A systematic review. Developmental Medicine and Child Neurology. 2014;56:22. doi: 10.1111/dmcn.12368.

1552. Babiloni C, Vecchio F, Buffo P, Iacoboni M, Pistoia F, Sacco S, et al. Mechanisms of Cortical Neural Synchronization Related to Healthy and Impaired Consciousness: Evidence by Quantitative Electroencephalographic Studies. Current Pharmaceutical Design. 2014;20(26):4225-38. PubMed PMID: WOS:000338508000009.

1553. Bachtiar V, Stagg CJ. THE ROLE OF INHIBITION IN HUMAN MOTOR CORTICAL PLASTICITY. Neuroscience. 2014;278:93-104. doi: 10.1016/j.neuroscience.2014.07.059. PubMed PMID: WOS:000342058700010.

1554. Barlow KM, Brooks BL, MacMaster FP, Kirton A, Seeger T, Esser M, et al. A double-blind, placebo-controlled intervention trial of

3 and 10 mg sublingual melatonin for post-concussion syndrome in youths (PLAYGAME): study protocol for a randomized controlled trial. Trials. 2014;15:271. Epub 2014/07/09. doi: 10.1186/1745-6215-15-271. PubMed PMID: 25001947; PubMed Central PMCID: PMCPMC4227124.

1555. Barry MD, Boddington LJ, Igelström KM, Gray JP, Shemmell J, Tseng KY, et al. Utility of intracerebral theta burst electrical stimulation to attenuate interhemispheric inhibition and to promote motor recovery after cortical injury in an animal model. Exp Neurol. 2014;261:258-66. Epub 2014/06/07. doi: 10.1016/j.expneurol.2014.05.023. PubMed PMID: 24905955.

1556. Bate S, Bennetts RJ. The rehabilitation of face recognition impairments: a critical review and future directions. Frontiers in Human Neuroscience. 2014;8. doi: 10.3389/fnhum.2014.00491. PubMed PMID: WOS:000340564400001.

1557. Bécsi B, Kiss A, Erdodi F. Interaction of protein phosphatase inhibitors with membrane lipids assessed by surface plasmon resonance based binding technique. Chemistry and Physics of Lipids. 2014;183:68-76. doi: 10.1016/j.chemphyslip.2014.05.009.

1558. Birbaumer N. Neural Signatures of Modified Memories. Neuron. 2014;81(1):3-5. doi: 10.1016/j.neuron.2013.12.019. PubMed PMID: WOS:000329559000002.

1559. Bolton DAE, Staines WR. Attention-based modulation of tactile stimuli: A comparison between prefrontal lesion patients and healthy age-matched controls. Neuropsychologia. 2014;57(1):101-11. doi: 10.1016/j.neuropsychologia.2014.03.003.

1560. Bonni S, Perri R, Fadda L, Tornaiuolo F, Koch G, Caltagirone C, et al. Selective deficit of spatial short-term memory: Role of storage and rehearsal mechanisms. Cortex. 2014;59:22-32. doi: 10.1016/j.cortex.2014.06.004. PubMed PMID: WOS:000343838500003.

1561. Brody AL, Okita K, Shieh J, Liang L, Hubert R, Mamoun M, et al. Radiation dosimetry and biodistribution of the translocator protein radiotracer [(11)C]DAA1106 determined with PET/CT in healthy human volunteers. Nucl Med Biol. 2014;41(10):871-5. Epub 2014/08/27. doi: 10.1016/j.nucmedbio.2014.07.004. PubMed PMID: 25156039; PubMed Central PMCID: PMCPMC4192057.

1562. Brody AL, Okita K, Shieh J, Liang L, Hubert R, Mamoun M, et al. Radiation dosimetry and biodistribution of the translocator protein radiotracer C-11 DAA1106 determined with PET/CT in healthy human volunteers. Nuclear Medicine and Biology. 2014;41(10):871-5. doi: 10.1016/j.nucmedbio.2014.07.004. PubMed PMID: WOS:000343614800013.

1563. Burke E, Dodakian L, See J, McKenzie A, Riley JD, Le V, et

al. A multimodal approach to understanding motor impairment and disability after stroke. Journal of Neurology. 2014;261(6):1178-86. doi: 10.1007/s00415-014-7341-8. PubMed PMID: WOS:000337750700017.

1564. Burke E, Dodakian L, See J, Riley JD, McKenzie A, Le V, et al. Different predictors of treatment gains in lacunar and non-lacunar stroke. Stroke. 2014;45.

1565. Cai B, Xia W, Bredenberg S, Engqvist H. Self-setting bioceramic microscopic protrusions for transdermal drug delivery. Journal of Materials Chemistry B. 2014;2(36):5992-8. doi: 10.1039/c4tb00764f. PubMed PMID: WOS:000341300300007.

1566. Castel-Lacanal E, Tarri M, Loubinoux I, Gasq D, de Boissezon X, Marque P, et al. Transcranial magnetic stimulation in brain injury. Ann Fr Anesth Reanim. 2014;33(2):83-7. Epub 2014/01/01. doi: 10.1016/j.annfar.2013.11.006. PubMed PMID: 24378049.

1567. Chen L, Zhu Y, Yang D, Zou R, Wu J, Tian H. Synthesis and antibacterial activities of antibacterial peptides with a spiropyran fluorescence probe. Scientific Reports. 2014;4. doi: 10.1038/srep06860.

1568. Chennu S, Finoia P, Kamau E, Allanson J, Williams GB, Monti MM, et al. Spectral Signatures of Reorganised Brain Networks

in Disorders of Consciousness. PLoS Computational Biology. 2014;10(10). doi: 10.1371/journal.pcbi.1003887.
1569. Corbetta M. Hemispatial neglect: Clinic, pathogenesis, and treatment. Seminars in Neurology. 2014;34(5):514-23. doi: 10.1055/s-0034-1396005.
1570. Coslett H, Faseyitan O, Medina J, Iannacone M. Effects of gaze, head and hand location on motor function: Evidence from TMS in normal subjects. Neurology. 2014;82(10).
1571. Cossu G. Therapeutic options to enhance coma arousal after traumatic brain injury: state of the art of current treatments to improve coma recovery. Br J Neurosurg. 2014;28(2):187-98. Epub 2013/10/05. doi: 10.3109/02688697.2013.841845. PubMed PMID: 24090192.
1572. Crescentini C, Aglioti SM, Fabbro F, Urgesi C. Virtual lesions of the inferior parietal cortex induce fast changes of implicit religiousness/spirituality. Cortex. 2014;54(1):1-15. doi: 10.1016/j.cortex.2014.01.023.
1573. Deer T. Spinal mapping and neuromodulation of the DRG. Regional Anesthesia and Pain Medicine. 2014;39(5):e35-e6. doi: 10.1097/AAP.0000000000000142.
1574. Deer TR, Mekhail N, Petersen E, Krames E, Staats P, Pope J, et al. The appropriate use of neurostimulation: stimulation of the intracranial and extracranial space and head for chronic pain.

Neuromodulation Appropriateness Consensus Committee. Neuromodulation. 2014;17(6):551-70; discussion 70. Epub 2014/08/13. doi: 10.1111/ner.12215. PubMed PMID: 25112890.

1575. Deer TR, Mekhail N, Petersen E, Krames E, Staats P, Pope J, et al. The Appropriate Use of Neurostimulation: Stimulation of the Intracranial and Extracranial Space and Head for Chronic Pain. Neuromodulation. 2014;17(6):551-70. doi: 10.1111/ner.12215. PubMed PMID: WOS:000340500200004.

1576. Defina P, Zakrzewski C, Prestigiacomo C, Machado C, Bernad P, Halper J, et al. Re-evaluating brain death: The potential for treatment and recovery after brain injury. Neurology. 2014;82(10).

1577. Deng Y-L, Juang Y-J. Polydimethyl siloxane wet etching for three dimensional fabrication of microneedle array and high-aspect-ratio micropillars. Biomicrofluidics. 2014;8(2). doi: 10.1063/1.4871038. PubMed PMID: WOS:000336082100027.

1578. Di Perri C, Heine L, Amico E, Soddu A, Laureys S, Demertzi A. Technology-based assessment in patients with disorders of consciousness. Ann Ist Super Sanita. 2014;50(3):209-20. Epub 2014/10/09. doi: 10.4415/ann_14_03_03. PubMed PMID: 25292268.

1579. Di Perri C, Stender J, Laureys S, Gosseries O. Functional neuroanatomy of disorders of consciousness. Epilepsy Behav. 2014;30:28-32. Epub 2013/10/09. doi: 10.1016/j.yebeh.2013.09.014.

PubMed PMID: 24100252.

1580. Di Perri C, Thibaut A, Heine L, Soddu A, Demertzi A, Laureys S. Measuring consciousness in coma and related states. World J Radiol. 2014;6(8):589-97. Epub 2014/08/30. doi: 10.4329/wjr.v6.i8.589. PubMed PMID: 25170396; PubMed Central PMCID: PMCPMC4147439.

1581. Dominguez LG, Radhu N, Farzan F, Daskalakis ZJ. Characterizing long interval cortical inhibition over the time-frequency domain. PLoS ONE. 2014;9(3). doi: 10.1371/journal.pone.0092354.

1582. Dougherty ET, Turner JC, Vogel F. Multiscale coupling of transcranial direct current stimulation to neuron electrodynamics: modeling the influence of the transcranial electric field on neuronal depolarization. Comput Math Methods Med. 2014;2014:360179. Epub 2014/11/19. doi: 10.1155/2014/360179. PubMed PMID: 25404950; PubMed Central PMCID: PMCPMC4227389.

1583. Du Plooy W, Mathibe L. Hospitalization of patients due to traditional medicine poisoning. Basic and Clinical Pharmacology and Toxicology. 2014;115:357.

1584. Eisner W, Fiegele T, Sohm F. Possible reason for the loss of effectivity of motor cortex stimulation for treatment of neuropathic pain syndromes. Stereotactic and Functional Neurosurgery.

2014;92:s37. doi: 10.1159/000366073.
1585. Ekusheva EV. Ipsilateral motor evoked potentials to transcranial magnetic stimulation in right and left hemisphere stroke. European Journal of Neurology. 2014;21:486.
1586. Ekusheva EV. Ipsilateral motor evoked potentials to transcranial magnetic stimulation in right-and left hemisphere stroke. Journal of Neurology. 2014;261:S322. doi: 10.1007/s00415-014-7337-4.
1587. Elahi F, Reddy C. High cervical epidural neurostimulation for post-traumatic headache management. Pain Physician. 2014;17(4):E537-41. Epub 2014/07/24. PubMed PMID: 25054404.
1588. Etheredge S, Zasler N. Cefaly neuromodulation for posttraumatic neuralgic headache. Brain Injury. 2014;28(5-6):791. doi: 10.3109/02699052.2014.892379.
1589. Farzan F. Single-pulse transcranial magnetic stimulation (TMS) protocols and outcome measures. Neuromethods2014. p. 69-115.
1590. Fayet G, Pegat B, Kassam R, Magot A, Roualdes V, N'Guyen JP, et al. Quantitative assessment of the cerebello-cortical inhibition by double-pulse transcranial magnetic stimulation in patients with cerebellar alterations. Clinical Neurophysiology. 2014;125:S241.
1591. Finsterer J, Stöllberger C, Güler N. Non-compaction

delineates amyotrophic lateral sclerosis from metabolic myopathy. International Journal of Cardiology. 2014;176(1):277-9. doi: 10.1016/j.ijcard.2014.06.092.

1592. Formisano R, Zasler N. Post-traumatic parkinsonism. Brain Injury. 2014;28(5-6):586. doi: 10.3109/02699052.2014.892379.

1593. Fox M, Buckner R, Lozano A, Pascual-Leone A. Connecting invasive and noninvasive brain stimulation. Neurology. 2014;82(10).

1594. Fox MD, Buckner RL, Liu H, Chakravarty MM, Lozano AM, Pascual-Leone A. Resting-state networks link invasive and noninvasive brain stimulation across diverse psychiatric and neurological diseases. Proc Natl Acad Sci U S A. 2014;111(41):E4367-75. Epub 2014/10/01. doi: 10.1073/pnas.1405003111. PubMed PMID: 25267639; PubMed Central PMCID: PMCPMC4205651.

1595. Frank S, Waschke A, Reichart R. RTMS in treatment of neuropathic facial pain-a prospective study of 20 patients. Clinical Neurophysiology. 2014;125:S242.

1596. Fridman EA, Schiff ND. Neuromodulation of the conscious state following severe brain injuries. Curr Opin Neurobiol. 2014;29:172-7. Epub 2014/10/07. doi: 10.1016/j.conb.2014.09.008. PubMed PMID: 25285395; PubMed Central PMCID: PMCPMC6519077.

1597. Gaede G, Hellweg R, Zimmermann H, Brandt AU, Dörr J, Bellmann-Strobl J, et al. Effects of deep repetitive transcranial magnetic stimulation on brain-derived neurotrophic factor serum concentration in healthy volunteers. Neuropsychobiology. 2014;69(2):112-9. doi: 10.1159/000358088.

1598. Galldiks N, Ruge MI, Hamisch C, Stoffels G, Ceccon G, Rapp M, et al. Differentiation of local recurrent brain metastasis from radiation-induced changes using O-(2-[18F]fluoroethyl)-l-tyrosine PET. Neuro-Oncology. 2014;16:ii16. doi: 10.1093/neuonc/nou174.

1599. Gao GY, Bao Y, Liang Y, Ding S, Jiang J. Right median nerve electrical stimulation improves the outcome of traumatic coma patients. Journal of neurotrauma. 2014;31(5):A43. doi: 10.1089/neu.2014.9937. PubMed PMID: CN-01058918.

1600. George MS, Raman R, Benedek DM, Pelic CG, Grammer GG, Stokes KT, et al. A two-site pilot randomized 3 day trial of high dose left prefrontal repetitive transcranial magnetic stimulation (rTMS) for suicidal inpatients. Brain Stimul. 2014;7(3):421-31. Epub 2014/04/16. doi: 10.1016/j.brs.2014.03.006. PubMed PMID: 24731434.

1601. Gibson N, Lay B, Blair E, Hamer P. The functional benefit and peripheral and central pathway changes derived from resistance training augmented with botulinum toxin A for the upper limb in children with spastic hemiplegia: a randomised controlled trial.

Developmental medicine and child neurology. 2014;56:53-4. doi: 10.1111/dmcn.12368. PubMed PMID: CN-01023244.
1602. Gimeno H, Tustin K, Lumsden D, Ashkan K, Selway R, Lin JP. Evaluation of functional goal outcomes using the Canadian Occupational Performance Measure (COPM) following Deep Brain Stimulation (DBS) in childhood dystonia. European Journal of Paediatric Neurology. 2014;18(3):308-16. doi: 10.1016/j.ejpn.2013.12.010.
1603. Giovannelli F, Chiaramonti R, Bianco G, Godone M, Battista D, Cardinali C, et al. Lack of behavioural effects of high-frequency rTMS in vegetative state: a randomised, double blind, sham-controlled, cross-over study. Clinical neurophysiology. 2014;125:S243. PubMed PMID: CN-01059912.
1604. Glannon W. Neuromodulation, Agency and Autonomy. Brain Topography. 2014;27(1):46-54. doi: 10.1007/s10548-012-0269-3. PubMed PMID: WOS:000329226500006.
1605. Gobbo M, Falciati L. Neuromodulation and cognitive rehabilitation: Addressing the methodological issue of circadian rhythms. Frontiers in Psychiatry. 2014;5(OCT). doi: 10.3389/fpsyt.2014.00150.
1606. Goldwasser T, Bressan S, Oakley E, Babl F. Use of sedation in children requiring a CT scan after head injury. EMA - Emergency

Medicine Australasia. 2014;26:9-10. doi: 10.1111/1742-6723.12243.

1607. Gonullu H, Aslan M, Karadas S, Kat C, Duran L, Milanlioglu A, et al. Serum prolidase enzyme activity and oxidative stress levels in patients with acute hemorrhagic stroke. Scandinavian Journal of Clinical and Laboratory Investigation. 2014;74(3):199-205. doi: 10.3109/00365513.2013.873949.

1608. Gosseries O, Laureys S. Current knowledge on severe acquired brain injury with disorders of consciousness. Brain Injury. 2014;28(9):1139-40. doi: 10.3109/02699052.2014.932554. PubMed PMID: WOS:000340455700001.

1609. Gosseries O, Thibaut A, Boly M, Rosanova M, Massimini M, Laureys S. Assessing consciousness in coma and related states using transcranial magnetic stimulation combined with electroencephalography. Ann Fr Anesth Reanim. 2014;33(2):65-71. Epub 2014/01/08. doi: 10.1016/j.annfar.2013.11.002. PubMed PMID: 24393302.

1610. Gosseries O, Zasler ND, Laureys S. Recent advances in disorders of consciousness: Focus on the diagnosis. Brain Injury. 2014;28(9):1141-50. doi: 10.3109/02699052.2014.920522. PubMed PMID: WOS:000340455700002.

1611. Grefkes C, Ward NS. Cortical Reorganization After Stroke: How Much and How Functional? Neuroscientist. 2014;20(1):56-70.

doi: 10.1177/1073858413491147. PubMed PMID: WOS:000329919300011.

1612. Guerra A, Costantini EM, Maatta S, Ponzo D, Ferreri F. Disorders of consciousness and electrophysiological treatment strategies: a review of the literature and new perspectives. Curr Pharm Des. 2014;20(26):4248-67. Epub 2013/09/13. PubMed PMID: 24025061.

1613. Guerra-Carrillo B, Mackey AP, Bunge SA. Resting-State fMRI: A Window into Human Brain Plasticity. Neuroscientist. 2014;20(5):522-33. doi: 10.1177/1073858414524442. PubMed PMID: WOS:000342640800013.

1614. Guller Y, Giacino J. Potential applications of concurrent transcranial magnetic stimulation and functional magnetic resonance imaging in acquired brain injury and disorders of consciousness. Brain Inj. 2014;28(9):1190-6. Epub 2014/08/08. doi: 10.3109/02699052.2014.920527. PubMed PMID: 25099023.

1615. Gun TK, Gul JD, Hee EP. EFFECTS OF DUAL MODE NON INVASIVE BRAIN STIMULATION ON MOTOR FUNCTION IN PATIENTS WITH CHRONIC TRAUMATIC BRAIN INJURY. Journal of Neurotrauma. 2014;31(12):A45-A. PubMed PMID: WOS:000338443600120.

1616. Harton B, Wang X, Pape T, Parrish T. EEG power spectrum

changes induced by transcranial magnetic stimulation in two vegetative patients. Brain Injury. 2014;28(5-6):772. doi: 10.3109/02699052.2014.892379.

1617. Heathcock J, Lo W, Chaudhari A. Endpoint kinematics and hand strength following transcranial magnetic stimulation in children with hemiparesis. Developmental Medicine and Child Neurology. 2014;56:64-5. doi: 10.1111/dmcn.12368.

1618. Herrold AA, Kletzel SL, Harton BC, Chambers RA, Jordan N, Pape TL. Transcranial magnetic stimulation: potential treatment for co-occurring alcohol, traumatic brain injury and posttraumatic stress disorders. Neural Regen Res. 2014;9(19):1712-30. Epub 2014/11/26. doi: 10.4103/1673-5374.143408. PubMed PMID: 25422632; PubMed Central PMCID: PMCPMC4238159.

1619. Hu H, Zhou Y, Leng T, Liu A, Wang Y, You X, et al. The major cholesterol metabolite cholestane-3β,5α,6β-triol functions as an endogenous neuroprotectant. Journal of Neuroscience. 2014;34(34):11426-38. doi: 10.1523/JNEUROSCI.0344-14.2014.

1620. Huang B, Zhou ZL, Wang LL, Zuo C, Lu Y, Chen Y. Electrical response grading versus House-Brackmann scale for evaluation of facial nerve injury after Bell's palsy: a comparative study. J Integr Med. 2014;12(4):367-71. Epub 2014/07/31. doi: 10.1016/s2095-4964(14)60036-4. PubMed PMID: 25074886.

1621. Hwang HS, Yi HJ, Lee SG, Sheen SH, Moon SM, Shin IY. Paroxysmal autonomic instability in traumatic brain injuries at neurosurgical intensive care unit. Journal of Neurotrauma. 2014;31(5):A64. doi: 10.1089/neu.2014.9937.

1622. Irct2014042817468N. The effect of transcranial direct current stimulation on pain and pain anxiety of burn in Patients with burns. https://trialsearchwhoint/Trial2aspx?TrialID=IRCT2014042817468N1. 2014. PubMed PMID: CN-01851089.

1623. Irct2014062318192N. Effect of transcranial Direct Current stimulation on inhibitory control. https://trialsearchwhoint/Trial2aspx?TrialID=IRCT2014062318192N1. 2014. PubMed PMID: CN-01846680.

1624. Jang SH. THE CORTICOSPINAL TRACT FROM THE VIEWPOINT OF BRAIN REHABILITATION. Journal of Rehabilitation Medicine. 2014;46(3):193-9. doi: 10.2340/16501977-1782. PubMed PMID: WOS:000332903400001.

1625. Jennings S, Russell N, Jennings B, Slee V, Sterling L, Castells M, et al. The mastocytosis society survey on mast cell disorders: Patient experiences and perceptions. Journal of Allergy and Clinical Immunology: In Practice. 2014;2(1):70-6. doi: 10.1016/j.jaip.2013.09.004.

1626. Jensen MP, Sherlin LH, Fregni F, Gianas A, Howe JD,

Hakimian S. Baseline brain activity predicts response to neuromodulatory pain treatment. Pain medicine (Malden, Mass). 2014;15(12):2055-63. doi: 10.1111/pme.12546. PubMed PMID: CN-01113278.

1627. Jeon HA. Hierarchical processing in the prefrontal cortex in a variety of cognitive domains. Frontiers in Systems Neuroscience. 2014;8(NOV):1-8. doi: 10.3389/fnsys.2014.00223.

1628. Jiang L, Ji Y, Voulalas PJ, Keaser M, Xu S, Gullapalli RP, et al. Motor Cortex Stimulation Suppresses Cortical Responses to Noxious Hindpaw Stimulation After Spinal Cord Lesion in Rats. Brain Stimulation. 2014;7(2):182-9. doi: 10.1016/j.brs.2013.12.013. PubMed PMID: WOS:000333442200005.

1629. Jiménez-Ortega L, García-Milla M, Fondevila S, Casado P, Hernández-Gutiérrez D, Martín-Loeches M. Automaticity of higher cognitive functions: Neurophysiological evidence for unconscious syntactic processing of masked words. Biological Psychology. 2014;103:83-91. doi: 10.1016/j.biopsycho.2014.08.011.

1630. Jolly S, Pringle N, Zhuang H, Li H, Richardson WD. Studies of a glial-specific G-protein-coupled receptor GPR37L1. FEBS Journal. 2014;281:382-3. doi: 10.1111/febs.12919.

1631. Julkunen P, Koenoenen M, Tarkka I, Maeaettae S, Vanninen R, Jaekaelae P, et al. Changes in cortical reactivity during stroke

recovery-a TMS-EEG study. Clinical Neurophysiology. 2014;125:S142-S3.
1632. Kaczmarek K, Tyler M, Skinner K, Verbny Y, Danilov Y. Cranial nerve non-invasive neuromodulation (CN-NINM) for symptomatic treatment of mild and moderate traumatic brain injury. Brain Injury. 2014;28(5-6):605-. PubMed PMID: WOS:000335017000219.
1633. Kakuda W, Abo M, Shimizu M, Sasanuma J, Okamoto T, Hara H, et al. Combination treatment of lowfrequency repetitive transcranial magnetic stimulation and intensive occupational therapy for upper limb hemiparesis after stroke: Clinical results in more than a thousand patients. Brain Injury. 2014;28(5-6):586-7. doi: 10.3109/02699052.2014.892379.
1634. Kelly C, Castellanos FX. Strengthening Connections: Functional Connectivity and Brain Plasticity. Neuropsychology Review. 2014;24(1):63-76. doi: 10.1007/s11065-014-9252-y. PubMed PMID: WOS:000335812400006.
1635. Khan B, Hodics T, Hervey N, Kondraske G, Stowe AM, Alexandrakis G. Personalizing non-invasive cortical stimulation for improving motor performance with perturbation transcranial direct current stimulation (ptDCS). Neuromodulation. 2014;17(5):e114. doi: 10.1111/ner.12232.

1636. Kim DR, Wang E. Prevention of supine hypotensive syndrome in pregnant women treated with transcranial magnetic stimulation. Psychiatry Research. 2014;218(1-2):247-8. doi: 10.1016/j.psychres.2014.04.001. PubMed PMID: WOS:000338614500042.

1637. Kirton A, Anderson J, Herrero M, Carsolio L, Mineyko A, Keess J, et al. Repetitive transcranial magnetic stimulation for perinatal stroke-induced cerebral palsy: Shortterm outcomes from the PLASTIC CHAMPS trial. Developmental Medicine and Child Neurology. 2014;56:84. doi: 10.1111/dmcn.12540.

1638. Klein PA, Petitjean C, Olivier E, Duque J. Top-down suppression of incompatible motor activations during response selection under conflict. NeuroImage. 2014;86:138-49. doi: 10.1016/j.neuroimage.2013.08.005.

1639. Koganemaru S, Ikeda A, Takahashi R, Fukuyama H, Mima T. Case report: Seizure induction by single-pulse transcranial magnetic stimulation after anodal transcranial direct current stimulation in a stroke patient. Clinical Neurophysiology. 2014;125:S225.

1640. Kronenburg A, van Doormaal T, van Eijsden P, van der Zwan A, Leijten F, Han KS. Surgery for a giant arteriovenous malformation without motor deterioration: preoperative transcranial magnetic stimulation in a non-cooperative patient. J Neurosurg Pediatr.

2014;14(1):38-42. Epub 2014/05/29. doi: 10.3171/2014.4.Peds13592. PubMed PMID: 24866824.

1641. Kronenburg A, Van Doormaal T, Van Eijsden P, Van Der Zwan A, Leijten F, Han KS. Surgery for a giant arteriovenous malformation without motor deterioration: Preoperative transcranial magnetic stimulation in a non-cooperative patient: Case report. Journal of Neurosurgery: Pediatrics. 2014;14(1):38-42. doi: 10.3171/2014.4.PEDS13592.

1642. Kukurin G. The use of non-invasive brain stimulation in mTBI treatment: A case series. Brain Injury. 2014;28(5-6):526-7. doi: 10.3109/02699052.2014.892379.

1643. Kwon HG, Jang SH. Delayed gait disturbance due to injury of the corticoreticular pathway in a patient with mild traumatic brain injury. Brain Injury. 2014;28(4):511-4. doi: 10.3109/02699052.2014.887228.

1644. Lairamore CI, Garrison MK, Bourgeon L, Mennemeier M. Effects of functional electrical stimulation on gait recovery post-neurological injury during inpatient rehabilitation. Percept Mot Skills. 2014;119(2):591-608. Epub 2014/08/26. doi: 10.2466/15.25.PMS.119c19z5. PubMed PMID: 25153616; PubMed Central PMCID: PMCPMC4341891.

1645. Lancioni GE, Bosco A, Belardinelli MO, Singh NN, O'Reilly

ME, Sigafoos J, et al. Technology-based intervention programs to promote stimulation control and communication in post-coma persons with different levels of disability. Frontiers in Human Neuroscience. 2014;8. doi: 10.3389/fnhum.2014.00048. PubMed PMID: WOS:000331945400001.

1646. Langsjo JW, Revonsuo A, Scheinin H. Harnessing Anesthesia and Brain Imaging for the Study of Human Consciousness. Current Pharmaceutical Design. 2014;20(26):4211-24. PubMed PMID: WOS:000338508000008.

1647. Lau BK, Vaughan CW. Targeting the endogenous cannabinoid system to treat neuropathic pain. Frontiers in Pharmacology. 2014;5 MAR. doi: 10.3389/fphar.2014.00028.

1648. Leal B, Zenatti L, Rangel R, Chaves J, Carvalho C, Bettencourt A, et al. Adenosinergic system in mesial temporal lobe epilepsy. Epilepsia. 2014;55:80. doi: 10.1111/epi.12675.

1649. Lefaucheur J-P, Andre-Obadia N, Antal A, Ayache SS, Baeken C, Benninger DH, et al. Evidence-based guidelines on the therapeutic use of repetitive transcranial magnetic stimulation (rTMS). Clinical Neurophysiology. 2014;125(11):2150-206. doi: 10.1016/j.clinph.2014.05.021. PubMed PMID: WOS:000345519600005.

1650. Lemaire JJ, Sontheimer A, Nezzar H, Pontier B, Luauté J,

Roche B, et al. Electrical modulation of neuronal networks in brain-injured patients with disorders of consciousness: a systematic review. Ann Fr Anesth Reanim. 2014;33(2):88-97. Epub 2013/12/24. doi: 10.1016/j.annfar.2013.11.007. PubMed PMID: 24361283.

1651. León-Carrión J, León-Domínguez U, Halper J, Pollonini L, Zouridakis G, Domínguez-Morales MR. Restoring cortical connectivity directionality and synchronization is essential to treating disorder of consciousness. Current Pharmaceutical Design. 2014;20(26):4268-74.

1652. Leśniak M, Polanowska K, Seniów J, Członkowska A. Effects of repeated anodal tDCS coupled with cognitive training for patients with severe traumatic brain injury: a pilot randomized controlled trial. J Head Trauma Rehabil. 2014;29(3):E20-9. Epub 2013/06/13. doi: 10.1097/HTR.0b013e318292a4c2. PubMed PMID: 23756431.

1653. Leung A, Shukla S, Song DD, Lin L, Tsai AM, Polston G, et al. rTMS in Reducing Mild TBI Related Headache- a Pilot Study. Headache. 2014;54:57-. PubMed PMID: WOS:000337950600111.

1654. Li H, Zhang T. A synopsis of recent influential papers published in mental health journals (2012-2013) in Mainland China. Asian Journal of Psychiatry. 2014;10:105-8. doi: 10.1016/j.ajp.2014.01.012.

1655. Liberati G, Hünefeldt T, Belardinelli MO. Questioning the

dichotomy between vegetative state and minimally conscious state: A review of the statistical evidence. 2014;8(November):1-14. doi: 10.3389/fnhum.2014.00865.

1656. Liew S-L, Santarnecchi E, Buch ER, Cohen LG. Non-invasive brain stimulation in neurorehabilitation: local and distant effects for motor recovery. Frontiers in Human Neuroscience. 2014;8. doi: 10.3389/fnhum.2014.00378. PubMed PMID: WOS:000339945300002.

1657. Lipinski LJ, Van Gompel J, Smith J, Spinner RJ. Spinal accessory neuropathy following dual tunneling of lead extensions for motor cortex stimulation. Stereotact Funct Neurosurg. 2014;92(3):178-81. Epub 2014/06/20. doi: 10.1159/000360863. PubMed PMID: 24943465.

1658. Lippa CF. Head injury and neuropsychological issues. American Journal of Alzheimer's Disease and other Dementias. 2014;29(3):197-8. doi: 10.1177/1533317514531362.

1659. Lohse-Busch H, Reime U, Falland R. Symptomatic treatment of unresponsive wakefulness syndrome with transcranially focused extracorporeal shock waves. NeuroRehabilitation. 2014;35(2):235-44. Epub 2014/07/06. doi: 10.3233/nre-141115. PubMed PMID: 24990026.

1660. López-Liria R, Fernández-Alonso M, Vega-Ramírez FA,

Salido-Campos M, Padilla-Góngora D. [Treatment and rehabilitation of dysphagia following cerebrovascular disease]. Rev Neurol. 2014;58(6):259-67. Epub 2014/03/13. PubMed PMID: 24610693.

1661. Maggioni G, Passaro I, Colombo R, Sterpi I, Giustini A, Pistarini C. Robotic rehabilitation therapy combined with transcranial direct current neuromodulation for the recovery of the upper limb in the subacute phase of stroke. Annals of Physical and Rehabilitation Medicine. 2014;57:e12. doi: 10.1016/j.rehab.2014.03.038.

1662. Mahayana IT, Hartono, Tcheang L, Chen CY, Juan CH, Muggleton NG. Posterior parietal cortex and visuospatial control in near and far space. Translational Neuroscience. 2014;5(4):269-74. doi: 10.2478/s13380-014-0229-3.

1663. Mall V. Neuromodulation in hand function training. Developmental Medicine and Child Neurology. 2014;56:5-6. doi: 10.1111/dmcn.12482-1.

1664. Maller JJ. Neuroplasticity in normal and brain injured patients: potential relevance of ear wiggling locus of control and cortical projections. Med Hypotheses. 2014;83(6):838-43. Epub 2014/12/04. doi: 10.1016/j.mehy.2014.11.003. PubMed PMID: 25468045.

1665. Mason RA, Prat CS, Just MA. Neurocognitive brain response to transient impairment of Wernicke's area. Cereb Cortex. 2014;24(6):1474-84. Epub 2013/01/17. doi: 10.1093/cercor/bhs423.

PubMed PMID: 23322403; PubMed Central PMCID: PMCPMC4014179.
1666. Masopust V, Rokyta R, Benes V. Neuromodulation. Ceska a Slovenska Neurologie a Neurochirurgie. 2014;77(2):138-51. PubMed PMID: WOS:000333662100001.
1667. Matsumoto-Miyazaki J, Yonezawa S, Nishiyama N, Okumura R, Fukuyama S, Kanematsu Y, et al. Acupuncture treatment increases motor evoked potentials induced by using transcranial magnetic stimulation in patients with chronic disorder of consciousness following severe traumatic brain injury. Brain Injury. 2014;28(5-6):597-8. PubMed PMID: WOS:000335017000202.
1668. Mazzi C, Mancini F, Savazzi S. Can IPS reach visual awareness without V1? Evidence from TMS in healthy subjects and hemianopic patients. Neuropsychologia. 2014;64:134-44. doi: 10.1016/j.neuropsychologia.2014.09.026.
1669. McCullagh P, Lightbody G, Zygierewicz J, Kernohan WG. Ethical challenges associated with the development and deployment of brain computer interface technology. Neuroethics. 2014;7(2):109-22. doi: 10.1007/s12152-013-9188-6.
1670. Merlo L, Cimino F, Angileri FF, La Torre D, Conti A, Cardali SM, et al. Alteration in synaptic junction proteins following traumatic brain injury. J Neurotrauma. 2014;31(16):1375-85. Epub 2014/03/26.

doi: 10.1089/neu.2014.3385. PubMed PMID: 24661152.

1671. Middleton A, Fritz SL, Liuzzo DM, Newman-Norlund R, Herter TM. Using clinical and robotic assessment tools to examine the feasibility of pairing tDCS with upper extremity physical therapy in patients with stroke and TBI: a consideration-of-concept pilot study. NeuroRehabilitation. 2014;35(4):741-54. Epub 2014/10/18. doi: 10.3233/nre-141178. PubMed PMID: 25323084; PubMed Central PMCID: PMCPMC4268358.

1672. Middleton A, Liuzzo D, Newman-Norlund R, Fritz S, Herter T. Quantifying the feasibility of pairing tDCS with standard physical therapy following neurological insult, using clinical and robotic assessment tools. Neurorehabilitation and Neural Repair. 2014;28(4):NP1-NP2. doi: 10.1177/1545968314529927.

1673. Miller NR, Yasen AL, Maynard LF, Chou LS, Howell DR, Christie AD. Acute and longitudinal changes in motor cortex function following mild traumatic brain injury. Brain Inj. 2014;28(10):1270-6. Epub 2014/05/21. doi: 10.3109/02699052.2014.915987. PubMed PMID: 24841536.

1674. Min KS, Lee CJ, Jun SB, Kim J, Lee SE, Shin J, et al. A liquid crystal polymer-based neuromodulation system: an application on animal model of neuropathic pain. Neuromodulation. 2014;17(2):160-9. Epub 2013/09/13. doi: 10.1111/ner.12093. PubMed

PMID: 24024655.

1675. Minter MR, Zhang M, Ates RC, Taylor JM, Crack PJ. Type-1 interferons contribute to oxygen glucose deprivation induced neuro-inflammation in BE(2)M17 human neuroblastoma cells. Journal of Neuroinflammation. 2014;11. doi: 10.1186/1742-2094-11-43.

1676. Mistry S, Michou E, Vasant D, Ghannouchi I, Verin E, Hamdy S. New techniques-future. Journal of Gastroenterology and Hepatology Research. 2014;3(5):1080-7. doi: 10.6051/j.issn.2224-3992.2014.03.408-14.

1677. Mitchell AS, Murray Sherman S, Sommer MA, Mair RG, Vertes RP, Chudasama Y. Advances in understanding mechanisms of thalamic relays in cognition and behavior. Journal of Neuroscience. 2014;34(46):15340-6. doi: 10.1523/JNEUROSCI.3289-14.2014.

1678. Moeller DR, Duffey JM, Goolsby AM, Gallimore JT. Use of a Removable Mandibular Neuroprosthesis for the Reduction of Posttraumatic Stress Disorder (PTSD) and Mild Traumatic Brain Injury/PTSD/Associated Nightmares, Headaches, and Sleep Disturbances. Journal of special operations medicine : a peer reviewed journal for SOF medical professionals. 2014;14(3):64-73.

1679. Momosaki R, Abo M, Watanabe S, Kakuda W, Yamada N, Mochio K. Influence of functional magnetic stimulation on swallowing function. Brain Injury. 2014;28(5-6):562. doi:

10.3109/02699052.2014.892379.

1680. Moore NZ, Lempka SF, Machado A. Central neuromodulation for refractory pain. Neurosurg Clin N Am. 2014;25(1):77-83. Epub 2013/11/23. doi: 10.1016/j.nec.2013.08.011. PubMed PMID: 24262901; PubMed Central PMCID: PMCPMC3867973.

1681. Moreno-Duarte I, Morse LR, Alam M, Bikson M, Zafonte R, Fregni F. Targeted therapies using electrical and magnetic neural stimulation for the treatment of chronic pain in spinal cord injury. Neuroimage. 2014;85 Pt 3:1003-13. Epub 2013/06/04. doi: 10.1016/j.neuroimage.2013.05.097. PubMed PMID: 23727533.

1682. Morton DL, El-Deredy W, Jones AK. Placebo analgesia: Cognition or perception. Handbook of Experimental Pharmacology2014. p. 71-80.

1683. Moy SL. Concussion in athletes: From pathophysiology to current guidelines and recommendations. Osteopathic Family Physician. 2014;6(2):8-13. doi: 10.1016/ofp.v6i2.11.

1684. Muthukumaraswamy SD. The use of magnetoencephalography in the study of psychopharmacology (pharmaco-MEG). Journal of Psychopharmacology. 2014;28(9):815-29. doi: 10.1177/0269881114536790. PubMed PMID: WOS:000340182700001.

1685. Najib U, Horvath JC. Transcranial Magnetic Stimulation

(TMS) safety considerations and recommendations. 2014. p. 15-30.

1686. Nam HJ, Shin WC, Kwon YN, Lee JS. Cortical excitability difference in postictal state of epileptic seizure and syncope associated with loss of consciousness. Epilepsy Currents. 2014;14:45.

1687. Napolitani M, Bodart O, Canali P, Seregni F, Casali A, Laureys S, et al. Transcranial magnetic stimulation combined with high-density EEG in altered states of consciousness. Brain Inj. 2014;28(9):1180-9. Epub 2014/08/08. doi: 10.3109/02699052.2014.920524. PubMed PMID: 25099022.

1688. Nardone R, Höller Y, Leis S, Höller P, Thon N, Thomschewski A, et al. Invasive and non-invasive brain stimulation for treatment of neuropathic pain in patients with spinal cord injury: a review. J Spinal Cord Med. 2014;37(1):19-31. Epub 2013/10/05. doi: 10.1179/2045772313y.0000000140. PubMed PMID: 24090372; PubMed Central PMCID: PMCPMC4066547.

1689. Naryshkin AG, Gorelik AL, Skoromets TA, Egorov A, Vtorov AV, Martynov IV. [On possibility of partial restore integrative brain activity in patients with vegetative state]. Fiziol Cheloveka. 2014;40(5):69-79. Epub 2015/02/26. PubMed PMID: 25711097.

1690. Nct. rTMS to Improve Cognitive Function in TBI. https://clinicaltrialsgov/show/NCT02152540. 2014. PubMed PMID: CN-01596932.

1691. Nct. Brain Stimulation for Traumatic Brain Injury. https://clinicaltrialsgov/show/NCT02167971. 2014. PubMed PMID: CN-02037454.

1692. Nct. Brain Stimulation for Mild Traumatic Brain Injury. https://clinicaltrialsgov/show/NCT02292589. 2014. PubMed PMID: CN-01550264.

1693. Nct. Noninvasive Neuromodulation for Treatment of Symptoms Due to Mild or Moderate Traumatic Brain Injury. https://clinicaltrialsgov/show/NCT02158494. 2014. PubMed PMID: CN-01546282.

1694. Nct. Cranial-nerve Non-invasive Neuromodulation (CN-NINM) for Balance Deficits After Mild Traumatic Brain Injury (Full Trial). https://clinicaltrialsgov/show/NCT02125591. 2014. PubMed PMID: CN-01545356.

1695. Nct. Cranial-nerve Non-invasive Neuromodulation (CN-NINM) for Balance Deficits After Mild Traumatic Brain Injury (Pilot Trial). https://clinicaltrialsgov/show/NCT02109198. 2014. PubMed PMID: CN-01544918.

1696. Nct. Brain Mechanisms Underlying Reading Improvement in Central Alexia. https://clinicaltrialsgov/show/NCT02062619. 2014. PubMed PMID: CN-01586876.

1697. Nct. Action Observation Treatment (AOT) as a Rehabilitation

Tool. https://clinicaltrialsgov/show/NCT02235350. 2014. PubMed PMID: CN-01548556.

1698. Nct. Prolonged-release Fampridine as Adjunct Therapy to Active Motor Training in MS Patients. https://clinicaltrialsgov/show/NCT02146534. 2014. PubMed PMID: CN-01545966.

1699. Nct. Treating Refractory Schizophrenia With rTMS. https://clinicaltrialsgov/show/NCT02242578. 2014. PubMed PMID: CN-01548763.

1700. Nct. The Effects of Exercise Training on Shoulder Neuromuscular Control. https://clinicaltrialsgov/show/NCT02164305. 2014. PubMed PMID: CN-01546444.

1701. Neva JL, Singh AM, Vesia M, Staines WR. Selective modulation of left primary motor cortex excitability after continuous theta burst stimulation to right primary motor cortex and bimanual training. Behavioural Brain Research. 2014;269:138-46. doi: 10.1016/j.bbr.2014.04.041. PubMed PMID: WOS:000337866400018.

1702. Neva JL, Vesia M, Singh AM, Staines WR. Modulation of left primary motor cortex excitability after bimanual training and intermittent theta burst stimulation to left dorsal premotor cortex. Behavioural Brain Research. 2014;261:289-96. doi: 10.1016/j.bbr.2013.12.029. PubMed PMID: WOS:000331497000036.

1703. Nishijima DK. Tackling the persistent effects of concussion. Science Translational Medicine. 2014;6(255). doi: 10.1126/scitranslmed.3010413.

1704. Notaro P, Buratti E, Meroni A, Montagna MC, Rubino FG, Voltolini A. The effects of peripheral occipital nerve stimulation for the treatment of patients suffering from chronic migraine: a single center experience. Pain Physician. 2014;17(3):E369-74. Epub 2014/05/23. PubMed PMID: 24850118.

1705. O'Neill B, Gardani M, Findlay G, Whyte T, Cullen T. Challenging behaviour and sleep cycle disorder following brain injury: A preliminary response to agomelatine treatment. Brain Injury. 2014;28(3):378-81. doi: 10.3109/02699052.2013.865264.

1706. Odinak MM, Zhivolupov SA, Panomarev VV, Rashidov NA, Samartsev IN. Recovery of consciousness as manifestation of neuroplasticity. Zh Vopr Neirokhir Im N N Burdenko. 2014;78(1):33-41; discussion Epub 2014/04/26. PubMed PMID: 24761594.

1707. Ostergard T, Miller JP. Deep brain stimulation: new directions. J Neurosurg Sci. 2014;58(4):191-8. Epub 2014/11/25. PubMed PMID: 25418273.

1708. Pąchalska M, Buliński L, Jauer-Niworowska O, Rasmus A, Daniluk B, Mirski A, et al. Neuropsychological functioning after

traumatic injury to the orbito-frontal area: A Polish Phineas Gage. Acta Neuropsychologica. 2014;12(4):503-16. doi: 10.5604/17307503.1138851.
1709. Pan B, Liu J, Zhang Y, Sun Y, Wu Q, Zhao K, et al. Acute ablation of DP thymocytes induces up-regulation of IL-22 and Foxn1 in TECs. Clin Immunol. 2014;150(1):101-8. Epub 2013/12/18. doi: 10.1016/j.clim.2013.11.002. PubMed PMID: 24333537.
1710. Papa L, LaMee A, Tan CN, Hill-Pryor C. Systematic review and meta-analysis of noninvasive cranial nerve neuromodulation for nervous system disorders. Arch Phys Med Rehabil. 2014;95(12):2435-43. Epub 2014/05/13. doi: 10.1016/j.apmr.2014.04.018. PubMed PMID: 24814561.
1711. Pape T. rTMS related changes in neural network functional connectivity for subjects in vegetative state after TBI. Archives of Physical Medicine and Rehabilitation. 2014;95(10):e73-e4.
1712. Pape T, Rosenow J, Patil V, Steiner M, Harton B, Wang X, et al. TMS related changes in functional connectivity of default mode network and associated neurobehavioural gains for subjects in vegetative state after traumatic brain injury. Brain Injury. 2014;28(5-6):708-. PubMed PMID: WOS:000335017000463.
1713. Pape TL, Rosenow JM, Patil V, Steiner M, Harton B, Guernon A, et al. RTMS safety for two subjects with disordered consciousness

after traumatic brain injury. Brain Stimul. 2014;7(4):620-2. Epub 2014/05/20. doi: 10.1016/j.brs.2014.03.007. PubMed PMID: 24836500.

1714. Parmar VK, Gee L, Smith H, Pilitsis JG. Supraspinal stimulation for treatment of refractory pain. Clin Neurol Neurosurg. 2014;123:155-63. Epub 2014/06/24. doi: 10.1016/j.clineuro.2014.05.026. PubMed PMID: 24956545.

1715. Patuzzo S, Manganotti P. Deep Brain Stimulation in Persistent Vegetative States: Ethical Issues Governing Decision Making. Behavioural Neurology. 2014;2014. doi: 10.1155/2014/641213. PubMed PMID: WOS:000333597000001.

1716. Pearce AJ, Corp DT, Davies CB, Major BP, Maller JJ. Second time around: Corticospinal responses following repeated sports-related concussions within the same season. A transcranial magnetic stimulation study. Journal of Acute Disease. 2014;3(3):186-93. doi: 10.1016/S2221-6189(14)60042-1.

1717. Pearce AJ, Hoy K, Rogers MA, Corp DT, Maller JJ, Drury HG, et al. The long-term effects of sports concussion on retired Australian football players: a study using transcranial magnetic stimulation. J Neurotrauma. 2014;31(13):1139-45. Epub 2014/03/04. doi: 10.1089/neu.2013.3219. PubMed PMID: 24579780.

1718. Pérez De Los Cobos F, Chatterjee M, Egger V. Long-term

plasticity at the olfactory bulb mitral-granule cell synapse. Chemical Senses. 2014;39(1):107. doi: 10.1093/chemse/bjt055.

1719. Pistoia F, Sacco S, Sarà M, Franceschini M, Carolei A. Intrathecal Baclofen: Effects on Spasticity, Pain, and Consciousness in Disorders of Consciousness and Locked-in Syndrome. Current Pain and Headache Reports. 2014;19(1). doi: 10.1007/s11916-014-0466-8.

1720. Plow EB, Cunningham DA, Varnerin N, Janini D, Sakaie K, Frost F. Cortical plasticity to improve rehabilitative outcomes in patients with quadriplegia. Journal of spinal cord medicine. 2014;37(4):473-4. doi: 10.1179/1079026814Z. PubMed PMID: CN-01056930.

1721. Powers KC, Cinelli ME, Kalmar JM. Cortical hypoexcitability persists beyond the symptomatic phase of a concussion. Brain Inj. 2014;28(4):465-71. Epub 2014/04/08. doi: 10.3109/02699052.2014.888759. PubMed PMID: 24702432.

1722. Prager J, Deer T, Levy R, Bruel B, Buchser E, Caraway D, et al. Best practices for intrathecal drug delivery for pain. Neuromodulation. 2014;17(4):354-72. doi: 10.1111/ner.12146.

1723. Preston C, Newport R. Noisy visual feedback training impairs detection of self-generated movement error: Implications for anosognosia for hemiplegia. Frontiers in Human Neuroscience. 2014;8(JUNE). doi: 10.3389/fnhum.2014.00456.

1724. Prichard G, Weiner C, Fritsch B, Reis J. Effects of Different Electrical Brain Stimulation Protocols on Subcomponents of Motor Skill Learning. Brain Stimulation. 2014;7(4):532-40. doi: 10.1016/j.brs.2014.04.005. PubMed PMID: WOS:000339984300006.
1725. Raicevic A. Clinical modeling of neurolysis. Annals of Physical and Rehabilitation Medicine. 2014;57:e22. doi: 10.1016/j.rehab.2014.03.077.
1726. Railo H, Andersson E, Kaasinen V, Laine T, Koivisto M. Unlike in clinical blindsight patients, unconscious processing of chromatic information depends on early visual cortex in healthy humans. Brain Stimul. 2014;7(3):415-20. Epub 2014/04/05. doi: 10.1016/j.brs.2014.01.060. PubMed PMID: 24698972.
1727. Reddy A, Cho J, Ling S, Reddy V, Shlykov M, Saier MH. Reliability of nine programs of topological predictions and their application to integral membrane channel and carrier proteins. Journal of Molecular Microbiology and Biotechnology. 2014;24(3):161-90. doi: 10.1159/000363506.
1728. Rezai AR, Sharma M. Deep Brain Stimulation (DBS): Current and emerging applications. Japanese Journal of Neurosurgery. 2014;23(8):648-60. doi: 10.7887/jcns.23.648.
1729. Ribeiro AS, Ramos A, Bermejo E, Casero M, Corrales JM, Grantham S. Effects of different musical stimuli in vital signs and

facial expressions in patients with cerebral damage: a pilot study. J Neurosci Nurs. 2014;46(2):117-24. Epub 2014/02/22. doi: 10.1097/jnn.0000000000000037. PubMed PMID: 24556659.

1730. Roberts L, Cacace AT. Jendrassik maneuver facilitates cVEMP amplitude: Some preliminary observations. Journal of the American Academy of Audiology. 2014;25(3):237-43. doi: 10.3766/jaaa.25.3.2.

1731. Roebuck H, Bourke P, Guo K. Role of lateral and feedback connections in primary visual cortex in the processing of spatiotemporal regularity - A TMS study. Neuroscience. 2014;263:231-9. doi: 10.1016/j.neuroscience.2014.01.027.

1732. Rollnik JD, Altenmueller E. Music in disorders of consciousness. Frontiers in Neuroscience. 2014;8. doi: 10.3389/fnins.2014.00190. PubMed PMID: WOS:000346496900001.

1733. Sabel BA, Brösel D, Krohn F, Schlaug C, Rossini PM, Tatlisumak T, et al. REVIS-network: Restoration of vision after stroke with noninvasive alternating current stimulation to improve visual impairment. Brain Injury. 2014;28(5-6):764. doi: 10.3109/02699052.2014.892379.

1734. Sachs AJ, Babu H, Su YF, Miller KJ, Henderson JM. Lack of efficacy of motor cortex stimulation for the treatment of neuropathic pain in 14 patients. Neuromodulation. 2014;17(4):303-11. doi: 10.1111/ner.12181.

1735. Sarasso S, Rosanova M, Casali AG, Casarotto S, Fecchio M, Boly M, et al. Quantifying cortical EEG responses to TMS in (un)consciousness. Clin EEG Neurosci. 2014;45(1):40-9. Epub 2014/01/10. doi: 10.1177/1550059413513723. PubMed PMID: 24403317.

1736. Schintu S, Hadj-Bouziane F, Dal Monte O, Knutson KM, Pardini M, Wassermann EM, et al. Object and space perception - Is it a matter of hemisphere? Cortex. 2014;57:244-53. doi: 10.1016/j.cortex.2014.04.009. PubMed PMID: WOS:000340317500018.

1737. Seidl SE, Izadi A, Lee DJ, Melnik M, Ekstrom AD, Shahlaie K, et al. STIMULATION OF THE MEDIAL SEPTUM DRIVES HIPPOCAMPAL THETA OSCILLATIONS LEADING TO PERSISTENT INCREASES IN THETA PHASE COHERENCE. Journal of Neurotrauma. 2014;31(12):A98-A9. PubMed PMID: WOS:000338443600267.

1738. Shabalov V, Isagulyan E, Salova E, Tomskiy A. Central Cortex Stimulation (CCS) in the complex treatment of severe pain. Stereotactic and functional neurosurgery Conference: 21st congress of the european society for stereotactic and functional neurosurgery: implementing research in clinical applications maastricht netherlands Conference start: 20140917 conference end: 20140920 Conference

publication: (varpagings). 2014;92:s122. doi: 10.1159/000367644. PubMed PMID: CN-01173725.

1739. Shah PP, Norise C, Garcia G, Torres J, Faseyitan O, Hamilton RH. Lesion correlates of transcranial direct current stimulation in chronic nonfluent aphasia. Annals of Neurology. 2014;76:S25-S6. doi: 10.1002/ana.24247.

1740. Shin DS, Hwang SC, Kim BT, Jeong JH, Im SB, Shin WH. Serial Brain CT Scans in Severe Head Injury without Intracranial Pressure Monitoring. Korean J Neurotrauma. 2014;10(1):26-30. Epub 2014/04/01. doi: 10.13004/kjnt.2014.10.1.26. PubMed PMID: 27169029; PubMed Central PMCID: PMCPMC4852589.

1741. Shin SS, Dixon CE, Okonkwo DO, Richardson RM. Neurostimulation for traumatic brain injury. J Neurosurg. 2014;121(5):1219-31. Epub 2014/08/30. doi: 10.3171/2014.7.Jns131826. PubMed PMID: 25170668.

1742. Sidyakina I, Dobrushina O, Degterev D. Navigated fMRI-guided rTMS in rehabilitation of swallowing: A case demonstration. Annals of Physical and Rehabilitation Medicine. 2014;57:e265. doi: 10.1016/j.rehab.2014.03.962.

1743. Silva D, Colvin L, Hagemann E, Stanley F, Bower C. Children diagnosed with attention deficit disorder and their hospitalisations: population data linkage study. Eur Child Adolesc Psychiatry.

2014;23(11):1043-50. Epub 2014/04/29. doi: 10.1007/s00787-014-0545-8. PubMed PMID: 24770488.

1744. Silvanto J. The use of brain stimulation in the rehabilitation of visual and attentional functions. Clinical Neurophysiology. 2014;125:S5.

1745. Singh J, Kaur M, Kulshrestha A, Bajwa SJS. Recent advances in pre-eclampsia management: An anesthesiologist's perspective! Anaesthesia, Pain and Intensive Care. 2014;18(2):209-14.

1746. Smorenburg A, Kuo H, Ferre CL, Brandão M, Carmel J, Gordon A, et al. Wired for recovery? How corticospinal system connectivity impacts on the efficacy of bimanual therapy in children with unilateral cerebral palsy. Developmental Medicine and Child Neurology. 2014;56:81-2. doi: 10.1111/dmcn.12540.

1747. Solbakk AK, Funderud I, Løvstad M, Endestad T, Meling T, Lindgren M, et al. Impact of orbitofrontal lesions on electrophysiological signals in a stop signal task. Journal of Cognitive Neuroscience. 2014;26(7):1528-45. doi: 10.1162/jocn_a_00561.

1748. Song XJ, Luo MF, Jiang J, Zhang JL. [Effects of electroacupuncture stimulation of "Zusanli" (ST 36) on the regional vascular intercellular Adhesion Molecule-1 mRNA expression and mast cell activity in normal rats]. Zhen Ci Yan Jiu. 2014;39(6):461-5. Epub 2015/01/31. PubMed PMID: 25632570.

1749. Spreij LA, Visser-Meily JMA, van Heugten CM, Nijboer TCW. Novel insights into the rehabilitation of memory post acquired brain injury: A systematic review. Frontiers in Human Neuroscience. 2014;8(DEC). doi: 10.3389/fnhum.2014.00993.

1750. Strigo IA, Spadoni AD, Lohr J, Simmons AN. Too hard to control: Compromised pain anticipation and modulation in mild traumatic brain injury. Translational Psychiatry. 2014;4. doi: 10.1038/tp.2013.116.

1751. Su J, Wang W, Sun L, Li T, Kong D, Kang J. Raphe serotonergic neurons modulate genioglossus corticomotor activity in intermittent hypoxic rats. Respiratory Research. 2014;15(1). doi: 10.1186/1465-9921-15-76.

1752. Sukul VV, Slavin KV. Deep brain and motor cortex stimulation. Curr Pain Headache Rep. 2014;18(7):427. Epub 2014/05/13. doi: 10.1007/s11916-014-0427-2. PubMed PMID: 24817154.

1753. Suykerbuyk J, Demeester M. Duloxetine as a treatment of mood instability after acquired brain injury. Brain Injury. 2014;28(5-6):581. doi: 10.3109/02699052.2014.892379.

1754. Sweet JA, Eakin KC, Munyon CN, Miller JP. Improved learning and memory with theta-burst stimulation of the fornix in rat model of traumatic brain injury. Hippocampus. 2014;24(12):1592-600.

Epub 2014/08/05. doi: 10.1002/hipo.22338. PubMed PMID: 25087862.

1755. Swick D, Chatham CH. Ten years of inhibition revisited. Frontiers in Human Neuroscience. 2014;8(MAY). doi: 10.3389/fnhum.2014.00329.

1756. Tang Y, Shang Q, Zhou LH. [Vegetative state treated with acupoint injection combined with plum-blossom needle in children: a randomized controlled trial]. Zhongguo Zhen Jiu. 2014;34(5):421-5. Epub 2014/07/16. PubMed PMID: 25022107.

1757. Taub E, Uswatte G, Mark VW. The functional significance of cortical reorganization and the parallel development of CI therapy. Frontiers in Human Neuroscience. 2014;8(JUNE). doi: 10.3389/fnhum.2014.00396.

1758. Tazoe T, Perez MA. Selective activation of ipsilateral motor pathways in intact humans. Journal of neuroscience. 2014;34(42):13924-34. doi: 10.1523/JNEUROSCI.1648-14.2014. PubMed PMID: CN-01053633.

1759. Thibaut A, Bruno M-A, Ledoux D, Demertzi A, Laureys S. tDCS in patients with disorders of consciousness. Neurology. 2014;82(13):1112-8. doi: 10.1212/wnl.0000000000000260. PubMed PMID: WOS:000335977100006.

1760. Thibaut A, Bruno MA, Ledoux D, Demertzi A, Laureys S.

tDCS in patients with disorders of consciousness: sham-controlled randomized double-blind study. Neurology. 2014;82(13):1112-8. Epub 2014/02/28. doi: 10.1212/wnl.0000000000000260. PubMed PMID: 24574549.

1761. Thibaut A, Bruno MA, Ledoux D, Demertzi A, Laureys S. tDCS in patients with disorders of consciousness: sham-controlled randomized double-blind study. Neurology. 2014;82(13):1112-8. Epub 2014/02/28. doi: 10.1212/wnl.0000000000000260. PubMed PMID: 24574549.

1762. Thompson FJ, Hou J, Mustafa G, Wilkie Z, Tsuda S, Nelson R, et al. THERAPEUTIC TMS REDUCES TBI SPASTICITY, ANXIETY, AND PAIN. Journal of Neurotrauma. 2014;31(12):A47-A. PubMed PMID: WOS:000338443600126.

1763. Tonello F, Francesco C, Tebaldi F, Cantagallo A. High technology for aphasia rehabilitation. Annals of Physical and Rehabilitation Medicine. 2014;57:e144. doi: 10.1016/j.rehab.2014.03.1595.

1764. Tracey I, Flower R. The warrior in the machine: Neuroscience goes to war. Nature Reviews Neuroscience. 2014;15(12):825-34. doi: 10.1038/nrn3835.

1765. Tran MTD, Skovbjerg S, Arendt-Nielsen L, Bech P, Lunde M, Elberling J. Two of three patients with multiple chemical sensitivity

had less symptoms and secondary hyperalgesia after transcranially applied pulsed electromagnetic fields. Scandinavian Journal of Pain. 2014;5(2):104-9. doi: 10.1016/j.sjpain.2013.11.008.

1766. Treffert DA. Accidental genius. Sci Am. 2014;311(2):52-7. Epub 2014/08/07. doi: 10.1038/scientificamerican0814-52. PubMed PMID: 25095469.

1767. Tremblay S, Beaulé V, Proulx S, Tremblay S, Marjańska M, Doyon J, et al. Multimodal assessment of primary motor cortex integrity following sport concussion in asymptomatic athletes. Clin Neurophysiol. 2014;125(7):1371-9. Epub 2014/01/28. doi: 10.1016/j.clinph.2013.11.040. PubMed PMID: 24462505; PubMed Central PMCID: PMCPMC4381958.

1768. Tremblay S, Lepage J-F, Latulipe-Loiselle A, Fregni F, Pascual-Leone A, Theoret H. The Uncertain Outcome of Prefrontal tDCS. Brain Stimulation. 2014;7(6):773-83. doi: 10.1016/j.brs.2014.10.003. PubMed PMID: WOS:000346455200002.

1769. Uhrig L, Dehaene S, Jarraya B. Cerebral mechanisms of general anesthesia. Annales Francaises d'Anesthesie et de Reanimation. 2014;33(2):72-82. doi: 10.1016/j.annfar.2013.11.005.

1770. Ulyanova AV, Koch PF, Grovola MR, Harris JP, Cullen DK, Wolf JA. The development of epileptogenic activity after diffuse brain injury in swine. Journal of Neurotrauma. 2014;31(12):A2. doi:

10.1089/neu.2014.9935.abstracts.
1771. Urbanski M, Coubard OA, Bourlon C. Visualizing the blind brain: brain imaging of visual field defects from early recovery to rehabilitation techniques. Front Integr Neurosci. 2014;8:74. Epub 2014/10/18. doi: 10.3389/fnint.2014.00074. PubMed PMID: 25324739; PubMed Central PMCID: PMCPMC4179723.
1772. Urgesi C, Candidi M, Avenanti A. Neuroanatonnical substrates of action perception and understanding: an anatomic likelihood estimation meta-analysis of lesion-symptom mapping studies in brain injured patients. Frontiers in Human Neuroscience. 2014;8. doi: 10.3389/fnhum.2014.00344. PubMed PMID: WOS:000339500600001.
1773. Urgesi C, Candidi M, Avenanti A. Neuroanatomical substrates of action perception and understanding: An anatomic likelihood estimation meta-analysis of lesion-symptom mapping studies in brain injured patients. Frontiers in Human Neuroscience. 2014;8(MAY). doi: 10.3389/fnhum.2014.00344.
1774. Vanzan S, Ferguson H, Bodani M, Sakel M. The effects of caloric vestibular stimulation (CVS) on low awareness states. Archives of Physical Medicine and Rehabilitation. 2014;95(10):e70-e1.
1775. Vernet M, Quentin R, Chanes L, Mitsumasu A, Valero-Cabré

A. Frontal eye field, where art thou? Anatomy, function, and non-invasive manipulation of frontal regions involved in eye movements and associated cognitive operations. Frontiers in Integrative Neuroscience. 2014;8(AUG). doi: 10.3389/fnint.2014.00066.

1776. Vidal AC, Banca P, Gil Pascoal A, Cordeiro G, Sargento-Freitas J, Gouveia A, et al. Acute neuroimaging cortical interhemispheric modulation by motion induced interactions of the upper limbs in acute ischemic stroke. International Journal of Stroke. 2014;9:58. doi: 10.1111/ijs.12375.

1777. Volman V, Ng LJ. Primary paranode demyelination modulates slowly developing axonal depolarization in a model of axonal injury. Journal of Computational Neuroscience. 2014;37(3):439-57. doi: 10.1007/s10827-014-0515-7.

1778. Wang P-C, Paik S-J, Kim S-H, Allen MG. Hypodermic-Needle-Like Hollow Polymer Microneedle Array: Fabrication and Characterization. Journal of Microelectromechanical Systems. 2014;23(4):991-8. doi: 10.1109/jmems.2014.2307320. PubMed PMID: WOS:000341573300027.

1779. Waninger KN, Gloyeske BM, Hauth JM, Vanic KA, Yen DM. Intratympanic hemorrhage and concussion in a football offensive lineman. Journal of Emergency Medicine. 2014;46(3):371-2. doi:

10.1016/j.jemermed.2013.08.043.
1780. Weatherall A, Garner A, Lovell N, Redmond S, Lee A, Skowno J, et al. Study protocol for the PHANTOM study: Prehospital assessment of noninvasive tissue oximetry monitoring. Scandinavian Journal of Trauma, Resuscitation and Emergency Medicine. 2014;22(1). doi: 10.1186/s13049-014-0057-z.
1781. Wertheimer T, Kloss CC, Velardi E, Brede C, Ginsberg M, Kreines FM, et al. Endothelial Cells Promote Endogenous Thymic Regeneration after Injury Via BMP4 Signaling. Blood. 2014;124(21). PubMed PMID: WOS:000349243501095.
1782. Whyte J. Disorders of consciousness: the changing landscape of treatment. Neurology. 2014;82(13):1106-7. Epub 2014/02/28. doi: 10.1212/wnl.0000000000000276. PubMed PMID: 24574547.
1783. Wojtecki L, Petri D, Elben S, Hirschmann J, Yelnik J, Eickhoff S, et al. Modulation of central thalamic oscillations during emotional-cognitive processing in chronic disorder of consciousness. Cortex. 2014;60:94-102. doi: 10.1016/j.cortex.2014.09.007.
1784. Wolf JA, Browne K, Grovola M, Cullen DK, Ulyanova AV. Hippocampal circuitry disruptions following diffuse brain injury in swine. Epilepsy Currents. 2014;14:17.
1785. Wu Q, Marescaux C, Qin X, Kessler R, Yang J. Heterogeneity of radiological spectrum in tacrolimus-associated encephalopathy after

lung transplantation. Behavioural Neurology. 2014;2014. doi: 10.1155/2014/931808.

1786. Xerri C, Zennou-Azogui Y, Sadlaoud K, Sauvajon D. INTERPLAY BETWEEN INTRA- AND INTERHEMISPHERIC REMODELING OF NEURAL NETWORKS AS A SUBSTRATE OF FUNCTIONAL RECOVERY AFTER STROKE: ADAPTIVE VERSUS MALADAPTIVE REORGANIZATION. Neuroscience. 2014;283:178-201. doi: 10.1016/j.neuroscience.2014.06.066. PubMed PMID: WOS:000345513100013.

1787. Xu G, Lan Y, Zhang Q, He X. Acute modulatory effects of low frequency repetitive transcranial magnetic stimulation on the frontoparietal network. Brain Injury. 2014;28(5-6):676. doi: 10.3109/02699052.2014.892379.

1788. Yamada N, Kakuda W, Kondo T, Shimizu M, Mitani S, Abo M. Consecutive application of continuous theta burst stimulation combined with intensive occupational therapy for upper limb hemiparesis after stroke: A preliminary study. Brain Injury. 2014;28(5-6):620-1. doi: 10.3109/02699052.2014.892379.

1789. Yeung ST, Myczek K, Kang AP, Chabrier MA, Baglietto-Vargas D, Laferla FM. Impact of hippocampal neuronal ablation on neurogenesis and cognition in the aged brain. Neuroscience. 2014;259:214-22. Epub 2013/12/10. doi:

10.1016/j.neuroscience.2013.11.054. PubMed PMID: 24316470; PubMed Central PMCID: PMCPMC4438704.

1790. Yoon EJ, Kim YK, Kim HR, Kim SE, Lee Y, Shin HI. Transcranial direct current stimulation to lessen neuropathic pain after spinal cord injury: a mechanistic PET study. Neurorehabilitation and neural repair. 2014;28(3):250-9. doi: 10.1177/1545968313507632. PubMed PMID: CN-01120453.

1791. Young SJ, Bertucco M, Sanger TD. Cathodal transcranial direct current stimulation in children with dystonia: a sham-controlled study. J Child Neurol. 2014;29(2):232-9. Epub 2013/06/14. doi: 10.1177/0883073813492385. PubMed PMID: 23760989.

1792. Yu Y, Liang E, Zhang L, Zhu Z, Yan H. Effect of early rehabilitation in patients with severe traumatic brain injury. Chinese Journal of Trauma. 2014;30(5):400-3. PubMed PMID: CSCD:5162287.

1793. Zado ES, Pammer M, Marchlinski FE. First report of a shock from a subcutaneous ICD during transcutaneous electrical nerve stimulation (TENS). Heart Rhythm. 2014;11(5):S1. doi: 10.1016/j.hrthm.2014.03.025.

1794. Zanetti O. Non-pharmacological intervention for memory decline. Neurobiology of Aging. 2014;35:S26.

1795. Zhang X, Bian JS. Hydrogen sulfide: a neuromodulator and

neuroprotectant in the central nervous system. ACS Chem Neurosci. 2014;5(10):876-83. Epub 2014/09/18. doi: 10.1021/cn500185g. PubMed PMID: 25230373.
1796. Zhang X, Chen H, Liang E. Neuromodulation (cervical spinal cord stimulation, cSCS) on a comatose patient due to traumatic brain injury-One case report. Brain Injury. 2014;28(5-6):531-. PubMed PMID: WOS:000335017000039.
1797. Zhang X, Fu LD, Geng YH, Zhai X, Liu YH. Analysis of the effect of repeated-pulse transcranial magnetic stimulation at the Guangming point on electroencephalograms. Neural Regeneration Research. 2014;9(5):549-54. doi: 10.4103/1673-5374.130082.
1798. Zhou HJ, Tang T, Zhong JH, Luo JK, Cui HJ, Zhang QM, et al. Electroacupuncture improves recovery after hemorrhagic brain injury by inducing the expression of angiopoietin-1 and -2 in rats. BMC Complement Altern Med. 2014;14:127. Epub 2014/04/09. doi: 10.1186/1472-6882-14-127. PubMed PMID: 24708627; PubMed Central PMCID: PMCPMC4012070.
1799. Zimerman M, Heise KF, Gerloff C, Cohen LG, Hummel FC. Disrupting the ipsilateral motor cortex interferes with training of a complex motor task in older adults. Cerebral Cortex. 2014;24(4):1030-6. doi: 10.1093/cercor/bhs385.
1800. Abdullah JM, Idris Z, Ahmad AL, Omar H. Transcranial

magnetic stimulation (TMS) for neurorehabilitation and clinical application. Brain Stimulation. 2015;8(2):413.

1801. Agashe S, Foreman J. The Efficacy of Transcranial Magnetic Stimulation in Long Term Rehabilitation Following Traumatic Brain Injury - A Case Report. Annals of Neurology. 2015;78:S44-S. PubMed PMID: WOS:000362668600096.

1802. Alústiza I, Pujol N, Molero P, Ortuño F. Temporal processing in schizophrenia: Review. Schizophrenia Research: Cognition. 2015;2(4):185-8. doi: 10.1016/j.scog.2015.10.001.

1803. Amen DG, Raji CA, Willeumier K, Taylor D, Tarzwell R, Newberg A, et al. Functional Neuroimaging Distinguishes Posttraumatic Stress Disorder from Traumatic Brain Injury in Focused and Large Community Datasets. Plos One. 2015;10(7). doi: 10.1371/journal.pone.0129659. PubMed PMID: WOS:000358153000031.

1804. Ayata C, Lauritzen M. Spreading depression, spreading depolarizations, and the cerebral vasculature. Physiological Reviews. 2015;95(3):953-93. doi: 10.1152/physrev.00027.2014.

1805. Bhaskaran A, Barry MAT, I. Al Raisi S, Chik W, Nguyen DT, Pouliopoulos J, et al. Magnetic guidance versus manual control: comparison of radiofrequency lesion dimensions and evaluation of the effect of heart wall motion in a myocardial phantom. Journal of

Interventional Cardiac Electrophysiology. 2015;44(1):1-8. doi: 10.1007/s10840-015-0023-3.
1806. Blume C, del Giudice R, Wislowska M, Lechinger J, Schabus M. Across the consciousness continuum—from unresponsive wakefulness to sleep. Frontiers in Human Neuroscience. 2015;9(MAR). doi: 10.3389/fnhum.2015.00105.
1807. Bodart O, Wannez S, Thibaut A, Annen J, Casali AG, Casarotto S, et al. Multimodal TMS/EEG, FDG-PET and active fMRI assessments in patients with disorders of consciousness and locked-in syndrome. European Journal of Neurology. 2015;22:102-3. PubMed PMID: WOS:000357033500163.
1808. Bogdanova Y, Gilbert KS, Kark S, Ho VT, Yee MK, Brown TA, et al. Sleep problems, treatment and recovery in veterans with blast exposure, TBI and PTSD. Archives of physical medicine and rehabilitation. 2015;96(10):e3-e4. PubMed PMID: CN-01164070.
1809. Bogdanova Y, Martin P, Ho M, Yee M, Ho V, Knight J, et al. Improved sleep and cognition after transcranial or intranasal, red/near-infrared led treatment in chronic TBI: Pilot case series. Journal of Head Trauma Rehabilitation. 2015;30(3):E101-E2. doi: 10.1097/HTR.0000000000000150.
1810. Bolognini N, Russo C, Vallar G. Crossmodal illusions in neurorehabilitation. Frontiers in Behavioral Neuroscience.

2015;9(AUGUST). doi: 10.3389/fnbeh.2015.00212.

1811. Bolu A, Balikci A, Erdem M, Oznur T, Celik C, Uzun O. Cortical Excitability and Agressive Behavior in Post-Traumatic Stress Disorder. Noropsikiyatri Arsivi-Archives of Neuropsychiatry. 2015;52(1):73-7. doi: 10.5152/npa.2015.7031. PubMed PMID: WOS:000351851100015.

1812. Broeer S, Loescher W. Novel combinations of phenotypic biomarkers predict development of epilepsy in the lithium-pilocarpine model of temporal lobe epilepsy in rats. Epilepsy & Behavior. 2015;53:98-107. doi: 10.1016/j.yebeh.2015.09.028. PubMed PMID: WOS:000366328200017.

1813. Burns N, Bavishi S, Bockbrader M, Basobas B, Nielsen AV. Transcranial direct current stimulation for minimally conscious state: a case report. PM and r. 2015;7(9):S110-. PubMed PMID: CN-01106589.

1814. Burnstock G. Physiopathological roles of P2X receptors in the central nervous system. Current Medicinal Chemistry. 2015;22(7):819-44. doi: 10.2174/0929867321666140706130415.

1815. Büyükçoban S, Arıcı MA, Koca U, Kalkan Ş. A Case Report of Toxic Brain Syndrome Caused by Methyl Bromide. Turk J Anaesthesiol Reanim. 2015;43(2):134-7. Epub 2015/04/01. doi: 10.5152/tjar.2014.84756. PubMed PMID: 27366483; PubMed Central

PMCID: PMCPMC4917157.
1816. Calzolari E, Bolognini N, Casati C, Marzoli SB, Vallar G. Restoring abnormal aftereffects of prismatic adaptation through neuromodulation. Neuropsychologia. 2015;74:162-9. Epub 2015/04/29. doi: 10.1016/j.neuropsychologia.2015.04.022. PubMed PMID: 25912762.
1817. Carman AJ, Ferguson R, Cantu R, Comstock RD, Dacks PA, Dekosky ST, et al. Expert consensus document: Mind the gaps-advancing research into short-term and long-term neuropsychological outcomes of youth sports-related concussions. Nature Reviews Neurology. 2015;11(4):230-44. doi: 10.1038/nrneurol.2015.30.
1818. Chaieb L, Antal A, Masurat F, Paulus W. Neuroplastic effects of transcranial near-infrared stimulation (tNIRS) on the motor cortex. Front Behav Neurosci. 2015;9:147. Epub 2015/06/18. doi: 10.3389/fnbeh.2015.00147. PubMed PMID: 26082699; PubMed Central PMCID: PMCPMC4451368.
1819. Chang S, Ranjan M, Slotty PJ, Honey CR. The Influence of Positioning and Muscle Activity on Motor Threshold during Motor Cortex Stimulation Programming. Stereotactic and Functional Neurosurgery. 2015;93(2):122-6. doi: 10.1159/000369355. PubMed PMID: WOS:000351486500007.

1820. Chantsoulis M, Mirski A, Rasmus A, Kropotov JD, Pachalska M. Neuropsychological rehabilitation for traumatic brain injury patients. Annals of Agricultural and Environmental Medicine. 2015;22(2):368-79. doi: 10.5604/12321966.1152097. PubMed PMID: WOS:000356635800036.

1821. Chen GS, Huang KF, Huang CC, Wang JY. Thaliporphine derivative improves acute lung injury after traumatic brain injury. BioMed Research International. 2015;2015. doi: 10.1155/2015/729831.

1822. Chen M-C, Ling M-H, Kusuma SJ. Poly-gamma-glutamic acid microneedles with a supporting structure design as a potential tool for transdermal delivery of insulin. Acta Biomaterialia. 2015;24:106-16. doi: 10.1016/j.actbio.2015.06.021. PubMed PMID: WOS:000360867700011.

1823. Chen Y, Kosmas P, Anwar PS, Huang L. A Touch-Communication Framework for Drug Delivery Based on a Transient Microbot System. Ieee Transactions on Nanobioscience. 2015;14(4):397-408. doi: 10.1109/tnb.2015.2395539. PubMed PMID: WOS:000359565800009.

1824. Chi CI. The improvement of the episodic memory in the older adults and its cognitive neural mechanism. http://wwwchictrorgcn/showprojaspx?proj=11410. 2015. PubMed

PMID: CN-01308112.
1825. Chung MG, Lo WD. Noninvasive brain stimulation: the potential for use in the rehabilitation of pediatric acquired brain injury. Arch Phys Med Rehabil. 2015;96(4 Suppl):S129-37. Epub 2014/12/03. doi: 10.1016/j.apmr.2014.10.013. PubMed PMID: 25448248.
1826. Cincotta M, Giovannelli F, Chiaramonti R, Bianco G, Godone M, Battista D, et al. No effects of 20 Hz-rTMS of the primary motor cortex in vegetative state: A randomised, sham-controlled study. Cortex. 2015;71:368-76. Epub 2015/08/25. doi: 10.1016/j.cortex.2015.07.027. PubMed PMID: 26301875.
1827. Cipollari S, Veniero D, Razzano C, Caltagirone C, Koch G, Marangolo P. Combining TMS-EEG with transcranial direct current stimulation language treatment in aphasia. Expert Review of Neurotherapeutics. 2015;15(7):833-45. doi: 10.1586/14737175.2015.1049998.
1828. Corbett D, Jeffers M, Nguemeni C, Gomez-Smith M, Livingston-Thomas J. Lost in translation: rethinking approaches to stroke recovery. In: Dancause N, Nadeau S, Rossignol S, editors. Sensorimotor Rehabilitation at the Crossroads of Basic and Clinical Sciences. Progress in Brain Research. 2182015. p. 413-34.
1829. Costa V, Lugert S, Jagasia R. Role of adult hippocampal neurogenesis in cognition in physiology and disease: pharmacological

targets and biomarkers. Handbook of Experimental Pharmacology2015. p. 99-155.

1830. Culling B, Dahlgren N, Dillow J, Weinstein A. Neuromodulation for chronic bilateral occipital Neuralgia; a common technique with a not so common outcome. Regional Anesthesia and Pain Medicine. 2015;40(5).

1831. Da Silva EU, Nilton Jr LA, Veiga JCE, Silva JMDA, De Souza HC. Use of tDCS as motor cortex stimulation predictor for gait disorders in advanced Parkinson's disease. Movement Disorders. 2015;30:S78. doi: 10.1002/mds.26295.

1832. Da Silva EU, Nilton Jr LA, Veiga JCE, Silva JMDA, De Souza HC. Motor cortex stimulation for gait disorders in advanced Parkinson's disease. Movement Disorders. 2015;30:S214-S5. doi: 10.1002/mds.26295.

1833. Danilov Y, Kaczmarek K, Skinner K, Tyler M. Frontiers in Neuroengineering

Cranial Nerve Noninvasive Neuromodulation: New Approach to Neurorehabilitation. In: Kobeissy FH, editor. Brain Neurotrauma: Molecular, Neuropsychological, and Rehabilitation Aspects. Boca Raton (FL): CRC Press/Taylor & Francis

© 2015 by Taylor & Francis Group, LLC.; 2015.

1834. Danilov Y, Verbny Y, Kaczmarek K, Skinner K, Tyler M. Eye

movement rehabilitation by CN-ninm intervention: A set of case studies. Journal of Head Trauma Rehabilitation. 2015;30(3):E77. doi: 10.1097/HTR.0000000000000150.
1835. Defrin R, Riabinin M, Feingold Y, Schreiber S, Pick CG. Deficient pain modulatory systems in patients with mild traumatic brain and chronic post-traumatic headache: Implications for its mechanism. Journal of Neurotrauma. 2015;32(1):28-37. doi: 10.1089/neu.2014.3359.
1836. Dhaliwal SK, Meek BP, Modirrousta MM. Non-Invasive Brain Stimulation for the Treatment of Symptoms Following Traumatic Brain Injury. Front Psychiatry. 2015;6:119. Epub 2015/09/18. doi: 10.3389/fpsyt.2015.00119. PubMed PMID: 26379560; PubMed Central PMCID: PMCPMC4549551.
1837. Dudakov J, Wertheimer T, Velardi E, Brede C, Young L, Ginsberg M, et al. Endothelial cells promote endogenous thymic regeneration via BMP4 signaling and activation of Foxn1 (HEM7P.224). Journal of Immunology. 2015;194(1).
1838. Durka PJ, Malinowska U, Zieleniewska M, O'Reilly C, Różański PT, Żygierewicz J. Spindles in Svarog: Framework and software for parametrization of EEG transients. Frontiers in Human Neuroscience. 2015;9(MAY). doi: 10.3389/fnhum.2015.00258.
1839. Eghtesadi M, Boudreau G, Leroux E, Fournier Gosselin MP.

Peripheral neuromodulation for headache attributed to an upper cervical radiculopathy. Cephalalgia. 2015;35(6):128. doi: 10.1177/0333102415581304.
1840. Endisch C, Storm C, Ploner CJ, Leithner C. Somatosensory evoked high-frequency oscillations and prognostication after cardiac arrest. Critical Care. 2015;19:S151. doi: 10.1186/cc14511.
1841. Espahbodi M, Sweeney AD, Lennon KJ, Wanna GB. Facial nerve stimulation associated with cochlear implant use following temporal bone fractures. Am J Otolaryngol. 2015;36(4):578-82. Epub 2015/05/02. doi: 10.1016/j.amjoto.2015.04.003. PubMed PMID: 25929977.
1842. Fecteau S, Dickler M, Pelayo R, Kumru H, Bernabeu M, Opisso Salleras E, et al. Cortical Excitability During Passive Action Observation in Hospitalized Adults With Subacute Moderate to Severe Traumatic Brain Injury: A Preliminary TMS Study. Neurorehabil Neural Repair. 2015;29(6):548-56. Epub 2014/12/17. doi: 10.1177/1545968314558603. PubMed PMID: 25505219; PubMed Central PMCID: PMCPMC4465410.
1843. Fecteau S, Dickler M, Pelayo R, Kumru H, Bernabeu M, Opisso Salleras E, et al. Cortical Excitability during Passive Action Observation in Hospitalized Adults with Subacute Moderate to Severe Traumatic Brain Injury. Neurorehabilitation and Neural Repair.

2015;29(6):548-56. doi: 10.1177/1545968314558603.

1844. Fins JJ. Nanotechnology, neuromodulation & the immune response: discourse, materiality & ethics. Biomed Microdevices. 2015;17(2):28. Epub 2015/02/15. doi: 10.1007/s10544-015-9934-0. PubMed PMID: 25681046.

1845. Fischer DB, Truog RD. What is a reflex? A guide for understanding disorders of consciousness. Neurology. 2015;85(6):543-8. doi: 10.1212/WNL.0000000000001748.

1846. Ford JA, Orfila JE, Dietz RM, Yang Y, Haeger S, Herson PS, et al. The mechanistic investigation of neurocognitive dysfunction after sepsis. American Journal of Respiratory and Critical Care Medicine. 2015;191.

1847. Fregni F, Nitsche MA, Loo CK, Brunoni AR, Marangolo P, Leite J, et al. Regulatory considerations for the clinical and research use of transcranial direct current stimulation (tDCS): Review and recommendations from an expert panel. Clinical Research and Regulatory Affairs. 2015;32(1):22-35. doi: 10.3109/10601333.2015.980944.

1848. Fritz JV, McVige J, Mechtler L. Coding in behavioral neurology and neuropsychiatry. CONTINUUM Lifelong Learning in Neurology. 2015;21(3):844-60. doi: 10.1212/01.CON.0000466672.94853.19.

1849. Fuente A. The olivocochlear system and protection from acoustic trauma: A mini literature review. Frontiers in Systems Neuroscience. 2015;9(JUNE). doi: 10.3389/fnsys.2015.00094.

1850. Gentile A, Mori F, Bernardini S, Centonze D. Role of amyloid-β CSF levels in cognitive deficit in MS. Clinica Chimica Acta. 2015;449:23-30. doi: 10.1016/j.cca.2015.01.035.

1851. Gertler P, Tate RL, Cameron ID. Non-pharmacological interventions for depression in adults and children with traumatic brain injury. Cochrane Database Syst Rev. 2015;2015(12):Cd009871. Epub 2015/12/15. doi: 10.1002/14651858.CD009871.pub2. PubMed PMID: 26663136; PubMed Central PMCID: PMCPMC8761477.

1852. Gilbert KS, Kark SM, Gehrman P, Bogdanova Y. Sleep disturbances, TBI and PTSD: Implications for treatment and recovery. Clin Psychol Rev. 2015;40:195-212. Epub 2015/07/15. doi: 10.1016/j.cpr.2015.05.008. PubMed PMID: 26164549; PubMed Central PMCID: PMCPMC5153364.

1853. Gillick BT, Rich T, Chen M, Meekins GD. Case report of vasovagal syncope associated with single pulse transcranial magnetic stimulation in a healthy adult participant. BMC Neurol. 2015;15:248. Epub 2015/12/03. doi: 10.1186/s12883-015-0510-2. PubMed PMID: 26627895; PubMed Central PMCID: PMCPMC4666029.

1854. Goldsworthy MR, Pitcher JB, Ridding MC. Spaced

Noninvasive Brain Stimulation: Prospects for Inducing Long-Lasting Human Cortical Plasticity. Neurorehabilitation and Neural Repair. 2015;29(8):714-21. doi: 10.1177/1545968314562649.

1855. Goldwasser T, Bressan S, Oakley E, Arpone M, Babl FE. Use of sedation in children receiving computed tomography after head injuries. Eur J Emerg Med. 2015;22(6):413-8. Epub 2014/09/16. doi: 10.1097/mej.0000000000000201. PubMed PMID: 25222427.

1856. Gosseries O, Sarasso S, Casarotto S, Boly M, Schnakers C, Napolitani M, et al. On the cerebral origin of EEG responses to TMS: insights from severe cortical lesions. Brain Stimul. 2015;8(1):142-9. Epub 2014/12/08. doi: 10.1016/j.brs.2014.10.008. PubMed PMID: 25481074.

1857. Guerriero RM, Giza CC, Rotenberg A. Glutamate and GABA Imbalance Following Traumatic Brain Injury. Current Neurology and Neuroscience Reports. 2015;15(5). doi: 10.1007/s11910-015-0545-1. PubMed PMID: WOS:000352317600009.

1858. Guffens F, Maes A, Van Laere K, Goffin K. Dynamic O-(2-18F-Fluoroethyl)-l-Tyrosine PET improves differentiation of local recurrent brain metastasis from radiation necrosis. Journal of Nuclear Medicine. 2015;56.

1859. Gummadavelli A, Kundishora AJ, Willie JT, Andrews JP, Gerrard JL, Spencer DD, et al. Neurostimulation to improve level of

consciousness in patients with epilepsy. Neurosurg Focus. 2015;38(6):E10. Epub 2015/06/02. doi: 10.3171/2015.3.Focus1535. PubMed PMID: 26030698; PubMed Central PMCID: PMCPMC6300138.

1860. Guo T, Li H, Lv Y, Lu H, Niu J, Sun J, et al. Pulsed Transcranial Ultrasound Stimulation Immediately After The Ischemic Brain Injury is Neuroprotective. IEEE Trans Biomed Eng. 2015;62(10):2352-7. Epub 2015/05/03. doi: 10.1109/tbme.2015.2427339. PubMed PMID: 25935023.

1861. Hameed MQ, Hsieh TH, Morales-Quezada JL, Goodrich GS, Rotenberg A. Ceftriaxone treatment in a rat post traumatic epilepsy model preserves cortical inhibitory interneuron function and parvalbumin expression. Epilepsy Currents. 2015;15:393.

1862. Heise CW, Beutler D, Bosak A, Orme G, Loli A, Graeme K. Massive Atenolol, Lisinopril, and Chlorthalidone Overdose Treated with Endoscopic Decontamination, Hemodialysis, Impella Percutaneous Left Ventricular Assist Device, and ECMO. Journal of Medical Toxicology. 2015;11(1):110-4. doi: 10.1007/s13181-014-0419-y.

1863. Hilz MJ, Aurnhammer F, Flanagan SR, Intravooth T, Wang R, Hösl KM, et al. Eyeball Pressure Stimulation Unveils Subtle Autonomic Cardiovascular Dysfunction in Persons with a History of

Mild Traumatic Brain Injury. Journal of Neurotrauma. 2015;32(22):1796-804. doi: 10.1089/neu.2014.3842.
1864. Ille S, Sollmann N, Hauck T, Maurer S, Tanigawa N, Obermueller T, et al. Impairment of non-invasive language mapping by lesion location a fMRI, rTMS, and DCS study. Clinical Neurophysiology. 2015;126(8):e130. doi: 10.1016/j.clinph.2015.04.205.
1865. Ille S, Wittig R, Kulchytska N, Sollmann N, Hauck T, Maurer S, et al. Correlation of hemispheric dominance of language examined by rTMS and postoperative course of language function in patients with left-sided Perisylvian brains lesions. Clinical Neurophysiology. 2015;126(8):e129-e30. doi: 10.1016/j.clinph.2015.04.204.
1866. Irct2014031611234N. Cumulative effects of transcranial direct current stimulation on meth craving. https://trialsearchwhoint/Trial2aspx?TrialID=IRCT2014031611234N2. 2015. PubMed PMID: CN-01804843.
1867. Isagulyan ED, Tomsky AA, Dekopov AV, Salova EM, Troshina EM, Dorokhov EV, et al. Results of motor cortex stimulation in the treatment of chronic pain syndromes. Zh Vopr Neirokhir Im N N Burdenko. 2015;79(6):46-60. Epub 2015/01/01. doi: 10.17116/neiro201579646-60. PubMed PMID: 26977794.
1868. Jedlicka S, Ott S, Krammer M, Lumenta CB. The value of

navigated transcranial magnetic stimulation in clinical neurosurgical practice. Brain Stimulation. 2015;8(2):428.

1869. Ji G, Liu M, Zhao XF, Liu XY, Guo QL, Guan ZF, et al. NF-κB Signaling is Involved in the Effects of Intranasally Engrafted Human Neural Stem Cells on Neurofunctional Improvements in Neonatal Rat Hypoxic-Ischemic Encephalopathy. CNS Neuroscience and Therapeutics. 2015;21(12):926-35. doi: 10.1111/cns.12441.

1870. Johnen VM, Neubert F-X, Buch ER, Verhagen LM, O'Reilly J, Mars RB, et al. Causal manipulation of functional connectivity in a specific neural pathway during behaviour and at rest. Elife. 2015;4. doi: 10.7554/eLife.04585. PubMed PMID: WOS:000349460700002.

1871. Jprn U. Acupuncture treatment for patients with chronic disorder of consciousness and severe cognitive disorder (higher brain dysfunction) following traffic traumatic brain injury. https://trialsearchwhoint/Trial2aspx?TrialID=JPRN-UMIN000018797. 2015. PubMed PMID: CN-01881418.

1872. Karuppagounder SS, Kumar A, Shao DS, Zille M, Bourassa MW, Caulfield JT, et al. Metabolism and epigenetics in the nervous system: Creating cellular fitness and resistance to neuronal death in neurological conditions via modulation of oxygen-, iron-, and 2-oxoglutarate-dependent dioxygenases. Brain Research. 2015;1628:273-87. doi: 10.1016/j.brainres.2015.07.030.

1873. Ketelslegers IA, Van Pelt ED, Bryde S, Neuteboom RF, Catsman-Berrevoets CE, Hamann D, et al. Anti-MOG antibodies plead against MS diagnosis in an Acquired Demyelinating Syndromes cohort. Multiple Sclerosis. 2015;21(12):1513-20. doi: 10.1177/1352458514566666.

1874. Kim SK, Nam SJ, Jang H, Kim A, Lee JJ. TM-MC: A database of medicinal materials and chemical compounds in Northeast Asian traditional medicine. BMC Complementary and Alternative Medicine. 2015;15(1). doi: 10.1186/s12906-015-0758-5.

1875. Kopachka MM, Sharova EV, Troshina EM, Oknina LB, Romanov AC, Chelyapina MV, et al. Repetitive transcranial magnetic stimulation (RTMS)-induced asymmetric changes in acoustic event-related potentials (AERP) in normal subjects and in patients experiencing posttraumatic unconsciousness states. Asimmetria - Journal of Asymmetry. 2015;9(2):18-29.

1876. Kornfeld S, Rodríguez JAD, Everts R, Kaelin-Lang A, Wiest R, Weisstanner C, et al. Cortical reorganisation of cerebral networks after childhood stroke: Impact on outcome. BMC Neurology. 2015;15(1). doi: 10.1186/s12883-015-0309-1.

1877. Koski L, Kolivakis T, Yu C, Chen JK, Delaney S, Ptito A. Noninvasive brain stimulation for persistent postconcussion symptoms in mild traumatic brain injury. J Neurotrauma. 2015;32(1):38-44. Epub

2014/06/24. doi: 10.1089/neu.2014.3449. PubMed PMID: 24955920.

1878. Ksiazek-Winiarek DJ, Szpakowski P, Glabinski A. Neural Plasticity in Multiple Sclerosis: The Functional and Molecular Background. Neural Plasticity. 2015;2015. doi: 10.1155/2015/307175.

1879. Làdavas E, Giulietti S, Avenanti A, Bertini C, Lorenzini E, Quinquinio C, et al. A-tDCS on the ipsilesional parietal cortex boosts the effects of prism adaptation treatment in neglect. Restorative Neurology and Neuroscience. 2015;33(5):647-62. doi: 10.3233/RNN-140464.

1880. Lancioni GE, Singh NN, O'Reilly MF, Sigafoos J, D'Amico F, Buonocunto F, et al. Assistive technology to help persons in a minimally conscious state develop responding and stimulation control: Performance assessment and social rating. Neurorehabilitation. 2015;37(3):393-403. doi: 10.3233/nre-151269. PubMed PMID: WOS:000366324100006.

1881. Lee A, Chang WH, Lee MJ, Kim MS, Kim YH. Dual-mode noninvasive brain stimulation over prefrontal cortices on working memory in stroke patients. Brain Stimulation. 2015;8(2):359.

1882. Lee DJ, Gurkoff GG, Izadi A, Seidl SE, Echeverri A, Melnik M, et al. Septohippocampal Neuromodulation Improves Cognition after Traumatic Brain Injury. J Neurotrauma. 2015;32(22):1822-32. Epub 2015/06/23. doi: 10.1089/neu.2014.3744. PubMed PMID:

26096267; PubMed Central PMCID: PMCPMC4702430.

1883. Lee IC, He J-S, Tsai M-T, Lin K-C. Fabrication of a novel partially dissolving polymer microneedle patch for transdermal drug delivery. Journal of Materials Chemistry B. 2015;3(2):276-85. doi: 10.1039/c4tb01555j. PubMed PMID: WOS:000346061000014.

1884. Lefebvre G, Tremblay S, Théoret H. Probing the effects of mild traumatic brain injury with transcranial magnetic stimulation of the primary motor cortex. Brain Inj. 2015;29(9):1032-43. Epub 2015/05/23. doi: 10.3109/02699052.2015.1028447. PubMed PMID: 25996466.

1885. Lefebvre J, Murray MM. Shaping pathological cortical dynamics with high-frequency neurostimulation. BMC Neuroscience. 2015;16.

1886. Lei J, Wang L, Gao G, Cooper E, Jiang J. Right Median Nerve Electrical Stimulation for Acute Traumatic Coma Patients. J Neurotrauma. 2015;32(20):1584-9. Epub 2015/02/11. doi: 10.1089/neu.2014.3768. PubMed PMID: 25664378.

1887. Lemmens S, Brône B, Dooley D, Hendrix S, Geurts N. Alpha-adrenoceptor modulation in central nervous system trauma: pain, spasms, and paralysis--an unlucky triad. Med Res Rev. 2015;35(4):653-77. Epub 2014/12/30. doi: 10.1002/med.21337. PubMed PMID: 25546087.

1888. Lemmens S, Brone B, Dooley D, Hendrix S, Geurts N. Alpha-Adrenoceptor Modulation in Central Nervous System Trauma: Pain, Spasms, and Paralysis - An Unlucky Triad. Medicinal Research Reviews. 2015;35(4):653-77. doi: 10.1002/med.21337. PubMed PMID: WOS:000356068800001.

1889. Leong SR, Liang WC, Wu Y, Crocker L, Cheng E, Sampath D, et al. An Anti-B7-H4 antibody-drug conjugate for the treatment of breast cancer. Molecular Pharmaceutics. 2015;12(6):1717-29. doi: 10.1021/mp5007745.

1890. Li LM, Uehara K, Hanakawa T. The contribution of interindividual factors to variability of response in transcranial direct current stimulation studies. Frontiers in Cellular Neuroscience. 2015;9. doi: 10.3389/fncel.2015.00181. PubMed PMID: WOS:000354803600003.

1891. Li S, Zaninotto AL, Neville IS, Paiva WS, Nunn D, Fregni F. Clinical utility of brain stimulation modalities following traumatic brain injury: current evidence. Neuropsychiatr Dis Treat. 2015;11:1573-86. Epub 2015/07/15. doi: 10.2147/ndt.S65816. PubMed PMID: 26170670; PubMed Central PMCID: PMCPMC4494620.

1892. Liamlahi R, Hagmann C, Beck I, Latal B, Knirsch W. Delayed brain maturation correlates with motor impairment at one year of age

in children with severe congenital heart disease. Cardiology in the Young. 2015;25:S27. doi: 10.1017/S1047951115000529.

1893. Liao Z, Pu T, Gu H, Liu W. Coupled motion of cervical spine in three level hybrid constructs. Bio-Medical Materials and Engineering. 2015;26:S637-S45. doi: 10.3233/BME-151355.

1894. Lin SC, Brown RE, Shuler MGH, Petersen CCH, Kepecs A. Optogenetic dissection of the basal forebrain neuromodulatory control of cortical activation, plasticity, and cognition. Journal of Neuroscience. 2015;35(41):13896-903. doi: 10.1523/JNEUROSCI.2590-15.2015.

1895. Litvinenko IV, Odinak MM, Emelin AY, Vorobyev SV, Yurin AA. Role of transcranial magnetic stimulation in diagnostics of pathophysiological mechanisms of cognitive impairment in traumatic brain injury. Bulletin of the Russian Military Medical Academy. 2015(3):249-54. PubMed PMID: RSCI:25808282.

1896. Liu CY, Law M, Romano R. Rational approach to understanding and preventing sports-related traumatic brain injuries. World Neurosurgery. 2015;84(6):1556-7. doi: 10.1016/j.wneu.2015.07.076.

1897. Liu J, Zhao F, Wu J, Wu S, Yang A, Li X, et al. Features of survived casualties and treatment strategies in Kunshan August 2" explosion aaccident. Chinese Journal of Emergency Medicine.

2015;24(9):952-7. PubMed PMID: CSCD:5510601.
1898. Liu Z, Lee Y, Jang JH, Li Y, Han X, Yokoi K, et al. Microfluidic cytometric analysis of cancer cell transportability and invasiveness. Scientific Reports. 2015;5. doi: 10.1038/srep14272.
1899. Lõo S, Kuusela L, Valanne L, Vaalto S, Nevalainen P, Lauronen L, et al. Structural MRI, transcranial magnetic stimulation, magnetoencephalography and DTI tractography findings in relation to sensorimotor outcome after perinatal stroke. European Journal of Paediatric Neurology. 2015;19:S9-S10.
1900. Lorente L, Martín MM, Almeida T, Abreu-González P, Ramos L, Argueso M, et al. Total antioxidant capacity is associated with mortality of patients with severe traumatic brain injury. BMC Neurology. 2015;15(1). doi: 10.1186/s12883-015-0378-1.
1901. Lu H, Kobilo T, Robertson C, Tong S, Celnik P, Pelled G. Transcranial magnetic stimulation facilitates neurorehabilitation after pediatric traumatic brain injury. Sci Rep. 2015;5:14769. Epub 2015/10/07. doi: 10.1038/srep14769. PubMed PMID: 26440604; PubMed Central PMCID: PMCPMC4594036.
1902. Luauté J, Morlet D, Mattout J. BCI in patients with disorders of consciousness: Clinical perspectives. Annals of Physical and Rehabilitation Medicine. 2015;58(1):29-34. doi: 10.1016/j.rehab.2014.09.015.

1903. Major BP, Rogers MA, Pearce AJ. Using transcranial magnetic stimulation to quantify electrophysiological changes following concussive brain injury: a systematic review. Clin Exp Pharmacol Physiol. 2015;42(4):394-405. Epub 2015/01/22. doi: 10.1111/1440-1681.12363. PubMed PMID: 25603731.

1904. Marek Gołe'biewski W, Cyrta M, Michalczyk A. Synthesis and fungistatic activity of aryl aldoxime derivatives. Journal de Mycologie Medicale. 2015;25(1):50-6. doi: 10.1016/j.mycmed.2014.10.026.

1905. Marins TF, Rodrigues EC, Engel A, Hoefle S, Basílio R, Lent R, et al. Enhancing motor network activity using real-time functional mri neurofeedback of left premotor cortex. Frontiers in Behavioral Neuroscience. 2015;9(DEC). doi: 10.3389/fnbeh.2015.00341.

1906. Martial C, Thibaut A, Bruno MA, Wannez S, Chatelle C, Anne-Francoise D, et al. Controlled clinical trial of repeated left prefrontal tDCS in patients with chronic minimally conscious state. European journal of neurology. 2015;22:408-9. doi: 10.1111/ene.12807. PubMed PMID: CN-01086684.

1907. Miller JP, Sweet JA, Bailey CM, Munyon CN, Luders HO, Fastenau PS. Visual-spatial memory may be enhanced with theta burst deep brain stimulation of the fornix: a preliminary investigation with four cases. Brain. 2015;138(Pt 7):1833-42. Epub 2015/06/25. doi:

10.1093/brain/awv095. PubMed PMID: 26106097.

1908. Morey RA, Lancaster SC, Haswell CC. Trauma Re-experiencing Symptoms Modulate Topology of Intrinsic Functional Networks. Biological Psychiatry. 2015;78(3):156-8. doi: 10.1016/j.biopsych.2015.06.001. PubMed PMID: WOS:000357489000004.

1909. Morries LD, Cassano P, Henderson TA. Treatments for traumatic brain injury with emphasis on transcranial near-infrared laser phototherapy. Neuropsychiatric Disease and Treatment. 2015;11:2159-75. doi: 10.2147/NDT.S65809.

1910. Muralidharan P, Malapit M, Mallory E, Hayes D, Jr., Mansour HM. Inhalable nanoparticulate powders for respiratory delivery. Nanomedicine-Nanotechnology Biology and Medicine. 2015;11(5):1189-99. doi: 10.1016/j.nano.2015.01.007. PubMed PMID: WOS:000363967100016.

1911. Nantes JC, Zhong J, Holmes SA, Narayanan S, Lapierre Y, Koski L. Intracortical inhibition in multiple sclerosis: Relation to clinical subtype, motor disability, and cortical damage. Multiple Sclerosis. 2015;23(11):223. doi: 10.1177/1352458515602642.

1912. Nardone A, Sampson E, Stauffer C, Leopold A, Jacobs K, Hendricks DJ, et al. Project Career: A qualitative examination of five college students with traumatic brain injuries. NeuroRehabilitation.

2015;37(3):459-69. Epub 2015/11/01. doi: 10.3233/nre-151274. PubMed PMID: 26518535.

1913. Naro A, Calabrò RS, Russo M, Leo A, Pollicino P, Quartarone A, et al. Can transcranial direct current stimulation be useful in differentiating unresponsive wakefulness syndrome from minimally conscious state patients? Restor Neurol Neurosci. 2015;33(2):159-76. Epub 2015/01/16. doi: 10.3233/rnn-140448. PubMed PMID: 25588461.

1914. Naro A, Leo A, Bramanti P, Calabrò RS. Moving Toward Conscious Pain Processing Detection in Chronic Disorders of Consciousness: Anterior Cingulate Cortex Neuromodulation. J Pain. 2015;16(10):1022-31. Epub 2015/07/26. doi: 10.1016/j.jpain.2015.06.014. PubMed PMID: 26208761.

1915. Naro A, Leo A, Cannavò A, Buda A, Bruno R, Salviera C, et al. Audiomotor Integration in Minimally Conscious State: Proof of Concept! Neural Plast. 2015;2015:391349. Epub 2015/10/02. doi: 10.1155/2015/391349. PubMed PMID: 26425370; PubMed Central PMCID: PMCPMC4573993.

1916. Naro A, Leo A, Filoni S, Bramanti P, Calabrò RS. Visuo-motor integration in unresponsive wakefulness syndrome: A piece of the puzzle towards consciousness detection? Restor Neurol Neurosci. 2015;33(4):447-60. Epub 2015/09/28. doi:

10.3233/rnn-150525. PubMed PMID: 26409404; PubMed Central PMCID: PMCPMC4923741.

1917. Naro A, Leo A, Russo M, Quartarone A, Bramanti P, Calabrò RS. Shaping thalamo-cortical plasticity: a marker of cortical pain integration in patients with post-anoxic unresponsive wakefulness syndrome? Brain Stimul. 2015;8(1):97-104. Epub 2014/09/28. doi: 10.1016/j.brs.2014.09.001. PubMed PMID: 25260422.

1918. Naro A, Pisani LR, Leo A, Molonia F, Bramanti P, Calabrò RS. Treatment of refractory generalized status epilepticus in a patient with unresponsive wakefulness syndrome: Is neuromodulation the future? Epilepsy Behav. 2015;50:96-7. Epub 2015/07/27. doi: 10.1016/j.yebeh.2015.06.038. PubMed PMID: 26209943.

1919. Naro A, Russo M, Leo A, Bramanti P, Quartarone A, Calabrò RS. A Single Session of Repetitive Transcranial Magnetic Stimulation Over the Dorsolateral Prefrontal Cortex in Patients With Unresponsive Wakefulness Syndrome: Preliminary Results. Neurorehabil Neural Repair. 2015;29(7):603-13. Epub 2014/12/30. doi: 10.1177/1545968314562114. PubMed PMID: 25539781.

1920. Nct. Asia Coma Electrical Stimulation (the ACES Trial). https://clinicaltrialsgov/show/NCT02645578. 2015. PubMed PMID: CN-01554851.

1921. Nct. LED Treatment to Improve Cognition and Promote

Recovery in TBI. https://clinicaltrialsgov/show/NCT02404402. 2015. PubMed PMID: CN-01598036.

1922. Nct. Treatment and Recovery Monitoring of Post TBI Symptoms. https://clinicaltrialsgov/show/NCT02426749. 2015. PubMed PMID: CN-01591562.

1923. Nct. Transcranial Magnetic Stimulation (TMS) to Treat mTBI and PTSD. https://clinicaltrialsgov/show/NCT02458521. 2015. PubMed PMID: CN-01590261.

1924. Nct. rTMS: A Treatment to Restore Function After Severe TBI. https://clinicaltrialsgov/show/NCT02366754. 2015. PubMed PMID: CN-01480415.

1925. Nct. Transcranial Direct Current Stimulation for the Treatment of Deficits After Traumatic Brain Injury. https://clinicaltrialsgov/show/NCT02613936. 2015. PubMed PMID: CN-01589330.

1926. Nct. Effect of Repeated tDCS Sessions on Cognitive Improvement in Patients With Disorders of Consciousness. https://clinicaltrialsgov/show/NCT02394691. 2015. PubMed PMID: CN-01597943.

1927. Nct. Fronto-parietal tDCS in Severely Brain Injured Patients With Disorders of Consciousness. https://clinicaltrialsgov/show/NCT02626403. 2015. PubMed PMID:

CN-01598970.

1928. Nct. The Consciousness Variation of Deep Brain and Transcranial Magnetic Stimulation for Disorders of Consciousness Patients. https://clinicaltrialsgov/show/NCT02667899. 2015. PubMed PMID: CN-01555416.

1929. Nct. Conditioning Neural Circuits to Improve Upper Extremity Function. https://clinicaltrialsgov/show/NCT02611375. 2015. PubMed PMID: CN-01553883.

1930. Nct. Effects of the Transcranial Stimulation on Prevention of Cognitive Dysfunction in Cardiac Surgery. https://clinicaltrialsgov/show/NCT02549560. 2015. PubMed PMID: CN-01492241.

1931. Nct. Primed vs Unprimed Facilitatory and Depressive Paired Associative Stimulation. https://clinicaltrialsgov/show/NCT02619643. 2015. PubMed PMID: CN-01589390.

1932. Neggers SFW, Petrov PI, Mandija S, Sommer IEC, van den Berg NAT. Understanding the biophysical effects of transcranial magnetic stimulation on brain tissue: The bridge between brain stimulation and cognition. In: Bestmann S, editor. Computational Neurostimulation. Progress in Brain Research. 2222015. p. 229-59.

1933. Neva JL, Vesia M, Singh AM, Staines WR. Bilateral primary motor cortex circuitry is modulated due to theta burst stimulation to

left dorsal premotor cortex and bimanual training. Brain Research. 2015;1618:61-74. doi: 10.1016/j.brainres.2015.05.028. PubMed PMID: WOS:000359957600008.

1934. Neville IS, Hayashi CY, El Hajj SA, Zaninotto AL, Sabino JP, Sousa LM, Jr., et al. Repetitive Transcranial Magnetic Stimulation (rTMS) for the cognitive rehabilitation of traumatic brain injury (TBI) victims: study protocol for a randomized controlled trial. Trials. 2015;16:440. Epub 2015/10/07. doi: 10.1186/s13063-015-0944-2. PubMed PMID: 26438108; PubMed Central PMCID: PMCPMC4594992.

1935. Ngernyam N, Jensen MP, Arayawichanon P, Auvichayapat N, Tiamkao S, Janjarasjitt S, et al. The effects of transcranial direct current stimulation in patients with neuropathic pain from spinal cord injury. Clinical neurophysiology. 2015;126(2):382-90. doi: 10.1016/j.clinph.2014.05.034. PubMed PMID: CN-01048957.

1936. Nielson DM, McKnight CA, Patel RN, Kalnin AJ, Mysiw WJ. Preliminary guidelines for safe and effective use of repetitive transcranial magnetic stimulation in moderate to severe traumatic brain injury. Arch Phys Med Rehabil. 2015;96(4 Suppl):S138-44. Epub 2014/10/05. doi: 10.1016/j.apmr.2014.09.010. PubMed PMID: 25281871.

1937. Nielson DM, McKnight CA, Patel RN, Kalnin AJ, Mysiw WJ.

Preliminary Guidelines for Safe and Effective Use of Repetitive Transcraniat Magnetic Stimulation in Moderate to Severe Traumatic Brain Injury. Archives of Physical Medicine and Rehabilitation. 2015;96(4):S138-S44. doi: 10.1016/j.apmr.2014.09.010. PubMed PMID: WOS:000352179900007.

1938. Page SJ, Cunningham DA, Plow E, Blazak B. It takes two: noninvasive brain stimulation combined with neurorehabilitation. Arch Phys Med Rehabil. 2015;96(4 Suppl):S89-93. Epub 2015/03/31. doi: 10.1016/j.apmr.2014.09.019. PubMed PMID: 25813373; PubMed Central PMCID: PMCPMC4445084.

1939. Page SJ, Cunningham DA, Plow E, Blazak B. It Takes Two: Noninvasive Brain Stimulation Combined With Neurorehabilitation INTRODUCTION. Archives of Physical Medicine and Rehabilitation. 2015;96(4):S89-S93. doi: 10.1016/j.apmr.2014.09.019. PubMed PMID: WOS:000352179900001.

1940. Paiva WS, Neville IS, Fregni F, Teixeira MJ. Is transcranial magnetic stimulation useful in posttraumatic disorders? Neural Regeneration Research. 2015;10(9):1528-. doi: 10.4103/1673-5374.165529. PubMed PMID: WOS:000362625000041.

1941. Palhano-Fontes F, Andrade KC, Tofoli LF, Jose ACS, Crippa AS, Hallak JEC, et al. The psychedelic state induced by Ayahuasca

modulates the activity and connectivity of the Default Mode Network. PLoS ONE. 2015;10(2). doi: 10.1371/journal.pone.0118143.

1942. Pathak R, Shao L, Ghosh SP, Zhou D, Boerma M, Weiler H, et al. Thrombomodulin contributes to gamma tocotrienol-mediated lethality protection and hematopoietic cell recovery in irradiated mice. PLoS ONE. 2015;10(4). doi: 10.1371/journal.pone.0122511.

1943. Pearce AJ, Hoy K, Rogers MA, Corp DT, Davies CB, Maller JJ, et al. Acute motor, neurocognitive and neurophysiological change following concussion injury in Australian amateur football. A prospective multimodal investigation. J Sci Med Sport. 2015;18(5):500-6. Epub 2014/08/12. doi: 10.1016/j.jsams.2014.07.010. PubMed PMID: 25104044.

1944. Picillo M, Moro E, Edwards M, Di Lazzaro V, Lozano AM, Fasano A. Subdural continuous theta burst stimulation of the motor cortex in essential tremor. Neurological Sciences. 2015;36(2):S190-S1. doi: 10.1007/s10072-015-2346-3.

1945. Pisani LR, Naro A, Leo A, Aricò I, Pisani F, Silvestri R, et al. Repetitive transcranial magnetic stimulation induced slow wave activity modification: A possible role in disorder of consciousness differential diagnosis? Conscious Cogn. 2015;38:1-8. Epub 2015/10/27. doi: 10.1016/j.concog.2015.09.012. PubMed PMID: 26496476.

1946. Pistarini C. Robot therapy and tDCS for recovery of movement in stroke patients. Neurorehabilitation and Neural Repair. 2015;29(7):NP4. doi: 10.1177/1545968315589320.

1947. Potter-Baker KA, Bonnett CE, Chabra P, Roelle S, Varnerin N, Cunningham DA, et al. A game of hide and seek: Is it possible to recruit more patients for NIBS studies in stroke? J Neurol Sci. 2015;358(1-2):472-4. Epub 2015/09/01. doi: 10.1016/j.jns.2015.08.030. PubMed PMID: 26320611; PubMed Central PMCID: PMCPMC4629798.

1948. Quinlan EB, Dodakian L, See J, McKenzie A, Le V, Wojnowicz M, et al. Neural Function, Injury, and Stroke Subtype Predict Treatment Gains after Stroke. Annals of Neurology. 2015;77(1):132-45. doi: 10.1002/ana.24309. PubMed PMID: WOS:000348423200012.

1949. Rabau S, Van De Heyning P. The added effect of transcranial direct current stimulation for the treatment of acute tinnitus. Brain Stimulation. 2015;8(2):410.

1950. Raji CA, Willeumier K, Taylor D, Tarzwell R, Newberg A, Henderson TA, et al. Functional neuroimaging with default mode network regions distinguishes PTSD from TBI in a military veteran population. Brain Imaging and Behavior. 2015;9(3):527-34. doi: 10.1007/s11682-015-9385-5. PubMed PMID:

WOS:000361604300013.
1951. Rao VR, Parko KL. Clinical approach to posttraumatic epilepsy. Semin Neurol. 2015;35(1):57-63. Epub 2015/02/26. doi: 10.1055/s-0035-1544239. PubMed PMID: 25714868.
1952. Reti IM, Schwarz N, Bower A, Tibbs M, Rao V. Transcranial magnetic stimulation: A potential new treatment for depression associated with traumatic brain injury. Brain Inj. 2015;29(7-8):789-97. Epub 2015/05/08. doi: 10.3109/02699052.2015.1009168. PubMed PMID: 25950260.
1953. Richardson SP, Tinaz S, Chen R. Repetitive transcranial magnetic stimulation in cervical dystonia: Effect of site and repetition in a randomized pilot trial. PLoS ONE. 2015;10(4). doi: 10.1371/journal.pone.0124937.
1954. Rodger J, Sherrard RM. Optimising repetitive transcranial magnetic stimulation for neural circuit repair following traumatic brain injury. Neural Regen Res. 2015;10(3):357-9. Epub 2015/04/17. doi: 10.4103/1673-5374.153676. PubMed PMID: 25878575; PubMed Central PMCID: PMCPMC4396089.
1955. Sánchez-Porras R, Zheng Z, Sakowitz OW. Pharmacological modulation of spreading depolarizations. Acta Neurochirurgica, Supplementum2015. p. 153-7.
1956. Sanders R, Bruno M, Bonhomme V, Rosanova M, Laureys S,

Tononi G, et al. Propofol-induced unresponsiveness is associated with loss of deterministic high density electroencephalogram responses to transcranial magnetic stimulation. Journal of Neurosurgical Anesthesiology. 2015;27(4):447. doi: 10.1097/ANA.0000000000000217.
1957. Sarasso S, Boly M, Napolitani M, Gosseries O, Charland-Verville V, Casarotto S, et al. Consciousness and Complexity during Unresponsiveness Induced by Propofol, Xenon, and Ketamine. Current Biology. 2015;25(23):3099-105. doi: 10.1016/j.cub.2015.10.014. PubMed PMID: WOS:000366388600025.
1958. Sato N, Morishita R. The roles of lipid and glucose metabolism in modulation of β-amyloid, tau, and neurodegeneration in the pathogenesis of Alzheimer disease. Frontiers in Aging Neuroscience. 2015;7(OCT). doi: 10.3389/fnagi.2015.00199.
1959. Scalone L, Zucco F, Ciampichini R, Lavano A, Costantini A, De Rose M, et al. Relationship between pain, functional disability and health-related quality of life in patient with failed back surgery syndrome undergoing spinal cord stimulation: Results from the precise study. Value in Health. 2015;18(7):A647.
1960. Schartner M, Seth A, Noirhomme Q, Boly M, Bruno M-A, Laureys S, et al. Complexity of Multi-Dimensional Spontaneous EEG Decreases during Propofol Induced General Anaesthesia. Plos One.

2015;10(8). doi: 10.1371/journal.pone.0133532. PubMed PMID: WOS:000359121100020.
1961. Schatzberg AF. Issues encountered in recent attempts to develop novel antidepressant agents. In: Fink ME, Barchas JD, Sheikh JI, editors. Affective Disorders and Traumatic Brain Injury. Annals of the New York Academy of Sciences. 13452015. p. 67-73.
1962. Seo JP, Jang SH. Traumatic axonal injury of the corticospinal tract in the subcortical white matter in patients with mild traumatic brain injury. Brain Inj. 2015;29(1):110-4. Epub 2014/10/31. doi: 10.3109/02699052.2014.973447. PubMed PMID: 25356741.
1963. Sergeeva EG, Bola M, Wagner S, Lazik S, Voigt N, Mawrin C, et al. Repetitive Transcorneal Alternating Current Stimulation Reduces Brain Idling State After Long-term Vision Loss. Brain Stimul. 2015;8(6):1065-73. Epub 2015/07/07. doi: 10.1016/j.brs.2015.06.006. PubMed PMID: 26145756.
1964. Sharma V, Kaur H, Gupta D. Right median nerve stimulation for improving consciousness: A case series. Indian Journal of Neurotrauma. 2015;12(2):144-8. doi: 10.1055/s-0035-1569473.
1965. Shyshov AS, Petrova IS, Grishina TP, Frolova KS, Blank IA, Inozemtseva AB, et al. Cerebrospinal fluid in clinical aspects of tuberculosis of the central nervous system. Zhurnal Nevrologii i Psihiatrii imeni SS Korsakova. 2015;2015(3):17-24. doi:

10.17116/jnevro20151153117-24.
1966. Sidiakina IV, Dobrushina OR, Liadov KV, Shapovalenko TV, Romashin OV. The role of evidence-based medicine in the neurorehabilitation: the innovative technologies (a review). Voprosy kurortologii, fizioterapii, i lechebnoi fizicheskoi kultury. 2015;92(3):53-6. doi: 10.17116/kurort2015353-56. PubMed PMID: MEDLINE:26285335.
1967. Simon DT, Larsson KC, Nilsson D, Burstrom G, Galter D, Berggren M, et al. An organic electronic biomimetic neuron enables auto-regulated neuromodulation. Biosensors & Bioelectronics. 2015;71:359-64. doi: 10.1016/j.bios.2015.04.058. PubMed PMID: WOS:000356646000052.
1968. Singh R, Telleria M, Patin D. Neuromodulation therapy for the treatment of refractory complex regional pain syndrome with diagonal spread. Journal of Pain. 2015;16(4):S71.
1969. Slotty PJ, Eisner W, Honey CR, Wille C, Vesper J. Long-Term Follow-Up of Motor Cortex Stimulation for Neuropathic Pain in 23 Patients. Stereotactic and Functional Neurosurgery. 2015;93(3):199-205. doi: 10.1159/000375177. PubMed PMID: WOS:000355200800008.
1970. Sokal P, Harat M, Zieliński P, Furtak J, Paczkowski D, Rusinek M. Motor cortex stimulation in patients with chronic central

pain. Adv Clin Exp Med. 2015;24(2):289-96. Epub 2015/05/02. doi: 10.17219/acem/40452. PubMed PMID: 25931362.
1971. Sollmann N, Hauck T, Ille S, Maurer S, Negwer C, Meyer B, et al. Preoperative repetitive navigated transcranial magnetic stimulation for language mapping and outcome improvement in brain tumor patients. Clinical Neurophysiology. 2015;126(8):e124. doi: 10.1016/j.clinph.2015.04.195.
1972. Sollmann N, Ille S, Hauck T, Maurer S, Negwer C, Zimmer C, et al. The impact of preoperative language mapping by repetitive navigated transcranial magnetic stimulation on the clinical course of brain tumor patients. BMC Cancer. 2015;15(1). doi: 10.1186/s12885-015-1299-5.
1973. Spagnuolo MS, Mollica MP, Maresca B, Cavaliere G, Cefaliello C, Trinchese G, et al. High fat diet and inflammation – Modulation of haptoglobin level in rat brain. Frontiers in Cellular Neuroscience. 2015;9(DEC). doi: 10.3389/fncel.2015.00479.
1974. Speth J, Frenzel C, Harley T. Transcranial direct current stimulation of areas above the motor cortex (C3) in waking resting state and REM sleep induces motor imagery. Brain Stimulation. 2015;8(2):404-5.
1975. Speth J, Speth C, Harley TA. Transcranial direct current stimulation of the motor cortex in waking resting state induces motor

imagery. Conscious Cogn. 2015;36:298-305. Epub 2015/07/24. doi: 10.1016/j.concog.2015.07.005. PubMed PMID: 26204566.

1976. Sridhar K. A summary of some of the recently published, seminal papers in Neuroscience. Neurology India. 2015;63(2):250-4. doi: 10.4103/0028-3886.156294.

1977. Storm C, Ploner CJ, Leithner C. Amplitudes of cortical somatosensory evoked potentials and outcome prediction after cardiac arrest. Critical Care. 2015;19:S153. doi: 10.1186/cc14516.

1978. Sun M-K, Nelson TJ, Alkon DL. Towards universal therapeutics for memory disorders. Trends in Pharmacological Sciences. 2015;36(6):384-94. doi: 10.1016/j.tips.2015.04.004. PubMed PMID: WOS:000356636900007.

1979. Syroezhkin FA, Nikitin NI, Dvorianchikov VV, Golovanov AE, Morozova MV, Letyagin AI. Neural plasticity in current tinnitus conception: opportunity for audial training. Bulletin of the Russian Military Medical Academy. 2015(3):94-8. PubMed PMID: RSCI:25808252.

1980. Takamiya A, Kishimoto T, Watanabe K, Mimura M, Kito S. Transcranial Magnetic Stimulation for Bipolar Disorder with Catatonic Stupor: A Case Report. Brain Stimul. 2015;8(6):1236-7. Epub 2015/10/03. doi: 10.1016/j.brs.2015.09.004. PubMed PMID: 26428351.

1981. Tang AD, Makowiecki K, Bartlett C, Rodger J. Low intensity repetitive transcranial magnetic stimulation does not induce cell survival or regeneration in a mouse optic nerve crush model. PLoS One. 2015;10(5):e0126949. Epub 2015/05/21. doi: 10.1371/journal.pone.0126949. PubMed PMID: 25993112; PubMed Central PMCID: PMCPMC4438867.

1982. Tekriwal A, Baltuch G. Deep Brain Stimulation: Expanding Applications. Neurologia Medico-Chirurgica. 2015;55(12):861-77. doi: 10.2176/nmc.ra.2015-0172. PubMed PMID: WOS:000367428900001.

1983. Thibaut A, Di Perri C, Chatelle C, Bruno MA, Bahri MA, Wannez S, et al. Clinical Response to tDCS Depends on Residual Brain Metabolism and Grey Matter Integrity in Patients With Minimally Conscious State. Brain Stimul. 2015;8(6):1116-23. Epub 2015/10/17. doi: 10.1016/j.brs.2015.07.024. PubMed PMID: 26471400.

1984. Tremblay S, Vernet M, Bashir S, Pascual-Leone A, Théoret H. Theta burst stimulation to characterize changes in brain plasticity following mild traumatic brain injury: A proof-of-principle study. Restor Neurol Neurosci. 2015;33(5):611-20. Epub 2015/03/05. doi: 10.3233/rnn-140459. PubMed PMID: 25735241; PubMed Central PMCID: PMCPMC4850740.

1985. Ulam F, Shelton C, Richards L, Davis L, Hunter B, Fregni F,

et al. Cumulative effects of transcranial direct current stimulation on EEG oscillations and attention/working memory during subacute neurorehabilitation of traumatic brain injury. Clin Neurophysiol. 2015;126(3):486-96. Epub 2014/06/21. doi: 10.1016/j.clinph.2014.05.015. PubMed PMID: 24947595.

1986. Urbano Da Silva E, Lara NAJR, Veiga JCE, De Souza HC. Motor cortex stimlation in parkinsonian patients with refractory freezing of gait and balance disorders responsive to transcranial direct current stimulation. Neurodegenerative Diseases. 2015;15:1962. doi: 10.1159/000381736.

1987. Varipapa RJ. Continuous theta burst stimulation (CTBs) applied to the dorsolateral prefrontal cortex modulates late somatosensory evoked potentials (SEP) in normal subjects. Journal of Clinical Neurophysiology. 2015;32(4):395. doi: 10.1097/WNP.0000000000000175.

1988. Votta-Velis G. Refresher course: Phantom pain: Treatment algorithms. Regional Anesthesia and Pain Medicine. 2015;40(5):e9-e11. doi: 10.1097/AAP.0000000000000308.

1989. Wertheimer T, Velardi E, Brede C, Xiao S, Kloss CC, Beilhack A, et al. Production of BMP4 By Endothelial Cells Is Crucial for Endogenous Thymic Regeneration. Blood. 2015;126(23). PubMed PMID: WOS:000368019002074.

1990. Wessel MJ, Zimerman M, Hummel FC. Non-invasive brain stimulation: an interventional tool for enhancing behavioral training after stroke. Frontiers in Human Neuroscience. 2015;9. doi: 10.3389/fnhum.2015.00265. PubMed PMID: WOS:000356060400001.

1991. Wilkinson L, Steel A, Mooshagian E, Zimmermann T, Keisler A, Lewis JD, et al. Online feedback enhances early consolidation of motor sequence learning and reverses recall deficit from transcranial stimulation of motor cortex. Cortex. 2015;71:134-47. doi: 10.1016/j.cortex.2015.06.012.

1992. Xie Y, Zhang T, Chen ACN. Repetitive Transcranial Magnetic Stimulation for the Recovery of Stroke Patients With Disturbance of Consciousness. Brain Stimulation. 2015;8(3):674-5. doi: 10.1016/j.brs.2015.01.406. PubMed PMID: WOS:000355772300038.

1993. Xu J, Wang D, Wang J, Wang J, Hu L. Preliminary Quantitative Evaluation of Potential Risk for HCCB TBS Tritium Extraction System Based on Fault Tree Analysis. Journal of Fusion Energy. 2015;34(6):1369-77. doi: 10.1007/s10894-015-9972-x. PubMed PMID: WOS:000363490000022.

1994. Xu T, Zhang WG, Sun J, Zhang Y, Lu JF, Han HB, et al. Protective effects of thrombomodulin on microvascular permeability after subarachnoid hemorrhage in mouse model. Neuroscience.

2015;299:18-27. doi: 10.1016/j.neuroscience.2015.04.058.
1995. Yin Z, Shen Y, Reinhardt JD, Chen C-F, Jiang X, Dai W, et al. 5 Hz Repetitive Transcranial Magnetic Stimulation with Maximum Voluntary Muscle Contraction Facilitates Cerebral Cortex Excitability of Normal Subjects. Cns & Neurological Disorders-Drug Targets. 2015;14(10):1298-303. doi: 10.2174/1871527315666151111124216. PubMed PMID: WOS:000365808000009.
1996. Yoon KJ, Lee YT, Chung PW, Lee YK, Kim DY, Chun MH. Effects of Repetitive Transcranial Magnetic Stimulation on Behavioral Recovery during Early Stage of Traumatic Brain Injury in Rats. J Korean Med Sci. 2015;30(10):1496-502. Epub 2015/10/02. doi: 10.3346/jkms.2015.30.10.1496. PubMed PMID: 26425049; PubMed Central PMCID: PMCPMC4575941.
1997. Yoon YS, Cho KH, Kim ES, Lee MS, Lee KJ. Effect of Epidural Electrical Stimulation and Repetitive Transcranial Magnetic Stimulation in Rats With Diffuse Traumatic Brain Injury. Ann Rehabil Med. 2015;39(3):416-24. Epub 2015/07/15. doi: 10.5535/arm.2015.39.3.416. PubMed PMID: 26161348; PubMed Central PMCID: PMCPMC4496513.
1998. Yuan J, Wu Y, Li JY, Zhang L, Chen X, Chen HX. [Protection of Transcutaneous Acupoint Electrical Stimulation for Brain Injury Undergoing Intervention: a Clinical Observation]. Zhongguo Zhong

Xi Yi Jie He Za Zhi. 2015;35(8):971-4. Epub 2015/10/22. PubMed PMID: 26485913.

1999. Zakhvataev VE. Possible scenarios of the influence of low-dose ionizing radiation on neural functioning. Medical Hypotheses. 2015;85(6):723-35. doi: 10.1016/j.mehy.2015.10.020.

2000. Zanakis S, Tasoulas J, Angelidis I, Dendrinos C. Tooth in the line of angle fractures: the impact in the healing process. A retrospective study of 112 patients. J Craniomaxillofac Surg. 2015;43(1):113-6. Epub 2014/12/04. doi: 10.1016/j.jcms.2014.10.020. PubMed PMID: 25465485.

2001. Zhao W, Wang C, Li Z, Chen L, Li J, Cui W, et al. Efficacy and safety of transcutaneous electrical acupoint stimulation to treat muscle spasticity following brain injury: a double-blinded, multicenter, randomized controlled trial. PLoS One. 2015;10(2):e0116976. Epub 2015/02/03. doi: 10.1371/journal.pone.0116976. PubMed PMID: 25643051; PubMed Central PMCID: PMCPMC4314074.

2002. Zhong YJ, Feng Z, Wang L, Wei TQ. Wake-promoting actions of median nerve stimulation in TBI-induced coma: An investigation of orexin-A and orexin receptor 1 in the hypothalamic region. Mol Med Rep. 2015;12(3):4441-7. Epub 2015/06/11. doi: 10.3892/mmr.2015.3898. PubMed PMID: 26059340.

2003. Zhou J, Zhou C, Li J, Zhang M. Cognitive style modulates

conscious but not unconscious thought: Comparing the deliberation-without-attention effect in analytics and wholists. Consciousness and Cognition. 2015;36:54-60. doi: 10.1016/j.concog.2015.05.018.

2004. Zhou X, Wang YH, Ghosh S, Tang WF, Pang BB, Liu MQ, et al. Genomic characterization of G3P[6], G4P[6] and G4P[8] human rotaviruses from Wuhan, China: Evidence for interspecies transmission and reassortment events. Infection, Genetics and Evolution. 2015;33:55-71. doi: 10.1016/j.meegid.2015.04.010.

2005. Zimerman M, Weide S, Wessel M, Schulz R, Timmermann JE, Bönstrup M, et al. Interactions between primary and secondary motor areas for recovered hand functions after stroke. Clinical Neurophysiology. 2015;126(8):e84-e5. doi: 10.1016/j.clinph.2015.04.115.

2006. Cyclops. Canadian Journal of Ophthalmology. 2016;51(3):135. doi: 10.1016/j.jcjo.2016.03.012.

2007. Abstracts from the 19th Annual Meeting of the North American Neuromodulation Society, NANS 2015. Neuromodulation. 2016;19(3).

2008. A Skutchanova A, Saniova B. The Enigma of Consciousness - Philosophic and Scientific Approach. Acta Medica Martiniana. 2016;16(2):37-43. doi: 10.1515/acm-2016-0010.

2009. Amaro LJA, De Brito VF, De Ribamar Castro Veloso J, Tavares JR, De Sousa FLR, Neto FXP, et al. Deafness post traumatic brain injury. International Archives of Otorhinolaryngology. 2016;20:S26.

2010. Angelakis E, Konstantinidi M, Sakas D. Clinical improvement with transcranial direct current stimulation (tDCS) in disorders of consciousness due to TBI. Brain Injury. 2016;30(5-6):613-4. PubMed PMID: WOS:000376388200304.

2011. Avakov V, Shakhova I. Non-invasive brain and kidney cooling in severe multi system brain injury complicated by septic shock. Journal of Cerebral Blood Flow and Metabolism. 2016;36:802-3. doi: 10.1177/0271678X16645239.

2012. Babyar S, Santos-Pontelli T, Will-Lemos T, Mazin S, Bikson M, Truong DQ, et al. Center of Pressure Speed Changes with tDCS Versus GVS in Patients with Lateropulsion after Stroke. Brain Stimulation. 2016;9(5):796-8. doi: 10.1016/j.brs.2016.06.053.

2013. Bai Y, Xia X, Kang J, Yin X, Yang Y, He J, et al. Evaluating the Effect of Repetitive Transcranial Magnetic Stimulation on Disorders of Consciousness by Using TMS-EEG. Front Neurosci. 2016;10:473. Epub 2016/11/05. doi: 10.3389/fnins.2016.00473. PubMed PMID: 27812319; PubMed Central PMCID: PMCPMC5071327.

2014. Balazs S, Kermanshahi K, Binder H, Rattay F, Bodis-Wollner I. Gamma-Band Modulation and Coherence in the EEG by Involuntary Eye Movements in Patients in Unresponsive Wakefulness Syndrome. Clinical EEG and Neuroscience. 2016;47(3):196-206. doi: 10.1177/1550059415601756.

2015. Barlow KM. Postconcussion Syndrome: A Review. Journal of Child Neurology. 2016;31(1):57-67. doi: 10.1177/0883073814543305. PubMed PMID: WOS:000366650400006.

2016. Biagi L, Cioni G, Fogassi L, Guzzetta A, Sgandurra G, Tosetti M. Action observation network in childhood: a comparative fMRI study with adults. Developmental Science. 2016;19(6):1075-86. doi: 10.1111/desc.12353.

2017. Bick SK, Eskandar EN. Neuromodulation for restoring memory. Neurosurg Focus. 2016;40(5):E5. Epub 2016/05/03. doi: 10.3171/2016.3.Focus162. PubMed PMID: 27132526.

2018. Bikson M, Grossman P, Thomas C, Zannou AL, Jiang J, Adnan T, et al. Safety of Transcranial Direct Current Stimulation: Evidence Based Update 2016. Brain Stimulation. 2016;9(5):641-61. doi: 10.1016/j.brs.2016.06.004. PubMed PMID: WOS:000382499100002.

2019. Boes AD, Stern AP, Bernstein M, Hooker JE, Connor A, Press DZ, et al. H-Coil Repetitive Transcranial Magnetic Stimulation

Induced Seizure in an Adult with Major Depression: A Case Report. Brain Stimulation. 2016;9(4):632-3. doi: 10.1016/j.brs.2016.04.013.

2020. Bragin D, Bragina O, Statom G, Nemoto E. THERAPEUTIC EVALUATION OF TRANSCRANIAL DIRECT CURRENT STIMULATION IN A MOUSE MODEL OF TRAUMATIC BRAIN INJURY. Journal of Neurotrauma. 2016;33(13):A90-A1. PubMed PMID: WOS:000378336200247.

2021. Brill J, Shao Z, Puche AC, Wachowiak M, Shipley MT. Serotonin increases synaptic activity in olfactory bulb glomeruli. J Neurophysiol. 2016;115(3):1208-19. Epub 2015/12/15. doi: 10.1152/jn.00847.2015. PubMed PMID: 26655822; PubMed Central PMCID: PMCPMC4808087.

2022. Burnett CJ, Krashes MJ. Resolving behavioral output via chemogenetic designer receptors exclusively activated by designer drugs. Journal of Neuroscience. 2016;36(36):9268-82. doi: 10.1523/JNEUROSCI.1333-16.2016.

2023. Casarotto S, Comanducci A, Rosanova M, Sarasso S, Fecchio M, Napolitani M, et al. Stratification of unresponsive patients by an independently validated index of brain complexity. Ann Neurol. 2016;80(5):718-29. Epub 2016/11/03. doi: 10.1002/ana.24779. PubMed PMID: 27717082; PubMed Central PMCID: PMCPMC5132045.

2024. Cassano P, Petrie S, Mischoulon D, Ionescu D, Cusin C, Katnani H, et al. Transcranial photobiomodulation for the treatment of major depressive disorder: the ELATED-2 clinical trial. Neuropsychopharmacology Conference: 55th annual meeting of the american college of neuropsychopharmacology, ACNP 2016 United states Conference start: 20161204 Conference end: 20161208. 2016;41:S354-S5. doi: 10.1038/npp.2016.241. PubMed PMID: CN-01296356.

2025. Cavaliere C, Aiello M, Di Perri C, Amico E, Martial C, Thibaut A, et al. Functional Connectivity Substrates for tDCS Response in Minimally Conscious State Patients. Front Cell Neurosci. 2016;10:257. Epub 2016/11/20. doi: 10.3389/fncel.2016.00257. PubMed PMID: 27857682; PubMed Central PMCID: PMCPMC5093112.

2026. Charbit J, Lakhal K, Deras P, Dehon A, Latry P, Boissier E, et al. Influence of surgical bleeding on the relationship between admission coagulopathy and risk of massive transfusion: lesson from 704 severe trauma patients. Vox Sanguinis. 2016;111(2):151-60. doi: 10.1111/vox.12401.

2027. Chen HI, Burke JF, Cohen AS. Editorial: Traumatic Brain Injury As a Systems Neuroscience Problem. Front Syst Neurosci. 2016;10:100. Epub 2016/12/27. doi: 10.3389/fnsys.2016.00100.

PubMed PMID: 28018187; PubMed Central PMCID: PMCPMC5145880.

2028. Chevalier A, Armstrong K, Norwood-Williams C, Gokal R. DC Electroacupuncture Effects on Scars and Sutures of a Patient with Postconcussion Pain. Medical Acupuncture. 2016;28(4):223-9. doi: 10.1089/acu.2016.1188.

2029. Chi CI. An applied research of peripheral nerve rewiring in treating hemiplegic hand after central neurologic injury. https://trialsearchwhoint/Trial2aspx?TrialID=ChiCTR-IIR-16007919. 2016. PubMed PMID: CN-01815991.

2030. Cioni B, Tufo T, Bentivoglio A, Trevisi G, Piano C. Motor cortex stimulation for movement disorders. Journal of Neurosurgical Sciences. 2016;60(2):230-41. PubMed PMID: WOS:000384947200012.

2031. Clayton E, Kinley-Cooper SK, Weber RA, Adkins DL. Brain stimulation: Neuromodulation as a potential treatment for motor recovery following traumatic brain injury. Brain Res. 2016;1640(Pt A):130-8. Epub 2016/02/09. doi: 10.1016/j.brainres.2016.01.056. PubMed PMID: 26855256; PubMed Central PMCID: PMCPMC5090977.

2032. Colella M, Biran V, Baud O. Melatonin and the newborn brain. Early Human Development. 2016;102:1-3. doi:

10.1016/j.earlhumdev.2016.09.001.

2033. Cruccu G, Attal N. Neurostimulation in neuropathic pain. European Journal of Neurology. 2016;23:902. doi: 10.1111/ene.13096.

2034. Cruccu G, Garcia-Larrea L, Hansson P, Keindl M, Lefaucheur JP, Paulus W, et al. EAN guidelines on central neurostimulation therapy in chronic pain conditions. Eur J Neurol. 2016;23(10):1489-99. Epub 2016/08/12. doi: 10.1111/ene.13103. PubMed PMID: 27511815.

2035. Davidson TW, Tremblay F. Evidence of alterations in transcallosal motor inhibition as a possible long-term consequence of concussions in sports: A transcranial magnetic stimulation study. Clin Neurophysiol. 2016;127(10):3364-75. Epub 2016/08/18. doi: 10.1016/j.clinph.2016.07.012. PubMed PMID: 27531393.

2036. De Laat F, Santegoets K, Peijnenburg R, Zuiderwijk E, Utens C. Median nerve stimulation in children and young adults in vegetative or minimally conscious state: study protocol for a randomiszed controlled trial. Brain injury. 2016;30(5-6):508-. doi: 10.3109/02699052.2016.1162060. PubMed PMID: CN-01304359.

2037. de Tommaso M. Response: Commentary: Cortical responses to salient nociceptive and not nociceptive stimuli in vegetative and minimal conscious state. Frontiers in Human Neuroscience. 2016;10(JAN2016). doi: 10.3389/fnhum.2016.00012.

2038. Debruyne P. "Cardio-Neuromodulation" With a Multielectrode

Irrigated Catheter: A Potential New Approach for Patients With Cardio-Inhibitory Syncope. J Cardiovasc Electrophysiol. 2016;27(9):1110-3. Epub 2016/06/17. doi: 10.1111/jce.13031. PubMed PMID: 27307200.

2039. Del Bigio MR, Di Curzio DL. Nonsurgical therapy for hydrocephalus: A comprehensive and critical review. Fluids and Barriers of the CNS. 2016;13(1). doi: 10.1186/s12987-016-0025-2.

2040. Di Virgilio TG, Hunter A, Wilson L, Stewart W, Goodall S, Howatson G, et al. Evidence for Acute Electrophysiological and Cognitive Changes Following Routine Soccer Heading. EBioMedicine. 2016;13:66-71. Epub 2016/10/30. doi: 10.1016/j.ebiom.2016.10.029. PubMed PMID: 27789273; PubMed Central PMCID: PMCPMC5264439.

2041. DosSantos MF, Ferreira N, Toback RL, Carvalho AC, DaSilva AF. Potential Mechanisms Supporting the Value of Motor Cortex Stimulation to Treat Chronic Pain Syndromes. Front Neurosci. 2016;10:18. Epub 2016/02/24. doi: 10.3389/fnins.2016.00018. PubMed PMID: 26903788; PubMed Central PMCID: PMCPMC4749700.

2042. Drougkas D, Konstantinidi M, Kalkanis D, Sakas DE, Datseris IE, Angelakis E. Changes in brain metabolism (18-FDG-PET) after transcranial direct current stimulation (Tdcs) in TBI patients with

disorders of consciousness (DOC). Brain Injury. 2016;30(5-6):735-6. PubMed PMID: WOS:000376388200566.

2043. Dubljević V. 21 Selected Abstracts from the Montreal Neuroethics Conference for Young Researchers. Neuroethics. 2016;9(2):137-45. doi: 10.1007/s12152-016-9262-y.

2044. Dumel G, Bourassa ME, Desjardins M, Voarino N, Charlebois-Plante C, Doyon J, et al. Multisession Anodal tDCS Protocol Improves Motor System Function in an Aging Population. Neural Plasticity. 2016;2016. doi: 10.1155/2016/5961362. PubMed PMID: WOS:000371058200001.

2045. Economou N-T, Papoutsi SN, Ilias I, Konstantinidi M, Sakas D, Dikeos D, et al. Sleep in disorders of consciousness due to traumatic brain injury before and after transcranial direct current stimulation. Brain Injury. 2016;30(5-6):790-. PubMed PMID: WOS:000376388200686.

2046. Economou NT, Papoutsi S, Konstantinidi M, Sakas D, Dikeos D, Angelakis E. Sleep structure changes in disorders of consciousness due to traumatic brain injury before and after transcranial direct current stimulation. Journal of Sleep Research. 2016;25:315-. PubMed PMID: WOS:000383445201330.

2047. Elzamarany E, Afifi L, El-Fayoumy NM, Salah H, Nada M. Motor cortex rTMS improves dexterity in relapsing-remitting and

secondary progressive multiple sclerosis. Acta Neurologica Belgica. 2016;116(2):145-50. doi: 10.1007/s13760-015-0540-y.
2048. Endisch C, Waterstraat G, Storm C, Ploner CJ, Curio G, Leithner C. Cortical somatosensory evoked high-frequency (600 Hz) oscillations predict absence of severe hypoxic encephalopathy after resuscitation. Clinical Neurophysiology. 2016;127(7):2561-9. doi: 10.1016/j.clinph.2016.04.014. PubMed PMID: WOS:000377988900003.
2049. Feldmann LK, Le Prieult F, Felzen V, Thal S, Behl C, Engelhard K, et al. Traumatic brain injury induces changes in synaptic longterm plasticity connected to alterations in protein degradation systems. Acta Physiologica. 2016;216:163.
2050. Feng Z, Du Q. Mechanisms responsible for the effect of median nerve electrical stimulation on traumatic brain injury-induced coma: orexin-A-mediated N-methyl-D-aspartate receptor subunit NR1 upregulation. Neural Regen Res. 2016;11(6):951-6. Epub 2016/08/03. doi: 10.4103/1673-5374.184494. PubMed PMID: 27482224; PubMed Central PMCID: PMCPMC4962593.
2051. Feng Z, Du Q. Mechanisms responsible for the effect of median nerve electrical stimulation on traumatic brain injuryinduced coma: Orexin-A-mediated N-methyl-D-aspartate receptor subunit NR1 upregulation. Neural Regeneration Research. 2016;11(6):951-6.

doi: 10.4103/1673-5374.184494.

2052. Flamand V, Smorenburg A, Kuo H, Marneweck M, Ferre C, Bleyenheuft Y, et al. Underpinnings of intracortical motor circuits physiology in children with unilateral cerebral palsy. Developmental Medicine and Child Neurology. 2016;58:70-1. doi: 10.1111/dmcn.51_13224.

2053. Flamez A, Cordenier A, De Raedt S, Michiels V, Smetcoren S, Van Merhaegen-Wieleman A, et al. Bilateral low frequency rTMS of the primary motor cortex may not be a suitable treatment for levodopa-induced dyskinesias in late stage Parkinson's disease. Parkinsonism and Related Disorders. 2016;22:62-7. doi: 10.1016/j.parkreldis.2015.11.009.

2054. Formaggio E, Cavinato M, Storti SF, Tonin P, Piccione F, Manganotti P. Assessment of Event-Related EEG Power After Single-Pulse TMS in Unresponsive Wakefulness Syndrome and Minimally Conscious State Patients. Brain Topogr. 2016;29(2):322-33. Epub 2015/11/23. doi: 10.1007/s10548-015-0461-3. PubMed PMID: 26590568.

2055. Fujimoto S, Kon N, Otaka Y, Yamaguchi T, Nakayama T, Kondo K, et al. Transcranial direct current stimulation over the primary and secondary somatosensory cortices transiently improves tactile spatial discrimination in stroke patients. Frontiers in

Neuroscience. 2016;10(MAR). doi: 10.3389/fnins.2016.00128.

2056. Gall C, Schmidt S, Schittkowski MP, Antal A, Ambrus GG, Paulus W, et al. Alternating Current Stimulation for Vision Restoration after Optic Nerve Damage: A Randomized Clinical Trial. PLoS One. 2016;11(6):e0156134. Epub 2016/06/30. doi: 10.1371/journal.pone.0156134. PubMed PMID: 27355577; PubMed Central PMCID: PMCPMC4927182.

2057. Gallovic MD, Bandyopadhyay S, Borteh H, Montjoy DG, Collier MA, Peine KJ, et al. Microparticles formulated from a family of novel silylated polysaccharides demonstrate inherent immunostimulatory properties and tunable hydrolytic degradability. Journal of Materials Chemistry B. 2016;4(24):4302-12. doi: 10.1039/c6tb00745g. PubMed PMID: WOS:000378936400008.

2058. Gaur GS. Concepts of synaptic plasticity, neuroplasticity and neurogenesis. Indian Journal of Physiology and Pharmacology. 2016;60(5):12.

2059. Girgis F, Pace J, Sweet J, Miller JP. Hippocampal Neurophysiologic Changes after Mild Traumatic Brain Injury and Potential Neuromodulation Treatment Approaches. Front Syst Neurosci. 2016;10:8. Epub 2016/02/24. doi: 10.3389/fnsys.2016.00008. PubMed PMID: 26903824; PubMed Central PMCID: PMCPMC4746250.

2060. Glannon W. Ethical issues in neuroprosthetics. Journal of Neural Engineering. 2016;13(2). doi: 10.1088/1741-2560/13/2/021002. PubMed PMID: WOS:000375683800003.

2061. Glass L, Moody L, Grafman J, Krueger F. Neural signatures of third-party punishment: evidence from penetrating traumatic brain injury. Social Cognitive and Affective Neuroscience. 2016;11(2):253-62. doi: 10.1093/scan/nsv105. PubMed PMID: WOS:000374226900007.

2062. González-Saldaña N, Hernández-Porras M, Macías-Parra M, Monroy-Colín VA, Acebo-Arcentales JJ, Juárez-Olguín H. Tuberculous meningitis: Symptoms, diagnosis and evaluation experienced in 532 patients in a pediatric hospital. Asian Pacific Journal of Tropical Disease. 2016;6(3):208-11. doi: 10.1016/S2222-1808(15)61015-5.

2063. Gosseries O, Pistoia F, Charland-Verville V, Carolei A, Sacco S, Laureys S. The Role of Neuroimaging Techniques in Establishing Diagnosis, Prognosis and Therapy in Disorders of Consciousness. Open Neuroimag J. 2016;10:52-68. Epub 2016/06/28. doi: 10.2174/1874440001610010052. PubMed PMID: 27347265; PubMed Central PMCID: PMCPMC4894918.

2064. Goulet-Kennedy J, Labbe S, Fecteau S. The involvement of the striatum in decision making. Dialogues in Clinical Neuroscience.

2016;18(1):55-63.
2065. Goyal E, Kadiani A, Marella S, Saldanha D. Interventional psychiatry. history, current status and future directions. Indian Journal of Psychiatry. 2016;58(5):S14-S5.
2066. Grecco LAC, Oliveira CS, Galli M, Cosmo C, Duarte NAC, Zanon N, et al. Spared primary motor cortex and the presence of MEP in cerebral palsy dictate the responsiveness to tDCS during gait training. Frontiers in Human Neuroscience. 2016;10. doi: 10.3389/fnhum.2016.00361.
2067. Gu JJ, Chen JB, Zhang JH, Zhang H, Wang SS. Recombinant human soluble thrombomodulin protects against brain injury in a CVST rat model, via downregulation of the HMGB1-RAGE axis. Molecular Medicine Reports. 2016;14(6):5217-22. doi: 10.3892/mmr.2016.5891.
2068. Guan C, Guo G, Li Z. Transcranial magnetic stimulation promotes cognition among epileptics after traumatic brain injury. Chinese Journal of Physical Medicine and Rehabilitation. 2016;38(5):349-52. PubMed PMID: CSCD:5798451.
2069. Guo S, Lu X, Zhuo J, Xu S, Gullapalli R, Gandhi D. Water diffusivity changes with varying focused ultrasound exposure levels in rat brains. Journal of Therapeutic Ultrasound. 2016;4(1). doi: 10.1186/s40349-016-0076-5.

2070. Hadanny A, Efrati S. Treatment of persistent post-concussion syndrome due to mild traumatic brain injury: current status and future directions. Expert Review of Neurotherapeutics. 2016;16(8):875-87. doi: 10.1080/14737175.2016.1205487. PubMed PMID: WOS:000381575200004.
2071. Hampel KG, Jahanbekam A, Elger CE, Surges R. Seizure-related modulation of systemic arterial blood pressure in focal epilepsy. Epilepsia. 2016;57(10):1709-18. doi: 10.1111/epi.13504.
2072. Heiss WD. Imaging effects related to language improvements by rTMS. Restorative Neurology and Neuroscience. 2016;34(4):531-6. doi: 10.3233/RNN-150631.
2073. Henkin RI, Abdelmeguid M, Knoppel AB, Potolicchio SJ. Phantageusia: The Presence of Taste Distortions Inhibited by Repetitive Transcranial Magnetic Stimulation (rTMS). Faseb Journal. 2016;30. PubMed PMID: WOS:000406444000300.
2074. Hesse S, Werner C, Byhahn M. Non-invasive brain stimulation to improve alertness and awareness of patients with disorders of consciousness: transcranial near-infrared laser therapy vs. Transcranial shock-wave therapy. Neurologie und rehabilitation. 2016;22(4):293-300. PubMed PMID: CN-01298758.
2075. Honey CM, Tronnier VM, Honey CR. Deep brain stimulation versus motor cortex stimulation for neuropathic pain: A minireview of

the literature and proposal for future research. Comput Struct Biotechnol J. 2016;14:234-7. Epub 2016/07/15. doi: 10.1016/j.csbj.2016.06.003. PubMed PMID: 27413477; PubMed Central PMCID: PMCPMC4925438.

2076. Ilić TV, Milanović S, Komatina N, Raičević R. Prefrontal transcranial direct current stimulation in patients with disorders of consciousness-a case series study. Clinical Neurophysiology. 2016;127(3):e98. doi: 10.1016/j.clinph.2015.11.330.

2077. Ille S, Sollmann N, Ringel F, Meyer B, Krieg SM. Resection of highly language-eloquent brain lesions purely based on rTMS language mapping without awake surgery. Neuro-Oncology. 2016;18:vi191. doi: 10.1093/neuonc/now212.804.

2078. Irct201605299014N. Effect of transcranial direct current stimulation versus no stimulation on working memory in elders with normal cognitive status. https://trialsearchwhoint/Trial2aspx?TrialID=IRCT201605299014N101. 2016. PubMed PMID: CN-01902289.

2079. Irct2015120625384N. Efficacy of transcranial direct current stimulation in reduction of anxiety and crystalline heroin craving. https://trialsearchwhoint/Trial2aspx?TrialID=IRCT2015120625384N1. 2016. PubMed PMID: CN-01805923.

2080. Irct2016012526189N. Evaluation of TDCS for augmentation

treatment of major depressive disorder with poor treatment response. https://trialsearchwhoint/Trial2aspx?TrialID=IRCT2016012526189N1. 2016. PubMed PMID: CN-01878489.
2081. Irct2016013026257N. Effect of transcranial stimulation on healthy older subjects function. https://trialsearchwhoint/Trial2aspx?TrialID=IRCT2016013026257N1. 2016. PubMed PMID: CN-01874119.
2082. Iscan Z, Nazarova M, Fedele T, Blagovechtchenski E, Nikulin VV. Pre-stimulus Alpha Oscillations and Inter-subject Variability of Motor Evoked Potentials in Single- and Paired-Pulse TMS Paradigms. Frontiers in Human Neuroscience. 2016;10. doi: 10.3389/fnhum.2016.00504. PubMed PMID: WOS:000385888100001.
2083. Jprn U. the effect of transcranial magnetic stimulation therapy in higher brain dysfunction patients. https://trialsearchwhoint/Trial2aspx?TrialID=JPRN-UMIN000023189. 2016. PubMed PMID: CN-01827678.
2084. Juang Y-J, Deng Y-L, Lee IC. Membrane filtration: An unconventional route for fabrication of the flexible and dissolvable, polymer microneedle patches. Biomicrofluidics. 2016;10(4). doi: 10.1063/1.4960206. PubMed PMID: WOS:000383911400021.
2085. Kang IS, Lee W, Choi BW, Choi D, Hong MK, Jang Y, et al.

Semiquantitative assessment of tibial artery calcification by computed tomography angiography and its ability to predict infrapopliteal angioplasty outcomes. Journal of Vascular Surgery. 2016;64(5):1335-43. doi: 10.1016/j.jvs.2016.04.047.

2086. Keane KM, Haskell-Ramsay CF, Veasey RC, Howatson G. Montmorency Tart cherries (Prunus cerasus L.) modulate vascular function acutely, in the absence of improvement in cognitive performance. British Journal of Nutrition. 2016;116(11):1935-44. doi: 10.1017/S0007114516004177.

2087. Kesar TM, McDonald HS, Eicholtz SP, Borich MR. Case Report of Syncope During a Single Pulse Transcranial Magnetic Stimulation Experiment in a Healthy Adult Participant. Brain Stimul. 2016;9(3):471-2. Epub 2016/04/07. doi: 10.1016/j.brs.2016.03.013. PubMed PMID: 27050115; PubMed Central PMCID: PMCPMC4928708.

2088. Khandan O, Kahook MY, Rao MP. Fenestrated microneedles for ocular drug delivery. Sensors and Actuators B-Chemical. 2016;223:15-23. doi: 10.1016/j.snb.2015.09.071. PubMed PMID: WOS:000366458700003.

2089. Kim JW, Bae KY, Kim SW, Kang HJ, Shin IS, Yoon JS, et al. Treatment-Resistant Depression Entering Remission Following a Seizure during the Course of Repetitive Transcranial Magnetic

Stimulation. Psychiatry Investig. 2016;13(4):468-71. Epub 2016/08/03. doi: 10.4306/pi.2016.13.4.468. PubMed PMID: 27482250; PubMed Central PMCID: PMCPMC4965659.

2090. Kim MO, Adji A, O'Rourke MF, Avolio AP, Smielewski P, Pickard JD, et al. Change in pulsatile cerebral arterial pressure and flow waves as a therapeutic strategy? Acta Neurochirurgica, Supplementum2016. p. 167-70.

2091. Kimura H, Kanahara N, Takase M, Yoshida T, Watanabe H, Iyo M. A randomized, sham-controlled study of high frequency rTMS for auditory hallucination in schizophrenia. Psychiatry Research. 2016;241:190-4. doi: 10.1016/j.psychres.2016.04.119. PubMed PMID: WOS:000379370900032.

2092. Kondratyeva EA, Avdunina IA, Kondratyev AN, Ulitin AU, Ivanova NE, Petrova MV, et al. [Vegetative State: Difficulty in Identifying Consciousness and Predicting Outcome]. Vestn Ross Akad Med Nauk. 2016;71(4):273-80. Epub 2016/01/01. doi: 10.15690/vramn728. PubMed PMID: 29297644.

2093. Kotecha R, Berriochoa CA, Murphy ES, Machado AG, Chao ST, Suh JH, et al. Report of whole-brain radiation therapy in a patient with an implanted deep brain stimulator: important neurosurgical considerations and radiotherapy practice principles. Journal of Neurosurgery. 2016;124(4):966-70. doi: 10.3171/2015.2.Jns142951.

PubMed PMID: WOS:000372669100010.
2094. Kusamori K, Katsumi H, Sakai R, Hayashi R, Hirai Y, Tanaka Y, et al. Development of a drug-coated microneedle array and its application for transdermal delivery of interferon alpha. Biofabrication. 2016;8(1). doi: 10.1088/1758-5090/8/1/015006. PubMed PMID: WOS:000373289000010.
2095. Långsjö JW, Lehtimäki K, Ruohonen J, Sajanti A, Sandell S, Heikkilä HT, et al. Critical neural targets for (the level of) human consciousness: Arousal arrest and unconsciousness after sumatriptan administration. Brain Inj. 2016;30(13-14):1731-6. Epub 2016/09/30. doi: 10.1080/02699052.2016.1199908. PubMed PMID: 27680211.
2096. Laureys S. Transcranial direct current stimulation: a promising new avenue. European Journal of Neurology. 2016;23:5-. PubMed PMID: WOS:000431634200012.
2097. Lefaucheur J-P. A comprehensive database of published tDCS clinical trials (2005-2016). Neurophysiologie Clinique-Clinical Neurophysiology. 2016;46(6):319-98. doi: 10.1016/j.neucli.2016.10.002. PubMed PMID: WOS:000393255800001.
2098. Legostaeva L, Zmeykina E, Kremneva E, Poydasheva A, Chervyakov A, Sergeev D, et al. FMRI-navigated rTMS original protocol for disorders of consciousness treatment. European Journal of

Neurology. 2016;23:460-. PubMed PMID: WOS:000431634202217.
2099. Legostaeva LA, Zmeykina EA, Poydasheva AG, Sergeev DV, Sinitsyn DO, Kremneva EI, et al. Новый подход к изучению сознания с позиции теории интегрированной информации (обзор) A New Approach to the Study of Consciousness from the Theory of Integrated Information Point of View (Review). Modern Technologies in Medicine. 2016;8(4):251-9. doi: 10.17691/stm2016.8.4.30. PubMed PMID: RSCI:28290656.
2100. Legostaeva LA, Zmeykina EA, Poydasheva AG, Sergeev DV, Sinitsyn DO, Kremneva EI, et al. A new approach to the study of consciousness from the theory of integrated information point of view. Sovremennye Tehnologii v Medicine. 2016;8(4):251-8. doi: 10.17691/stm2016.8.4.30.
2101. León D, Rodríguez N, Pelayo R, Martinell M, Berbaneu M, Tormos JM. Treatment of chronic non-fluent aphasia with rTMS combined with intensive speech therapy. Brain Injury. 2016;30(5-6):621. doi: 10.3109/02699052.2016.1162060.
2102. Leung A, Fallah A, Shukla S, Lin L, Tsia A, Song D, et al. rTMS in Alleviating Mild TBI Related Headaches--A Case Series. Pain Physician. 2016;19(2):E347-54. Epub 2016/01/28. PubMed PMID: 26815263.
2103. Leung A, Fallah A, Shukla S, Lin L, Tsia A, Song D, et al.

rTMS in Alleviating Mild TBI Related Headaches - A Case Series. Pain Physician. 2016;19(2):E347-E53. PubMed PMID: WOS:000368991000010.

2104. Leung A, Fallah A, Shukla S, Lin L, Tsia A, Song D, et al. RTMS in alleviating mild TBI related headaches – A case series. Pain Physician. 2016;19(2):E347-E53.

2105. Leung A, Shukla S, Fallah A, Song D, Lin L, Golshan S, et al. Repetitive Transcranial Magnetic Stimulation in Managing Mild Traumatic Brain Injury-Related Headaches. Neuromodulation. 2016;19(2):133-41. Epub 2015/11/12. doi: 10.1111/ner.12364. PubMed PMID: 26555886.

2106. Leung A, Shukla S, Yang E, Canlas B, Kadokana M, Heald J, et al. Diminished supraspinal pain modulation in patients with mild traumatic brain injury. Molecular Pain. 2016;12. doi: 10.1177/1744806916662661. PubMed PMID: WOS:000386444700001.

2107. Li J, Meng XM, Li RY, Zhang R, Zhang Z, Du YF. Effects of different frequencies of repetitive transcranial magnetic stimulation on the recovery of upper limb motor dysfunction in patients with subacute cerebral infarction. Neural Regeneration Research. 2016;11(10):1584-90. doi: 10.4103/1673-5374.193236.

2108. Lin N, Antiel R, Waqar L, Hoffman-Craven C, Monos S,

D'Agostino J, et al. Brain maturation and neurodevelopmental outcomes in infants with congenital diaphragmatic hernia. Annals of Neurology. 2016;80:S401-S3. doi: 10.1002/pon.4272.

2109. Liu P, Gao J, Pan S, Meng F, Pan G, Li J, et al. Effects of High-Frequency Repetitive Transcranial Magnetic Stimulation on Cerebral Hemodynamics in Patients with Disorders of Consciousness: A Sham-Controlled Study. Eur Neurol. 2016;76(1-2):1-7. Epub 2016/06/23. doi: 10.1159/000447325. PubMed PMID: 27332827.

2110. Liu Y, Yang H, Tang X, Bai W, Wang G, Tian X. Repetitive transcranial magnetic stimulation regulates L-type Ca2+ channel activity inhibited by early sevoflurane exposure. Brain Research. 2016;1646:207-18. doi: 10.1016/j.brainres.2016.05.045. PubMed PMID: WOS:000381844700023.

2111. Loescher W. Fit for purpose application of currently existing animal models in the discovery of novel epilepsy therapies. Epilepsy Research. 2016;126:157-84. doi: 10.1016/j.eplepsyres.2016.05.016. PubMed PMID: WOS:000383825200022.

2112. Lorente L, Martín MM, Pérez-Cejas A, Abreu-González P, Ramos L, Argueso M, et al. Association between total antioxidant capacity and mortality in ischemic stroke patients. Annals of Intensive Care. 2016;6(1). doi: 10.1186/s13613-016-0143-7.

2113. Luvizutto GJ, Dos Santos MRL, Rodrigues RT, Fogaroli MO,

Braga GP, Bazan SGZ, et al. Relation between anthropometric variables and respiratory muscular strength in the acute phase of stroke. International Journal of Stroke. 2016;11:133. doi: 10.1177/1747493016670567.

2114. Magrone T, Russo MA, Jirillo E. Role of immune cells in the course of central nervous system injury: Modulation with natural products. Current Pharmaceutical Design. 2016;22(6):701-8. doi: 10.2174/1381612822666151204000959.

2115. Mahler DA. A Perspective on Acupuncture Techniques for Relief of Dyspnea in Chronic Obstructive Pulmonary Disease. Medical Acupuncture. 2016;28(1):28-32. doi: 10.1089/acu.2015.1117.

2116. Maistreli S, Kourlaba G, Georgopoulos D, Maniadakis N. Budget impact analysis of the incorporation of a computerized temperature management system in the treatment of therapeutic hypothermia in Greece. Value in Health. 2016;19(7):A630-A1.

2117. Manogaran P, Vavasour I, Borich M, Kolind SH, Lange AP, Rauscher A, et al. Corticospinal tract integrity measured using transcranial magnetic stimulation and magnetic resonance imaging in neuromyelitis optica and multiple sclerosis. Multiple Sclerosis Journal. 2016;22(1):43-50. doi: 10.1177/1352458515579441. PubMed PMID: WOS:000367252500010.

2118. Marangolo P, Fiori V, Sabatini U, De Pasquale G, Razzano C,

Caltagirone C, et al. Bilateral Transcranial Direct Current Stimulation Language Treatment Enhances Functional Connectivity in the Left Hemisphere: Preliminary Data from Aphasia. J Cogn Neurosci. 2016;28(5):724-38. Epub 2016/01/26. doi: 10.1162/jocn_a_00927. PubMed PMID: 26807842.
2119. Martens G. Double blind randomized controlled clinical trial of fronto-parietal tdcs in patients with disorders of consciousness. Archives of physical medicine and rehabilitation. 2016;97(10):e33-e4. PubMed PMID: CN-01721885.
2120. Martial C, Thibaut A, Bruno MA, Wannez S, Chatelle C, Donneau AF, et al. Five days of left prefrontal transcranial direct current stimulation improve signs of consciousness in 50% of chronic minimally conscious state. Brain injury. 2016;30(5-6):520-1. doi: 10.3109/02699052.2016.1162060. PubMed PMID: CN-01304309.
2121. Martino Cinnera A, Bonnì S, Iosa M, Ponzo V, Fusco A, Caltagirone C, et al. Clinical effects of non-invasive cerebellar magnetic stimulation treatment combined with neuromotor rehabilitation in traumatic brain injury. A single case study. Funct Neurol. 2016;31(2):117-20. Epub 2016/07/01. PubMed PMID: 27358225.
2122. Matsumoto-Miyazaki J, Asano Y, Yonezawa S, Nomura Y, Ikegame Y, Aki T, et al. Acupuncture Increases the Excitability of the

Cortico-Spinal System in Patients with Chronic Disorders of Consciousness Following Traumatic Brain Injury. J Altern Complement Med. 2016;22(11):887-94. Epub 2016/09/24. doi: 10.1089/acm.2014.0356. PubMed PMID: 27662495.

2123. Matsushima K, Inaba K, Cho J, Mohammed H, Herr K, Leichtle S, et al. Therapeutic anticoagulation in patients with traumatic brain injury. Journal of Surgical Research. 2016;205(1):186-91. doi: 10.1016/j.jss.2016.06.042.

2124. Matteo BM, Viganò B, Cerri CG, Perin C. Visual field restorative rehabilitation after brain injury. J Vis. 2016;16(9):11. Epub 2016/07/30. doi: 10.1167/16.9.11. PubMed PMID: 27472498.

2125. Meng L, Jiang J, Jin C, Liu J, Zhao Y, Wang W, et al. Trauma-specific Grey Matter Alterations in PTSD. Sci Rep. 2016;6:33748. Epub 2016/09/22. doi: 10.1038/srep33748. PubMed PMID: 27651030; PubMed Central PMCID: PMCPMC5030628.

2126. Meola A. Letter to the Editor. Brain Injury. 2016;30(13-14):1515-6. doi: 10.1080/02699052.2016.1199912.

2127. Momosaki R, Kinoshita S, Kakuda W, Yamada N, Abo M. Noninvasive brain stimulation for dysphagia after acquired brain injury: a systematic review. J Med Invest. 2016;63(3-4):153-8. Epub 2016/09/21. doi: 10.2152/jmi.63.153. PubMed PMID: 27644550.

2128. Montenegro RA, Farinatti PTV, De Lima PFM, Okano AH,

Menêses AL, De Oliveira-Neto L, et al. Motor cortex tDCS does not modulate perceived exertion within multiple-sets of resistance exercises. Isokinetics and Exercise Science. 2016;24(1):17-24. doi: 10.3233/IES-150593.

2129. Morecraft RJ, Ge J, Stilwell-Morecraft KS, McNeal DW, Hynes SM, Pizzimenti MA, et al. Frontal and frontoparietal injury differentially affect the ipsilateral corticospinal projection from the nonlesioned hemisphere in monkey (Macaca mulatta). Journal of Comparative Neurology. 2016;524(2):380-407. doi: 10.1002/cne.23861. PubMed PMID: WOS:000366604700010.

2130. Morishita T, Inoue T. Brain Stimulation Therapy for Central Post-Stroke Pain from a Perspective of Interhemispheric Neural Network Remodeling. Front Hum Neurosci. 2016;10:166. Epub 2016/05/06. doi: 10.3389/fnhum.2016.00166. PubMed PMID: 27148019; PubMed Central PMCID: PMCPMC4838620.

2131. Naim-Feil J, Bradshaw JL, Rogasch NC, Daskalakis ZJ, Sheppard DM, Lubman DI, et al. Cortical inhibition within motor and frontal regions in alcohol dependence post-detoxification: A pilot TMS-EEG study. World Journal of Biological Psychiatry. 2016;17(7):547-56. doi: 10.3109/15622975.2015.1066512.

2132. Naro A, Bramanti P, Leo A, Russo M, Calabrò RS. Transcranial Alternating Current Stimulation in Patients with Chronic

Disorder of Consciousness: A Possible Way to Cut the Diagnostic Gordian Knot? Brain Topogr. 2016;29(4):623-44. Epub 2016/04/12. doi: 10.1007/s10548-016-0489-z. PubMed PMID: 27062669.

2133. Naro A, Leo A, Buda A, Manuli A, Bramanti A, Bramanti P, et al. Do you see me? The role of visual fixation in chronic disorders of consciousness differential diagnosis. Brain Res. 2016;1653:59-66. Epub 2016/10/25. doi: 10.1016/j.brainres.2016.10.015. PubMed PMID: 27773729.

2134. Naro A, Leo A, Buda A, Manuli A, Bramanti A, Bramanti P, et al. Unravelling motor networks in patients with chronic disorders of consciousness: A promising minimally invasive approach. Brain Res. 2016;1646:262-8. Epub 2016/06/12. doi: 10.1016/j.brainres.2016.06.012. PubMed PMID: 27288702.

2135. Naro A, Leo A, Buda A, Manuli A, Bramanti A, Bramanti P, et al. Do you see me? The role of visual fixation consciousness differential diagnosis. Brain Research. 2016;1653:59-66. doi: 10.1016/j.brainres.2016.10.015. PubMed PMID: WOS:000389730100007.

2136. Naro A, Russo M, Leo A, Cannavò A, Manuli A, Bramanti A, et al. Cortical connectivity modulation induced by cerebellar oscillatory transcranial direct current stimulation in patients with chronic disorders of consciousness: A marker of covert cognition?

Clin Neurophysiol. 2016;127(3):1845-54. Epub 2016/01/13. doi: 10.1016/j.clinph.2015.12.010. PubMed PMID: 26754875.

2137. Naros G, Geyer M, Koch S, Mayr L, Ellinger T, Grimm F, et al. Enhanced motor learning with bilateral transcranial direct current stimulation: Impact of polarity or current flow direction? Clinical Neurophysiology. 2016;127(4):2119-26. doi: 10.1016/j.clinph.2015.12.020.

2138. Nct. tDCS on Precuneus in MCS: Repeated Stimulations. https://clinicaltrialsgov/show/NCT02702362. 2016. PubMed PMID: CN-01556348.

2139. Nct. fMRI-neuronavigated rTMS for the Treatment of Major Depression Associated With TBI. https://clinicaltrialsgov/show/NCT02980484. 2016. PubMed PMID: CN-02039937.

2140. Nct. Neurorehabilitation After Traumatic Brain Injury: Functional Magnetic Resonance Imaging Study. https://clinicaltrialsgov/show/NCT02698449. 2016. PubMed PMID: CN-01556236.

2141. Nct. Transcranial Direct Current Stimulation (TDCS) as an Intervention for Patients With Traumatic Brain Injury. https://clinicaltrialsgov/show/NCT02849223. 2016. PubMed PMID: CN-01583032.

2142. Nct. Two Intensities of Transcranial Direct Current Stimulation to Improve Consciousness in Severely Brain Injured Patients. https://clinicaltrialsgov/show/NCT03007784. 2016. PubMed PMID: CN-01560967.
2143. Nct. Corticospinal Excitability After rTMS in Spinal Cord Injury Patients. https://clinicaltrialsgov/show/NCT03014999. 2016. PubMed PMID: CN-01592507.
2144. Nct. Impact of Celecoxib on Electrophysiological Property in Brain of Healthy Volunteer. https://clinicaltrialsgov/show/NCT02711579. 2016. PubMed PMID: CN-02036454.
2145. Nct. tDCS Associated With Peripheral Electrical Stimulation for Pain Control in Individuals With Sickle Cell Disease. https://clinicaltrialsgov/show/NCT02813629. 2016. PubMed PMID: CN-02042100.
2146. Nct. Investigating the Physiological, Cognitive, and Psychological Effects of a Cognitive-Based Intervention. https://clinicaltrialsgov/show/NCT02906449. 2016. PubMed PMID: CN-01520917.
2147. Nct. Augmentation of Locomotor Adaptation Post-Stroke. https://clinicaltrialsgov/show/NCT02892084. 2016. PubMed PMID: CN-01580117.

2148. Nekkanti S, Shaik R, Mondem S, Meruva N, Elumalai G. Effect of right median nerve stimulation on level of consciousness in traumatic brain injury subjects. Asian journal of pharmaceutical research and health care. 2016;8(3):67-71. doi: 10.18311/ajprhc/2016/751. PubMed PMID: CN-01165502.

2149. Neren D, Johnson MD, Legon W, Bachour SP, Ling G, Divani AA. Vagus Nerve Stimulation and Other Neuromodulation Methods for Treatment of Traumatic Brain Injury. Neurocrit Care. 2016;24(2):308-19. Epub 2015/09/25. doi: 10.1007/s12028-015-0203-0. PubMed PMID: 26399249.

2150. Neville IS, Hayashi CY, Rodrigues P, Galhardoni RRG, Brunoni AR, Zaninotto AL, et al. Cortical excitability assessment on traumatic brain injury (10649). Neuromodulation. 2016;19(3):e2. doi: 10.1111/ner.12428.

2151. Neville IS, Hayashi CY, Rodrigues P, Galhardoni RRG, Sousa Jr LM, Rios RM. High-frequency rTMS for traumatic brain injury: tolerability and safety considerations (10743). Neuromodulation. 2016;19(3):e71. doi: 10.1111/ner.12428. PubMed PMID: CN-01214457.

2152. Neville IS, Hayashi CY, Sousa Jr LM, Galhardoni RRG, Moraes TM, Rodrigues P, et al. Application of rTMS on victims of Bifrontal Cerebral Contusions. (10746). Neuromodulation.

2016;19(3):e71. doi: 10.1111/ner.12428.

2153. Nieminen JO, Gosseries O, Massimini M, Saad E, Sheldon AD, Boly M, et al. Consciousness and cortical responsiveness: a within-state study during non-rapid eye movement sleep. Sci Rep. 2016;6:30932. Epub 2016/08/06. doi: 10.1038/srep30932. PubMed PMID: 27491799; PubMed Central PMCID: PMCPMC4974655.

2154. Okie S. TBI's long-term follow-up-slow progress in science and recovery. New England Journal of Medicine. 2016;375(2):180-4. doi: 10.1056/NEJMms1604272.

2155. Otal B, Dutta A, Foerster Á, Ripolles O, Kuceyeski A, Miranda PC, et al. Opportunities for guided multichannel non-invasive transcranial current stimulation in poststroke rehabilitation. Frontiers in Neurology. 2016;7(FEB). doi: 10.3389/fneur.2016.00021.

2156. Padilla R, Domina A. Effectiveness of Sensory Stimulation to Improve Arousal and Alertness of People in a Coma or Persistent Vegetative State After Traumatic Brain Injury: A Systematic Review. Am J Occup Ther. 2016;70(3):7003180030p1-8. Epub 2016/04/19. doi: 10.5014/ajot.2016.021022. PubMed PMID: 27089287.

2157. Panuccio G, Semprini M, Chiappalone M. Intelligent biohybrid systems for functional brain repair. New Horizons in Translational Medicine. 2016;3(3-4):162-74. doi: 10.1016/j.nhtm.2016.10.001.

2158. Pape T, Rosenow J, Herrold A, Harton B, Patil V, Guernon A. Neurobiological model for use of rTMS + amantadine as a treatment to modulate and shape neural repair for persons in states of disordered consciousness. Brain Injury. 2016;30(5-6):615-6. doi: 10.3109/02699052.2016.1162060.

2159. Parenti L, Barloscio D, Bartolotta M, Di Rollo A, Sartucci F, Bocci T. Intracortical and interhemispheric changes induced by Transcutaneous spinal Direct Current Stimulation (tsDCS). Clinical Neurophysiology. 2016;127(12):e339. doi: 10.1016/j.clinph.2016.10.077.

2160. Pearce AJ. The Neurophysiological Response Following Sub-Concussive Soccer Heading. EBioMedicine. 2016;13:3-4. Epub 2016/11/08. doi: 10.1016/j.ebiom.2016.10.043. PubMed PMID: 27818040; PubMed Central PMCID: PMCPMC5264430.

2161. Pevzner A, Izadi A, Lee DJ, Shahlaie K, Gurkoff GG. Making Waves in the Brain: What Are Oscillations, and Why Modulating Them Makes Sense for Brain Injury. Front Syst Neurosci. 2016;10:30. Epub 2016/04/20. doi: 10.3389/fnsys.2016.00030. PubMed PMID: 27092062; PubMed Central PMCID: PMCPMC4823270.

2162. Plenger P, Krishnan K, Cloud M, Bosworth C, Qualls D, de la Plata CM. fNIRS-based investigation of the Stroop task after TBI. Brain Imaging and Behavior. 2016;10(2):357-66. doi:

10.1007/s11682-015-9401-9. PubMed PMID: WOS:000382390000004.

2163. Poole VN, Robinson ME, Singleton O, DeGutis J, Milberg WP, McGlinchey RE, et al. Intrinsic functional connectivity predicts individual differences in distractibility. Neuropsychologia. 2016;86:176-82. doi: 10.1016/j.neuropsychologia.2016.04.023. PubMed PMID: WOS:000378453400018.

2164. Pruitt DT, Schmid AN, Kim LJ, Abe CM, Trieu JL, Choua C, et al. Vagus Nerve Stimulation Delivered with Motor Training Enhances Recovery of Function after Traumatic Brain Injury. J Neurotrauma. 2016;33(9):871-9. Epub 2015/06/11. doi: 10.1089/neu.2015.3972. PubMed PMID: 26058501; PubMed Central PMCID: PMCPMC4860663.

2165. Pycroft L, Boccard SG, Owen SLF, Stein JF, Fitzgerald JJ, Green AL, et al. Brainjacking: Implant Security Issues in Invasive Neuromodulation. World Neurosurg. 2016;92:454-62. Epub 2016/05/18. doi: 10.1016/j.wneu.2016.05.010. PubMed PMID: 27184896.

2166. Rabie A, Metman LV, Fakhry M, Eassa AYE, Fouad W, Shakal A, et al. Improvement of advanced Parkinson’s disease manifestations with deep brain stimulation of the subthalamic nucleus: A single institution experience. Brain Sciences. 2016;6(4). doi:

10.3390/brainsci6040058.
2167. Rahimpour S, Lad SP. Frontiers in Neuroscience
Traumatic Brain Injury and Potential for Neuromodulation. In: Laskowitz D, Grant G, editors. Translational Research in Traumatic Brain Injury. Boca Raton (FL): CRC Press/Taylor and Francis Group © 2016 by Taylor & Francis Group, LLC.; 2016.
2168. Raithatha R, Carrico C, Powell ES, Westgate PM, Chelette Ii KC, Lee K, et al. Non-invasive brain stimulation and robot-assisted gait training after incomplete spinal cord injury: a randomized pilot study. NeuroRehabilitation. 2016;38(1):15-25. doi: 10.3233/NRE-151291. PubMed PMID: CN-01137986.
2169. Rajan TS, Cuzzocrea S, Bruschetta D, Quartarone A. Repetitive Transcranial Magnetic Stimulation as a Novel Therapy in Animal Models of Traumatic Brain Injury. Methods Mol Biol. 2016;1462:433-43. Epub 2016/09/09. doi: 10.1007/978-1-4939-3816-2_24. PubMed PMID: 27604732.
2170. Ranft A, Golkowski D, Kiel T, Riedl V, Kohl P, Rohrer G, et al. Neural Correlates of Sevoflurane-induced Unconsciousness Identified by Simultaneous Functional Magnetic Resonance Imaging and Electroencephalography. Anesthesiology. 2016;125(5):861-72. doi: 10.1097/ALN.0000000000001322.
2171. Rasche D, Tronnier VM. Clinical Significance of Invasive

Motor Cortex Stimulation for Trigeminal Facial Neuropathic Pain Syndromes. Neurosurgery. 2016;79(5):655-66. Epub 2016/10/21. doi: 10.1227/neu.0000000000001353. PubMed PMID: 27465843.

2172. Raucci U, Tomasello C, Marri M, Salzano M, Gasparini A, Conicella E. Scrambler Therapy® MC-5A for Complex Regional Pain Syndrome: Case Reports. Pain Practice. 2016;16(7):E103-E9. doi: 10.1111/papr.12474.

2173. Rigon A, Voss MW, Turkstra LS, Mutlu B, Duff MC. Frontal and Temporal Structural Connectivity Is Associated with Social Communication Impairment Following Traumatic Brain Injury. J Int Neuropsychol Soc. 2016;22(7):705-16. Epub 2016/07/14. doi: 10.1017/s1355617716000539. PubMed PMID: 27405965; PubMed Central PMCID: PMCPMC5823263.

2174. Roberts A, Sithole A, Sedghi M, Walker CA, Quinn TM. Minimal adverse effects profile following implantation of periauricular percutaneous electrical nerve field stimulators: a retrospective cohort study. Med Devices (Auckl). 2016;9:389-93. Epub 2016/11/16. doi: 10.2147/mder.S107426. PubMed PMID: 27843360; PubMed Central PMCID: PMCPMC5098779.

2175. Romero G, Christiansen MG, Barbosa LS, Garcia F, Anikeeva P. Localized Excitation of Neural Activity via Rapid Magnetothermal Drug Release. Advanced Functional Materials. 2016;26(35):6471-8.

doi: 10.1002/adfm.201602189. PubMed PMID: WOS:000384809100013.

2176. Rosa MA, Abdo GL, Rosa MO. Efficacy and safety of TMS during eight years in a Brazilian service: Are safety guidelines too strict? Journal of ECT. 2016;32(3):218.

2177. Rudisch J, Butler J, Izadi H, Zielinski IM, Aarts P, Birtles D, et al. Kinematic parameters of hand movement during a disparate bimanual movement task in children with unilateral Cerebral Palsy. Human Movement Science. 2016;46:239-50. doi: 10.1016/j.humov.2016.01.010. PubMed PMID: WOS:000370098600023.

2178. Sacco K, Galetto V, Dimitri D, Geda E, Perotti F, Zettin M, et al. Concomitant Use of Transcranial Direct Current Stimulation and Computer-Assisted Training for the Rehabilitation of Attention in Traumatic Brain Injured Patients: Behavioral and Neuroimaging Results. Front Behav Neurosci. 2016;10:57. Epub 2016/04/12. doi: 10.3389/fnbeh.2016.00057. PubMed PMID: 27065823; PubMed Central PMCID: PMCPMC4814724.

2179. Sahoo S, Rout CS. Facile Electrochemical Synthesis of Porous Manganese-Cobalt-Sulfide Based Ternary Transition Metal Sulfide Nanosheets Architectures for High Performance Energy Storage Applications. Electrochimica Acta. 2016;220:57-66. doi:

10.1016/j.electacta.2016.10.043. PubMed PMID: WOS:000389090800007.
2180. Sasso V, Bisicchia E, Latini L, Ghiglieri V, Cacace F, Carola V, et al. Repetitive transcranial magnetic stimulation reduces remote apoptotic cell death and inflammation after focal brain injury. J Neuroinflammation. 2016;13(1):150. Epub 2016/06/16. doi: 10.1186/s12974-016-0616-5. PubMed PMID: 27301743; PubMed Central PMCID: PMCPMC4908713.
2181. Sasso V, Bisicchia E, Latini L, Ghiglieri V, Molinari M, Viscomi M. Repetitive transcranial magnetic stimulation protects neurons form remote degeneration after focal brain injury in rats. Clinical Neuropathology. 2016;35(3):146-7.
2182. Sbicigo JB, Piccolo LR, Becker N, Vedana SN, Rodrigues JC, Salles JF, et al. Current perspectives on the anatomo-clinical method in neuropsychology. Psychology and Neuroscience. 2016;9(2):198-214. doi: 10.1037/pne0000050.
2183. Schiff ND. Central thalamic deep brain stimulation to support anterior forebrain mesocircuit function in the severely injured brain. J Neural Transm (Vienna). 2016;123(7):797-806. Epub 2016/04/27. doi: 10.1007/s00702-016-1547-0. PubMed PMID: 27113938.
2184. Schorr B, Schlee W, Arndt M, Bender A. Coherence in resting-state EEG as a predictor for the recovery from unresponsive

wakefulness syndrome. Journal of Neurology. 2016;263(5):937-53. doi: 10.1007/s00415-016-8084-5. PubMed PMID: WOS:000376141000012.

2185. Scott R, Van-Ruiten H, Baker M, Mitra D, McFarland R, Ramesh V. Explosive, new-onset epilepsia partialis continua in a teenage girl caused by mitochondrial disease-treatment with transcranial direct current electrical stimulation. Developmental Medicine and Child Neurology. 2016;58:3. doi: 10.1111/dmcn.12997.

2186. Seeger T, Kirton A, Barlow K. Cortical excitability after mild traumatic brain injury in children. Neurology. 2016;86(16).

2187. Sharma M, Naik V, Deogaonkar M. Emerging applications of deep brain stimulation. J Neurosurg Sci. 2016;60(2):242-55. Epub 2016/01/21. PubMed PMID: 26788743.

2188. Sharpee TO, Destexhe A, Kawato M, Sekulić V, Skinner FK, Wójcik DK, et al. 25th Annual Computational Neuroscience Meeting: CNS-2016. BMC Neurosci. 2016;17 Suppl 1(Suppl 1):54. Epub 2016/08/19. doi: 10.1186/s12868-016-0283-6. PubMed PMID: 27534393; PubMed Central PMCID: PMCPMC5001212.

2189. Shehata HS, Esmail EH, Abdelalim A, El-Jaafary S, Elmazny A, Sabbah A, et al. Repetitive transcranial magnetic stimulation versus botulinum toxin injection in chronic migraine prophylaxis: A pilot randomized trial. Journal of Pain Research. 2016;9:771-7. doi:

10.2147/JPR.S116671.
2190. Shen Q, Fang Y, Wu J, Li N, You P, Liu P, et al. Effect of hyperbaric oxygen combined with repetitive transcranial magnetic stimulation in the treatment of coma following craniocerebral trauma. Chinese Journal of Nautical Medicine and Hyperbaric Medicine. 2016;23(1):9-12. PubMed PMID: CSCD:5669180.
2191. Simpson M, Churilov L, Bardsley B, Heriot E, Dimovitis J, Carey L, et al. The Effects of modified-release fampridine on upper limb impairment in multiple sclerosis. Multiple sclerosis (Houndmills, Basingstoke, England). 2016;22:685-. doi: 10.1177/1352458516663086. PubMed PMID: CN-01212377.
2192. Singh H, Cooper R, Lee CW, Dempsey L, Brigadoi S, Edwards A, et al. Neurovascular interactions in the neurologically compromised neonatal brain. Advances in Experimental Medicine and Biology2016. p. 485-92.
2193. Sobreira G, Aleixo MA, Moreia C, Oliveira J. Adverse effects in repetitive transcranial magnetic stimulation-prevention and management. European Psychiatry. 2016;33:S555-S6. doi: 10.1016/j.eurpsy.2016.01.2052.
2194. Sollmann N, Kubitscheck A, Maurer S, Ille S, Hauck T, Kirschke JS, et al. Preoperative language mapping by repetitive navigated transcranial magnetic stimulation and diffusion tensor

imaging fiber tracking and their comparison to intraoperative stimulation. Neuroradiology. 2016;58(8):807-18. doi: 10.1007/s00234-016-1685-y.

2195. Sorato Bezerra O, Babis Magoulas C, Schuh Teixeira Da Rosa AL, Primo De Carvalho Alves L. Efficacy of low-frequency rTMS on motor recovery after-stroke. European Journal of Neurology. 2016;23:224. doi: 10.1111/ene.13092.

2196. Speth J, Speth C. Motor imagery in REM sleep is increased by transcranial direct current stimulation of the left motor cortex (C3). Neuropsychologia. 2016;86:57-65. Epub 2016/04/16. doi: 10.1016/j.neuropsychologia.2016.04.010. PubMed PMID: 27079954.

2197. Staudt M, Küpper H, Pieper T, Gröschel S, Winkler P, Holthausen H, et al. Prediction of contralateral hand function after hemispherectomy for refractory epilepsies: Experience from 102 pediatric cases. Neuropediatrics. 2016;47. doi: 10.1055/s-0036-1583712.

2198. Suntrup-Krueger S, Bittner S, Recker S, Meuth SG, Warnecke T, Suttrup I, et al. Electrical pharyngeal stimulation increases substance P level in saliva. Neurogastroenterology and Motility. 2016;28(6):855-60. doi: 10.1111/nmo.12783.

2199. Takechi K, Hori S, Yamashiro K, Yuki T, Hori A. A case of successful drug-eluting microsphere transcatheter arterial

embolization for hepatocellular carcinoma with associated tumor thrombus in the inferior vena cava and right atrium. Japanese Journal of Cancer and Chemotherapy. 2016;43(11):1385-8.

2200. Tan CSH, Ho CFY, Heng SS, Wu JS, Tan BKH, Ng YK, et al. Clinacanthus nutans Extracts Modulate Epigenetic Link to Cytosolic Phospholipase A2 Expression in SH-SY5Y Cells and Primary Cortical Neurons. NeuroMolecular Medicine. 2016;18(3):441-52. doi: 10.1007/s12017-016-8404-z.

2201. Tanaka J. Editorial. Visual Cognition. 2016;24(1):1. doi: 10.1080/13506285.2016.1213000.

2202. Tang WC, Hsu YC, Wang CC, Hu CY, Chio CC, Kuo JR. Early electroacupuncture treatment ameliorates neuroinflammation in rats with traumatic brain injury. BMC Complement Altern Med. 2016;16(1):470. Epub 2016/11/18. doi: 10.1186/s12906-016-1457-6. PubMed PMID: 27852302; PubMed Central PMCID: PMCPMC5112630.

2203. Thibaut A. Controlled clinical trial of 4 weeks of home-based tDCS in patients with chronic minimally conscious state. Archives of physical medicine and rehabilitation. 2016;97(10):e23-. PubMed PMID: CN-01784253.

2204. Thiel A, Zumbansen A. The pathophysiology of post-stroke aphasia: A network approach. Restorative Neurology and

Neuroscience. 2016;34(4):507-18. doi: 10.3233/RNN-150632.

2205. Tidoni E, Candidi M. Commentary: Understanding intentions from actions: Direct perception, inference, and the roles of mirror and mentalizing systems. Frontiers in Behavioral Neuroscience. 2016;10(FEB). doi: 10.3389/fnbeh.2016.00013.

2206. Tsujita-Inoue K, Hirota M, Atobe T, Ashikaga T, Tokura Y, Kouzuki H. Development of novel in vitro photosafety assays focused on the Keap1-Nrf2-ARE pathway. Journal of Applied Toxicology. 2016;36(7):956-68. doi: 10.1002/jat.3234.

2207. Turner DA. Frontiers in Neuroscience
Enhanced Functional Outcome from Traumatic Brain Injury with Brain–Machine Interface Neuromodulation: Neuroprosthetic Scaling in Relation to Injury Severity. In: Laskowitz D, Grant G, editors. Translational Research in Traumatic Brain Injury. Boca Raton (FL): CRC Press/Taylor and Francis Group
© 2016 by Taylor & Francis Group, LLC.; 2016.

2208. Vaalto S, Loo S, Mãenpãã H. Lateralization differences in hand and leg motor representations in patients with cerebral palsy. Clinical Neurophysiology. 2016;127(3):e34. doi: 10.1016/j.clinph.2015.11.105.

2209. Vaessen MJ, Saj A, Loublad K-O, Gschwind M, Vuilleumier P. Structural white-matter connections mediating distinct behavioral

components of spatial neglect in right brain-damaged patients. Cortex. 2016;77:54-68. doi: 10.1016/j.cortex.2015.12.008. PubMed PMID: WOS:000374081600005.

2210. Valenza MC, Torres-Sánchez I, Cabrera-Martos I, Valenza-Demet G, Cano-Cappellacci M. Acute Effects of Contract-Relax Stretching vs. TENS in Young Subjects with Anterior Knee Pain: A Randomized Controlled Trial. Journal of Strength and Conditioning Research. 2016;30(8):2271-8. doi: 10.1097/JSC.0000000000000215.

2211. van den Heuvel CNAM, Tijssen MAJ, van de Warrenburg BPC, Delnooz CCS. The Symptomatic Treatment of Acquired Dystonia: A Systematic Review. Movement Disorders Clinical Practice. 2016;3(6):548-58. doi: 10.1002/mdc3.12400.

2212. Vannini B, Bocci T, Caleo M, Vergari M, Rossi S, Priori A, et al. An unexpected target of spinal direct current stimulation: Interhemispheric connectivity in humans. Clinical Neurophysiology. 2016;127(4):e158. doi: 10.1016/j.clinph.2015.09.122.

2213. Ventura RE, Balcer LJ, Galetta SL, Rucker JC. Ocular motor assessment in concussion: Current status and future directions. Journal of the Neurological Sciences. 2016;361:79-86. doi: 10.1016/j.jns.2015.12.010. PubMed PMID: WOS:000370093400016.

2214. Wagner D, Eslinger PJ, Barrett AM. Decreased Leftward

'Aiming' Motor-Intentional Spatial Cuing in Traumatic Brain Injury. Neuropsychology. 2016;30(6):731-41. doi: 10.1037/neu0000252. PubMed PMID: WOS:000383879300008.

2215. Walsh EM, Blabas B, Guernon A. Reliability of motor threshold testing. Archives of Physical Medicine and Rehabilitation. 2016;97(10):e80.

2216. Wang QL, Zhu DD, Chen Y, Guo XD. A fabrication method of microneedle molds with controlled microstructures. Materials Science & Engineering C-Materials for Biological Applications. 2016;65:135-42. doi: 10.1016/j.msec.2016.03.097. PubMed PMID: WOS:000376833200016.

2217. Watson JC, Sandroni P. Central Neuropathic Pain Syndromes. Mayo Clinic Proceedings. 2016;91(3):372-85. doi: 10.1016/j.mayocp.2016.01.017.

2218. Werner C, Byhahn M, Hesse S. Non-invasive brain stimulation to promote alertness and awareness in chronic patients with disorders of consciousness: Low-level, near-infrared laser stimulation vs. focused shock wave therapy. Restor Neurol Neurosci. 2016;34(4):561-9. Epub 2016/04/16. doi: 10.3233/rnn-150624. PubMed PMID: 27080072.

2219. Willemse RB, Hillebrand A, Ronner HE, Vandertop WP, Stam CJ. Magnetoencephalographic study of hand and foot sensorimotor

organization in 325 consecutive patients evaluated for tumor or epilepsy surgery. Neuroimage-Clinical. 2016;10:46-53. doi: 10.1016/j.nicl.2015.11.002. PubMed PMID: WOS:000373189000005.

2220. Wolf JA, Koch PF. Disruption of Network Synchrony and Cognitive Dysfunction After Traumatic Brain Injury. Front Syst Neurosci. 2016;10:43. Epub 2016/06/01. doi: 10.3389/fnsys.2016.00043. PubMed PMID: 27242454; PubMed Central PMCID: PMCPMC4868948.

2221. Woods DL, Wyma JM, Herron TJ, Yund EW. The Effects of Repeat Testing, Malingering, and Traumatic Brain Injury on Computerized Measures of Visuospatial Memory Span. Frontiers in Human Neuroscience. 2016;9. doi: 10.3389/fnhum.2015.00690. PubMed PMID: WOS:000367645700001.

2222. Xia X, Yang Y, Bai Y, He J. Transcranial magnetic stimulation combined with high -density EEG in assessment of patients with disorders of consciousness. Chinese Journal of Neuromedicine. 2016;15(4):425-8. PubMed PMID: CSCD:5689961.

2223. Yang B, Wang Z, Sheng C, Wang Y, Zhou J, Xiong XG, et al. Evidence-based review of oral traditional Chinese medicine compound recipe administration for treating weight drop-induced experimental traumatic brain injury. BMC Complementary and Alternative Medicine. 2016;16(1). doi: 10.1186/s12906-016-1076-2.

2224. Yang C, Wang L, Feng Z. Arousal effects of median nerve electrical stimulation on coma patients after traumatic brain injury. Chinese Journal of Rehabilitation Medicine. 2016;31(11):1195-9,207. PubMed PMID: CSCD:5850560.

2225. Yang N, Yue Y, Pan JZ, Zuo MZ, Shi Y, Zhou SZ, et al. Changes in the Bispectral Index in Response to Loss of Consciousness and No Somatic Movement to Nociceptive Stimuli in Elderly Patients. Chin Med J (Engl). 2016;129(4):410-6. Epub 2016/02/18. doi: 10.4103/0366-6999.176083. PubMed PMID: 26879014; PubMed Central PMCID: PMCPMC4800841.

2226. Yang Y, Cheng X, Xu Q, Li R, Liu Z, Wang L, et al. The maintenance of modified electroconvulsive therapy combined with risperidone is better than risperidone alone in preventing relapse of schizophrenia and improving cognitive function. Arquivos de Neuro-Psiquiatria. 2016;74(10):823-8. doi: 10.1590/0004-282X20160130.

2227. Yoon KJ, Lee YT, Chae SW, Park CR, Kim DY. Effects of anodal transcranial direct current stimulation (tDCS) on behavioral and spatial memory during the early stage of traumatic brain injury in the rats. J Neurol Sci. 2016;362:314-20. Epub 2016/03/06. doi: 10.1016/j.jns.2016.02.005. PubMed PMID: 26944170.

2228. You W, Tang Q, Wang L, Lei J, Feng J, Mao Q, et al. Effect of

right median nerve electric stimulation on brain electrical activity of traumatic coma patients. Chinese Journal of Trauma. 2016;32(2):120-3. PubMed PMID: CSCD:5626226.

2229. Yozbatiran N, Keser Z, Davis M, Stampas A, O'Malley MK, Cooper-Hay C, et al. Transcranial direct current stimulation (tDCS) of the primary motor cortex and robot-assisted arm training in chronic incomplete cervical spinal cord injury: a proof of concept sham-randomized clinical study. NeuroRehabilitation. 2016;39(3):401-11. doi: 10.3233/NRE-161371. PubMed PMID: CN-01210967.

2230. Zarea A, Charbonnier C, Rovelet-Lecrux A, Nicolas G, Rousseau S, Borden A, et al. Seizures in dominantly inherited Alzheimer disease. Neurology. 2016;87(9):912-9. doi: 10.1212/WNL.0000000000003048.

2231. Zhu DD, Wang QL, Liu XB, Guo XD. Rapidly separating microneedles for transdermal drug delivery. Acta Biomaterialia. 2016;41:312-9. doi: 10.1016/j.actbio.2016.06.005. PubMed PMID: WOS:000381950500029.

2232. Zhu J, Shen Q, Cao Y, Chen X, Zhao X. The fabrication and property of a novel coated out-of-plane microneedle arrays. Microsystem Technologies-Micro-and Nanosystems-Information Storage and Processing Systems. 2016;22(1):143-9. doi:

10.1007/s00542-015-2448-0. PubMed PMID: WOS:000367812600020.

2233. Zong H, Ma F, Zhang L, Lu H, Gong J, Cai M, et al. Hindlimb spasticity after unilateral motor cortex lesion in rats is reduced by contralateral nerve root transfer. Bioscience Reports. 2016;36(6). doi: 10.1042/BSR20160412.

2234. Zou J, Wang M, Uchiumi O, Shui Y, Ishigaki Y, Liu X, et al. Learning impairment by minimal cortical injury in a mouse model of Alzheimer's disease. Brain Research. 2016;1637:56-63. doi: 10.1016/j.brainres.2016.02.017. PubMed PMID: WOS:000374610700005.

2235. 25. Jahrestagung der Deutschen Gesellschaft fur Neurorehabilitation e. V., DGNR 2017. Neurologie und rehabilitation. 2017;23. PubMed PMID: CN-01462366.

2236. Abstracts of the 62nd National Congress of the Italian Society for Clinical Neurophysiology. Clinical Neurophysiology. 2017;128(12).

2237. Abstracts of the 61st Scientific Annual Meeting of the German Society for Clinical Neurophysiology and Functional Imaging, DGKN 2017. Clinical Neurophysiology. 2017;128(10).

2238. 2017 Symposium “Brain Plasticity in Epilepsy”. Acta Neurologica Belgica. 2017;117(3).

2239. International Society for ECT and Neurostimulation Annual Meeting Abstracts 2017. Journal of ECT. 2017;33(3).
2240. Abreu V, Vaz R, Rebelo V, Rosas MJ, Chamadoira C, Gillies MJ, et al. Thalamic Deep Brain Stimulation for Neuropathic Pain: efficacy at Three Years' Follow-Up. Neuromodulation. 2017;20(5):504-13. doi: 10.1111/ner.12620. PubMed PMID: CN-01396083.
2241. Actrn. Theta Burst Stimulation in fibromyalgia. https://trialsearchwhoint/Trial2aspx?TrialID=ACTRN12617000487314. 2017. PubMed PMID: CN-01817044.
2242. Adamson M, Main K, Milazzo AC, Bickart K, Furst A, Ashford J, et al. Detection and treatment of chronic sequalae of brain injury in veterans. Journal of Head Trauma Rehabilitation. 2017;32(6):E95. doi: 10.1097/HTR.0000000000000241.
2243. Ahn S, Son BC, Choi JG, Ha SW, Ko HC. Long term results of simultaneous trial of deep brain and motor cortex stimulation in refractory neuropathic pain. Stereotactic and Functional Neurosurgery. 2017;95:347. doi: 10.1159/000478281.
2244. Alohali M, Alhussien A, Alghamdi F, Alatawi A, Almusned T, Al-Hussain F, et al. Safety and tolerability of transcranial direct current stimulation in midle east. Brain stimulation. 2017;10(4):e46-e7. doi: 10.1016/j.brs.2017.04.094. PubMed PMID: CN-01399454.

2245. An M, Liu H. Dissolving Microneedle Arrays for Transdermal Delivery of Amphiphilic Vaccines. Small. 2017;13(26). doi: 10.1002/smll.201700164. PubMed PMID: WOS:000404986200005.
2246. Andrabi SS, Parvez S, Tabassum H. Neurosteroids and ischemic stroke: Progesterone a promising agent in reducing the brain injury in Ischemic stroke. Journal of Environmental Pathology, Toxicology and Oncology. 2017;36(3):191-205. doi: 10.1615/JEnvironPatholToxicolOncol.2017017156.
2247. Antal A, Paulus W, Rohde V. New Results on Brain Stimulation in Chronic Pain. Aktuelle Neurologie. 2017;44(10):728-32. doi: 10.1055/s-0043-119975.
2248. Attard Trevisan A, Rotonda M, Antigua L, Cavallari P. Towards a personalised model for transcranial direct current stimulation (tDCS). Brain Stimulation. 2017;10(2):378. doi: 10.1016/j.brs.2017.01.119.
2249. Bai Y, Xia X, Kang J, Yang Y, He J, Li X. TDCS modulates cortical excitability in patients with disorders of consciousness. Neuroimage Clin. 2017;15:702-9. Epub 2017/07/14. doi: 10.1016/j.nicl.2017.01.025. PubMed PMID: 28702347; PubMed Central PMCID: PMCPMC5487253.
2250. Bai Y, Xia X, Li X. A Review of Resting-State Electroencephalography Analysis in Disorders of Consciousness.

Frontiers in Neurology. 2017;8. doi: 10.3389/fneur.2017.00471. PubMed PMID: WOS:000410076900002.

2251. Banús J, Martens G, Salvador R, Thibaut A, Ripolles O, Antonopoulos G, et al. The effects of lesions in e-field distribution during frontoparietal TDCS. Brain Stimulation. 2017;10(2):442-3. doi: 10.1016/j.brs.2017.01.317.

2252. Bassi MS, Gilio L, Buttari F, Maffei P, Marfia GA, Restivo DA, et al. Remodeling functional connectivity in multiple sclerosis: A challenging therapeutic approach. Frontiers in Neuroscience. 2017;11(DEC). doi: 10.3389/fnins.2017.00710.

2253. Bernier RA, Roy A, Venkatesan UM, Grossner EC, Brenner EK, Hillary FG. Dedifferentiation Does not account for hyperconnectivity after Traumatic Brain injury. Frontiers in Neurology. 2017;8. doi: 10.3389/fneur.2017.00297. PubMed PMID: WOS:000406481700001.

2254. Bestmann S, Ward N. Are current flow models for transcranial electrical stimulation fit for purpose? Brain Stimulation. 2017;10(4):865-6. doi: 10.1016/j.brs.2017.04.002.

2255. Bilotta F, Lauretta MP, Tewari A, Haque M, Hara N, Uchino H, et al. Insulin and the Brain : A Sweet Relationship with Intensive Care. Journal of Intensive Care Medicine. 2017;32(1):48-58. doi: 10.1177/0885066615594341.

2256. Bivona G, Sasso BL, Agnello L, Ciaccio M. From the blood-brain barrier to behavior. Acta Neuropsychologica. 2017;15(1):63-7. doi: 10.5604/12321966.1237451.

2257. Bockbrader MA. Physician-monitored home tDCS for post-brain injury fatigue, depression and headache: A case report. Brain Stimulation. 2017;10(4):e33-e4. doi: 10.1016/j.brs.2017.04.052.

2258. Bodart O, Amico E, Wannez S, Gomez F, Casarotto S, Rosanova M, et al. Global structural and effective connectivity in patients with chronic disorders of consciousness. Brain Stimulation. 2017;10(2):353. doi: 10.1016/j.brs.2017.01.035.

2259. Bodart O, Gosseries O, Wannez S, Thibaut A, Annen J, Boly M, et al. Measures of metabolism and complexity in the brain of patients with disorders of consciousness. Neuroimage Clin. 2017;14:354-62. Epub 2017/02/28. doi: 10.1016/j.nicl.2017.02.002. PubMed PMID: 28239544; PubMed Central PMCID: PMCPMC5318348.

2260. Boes A, Labar D, Kuceyeski A, Pascual-Leone A, Fox M, Edwards D. Lesion mapping of bilateral motor threshold in chronic post-stroke hemiparesis. Neurology. 2017;88(16).

2261. Bognár A, Csete G, Németh M, Csibri P, Kincses TZ, Sáry G. Transcranial stimulation of the orbitofrontal cortex affects decisions about magnocellular optimized stimuli. Frontiers in Neuroscience.

2017;11(APR). doi: 10.3389/fnins.2017.00234.

2262. Bologna M, Berardelli A. Cerebellum: An explanation for dystonia? Cerebellum and Ataxias. 2017;4(1). doi: 10.1186/s40673-017-0064-8.

2263. Boly M, Massimini M, Tsuchiya N, Postle BR, Koch C, Tononi G. Are the Neural Correlates of Consciousness in the Front or in the Back of the Cerebral Cortex? Clinical and Neuroimaging Evidence. Journal of Neuroscience. 2017;37(40):9603-13. doi: 10.1523/jneurosci.3218-16.2017. PubMed PMID: WOS:000412182900002.

2264. Bragina O, Lara D, Yang Y, Shuttleworth C, Nemoto E, Bragin D. REPETITIVE ANODAL TRANSCRANIAL DIRECT CURRENT STIMULATION IMPROVES OUTCOME AT THREE WEEKS BUT NOT ONE WEEK AFTER TBI IN MICE. Journal of Neurotrauma. 2017;34(13):A71-A2. PubMed PMID: WOS:000404530400193.

2265. Bragina OA, Lara DA, Yang Y, Nemoto EE, Shuttleworth CW, Semyachkina-Glushkovskaya OV, et al. Anodal transcranial direct current stimulation increases cerebral blood flow, tissue oxygenation and improves neurologic outcome in mice after traumatic brain injury. Brain Stimulation. 2017;10(4):e75-e6. doi: 10.1016/j.brs.2017.04.115.

2266. Bragina OA, Yang Y, Shuttleworth CW, Nemoto EM, Semyachkina-Glushkovskaya OV, Bragin DE. Repetitive anodal

transcranial direct current stimulation (tDCS) improves neurologic outcome in mice after traumatic brain injury (TBI) by increasing cerebral blood flow and tissue oxygenation. Journal of Cerebral Blood Flow and Metabolism. 2017;37:408-. PubMed PMID: WOS:000400157400588.

2267. Branscheidt M, Hoppe J, Freundlieb N, Zwitserlood P, Liuzzi G. tDCS over the motor cortex shows differential effects on action and object words in associative word learning in healthy aging. Frontiers in Aging Neuroscience. 2017;9(MAY). doi: 10.3389/fnagi.2017.00137.

2268. Buch ER, Liew S-L, Cohen LG. Plasticity of Sensorimotor Networks: Multiple Overlapping Mechanisms. Neuroscientist. 2017;23(2):185-96. doi: 10.1177/1073858416638641. PubMed PMID: WOS:000398048800011.

2269. Cakmak YO, Ekinci G, Heinecke A, Cavdar S. A Possible Role of Prolonged Whirling Episodes on Structural Plasticity of the Cortical Networks and Altered Vertigo Perception: The Cortex of Sufi Whirling Dervishes. Frontiers in Human Neuroscience. 2017;11. doi: 10.3389/fnhum.2017.00003. PubMed PMID: WOS:000392444300001.

2270. Celnik P. The role of the cerebellum on motor recovery following stroke. Brain Stimulation. 2017;10(2):500. doi:

10.1016/j.brs.2017.01.459.

2271. Cha M, Um SW, Kwon M, Nam TS, Lee BH. Repetitive motor cortex stimulation reinforces the pain modulation circuits of peripheral neuropathic pain. Scientific Reports. 2017;7. doi: 10.1038/s41598-017-08208-2. PubMed PMID: WOS:000407443300034.

2272. Chantigian D, Rich T, Chen C-Y, Lixandrao M, Gillick B, Keller-Ross M. The Effect of Transcranial Magnetic Stimulation on the Autonomic Nervous System in Pediatric Stroke. Faseb Journal. 2017;31. PubMed PMID: WOS:000405986502579.

2273. Chantsoulis M, Półrola P, Góral-Półrola J, Hajdukiewicz A, Supiński J, Kropotov JD, et al. Application of ERPs neuromarkers for assessment and treatment of a patient with chronic crossed aphasia after severe TBI and long-term coma - Case Report. Ann Agric Environ Med. 2017;24(1):141-7. Epub 2017/04/06. doi: 10.5604/12321966.1232770 PubMed PMID: 28378976.

2274. Chantsoulis M, Półrola P, Góral-Półrola J, Hajdukiewicz A, Supiński J, Kropotov JD, et al. Application of ERPs neuromarkers for assessment and treatment of a patient with chronic crossed aphasia after severe TBI and long-term coma – Case report. Annals of Agricultural and Environmental Medicine. 2017;24(1):141-7. doi: 10.5604/12321966.1232770.

2275. Chari A, Hentall ID, Papadopoulos MC, Pereira EA. Surgical Neurostimulation for Spinal Cord Injury. Brain Sci. 2017;7(2). Epub 2017/02/18. doi: 10.3390/brainsci7020018. PubMed PMID: 28208601; PubMed Central PMCID: PMCPMC5332961.
2276. Chen M-C, Chan H-A, Ling M-H, Su L-C. Implantable polymeric microneedles with phototriggerable properties as a patient-controlled transdermal analgesia system. Journal of Materials Chemistry B. 2017;5(3):496-503. doi: 10.1039/c6tb02718k. PubMed PMID: WOS:000394431600012.
2277. Cho IT, Cho YW, Kwak SG, Chang MC. Comparison between ultrasound-guided interfascial pulsed radiofrequency and ultrasound-guided interfascial block with local anesthetic in myofascial pain syndrome of trapezius muscle. Medicine (United States). 2017;96(5). doi: 10.1097/MD.0000000000006019.
2278. Conde V, Andreasen SH, Petersen TH, Larsen KB, Madsen K, Andersen KW, et al. Alterations in the brain's connectome during recovery from severe traumatic brain injury: protocol for a longitudinal prospective study. BMJ Open. 2017;7(6):e016286. Epub 2017/06/16. doi: 10.1136/bmjopen-2017-016286. PubMed PMID: 28615277; PubMed Central PMCID: PMCPMC5541610.
2279. Crosson B, Hampstead BM, Krishnamurthy LC, Krishnamurthy V, McGregor KM, Nocera JR, et al. Advances in

neurocognitive rehabilitation research from 1992 to 2017: The ascension of neural plasticity. Neuropsychology. 2017;31(8):900-20. Epub 2017/09/01. doi: 10.1037/neu0000396. PubMed PMID: 28857600; PubMed Central PMCID: PMCPMC5788715.

2280. Ctri. Effect of Magnet treatment in Head injury patients. https://trialsearchwhoint/Trial2aspx?TrialID=CTRI/2017/02/007789. 2017. PubMed PMID: CN-01818882.

2281. D'Imperio D, Tomelleri G, Moretto G, Moro V. Modulation of somatoparaphrenia following left-hemisphere damage. Neurocase. 2017;23(2):162-70. doi: 10.1080/13554794.2017.1329444.

2282. Da Silva A. New insights in vivo into the treatment of migraine and other chronic pain disorders using non-invasive neuromodulation. Brain Stimulation. 2017;10(2):387. doi: 10.1016/j.brs.2017.01.143.

2283. Dang B, Chen W, He W, Chen G. Rehabilitation Treatment and Progress of Traumatic Brain Injury Dysfunction. Neural Plast. 2017;2017:1582182. Epub 2017/05/12. doi: 10.1155/2017/1582182. PubMed PMID: 28491478; PubMed Central PMCID: PMCPMC5405588.

2284. Dario A, Baldeschi GC, DeCarolis G, Luxardo N, Nosella P, Natale MNMNM, et al. New implantable neuromodulation system for peripheral nerve stimulation: Efficacy on pain and mental status.

Neuromodulation. 2017;20(7):e210. doi: 10.1111/ner.12639.
2285. de Amorim RLO, Brunoni AR, de Oliveira MAF, Zaninotto ALC, Nagumo MM, Guirado VMP, et al. Transcranial Direct Current Stimulation for Post-Concussion Syndrome: Study Protocol for a Randomized Crossover Trial. Front Neurol. 2017;8:164. Epub 2017/05/18. doi: 10.3389/fneur.2017.00164. PubMed PMID: 28512443; PubMed Central PMCID: PMCPMC5411433.
2286. de Oliveira FF, Correia Marin SdM, Ferreira Bertolucci PH. Neurological impressions on the organization of language networks in the human brain. Brain Injury. 2017;31(2):140-50. doi: 10.1080/02699052.2016.1199914. PubMed PMID: WOS:000395163500002.
2287. Debruyne P, Wijns W. Cardio-Neuromodulation: The Right-Sided Approach. JACC Clin Electrophysiol. 2017;3(9):1056-7. Epub 2018/05/16. doi: 10.1016/j.jacep.2016.12.027. PubMed PMID: 29759711.
2288. Demuth HU, Dijkhuizen RM, Farr TD, Gelderblom M, Horsburgh K, Iadecola C, et al. Recent progress in translational research on neurovascular and neurodegenerative disorders. Restorative Neurology and Neuroscience. 2017;35(1):87-103. doi: 10.3233/RNN-160690.
2289. Dimitri D, De Filippis D, Galetto V, Zettin M. Evaluation of

the effectiveness of transcranial direct current stimulation (tDCS) and psychosensory stimulation through DOCS scale in a minimally conscious subject. Neurocase. 2017;23(2):96-104. Epub 2017/03/30. doi: 10.1080/13554794.2017.1305112. PubMed PMID: 28347207.

2290. Dong X, Wei T, Papa EV, Feng Z. Median nerve electrical stimulation causes wake-promotion by downregulating the expression of gamma-aminobutyric acid b receptor via orexins pathway in comatose rats caused by traumatic brain injury. International Journal of Clinical and Experimental Medicine. 2017;10(10):14571-8. PubMed PMID: WOS:000414299700046.

2291. Dong X, Wei T, Papa EV, Feng Z. Median nerve electrical stimulation causes wake-promotion by downregulating the expression of γ-aminobutyric acid b receptor via orexins pathway in comatose rats caused by traumatic brain injury. International Journal of Clinical and Experimental Medicine. 2017;10(10):14571-8.

2292. Du Q, Feng Z. The effects of electrical stimulation on the expression of H1 receptor in the prefrontal cortex of brain injured, comatose rats. Chinese Journal of Physical Medicine and Rehabilitation. 2017;39(2):81-5. PubMed PMID: CSCD:5932756.

2293. Eapen BC, Murphy DP, Cifu DX. Neuroprosthetics in amputee and brain injury rehabilitation. Experimental Neurology. 2017;287:479-85. doi: 10.1016/j.expneurol.2016.08.004. PubMed

PMID: WOS:000391158800006.
2294. Edwards EK, Christie AD. Assessment of motor cortex excitability and inhibition during a cognitive task in individuals with concussion. Brain Inj. 2017;31(10):1348-55. Epub 2017/06/29. doi: 10.1080/02699052.2017.1327671. PubMed PMID: 28657363.
2295. Eisner W, Quirbach S, Sohm F, Kerschbaumer J. Possible reason for the loss of effectivity of motor cortex stimulation. Stereotactic and Functional Neurosurgery. 2017;95:368. doi: 10.1159/000478281.
2296. Estraneo A, Fiorenza S, Pascarella A, Moretta P, Masotta O, Loreto V, et al. Transcranial direct current stimulation in prolonged disorders of consciousness. Clinical neurophysiology. 2017;128(12):e417-. doi: 10.1016/j.clinph.2017.09.016. PubMed PMID: CN-01438055.
2297. Estraneo A, Pascarella A, Moretta P, Masotta O, Fiorenza S, Chirico G, et al. Repeated transcranial direct current stimulation in prolonged disorders of consciousness: A double-blind cross-over study. J Neurol Sci. 2017;375:464-70. Epub 2017/03/23. doi: 10.1016/j.jns.2017.02.036. PubMed PMID: 28320187.
2298. Estraneo A, Pascarella A, Moretta P, Masotta O, Fiorenza S, Chirico G, et al. Transcranial direct current stimulation in prolonged disorders of consciousness: a randomized controlled pilot study. Brain

injury. 2017;31(6-7):855-. doi: 10.1080/02699052.2017.1312145. PubMed PMID: CN-01399411.

2299. Ferrari PF, Barbot A, Bianchi B, Ferri A, Garofalo G, Bruno N, et al. A proposal for new neurorehabilitative intervention on Moebius Syndrome patients after 'smile surgery'. Proof of concept based on mirror neuron system properties and hand-mouth synergistic activity. Neuroscience and Biobehavioral Reviews. 2017;76:111-22. doi: 10.1016/j.neubiorev.2017.01.050.

2300. Fomina T, Weichwald S, Synofzik M, Just J, Schols L, Scholkopf B, et al. Absence of EEG correlates of self-referential processing depth in ALS. PLoS ONE. 2017;12(6). doi: 10.1371/journal.pone.0180136.

2301. Gao YL, Zhang Y, Cao JP, Wu SB, Cai XH, Zhang YC, et al. Regulation of the endoplasmic reticulum stress response and neuroprotective effects of acupuncture on brain injury caused by heroin addiction. Acupunct Med. 2017;35(5):366-73. Epub 2017/05/27. doi: 10.1136/acupmed-2016-011220. PubMed PMID: 28546432.

2302. Garcia-Rill E. Bottom-up gamma and stages of waking. Medical Hypotheses. 2017;104:58-62. doi: 10.1016/j.mehy.2017.05.023.

2303. Gilmore N, Dwyer M, Kiran S. A systematic review of

standardized outcome measures used in aphasia rehabilitation, including meta-analyses. Archives of Physical Medicine and Rehabilitation. 2017;98(10):e137. doi: 10.1016/j.apmr.2017.08.447.

2304. Ginanneschi F, Mignarri A, Lucchiari S, Ulzi G, Comi GP, Rossi A, et al. Neuromuscular excitability changes produced by sustained voluntary contraction and response to mexiletine in myotonia congenita. Neurophysiologie Clinique. 2017;47(3):247-52. doi: 10.1016/j.neucli.2017.01.003.

2305. Giordano J, Bikson M, Kappenman ES, Clark VP, Coslett HB, Hamblin MR, et al. Mechanisms and effects of transcranial direct current stimulation. Dose-Response. 2017;15(1). doi: 10.1177/1559325816685467.

2306. Goadsby PJ, Holland PR, Martins-Oliveira M, Hoffmann J, Schankin C, Akerman S. Pathophysiology of migraine: A disorder of sensory processing. Physiological Reviews. 2017;97(2):553-622. doi: 10.1152/physrev.00034.2015.

2307. Gould TD, Georgiou P, Brenner LA, Brundin L, Can A, Courtet P, et al. Animal models to improve our understanding and treatment of suicidal behavior. Translational Psychiatry. 2017;7. doi: 10.1038/tp.2017.50. PubMed PMID: WOS:000402329700007.

2308. Guo F, Lou J, Han X, Deng Y, Huang X. Repetitive transcranial magnetic stimulation ameliorates cognitive impairment by

enhancing neurogenesis and suppressing apoptosis in the hippocampus in rats with ischemic stroke. Frontiers in Physiology. 2017;8(AUG). doi: 10.3389/fphys.2017.00559.

2309. Guo J, Garrido L, Liu RR, Susilo T, Barton JJS, Duchaine B. Normal voice processing after posterior superior temporal sulcus lesion. Neuropsychologia. 2017;105:215-22. doi: 10.1016/j.neuropsychologia.2017.03.008. PubMed PMID: WOS:000416185000019.

2310. Hanna K, Sambrook P, Armfield J, Brennan D. Online resources provision and wisdom teeth extractions: a pilot randomized controlled trial. Journal of dental research. 2017;96(Spec Iss B). PubMed PMID: CN-02344379.

2311. Hara T, Abo M, Sasaki N, Yamada N, Niimi M, Kenmoku M, et al. Improvement of higher brain dysfunction after brain injury by repetitive transcranial magnetic stimulation and intensive rehabilitation therapy: case report. Neuroreport. 2017;28(13):800-7. Epub 2017/07/14. doi: 10.1097/wnr.0000000000000830. PubMed PMID: 28704292.

2312. Henderson TA, Morries LD. Multi-Watt Near-Infrared Phototherapy for the Treatment of Comorbid Depression: An Open-Label Single-Arm Study. Frontiers in Psychiatry. 2017;8. doi: 10.3389/fpsyt.2017.00187. PubMed PMID: WOS:000411935500001.

2313. Henrich-Noack P, Sergeeva EG, Sabel BA. Non-invasive electrical brain stimulation: From acute to late-stage treatment of central nervous system damage. Neural Regeneration Research. 2017;12(10):1590-4. doi: 10.4103/1673-5374.217322.

2314. Higginson CI, Thompson T, Benjamin AE, Rosales AC, Zane KL, Valenti M, et al. Construct validity of the Functional Assessment of Verbal Reasoning and Executive Strategies (FAVRES). Brain Injury. 2017;31(13-14):1807-12. doi: 10.1080/02699052.2017.1347278.

2315. Hone-Blanchet A, Mondino M, Fecteau S. Repetitive transcranial magnetic stimulation reduces anxiety symptoms, drug cravings, and elevates 1H-MRS brain metabolites: A case report. Brain Stimulation. 2017;10(4):856-8. doi: 10.1016/j.brs.2017.03.007.

2316. Hsieh TH, Lee HHC, Hameed MQ, Pascual-Leone A, Hensch TK, Rotenberg A. Trajectory of Parvalbumin Cell Impairment and Loss of Cortical Inhibition in Traumatic Brain Injury. Cereb Cortex. 2017;27(12):5509-24. Epub 2016/12/03. doi: 10.1093/cercor/bhw318. PubMed PMID: 27909008; PubMed Central PMCID: PMCPMC6075565.

2317. Huang C-J, Huang Y-H, Lin C-H. Factors Related to the Changes in Quality of Life for Patients With Depression After an Acute Course of Electroconvulsive Therapy. Journal of Ect. 2017;33(2):126-33. doi: 10.1097/yct.0000000000000358. PubMed

PMID: WOS:000401798400019.
2318. Huang MX, Swan AR, Quinto AA, Matthews S, Harrington DL, Nichols S, et al. A pilot treatment study for mild traumatic brain injury: Neuroimaging changes detected by MEG after low-intensity pulse-based transcranial electrical stimulation. Brain Inj. 2017;31(13-14):1951-63. Epub 2017/09/20. doi: 10.1080/02699052.2017.1363409. PubMed PMID: 28925734.
2319. Huang Z, Thiebaud N, Fadool DA. Differential serotonergic modulation across the main and accessory olfactory bulbs. J Physiol. 2017;595(11):3515-33. Epub 2017/02/24. doi: 10.1113/jp273945. PubMed PMID: 28229459; PubMed Central PMCID: PMCPMC5451723.
2320. Hurme M, Koivisto M, Revonsuo A, Railo H. Early processing in primary visual cortex is necessary for conscious and unconscious vision while late processing is necessary only for conscious vision in neurologically healthy humans. Neuroimage. 2017;150:230-8. Epub 2017/03/04. doi: 10.1016/j.neuroimage.2017.02.060. PubMed PMID: 28254455.
2321. Ilyichev VP, Martynov IV, Gorelik AL, Naryshkin AG. Experience of complex application of neuromodulation technologies in cases of severe cerebral lesions. Brain Stimulation. 2017;10(2):406-7. doi: 10.1016/j.brs.2017.01.206.

2322. Irct2017011932053N. Investigating the effect of rehabilitation with and without rTMS (repetitive transcranial magnetic stimulation) on upper extremity motor deficit of traumatic brain injury. https://trialsearchwhoint/Trial2aspx?TrialID=IRCT2017011932053N1. 2017. PubMed PMID: CN-01807694.

2323. Irct2017020311885N. Effects of Transcranial direct currency stimulation (tDCS) on the treatment of depressive and anxiety symptoms and improve quality of sleep in patients with chronic renal diseases on dialysis. https://trialsearchwhoint/Trial2aspx?TrialID=IRCT2017020311885N8. 2017. PubMed PMID: CN-01840221.

2324. Irct2017030532884N. Effect of combining tDCS and behavioral intervention on children with autism spectrum disorder. https://trialsearchwhoint/Trial2aspx?TrialID=IRCT2017030532884N1. 2017. PubMed PMID: CN-01815633.

2325. Ismail FY, Fatemi A, Johnston MV. Cerebral plasticity: Windows of opportunity in the developing brain. Eur J Paediatr Neurol. 2017;21(1):23-48. Epub 2016/08/28. doi: 10.1016/j.ejpn.2016.07.007. PubMed PMID: 27567276.

2326. Jablawi F, Kweider H, Nikoubashman O, Clusmann H, Schubert GA. Twist Drill Procedure for Chronic Subdural Hematoma Evacuation: An Analysis of Predictors for Treatment Success. World

Neurosurg. 2017;100:480-6. Epub 2017/01/23. doi: 10.1016/j.wneu.2017.01.037. PubMed PMID: 28109862.

2327. Jackson MP, Bikson M, Liebetanz D, Nitsche M. Toward comprehensive tDCS safety standards. Brain Behavior and Immunity. 2017;66:413-. doi: 10.1016/j.bbi.2017.08.007. PubMed PMID: WOS:000414236600038.

2328. Jalan A, Shinde D, Kudalkar K, Jalan R, Borugale M, Tawde R, et al. Carnitine palmitoyl transferase deficiency in Indian patients. Journal of Inborn Errors of Metabolism and Screening. 2017;5:219. doi: 10.1177/2326409817722292.

2329. Jobst BC, Kapur R, Barkley GL, Bazil CW, Berg MJ, Bergey GK, et al. Brain-responsive neurostimulation in patients with medically intractable seizures arising from eloquent and other neocortical areas. Epilepsia. 2017;58(6):1005-14. doi: 10.1111/epi.13739.

2330. Jung D, Rejinold NS, Kwak J-E, Park S-H, Kim Y-C. Nano-patterning of a stainless steel microneedle surface to improve the dip-coating efficiency of a DNA vaccine and its immune response. Colloids and Surfaces B-Biointerfaces. 2017;159:54-61. doi: 10.1016/j.colsurfb.2017.07.059. PubMed PMID: WOS:000418729600008.

2331. Kamins J, Bigler E, Covassin T, Henry L, Kemp S, Leddy JJ,

et al. What is the physiological time to recovery after concussion? A systematic review. Br J Sports Med. 2017;51(12):935-40. Epub 2017/04/30. doi: 10.1136/bjsports-2016-097464. PubMed PMID: 28455363.
2332. Kaminski E, Hoff M, Rjosk V, Steele CJ, Gundlach C, Sehm B, et al. Anodal transcranial direct current stimulation does not facilitate dynamic balance task learning in healthy old adults. Frontiers in human neuroscience. 2017;11(no pagination). doi: 10.3389/fnhum.2017.00016. PubMed PMID: CN-01336954.
2333. Kendall DA, Yudowski GA. Cannabinoid Receptors in the Central Nervous System: Their Signaling and Roles in Disease. Frontiers in Cellular Neuroscience. 2017;10. doi: 10.3389/fncel.2016.00294. PubMed PMID: WOS:000391218600001.
2334. Khan IR, Rehan M, Badjatia N, Parikh G, Kon Z, Herr D, et al. Near infrared spectroscopy as a non-invasive brain injury monitor in adult extracorporeal membrane oxygenation. Neurocritical Care. 2017;27(2):S248. doi: 10.1007/s12028-017-0465-9.
2335. Khan S, Fusco H. Review of Non-invasive Brain Stimulation and Stem Cells in the Treatment of Traumatic Brain Injury. Journal of Head Trauma Rehabilitation. 2017;32(6):E80-E. PubMed PMID: WOS:000418588100041.
2336. Kim HJ, Han SJ. Anodal Transcranial Direct Current

Stimulation Provokes Neuroplasticity in Repetitive Mild Traumatic Brain Injury in Rats. Neural Plast. 2017;2017:1372946. Epub 2017/08/05. doi: 10.1155/2017/1372946. PubMed PMID: 28770112; PubMed Central PMCID: PMCPMC5523234.

2337. Kimberley TJ, Novak I, Boyd L, Fowler E, Larsen D. Stepping Up to Rethink the Future of Rehabilitation: IV STEP Considerations and Inspirations. Journal of Neurologic Physical Therapy. 2017;41(3):S63-S72. doi: 10.1097/npt.0000000000000182. PubMed PMID: WOS:000404956200010.

2338. King R, Seeger T, Zewdie E, Ciechanski P, Damji O, Kirton A, et al. Longitudinal follow-up of cortical excitability in paediatric traumatic brain injury. Brain Injury. 2017;31(6-7):955-6. doi: 10.1080/02699052.2017.1312145.

2339. Kovacic K, Hainsworth K, Sood M, Chelimsky G, Unteutsch R, Nugent M, et al. Neurostimulation for abdominal pain-related functional gastrointestinal disorders in adolescents: a randomised, double-blind, sham-controlled trial. Lancet Gastroenterol Hepatol. 2017;2(10):727-37. Epub 2017/08/23. doi: 10.1016/s2468-1253(17)30253-4. PubMed PMID: 28826627.

2340. Kraeutner SN, Ingram TGJ, Boe SG. The effector independent nature of motor imagery: Evidence from rTMS induced inhibition to the primary motor cortices. Neuropsychologia. 2017;97:1-8. doi:

10.1016/j.neuropsychologia.2017.01.023.
2341. Kregel E, Walz M, Nelson A, Con-Way T. Intrathecal catheters for targeted drug delivery: Computer modeling of bending the ascenda® catheter. Neuromodulation. 2017;20(7):e236-e7. doi: 10.1111/ner.12639.
2342. Krieg S, Bulubas L, Sollmann N, Tanigawa N, Meyer B. Plasticity of motor representations in patients with brain lesions: A navigated TMS study. Journal of Neurosurgery. 2017;126(4):A1415. doi: 10.3171/2017.4.JNS.AANS2017abstracts.
2343. Kukke SN, Brewer CC, Zalewski C, King KA, Damiano D, Alter KE, et al. Hearing Safety From Single- and Double-Pulse Transcranial Magnetic Stimulation in Children and Young Adults. J Clin Neurophysiol. 2017;34(4):340-7. Epub 2017/06/24. doi: 10.1097/wnp.0000000000000372. PubMed PMID: 28644204; PubMed Central PMCID: PMCPMC5484061.
2344. Kurt E, Henssen D, Steegers M, Staal M, Beese U, Maarrawi J, et al. Motor Cortex Stimulation in Patients Suffering from Chronic Neuropathic Pain: Summary of Expert Meeting and Premeeting Questionnaire, Combined with Literature Review. World Neurosurg. 2017;108:254-63. Epub 2017/09/09. doi: 10.1016/j.wneu.2017.08.168. PubMed PMID: 28882715.
2345. Landgraf MN, König H, Hannibal I, Langhagen T, Bonfert

MV, Klose B, et al. [Migraine in children and adolescents-brain and muscle? : Another example of why children are not small adults]. Nervenarzt. 2017;88(12):1402-10. Epub 2017/09/29. doi: 10.1007/s00115-017-0428-6. PubMed PMID: 28956079.
2346. Lascano AM, Lalive PH, Hardmeier M, Fuhr P, Seeck M. Clinical evoked potentials in neurology: a review of techniques and indications. Journal of Neurology Neurosurgery and Psychiatry. 2017;88(8):688-96. doi: 10.1136/jnnp-2016-314791. PubMed PMID: WOS:000405379100099.
2347. Lavano A, Guzzi G, M DER, Romano M, Della Torre A, Vescio G, et al. Minimally invasive motor cortex stimulation for Parkinson's disease. J Neurosurg Sci. 2017;61(1):77-87. Epub 2015/04/18. doi: 10.23736/s0390-5616.16.03246-x. PubMed PMID: 25881652.
2348. Lee HY, Kim TW. Clinical effects of intermittent theta-burst stimulation of the anterior temporal lobe to promote visual motor integration and perceptual reasoning in pediatric traumatic brain injury: A case study. Brain Stimulation. 2017;10(2):492-3. doi: 10.1016/j.brs.2017.01.440.
2349. Lee IC, Lin W-M, Shu J-C, Tsai S-W, Chen C-H, Tsai M-T. Formulation of two-layer dissolving polymeric microneedle patches for insulin transdermal delivery in diabetic mice. Journal of

Biomedical Materials Research Part A. 2017;105(1):84-93. doi: 10.1002/jbm.a.35869. PubMed PMID: WOS:000389145400009.

2350. Lee JW, Lee S, Cho HW, Ma Y, Yoo KH, Sung KW, et al. Incorporation of high-dose 131I-metaiodobenzylguanidine treatment into tandem high-dose chemotherapy and autologous stem cell transplantation for high-risk neuroblastoma: results of the SMC NB-2009 study. Journal of Hematology and Oncology. 2017;10(1). doi: 10.1186/s13045-017-0477-0.

2351. Lefaucheur J-P, Antal A, Ayache SS, Benninger DH, Brunelin J, Cogiamanian F, et al. Evidence-based guidelines on the therapeutic use of transcranial direct current stimulation (tDCS). Clinical Neurophysiology. 2017;128(1):56-92. doi: 10.1016/j.clinph.2016.10.087. PubMed PMID: WOS:000396377300008.

2352. Lefebvre S, Liew SL. Anatomical parameters of tDCS to modulate the motor system after stroke: A review. Frontiers in Neurology. 2017;8(FEB). doi: 10.3389/fneur.2017.00029.

2353. Legostaeva L, Mochalova E, Poydasheva A, Chervyakov A, Kremneva E, Sergeev D, et al. Feasibility of individual diagnostic approach for patients with chronic disorders of consciousness. European Journal of Neurology. 2017;24:147. doi: 10.1111/ene.13367.

2354. Lewis GN, Hume PA, Stavric V, Brown SR, Taylor D. New

Zealand rugby health study: motor cortex excitability in retired elite and community level rugby players. N Z Med J. 2017;130(1448):34-44. Epub 2017/01/13. PubMed PMID: 28081555.
2355. Li C, Amrit C, Afridi A, Narayan R. Trigeminal nerve stimulation attenuates secondary brain injury after traumatic brain injury and hemorrhagic shock. Journal of Neurotrauma. 2017;34(13):A123. doi: 10.1089/neu.2017.29011.abstracts.
2356. Li L, Violante I, Zimmerman K, Ross E, Leech R, Hampshire A, et al. EXPLORING THE TARGETED APPLICATION OF TDCS AFTER TBI. Journal of Neurology Neurosurgery and Psychiatry. 2017;88:A82-A. doi: 10.1136/jnnp-2017-ABN.286. PubMed PMID: WOS:000471110600287.
2357. Li LM, Violante I, Zimmerman K, Ross E, Leech R, Hampshire A, et al. Promoting network recovery after traumatic brain injury: exploring the targeted application of transcranial direct current stimulation. Neurology. 2017;88. PubMed PMID: WOS:000577381500017.
2358. Liao L, Teng X, Gao L, Gu L, Zhou B. Effect of Repetitive Transcranial Magnetic Stimulation Combined with Eye-acupuncture on Cognitive Dysfunction after Traumatic Brain Injury. Chinese Journal of Rehabilitation Theory and Practice. 2017;23(1):92-6. PubMed PMID: CSCD:5922145.

2359. Lin C-L, Dumont AS, Zhang JH, Zuccarello M, Chen C-S. Improving and Predicting Outcomes of Traumatic Brain Injury: Neuroplasticity, Imaging Modalities, and Perspective Therapy. Neural Plasticity. 2017;2017. doi: 10.1155/2017/4752546. PubMed PMID: WOS:000404928700001.

2360. Liu LA, Wang ZQ, Fu JJ, Du QQ, Zhang YY, Jiao CC, et al. [Comparative Observation on Electroacupuncture and Manual Acupuncture in Rabbits with Facial Nerve Injury by Electron Microscope]. Zhen Ci Yan Jiu. 2017;42(5):423-8. Epub 2017/11/07. PubMed PMID: 29105471.

2361. Lorente L, Martín MM, Abreu-González P, Pérez-Cejas A, Ramos L, Argueso M, et al. Serum melatonin levels in survivor and non-survivor patients with traumatic brain injury. BMC Neurology. 2017;17(1). doi: 10.1186/s12883-017-0922-2.

2362. Love S, Jaouen V, Annan M, Cottier JP, Vercouillie J, Guilloteau D, et al. Robust supervised clustering analysis of 18F DPA-714 dynamic clinical PET data. Journal of Nuclear Medicine. 2017;58.

2363. Lu CC, Dong SL, Lin HH, Ni YC, Jan ML, Chuang KS. Noninvasive measurement of radiopharmaceutical time-activity data using external thermoluminescent dosimeters (TLDs). Physics in Medicine and Biology. 2017;62(4):N58-N72. doi:

10.1088/1361-6560/aa54a9.

2364. Lu X, Bao X, Li J, Zhang G, Guan J, Gao Y, et al. High-frequency repetitive transcranial magnetic stimulation for treating moderate traumatic brain injury in rats: A pilot study. Exp Ther Med. 2017;13(5):2247-54. Epub 2017/06/02. doi: 10.3892/etm.2017.4283. PubMed PMID: 28565833; PubMed Central PMCID: PMCPMC5443170.

2365. Lynall RC, Pietrosimone B, Kerr ZY, Mauntel TC, Mihalik JP, Guskiewicz KM. Osteoarthritis Prevalence in Retired National Football League Players With a History of Concussion and Lower Extremity Injury. Journal of Athletic Training. 2017;52(6):518-25. doi: 10.4085/1062-6050-52.2.03. PubMed PMID: WOS:000404154700005.

2366. Madsen C, Vaughan M, Koehlmoos TP. Use of Integrative Medicine in the United States Military Health System. Evidence-based Complementary and Alternative Medicine. 2017;2017. doi: 10.1155/2017/9529257.

2367. Mancuso M, Abbruzzese L, Canova S, Landi G, Rossi S, Santarnecchi E. Transcranial Random Noise Stimulation Does Not Improve Behavioral and Neurophysiological Measures in Patients with Subacute Vegetative-Unresponsive Wakefulness State (VS-UWS). Front Hum Neurosci. 2017;11:524. Epub 2017/11/23. doi:

10.3389/fnhum.2017.00524. PubMed PMID: 29163104; PubMed Central PMCID: PMCPMC5681535.

2368. Mandal S, Khandalavala K, Pham R, Bruck P, Varghese M, Kochvar A, et al. Cellulose Acetate Phthalate and Antiretroviral Nanoparticle Fabrications for HIV Pre-Exposure Prophylaxis. Polymers. 2017;9(9). doi: 10.3390/polym9090423. PubMed PMID: WOS:000411524400037.

2369. Marei A, Rashed H. One session of repetitive transcranial magnetic stimulation (rTMS) can improve catatonia features associated with major depressive disorder (MDD): Case study. Brain Stimulation. 2017;10(2):519. doi: 10.1016/j.brs.2017.01.515.

2370. Markiewcz R. The use of EEG Biofeedback/Neurofeedback in psychiatric rehabilitation. Psychiatr Pol. 2017;51(6):1095-106. Epub 2018/02/13. doi: 10.12740/pp/68919. PubMed PMID: 29432505.

2371. Martens G, Thibaut A, Huang W, Di H, Laureys S. Repeated stimulation of the posterior parietal cortex in patients in minimally conscious state: a sham-controlled randomized clinical trial. Brain stimulation. 2017;10(2):370-. doi: 10.1016/j.brs.2017.01.090. PubMed PMID: CN-01361661.

2372. Martens G, Thibaut A, Huang W, Laureys S. Repeated transcranial direct current stimulation of the posterior parietal cortex in patients in minimally conscious state: a shamcontrolled randomized

clinical trial. Brain injury. 2017;31(6-7):753-. doi: 10.1080/02699052.2017.1312145. PubMed PMID: CN-01399420.

2373. Martens G, Thibaut A, Laureys S. Controlled clinical trial of 4 weeks of homebased tDCS in patients with chronic minimally conscious state. Brain injury. 2017;31(6-7):753-4. doi: 10.1080/02699052.2017.1312145. PubMed PMID: CN-01399419.

2374. Maslenikov N, Tsukarzi EE, Mosolov SN. Treatment-resistant depression in schizophrenia: Clinical response predictors of repetitive transcranial magnetic stimulation (rTMS). European Neuropsychopharmacology. 2017;27:S693-S4.

2375. Mattogno PP, Barbagallo G, Iacopino G, Pappalardo G, G LAR, Signorelli F, et al. Recovery from Chronic Diseases of Consciousness: State of the Art in Neuromodulation for Persistent Vegetative State and Minimally Conscious State. Acta Neurochir Suppl. 2017;124:19-25. Epub 2017/01/26. doi: 10.1007/978-3-319-39546-3_4. PubMed PMID: 28120048.

2376. Maurer-Karattup P, Morrissey AM. Comparison of treatment for disorders of consciousness (DOC) across countries-results of an international survey. Brain Injury. 2017;31(6-7):841. doi: 10.1080/02699052.2017.1312145.

2377. McGarry A, Kieburtz K, Abler V, Grachev ID, Gandhi S, Auinger P, et al. Safety and Exploratory Efficacy at 36 Months in

Open-HART, an Open-Label Extension Study of Pridopidine in Huntington's Disease. Journal of huntington's disease. 2017;6(3):189-99. doi: 10.3233/JHD-170241. PubMed PMID: CN-01422679.

2378. Meehan SK, Mirdamadi JL, Martini DN, Broglio SP. Changes in Cortical Plasticity in Relation to a History of Concussion during Adolescence. Front Hum Neurosci. 2017;11:5. Epub 2017/02/02. doi: 10.3389/fnhum.2017.00005. PubMed PMID: 28144218; PubMed Central PMCID: PMCPMC5239801.

2379. Meng ZY, Song WQ. Low frequency repetitive transcranial magnetic stimulation improves motor dysfunction after cerebral infarction. Neural Regeneration Research. 2017;12(4):610-3. doi: 10.4103/1673-5374.205100.

2380. Menolascino SM, Belgin M, Izzo GN, Fisher LE. Maintenance tDCS: A case of full and durable recovery from myalgic encephalomyelitis/chronic fatigue syndrome (ME/CFS). Brain Stimulation. 2017;10(4):e69-e71. doi: 10.1016/j.brs.2017.04.112.

2381. Mitchell C, Bowen A, Tyson S, Butterfint Z, Conroy P. Interventions for dysarthria due to stroke and other adult-acquired, non-progressive brain injury. Cochrane Database Syst Rev. 2017;1(1):Cd002088. Epub 2017/01/26. doi: 10.1002/14651858.CD002088.pub3. PubMed PMID: 28121021;

PubMed Central PMCID: PMCPMC6464736 Institute for Health Research Doctoral Research Fellowship (DRF-2014-07-043) and is registered with the Health and Care Professions Council, UK. Audrey Bowen's salary is part funded by Stroke Association and partly by the National Institute for Health Research Collaboration for Leadership in Applied Health Research and Care (NIHR CLAHRC) Greater Manchester. Audrey Bowen has been involved in a study included in this review (Bowen 2012). She did not contribute to the assessment or interpretation of this study. Sarah Tyson: none known. Zoe Butterfint: none known. Paul Conroy is a speech and language therapist, member of the Royal College of Speech and Language Therapists, and is registered with the Health and Care Professions Council, UK. This report presents independent research funded by the National Institute for Health Research (NIHR). The views expressed are those of the review authors and not necessarily those of the NHS, the NIHR, or the Department of Health.

2382. Mochalova E, Legostaeva L, Poydasheva A, Kremneva E, Chervyakov A, Sergeev D, et al. Decreased level of consciousness in thalamic hemorrhage patient. European Journal of Neurology. 2017;24:539-40. PubMed PMID: WOS:000405530101161.

2383. Moisset X. Noninvasive brain stimulation: a new therapeutic option in chronic pain? Douleur et Analgesie. 2017;30(4):192-8. doi:

10.1007/s11724-017-0532-1.

2384. Moisset X, Bouhassira D. Non-invasive brain neurostimulation for neuropathic pain. Douleurs. 2017;18(2):71-7. doi: 10.1016/j.douler.2017.03.007.

2385. Moore N, Patel B, Zuabi N, Langdorf MI, Rodriguez RM. Feasibility of Informed Consent for Computed Tomography in Acute Trauma Patients. Academic Emergency Medicine. 2017;24(5):637-40. doi: 10.1111/acem.13164.

2386. Moustafa IM, Diab AA, Harrison DE. The effect of normalizing the sagittal cervical configuration on dizziness, neck pain, and cervicocephalic kinesthetic sensibility: a 1-year randomized controlled study. European journal of physical and rehabilitation medicine. 2017;53(1):57-71. doi: 10.23736/S1973-9087.16.04179-4. PubMed PMID: CN-01413906.

2387. Murphy C, Zafar H, Sharif F. An updated review of cardiac devices in heart failure. Ir J Med Sci. 2017;186(4):909-19. Epub 2017/03/28. doi: 10.1007/s11845-017-1597-9. PubMed PMID: 28343276.

2388. Nada M, Ghany WA, Fayed Z, Elbahy K, Ghanem E. Comparative study between the outcomes of neuroablative and neuromodulation techniques in the treatment of secondary dystonia. Stereotactic and Functional Neurosurgery. 2017;95:244. doi:

10.1159/000478281.
2389. Nagy A, Lipoldová J, Novák M, Štěpánová R. Occurrence of implantable cardioverter-defibrillator therapy in clinical practice. Cor et Vasa. 2017;59(3):e215-e21. doi: 10.1016/j.crvasa.2016.12.014.
2390. Naro A, Bruno R, Leo A, Russo M, Salviera C, Bramanti A, et al. Twist and turn into chronic disorders of consciousness: Potential role of the auditory stapedial reflex. Restor Neurol Neurosci. 2017;35(1):77-85. Epub 2017/01/07. doi: 10.3233/rnn-160655. PubMed PMID: 28059800.
2391. Naro A, Leo A, Manuli A, Cannavò A, Bramanti A, Bramanti P, et al. How far can we go in chronic disorders of consciousness differential diagnosis? The use of neuromodulation in detecting internal and external awareness. Neuroscience. 2017;349:165-73. Epub 2017/03/14. doi: 10.1016/j.neuroscience.2017.02.053. PubMed PMID: 28285941.
2392. Naro A, Leo A, Russo M, Casella C, Buda A, Crespantini A, et al. Breakthroughs in the spasticity management: Are non-pharmacological treatments the future? J Clin Neurosci. 2017;39:16-27. Epub 2017/03/07. doi: 10.1016/j.jocn.2017.02.044. PubMed PMID: 28262404.
2393. Naros G, Gharabaghi A. Physiological and behavioral effects of β-tACS on brain self-regulation in chronic stroke. Brain

Stimulation. 2017;10(2):251-9. doi: 10.1016/j.brs.2016.11.003.
2394. Nct. Transcranial Direct Current Stimulation and Computer-based Training for the Traumatic Brain Injury Rehabilitation. https://clinicaltrialsgov/show/NCT03382626. 2017. PubMed PMID: CN-01566963.
2395. Nct. Managing MTBI-related Headaches With rTMS. https://clinicaltrialsgov/show/NCT03314584. 2017. PubMed PMID: CN-01564973.
2396. Nct. Transcranial Magnetic Stimulation in Patients With Disorders of Consciousness. https://clinicaltrialsgov/show/NCT03385278. 2017. PubMed PMID: CN-02038968.
2397. Nct. Neurophysiological Effects of tPCS and tDCS on Patients With Disorders of Consciousness. https://clinicaltrialsgov/show/NCT03115021. 2017. PubMed PMID: CN-01575917.
2398. Nct. Long-term Effect of tDCS in Patients With Disorders of Consciousness. https://clinicaltrialsgov/show/NCT03114397. 2017. PubMed PMID: CN-01575908.
2399. Nct. Transcranial Direct Current Stimulation to Enhance Rehabilitation in Individuals With Rotator Cuff Tendinopathy. https://clinicaltrialsgov/show/NCT03104218. 2017. PubMed PMID:

CN-01563191.
2400. Nicoletti CG, Monteleone F, Marfia GA, Centonze D, Mori F. Enhancing brain plasticity to contrast clinical progression in MS: A pilot study assessing the safety and efficacy of D-Aspartate. Multiple Sclerosis Journal. 2017;23(3):657-8. doi: 10.1177/1352458517731406.
2401. Nuta J, Itonaga M, Tamura T, Kitano M, Hirono S, Kawai M, et al. Usefulness of contrast-enhanced harmonic endoscopic ultrasonography for identification of mural nodules in intraductal papillary mucinous neoplasms. Gastrointestinal Endoscopy. 2017;85(5):AB343.
2402. O'Neil-Pirozzi TM, Doruk D, Thomson JM, Fregni F. Immediate memory and electrophysiologic effects of prefrontal cortex transcranial direct current stimulation on neurotypical individuals and individuals with chronic traumatic brain injury: a pilot study. Int J Neurosci. 2017;127(7):592-600. Epub 2016/07/28. doi: 10.1080/00207454.2016.1216415. PubMed PMID: 27453334.
2403. O'Shea J, Revol P, Cousijn H, Near J, Petitet P, Jacquin-Courtois S, et al. Induced sensorimotor cortex plasticity remediates chronic treatment-resistant visual neglect. Elife. 2017;6. doi: 10.7554/eLife.26602. PubMed PMID: WOS:000410296800001.
2404. Oakley E, May R, Hoeppner T, Sinn K, Furyk J, Craig S, et al.

Computed tomography for head injuries in children: Change in Australian usage rates over time. EMA - Emergency Medicine Australasia. 2017;29(2):192-7. doi: 10.1111/1742-6723.12732.

2405. Odegaard B, Knight RT, Lau H. Should a few null findings falsify prefrontal theories of conscious perception? Journal of Neuroscience. 2017;37(40):9593-602. doi: 10.1523/JNEUROSCI.3217-16.2017.

2406. Ohi K, Kuwata A, Shimada T, Yasuyama T, Nitta Y, Uehara T, et al. Response to benzodiazepines and the clinical course in malignant catatonia associated with schizophrenia: A case report. Medicine (United States). 2017;96(16). doi: 10.1097/MD.0000000000006566.

2407. Okamura Y, Mishima S, Kashiwakura JI, Sasaki-Sakamoto T, Toyoshima S, Kuroda K, et al. The dual regulation of substance P-mediated inflammation via human synovial mast cells in rheumatoid arthritis. Allergology International. 2017;66:S9-S20. doi: 10.1016/j.alit.2017.03.002.

2408. Oliveira-Maia AJ, Press D, Pascual-Leone A. Modulation of motor cortex excitability predicts antidepressant response to prefrontal cortex repetitive transcranial magnetic stimulation. Brain Stimulation. 2017;10(4):787-94. doi: 10.1016/j.brs.2017.03.013.

2409. Orfila J, Dietz R, Patsos O, Schmidt R, Traystman R, Herson P.

Extended therapeutic window of a novel peptide inhibitor of TRPM2 channels on memory following traumatic brain injury. Journal of Cerebral Blood Flow and Metabolism. 2017;37(1):411. doi: 10.1177/0271678X17695986.

2410. Ortenzi M, Ghiselli R, Cardinali L, Guerrieri M. Surgical treatment of gastric stromal tumors: laparoscopic versus open approach. Annali italiani di chirurgia. 2017;88.

2411. Pape T, Rosenow J, Blabas B, Kletzel S, Herrold A, Parrish T, et al. Combining rTMS and amantadine to enable neurobehavioural recovery for persons living in chronic states of disordered consciousness after TBI. Brain Injury. 2017;31(6-7):994-5. PubMed PMID: WOS:000406734000550.

2412. Parazzini M, Chiaramello E, Fiocchi S, Ravazzani P. Computational modelling studies for non-invasive brain stimulation. European Archives of Psychiatry and Clinical Neuroscience. 2017;267:S135-S6. doi: 10.1007/s00406-017-0856-0.

2413. Pashkovskaya O, Polovnikov E. A study on the patient positioning accuracy for stereotactic radiotherapy of brain lesions. Radiotherapy and Oncology. 2017;123:S994.

2414. Pearce AJ, Rist B, Cohen A. Electrophysiological changes in retired Australian rugby players with a history of repeated sports concussion: A study using transcranial magnetic stimulation. Brain

Injury. 2017;31(6-7):825-6. doi: 10.1080/02699052.2017.1312145.
2415. Pearn ML, Niesman IR, Egawa J, Sawada A, Almenar-Queralt A, Shah SB, et al. Pathophysiology Associated with Traumatic Brain Injury: Current Treatments and Potential Novel Therapeutics. Cell Mol Neurobiol. 2017;37(4):571-85. Epub 2016/07/08. doi: 10.1007/s10571-016-0400-1. PubMed PMID: 27383839.
2416. Peng CW, Chen SC, Hsieh TH. Influence of cortical electrical stimulation on neural plasticity in a rat model of traumatic brain injury. Brain Stimulation. 2017;10(2):362. doi: 10.1016/j.brs.2017.01.066.
2417. Potter-Baker KA, Frost F, Plow EB. It's all in your head: driving cortical plasticity to improve muscle contraction below the level of injury. Journal of spinal cord medicine. 2017;40(5):592-. doi: 10.1080/10790268.2017.1351703. PubMed PMID: CN-01416408.
2418. Pruitt DT, Danaphongse TT, Schmid AN, Morrison RA, Kilgard MP, Rennaker RL, II, et al. Traumatic Brain Injury Occludes Training-Dependent Cortical Reorganization in the Contralesional Hemisphere. Journal of Neurotrauma. 2017;34(17):2495-503. doi: 10.1089/neu.2016.4796. PubMed PMID: WOS:000408310200002.
2419. Quinn D. TRANSCRANIAL DIRECT CURRENT STIMULATION FACILITATES WORKING MEMORY AFTER MILD-MODERATE TRAUMATIC BRAIN INJURY. Journal of Neurotrauma. 2017;34(13):A143-A. PubMed PMID:

WOS:000404530400392.
2420. Quoilin C, De Timary P, Duque J. Impaired inhibitory control in alcohol-dependence: Relationship with brain damage and relapse. Alcoholism: Clinical and Experimental Research. 2017;41:84A. doi: 10.1111/acer.13391.
2421. Ragazzoni A, Cincotta M, Giovannelli F, Cruse D, Young GB, Miniussi C, et al. Clinical neurophysiology of prolonged disorders of consciousness: From diagnostic stimulation to therapeutic neuromodulation. Clin Neurophysiol. 2017;128(9):1629-46. Epub 2017/07/21. doi: 10.1016/j.clinph.2017.06.037. PubMed PMID: 28728060.
2422. Rajczak E, Pecoraro VL, Juskowiak B. Sm(iii)[12-MCGa(III)shi-4] as a luminescent probe for G-quadruplex structures. Metallomics. 2017;9(12):1735-44. doi: 10.1039/c7mt00232g.
2423. Rizzo J-R, Hosseini M, Wong EA, Mackey WE, Fung JK, Ahdoot E, et al. The Intersection between Ocular and Manual Motor Control: Eye-Hand Coordination in Acquired Brain Injury. Frontiers in Neurology. 2017;8. doi: 10.3389/fneur.2017.00227. PubMed PMID: WOS:000403264500001.
2424. Rothwell J. Understanding and improving mechanisms of non-invasive brain stimulation. Brain Stimulation. 2017;10(2):540. doi:

10.1016/j.brs.2017.01.572.

2425. Rovlias A, Blionas A, Melissaris S, Papoutsakis D. Strategies and public health impact of head injuries prevention. Brain Injury. 2017;31(6-7):983-4. doi: 10.1080/02699052.2017.1312145.

2426. Rutherford G, Mansouri B, Zhang W, Wang X, Sulieman A, Ranjbar O, et al. RTMS as a treatment for mild traumatic brain injury. Brain stimulation. 2017;10(2):481-. doi: 10.1016/j.brs.2017.01.409. PubMed PMID: CN-01361738.

2427. Saadipour K, MacLean M, Pirkle S, Ali S, Lopez-Redondo ML, Stokes DL, et al. The transmembrane domain of the p75 neurotrophin receptor stimulates phosphorylation of the TrkB tyrosine kinase receptor. Journal of Biological Chemistry. 2017;292(40):16594-604. doi: 10.1074/jbc.M117.788729.

2428. Sánchez-Kuhn A, Medina Y, Pérez-García M, Martínez-Sola M, De Haro P, Flores P, et al. Neurorehabilitation treatment of dysphagia after-stroke with transcranial direct current stimulation: A clinical case. Brain Stimulation. 2017;10(2):431. doi: 10.1016/j.brs.2017.01.283.

2429. Santarnecchi E, Pascual-Leone A. The Illusion of the Perfect Brain Enhancer. Cerebrum. 2017;2017. Epub 2018/09/14. PubMed PMID: 30210662; PubMed Central PMCID: PMCPMC6132045.

2430. Sato K, Kobayashi Y, Nakamura A, Fukushima D, Satomi S.

Early post-transplant hyperbilirubinemia is a possible predictive factor for developing neurological complications in pediatric living donor liver transplant patients receiving tacrolimus. Pediatric Transplantation. 2017;21(2). doi: 10.1111/petr.12843.

2431. Scangos K, Boudewyn M, Roberts B, Ragland J, Ranganath C, Carter C. Effects of tDCS on Cognitive Control and Cortical Network Oscillations in Schizophrenia. Neuropsychopharmacology. 2017;42:S230-S. PubMed PMID: WOS:000416846301186.

2432. Schnakers C, Monti MM. Disorders of consciousness after severe brain injury: therapeutic options. Curr Opin Neurol. 2017;30(6):573-9. Epub 2017/09/14. doi: 10.1097/wco.0000000000000495. PubMed PMID: 28901969.

2433. Sebastianelli L, Saltuari L, Nardone R. How the brain can rewire itself after an injury: the lesson from hemispherectomy. Neural Regeneration Research. 2017;12(9):1426-7. doi: 10.4103/1673-5374.215247. PubMed PMID: WOS:000412978200008.

2434. Seeger TA, Kirton A, Esser MJ, Gallagher C, Dunn J, Zewdie E, et al. Cortical excitability after pediatric mild traumatic brain injury. Brain Stimul. 2017;10(2):305-14. Epub 2016/12/06. doi: 10.1016/j.brs.2016.11.011. PubMed PMID: 27916406.

2435. Sender NS, Govindan RB, Whitehead MT, Massaro AN,

Metzler M, Wang J, et al. Cerebral modulation of the autonomic nervous system in term infants. Journal of Perinatology. 2017;37(5):558-62. doi: 10.1038/jp.2016.248.
2436. Shamanur SB, Prabhu KV. Pseudohypoaldosteronism type 1: A challenging diagnosis. Hormone Research in Paediatrics. 2017;88:76. doi: 10.1159/000481424.
2437. Shaw M, Dobbs B, Pawlak N, Pau W, Sherman K, Bikson M, et al. Updated safety and tolerability of remotely-supervised transcranial direct current stimulation (RS-tDCS). Brain stimulation. 2017;10(4):e60-e1. doi: 10.1016/j.brs.2017.04.106. PubMed PMID: CN-01399491.
2438. Sheehan D, Fickling S, Livingstone A, Tannouri P. Symstematic review and proposed study design for neuromodulation therapy in dementia. Irish Journal of Medical Science. 2017;186(1):S15-S6. doi: 10.1007/s11845-016-1532-5.
2439. Shin HW, Hallett M, Sohn Y. Low-frequency repetitive transcranial cerebellar magnetic stimulation as an 'add-on' therapy in patients with Essential Tremor. Movement disorders. 2017;32:507-9. doi: 10.1002/mds.27087. PubMed PMID: CN-01712971.
2440. Shin SS, Pelled G. Novel Neuromodulation Techniques to Assess Interhemispheric Communication in Neural Injury and Neurodegenerative Diseases. Front Neural Circuits. 2017;11:15. Epub

2017/03/25. doi: 10.3389/fncir.2017.00015. PubMed PMID: 28337129; PubMed Central PMCID: PMCPMC5343068.

2441. Siddiqi SH, Hacker CD, Kandala S, Laumann TO, Trapp NT, Brody DL, et al. Individualized precision targeting of resting-state networks with transcranial magnetic stimulation. Journal of ECT. 2017;33(3):212. doi: 10.1097/YCT.0000000000000429.

2442. Siddiqi SH, Trapp NT, Kandala S, Hacker CD, Hong X, Carter AC, et al. Individualized connectome mapping to target rTMS for treatment-resistant depression in traumatic brain injury. Journal of neuropsychiatry and clinical neurosciences. 2017;29(3):e27 - . doi: 10.1176/appi.neuropsych.16110299. PubMed PMID: CN-01470441.

2443. Siddiqi SH, Trapp NT, Kandala S, Laumann TO, Hacker CD, Hong X, et al. Repetitive transcranial magnetic stimulation with resting-state network targeting for depression in traumatic brain injury. Journal of ECT. 2017;33(3):212-. doi: 10.1097/YCT.0000000000000429. PubMed PMID: CN-01461833.

2444. Simons A, Hamdy S. The Use of Brain Stimulation in Dysphagia Management. Dysphagia. 2017;32(2):209-15. doi: 10.1007/s00455-017-9789-z. PubMed PMID: WOS:000399169300001.

2445. Sollmann N, Wildschutz N, Boeckh-Behrens T, Meyer B, Krieg SM. Associations between clinical outcome and navigated TMS

characteristics in patients with motor-eloquent brain lesions: A combined NTMS efiber tracking approach. Brain Stimulation. 2017;10(2):348. doi: 10.1016/j.brs.2017.01.016.

2446. Song W, Martin JH. Spinal cord direct current stimulation differentially modulates neuronal activity in the dorsal and ventral spinal cord. Journal of Neurophysiology. 2017;117(3):1143-55. doi: 10.1152/jn.00584.2016. PubMed PMID: WOS:000398162500023.

2447. Soplata AE, McCarthy MM, Sherfey J, Lee S, Purdon PL, Brown EN, et al. Thalamocortical control of propofol phase-amplitude coupling. PLoS Computational Biology. 2017;13(12). doi: 10.1371/journal.pcbi.1005879.

2448. Srejic U, Larson P, Bickler PE. Little Black Boxes: Noncardiac Implantable Electronic Medical Devices and Their Anesthetic and Surgical Implications. Anesth Analg. 2017;125(1):124-38. Epub 2017/05/11. doi: 10.1213/ane.0000000000001983. PubMed PMID: 28489640.

2449. Stip E, Lespérance P, Farmer O, Fournier-Gosselin MP. First clinical use of epidural stimulation in catatonia. Brain Stimulation. 2017;10(4):859-61. doi: 10.1016/j.brs.2017.03.006.

2450. Streijger F, Skinnider MA, Rogalski JC, Balshaw R, Shannon CP, Prudova A, et al. A Targeted Proteomics Analysis of Cerebrospinal Fluid after Acute Human Spinal Cord Injury. Journal of Neurotrauma.

2017;34(12):2054-68. doi: 10.1089/neu.2016.4879. PubMed PMID: WOS:000402880600006.

2451. Strisland F, Vedum J, Liverud A, Dalgard S, Brodreskift T, Albert B, et al. Portable qEEG and HD-tCS Device for Point-of-Injury Traumatic Brain Injury Diagnostics. Studies in health technology and informatics. 2017;237:198-203. PubMed PMID: MEDLINE:28479568.

2452. Su L-C, Chen M-C. Efficient delivery of nanoparticles to deep skin layers using dissolvable microneedles with an extended-length design. Journal of Materials Chemistry B. 2017;5(18):3355-63. doi: 10.1039/c7tb00451f. PubMed PMID: WOS:000400988200011.

2453. Sun Y, Wong W, Farzan F, Blumberger DM, Daskalakis ZJ. Neuromodulation of subgenual cingulate activity localizable from EEG. BMC Neuroscience. 2017;18. doi: 10.1186/s12868-017-0371-2.

2454. Suponeva NA, Bakulin IS, Poydasheva AG, Piradov MA. Safety of transcranial magnetic stimulation: review of international guidelines and new findings. Neuromuscular Diseases. 2017;7(2):21-36. doi: 10.17650/2222-8721-2017-7-2-21-36. PubMed PMID: RSCI:30013882.

2455. Surmeli T, Eralp E, Mustafazade I, Kos IH, Ozer GE, Surmeli OH. Quantitative EEG Neurometric Analysis-Guided Neurofeedback Treatment in Postconcussion Syndrome (PCS): Forty Cases. How Is

Neurometric Analysis Important for the Treatment of PCS and as a Biomarker? Clinical Eeg and Neuroscience. 2017;48(3):217-30. doi: 10.1177/1550059416654849. PubMed PMID: WOS:000403282400007.

2456. Suykerbuyk JK, Demeester MAUD. A double-blind placebo controlled pilot-study of duloxetine as treatment of mood instability after acquired brain injury (ABI). Journal of Head Trauma Rehabilitation. 2017;32(6):E76. doi: 10.1097/HTR.0000000000000241.

2457. Takemi M, Castagnola E, Ansaldo A, Ricci D, Fadiga L, Taoka M, et al. Rapid Identification of Cortical Motor Areas in Rodents by High-Frequency Automatic Cortical Stimulation and Novel Motor Threshold Algorithm. Frontiers in Neuroscience. 2017;11. doi: 10.3389/fnins.2017.00580. PubMed PMID: WOS:000413158000001.

2458. Tejale HG, Khandheria B, Smalligan RD. Eagle in a tailspin (stylohyoid syndrome leads to vertigo). Journal of General Internal Medicine. 2017;32(2):S491.

2459. Tendler A, Roth Y, Barnea-Ygael N, Zangen A. How to use the H1 deep transcranial magnetic stimulation coil for conditions other than depression. Journal of Visualized Experiments. 2017;2017(119). doi: 10.3791/55100.

2460. Than A, Liang K, Xu S, Sun L, Duan H, Xi F, et al. Transdermal Delivery of Anti-Obesity Compounds to Subcutaneous Adipose Tissue with Polymeric Microneedle Patches. Small Methods. 2017;1(11). doi: 10.1002/smtd.201700269. PubMed PMID: WOS:000417488900010.

2461. Theus MH, Brickler T, Meza AL, Coutermarsh-Ott S, Hazy A, Gris D, et al. Loss of NLRX1 exacerbates neural tissue damage and NF-κB signaling following brain injury. Journal of Immunology. 2017;199(10):3547-58. doi: 10.4049/jimmunol.1700251.

2462. Thibaut A, Martens G, Laureys S. Multichannel tDCS of the frontoparietal network in patients with disorders of consciousness: a double blind sham controlled randomized clinical trial. Brain stimulation. 2017;10(4):e40-. doi: 10.1016/j.brs.2017.04.074. PubMed PMID: CN-01399464.

2463. Thibaut A, Wannez S, Donneau AF, Chatelle C, Gosseries O, Bruno MA, et al. Controlled clinical trial of repeated prefrontal tDCS in patients with chronic minimally conscious state. Brain Inj. 2017;31(4):466-74. Epub 2017/03/11. doi: 10.1080/02699052.2016.1274776. PubMed PMID: 28281845.

2464. Tobimatsu S. Neurophysiology CNS. Journal of the Neurological Sciences. 2017;381:26-. doi: 10.1016/j.jns.2017.08.115. PubMed PMID: WOS:000427450300091.

2465. Trachsel DS, Tejada MA, Grosfjeld Christensen V, Pedersen PJ, Kanters JK, Calloe K, et al. Trimethoprim-sulfadiazine has Kv11.1 potassium channel blocking activitiy in horses. Journal of Veterinary Internal Medicine. 2017;31(2):611.

2466. Trapp NT, Siddiqi SH, Carter AC, Brody DL. Functional magnetic resonance imaging-navigated repetitive transcranial magnetic stimulation of the dorsal attention network alters functional connectivity in a predictable manner. Journal of ECT. 2017;33(3):215. doi: 10.1097/YCT.0000000000000429.

2467. Tu-Chan AP, Natraj N, Godlove J, Abrams G, Ganguly K. Effects of somatosensory electrical stimulation on motor function and cortical oscillations. J Neuroeng Rehabil. 2017;14(1):113. Epub 2017/11/15. doi: 10.1186/s12984-017-0323-1. PubMed PMID: 29132379; PubMed Central PMCID: PMCPMC5683582.

2468. Tyler WJ. Transdermal electrical neuromodulation of the trigeminal sensory nuclear complex improves sleep quality. Sleep medicine. 2017;40:e330-. PubMed PMID: CN-01463341.

2469. Ulyanova A, Koch P, Grovola M, Russo R, Smith D, Johnson V, et al. In vivo electrophysiological alterations of the hippocampus in a porcine model of diffuse axonal injury. Journal of Neurotrauma. 2017;34(13):A119. doi: 10.1089/neu.2017.29011.abstracts.

2470. Vanhoecke J, Hariz M. Deep brain stimulation for disorders of

consciousness: Systematic review of cases and ethics. Brain Stimul. 2017;10(6):1013-23. Epub 2017/10/03. doi: 10.1016/j.brs.2017.08.006. PubMed PMID: 28966051.

2471. Vanzan S, Wilkinson D, Ferguson H, Pullicino P, Sakel M. Behavioural improvement in a minimally conscious state after caloric vestibular stimulation: evidence from two single case studies. Clin Rehabil. 2017;31(4):500-7. Epub 2016/04/29. doi: 10.1177/0269215516646167. PubMed PMID: 27121862.

2472. Verdugo-Diaz L, Estrada-Rojo F, Garcia-Espinoza A, Hernandez-Lopez E, Hernandez-Chavez A, Guzman-Uribe C, et al. Effect of Intermediate-Frequency Repetitive Transcranial Magnetic Stimulation on Recovery following Traumatic Brain Injury in Rats. Biomed Res Int. 2017;2017:4540291. Epub 2018/01/11. doi: 10.1155/2017/4540291. PubMed PMID: 29318150; PubMed Central PMCID: PMCPMC5727566.

2473. Votta-Velis EG. Pro con debate 5: Intrathecal drug delivery is the way forward. Regional Anesthesia and Pain Medicine. 2017;42(5):e7. doi: 10.1097/AAP.0000000000000656.

2474. Wang D, Mai R, Wang M, Tan T, Zhu F, Chen S, et al. Effect of Low Frequency Repetitive Transcranial Magnetic Stimulation at Uninjured Side on Memory and Quality of Life in Patients with Traumatic Brain Injury. Chinese Journal of Rehabilitation Theory and

Practice. 2017;23(10):1180-4. PubMed PMID: CSCD:6107379.
2475. Wang J, Lim Y-F, Ho GW. Manganese Copper Sulfide Nanocomposites: Structure Tailoring and Photo/Electrocatalytic Hydrogen Generation. Chemcatchem. 2017;9(21):4148-54. doi: 10.1002/cctc.201700865. PubMed PMID: WOS:000417441400019.
2476. Wardini R, Moses M. Portable neuromodulation stimulation (PoNSTM) therapy efficacy for the treatment of traumatic brain injury compared to standard of care. Brain injury Conference: 12th world congress on brain injury of the international brain injury association United states. 2017;31(6-7):806. doi: 10.1080/02699052.2017.1312145. PubMed PMID: CN-01399406.
2477. Watanabe M, Michou E, Raginis-Zborowska A, Magara J, Inoue M, Hamdy S. Ten Hz repetitive cerebellar magnetic stimulation reverses cortical suppression in the healthy human pharyngeal motor system. Gut. 2017;66:A269. doi: 10.1136/gutjnl-2017-314472.525.
2478. Wertheimer T, Velardi E, Tsai J, Cooper K, Ottmueller KJ, Mokthari Z, et al. Mechanisms Governing Endogenous Thymic Regeneration. Blood. 2017;130. PubMed PMID: WOS:000432419400121.
2479. Wilke S, List J, Mekle R, Lindenberg R, Bukowski M, Ott S, et al. No Effect of Anodal Transcranial Direct Current Stimulation on Gamma-Aminobutyric Acid Levels in Patients with Recurrent Mild

Traumatic Brain Injury. J Neurotrauma. 2017;34(2):281-90. Epub 2016/07/10. doi: 10.1089/neu.2016.4399. PubMed PMID: 27393381.

2480. Wilkerson GB, Grooms DR, Acocello SN. Neuromechanical Considerations for Postconcussion Musculoskeletal Injury Risk Management. Current Sports Medicine Reports. 2017;16(6):419-27. doi: 10.1249/JSR.0000000000000430.

2481. Winkelman MJ. The mechanisms of psychedelic visionary experiences: Hypotheses from evolutionary psychology. Frontiers in Neuroscience. 2017;11(SEP). doi: 10.3389/fnins.2017.00539.

2482. Wise N. Transcranial photobiomodulation for stress: Why, how, and where to treat the brain to relax the body. Lasers in Medical Science. 2017;32(8):1703. doi: 10.1007/s10103-017-2327-2.

2483. Wu X, Zhang C, Feng J, Mao Q, Gao G, Jiang J. Right median nerve electrical stimulation for acute traumatic coma (the Asia Coma Electrical Stimulation trial): study protocol for a randomised controlled trial. Trials. 2017;18(1):311. Epub 2017/07/12. doi: 10.1186/s13063-017-2045-x. PubMed PMID: 28693604; PubMed Central PMCID: PMCPMC5504835.

2484. Xia X, Bai Y, Zhou Y, Yang Y, Xu R, Gao X, et al. Effects of 10 Hz Repetitive Transcranial Magnetic Stimulation of the Left Dorsolateral Prefrontal Cortex in Disorders of Consciousness. Front Neurol. 2017;8:182. Epub 2017/05/19. doi: 10.3389/fneur.2017.00182.

PubMed PMID: 28515709; PubMed Central PMCID: PMCPMC5413493.

2485. Xia X, Bai Y, Zhou Y, Yang Y, Xu R, Gao X, et al. Effect of 10 Hz Repetitive Transcranial Magnetic Stimulation of the Left Dorsolateral Prefrontal Cortex in Disorders of Consciousness. Frontiers in Neurology. 2017;8. doi: 10.3389/fneur.2017.00182. PubMed PMID: WOS:000400511900001.

2486. Xia X, Liu Y, Bai Y, Liu Z, Yang Y, Guo Y, et al. Long-lasting repetitive transcranial magnetic stimulation modulates electroencephalography oscillation in patients with disorders of consciousness. Neuroreport. 2017;28(15):1022-9. Epub 2017/09/14. doi: 10.1097/wnr.0000000000000886. PubMed PMID: 28902713.

2487. Yamamoto T, Watanabe M, Obuchi T, Kobayashi K, Oshima H, Fukaya C, et al. Spinal Cord Stimulation for Vegetative State and Minimally Conscious State: Changes in Consciousness Level and Motor Function. Acta Neurochir Suppl. 2017;124:37-42. Epub 2017/01/26. doi: 10.1007/978-3-319-39546-3_6. PubMed PMID: 28120050.

2488. Yan Y, Fu J, Wang T, Lu X. Controlled release of silyl ether camptothecin from thiol-ene click chemistry-functionalized mesoporous silica nanoparticles. Acta Biomaterialia. 2017;51:471-8. doi: 10.1016/j.actbio.2017.01.062. PubMed PMID:

WOS:000398014000037.
2489. Yang H, Jiang K, Fang D. Effect of Median Nerve Electrical Stimulation on Hemodynamics in Severe Brain Injury Patients with Coma. Acta Medicinae Universitatis Scientiae et Technologiae Huazhong. 2017;46(4):462-5. PubMed PMID: CSCD:6060883.
2490. Yasen AL, Howell DR, Chou LS, Pazzaglia AM, Christie AD. Cortical and Physical Function after Mild Traumatic Brain Injury. Med Sci Sports Exerc. 2017;49(6):1066-71. Epub 2017/05/17. doi: 10.1249/mss.0000000000001217. PubMed PMID: 28509819.
2491. Young LR, Yu W, Holloway M, Rodgers BN, Chapman SB, Krawczyk DC. Amygdala activation as a marker for selective attention toward neutral faces in a chronic traumatic brain injury population. Neuropsychologia. 2017;104:214-22. doi: 10.1016/j.neuropsychologia.2017.08.026.
2492. Yu W, Jiang G, Liu D, Li L, Chen H, Liu Y, et al. Fabrication of biodegradable composite microneedles based on calcium sulfate and gelatin for transdermal delivery of insulin. Materials Science & Engineering C-Materials for Biological Applications. 2017;71:725-34. doi: 10.1016/j.msec.2016.10.063. PubMed PMID: WOS:000390967200086.
2493. Yu W, Jiang G, Liu D, Li L, Tong Z, Yao J, et al. Transdermal delivery of insulin with bioceramic composite microneedles fabricated

by gelatin and hydroxyapatite. Materials Science & Engineering C-Materials for Biological Applications. 2017;73:425-8. doi: 10.1016/j.msec.2016.12.111. PubMed PMID: WOS:000394064800050.

2494. Yu W, Jiang G, Zhang Y, Liu D, Xu B, Zhou J. Polymer microneedles fabricated from alginate and hyaluronate for transdermal delivery of insulin. Materials Science & Engineering C-Materials for Biological Applications. 2017;80:187-96. doi: 10.1016/j.msec.2017.05.143. PubMed PMID: WOS:000410254400025.

2495. Yu Y, Rong P, Zhu B. Present Situation and Prospect of Transcutaneous Auricular Vagus Nerve Stimulation in Encephalopathy Treatment. Modernization of Traditional Chinese Medicine and Materia Medica--World Science and Technology. 2017;19(3):462-8. PubMed PMID: CSCD:6013096.

2496. Yu YT, Yang Y, Wang LB, Fang JL, Chen YY, He JH, et al. Transcutaneous auricular vagus nerve stimulation in disorders of consciousness monitored by fMRI: The first case report. Brain Stimul. 2017;10(2):328-30. Epub 2016/12/27. doi: 10.1016/j.brs.2016.12.004. PubMed PMID: 28017322.

2497. Zhang J. Multivariate analysis and machine learning in cerebral palsy research. Frontiers in Neurology. 2017;8(DEC). doi:

10.3389/fneur.2017.00715.

2498. Zhang P, Cao Y, Chen H, Zhou B, Hu W, Zhang L. Preparation and evaluation of glycyrrhetinic acid-modified and honokiol-loaded acoustic nanodroplets for targeted tumor imaging and therapy with low-boiling-point phase-change perfluorocarbon. Journal of Materials Chemistry B. 2017;5(29):5845-53. doi: 10.1039/c7tb01215b. PubMed PMID: WOS:000406616400015.

2499. Zhang X, Hu Y, Tao W, Zhu H, Xiao D, Li Y. The Effect of Motor Cortex Stimulation on Central Poststroke Pain in a Series of 16 Patients With a Mean Follow-Up of 28 Months. Neuromodulation. 2017;20(5):492-6. Epub 2017/01/20. doi: 10.1111/ner.12547. PubMed PMID: 28102925.

2500. Zhang Y, Song W, Du J, Huo S, Shan G, Li R. Transcranial Direct Current Stimulation in Patients with Prolonged Disorders of Consciousness: Combined Behavioral and Event-Related Potential Evidence. Front Neurol. 2017;8:620. Epub 2017/12/07. doi: 10.3389/fneur.2017.00620. PubMed PMID: 29209270; PubMed Central PMCID: PMCPMC5702306.

2501. Zhu DD, Chen BZ, He MC, Guo XD. Structural optimization of rapidly separating microneedles for efficient drug delivery. Journal of Industrial and Engineering Chemistry. 2017;51:178-84. doi: 10.1016/j.jiec.2017.02.030. PubMed PMID: WOS:000401208400019.

2502. Zhu Y, Deng L, Tang H, Gao X, Wang Y, Guo K, et al. Electroacupuncture improves neurobehavioral function and brain injury in rat model of intracerebral hemorrhage. Brain Res Bull. 2017;131:123-32. Epub 2017/04/12. doi: 10.1016/j.brainresbull.2017.04.003. PubMed PMID: 28395933.
2503. Abstracts of the 62nd Scientific Annual Meeting of the German Society for Clinical Neurophysiology and Functional Imaging, DGKN. Clinical Neurophysiology. 2018;129(8).
2504. Aima M, DeWerd LA, Mitch MG, Hammer CG, Culberson WS. Dosimetric characterization of a new directional low-dose rate brachytherapy source. Medical Physics. 2018;45(8):3848-60. doi: 10.1002/mp.12994.
2505. Alawieh A, Zhao J, Feng W. Factors affecting post-stroke motor recovery: Implications on neurotherapy after brain injury. Behavioural Brain Research. 2018;340:94-101. doi: 10.1016/j.bbr.2016.08.029. PubMed PMID: WOS:000424173400011.
2506. Alkanfari I, Gupta K, Jahan T, Ali H. Naturally occurring missense MRGPRX2 variants display loss of function phenotype for mast cell degranulation in response to substance P, hemokinin-1, human b-defensin-3, and icatibant. Journal of Immunology. 2018;201(2):343-9. doi: 10.4049/jimmunol.1701793.
2507. Alsharidah M, Al-Hussain F, Iqbal M, Hamza A, Yoo WK,

Bashir S. The effect of transcranial direct current stimulation combined with functional task training on motor recovery in stroke patients. European Review for Medical and Pharmacological Sciences. 2018;22(21):7385-92. PubMed PMID: WOS:000451033900035.

2508. Andersen TE, Andersen AJ, Petersen RS, Nielsen LH, Keller SS. Drug loaded biodegradable polymer microneedles fabricated by hot embossing. Microelectronic Engineering. 2018;195:57-61. doi: 10.1016/j.mee.2018.03.024. PubMed PMID: WOS:000433641200010.

2509. Anghinah R, Paiva WS, Falk TH, Fregni F. Editorial: NEUROTRAUMA: from emergency room to back to day-by-day life. Frontiers in Neurology. 2018;9(SEP). doi: 10.3389/fneur.2018.00776.

2510. Bai Y, Xia X, Wang Y, Guo Y, Yang Y, He J, et al. Fronto-parietal coherence response to tDCS modulation in patients with disorders of consciousness. Int J Neurosci. 2018;128(7):587-94. Epub 2017/11/22. doi: 10.1080/00207454.2017.1403440. PubMed PMID: 29160761.

2511. Baltus A, Wagner S, Wolters CH, Herrmann CS. Optimized auditory transcranial alternating current stimulation improves individual auditory temporal resolution. Brain Stimulation. 2018;11(1):118-24. doi: 10.1016/j.brs.2017.10.008.

2512. Baniqued PL, Gallen CL, Voss MW, Burzynska AZ, Wong CN, Cooke GE, et al. Brain network modularity predicts exercise-related

executive function gains in older adults. Frontiers in aging neuroscience. 2018;9(JAN) (no pagination). doi: 10.3389/fnagi.2017.00426. PubMed PMID: CN-01449590.

2513. Barilka V, Matlan V, Shalay O, Yevstachevych Y, Prymak S, Loginsky V. The impaired production of TNF, TGF beta 1 as an important reason of defective natural killer ability in non-Hodgkin lymphoma patients with spleen affection. HemaSphere. 2018;2:1038-9. doi: 10.1097/HS9.0000000000000060.

2514. Bell AH, Bultitude JH. Methods matter: A primer on permanent and reversible interference techniques in animals for investigators of human neuropsychology. Neuropsychologia. 2018;115:211-9. Epub 2017/09/26. doi: 10.1016/j.neuropsychologia.2017.09.019. PubMed PMID: 28943365; PubMed Central PMCID: PMCPMC6018620.

2515. Bestelmeyer PEG, Davis NJ, Poliva O, Rafal RD. Neuromodulation of Right Auditory Cortex Selectively Increases Activation in Speech-Related Brain Areas in Brainstem Auditory Agnosia. Cogn Behav Neurol. 2018;31(3):151-5. Epub 2018/09/22. doi: 10.1097/wnn.0000000000000162. PubMed PMID: 30239465.

2516. Bianchi F, Malboubi M, Li Y, George JH, Jerusalem A, Szele F, et al. Rapid and efficient differentiation of functional motor neurons from human iPSC for neural injury modelling. Stem Cell Research.

2018;32:126-34. doi: 10.1016/j.scr.2018.09.006. PubMed PMID: WOS:000447301200019.

2517. Bickart K, Main K, Milazzo A-C, Newsom M, Sours C, Ashford JW, et al. Large-Scale Frontotemporal Limbic Networks in TBI: Vulnerability and Response to Non-Invasive Brain Stimulation. Neurology. 2018;90. PubMed PMID: WOS:000453090806024.

2518. Black L, Gaebler-Spira D. Nonsurgical Treatment Options for Upper Limb Spasticity. Hand Clin. 2018;34(4):455-64. Epub 2018/10/06. doi: 10.1016/j.hcl.2018.06.003. PubMed PMID: 30286960.

2519. Blandiaux S, Raimondo F, Wolff A, Sanz L, Bodart O, Barra A, et al. PCI & Auditory ERPs for the diagnosis of disorders of consciousness: an EEG-based methods comparison study. European Journal of Neurology. 2018;25:399-. PubMed PMID: WOS:000435272002205.

2520. Bodart O, Amico E, Gómez F, Casali AG, Wannez S, Heine L, et al. Global structural integrity and effective connectivity in patients with disorders of consciousness. Brain Stimul. 2018;11(2):358-65. Epub 2017/11/23. doi: 10.1016/j.brs.2017.11.006. PubMed PMID: 29162503.

2521. Bodi I, Giamouriadis A, Sibtain N, Laxton R, King A, Vergani F. Primary intracerebral INI1-deficient rhabdoid tumor with CD34

immunopositivity in a young adult. Surgical Neurology International. 2018;9(1). doi: 10.4103/sni.sni_334_17.
2522. Boly M. Quantifying consciousness. Clinical EEG and Neuroscience. 2018;49(6):NP9. doi: 10.1177/1550059418756508.
2523. Bragina OA, Lara DA, Nemoto EM, Shuttleworth CW, Semyachkina-Glushkovskaya OV, Bragin DE. Increases in Microvascular Perfusion and Tissue Oxygenation via Vasodilatation After Anodal Transcranial Direct Current Stimulation in the Healthy and Traumatized Mouse Brain. Adv Exp Med Biol. 2018;1072:27-31. Epub 2018/09/05. doi: 10.1007/978-3-319-91287-5_5. PubMed PMID: 30178319; PubMed Central PMCID: PMCPMC6294145.
2524. Bragina OA, Nemoto EM, Bragin DE. Anodal transcranial direct current stimulation (tDCS) improves impaired cerebrovascular reactivity in traumatized mouse brain. Journal of Neurotrauma. 2018;35(16):A264. doi: 10.1089/neu.2018.29013.abstracts.
2525. Branscheidt M, Hoppe J, Zwitserlood P, Liuzzi G. TDCS over the motor cortex improves lexical retrieval of action words in poststroke aphasia. Journal of Neurophysiology. 2018;119(2):621-30. doi: 10.1152/jn.00285.2017.
2526. Bui Quoc B, Ngoc Hoang L, Diem Huong Tran N, Tuong Vi T, Linh Phuong Tran P, Long Giang B, et al. Evolution and present scenario of multifunctionalized mesoporous nanosilica platform: A

mini review. Materials Science & Engineering C-Materials for Biological Applications. 2018;91:912-28. doi: 10.1016/j.msec.2018.07.008. PubMed PMID: WOS:000442192000092.
2527. Casarotto S, Comanducci A, Casali AG, Rosanova M, Massimini M. A non-invasive perturbation approach to disorders of consciousness. International Journal of Psychophysiology. 2018;131:S65. doi: 10.1016/j.ijpsycho.2018.07.193.
2528. Catić-Đorđević A, Pavlović I, Pavlović D, Stefanović N, Mikov M, Cvetković T, et al. Evaluation of gender-based limited sampling methods for tacrolimus exposure after renal transplantation using the Monte Carlo simulation. Pharmazie. 2018;73(8):482-5. doi: 10.1691/ph.2018.8461.
2529. Cavanna F, Vilas MG, Palmucci M, Tagliazucchi E. Dynamic functional connectivity and brain metastability during altered states of consciousness. Neuroimage. 2018;180(Pt B):383-95. Epub 2017/10/08. doi: 10.1016/j.neuroimage.2017.09.065. PubMed PMID: 28986208.
2530. Chen BZ, Ashfaq M, Zhu DD, Zhang XP, Guo XD. Controlled Delivery of Insulin Using Rapidly Separating Microneedles Fabricated from Genipin-Crosslinked Gelatin. Macromolecular Rapid Communications. 2018;39(20). doi: 10.1002/marc.201800075. PubMed PMID: WOS:000447653600005.

2531. Chen C-H, Shyu VB-H, Chen C-T. Dissolving Microneedle Patches for Transdermal Insulin Delivery in Diabetic Mice: Potential for Clinical Applications. Materials. 2018;11(9). doi: 10.3390/ma11091625. PubMed PMID: WOS:000446395200141.

2532. Chen M-C, Lai K-Y, Ling M-H, Lin C-W. Enhancing immunogenicity of antigens through sustained intradermal delivery using chitosan microneedles with a patch-dissolvable design. Acta Biomaterialia. 2018;65:66-75. doi: 10.1016/j.actbio.2017.11.004. PubMed PMID: WOS:000418315900006.

2533. Chi L, Li H, Zhao L, Pang Y. The clinical curative effect of transcranial direct current stimulation on patients with disorders of consciousness. Chinese Journal of Physical Medicine and Rehabilitation. 2018;40(9):652-6. PubMed PMID: CSCD:6459795.

2534. ChiCtr. A prospective study of prognosis in Children with Disorder of consciousness and Wake-promoting actions of median nerve stimulation of DoC in children. https://trialsearchwhoint/Trial2aspx?TrialID=ChiCTR1800019330. 2018. PubMed PMID: CN-01950673.

2535. ChiCtr. Effects of Transcranial Direct Current Stimulation on Patients With Disorders of Consciousness. https://trialsearchwhoint/Trial2aspx?TrialID=ChiCTR1800014808. 2018. PubMed PMID: CN-01899537.

2536. Chieffi S, Ilardi CR, Iavarone A. Parietal lobe dysfunction in schizophrenia: A review. Current Psychiatry Reviews. 2018;14(2):71-83. doi: 10.2174/1573400514666180703150804.
2537. Choi GS, Kwak SG, Lee HD, Chang MC. Effect of high-frequency repetitive transcranial magnetic stimulation on chronic central pain after mild traumatic brain injury: A pilot study. J Rehabil Med. 2018;50(3):246-52. Epub 2018/02/03. doi: 10.2340/16501977-2321. PubMed PMID: 29392332.
2538. Christova M, Gallasch E, Pinter D. Cerebellar contributions to somatosensory induced neuroplasticity. Clinical Neurophysiology. 2018;129:e141. doi: 10.1016/j.clinph.2018.04.357.
2539. Corbett M, South E, Harden M, Eldabe S, Pereira E, Sedki I, et al. Brain and spinal stimulation therapies for phantom limb pain: a systematic review. Health Technol Assess. 2018;22(62):1-94. Epub 2018/11/09. doi: 10.3310/hta22620. PubMed PMID: 30407905; PubMed Central PMCID: PMCPMC6253324 USA) and personal fees from Abbott Laboratories (Chicago, IL, USA) and Boston Scientific (Marlborough, MA, USA) outside the submitted work.
2540. Corr PJ, Mobbs D. From epiphenomenon to biologically important phenomena. Personality Neuroscience. 2018;1:1-4. doi: 10.1017/pen.2017.1.
2541. Coyle HL, Ponsford J, Hoy KE. Understanding individual

variability in symptoms and recovery following mTBI: A role for TMS-EEG? Neurosci Biobehav Rev. 2018;92:140-9. Epub 2018/06/10. doi: 10.1016/j.neubiorev.2018.05.027. PubMed PMID: 29885426.

2542. D'Andola M, Rebollo B, Casali AG, Weinert JF, Pigorini A, Villa R, et al. Bistability, Causality, and Complexity in Cortical Networks: An In Vitro Perturbational Study. Cerebral Cortex. 2018;28(7):2233-42. doi: 10.1093/cercor/bhx122. PubMed PMID: WOS:000437165800002.

2543. Damar U, Gersner R, Johnstone JT, Kapur K, Collins S, Schachter S, et al. Alterations in the Timing of Huperzine A Cerebral Pharmacodynamics in the Acute Traumatic Brain Injury Setting. J Neurotrauma. 2018;35(2):393-7. Epub 2017/10/22. doi: 10.1089/neu.2017.5258. PubMed PMID: 29054135; PubMed Central PMCID: PMCPMC5784790.

2544. Danilov Y, Paltin D. Translingual neurostimulation (TLNS): Perspective on a novel approach to neurorehabilitation after brain injury. 2018. p. 307-27.

2545. Darracq M, Funk CM, Polyakov D, Riedner B, Gosseries O, Nieminen JO, et al. Evoked Alpha Power is Reduced in Disconnected Consciousness During Sleep and Anesthesia. Sci Rep. 2018;8(1):16664. Epub 2018/11/11. doi: 10.1038/s41598-018-34957-9. PubMed PMID: 30413741; PubMed Central PMCID:

PMCPMC6226534 well as honoraria from Medtronic during the past five years. V.B. is the Editor-in-Chief of the Acta Anaesthesiologica Belgica. G.T. receives unrestricted grant funding from Philips Healthcare and serves on the advisory board of the Allen Institute for Brain Science. All other authors declare no competing interests.

2546. de Assis Aquino Gondim F, Bastante P, Gondim WC, Filha JGH, Thomas FP. Medical and neuropsychiatric phenomena depicted in two Spanish medieval texts of Marian miracles. Neurological Sciences. 2018;39(3):565-9. doi: 10.1007/s10072-017-3183-3.

2547. Debruyne P, Rossenbacker T, Collienne C, Roosen J, Ector B, Janssens L, et al. Unifocal Right-Sided Ablation Treatment for Neurally Mediated Syncope and Functional Sinus Node Dysfunction Under Computed Tomographic Guidance. Circ Arrhythm Electrophysiol. 2018;11(9):e006604. Epub 2018/10/26. doi: 10.1161/circep.118.006604. PubMed PMID: 30354289.

2548. Demeulenaere M, Devreese K, Vanbelleghem H, De Zaeytijd J, Vande Walle J, Van Biesen W, et al. Thrombomodulin and Endothelial Dysfunction: A Disease-Modifier Shared between Malignant Hypertension and Atypical Hemolytic Uremic Syndrome. Nephron. 2018;140(1):63-73. doi: 10.1159/000490201.

2549. Dieguez S, Kaeser M, Roux C, Cottet J, Annoni JM, Schmidlin E. Birth and death of a phantom. Annals of Clinical and

Translational Neurology. 2018;5(1):98-101. doi: 10.1002/acn3.495.

2550. Dogra P, Adolphi NL, Wang Z, Lin Y-S, Butler KS, Durfee PN, et al. Establishing the effects of mesoporous silica nanoparticle properties on in vivo disposition using imaging-based pharmacokinetics. Nature Communications. 2018;9. doi: 10.1038/s41467-018-06730-z. PubMed PMID: WOS:000448807000022.

2551. Domen K, Takebayashi T, Takahashi K, Moriwaki M. A pilot randomized controlled trial of constraint-induced movement therapy combined with transcranial direct current stimulation and peripheral neuromuscular stimulation. Neurorehabilitation and Neural Repair. 2018;32(4-5):340. doi: 10.1177/1545968318765497.

2552. Duchalais E, Guilluy C, Nedellec S, Touvron M, Bessard A, Touchefeu Y, et al. Colorectal Cancer Cells Adhere to and Migrate Along the Neurons of the Enteric Nervous System. Cell Mol Gastroenterol Hepatol. 2018;5(1):31-49. Epub 2017/12/01. doi: 10.1016/j.jcmgh.2017.10.002. PubMed PMID: 29188232; PubMed Central PMCID: PMCPMC5696385.

2553. Epperson M, Horn P, Wu S, Pedapati E, Guthrie M, Huddleston D, et al. Motor cortex physiology of movement preparation in ADHD children. Developmental Medicine and Child Neurology. 2018;60:95-6. doi: 10.1111/dmcn.30_14018.

2554. Evans LR, Fitzgerald MC, Varma D, Mitra B. A novel approach to improving the interpretation of CT brain in trauma. Injury. 2018;49(1):56-61. Epub 2017/09/09. doi: 10.1016/j.injury.2017.08.056. PubMed PMID: 28882376.

2555. Faried A, Ramdan A, Arifin MZ, Nataprawira HM. Characteristics and surgical outcomes of tuberculous meningitis and of tuberculous spondylitis in pediatric patients at Dr. Hasan Sadikin Hospital, Bandung: A single center experience. Interdisciplinary Neurosurgery: Advanced Techniques and Case Management. 2018;11:37-40. doi: 10.1016/j.inat.2017.09.008.

2556. Finley J. Transposable elements, placental development, and oocyte activation: Cellular stress and AMPK links jumping genes with the creation of human life. Medical Hypotheses. 2018;118:44-54. doi: 10.1016/j.mehy.2018.05.015.

2557. Fouzia NA, Edison ES, Lakshmi KM, Korula A, Velayudhan SR, Balasubramanian P, et al. Long-term outcome of mixed chimerism after stem cell transplantation for thalassemia major conditioned with busulfan and cyclophosphamide. Bone Marrow Transplantation. 2018;53(2):169-74. doi: 10.1038/bmt.2017.231.

2558. Fox KCR, Yih J, Raccah O, Pendekanti SL, Limbach LE, Maydan DD, et al. Changes in subjective experience elicited by direct stimulation of the human orbitofrontal cortex. Neurology.

2018;91(16):E1519-E27. doi: 10.1212/WNL.0000000000006358.

2559. Fox MD. Mapping Symptoms to Brain Networks with the Human Connectome. New England Journal of Medicine. 2018;379(23):2237-45. doi: 10.1056/NEJMra1706158. PubMed PMID: WOS:000452259200009.

2560. Fujioka H, Urasaki E, Izumihara A, Yamashita K. Epidural motor cortex stimulation for intractable leg pain. Clinical Neurophysiology. 2018;129(3):636-7. doi: 10.1016/j.clinph.2018.01.007. PubMed PMID: WOS:000425876800014.

2561. Gallagher CN, Esser MJ, Nguyen R, Casha S, Sutherland G, Yeates KO. Neurotrauma Research at the University of Calgary. Journal of Neurotrauma. 2018;35(16):1990-2.

2562. Gallasch E, Rafolt D, Postruznik M, Fresnoza S, Christova M. Decrease of motor cortex excitability following exposure to a 20 Hz magnetic field as generated by a rotating permanent magnet. Clinical Neurophysiology. 2018;129(7):1397-402. doi: 10.1016/j.clinph.2018.03.045.

2563. Gao F, Zhang Q, Li Y, Tai Y, Xin X, Wang X, et al. Transcutaneous electrical acupoint stimulation for prevention of postoperative delirium in geriatric patients with silent lacunar infarction: a preliminary study. Clin Interv Aging. 2018;13:2127-34.

Epub 2018/11/15. doi: 10.2147/cia.S183698. PubMed PMID: 30425466; PubMed Central PMCID: PMCPMC6205526.

2564. Geraldo AF, Pereira J, Nunes P, Reimão S, Sousa R, Castelo-Branco M, et al. Beyond fractional anisotropy in amyotrophic lateral sclerosis: the value of mean, axial, and radial diffusivity and its correlation with electrophysiological conductivity changes. Neuroradiology. 2018;60(5):505-15. doi: 10.1007/s00234-018-2012-6.

2565. Gincberg G, Shohami E, Trembovler V, Alexandrovich AG, Lazarovici P, Elchalal U. Nerve growth factor plays a role in the neurotherapeutic effect of a CD45 + pan-hematopoietic subpopulation derived from human umbilical cord blood in a traumatic brain injury model. Cytotherapy. 2018;20(2):245-61. doi: 10.1016/j.jcyt.2017.11.008.

2566. Grangeon L, O'Connor E, Matharu M. Cohort study of post-traumatic cluster headache. Headache. 2018;58:201-2.

2567. Guernon A, Larson E, Sayyad A, Brady S, Bou S, Pape T. Innovative approach using neuromodulation techniques as a treatment for TBI related cognitive impairments. Journal of head trauma rehabilitation. 2018;33(3):E93-E4. doi: 10.1097/HTR.0000000000000401. PubMed PMID: CN-01612454.

2568. Guger C, Spataro R, Pellas F, Allison BZ, Heilinger A, Ortner R, et al. Assessing Command-Following and Communication With

Vibro-Tactile P300 Brain-Computer Interface Tools in Patients With Unresponsive Wakefulness Syndrome. Front Neurosci. 2018;12:423. Epub 2018/07/17. doi: 10.3389/fnins.2018.00423. PubMed PMID: 30008659; PubMed Central PMCID: PMCPMC6034093.

2569. Guzman Garcia L, Santamarina E, Toledo M, Raguer N, Sueiras M, Abraira del Fresno L, et al. Low Frequency Repetitive Transcranial Magnetic Stimulation for Refractory Status Epilepticus and Multifocal Highly Drug-Resistant Epilepsy. Epilepsia. 2018;59:S166-S. PubMed PMID: WOS:000451817900346.

2570. Guzmán-Mejía F, López-Rubalcava C, González-Espinosa C. Stimulation of nAchRα7 Receptor Inhibits TNF Synthesis and Secretion in Response to LPS Treatment of Mast Cells by Targeting ERK1/2 and TACE Activation. J Neuroimmune Pharmacol. 2018;13(1):39-52. Epub 2017/08/20. doi: 10.1007/s11481-017-9760-7. PubMed PMID: 28822039.

2571. Guzman-Mejia F, Lopez-Rubalcava C, Gonzalez-Espinosa C. Stimulation of nAchR alpha 7 Receptor Inhibits TNF Synthesis and Secretion in Response to LPS Treatment of Mast Cells by Targeting ERK1/2 and TACE Activation. Journal of Neuroimmune Pharmacology. 2018;13(1):39-52. doi: 10.1007/s11481-017-9760-7. PubMed PMID: WOS:000423591600004.

2572. Hamoudi M, Schambra HM, Fritsch B, Schoechlin-Marx A,

Weiller C, Cohen LG, et al. Transcranial Direct Current Stimulation Enhances Motor Skill Learning but Not Generalization in Chronic Stroke. Neurorehabilitation and Neural Repair. 2018;32(4-5):295-308. doi: 10.1177/1545968318769164.

2573. Han D, She J, Guo H, Sun C, Li H. Ablation or pacing, which goes first for patients with tachycardia-bradycardia syndrome: Two case comparison and clinical enlightenment. Journal of Interventional Cardiac Electrophysiology. 2018;51(1):S112-S3. doi: 10.1007/s10840-018-0338-y.

2574. Hartl E, Bötzel K, Mehrkens JH, Noachtar S. Seizure reductions outlast DBS explantation. Brain Stimulation. 2018;11(3):636-8. doi: 10.1016/j.brs.2018.01.033.

2575. Hayashi CY, Neville IS, Rodrigues PA, Galhardoni R, Brunoni AR, Zaninotto AL, et al. Altered Intracortical Inhibition in Chronic Traumatic Diffuse Axonal Injury. Front Neurol. 2018;9:189. Epub 2018/04/13. doi: 10.3389/fneur.2018.00189. PubMed PMID: 29643831; PubMed Central PMCID: PMCPMC5882787.

2576. He F, Wu M, Meng F, Hu Y, Gao J, Chen Z, et al. Effects of 20 Hz Repetitive Transcranial Magnetic Stimulation on Disorders of Consciousness: A Resting-State Electroencephalography Study. Neural Plast. 2018;2018:5036184. Epub 2018/05/18. doi: 10.1155/2018/5036184. PubMed PMID: 29770146; PubMed Central

PMCID: PMCPMC5889874.
2577. He F, Wu M, Meng F, Hu Y, Gao J, Chen Z, et al. Effects of 20Hz Repetitive Transcranial Magnetic Stimulation on Disorders of Consciousness: A Resting-State Electroencephalography Study. Neural Plasticity. 2018;2018. doi: 10.1155/2018/5036184. PubMed PMID: WOS:000429681300001.
2578. Henssen D, Dao J, Witkam R, Comes D, Van Cappellen Van Walsum AM, Kozicz T, et al. Phenotypical and neurosurgical characteristics of non-responders to motor cortex stimulation as a treatment of pain: A systematic review and metaanalysis of individual patient data. Pain Practice. 2018;18:131.
2579. Henssen DJHA, Kurt E, van Walsum A-MvC, Arnts I, Doorduin J, Kozicz T, et al. Long-term effect of motor cortex stimulation in patients suffering from chronic neuropathic pain: An observational study. Plos One. 2018;13(1). doi: 10.1371/journal.pone.0191774. PubMed PMID: WOS:000423617800034.
2580. Hermann B, Raimondo F, Valente M, Rohaut B, Perez P, Sitt JD, et al. Electrophysiological effects of transcranial direct current stimulation in patients suffering from disorders of consciousness : An open-label pilot study. Neurocritical Care. 2018;29(1):S312. doi: 10.1007/s12028-018-0606-9.

2581. Holeckova I, Vales J, Mracek J, Rihanek P, Moucek R, Mautner P, et al. The changes in motor cortex localisation and organisation in motor eloquent tumorous brain lesions detected by navigated transcranial magnetic stimulation. Clinical Neurophysiology. 2018;129:e46. doi: 10.1016/j.clinph.2018.04.114.
2582. Hu L, Liao Z, Hu Q, Maffucci KG, Qu Y. Novel Bletilla striata polysaccharide microneedles: Fabrication, characterization, and in vitro transcutaneous drug delivery. International Journal of Biological Macromolecules. 2018;117:928-36. doi: 10.1016/j.ijbiomac.2018.05.097. PubMed PMID: WOS:000442057700107.
2583. Hussein AE, Esfahani DR, Moisak GI, Rzaev JA, Slavin KV. Motor Cortex Stimulation for Deafferentation Pain. Curr Pain Headache Rep. 2018;22(6):45. Epub 2018/05/26. doi: 10.1007/s11916-018-0697-1. PubMed PMID: 29796941.
2584. Iliceto A, Seiler RL, Sarkar K. Repetitive Transcranial Magnetic Stimulation for Treatment of Depression in a Patient With Severe Traumatic Brain Injury. Ochsner J. 2018;18(3):264-7. Epub 2018/10/03. doi: 10.31486/toj.17.0075. PubMed PMID: 30275792; PubMed Central PMCID: PMCPMC6162118.
2585. Iovino L, Cooper K, Kinsella S, DeRoos P, Jain R, Galimberti S, et al. Zinc Supplementation Improves T Cell Reconstitution after

Allogeneic HSCT By Stimulating Endogenous Pathways of Thymic Regeneration. Blood. 2018;132. doi: 10.1182/blood-2018-99-112707. PubMed PMID: WOS:000454842801110.

2586. Irct20170808035562N. ?Effect of transcranial direct current stimulation on drug craving of addicts. https://trialsearchwhoint/Trial2aspx?TrialID=IRCT20170808035562N2. 2018. PubMed PMID: CN-01909612.

2587. Irct20171223038019N. Effect of rTMS on severity of Delirium. https://trialsearchwhoint/Trial2aspx?TrialID=IRCT20171223038019N1. 2018. PubMed PMID: CN-01902368.

2588. Irvine KA, David Clark J. Chronic pain after traumatic brain injury: Pathophysiology and pain mechanisms. Pain Medicine (United States). 2018;19(7):1315-33. doi: 10.1093/pm/pnx153.

2589. Ishai R, Knoll RM, Chen JX, Wong K, Reinshagen KL, Nadol JB, Jr., et al. Otopathologic Changes in the Cochlea following Head Injury without Temporal Bone Fracture. Otolaryngol Head Neck Surg. 2018;159(3):526-34. Epub 2018/06/13. doi: 10.1177/0194599818769861. PubMed PMID: 29890895.

2590. Izrael M, Slutsky SG, Admoni T, Cohen L, Granit A, Hasson A, et al. Safety and efficacy of human embryonic stem cell-derived astrocytes following intrathecal transplantation in SOD1(G93A) and

NSG animal models. Stem Cell Research & Therapy. 2018;9. doi: 10.1186/s13287-018-0890-5. PubMed PMID: WOS:000434911700001.
2591. Jang SH, Kwon Y. Injury of leg somatotopy of corticospinal tract at corona radiata by ventriculoperitoneal shunt: A case report. Medicine (Baltimore). 2018;97(10):e9983. Epub 2018/03/09. doi: 10.1097/md.0000000000009983. PubMed PMID: 29517704; PubMed Central PMCID: PMCPMC5882449 through the National Research Foundation of Korea (NRF) funded by the Ministry of Science, ICT, and Future Planning.Disclosures: Financial disclosure statements have been obtained, and no conflicts of interest have been reported by the authors or by any individuals in control of the content of this article.
2592. Jang SH, Seo YS. Effect of Neuromuscular Electrical Stimulation Training on the Finger Extensor Muscles for the Contralateral Corticospinal Tract in Normal Subjects: A Diffusion Tensor Tractography Study. Frontiers in Human Neuroscience. 2018;12. doi: 10.3389/fnhum.2018.00432. PubMed PMID: WOS:000450705800001.
2593. Jasey N, Ward I, Lequerica A, Chiaravalloti ND. The therapeutic value of cranioplasty in individuals with brain injury. Brain Injury. 2018;32(3):318-24. doi: 10.1080/02699052.2017.1419283.

2594. Jen E, Lin C-W, Hsieh T-H, Chiu Y-C, Lu T-C, Chen S-C, et al. Prototype Deep Brain Stimulation System with Closed-Loop Control Feedback for Modulating Bladder Functions in Traumatic Brain Injured Animals. Journal of Medical and Biological Engineering. 2018;38(3):337-49. doi: 10.1007/s40846-017-0326-1. PubMed PMID: WOS:000432631500001.

2595. Jiang T. Recent Progress in Basic and Clinical Research on Disorders of Consciousness. Neuroscience Bulletin. 2018;34(4):589-91. doi: 10.1007/s12264-018-0264-0. PubMed PMID: WOS:000440164500001.

2596. Johansson M, Ricci F, Aung N, Sutton R, Melander O, Fedorowski A. Proteomic Profiling for Cardiovascular Biomarker Discovery in Orthostatic Hypotension. Hypertension. 2018;71(3):465-72. doi: 10.1161/HYPERTENSIONAHA.117.10365.

2597. Jones W, Silkworth W, Dusto N, Pelak VS, Berliner J, Buard I, et al. Central Visual Oscillopsia: Case Report and Review of the Literature. Cogn Behav Neurol. 2018;31(2):86-95. Epub 2018/06/22. doi: 10.1097/wnn.0000000000000151. PubMed PMID: 29927799.

2598. Joutsa J, Shih LC, Horn A, Reich MM, Wu O, Rost NS, et al. Identifying therapeutic targets from spontaneous beneficial brain lesions. Ann Neurol. 2018;84(1):153-7. Epub 2018/07/18. doi: 10.1002/ana.25285. PubMed PMID: 30014594.

2599. Jung KI, Ohn SH, Lee H. GLOBAL SYNCHRONIZATION INDEX AS A NEW INDICATOR FOR TRACKING COGNITIVE FUNCTION CHANGES BY TRANSCRANIAL DIRECT CURRENT STIMULATION IN A TRAUMATIC BRAIN INJURED PATIENT: A CASE REPORT. Alzheimer's and Dementia. 2018;14(7):P289-P90. doi: 10.1016/j.jalz.2018.06.058.

2600. Kaczmarek BLJ, Markiewicz K. Current and traditional views on the brain works. Acta Neuropsychologica. 2018;16(2):201-12. doi: 10.5604/01.3001.0012.2052.

2601. Karim R, Lepeltier E, Esnault L, Pigeon P, Lemaire L, Lepinoux-Chambaud C, et al. Enhanced and preferential internalization of lipid nanocapsules into human glioblastoma cells: effect of a surface-functionalizing NFL peptide. Nanoscale. 2018;10(28):13485-501. doi: 10.1039/c8nr02132e. PubMed PMID: WOS:000439319000022.

2602. Kelchen MN, Brogden NK. Effect of dosing regimen and microneedle pretreatment on in vitro skin retention of topically applied beta-blockers. Biomedical Microdevices. 2018;20(4). doi: 10.1007/s10544-018-0348-7. PubMed PMID: WOS:000452462600002.

2603. Kharchenko EP, Tel'nova MN. Brain Plasticity: Limitations and Possibilities. Neuroscience and Behavioral Physiology.

2018;48(5):603-7. doi: 10.1007/s11055-018-0606-4.
2604. Khatoun A, Breukers J, De Beeck SO, Nica IG, Aerts JM, Seynaeve L, et al. Using high-amplitude and focused transcranial alternating current stimulation to entrain physiological tremor. Scientific Reports. 2018;8(1). doi: 10.1038/s41598-018-23290-w.
2605. Khodaei S, Avramescu S, Lecker I, Whissell P, Wang DS, Baker AJ, et al. Inhibiting α5GABAA receptors restores synaptic plasticity in the hippocampus after traumatic brain injury in mice. Anesthesia and Analgesia. 2018;126(4):775-6.
2606. Kim C, Park JM, Kong TH, Lee S, Seo KW, Choi Y, et al. Double-Injected Human Stem Cells Enhance Rehabilitation in TBI Mice Via Modulation of Survival and Inflammation. Molecular Neurobiology. 2018;55(6):4870-84. doi: 10.1007/s12035-017-0683-3.
2607. Kim CH, Shin JE. Hemorrhage within the tympanic membrane without perforation. Journal of Otolaryngology - Head and Neck Surgery. 2018;47(1). doi: 10.1186/s40463-018-0300-0.
2608. Kim H, Koo YS, Shin MJ, Kim SY, Shin YB, Choi BT, et al. Combination of Constraint-Induced Movement Therapy with Electroacupuncture Improves Functional Recovery following Neonatal Hypoxic-Ischemic Brain Injury in Rats. Biomed Res Int. 2018;2018:8638294. Epub 2018/03/24. doi: 10.1155/2018/8638294. PubMed PMID: 29568769; PubMed Central PMCID:

PMCPMC5820667.
2609. King D. Use of the King-Devick test and Brain Gauge for the management of concussion. Journal of Science and Medicine in Sport. 2018;21:S38-S9. doi: 10.1016/j.jsams.2018.09.089.
2610. Koenig MA, Kaplan PW. Measuring Depth in Still Water: Electrophysiologic Indicators of Residual Consciousness in the Unresponsive Patient. Epilepsy Curr. 2018;18(3):147-50. Epub 2018/06/29. doi: 10.5698/1535-7597.18.3.147. PubMed PMID: 29950932; PubMed Central PMCID: PMCPMC6017691.
2611. Kuffler DP. Coping with Phantom Limb Pain. Mol Neurobiol. 2018;55(1):70-84. Epub 2017/09/19. doi: 10.1007/s12035-017-0718-9. PubMed PMID: 28921418.
2612. Kuffler DP. Origins of Phantom Limb Pain. Mol Neurobiol. 2018;55(1):60-9. Epub 2017/09/19. doi: 10.1007/s12035-017-0717-x. PubMed PMID: 28921474.
2613. Kundu A, Ausaf T, Rajaraman S. 3D Printing, Ink Casting and Micromachined Lamination (3D PICLM): A Makerspace Approach to the Fabrication of Biological Microdevices. Micromachines. 2018;9(2). doi: 10.3390/mi9020085. PubMed PMID: WOS:000427530300045.
2614. Kundu B, Brock AA, Englot DJ, Butson CR, Rolston JD. Deep brain stimulation for the treatment of disorders of consciousness

and cognition in traumatic brain injury patients: a review. Neurosurg Focus. 2018;45(2):E14. Epub 2018/08/02. doi: 10.3171/2018.5.Focus18168. PubMed PMID: 30064315; PubMed Central PMCID: PMCPMC6193266.

2615. Kureshi N, Clarke DB. Neurotrauma Research at Dalhousie University: A Snapshot of Past and Present Developments. Journal of Neurotrauma. 2018;35(16):1993-4. PubMed PMID: WOS:000441527400762.

2616. Latchoumane C-FV, Jackson L, Sendi MSE, Tehrani KF, Mortensen LJ, Stice SL, et al. Chronic Electrical Stimulation Promotes the Excitability and Plasticity of ESC-derived Neurons following Glutamate-induced Inhibition In vitro. Scientific Reports. 2018;8. doi: 10.1038/s41598-018-29069-3. PubMed PMID: WOS:000439102400013.

2617. Laureys S. Neuromodulation: Outlook into the future. European Journal of Neurology. 2018;25:11.

2618. Lee G, Ma Y, Lee Y-h, Jung H. Clinical Evaluation of a Low-pain Long Microneedle for Subcutaneous Insulin Injection. Biochip Journal. 2018;12(4):309-16. doi: 10.1007/s13206-018-2411-0. PubMed PMID: WOS:000453682800007.

2619. Lee HN, Oh Y-B, Kim G-W, Hui WY, Hwan KM, Hwan SJ, et al. Localization of Bilateral Hemisphere Lesion Using Combined

Transcranial Magnetic Stimulation and Diffusion Tensor Imaging: Report of Two Cases. Journal of Electrodiagnosis and Neuromuscular Diseases. 2018;20(2):106-11. doi: 10.18214/jkaem.2018.20.2.106. PubMed PMID: KJD:ART002425419.

2620. Lee SA, Kim MK. Effect of Low Frequency Repetitive Transcranial Magnetic Stimulation on Depression and Cognition of Patients with Traumatic Brain Injury: A Randomized Controlled Trial. Med Sci Monit. 2018;24:8789-94. Epub 2018/12/05. doi: 10.12659/msm.911385. PubMed PMID: 30513530; PubMed Central PMCID: PMCPMC6289027.

2621. Lefebvre G, Chamard E, Proulx S, Tremblay S, Halko M, Soman S, et al. Increased Myo-Inositol in Primary Motor Cortex of Contact Sports Athletes without a History of Concussion. Journal of Neurotrauma. 2018;35(7):953-62. doi: 10.1089/neu.2017.5254. PubMed PMID: WOS:000424833600001.

2622. Legarreta AD, Monk SH, Kirby PW, Brett BL, Yengo-Kahn AM, Bhatia A, et al. Long-Term Neuroimaging Findings in American Football Players: Systematic Review. World Neurosurg. 2018;120:e365-e79. Epub 2018/08/26. doi: 10.1016/j.wneu.2018.08.079. PubMed PMID: 30144599.

2623. Leung A, Metzger-Smith V, He Y, Cordero J, Ehlert B, Song D, et al. Left Dorsolateral Prefrontal Cortex rTMS in Alleviating MTBI

Related Headaches and Depressive Symptoms. Neuromodulation. 2018;21(4):390-401. Epub 2017/05/31. doi: 10.1111/ner.12615. PubMed PMID: 28557049.

2624. Li F, Ball S, Katz B, Smith A. Case report of syncope during a transcranial direct current stimulation experiment in a healthy adult participant. Brain Stimul. 2018;11(5):1201-2. Epub 2018/08/28. doi: 10.1016/j.brs.2018.06.008. PubMed PMID: 30146042.

2625. Li Y, Luo X, Wan M, Li J, Wang H, Wei D, et al. The effectiveness of non-invasive brain stimulation on arousal and alertness in patients in coma or persistent vegetative state after traumatic brain injury: Protocol of systematic review and network meta-analysis. Medicine (Baltimore). 2018;97(37):e12321. Epub 2018/09/15. doi: 10.1097/md.0000000000012321. PubMed PMID: 30212977; PubMed Central PMCID: PMCPMC6155968.

2626. Liao C, Sun W, Feng Z. Effects of transcranial direct current stimulation on consciousness and expression of brain-derived neurotrophic factor in coma rats following traumatic brain injury. Chinese Journal of Rehabilitation Medicine. 2018;33(3):269-73,85. PubMed PMID: CSCD:6203324.

2627. Liu D, Yu B, Jiang G, Yu W, Zhang Y, Xu B. Fabrication of composite microneedles integrated with insulin-loaded CaCO3 microparticles and PVP for transdermal delivery in diabetic rats.

Materials Science & Engineering C-Materials for Biological Applications. 2018;90:180-8. doi: 10.1016/j.msec.2018.04.055. PubMed PMID: WOS:000435619600019.

2628. Liu J, Xu F, Huang J, Xu J, Liu Y, Yao Y, et al. Low-intensity focused ultrasound (LIFU)-activated nanodroplets as a theranostic agent for noninvasive cancer molecular imaging and drug delivery. Biomaterials Science. 2018;6(11). doi: 10.1039/c8bm00726h. PubMed PMID: WOS:000448822800004.

2629. Liu J, Xue X, Wu Y, Yang C, Li N, Li H. Efficacy and safety of electro-acupuncture treatment in improving the consciousness of patients with traumatic brain injury: study protocol for a randomized controlled trial. Trials. 2018;19(1):296. Epub 2018/05/31. doi: 10.1186/s13063-018-2687-3. PubMed PMID: 29843761; PubMed Central PMCID: PMCPMC5975471.

2630. Liu X, Meng F, Gao J, Zhang L, Zhou Z, Pan G, et al. Behavioral and Resting State Functional Connectivity Effects of High Frequency rTMS on Disorders of Consciousness: A Sham-Controlled Study. Front Neurol. 2018;9:982. Epub 2018/12/07. doi: 10.3389/fneur.2018.00982. PubMed PMID: 30519211; PubMed Central PMCID: PMCPMC6258881.

2631. Liu X, Quan N. Microglia and CNS Interleukin-1: Beyond Immunological Concepts. Frontiers in Neurology. 2018;9. doi:

10.3389/fneur.2018.00008. PubMed PMID: WOS:000423072300002.
2632. Luzuriaga MA, Berry DR, Reagan JC, Smaldone RA, Gassensmith JJ. Biodegradable 3D printed polymer microneedles for transdermal drug delivery. Lab on a Chip. 2018;18(8):1223-30. doi: 10.1039/c8lc00098k. PubMed PMID: WOS:000430406400007.
2633. Maines M, Zorzi A, Tomasi G, Angheben C, Catanzariti D, Piffer L, et al. Clinical impact, safety, and accuracy of the remotely monitored implantable loop recorder Medtronic Reveal LINQ TM. Europace. 2018;20(6):1050-7. doi: 10.1093/europace/eux187.
2634. Mak A, Dharmadhikari B, Wong LW, Sreedharan SK, Schwarz H. The differential impact of the abrogation of the costimulatory molecule CD137 ligand on renal and cerebral manifestations in C57BL/6.FASLPR-/- mice. Arthritis and Rheumatology. 2018;70:2318-9. doi: 10.1002/art.40700.
2635. Maltese A, Salerno M, Cerroni F, Romano P, Di Folco A, Di Filippo T, et al. THE TRANSCRANIAL STIMULATION WITH DIRECT CURRENTS (TDCS): AN HISTORICAL AND CONCEPTUAL MINIREVIEW. Acta Medica Mediterranea. 2018;34(3):697-702. doi: 10.19193/0393-6384_2018_3_107. PubMed PMID: WOS:000434980000012.
2636. Manoj NK, Singh U, Shahid NA. Pilot study on outcome of brief right median nerve stimulation and multimodal sensory

stimulation in comatose patients. Neurorehabilitation and Neural Repair. 2018;32(4-5):475-6. doi: 10.1177/1545968318765498.
2637. Marcelo G, Ariana-Machado J, Enea M, Carmo H, Rodriguez-Gonzalez B, Capelo JL, et al. Toxicological Evaluation of Luminescent Silica Nanoparticles as New Drug Nanocarriers in Different Cancer Cell Lines. Materials. 2018;11(8). doi: 10.3390/ma11081310. PubMed PMID: WOS:000444112800047.
2638. Martens G, Lejeune N, O'Brien AT, Fregni F, Martial C, Wannez S, et al. Randomized controlled trial of home-based 4-week tDCS in chronic minimally conscious state. Brain Stimul. 2018;11(5):982-90. Epub 2018/05/16. doi: 10.1016/j.brs.2018.04.021. PubMed PMID: 29759943.
2639. Matheson N, Fisher S, Shemmell J, Reynolds J. The cellular effects of rTMS in healthy and stroke-damaged brains: An in vivo intracellular electrophysiological study in the rat. Neurorehabilitation and Neural Repair. 2018;32(12):1080. doi: 10.1177/1545968318817151.
2640. Mayer AR, Kaushal M, Dodd AB, Hanlon FM, Shaff NA, Mannix R, et al. Advanced biomarkers of pediatric mild traumatic brain injury: Progress and perils. Neurosci Biobehav Rev. 2018;94:149-65. Epub 2018/08/14. doi: 10.1016/j.neubiorev.2018.08.002. PubMed PMID: 30098989; PubMed

Central PMCID: PMCPMC6221189.
2641. Meiron O, Gale R, Namestnic J, Bennet-Back O, David J, Gebodh N, et al. High-Definition transcranial direct current stimulation in early onset epileptic encephalopathy: a case study. Brain Injury. 2018;32(1):135-43. doi: 10.1080/02699052.2017.1390254.
2642. Mishra R, Maiti TK, Bhattacharyya TK. Development of SU-8 hollow microneedles on a silicon substrate with microfluidic interconnects for transdermal drug delivery. Journal of Micromechanics and Microengineering. 2018;28(10). doi: 10.1088/1361-6439/aad301. PubMed PMID: WOS:000440308700002.
2643. Mishra R, Matti TK, Bhattacharyya TK. Design and Scalable Fabrication of Hollow SU-8 Microneedles for Transdermal Drug Delivery. Ieee Sensors Journal. 2018;18(14):5635-44. doi: 10.1109/jsen.2018.2840335. PubMed PMID: WOS:000437024600002.
2644. Nagai M, Tanaka N, Oouchida Y, Izumi SI. Effect of Cathodal Transcranial Direct Current Stimulation on a Child with Involuntary Movement after Hypoxic Encephalopathy. Case Reports in Medicine. 2018;2018. doi: 10.1155/2018/8934253.
2645. Naro A, Bramanti A, Leo A, Bramanti P, Calabrò RS. Metaplasticity: A Promising Tool to Disentangle Chronic Disorders of

Consciousness Differential Diagnosis. Int J Neural Syst. 2018;28(6):1750059. Epub 2018/01/27. doi: 10.1142/s0129065717500599. PubMed PMID: 29370729.

2646. Naro A, Bramanti A, Leo A, Cacciola A, Manuli A, Bramanti P, et al. Shedding new light on disorders of consciousness diagnosis: The dynamic functional connectivity. Cortex. 2018;103:316-28. Epub 2018/04/24. doi: 10.1016/j.cortex.2018.03.029. PubMed PMID: 29684751.

2647. Naro A, Calabrò RS, Leo A, Russo M, Milardi D, Cannavò A, et al. Bridging the Gap Towards Awareness Detection in Disorders of Consciousness: An Experimental Study on the Mirror Neuron System. Brain Topogr. 2018;31(4):623-39. Epub 2018/02/09. doi: 10.1007/s10548-018-0628-9. PubMed PMID: 29417320.

2648. Naro A, Chillura A, Portaro S, Bramanti A, De Luca R, Bramanti P, et al. Novel approaches to the diagnosis of chronic disorders of consciousness: Detecting peripersonal space by using ultrasonics. Frontiers in Neurology. 2018;9(FEB). doi: 10.3389/fneur.2018.00047.

2649. Nct. Efficacy of Repetitive Transcranial Magnetic Stimulation for Improvement of Memory in Older Adults With TBI. https://clinicaltrialsgov/show/NCT03727737. 2018. PubMed PMID: CN-01794719.

2650. Nct. fMRI-neuronavigated rTMS Treatment for Symptoms of Depression Associated With Concussive TBI in the Military Population. https://clinicaltrialsgov/show/NCT03523507. 2018. PubMed PMID: CN-01574383.

2651. Nct. rTMS for Cognitive Rehabilitation After TBI. https://clinicaltrialsgov/show/NCT03642158. 2018. PubMed PMID: CN-01662504.

2652. Nct. rTMS Treatment of Persistent Headache and Post Concussion Symptoms Attributed to Mild Traumatic Injury to the Head. https://clinicaltrialsgov/show/NCT03691272. 2018. PubMed PMID: CN-01663757.

2653. Nct. The Effects of Low-intensity Blood-flow Restricted Exercise on Upper Limb Function Following Spinal Cord Injury. https://clinicaltrialsgov/show/NCT03690700. 2018. PubMed PMID: CN-01663748.

2654. Nct. Investigation and Modulation of the Mu-opioid Mechanisms in TMD (in Vivo). https://clinicaltrialsgov/show/NCT03724032. 2018. PubMed PMID: CN-01964986.

2655. Nct. Grasping Function After Spinal Cord Injury. https://clinicaltrialsgov/show/NCT03447509. 2018. PubMed PMID: CN-01589533.

2656. Nct. Non-Invasive Stimulation for Improving Motor Function. https://clinicaltrialsgov/show/NCT03592173. 2018. PubMed PMID: CN-01661238.

2657. Nct. The Effects of rTMS in Rehabilitation Following Spinal Cord Injury. https://clinicaltrialsgov/show/NCT03690726. 2018. PubMed PMID: CN-01663749.

2658. Nct. Combined Robotic Training and tDCS in Chronic SCI. https://clinicaltrialsgov/show/NCT03555838. 2018. PubMed PMID: CN-01609221.

2659. Nct. Treatment of Memory Disorders in Gulf War Illness With High Definition Transcranial Direct Cortical Stimulation. https://clinicaltrialsgov/show/NCT03542383. 2018. PubMed PMID: CN-01659983.

2660. Nct. Activity-Dependent Transspinal Stimulation in SCI. https://clinicaltrialsgov/show/NCT03669302. 2018. PubMed PMID: CN-01663224.

2661. Nct. Neuromodulation for Exercise Adherence. https://clinicaltrialsgov/show/NCT03733041. 2018. PubMed PMID: CN-01658712.

2662. Nct. Post-concussion and Transcranial Direct Current Stimulation. https://clinicaltrialsgov/show/NCT03481153. 2018. PubMed PMID: CN-01589260.

2663. Nct. CONsciousness Transcranial Electric STimulation. https://clinicaltrialsgov/show/NCT03576248. 2018. PubMed PMID: CN-01660835.

2664. Nelson DV, Esty ML. Minute Pulsed Electromagnetic Neurostimulation for Mixed Trauma Syndromes. J Evid Based Integr Med. 2018;23:2515690x18770136. Epub 2018/04/26. doi: 10.1177/2515690x18770136. PubMed PMID: 29692181; PubMed Central PMCID: PMCPMC5987890.

2665. Nemanich S, Rich T, Mueller B, Opitz A, Gillick B. Multimodal neurophysiologic and neuroanatomic assessment of brain connectivity in pediatric stroke. Developmental Medicine and Child Neurology. 2018;60:19-20. doi: 10.1111/dmcn.27_14017.

2666. Neville IS, Gomes-Osman J, Amorim RLO, Hayashi CY, Galhardoni R, Zaninotto AL, et al. How can transcranial magnetic stimulation change the way we treat traumatic brain injury? International Journal of Clinical and Experimental Medicine. 2018;11(8):7643-50. PubMed PMID: WOS:000443533700014.

2667. Orfila JE, Clevenger AC, Dietz RM, Patsos OP, Schroeder C, Schmidt R, et al. A novel peptide inhibitor of TRPM2 channels reduces memory deficits and improves LTP following traumatic brain injury in mice. Journal of Neurotrauma. 2018;35(16):A138. doi: 10.1089/neu.2018.29013.abstracts.

2668. Oxley TJ, Opie NL, Rind GS, Liyanage K, John SE, Ronayne S, et al. An ovine model of cerebral catheter venography for implantation of an endovascular neural interface. Journal of Neurophysiology. 2018;128(4):1020-7. doi: 10.3171/2016.11.JNS161754.
2669. Padmanabhan P, Kataria P, Padmanaban V, Srinivas SP, Ambrosio R. Accuracy of integrated corneal biomechanical and tomographic data for detecting subclinical keratectasia. Investigative ophthalmology & visual science. 2018;59(9). PubMed PMID: CN-01960158.
2670. Paxman E, Stilling J, Mercier L, Debert CT. Repetitive transcranial magnetic stimulation (rTMS) as a treatment for chronic dizziness following mild traumatic brain injury. BMJ Case Rep. 2018;2018. Epub 2018/11/07. doi: 10.1136/bcr-2018-226698. PubMed PMID: 30396889; PubMed Central PMCID: PMCPMC6229180.
2671. Pearce AJ, Rist B, Fraser CL, Cohen A, Maller JJ. Neurophysiological and cognitive impairment following repeated sports concussion injuries in retired professional rugby league players. Brain Inj. 2018;32(4):498-505. Epub 2018/02/02. doi: 10.1080/02699052.2018.1430376. PubMed PMID: 29388850.
2672. Pei LX, Zhang W, Song YF, Geng H, Chen L, Wu XL, et al. [Electroacupuncture of "Tianshu" (ST 25) Suppresses Visceral Pain

Possibly by Down-regulating Mast Cell Activation, and Tryptase and SP Expression in Rats with Post-infectious Irritable Bowel Syndrome]. Zhen Ci Yan Jiu. 2018;43(7):419-23. Epub 2018/08/11. doi: 10.13702/j.1000-0607.180211. PubMed PMID: 30094977.

2673. Piccillo GA, Mondati EGM, Gasbarrini G. A severe and rare kind of headache drastically reduced the quality of life of a young unfortunate woman…. Italian Journal of Medicine. 2018;12(2):95. doi: 10.4081/itjm.2018.s2.

2674. Pixa NH, Pollok B. Effects of tDCS on bimanual motor skills: A brief review. Frontiers in Behavioral Neuroscience. 2018;12. doi: 10.3389/fnbeh.2018.00063.

2675. Popova M, Trendafilova I, Szegedi A, Momekova D, Mihaly J, Momekov G, et al. Novel SO3H functionalized magnetic nanoporous silica/polymer nanocomposite as a carrier in a dual-drug delivery system for anticancer therapy. Microporous and Mesoporous Materials. 2018;263:96-105. doi: 10.1016/j.micromeso.2017.12.005. PubMed PMID: WOS:000434239700012.

2676. Portaro S, Naro A, Bramanti A, Leo A, Manuli A, Balletta T, et al. Beyond the muscular involvement in non-dystrophic myotonias: The emerging role of neuromodulation. Restorative Neurology and Neuroscience. 2018;36(4):459-67. doi: 10.3233/RNN-170796.

2677. Poydasheva A, Legostaeva L, Mochalova E, Kremneva E,

Sinitsyn D, Sergeev D, et al. RTMS application in disorders of consciousness. Clinical Neurophysiology. 2018;129:e153. doi: 10.1016/j.clinph.2018.04.389.

2678. Qu X, Dong Y, Jin H, Hou L. Research progress in non-invasive brain stimulation technique for rehabilitation of traumatic brain injury. Chinese Journal of Trauma. 2018;34(8):759-63. PubMed PMID: CSCD:6313390.

2679. Quesada C, Pommier B, Fauchon C, Bradley C, Créac'h C, Vassal F, et al. Robot-Guided Neuronavigated Repetitive Transcranial Magnetic Stimulation (rTMS) in Central Neuropathic Pain. Arch Phys Med Rehabil. 2018;99(11):2203-15.e1. Epub 2018/05/12. doi: 10.1016/j.apmr.2018.04.013. PubMed PMID: 29750900.

2680. Quinn DK, Mayer AR, Master CL, Fann JR. Prolonged postconcussive symptoms. American Journal of Psychiatry. 2018;175(2):103-11. doi: 10.1176/appi.ajp.2017.17020235.

2681. Rai S, Drislane FW. Treatment of Refractory and Super-refractory Status Epilepticus. Neurotherapeutics. 2018;15(3):697-712. doi: 10.1007/s13311-018-0640-5. PubMed PMID: WOS:000441999200014.

2682. Raij T, Nummenmaa A, Marin MF, Porter D, Furtak S, Setsompop K, et al. Prefrontal Cortex Stimulation Enhances Fear Extinction Memory in Humans. Biological Psychiatry.

2018;84(2):129-37. doi: 10.1016/j.biopsych.2017.10.022.

2683. Raine A. Antisocial Personality as a Neurodevelopmental Disorder. Annual Review of Clinical Psychology2018. p. 259-89.

2684. Ramirez-Zamora A, Giordano JJ, Gunduz A, Brown P, Sanchez JC, Foote KD, et al. Evolving applications, technological challenges and future opportunities in neuromodulation: Proceedings of the fifth annual deep brain stimulation think tank. Frontiers in Neuroscience. 2018;11(JAN). doi: 10.3389/fnins.2017.00734.

2685. Rivera-Lillo G, Rojas-Líbano D, Burgos P, Egaña JI, Chennu S, Maldonado PE. Reduced delta-band modulation underlies the loss of P300 responses in disorders of consciousness. Clinical Neurophysiology. 2018;129(12):2613-22. doi: 10.1016/j.clinph.2018.09.104.

2686. Romeo-Guitart D, Leiva-Rodríguez T, Espinosa-Alcantud M, Sima N, Vaquero A, Domínguez-Martín H, et al. SIRT1 activation with neuroheal is neuroprotective but SIRT2 inhibition with AK7 is detrimental for disconnected motoneurons. Cell Death Dis. 2018;9(5):531. Epub 2018/05/12. doi: 10.1038/s41419-018-0553-6. PubMed PMID: 29748539; PubMed Central PMCID: PMCPMC5945655 under PCT extension.

2687. Rosanova M, Fecchio M, Casarotto S, Sarasso S, Casali AG, Pigorini A, et al. Sleep-like cortical OFF-periods disrupt causality and

complexity in the brain of unresponsive wakefulness syndrome patients. Nat Commun. 2018;9(1):4427. Epub 2018/10/26. doi: 10.1038/s41467-018-06871-1. PubMed PMID: 30356042; PubMed Central PMCID: PMCPMC6200777.

2688. Rosenow JM, Blabas BC, Keltzel S, Herrold A, Parrish T, Guernon A, et al. RTMS and amantadine for ppersons in chronic states of disordered consciousness after TBI. Neuromodulation. 2018;21(3):e79. doi: 10.1111/ner.12774.

2689. Rossini PM. Connectivity disturbances in stroke as revealed by integrated multimodal neuroimaging by fMRI, EEG and TMS-EEG. European Journal of Neurology. 2018;25:633.

2690. Rothen N, Schwartzman DJ, Bor D, Seth AK. Coordinated neural, behavioral, and phenomenological changes in perceptual plasticity through overtraining of synesthetic associations. Neuropsychologia. 2018;111:151-62. doi: 10.1016/j.neuropsychologia.2018.01.030. PubMed PMID: WOS:000430644300019.

2691. Ryan K, Schiller K, Rezaie R, Baughman BC, Narayana S. A single season of soccer results in no significant neurophysiological changes in adolescent female athletes. Neurology. 2018;91(23). doi: 10.1212/01.wnl.0000550672.77974.75. PubMed PMID: WOS:000541962800039.

2692. Ryan K, Wawrzyn K, Gati JS, Chronik BA, Wong D, Duggal N, et al. 1H MR spectroscopy of the motor cortex immediately following transcranial direct current stimulation at 7 Tesla. PLoS ONE. 2018;13(8). doi: 10.1371/journal.pone.0198053.

2693. Ryan NP, Genc S, Beauchamp MH, Yeates KO, Hearps S, Catroppa C, et al. White matter microstructure predicts longitudinal social cognitive outcomes after paediatric traumatic brain injury: a diffusion tensor imaging study. Psychol Med. 2018;48(4):679-91. Epub 2017/08/07. doi: 10.1017/s0033291717002057. PubMed PMID: 28780927.

2694. Sanders RD, Banks MI, Darracq M, Moran R, Sleigh J, Gosseries O, et al. Propofol-induced unresponsiveness is associated with impaired feedforward connectivity in cortical hierarchy. Br J Anaesth. 2018;121(5):1084-96. Epub 2018/10/20. doi: 10.1016/j.bja.2018.07.006. PubMed PMID: 30336853; PubMed Central PMCID: PMCPMC6208295.

2695. Sanger TD, Liker M, Arguelles E, Deshpande R, Maskooki A, Ferman D, et al. Pediatric deep brain stimulation using awake recording and stimulation for target selection in, we inpatient neuromodulation monitoring unit. Brain Sciences. 2018;8(7). doi: 10.3390/brainsci8070135.

2696. Schellack N, Masuku P, Mogale K, Mokwele N, Skosana P.

Hands on management of muscle pain and injuries. SA Pharmaceutical Journal. 2018;85(6):27-36.
2697. Schmidt J, Brown KE, Feldman SJ, Babul S, Zwicker JG, Boyd LA. Evidence of altered interhemispheric communication in pediatric mild traumatic brain injury. Neurorehabilitation and Neural Repair. 2018;32(12):1102. doi: 10.1177/1545968318817151.
2698. Schmidt J, Hayward KS, Brown KE, Zwicker JG, Ponsford J, van Donkelaar P, et al. Imaging in Pediatric Concussion: A Systematic Review. Pediatrics. 2018;141(5). doi: 10.1542/peds.2017-3406. PubMed PMID: WOS:000431231300028.
2699. Schmidt J, Rubino C, Boyd LA, Virji-Babul N. The Role of Physical Activity in Recovery From Concussion in Youth: A Neuroscience Perspective. Journal of Neurologic Physical Therapy. 2018;42(3):155-62. doi: 10.1097/npt.0000000000000226. PubMed PMID: WOS:000436873100007.
2700. Sedaghat MR, Momeni-Moghaddam H, Naroo SS, Ghavamsaeedi H, Vahedi A. Dysfunctional lens syndrome. International Ophthalmology. 2018;38(4):1759-63. doi: 10.1007/s10792-017-0622-3.
2701. Sekar S, Zhang Y, Taghibiglou C. Role of low field magnetic stimulation on cognitive and motor functions in traumatic brain injury mouse model. Journal of Neurotrauma. 2018;35(16):A137. doi:

10.1089/neu.2018.29013.abstracts.

2702. Servia L, Serrano JCE, Pamplona R, Badia M, Montserrat N, Portero-Otin M, et al. Location-dependent effects of trauma on oxidative stress in humans. PLoS ONE. 2018;13(10). doi: 10.1371/journal.pone.0205519.

2703. Seven YB, Nichols NL, Kelly MN, Hobson OR, Satriotomo I, Mitchell GS. Compensatory plasticity in diaphragm and intercostal muscle utilization in a rat model of ALS. Exp Neurol. 2018;299(Pt A):148-56. Epub 2017/10/24. doi: 10.1016/j.expneurol.2017.10.015. PubMed PMID: 29056361; PubMed Central PMCID: PMCPMC5951687.

2704. Sgandurra G, Biagi L, Guzzetta A, Fogassi L, Ferrari A, Cioni G, et al. Reorganization of action observation network in children with unilateral cerebral palsy: An fMRI study. Developmental Medicine and Child Neurology. 2018;60:16-7. doi: 10.1111/dmcn.22_14017.

2705. Shin SS, Krishnan V, Stokes W, Robertson C, Celnik P, Chen Y, et al. Transcranial magnetic stimulation and environmental enrichment enhances cortical excitability and functional outcomes after traumatic brain injury. Brain Stimul. 2018;11(6):1306-13. Epub 2018/08/08. doi: 10.1016/j.brs.2018.07.050. PubMed PMID: 30082198; PubMed Central PMCID: PMCPMC6204305.

2706. Siddiqi SH, Trapp NT, Hacker CD, Laumann TO, Kandala S,

Hong X, et al. Repetitive transcranial magnetic stimulation with resting state network targeting for treatment-resistant depression in TBI. Journal of neurotrauma. 2018;35(16):A15-. doi: 10.1089/neu.2018.29013.abstracts. PubMed PMID: CN-01647811.

2707. Simon-Martinez C, Jaspers E, Mailleux L, Ortibus E, Klingels K, Wenderoth N, et al. Corticospinal tract wiring and brain lesion characteristics in unilateral cerebral palsy: Determinants of upper limb motor and sensory function. Neurorehabilitation and Neural Repair. 2018;32(12):1087. doi: 10.1177/1545968318817151.

2708. Simon-Martinez C, Mailleux L, Ortibus E, Fehrenbach A, Sgandurra G, Cioni G, et al. Combining constraint-induced movement therapy and action-observation training in children with unilateral cerebral palsy: a randomized controlled trial. BMC Pediatr. 2018;18(1):250. Epub 2018/08/02. doi: 10.1186/s12887-018-1228-2. PubMed PMID: 30064396; PubMed Central PMCID: PMCPMC6069849.

2709. Smith AT, Gorassini MA. Hyperexcitability of brain stem pathways in cerebral palsy. Journal of Neurophysiology. 2018;120(3):1428-37. doi: 10.1152/jn.00185.2018.

2710. Sokhadze EM, Lamina EV, Casanova EL, Kelly DP, Opris I, Tasman A, et al. Exploratory study of rTMS neuromodulation effects on electrocortical functional measures of performance in an oddball

test and behavioral symptoms in autism. Frontiers in Systems Neuroscience. 2018;12. doi: 10.3389/fnsys.2018.00020.

2711. Sollmann N, Wildschuetz N, Kelm A, Conway N, Moser T, Bulubas L, et al. Associations between clinical outcome and navigatedtranscranial magnetic stimulation characteristics inpatients with motor-eloquent brain lesions: A combinednavigated transcranial magnetic stimulation-diffusiontensor imaging fiber tracking approach. Journal of Neurophysiology. 2018;128(3):800-10. doi: 10.3171/2016.11.JNS162322.

2712. Song M, Zhang Y, Cui Y, Yang Y, Jiang T. Brain Network Studies in Chronic Disorders of Consciousness: Advances and Perspectives. Neuroscience Bulletin. 2018;34(4):592-604. doi: 10.1007/s12264-018-0243-5. PubMed PMID: WOS:000440164500002.

2713. Steele VR, Maxwell AM, Ross TJ, Moussawi K, Abulseoud OO, Stein EA, et al. Report of transient events in a cocaine-dependent volunteer who received iTBS. Brain Stimulation. 2018;11(3):631-3. doi: 10.1016/j.brs.2018.01.004.

2714. Stilling J, Duszynski C, Oni I, Paxman E, Dunn J, Debert C. Using functional near-infrared spectroscopy to study the effect of repetitive transcranial magnetic stimulation in concussion. Journal of Neurotrauma. 2018;35(16):A103-A4. doi:

10.1089/neu.2018.29013.abstracts.
2715. Subramanian S, Fountain M, Hood A. Interventions to augment upper extremity motor improvement in individuals with a traumatic brain injury: A systematic review. Neurorehabilitation and Neural Repair. 2018;32(12):1110-1. doi: 10.1177/1545968318817151.
2716. Sueri C, Gasparini S, Balestrini S, Labate A, Gambardella A, Russo E, et al. Diagnostic Biomarkers of Epilepsy. Curr Pharm Biotechnol. 2018;19(6):440-50. Epub 2018/07/14. doi: 10.2174/1389201019666180713095251. PubMed PMID: 30003857.
2717. Sundaravadhanan S. Neurotrauma: A Futuristic Perspective. Indian Journal of Neurotrauma. 2018;15(2-3):78-81. doi: 10.1055/s-0039-1694297.
2718. Surowiecka-Pastewka A, Witkowski W, Kawecki M. A new triage method for burn disasters: Fast triage in burns (FTB). Medical Science Monitor. 2018;24:1894-901. doi: 10.12659/MSM.905197.
2719. Taghdiri F, Chung J, Irwin S, Multani N, Tarazi A, Ebraheem A, et al. Decreased Number of Self-Paced Saccades in Post-Concussion Syndrome Associated with Higher Symptom Burden and Reduced White Matter Integrity. Journal of Neurotrauma. 2018;35(5):719-29. doi: 10.1089/neu.2017.5274. PubMed PMID: WOS:000424839300001.
2720. Takeuchi K, Kim B. Functionalized microneedles for

continuous glucose monitoring. Nano Convergence. 2018;5. doi: 10.1186/s40580-018-0161-2. PubMed PMID: WOS:000451707500001.

2721. Tanei T, Kajita Y, Maesawa S, Nakatsubo D, Aoki K, Noda H, et al. Long-term Effect and Predictive Factors of Motor Cortex and Spinal Cord Stimulation for Chronic Neuropathic Pain. Neurol Med Chir (Tokyo). 2018;58(10):422-34. Epub 2018/08/31. doi: 10.2176/nmc.oa.2018-0106. PubMed PMID: 30158352; PubMed Central PMCID: PMCPMC6186764.

2722. Tasca CI, Lanznaster D, Oliveira KA, Fernández-Dueñas V, Ciruela F. Neuromodulatory effects of guanine-based purines in health and disease. Frontiers in Cellular Neuroscience. 2018;12. doi: 10.3389/fncel.2018.00376.

2723. Tctr. Comparison of the mindfulness and transcranial stimulation on brain injury. https://trialsearchwhoint/Trial2aspx?TrialID=TCTR20180827003. 2018. PubMed PMID: CN-01992880.

2724. Thibaut A, Chatelle C, Vanhaudenhuyse A, Martens G, Cassol H, Martial C, et al. Transcranial direct current stimulation unveils covert consciousness. Brain Stimulation. 2018;11(3):642-4. doi: 10.1016/j.brs.2018.02.002.

2725. Thibaut A, Chennu S, Chatelle C, Martens G, Annen J, Cassol

H, et al. Theta network centrality correlates with tDCS response in disorders of consciousness. Brain Stimul. 2018;11(6):1407-9. Epub 2018/09/23. doi: 10.1016/j.brs.2018.09.002. PubMed PMID: 30241773.

2726. Thibaut A, Martens G, Kroupi E, Aureli Soria-Frisch A, Ruffini G, Laureys S. Stimulation of the frontoparietal network using tDCS in patients with disorders of consciousness: a double blind sham controlled randomised clinical trial. European journal of neurology. 2018;25:562-. PubMed PMID: CN-01630749.

2727. Thomas R, Mathew R. Right median nerve electrical stimulation (RMNS) to improve arousal in acquired brain injury patients a randomised controlled trial. Neurorehabilitation and neural repair. 2018;32(4-5):476-. doi: 10.1177/1545968318765498. PubMed PMID: CN-01785701.

2728. Tjen ALSC, Fu LW, Guo ZL, Longhurst JC. Modulation of Neurally Mediated Vasodepression and Bradycardia by Electroacupuncture through Opioids in Nucleus Tractus Solitarius. Sci Rep. 2018;8(1):1900. Epub 2018/02/01. doi: 10.1038/s41598-018-19672-9. PubMed PMID: 29382866; PubMed Central PMCID: PMCPMC5789879.

2729. Torrente A, Buscarnera S, Curto S, Di Stefano V, Cosentino G, Giglia G, et al. Transcranial direct current stimulation in post-stroke

aphasia rehabilitation: Bilateral vs unilateral online stimulation. European Journal of Neurology. 2018;25:565.

2730. Tremblay S, Pascual-Leone A, Théoret H. A review of the effects of physical activity and sports concussion on brain function and anatomy. Int J Psychophysiol. 2018;132(Pt A):167-75. Epub 2017/09/13. doi: 10.1016/j.ijpsycho.2017.09.005. PubMed PMID: 28893565.

2731. Trofimov AO, Kalentiev G, Karelsky M, Ksenofontova C, Ruzavina A, Yuriev M, et al. Cerebral Hemodynamics After Transcranial Direct Current Stimulation (tDCS) in Patients with Consequences of Traumatic Brain Injury. Adv Exp Med Biol. 2018;1072:59-62. Epub 2018/09/05. doi: 10.1007/978-3-319-91287-5_10. PubMed PMID: 30178324; PubMed Central PMCID: PMCPMC6312702.

2732. Turolla A, Venneri A, Farina D, Cagnin A, Cheung VCK. Rehabilitation Induced Neural Plasticity after Acquired Brain Injury. Neural Plasticity. 2018;2018. doi: 10.1155/2018/6565418. PubMed PMID: WOS:000432801000001.

2733. Ullah A, Kim CM, Kim GM. Porous polymer coatings on metal microneedles for enhanced drug delivery. Royal Society Open Science. 2018;5(4). doi: 10.1098/rsos.171609. PubMed PMID: WOS:000431110100020.

2734. Vallhov H, Xia W, Engqvist H, Scheynius A. Bioceramic microneedle arrays are able to deliver OVA to dendritic cells in human skin. Journal of Materials Chemistry B. 2018;6(42):6808-16. doi: 10.1039/c8tb01476k. PubMed PMID: WOS:000451177200011.

2735. Vargas-Saturno L, Ayala-Grosso C. Adaptive neurogenesis in the cerebral cortex and contralateral subventricular zone induced by unilateral cortical devascularization: Possible modulation by dopamine neurotransmission. Eur J Neurosci. 2018;48(12):3514-33. Epub 2018/11/08. doi: 10.1111/ejn.14260. PubMed PMID: 30402991.

2736. Verma MK, Goel R, Krishnadas N, Nemmani KVS. Targeting glucose-dependent insulinotropic polypeptide receptor for neurodegenerative disorders. Expert Opin Ther Targets. 2018;22(7):615-28. Epub 2018/06/19. doi: 10.1080/14728222.2018.1487952. PubMed PMID: 29911915.

2737. Vitale A, Lai Q, Farinati F, Bucci L, Giannini EG, Napoli L, et al. Utility of Tumor Burden Score to Stratify Prognosis of Patients with Hepatocellular Cancer: Results of 4759 Cases from ITA.LI.CA Study Group. Journal of Gastrointestinal Surgery. 2018;22(5):859-71. doi: 10.1007/s11605-018-3688-y.

2738. Vyas S. A conversation with Mehmet Fatih Yanik. 2018. p. 290-2.

2739. Wang T, Mantini D, Gillebert CR. The potential of real-time

fMRI neurofeedback for stroke rehabilitation: A systematic review. Cortex. 2018;107:148-65. doi: 10.1016/j.cortex.2017.09.006.
2740. Weinstein M, Green D, Rudisch J, Zielinski IM, Benthem-Muñiz M, Jongsma MLA, et al. Understanding the relationship between brain and upper limb function in children with unilateral motor impairments: A multimodal approach. European Journal of Paediatric Neurology. 2018;22(1):143-54. doi: 10.1016/j.ejpn.2017.09.012.
2741. Whalen AJ, Xiao Y, Kadji H, Dahlem MA, Gluckman BJ, Schiff SJ. Control of Spreading Depression with Electrical Fields. Scientific Reports. 2018;8. doi: 10.1038/s41598-018-26986-1. PubMed PMID: WOS:000434640800023.
2742. Wieckowska A, Gajewska-Wozniak O, Glowacka A, Ji B, Grycz K, Czarkowska-Bauch J, et al. Spinalization and locomotor training differentially affect muscarinic acetylcholine receptor type 2 abutting on -motoneurons innervating the ankle extensor and flexor muscles. Journal of Neurochemistry. 2018;147(3):361-79. doi: 10.1111/jnc.14567. PubMed PMID: WOS:000450112100005.
2743. Woytowicz EJ, Sours C, Gullapalli RP, Rosenberg J, Westlake KP. Modulation of working memory load distinguishes individuals with and without balance impairments following mild traumatic brain injury. Brain Injury. 2018;32(2):191-9. doi:

10.1080/02699052.2017.1403045.
2744. Wu M, Wu Y, Yu Y, Gao J, Meng F, He F, et al. Effects of theta burst stimulation of the left dorsolateral prefrontal cortex in disorders of consciousness. Brain Stimul. 2018;11(6):1382-4. Epub 2018/08/19. doi: 10.1016/j.brs.2018.07.055. PubMed PMID: 30120046.
2745. Wu V, Cooke B, Eitutis S, Simpson MTW, Beyea JA. Approach to tinnitus management. Canadian Family Physician. 2018;64(7):e293-e8.
2746. Xia X, Yang Y, Guo Y, Bai Y, Dang Y, Xu R, et al. Current Status of Neuromodulatory Therapies for Disorders of Consciousness. Neurosci Bull. 2018;34(4):615-25. Epub 2018/06/20. doi: 10.1007/s12264-018-0244-4. PubMed PMID: 29916112; PubMed Central PMCID: PMCPMC6060218.
2747. Yagi N, Itoh H, Hisamatsu T, Tomita Y, Kimura H, Fujii Y, et al. A challenge for mutation specific risk stratification in long QT syndrome type 1. Journal of Cardiology. 2018;72(1):56-65. doi: 10.1016/j.jjcc.2017.12.011.
2748. Yamasaki F, Kolakshyapati M, Hashizume A, Ochi K, Ueno H, Kaichi Y, et al. Histogram analysis in ring-enhancing intracranial lesions. Neuroradiology. 2018;60(10):1119. doi: 10.1007/s00234-018-2086-1.
2749. Yankevich DS, Borodin MM, Usoltseva NI, Shevtsova EE,

Kovaleva GA, Gorbeshko GA. Personalized patient neurorehabilitation in early post-coma period. The uniqueness of a single case. International Journal of Pharmaceutical Research. 2018;10(1):305-19.

2750. Yasen AL, Edwards EK, Eick G, Sterner K, Christie AD. Motor Cortex Function in Symptomatic and Asymptomatic Individuals Following Mild Traumatic Brain Injury. Medicine and Science in Sports and Exercise. 2018;50(5):564-. PubMed PMID: WOS:000456870502370.

2751. Yavari F, van Thriel C, Nitsche MA, Kuo MF. Effect of acute exposure to toluene on cortical excitability, neuroplasticity, and motor learning in healthy humans. Archives of Toxicology. 2018;92(10):3149-62. doi: 10.1007/s00204-018-2277-x.

2752. Yona Rabinovich Y, Shaklai S, Sacher Y, Cismariu Potash K, Ressel Zviely A. The effects of TDCS stimulation on motor learning in healthy and severe sub-acute traumatic brain injury adults. Annals of physical and rehabilitation medicine. 2018;(no pagination). doi: 10.1016/j.rehab.2018.05.086. PubMed PMID: CN-01610899.

2753. Yu K, Seal ML, Reyes J, Godfrey C, Anderson V, Adamson C, et al. Brain volumetric correlates of inhibition and cognitive flexibility 16 years following childhood traumatic brain injury. J Neurosci Res. 2018;96(4):642-51. Epub 2017/07/05. doi: 10.1002/jnr.24087.

PubMed PMID: 28675465.
2754. Yu KP, Yoon YS, Lee JG, Oh JS, Lee JS, Seog T, et al. Effects of Electric Cortical Stimulation (ECS) and Transcranial Direct Current Stimulation (tDCS) on Rats With a Traumatic Brain Injury. Ann Rehabil Med. 2018;42(4):502-13. Epub 2018/09/06. doi: 10.5535/arm.2018.42.4.502. PubMed PMID: 30180518; PubMed Central PMCID: PMCPMC6129703.
2755. Zardouz S, Leung A. Long term feasibility and efficacy of RTMS in alleviating MTBI headaches. Pain Medicine (United States). 2018;19(4):855-6. doi: 10.1093/pm/pny044.
2756. Zarshenas S, Cullen N. Association between cognitive ability and functional outcomes following traumatic brain injury- using a computer-based neurocognitive battery. Brain Injury. 2018;32(13-14):1678-83. doi: 10.1080/02699052.2018.1517223.
2757. Zhang D, Fu Q, Dionigi G, Wang T, Xin J, Zhang J, et al. Intraoperative Neural Monitoring in Endoscopic Thyroidectomy Via Bilateral Areola Approach. Surg Laparosc Endosc Percutan Tech. 2018;28(5):303-8. Epub 2018/06/12. doi: 10.1097/sle.0000000000000542. PubMed PMID: 29889110.
2758. Zhang X, Hou J, Duan G. Effects of transcranial direct current stimulation on psychomotor inhibition state in patients with traumatic brain injury. Chinese Journal of Rehabilitation Medicine.

2018;33(12):1405-9. PubMed PMID: CSCD:6395336.
2759. Zhang X, Zhu H, Tao W, Li Y, Hu Y. Motor Cortex Stimulation Therapy for Relief of Central Post-Stroke Pain: A Retrospective Study with Neuropathic Pain Symptom Inventory. Stereotactic and Functional Neurosurgery. 2018;96(4):239-43. doi: 10.1159/000492056. PubMed PMID: WOS:000446819900004.
2760. Zhang XY, Sui YF, Guo TC, Wang SH, Hu Y, Lu YS. Effect of Paired Associative Stimulation on Motor Cortex Excitability in Rats. Current Medical Science. 2018;38(5):903-9. doi: 10.1007/s11596-018-1960-8.
2761. Zhang Y, Song W. Transcranial direct current stimulation in disorders of consciousness: a review. Int J Neurosci. 2018;128(3):255-61. Epub 2017/09/19. doi: 10.1080/00207454.2017.1381094. PubMed PMID: 28918680.
2762. Zhang Y, Yang Y, Si J, Xia X, He J, Jiang T. Influence of inter-stimulus interval of spinal cord stimulation in patients with disorders of consciousness: A preliminary functional near-infrared spectroscopy study. Neuroimage Clin. 2018;17:1-9. Epub 2018/04/06. doi: 10.1016/j.nicl.2017.09.017. PubMed PMID: 29619317; PubMed Central PMCID: PMCPMC5883216.
2763. Zhao J. Disorders of Consciousness in China. Neuroscience Bulletin. 2018;34(4):605-14. doi: 10.1007/s12264-018-0263-1.

PubMed PMID: WOS:000440164500003.
2764. Zhao J, Ni Y, Lin H. A study on resting state fMRI of brain function network in patients with minimally conscious state by transcranial direct current stimulation. Chinese Journal of Rehabilitation Medicine. 2018;33(3):259-63. PubMed PMID: CSCD:6203322.
2765. Zhao J, Ni Y, Lin H, Liu H, Lu X, Dong J, et al. A comparative study of the effect of a single transcranial direct current stimulation on resting state of brain functional network in patients in a minimally conscious state. Chinese Journal of Physical Medicine and Rehabilitation. 2018;40(9):647-51. PubMed PMID: CSCD:6459794.
2766. Zhao X, Luo W, Hu J, Zuo L, Wang J, Hu R, et al. Cardiomyocyte-targeted and 17 beta-estradiol-loaded acoustic nanoprobes as a theranostic platform for cardiac hypertrophy. Journal of Nanobiotechnology. 2018;16. doi: 10.1186/s12951-018-0360-3. PubMed PMID: WOS:000428929500004.
2767. Zheng MX, Hua XY, Feng JT, Li T, Lu YC, Shen YD, et al. Trial of Contralateral Seventh Cervical Nerve Transfer for Spastic Arm Paralysis. N Engl J Med. 2018;378(1):22-34. Epub 2017/12/21. doi: 10.1056/NEJMoa1615208. PubMed PMID: 29262271.
2768. Zhou Y, Coughlin J, Du Y, Liu M, Leal J, Wong D, et al. A genetic polymorphism based kinetic analysis of [11C]DPA-713

dynamic PET for precision imaging of brain injury in National Football League players. Journal of Nuclear Medicine. 2018;59.
2769. CTMSS 2019 Annual Meeting. Brain stimulation. 2019;12(4):A1-A10. doi: 10.1016/s1935-861x(19)30251-7. PubMed PMID: CN-01976548.
2770. 2019 ACRM Annual Conference. Archives of Physical Medicine and Rehabilitation. 2019;100(10):A1-A22. doi: 10.1016/s0003-9993(19)31009-3.
2771. European Congress of Clinical Neurophysiology. Clinical Neurophysiology. 2019;130(7):e21-e116. doi: 10.1016/j.clinph.2019.04.317.
2772. 3rd International Brain Stimulation Conference. Brain Stimulation. 2019;12(2).
2773. 63rd National Congress of the Italian Society for Clinical Neurophysiology. Clinical Neurophysiology. 2019;130(1).
2774. Aanen IP, Elders BBLJ, Pullens B, Wijnen RMH. Tracheomalacia after esophageal repair: A systematic review. Diseases of the Esophagus. 2019;32:15. doi: 10.1093/dote/doz047.41.
2775. Abraham M, Poulopoulos N, Larson E. Clinical Utility of Transcranial Direct Current Stimulation (tDCS) Following Traumatic Brain Injury and Stroke. Archives of Physical Medicine and Rehabilitation. 2019;100(10):e149. doi: 10.1016/j.apmr.2019.08.454.

2776. Abubaker SS, Zhang Y. Optimization Design and Fabrication of Polymer Micro Needle by Hot Embossing Method. International Journal of Precision Engineering and Manufacturing. 2019;20(4):631-40. doi: 10.1007/s12541-019-00095-z. PubMed PMID: WOS:000464704300012.

2777. Actrn. Effects of transcranial direct current stimulation for improving attention after traumatic brain injury. https://trialsearchwhoint/Trial2aspx?TrialID=ACTRN1261900152319 0. 2019. PubMed PMID: CN-02064925.

2778. Actrn. A clinical research study investigating repetitive transcranial magnetic stimulation (rTMS) in treating persistent post-concussion symptoms in children compared to placebo/dummy treatment. https://trialsearchwhoint/Trial2aspx?TrialID=ACTRN1261900111819 0. 2019. PubMed PMID: CN-01975102.

2779. Adamson M, Siddiqi S, McNerney W, Swaminath G, Wu L, Wortman K, et al. Plasticity mechanisms underlying repetitive transcranial magnetic stimulation for improving cognition in brain injury. Neuromodulation. 2019;22(7):e417. doi: 10.1111/ner.12958.

2780. Ahmed M, Siddiqi S, Haque S, Chau J, Amin H. Safety and efficacy of repetitive Transcranial Magnetic Stimulation (rTMS) for depression in mild to moderate Traumatic Brain Injury (TBI): a

non-controlled study. Brain Stimulation. 2019;12(2):585. doi: 10.1016/j.brs.2018.12.943.

2781. Akyüz O, Bodakçi MN, Tefekli AH. Thermal cautery-assisted circumcision and principles of its use to decrease complication rates. Journal of Pediatric Urology. 2019;15(2):186.e1-.e8. doi: 10.1016/j.jpurol.2019.01.003.

2782. Al-Sultan F, Sultan Alsultan A. Safety of transcranial direct current stimulation during fasting in healthy adults. Journal of Clinical Neurophysiology. 2019;36(6):503. doi: 10.1097/WNP.0000000000000642.

2783. Allart E, Devanne H, Delval A. Contribution of transcranial magnetic stimulation in assessing parietofrontal connectivity during gesture production in healthy individuals and brain-injured patients. Neurophysiol Clin. 2019;49(2):115-23. Epub 2019/01/03. doi: 10.1016/j.neucli.2018.12.005. PubMed PMID: 30600138.

2784. Almousa A, Alajaji R, Alaboudi M, Al-sultan F, Bashir S. Safety of transcranial direct current stimulation across three brain regions in fasting. Brain Stimulation. 2019;12(2):402. doi: 10.1016/j.brs.2018.12.296.

2785. Ansado J, Blunt A, Chen JK, Koski L, Ptito A. Impact of non-invasive brain stimulation on transcallosal modulation in mild traumatic brain injury: a multimodal pilot investigation. Brain Inj.

2019;33(8):1021-31. Epub 2019/06/07. doi: 10.1080/02699052.2019.1605620. PubMed PMID: 31170014.

2786. Askalsky P, Iosifescu DV. Transcranial Photobiomodulation For The Management Of Depression: Current Perspectives. Neuropsychiatric Disease and Treatment. 2019;15:3255-72. doi: 10.2147/ndt.S188906. PubMed PMID: WOS:000500986900001.

2787. Awosika OO, Sandrini M, Volochayev R, Thompson RM, Fishman N, Wu T, et al. Transcutaneous spinal direct current stimulation improves locomotor learning in healthy humans. Brain Stimulation. 2019;12(3):628-34. doi: 10.1016/j.brs.2019.01.017. PubMed PMID: CN-01794166.

2788. Ay H. Post-extubation dysphagia (PED). Turk Beyin Damar Hastaliklar Dergisi. 2019;25:91-5.

2789. Badran B, Jenkins D, DeVries W, Dancy M, Cook D, Mappin G, et al. Development of Closed-loop Transcutaneous Auricular Vagus Nerve Stimulation (taVNS) as a Neurorehabilitation Tool. Brain Stimulation. 2019;12(2):523. doi: 10.1016/j.brs.2018.12.721.

2790. Bai Y, Xia X, Wang Y, He J, Li X. Assessment Effects of Repetitive Transcranial Magnetic Stimulation in Patients with Disorders of Consciousness by EEG. Chinese Journal of Biomedical Engineering. 2019;38(6):687-94. PubMed PMID: CSCD:6645150.

2791. Bakulin I, Poydasheva A, Lagoda D, Zabirova A, Evdokimov

K, Suponeva N, et al. P79-S Tolerability of repetitive transcranial magnetic stimulation: Analysis of 727 sessions with standardized questionnaires. Clinical Neurophysiology. 2019;130(7):e116. doi: 10.1016/j.clinph.2019.04.607.

2792. Barbero-Castillo A, Weinert JF, Camassa A, Perez-Mendez L, Caldas-Martinez S, Mattia M, et al. Proceedings #31: Cortical Network Complexity under Different Levels of Excitability Controlled by Electric Fields. Brain Stimulation. 2019;12(2):e97-e9. doi: 10.1016/j.brs.2018.12.200.

2793. Bareham CA, Allanson J, Roberts N, Hutchinson PJA, Pickard JD, Menon DK, et al. Longitudinal assessments highlight long-term behavioural recovery in disorders of consciousness. Brain communications. 2019;1(1):fcz017-fcz. doi: 10.1093/braincomms/fcz017. PubMed PMID: MEDLINE:31886461.

2794. Batista J, Porto H. Abstract #131: tDCS in Post Firearm Brain Injury - Case Report. Brain Stimulation. 2019;12(2):e45. doi: 10.1016/j.brs.2018.12.138.

2795. Beisegel J, Denkert C, Koehler E, Krnc M, Pivac N, Scheffler R, et al. On the End-Vertex Problem of Graph Searches. Discrete Mathematics and Theoretical Computer Science. 2019;21(1). PubMed PMID: WOS:000480435300008.

2796. Belkin VA, Pozdnyakov DG, Belkin AA. Diagnosis of the

phenomenon of cognitive-motor dissociation in patients with chronic consciousness disorders. Nevrologiya, Neiropsikhiatriya, Psikhosomatika. 2019;11:46-51. doi: 10.14412/2074-2711-2019-3S-46-51.

2797. Bensaid S, Modolo J, Merlet I, Wendling F, Benquet P. COALIA: A Computational Model of Human EEG for Consciousness Research. Front Syst Neurosci. 2019;13:59. Epub 2019/12/05. doi: 10.3389/fnsys.2019.00059. PubMed PMID: 31798421; PubMed Central PMCID: PMCPMC6863981.

2798. Berger A, Horst F, Müller S, Steinberg F, Doppelmayr M. Current state and future prospects of EEG and fNIRS in robot-assisted gait rehabilitation: A brief review. Frontiers in Human Neuroscience. 2019;13. doi: 10.3389/fnhum.2019.00172.

2799. Berger P. A review of physical modalities and the potential to expand the treatment of patients with traumatic brain injury. Acupunct Med. 2019;37(6):365-9. Epub 2019/04/30. doi: 10.1177/0964528419844264. PubMed PMID: 31032621.

2800. Berger P. A rehabilitation approach to a patient with traumatic brain injury. Acupunct Med. 2019;37(6):370-1. Epub 2019/11/19. doi: 10.1177/0964528419884947. PubMed PMID: 31735056.

2801. Bernier RA, Hillary FG. Traumatic brain injury and frontal lobe plasticity. Handbook of clinical neurology. 2019;163:411-31. doi:

10.1016/b978-0-12-804281-6.00022-7. PubMed PMID: MEDLINE:31590744.
2802. Bodnar C, Bachstetter A. Understanding interleukin-1 receptor 1 localization after traumatic brain injury. Journal of Neurochemistry. 2019;150:170-. PubMed PMID: WOS:000478639800423.
2803. Bourdillon P, Hermann B, Sitt JD, Naccache L. Electromagnetic Brain Stimulation in Patients With Disorders of Consciousness. Front Neurosci. 2019;13:223. Epub 2019/04/03. doi: 10.3389/fnins.2019.00223. PubMed PMID: 30936822; PubMed Central PMCID: PMCPMC6432925.
2804. Boyd R, Pagnozzi A, Fripp J, Rose S. Understanding the impact of bilateral brain injury in children with cerebral palsy. Developmental Medicine and Child Neurology. 2019;61:54. doi: 10.1111/dmcn.14353.
2805. Brenner M, Messing A, Olsen ML. AP-1 and the injury response of the GFAP gene. Journal of Neuroscience Research. 2019;97(2):149-61. doi: 10.1002/jnr.24338.
2806. Burchiel KJ, Raslan AM. Contemporary concepts of pain surgery. J Neurosurg. 2019;130(4):1039-49. Epub 2019/04/02. doi: 10.3171/2019.1.Jns181620. PubMed PMID: 30933905.
2807. Burroughs L, Mitroi J, Bolbecker A, Moussa-Tooks A, Lundin N, O'Donnell B, et al. Transcranial direct current stimulation of the

human cerebellum during associative learning. Brain Stimulation. 2019;12(2):478. doi: 10.1016/j.brs.2018.12.558.
2808. Cacciola A, Naro A, Milardi D, Bramanti A, Malatacca L, Spitaleri M, et al. Functional brain network topology discriminates between patients with minimally conscious state and unresponsivewakefulness syndrome. Journal of Clinical Medicine. 2019;8(3). doi: 10.3390/jcm8030306.
2809. Caglayan AB, Beker MC, Caglayan B, Yalcin E, Caglayan A, Yulug B, et al. Acute and post-acute neuromodulation induces stroke recovery by promoting survival signaling, neurogenesis, and pyramidal tract plasticity. Frontiers in Cellular Neuroscience. 2019;13. doi: 10.3389/fncel.2019.00144.
2810. Cai T, Xia X, Zhang H, Guo Y, Bai Y. High-definition transcranial direct current stimulation modulates neural activities in patients with prolonged disorders of consciousness. Brain Stimul. 2019;12(6):1619-21. Epub 2019/09/15. doi: 10.1016/j.brs.2019.08.017. PubMed PMID: 31519492.
2811. Cain J, Monti M. Altered cortical blood flow during sonication of high-order thalamus using low intensity focused ultrasound pulsation. Brain Stimulation. 2019;12(2):480-1. doi: 10.1016/j.brs.2018.12.568.
2812. Capone F, Miccininilli S, Pellegrino G, Zollo L, Guglielmelli

E, Sterzi S, et al. Combining Transcutaneous Vagus Nerve Stimulation and Upper-Limb Robotic Rehabilitation in Chronic Stroke Patients. Brain Stimulation. 2019;12(2):413. doi: 10.1016/j.brs.2018.12.334.

2813. Carey L, Nilsson M, Boyd L. Learning following Brain Injury: Neural Plasticity Markers. Neural Plasticity. 2019;2019. doi: 10.1155/2019/4838159. PubMed PMID: WOS:000486427000001.

2814. Cary JH, Li BS, Maibach HI. Dermatotoxicology of microneedles (MNs) in man. Biomedical Microdevices. 2019;21(3). doi: 10.1007/s10544-019-0371-3. PubMed PMID: WOS:000474371300001.

2815. Castagna A, Di Tella S, Blasi V, Cabinio M, Baglio F. Integrity of Mirror Neuron System in cervical dystonia: An fMRI study. Movement Disorder. 2019;34:S775.

2816. Cavinato M, Genna C, Formaggio E, Gregorio C, Storti SF, Manganotti P, et al. Behavioural and electrophysiological effects of tDCS to prefrontal cortex in patients with disorders of consciousness. Clin Neurophysiol. 2019;130(2):231-8. Epub 2018/12/24. doi: 10.1016/j.clinph.2018.10.018. PubMed PMID: 30580246.

2817. Chai Z, Ma C, Jin X. Cortical stimulation for treatment of neurological disorders of hyperexcitability: A role of homeostatic plasticity. Neural Regeneration Research. 2019;14(1):34-8. doi: 10.4103/1673-5374.243696.

2818. Chattopadhyay A, Uikey MS, Verma R, Kumar N. Intermittent theta burst stimulation (iTBS) for managing treatment resistant depression. Indian Journal of Psychiatry. 2019;61(9):S624.

2819. Chemali Z, Nehme R, Fricchione G. Sensory neurologic disorders: Tinnitus. Handbook of clinical neurology. 2019;165:365-81. doi: 10.1016/b978-0-444-64012-3.00022-8. PubMed PMID: MEDLINE:31727224.

2820. Choi YA, Shin HI. Effects of anodal transcutaneous spinal direct current stimulation on chronic neuropathic pain after spinal cord injury: a pilot study. Neuromodulation. 2019;22(7):e492-. doi: 10.1111/ner.12958. PubMed PMID: CN-02164317.

2821. Chrysikou EG, Gorey C, Aupperle RL. Proceedings #19: effects of Transcranial Direct Current Stimulation over Prefrontal Cortex for Decision Making During Approach Avoidance Conflict. Brain stimulation. 2019;12(2):e81-e3. doi: 10.1016/j.brs.2018.12.188. PubMed PMID: CN-01788703.

2822. Coben R, Armes C. Meta-analysis of mulitvariate coherence neurofeedback research. NeuroRegulation. 2019;6(4):214-5. doi: 10.15540/nr.6.4.197.

2823. Cole G, Ali AA, McErlean E, Mulholland EJ, Short A, McCrudden CM, et al. DNA vaccination via RALA nanoparticles in a microneedle delivery system induces a potent immune response

against the endogenous prostate cancer stem cell antigen. Acta Biomaterialia. 2019;96:480-90. doi: 10.1016/j.actbio.2019.07.003. PubMed PMID: WOS:000486132900038.

2824. Colombo MA, Napolitani M, Boly M, Gosseries O, Casarotto S, Rosanova M, et al. The spectral exponent of the resting EEG indexes the presence of consciousness during unresponsiveness induced by propofol, xenon, and ketamine. Neuroimage. 2019;189:631-44. Epub 2019/01/15. doi: 10.1016/j.neuroimage.2019.01.024. PubMed PMID: 30639334.

2825. Comolatti R, Pigorini A, Casarotto S, Fecchio M, Faria G, Sarasso S, et al. A fast and general method to empirically estimate the complexity of brain responses to transcranial and intracranial stimulations. Brain Stimulation. 2019;12(5):1280-9. doi: 10.1016/j.brs.2019.05.013.

2826. Cunningham D, Rickert A, Plow E, Knutson J. Chronic stroke hemispheric dominance and task specific fmri laterality of cortical motor activation. Neurorehabilitation and Neural Repair. 2019;33(12):1082. doi: 10.1177/1545968319893871.

2827. Cutsuridis V. Memory prosthesis: Is it time for a deep neuromimetic computing approach? Frontiers in Neuroscience. 2019;13(JUL). doi: 10.3389/fnins.2019.00667.

2828. da Costa Ferreira Pinto PH, Nigri F, Caparelli-Dáquer EM,

dos Santos Viana J. Computed tomography-guided navigated transcranial magnetic stimulation for preoperative brain motor mapping in brain lesion resection: A case report. Surgical Neurology International. 2019;10. doi: 10.25259/SNI-124-2019.

2829. Das N, Spence JS, Aslan S, Vanneste S, Mudar R, Rackley A, et al. Cognitive Training and Transcranial Direct Current Stimulation in Mild Cognitive Impairment: A Randomized Pilot Trial. Frontiers in Neuroscience. 2019;13. doi: 10.3389/fnins.2019.00307. PubMed PMID: WOS:000464451200001.

2830. Dave A, Ganesh A, Adil MM, Tsao JW. Practice Current: How do you diagnose and treat post-concussive headache? Neurology Clinical practice. 2019;9(3):263-70. doi: 10.1212/cpj.0000000000000656. PubMed PMID: MEDLINE:31341715.

2831. De Launay KQ, Cheung ST, Riggs L, Reed N, Beal DS. Abstract #63: the Effect of tDCS on Cognitive Performance in Youth with Concussion: a Pilot Study. Brain stimulation. 2019;12(2):e22-e3. doi: 10.1016/j.brs.2018.12.070. PubMed PMID: CN-01791663.

2832. Debruyne P, Rossenbacker T, Collienne C, Janssens L, Roosen J, Vankelecom B, et al. Unifocal right-sided ablation for neurally mediated syncope and functional sinus node dysfunction. Acta Cardiologica. 2019;74:3-4. doi: 10.1080/00015385.2018.1558767.

2833. Di Virgilio TG, Ietswaart M, Wilson L, Donaldson DI, Hunter AM. Understanding the Consequences of Repetitive Subconcussive Head Impacts in Sport: Brain Changes and Dampened Motor Control Are Seen After Boxing Practice. Front Hum Neurosci. 2019;13:294. Epub 2019/09/26. doi: 10.3389/fnhum.2019.00294. PubMed PMID: 31551732; PubMed Central PMCID: PMCPMC6746992.
2834. Dixon B, Turner R, Christou C. Assessment of a Non-Invasive Brain Oximeter in a Sheep Model of Acute Brain Injury. Med Devices (Auckl). 2019;12:479-87. Epub 2019/12/12. doi: 10.2147/mder.S235804. PubMed PMID: 31824197; PubMed Central PMCID: PMCPMC6900466.
2835. Dobney DM, Miller MB, Tufts E. Non-pharmacological rehabilitation interventions for concussion in children: a scoping review. Disabil Rehabil. 2019;41(6):727-39. Epub 2017/11/22. doi: 10.1080/09638288.2017.1400595. PubMed PMID: 29157025.
2836. Dodeja AK, Thomas C, Daniels CJ, Kertesz N, Kamp A. Detection of arrhythmias in adult congenital heart disease patients with LINQTM implantable loop recorder. Congenital Heart Disease. 2019;14(5):745-51. doi: 10.1111/chd.12815.
2837. Duarte N, Grecco L, Lopes J, Galli M, Oliveira C. The effect of transcranial direct current stimulation on mobility in a child with cerebral palsy: case report. Brain Stimulation. 2019;12(2):429. doi:

10.1016/j.brs.2018.12.390.

2838. Dunn MS, Beck B, Simpson PM, Cameron PA, Kennedy M, Maiden M, et al. Comparing the outcomes of isolated, serious traumatic brain injury in older adults managed at major trauma centres and neurosurgical services: A registry-based cohort study. Injury. 2019;50(9):1534-9. Epub 2019/06/18. doi: 10.1016/j.injury.2019.06.012. PubMed PMID: 31204027.

2839. Ebbing J, van de Lindt D. Right Sided (RDLPFC) Low Frequency (1Hz) rTMS in the Third Trimester of Pregnancy; A Case Report. Brain Stimulation. 2019;12(4):e134-e5. doi: 10.1016/j.brs.2019.03.041.

2840. Elbanna ST, Elshennawy S, Ayad MN. Noninvasive Brain Stimulation for Rehabilitation of Pediatric Motor Disorders Following Brain Injury: Systematic Review of Randomized Controlled Trials. Arch Phys Med Rehabil. 2019;100(10):1945-63. Epub 2019/05/13. doi: 10.1016/j.apmr.2019.04.009. PubMed PMID: 31078616.

2841. Etkin A, Maron-Katz A, Wu W, Fonzo GA, Huemer J, Vértes PE, et al. Using fMRI connectivity to define a treatment-resistant form of post-traumatic stress disorder. Science Translational Medicine. 2019;11(486). doi: 10.1126/scitranslmed.aal3236.

2842. Faye A, Jacquin-Courtois S, Reynaud E, Lesourd M, Besnard J, Osiurak F. Numerical cognition: A meta-analysis of neuroimaging,

transcranial magnetic stimulation and brain-damaged patients studies. Neuroimage Clin. 2019;24:102053. Epub 2019/12/05. doi: 10.1016/j.nicl.2019.102053. PubMed PMID: 31795045; PubMed Central PMCID: PMCPMC6978218.

2843. Fecchio M, Rosanova M, Casarotto S, Sarasso S, Casali AG, Pigorini A, et al. Sleep-like bistability, loss of causality and complexity in the cerebral cortex of unresponsive wakefulness syndrome patients. Brain Stimulation. 2019;12(2):432. doi: 10.1016/j.brs.2018.12.401.

2844. Feldmann LK, Le Prieult F, Felzen V, Thal SC, Engelhard K, Behl C, et al. Proteasome and Autophagy-Mediated Impairment of Late Long-Term Potentiation (l-LTP) after Traumatic Brain Injury in the Somatosensory Cortex of Mice. Int J Mol Sci. 2019;20(12). Epub 2019/06/27. doi: 10.3390/ijms20123048. PubMed PMID: 31234472; PubMed Central PMCID: PMCPMC6627835.

2845. Fernandes SR, Pereira M, Salvador R, Miranda PC, De Carvalho M. Cervical trans-spinal direct current stimulation: A modelling-experimental approach. Journal of NeuroEngineering and Rehabilitation. 2019;16(1). doi: 10.1186/s12984-019-0589-6.

2846. Ferre C, Karen C, Gordon A, Friel K. Bilateral connectivity in children with unilateral brain injury: Implications for upper-extremity function. Developmental Medicine and Child Neurology. 2019;61:128.

doi: 10.1111/dmcn.14354.
2847. Filla MS, Faralli JA, Desikan H, Peotter JL, Wannow AC, Peters DM. Activation of αvβ3 integrin alters fibronectin fibril formation in human trabecular meshwork cells in a ROCK-independent manner. Investigative Ophthalmology and Visual Science. 2019;60(12):3897-913. doi: 10.1167/iovs.19-27171.
2848. Fonseca DFS, Vilela C, Silvestre AJD, Freire CSR. A compendium of current developments on polysaccharide and protein-based microneedles. International Journal of Biological Macromolecules. 2019;136:704-28. doi: 10.1016/j.ijbiomac.2019.04.163. PubMed PMID: WOS:000482533000073.
2849. Fridman EA, Osborne JR, Mozley PD, Victor JD, Schiff ND. Presynaptic dopamine deficit in minimally conscious state patients following traumatic brain injury. Brain. 2019;142(7):1887-93. Epub 2019/09/11. doi: 10.1093/brain/awz118. PubMed PMID: 31505542; PubMed Central PMCID: PMCPMC6598636.
2850. Gao S, Proekt A, Renier N, Calderon D, Pfaff D. Gigantocellular Neurons Awaken the Brain from Deep Pharmacologically-Induced Coma. Brain Stimulation. 2019;12(2):428. doi: 10.1016/j.brs.2018.12.386.
2851. Gaware SA, Rokade KA, Bala P, Kale SN. Microneedles of

chitosan-porous carbon nanocomposites: Stimuli (pH and electric field)-initiated drug delivery and toxicological studies. Journal of Biomedical Materials Research Part A. 2019;107(8):1582-96. doi: 10.1002/jbm.a.36672. PubMed PMID: WOS:000471813900002.

2852. Ghonimi NAM, Mahdy ME, Abdel Salam OA. Total Antioxidant Capacity Predicts Outcome in Acute Ischemic Stroke Subtypes in Egyptian Patients. Journal of Stroke and Cerebrovascular Diseases. 2019;28(7):1911-7. doi: 10.1016/j.jstrokecerebrovasdis.2019.03.053.

2853. Giuffre A, Zewdie E, Kahl C, Kirton A. Robotic Transcranial Magnetic Stimulation (TMS) Motor Mapping in Children. Brain Stimulation. 2019;12(2):549. doi: 10.1016/j.brs.2018.12.813.

2854. Glize B, Bigourdan A, Villain M, Munsch F, Tourdias T, de Gabory I, et al. Motor evoked potential of upper-limbs is predictive of aphasia recovery. Aphasiology. 2019;33(1):105-20. doi: 10.1080/02687038.2018.1444137.

2855. Goldman JS, Tort-Colet N, di Volo M, Susin E, Bouté J, Dali M, et al. Bridging Single Neuron Dynamics to Global Brain States. Frontiers in Systems Neuroscience. 2019;13. doi: 10.3389/fnsys.2019.00075.

2856. Guo F, Ouyang T, Peng T, Zhang X, Xie B, Yang X, et al. Enhanced oral absorption of insulin using colon-specific nanoparticles

co-modified with amphiphilic chitosan derivatives and cell-penetrating peptides. Biomaterials Science. 2019;7(4):1493-506. doi: 10.1039/c8bm01485j. PubMed PMID: WOS:000463746600021.

2857. Guo S, Yin Z, Lu G, Huang X. A comparative study of repetitive transcranial magnetic stimulation at different frequencies in arousal for coma patients after brain stem injury. Chinese Journal of Neuromedicine. 2019;18(6):550-4. PubMed PMID: CSCD:6521188.

2858. Guo Y, Bai Y, Xia X, Li J, Wang X, Dai Y, et al. Effects of Long-Lasting High-Definition Transcranial Direct Current Stimulation in Chronic Disorders of Consciousness: A Pilot Study. Front Neurosci. 2019;13:412. Epub 2019/05/23. doi: 10.3389/fnins.2019.00412. PubMed PMID: 31114475; PubMed Central PMCID: PMCPMC6502996.

2859. Gupte R, Christian S, Keselman P, Habiger J, Brooks WM, Harris JL. Evaluation of taurine neuroprotection in aged rats with traumatic brain injury. Brain Imaging and Behavior. 2019;13(2):461-71. doi: 10.1007/s11682-018-9865-5.

2860. Hagelskjær V, Krohn M, Christensen PS, Christensen JR. Canadian occupational performance measure supported by talking mats: An evaluation of the clinical utility. Occupational Therapy International. 2019;2019. doi: 10.1155/2019/9367315.

2861. Hall BR, Armijo PR, Leinicke JA, Langenfeld SJ, Oleynikov

D. Prolonged non-operative management of clostridium difficile colitis is associated with increased mortality, complications, and cost. American Journal of Surgery. 2019;217(6):1042-6. doi: 10.1016/j.amjsurg.2019.01.017.

2862. Hameed MQ, Hsieh TH, Morales-Quezada L, Lee HHC, Damar U, MacMullin PC, et al. Ceftriaxone Treatment Preserves Cortical Inhibitory Interneuron Function via Transient Salvage of GLT-1 in a Rat Traumatic Brain Injury Model. Cereb Cortex. 2019;29(11):4506-18. Epub 2018/12/28. doi: 10.1093/cercor/bhy328. PubMed PMID: 30590449; PubMed Central PMCID: PMCPMC7150617.

2863. Hannah R, Iacovou A, Rothwell JC. Direction of TDCS current flow in human sensorimotor cortex influences behavioural learning. Brain Stimulation. 2019;12(3):684-92. doi: 10.1016/j.brs.2019.01.016.

2864. Hansson JHS. A hypothesis regarding how sleep can calibrate neuronal excitability in the central nervous system and thereby offer stability, sensitivity and the best possible cognitive function. Medical Hypotheses. 2019;131. doi: 10.1016/j.mehy.2019.109307.

2865. Hara T, Burhan A, Abo M, Watanabe S, Akimoto H, Fukui R. Improvement of higher brain dysfunction after brain injurywith strategic application of repetitive transcranial magnetic stimulation

and intensive rehabilitation therapy: a report of three cases. Brain Stimulation. 2019;12(2):391. doi: 10.1016/j.brs.2018.12.255.
2866. Hayashi C, Neville IS, Rodrigues P, Galhardoni RR, de Andrade DC, Zaninotto AL, et al. Abstract #77: repetitive Transcranial Magnetic Stimulation (rTMS) in chronic diffuse axonal injury: a randomized controlled trial. Brain stimulation. 2019;12(2):e27-. doi: 10.1016/j.brs.2018.12.084. PubMed PMID: CN-01787181.
2867. He DP, Zhang J, Bai ZF. Percutaneous Electrical Nerve Stimulation for Chronic Knee Pain: A Randomized, Sham-controlled Trial. Altern Ther Health Med. 2019;25(2):30-4. Epub 2017/11/05. PubMed PMID: 29101777.
2868. He R, Zheng Be, Wu H. The local change of EEG in the severe brain injury patients after rTMS intervention. Chinese Journal of Rehabilitation Medicine. 2019;34(12):1418-22. PubMed PMID: CSCD:6629230.
2869. Henriques IAD, Lattari E, Torres G, Rodrigues GM, Oliveira BRR, Neto GAM, et al. Can transcranial direct current stimulation improve range of motion and modulate pain perception in healthy individuals? Neuroscience Letters. 2019;707. doi: 10.1016/j.neulet.2019.134311.
2870. Herrold A, Jordan N, Parrish T, Cobia D, Chambers RA, Sander A, et al. Multi-Modal Neuroimaging Characterization of

Co-Occurring Mild Traumatic Brain Injury and Alcohol Use Disorder. Biological Psychiatry. 2019;85(10):S293-S4. doi: 10.1016/j.biopsych.2019.03.744. PubMed PMID: WOS:000472661000719.

2871. Herrold AA, Jordan N, Parrish T, Cobia D, Wang X, Chambers RA, et al. USING MULTI-MODAL NEUROIMAGING TO INFORM NEUROMODULATORY TREATMENT FOR CO-OCCURRING ALCOHOL USE DISORDER AND MILD TRAUMATIC BRAIN INJURY. Alcoholism-Clinical and Experimental Research. 2019;43:308A-A. PubMed PMID: WOS:000468963104199.

2872. Hofer AS, Schwab ME. Enhancing rehabilitation and functional recovery after brain and spinal cord trauma with electrical neuromodulation. Curr Opin Neurol. 2019;32(6):828-35. Epub 2019/10/01. doi: 10.1097/wco.0000000000000750. PubMed PMID: 31567546; PubMed Central PMCID: PMCPMC6855343.

2873. Horton G, Ibrahim O, Jansen M, Milev R, Beyea J, Trier J. Repetitive Trans-Cranial Magnetic Stimulation (rTMS) for the Treatment of Chronic Tinnitus in Patients with and without A History of Traumatic Brain Injury (TBI). Brain Injury. 2019;33:58-9. PubMed PMID: WOS:000466897000131.

2874. Hosseini M, McConathey EM, Ungrady M, Grossman M,

Coslett HB, Hamilton RH. Proceedings #10: transcranial Direct Current Stimulation Mediates Improvements in Verbal Fluency for Patients with Primary Progressive Aphasia. Brain stimulation. 2019;12(2):e69-e71. doi: 10.1016/j.brs.2018.12.179. PubMed PMID: CN-01793220.

2875. Houghton DC, Uhde TW, Borckardt JJ, Cortese BM. Exploratory Investigation of a Brief Cognitive Behavioral Intervention and Transcranial Direct Current Stimulation on Odor Sensitivity. Psychosom Med. 2019;81(4):389-95. Epub 2019/02/15. doi: 10.1097/psy.0000000000000679. PubMed PMID: 30762663; PubMed Central PMCID: PMCPMC6961818.

2876. Hoy KE, McQueen S, Elliot D, Herring SE, Maller JJ, Fitzgerald PB. A Pilot Investigation of Repetitive Transcranial Magnetic Stimulation for Post-Traumatic Brain Injury Depression: Safety, Tolerability, and Efficacy. J Neurotrauma. 2019;36(13):2092-8. Epub 2019/02/05. doi: 10.1089/neu.2018.6097. PubMed PMID: 30712461.

2877. Hu F, Zheng L, Liang E, Ding L, Wu L, Chen G, et al. Right anterior ganglionated plexus: The primary target of cardioneuroablation? Heart Rhythm. 2019;16(10):1545-51. doi: 10.1016/j.hrthm.2019.07.018. PubMed PMID: WOS:000488200900015.

2878. Huang X, Luo Y, Liu Z, Zhang C, Zhong H, Xue J, et al. Influence of Two-Stage Combinations of Constructed Wetlands on the Removal of Antibiotics, Antibiotic Resistance Genes and Nutrients from Goose Wastewater. Int J Environ Res Public Health. 2019;16(20). Epub 2019/10/24. doi: 10.3390/ijerph16204030. PubMed PMID: 31640268; PubMed Central PMCID: PMCPMC6843979.

2879. Huang Y, Thomas C, Datta A, Parra LC. Proceedings #23: Inaccurate segmentation of lesioned brains can significantly affect targeted transcranial electrical stimulation on stroke patients. Brain Stimulation. 2019;12(2):e87-e9. doi: 10.1016/j.brs.2018.12.192.

2880. Huo C, Li X, Jing J, Ma Y, Li W, Wang Y, et al. Median Nerve Electrical Stimulation–Induced Changes in Effective Connectivity in Patients With Stroke as Assessed With Functional Near-Infrared Spectroscopy. Neurorehabilitation and Neural Repair. 2019;33(12):1008-17. doi: 10.1177/1545968319875952.

2881. Hurme M, Koivisto M, Revonsuo A, Railo H. V1 activity during feedforward and early feedback processing is necessary for both conscious and unconscious motion perception. Neuroimage. 2019;185:313-21. Epub 2018/10/27. doi: 10.1016/j.neuroimage.2018.10.058. PubMed PMID: 30366074.

2882. Iovino L, Cooper K, Kinsella S, deRoos P, Jain R, Dudakov JA. Zinc Improves Thymic Regeneration after Allogeneic HSCT By

Stimulating BMP4 Production from Endothelial Cells. Biology of Blood and Marrow Transplantation. 2019;25(3):S333. doi: 10.1016/j.bbmt.2018.12.537.

2883. Iovino L, Kinsella S, Cooper K, Jain R, DeRoos P, Gagnon A, et al. Zinc Treatment Stimulates Thymic Regeneration after Bone Marrow Transplant. Blood. 2019;134. doi: 10.1182/blood-2019-129290. PubMed PMID: WOS:000577164603154.

2884. Irct20130416013027N. The survey of transcranial direct current stimulation efficacy on the post-traumatic sleep quality, insomnia severity in the patients with mild traumatic brain injury during sub-acute phase. https://trialsearchwhoint/Trial2aspx?TrialID=IRCT20130416013027N2. 2019. PubMed PMID: CN-02068957.

2885. Irct20171215037872N. Efficacy of Repetitive Transcranial Magnetic Stimulation to Reduce Muscle Spasticity. https://trialsearchwhoint/Trial2aspx?TrialID=IRCT20171215037872N1. 2019. PubMed PMID: CN-01972328.

2886. Isrctn. Can treatment with Cerebrolysin and magnetic stimulation of the brain improve recovery after traumatic brain injury? https://trialsearchwhoint/Trial2aspx?TrialID=ISRCTN10335247. 2019. PubMed PMID: CN-01947397.

2887. Jang SH, Kwon YH. Neuroimaging characterization of recovery of impaired consciousness in patients with disorders of consciousness. Neural Regen Res. 2019;14(7):1202-7. Epub 2019/02/26. doi: 10.4103/1673-5374.251299. PubMed PMID: 30804246; PubMed Central PMCID: PMCPMC6425825.
2888. Jang SH, Seo YS. Diagnosis of Conversion Disorder Using Diffusion Tensor Tractography and Transcranial Magnetic Stimulation in a Patient with Mild Traumatic Brain Injury. Diagnostics (Basel). 2019;9(4). Epub 2019/10/28. doi: 10.3390/diagnostics9040155. PubMed PMID: 31652549; PubMed Central PMCID: PMCPMC6963761.
2889. Jha KA, Pentecost M, Lenin R, Gentry J, Klaic L, Del Mar N, et al. TSG-6 in conditioned media from adipose mesenchymal stem cells protects against visual deficits in mild traumatic brain injury model through neurovascular modulation. Stem Cell Research and Therapy. 2019;10(1). doi: 10.1186/s13287-019-1436-1.
2890. Js G, E J, Ja T, Kh B, Kc O, Gover-Chamlou A, et al. Evolution of a Study of Bilateral Prefrontal Transcranial Magnetic Stimulation (TMS) to Treat the Symptoms of Mild TBI (mTBI) and PTSD: research Methods, Participant Demographics, and Tolerability. Brain stimulation. 2019;12(4):e135-. doi: 10.1016/j.brs.2019.03.042. PubMed PMID: CN-01962710.

2891. Kalita J, Kumar S, Misra UK. Effect of rTMS and amitriptyline versus rTMS alone in chronic migraine: A randomized controlled trial. European Journal of Neurology. 2019;26:218. doi: 10.1111/ene.14018.
2892. Kalita J, Misra UK, Dubey AK. Role of Oxidative Stress in Tuberculous Meningitis: a Clinico-Radiological Correlation. Journal of Molecular Neuroscience. 2019;68(2):287-94. doi: 10.1007/s12031-019-01304-0.
2893. Kaltiainen H, Liljeström M, Helle L, Salo A, Hietanen M, Renvall H, et al. Mild traumatic brain injury affects cognitive processing and modifies oscillatory brain activity during attentional tasks. Journal of Neurotrauma. 2019;36(14):2222-32. doi: 10.1089/neu.2018.6306.
2894. Karamyan VT. Peptidase neurolysin is an endogenous cerebroprotective mechanism in acute neurodegenerative disorders. Medical Hypotheses. 2019;131. doi: 10.1016/j.mehy.2019.109309.
2895. Kataria P, Padmanabhan P, Gopalakrishnan A, Padmanaban V, Mahadik S, Ambrósio R, Jr. Accuracy of Scheimpflug-derived corneal biomechanical and tomographic indices for detecting subclinical and mild keratectasia in a South Asian population. J Cataract Refract Surg. 2019;45(3):328-36. Epub 2018/12/12. doi: 10.1016/j.jcrs.2018.10.030. PubMed PMID: 30527442.

2896. Kaur J, Mania I. Deep transcranial magnetic stimulation (dTMS) for treatment of major depressive disorder (MDD) status post-surgical removal of medulloblastoma: A case report of safety. Brain Stimulation. 2019;12(4):1061-2. doi: 10.1016/j.brs.2019.03.010.
2897. Kawamura M, Takahashi N, Kobayashi Y. Effect of Repetitive Transcranial Magnetic Stimulation on the Right Superior Temporal Gyrus for Severe Aphasia Caused by Damage to the Left Inferior Frontal Gyrus. Case Reports in Neurology. 2019;11(2):189-98. doi: 10.1159/000500669.
2898. Kaye HL, Block G, Zhang H, Sollmann N, Krieg S, Rotenberg A. Attraction of the motor map toward a seizure focus. Journal of Clinical Neurophysiology. 2019;36(6):485. doi: 10.1097/WNP.0000000000000642.
2899. Keller-Ross ML, Chantigian DP, Rich TL, Chen M, Chen C-Y, Gillick BT. Stability of the cardiovascular response during single-pulse TMS in perinatal stroke. Brain Stimulation. 2019;12(2):371-3. doi: 10.1016/j.brs.2018.11.010. PubMed PMID: WOS:000456437000023.
2900. Khammash D, Simmonite M, Polk TA, Taylor SF, Meehan SK. Probing short-latency cortical inhibition in the visual cortex with transcranial magnetic stimulation: A reliability study. Brain Stimulation. 2019;12(3):702-4. doi: 10.1016/j.brs.2019.01.013.

2901. Khan F, Amatya B, Bensmail D, Yelnik A. Non-pharmacological interventions for spasticity in adults: An overview of systematic reviews. Ann Phys Rehabil Med. 2019;62(4):265-73. Epub 2017/10/19. doi: 10.1016/j.rehab.2017.10.001. PubMed PMID: 29042299.
2902. Khan IS, D'Agostino EN, Calnan DR, Lee JE, Aronson JP. Deep Brain Stimulation for Memory Modulation: A New Frontier. World Neurosurg. 2019;126:638-46. Epub 2019/01/18. doi: 10.1016/j.wneu.2018.12.184. PubMed PMID: 30654156.
2903. Khan OH. Incorporating Navigated Transcranial Magnetic Stimulation (nTMS) into the neurosurgical practice: Oncological, vascular and research potentials. Canadian Journal of Neurological Sciences. 2019;46:S38-S9. doi: 10.1017/cjn.2019.189.
2904. Kim SK, Lee S, Lee MK, Lee S. A systems pharmacology approach to investigate the mechanism of Oryeong-san formula for the treatment of hypertension. Journal of Ethnopharmacology. 2019;244. doi: 10.1016/j.jep.2019.112129.
2905. Kim WS, Lee K, Kim S, Cho S, Paik NJ. Transcranial direct current stimulation for the treatment of motor impairment following traumatic brain injury. J Neuroeng Rehabil. 2019;16(1):14. Epub 2019/01/27. doi: 10.1186/s12984-019-0489-9. PubMed PMID: 30683136; PubMed Central PMCID: PMCPMC6347832.

2906. Kim Y, Bhattaccharjee SA, Beck-Broichsitter M, Banga AK. Fabrication and characterization of hyaluronic acid microneedles to enhance delivery of magnesium ascorbyl phosphate into skin. Biomedical Microdevices. 2019;21(4). doi: 10.1007/s10544-019-0455-0. PubMed PMID: WOS:000499456800002.

2907. King R, Kirton A, Zewdie E, Seeger TA, Ciechanski P, Barlow KM. Longitudinal Assessment of Cortical Excitability in Children and Adolescents With Mild Traumatic Brain Injury and Persistent Post-concussive Symptoms. Front Neurol. 2019;10:451. Epub 2019/06/04. doi: 10.3389/fneur.2019.00451. PubMed PMID: 31156530; PubMed Central PMCID: PMCPMC6533942.

2908. Knoll RM, Ishai R, Trakimas DR, Chen JX, Nadol JB, Jr., Rauch SD, et al. Peripheral Vestibular System Histopathologic Changes following Head Injury without Temporal Bone Fracture. Otolaryngol Head Neck Surg. 2019;160(1):122-30. Epub 2018/10/03. doi: 10.1177/0194599818795695. PubMed PMID: 30274548.

2909. Knoll RM, Reinshagen KL, Barber SR, Ghanad I, Swanson R, Smith DH, et al. High Resolution Computed Tomography Atlas of the Porcine Temporal Bone and Skull Base: Anatomical Correlates for Traumatic Brain Injury Research. J Neurotrauma. 2019;36(7):1029-39. Epub 2018/07/05. doi: 10.1089/neu.2018.5808. PubMed PMID:

29969939; PubMed Central PMCID: PMCPMC8349728.
2910. Koh S, Ambrósio R, Jr., Inoue R, Maeda N, Miki A, Nishida K. Detection of subclinical corneal ectasia using corneal tomographic and biomechanical assessments in a Japanese population. Journal of Refractive Surgery. 2019;35(6):383-90. doi: 10.3928/1081597X-20190417-01.
2911. Kopachka M, Sharova E, Alexandrova E, Troshina E, Zaitsev O, Chelyapina M, et al. P76-S Therapeutic possibilities of transcranial magnetic stimulation in patients after traumatic brain injury (updated report). Clinical Neurophysiology. 2019;130(7):e115. doi: 10.1016/j.clinph.2019.04.604.
2912. Kopachka MM, Sharova EV, Aleksandrova EV, Troshina EM, Zaytsev OS, Kravchuk AD, et al. [In search of an effective algorithm for rhythmic transcranial magnetic stimulation in neurorehabilitation after severe traumatic brain injury]. Zh Vopr Neirokhir Im N N Burdenko. 2019;83(6):111-9. Epub 2020/02/08. doi: 10.17116/neiro201983061111. PubMed PMID: 32031174.
2913. Kowalski J, Nemanich S, Nawshin T, Sutter E, Chen M, Peyton C, et al. Corticospinal connectivity patterns and motor outcome in infants with early brain injury. Developmental Medicine and Child Neurology. 2019;61:18-9. doi: 10.1111/dmcn.14244.
2914. Krieger D, Soose R, Shepard P, Puccio A, Beers S, Schneider

W, et al. Reliable magnetoencephalographic measures in chronic post-concussive syndrome-a teamtbi study. Journal of Neurotrauma. 2019;36(13):A115. doi: 10.1089/neu.2019.29100.abstracts.

2915. Kulju T, Haapasalo J, Rainesalo S, Lehtimäki K, Peltola J. Autostimulation in Vagus Nerve Stimulator Treatment: Modulating Neuromodulation. Neuromodulation. 2019;22(5):630-7. doi: 10.1111/ner.12897.

2916. Kulyk AV. The system of differentiated medical neurorehabilitation in the traumatic post-coma conditions of long-term disorders of consciousness. Acta Neuropsychologica. 2019;17(1):9-24. doi: 10.5604/01.3001.0013.1405.

2917. Kumar D, Prabhakaran J, Kim J, Castrillon J, Molotkov A, Dileep H, et al. In vivo evaluation of microtubul ligand [11C]MPC-6827 in animal models of neurodegenerative disorders. Journal of Nuclear Medicine. 2019;60.

2918. Lechner A, Spataro R, Guger C. Using vibro-tactile brain-computer interfaces in patients with unresponsive wakefulness syndrome. European Journal of Neurology. 2019;26:932-. PubMed PMID: WOS:000474481004141.

2919. Lee KM, Greenhalgh WM, Sargent P, Chae H, Klimp S, Engel S, et al. Unique Features of the US Department of Defense Multidisciplinary Concussion Clinics. Journal of Head Trauma

Rehabilitation. 2019;34(6):402-8. doi: 10.1097/HTR.0000000000000526.
2920. Lee M, Baird B, Gosseries O, Nieminen JO, Boly M, Postle BR, et al. Connectivity differences between consciousness and unconsciousness in non-rapid eye movement sleep: a TMS-EEG study. Sci Rep. 2019;9(1):5175. Epub 2019/03/28. doi: 10.1038/s41598-019-41274-2. PubMed PMID: 30914674; PubMed Central PMCID: PMCPMC6435892.
2921. Lee M, Baird B, Gosseries O, Nieminen JO, Boly M, Postle BR, et al. Connectivity differences between consciousness and unconsciousness in non-rapid eye movement sleep: a TMS–EEG study. Scientific Reports. 2019;9(1). doi: 10.1038/s41598-019-41274-2.
2922. Legostaeva L, Poydasheva A, Iazeva E, Sinitsyn D, Sergeev D, Bakulin I, et al. Stimulation of the Angular Gyrus Improves the Level of Consciousness. Brain Sci. 2019;9(5). Epub 2019/05/09. doi: 10.3390/brainsci9050103. PubMed PMID: 31064138; PubMed Central PMCID: PMCPMC6562708.
2923. Legostaeva L, Sinitsyn D, Sergeev D, Iazeva E, Kremneva E, Poydasheva A, et al. Comparison of the neurophysiological and neuroimaging data provides more accurate disorder of consciousness diagnostics. European Journal of Neurology. 2019;26:181-. PubMed PMID: WOS:000474481000286.

2924. Leopold A, Rumrill P, Hendricks DJ, Nardone A, Sampson E, Minton D, et al. A mixed-methodological examination of participant experiences, activities, and outcomes in a technology and employment project for postsecondary students with traumatic brain injuries. Journal of Vocational Rehabilitation. 2019;50(1):3-11. doi: 10.3233/JVR-180983.

2925. Levi V, Cordella R, D'Ammando A, Tringali G, Dones I, Messina G, et al. Dorsal anterior cingulate cortex (ACC) deep brain stimulation (DBS): a promising surgical option for the treatment of refractory thalamic pain syndrome (TPS). Acta Neurochirurgica. 2019;161(8):1579-88. doi: 10.1007/s00701-019-03975-5.

2926. Levy R. Motor cortex stimulation for pain and related conditions: Pro. Neuromodulation. 2019;22(7):e316. doi: 10.1111/ner.12958.

2927. Li D, Mashour GA. Cortical dynamics during psychedelic and anesthetized states induced by ketamine. NeuroImage. 2019;196:32-40. doi: 10.1016/j.neuroimage.2019.03.076.

2928. Li H, Wang Z, He Y, Meng S, Xu Y, Chen S, et al. Rational synthesis of Mn(x)Cd(1-x)S for enhanced photocatalytic H(2) evolution: Effects of S precursors and the feed ratio of Mn/Cd on its structure and performance. J Colloid Interface Sci. 2019;535:469-80. Epub 2018/10/16. doi: 10.1016/j.jcis.2018.10.018. PubMed PMID:

30321782.

2929. Li H, Wang Z, He Y, Meng S, Xu Y, Chen S, et al. Rational synthesis of MnxCd1-xS for enhanced photocatalytic H-2 evolution: Effects of S precursors and the feed ratio of Mn/Cd on its structure and performance. Journal of Colloid and Interface Science. 2019;535:469-80. doi: 10.1016/j.jcis.2018.10.018. PubMed PMID: WOS:000452811600050.

2930. Li L, Violante I, Zimmerman K, Leech R, Hampsire A, Carmichael D, et al. Exploring the targeted application of transcranial direct current stimulation (tDCS) for cognitive modulation after brain injury. Brain stimulation. 2019;12(2):454-. doi: 10.1016/j.brs.2018.12.475. PubMed PMID: CN-01790184.

2931. Li LM, Violante IR, Zimmerman K, Leech R, Hampshire A, Patel M, et al. Traumatic axonal injury influences the cognitive effect of non-invasive brain stimulation. Brain. 2019;142(10):3280-93. Epub 2019/09/11. doi: 10.1093/brain/awz252. PubMed PMID: 31504237; PubMed Central PMCID: PMCPMC6794939.

2932. Li S, Dong X, Sun W, Zhao N, Yu G, Shuai L. Effects of transcranial direct current stimulation on patients with disorders of consciousness after traumatic brain injury: study protocol for a randomized, double-blind controlled trial. Trials. 2019;20(1):596. Epub 2019/10/19. doi: 10.1186/s13063-019-3680-1. PubMed PMID:

31623656; PubMed Central PMCID: PMCPMC6796458.

2933. Li S, Lu K, Pan M, Zhang R. A new similarity analysis of EEG signals for automaticepileptic seizure detection. Journal of Northwest University Natural Science Edition. 2019;49(2):309-17. PubMed PMID: CSCD:6466355.

2934. Li Z, Wang X, Wang X, Lin Y, Meng A, Yang L, et al. Mn-Cd-S@amorphous-Ni3S2 hybrid catalyst with enhanced photocatalytic property for hydrogen production and electrocatalytic OER. Applied Surface Science. 2019;491:799-806. doi: 10.1016/j.apsusc.2019.05.313. PubMed PMID: WOS:000479082900090.

2935. Lim Y, Baek KH, Kim HJ, Lee S, Lee JW, Kang MI. Changes in trabecular bone score and bone mineral density following allogeneic hematopoietic stem cell transplantation. Bone. 2019;124:40-6. Epub 2019/04/09. doi: 10.1016/j.bone.2019.04.004. PubMed PMID: 30959188.

2936. Lin Y, Huang Q, Han T, Su Y, Gao D, Chen W, et al. Evaluating the Effect of Simultaneous Transcranial Direct Current stimulation and Repetitive Transcranial Magnetic Stimulation on Minimally conscious state by Using EEG and Functional MRI. Brain Stimulation. 2019;12(2):403-4. doi: 10.1016/j.brs.2018.12.303.

2937. Lin Y, Liu T, Huang Q, Su Y, Chen W, Gao D, et al.

Electroencephalography and Functional Magnetic Resonance Imaging-Guided Simultaneous Transcranial Direct Current Stimulation and Repetitive Transcranial Magnetic Stimulation in a Patient With Minimally Conscious State. Front Neurosci. 2019;13:746. Epub 2019/08/17. doi: 10.3389/fnins.2019.00746. PubMed PMID: 31417339; PubMed Central PMCID: PMCPMC6685103.

2938. Lindsey GL, Yesen A, Christie AD. The Impact of Physical Activity and Sleep on Physiology Following a mTBI. Int J Exerc Sci. 2019;12(3):919-31. Epub 2019/09/17. PubMed PMID: 31523349; PubMed Central PMCID: PMCPMC6719821.

2939. Liu F, Lin Z, Jin Q, Wu Q, Yang C, Chen H-J, et al. Protection of Nanostructures-Integrated Microneedle Biosensor Using Dissolvable Polymer Coating. Acs Applied Materials & Interfaces. 2019;11(5):4809-19. doi: 10.1021/acsami.8b18981. PubMed PMID: WOS:000458347900012.

2940. Liu H, Li Z, Yao Z, Liu Y, Zhang Q, Sun Y, et al. Designed MnS/Co(9)S(8) micro-flowers composites with serrate edges as high-performance electrodes for asymmetric supercapacitor. J Colloid Interface Sci. 2019;551:119-29. Epub 2019/05/11. doi: 10.1016/j.jcis.2019.05.024. PubMed PMID: 31075626.

2941. Liu J, Fan Y, Kim D, Zhong T, Yi P, Fan C, et al. Neuroprotective effect of catechins derivatives isolated from Anhua

dark tea on NMDA-induced excitotoxicity in SH-SY5Y cells. Fitoterapia. 2019;137. doi: 10.1016/j.fitote.2019.104240.

2942. Liu Q, Li R, Qu W, Li B, Yang W, Cui R. Pharmacological and non-pharmacological interventions of depression after traumatic brain injury: A systematic review. European Journal of Pharmacology. 2019;865. doi: 10.1016/j.ejphar.2019.172775. PubMed PMID: WOS:000500178300041.

2943. Liu T, Lu Y, Yu J, Kuang W, Wang X, Jiang Y, et al. Effect of auricular electroacupuncture combined with body acupuncture in improving the consciousness of patients after traumatic brain injury: Study protocol for a randomized controlled trial. Medicine (Baltimore). 2019;98(30):e16587. Epub 2019/07/28. doi: 10.1097/md.0000000000016587. PubMed PMID: 31348298; PubMed Central PMCID: PMCPMC6709251.

2944. Liu Y, Fan S, Huang J, Wang P, Yin Y, Xue L, et al. Clinical effect of hyperbaric oxygen combined with transcranial electrical stimulation and memantine hydrochloride on cognitive impairment in the patients with craniocerebral injury. Chinese Journal of Nautical Medicine and Hyperbaric Medicine. 2019;26(4):320-3. PubMed PMID: CSCD:6573524.

2945. Locatelli D, Veiceschi P, Castelnuovo P, Tanriover N, Evliyaoglu O, Canaz H, et al. Transsphenoidal surgery for pituitary

adenomas in pediatric patients: a multicentric retrospective study. Childs Nerv Syst. 2019;35(11):2119-26. Epub 2019/05/13. doi: 10.1007/s00381-019-04179-z. PubMed PMID: 31079182.

2946. Lowe J, Goodman C, Larson E, Guernon A. Safety and Feasibility of tDCS with Computerized Attention Training after TBI. Archives of physical medicine and rehabilitation. 2019;100(10):e118-. doi: 10.1016/j.apmr.2019.08.351. PubMed PMID: CN-01989744.

2947. Luber B. Optimization of TMS Delivery in the Temporal Domain. Neuropsychopharmacology. 2019;44(SUPPL 1):74-5. PubMed PMID: WOS:000509665600159.

2948. Luo Z, Sun W, Fang J, Lee K, Li S, Gu Z, et al. Biodegradable Gelatin Methacryloyl Microneedles for Transdermal Drug Delivery. Advanced Healthcare Materials. 2019;8(3). doi: 10.1002/adhm.201801054. PubMed PMID: WOS:000459627200002.

2949. Ly LT, Benthall KN, Pale T, McClellan AD. Restoration of the Topological Organization of the Trigeminal System Following Trigeminal Nerve Root Injury in the Lamprey. Neuroscience. 2019;423:216-31. Epub 2019/09/05. doi: 10.1016/j.neuroscience.2019.08.044. PubMed PMID: 31484046.

2950. Mahncke HW, Kim SJ, Rose A, Stasio C, Buckley P, Caroff S, et al. Evaluation of a plasticity-based cognitive training program in schizophrenia: Results from the eCaesar trial. Schizophrenia Research.

2019;208:182-9. doi: 10.1016/j.schres.2019.03.006.

2951. Marcó-García S, Ferrer-Quintero M, Usall J, Ochoa S, Del Cacho N, Huerta-Ramos E. Facial emotion recognition in neurological disorders: A narrative review. Revista de Neurologia. 2019;69(5):207-19. doi: 10.33588/rn.6905.2019047.

2952. Martens G, Barra A, Carriere M, Soria-Frisch A, Ruffini G, Ibanez D, et al. Closed-loop application of tDCS to promote responsiveness in patients with disorders of consciousness. Brain stimulation. 2019;12(2):459-. doi: 10.1016/j.brs.2018.12.493. PubMed PMID: CN-01787170.

2953. Martens G, Barra A, Laureys S, Thibaut A. Recent advance in the treatment of patients with disorders of consciousness: a review of transcranial direct current stimulation efficacy. Brain Stimulation. 2019;12(2):428. doi: 10.1016/j.brs.2018.12.385.

2954. Martens G, Fregni F, Carrière M, Barra A, Laureys S, Thibaut A. Single tDCS session of motor cortex in patients with disorders of consciousness: a pilot study. Brain Inj. 2019;33(13-14):1679-83. Epub 2019/09/17. doi: 10.1080/02699052.2019.1667537. PubMed PMID: 31523995.

2955. Martens K, Pechacek KP, Zhu B, Modrak CG, Milleson VJ, O'Hearn CO, et al. TRANSCRANIAL DIRECT-CURRENT STIMULATION REDUCES IMPULSIVITY AFTER

EXPERIMENTAL TBI. Journal of Neurotrauma. 2019;36(13):A84-A. PubMed PMID: WOS:000473049600230.
2956. Martens KM, Pechacek KM, Modrak CG, Milleson VJ, Zhu B, Vonder Haar C. Cathodal Transcranial Direct-Current Stimulation Selectively Decreases Impulsivity after Traumatic Brain Injury in Rats. J Neurotrauma. 2019;36(19):2827-30. Epub 2019/05/11. doi: 10.1089/neu.2019.6470. PubMed PMID: 31072218; PubMed Central PMCID: PMCPMC6744944.
2957. Masuda F. Potentials of rTMS for neurodevelopmental disorders and road to clarification of TMS neuropathology. Brain Stimulation. 2019;12(2):476. doi: 10.1016/j.brs.2018.12.553.
2958. Mattavelli G, Pisoni A, Romero Lauro LJ, Marino BF, Bonomi M, Rosanova M, et al. TMS-EEG approach unveils brain mechanisms underlying conscious and unconscious face perception. Brain Stimul. 2019;12(4):1010-9. Epub 2019/03/11. doi: 10.1016/j.brs.2019.02.022. PubMed PMID: 30852122.
2959. McCall IC, Lau C, Minielly N, Illes J. Owning Ethical Innovation: Claims about Commercial Wearable Brain Technologies. Neuron. 2019;102(4):728-31. doi: 10.1016/j.neuron.2019.03.026. PubMed PMID: WOS:000468597000009.
2960. Meek BP, Hill SR, Modirrousta M. Abstract #79: Efficacy of Low-Frequency rTMS over the Right Dorsolateral Prefrontal Cortex

following Mild Traumatic Brain Injury. Brain Stimulation. 2019;12(2):e28. doi: 10.1016/j.brs.2018.12.086.

2961. Meng H, Zhao H, Cao X, Hao J, Zhang H, Liu Y, et al. Double-negative T cells remarkably promote neuroinflammation after ischemic stroke. Proceedings of the National Academy of Sciences of the United States of America. 2019;116(12):5558-63. doi: 10.1073/pnas.1814394116.

2962. Mensen A, Thibaut A, Bodart O, Wannez S, Laureys S, Gosseries O. Decreased Evoked Slow-Activity After tDCS in Patients with Severe Brain Injuries and Altered State of Consciousness. Brain Injury. 2019;33:16-. PubMed PMID: WOS:000466897000038.

2963. Metzger-Smith V, Vaninetti M, Lim M, Khalaf A, Yang E, Song D, et al. PREFRONTAL MODULATORY FUNCTIONAL CHANGES AFTER RTMS IN MTBI PATIENTS WITH HEADACHES. Journal of Neurotrauma. 2019;36(13):A66-A. PubMed PMID: WOS:000473049600180.

2964. Michou E, Sasegbon A, Hamdy S. Direct and indirect therapy: Neurostimulation for the treatment of dysphagia after stroke. 2019. p. 731-61.

2965. Mo JJ, Hu WH, Zhang C, Wang X, Liu C, Zhao BT, et al. Motor cortex stimulation: a systematic literature-based analysis of effectiveness and case series experience. BMC Neurol. 2019;19(1):48.

Epub 2019/03/31. doi: 10.1186/s12883-019-1273-y. PubMed PMID: 30925914; PubMed Central PMCID: PMCPMC6440080.

2966. Moretti A, de Sire A, Curci C, Toro G, Gimigliano F, Iolascon G. Effectiveness of denosumab on back pain-related disability and quality-of-life in patients with vertebral fragility fractures. Curr Med Res Opin. 2019;35(1):151-5. Epub 2018/11/09. doi: 10.1080/03007995.2018.1545636. PubMed PMID: 30406685.

2967. Mosiello G, Ludy Lopes D, Musciagna F, Del Conte F, Iacobelli B, Chiminello R, et al. Conservative treatment of bowel dysfunction in pediatric population: Role of a correct training for improving effectiveness of transanal irrigation. Neurourology and Urodynamics. 2019;38:S107-S8. doi: 10.1002/nau.24118.

2968. Moussavi Z, Suleiman A, Rutherford G, Ranjbar Pouya O, Dastgheib Z, Zhang W, et al. A Pilot Randomised Double-Blind Study of the Tolerability and efficacy of repetitive Transcranial Magnetic Stimulation on Persistent Post-Concussion Syndrome. Sci Rep. 2019;9(1):5498. Epub 2019/04/04. doi: 10.1038/s41598-019-41923-6. PubMed PMID: 30940870; PubMed Central PMCID: PMCPMC6445141.

2969. Muir O, MacMillan C, Khan M, Sang L, Aron T. Safety and tolerability of dTMS treatment in 18-20 year old patients: case series. Brain Stimulation. 2019;12(4):e137-e8. doi:

10.1016/j.brs.2019.03.049.

2970. Muresanu D. Advances in post stroke neurorehabilitation - From neuro fundamentals to evidence based medicine. Journal of the Neurological Sciences. 2019;405:75. doi: 10.1016/j.jns.2019.10.209.

2971. Nagel SJ, Helland L, Woodroffe RW, Frizon LA, Holland MT, Machado AG, et al. Durotomy Surrogate and Seals for Intradural Spinal Cord Stimulators: Apparatus and Review of Clinical Methods and Materials. Neuromodulation. 2019;22(8):916-29. Epub 2019/01/12. doi: 10.1111/ner.12913. PubMed PMID: 30632655.

2972. Namgung E, Kim M, Yoon S. Repetitive transcranial magnetic stimulation in trauma-related conditions. Neuropsychiatr Dis Treat. 2019;15:701-12. Epub 2019/04/03. doi: 10.2147/ndt.S189498. PubMed PMID: 30936700; PubMed Central PMCID: PMCPMC6430993.

2973. Narayana S, Charles C, Collins K, Tsao JW, Stanfill AG, Baughman B. Neuroimaging and Neuropsychological Studies in Sports-Related Concussions in Adolescents: Current State and Future Directions. Front Neurol. 2019;10:538. Epub 2019/06/11. doi: 10.3389/fneur.2019.00538. PubMed PMID: 31178818; PubMed Central PMCID: PMCPMC6542940.

2974. Nardone R, Langthaler P, Orioli A, Versace V, Scarano G, Brigo F, et al. Ipsilateral motor evoked potentials in a patient with

unihemispheric cortical atrophy due to Rasmussen encephalitis. Neural Regeneration Research. 2019;14(6):1025-8. doi: 10.4103/1673-5374.250581.
2975. Nawshin T, Behan M, Nemanich S, Kowalski J, Sutter E, Boxrud M, et al. Factors influencing parent and caregiver decisions to enroll children to participate in perceived high-risk brain research. Developmental Medicine and Child Neurology. 2019;61:28. doi: 10.1111/dmcn.14244.
2976. Nct. Prefontal tDCS in Patients With Disorders of Consciousness: Neurophysiological and Behavioural Outcomes. https://clinicaltrialsgov/show/NCT03823508. 2019. PubMed PMID: CN-01918993.
2977. Nct. Transcutaneous Auricular Vagal Nerve Stimulation for Post-coma Patients With Disorders of Consciousness. https://clinicaltrialsgov/show/NCT04065386. 2019. PubMed PMID: CN-01983669.
2978. Nct. rTMS Target Identification for Functional Disability in AUD+mTBI. https://clinicaltrialsgov/show/NCT04043442. 2019. PubMed PMID: CN-01966108.
2979. Nct. Pilot rTMS for AUD+mTBI. https://clinicaltrialsgov/show/NCT03995173. 2019. PubMed PMID: CN-01946067.

2980. Nct. High-Definition Transcranial Direct Current Stimulation (HD-tDCS) for Sensory Deficits in Complex Traumatic Brain Injury. https://clinicaltrialsgov/show/NCT03799458. 2019. PubMed PMID: CN-01965210.

2981. Nct. Neurofeedback Impact on Veterans With mTBI. https://clinicaltrialsgov/show/NCT04195685. 2019. PubMed PMID: CN-02053115.

2982. Nct. Neuromodulation and Neurorehabilitation for mTBI Plus PTSD. https://clinicaltrialsgov/show/NCT03819608. 2019. PubMed PMID: CN-01706661.

2983. Nct. Closed-loop tDCS in Patients in Minimally Conscious State. https://clinicaltrialsgov/show/NCT03810079. 2019. PubMed PMID: CN-01795963.

2984. Nct. Multichannel tDCS to Reduce Hypertonia in Patients With Prolonged DOC. https://clinicaltrialsgov/show/NCT03797573. 2019. PubMed PMID: CN-01795773.

2985. Nct. Remotely Supervised Transcranial Direct Current Stimulation for Persistent Post-traumatic Headache. https://clinicaltrialsgov/show/NCT04012853. 2019. PubMed PMID: CN-01953145.

2986. Nct. Spinal Cord Stimulation in Patients With Disorders of Consciousness. https://clinicaltrialsgov/show/NCT04010838. 2019.

PubMed PMID: CN-01953095.

2987. Nct. Cortical Contributions to Motor Sequence Learning. https://clinicaltrialsgov/show/NCT04138953. 2019. PubMed PMID: CN-02001358.

2988. Nct. Computerised Working Memory Training in Acquired Brain Injury. https://clinicaltrialsgov/show/NCT04010149. 2019. PubMed PMID: CN-01953080.

2989. Nct. TMS and Exposure Therapy. https://clinicaltrialsgov/show/NCT04019054. 2019. PubMed PMID: CN-01953302.

2990. Nct. Improving Hand Recovery With Neuromodulation in Tetraplegia. https://clinicaltrialsgov/show/NCT03954496. 2019. PubMed PMID: CN-01932763.

2991. Nct. Subacromial Corticosteroid Injections and tDCS in Rotator Cuff Tendinopathy. https://clinicaltrialsgov/show/NCT03967574. 2019. PubMed PMID: CN-01945158.

2992. Nct. Acute Effects of Pharmacological Neuromodulation on Leg Motor Activity in Patients With SCI Treated With EES. https://clinicaltrialsgov/show/NCT04052776. 2019. PubMed PMID: CN-01966345.

2993. Nct. Therapeutic Effects of Transcranial Alternative Current

Stimulation (tACS) in Chronic Post Stroke Hemianopia. https://clinicaltrialsgov/show/NCT04043689. 2019. PubMed PMID: CN-01966116.
2994. Nct. Efficacy of Equine Assisted Therapy on Negative Symptoms in Patients With Schizophrenia. https://clinicaltrialsgov/show/NCT03935243. 2019. PubMed PMID: CN-01931768.
2995. Nemanich S, Zewdie E, Kuo HC, Francis S, MacKinnon C, Meekins G, et al. Atypical motor evoked potentials from single-pulse transcranial magnetic stimulation in children with hemiparetic cerebral palsy. Neurorehabilitation and Neural Repair. 2019;33(12):1073. doi: 10.1177/1545968319893871.
2996. Nemanich ST, Chen CY, Chen M, Zorn E, Mueller B, Peyton C, et al. Safety and Feasibility of Transcranial Magnetic Stimulation as an Exploratory Assessment of Corticospinal Connectivity in Infants After Perinatal Brain Injury: An Observational Study. Phys Ther. 2019;99(6):689-700. Epub 2019/02/27. doi: 10.1093/ptj/pzz028. PubMed PMID: 30806664; PubMed Central PMCID: PMCPMC6545276.
2997. Nemanich ST, Kowalski JL, Chen M, Zorn E, Georgieff M, Gillick BT. Proceedings #1: Longitudinal Assessment of Cortical Excitability in an Infant with Perinatal Brain injury. Brain Stimulation.

2019;12(2):e56-e7. doi: 10.1016/j.brs.2018.12.170.

2998. Nemanich ST, Mueller BA, Gillick BT. Neurite orientation dispersion and density imaging quantifies corticospinal tract microstructural organization in children with unilateral cerebral palsy. Human Brain Mapping. 2019;40(17):4888-900. doi: 10.1002/hbm.24744.

2999. Neville IS, Zaninotto AL, Hayashi CY, Rodrigues PA, Galhardoni R, Ciampi de Andrade D, et al. Repetitive TMS does not improve cognition in patients with TBI: A randomized double-blind trial. Neurology. 2019;93(2):e190-e9. Epub 2019/06/09. doi: 10.1212/wnl.0000000000007748. PubMed PMID: 31175209; PubMed Central PMCID: PMCPMC6656650.

3000. Neville IS, Zaninotto AL, Hayashi CY, Rodrigues PA, Galhardoni R, de Andrade DC, et al. Repetitive TMS does not improve cognition in patients with TBI. Neurology. 2019;93(2):E190-E9. doi: 10.1212/wnl.0000000000007748. PubMed PMID: WOS:000480783800009.

3001. Nicolini C, Toepp S, Harasym D, Michalski B, Fahnestock M, Gibala MJ, et al. No changes in corticospinal excitability, biochemical markers, and working memory after six weeks of high-intensity interval training in sedentary males. Physiol Rep. 2019;7(11):e14140. Epub 2019/06/09. doi: 10.14814/phy2.14140. PubMed PMID:

31175708; PubMed Central PMCID: PMCPMC6555846.

3002. Noller CM, Levine YA, Urakov TM, Aronson JP, Nash MS. Vagus Nerve Stimulation in Rodent Models: An Overview of Technical Considerations. Front Neurosci. 2019;13:911. Epub 2019/09/26. doi: 10.3389/fnins.2019.00911. PubMed PMID: 31551679; PubMed Central PMCID: PMCPMC6738225.

3003. Oknina L, Zigmantovich A, Kopachka M, Zaitsev O, Masherov E, Alexandrova E. The reorganization of functional connectivity under transcranial magnetic stimulation in unconscious patients with severe brain injury. Folia Neuropathologica. 2019;57(3):308. doi: 10.5114/fn.2019.88492.

3004. Oliveira E, Santos HM, Jorge S, Rodriguez-Gonzalez B, Novio F, Lorenzo J, et al. Sustainable synthesis of luminescent CdTe quantum dots coated with modified silica mesoporous nanoparticles: Towards new protein scavengers and smart drug delivery carriers. Dyes and Pigments. 2019;161:360-9. doi: 10.1016/j.dyepig.2018.09.047. PubMed PMID: WOS:000449241200044.

3005. Ondek K, Izadi A, Girgis F, Saez I, Shahlaie K, Gurkoff G. Theta stimulation to treat cognitive dysfunction in rodent models of neurologic disorders. Brain Stimulation. 2019;12(2):582. doi: 10.1016/j.brs.2018.12.933.

3006. Opie GM, Foo N, Killington M, Ridding MC, Semmler JG. Transcranial Magnetic Stimulation-Electroencephalography Measures of Cortical Neuroplasticity Are Altered after Mild Traumatic Brain Injury. J Neurotrauma. 2019;36(19):2774-84. Epub 2019/03/09. doi: 10.1089/neu.2018.6353. PubMed PMID: 30848163.
3007. Ort A, Preller K, Tononi G, Massimini M, Vollenweider FX. Characterization of psilocybin-induced altered state consciousness using TMS-EEG and integrated information theory (IIT). Neuropsychobiology. 2019;77(3):157. doi: 10.1159/000496817.
3008. Padmanabhan JL, Cooke D, Joutsa J, Siddiqi SH, Ferguson M, Darby RR, et al. A Human Depression Circuit Derived From Focal Brain Lesions. Biological Psychiatry. 2019;86(10):749-58. doi: 10.1016/j.biopsych.2019.07.023. PubMed PMID: WOS:000491238900007.
3009. Padua L, Cuccagna C, Pazzaglia C. Novel sensory paradigms for neuromodulation in disorders of consciousness in traumatic brain injury. Curr Opin Neurol. 2019;32(6):844-9. Epub 2019/10/01. doi: 10.1097/wco.0000000000000747. PubMed PMID: 31567499.
3010. Papa L, Ptito A. Translingual stimulation (TLNS) with the portable neuromodulation stimulator (PoNSTM) for the treatment of chronic balance deficit due to mild-to-moderate traumatic brain injury (mmTBI). Annals of neurology. 2019;86:S173-. PubMed PMID:

CN-02095662.

3011. Papa L, Ptito A. Translingual neurostimulation with the portable neuromodulation stimulator (PoNSâ„¢) for the treatment of chronic balance deficit due to mild-to-moderate traumatic brain injury. PM and r. 2019;11:S89-S90. doi: 10.1002/pmrj.12271. PubMed PMID: CN-02119961.

3012. Papa L, Ptito A. Translingual neurostimulation with the portable neuromodulation stimulator (PoNS™) for the treatment of chronic balance deficit due to mild-to-moderate traumatic brain injury. PM and R. 2019;11:S89-S90. doi: 10.1002/pmrj.12271.

3013. Papa L, Yochelson M, Walker WC. Improvements across functional outcomes in patients treated for chronic balance deficit after mild-tomoderate traumatic brain injury with translingual neurostimulation. Neuromodulation. 2019;22(7):e445-. doi: 10.1111/ner.12958. PubMed PMID: CN-02130515.

3014. Pape TB, Rosenow J, Herrold A, Livengood S, Kletzel S, Guernon A, et al. ReEnabling ConsciOus behaViors by Engaging dopamineRgic pathwaYs (RECOVERY). Brain stimulation. 2019;12(2):559-. doi: 10.1016/j.brs.2018.12.849. PubMed PMID: CN-01790175.

3015. Park JS, Cho Y, You Y, Min JH, Jeong W, Ahn HJ, et al. Optimal timing to measure optic nerve sheath diameter as a prognostic

predictor in post-cardiac arrest patients treated with targeted temperature management. Resuscitation. 2019;143:173-9. doi: 10.1016/j.resuscitation.2019.07.004.

3016. Parravano DC, Ciampi DA, Fonoff ET, Monaco B, Navarro J, Yeng LT, et al. Quality of Life After Motor Cortex Stimulation: Clinical Results and Systematic Review of the Literature. Neurosurgery. 2019;84(2):451-6. doi: 10.1093/neuros/nyy060. PubMed PMID: WOS:000463732100026.

3017. Paxman E, Stilling J, Mercier L, Debert C. Repetitive Transcranial Magnetic Stimulation (rTMS) as an Effective Intervention for Chronic Dizziness Following Mild Traumatic Brain Injury: a Case Study. Brain stimulation. 2019;12(2):583-4. doi: 10.1016/j.brs.2018.12.937. PubMed PMID: CN-01788612.

3018. Paydar A, Schoenfield JS, Yan E, Kamali A, Kochan T, Harris NG. PROLONGED NEUROMODULATION AFTER EXPERIMENTAL TBI ENHANCES FUNCTIONAL CONNECTIVITY BUT DOES NOT IMPROVE EVOKED ACTIVATION. Journal of Neurotrauma. 2019;36(13):A142-A3. PubMed PMID: WOS:000473049600393.

3019. Pearce AJ, Tommerdahl M, King DA. Neurophysiological abnormalities in individuals with persistent post-concussion symptoms. Neuroscience. 2019;408:272-81. Epub 2019/04/21. doi:

10.1016/j.neuroscience.2019.04.019. PubMed PMID: 31004695.
3020. Pellegrini M, Zoghi M, Jaberzadeh S. Case Report of a Syncope Episode in a Healthy Male Adult Participant During Single-Pulse Transcranial Magnetic Stimulation. J ect. 2019;35(2):e11-e2. Epub 2018/08/17. doi: 10.1097/yct.0000000000000526. PubMed PMID: 30113987.
3021. Peng CW, Shih-Ching C. Motor plasticity effects induced by cortical electrical stimulation in traumatic brain injured rats. Neuromodulation. 2019;22(7):e376. doi: 10.1111/ner.12958.
3022. Pigorini A, Parmigiani S, Russo S, Mikulan E, Cattani A, Sarasso S, et al. P50-S State dependent changes in cortical reactivity: Comparison between transcranial and intracranial stimulation. Clinical Neurophysiology. 2019;130(7):e109. doi: 10.1016/j.clinph.2019.04.586.
3023. Podda M, Paciello F, Cocco S, Rolesi R, Troiani D, Fetoni A, et al. Transcranial direct current stimulation affects auditory cortex plasticity in normal-hearing and noise-exposed rats. Brain Stimulation. 2019;12(2):456. doi: 10.1016/j.brs.2018.12.481.
3024. Pommier B, Quesada C, Fauchon C, Nuti C, Vassal F, Peyron R. Added value of multiple versus single sessions of repetitive transcranial magnetic stimulation in predicting motor cortex stimulation efficacy for refractory neuropathic pain. Journal of

Neurosurgery. 2019;130(5):1750-61. doi: 10.3171/2017.12.Jns171333. PubMed PMID: WOS:000466401100040.

3025. Popescu M, Popescu EA, DeGraba TJ, Fernandez-Fidalgo DJ, Riedy G, Hughes JD. Post-traumatic stress disorder is associated with altered modulation of prefrontal alpha band oscillations during working memory. Clinical Neurophysiology. 2019;130(10):1869-81. doi: 10.1016/j.clinph.2019.06.227.

3026. Portinho PC. High-risk patient capability for osteoporotic fractures / FUSEX ＊ proposal for the inclusion of patients in a national database. (relevance of FLS-fracture liaison services). Osteoporosis International. 2019;30(SUPPL 2):S454-S5. doi: 10.1007/s00198-019-04993-w.

3027. Poydasheva AG, Bakulin IS, Legostaeva LA, Suponeva NA, Piradov MA. TMS-EEG: CURRENT POSSIBILITIES AND FUTURE PROSPECTS. Zhurnal Vysshei Nervnoi Deyatelnosti Imeni I P Pavlova. 2019;69(3):267-79. doi: 10.1134/s0044467719030092. PubMed PMID: WOS:000473092700001.

3028. Ptito A. Treatment of chronic symptoms of mild/moderate traumatic brain injury with the portable neuromodulation stimulator. Neuromodulation. 2019;22(3):E88-. doi: 10.1111/ner.12950. PubMed PMID: CN-01941442.

3029. Ptito A, Papa L, Skinner K, Tyler M. Predicting therapeutic

responses to cranial nerve noninvasive translingual neurostimulation in persons with mild-to-moderate traumatic brain injury using pooled data from two randomized clinical trials. Neuromodulation. 2019;22(7):e446-. doi: 10.1111/ner.12958. PubMed PMID: CN-02164356.

3030. Ptito A, Skinner K, Papa L. The Portable Neuromodulation Stimulator for Chronic Symptoms Due to Mild-to-Moderate Traumatic Brain Injury. Archives of physical medicine and rehabilitation. 2019;100(10):e132-e3. doi: 10.1016/j.apmr.2019.08.402. PubMed PMID: CN-01987494.

3031. Quentin R, Awosika O, Cohen LG. Plasticity and recovery of function. Handbook of clinical neurology. 2019;163:473-83. doi: 10.1016/b978-0-12-804281-6.00025-2. PubMed PMID: MEDLINE:31590747.

3032. Quinn D, Upston J, Jones T, Richardson J, Worth L, Fratzke V, et al. Abstract #1: Individualizing HD-tDCS With fMRI and E-Field Modeling: Pilot Data from the NAVIGATE-TBI Study. Brain Stimulation. 2019;12(2):e1. doi: 10.1016/j.brs.2018.12.008.

3033. Quinn D, Upston J, Jones TR, Fratzke V, Brandt E, Gill D, et al. Cerebral perfusion and postconcussive symptom reduction after cognitive training in chronic mmTBI patients. Journal of Neurotrauma. 2019;36(13):A48-A9. doi: 10.1089/neu.2019.29100.abstracts.

3034. Rackoll T, Darkow R, Wusten K, Floel A, Gorsler A. Proof of principle: traumatic brain injury speech rehabilitation under direct current stimulation (BISCUIT). Clinical neurophysiology. 2019;130(8):e149-. doi: 10.1016/j.clinph.2019.04.663. PubMed PMID: CN-02081158.

3035. Rao V, Bechtold K, McCann U, Roy D, Peters M, Vaishnavi S, et al. Low-Frequency Right Repetitive Transcranial Magnetic Stimulation for the Treatment of Depression After Traumatic Brain Injury: A Randomized Sham-Controlled Pilot Study. J Neuropsychiatry Clin Neurosci. 2019;31(4):306-18. Epub 2019/04/26. doi: 10.1176/appi.neuropsych.17110338. PubMed PMID: 31018810.

3036. Rastogi P, Lee EG, Hadimani RL, Jiles DC. Transcranial Magnetic Stimulation: Development of a Novel Deep-Brain Triple-Halo Coil. Ieee Magnetics Letters. 2019;10. doi: 10.1109/lmag.2019.2903993. PubMed PMID: WOS:000463473100001.

3037. Rauca VF, Vlase L, Casian T, Sesarman A, Gheldiu AM, Mocan A, et al. Biologically active Ajuga species extracts modulate supportive processes for cancer cell development. Frontiers in Pharmacology. 2019;10(APR). doi: 10.3389/fphar.2019.00334.

3038. Reilmann R, McGarry A, Grachev ID, Savola JM, Borowsky B, Eyal E, et al. Safety and efficacy of pridopidine in patients with

Huntington's disease (PRIDE-HD): a phase 2, randomised, placebo-controlled, multicentre, dose-ranging study. Lancet Neurol. 2019;18(2):165-76. Epub 2018/12/20. doi: 10.1016/s1474-4422(18)30391-0. PubMed PMID: 30563778.

3039. Rezaeizadeh H, Rahimi R, Abbasi M. Fatigue due to multiple sclerosis: A comparison between Persian medicine and conventional medicine. Galen Medical Journal. 2019;8. doi: 10.31661/gmj.v0i0.1139.

3040. Rong P, Zhang Y, Li S, Wang Y. Present Status and Prospect of Transcutaneous Auricular Vagus Nerve Stimulation in the Treatment of Encephalopathy and Relating Diseases. Modernization of Traditional Chinese Medicine and Materia Medica--World Science and Technology. 2019;21(9):1799-804. PubMed PMID: CSCD:6672742.

3041. Rosenow JM, Kletzel S, Herrold A, Guernon A, Patil V, Bender-Pape TL. Amantadine + RTMS as a neurotherapeutic for disordered consciousness after TBI: safety findings. Neuromodulation. 2019;22(3):E202-. doi: 10.1111/ner.12950. PubMed PMID: CN-01941712.

3042. Rosenthal ZP, Raut R, Voss T, Bauer AQ, Culver J, Raichle M, et al. Local perturbations in cortical excitability propagate differentially through large-scale functional networks. Annals of

Neurology. 2019;86:S42-S3.

3043. Ruiz de Miras J, Soler F, Iglesias-Parro S, Ibáñez-Molina AJ, Casali AG, Laureys S, et al. Fractal dimension analysis of states of consciousness and unconsciousness using transcranial magnetic stimulation. Comput Methods Programs Biomed. 2019;175:129-37. Epub 2019/05/21. doi: 10.1016/j.cmpb.2019.04.017. PubMed PMID: 31104702.

3044. Sa M, Singh R, Pujar S, D'Arco F, Desai N, Eltze C, et al. Centromedian thalamic nuclei deep brain stimulation and Anakinra treatment for FIRES – Two different outcomes. European Journal of Paediatric Neurology. 2019;23(5):749-54. doi: 10.1016/j.ejpn.2019.08.001.

3045. Saleem GT, Ewen JB, Crasta JE, Slomine BS, Cantarero GL, Suskauer SJ. Single-arm, open-label, dose escalation phase I study to evaluate the safety and feasibility of transcranial direct current stimulation with electroencephalography biomarkers in paediatric disorders of consciousness: a study protocol. BMJ Open. 2019;9(8):e029967. Epub 2019/08/12. doi: 10.1136/bmjopen-2019-029967. PubMed PMID: 31401607; PubMed Central PMCID: PMCPMC6701812.

3046. Saleh Velez F, Bonin Pinto C, Ortiz JG, Mansour A, Lazaridis C. The PASHT trial: randomized crossover sham-controlled trial

evaluating safety and feasibility of transcranial direct current stimulation (TDCS) for paroxysmal sympathetic hyperactivity post stroke and TBI. European stroke journal. 2019;4:94-. doi: 10.1177/2396987319845578. PubMed PMID: CN-01961249.

3047. Schenker E. Proceedings #7: trigeminal and Occipital Neuromodulation for Rapid Pain Reduction in Occipital Migraines. Brain stimulation. 2019;12(2):e65-e6. doi: 10.1016/j.brs.2018.12.176. PubMed PMID: CN-01925716.

3048. Schlaug G. Abstract #161: When stimulating the unaffected (healthy) hemisphere in stroke patients makes sense. Brain Stimulation. 2019;12(2):e55. doi: 10.1016/j.brs.2018.12.168.

3049. Schmidt J, Brown K, Feldman S, Babul S, Zwicker J, Boyd L. Evidence of altered inter-hemispheric communication in paediatric mild traumatic brain injury. Brain Stimulation. 2019;12(2):483. doi: 10.1016/j.brs.2018.12.576.

3050. Scott G, Carhart-Harris RL. Psychedelics as a treatment for disorders of consciousness. Neuroscience of Consciousness. 2019;2019(1). doi: 10.1093/nc/niz003.

3051. Scott-King JM, Tieu S, Chiew AL, Lui J, Kirby KA, Chan BS. Clinical decision rule for non-traumatic computed tomography of the brain. EMA - Emergency Medicine Australasia. 2019;31(6):974-81. doi: 10.1111/1742-6723.13292.

3052. Sdoia S, Conversi D, Pecchinenda A, Ferlazzo F. Access to consciousness of briefly presented visual events is modulated by transcranial direct current stimulation of left dorsolateral prefrontal cortex. Sci Rep. 2019;9(1):10950. Epub 2019/07/31. doi: 10.1038/s41598-019-47527-4. PubMed PMID: 31358875; PubMed Central PMCID: PMCPMC6662720.

3053. Sekar S, Zhang Y, Miranzadeh Mahabadi H, Parvizi A, Taghibiglou C. Low-Field Magnetic Stimulation Restores Cognitive and Motor Functions in the Mouse Model of Repeated Traumatic Brain Injury: Role of Cellular Prion Protein. J Neurotrauma. 2019;36(22):3103-14. Epub 2019/04/26. doi: 10.1089/neu.2018.5918. PubMed PMID: 31020907.

3054. Seon-Woo H-S, Kim HJ, Roh JY, Park J-H. Dissolving Microneedle Systems for the Oral Mucosal Delivery of Triamcinolone Acetonide to Treat Aphthous Stomatitis. Macromolecular Research. 2019;27(3):282-9. doi: 10.1007/s13233-019-7031-6. PubMed PMID: WOS:000467619900009.

3055. Sergeeva A, Kremneva E, Morozova S, Sinitsyn D, Legostaeva L, Poydasheva A, et al. Effect of repetitive transcranial magnetic stimulation on resting-state functional connectivity in patients with chronic disorders of consciousness. Neuroradiology. 2019;61(1):S75. doi: 10.1007/s00234-019-02263-4.

3056. Seritan AL, Ureste P, Duong T, Ostrem JL. Psychopharmacology for patients with Parkinson's disease and deep brain stimulation: Lessons learned in an academic center. Current Psychopharmacology. 2019;8(1):41-54. doi: 10.2174/2211556007666180328142953.

3057. Sha R, Zhang B, Han X, Peng J, Zheng C, Zhang F, et al. Electroacupuncture Alleviates Ischemic Brain Injury by Inhibiting the miR-223/NLRP3 Pathway. Med Sci Monit. 2019;25:4723-33. Epub 2019/06/27. doi: 10.12659/msm.917213. PubMed PMID: 31237865; PubMed Central PMCID: PMCPMC6607941.

3058. Shelley-Tremblay JF, Eyer JC, Hill BD. A laboratory word memory test analogue differentiates intentional feigning from true responding using the p300 event-related potential. Brain Sciences. 2019;9(5). doi: 10.3390/brainsci9050109.

3059. Shen L, Ouyang H, Yang C. The effect of high- frequency repetitive transcranial magnetic stimulation on consciousness recovery in traumatic brain injury patients with disorders of consciousness. Chinese Journal of Rehabilitation Medicine. 2019;34(12):1411-7. PubMed PMID: CSCD:6629229.

3060. Shinde A, Munsch F, Alsop D, Schlaug G. Abstract #18: Establishing a dose response for transcranial direct current stimulation. Brain Stimulation. 2019;12(2):e7. doi: 10.1016/j.brs.2018.12.025.

3061. Siddiqi S, Trapp N, Hacker C, Kandala S, Leuthardt E, Carter A, et al. Functional connectivity changes with targeted rTMS of the dorsal attention network in TBI-associated depression. Brain stimulation. 2019;12(2):538-. doi: 10.1016/j.brs.2018.12.773. PubMed PMID: CN-01787184.
3062. Siddiqi SH, Trapp NT, Hacker CD, Laumann TO, Kandala S, Hong X, et al. Repetitive Transcranial Magnetic Stimulation with Resting-State Network Targeting for Treatment-Resistant Depression in Traumatic Brain Injury: A Randomized, Controlled, Double-Blinded Pilot Study. J Neurotrauma. 2019;36(8):1361-74. Epub 2018/11/02. doi: 10.1089/neu.2018.5889. PubMed PMID: 30381997; PubMed Central PMCID: PMCPMC6909726.
3063. Siddiqi SH, Trapp NT, Hacker CD, Laumann TO, Kandala S, Hong X, et al. Abstract #156: functional connectivity changes with targeted rTMS of the dorsal attention network in TBI-associated depression. Brain stimulation. 2019;12(2):e53-. doi: 10.1016/j.brs.2018.12.163. PubMed PMID: CN-01788649.
3064. Siddiqi SH, Trapp NT, Shahim P, Hacker CD, Laumann TO, Kandala S, et al. Individualized Connectome-Targeted Transcranial Magnetic Stimulation for Neuropsychiatric Sequelae of Repetitive Traumatic Brain Injury in a Retired NFL Player. J Neuropsychiatry Clin Neurosci. 2019;31(3):254-63. Epub 2019/04/05. doi:

10.1176/appi.neuropsych.18100230. PubMed PMID: 30945588.
3065. Simon-Martinez C, Decraene L, Van Hoof E, Zielinski I, Hoare B, Steenbergen B, et al. The impact of timing of the brain lesion and corticospinal tract organization on mirror movement characteristics in children with unilateral cerebral palsy. Developmental Medicine and Child Neurology. 2019;61:16. doi: 10.1111/dmcn.14244.
3066. Sinitsyn D, Kremneva E, Legostaeva L, Yazeva E, Sergeev D, Poidasheva A, et al. TMS-EEG and resting-state fmri accuracy comparison in differentiation of chronic disorders of consciousness. Neuroradiology. 2019;61(1):S33-S4. doi: 10.1007/s00234-019-02263-4.
3067. Skinner K, Papa L, Ptito A. Cranial Nerve Noninvasive Neuromodulation Using Translingual Stimulation with the Portable Neuromodulation Stimulator (PoNS (R)) for the Treatment of Chronic Balance Deficit Due to Mild-to-Moderate Traumatic Brain Injury. Brain Injury. 2019;33:317-8. PubMed PMID: WOS:000466897000639.
3068. Skinner K, Prabhakaran V. Neurorehabilitation using the portable neuromodulation stimulator (PoNSâ„¢) for treating participants with chronic balance deficit due to mild-to-moderate traumatic brain injury: a 26-week study. PM and r. 2019;11:S34-. doi:

10.1002/pmrj.12271. PubMed PMID: CN-02120108.

3069. Skinner K, Prabhakaran V. Neurorehabilitation using the portable neuromodulation stimulator (PoNS™) for treating participants with chronic balance deficit due to mild-to-moderate traumatic brain injury: A 26-week study. PM and R. 2019;11:S34. doi: 10.1002/pmrj.12271.

3070. Skinner K, Tyler M. Long-Term Treatment of Balance Deficits After Mild-to-Moderate TBI With the Portable Neuromodulation Stimulator. Archives of physical medicine and rehabilitation. 2019;100(10):e9-. doi: 10.1016/j.apmr.2019.08.015. PubMed PMID: CN-01989746.

3071. Smith TJ, Kamson D, Mealy MA, D'Amato S. Treatment of central and spinal cord neuropathic pain with Scrambler Therapy. Journal of Clinical Oncology. 2019;37(31). doi: 10.1200/JCO.2019.37.31-suppl.127.

3072. Sohn E. Decoding the neuroscience of consciousness. Nature. 2019;571(7766):S2-s5. Epub 2019/07/26. doi: 10.1038/d41586-019-02207-1. PubMed PMID: 31341312.

3073. Sohn E. DECODING CONSCIOUSNESS. Nature. 2019;571(7766):S2-S5. doi: 10.1038/d41586-019-02207-1. PubMed PMID: WOS:000477016700019.

3074. Sole JV. Transient loss of consciousness with brain stimulation.

Brain Stimulation. 2019;12(2):435. doi: 10.1016/j.brs.2018.12.410.
3075. Solingapuram Sai KK, Prabhakaran J, Hines J, Norman S, Rajagopal SA, Mintz A, et al. Synthesis and initial in vivo evaluations of [11C]WX-MB-18B, a microtubule imaging agent. Journal of Nuclear Medicine. 2019;60.
3076. Sollmann N, Schramm S, Ille S, Meyer B, Krieg S. Application of navigated transcranial magnetic stimulation to map the supplementary motor area in healthy subjects. Brain Stimulation. 2019;12(2):466. doi: 10.1016/j.brs.2018.12.518.
3077. Stilling JM, Duszynski CC, Oni I, Paxman E, Dunn JF, Debert CT. Using Functional Near-Infrared Spectroscopy to Study the Effect of Repetitive Transcranial Magnetic Stimulation in Concussion: A Two-Patient Case Study. Frontiers in Neurology. 2019;10. doi: 10.3389/fneur.2019.00476. PubMed PMID: WOS:000467346400001.
3078. Straudi S, Bonsangue V, Mele S, Craighero L, Montis A, Fregni F, et al. Bilateral M1 anodal transcranial direct current stimulation in post traumatic chronic minimally conscious state: a pilot EEG-tDCS study. Brain Inj. 2019;33(4):490-5. Epub 2019/01/12. doi: 10.1080/02699052.2019.1565894. PubMed PMID: 30632807.
3079. Straughn J, Denais Z. Application of Right Median Nerve Stimulation in an Inpatient Rehabilitation Setting for Disorders of Consciouness. Archives of physical medicine and rehabilitation.

2019;100(10):e44-. doi: 10.1016/j.apmr.2019.08.115. PubMed PMID: CN-01987488.

3080. Stultz DJ, Osburn S, Burns T, Walton R, Pawlowska-Wajswol S. TMS treatment in a patient with seizures and refractory depression. Brain Stimulation. 2019;12(6):1603-4. doi: 10.1016/j.brs.2019.08.009.

3081. Stultz DJ, Thistlethwaite D, Voltin R, Osburn S, Walton R, Burns T, et al. Continued TMS Treatment After A Seizure. Brain Stimulation. 2019;12(4):e139. doi: 10.1016/j.brs.2019.03.056.

3082. Tanaka N, Tsutsumi R, Shirota Y, Shimizu T, Ohminami S, Terao Y, et al. Effects of L-DOPA on quadripulse magnetic stimulation-induced long-term potentiation in older adults. Neurobiology of Aging. 2019;84:217-24. doi: 10.1016/j.neurobiolaging.2019.08.005. PubMed PMID: WOS:000501576800024.

3083. Tang-Schomer MD, Kaplan DL, Whalen MJ. Film interface for drug testing for delivery to cells in culture and in the brain. Acta Biomater. 2019;94:306-19. Epub 2019/03/06. doi: 10.1016/j.actbio.2019.02.052. PubMed PMID: 30836199; PubMed Central PMCID: PMCPMC6642835.

3084. Tay EMY, Preisz P, Day RO. Role and impact of brain computed tomography in the management of drug overdoses and guideline recommendations. EMA - Emergency Medicine Australasia.

2019;31(6):1053-8. doi: 10.1111/1742-6723.13316.

3085. Thibaut A, Piarulli A, Martens G, Chatelle C, Laureys S. Effect of multichannel transcranial direct current stimulation to reduce hypertonia in individuals with prolonged disorders of consciousness: A randomized controlled pilot study. Ann Phys Rehabil Med. 2019;62(6):418-25. Epub 2019/07/10. doi: 10.1016/j.rehab.2019.05.009. PubMed PMID: 31283989.

3086. Thibaut A, Schiff N, Giacino J, Laureys S, Gosseries O. Therapeutic interventions in patients with prolonged disorders of consciousness. Lancet Neurol. 2019;18(6):600-14. Epub 2019/04/21. doi: 10.1016/s1474-4422(19)30031-6. PubMed PMID: 31003899.

3087. Toccaceli G, Barbagallo G, Peschillo S. Low-intensity focused ultrasound for the treatment of brain diseases: Safety and feasibility. Theranostics. 2019;9(2):537-9. doi: 10.7150/thno.31765.

3088. Trakimas DR, Knoll RM, Ishai R, Lee DJ, Jung DH, Nadol JB, Jr., et al. Otopathology of Unilateral Cochlear Implantation in Patients With Bilateral Temporal Bone Fracture. Otol Neurotol. 2019;40(1):e14-e9. Epub 2018/12/12. doi: 10.1097/mao.0000000000002057. PubMed PMID: 30531637; PubMed Central PMCID: PMCPMC6939457.

3089. Tyler M, Skinner K, Prabhakaran V. Long-term treatment trial of the portable neuromodulation stimulator (PoNSTM) for treatment

of chronic balance deficit due to mild-to-moderate traumatic brain injury (mmTBI). Annals of neurology. 2019;86:S80-S1. PubMed PMID: CN-02095964.

3090. Tyler M, Skinner K, Prabhakaran V. Neurorehabilitation Using the Portable Neuromodulation Stimulator (PoNS (R)) for Treating Patients with Chronic Balance Deficit Due to Mild-to-Moderate Traumatic Brain Injury (mTBI): A 26-Week Study. Brain Injury. 2019;33:228-9. PubMed PMID: WOS:000466897000464.

3091. Tyler M, Skinner K, Prabhakaran V, Kaczmarek K, Danilov Y. Translingual Neurostimulation for the Treatment of Chronic Symptoms Due to Mild-to-Moderate Traumatic Brain Injury. Arch Rehabil Res Clin Transl. 2019;1(3-4):100026. Epub 2019/09/27. doi: 10.1016/j.arrct.2019.100026. PubMed PMID: 33543056; PubMed Central PMCID: PMCPMC7853385.

3092. Uchiyama M, Park G, Monsanto RC, Paparella MM, Cureoglu S. Pathologic changes of inner ear following head trauma. Otolaryngology - Head and Neck Surgery. 2019;161(2):P255-P6. doi: 10.1177/0194599819858142.

3093. Uchiyama T, Fukawa N, Kato A. Long term follow up of intrathecal baclofen therapy for spastic and dystonic patients. Neuromodulation. 2019;22(7):e583. doi: 10.1111/ner.12958.

3094. Verley DR, Torolira D, Hessell BA, Sutton RL, Harris NG.

Cortical Neuromodulation of Remote Regions after Experimental Traumatic Brain Injury Normalizes Forelimb Function but is Temporally Dependent. J Neurotrauma. 2019;36(5):789-801. Epub 2018/07/18. doi: 10.1089/neu.2018.5769. PubMed PMID: 30014759; PubMed Central PMCID: PMCPMC6387565.

3095. Vidal AC, Sequeira A, Costa C, Pinto S. Neuromodulation and physiotherapy: State of art. Annals of Medicine. 2019;51:S33-S4. doi: 10.1080/07853890.2018.1560076.

3096. Vidal AC, Sequeira A, Costa C, Pinto S. Music as an acoustic neuromodulation tool in physiotherapy post-stroke: A case study. Annals of Medicine. 2019;51:S223. doi: 10.1080/07853890.2018.1560735.

3097. Violante I, Li L, Sharp D. Theta-tACS normalizes brain network activity in patients with traumatic brain injury. Brain Stimulation. 2019;12(2):498. doi: 10.1016/j.brs.2018.12.629.

3098. Walker LAS, Lindsay-Brown AP, Berard JA. Cognitive Fatigability Interventions in Neurological Conditions: A Systematic Review. Neurol Ther. 2019;8(2):251-71. Epub 2019/10/06. doi: 10.1007/s40120-019-00158-3. PubMed PMID: 31586303; PubMed Central PMCID: PMCPMC6858900.

3099. Wang L, Zhang Y, Wang X, Ye Z. Electroacupuncture-induced cannabinoid receptor expression in repair of abducens nerve. Int J

Neurosci. 2019;129(9):923-9. Epub 2019/03/20. doi: 10.1080/00207454.2019.1593980. PubMed PMID: 30889365.

3100. Wang N, Ke Y, Du J, Liu W, Kong L, Zhao X, et al., editors. High-Definition Transcranial Direct Current Stimulation (HD-tDCS) enhances working memory training. Proceedings of the Annual International Conference of the IEEE Engineering in Medicine and Biology Society, EMBS; 2019.

3101. Wang P, Zhang J, Yu J, Smith C, Feng W. Brain Modulatory Effects by Low-Intensity Transcranial Ultrasound Stimulation (TUS): A Systematic Review on Both Animal and Human Studies. Front Neurosci. 2019;13:696. Epub 2019/08/10. doi: 10.3389/fnins.2019.00696. PubMed PMID: 31396029; PubMed Central PMCID: PMCPMC6667677.

3102. Wang W, Xu Y. Neuromodulation in minimally conscious state. Neuromodulation. 2019;22(7):e337. doi: 10.1111/ner.12958.

3103. Wang WT, Sun L, Sun CH. PDIA3-regulted inflammation and oxidative stress contribute to the traumatic brain injury (TBI) in mice. Biochemical and Biophysical Research Communications. 2019;518(4):657-63. doi: 10.1016/j.bbrc.2019.08.100.

3104. Wang Y, Sui G, Teng D, Wang Q, Qu J, Zhu L, et al. Low intensity focused ultrasound (LIFU) triggered drug release from cetuximab-conjugated phase-changeable nanoparticles for precision

theranostics against anaplastic thyroid carcinoma. Biomaterials Science. 2019;7(1):196-210. doi: 10.1039/c8bm00970h. PubMed PMID: WOS:000454085700012.
3105. Weightman M, Brittain JS, Jenkinson N. Differentiating the roles of the cerebellum and motor cortex during visuomotor adaptation using either hand or whole arm reaching movements. Brain and Neuroscience Advances. 2019;3:134-5. doi: 10.1177/2398212819855490.
3106. Willms M, Brucar L, Muller A, Vila-Rodrigues F, Rosenblatt C, Babul NV. Exploring tDCS-induced changes in EEG power and network connectivity in youth concussion: preliminary findings. Brain stimulation. 2019;12(2):468-. doi: 10.1016/j.brs.2018.12.525. PubMed PMID: CN-01788611.
3107. Wilson RL, François M, Jankovic-Karasoulos T, McAninch D, McCullough D, Leifert WR, et al. Characterization of 5-methylcytosine and 5-hydroxymethylcytosine in human placenta cell types across gestation. Epigenetics. 2019;14(7):660-71. doi: 10.1080/15592294.2019.1609866.
3108. Wu M, Yu Y, Luo L, Wu Y, Gao J, Ye X, et al. Efficiency of Repetitive Transcranial Direct Current Stimulation of the Dorsolateral Prefrontal Cortex in Disorders of Consciousness: A Randomized Sham-Controlled Study. Neural Plast. 2019;2019:7089543. Epub

2019/07/17. doi: 10.1155/2019/7089543. PubMed PMID: 31308848; PubMed Central PMCID: PMCPMC6594311.

3109. Wu X, Sun NN, Lü MH, Su SH, Wang DH, Zhang SJ, et al. [Effect of different courses of electroacupuncture intervention on recognition memory and proliferation and differentiation of hippocampal neural stem cells in mice with radiation-induced brain injury]. Zhen Ci Yan Jiu. 2019;44(11):787-92. Epub 2019/11/30. doi: 10.13702/j.1000-0607.190017. PubMed PMID: 31777226.

3110. Xia X, Wang Y, Li C, Li X, He J, Bai Y. Transcranial magnetic stimulation-evoked connectivity reveals modulation effects of repetitive transcranial magnetic stimulation on patients with disorders of consciousness. Neuroreport. 2019;30(18):1307-15. Epub 2019/11/13. doi: 10.1097/wnr.0000000000001362. PubMed PMID: 31714484.

3111. Xu J, Branscheidt M, Schambra H, Steiner L, Widmer M, Diedrichsen J, et al. Rethinking interhemispheric imbalance as a target for stroke neurorehabilitation. Annals of Neurology. 2019;85(4):502-13. doi: 10.1002/ana.25452.

3112. Xu Y, Li P, Zhang S, Wang Y, Zhao X, Wang X, et al. Cervical Spinal Cord Stimulation for the Vegetative State: A Preliminary Result of 12 Cases. Neuromodulation. 2019;22(3):347-54. doi: 10.1111/ner.12903.

3113. Yang H, Wu X, Zhou Z, Chen X, Kong M. Enhanced transdermal lymphatic delivery of doxorubicin via hyaluronic acid based transfersomes/microneedle complex for tumor metastasis therapy. International Journal of Biological Macromolecules. 2019;125:9-16. doi: 10.1016/j.ijbiomac.2018.11.230. PubMed PMID: WOS:000458222000002.

3114. Yang J, Shang B, Shi H, Zhu S, Lu G, Dai F. The role of toll-like receptor 4 and mast cell in the ameliorating effect of electroacupuncture on visceral hypersensitivity in rats. Neurogastroenterol Motil. 2019;31(6):e13583. Epub 2019/03/28. doi: 10.1111/nmo.13583. PubMed PMID: 30916854.

3115. Yang Z, Zhang Y, Sun S. Deciphering the SUMO code in the kidney. J Cell Mol Med. 2019;23(2):711-9. Epub 2018/12/07. doi: 10.1111/jcmm.14021. PubMed PMID: 30506859; PubMed Central PMCID: PMCPMC6349152.

3116. Ye L, Zhang D, Shao M, Zhao P, Yin B, Zhuang J, et al. Lower posttraumatic α-synuclein level associated with altered default mode network connectivity following acute mild traumatic brain injury. Frontiers in Neural Circuits. 2019;13. doi: 10.3389/fncir.2019.00026.

3117. Yong Y, Guo J, Zheng D, Li Y, Chen W, Wang J, et al. Electroacupuncture pretreatment attenuates brain injury in a mouse

model of cardiac arrest and cardiopulmonary resuscitation via the AKT/eNOS pathway. Life Sci. 2019;235:116821. Epub 2019/09/03. doi: 10.1016/j.lfs.2019.116821. PubMed PMID: 31476306.

3118. Yu Y, Yang Y, Gan S, Guo S, Fang J, Wang S, et al. Auditory-function-preserved patients with disorders of consciousness are reversible by transcutaneous auricular vagus nerve stimulation: A pilot study monitored by ASL fMRI. Brain Stimulation. 2019;12(2):388-9. doi: 10.1016/j.brs.2018.12.245.

3119. Zaninotto AL, El-Hagrassy MM, Green JR, Babo M, Paglioni VM, Benute GG, et al. Transcranial direct current stimulation (tDCS) effects on traumatic brain injury (TBI) recovery: A systematic review. Dement Neuropsychol. 2019;13(2):172-9. Epub 2019/07/10. doi: 10.1590/1980-57642018dn13-020005. PubMed PMID: 31285791; PubMed Central PMCID: PMCPMC6601308.

3120. Zewdie E, Ciechanski P, Kuo H, Giuffre A, Kahl C, King R, et al. Safety and tolerability of non-invasive neurostimulation in children. Brain Stimulation. 2019;12(2):550. doi: 10.1016/j.brs.2018.12.817.

3121. Zhang J, Shi C, Wang H, Gao C, Chang P, Chen X, et al. Hydrogen sulfide protects against cell damage through modulation of PI3K/Akt/Nrf2 signaling. International Journal of Biochemistry and Cell Biology. 2019;117. doi: 10.1016/j.biocel.2019.105636.

3122. Zhang X, Cheng J, Zeng J, Hong J, Chen F, Li M, et al.

Predicting role of disturbance coefficient in post brain traumatic hydrocephalus. Chinese Journal of Neurosurgery. 2019;35(2):153-6. PubMed PMID: CSCD:6438369.

3123. Zhang Y, Chai D, Gao M, Xu B, Jiang G. Thermal ablation of separable microneedles for transdermal delivery of metformin on diabetic rats. International Journal of Polymeric Materials and Polymeric Biomaterials. 2019;68(14):850-8. doi: 10.1080/00914037.2018.1517347. PubMed PMID: WOS:000468504100006.

3124. Zhang Y, Wu S, Xie L, Yu S, Zhang L, Liu C, et al. Ketamine within clinically effective range inhibits glutamate transmission from astrocytes to neurons and disrupts synchronization of astrocytic SICs. Frontiers in Cellular Neuroscience. 2019;13. doi: 10.3389/fncel.2019.00240. PubMed Central PMCID: PMCFujian Gutian(China)
Jiangsu Nhwa(China)
Astra Zeneca(United States).

3125. Zheng H, Zhang Z, Jiang S, Yan B, Shi X, Xie Y, et al. A shape-memory and spiral light-emitting device for precise multisite stimulation of nerve bundles. Nature Communications. 2019;10(1). doi: 10.1038/s41467-019-10418-3.

3126. Zhong S, Zhang J, Zhang L, Ye X. Rencent advance in

transcranial direct current stimulation in rehabilitation of patients with disorder of consciousness. Chinese Journal of Neuromedicine. 2019;18(3):297-301. PubMed PMID: CSCD:6470883.

3127. Zhong Y, Zhang Y, Xu J, Zhou J, Liu J, Ye M, et al. Low-Intensity Focused Ultrasound-Responsive Phase-Transitional Nanoparticles for Thrombolysis without Vascular Damage: A Synergistic Nonpharmaceutical Strategy. Acs Nano. 2019;13(3):3387-403. doi: 10.1021/acsnano.8b09277. PubMed PMID: WOS:000462950500065.

3128. Ziliotto N, Marchetti G, Straudi S, Tisato V, Lavezzi S, Manfredini F, et al. Soluble neural cell adhesion molecule and behavioural recovery in minimally conscious patients undergoing transcranial direct current stimulation. Clin Chim Acta. 2019;495:374-6. Epub 2019/05/12. doi: 10.1016/j.cca.2019.05.008. PubMed PMID: 31077650.

3129. Zurawski E, Behm K, Dunlap C, Koo J, Ismail F, Boulias C, et al. Interrater Reliability of the Modified Ashworth Scale with Standardized Movement Speeds: A Pilot Study. Physiotherapy Canada. 2019;71(4):348-54. doi: 10.3138/ptc-2018-0086. PubMed PMID: WOS:000501336200007.

3130. 40th International Symposium on Intensive Care and Emergency Medicine. Critical Care. 2020;24.

3131. Actrn. Effect of strong magnetic fields on the communication between brain and arm after spinal cord injury. https://trialsearchwhoint/Trial2aspx?TrialID=ACTRN12620000746932. 2020. PubMed PMID: CN-02165428.
3132. Actrn. Effect of individualised transcranial magnetic stimulation (TMS) on cognitive functioning in healthy volunteers. https://trialsearchwhoint/Trial2aspx?TrialID=ACTRN12620000162910. 2020. PubMed PMID: CN-02165087.
3133. Adamson M, McNerney MW, Siddiqi S, Swaminath G, Wu LL, Hernandez B, et al. Default Mode Network Connectivity and Brain Derived Trophic Factor in Brain Stimulation of TBI patients. Biological psychiatry. 2020;87(9):S158-. doi: 10.1016/j.biopsych.2020.02.419. PubMed PMID: CN-02120338.
3134. Ahmed M, Balse A, Diep M, Amin H, Haque S. Oculomotor Dysfunction Improvement in Moderate TBI Patients With Repetitive Transcranial Magnetic Stimulation (rTMS). Journal of Head Trauma Rehabilitation. 2020;35(2):E228-E. PubMed PMID: WOS:000525012500164.
3135. Ahn HJ, Bashir S, Martin P, Naeser MA, Pascual-Leone A, Yoo WK. cTBS effect on language reorganization verified by fMRI and chronometry TMS in an aphasia patient. Neurology and Clinical Neuroscience. 2020;8(2):74-8. doi: 10.1111/ncn3.12353.

3136. Al-Hussainy ADO, Al-Gazally ME, Ewadh W. Oxidative stress, Anti-melanocyte and anti tyrosinase antibody in vitiligo and response to treatment. Annals of Tropical Medicine and Public Health. 2020;23(5):500-7. doi: 10.36295/ASRO.2020.23524.
3137. Amer M, Chen RK. Self-Adhesive Microneedles with Interlocking Features for Sustained Ocular Drug Delivery. Macromolecular Bioscience. 2020;20(6). doi: 10.1002/mabi.202000089. PubMed PMID: WOS:000542595000003.
3138. Anderson J, Parr NJ, Vela K. VA Evidence-based Synthesis Program Reports. Evidence Brief: Transcranial Magnetic Stimulation (TMS) for Chronic Pain, PTSD, TBI, Opioid Addiction, and Sexual Trauma. Washington (DC): Department of Veterans Affairs (US); 2020.
3139. Angkawinitwong U, Courtenay AJ, Rodgers AM, Larraneta E, McCarthy HO, Brocchini S, et al. A Novel Transdermal Protein Delivery Strategy via Electrohydrodynamic Coating of PLGA Microparticles onto Microneedles. Acs Applied Materials & Interfaces. 2020;12(11):12478-88. doi: 10.1021/acsami.9b22425. PubMed PMID: WOS:000526543400010.
3140. Arnsten AFT. Guanfacine's mechanism of action in treating prefrontal cortical disorders: Successful translation across species. Neurobiology of Learning and Memory. 2020;176. doi:

10.1016/j.nlm.2020.107327.
3141. Assecondi S, Hu R, Eskes G, Read M, Griffiths C, Shapiro K. BRAINSTORMING: A study protocol for a randomised double-blind clinical trial to assess the impact of concurrent brain stimulation (tDCS) and working memory training on cognitive performance in Acquired Brain Injury (ABI). BMC Psychol. 2020;8(1):125. Epub 2020/11/28. doi: 10.1186/s40359-020-00454-w. PubMed PMID: 33243286; PubMed Central PMCID: PMCPMC7694939.
3142. Ayache SS, Chalah MA. Fatigue and affective manifestations in multiple sclerosis—a cluster approach. Brain Sciences. 2020;10(1). doi: 10.3390/brainsci10010010.
3143. Badran BW, Jenkins DD, Cook D, Thompson S, Dancy M, DeVries WH, et al. Transcutaneous Auricular Vagus Nerve Stimulation-Paired Rehabilitation for Oromotor Feeding Problems in Newborns: An Open-Label Pilot Study. Frontiers in Human Neuroscience. 2020;14. doi: 10.3389/fnhum.2020.00077. PubMed PMID: WOS:000525569400001.
3144. Bagnato S, Galardi G, Ribaudo F, Boccagni C, Fiorilla TV, Rubino F, et al. Serum BDNF Levels Are Reduced in Patients with Disorders of Consciousness and Are Not Modified by Verticalization with Robot-Assisted Lower-Limb Training. Neural Plasticity. 2020;2020. doi: 10.1155/2020/5608145.

3145. Bai S, Liao J, Zhang B, Zhao M, You B, Li P, et al. Multimodal and multifunctional nanoparticles with platelet targeting ability and phase transition efficiency for the molecular imaging and thrombolysis of coronary microthrombi. Biomaterials Science. 2020;8(18):5047-60. doi: 10.1039/d0bm00818d. PubMed PMID: WOS:000569877500008.

3146. Baik SW, Kim GW, Ko MH, Seo JH, Won YH, Park SH. An Unusual, Intermediate-Sized Lesion Affecting Motor Organization in a Patient With Schizencephaly: A Case Report. Frontiers in Human Neuroscience. 2020;14. doi: 10.3389/fnhum.2020.00258.

3147. Bakulin I, Legostaeva L, Poydasheva A, Iazeva E, Sinitsyn D, Sergeev D, et al. TMS-EEG analysis of rTMS efficacy in chronic disorders of consciousness. Clinical Neurophysiology. 2020;131(4):e107. doi: 10.1016/j.clinph.2019.12.276.

3148. Balcik ZE, Senadim S, Keskek A, Ozudogru A, Koksal A, Soysal A, et al. Does restricted diffusion in the splenium indicate an acute infarct? Acta Neurologica Belgica. 2020;120(5):1085-9. doi: 10.1007/s13760-017-0876-6.

3149. Bao SC, Khan A, Song R, Tong RKY. Rewiring the lesioned brain: Electrical stimulation for post-stroke motor restoration. Journal of Stroke. 2020;22(1):47-63. doi: 10.5853/jos.2019.03027.

3150. Bapna A, Adin C, Engelman ZJ, Fudim M. Increasing Blood

Pressure by Greater Splanchnic Nerve Stimulation: a Feasibility Study. Journal of Cardiovascular Translational Research. 2020;13(4):509-18. doi: 10.1007/s12265-019-09929-7. PubMed PMID: WOS:000494380000001.

3151. Beedham W, Belli A, Ingaralingam S, Haque S, Upthegrove R. The management of depression following traumatic brain injury: A systematic review with meta-analysis. Brain Inj. 2020;34(10):1287-304. Epub 2020/08/19. doi: 10.1080/02699052.2020.1797169. PubMed PMID: 32809854.

3152. Begemann MJ, Brand BA, Ćurčić-Blake B, Aleman A, Sommer IE. Efficacy of non-invasive brain stimulation on cognitive functioning in brain disorders: a meta-analysis. Psychol Med. 2020;50(15):2465-86. Epub 2020/10/20. doi: 10.1017/s0033291720003670. PubMed PMID: 33070785; PubMed Central PMCID: PMCPMC7737055.

3153. Bender Pape TL, Herrold AA, Guernon A, Aaronson A, Rosenow JM. Neuromodulatory Interventions for Traumatic Brain Injury. J Head Trauma Rehabil. 2020;35(6):365-70. Epub 2020/11/10. doi: 10.1097/htr.0000000000000643. PubMed PMID: 33165150.

3154. Bernini A, Masoodi M, Solari D, Miroz JP, Carteron L, Christinat N, et al. Modulation of cerebral ketone metabolism following traumatic brain injury in humans. Journal of Cerebral Blood

Flow and Metabolism. 2020;40(1):177-86. doi: 10.1177/0271678X18808947.

3155. Biagi MC, Salvador R, Thibaut A, Martens G, Laureys S, Santarnecchi E, et al. Targeting consciousness brain networks with transcranial current stimulation in minimally conscious state patients: what we gain from a personalized intervention. Clinical Neurophysiology. 2020;131(4):e33-e5. doi: 10.1016/j.clinph.2019.12.150.

3156. Billeri L, Filoni S, Russo EF, Portaro S, Militi D, Calabro RS, et al. Toward Improving Diagnostic Strategies in Chronic Disorders of Consciousness: An Overview on the (Re-)Emergent Role of Neurophysiology. Brain Sciences. 2020;10(1). doi: 10.3390/brainsci10010042. PubMed PMID: WOS:000513130500027.

3157. Bolu A, Gündoğmuş I, Öznur T, Çelik C. Treatment of insomnia with repetitive transcranial magnetic stimulation (Rtms) in a patient with posttraumatic stress disorder (ptsd). Revista de Psiquiatria Clinica. 2020;47(5):157-8. doi: 10.1590/0101-60830000000252.

3158. Bragina OA, Semyachkina-Glushkovskaya OV, Nemoto EM, Bragin DE. Anodal Transcranial Direct Current Stimulation Improves Impaired Cerebrovascular Reactivity in Traumatized Mouse Brain. Adv Exp Med Biol. 2020;1232:47-53. Epub 2020/01/02. doi: 10.1007/978-3-030-34461-0_7. PubMed PMID: 31893393; PubMed

Central PMCID: PMCPMC7307636.
3159. Briand MM, Gosseries O, Staumont B, Laureys S, Thibaut A. Transcutaneous Auricular Vagal Nerve Stimulation and Disorders of Consciousness: A Hypothesis for Mechanisms of Action. Front Neurol. 2020;11:933. Epub 2020/09/29. doi: 10.3389/fneur.2020.00933. PubMed PMID: 32982941; PubMed Central PMCID: PMCPMC7477388.
3160. Buday J, Albrecht J, Podgorná G, Mareš T, Le TH, Čapek V, et al. Seizure threshold manipulation in electroconvulsive therapy via repetitive transcranial magnetic stimulation. A novel way of augmentation? Brain Stimulation. 2020;13(6):1631-8. doi: 10.1016/j.brs.2020.09.008.
3161. Buhagiar F, Fitzgerald M, Bell J, Allanson F, Pestell C. Neuromodulation for Mild Traumatic Brain Injury Rehabilitation: A Systematic Review. Front Hum Neurosci. 2020;14:598208. Epub 2020/12/29. doi: 10.3389/fnhum.2020.598208. PubMed PMID: 33362494; PubMed Central PMCID: PMCPMC7759622.
3162. Bulygin KV, Beeraka NM, Saitgareeva AR, Nikolenko VN, Gareev I, Beylerli O, et al. Can mirnas be considered as diagnostic and therapeutic molecules in ischemic stroke pathogenesis?—current status. International Journal of Molecular Sciences. 2020;21(18):1-25. doi: 10.3390/ijms21186728.

3163. Burhan AM, Patience JA, Teselink JGP, Marlatt NM, Babapoor-Farrokhran S, Palaniyappan L. Bilateral sequential theta burst stimulation for multiple-therapy-resistant depression: A naturalistic observation study. J Psychiatr Res. 2020;130:342-6. Epub 2020/09/04. doi: 10.1016/j.jpsychires.2020.08.009. PubMed PMID: 32882575.

3164. Campanella S, Schroder E, Vanderhasselt MA, Monnart A, Rabbijns M, Kornreich C, et al. Training inhibition through neuromodulation: Combined evidence from tdcs and erps through a go-nogo task in a healthy population. Clinical EEG and Neuroscience. 2020;51(3):NP16. doi: 10.1177/1550059415622686.

3165. Campos R, Gomez R, Velarde L. SACRAL NEUROMODULATION WITH INTERSTIM (R) FOR URINARY VOIDING DYSFUNCTION IN POST-TRAUMATIC BRAIN INJURY PATIENTS. Journal of Urology. 2020;203:E698-E. PubMed PMID: WOS:000527010303051.

3166. Campos R, Gomez R, Velarde L. Sacral neuromodulation with Interstim® for urinary voiding dysfunction in post-traumatic brain injury patients. Journal of Urology. 2020;203:e698.

3167. Cantarero G, Choynowski J, St Pierre M, Anaya M, Statton M, Stokes W, et al. Repeated Concussions Impair Behavioral and Neurophysiological Changes in the Motor Learning System.

Neurorehabil Neural Repair. 2020;34(9):804-13. Epub 2020/07/30. doi: 10.1177/1545968320943578. PubMed PMID: 32723160; PubMed Central PMCID: PMCPMC7501144.

3168. Carriere M, Barra A, Mortaheb S, Binda Fossati M, Martens G, Bodien Y, et al. Neurophysiological effects and behavioral outcomes after tPCS and tDCS in a patient in minimally conscious state. Clinical neurophysiology. 2020;131(4):e116-e7. doi: 10.1016/j.clinph.2019.12.292. PubMed PMID: CN-02096994.

3169. Carrière M, Mortaheb S, Raimondo F, Annen J, Barra A, Binda Fossati MC, et al. Neurophysiological Correlates of a Single Session of Prefrontal tDCS in Patients with Prolonged Disorders of Consciousness: A Pilot Double-Blind Randomized Controlled Study. Brain Sci. 2020;10(7). Epub 2020/07/28. doi: 10.3390/brainsci10070469. PubMed PMID: 32708119; PubMed Central PMCID: PMCPMC7408434.

3170. Cassidy JM, Wodeyar A, Wu J, Kaur K, Masuda AK, Srinivasan R, et al. Low-Frequency Oscillations Are a Biomarker of Injury and Recovery after Stroke. Stroke. 2020:1442-50. doi: 10.1161/STROKEAHA.120.028932.

3171. Castricum J, Tulen JHM, Taal W, Ottenhoff MJ, Kushner SA, Elgersma Y. Motor cortical excitability and plasticity in patients with neurofibromatosis type 1. Clinical Neurophysiology.

2020;131(11):2673-81. doi: 10.1016/j.clinph.2020.08.016.

3172. Chae J-S, Yuwon C, Kwon KM. The Effects of Proprioceptive Exercise Combined with Cognitive Task on the Balance and Ankle Function of Chronic Ankle Instability Adult. Journal of the Korean Society of Physical Medicine. 2020;15(1):65-76. doi: 10.13066/kspm.2020.15.1.65. PubMed PMID: KJD:ART002564681.

3173. Channagiri V, Aziz D, Iannaccone F, Kaufman A. Percutaneous peripheral nerve stimulation for treatment of brachial plexopathy secondary to pancoast tumor: A case report. Interventional Pain Management Reports. 2020;4(5):169-74. doi: 10.36076/PMCR.2020/4/169.

3174. Chapman K, Van Roosendaal B, Schrippa F. Unilateral dorsal root ganglion stimulator (drg-s) lead placement with resolution of bilateral lower extremity symptoms in peripheral diabetic neuropathy: A case report. Pain Practice. 2020;20(SUPPL 1):42. doi: 10.1111/papr.12886.

3175. Chen G, Cao Y, Tang Y, Yang X, Liu Y, Huang D, et al. Advanced Near-Infrared Light for Monitoring and Modulating the Spatiotemporal Dynamics of Cell Functions in Living Systems. Advanced Science. 2020;7(8). doi: 10.1002/advs.201903783. PubMed PMID: WOS:000516660100001.

3176. Chen J, Wang Y. Personalized dynamic transport of magnetic

nanorobots inside the brain vasculature. Nanotechnology. 2020;31(49). doi: 10.1088/1361-6528/abb392. PubMed PMID: WOS:000576444600001.

3177. Chiabi A, Djimafo ANM, Nguefack S, Mah E, Nguefack Dongmo F, Angwafo F, III. Severe malaria in Cameroon: Pattern of disease in children at the Yaounde Gynaeco-Obstetric and Pediatric hospital. Journal of Infection and Public Health. 2020;13(10):1469-72. doi: 10.1016/j.jiph.2020.02.038.

3178. Choi KJ, Nam GB, Zo JH, Lee JH, Cho MS, Kim J, et al. Comparison catheter ablation versus permanent pacemaker implantation in patients with tachycardia-bradycardia syndrome: prospective, randomized trial. European heart journal. 2020;41(SUPPL 2):712-. doi: 10.1093/ehjci/ehaa946.0712. PubMed PMID: CN-02243003.

3179. Chowdhury FA, Caciagli L, Whatley BP, McLaughlin C, Sanders B, Wehner T, et al. Preoperative language mapping using navigated TMS compared with extra-operative direct cortical stimulation using intracranial electrodes: A case report. Seizure. 2020;76:96-9. doi: 10.1016/j.seizure.2020.01.019.

3180. Chu S, Uplekar N, Liu S, Ghodssi R. Complementary Capillary System Integrated Microneedles for Autonomously Localized Therapeutics Loading. Journal of Microelectromechanical

Systems. 2020;29(5):912-7. doi: 10.1109/jmems.2020.2999255. PubMed PMID: WOS:000576466500043.

3181. Conti A, Kamimura HAS, Novell A, Duggento A, Toschi N. Magnetic Resonance Methods for Focused Ultrasound-Induced Blood-Brain Barrier Opening. Frontiers in Physics. 2020;8. doi: 10.3389/fphy.2020.547674. PubMed PMID: WOS:000579081100001.

3182. Cotner B, Ching D, Nakase-Richardson R, Murphy J, Martin A, Takagishi SC, et al. An Environmental Scan to Identify Current Chronic Pain Treatment Practices for Persons with Traumatic Brain Injury. Archives of Physical Medicine and Rehabilitation. 2020;101(11):e115. doi: 10.1016/j.apmr.2020.09.350.

3183. Cotovio G, Talmasov D, Barahona-Corrêa JB, Hsu J, Senova S, Ribeiro R, et al. Mapping mania symptoms based on focal brain damage. J Clin Invest. 2020;130(10):5209-22. Epub 2020/08/25. doi: 10.1172/jci136096. PubMed PMID: 32831292; PubMed Central PMCID: PMCPMC7524493.

3184. Ctri. Non-invasive Brain Stimulation to Improve Cognition In Traumatic Brain Injury Patients. https://trialsearchwhoint/Trial2aspx?TrialID=CTRI/2020/04/024781. 2020. PubMed PMID: CN-02167794.

3185. Ctri. Effect of direct current stimulation of brain on altered consciousness patients.

https://trialsearchwhoint/Trial2aspx?TrialID=CTRI/2020/07/026553. 2020. PubMed PMID: CN-02185320.

3186. D'Arcy RCN, Greene T, Greene D, Frehlick Z, Fickling SD, Campbell N, et al. Portable neuromodulation induces neuroplasticity to re-activate motor function recovery from brain injury: a high-density MEG case study. J Neuroeng Rehabil. 2020;17(1):158. Epub 2020/12/03. doi: 10.1186/s12984-020-00772-5. PubMed PMID: 33261623; PubMed Central PMCID: PMCPMC7708191.

3187. de Almeida AC, Massote MA, Ichinose RM, Miranda de Sá AMFL. Spectral F Test for detecting TMS/EEG responses. Biomedical Signal Processing and Control. 2020;58. doi: 10.1016/j.bspc.2019.101840.

3188. de Tommaso M, Betti V, Bocci T, Bolognini N, Di Russo F, Fattapposta F, et al. Pearl and pitfalls in brain functional analysis by event-related potentials: a narrative review by the Italian Psychophysiology and Cognitive Neuroscience Society on methodological limits and clinical reliability-part II. Neurol Sci. 2020;41(12):3503-15. Epub 2020/07/20. doi: 10.1007/s10072-020-04527-x. PubMed PMID: 32683566.

3189. Delaney SL, Gendreau JL, D'Souza M, Feng AY, Ho AL. Optogenetic Modulation for the Treatment of Traumatic Brain Injury. Stem Cells Dev. 2020;29(4):187-97. Epub 2019/09/29. doi:

10.1089/scd.2019.0187. PubMed PMID: 31559914.

3190. Ding Y, Li H, Hong T, Yao P. Efficacy of Pulsed Radiofrequency to Cervical Nerve Root for Postherpetic Neuralgia in Upper Extremity. Front Neurosci. 2020;14:377. Epub 2020/05/07. doi: 10.3389/fnins.2020.00377. PubMed PMID: 32372914; PubMed Central PMCID: PMCPMC7186594.

3191. Dixon B, MacLeod DB. Assessment of a Non Invasive Brain Oximeter in Volunteers Undergoing Acute Hypoxia. Med Devices (Auckl). 2020;13:183-94. Epub 2020/07/17. doi: 10.2147/mder.S250102. PubMed PMID: 32669881; PubMed Central PMCID: PMCPMC7335769.

3192. Dombovy-Johnson ML, Hagedorn JM, Wilson RE, Canzanello NC, Pingree MJ, Watson JC. Spinal Cord Stimulation for Neuropathic Pain Treatment in Brachial Plexus Avulsions: A Literature Review and Report of Two Cases. Neuromodulation. 2020;23(5):704-12. Epub 2020/02/19. doi: 10.1111/ner.13128. PubMed PMID: 32069374.

3193. Duong YN, Kilgard M, Hays S, Rennaker R, Pruitt D, Meyers E, et al. Usability of RePlay: An Automated Upper Limb Rehabilitation System for Brain Injury Tele-rehabilitation. Archives of Physical Medicine and Rehabilitation. 2020;101(11):e104. doi: 10.1016/j.apmr.2020.09.318.

3194. Dwivedi P, Kiran S, Han S, Dwivedi M, Khatik R, Fan R, et al. Magnetic Targeting and Ultrasound Activation of Liposome-Microbubble Conjugate for Enhanced Delivery of Anticancer Therapies. Acs Applied Materials & Interfaces. 2020;12(21):23737-51. doi: 10.1021/acsami.0c05308. PubMed PMID: WOS:000537731900009.
3195. Dwyer B, Zasler N. Post-traumatic cephalalgia. Neurorehabilitation. 2020;47(3):327-42. doi: 10.3233/nre-208006. PubMed PMID: WOS:000592868600007.
3196. Edlow BL, Barra ME, Zhou DW, Foulkes AS, Snider SB, Threlkeld ZD, et al. Personalized Connectome Mapping to Guide Targeted Therapy and Promote Recovery of Consciousness in the Intensive Care Unit. Neurocrit Care. 2020;33(2):364-75. Epub 2020/08/15. doi: 10.1007/s12028-020-01062-7. PubMed PMID: 32794142; PubMed Central PMCID: PMCPMC8336723.
3197. Elias GJB, De Vloo P, Germann J, Boutet A, Gramer RM, Joel SE, et al. Mapping the network underpinnings of central poststroke pain and analgesic neuromodulation. Pain. 2020;161(12):2805-19. Epub 2020/07/23. doi: 10.1097/j.pain.0000000000001998. PubMed PMID: 32694384.
3198. Ellison A, Dunne S, Lane AR. Real-world applications in vision and attention: How to help patients find their (golf) balls again.

In: Parkin BL, editor. Real-World Applications in Cognitive Neuroscience. Progress in Brain Research. 2532020. p. 169-200.
3199. Elmaadawi AZ, Robert Strawn J, Aaronson ST, Randolph Schrodt G, Holbert RC, Verdoliva S, et al. 24.3 HIGH-FREQUENCY TRANSCRANIAL MAGNETIC STIMULATION FOR TREATMENT-RESISTANT DEPRESSION IN ADOLESCENTS: THE FIRST MULTICENTER ACTIVE VS SHAM MULTICENTER TRIAL. Journal of the American Academy of Child and Adolescent Psychiatry. 2020;59(10):S199-S200. doi: 10.1016/j.jaac.2020.08.235. PubMed PMID: CN-02192226.
3200. Euctr DK. Aciclovir for treatment of meningitis caused by HSV-2 virus. https://trialsearchwhoint/Trial2aspx?TrialID=EUCTR2020-000033-41-DK. 2020. PubMed PMID: CN-02169561.
3201. Fang J-H, Liu C-H, Hsu R-S, Chen Y-Y, Chiang W-H, Wang H-MD, et al. Transdermal Composite Microneedle Composed of Mesoporous Iron Oxide Nanoraspberry and PVA for Androgenetic Alopecia Treatment. Polymers. 2020;12(6). doi: 10.3390/polym12061392. PubMed PMID: WOS:000550735500001.
3202. Fard MT, Mansouri SS, Jafari A, Vousooghi N. Role of modafinil in the treatment of patients with methamphetamine dependence; An update on randomized, controlled clinical trial.

Tropical Journal of Pharmaceutical Research. 2020;19(10):2179-85. doi: 10.4314/tjpr.v19i10.23.
3203. Feng Y, Zhang J, Zhou Y, Bai Z, Yin Y. Noninvasive brain stimulation for patients with a disorder of consciousness: a systematic review and meta-analysis. Rev Neurosci. 2020. Epub 2020/08/28. doi: 10.1515/revneuro-2020-0033. PubMed PMID: 32845870.
3204. Feng YH, Liu JL, Zhu DD, Hao YY, Guo XD. Multiscale simulations of drug distributions in polymer dissolvable microneedles. Colloids and Surfaces B-Biointerfaces. 2020;189. doi: 10.1016/j.colsurfb.2020.110844. PubMed PMID: WOS:000527314700027.
3205. Fickling SD, Greene T, Greene D, Frehlick Z, Campbell N, Etheridge T, et al. Brain Vital Signs Detect Cognitive Improvements During Combined Physical Therapy and Neuromodulation in Rehabilitation From Severe Traumatic Brain Injury: A Case Report. Front Hum Neurosci. 2020;14:347. Epub 2020/11/03. doi: 10.3389/fnhum.2020.00347. PubMed PMID: 33132868; PubMed Central PMCID: PMCPMC7513585.
3206. Fisher BE. Beyond Limits: Unmasking Potential Through Movement Discovery. Phys Ther. 2020;100(5):747-56. Epub 2020/04/28. doi: 10.1093/ptj/pzaa018. PubMed PMID: 32339243.
3207. Fong ZH, Sündermann O. Modulating disgust in mental

contamination: Experimental evidence for the role of disgust. Journal of Behavior Therapy and Experimental Psychiatry. 2020;68. doi: 10.1016/j.jbtep.2020.101567.
3208. Fornia L, Puglisi G, Leonetti A, Bello L, Berti A, Cerri G, et al. Direct electrical stimulation of the premotor cortex shuts down awareness of voluntary actions. Nature Communications. 2020;11(1). doi: 10.1038/s41467-020-14517-4.
3209. Foysal KMR, Baker SN. Induction of plasticity in the human motor system by motor imagery and transcranial magnetic stimulation. Journal of Physiology-London. 2020;598(12):2385-96. doi: 10.1113/jp279794. PubMed PMID: WOS:000531304600001.
3210. Franzini A, Messina G, Levi V, D'Ammando A, Cordella R, Moosa S, et al. Deep brain stimulation of the posterior limb of the internal capsule in the treatment of central poststroke neuropathic pain of the lower limb: case series with long-term follow-up and literature review. Journal of Neurosurgery. 2020;133(3):830-8. doi: 10.3171/2019.5.Jns19227. PubMed PMID: WOS:000586088300029.
3211. Fujiki M, Yee KM, Steward O. Non-invasive High Frequency Repetitive Transcranial Magnetic Stimulation (hfrTMS) Robustly Activates Molecular Pathways Implicated in Neuronal Growth and Synaptic Plasticity in Select Populations of Neurons. Frontiers in Neuroscience. 2020;14. doi: 10.3389/fnins.2020.00558. PubMed

PMID: WOS:000546087600001.
3212. Gandois L, Hoyt AM, Mounier S, Le Roux G, Harvey CF, Claustres A, et al. From canals to the coast: dissolved organic matter and trace metal composition in rivers draining degraded tropical peatlands in Indonesia. Biogeosciences. 2020;17(7):1897-909. doi: 10.5194/bg-17-1897-2020. PubMed PMID: WOS:000525367000001.
3213. Gao BY, Sun CC, Xia GH, Zhou ST, Zhang Y, Mao YR, et al. Paired associated magnetic stimulation promotes neural repair in the rat middle cerebral artery occlusion model of stroke. Neural Regeneration Research. 2020;15(11):2047-56. doi: 10.4103/1673-5374.282266.
3214. Gao F, Ostrem JL, Wang DD. Treatment of post-hypoxic myoclonus using pallidal deep brain stimulation placed using interventional MRI methods. Tremor and Other Hyperkinetic Movements. 2020;10:1-6. doi: 10.5334/tohm.544.
3215. Garland JS, Gbade-Alabi O, Taylor JA, Atwal N, Pasquina PF. A Study of Bilateral Prefrontal Repetitive Transcranial Magnetic Stimulation (rTMS) to Treat the Symptoms of Mild TBI (mTBI) and PTSD: preliminary Tolerability and Effectiveness. Brain stimulation. 2020;13(6):1848-. doi: 10.1016/j.brs.2020.06.037. PubMed PMID: CN-02212144.
3216. Garland JS, Jaskot EM, Taylor J, Buccellato K, Atwal N,

Pasquina PF. 135 Evolution of a Study of Bilateral Prefrontal Transcranial Magnetic Stimulation (TMS) to Treat the Symptoms of Mild TBI (mTBI) and PTSD. CNS spectrums. 2020;25(2):285-6. doi: 10.1017/S109285292000516. PubMed PMID: CN-02121330.

3217. Garland JS, Jaskot EM, Taylor J, Buccellato K, Atwal N, Pasquina PF. Evolution of a study of bilateral prefrontal transcranial magnetic stimulation (TMS) to treat the symptoms of mild TBI (mTBI) and PTSD. CNS spectrums. 2020;25(2):285-6. doi: 10.1017/S1092852920000188. PubMed PMID: CN-02121329.

3218. Giuffre A, Zewdie E, Kuo HC, Carlson H, Kirton A. The effects of conventional and high definition transcranial direct current stimulation on motor learning and robotic Transcranial Magnetic Stimulation (TMS) motor maps in children. Clinical Neurophysiology. 2020;131(4):e147. doi: 10.1016/j.clinph.2019.12.342.

3219. Giummarra MJ, Murgatroyd D, Tran Y, Adie S, Mittal R, Ponsford J, et al. Health and return to work in the first two years following road traffic injury: a comparison of outcomes between compensation claimants in Victoria and New South Wales, Australia. Injury. 2020;51(10):2199-208. doi: 10.1016/j.injury.2020.07.002.

3220. Gosseries O, Fecchio M, Wolff A, Sanz LRD, Sombrun C, Vanhaudenhuyse A, et al. Behavioural and brain responses in cognitive trance: A TMS-EEG case study. Clin Neurophysiol.

2020;131(2):586-8. Epub 2019/12/18. doi: 10.1016/j.clinph.2019.11.011. PubMed PMID: 31843502.

3221. Gruen DS, Brown JB, Guyette FX, Vodovotz Y, Johansson PI, Stensballe J, et al. Prehospital plasma is associated with distinct biomarker expression following injury. JCI Insight. 2020;5(8). doi: 10.1172/jci.insight.135350.

3222. Gu T, Wang RH, Wu T, Wang D, Yang Q, Yang Q, et al. [Effect of electroacupuncture on neuronal apoptosis in rats with traumatic brain injury based on PI3K/Akt signaling pathway]. Zhongguo Zhen Jiu. 2020;40(8):851-6. Epub 2020/09/02. doi: 10.13703/j.0255-2930.20191217-k0004. PubMed PMID: 32869594.

3223. Gu T, Wang X, Yang H, She XN, Chen KH, Wu T, et al. [Impacts of electroacupuncture on neurological function and protein expressions of apoptosis-related Cyt-C and Caspase-9 in rats with traumatic brain injury]. Zhongguo Zhen Jiu. 2020;40(7):749-55. Epub 2020/07/11. doi: 10.13703/j.0255-2930.20190521-0001. PubMed PMID: 32648400.

3224. Gu T, Wu T, Wang RH, Yang H, Ke ZH, Wang D, et al. [Effect of electroacupuncture on expression of corticocerebral autophagy-related proteins in rats with traumatic brain injury]. Zhen Ci Yan Jiu. 2020;45(7):524-8. Epub 2020/07/25. doi: 10.13702/j.1000-0607.190824. PubMed PMID: 32705824.

3225. Gudmundsson L, Vohryzek J, Fornari E, Clarke S, Hagmann P, Crottaz-Herbette S. A brief exposure to rightward prismatic adaptation changes resting-state network characteristics of the ventral attentional system. PLoS ONE. 2020;15(6). doi: 10.1371/journal.pone.0234382.
3226. Gutiérrez M, Feijóo G, Delucchi LJ. Neurophysiological evaluation of canine congenital hydrocephalus in three dogs. Veterinary Record Case Reports. 2020;8(1). doi: 10.1136/vetreccr-2019-000949.
3227. Haddad AF, Young JS, Berger MS, Tarapore PE. Preoperative Applications of Navigated Transcranial Magnetic Stimulation. Frontiers in Neurology. 2020;11. doi: 10.3389/fneur.2020.628903.
3228. Hakon J, Moghiseh M, Poulsen I, Øland CML, Hansen CP, Sabers A. Transcutaneous Vagus Nerve Stimulation in Patients With Severe Traumatic Brain Injury: A Feasibility Trial. Neuromodulation. 2020;23(6):859-64. Epub 2020/04/01. doi: 10.1111/ner.13148. PubMed PMID: 32227429.
3229. Hampstead BM, Bahar-Fuchs A. Neurophysiological mechanisms and outcomes of nonpharmacologic interventions for neurological disease or injury: Introduction to special issue. International Journal of Psychophysiology. 2020;154:1-2. doi: 10.1016/j.ijpsycho.2020.02.010. PubMed PMID: WOS:000540674600001.

3230. Han D, Morde RS, Mariani S, La Mattina AA, Vignali E, Yang C, et al. 4D Printing of a Bioinspired Microneedle Array with Backward-Facing Barbs for Enhanced Tissue Adhesion. Advanced Functional Materials. 2020;30(11). doi: 10.1002/adfm.201909197. PubMed PMID: WOS:000510696800001.
3231. Han JM, Hong KO, Yang IH, Ahn CH, Jin B, Lee W, et al. Oridonin induces the apoptosis of mucoepidermoid carcinoma cell lines in a myeloid cell leukemia-1-dependent manner. International Journal of Oncology. 2020;57(1):385-2020. doi: 10.3892/ijo.2020.5061.
3232. Handy JD, Wright WG, Haskell A, Servatius L, Servatius RJ. Enhanced Acquisition and Retention of Conditioned Eyeblink Responses in Veterans Expressing PTSD Symptoms: Modulation by Lifetime History of Mild Traumatic Brain Injury. Frontiers in Behavioral Neuroscience. 2020;14. doi: 10.3389/fnbeh.2020.595007.
3233. Harounian J, Berisha D, Mahmood A, Portenoy RK, Bikson M, Datta A, et al. TDCS-telehealth technology and protocol for successful TDCS administration by octogenarians for symptom control in home settings. Neuromodulation. 2020;23(5):e328-e30. doi: 10.1111/ner.13152.
3234. Harter K, Hammel G, Fleming M, Traidl-Hoffmann C. Multiple chemical sensitivity (MCS) – a guide for dermatologists on

how to manage affected individuals. JDDG - Journal of the German Society of Dermatology. 2020;18(2):119-30. doi: 10.1111/ddg.14027.
3235. He R, Fan J, Wang H, Zhong Y, Ma J. Differentiating Responders and Non-responders to rTMS Treatment for Disorder of Consciousness Using EEG After-Effects. Front Neurol. 2020;11:583268. Epub 2020/12/18. doi: 10.3389/fneur.2020.583268. PubMed PMID: 33329325; PubMed Central PMCID: PMCPMC7714935.
3236. Henderson TA, van Lierop MJ, McLean M, Uszler JM, Thornton JF, Siow Y-H, et al. Functional Neuroimaging in Psychiatry-Aiding in Diagnosis and Guiding Treatment. What the American Psychiatric Association Does Not Know. Frontiers in Psychiatry. 2020;11. doi: 10.3389/fpsyt.2020.00276. PubMed PMID: WOS:000530505100001.
3237. Hendriks T, Kunst HPM, Huppelschoten M, Doorduin J, Ter Laan M. TcMEP threshold change is superior to A-train detection when predicting facial nerve outcome in CPA tumour surgery. Acta Neurochir (Wien). 2020;162(5):1197-203. Epub 2020/03/09. doi: 10.1007/s00701-020-04275-z. PubMed PMID: 32146526; PubMed Central PMCID: PMCPMC7156349.
3238. Hermann B, Raimondo F, Hirsch L, Huang Y, Denis-Valente M, Pérez P, et al. Combined behavioral and electrophysiological

evidence for a direct cortical effect of prefrontal tDCS on disorders of consciousness. Sci Rep. 2020;10(1):4323. Epub 2020/03/11. doi: 10.1038/s41598-020-61180-2. PubMed PMID: 32152347; PubMed Central PMCID: PMCPMC7062738.

3239. Herrold AA, Siddiqi S, Parrish T, Cobia D, Chambers RA, Jordan N, et al. INTEGRATION OF CUE REACTIVITY TASK AND RESTING STATE MRI DATA TO DETERMINE TMS SITE OF STIMULATION FOR CO-OCCURRING AUD & MILD TBI. Alcoholism-Clinical and Experimental Research. 2020;44:71-. PubMed PMID: WOS:000540372300257.

3240. Herrold AA, Siddiqi SH, Livengood SL, Bender Pape TL, Higgins JP, Adamson MM, et al. Customizing TMS Applications in Traumatic Brain Injury Using Neuroimaging. J Head Trauma Rehabil. 2020;35(6):401-11. Epub 2020/11/10. doi: 10.1097/htr.0000000000000627. PubMed PMID: 33165153.

3241. Hill NM, Dewald JPA. The Upper Extremity Flexion Synergy Is Minimally Expressed in Young Individuals With Unilateral Cerebral Palsy Following an Early Brain Injury. Frontiers in Human Neuroscience. 2020;14. doi: 10.3389/fnhum.2020.590198.

3242. Hitti FL, Piazza M, Sinha S, Kvint S, Hudgins E, Baltuch G, et al. Surgical Outcomes in Post-Traumatic Epilepsy: A Single Institutional Experience. Oper Neurosurg (Hagerstown).

2020;18(1):12-8. Epub 2019/03/30. doi: 10.1093/ons/opz043. PubMed PMID: 30924499; PubMed Central PMCID: PMCPMC6911733.
3243. Hollis A, Zewdie E, Nettel-Aguirre A, Hilderley A, Kuo HC, Carlson HL, et al. Transcranial Static Magnetic Field Stimulation of the Motor Cortex in Children. Frontiers in Neuroscience. 2020;14. doi: 10.3389/fnins.2020.00464.
3244. Hou J, Nelson R, Mohammad N, Mustafa G, Plant D, Thompson FJ, et al. Effect of Simultaneous Combined Treadmill Training and Magnetic Stimulation on Spasticity and Gait Impairments after Cervical Spinal Cord Injury. Journal of Neurotrauma. 2020;37(18):1999-2013. doi: 10.1089/neu.2019.6961. PubMed PMID: WOS:000556431300001.
3245. Houshangi A, Jamehbozorgi S, Yousefi M, Ghiasi R. Preparation of CoFe2O4/sawdust and NiFe2O4/sawdust magnetic nanocomposites for removal of oil from the water surface. Journal of the Chinese Chemical Society. 2020;67(2):288-97. doi: 10.1002/jccs.201800452. PubMed PMID: WOS:000481905000001.
3246. Howard L, Schwedt TJ. Posttraumatic headache: recent progress. Curr Opin Neurol. 2020;33(3):316-22. Epub 2020/04/19. doi: 10.1097/wco.0000000000000815. PubMed PMID: 32304441.
3247. Hsu W-L, Huang C-Y, Hsu Y-P, Hwang T-L, Chang S-H, Wang H-YJ, et al. On-skin glucose-biosensing and on-demand

insulin-zinc hexamers delivery using microneedles for syringe-free diabetes management. Chemical Engineering Journal. 2020;398. doi: 10.1016/j.cej.2020.125536. PubMed PMID: WOS:000561588300007.

3248. Huang Y, Hu K, Green AL, Ma X, Gillies MJ, Wang S, et al. Dynamic changes in rhythmic and arrhythmic neural signatures in the subthalamic nucleus induced by anaesthesia and tracheal intubation. British Journal of Anaesthesia. 2020;125(1):67-76. doi: 10.1016/j.bja.2020.03.014.

3249. Hurtado-Puerto AM, Nestor K, Eldaief M, Camprodon JA. Safety Considerations for Cerebellar Theta Burst Stimulation. Clin Ther. 2020;42(7):1169-90.e1. Epub 2020/07/18. doi: 10.1016/j.clinthera.2020.06.001. PubMed PMID: 32674957.

3250. Hutton ARJ, McCrudden MTC, Larraneta E, Donnelly RF. Influence of molecular weight on transdermal delivery of model macromolecules using hydrogel-forming microneedles: potential to enhance the administration of novel low molecular weight biotherapeutics. Journal of Materials Chemistry B. 2020;8(19):4202-9. doi: 10.1039/d0tb00021c. PubMed PMID: WOS:000536814200004.

3251. Iazeva E, Legostaeva L, Bakulin I, Poydasheva A, Shabalina A, Sergeev D, et al. RTMS increases BDNF levels in patients with posttraumatic chronic disorders of consciousness. European Journal of Neurology. 2020;27:399-. PubMed PMID: WOS:000534616801125.

3252. Iazeva EG, Legostaeva LA, Bakulin IS, Poydasheva AG, Abaimov DA, Suponeva NA, et al. Effect of neuromodulation on neurotrophic factors in patients with chronic disorders of conciousness. Bulletin of Russian State Medical University. 2020(5):37-43. doi: 10.24075/brsmu.2020.056.

3253. Iovino L, Kinsella S, Cooper K, deRoos P, Ugrai TM, Gagnon A, et al. Thymic Reconstitution after Damage Is Influenced By Zinc Status. Biology of Blood and Marrow Transplantation. 2020;26(3):S304-S5. doi: 10.1016/j.bbmt.2019.12.403.

3254. Iovoli AJ, Ostrowski A, Rivers CI, Hermann GM, Groman A, Miller A, et al. Two- Versus Four-Times Weekly Acupuncture-Like Transcutaneous Electrical Nerve Stimulation for Treatment of Radiation-Induced Xerostomia: A Pilot Study. Journal of alternative and complementary medicine (New York, NY). 2020;26(4):323-8. doi: 10.1089/acm.2019.0131. PubMed PMID: CN-02073103.

3255. Irct20191016045135N. impact of Transcranial direct current stimulation (TDCS) on moral decision making in stroke patients. https://trialsearchwhoint/Trial2aspx?TrialID=IRCT20191016045135N1. 2020. PubMed PMID: CN-02238533.

3256. Irct20191229045931N. Effectiveness of Combined Transcranial Direct current Stimulation (tDCS) with Pharmacotherapy and Pharmacotherapy in Bipolar Disorders.

https://trialsearchwhoint/Trial2aspx?TrialID=IRCT20191229045931N1. 2020. PubMed PMID: CN-02187673.

3257. Iwanski S, Lesniak M, Polanowska K, Bembenek J, Czepiel W, Seniów J. Neuronavigated 1 Hz rTMS of the left angular gyrus combined with visuospatial therapy in post-stroke neglect. NeuroRehabilitation. 2020;46(1):83-93. doi: 10.3233/NRE-192951.

3258. Jagtap P, Chawa M, Pasovic V, Gautam M. A Review of Transcranial Magnetic Stimulation for Cognitive Impairment: Evidence for Expansion of Clinical Indications. Brain Stimulation. 2020;13(6):1855. doi: 10.1016/j.brs.2020.06.058.

3259. Jagtap P, Chawa M, Pasovic V, Gautam M. A review of non-invasive brain stimulation for cognitive impairment: Evidence for novel and widespread applications. Neuromodulation. 2020;23(3):e67-e8. doi: 10.1111/ner.13133.

3260. Jang SH, Kwon HG. Relationship between depression and dorsolateral prefronto-thalamic tract injury in patients with mild traumatic brain injury. Sci Rep. 2020;10(1):19728. Epub 2020/11/14. doi: 10.1038/s41598-020-76889-3. PubMed PMID: 33184443; PubMed Central PMCID: PMCPMC7661494.

3261. Jang SH, Kwon YH. Effect of repetitive transcranial magnetic stimulation on the ascending reticular activating system in a patient with disorder of consciousness: a case report. BMC Neurol.

2020;20(1):37. Epub 2020/01/31. doi: 10.1186/s12883-020-1607-9. PubMed PMID: 31996169; PubMed Central PMCID: PMCPMC6988329.

3262. Jang SH, Seo YS. Recovery of Injured Optic Radiations in a Patient with Hypoxic-Ischaemic Brain Injury. Neuroophthalmology. 2020;44(4):270-3. Epub 2020/10/06. doi: 10.1080/01658107.2019.1676263. PubMed PMID: 33012915; PubMed Central PMCID: PMCPMC7518302.

3263. Jang SH, Seo YS, Lee SJ. Increased thalamocortical connectivity to the medial prefrontal cortex with recovery of impaired consciousness in a stroke patient: A case report. Medicine (Baltimore). 2020;99(18):e19937. Epub 2020/05/03. doi: 10.1097/md.0000000000019937. PubMed PMID: 32358365; PubMed Central PMCID: PMCPMC7440307.

3264. Janicak P, Heart K, McGugan B. Post market rate of seizures during TMS treatment with NeuroStar system appears to be lower than previously estimated. CNS Spectrums. 2020;25(2):306. doi: 10.1017/S1092852920000188.

3265. Jiang Z, Xiong H, Yang S, Lu Y, Deng Y, Yao J, et al. Jet-Lagged Nanoparticles Enhanced Immunotherapy Efficiency through Synergistic Reconstruction of Tumor Microenvironment and Normalized Tumor Vasculature. Advanced Healthcare Materials.

2020;9(12). doi: 10.1002/adhm.202000075. PubMed PMID: WOS:000530633300001.

3266. Jing Z, Ling Y, Yi F. Multipoint transcutaneous electrical stimulation reduces median effective plasma concentration of propofol: A randomised clinical trial. Indian Journal of Anaesthesia. 2020;64(6):483-7. doi: 10.4103/ija.IJA_775_19.

3267. Kaboodvand N, Griffiths J. Influence of anatomical connectivity and intrinsic dynamics in a connectome based neural mass model of TMS-evoked potentials. BMC Neuroscience. 2020;21(SUPPL 1). doi: 10.1186/s12868-020-00593-1.

3268. Kaga T, Kato H, Hatano Y, Kawaguchi M, Furui T, Morishige KI, et al. Can MRI features differentiate ovarian mucinous carcinoma from mucinous borderline tumor? Eur J Radiol. 2020;132:109281. Epub 2020/09/23. doi: 10.1016/j.ejrad.2020.109281. PubMed PMID: 32961452.

3269. Kang DH, Kim GW. Changes in Diffuse Tensor Imaging and Therapeutic Effect of Repetitive Transcranial Magnetic Stimulation in Traumatic Brain Injury with Central Pain. Brain Sci. 2020;10(12). Epub 2020/12/06. doi: 10.3390/brainsci10120929. PubMed PMID: 33276440; PubMed Central PMCID: PMCPMC7759834.

3270. Kct. Effect of transcranial direct current stimulation on cognitive function in traumatic brain injury patients.

https://trialsearchwhoint/Trial2aspx?TrialID=KCT0005302. 2020. PubMed PMID: CN-02188991.

3271. Khaleghi A, Zarafshan H, Vand SR, Mohammadi MR. Effects of Non-invasive Neurostimulation on Autism Spectrum Disorder: A Systematic Review. Clinical Psychopharmacology and Neuroscience. 2020;18(4):527-52. doi: 10.9758/CPN.2020.18.4.527.

3272. Khan A, Peechatka A, Dias NR, Lima V, Seddo M, Inja A, et al. Patient perspective in the development of electronic patient-reported outcomes (ePROs) in seizure disorders: A patient-centric approach. Patient Preference and Adherence. 2020;14:13-21. doi: 10.2147/PPA.S222642.

3273. Kim M, Lee U. Alpha oscillation, criticality, and responsiveness in complex brain networks. Network Neuroscience. 2020;4(1):155-73. doi: 10.1162/netn_a_00113. PubMed PMID: WOS:000513793200007.

3274. Kinfe T, Buchfelder M. Incisionless MR-guided focused ultrasound deep brain modulation: Technical considerations and current therapeutic approaches in psychiatric disorders. Clinical Neurophysiology. 2020;131(4):e188-e9. doi: 10.1016/j.clinph.2019.12.030.

3275. King ML, Manzel K, Bruss J, Tranel D. Neural correlates of improvements in personality and behavior following a neurological

event. Neuropsychologia. 2020;145. doi: 10.1016/j.neuropsychologia.2017.11.023.

3276. Kinsella S, Evandy C, Hopwo KS, Cooper K, Iovino L, Smith CW, et al. Damage-Induced Pyroptotic Cell Death Facilitates Regeneration of the Thymus. Blood. 2020;136. doi: 10.1182/blood-2020-143051. PubMed PMID: WOS:000607547203078.

3277. Kletzel SL, Aaronson AL, Guernon A, Carbone C, Chaudhry N, Walsh E, et al. Safety Considerations for the Use of Transcranial Magnetic Stimulation as Treatment for Coma Recovery in People With Severe Traumatic Brain Injury. J Head Trauma Rehabil. 2020;35(6):430-8. Epub 2020/11/10. doi: 10.1097/htr.0000000000000636. PubMed PMID: 33165155; PubMed Central PMCID: PMCPMC8908773.

3278. Knoll RM, Ishai R, Lubner RJ, Trakimas DR, Brodsky JR, Jung DH, et al. Peripheral Vestibular Organ Degeneration After Temporal Bone Fracture: A Human Otopathology Study. Laryngoscope. 2020;130(3):752-60. Epub 2019/05/11. doi: 10.1002/lary.28010. PubMed PMID: 31074866.

3279. Knoll RM, Lubner RJ, Remenschneider AK, Kozin ED. Histopathology of audiovestibular dysfunction following head injury: A case report. Otolaryngology - Head and Neck Surgery. 2020;163(1

SUPPL):P233. doi: 10.1177/0194599820934781.
3280. Kojima Y, Sendo R, Ohno S, Sugimura M. Ultrasound-guided inferior alveolar nerve block for trismus during dental treatment: a case report. JA Clinical Reports. 2020;6(1). doi: 10.1186/s40981-020-00400-0.
3281. Kondziella D, Bender A, Diserens K, van Erp W, Estraneo A, Formisano R, et al. European Academy of Neurology guideline on the diagnosis of coma and other disorders of consciousness. Eur J Neurol. 2020;27(5):741-56. Epub 2020/02/25. doi: 10.1111/ene.14151. PubMed PMID: 32090418.
3282. Kopell B, Leung A, Levy R, Poree L, Wasserman E, Adamson MM, et al. Transcranial Magnetic Stimulation for Pain, Headache and co-morbid depression: INS-NANS Expert Consensus Panel Review and Recommendation. Brain stimulation. 2020;13(6):1851-. doi: 10.1016/j.brs.2020.06.046. PubMed PMID: CN-02212142.
3283. Kotob M. Adding illness to injury: A complicated football case. Clinical Journal of Sport Medicine. 2020;30(2):e44-e5. doi: 10.1097/JSM.0000000000000837.
3284. Kretzschmar M, Reining M. Opioid withdrawal during DRGS treatment of neuropathic pain after PNI: 36-months followup in 21 patients. Neuromodulation. 2020;23(3):e23. doi: 10.1111/ner.13133.
3285. Krewer C, Thibaut A. Non-invasive brain stimulation for

treatment of severe disorders of consciousness in people with acquired brain injury. Cochrane Database of Systematic Reviews. 2020;2020(2). doi: 10.1002/14651858.CD013533.

3286. Kutzer T, Dick M, Scudamore T, Wiener M, Schwartz T. Antidepressant efficacy and side effect burden: An updated guide for clinicians. Drugs in Context. 2020;9. doi: 10.7573/DIC.2020-2-2.

3287. Lang S, Yan X, Wang C, Wu Y, Liu X, Ma C, et al. The poly-ADP ribose polymerase-1/apoptosis-inducing factor pathway may help mediate the protective effect of electroacupuncture on early brain injury after subarachnoid hemorrhage. Neuroreport. 2020;31(8):605-12. Epub 2020/04/18. doi: 10.1097/wnr.0000000000001445. PubMed PMID: 32301816.

3288. Latchoumane CV, Barany DA, Karumbaiah L, Singh T. Neurostimulation and Reach-to-Grasp Function Recovery Following Acquired Brain Injury: Insight From Pre-clinical Rodent Models and Human Applications. Front Neurol. 2020;11:835. Epub 2020/08/28. doi: 10.3389/fneur.2020.00835. PubMed PMID: 32849253; PubMed Central PMCID: PMCPMC7396659.

3289. Lei K, Kunnel A, Metzger-Smith V, Golshan S, Javors J, Wei J, et al. Diminished corticomotor excitability in Gulf War Illness related chronic pain symptoms; evidence from TMS study. Scientific Reports. 2020;10(1). doi: 10.1038/s41598-020-75006-8. PubMed PMID:

WOS:000587657600016.
3290. León-Lozano MZ, Arnaez J, Valls A, Arca G, Agut T, Alarcón A, et al. Cerebrospinal fluid levels of neuron-specific enolase predict the severity of brain damage in newborns with neonatal hypoxic-ischemic encephalopathy treated with hypothermia. PLoS ONE. 2020;15(6). doi: 10.1371/journal.pone.0234082.
3291. Leung A. Addressing chronic persistent headaches after MTBI as a neuropathic pain state. Journal of Headache and Pain. 2020;21(1). doi: 10.1186/s10194-020-01133-2. PubMed PMID: WOS:000543404700002.
3292. Leung A, Ettenhofer M, Sargent P, Liu J, Poree L, Kopell B, et al. Transcranial magnetic stimulation for persistent post-traumatic headache-an INS/NANS expert consensus panel review and recommendation. Neuromodulation. 2020;23(3):e35-e6. doi: 10.1111/ner.13133. PubMed PMID: CN-02243846.
3293. Leung A, Shirvalkar P, Chen R, Kuluva J, Vaninetti M, Bermudes R, et al. Transcranial Magnetic Stimulation for Pain, Headache, and Comorbid Depression: INS-NANS Expert Consensus Panel Review and Recommendation. Neuromodulation. 2020;23(3):267-90. Epub 2020/03/27. doi: 10.1111/ner.13094. PubMed PMID: 32212288.
3294. Levy-Lamdan O, Zifman N, Sasson E, Efrati S, Hack DC,

Tanne D, et al. Evaluation of White Matter Integrity Utilizing the DELPHI (TMS-EEG) System. Front Neurosci. 2020;14:589107. Epub 2021/01/08. doi: 10.3389/fnins.2020.589107. PubMed PMID: 33408607; PubMed Central PMCID: PMCPMC7779791.

3295. Li Z, Zhang L, Zhang H, Yue L, Hu L. Pain-related gamma band oscillations: Progress and prospect. Chinese Science Bulletin. 2020;65(25):2752-62. PubMed PMID: CSCD:6818533.

3296. Liepert J, Stürner J, Büsching I, Sehle A, Schoenfeld MA. Effects of a single mental chronometry training session in subacute stroke patients – a randomized controlled trial. BMC Sports Science, Medicine and Rehabilitation. 2020;12(1). doi: 10.1186/s13102-020-00212-w.

3297. Lim K-S, Chia Z-J, Myint M-Z, Ara KJ, Chee Y-C, Heng W-T, et al. Epilepsy in Southeast Asia, how much have we closed the management gap in past two decades? Neurology Asia. 2020;25(4):425-38. PubMed PMID: WOS:000616431700001.

3298. Litvinenko IV, Zhivolupov SA, Samartsev IN, Kravchuk AY, Vorobyova MN, Yakovlev EV, et al. The Cholinergic Profile as a Target for Rational Therapy of Central Nervous System Diseases and Injuries. Neuroscience and Behavioral Physiology. 2020;50(9):1112-8. doi: 10.1007/s11055-020-01012-y.

3299. Liu J, Wang XL, Zi L, Yang CH, Li HP, Li N. [Effect of early

electroacupuncture intervention on conscious state of patients after traumatic brain injury surgery]. Zhongguo Zhen Jiu. 2020;40(5):479-82. Epub 2020/05/13. doi: 10.13703/j.0255-2930.20190506-0005. PubMed PMID: 32394653.
3300. Liu L, Feigin V, Sacco RL, Koroshetz WJ. Promoting global collaboration for brain health research. The BMJ. 2020;371. doi: 10.1136/bmj.m3753.
3301. Liu P, Du H, Chen Y, Wang H, Mao J, Zhang L, et al. Polymer microneedles with interconnected porous structures via a phase inversion route for transdermal medical applications. Journal of Materials Chemistry B. 2020;8(10):2032-9. doi: 10.1039/c9tb02837d. PubMed PMID: WOS:000520502600017.
3302. Liu S, Han X, Liu H, Zhao Y, Li H, Rupenthal DI, et al. Incorporation of ion exchange functionalized-montmorillonite into solid lipid nanoparticles with low irritation enhances drug bioavailability for glaucoma treatment. Drug Delivery. 2020;27(1):652-61. doi: 10.1080/10717544.2020.1756984. PubMed PMID: WOS:000530985800001.
3303. Liu T, Jiang G, Song G, Zhu J, Yang Y. Fabrication of separable microneedles with phase change coating for NIR-triggered transdermal delivery of metformin on diabetic rats. Biomedical Microdevices. 2020;22(1). doi: 10.1007/s10544-019-0468-8. PubMed

PMID: WOS:000511954500001.
3304. Locke MB, Toepp SL, Turco CV, Harasym DH, Rathbone MP, Noseworthy MD, et al. Altered motor system function in post-concussion syndrome as assessed via transcranial magnetic stimulation. Clinical neurophysiology practice. 2020;5:157-64. doi: 10.1016/j.cnp.2020.07.004. PubMed PMID: MEDLINE:32939420.
3305. Lorente L, Martín MM, Pérez-Cejas A, González-Rivero AF, Abreu-González P, Ramos L, et al. Traumatic brain injury patients mortality and serum total antioxidant capacity. Brain Sciences. 2020;10(2). doi: 10.3390/brainsci10020110.
3306. Lucente G, Valls-Sole J, Murillo N, Rothwell J, Coll J, Davalos A, et al. Noninvasive Brain Stimulation and Noninvasive Peripheral Stimulation for Neglect Syndrome Following Acquired Brain Injury. Neuromodulation. 2020;23(3):312-23. Epub 2019/11/15. doi: 10.1111/ner.13062. PubMed PMID: 31725939.
3307. Luneva I, Ryabinkina J, Piradov MA. Locked-in syndrome after bacterial ventriculitis. European Journal of Neurology. 2020;27:1180.
3308. Luo F, Li Y, Norgbey E, Li R, Ya Z, Nwankwegu AS, et al. A study on the occurrence of black water in reservoirs in Eucalyptus Plantation region. Environ Sci Pollut Res Int. 2020;27(28):34927-40. Epub 2020/06/25. doi: 10.1007/s11356-020-09613-3. PubMed PMID:

32577983.

3309. Lutkenhoff ES, Johnson MA, Casarotto S, Massimini M, Monti MM. Subcortical atrophy correlates with the perturbational complexity index in patients with disorders of consciousness. Brain Stimul. 2020;13(5):1426-35. Epub 2020/07/28. doi: 10.1016/j.brs.2020.07.012. PubMed PMID: 32717393.

3310. Ma XJ, Cheng OM, Xiao H, Song D, Jiang QS, Qiu H. Transcranial direct current stimulation promotes hippocampal neurogenesis in mice with cerebral ischemia involving up-regulation of nmda receptors. Chinese Pharmacological Bulletin. 2020;36(2):175-81. doi: 10.3969/j.issn.1001-1978.2020.02.07.

3311. Mailleux L, Simon-Martinez C, Radwan A, Blommaert J, Gooijers J, Wenderoth N, et al. White matter characteristics of motor, sensory and interhemispheric tracts underlying impaired upper limb function in children with unilateral cerebral palsy. Brain Struct Funct. 2020;225(5):1495-509. Epub 2020/04/23. doi: 10.1007/s00429-020-02070-1. PubMed PMID: 32318818.

3312. Maj R, Osório TG, Borio G, Iacopino S, Ströker E, Sieira J, et al. A novel strategy to treat vaso-vagal syncope: Cardiac neuromodulation by cryoballoon pulmonary vein isolation. Indian Pacing Electrophysiol J. 2020;20(4):154-9. Epub 2020/04/01. doi: 10.1016/j.ipej.2020.03.008. PubMed PMID: 32224089; PubMed

Central PMCID: PMCPMC7371945.
3313. Makhdoom SN, Campbell KM, Carvalho RM, Manso AP. Effects of curing modes on depth of cure and microtensile bond strength of bulk fill composites to dentin. J Appl Oral Sci. 2020;28:e20190753. Epub 2020/07/09. doi: 10.1590/1678-7757-2019-0753. PubMed PMID: 32638829; PubMed Central PMCID: PMCPMC7340207.
3314. Man VH, Li MS, Derreumaux P, Wang J, Nguyen TT, Nangia S, et al. Molecular mechanism of ultrasound interaction with a blood brain barrier model. Journal of Chemical Physics. 2020;153(4). doi: 10.1063/5.0010667. PubMed PMID: WOS:000556316200003.
3315. Marion S, Leonid C, Belinda B, Joanne D, Elise H, Leeanne C, et al. Effects of modified-release fampridine on upper limb impairment in patients with Multiple Sclerosis. Mult Scler Relat Disord. 2020;40:101971. Epub 2020/02/18. doi: 10.1016/j.msard.2020.101971. PubMed PMID: 32062444.
3316. Martens G, Kroupi E, Bodien Y, Frasso G, Annen J, Cassol H, et al. Behavioral and electrophysiological effects of network-based frontoparietal tDCS in patients with severe brain injury: A randomized controlled trial. Neuroimage Clin. 2020;28:102426. Epub 2020/09/26. doi: 10.1016/j.nicl.2020.102426. PubMed PMID: 32977212; PubMed Central PMCID: PMCPMC7511767.

3317. McGarry A, Auinger P, Kieburtz K, Geva M, Mehra M, Abler V, et al. Additional Safety and Exploratory Efficacy Data at 48 and 60 Months from Open-HART, an Open-Label Extension Study of Pridopidine in Huntington Disease. Journal of huntington's disease. 2020;9(2):173-84. doi: 10.3233/JHD-190393. PubMed PMID: CN-02142063.
3318. McMillan A, Kocharyan A, Dekker SE, Kikano EG, Garg A, Huang VW, et al. Comparison of Materials Used for 3D-Printing Temporal Bone Models to Simulate Surgical Dissection. Annals of Otology, Rhinology and Laryngology. 2020. doi: 10.1177/0003489420918273.
3319. Mencarelli L, Biagi MC, Salvador R, Romanella S, Ruffini G, Rossi S, et al. Network Mapping of Connectivity Alterations in Disorder of Consciousness: Towards Targeted Neuromodulation. J Clin Med. 2020;9(3). Epub 2020/03/22. doi: 10.3390/jcm9030828. PubMed PMID: 32197485; PubMed Central PMCID: PMCPMC7141258.
3320. Meng HJ, Zhang LL, Luo SS, Cao N, Zhang J, Pi YL. Modulation of hand motor skill performance induced by motor practice combined with matched or mismatched hand posture motor imagery. Physiology and Behavior. 2020;225. doi: 10.1016/j.physbeh.2020.113084.

3321. Mensen A, Bodart O, Thibaut A, Wannez S, Annen J, Laureys S, et al. Decreased Evoked Slow-Activity After tDCS in Disorders of Consciousness. Front Syst Neurosci. 2020;14:62. Epub 2020/10/27. doi: 10.3389/fnsys.2020.00062. PubMed PMID: 33100977; PubMed Central PMCID: PMCPMC7546425.
3322. Miller CP, Prener M, Dissing S, Paulson OB. Transcranial low-frequency pulsating electromagnetic fields (T-PEMF) as post-concussion syndrome treatment. Acta Neurol Scand. 2020;142(6):597-604. Epub 2020/06/20. doi: 10.1111/ane.13300. PubMed PMID: 32559313; PubMed Central PMCID: PMCPMC7689717.
3323. Miller SM. Endoscopic recurrent laryngeal neuropathy grade prevalence in a sample of thoroughbred yearlings at public auction in South Africa (2013-2019). J S Afr Vet Assoc. 2020;91(0):e1-e5. Epub 2020/05/07. doi: 10.4102/jsava.v91i0.2013. PubMed PMID: 32370531; PubMed Central PMCID: PMCPMC7203182.
3324. Modak M, Bobbala S, Lescott C, Liu Y-G, Nandwana V, Dravid VP, et al. Magnetic Nanostructure-Loaded Bicontinuous Nanospheres Support Multicargo Intracellular Delivery and Oxidation-Responsive Morphological Transitions. Acs Applied Materials & Interfaces. 2020;12(50):55584-95. doi: 10.1021/acsami.0c15920. PubMed PMID: WOS:000600202300005.

3325. Motes MA, Spence JS, Yeatman K, Jones PM, Lutrell M, O'Hair R, et al. High-Definition Transcranial Direct Current Stimulation to Improve Verbal Retrieval Deficits in Chronic Traumatic Brain Injury. J Neurotrauma. 2020;37(1):170-7. Epub 2019/07/30. doi: 10.1089/neu.2018.6331. PubMed PMID: 31354040.
3326. Moussi K, Bukhamsin A, Hidalgo T, Kosel J. Biocompatible 3D Printed Microneedles for Transdermal, Intradermal, and Percutaneous Applications. Advanced Engineering Materials. 2020;22(2). doi: 10.1002/adem.201901358. PubMed PMID: WOS:000502981900001.
3327. Moussi K, Kavaldzhiev M, Perez JE, Alsharif N, Merzaban J, Kosel J. 3D Printed Microneedle Array for Electroporation. Annual International Conference of the IEEE Engineering in Medicine and Biology Society IEEE Engineering in Medicine and Biology Society Annual International Conference. 2020;2020:2202-5. doi: 10.1109/embc44109.2020.9175748. PubMed PMID: MEDLINE:33018444.
3328. Mukherjee B, Nayak S. Nonorganic visual loss in a child. Saudi Journal of Ophthalmology. 2020;34(3):220-2. doi: 10.4103/1319-4534.310419.
3329. Murovec N, Heilinger A, Xu R, Ortner R, Spataro R, La Bella V, et al. Effects of a Vibro-Tactile P300 Based Brain-Computer

Interface on the Coma Recovery Scale-Revised in Patients With Disorders of Consciousness. Front Neurosci. 2020;14:294. Epub 2020/04/25. doi: 10.3389/fnins.2020.00294. PubMed PMID: 32327970; PubMed Central PMCID: PMCPMC7161577.

3330. Nabergoj M, Mauff K, Beelen DW, Ganser A, Kroger N, Bornhauser M, et al. Allogeneic Hematopoietic Cell Transplantation in Patients with Therapy-Related Myeloid Neoplasm after Breast Cancer: A Study of the Chronic Malignancies Working Party of the EBMT. Blood. 2020;136. doi: 10.1182/blood-2020-137773. PubMed PMID: WOS:000607547204058.

3331. Narapareddy BR, Narapareddy L, Lin A, Wigh S, Nanavati J, Dougherty J, 3rd, et al. Treatment of Depression After Traumatic Brain Injury: A Systematic Review Focused on Pharmacological and Neuromodulatory Interventions. Psychosomatics. 2020;61(5):481-97. Epub 2020/07/15. doi: 10.1016/j.psym.2020.04.012. PubMed PMID: 32660873.

3332. Nardone R, Sebastianelli L, Brigo F, Golaszewski S, Trinka E, Pucks-Faes E, et al. Effects of intrathecal baclofen therapy in subjects with disorders of consciousness: a reappraisal. J Neural Transm (Vienna). 2020;127(9):1209-15. Epub 2020/07/28. doi: 10.1007/s00702-020-02233-8. PubMed PMID: 32710152.

3333. Nardone R, Sebastianelli L, Versace V, Brigo F, Golaszewski

S, Manganotti P, et al. Repetitive transcranial magnetic stimulation in traumatic brain injury: Evidence from animal and human studies. Brain Res Bull. 2020;159:44-52. Epub 2020/04/07. doi: 10.1016/j.brainresbull.2020.03.016. PubMed PMID: 32251693.

3334. Naros G, Lehnertz T, Leão MT, Ziemann U, Gharabaghi A. Brain State-dependent Gain Modulation of Corticospinal Output in the Active Motor System. Cerebral Cortex. 2020;30(1):371-81. doi: 10.1093/cercor/bhz093.

3335. Nasios G, Bakirtzis C, Messinis L. Cognitive Impairment and Brain Reorganization in MS: Underlying Mechanisms and the Role of Neurorehabilitation. Frontiers in Neurology. 2020;11. doi: 10.3389/fneur.2020.00147.

3336. Nct. Spectral Correlates of Impulsivity in Patients With Traumatic Brain Injury. https://clinicaltrialsgov/show/NCT04239274. 2020. PubMed PMID: CN-02079648.

3337. Nct. Repetitive Transcranial Magnetic Stimulation in Traumatic Brain Injury. https://clinicaltrialsgov/show/NCT04573413. 2020. PubMed PMID: CN-02181687.

3338. Nct. Treatment of Post-concussion Syndrome With TMS: Using FNIRS as a Biomarker of Response. https://clinicaltrialsgov/show/NCT04568369. 2020. PubMed PMID: CN-02181558.

3339. Nct. Noninvasive Brain Stimulation on Memory in Individuals With Mild Cognitive Impairment and History of Brain Injury. https://clinicaltrialsgov/show/NCT04504630. 2020. PubMed PMID: CN-02145932.

3340. Nct. The Effects of Transcranial Direct Current Stimulation (tDCS) and Cognitive Training in Patients With TBI. https://clinicaltrialsgov/show/NCT04540783. 2020. PubMed PMID: CN-02148383.

3341. Nct. Neural, Behavioural, and Clinical Effects of tDCS in PDOC; Feasibility Study. https://clinicaltrialsgov/show/NCT04248946. 2020. PubMed PMID: CN-02079911.

3342. Nct. Transcranial Direct Current Stimulation (tDCS) Neuromodulation of Executive Function Across Neuropsychiatric Populations. https://clinicaltrialsgov/show/NCT04296604. 2020. PubMed PMID: CN-02088785.

3343. Nct. Does Physiotherapy Plus Non-invasive Brain Stimulation Improve Motor Recovery in Children With Acquired Brain Injury? https://clinicaltrialsgov/show/NCT04422886. 2020. PubMed PMID: CN-02125354.

3344. Nct. BoMI for Muscle Control. https://clinicaltrialsgov/show/NCT04641793. 2020. PubMed PMID: CN-02209200.

3345. Nct. Low Intensity Focused Ultrasound Pulses (LIFUP) to Modulate Pain. https://clinicaltrialsgov/show/NCT04339972. 2020. PubMed PMID: CN-02091502.
3346. Nct. Physiological Changes Induced Through MEP Conditioning in People With SCI. https://clinicaltrialsgov/show/NCT04286191. 2020. PubMed PMID: CN-02088552.
3347. Nct. Balance Training With tDCS for CAI. https://clinicaltrialsgov/show/NCT04390048. 2020. PubMed PMID: CN-02180589.
3348. Nct. Treating Self Injurious Behavior: A Novel Brain Stimulation Approach. https://clinicaltrialsgov/show/NCT04244786. 2020. PubMed PMID: CN-02079803.
3349. Nct. The Effect of Dorsolateral Prefrontal Cortex Theta Burst Stimulation on Alcohol Cue Reactivity and Cognitive Control: a Double-blind, Sham Controlled Study of Heavy Alcohol Drinkers With a History of Alcohol Related Injury. https://clinicaltrialsgov/show/NCT04223154. 2020. PubMed PMID: CN-02079220.
3350. Nct. rTMS and EEG in DOC Patients. https://clinicaltrialsgov/show/NCT04401319. 2020. PubMed PMID: CN-02180660.

3351. Nct. Modulating Neuropathic Pain With Transcranial Direct Current Stimulation. https://clinicaltrialsgov/show/NCT04581525. 2020. PubMed PMID: CN-02174612.

3352. Nct. Transcranial Direct Current Stimulation for Attention Deficit. https://clinicaltrialsgov/show/NCT04278430. 2020. PubMed PMID: CN-02088357.

3353. Nct. Neuromuscular Control in Individuals Following ACL-Reconstruction. https://clinicaltrialsgov/show/NCT04495075. 2020. PubMed PMID: CN-02145754.

3354. Nguyen S, Santhakumar V. From Plugging the Dam to Fueling the Firing: Platelets Breach the Barrier to Seize the Brain. Epilepsy Currents. 2020;20(5):300-2. doi: 10.1177/1535759720948117.

3355. Nieves MD, Furmanski O, Doughty ML. Host sex and transplanted human induced pluripotent stem cell phenotype interact to influence sensorimotor recovery in a mouse model of cortical contusion injury. Brain Research. 2020;1748. doi: 10.1016/j.brainres.2020.147120.

3356. Noe E, Ferri J, Colomer C, Moliner B, O'Valle M, Ugart P, et al. Feasibility, safety and efficacy of transauricular vagus nerve stimulation in a cohort of patients with disorders of consciousness. Brain Stimulation. 2020;13(2):427-9. doi: 10.1016/j.brs.2019.12.005. PubMed PMID: WOS:000509471600028.

3357. Oberman LM, Exley S, Philip NS, Siddiqi SH, Adamson MM, Brody DL. Use of Repetitive Transcranial Magnetic Stimulation in the Treatment of Neuropsychiatric and Neurocognitive Symptoms Associated With Concussion in Military Populations. J Head Trauma Rehabil. 2020;35(6):388-400. Epub 2020/11/10. doi: 10.1097/htr.0000000000000628. PubMed PMID: 33165152; PubMed Central PMCID: PMCPMC7664843.
3358. Okidi R, Ogwang DM, Okello TR, Ezati D, Kyegombe W, Nyeko D, et al. Factors affecting mortality after traumatic brain injury in a resource-poor setting. BJS open. 2020;4(2):320-5. doi: 10.1002/bjs5.50243.
3359. Olaya J, Noé E, Navarro MD, O'valle M, Colomer C, Moliner B, et al. When, how, and to what extent are individuals with unresponsive wakefulness syndrome able to progress? Functional independence. Brain Sciences. 2020;10(12):1-14. doi: 10.3390/brainsci10120990.
3360. Osali A. Aerobic exercise and nano-curcumin supplementation improve inflammation in elderly females with metabolic syndrome. Diabetology and Metabolic Syndrome. 2020;12(1). doi: 10.1186/s13098-020-00532-4.
3361. Pagnozzi AM, Pannek K, Fripp J, Fiori S, Boyd RN, Rose S. Understanding the impact of bilateral brain injury in children with

unilateral cerebral palsy. Hum Brain Mapp. 2020;41(10):2794-807. Epub 2020/03/07. doi: 10.1002/hbm.24978. PubMed PMID: 32134174; PubMed Central PMCID: PMCPMC7294067.

3362. Park JH, Lee JH, Lee S, Shin JY, Kim TH. Adverse events related to electroacupuncture: a systematic review of single case studies and case series. Acupunct Med. 2020;38(6):407-16. Epub 2020/05/19. doi: 10.1177/0964528420920287. PubMed PMID: 32418438.

3363. Pearce AJ, Kidgell DJ, Frazer AK, King DA, Buckland ME, Tommerdahl M. Corticomotor correlates of somatosensory reaction time and variability in individuals with post concussion symptoms. Somatosens Mot Res. 2020;37(1):14-21. Epub 2019/12/07. doi: 10.1080/08990220.2019.1699045. PubMed PMID: 31809669.

3364. Pena J, Sampedro A, Ibarretxe-Bilbao N, Zubiaurre-Elorza L, Aizpurua A, Ojeda N. The effect of transcranial random noise stimulation (tRNS) over bilateral posterior parietal cortex on divergent and convergent thinking. Scientific Reports. 2020;10(1). doi: 10.1038/s41598-020-72532-3. PubMed PMID: WOS:000573849300087.

3365. Pender SC, Smith AM, Finnoff JT, Huston J, Stuart MJ. Concussions in Ice Hockey - Moving Toward Objective Diagnoses and Point-of-care Treatment: A Review. Current Sports Medicine

Reports. 2020;19(9):380-6. doi: 10.1249/JSR.0000000000000752.
3366. Perin C, Vigano B, Piscitelli D, Matteo BM, Meroni R, Cerri CG. Non-invasive current stimulation in vision recovery: A review of the literature. Restorative Neurology and Neuroscience. 2020;38(3):239-50. doi: 10.3233/RNN-190948.
3367. Petrova M. Craniocerebral hypothermia as a neuromodulation method in chronically critically ill patients. Critical Care. 2020;24. doi: 10.1186/s13054-020-2772-3.
3368. Phillips A, Sami S, Adamson M. Sex Differences in Neuromodulation Treatment Approaches for Traumatic Brain Injury: A Scoping Review. J Head Trauma Rehabil. 2020;35(6):412-29. Epub 2020/11/10. doi: 10.1097/htr.0000000000000631. PubMed PMID: 33165154.
3369. Phillips N, Dalziel SR, Borland ML, Dalton S, Lyttle MD, Bressan S, et al. Imaging and admission practices in paediatric head injury across emergency departments in Australia and New Zealand: A PREDICT study. Emerg Med Australas. 2020;32(2):240-9. Epub 2019/11/28. doi: 10.1111/1742-6723.13396. PubMed PMID: 31773866.
3370. Pichini S, Di Trana A, Torrens M, Scherbaum N, Zaami S. Editorial: New Trends of Substance Abuse: Looking for New Psychotropic Effects of Chem Sex Drugs, Cognitive Enhancers, and

New Psychoactive Substances. Frontiers in Psychiatry. 2020;11. doi: 10.3389/fpsyt.2020.612192.

3371. Plamadeala C, Gosain SR, Hischen F, Buchroithner B, Puthukodan S, Jacak J, et al. Bio-inspired microneedle design for efficient drug/vaccine coating. Biomedical Microdevices. 2020;22(1). doi: 10.1007/s10544-019-0456-z. PubMed PMID: WOS:000511510400001.

3372. Pommier B, Quesada C, Nuti C, Peyron R, Vassal F. Is the analgesic effect of motor cortex stimulation somatotopically driven or not? Neurophysiol Clin. 2020;50(3):195-203. Epub 2020/05/22. doi: 10.1016/j.neucli.2020.04.002. PubMed PMID: 32434693.

3373. Poydasheva A, Sergeeva A, Kremneva E, Morozova S, Legostaeva L, Bakulin I, et al. Functional connectivity changes after rTMS over angular gyrus in chronic DOC patients. Clinical Neurophysiology. 2020;131(4):e129-e30. doi: 10.1016/j.clinph.2019.12.314.

3374. Pushchina EV, Marinina KS, Myasoyedov SD. Hydrogen Sulfide and Pathophysiology of the CNS. Neurophysiology. 2020;52(4):308-21. doi: 10.1007/s11062-021-09887-4.

3375. Qian Q, Ling YT, Zhong H, Zheng YP, Alam M. Restoration of arm and hand functions via noninvasive cervical cord neuromodulation after traumatic brain injury: a case study. Brain Inj.

2020;34(13-14):1771-80. Epub 2020/12/03. doi: 10.1080/02699052.2020.1850864. PubMed PMID: 33264033.

3376. Qu M, Kim H-J, Zhou X, Wang C, Jiang X, Zhu J, et al. Biodegradable microneedle patch for transdermal gene delivery. Nanoscale. 2020;12(32):16724-9. doi: 10.1039/d0nr02759f. PubMed PMID: WOS:000561429100009.

3377. Quinn DK, Upston J, Jones T, Brandt E, Story-Remer J, Fratzke V, et al. Cerebral Perfusion Effects of Cognitive Training and Transcranial Direct Current Stimulation in Mild-Moderate TBI. Front Neurol. 2020;11:545174. Epub 2020/10/30. doi: 10.3389/fneur.2020.545174. PubMed PMID: 33117255; PubMed Central PMCID: PMCPMC7575722.

3378. Read J, Kirsch I, McGrath L. Electroconvulsive Therapy for Depression: A Review of the Quality of ECT versus Sham ECT Trials and Meta-Analyses. Ethical Human Psychology and Psychiatry. 2020;21(2):64-103. doi: 10.1891/EHPP-D-19-00014.

3379. Rhoads M, Harper R, Snodgrass D, Yan-Go F, Sauerland E, Harper R. Neuromodulation of afferent fibers for Postural Orthostatic Trachycardia Syndrome (POTS). Faseb Journal. 2020;34. doi: 10.1096/fasebj.2020.34.s1.06800. PubMed PMID: WOS:000546107905210.

3380. Roach AE, Hines C, Stafford J, Mooney S. Repetitive

Transcranial Magnetic Stimulation for Treatment-Resistant Depression in Active-Duty Service Members Improves Depressive Symptoms. Journal of Ect. 2020;36(4):279-84. doi: 10.1097/yct.0000000000000680. PubMed PMID: WOS:000594475200022.

3381. Rodrigues PA, Zaninotto AL, Ventresca HM, Neville IS, Hayashi CY, Brunoni AR, et al. The Effects of Repetitive Transcranial Magnetic Stimulation on Anxiety in Patients With Moderate to Severe Traumatic Brain Injury: A Post-hoc Analysis of a Randomized Clinical Trial. Front Neurol. 2020;11:564940. Epub 2020/12/22. doi: 10.3389/fneur.2020.564940. PubMed PMID: 33343483; PubMed Central PMCID: PMCPMC7746857.

3382. Rojas SS, Veiga VC, Carvalho J, Lisboa AC, Ordinola AA, Nogueira CP, et al. Non-invasive brain compliance findings in patients with COVID-19 in a Brazilian intensive care unit. Critical Care. 2020;24(SUPPL 2). doi: 10.1186/s13054-020-03187-9.

3383. Romero-Martínez Á, Bressanutti S, Moya-Albiol L. A Systematic Review of the Effectiveness of Non-Invasive Brain Stimulation Techniques to Reduce Violence Proneness by Interfering in Anger and Irritability. J Clin Med. 2020;9(3). Epub 2020/03/28. doi: 10.3390/jcm9030882. PubMed PMID: 32213818; PubMed Central PMCID: PMCPMC7141522.

3384. Ronconi L, Melcher D, Junghöfer M, Wolters CH, Busch NA. Testing the effect of tACS over parietal cortex in modulating endogenous alpha rhythm and temporal integration windows in visual perception. European Journal of Neuroscience. 2020. doi: 10.1111/ejn.15017.

3385. Rosenfelder M, Wolff A, Niimi M, Sanz L, Martens G, Laureys S, et al. Repetitive transcranial magnetic stimulation and electroencephalography in patients with disorders of consciousness: protocol of a multi-center RCT. Clinical neurophysiology. 2020;131(4):e145-e6. doi: 10.1016/j.clinph.2019.12.338. PubMed PMID: CN-02100918.

3386. Rosenstock T, Picht T, Schneider H, Vajkoczy P, Thomale UW. Pediatric navigated transcranial magnetic stimulation motor and language mapping combined with diffusion tensor imaging tractography: clinical experience. J Neurosurg Pediatr. 2020;26(5):583-93. Epub 2020/07/25. doi: 10.3171/2020.4.Peds20174. PubMed PMID: 32707554.

3387. Roy R, Pattnaik S, Sivagurunathan S, Chidambaram S. Small ncRNA binding protein, PIWI: A potential molecular bridge between blood brain barrier and neuropathological conditions. Medical Hypotheses. 2020;138. doi: 10.1016/j.mehy.2020.109609.

3388. Rudroff T, Workman CD, Fietsam AC, Boles Ponto LL.

Imaging transcranial direct current stimulation (TDCS) with positron emission tomography (PET). Brain Sciences. 2020;10(4). doi: 10.3390/brainsci10040236.

3389. San Luciano M, Viehoever A, Bledsoe I, Dodenhoff K, Gittings M, Viser A, et al. Thalamic deep brain stimulation for acquired dystonia in children and young adults: A phase i clinical trial. Movement Disorders. 2020;35(SUPPL 1):S46. doi: 10.1002/mds.28268.

3390. Santiago JE, Cameron AP, Navarrete RA. Addressing Sphincter Dysfunction in the Female with Neurogenic Lower Urinary Tract Dysfunction. Current Bladder Dysfunction Reports. 2020;15(3):142-52. doi: 10.1007/s11884-020-00597-x.

3391. Sarasso S, D'Ambrosio S, Fecchio M, Casarotto S, Viganò A, Landi C, et al. Local sleep-like cortical reactivity in the awake brain after focal injury. Brain. 2020;143(12):3672-84. Epub 2020/11/15. doi: 10.1093/brain/awaa338. PubMed PMID: 33188680; PubMed Central PMCID: PMCPMC7805800.

3392. Schwedt TJ, Digre K, Tepper SJ, Spare NM, Ailani J, Birlea M, et al. The American Registry for Migraine Research: Research Methods and Baseline Data for an Initial Patient Cohort. Headache. 2020;60(2):337-47. doi: 10.1111/head.13688.

3393. Scott E, Kidgell DJ, Frazer AK, Pearce AJ. The

Neurophysiological Responses of Concussive Impacts: A Systematic Review and Meta-Analysis of Transcranial Magnetic Stimulation Studies. Frontiers in Human Neuroscience. 2020;14. doi: 10.3389/fnhum.2020.00306.
3394. Senatus P, Zurek S, Deogaonkar M. Deep Brain Stimulation and Motor Cortex Stimulation for Chronic Pain. Neurol India. 2020;68(Supplement):S235-s40. Epub 2020/12/16. doi: 10.4103/0028-3886.302471. PubMed PMID: 33318357.
3395. Sendra C, Salvador R, Lekadir K, Ruffini G, Pedro JB. StarNEt: A convolutional neural network for automated white and gray matter segmentation from magnetic resonance imaging in the human brain. Clinical Neurophysiology. 2020;131(4):e55-e6. doi: 10.1016/j.clinph.2019.12.190.
3396. Siddiqi S, Horn A, Hsu J, Choi KS, Mayberg H, Grafman J, et al. A Convergent Causal Substrate for Depression Symptoms Across Lesions and Brain Stimulation. Biological Psychiatry. 2020;87(9):S46-S. PubMed PMID: WOS:000535308200110.
3397. Siddiqi SH, Horn A, Schaper F, Hsu J, Padmanabhan J, Cash RFH, et al. A convergent circuit for depression across brain lesions and brain stimulation. Journal of Neuropsychiatry and Clinical Neurosciences. 2020;32(3):e22.
3398. Simon-Martinez C, Decraene L, Van Hoof E, Zielinski I,

Hoare B, Steenbergen B, et al. The impact of brain lesion features and corticospinal tract wiring pattern on mirror movement characteristics in children with unilateral cerebral palsy. Developmental Medicine and Child Neurology. 2020;62(SUPPL 4):32-3. doi: 10.1111/dmcn.14688.

3399. Simon-Martinez C, Mailleux L, Hoskens J, Ortibus E, Jaspers E, Wenderoth N, et al. Randomized controlled trial combining constraint-induced movement therapy and action-observation training in unilateral cerebral palsy: clinical effects and influencing factors of treatment response. Therapeutic Advances in Neurological Disorders. 2020;13. doi: 10.1177/1756286419898065.

3400. Singh KR, Sharma RM, Bhattacharyya D. Role of rTMS as an adjunctive treatment in treatment resistant OCD: a randomised double blind and sham control trial. Indian journal of psychiatry. 2020;62(7):S97-S8. PubMed PMID: CN-02101348.

3401. Singla RK, Guimarães AG, Zengin G. Editorial: Application of Plant Secondary Metabolites to Pain Neuromodulation. Frontiers in Pharmacology. 2020;11. doi: 10.3389/fphar.2020.623399.

3402. Sinitsyn DO, Poydasheva AG, Bakulin IS, Legostaeva LA, Iazeva EG, Sergeev DV, et al. Detecting the Potential for Consciousness in Unresponsive Patients Using the Perturbational Complexity Index. Brain Sci. 2020;10(12). Epub 2020/12/03. doi:

10.3390/brainsci10120917. PubMed PMID: 33260944; PubMed Central PMCID: PMCPMC7760168.

3403. Sollmann N, Fratini A, Zhang H, Zimmer C, Meyer B, Krieg SM. Associations between clinical outcome and tractography based on navigated transcranial magnetic stimulation in patients with language-eloquent brain lesions. Journal of Neurosurgery. 2020;132(4):1033-42. doi: 10.3171/2018.12.JNS182988.

3404. Sorouri Khorashad B, Khazai B, Talaei A, Acar F, Hudson AR, Borji N, et al. Neuroanatomy of transgender persons in a Non-Western population and improving reliability in clinical neuroimaging. Journal of Neuroscience Research. 2020;98(11):2166-77. doi: 10.1002/jnr.24702.

3405. Srivastav AK, Sharma N, Samuel AJ. tDCS combined with cognitive training in a patient with chronic traumatic head injury. Neurophysiol Clin. 2020;50(2):133-4. Epub 2020/03/10. doi: 10.1016/j.neucli.2020.02.004. PubMed PMID: 32147282.

3406. Stagg CJ. Exploring the infinite parameter space: rethinking assumptions underpinning the use of transcranial direct current stimulation to induce long-term effects. Journal of Physiology-London. 2020;598(4):621-2. doi: 10.1113/jp279295. PubMed PMID: WOS:000512369900001.

3407. Stevenson R, Samokhina E, Rossetti I, Morley JW, Buskila Y.

Neuromodulation of Glial Function During Neurodegeneration. Frontiers in Cellular Neuroscience. 2020;14. doi: 10.3389/fncel.2020.00278.
3408. Stilling J, Paxman E, Mercier L, Gan LS, Wang M, Amoozegar F, et al. Treatment of Persistent Post-Traumatic Headache and Post-Concussion Symptoms Using Repetitive Transcranial Magnetic Stimulation: A Pilot, Double-Blind, Randomized Controlled Trial. J Neurotrauma. 2020;37(2):312-23. Epub 2019/09/19. doi: 10.1089/neu.2019.6692. PubMed PMID: 31530227.
3409. Stokes W, Runnalls K, Choynowki J, St Pierre M, Anaya M, Statton MA, et al. Altered corticomotor latencies but normal motor neuroplasticity in concussed athletes. J Neurophysiol. 2020;123(5):1600-5. Epub 2020/02/20. doi: 10.1152/jn.00774.2019. PubMed PMID: 32073936; PubMed Central PMCID: PMCPMC7444927.
3410. Stoppacher S, Scheruebel S, Ucal M, Kornmuller K, Glowacki E, Schindl R, et al. Modeling External Stimulation of Excitable Cells Using a Novel Light-Activated Organic Semiconductor Technology. Studies in health technology and informatics. 2020;271:9-16. doi: 10.3233/shti200068. PubMed PMID: MEDLINE:32578535.
3411. Strüber D, Herrmann CS. Modulation of gamma oscillations as a possible therapeutic tool for neuropsychiatric diseases: A review

and perspective. Int J Psychophysiol. 2020;152:15-25. Epub 2020/04/03. doi: 10.1016/j.ijpsycho.2020.03.003. PubMed PMID: 32240665.

3412. Stultz DJ, Osburn S, Burns T, Pawlowska-Wajswol S, Walton R. Transcranial Magnetic Stimulation (TMS) Safety with Respect to Seizures: A Literature Review. Neuropsychiatr Dis Treat. 2020;16:2989-3000. Epub 2020/12/17. doi: 10.2147/ndt.S276635. PubMed PMID: 33324060; PubMed Central PMCID: PMCPMC7732158.

3413. Sun C, Li Y, Yates EA, Fernig DG. SimpleDSFviewer: A tool to analyze and view differential scanning fluorimetry data for characterizing protein thermal stability and interactions. Protein Science. 2020;29(1):19-27. doi: 10.1002/pro.3703.

3414. Sun J, Shi J, Hou J, Guo C, Heng X, Qi G. Electroacupuncture protects rats from ischemic brain injury via coffilin in mice. J Cell Biochem. 2020;121(1):43-8. Epub 2019/10/11. doi: 10.1002/jcb.27781. PubMed PMID: 31599049.

3415. Swanson BT, Creighton D. The degenerative lumbar disc: not a disease, but still an important consideration for OMPT practice: a review of the history and science of discogenic instability. Journal of Manual and Manipulative Therapy. 2020;28(4):191-200. doi: 10.1080/10669817.2020.1758520.

3416. Sydnor VJ, Bouix S, Pasternak O, Hartl E, Levin-Gleba L, Reid B, et al. Mild traumatic brain injury impacts associations between limbic system microstructure and post-traumatic stress disorder symptomatology. Neuroimage-Clinical. 2020;26. doi: 10.1016/j.nicl.2020.102190. PubMed PMID: WOS:000533149400014.
3417. Talbot CE, Zhao K, Ward M, Kandinov A, Mammis A, Paskhover B. Neuromodulation of the lingual nerve: a novel technique. J Neurosurg. 2020;134(3):1271-5. Epub 2020/04/25. doi: 10.3171/2020.2.Jns193109. PubMed PMID: 32330885.
3418. Talmasov D, Cotovio G, Barahona-Corrêa JB, Hsu J, Senova S, Ribeiro R, et al. Lesion network mapping of mania symptoms caused by focal brain lesions. Annals of Neurology. 2020;88(SUPPL 25):S70-S1. doi: 10.1002/ana.25865.
3419. Talmasov D, Cotovio G, Bernardo BarahonaCorrêa J, Hsu J, Senova S, Ribeiro R, et al. Network localization of mania symptoms based on focal bain lesions. Journal of Neuropsychiatry and Clinical Neurosciences. 2020;32(3):e23.
3420. Tan M, Chen Y, Guo Y, Yang C, Liu M, Guo D, et al. A low-intensity focused ultrasound-assisted nanocomposite for advanced triple cancer therapy: local chemotherapy, therapeutic extracellular vesicles and combined immunotherapy. Biomaterials Science.

2020;8(23):6703-17. doi: 10.1039/d0bm00804d. PubMed PMID: WOS:000592126300019.

3421. Taylor RM, Maharjan D, Moreu F, Baca JT. Parametric study of 3D printed microneedle (MN) holders for interstitial fluid (ISF) extraction. Microsystem Technologies-Micro-and Nanosystems-Information Storage and Processing Systems. 2020;26(6):2067-73. doi: 10.1007/s00542-020-04758-0. PubMed PMID: WOS:000510351400003.

3422. Tegeler CL, Howard LJ, Brown KL, Kellar DC, Shaltout HA, Gerdes L, et al. Exploratory use of artificial intelligence driven acoustic neuromodulation improved sleep, depression, anxiety, and stress in adults with persisting post-concussion symptoms. Sleep. 2020;43(SUPPL 1):A207-A8. doi: 10.1093/sleep/zsaa056.539.

3423. Tegeler CL, Shaltout HA, Lee SW, Simpson SL, Gerdes L, Tegeler CH. High-resolution, relational, resonance-based, electroencephalic mirroring (HIRREM) improves symptoms and autonomic function for insomnia: A randomized, placebo-controlled clinical trial. Brain and Behavior. 2020;10(11). doi: 10.1002/brb3.1826.

3424. Teghil A, Di Vita A, Pietranelli V, Matano A, Boccia M. Duration reproduction in regular and irregular contexts after unilateral brain damage: Evidence from voxel-based lesion-symptom mapping

and atlas-based hodological analysis. Neuropsychologia. 2020;147. doi: 10.1016/j.neuropsychologia.2020.107577. PubMed PMID: WOS:000581761400011.

3425. Texakalidis P, Tora M, McMahon T, Greven A, Anthony C, Boulis N. Percutaneous trigeminal ganglion stimulation for intractable facial pain. Journal of Neurosurgery. 2020;132(4):97. doi: 10.3171/2020.4.JNS.AANS2020abstracts.

3426. Texakalidis P, Tora MS, McMahon JT, Greven A, Anthony CL, Nagarajan P, et al. Percutaneous Trigeminal Stimulation for Intractable Facial Pain: A Case Series. Neurosurgery. 2020;87(3):547-54. Epub 2020/04/04. doi: 10.1093/neuros/nyaa065. PubMed PMID: 32243534.

3427. Tiotiu A, Badi Y, Kermani NZ, Hansbro P, Wheelock C, Dahlen SE, et al. Differential mast cell activation by transcriptomic signature analysis in the U-BIOPRED severe asthma cohort. European Respiratory Journal. 2020;56. doi: 10.1183/13993003.congress-2020.3827.

3428. Trapp N, Bruss J, Abu-Halawa O, Li S, Albazron F, Manzel K, et al. Large-scale lesion symptom mapping of depression: Anatomy of risk and resilience. Neuropsychopharmacology. 2020;45:316-7. doi: 10.1038/s41386-020-00892-5.

3429. Tseng CT, Brougher J, Gaulding SJ, Hassan BS, Thorn CA. Vagus nerve stimulation promotes cortical reorganization and reduces

task-dependent calorie intake in male and female rats. Brain Res. 2020;1748:147099. Epub 2020/09/09. doi: 10.1016/j.brainres.2020.147099. PubMed PMID: 32896520.

3430. Tsilimigras DI, Mehta R, Guglielmi A, Ratti F, Marques HP, Soubrane O, et al. Recurrence beyond the Milan criteria after curative-intent resection of hepatocellular carcinoma: A novel tumor-burden based prediction model. Journal of Surgical Oncology. 2020;122(5):955-63. doi: 10.1002/jso.26091.

3431. Tucak A, Sirbubalo M, Hindija L, Rahic O, Hadziabdic J, Muhamedagic K, et al. Microneedles: Characteristics, Materials, Production Methods and Commercial Development. Micromachines. 2020;11(11). doi: 10.3390/mi11110961. PubMed PMID: WOS:000594026600001.

3432. Tzur R, David M, Barron J, Jaul E, Meiron O. Prefrontal cortex transcranial direct-current stimulation putatively enhances electroencephalography classification resolution of binary responses in a minimal conscious state patient. Clin Neurophysiol. 2020;131(9):2148-9. Epub 2020/07/19. doi: 10.1016/j.clinph.2020.06.009. PubMed PMID: 32682242.

3433. Udovin LD, Kobiec T, Herrera MI, Toro-Urrego N, Kusnier CF, Kölliker-Frers RA, et al. Partial Reversal of Striatal Damage by Palmitoylethanolamide Administration Following Perinatal Asphyxia.

Frontiers in Neuroscience. 2020;13. doi: 10.3389/fnins.2019.01345.
3434. Vallence AM, Fujiyama H, Rurak BK, Wansbrough K, Haigh Y. Suspected vasovagal syncope during single-pulse TMS in healthy adults: Three case reports. Clin Neurophysiol. 2020;131(11):2540-1. Epub 2020/09/15. doi: 10.1016/j.clinph.2020.08.007. PubMed PMID: 32927208.
3435. Van Dam J, Goldsworthy M. Case report of a vasovagal pre-syncope event during single-pulse transcranial magnetic stimulation in a healthy adult participant. Clin Neurophysiol. 2020;131(4):981-2. Epub 2020/02/06. doi: 10.1016/j.clinph.2019.12.401. PubMed PMID: 32008908.
3436. Vaninetti M, Lim M, Khalaf A, Smith VM, Flowers M, Kunnel A, et al. Cortical pain modulatory functional enhancement after rTMS associated with durable headache improvement in mTBI patients. Brain stimulation. 2020;13(6):1853-. doi: 10.1016/j.brs.2020.06.051. PubMed PMID: CN-02264756.
3437. Volkers R, Giesen E, van der Heiden M, Kerperien M, Lange S, Kurt E, et al. Invasive Motor Cortex Stimulation Influences Intracerebral Structures in Patients With Neuropathic Pain: An Activation Likelihood Estimation Meta-Analysis of Imaging Data. Neuromodulation. 2020;23(4):436-43. doi: 10.1111/ner.13119. PubMed PMID: WOS:000511365400001.

3438. Wan H-P, Ni Y-Q. A New Approach for Interval Dynamic Analysis of Train-Bridge System Based on Bayesian Optimization. Journal of Engineering Mechanics. 2020;146(5). doi: 10.1061/(asce)em.1943-7889.0001735. PubMed PMID: WOS:000521194600005.
3439. Wang H-y, Hou L, Li H-l, Wang X, Cao Y, Zhang B-y, et al. A nanosystem loaded with perfluorohexane and rose bengal coupled upconversion nanoparticles for multimodal imaging and synergetic chemo-photodynamic therapy of cancer. Biomaterials Science. 2020;8(9):2488-506. doi: 10.1039/c9bm02081k. PubMed PMID: WOS:000532529400010.
3440. Wang M, Han Y, Yu X, Liang L, Chang H, Yeo DC, et al. Upconversion Nanoparticle Powered Microneedle Patches for Transdermal Delivery of siRNA. Advanced Healthcare Materials. 2020;9(2). doi: 10.1002/adhm.201900635. PubMed PMID: WOS:000499713200001.
3441. Wang M, Yang Q, Li M, Zou H, Wang Z, Ran H, et al. Multifunctional Nanoparticles for Multimodal Imaging-Guided Low-Intensity Focused Ultrasound/Immunosynergistic Retinoblastoma Therapy. Acs Applied Materials & Interfaces. 2020;12(5):5642-57. doi: 10.1021/acsami.9b22072. PubMed PMID: WOS:000512216900044.

3442. Wang X, Guo Y, Zhang Y, Li J, Gao Z, Li Y, et al. Combined Behavioral and Mismatch Negativity Evidence for the Effects of Long-Lasting High-Definition tDCS in Disorders of Consciousness: A Pilot Study. Front Neurosci. 2020;14:381. Epub 2020/05/16. doi: 10.3389/fnins.2020.00381. PubMed PMID: 32410950; PubMed Central PMCID: PMCPMC7198816.

3443. Wang Y, Bai Y, Xia X, Yang Y, He J, Li X. Spinal cord stimulation modulates complexity of neural activities in patients with disorders of consciousness. International Journal of Neuroscience. 2020;130(7):662-70. doi: 10.1080/00207454.2019.1702543. PubMed PMID: WOS:000503531500001.

3444. Wang Y, Wang H, Zhu XX, Guan Y, Zhang Y. Smart microneedle patches for rapid, and painless transdermal insulin delivery. Journal of Materials Chemistry B. 2020;8(40):9335-42. doi: 10.1039/d0tb01822h. PubMed PMID: WOS:000580584800012.

3445. Wang Y, Xia X, Yang Y, Bai Y, He J, Li X. Recent advance in non-invasive neuromodulation in disorders of consciousness. Chinese Journal of Neuromedicine. 2020;19(9):966-71. PubMed PMID: CSCD:6807579.

3446. Webler RD, Hamady C, Molnar C, Johnson K, Bonilha L, Anderson BS, et al. Decreased interhemispheric connectivity and increased cortical excitability in unmedicated schizophrenia: A

prefrontal interleaved TMS fMRI study. Brain Stimulation. 2020;13(5):1467-75. doi: 10.1016/j.brs.2020.06.017.
3447. Wei S, Vaska P. Evaluation of quantitative, efficient image reconstruction for VersaPET, a compact PET system. Medical Physics. 2020;47(7):2852-68. doi: 10.1002/mp.14158.
3448. Wende T, Hoffmann KT, Meixensberger J. Tractography in Neurosurgery: A Systematic Review of Current Applications. J Neurol Surg A Cent Eur Neurosurg. 2020;81(5):442-55. Epub 2020/03/17. doi: 10.1055/s-0039-1691823. PubMed PMID: 32176926.
3449. Wilson C, Selwyn R, Quinn D, Elojeimy S. Detection of Regional Neurometabolic Differences in Individual TBI Subjects After tDCS Therapy. Journal of Nuclear Medicine. 2020;61. PubMed PMID: WOS:000568290501467.
3450. Wilson CL, Tavender EJ, Phillips NT, Hearps SJC, Foster K, O'Brien SL, et al. Variation in CT use for paediatric head injuries across different types of emergency departments in Australia and New Zealand. Emergency Medicine Journal. 2020;37(11):686-9. doi: 10.1136/emermed-2020-209719.
3451. Wu XL, Liu LX, Yang LY, Zhang T. Comprehensive rehabilitation in a patient with corpus callosum syndrome after traumatic brain injury: Case report. Medicine (Baltimore). 2020;99(28):e21218. Epub 2020/07/16. doi:

10.1097/md.0000000000021218. PubMed PMID: 32664173; PubMed Central PMCID: PMCPMC7360258.

3452. Wu Y, Wang L, Yang F, Xi W. Neural Circuits for Sleep-Wake Regulation. Adv Exp Med Biol. 2020;1284:91-112. Epub 2020/08/28. doi: 10.1007/978-981-15-7086-5_8. PubMed PMID: 32852742.

3453. Xie L, Zeng H, Sun J, Qian W. Engineering Microneedles for Therapy and Diagnosis: A Survey. Micromachines. 2020;11(3). doi: 10.3390/mi11030271. PubMed PMID: WOS:000526546000102.

3454. xmkyy RBR. Neurostimulation and memory training in depression. https://trialsearchwhoint/Trial2aspx?TrialID=RBR-3xmkyy. 2020. PubMed PMID: CN-02189333.

3455. Xu C-M, Tang M, Feng J, Xia H-F, Wu L-L, Pang D-W, et al. A liquid biopsy-guided drug release system for cancer theranostics: integrating rapid circulating tumor cell detection and precision tumor therapy. Lab on a Chip. 2020;20(8):1418-25. doi: 10.1039/d0lc00149j. PubMed PMID: WOS:000527017200003.

3456. Xu G, Sheng Q, Xin Q, Song Y, Zhang G, Yuan L, et al. EEG Assessment in a 2-Year-Old Child with Prolonged Disorders of Consciousness: 3 Years' Follow-up. Comput Intell Neurosci. 2020;2020:8826238. Epub 2020/12/10. doi: 10.1155/2020/8826238. PubMed PMID: 33293944; PubMed Central PMCID:

PMCPMC7718066.
3457. Yang A, Qiao B, Strohm EM, Cao J, Wang Z, Yuan X, et al. Thrombin-responsive engineered nanoexcavator with full-thickness infiltration capability for pharmaceutical-free deep venous thrombosis theranostics. Biomaterials Science. 2020;8(16):4545-58. doi: 10.1039/d0bm00917b. PubMed PMID: WOS:000558225900013.
3458. Yang Q, An PF, Wang RH, Wang D, Zhang RC. [Effect of electroacupuncture at different stages on the expression of Fas and FasL in brain tissue of rats with traumatic brain injury]. Zhen Ci Yan Jiu. 2020;45(9):714-9. Epub 2020/09/23. doi: 10.13702/j.1000-0607.190863. PubMed PMID: 32959553.
3459. Yang S, Chang MC. Effect of Repetitive Transcranial Magnetic Stimulation on Pain Management: A Systematic Narrative Review. Frontiers in Neurology. 2020;11. doi: 10.3389/fneur.2020.00114. PubMed PMID: WOS:000518616400001.
3460. Yao W, Li D, Zhao Y, Zhan Z, Jin G, Liang H, et al. 3D Printed Multi-Functional Hydrogel Microneedles Based on High-Precision Digital Light Processing. Micromachines. 2020;11(1). doi: 10.3390/mi11010017. PubMed PMID: WOS:000514309100017.
3461. Yasen AL, Eick GN, Sterner KN, Christie AD. The Influence Of Apoe Genotype On Motor Cortex Function Following Mtbi. Medicine and Science in Sports and Exercise. 2020;52(17):942-.

PubMed PMID: WOS:000590026304178.
3462. Yasen AL, Lim MM, Weymann KB, Christie AD. Excitability, Inhibition, and Neurotransmitter Levels in the Motor Cortex of Symptomatic and Asymptomatic Individuals Following Mild Traumatic Brain Injury. Front Neurol. 2020;11:683. Epub 2020/08/09. doi: 10.3389/fneur.2020.00683. PubMed PMID: 32765402; PubMed Central PMCID: PMCPMC7381132.
3463. Yuan X, Yang Y, Cao N, Jiang C. Promotion of poststroke motor-function recovery with repetitive transcranial magnetic stimulation by regulating the interhemispheric imbalance. Brain Sciences. 2020;10(9):1-14. doi: 10.3390/brainsci10090648.
3464. Zare EN, Jamaledin R, Naserzadeh P, Afjeh-Dana E, Ashtari B, Hosseinzadeh M, et al. Metal-Based Nanostructures/PLGA Nanocomposites: Antimicrobial Activity, Cytotoxicity, and Their Biomedical Applications. Acs Applied Materials & Interfaces. 2020;12(3):3279-300. doi: 10.1021/acsami.9b19435. PubMed PMID: WOS:000509428300001.
3465. Zewdie E, Ciechanski P, Kuo HC, Giuffre A, Kahl C, King R, et al. Safety and tolerability of transcranial magnetic and direct current stimulation in children: Prospective single center evidence from 3.5 million stimulations. Brain Stimul. 2020;13(3):565-75. Epub 2020/04/15. doi: 10.1016/j.brs.2019.12.025. PubMed PMID:

32289678.
3466. Zhang D, Fu Y, Dionigi G, Pontin A, Caruso E, Antonella P, et al. Human cadaveric model for studying the preservation of mental nerve during transoral endoscopic thyroidectomy. Surg Radiol Anat. 2020;42(1):55-62. Epub 2019/08/25. doi: 10.1007/s00276-019-02306-8. PubMed PMID: 31444547.
3467. Zhang D, Li S, Dionigi G, Wang T, Zhang J, Xue G, et al. Consequences of Trocar Dynamics on Mental Nerve in Transoral Thyroidectomy. Surg Laparosc Endosc Percutan Tech. 2020;30(4):305-11. Epub 2020/05/28. doi: 10.1097/sle.0000000000000795. PubMed PMID: 32459705.
3468. Zhang K-Y, Rui G, Zhang J-P, Guo L, An G-Z, Lin J-J, et al. Cathodal tDCS exerts neuroprotective effect in rat brain after acute ischemic stroke. Bmc Neuroscience. 2020;21(1). doi: 10.1186/s12868-020-00570-8. PubMed PMID: WOS:000535612200002.
3469. Zhang Q, Liu J, Cao R, Jin Y. Acupuncture for Patients in Coma after Traumatic Brain Injury: Systematic Review and Meta-Analysis. Altern Ther Health Med. 2020;26(4):50-7. Epub 2020/02/24. PubMed PMID: 32088667.
3470. Zhang R, Zhang L, Guo Y, Shi L, Gao J, Wang X, et al. Effects of High-Definition Transcranial Direct-Current Stimulation on

Resting-State Functional Connectivity in Patients With Disorders of Consciousness. Front Hum Neurosci. 2020;14:560586. Epub 2020/10/27. doi: 10.3389/fnhum.2020.560586. PubMed PMID: 33100996; PubMed Central PMCID: PMCPMC7546763.

3471. Zhang X, Chen G, Liu Y, Sun L, Sun L, Zhao Y. Black Phosphorus-Loaded Separable Microneedles as Responsive Oxygen Delivery Carriers for Wound Healing. Acs Nano. 2020;14(5):5901-8. doi: 10.1021/acsnano.0c01059. PubMed PMID: WOS:000537682300073.

3472. Zhang X, Liu B, Li N, Li Y, Hou J, Duan G, et al. Transcranial Direct Current Stimulation Over Prefrontal Areas Improves Psychomotor Inhibition State in Patients With Traumatic Brain Injury: A Pilot Study. Front Neurosci. 2020;14:386. Epub 2020/06/09. doi: 10.3389/fnins.2020.00386. PubMed PMID: 32508560; PubMed Central PMCID: PMCPMC7251071.

3473. Zhang XH, Li FF, Qi Y, Ming CR, Li Y, Pan ST, et al. [Electroacupuncture improves cutaneous allergic reaction by inhibiting degranulation of intrape-ritoneal mast cells, MAPK signaling and inflammatory factor levels in urticaria rats]. Zhen Ci Yan Jiu. 2020;45(4):299-304. Epub 2020/04/26. doi: 10.13702/j.1000-0607.190263. PubMed PMID: 32333535.

3474. Zhang Y, Du J, Li R. The application of P300 in evaluating

therapeutic efficacy of transcranial direct current stimulation in patients with minimally conscious state. Chinese Journal of Rehabilitation Medicine. 2020;35(1):22-8. PubMed PMID: CSCD:6656605.

3475. Zhang Y, Lu J, Du J, Huo S, Li R, Song W. Neural correlates of different behavioral response to transcranial direct current stimulation between patients in the unresponsive wakefulness syndrome and minimally conscious state. Neurol Sci. 2020;41(1):75-82. Epub 2019/08/20. doi: 10.1007/s10072-019-04034-8. PubMed PMID: 31422504.

3476. Zhao CG, Sun W, Ju F, Wang H, Sun XL, Mou X, et al. Analgesic Effects of Directed Repetitive Transcranial Magnetic Stimulation in Acute Neuropathic Pain After Spinal Cord Injury. Pain medicine (Malden, Mass). 2020;21(6):1216-23. doi: 10.1093/pm/pnz290. PubMed PMID: CN-02009667.

3477. Zhao K, Liu A. Effect of Right Median Nerve Stimulation on Clinical Efficacy and Prognosis of Patients with Consciousness Disorder Caused by Traumatic Brain Injury. Rehabilitation Medicine. 2020;30(3):192-6. PubMed PMID: CSCD:6757067.

3478. Zhao X, Zhang S, Yang G, Zhou Z, Gao Y. Exploring Trehalose on the Release of Levonorgestrel from Implantable PLGA Microneedles. Polymers. 2020;12(1). doi: 10.3390/polym12010059.

PubMed PMID: WOS:000519848300059.
3479. Zheng SM, Zhao FL, Luo YY, Lin XF, Wen MY. [Clinical effect of electroacupuncture at Baihui and Shuigou points in treatment of brain injury in patients with sepsis-associated encephalopathy]. Zhen Ci Yan Jiu. 2020;45(5):402-6. Epub 2020/05/25. doi: 10.13702/j.1000-0607.190781. PubMed PMID: 32447856.
3480. Zurita Saavedra M, Pérez Alonso A, Pérez Cabrera B, Haro García AM, Ruiz García VM, Mirón Pozo B, et al. Management of Splenic Injuries Utilizing a Multidisciplinary Protocol in 110 Consecutive Patients at a Level II Hospital. Cirugia Espanola. 2020;98(3):143-8. doi: 10.1016/j.ciresp.2019.08.007.
3481. 2021 American Neuropsychiatric Association Annual Meeting Abstracts. Journal of Neuropsychiatry and Clinical Neurosciences. 2021;33(3).
3482. Adamson M, McNerney MW, Siddiqi S, Swaminath G, Wu LL, Hernandez B, et al. Default mode network connectivity and brain derived neurotrophic factor in brain stimulation of traumatic brain injury patients. Brain stimulation. 2021;14(6):1633-. doi: 10.1016/j.brs.2021.10.146. PubMed PMID: CN-02345974.
3483. Ahorsu DK, Adjaottor ES, Lam BYH. Intervention Effect of Non-Invasive Brain Stimulation on Cognitive Functions among People with Traumatic Brain Injury: A Systematic Review and

Meta-Analysis. Brain Sci. 2021;11(7). Epub 2021/07/03. doi: 10.3390/brainsci11070840. PubMed PMID: 34202739; PubMed Central PMCID: PMCPMC8301762.

3484. Akkoc Altinok DC, Votinov M, Henzelmann F, Jo H, Eisert A, Habel U, et al. A combined administration of testosterone and arginine vasopressin affects aggressive behavior in males. Brain Sciences. 2021;11(12). doi: 10.3390/brainsci11121623.

3485. Akram Z, Daood U, Aati S, Ngo H, Fawzy AS. Formulation of pH-sensitive chlorhexidine-loaded/mesoporous silica nanoparticles modified experimental dentin adhesive. Materials Science & Engineering C-Materials for Biological Applications. 2021;122. doi: 10.1016/j.msec.2021.111894. PubMed PMID: WOS:000624405100002.

3486. Aksu T, Davila A, Gupta D. The "heart brain" and neuromodulation for vasovagal syncope. Auton Neurosci. 2021;236:102892. Epub 2021/10/20. doi: 10.1016/j.autneu.2021.102892. PubMed PMID: 34666205.

3487. Aksu T, Turagam M, Gautam S, Futyma P, Akella K, Baysal E, et al. Effects of permanent cardiac pacing on ventricular repolarization when compared to cardioneuroablation. J Electrocardiol. 2021;67:13-8. Epub 2021/05/14. doi: 10.1016/j.jelectrocard.2021.04.006. PubMed PMID: 33984570.

3488. Aloi D, Della Rocchetta AI, Ditchfield A, Coulborn S, Fernández-Espejo D. Therapeutic Use of Transcranial Direct Current Stimulation in the Rehabilitation of Prolonged Disorders of Consciousness. Front Neurol. 2021;12:632572. Epub 2021/04/27. doi: 10.3389/fneur.2021.632572. PubMed PMID: 33897592; PubMed Central PMCID: PMCPMC8058460.

3489. Alpsoy MY, Sönmez S, Orhan Z, Orhan EK, Aslıyüksek H, Orhan KS. Evaluation of patients with post-traumatic hearing loss: A retrospective review of 506 cases. Journal of International Advanced Otology. 2021;17(3):239-44. doi: 10.5152/iao.2021.9089.

3490. An Y, Yang R, Wang X, Han Y, Jia G, Hu C, et al. Facile Assembly of Thermosensitive Liposomes for Active Targeting Imaging and Synergetic Chemo-/Magnetic Hyperthermia Therapy. Frontiers in Bioengineering and Biotechnology. 2021;9. doi: 10.3389/fbioe.2021.691091. PubMed PMID: WOS:000687220800001.

3491. Anasobi AT, Macel LP, Hirsch AR. Inhibition of hallucinations through electrical stimulation of the cerebellum. Annals of Neurology. 2021;90(SUPPL 27):S40. doi: 10.1002/ana.26180.

3492. Andre-Obadia N, Magnin M, Garcia-Larrea L. Theta-burst versus 20 Hz repetitive transcranial magnetic stimulation in neuropathic pain: A head-to-head comparison. Clinical

Neurophysiology. 2021;132(10):2702-10. doi: 10.1016/j.clinph.2021.05.022. PubMed PMID: CN-02309283.

3493. Arai N, Nakanishi T, Nakajima S, Li X, Wada M, Daskalakis ZJ, et al. Insights of neurophysiology on unconscious state using combined transcranial magnetic stimulation and electroencephalography: A systematic review. Neurosci Biobehav Rev. 2021;131:293-312. Epub 2021/09/24. doi: 10.1016/j.neubiorev.2021.09.029. PubMed PMID: 34555384.

3494. Argyriou AA, Mitsikostas DD, Mantovani E, Litsardopoulos P, Panagiotopoulos V, Tamburin S. An updated brief overview on post-traumatic headache and a systematic review of the non-pharmacological interventions for its management. Expert Rev Neurother. 2021;21(4):475-90. Epub 2021/03/09. doi: 10.1080/14737175.2021.1900734. PubMed PMID: 33682560.

3495. Asimakidou E, Abut PA, Raabe A, Seidel K. Motor evoked potential warning criteria in supratentorial surgery: A scoping review. Cancers. 2021;13(11). doi: 10.3390/cancers13112803.

3496. Auxéméry Y, Gayraud F. Post-concussion syndrome: Still forgotten? From the disappearance of the DSM-5 to a multidisciplinary consultation. Encephale. 2021;47(5):491-4. doi: 10.1016/j.encep.2020.06.017.

3497. Ayache SS, Chalah MA. Cognitive fatigability in the healthy

brain: Neurophysiological substrates and the use of tDCS. Clinical Neurophysiology. 2021;132(7):1714-5. doi: 10.1016/j.clinph.2021.03.018. PubMed PMID: WOS:000659518700011.
3498. Azizi S, Irani A, Shahrokhi A, Rahimian E, Mirbagheri MM. Contribution of altered corticospinal microstructure to gait impairment in children with cerebral palsy. Clinical Neurophysiology. 2021;132(9):2211-21. doi: 10.1016/j.clinph.2021.06.016. PubMed PMID: WOS:000691755500026.
3499. Babity S, Laszlo E, Brambilla D. Polymer-Based Microneedles for Decentralized Diagnostics and Monitoring: Concepts, Potentials, and Challenges. Chemistry of Materials. 2021;33(18):7148-59. doi: 10.1021/acs.chemmater.1c01866. PubMed PMID: WOS:000703532600002.
3500. Bai Y, Lin Y, Ziemann U. Managing disorders of consciousness: the role of electroencephalography. J Neurol. 2021;268(11):4033-65. Epub 2020/09/12. doi: 10.1007/s00415-020-10095-z. PubMed PMID: 32915309; PubMed Central PMCID: PMCPMC8505374.
3501. Bareham CA, Oxner M, Gastrell T, Carmel D. Beyond the neural correlates of consciousness: using brain stimulation to elucidate causal mechanisms underlying conscious states and contents. Journal

of the Royal Society of New Zealand. 2021;51(1):143-70. doi: 10.1080/03036758.2020.1840405. PubMed PMID: WOS:000588817900001.

3502. Barra A, Martens G, Pacheco-Barrios K, Gosseries O, Fregni F, Laureys S, et al. Neuromodulation to promote recovery in patients with disorders of consciousness: a systematic review and meta-analysis. European Journal of Neurology. 2021;28:443-. PubMed PMID: WOS:000663065901002.

3503. Barth BB, Travis L, Spencer NJ, Grill WM. Control of colonic motility using electrical stimulation to modulate enteric neural activity. Am J Physiol Gastrointest Liver Physiol. 2021;320(4):G675-g87. Epub 2021/02/25. doi: 10.1152/ajpgi.00463.2020. PubMed PMID: 33624530; PubMed Central PMCID: PMCPMC8238160.

3504. Beisegel J, Denkert C, Koehler E, Krnc M, Pivac N, Scheffler R, et al. THE RECOGNITION PROBLEM OF GRAPH SEARCH TREES. Siam Journal on Discrete Mathematics. 2021;35(2):1418-46. doi: 10.1137/20m1313301. PubMed PMID: WOS:000674142200039.

3505. Bian N, Yuan Y, Li Y, Liu M, Li X. Low-Intensity Pulsed Ultrasound Stimulation Inhibits Cortical Spreading Depression. Cerebral Cortex. 2021;31(8):3872-80. doi: 10.1093/cercor/bhab055. PubMed PMID: WOS:000674446400021.

3506. Boissonnault È, Higgins J, LaGarde G, Barthélemy D,

Lamarre C, J HD. Brain stimulation in attention deficits after traumatic brain injury: a literature review and feasibility study. Pilot Feasibility Stud. 2021;7(1):115. Epub 2021/06/02. doi: 10.1186/s40814-021-00859-3. PubMed PMID: 34059152; PubMed Central PMCID: PMCPMC8165970.

3507. Boltzmann M, Schmidt SB, Gutenbrunner C, Krauss JK, Stangel M, Höglinger GU, et al. Auditory Stimulation Modulates Resting-State Functional Connectivity in Unresponsive Wakefulness Syndrome Patients. Frontiers in Neuroscience. 2021;15. doi: 10.3389/fnins.2021.554194.

3508. Bouton C, Bhagat N, Chandrasekaran S, Herrero J, Markowitz N, Espinal E, et al. Decoding Neural Activity in Sulcal and White Matter Areas of the Brain to Accurately Predict Individual Finger Movement and Tactile Stimuli of the Human Hand. Frontiers in Neuroscience. 2021;15. doi: 10.3389/fnins.2021.699631.

3509. Cambiaghi M, Cherchi L, Masin L, Infortuna C, Briski N, Caviasco C, et al. High-frequency repetitive transcranial magnetic stimulation enhances layer II/III morphological dendritic plasticity in mouse primary motor cortex. Behav Brain Res. 2021;410:113352. Epub 2021/05/13. doi: 10.1016/j.bbr.2021.113352. PubMed PMID: 33979657.

3510. Campbell AM, Elbogen EB, Johnson JL, Hamer RM, Belger

A. Event related potentials indexing the influence of emotion on cognitive processing in veterans with comorbid post-traumatic stress disorder and traumatic brain injury. Clinical Neurophysiology. 2021;132(7):1389-97. doi: 10.1016/j.clinph.2021.03.017.

3511. Campos ACP, Berzuino MB, Barbosa GR, Freire HMRC, Lopes PS, Assis DV, et al. Motor Cortex Stimulation Reversed Hypernociception, Increased Serotonin in Raphe Neurons, and Caused Inhibition of Spinal Astrocytes in a Parkinson's Disease Rat Model. Cells. 2021;10(5). doi: 10.3390/cells10051158. PubMed PMID: WOS:000654652600001.

3512. Chakraborty S, Saetta G, Simon C, Lenggenhager B, Ruddy K. Could Brain–Computer Interface Be a New Therapeutic Approach for Body Integrity Dysphoria? Frontiers in Human Neuroscience. 2021;15. doi: 10.3389/fnhum.2021.699830.

3513. Chan H, Hogue O, Newcomb M, Hermann J, Floden D, Machado A, et al. Prefrontal improvement by deep cerebellar stimulation in a traumatic brain injury model of rat. Brain Stimulation. 2021;14(6):1714-5. doi: 10.1016/j.brs.2021.10.416.

3514. Chantigian DP, Larson M, Chen M, Gillick BT, Cross TJ, Keller-Ross ML. Heart rate and blood pressure responses to corticomotor stimulation in young, healthy adults. Clin Neurophysiol Pract. 2021;6:206-8. Epub 2021/08/12. doi:

10.1016/j.cnp.2021.06.004. PubMed PMID: 34377873; PubMed Central PMCID: PMCPMC8327479.

3515. Chavarría AP, Vázquez RRV, Cherit JGD, Bello HH, Suastegui HC, Moreno-Castañeda L, et al. Antioxidants and pentoxifylline as coadjuvant measures to standard therapy to improve prognosis of patients with pneumonia by COVID-19. Computational and Structural Biotechnology Journal. 2021;19:1379-90. doi: 10.1016/j.csbj.2021.02.009.

3516. Chen B, Lin WQ, Li ZF, Zhong XY, Wang J, You XF, et al. Electroacupuncture Attenuates Ischemic Brain Injury and Cellular Apoptosis via Mitochondrial Translocation of Cofilin. Chin J Integr Med. 2021;27(9):705-12. Epub 2021/03/13. doi: 10.1007/s11655-021-3335-4. PubMed PMID: 33709239.

3517. Chen S, Xu D, Fan L, Fang Z, Wang X, Li M. Roles of N-Methyl-D-Aspartate Receptors (NMDARs) in Epilepsy. Frontiers in Molecular Neuroscience. 2021;14. doi: 10.3389/fnmol.2021.797253.

3518. Chen XL, Yu LP, Zhu Y, Wang TY, Han J, Chen XY, et al. Combined effect of hydrotherapy and transcranial direct-current stimulation on children with cerebral palsy: A protocol for a randomized controlled trial. Medicine (Baltimore). 2021;100(49):e27962. Epub 2021/12/11. doi: 10.1097/md.0000000000027962. PubMed PMID: 34889241; PubMed

Central PMCID: PMCPMC8663893.
3519. Chen Z, Liu J, Zhao X, Zheng Z, Bai L, Yang D, et al. Effects of Electroacupuncture on Consciousness Disorders Based on Transcranial Magnetic Stimulation: Study with Mismatch Negativity. Chinese Journal of Rehabilitation Theory and Practice. 2021;27(1):102-6. PubMed PMID: CSCD:6900230.
3520. Chiang HS, Kraut M, Vanneste S, Hart J. Modulation of the superior medial frontal cortex using anodal high definition transcranial direct current stimulation affects frontal midline theta oscillations during a Go-Nogo task in veterans with chronic traumatic brain injury. Brain Stimulation. 2021;14(6):1635. doi: 10.1016/j.brs.2021.10.152.
3521. Chiang HS, Motes M, O'Hair R, Vanneste S, Kraut M, Hart J, Jr. Baseline delayed verbal recall predicts response to high definition transcranial direct current stimulation targeting the superior medial frontal cortex. Neurosci Lett. 2021;764:136204. Epub 2021/09/04. doi: 10.1016/j.neulet.2021.136204. PubMed PMID: 34478816.
3522. Chiang HS, Shakal S, Vanneste S, Kraut M, Hart J, Jr. Case Report: Improving Verbal Retrieval Deficits With High Definition Transcranial Direct Current Stimulation Targeting the Pre-Supplementary Motor Area in a Patient With Chronic Traumatic Brain Injury. Front Neurol. 2021;12:678518. Epub 2021/08/03. doi: 10.3389/fneur.2021.678518. PubMed PMID: 34335447; PubMed

Central PMCID: PMCPMC8322436.
3523. ChiCtr. The awakening effect of neuromodulation technology on patients with disorders of consciousness: a multi-center, double blind, randomized controlled clinical trial. https://trialsearchwhoint/Trial2aspx?TrialID=ChiCTR2100043784. 2021. PubMed PMID: CN-02280865.
3524. ChiCtr. Repeated transcranial direct current stimulation in disorders of consciousness: a randomized, double-blind controlled trial study. https://trialsearchwhoint/Trial2aspx?TrialID=ChiCTR2100053108. 2021. PubMed PMID: CN-02349387.
3525. ChiCtr. Effectiveness of transcranial magnetic stimulation on awaking patients with disorders of consciousness: a randomized controlled trial. https://trialsearchwhoint/Trial2aspx?TrialID=ChiCTR2100044930. 2021. PubMed PMID: CN-02349205.
3526. Choi Y, Jung IC, Kim AR, Park HJ, Kwon O, Lee JH, et al. Feasibility and effect of electroacupuncture on cognitive function domains in patients with mild cognitive impairment: A pilot exploratory randomized controlled trial. Brain Sciences. 2021;11(6). doi: 10.3390/brainsci11060756.
3527. Chou P, Kuo CC. Anticonvulsant vs. Proconvulsant Effect of

in situ Deep Brain Stimulation at the Epileptogenic Focus. Frontiers in Systems Neuroscience. 2021;15. doi: 10.3389/fnsys.2021.607450.

3528. Claassen J, Akbari Y, Alexander S, Bader MK, Bell K, Bleck TP, et al. Proceedings of the First Curing Coma Campaign NIH Symposium: Challenging the Future of Research for Coma and Disorders of Consciousness. Neurocritical Care. 2021;35(SUPPL 1):4-23. doi: 10.1007/s12028-021-01260-x. PubMed PMID: WOS:000671346400002.

3529. Conte G, Sbaraini S, Morelli C, Casale S, Caschera L, Contarino VE, et al. A susceptibility-weighted imaging qualitative score of the motor cortex may be a useful tool for distinguishing clinical phenotypes in amyotrophic lateral sclerosis. European Radiology. 2021;31(3):1281-9. doi: 10.1007/s00330-020-07239-0.

3530. Contreras Lopez WO, Navarro PA, Gouveia FV, Fonoff ET, Lebrun I, Auada AVV, et al. Directional Deep Brain Stimulation of the Posteromedial Hypothalamus for Refractory Intermittent Explosive Disorder: A Case Series Using a Novel Neurostimulation Device and Intraoperative Microdialysis. World Neurosurgery. 2021;155:e19-e33. doi: 10.1016/j.wneu.2021.07.086.

3531. Coulborn S, Taylor C, Naci L, Owen AM, Fernández-Espejo D. Disruptions in effective connectivity within and between default mode network and anterior forebrain mesocircuit in prolonged

disorders of consciousness. Brain Sciences. 2021;11(6). doi: 10.3390/brainsci11060749.

3532. D'Arcy RCN, Sandhu JK, Marshall S, Besemann M. Mitigating Long-Term COVID-19 Consequences on Brain Health. Frontiers in Neurology. 2021;12. doi: 10.3389/fneur.2021.630986.

3533. Daia C, Scheau C, Spinu A, Andone I, Popescu C, Toader C, et al. Modulated neuroprotection in unresponsive wakefulness syndrome after severe traumatic brain injury. Brain Sciences. 2021;11(8). doi: 10.3390/brainsci11081044.

3534. Daood U, Balasankar MP, Ibrahim MA, Pichika MR, Mak K-K, Fawzy AS. PLGA nanoparticles loaded with quaternary ammonium silane and riboflavin for potential applications in adhesive dentistry. International Journal of Adhesion and Adhesives. 2021;105. doi: 10.1016/j.ijadhadh.2020.102797. PubMed PMID: WOS:000611906600006.

3535. Darmani G, Nieminen JO, Bergmann TO, Ramezanpour H, Ziemann U. A degraded state of consciousness in healthy awake humans? Brain Stimulation. 2021;14(3):710-2. doi: 10.1016/j.brs.2021.04.012. PubMed PMID: WOS:000654712000033.

3536. Das S, Nalini A, Laxmi TR, Raju TR. ALS-CSF-induced structural changes in spinal motor neurons of rat pups cause deficits in motor behaviour. Experimental Brain Research. 2021;239(1):315-27.

doi: 10.1007/s00221-020-05969-7.

3537. De Andrés J, Navarrete-Rueda F, Fabregat G, García-Gutiérrez MS, Monsalve-Dolz V, Harutyunyan A, et al. Differences in Gene Expression of Endogenous Opioid Peptide Precursor, Cannabinoid 1 and 2 Receptors and Interleukin Beta in Peripheral Blood Mononuclear Cells of Patients With Refractory Failed Back Surgery Syndrome Treated With Spinal Cord Stimulation: Markers of Therapeutic Outcomes? Neuromodulation. 2021;24(1):49-60. Epub 2020/02/07. doi: 10.1111/ner.13111. PubMed PMID: 32027775.

3538. De Freitas DJ, De Carvalho D, Paglioni VM, Brunoni AR, Valiengo L, Thome-Souza MS, et al. Effects of transcranial direct current stimulation (tDCS) and concurrent cognitive training on episodic memory in patients with traumatic brain injury: a double-blind, randomised, placebo-controlled study. BMJ Open. 2021;11(8):e045285. Epub 2021/08/28. doi: 10.1136/bmjopen-2020-045285. PubMed PMID: 34446480; PubMed Central PMCID: PMCPMC8395342.

3539. De Martino ML, De Bartolo M, Leemhuis E, Pazzaglia M. Rebuilding body–brain interaction from the vagal network in spinal cord injuries. Brain Sciences. 2021;11(8). doi: 10.3390/brainsci11081084.

3540. Deblieck C, Smeijers S, Morlion B, Datta A, Thomas C, Theys T. Case Report: Initial Evidence of Safety and Efficacy of High Definition-Transcranial Direct Current Stimulation in a Patient With Neuropathic Pain and Implanted Spinal Cord Stimulator. Front Pain Res (Lausanne). 2021;2:753464. Epub 2022/03/18. doi: 10.3389/fpain.2021.753464. PubMed PMID: 35295503; PubMed Central PMCID: PMCPMC8915614.

3541. Deli A, He S, Huang Y, Martin S, Aziz T, Denison T, et al. Entraining Human Wakefulness: Selective, Closed-Loop Stimulation of Brainstem Arousal Circuits. Stereotactic and Functional Neurosurgery. 2021;99(SUPPL 1):109. doi: 10.1159/000520618.

3542. Deltombe T, Schillebeeckx F, Ip A, Schinwelski M, Teixeira JM, Ashford S, et al. Worldwide clinician survey on practice patterns and perceptions on use of adjunct therapies following botulinum toxin injection for limb spasticity. Toxicon. 2021;190:S15-S6. doi: 10.1016/j.toxicon.2020.11.371.

3543. Deng ZD, McClintock S, Husain M, Lisanby S. Antidepressant response of electroconvulsive therapy and magnetic seizure therapy: response trajectories by symptom clusters. Neuropsychopharmacology. 2021;46:226-. doi: 10.1038/s41386-021-01237-6. PubMed PMID: CN-02356325.

3544. Di Gregorio F, La Porta F, Lullini G, Casanova E, Petrone V,

Simoncini L, et al. Efficacy of Repetitive Transcranial Magnetic Stimulation Combined With Visual Scanning Treatment on Cognitive-Behavioral Symptoms of Unilateral Spatial Neglect in Patients With Traumatic Brain Injury: Study Protocol for a Randomized Controlled Trial. Front Neurol. 2021;12:702649. Epub 2021/08/03. doi: 10.3389/fneur.2021.702649. PubMed PMID: 34335455; PubMed Central PMCID: PMCPMC8317998.

3545. Di Natale C, De Rosa D, Profeta M, Jamaledin R, Attanasio A, Lagreca E, et al. Design of biodegradable bi-compartmental microneedles for the stabilization and the controlled release of the labile molecule collagenase for skin healthcare. Journal of Materials Chemistry B. 2021;9(2):392-403. doi: 10.1039/d0tb02279a. PubMed PMID: WOS:000609867400016.

3546. Diep D, Lam ACL, Ko G. A Review of the Evidence and Current Applications of Portable Translingual Neurostimulation Technology. Neuromodulation. 2021;24(8):1377-87. doi: 10.1111/ner.13260. PubMed PMID: WOS:000566476900001.

3547. Dolev I. Delphi in the detection of neurological conditions and white matter pathologies. Journal of the Neurological Sciences. 2021;429. doi: 10.1016/j.jns.2021.118327.

3548. Dong J, Wang L, Xiong R, Liu X, Guo Z, Sun W, et al. [A 180-day mortality predictive score based on frailty syndrome in

elderly patients with sepsis: a Logistic regression analysis model]. Zhonghua Wei Zhong Bing Ji Jiu Yi Xue. 2021;33(3):257-62. Epub 2021/04/10. doi: 10.3760/cma.j.cn121430-20201027-00688. PubMed PMID: 33834964.

3549. Economidou SN, Uddin MJ, Marques MJ, Douroumis D, Sow WT, Li H, et al. A novel 3D printed hollow microneedle microelectromechanical system for controlled, personalized transdermal drug delivery. Additive Manufacturing. 2021;38. doi: 10.1016/j.addma.2020.101815. PubMed PMID: WOS:000617202300043.

3550. Edlow BL, Sanz LRD, Polizzotto L, Pouratian N, Rolston JD, Snider SB, et al. Therapies to Restore Consciousness in Patients with Severe Brain Injuries: A Gap Analysis and Future Directions. Neurocritical Care. 2021;35(SUPPL 1):68-85. doi: 10.1007/s12028-021-01227-y. PubMed PMID: WOS:000671346400007.

3551. Eilam-Stock T, George A, Charvet L. Telerehabilitation of Cognitive Functioning with Combined Transcranial Direct Current Stimulation and Computerized Adaptive Cognitive Training in Traumatic Brain Injury: A Case Report. Neurology. 2021;96(15). PubMed PMID: WOS:000729283604283.

3552. Eilam-Stock T, George A, Charvet LE. Cognitive

Telerehabilitation with Transcranial Direct Current Stimulation Improves Cognitive and Emotional Functioning Following a Traumatic Brain Injury: A Case Study. Arch Clin Neuropsychol. 2021;36(3):442-53. Epub 2021/04/23. doi: 10.1093/arclin/acaa059. PubMed PMID: 33885138.

3553. Engelhardt M, Karhu J, Picht T. Protocol for mapping of the supplementary motor area using repetitive navigated transcranial magnetic stimulation. Brain Stimulation. 2021;14(6):1670. doi: 10.1016/j.brs.2021.10.261.

3554. Eskildsen SJ, Poulsen I, Jakobsen D, Riberholt CG, Curtis DJ. Scoping review to identify and map non-pharmacological, non-surgical treatments for dysphagia following moderate-to-severe acquired brain injury. BMJ Open. 2021;11(12):e053244. Epub 2021/12/04. doi: 10.1136/bmjopen-2021-053244. PubMed PMID: 34857571; PubMed Central PMCID: PMCPMC8640633.

3555. Evans TC, DeGutis J, Rothlein D, Jagger-Rickels A, Yamashita A, Fortier CB, et al. Punishment and reward normalize error-related cognitive control in PTSD by modulating salience network activation and connectivity. Cortex. 2021;145:295-314. doi: 10.1016/j.cortex.2021.09.004.

3556. Fan K, Zeng L, Guo J, Xie S, Yu Y, Chen J, et al. Visualized podocyte-targeting and focused ultrasound responsive glucocorticoid

nano-delivery system against immune-associated nephropathy without glucocorticoid side effect. Theranostics. 2021;11(6):2670-90. doi: 10.7150/THNO.53083.

3557. Fann JR, Quinn DK, Hart T. Treatment of Psychiatric Problems After Traumatic Brain Injury. Biological psychiatry. 2021. doi: 10.1016/j.biopsych.2021.07.008. PubMed PMID: CN-02326463.

3558. Fatih P, Kucuker MU, Vande Voort JL, Doruk Camsari D, Farzan F, Croarkin PE. A Systematic Review of Long-Interval Intracortical Inhibition as a Biomarker in Neuropsychiatric Disorders. Frontiers in Psychiatry. 2021;12. doi: 10.3389/fpsyt.2021.678088.

3559. Fekete K, Tóth J, Horváth L, Márton S, Héja M, Csiba L, et al. Neurophysiological Examinations as Adjunctive Tool to Imaging Techniques in Spontaneous Intracerebral Hemorrhage: IRONHEART Study. Front Neurol. 2021;12:757078. Epub 2021/11/16. doi: 10.3389/fneur.2021.757078. PubMed PMID: 34777228; PubMed Central PMCID: PMCPMC8578792.

3560. Feller D, Vinante C, Trentin F, Innocenti T. The effectiveness of median nerve electrical stimulation in patients with disorders of consciousness: a systematic review. Brain Inj. 2021;35(4):385-94. Epub 2021/02/23. doi: 10.1080/02699052.2021.1887522. PubMed PMID: 33617359.

3561. Feng YH, Zhang XP, Li WX, Guo XD. Stability and Diffusion

Properties of Insulin in Dissolvable Microneedles: A Multiscale Simulation Study. Langmuir. 2021;37(30):9244-52. doi: 10.1021/acs.langmuir.1c01434. PubMed PMID: WOS:000683367100038.
3562. Fernández-Galán E, Rodriguez M, García De Herreros M, Pineda E, Martínez D, Nomdedeu M, et al. Role of s100 in the diagnosis of bone marrow metastasis from glioblastoma: A case report. Clinical Chemistry and Laboratory Medicine. 2021;59(SUPPL 1):S268. doi: 10.1515/cclm-2021-5013.
3563. Flanagan SD, Proessl F, Dunn-Lewis C, Sterczala AJ, Connaboy C, Canino MC, et al. Differences in brain structure and theta burst stimulation-induced plasticity implicate the corticomotor system in loss of function after musculoskeletal injury. Journal of neurophysiology. 2021;125(4):1006-21. doi: 10.1152/jn.00689.2020. PubMed PMID: CN-02259029.
3564. Fonseca DFS, Vilela C, Pinto RJB, Bastos V, Oliveira H, Catarino J, et al. Bacterial nanocellulose-hyaluronic acid microneedle patches for skin applications: In vitro and in vivo evaluation. Materials Science & Engineering C-Materials for Biological Applications. 2021;118. doi: 10.1016/j.msec.2020.111350. PubMed PMID: WOS:000600850700004.
3565. Formica C, De Salvo S, Corallo F, Alagna A, Logiudice AL,

Todaro A, et al. Role of neurorehabilitative treatment using transcranial magnetic stimulation in disorders of consciousness. J Int Med Res. 2021;49(2):300060520976472. Epub 2021/02/05. doi: 10.1177/0300060520976472. PubMed PMID: 33535855; PubMed Central PMCID: PMCPMC7869152.

3566. Fragío Gil JJ, Grau García E, Fernández Matilla M, Ortiz Sanjuan FM, Rodrigo Nicolás R, Román Ivorra JA. Decreased DNA hydroxymethylation and increased DNA demethylation are associated with high antioxidant response in systemic lupus erythematosus patients. Medicina Clinica. 2021;157(12):575-9. doi: 10.1016/j.medcli.2020.10.023.

3567. Frohlich J, Toker D, Monti MM. Consciousness among delta waves: a paradox? Brain. 2021;144:2257-77. doi: 10.1093/brain/awab095. PubMed PMID: WOS:000710930500015.

3568. Furlan JC, Pakosh M, Craven BC, Popovic MR. Insights on the Potential Mechanisms of Action of Functional Electrical Stimulation Therapy in Combination With Task-Specific Training: A Scoping Review. Neuromodulation. 2021. Epub 2021/05/26. doi: 10.1111/ner.13403. PubMed PMID: 34031937.

3569. Galafassi GZ, Simm Pires de Aguiar PH, Simm RF, Franceschini PR, Filho MP, Pagura JR, et al. Neuromodulation for Medically Refractory Neuropathic Pain: Spinal Cord Stimulation,

Deep Brain Stimulation, Motor Cortex Stimulation, and Posterior Insula Stimulation. World Neurosurg. 2021;146:246-60. Epub 2020/11/21. doi: 10.1016/j.wneu.2020.11.048. PubMed PMID: 33217591.

3570. Gatzinsky K, Bergh C, Liljegren A, Silander H, Samuelsson J, Svanberg T, et al. Repetitive transcranial magnetic stimulation of the primary motor cortex in management of chronic neuropathic pain: a systematic review. Scand J Pain. 2021;21(1):8-21. Epub 2020/09/07. doi: 10.1515/sjpain-2020-0054. PubMed PMID: 32892189.

3571. Gazaille C, Sicot M, Akiki M, Lautram N, Dupont A, Saulnier P, et al. Characterization of Biological Material Adsorption to the Surface of Nanoparticles without a Prior Separation Step: a Case Study of Glioblastoma-Targeting Peptide and Lipid Nanocapsules. Pharmaceutical Research. 2021;38(4):681-91. doi: 10.1007/s11095-021-03034-8. PubMed PMID: WOS:000637660100002.

3572. Ge X, Zhang Y, Xin T, Luan X. Effects of 10 Hz repetitive transcranial magnetic stimulation of the right dorsolateral prefrontal cortex in the vegetative state. Exp Ther Med. 2021;21(3):206. Epub 2021/01/28. doi: 10.3892/etm.2021.9626. PubMed PMID: 33500699; PubMed Central PMCID: PMCPMC7818534.

3573. Gracefire P. Advances in photobiomodulation using a

closed-loop design. NeuroRegulation. 2021;8(4):203-4. doi: 10.15540/nr.8.4.198.

3574. Granero OP, Cheema S, Lagrata S, Hyam J, Zrinzo L, Matharu M, et al. Long-Term Outcomes of Ventral Tegmental Area Deep Brain Stimulation for Trigeminal Autonomic Cephalalgias. Stereotactic and Functional Neurosurgery. 2021;99(SUPPL 1):128. doi: 10.1159/000520618.

3575. Guo Y, Liu C, Yang Y, Li Y, Shan Q, Xie J, et al. Effect of long-lasting repeated high-definition transcranial direct current stimulation on consciousness recovery in minimally conscious state patients. Journal of Third Military Medical University. 2021;43(15):1454-9. PubMed PMID: CSCD:7015498.

3576. Hall MR, Mathiasen R. Phonate a friend. Clinical Journal of Sport Medicine. 2021;31(2):e35. doi: 10.1097/JSM.0000000000000917.

3577. Han K, Dang H, Zhang H. Efficacy of Repetitive Transcranial Magnetic Stimulation on Cognitive Dysfunction after Traumatic Brain Injury: AMeta-analysis. Chinese Journal of Rehabilitation Theory and Practice. 2021;27(11):1282-90. PubMed PMID: CSCD:7111816.

3578. Hara T, Shanmugalingam A, McIntyre A, Burhan A. The Effect of Non-Invasive Brain Stimulation (NIBS) on Executive Functioning, Attention and Memory in Rehabilitation Patients with

Traumatic Brain Injury: a Systematic Review and Meta-analysis. Brain Stimulation. 2021;14(6):1716-7. doi: 10.1016/j.brs.2021.10.422.

3579. Hara T, Shanmugalingam A, McIntyre A, Burhan AM. The Effect of Non-Invasive Brain Stimulation (NIBS) on Executive Functioning, Attention and Memory in Rehabilitation Patients with Traumatic Brain Injury: A Systematic Review. Diagnostics (Basel). 2021;11(4). Epub 2021/04/04. doi: 10.3390/diagnostics11040627. PubMed PMID: 33807188; PubMed Central PMCID: PMCPMC8066265.

3580. Hardy DJ. Neuroergonomics: A perspective from neuropsychology, with a proposal about workload. Brain Sciences. 2021;11(5). doi: 10.3390/brainsci11050647.

3581. Hartikainen KM. Emotion-attention interaction in the right hemisphere. Brain Sciences. 2021;11(8). doi: 10.3390/brainsci11081006.

3582. Hays S, Swank C, Rennaker R, French J. Vagus nerve stimulation paired with rehabilitation to improve recovery of upper extremity function in stroke and spinal cord injury. Neurorehabilitation and Neural Repair. 2021;35(11):NP16. doi: 10.1177/15459683211027047.

3583. He RH, Wang HJ, Zhou Z, Fan JZ, Zhang SQ, Zhong YH. The influence of high-frequency repetitive transcranial magnetic

stimulation on endogenous estrogen in patients with disorders of consciousness. Brain Stimul. 2021;14(3):461-6. Epub 2021/03/08. doi: 10.1016/j.brs.2021.02.014. PubMed PMID: 33677157.

3584. Herrity AN, Aslan SC, Ugiliweneza B, Mohamed AZ, Hubscher CH, Harkema SJ. Improvements in Bladder Function Following Activity-Based Recovery Training With Epidural Stimulation After Chronic Spinal Cord Injury. Frontiers in Systems Neuroscience. 2021;14. doi: 10.3389/fnsys.2020.614691.

3585. Herrold A, Livengood S, Siddiqi S, Pape TB. Customizing rTMS treatment targeting for co-occurring alcohol use disorder and mild traumatic brain injury using multi-modal neuroimaging data as neurobiological markers. Brain Stimulation. 2021;14(6):1721. doi: 10.1016/j.brs.2021.10.437.

3586. Heslot C, Cogné M, Guillouët E, Perdrieau V, Lefevre-Dognin C, Glize B, et al. Management of unfavorable outcome after mild traumatic brain injury: Review of physical and cognitive rehabilitation and of psychological care in post-concussive syndrome. Neurochirurgie. 2021;67(3):283-9. Epub 2020/10/14. doi: 10.1016/j.neuchi.2020.09.001. PubMed PMID: 33049290.

3587. Horton GA, Ibrahim O, Jansen M, Trier J, Milev R, Beyea JA. Repetitive Transcranial Magnetic Stimulation For The Treatment of Chronic Tinnitus: A Preliminary Study of The Influence of Traumatic

Brain Injury on Treatment Response. Int Tinnitus J. 2021;25(1):51-8. Epub 2021/08/20. doi: 10.5935/0946-5448.20210011. PubMed PMID: 34410080.

3588. Hosseinian T, Yavari F, Biagi MC, Kuo MF, Ruffini G, Nitsche MA, et al. External induction and stabilization of brain oscillations in the human. Brain Stimul. 2021;14(3):579-87. Epub 2021/03/31. doi: 10.1016/j.brs.2021.03.011. PubMed PMID: 33781955; PubMed Central PMCID: PMCPMC8144019.

3589. Huang LD, Sung KB. COVID-encephalopathy treatment with transcranial near infrared laser stimulation. Brain Stimulation. 2021;14(6):1660. doi: 10.1016/j.brs.2021.10.227.

3590. Huffman S, Badran B, Dancy M, Austelle C, Kautz S, George M. At-Home Telemedicine Controlled taVNS Twice Daily for 4 weeks is Feasible and Safe for Long COVID Symptoms. Brain stimulation. 2021;14(6):1702-3. doi: 10.1016/j.brs.2021.10.367. PubMed PMID: CN-02345944.

3591. Hümmer K, Coenen J, Konstantinidis I, Lausberg H, Helmich I. Impaired recognition of nonverbal expressions of emotions in depressed individuals. Psychiatry Research. 2021;302. doi: 10.1016/j.psychres.2021.114031.

3592. Hunt S, Moss H, Dancy M, Badran BW, George MS, Jenkins D. Predicting response to transcutaneous auricular vagus nerve

stimulation (taVNS) paired with oromotor feeding in neonates: CNS metabolite biomarkers by MR spectroscopy (MRS). Brain Stimulation. 2021;14(6):1664-5. doi: 10.1016/j.brs.2021.10.243.
3593. Huynh-Le MP, Tibbs MD, Karunamuni R, Salans M, Tringale KR, Yip A, et al. Microstructural Injury to Corpus Callosum and Intrahemispheric White Matter Tracts Correlate With Attention and Processing Speed Decline After Brain Radiation. International Journal of Radiation Oncology Biology Physics. 2021;110(2):337-47. doi: 10.1016/j.ijrobp.2020.12.046.
3594. Imperatore JP, McCalley DM, Borckardt JJ, Brady KT, Hanlon CA. Non-invasive brain stimulation as a tool to decrease chronic pain in current opiate users: A parametric evaluation of two promising cortical targets. Drug and Alcohol Dependence. 2021;218. doi: 10.1016/j.drugalcdep.2020.108409.
3595. Inanici F, Brighton LN, Samejima S, Hofstetter CP, Moritz CT. Transcutaneous Spinal Cord Stimulation Restores Hand and Arm Function after Spinal Cord Injury. IEEE Transactions on Neural Systems and Rehabilitation Engineering. 2021;29:310-9. doi: 10.1109/TNSRE.2021.3049133.
3596. Isagulyan E, Tomsky A, Makashova E, Mikhailova V, Dorochov E, Salova E. Neuromodulation in Treating Complex Regional Pain Syndrome. Stereotactic and Functional Neurosurgery.

2021;99(SUPPL 1):119. doi: 10.1159/000520618.

3597. Ishii D, Ishibashi K, Takeda K, Yuine H, Yamamoto S, Kaku Y, et al. Interaction of the Left–Right Somatosensory Pathways in Patients With Thalamic Hemorrhage: A Case Report. Frontiers in Human Neuroscience. 2021;15. doi: 10.3389/fnhum.2021.761186.

3598. Ito S, Itoh H, Rakugi H, Okuda Y, Iijima S. Antihypertensive effects and safety of esaxerenone in patients with moderate kidney dysfunction. Hypertension Research. 2021;44(5):489-97. doi: 10.1038/s41440-020-00585-y.

3599. Jerban S, Kasibhatla A, Ma Y, Wu M, Chen Y, Guo T, et al. Detecting Articular Cartilage and Meniscus Deformation Effects Using Magnetization Transfer Ultrashort Echo Time (MT-UTE) Modeling during Mechanical Load Application: Ex Vivo Feasibility Study. Cartilage. 2021;13(1_suppl):665S-73S. doi: 10.1177/1947603520976771.

3600. Jerban S, Ma Y, Kasibhatla A, Wu M, Szeverenyi N, Guma M, et al. Ultrashort echo time adiabatic T1ρ (UTE-Adiab-T1ρ) is sensitive to human cadaveric knee joint deformation induced by mechanical loading and unloading. Magnetic Resonance Imaging. 2021;80:98-105. doi: 10.1016/j.mri.2021.04.014.

3601. Johnstone A, Grigoras I, Petitet P, Capitao LP, Stagg CJ. A single, clinically relevant dose of the GABA(B) agonist baclofen

impairs visuomotor learning. Journal of Physiology-London. 2021;599(1):307-22. doi: 10.1113/jp280378. PubMed PMID: WOS:000584821600001.

3602. Juárez-Belaúnde A, Orcajo E, Lejarreta S, Davila-Pérez P, León N, Oliviero A. Fatigue in patients with acquired brain damage. Neurologia (Engl Ed). 2021. Epub 2021/09/21. doi: 10.1016/j.nrl.2021.05.011. PubMed PMID: 34538507.

3603. Jung JY. The neural mechanisms of plasticity in semantic cognition: Variable neuro-displacement hypothesis. Asia-Pacific Psychiatry. 2021;13(SUPPL 1). doi: 10.1111/appy.12459.

3604. Kahana M, Wanda P, Ezzyat Y, Adamovich-Zeitlin R, Lega B, Jobst B, et al. Closed-Loop Neuromodulation Restores Memory after Traumatic Brain Injury. Neuromodulation. 2021;24(4):e249-e50. doi: 10.1111/ner.13385.

3605. Kalita J, Kumar S, Singh VK, Misra UK. A randomized controlled trial of high rate rtms versus rtms and amitriptyline in chronic migraine. Pain Physician. 2021;24(6):E733-E41. doi: 10.36076/ppj.2021.24.e733.

3606. Kamali AM, Gholamzadeh MJ, Mousavi SZ, Gharamaleki MV, Nami M. Improved Visual Function in a Case of Ultra-low Vision following Ischemic Encephalopathy Using Transcranial Electrical Stimulation; A Brief Report. Developmental Neurorehabilitation.

2021;24(1):68-72. doi: 10.1080/17518423.2020.1839978.

3607. Kang X, Herron T, Coetzee J, Adamson M. Effect of rTMS on fractional anisotropy, radial diffusivity, and axonal volume in select fiber tracts in patients with traumatic brain injury. Brain Stimulation. 2021;14(6):1639. doi: 10.1016/j.brs.2021.10.164.

3608. Kavakcı M. Efficacy of non-invasive brain stimulation methods for the treatment of cognitive impairment in traumatic brain injury and stroke. Turk Noroloji Dergisi. 2021;27(1):17-20. doi: 10.4274/tnd.2021.90688.

3609. Kct. The effect of personalized and optimized transcranial direct current stimulation on consciousness in patients with severe unconsciousness (minimal unconsciousness state). https://trialsearchwhoint/Trial2aspx?TrialID=KCT0006665. 2021. PubMed PMID: CN-02351977.

3610. Kierońska S, Świtońska M, Meder G, Piotrowska M, Sokal P. Tractography alterations in the arcuate and uncinate fasciculi in post-stroke aphasia. Brain Sciences. 2021;11(1):1-12. doi: 10.3390/brainsci11010053.

3611. Kinfe T, Buchfelder M. Incisionless MR-Guided Focused Ultrasound: Technical Considerations and Current Approaches in Psychiatric Disorders. Stereotactic and Functional Neurosurgery. 2021;99(SUPPL 1):132-3. doi: 10.1159/000520618.

3612. Köhnen M, Kriston L, Härter M, Baumeister H, Liebherz S. Effectiveness and acceptance of technology-based psychological interventions for the acute treatment of unipolar depression: Systematic review and meta-analysis. Journal of Medical Internet Research. 2021;23(6). doi: 10.2196/24584.

3613. Kolchina AN, Yatsyshina EE, Malysheva LV, Ledentsova EE, Lidyaeva EE, Khaletskaya OV. Diagnostics of Inherited Metabolic Diseases in Newborns with the Hyperammonemia Syndrome at the Onset of Disease (Pilot Study). Sovrem Tekhnologii Med. 2021;13(1):59-64. Epub 2021/09/14. doi: 10.17691/stm2021.13.1.07. PubMed PMID: 34513067; PubMed Central PMCID: PMCPMC8353695.

3614. Krenzlin H, Wesp D, Schmitt J, Frenz C, Kurz E, Masomi-Bornwasser J, et al. Decreased superoxide dismutase concentrations (Sod) in plasma and csf and increased circulating total antioxidant capacity (tac) are associated with unfavorable neurological outcome after aneurysmal subarachnoid hemorrhage. Journal of Clinical Medicine. 2021;10(6):1-10. doi: 10.3390/jcm10061188.

3615. Kullakçi H, Sonkaya AR. The investigation of the effects of repetitive transcranialmagnetic stimulation treatment on taste and smell sensations in depressed patients. Noropsikiyatri Arsivi. 2021;58(1):26-33. doi: 10.29399/npa.25087.

3616. Kuo CW, Chang MY, Liu HH, He XK, Chan SY, Huang YZ, et al. Cortical Electrical Stimulation Ameliorates Traumatic Brain Injury-Induced Sensorimotor and Cognitive Deficits in Rats. Front Neural Circuits. 2021;15:693073. Epub 2021/07/02. doi: 10.3389/fncir.2021.693073. PubMed PMID: 34194304; PubMed Central PMCID: PMCPMC8236591.
3617. Kurtin DL, Violante IR, Zimmerman K, Leech R, Hampshire A, Patel MC, et al. Investigating the interaction between white matter and brain state on tDCS-induced changes in brain network activity. Brain Stimul. 2021;14(5):1261-70. Epub 2021/08/27. doi: 10.1016/j.brs.2021.08.004. PubMed PMID: 34438046; PubMed Central PMCID: PMCPMC8460997.
3618. Lam D, Bogguri C, Cadena J, Ladd A, Sebastian A, Enright H, et al. Do reprogrammed brain cells hold the key to healing damaged networks after traumatic brain injury? Journal of Neurotrauma. 2021;38(14):A70. doi: 10.1089/neu.2021.29111.abstracts.
3619. Lan Q, Guo R, Chang J, Zheng J, Yu K, Chen J, editors. Fast Prediction of RF-induced Heating for Sacral Neuromodulation System Exposed to Multi-Channel 2 RF Field at 3T MRI. Proceedings of the Annual International Conference of the IEEE Engineering in Medicine and Biology Society, EMBS; 2021.
3620. Larsen J, Güler DB, Andersen MS, Rich K, Damm MV, Zhang

M, et al. Plasticity of cholinergic system in the spinal cord following traumatic brain injury. Clinical Neuropathology. 2021;40(4):S107-S8. doi: 10.5414/NPP40M01.

3621. Leisman G, Machado C, Melillo R. Cortical visual impairment in childhood: 'blindsight' and the sprague effect revisited. Brain Sciences. 2021;11(10). doi: 10.3390/brainsci11101279.

3622. Leung A. TMS for Persistent Post Traumatic Brain Injury Headache-A neuropathic Pain State. Brain Stimulation. 2021;14(6):1730. doi: 10.1016/j.brs.2021.10.470.

3623. Li D, Hu D, Xu H, Patra HK, Liu X, Zhou Z, et al. Progress and perspective of microneedle system for anti-cancer drug delivery. Biomaterials. 2021;264. doi: 10.1016/j.biomaterials.2020.120410. PubMed PMID: WOS:000587813600012.

3624. Li J, Curley WH, Guerin B, Dougherty DD, Dalca AV, Fischl B, et al. Mapping the subcortical connectivity of the human default mode network. Neuroimage. 2021;245:118758. Epub 2021/11/29. doi: 10.1016/j.neuroimage.2021.118758. PubMed PMID: 34838949; PubMed Central PMCID: PMCPMC8945548.

3625. Li LP, Chen JK, Coelho DH. Optimizing Location of Subdermal Recording Electrodes for Intraoperative Facial Nerve Monitoring. Laryngoscope. 2021;131(7):E2329-e34. Epub 2021/03/23. doi: 10.1002/lary.29518. PubMed PMID: 33749869.

3626. Li R. Study on the Process of Consciousness Recovery in DOC Patients Based on Microstate. International Journal of Psychophysiology. 2021;168:S204. doi: 10.1016/j.ijpsycho.2021.07.554.

3627. Li S, Xia D, Prausnitz MR. Efficient Drug Delivery into Skin Using a Biphasic Dissolvable Microneedle Patch with Water-Insoluble Backing. Advanced Functional Materials. 2021;31(44). doi: 10.1002/adfm.202103359. PubMed PMID: WOS:000664463100001.

3628. Li W, Yang Y, Huang J, Zhang H, Zeng D, Jiang L, et al. Electroacupuncture-induced plasticity between face and hand representations in motor cortex is associated with recovery of function after facial nerve injury. Acupunct Med. 2021;39(1):75-7. Epub 2020/06/17. doi: 10.1177/0964528420920298. PubMed PMID: 32539425.

3629. Li Y, He J, Yang B, Zhang H, Yang Z, Fu J, et al. Guidelines of clinical diagnosis and neurorestorative treatment for chronic disorders of consciousness (2021 China version). Journal of Neurorestoratology. 2021;9(1):50-9. doi: 10.26599/JNR.2021.9040006.

3630. Lisanby S. Non-invasive brain stimulation and psychiatric symptoms in neurological disorders. Journal of the Neurological Sciences. 2021;429. doi: 10.1016/j.jns.2021.117962. PubMed PMID:

WOS:000713637300325.

3631. Liu L, Wang Q, Liao H, Ye J, Huang J, Li S, et al. Soluble microneedle patch with photothermal and NO-release properties for painless and precise treatment of ischemic perforator flaps. Journal of Materials Chemistry B. 2021;9(37):7725-33. doi: 10.1039/d1tb00491c. PubMed PMID: WOS:000692365400001.

3632. Lo Re V, Varoli E, Hassan G, Avorio F, Mamone G, De Salvo S, et al. Role of transcranial magnetic stimulation (TMS) combined with electroencephalography (EEG) in disorders of consciousness (DOC). Journal of the Neurological Sciences. 2021;429. doi: 10.1016/j.jns.2021.118507. PubMed PMID: WOS:000713637301325.

3633. Longley V, Hazelton C, Heal C, Pollock A, Woodward-Nutt K, Mitchell C, et al. Non-pharmacological interventions for spatial neglect or inattention following stroke and other non-progressive brain injury. Cochrane Database of Systematic Reviews. 2021;2021(7). doi: 10.1002/14651858.CD003586.pub4.

3634. Longo MG, Tan CO, Chan ST, Welt J, Ahmadi E, Ratai E, et al. Effect of Transcranial Low-Level Light Therapy Among Patients With Moderate Traumatic Brain Injury. Biological psychiatry. 2021;89(9):S68-S9. doi: 10.1016/j.biopsych.2021.02.186. PubMed PMID: CN-02298120.

3635. Lopez-Ramirez MA, Kupor D, Marchiori L, Soto F, Rueda R,

Reynoso M, et al. Combinatorial microneedle patch with tunable release kinetics and dual fast-deep/sustained release capabilities. Journal of Materials Chemistry B. 2021;9(9):2189-99. doi: 10.1039/d1tb00141h. PubMed PMID: WOS:000627506200004.

3636. Lorente L, Martín MM, Pérez-Cejas A, González-Rivero AF, Sabatel R, Ramos-Gómez L, et al. High serum levels of TAC and early mortality in patients with spontaneous intracerebral haemorrhage. Neurological Sciences. 2021;42(4):1491-7. doi: 10.1007/s10072-020-04692-z.

3637. Loveridge A, Smith DS, McGeer JC. Dissolved Organic Matter Mitigates the Acute Toxicity of Thulium to Hyalella azteca but Ca, Mg and Na Do Not. Archives of Environmental Contamination and Toxicology. 2021;81(4):637-47. doi: 10.1007/s00244-021-00898-0.

3638. Luo Y, Granzhan A, Verga D, Mergny JL. FRET-MC: A fluorescence melting competition assay for studying G4 structures in vitro. Biopolymers. 2021;112(4). doi: 10.1002/bip.23415.

3639. Lv Y, Dai DC, Jiang HN, Wang L. [Effect of electroacupuncture on lumbar disc herniation with different multifidus fatty infiltration rates]. Zhongguo Zhen Jiu. 2021;41(6):593-7. Epub 2021/06/05. doi: 10.13703/j.0255-2930.20200817-k0006. PubMed PMID: 34085473.

3640. Ma C. The Research Progress of Neuromodulation on Wake-promoting in Patients with Disorders of Consciousness. Journal of Sun Yat-sen University Medical Sciences. 2021;42(4):487-93. PubMed PMID: CSCD:7036612.

3641. Maeda Y, Otsuka T, Mitsuhara T, Okazaki T, Yuge L, Takeda M. A novel bone-thinning technique for transcranial stimulation motor-evoked potentials in rats. Sci Rep. 2021;11(1):12496. Epub 2021/06/16. doi: 10.1038/s41598-021-91780-5. PubMed PMID: 34127706; PubMed Central PMCID: PMCPMC8203740.

3642. Magnusson JL, Leventhal DK. Revisiting the "Paradox of Stereotaxic Surgery": Insights Into Basal Ganglia-Thalamic Interactions. Frontiers in Systems Neuroscience. 2021;15. doi: 10.3389/fnsys.2021.725876.

3643. Mahmud M, Prendergast M, Hadi Z, Seemungal B. Modulating balance with galvanic vestibular stimulation in traumatic brain injury patients. European journal of neurology. 2021;28(SUPPL 1):146-. doi: 10.1111/ene.14973. PubMed PMID: CN-02293585.

3644. Mansur A, Rabiner EA, Tsukada H, Comley RA, Lewis Y, Huiban M, et al. Test–retest variability and reference region-based quantification of 18F-BCPP-EF for imaging mitochondrial complex I in the human brain. Journal of Cerebral Blood Flow and Metabolism. 2021;41(4):771-9. doi: 10.1177/0271678X20928149.

3645. Manto M, Kakei S, Mitoma H. The critical need to develop tools assessing cerebellar reserve for the delivery and assessment of non-invasive cerebellar stimulation. Cerebellum and Ataxias. 2021;8(1). doi: 10.1186/s40673-020-00126-w.

3646. Mao B, Wang Y, Balasubramanian T, Urioste R, Wafa T, Fitzgerald TS, et al. Assessment of auditory and vestibular damage in a mouse model after single and triple blast exposures. Hear Res. 2021;407:108292. Epub 2021/07/03. doi: 10.1016/j.heares.2021.108292. PubMed PMID: 34214947; PubMed Central PMCID: PMCPMC8276524.

3647. Martel MM, Eng AG, Bansal PS, Smith TE, Elkins AR, Goh PK. Multiple informant average integration of ADHD symptom ratings predictive of concurrent and longitudinal impairment. Psychological Assessment. 2021;33(5):443-51. doi: 10.1037/pas0000994.

3648. Martens G, Ibáñez-Soria D, Barra A, Soria-Frisch A, Piarulli A, Gosseries O, et al. A novel closed-loop EEG-tDCS approach to promote responsiveness of patients in minimally conscious state: A study protocol. Behav Brain Res. 2021;409:113311. Epub 2021/04/21. doi: 10.1016/j.bbr.2021.113311. PubMed PMID: 33878429.

3649. Max SM, Plewnia C, Zipfel S, Giel KE, Schag K. Combined antisaccade task and transcranial direct current stimulation to increase

response inhibition in binge eating disorder. European Archives of Psychiatry and Clinical Neuroscience. 2021;271(1):17-28. doi: 10.1007/s00406-020-01164-5.
3650. Mayorova LA, Petrova MV, Pryanikov IV, Grechko AV. CHRONIC DISORDERS OF CONSCIOUSNESS: DIAGNOSIS AND PROGNOSTICATION. Zhurnal Vysshei Nervnoi Deyatelnosti Imeni I P Pavlova. 2021;71(2):213-36. doi: 10.31857/s0044467721020076. PubMed PMID: WOS:000649024000005.
3651. Mayorova LA, Petrova MV, Pryanikov IV, Grechko AV. Chronic Disorders of Consciousness: Diagnosis and Prognosis. Neuroscience and Behavioral Physiology. 2021;51(8):1132-47. doi: 10.1007/s11055-021-01173-4.
3652. Meek BP, Hill S, Modirrousta M. Accelerated repetitive transcranial magnetic stimulation in the treatment of post-concussion symptoms due to mild traumatic brain injury: a pilot study. Brain Inj. 2021;35(1):48-58. Epub 2020/12/11. doi: 10.1080/02699052.2020.1857837. PubMed PMID: 33297788.
3653. Mehta UM, Ektare A, Jakhar J, Basavaraju R, Sanjay TN, Naik SS, et al. A transdiagnostic evaluation of cortical inhibition in severe mental disorders using Transcranial Magnetic Stimulation. Journal of Psychiatric Research. 2021;143:364-9. doi:

10.1016/j.jpsychires.2021.09.049.
3654. Mikellides G, Michael P, Schuhmann T, Sack AT. TMS-Induced Seizure during FDA-Approved Bilateral DMPFC Protocol for Treating OCD: A Case Report. Case reports in neurology. 2021;13(3):584-90. doi: 10.1159/000518999. PubMed PMID: MEDLINE:34703446.
3655. Milosevic M, Nakanishi T, Sasaki A, Yamaguchi A, Nomura T, Popovic MR, et al. Cortical Re-organization After Traumatic Brain Injury Elicited Using Functional Electrical Stimulation Therapy: A Case Report. Front Neurosci. 2021;15:693861. Epub 2021/09/08. doi: 10.3389/fnins.2021.693861. PubMed PMID: 34489624; PubMed Central PMCID: PMCPMC8417438.
3656. Mitra B, Ruggles T, Seah J, Miller C, Fitzgerald MC. Delayed intracranial hemorrhage after trauma. Brain Injury. 2021;35(4):484-9. doi: 10.1080/02699052.2021.1887520.
3657. Molero-Chamizo A, Salas Sánchez Á, Álvarez Batista B, Cordero García C, Andújar Barroso R, Rivera-Urbina GN, et al. Bilateral Motor Cortex tDCS Effects on Post-Stroke Pain and Spasticity: A Three Cases Study. Frontiers in Pharmacology. 2021;12. doi: 10.3389/fphar.2021.624582.
3658. Mollica A, Safavifar F, Fralick M, Giacobbe P, Lipsman N, Burke MJ. Transcranial Magnetic Stimulation for the Treatment of

Concussion: A Systematic Review. Neuromodulation. 2021;24(5):803-12. Epub 2020/11/14. doi: 10.1111/ner.13319. PubMed PMID: 33184973.

3659. Monti M. Ultrasonic stimulation in disorders of consciousness. Brain Stimulation. 2021;14(6):1746-7. doi: 10.1016/j.brs.2021.10.532.

3660. Moreau-Debord I, Serrano É, Quessy S, Dancause N. Rapid and Bihemispheric Reorganization of Neuronal Activity in Premotor Cortex after Brain Injury. J Neurosci. 2021;41(44):9112-28. Epub 2021/09/25. doi: 10.1523/jneurosci.0196-21.2021. PubMed PMID: 34556488; PubMed Central PMCID: PMCPMC8570830.

3661. Mosiello G, Safder S, Marshall D, Rolle U, Benninga MA. Neurogenic bowel dysfunction in children and adolescents. Journal of Clinical Medicine. 2021;10(8). doi: 10.3390/jcm10081669.

3662. Mota F, Ruiz-Bedoya C, Tucker E, De Jesus P, Flavahan K, Turner M, et al. Noninvasive Assessment of Intralesional Antimicrobial Concentration-Time Profiles in Pulmonary and Central Nervous System Tuberculosis using Dynamic 18F-Pretomanid Positron Emission Tomography. Open Forum Infectious Diseases. 2021;8(SUPPL 1):S789-S90. doi: 10.1093/ofid/ofab466.1603.

3663. Moussi K, Haneef AA, Alsiary RA, Diallo EM, Boone MA, Abu-Araki H, et al. A Microneedles Balloon Catheter for Endovascular Drug Delivery. Advanced Materials Technologies.

2021;6(8). doi: 10.1002/admt.202100037. PubMed PMID: WOS:000655686800001.

3664. Mülling K, Fischer AJ, Siakaeva E, Richter M, Bordbari S, Spyra I, et al. Neutrophil dynamics, plasticity and function in acute neurodegeneration following neonatal hypoxia–ischemia. Brain, Behavior, and Immunity. 2021;92:234-44. doi: 10.1016/j.bbi.2020.12.012.

3665. Munir A, Rymowicz R, Joshi P. A rare case of gamma-butyrolactone (GBL) withdrawal syndrome. American Journal on Addictions. 2021;30(3):238-9. doi: 10.1111/ajad.13173.

3666. Muttalib F, Fonseca Y, Stein P, Doctor A. The association between baseline heart rate variability and neurological outcome among children hospitalized due to cerebral malaria in malawi: A pilot cohort study. Pediatric Critical Care Medicine. 2021;22(SUPPL 1):49. doi: 10.1097/01.pcc.0000738448.18543.3b.

3667. Nagy SA, Kürtös Z, Németh N, Perlaki G, Csernela E, Lakner FE, et al. Childhood maltreatment results in altered deactivation of reward processing circuits in depressed patients: A functional magnetic resonance imaging study of a facial emotion recognition task. Neurobiology of Stress. 2021;15. doi: 10.1016/j.ynstr.2021.100399.

3668. Nct. Noninvasive Modulation of Motivational Brain Regions in Healthy Volunteers. https://clinicaltrialsgov/show/NCT04972786.

2021. PubMed PMID: CN-02296894.

3669. Nct. Foundations in Developing Precision Cognitive Restoration. https://clinicaltrialsgov/show/NCT05117502. 2021. PubMed PMID: CN-02352777.

3670. Nct. tDCS and Cognitive Training in Active Duty Service Members With Mild TBI: A Pilot Study. https://clinicaltrialsgov/show/NCT04925453. 2021. PubMed PMID: CN-02278434.

3671. Nct. Treating Mild Traumatic Brain Injury With High Definition Transcranial Direct Current Stimulation. https://clinicaltrialsgov/show/NCT04869059. 2021. PubMed PMID: CN-02255043.

3672. Nct. Effectiveness of NEUROM Combined With tDCS to Guide Motor Recovery in Paraplegics Patients. https://clinicaltrialsgov/show/NCT04790149. 2021. PubMed PMID: CN-02249762.

3673. Nct. Brain State-dependent Stimulation to Improve Movement. https://clinicaltrialsgov/show/NCT05103176. 2021. PubMed PMID: CN-02341444.

3674. Nct. Effects of Action Observation Training and Exercises Over 65 Years Old. https://clinicaltrialsgov/show/NCT04759690. 2021. PubMed PMID: CN-02249081.

3675. Nct. Effects of tDCS With Gait Training on Leg Performance in Incomplete Spinal Cord Injury. https://clinicaltrialsgov/show/NCT04910412. 2021. PubMed PMID: CN-02278092.

3676. Nct. Random Noise Stimulation to Enhance Cortical Drive & Improve Hand Function. https://clinicaltrialsgov/show/NCT04777149. 2021. PubMed PMID: CN-02269153.

3677. Nct. A Telerehabilitation Program for SCI. https://clinicaltrialsgov/show/NCT04977037. 2021. PubMed PMID: CN-02297003.

3678. Nct. Transspinal Stimulation Plus Locomotor Training for SCI. https://clinicaltrialsgov/show/NCT04807764. 2021. PubMed PMID: CN-02253036.

3679. Nct. Neural Basis of Sensory Learning: Brain Regions. https://clinicaltrialsgov/show/NCT05079516. 2021. PubMed PMID: CN-02340975.

3680. Nct. The Study of a Neural Interface and a Neurostimulation in the Rehabilitation of Upper Limb Movement Impairments. https://clinicaltrialsgov/show/NCT05115149. 2021. PubMed PMID: CN-02352723.

3681. Nct. Motor and Autonomic Concomitant Health Improvements With Neuromodulation and Exoskeleton Training: A

RCT in Individuals With SCI. https://clinicaltrialsgov/show/NCT04726059. 2021. PubMed PMID: CN-02234463.

3682. Nct. Efficacy of a Right-sided Ablation of the Anterior Ganglionated Plexus for Neurally Mediated Syncope. https://clinicaltrialsgov/show/NCT04755101. 2021. PubMed PMID: CN-02235204.

3683. Nct. Improving Visual Field Deficits With Noninvasive Brain Stimulation. https://clinicaltrialsgov/show/NCT05085210. 2021. PubMed PMID: CN-02341107.

3684. Nct. Cerebellar tDCS for SRPCS Treatment. https://clinicaltrialsgov/show/NCT04760899. 2021. PubMed PMID: CN-02249114.

3685. Nejati V, Movahed Alavi M, Nitsche MA. The Impact of Attention Deficit-hyperactivity Disorder Symptom Severity on the Effectiveness of Transcranial Direct Current Stimulation (tDCS) on Inhibitory Control. Neuroscience. 2021;466:248-57. doi: 10.1016/j.neuroscience.2021.05.008.

3686. Nishikawa Y, Takahashi T, Kawade S, Maeda N, Maruyama H, Hyngstrom A. The effect of electrical muscle stimulation on muscle mass and balance in older adults with dementia. Brain Sciences. 2021;11(3):1-8. doi: 10.3390/brainsci11030339. PubMed PMID:

CN-02264282.

3687. Noda Y. Clinical neurophysiology, neuroimaging, and neuromodulation of neuropsychiatric disorders. Journal of Personalized Medicine. 2021;11(11). doi: 10.3390/jpm11111193.

3688. Noé E, Ferri J, Olaya J, Navarro MD, O'valle M, Colomer C, et al. When, how, and to what extent are individuals with unresponsive wakefulness syndrome able to progress? Neurobehavioral progress. Brain Sciences. 2021;11(1):1-16. doi: 10.3390/brainsci11010126.

3689. Norgbey E, Li Y, Zhu Y, Nwankwegu AS, Bofah-Buah R, Nuamah L, et al. Combined use of high-resolution dialysis, diffusive gradient in thin films (DGT) technique, and conventional methods to assess trace metals in reservoir sediments. Environ Monit Assess. 2021;193(8):469. Epub 2021/07/07. doi: 10.1007/s10661-021-09247-z. PubMed PMID: 34226963.

3690. Ntikas M, Hunter AM, Gallagher IJ, Di Virgilio TG. Longer Neurophysiological vs. Clinical Recovery Following Sport Concussion. Frontiers in sports and active living. 2021;3:737712-. doi: 10.3389/fspor.2021.737712. PubMed PMID: MEDLINE:34957396.

3691. O'Donnell DM, Agin A. Management of headaches in children and adolescents. Current Problems in Pediatric and Adolescent Health Care. 2021;51(7). doi: 10.1016/j.cppeds.2021.101034.

3692. O'Leary GH, Jenkins DD, Coker-Bolt P, George MS, Kautz S,

Bikson M, et al. From adults to pediatrics: A review noninvasive brain stimulation (NIBS) to facilitate recovery from brain injury. Prog Brain Res. 2021;264:287-322. Epub 2021/06/26. doi: 10.1016/bs.pbr.2021.01.019. PubMed PMID: 34167660; PubMed Central PMCID: PMCPMC8855635.

3693. O'Neal CM, Schroeder LN, Wells AA, Chen S, Stephens TM, Glenn CA, et al. Patient Outcomes in Disorders of Consciousness Following Transcranial Magnetic Stimulation: A Systematic Review and Meta-Analysis of Individual Patient Data. Front Neurol. 2021;12:694970. Epub 2021/09/04. doi: 10.3389/fneur.2021.694970. PubMed PMID: 34475848; PubMed Central PMCID: PMCPMC8407074.

3694. Omidi M, Mansouri V, Amirabad LM, Tayebi L. Impact of Lipid/Magnesium Hydroxide Hybrid Nanoparticles on the Stability of Vascular Endothelial Growth Factor-Loaded PLGA Microspheres. Acs Applied Materials & Interfaces. 2021;13(21):24370-84. doi: 10.1021/acsami.0c22140. PubMed PMID: WOS:000659315800003.

3695. Onose G, Anghelescu A, Blendea CD, Ciobanu V, Daia CO, Firan FC, et al. Non-invasive, non-pharmacological/bio-technological interventions towards neurorestoration upshot after ischemic stroke, in adults-systematic, synthetic, literature review. Frontiers in Bioscience-Landmark. 2021;26(11):1204-39. doi: 10.52586/5020.

PubMed PMID: WOS:000727662100021.
3696. Orakpo N, Vieux U, Castro-Nuñez C. Case Report: Virtual Reality Neurofeedback Therapy as a Novel Modality for Sustained Analgesia in Centralized Pain Syndromes. Frontiers in Psychiatry. 2021;12. doi: 10.3389/fpsyt.2021.660105.
3697. Orlando B, Morano A, Irelli EC, Fanella M, Giallonardo AT, Di Bonaventura C. Unilateral catalepsy in non-convulsive status epilepticus. Epileptic Disorders. 2021;23(5):760-4. doi: 10.1684/epd.2021.1323.
3698. Ortelli P, Ferrazzoli D, Sebastianelli L, Maestri R, Dezi S, Spampinato D, et al. Fatigue and “brain fog” in the aftermath of mild COVID-19: A neuropsychological and TMS study. Journal of the Neurological Sciences. 2021;429. doi: 10.1016/j.jns.2021.119854.
3699. Pan J, Wu J, Liu J, Wu J, Wang F. A systematic review of sleep in patients with disorders of consciousness: From diagnosis to prognosis. Brain Sciences. 2021;11(8). doi: 10.3390/brainsci11081072.
3700. Pape TB. Preliminary evaluation of the clinical utility of miRNA as neurobiological markers of TBI diagnosis and rTMS treatment responsiveness. Brain Stimulation. 2021;14(6):1721. doi: 10.1016/j.brs.2021.10.438.
3701. Park G, Suh JH, Han SJ. Transcranial direct current

stimulation for balance and gait in repetitive mild traumatic brain injury in rats. BMC Neurosci. 2021;22(1):26. Epub 2021/04/19. doi: 10.1186/s12868-021-00633-4. PubMed PMID: 33865318; PubMed Central PMCID: PMCPMC8052850.

3702. Pascarella A, Manzo L, Estraneo A. Long-term clinical, neurophysiological and neuroimaging follow-up of a patient with hemiparkinson-hemiatrophy syndrome. Journal of the Neurological Sciences. 2021;429. doi: 10.1016/j.jns.2021.119461.

3703. Patterson Gentile C, Shah R, Irwin SL, Greene K, Szperka CL. Acute and chronic management of posttraumatic headache in children: A systematic review. Headache. 2021;61(10):1475-92. Epub 2021/12/05. doi: 10.1111/head.14236. PubMed PMID: 34862612.

3704. Pearce AJ, Kidgell DJ, Tommerdahl MA, Frazer AK, Rist B, Mobbs R, et al. Chronic Neurophysiological Effects of Repeated Head Trauma in Retired Australian Male Sport Athletes. Front Neurol. 2021;12:633320. Epub 2021/03/27. doi: 10.3389/fneur.2021.633320. PubMed PMID: 33767661; PubMed Central PMCID: PMCPMC7985524.

3705. Petersen A, Soderstrom M, Saha B, Sharma P. Animal models of traumatic brain injury: a review of pathophysiology to biomarkers and treatments. Experimental Brain Research. 2021;239(10):2939-50. doi: 10.1007/s00221-021-06178-6. PubMed PMID:

WOS:000679277000001.
3706. Pincherle A, Rosanova M, Diserens K. Editorial: New Advances in Diagnostic Tools and Rehabilitation of Disorders of Consciousness in the Acute Phase. Front Neurol. 2021;12:770791. Epub 2021/12/07. doi: 10.3389/fneur.2021.770791. PubMed PMID: 34867758; PubMed Central PMCID: PMCPMC8635187.
3707. Pink AE, Williams C, Alderman N, Stoffels M. The use of repetitive transcranial magnetic stimulation (rTMS) following traumatic brain injury (TBI): A scoping review. Neuropsychol Rehabil. 2021;31(3):479-505. Epub 2019/12/28. doi: 10.1080/09602011.2019.1706585. PubMed PMID: 31880207.
3708. Ptito A, Papa L, Gregory K, Folmer RL, Walker WC, Prabhakaran V, et al. A Prospective, Multicenter Study to Assess the Safety and Efficacy of Translingual Neurostimulation Plus Physical Therapy for the Treatment of a Chronic Balance Deficit Due to Mild-to-Moderate Traumatic Brain Injury. Neuromodulation. 2021;24(8):1412-21. doi: 10.1111/ner.13159. PubMed PMID: WOS:000529223000001.
3709. Qian F, Han Y, Han Z, Zhang D, Zhang L, Zhao G, et al. In Situ implantable, post-trauma microenvironment-responsive, ROS Depletion Hydrogels for the treatment of Traumatic brain injury. Biomaterials. 2021;270. doi: 10.1016/j.biomaterials.2021.120675.

3710. Qiu W, Bouakaz A, Konofagou EE, Zheng H. Ultrasound for the Brain: A Review of Physical and Engineering Principles, and Clinical Applications. Ieee Transactions on Ultrasonics Ferroelectrics and Frequency Control. 2021;68(1):6-20. doi: 10.1109/tuffc.2020.3019932. PubMed PMID: WOS:000602706700002.

3711. Rao J, Hu G, Yang W, Yu J, Xue C, Tian L, et al. Recovery of aphemia with pure word dumbness after treatment with repetitive transcranial magnetic stimulation: A case report. Journal of Neurolinguistics. 2021;59. doi: 10.1016/j.jneuroling.2021.100991. PubMed PMID: WOS:000656680400012.

3712. Requena MB, Permana AD, Vollet-Filho JD, Gonzalez-Vazquez P, Garcia MR, Goncalves De Faria CM, et al. Dissolving microneedles containing aminolevulinic acid improves protoporphyrinIXdistribution. Journal of Biophotonics. 2021;14(1). doi: 10.1002/jbio.202000128. PubMed PMID: WOS:000575099600001.

3713. Rezai A, Ranjan M, D'Haese PF, Haut M, Carpenter J, Najib U, et al. Opening of Blood Brain Barrier (BBB) in the Hippocampus and Entorhinal Cortex in Early Alzheimer's Disease with Focused Ultrasound (FUS). Stereotactic and Functional Neurosurgery. 2021;99(SUPPL 1):105-6. doi: 10.1159/000520618.

3714. Rosalia L, Ozturk C, Shoar S, Fan Y, Malone G, Cheema FH, et al. Device-Based Solutions to Improve Cardiac Physiology and Hemodynamics in Heart Failure With Preserved Ejection Fraction. JACC Basic Transl Sci. 2021;6(9-10):772-95. Epub 2021/11/11. doi: 10.1016/j.jacbts.2021.06.002. PubMed PMID: 34754993; PubMed Central PMCID: PMCPMC8559325.

3715. Rosanova M, Sarasso S. Sleep-like cortical bistability after focal and multifocal brain lesions. Brain Stimulation. 2021;14(6):1743. doi: 10.1016/j.brs.2021.10.518.

3716. Rouwhorst R, Dijkstra E, Van Oostrom I, Zwienenberg L, Sack A, Van Dijk H, et al. Vasovagal syncope as a specific side effect of rTMS: A frontal-vagal dose-finding study. Brain Stimulation. 2021;14(6):1648. doi: 10.1016/j.brs.2021.10.194.

3717. Rr P, Douch C, Aan Koh MJ, Lai AHM, Lim CT, Hartley L, et al. Speckled brain lesions in Incontinentia Pigmenti patients with acquired brain syndromes. European Journal of Paediatric Neurology. 2021;33:106-11. doi: 10.1016/j.ejpn.2021.05.012.

3718. Ruan L, Song G, Zhang X, Liu T, Sun Y, Zhu J, et al. Transdermal delivery of multifunctional CaO2@Mn-PDA nanoformulations by microneedles for NIR-induced synergistic therapy against skin melanoma. Biomaterials Science. 2021;9(20):6830-41. doi: 10.1039/d1bm01117k. PubMed PMID:

WOS:000693154800001.
3719. Rudroff T, Workman CD. Transcranial Direct Current Stimulation as a Treatment Tool for Mild Traumatic Brain Injury. Brain Sci. 2021;11(6). Epub 2021/07/03. doi: 10.3390/brainsci11060806. PubMed PMID: 34207004; PubMed Central PMCID: PMCPMC8235194.
3720. Rushby JA, De Blasio FM, Logan JA, Wearne T, Kornfeld E, Wilson EJ, et al. tDCS effects on task-related activation and working memory performance in traumatic brain injury: A within group randomized controlled trial. Neuropsychol Rehabil. 2021;31(5):814-36. Epub 2020/03/03. doi: 10.1080/09602011.2020.1733620. PubMed PMID: 32114899.
3721. Saepang K, Li SK, Chantasart D. Passive and iontophoretic transport of pramipexole dihydrochloride across human skin microchannels created by microneedles in vitro. Int J Pharm. 2021;609:121092. Epub 2021/09/17. doi: 10.1016/j.ijpharm.2021.121092. PubMed PMID: 34530098.
3722. Saetia S, Yoshimura N, Koike Y. Constructing Brain Connectivity Model Using Causal Network Reconstruction Approach. Frontiers in Neuroinformatics. 2021;15. doi: 10.3389/fninf.2021.619557.
3723. Saleem G, Slomine B, Suskauer S. Transcranial Direct Current

Stimulation Feasibility Challenges in Youth with Severe Brain Injury: A Case Report. Neuromodulation. 2021;24(4):e159. doi: 10.1111/ner.13385.

3724. San Luciano M, Robichaux-Viehoever A, Dodenhoff KA, Gittings ML, Viser AC, Racine CA, et al. Thalamic deep brain stimulation for acquired dystonia in children and young adults: a phase 1 clinical trial. Journal of Neurosurgery-Pediatrics. 2021;27(2):203-12. doi: 10.3171/2020.7.Peds20348. PubMed PMID: WOS:000615948400004.

3725. Sanesteban Beceiro E, Mayo Rodríguez P, García Yepes M, Gómez Esteban A, Cuadrado Pérez M, Porta-Etessam J, et al. Secondary Cluster Headache: Description of five cases. European Journal of Neurology. 2021;28(SUPPL 1):269. doi: 10.1111/ene.14968.

3726. Santaniello S, McConnell GC, Gale JT, Faghih RT, Kemere C, Hilliard JD, et al. Editorial: Towards the Next Generation of Deep Brain Stimulation Therapies: Technological Advancements, Computational Methods, and New Targets. Frontiers in Neuroscience. 2021;15. doi: 10.3389/fnins.2021.737737.

3727. Sanz LRD, Thibaut A, Edlow BL, Laureys S, Gosseries O. Update on neuroimaging in disorders of consciousness. Curr Opin Neurol. 2021;34(4):488-96. Epub 2021/06/01. doi:

10.1097/wco.0000000000000951. PubMed PMID: 34054109; PubMed Central PMCID: PMCPMC8938964.

3728. Schaper F, Nordberg J, Cohen A, Hsu J, Lin C, Ferguson M, et al. An epilepsy network derived from human brain lesions and deep brain stimulation. Brain Stimulation. 2021;14(6):1643-4. doi: 10.1016/j.brs.2021.10.178.

3729. Schmidt J, Brown KE, Feldman SJ, Babul S, Zwicker JG, Boyd LA. Evidence of altered interhemispheric communication after pediatric concussion. Brain Injury. 2021;35(10):1143-61. doi: 10.1080/02699052.2021.1929485. PubMed PMID: WOS:000684719800001.

3730. Schwab PJ, Miller A, Raphail AM, Levine A, Haslam C, Coslett HB, et al. Virtual reality tools for assessing unilateral spatial neglect: A novel opportunity for data collection. Journal of Visualized Experiments. 2021;2021(169). doi: 10.3791/61951.

3731. Schwarm FP, Ott M, Nagl J, Bender M, Stein M, Uhl E, et al. The Predictive Value of Transcutaneous Electrical Nerve Stimulation for Patient Selection in Peripheral Nerve Field Stimulation for Chronic Low Back Pain: A Prospective Study. Neuromodulation. 2021;24(6):1051-8. Epub 2020/08/07. doi: 10.1111/ner.13244. PubMed PMID: 32757257.

3732. Seeni RZ, Zheng M, Lio DCS, Wiraja C, Yusoff MFBM, Koh

WTY, et al. Targeted Delivery of Anesthetic Agents to Bone Tissues using Conductive Microneedles Enhanced Iontophoresis for Painless Dental Anesthesia. Advanced Functional Materials. 2021;31(47). doi: 10.1002/adfm.202105686. PubMed PMID: WOS:000686916800001.

3733. Serradell-Ribe N, Romero Munoz JP, Viejo-Sobera R, Marron EM. Brain plasticity in frontal traumatic brain injury. Biomarker of severity and intervention efficacy. Brain Stimulation. 2021;14(6):1648-9. doi: 10.1016/j.brs.2021.10.195. PubMed PMID: CN-02370734.

3734. Serruya MD, Rosenwasser RH. An artificial nervous system to treat chronic stroke. Artif Organs. 2021;45(8):804-12. Epub 2021/06/23. doi: 10.1111/aor.13998. PubMed PMID: 34156104.

3735. Sheline Y, Balderston N, Oathes D, Makhoul W, Duprat R, Beer J, et al. Intensive tms in treatment refractory patients with major depressive disorder: A pilot replication. Neuropsychopharmacology. 2021;46:284-5. doi: 10.1038/s41386-021-01237-6.

3736. Shen Y, Wang J, Yuan J, Yang L, Yu F, Wang X, et al. Anemia among Chinese patients with chronic kidney disease and its association with quality of life - results from the Chinese cohort study of chronic kidney disease (C-STRIDE). BMC nephrology. 2021;22(1):64. doi: 10.1186/s12882-021-02247-8.

3737. Shi Y, Song R, Wang Z, Zhang H, Zhu J, Yue Y, et al. Potential

clinical value of circular RNAs as peripheral biomarkers for the diagnosis and treatment of major depressive disorder. EBioMedicine. 2021;66. doi: 10.1016/j.ebiom.2021.103337.
3738. Shirvani S, Davoudi M, Shirvani M, Koleini P, Hojat Panah S, Shoshtari F, et al. Comparison of the effects of transcranial direct current stimulation and mindfulness-based stress reduction on mental fatigue, quality of life and aggression in mild traumatic brain injury patients: a randomized clinical trial. Ann Gen Psychiatry. 2021;20(1):33. Epub 2021/06/17. doi: 10.1186/s12991-021-00355-1. PubMed PMID: 34130721; PubMed Central PMCID: PMCPMC8207739.
3739. Shou Z, Li Z, Wang X, Chen M, Bai Y, Di H. Non-invasive brain intervention techniques used in patients with disorders of consciousness. Int J Neurosci. 2021;131(4):390-404. Epub 2020/04/03. doi: 10.1080/00207454.2020.1744598. PubMed PMID: 32238043.
3740. Siddappaji KK, Gopal S. Molecular mechanisms in Alzheimer's disease and the impact of physical exercise with advancements in therapeutic approaches. AIMS neuroscience. 2021;8(3):357-89. doi: 10.3934/Neuroscience.2021020. PubMed PMID: MEDLINE:34183987.
3741. Siddiqi S. Precision targeting of brain networks in TBI. Brain Stimulation. 2021;14(6):1721-2. doi: 10.1016/j.brs.2021.10.439.

3742. Siddiqi S. Connectome-targeted neuromodulation across symptoms and disorders. Brain Stimulation. 2021;14(6):1712. doi: 10.1016/j.brs.2021.10.407.

3743. Singhal D, Hahn CN, Feurstein S, Wee LYA, Moma L, Kutyna MM, et al. Targeted gene panels identify a high frequency of pathogenic germline variants in patients diagnosed with a hematological malignancy and at least one other independent cancer. Leukemia. 2021;35(11):3245-56. doi: 10.1038/s41375-021-01246-w.

3744. Sippel LM, Flanagan JC, Holtzheimer PE, Moran-Santa-Maria MM, Brady KT, Joseph JE. Effects of intranasal oxytocin on threat- and reward-related functional connectivity in men and women with and without childhood abuse-related PTSD. Psychiatry Research - Neuroimaging. 2021;317. doi: 10.1016/j.pscychresns.2021.111368. PubMed PMID: CN-02301315.

3745. Skalický P, Masopust V. Bilateral cortical stimulation for bilateral refractory atypical trigeminal neuropathic pain: Case report. Neuro Endocrinol Lett. 2021;42(2):87-90. Epub 2021/07/05. PubMed PMID: 34217165.

3746. Song C, Gao C, Zhao J, Wang Z. Construction of long-circulation EpCAM targeted drug delivery system and its application in the diagnosis and treatment of breast cancer. Journal of Biomaterials Applications. 2021;35(8):947-57. doi:

10.1177/0885328220965135. PubMed PMID: WOS:000622799000005.
3747. Song F, Gao H, Li D, Petrov AV, Petrov VV, Wen D, et al. Low intensity focused ultrasound responsive microcapsules for non-ablative ultrafast intracellular release of small molecules. Journal of Materials Chemistry B. 2021;9(10). doi: 10.1039/d0tb02788j. PubMed PMID: WOS:000629733200003.
3748. Spindler LRB, Luppi AI, Adapa RM, Craig MM, Coppola P, Peattie ARD, et al. Dopaminergic brainstem disconnection is common to pharmacological and pathological consciousness. Proceedings of the National Academy of Sciences of the United States of America. 2021;118(30). doi: 10.1073/pnas.2026289118. PubMed PMID: WOS:000685039000004.
3749. Spindler LRB, Luppi AI, Adapa RM, Craig MM, Coppola P, Peattie ARD, et al. Dopaminergic brainstem disconnection is common to pharmacological and pathological consciousness perturbation. Proceedings of the National Academy of Sciences of the United States of America. 2021;118(30). doi: 10.1073/pnas.2026289118.
3750. Stavropoulos I, Selway R, Hasegawa H, Hughes E, Rittey C, Jimenez-Jimenez D, et al. Low frequency centromedian thalamic nuclei deep brain stimulation for the treatment of super refractory status epilepticus: A case report and a review of the literature. Brain

Stimulation. 2021;14(2):226-9. doi: 10.1016/j.brs.2020.12.013. PubMed PMID: WOS:000632697000004.

3751. Stephens TM, Young IM, O'Neal CM, Dadario NB, Briggs RG, Teo C, et al. Akinetic mutism reversed by inferior parietal lobule repetitive theta burst stimulation: Can we restore default mode network function for therapeutic benefit? Brain Behav. 2021;11(8):e02180. Epub 2021/06/20. doi: 10.1002/brb3.2180. PubMed PMID: 34145791; PubMed Central PMCID: PMCPMC8413751.

3752. Storch S, Samantzis M, Balbi M. Driving Oscillatory Dynamics: Neuromodulation for Recovery After Stroke. Frontiers in Systems Neuroscience. 2021;15. doi: 10.3389/fnsys.2021.712664.

3753. Su Y, Zhang X, Lei L, Liu B, Wu S, Shen J. Tumor Microenvironment-Activatable Cyclic Cascade Reaction to Reinforce Multimodal Combination Therapy by Destroying the Extracellular Matrix. Acs Applied Materials & Interfaces. 2021;13(11):12960-71. doi: 10.1021/acsami.1c02011. PubMed PMID: WOS:000634759500033.

3754. Sun Q, Fang Y, Shi Y, Wang L, Peng X, Tan L. Inhibitory Top-Down Control Deficits in Schizophrenia With Auditory Verbal Hallucinations: A Go/NoGo Task. Frontiers in Psychiatry. 2021;12. doi: 10.3389/fpsyt.2021.544746.

3755. Szarka G, Balogh M, Tengölics Á J, Ganczer A, Völgyi B, Kovács-Öller T. The role of gap junctions in cell death and neuromodulation in the retina. Neural Regen Res. 2021;16(10):1911-20. Epub 2021/03/02. doi: 10.4103/1673-5374.308069. PubMed PMID: 33642359; PubMed Central PMCID: PMCPMC8343308.
3756. Taing AS, Mundy ME, Ponsford JL, Spitz G. Aberrant modulation of brain activity underlies impaired working memory following traumatic brain injury. NeuroImage: Clinical. 2021;31. doi: 10.1016/j.nicl.2021.102777.
3757. Talas DÜ, Beger O, Çömelekoğlu Ü, Çakır S, Taghipour P, Vayisoğlu Y. An insight to tympanic membrane perforation pressure through morphometry: A cadaver study. Diving and Hyperbaric Medicine. 2021;51(1):10-7. doi: 10.28920/dhm51.1.10-17.
3758. Tennant JR, Adams MS, Brown KE, Staines WR. Long-term effects of concussion on relevancy-based modulation of somatosensory-evoked potentials. Clinical Neurophysiology. 2021;132(10):2431-9. doi: 10.1016/j.clinph.2021.06.027. PubMed PMID: WOS:000704914800013.
3759. Toyohara J, Sakata M, Ishibashi K, Mossel P, Imai M, Wagatsuma K, et al. First clinical assessment of [18F]MC225, a novel fluorine-18 labelled PET tracer for measuring functional

P-glycoprotein at the blood–brain barrier. Annals of Nuclear Medicine. 2021;35(11):1240-52. doi: 10.1007/s12149-021-01666-9.

3760. Trofimov AO, Agarkova DI, Kopylov AA, Dubrovin A, Trofimova KA, Sheludyakov A, et al. NIRS-Based Assessment of Cerebral Oxygenation During High-Definition Anodal Transcranial Direct Current Stimulation in Patients with Posttraumatic Encephalopathy. Adv Exp Med Biol. 2021;1339:27-31. Epub 2022/01/14. doi: 10.1007/978-3-030-78787-5_4. PubMed PMID: 35023086.

3761. Trofimov AO, Kopylov AA, Martynov DS, Zorkova AV, Trofimova K, Cheremuhin PN, et al. The Changes in Brain Oxygenation During Transcranial Alternating Current Stimulation as Consequences of Traumatic Brain Injury: A Near-Infrared Spectroscopy Study. Adv Exp Med Biol. 2021;1269:235-9. Epub 2021/05/10. doi: 10.1007/978-3-030-48238-1_37. PubMed PMID: 33966223.

3762. Tsai PY, Chen YC, Wang JY, Chung KH, Lai CH. Effect of repetitive transcranial magnetic stimulation on depression and cognition in individuals with traumatic brain injury: a systematic review and meta-analysis. Sci Rep. 2021;11(1):16940. Epub 2021/08/22. doi: 10.1038/s41598-021-95838-2. PubMed PMID: 34417481; PubMed Central PMCID: PMCPMC8379199.

3763. Uchiyama M, Monsanto RDC, Sancak IG, Park GS, Schachern P, Kobayashi H, et al. Temporal Bone Pathology Secondary to Head Trauma-A Human Temporal Bone Study. Otol Neurotol. 2021;42(8):e1152-e9. Epub 2021/07/06. doi: 10.1097/mao.0000000000003192. PubMed PMID: 34224545.
3764. van 't Wout-Frank M, Philip NS. Simultaneous Application of Transcranial Direct Current Stimulation during Virtual Reality Exposure. Journal of visualized experiments : JoVE. 2021(167). doi: 10.3791/61795. PubMed PMID: CN-02271251.
3765. van den Bos MAJ, Higashihara M, Geevasinga N, Menon P, Kiernan MC, Vucic S. Pathophysiological associations of transcallosal dysfunction in ALS. European Journal of Neurology. 2021;28(4):1172-80. doi: 10.1111/ene.14653.
3766. Vaninetti M, Lim M, Khalaf A, Metzger-Smith V, Flowers M, Kunnel A, et al. fMRI findings in MTBI patients with headaches following rTMS. Sci Rep. 2021;11(1):9573. Epub 2021/05/07. doi: 10.1038/s41598-021-89118-2. PubMed PMID: 33953315; PubMed Central PMCID: PMCPMC8100290.
3767. Vaz AR, Vizinha D, Morais H, Colaco AR, Loch-Neckel G, Barbosa M, et al. Overexpression of miR-124 in Motor Neurons Plays a Key Role in ALS Pathological Processes. International Journal of Molecular Sciences. 2021;22(11). doi: 10.3390/ijms22116128.

PubMed PMID: WOS:000660210700001.
3768. Vesia M. Targeting brain circuits for goal-directed movements with multi-focal transcranial magnetic stimulation. Brain Stimulation. 2021;14(6):1713. doi: 10.1016/j.brs.2021.10.410.
3769. Villagomez A, Borja T, Pontón P, Barba P, Chiliquinga A, Yambarela I, et al. HISTOLOGICAL AND MICROBIOLOGICAL EVALUATION IN MICE OF CYANOACRYLATE (HISTOACRYL) AS POSSIBLE REGENERATIVE AGENT TO REPLACE TRADITIONAL SUTURES. Cytotherapy. 2021;23(4):17. doi: 10.1016/j.jcyt.2021.02.052.
3770. Voinescu A, Sui J, Stanton Fraser D. Virtual reality in neurorehabilitation: An umbrella review of meta-analyses. Journal of Clinical Medicine. 2021;10(7). doi: 10.3390/jcm10071478.
3771. Vuralli D, Karatas H, Yemisci M, Bolay H. Updated review on the link between cortical spreading depression and headache disorders. Expert Review of Neurotherapeutics. 2021;21(10):1069-84. doi: 10.1080/14737175.2021.1947797.
3772. Wambaugh JL. An expanding apraxia of speech (AOS) treatment evidence base: An update of recent developments. Aphasiology. 2021;35(4):442-61. doi: 10.1080/02687038.2020.1732289.
3773. Wampler S, Fuertes C, Reck A, Haar CV. Brain injury

increases impulsivity on the rodent gambling task but is not treated by neuromodulation in female rats. Journal of Neurotrauma. 2021;38(14):A30. doi: 10.1089/neu.2021.29111.abstracts.
3774. Wang H, Gao H. Protective effects of repetitive transcranial magnetic stimulation against 6-OHDA-induced Parkinson's symptoms in a mice model: the key role of miR-409-3p/PDHB axis. Int J Neurosci. 2021:1-8. Epub 2021/06/04. doi: 10.1080/00207454.2021.1938037. PubMed PMID: 34078216.
3775. Wang J, Zhu Jn, Ma X, Guo X, Huang D, Zheng Y. Application progress of low-intensity ultrasound in the treatment of neurological diseases. Journal of Ultrasound in Medicine. 2021;23(3):220-2. PubMed PMID: CSCD:6940599.
3776. Wang P, Peng ZL, Liu L, An L, Liu YX, Cao QJ, et al. Neural response to trauma-related and trauma-unrelated negative stimuli in remitted and persistent pediatric post-traumatic stress disorder. Brain and Behavior. 2021;11(7). doi: 10.1002/brb3.2173.
3777. Wang Q, Sun W, Qu Y, Feng C, Wang D, Yin H, et al. Development and Application of Medicine-Engineering Integration in the Rehabilitation of Traumatic Brain Injury. Biomed Res Int. 2021;2021:9962905. Epub 2021/07/09. doi: 10.1155/2021/9962905. PubMed PMID: 34235225; PubMed Central PMCID: PMCPMC8216791 publication of the paper.

3778. Wang Y, Bai Y, Xia X, Niu Z, Yang Y, He J, et al. Comparison of synchrosqueezing transform to alternative methods for time-frequency analysis of TMS-evoked EEG oscillations. Biomedical Signal Processing and Control. 2021;70. doi: 10.1016/j.bspc.2021.102975. PubMed PMID: WOS:000696963000002.

3779. Wang Y, Zhang W, Wu X, Luo X, Li S, Zhu G, et al. Cerebral Structural Abnormalities and Their Associations With Peripheral Cytokine Levels in a Group of Untreated Patients With Nasopharyngeal Carcinoma. Frontiers in Oncology. 2021;11. doi: 10.3389/fonc.2021.740033.

3780. Wen T, Lin Z, Zhao Y, Zhou Y, Niu B, Shi C, et al. Bioresponsive Nanoarchitectonics-Integrated Microneedles for Amplified Chemo-Photodynamic Therapy against Acne Vulgaris. Acs Applied Materials & Interfaces. 2021;13(41):48433-48. doi: 10.1021/acsami.1c15673. PubMed PMID: WOS:000710924900010.

3781. Wessel MJ, Beanato E, Popa T, Windel F, Menoud P, Beliaeva V, et al. Evidence for temporal interference (TI) stimulation effects on motor striatum. Brain Stimulation. 2021;14(6):1684-. doi: 10.1016/j.brs.2021.10.307. PubMed PMID: CN-02370782.

3782. Wileczek A, Reichert A, Kluk M, Sledz S, Sledz A, Kosior J, et al. Non-invasive and invasive autonomic tests to facilitate

cardioneuroablation and complex indications for transcutaneous lead extraction and discontinuation of permanent pacing. Europace. 2021;23(SUPPL 3):iii538. doi: 10.1093/europace/euab116.490.

3783. Wilson CL, Hearps SJC, Tavender EJ, Phillips NT, Lawton B, Kinnear F, et al. Factors predictive for computed tomography use and abnormality in paediatric head injuries in Australia and New Zealand. EMA - Emergency Medicine Australasia. 2021;33(1):157-60. doi: 10.1111/1742-6723.13694.

3784. Won-Seok K, Nam-Jong P. Safety Review for Clinical Application of Repetitive Transcranial Magnetic Stimulation. Brain & NeuroRehabilitation. 2021;14(1):1-8. doi: 10.12786/bn.2021.14.e6. PubMed PMID: KJD:ART002700635.

3785. Wu X, Li YH, Zhang WJ, Zhang SJ, Gao JF. [Effects of electroacupuncture with different courses on the synaptic structure and synaptic function-related proteins in mice with radiation-induced brain injury]. Sheng Li Xue Bao. 2021;73(6):909-16. Epub 2021/12/29. PubMed PMID: 34961865.

3786. Wu YH, Yu J, Hong LR, Luo BY. Neuromodulatory therapies for patients with prolonged disorders of consciousness. Chin Med J (Engl). 2021;134(7):765-76. Epub 2021/04/03. doi: 10.1097/cm9.0000000000001377. PubMed PMID: 33797467; PubMed Central PMCID: PMCPMC8104219.

3787. Xin Z, Abe Y, Liu S, Tanaka KF, Hosomi K, Saitoh Y, et al. Direct Impact of Motor Cortical Stimulation on the Blood Oxygen-level Dependent Response in Rats. Magnetic Resonance in Medical Sciences. 2021;20(1):83-90. doi: 10.2463/mrms.mp.2019-0156. PubMed PMID: WOS:000628817600011.

3788. Xing G, Zhang Y, Zhang Y, Adamson MM, Furst AJ, Ashford JW. Editorial: Evidence-Based Anti-Inflammatory, Anti-Hypoperfusion and Anti-Anxiety/Insomnia Therapies Show Promises for TBI-Induced Post-Traumatic Symptoms and Cognitive Deficits: Advances in Diagnosis and Treatment of TBI-Induced Neurodegeneration and Cognitive Deficits. Frontiers in Neurology. 2021;12. doi: 10.3389/fneur.2021.695629.

3789. Xing X, Jiang R, Lei S, Xu Q, Zhu M, Zhi Y, et al. [Electroacupuncture in the treatment of acute gastrointestinal injury in patients with severe traumatic brain injury: a prospective randomized controlled trial]. Zhonghua Wei Zhong Bing Ji Jiu Yi Xue. 2021;33(1):95-9. Epub 2021/02/11. doi: 10.3760/cma.j.cn121430-20200804-00562. PubMed PMID: 33565409.

3790. Xu H, Wang Y, Luo Y. OTULIN is a new target of EA treatment in the alleviation of brain injury and glial cell activation via

suppression of the NF-κB signalling pathway in acute ischaemic stroke rats. Mol Med. 2021;27(1):37. Epub 2021/04/11. doi: 10.1186/s10020-021-00297-0. PubMed PMID: 33836646; PubMed Central PMCID: PMCPMC8035756.

3791. Yan Q, Weng J, Shen S, Wang Y, Fang M, Zheng G, et al. Finite Element Analysis for Biodegradable Dissolving Microneedle Materials on Skin Puncture and Mechanical Performance Evaluation. Polymers. 2021;13(18). doi: 10.3390/polym13183043. PubMed PMID: WOS:000701147700001.

3792. Yang D, Chen M, Sun Y, Jin Y, Lu C, Pan X, et al. Microneedle-mediated transdermal drug delivery for treating diverse skin diseases. Acta Biomaterialia. 2021;121:119-33. doi: 10.1016/j.actbio.2020.12.004. PubMed PMID: WOS:000614529200007.

3793. Yang D, Shin YI, Hong KS. Systemic Review on Transcranial Electrical Stimulation Parameters and EEG/fNIRS Features for Brain Diseases. Front Neurosci. 2021;15:629323. Epub 2021/04/13. doi: 10.3389/fnins.2021.629323. PubMed PMID: 33841079; PubMed Central PMCID: PMCPMC8032955.

3794. Yang Y, Xu L, Jiang D, Chen BZ, Luo R, Liu Z, et al. Self-Powered Controllable Transdermal Drug Delivery System. Advanced Functional Materials. 2021;31(36). doi:

10.1002/adfm.202104092. PubMed PMID: WOS:000665868700001.

3795. Yanming Q, Peng W, Xuanxuan X, Zheng L. Analysis of risk factors of acute respiratory distress syndrome secondary to severe multiple trauma. Zhonghua Wei Zhong Bing Ji Jiu Yi Xue. 2021;33(3):299-304. doi: 10.3760/cma.j.cn121430-20201023-00685.

3796. Yao L, Baker JL, Schiff ND, Purpura KP, Shoaran M. Predicting task performance from biomarkers of mental fatigue in global brain activity. J Neural Eng. 2021;18(3). Epub 2020/10/28. doi: 10.1088/1741-2552/abc529. PubMed PMID: 33108778; PubMed Central PMCID: PMCPMC8122624.

3797. Yao S, Chi J, Wang Y, Zhao Y, Luo Y, Wang Y. Zn-MOF Encapsulated Antibacterial and Degradable Microneedles Array for Promoting Wound Healing. Advanced Healthcare Materials. 2021;10(12). doi: 10.1002/adhm.202100056. PubMed PMID: WOS:000646236000001.

3798. Yasen AL, Eick GN, Sterner KN, Christie AD. Motor Cortex Function in APOE4 Carriers and Noncarriers. Journal of Clinical Neurophysiology. 2021;38(6):553-7. doi: 10.1097/wnp.0000000000000738. PubMed PMID: WOS:000756261600018.

3799. Yeh N, Yang Y, Huang S, Ku P, Wang R. Effects of transcranial direct current stimulation followed by exercise on

neuropathic pain in chronic spinal cord injury: a double-blinded randomized controlled pilot trial. Spinal cord. 2021;59(6):684-92. doi: 10.1038/s41393-020-00560-x. PubMed PMID: CN-02308880.

3800. Yu J, Meng F, He F, Chen F, Bao W, Yu Y, et al. Metabolic abnormalities in patients with chronic disorders of consciousness. Aging and Disease. 2021;12(2):386-403. doi: 10.14336/AD.2020.0812.

3801. Yu W, Li X, Huang Y, Chen Y, Gao Q, Wang Y, et al. Build an implanted "arsenal": detachable microneedles for NIR-triggered cancer photothermo-chemotherapy. Biomaterials Science. 2021;9(13):4737-45. doi: 10.1039/d1bm00520k. PubMed PMID: WOS:000653853500001.

3802. Yu Y, Yang Y, Gan S, Guo S, Fang J, Wang S, et al. Cerebral Hemodynamic Correlates of Transcutaneous Auricular Vagal Nerve Stimulation in Consciousness Restoration: An Open-Label Pilot Study. Front Neurol. 2021;12:684791. Epub 2021/08/03. doi: 10.3389/fneur.2021.684791. PubMed PMID: 34335449; PubMed Central PMCID: PMCPMC8319239.

3803. Yuan A, Xia F, Bian Q, Wu H, Gu Y, Wang T, et al. Ceria Nanozyme-Integrated Microneedles Reshape the Perifollicular Microenvironment for Androgenetic Alopecia Treatment. Acs Nano. 2021;15(8):13759-69. doi: 10.1021/acsnano.1c05272. PubMed PMID:

WOS:000693105500108.
3804. Zamunér AR, Minonzio M, Shiffer D, Fornerone R, Cairo B, Porta A, et al. Relationships Between Cardiovascular Autonomic Profile and Work Ability in Patients With Pure Autonomic Failure. Frontiers in Human Neuroscience. 2021;15. doi: 10.3389/fnhum.2021.761501.
3805. Zardkhoshoui AM, Ameri B, Davarani SSH. alpha-MnS@Co3S4 hollow nanospheres assembled from nanosheets for hybrid supercapacitors. Chemical Engineering Journal. 2021;422. doi: 10.1016/j.cej.2021.129953. PubMed PMID: WOS:000669703700002.
3806. Zare MR, Khorram M, Barzegar S, Sarkari B, Asgari Q, Ahadian S, et al. Dissolvable carboxymethyl cellulose/polyvinylpyrrolidone microneedle arrays for transdermal delivery of Amphotericin B to treat cutaneous leishmaniasis. International Journal of Biological Macromolecules. 2021;182:1310-21. doi: 10.1016/j.ijbiomac.2021.05.075. PubMed PMID: WOS:000669497600014.
3807. Zettin M, Bondesan C, Nada G, Varini M, Dimitri D. Transcranial Direct-Current Stimulation and Behavioral Training, a Promising Tool for a Tailor-Made Post-stroke Aphasia Rehabilitation: A Review. Frontiers in Human Neuroscience. 2021;15. doi:

10.3389/fnhum.2021.742136.

3808. Zhang D, Wang JLJ, Wang P, Cheng Y, Yuan Y, Song W. Efficacy comparison of noninvasive brain stimulation techniques on picture naming ability of non-fluent aphasia patients after stroke with normal expression capacity. Chinese Journal of Cerebrovascular Diseases. 2021;18(2):84-90. doi: 10.3969/j.issn.1672-5921.2021.02.002.

3809. Zhang J, Kucyi A, Raya J, Nielsen AN, Nomi JS, Damoiseaux JS, et al. What have we really learned from functional connectivity in clinical populations? Neuroimage. 2021;242. doi: 10.1016/j.neuroimage.2021.118466. PubMed PMID: WOS:000693332400010.

3810. Zhang P, Ilagan R, Bai Y, Zhang X, Deng Y, Ding Y. Editorial: Plasticity and Reconstruction of Neural Network in Brain Injury. Frontiers in Cellular Neuroscience. 2021;15. doi: 10.3389/fncel.2021.710499. PubMed PMID: WOS:000669842700001.

3811. Zhang X, Liu B, Li Y, Duan G, Hou J, Wu D. Multi-Target and Multi-Session Transcranial Direct Current Stimulation in Patients With Prolonged Disorders of Consciousness: A Controlled Study. Front Neurosci. 2021;15:641951. Epub 2021/09/28. doi: 10.3389/fnins.2021.641951. PubMed PMID: 34566555; PubMed

Central PMCID: PMCPMC8456025.
3812. Zhang XH, Han P, Zeng YY, Wang YL, Lv HL. The Clinical Effect of Repetitive Transcranial Magnetic Stimulation on the Disturbance of Consciousness in Patients in a Vegetative State. Front Neurosci. 2021;15:647517. Epub 2021/05/18. doi: 10.3389/fnins.2021.647517. PubMed PMID: 33994925; PubMed Central PMCID: PMCPMC8119637.
3813. Zhang Y, Du W, Yin Y, Li H, Liu Z, Yang Y, et al. Impaired cerebral vascular and metabolic responses to parametric N-back tasks in subjective cognitive decline. Journal of Cerebral Blood Flow and Metabolism. 2021;41(10):2743-55. doi: 10.1177/0271678X211012153.
3814. Zhang Y, Wu M, Tan D, Liu Q, Xia R, Chen M, et al. A dissolving and glucose-responsive insulin-releasing microneedle patch for type 1 diabetes therapy. Journal of Materials Chemistry B. 2021;9(3). doi: 10.1039/d0tb02133d. PubMed PMID: WOS:000612856700008.
3815. Zhong M, Cywiak C, Metto AC, Liu X, Qian C, Pelled G. Multi-session delivery of synchronous rTMS and sensory stimulation induces long-term plasticity. Brain Stimulation. 2021;14(4):884-94. doi: 10.1016/j.brs.2021.05.005.
3816. Zhou L, Huang X, Li H, Guo R, Wang J, Zhang Y, et al.

Rehabilitation effect of rTMS combined with cognitive training on cognitive impairment after traumatic brain injury. Am J Transl Res. 2021;13(10):11711-7. Epub 2021/11/18. PubMed PMID: 34786098; PubMed Central PMCID: PMCPMC8581933.
3817. Zhu D, Wang M, Feng F, Nan N, Liu Y, Shi J, et al. Correlation between clinical features and total maturation score by magnetic resonance imaging in very low birth weight premature infants with brain injury. Ann Palliat Med. 2021;10(2):2089-97. Epub 2021/03/18. doi: 10.21037/apm-21-55. PubMed PMID: 33725767.
3818. Zigmantovich AS, Oknina LB, Kopachka MM, Masherow EL, Alexandrova EV. Functional Wavelet-Connectivity in Resting State Reflects Consciousness Recovery in Patients with Severe Traumatic Brain Injury. Fiziologiya cheloveka. 2021;47(2):22-31. doi: 10.31857/s0131164621020144. PubMed PMID: RSCI:44810855.
3819. Zimdahl JW, Thomas H, Bolland SJ, Leggett K, Barry KM, Rodger J, et al. Excitatory Repetitive Transcranial Magnetic Stimulation Over Prefrontal Cortex in a Guinea Pig Model Ameliorates Tinnitus. Frontiers in Neuroscience. 2021;15. doi: 10.3389/fnins.2021.693935.
3820. Aloi D, Jalali R, Tilsley P, Miall RC, Fernández-Espejo D. tDCS modulates effective connectivity during motor command following; a potential therapeutic target for disorders of consciousness.

Neuroimage. 2022;247:118781. Epub 2021/12/09. doi: 10.1016/j.neuroimage.2021.118781. PubMed PMID: 34879252; PubMed Central PMCID: PMCPMC8803542.

3821. Aloi D, Lafanechere M, Jalali R, Miall RC, Fernandez-Espejo D. tDCS Combined with Passive Mobilization Increases Brain Activity During Command-Following; a Proposed Intervention to Increase Behavioral Responsiveness in Prolonged Disorders of Consciousness. Brain Injury. 2022;36:130-. PubMed PMID: WOS:000761085700258.

3822. Bhome R, Zarkali A, Thomas GEC, Iglesias JE, Cole JH, Weil RS. Thalamic white matter macrostructure and subnuclei volumes in Parkinson’s disease depression. npj Parkinson's Disease. 2022;8(1). doi: 10.1038/s41531-021-00270-y.

3823. Borders JC, Grande AA, Troche MS. Statistical Power and Swallowing Rehabilitation Research: Current Landscape and Next Steps. Dysphagia. 2022. doi: 10.1007/s00455-022-10428-2. PubMed PMID: WOS:000762171900002.

3824. Chang Y, Venton BJ. Dual-Channel Electrochemical Measurements Reveal Rapid Adenosine is Localized in Brain Slices. Acs Chemical Neuroscience. 2022;13(4):477-85. doi: 10.1021/acschemneuro.1c00679. PubMed PMID: WOS:000756681000005.

3825. Chiang HS, Shakal S, Strain JF, Womack K, Kraut M, Vanneste S, et al. Reversal of unilateral hand movement dysfunction by high definition transcranial direct current stimulation in a patient with chronic traumatic brain injury. Brain Stimul. 2022;15(2):283-5. Epub 2022/01/24. doi: 10.1016/j.brs.2022.01.010. PubMed PMID: 35066190.

3826. Cho YS, Ryu O, Cho K, Kim D, Lim J, Hong SH, et al. The effect of charge-balanced transcutaneous electrical nerve stimulation on rodent facial nerve regeneration. Sci Rep. 2022;12(1):1388. Epub 2022/01/28. doi: 10.1038/s41598-022-05542-y. PubMed PMID: 35082405; PubMed Central PMCID: PMCPMC8791984.

3827. D'urso G, Toscano E, Sanges V, Sauvaget A, Sheffer CE, Riccio MP, et al. Cerebellar transcranial direct current stimulation in children with autism spectrum disorder: A pilot study on efficacy, feasibility, safety, and unexpected outcomes in tic disorder and epilepsy. Journal of Clinical Medicine. 2022;11(1). doi: 10.3390/jcm11010143.

3828. DaSilva AF, Datta A, Swami J, Kim DJ, Patil PG, Bikson M. The Concept, Development, and Application of a Home-Based High-Definition tDCS for Bilateral Motor Cortex Modulation in Migraine and Pain. Front Pain Res (Lausanne). 2022;3:798056. Epub 2022/03/18. doi: 10.3389/fpain.2022.798056. PubMed PMID:

35295794; PubMed Central PMCID: PMCPMC8915734.

3829. du Plessis S, Oni IK, Lapointe AP, Campbell C, Dunn JF, Debert CT. Treatment of Persistent Postconcussion Syndrome With Repetitive Transcranial Magnetic Stimulation Using Functional Near-Infrared Spectroscopy as a Biomarker of Response: Protocol for a Randomized Controlled Clinical Trial. JMIR Res Protoc. 2022;11(3):e31308. Epub 2022/03/23. doi: 10.2196/31308. PubMed PMID: 35315783.

3830. Duan YJ, Hua XY, Zheng MX, Wu JJ, Xing XX, Li YL, et al. Corticocortical paired associative stimulation for treating motor dysfunction after stroke: Study protocol for a randomised sham-controlled double-blind clinical trial. BMJ Open. 2022;12(1). doi: 10.1136/bmjopen-2021-053991.

3831. Eapen BC, Bowles AO, Sall J, Lang AE, Hoppes CW, Stout KC, et al. The management and rehabilitation of post-acute mild traumatic brain injury. Brain Inj. 2022:1-10. Epub 2022/02/15. doi: 10.1080/02699052.2022.2033848. PubMed PMID: 35152817.

3832. Ek-Huchim JP, Árcega-Cabrera F, May-Tec AL, Améndola-Pimenta M, Ceja-Moreno V, Rodríguez-Canul R. Red Blood Cell Cytotoxicity Associated to Heavy Metals and Hydrocarbons Exposure in Flounder Fish from Two Regions of the Gulf of Mexico. Bull Environ Contam Toxicol. 2022;108(1):78-84.

Epub 2021/03/25. doi: 10.1007/s00128-021-03176-w. PubMed PMID: 33759008.

3833. Fan J, Zhong Y, Wang H, Aierken N, He R. Repetitive transcranial magnetic stimulation improves consciousness in some patients with disorders of consciousness. Clin Rehabil. 2022:2692155221089455. Epub 2022/03/25. doi: 10.1177/02692155221089455. PubMed PMID: 35322709.

3834. Franke LM, Gitchel GT, Perera RA, Hadimani RL, Holloway KL, Walker WC. Randomized trial of rTMS in traumatic brain injury: improved subjective neurobehavioral symptoms and increases in EEG delta activity. Brain Inj. 2022:1-10. Epub 2022/02/11. doi: 10.1080/02699052.2022.2033845. PubMed PMID: 35143365.

3835. Garcia-Pallero M, Cardona D, Rueda-Ruzafa L, Rodriguez-Arrastia M, Roman P. Central nervous system stimulation therapies in phantom limb pain: a systematic review of clinical trials. Neural Regeneration Research. 2022;17(1):59-64. doi: 10.4103/1673-5374.314288. PubMed PMID: WOS:000661581500010.

3836. Hac NEF, Gold DR. Neuro-Visual and Vestibular Manifestations of Concussion and Mild TBI. Curr Neurol Neurosci Rep. 2022. Epub 2022/03/03. doi: 10.1007/s11910-022-01184-9. PubMed PMID: 35235169.

3837. Jang SH, Byun DH. A Review of Studies on the Role of Diffusion Tensor Magnetic Resonance Imaging Tractography in the Evaluation of the Fronto-Subcortical Circuit in Patients with Akinetic Mutism. Medical Science Monitor. 2022;28. doi: 10.12659/msm.936251. PubMed PMID: WOS:000758334200001.

3838. Keswani T, Obeidallah A, Nieves E, Sidoli S, Fazzari M, Taylor T, et al. Pipecolic Acid, a Putative Mediator of the Encephalopathy of Cerebral Malaria and the Experimental Model of Cerebral Malaria. J Infect Dis. 2022;225(4):705-14. Epub 2021/12/22. doi: 10.1093/infdis/jiab615. PubMed PMID: 34932816; PubMed Central PMCID: PMCPMC8844588.

3839. Khan A, Antonakakis M, Vogenauer N, Haueisen J, Wolters CH. Individually optimized multi-channel tDCS for targeting somatosensory cortex. Clinical Neurophysiology. 2022;134:9-26. doi: 10.1016/j.clinph.2021.10.016.

3840. Koh DTS, Chen JY, Tan SM, Tay KS, Singh IR, Yeo NEM. Mid-Term Functional and Radiological Outcomes of Total Ankle Replacement in an Asian Cohort. Journal of Foot and Ankle Surgery. 2022;61(2):363-8. doi: 10.1053/j.jfas.2021.09.014.

3841. Kornfeld S, Kalambaheti E, Antonucci MM. Rehabilitation of persistent symptoms and neurocognitive deficits following sports related concussion in a professional hockey player: Case study.

Neurology. 2022;98(1 SUPPL). doi: 10.1212/01.wnl.0000801980.87446.22.

3842. Krieger D, Shepard P, Soose R, Puccio A, Beers S, Schneider W, et al. MEG-Derived Symptom-Sensitive Biomarkers with Long-Term Test-Retest Reliability. Diagnostics. 2022;12(1). doi: 10.3390/diagnostics12010084. PubMed PMID: WOS:000758893600001.

3843. Mabaso SH, Bhana-Nathoo D, Lucas S. An audit of CT brain findings in adults with new-onset seizures in a resource restricted setting in South Africa. SA J Radiol. 2022;26(1):2294. Epub 2022/02/17. doi: 10.4102/sajr.v26i1.2294. PubMed PMID: 35169503; PubMed Central PMCID: PMCPMC8831926.

3844. Manganotti P, Ajčević M, Buoite Stella A. EEG as a marker of brain plasticity in clinical applications. Handb Clin Neurol. 2022;184:91-104. Epub 2022/01/18. doi: 10.1016/b978-0-12-819410-2.00029-1. PubMed PMID: 35034760.

3845. Mollica A, Dey A, Cairncross M, Silverberg N, Burke MJ. Neuropsychiatric Treatment for Mild Traumatic Brain Injury: Nonpharmacological Approaches. Semin Neurol. 2022. Epub 2022/02/04. doi: 10.1055/s-0041-1742143. PubMed PMID: 35114694.

3846. Mollica A, Greben R, Oriuwa C, Siddiqi SH, Burke MJ. Neuromodulation Treatments for Mild Traumatic Brain Injury and

Post-concussive Symptoms. Curr Neurol Neurosci Rep. 2022. Epub 2022/02/18. doi: 10.1007/s11910-022-01183-w. PubMed PMID: 35175543.

3847. Navot-Mintzer D, Ekstein D, Nof E, Fogelman R, Beinart R, Honig A, et al. Rare Cause of Syncope in an Athlete, Matter of Brain and Heart Electricity. Clin J Sport Med. 2022;32(1):e104-e5. Epub 2021/08/28. doi: 10.1097/jsm.0000000000000967. PubMed PMID: 34446650.

3848. Nct. Control Network Neuromodulation to Enhance Cognitive Training in Complex Traumatic Brain Injury. https://clinicaltrialsgov/show/NCT05236010. 2022. PubMed PMID: CN-02367654.

3849. Nct. Combined Transcranial Magnetic Stimulation and Therapy for mTBI Related Headaches. https://clinicaltrialsgov/show/NCT05176392. 2022. PubMed PMID: CN-02355597.

3850. Nct. Probing the Dorsolateral Prefrontal Cortex and Central Executive Network for Improving Neuromodulation in Depression. https://clinicaltrialsgov/show/NCT05224063. 2022. PubMed PMID: CN-02367408.

3851. Nct. Effects of Individualized rTMS in DOC Patients. https://clinicaltrialsgov/show/NCT05187000. 2022. PubMed PMID:

CN-02366658.
3852. Nct. Long-term Effects of Repetitive TMS in Chronic Neuropathic Pain in People With SCI. https://clinicaltrialsgov/show/NCT05226676. 2022. PubMed PMID: CN-02367457.
3853. Nct. Combining Cognitive Training With tDCS to Enhance Memory Inhibition in Young Binge Drinkers. https://clinicaltrialsgov/show/NCT05237414. 2022. PubMed PMID: CN-02367685.
3854. Nguyen AL, Kirkwood B, Hackett L, Buntine P. Validation of the ECHS non trauma cranial CT rule in Australia: A prospective cohort study. American Journal of Emergency Medicine. 2022;52:225-31. doi: 10.1016/j.ajem.2021.12.004.
3855. Nousia A, Martzoukou M, Liampas I, Siokas V, Bakirtzis C, Nasios G, et al. The Effectiveness of Non-Invasive Brain Stimulation Alone or Combined with Cognitive Training on the Cognitive Performance of Patients With Traumatic Brain Injury: A Systematic Review. Arch Clin Neuropsychol. 2022;37(2):497-512. Epub 2021/06/23. doi: 10.1093/arclin/acab047. PubMed PMID: 34155517.
3856. Nousia A, Martzoukou M, Liampas I, Siokas V, Bakirtzis C, Nasios G, et al. The Effectiveness of Non-Invasive Brain Stimulation Alone or Combined with Cognitive Training on the Cognitive

Performance of Patients With Traumatic Brain Injury: A Systematic Review. Archives of Clinical Neuropsychology. 2022;37(2):497-512. doi: 10.1093/arclin/acab047. PubMed PMID: WOS:000755899200001.

3857. Papa A, Pasquini S, Contri C, Gemma S, Campiani G, Butini S, et al. Polypharmacological Approaches for CNS Diseases: Focus on Endocannabinoid Degradation Inhibition. Cells. 2022;11(3). doi: 10.3390/cells11030471.

3858. Patel H, Naish K, Soreni N, Amlung M. The Effects of a Single Transcranial Direct Current Stimulation Session on Impulsivity and Risk Among a Sample of Adult Recreational Cannabis Users. Frontiers in Human Neuroscience. 2022;16. doi: 10.3389/fnhum.2022.758285.

3859. Pavitt S, Gelfand AA, Zorrilla N, Allen I, Riggins N. Efficacy and Safety of Repetitive Intravenous Sodium Valproate in Pediatric Patients With Refractory Chronic Headache Disorders: A Retrospective Review. Pediatric Neurology. 2022;128:52-7. doi: 10.1016/j.pediatrneurol.2021.12.008.

3860. Penezic A, Gasparovic B, Cuculic V, Strmecki S, Djakovac T, Mlakar M. Dissolved Trace Metals and Organic Matter Distribution in the Northern Adriatic, an Increasingly Oligotrophic Shallow Sea. Water. 2022;14(3). doi: 10.3390/w14030349. PubMed PMID:

WOS:000755544900001.

3861. Peng Y, Zhao J, Lu X, Dong J, Zhang S, Zhang J, et al. Efficacy of Transcranial Direct Current Stimulation Over Dorsolateral Prefrontal Cortex in Patients With Minimally Conscious State. Front Neurol. 2022;13:821286. Epub 2022/03/08. doi: 10.3389/fneur.2022.821286. PubMed PMID: 35250824; PubMed Central PMCID: PMCPMC8894202.

3862. Porcaro C, Nemirovsky IE, Riganello F, Mansour Z, Cerasa A, Tonin P, et al. Diagnostic Developments in Differentiating Unresponsive Wakefulness Syndrome and the Minimally Conscious State. Frontiers in Neurology. 2022;12. doi: 10.3389/fneur.2021.778951. PubMed PMID: WOS:000752778200001.

3863. Qureshi AY, Stevens RD. Mapping the Unconscious Brain: Insights From Advanced Neuroimaging. J Clin Neurophysiol. 2022;39(1):12-21. Epub 2021/09/03. doi: 10.1097/wnp.0000000000000846. PubMed PMID: 34474430.

3864. Ramage AE, Ray KL, Franz HM, Tate DF, Lewis JD, Robin DA. Cingulo-Opercular and Frontoparietal Network Control of Effort and Fatigue in Mild Traumatic Brain Injury. Frontiers in Human Neuroscience. 2022;15. doi: 10.3389/fnhum.2021.788091.

3865. Ramos-Fresnedo A, Perez-Vega C, Domingo RA, Cheshire

WP, Middlebrooks EH, Grewal SS. Motor Cortex Stimulation for Pain: A Narrative Review of Indications, Techniques, and Outcomes. Neuromodulation. 2022;25(2):211-21. Epub 2022/02/08. doi: 10.1016/j.neurom.2021.10.025. PubMed PMID: 35125140.

3866. Srihagulang C, Vongsfak J, Vaniyapong T, Chattipakorn N, Chattipakorn SC. Potential roles of vagus nerve stimulation on traumatic brain injury: Evidence from in vivo and clinical studies. Exp Neurol. 2022;347:113887. Epub 2021/10/09. doi: 10.1016/j.expneurol.2021.113887. PubMed PMID: 34624329.

3867. Stunnenberg BC, Berends J, Griggs RC, Statland J, Drost G, Nikles J, et al. N-of-1 Trials in Neurology A Systematic Review. Neurology. 2022;98(2):E174-E85. doi: 10.1212/WNL.0000000000012998.

3868. Sui K, Meneghetti M, Kaur J, Sørensen RJF, Berg RW, Markos C. Adaptive polymer fiber neural device for drug delivery and enlarged illumination angle for neuromodulation. J Neural Eng. 2022;19(1). Epub 2022/02/08. doi: 10.1088/1741-2552/ac5267. PubMed PMID: 35130533.

3869. Tiotiu A, Badi Y, Kermani NZ, Sanak M, Kolmert J, Wheelock CE, et al. Association of Differential Mast Cell Activation with Granulocytic Inflammation in Severe Asthma. Am J Respir Crit Care Med. 2022;205(4):397-411. Epub 2021/11/24. doi:

10.1164/rccm.202102-0355OC. PubMed PMID: 34813381.

3870. Wang Y, Niu Z, Xia X, Bai Y, Liang Z, He J, et al. Application of Fast Perturbational Complexity Index to the Diagnosis and Prognosis for Disorders of Consciousness. IEEE Trans Neural Syst Rehabil Eng. 2022;30:509-18. Epub 2022/02/26. doi: 10.1109/tnsre.2022.3154772. PubMed PMID: 35213312.

3871. Winters B, Kuluris B, Pathmanaban R, Vanderwalt H, Thibaut A, Schnakers C. Changes of Spasticity across Time in Prolonged Disorders of Consciousness: A Retrospective Study. Brain Sciences. 2022;12(2). doi: 10.3390/brainsci12020295.

3872. Wong JK, Deuschl G, Wolke R, Bergman H, Muthuraman M, Groppa S, et al. Proceedings of the Ninth Annual Deep Brain Stimulation Think Tank: Advances in Cutting Edge Technologies, Artificial Intelligence, Neuromodulation, Neuroethics, Pain, Interventional Psychiatry, Epilepsy, and Traumatic Brain Injury. Front Hum Neurosci. 2022;16:813387. Epub 2022/03/22. doi: 10.3389/fnhum.2022.813387. PubMed PMID: 35308605; PubMed Central PMCID: PMCPMC8931265.

3873. Wu M, Wu Y, Yu Y, Gao J, Meng F, He F, et al. Corrigendum to: "Effects of theta burst stimulation of the left dorsolateral prefrontal cortex in disorders of consciousness". Brain Stimul. 2022;15(2):426. Epub 2022/02/15. doi: 10.1016/j.brs.2022.02.001. PubMed PMID:

35158110.
3874. Xiong M, Qin Y, Chai B, Yan J, Fan G, Xu F, et al. Unveiling the role of Mn-Cd-S solid solution and MnS in MnxCd1-xS photocatalysts and decorating with CoP nanoplates for enhanced photocatalytic H-2 evolution. Chemical Engineering Journal. 2022;428. doi: 10.1016/j.cej.2021.131069. PubMed PMID: WOS:000711644200002.
3875. Xu R, Zhu GY, Zhu J, Wang Y, Xing XX, Chen LY, et al. Using Hebbian-Type Stimulation to Rescue Arm Function After Stroke: Study Protocol for a Randomized Clinical Trial. Frontiers in Neural Circuits. 2022;15. doi: 10.3389/fncir.2021.789095.
3876. Yang S, Kwak SG, Choi GS, Chang MC. Short-term Effect of Repetitive Transcranial Magnetic Stimulation on Diabetic Peripheral Neuropathic Pain. Pain Physician. 2022;25(2):E203-e9. Epub 2022/03/25. PubMed PMID: 35322973.
3877. Zhang T, Song B, Li Y, Duan R, Gong Z, Jing L, et al. Neurofilament Light Chain as a Biomarker for Monitoring the Efficacy of Transcranial Magnetic Stimulation on Alcohol Use Disorder. Frontiers in Behavioral Neuroscience. 2022;16. doi: 10.3389/fnbeh.2022.831901.
3878. Zhang Y, Chen W, Zhang T, Du J, Li R, Huo R, et al. P300 correlates with tDCS response in minimally conscious state patients.

Neurosci Lett. 2022;774:136534. Epub 2022/02/20. doi: 10.1016/j.neulet.2022.136534. PubMed PMID: 35181480.

3. Studies included in review

1. Cooper JB, Jane JA, Alves WM, Cooper EB. Right median nerve electrical stimulation to hasten awakening from coma. Brain Inj. 1999;13(4):261-7. Epub 1999/05/07. doi: 10.1080/026990599121638. PubMed PMID: 10230527.

2. Peri CV, Shaffrey ME, Farace E, Cooper E, Alves WM, Cooper JB, et al. Pilot study of electrical stimulation on median nerve in comatose severe brain injured patients: 3-month outcome. Brain Inj. 2001;15(10):903-10. Epub 2001/10/12. doi: 10.1080/02699050110065709. PubMed PMID: 11595086.

3. Cincotta M, Giovannelli F, Chiaramonti R, Bianco G, Godone M, Battista D, et al. No effects of 20 Hz-rTMS of the primary motor cortex in vegetative state: A randomised, sham-controlled study. Cortex. 2015;71:368-76. Epub 2015/08/25. doi: 10.1016/j.cortex.2015.07.027. PubMed PMID: 26301875.

4. Nekkanti S, Shaik R, Mondem S, Meruva N, Elumalai G. Effect of right median nerve stimulation on level of consciousness in traumatic brain injury subjects. Asian journal of pharmaceutical research and health care. 2016;8(3):67-71. doi: 10.18311/ajprhc/2016/751. PubMed PMID: CN-01165502.

5. Estraneo A, Pascarella A, Moretta P, Masotta O, Fiorenza S, Chirico G, et al. Repeated transcranial direct current stimulation in

prolonged disorders of consciousness: A double-blind cross-over study. J Neurol Sci. 2017;375:464-70. Epub 2017/03/23. doi: 10.1016/j.jns.2017.02.036. PubMed PMID: 28320187.

6. Martens G, Thibaut A, Huang W, Di H, Laureys S. Repeated stimulation of the posterior parietal cortex in patients in minimally conscious state: a sham-controlled randomized clinical trial. Brain stimulation. 2017;10(2):370-. doi: 10.1016/j.brs.2017.01.090. PubMed PMID: CN-01361661.

7. Thibaut A, Wannez S, Donneau AF, Chatelle C, Gosseries O, Bruno MA, et al. Controlled clinical trial of repeated prefrontal tDCS in patients with chronic minimally conscious state. Brain Inj. 2017;31(4):466-74. Epub 2017/03/11. doi: 10.1080/02699052.2016.1274776. PubMed PMID: 28281845.

8. Zhang Y, Song W, Du J, Huo S, Shan G, Li R. Transcranial Direct Current Stimulation in Patients with Prolonged Disorders of Consciousness: Combined Behavioral and Event-Related Potential Evidence. Front Neurol. 2017;8:620. Epub 2017/12/07. doi: 10.3389/fneur.2017.00620. PubMed PMID: 29209270; PubMed Central PMCID: PMCPMC5702306.

9. He F, Wu M, Meng F, Hu Y, Gao J, Chen Z, et al. Effects of 20Hz Repetitive Transcranial Magnetic Stimulation on Disorders of Consciousness: A Resting-State Electroencephalography Study. Neural

Plasticity. 2018;2018. doi: 10.1155/2018/5036184. PubMed PMID: WOS:000429681300001.

10. Martens G, Lejeune N, O'Brien AT, Fregni F, Martial C, Wannez S, et al. Randomized controlled trial of home-based 4-week tDCS in chronic minimally conscious state. Brain Stimul. 2018;11(5):982-90. Epub 2018/05/16. doi: 10.1016/j.brs.2018.04.021. PubMed PMID: 29759943.

11. Cavinato M, Genna C, Formaggio E, Gregorio C, Storti SF, Manganotti P, et al. Behavioural and electrophysiological effects of tDCS to prefrontal cortex in patients with disorders of consciousness. Clin Neurophysiol. 2019;130(2):231-8. Epub 2018/12/24. doi: 10.1016/j.clinph.2018.10.018. PubMed PMID: 30580246.

12. Wu M, Yu Y, Luo L, Wu Y, Gao J, Ye X, et al. Efficiency of Repetitive Transcranial Direct Current Stimulation of the Dorsolateral Prefrontal Cortex in Disorders of Consciousness: A Randomized Sham-Controlled Study. Neural Plast. 2019;2019:7089543. Epub 2019/07/17. doi: 10.1155/2019/7089543. PubMed PMID: 31308848; PubMed Central PMCID: PMCPMC6594311.

13. Zhang XH, Han P, Zeng YY, Wang YL, Lv HL. The Clinical Effect of Repetitive Transcranial Magnetic Stimulation on the Disturbance of Consciousness in Patients in a Vegetative State. Front Neurosci. 2021;15:647517. Epub 2021/05/18. doi:

10.3389/fnins.2021.647517. PubMed PMID: 33994925; PubMed Central PMCID: PMCPMC8119637.

14. Chen JM, Chen QF, Wang ZY, Chen YJ, Zhang NN, Xu JW, et al. Influence of High-Frequency Repetitive Transcranial Magnetic Stimulation on Neurobehavioral and Electrophysiology in Patients with Disorders of Consciousness. Neural Plast. 2022;2022:7195699. Epub 2022/11/29. doi: 10.1155/2022/7195699. PubMed PMID: 36437902; PubMed Central PMCID: PMCPMC9699789 authorship, and/or publication of this article.

15. Fan J, Zhong Y, Wang H, Aierken N, He R. Repetitive transcranial magnetic stimulation improves consciousness in some patients with disorders of consciousness. Clin Rehabil. 2022:2692155221089455. Epub 2022/03/25. doi: 10.1177/02692155221089455. PubMed PMID: 35322709.
